# DDX6 Is Essential for Oocyte Development and Maturation in *Locusta migratoria*

**DOI:** 10.3390/insects12010070

**Published:** 2021-01-14

**Authors:** Junxiu Wang, Tingting Li, Sufang Deng, Enbo Ma, Jianzhen Zhang, Shuping Xing

**Affiliations:** 1 Research Institute of Applied Biology, Shanxi University, Taiyuan 030006, Shanxi, China; wangjx0419@163.com (J.W.); ltt100518@163.com (T.L.); dengsufang_142@163.com (S.D.); maenbo2003@sxu.edu.cn (E.M.); zjz@sxu.edu.cn (J.Z.); 2 College of Life Science, Shanxi University, Taiyuan 030006, Shanxi, China; 3 College of Biological Sciences and Technology, Jinzhong University, Jinzhong 030600, Shanxi, China; 4 Shanxi Provincial Key Laboratory of Agricultural Integrated Pest Management, Taiyuan 030006, Shanxi, China

**Keywords:** DEAD-box helicase, DDX6, oocyte, vitellogenesis, *Locusta migratoria*

## Abstract

**Simple Summary:**

Insect reproduction is an important and complicated process required for producing healthy individuals and maintaining their population abundance. Thus, it could become a valuable target for insect biological control. To date, many factors and pathways have been revealed to be involved in this reproductive process, but it is still far from a full understanding of the molecular network underlying this process. We herein investigated a RNA helicase, DEAD-box protein 6 (DDX6) in *Locusta migratoria*, a global, destructive pest, and found that knockdown *LmDDX6* downregulated expression levels of juvenile hormone receptor gene methoprene-tolerant and its target genes 78-kDa glucose-regulated proteins, thus reducing *vitellogenin* expression and ultimately impairing the ovary development and oocyte maturation. These results demonstrate that LmDDX6 is a key player in female locust reproduction, providing, thus, a novel target for locust biological control.

**Abstract:**

DEAD-box protein 6 (DDX6) is a member of the DDX RNA helicase family that exists in all eukaryotes. It has been extensively studied in yeast and mammals and has been shown to be involved in messenger ribonucleoprotein assembly, mRNA storage, and decay, as well as in miRNA-mediated gene silencing. DDX6 participates in many developmental processes but the biological function of DDX6 in insects has not yet been adequately addressed. Herein, we characterized the *LmDDX6* gene that encodes the LmDDX6 protein in *Locusta migratoria*, a global, destructive pest. LmDDX6 possesses five motifs unique to the DDX6 subfamily. In the phylogenetic tree, LmDDX6 was closely related to its orthologs in *Apis dorsata* and *Zootermopsis nevadensis*. RT-qPCR data revealed high expression of *LmDDX6* in the ovary, muscle, and fat body, with a declining trend in the ovary after adult ecdysis. *LmDDX6* knockdown downregulated the expression levels of the juvenile hormone receptor *Met,* and genes encoding Met downstream targeted *Grp78-1* and *Grp78-2*, reduced *LmVg* expression, and impaired ovary development and oocyte maturation. These results demonstrate that *LmDDX6* plays an essential role in locust female reproduction and, thus, could be a novel target for locust biological control.

## 1. Introduction

Reproduction is an essential and complicated process required for producing healthy individuals and maintaining the population abundance of organisms. Insect female reproductive system is composed of two ovaries, containing a number of ovarioles, connected directly to the oviduct. The ovariole is the functional unit for egg production, and the number of ovarioles in each ovary varies widely, depending on the particular insect species [1,2]. In brief, the ovariole consists of the apical terminal filament, the germarium region linked to the terminal filament, and the vitellarium in the basal part [1]. Insects of different orders adopt distinct reproductive strategies. Thus far, three types of oogenesis have been described based on the presence and position of the nurse cells: (1) panoistic, (2) telotrophic meroistic, and (3) polytrophic meroistic [3,4]. Proper ovary development and oocyte maturation in insects are prerequisites for successful reproduction. Many intrinsic and extrinsic factors are involved in this process, including hormones, nutrition, and growth conditions [5,6,7,8].

Juvenile hormone (JH), one of the classical endocrine hormones produced by the *corpora allata*, a pair of endocrine glands in the retrocerebral complex behind the brain, has been long known to regulate female reproduction in many insects, exerting a central role in vitellogenesis, an indispensable process of ovary development and oocyte maturation [8]. As a gonadotropin, JH promotes female reproduction mainly by inducing the expression of *Vg* [8,9,10]. *Vg* is a key gene expressed largely in the fat body during vitellogenesis that encodes vitellogenin, a major precursor of the yolk protein, which is secreted into the hemolymph and taken up by maturing oocytes [11]. Besides the induction of *Vg* expression, JH enhances Vg uptake and promotes oocyte maturation [12]. In *Locusta migratoria*, several downstream genes of JH and its receptor complex have been found to regulate fat body polyploidization, including mini-chromosome maintenance 4/7 (*Mcm4/7*), cell division cycle 6 (*cdc6*), cyclin-dependent kinase 6 (*Cdk6*), and adenovirus E2 factor 1 (*E2f1*), reducing *LmVg* expression level and ultimately impairing oocyte growth and maturation [13,14,15]. Very recently, it has been found that JH promotes the expression of *Cdc2* and origin recognition complex subunit 5 (*Orc5*) via the LCMT1-PP2A-FoxO pathway, mediating fat body ploidy, and reduces the expression of *LmVg* [16]. In *Tribolium castaneum*, JH induces the expression level of the gene encoding insulin-like peptides in the fat body, phosphorylating fork head transcription factor FOXO, and promoting *Vg* expression [10]. In the American cockroach, it has been recently reported that the insulin/IGF signaling and targeting of rapamycin induce the expression of *Jhamt* and *Cyp15a*, the enzymes of the last two steps of JH biosynthesis, thus activating JH biosynthesis, eventually affecting vitellogenesis and oocyte maturation [17]. In *D. melanogaster*, JH enhances Vg uptake and promotes oocyte maturation [18].

Ecdysteroid is another classical hormone that controls oocyte development and maturation in insects [19]. The active form of ecdysteroid, 20-hydroxyecdysone, has been reported to contribute to *Drosophila* oogenesis, where it tightly controls the developmental checkpoint at stage 8, which allows the onset of vitellogenesis and egg maturation. Females with mutation in ecdysone receptor contained abnormal egg chambers [20]. In mosquitoes, inhibition of the target of rapamycin protein synthesis impeded *Vg* expression and reduced fecundity, suggesting that the nutritional signaling pathway contributes to mosquito ovary development and oocyte maturation [6]. In addition, miRNAs have been shown to be involved in female reproduction [21]. These data indicate that although there has been some progress in the elucidation of the molecular mechanism of ovary development and oocyte maturation in insects, the current body of knowledge is just the tip of the iceberg. To fully understand the molecular network underlying complicated reproductive processes in insects, more factors and pathways need to be taken into account.

DEAD-box proteins (DDXs) comprise a large family of RNA helicases that are conserved from bacteria to eukaryotes. They share nine common, conserved motifs in the helicase core [22]. Motif II that contains four amino acids (Asp-Glu-Ala-Asp, i.e., DEAD) is required for ATPase activity. Other motifs are involved in ATP binding and hydrolysis as well as in RNA binding [23]. Multiple studies have demonstrated that DDXs are involved in almost every aspect of RNA metabolism, from transcription, splicing, transport, ribosome biosynthesis, and translation to RNA decay, so they have multifaceted biological functions in cells. For example, in yeast, 15 of 25 DDXs have been demonstrated to regulate ribosome biogenesis [24]. DDX20/DP103 in mammalian cells has been shown to repress transcription [25,26]. Ded1/DDX3 is required for translation initiation [23,27], and Belle, the DDX3 ortholog in *Drosophila*, is required for male and female fertility [28,29]. DHH1/DDX6 is necessary for RNA decay [30]. Vasa/DDX4 is a germ cell marker required for fertility [31]. Many of these DDXs have been also shown to participate in tumorigenesis, antiviral reactions, and immune responses [32,33,34].

In our previous study, we isolated 32 *DDX* genes from *L. migratoria* and identified seven of these *LmDDXs* that were indispensable for nymph survival [35]. However, their detailed biological functions remained largely unknown. DDX6, an important translational repressor [30], has not yet been extensively studied in insects. Herein, we focused on the *LmDDX6* gene and characterized its function in oocyte development and maturation. Knockdown *of LmDDX6* reduced *LmVg* expression and downregulated expression levels of the methoprene-tolerant gene (*Met*) encoding the JH receptor and its downstream target gene *Grp78*, which ultimately resulted in oocyte abortion. These results confirmed that *LmDDX6* is a key player in locust female reproduction and, as such, it may be a new target for locust biological control.

## 2. Materials and Methods

### 2.1. Experimental Insects

Nymph locusts were purchased from a locust breeding center (Cangzhou, China) and reared in a cage with 50% relative humidity at 30 ± 2 °C under the 14 h:10 h (light:dark) photoperiod. Fresh wheat leaves and bran were fed to the locusts twice per day. Adult locusts after eclosion were used for the following experiments.

### 2.2. Motif Pattern Analysis

Motif pattern analysis was conducted by using online program MEME (http://meme-suite.org/tools/meme). The ortholog sequences of DDX6 used in this analysis are listed in Supplemental File S1. The parameters were as follows: minimum width = 10, maximum width = 10, and maximum number of motifs to find = 18.

### 2.3. Phylogenetic Analysis of DDX6

DDX6 protein sequences from different species were obtained from NCBI (National Center for Biotechnology Information) and a multiple-sequence alignment was performed by using ClustalW software. The phylogenetic tree was generated by MEGA 6 by using the neighbor-joining method with 1000 repetitions. The protein accession numbers are shown in Table 1.

### 2.4. RNA Extraction and RT-qPCR

Integument, fat body, ovary, foregut, midgut, hindgut, malpighian tubule, and muscle tissues of female adults 2 days post-adult eclosion (PAE) were first sampled. Ovaries from 0, 2, 4, 6, and 8 days PAE locusts were also collected. Three individuals were sampled for one replicate, and three replicates were repeated. Total RNA was extracted by using RNAiso Plus reagent (Takara, Japan). First-strand cDNA was synthesized with 1 µg of total RNA by using an RNA HiScript^®^ III RT SuperMix for qPCR (+ gDNA wiper) Kit (Vazyme, Nanjing, China) according to the manufacturer′s instructions. Real-time quantitative PCR (RT-qPCR) was performed to measure the relative transcript level by using a LightCycler^®^ 480 Instrument II (Roche, Basel, Switzerland) with 2 × ChamQ^TM^ Universal SYBR^®^ qPCR MasterMix. The RT-qPCR program was conducted at 94 °C for 2 min, followed by 40 cycles of 94 °C for 15 s and 60 °C for 31 s. The specific primer sequences are summarized in Table S1. Relative gene expression was calculated by the 2^−ΔΔCT^ method. The level of β-actin mRNA expression was used as internal control.

### 2.5. RNA Interference (RNAi) 

The synthesis of the *LmDDX6* double-stranded RNA (ds*LmDDX6*) was described previously [35]. In brief, the region (484 bp) for ds*LmDDX6* from *LmDDX6* gene was amplified by PCR using the specific dsRNA primers (Table S1), which contain the T7 RNA polymerase promoter sequence. Ds*LmDDX6* was synthesized by using T7 RiboMAX^™^ Express RNAi System (Promega, USA) and dissolved in nuclease-free water. The ds*GFP* was synthesized in parallel and served as mock control. Female adult locusts within 12 h after eclosion (0 PAE) were injected with 10 µg of dsRNA at the second to third segments of the abdomen. The silencing efficiency of *LmDDX6* in the ovary and fat body from female locusts at 4, 6, and 8 days PAE was analyzed by RT-qPCR.

### 2.6. Tissue Imaging

Epson Perfection V600 Photo was used for imaging ovary morphology. The morphology and length of the ovarioles were analyzed by using a Leica M205C microscope.

### 2.7. Data Analysis

The relative expression level of *LmDDX6* in various tissues was calculated using one-way analysis of variance (ANOVA), as appropriate, by using SPSS 16.0 software. The post hoc Tukey’s test was used if F value in one-way ANOVA reported a significant effect. The different letters indicate a significant difference. The comparison of the gene expression and the size of primary oocytes between the ds*LmDDDX6-* and the ds*GFP*-treated locusts were analyzed by the two-sample and two-tail t-test. All statistical analyses were conducted at the significance level of α = 0.05 (*p* < 0.05).

## 3. Results

### 3.1. Motif Patterns of LmDDX6 and Its Orthologs

**Figure 1:**
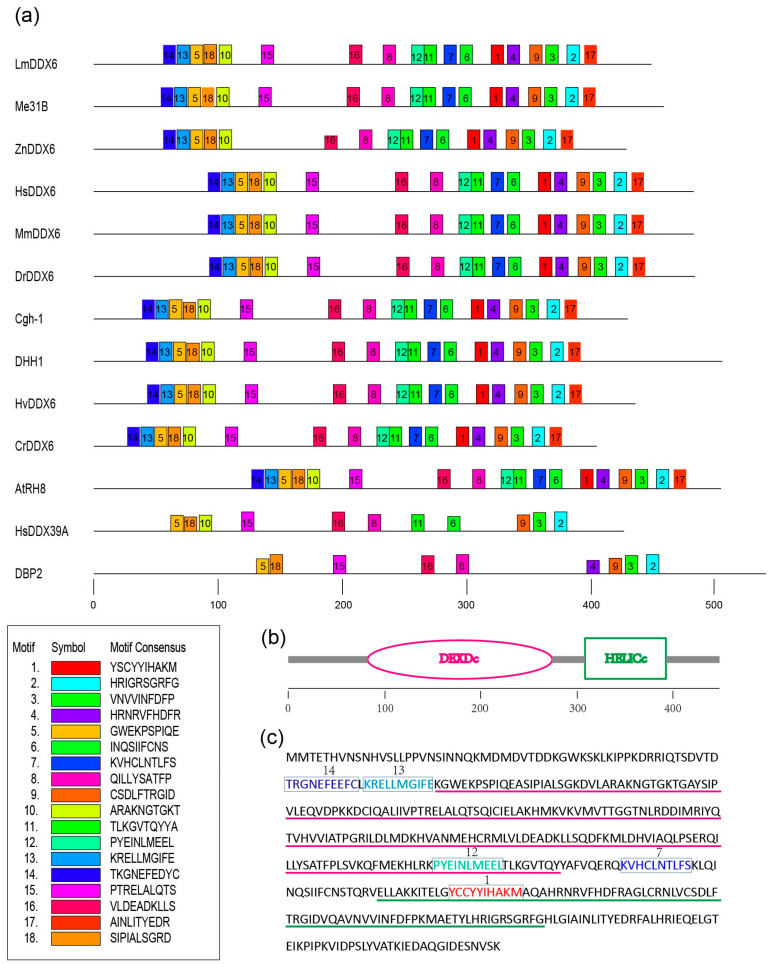
Motif analysis of DDX6 orthologs. Thirteen sequences from different phyla, including yeast (*Saccharomyces cerevisiae*, DHH1), cnidarian (*Hydra vulgaris*, HvDDX6), worm (*Caenorhabditis elegans*, Cgh-1), insects (*Locusta migratoria*, LmDDX6; *Drosophila melanogaster*, Me31B; *Zootermopsis nevadensis*, ZnDDX6), vertebrates (*Homo sapiens*, HsDDX6; *Mus musculus*, MmDDX6; *Danio rerio*, DrDDX6), and green plants (*Chlamydomonas reinhardtii*, CrDDX6; *Arabidopsis thaliana*, AtRH8), as well as sequences of two outgroup members, HsDDX39A from *H. sapiens* and DBP2 from *C. cerevisiae*, were analyzed using MEME (www.meme-suite.org) program. The parameters were as follows: minimum width = 10, maximum width = 10, and maximum number of motifs to find = 18. (**a**) Motif patterns of the selected sequences. The numbers with different colors indicate various motifs. The number with the vertical, short line denotes the number of amino acids. (**b**) Domain arrangement of LmDDX6 analyzed by SMART (smart.embl-heidelberg.de). (**c**) Amino acid sequence of LmDDX6, showing the five unique motifs marked in different colors. The pink and the green underlines indicate the DEXDc and HELICc domains, respectively.

*LmDDX6* encodes a protein of 449 amino acids (aa) that form the conserved DEXDc and HELICc domains with the N- and C-terminal regions of 78 aa and 58 aa, respectively ([35]; Table 1). Both the N- and C-terminal sequences are more variable than the conserved domains among the members of the DDX family or even of the same DDX subfamily. To search for some motifs that might be present only in the DDX6 subfamily, we first selected 13 sequences from different phyla, including yeast (*Saccharomyces cerevisiae*, DHH1), cnidarian (*Hydra vulgaris*, HvDDX6), worm (*Caenorhabditis elegans*, Cgh-1), insects (*L. migratoria*, LmDDX6; *D. melanogaster*, Me31B; *Zootermopsis nevadensis*, ZnDDX6), vertebrates (*Homo sapiens*, HsDDX6; *Mus musculus*, MmDDX6; *Danio rerio*, DrDDX6), and green plants (*Chlamydomonas reinhardtii*, CrDDX6; *Arabidopsis thaliana*, AtRH8), as well as sequences of two outgroup members, HsDDX39A from *H. sapiens* and DBP2 from *S. cerevisiae*. These sequences were analyzed as one group using MEME program (www.meme-suite.org). We found six motifs (motifs 14, 13, 12, 7, 1, and 17) in the DDX6 subfamily but not in HsDDX39A or DBP2 among the 18 motifs examined (Figure 1a). We then explored more sequences (48 as one group) and confirmed five out of the six motifs (motif 17 was removed this time, Figure S1). To obtain further confirmation, 27 sequences from algae, 191 sequences from insects, and 500 sequences from vertebrates, plants, and fungi, respectively, were downloaded and examined manually for the five motifs, one by one (Supplemental Files S2–S6). Without exception, all five motifs were maintained after this examination. Therefore, we discovered five new motifs highly conserved in the DDX6 subfamily, including two motifs (13, KRELLMGIFE and 14, TKGNEFEDYC) in the N-terminal region, one motif (12, PYEINLMEEL) in the DEXDc domain, one motif (1, YSCYYIHAKM) in the HELICc domain, and one motif (7, KVHCLNTLFS) in the linker region between the DEXDc and HELICc domains (Figure 1b,c). The specificities and biological roles of these motifs are currently unknown.

### 3.2. Phylogeny of LmDDX6 and Its Orthologs

**Figure 2:**
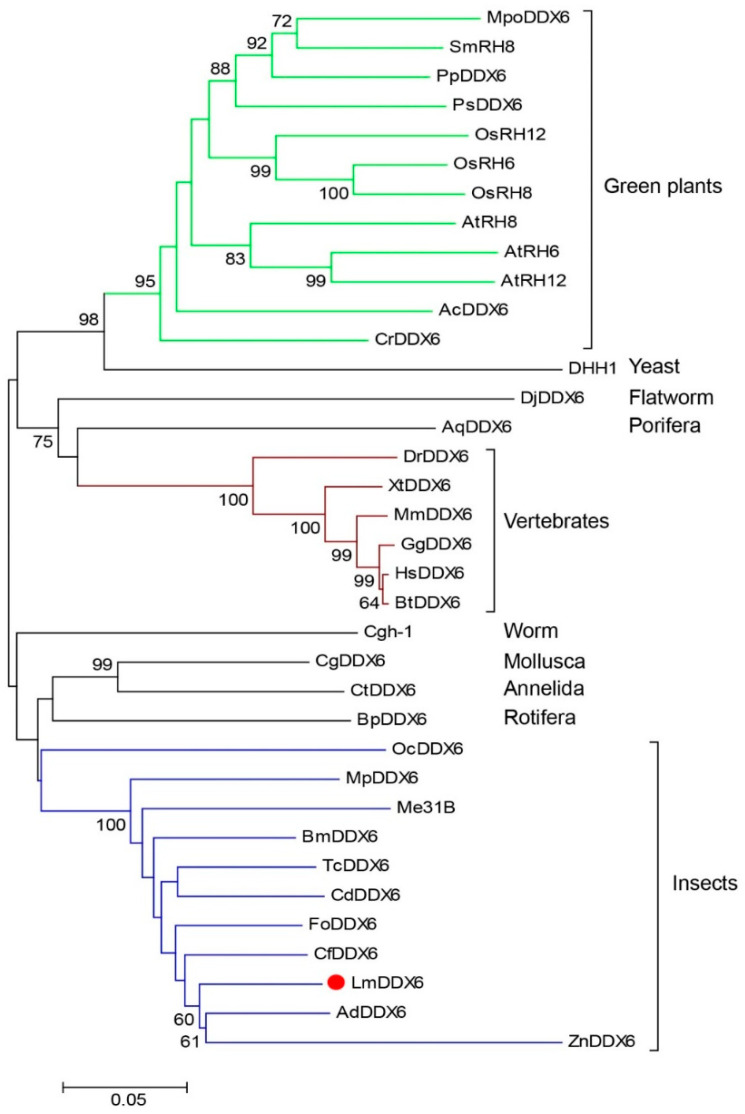
A phylogenetic tree of DDX6 orthologs. DDX6 protein sequences from different species were obtained from NCBI (National Center for Biotechnology Information), and a multiple-sequence alignment was performed using ClustalW software. The phylogenetic tree was generated by MEGA 6 using neighbor-joining method with 1000 repetitions. The filled, red circle indicates DDX6 from *L. migratoria*. Protein accession numbers are shown in Table 1.

To understand the relationship between LmDDX6 and other DDX6 subfamily members, we generated a phylogenetic tree (Figure 2) by using DDX6 sequences from 32 species selected from diverse phyla (Table 1). DDX6 sequences from green plants, vertebrates, and insects were classified into distinct clades. LmDDX6 in the insect clade was closely related to the orthologs from *Apis dorsata* and *Z. nevadensis*. Cgh-1 from worm and a group of DDX6 orthologs from Mollusca *(Crassostrea gigas*), Annelida (*Capitella teleta*), and Rotifera (*Brachionus plicatilis*) were more closely related to the insect clades. The flatworm (*Dugesia japonica*) and porifera (*Amphimedon queenslandica*) sequences were closely related to the vertebrate clade. DHH1, a yeast DDX6 ortholog, was strikingly related to the sequences from green plants. These data demonstrate that DDX6 appears in unicellular eukaryotes, such as *C. reinhardtii* and *S. cerevisiae*, and is retained by multicellular eukaryotes.

Intriguingly, when we searched for LmDDX6-like sequences in the BLAST bacterial database in NCBI, we found sequence WP_162815294.1 from *Microbacterium arborescen*, which showed an overall 90% identity with LmDDX6 and also contained the five motifs unique to the DDX6 subfamily. Interestingly, when a BLAST search of WP_162815294.1 was performed in NCBI, we found another sequence, from *Papilio polytes*, with 100% identity with WP_162815294.1. That sequence was a DDX6 ortholog from *P. polytes* and in the reconstructed phylogenetic tree (Figure S2) it was closely related to the sequence of BmDDX6, a DDX6 ortholog in *Bombyx mori*. Based on these data, we assume that sequence WP_162815294.1 from *M. arborescen* was probably a contamination from the sequence of *P. polytes*.

### 3.3. Expression Profile of LmDDX6 in Female Adults

**Figure 3:**
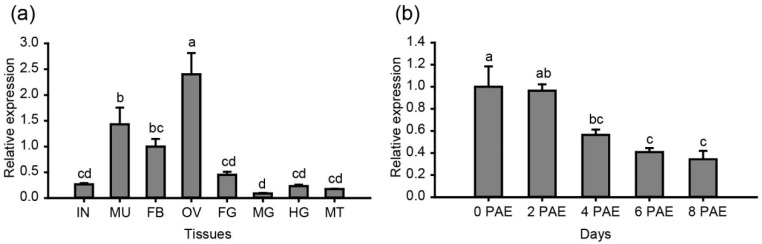
Expression profile of *LmDDX6* in female adult locusts. (**a**) *LmDDX6* mRNA expression in different tissues of female adult locusts at two days PAE. IN, integument; MU, muscle; FB, fat body, OV, ovary, FG, foregut, MG, midgut, HG, hindgut, MT, malpighian tubules. (**b**) Expression of *LmDDX6* at different stages of adult ovary development. Data are presented as the mean ± standard error of the mean of three independent biological replicates. Data points indicated by different letters are significantly different.

In our previous study, we detected high expression of *LmDDX6* in the testis and ovary and intermediate expression in the fat body and Malpighian tubules of five-instar nymphs [35]. To determine *LmDDX6* expression in adult females, we collected various tissues from female locusts at 2 days PAE and analyzed them by RT-qPCR. A high expression level of *LmDDX6* was detected in the ovary and an intermediate level of expression was detected in the fat body and muscle. Other tissues, including integument, foregut, midgut, hindgut, and Malpighian tubules showed lower *LmDDX6* expression levels (Figure 3a). To determine the relationship between the expression of *LmDDX6* and ovary development, we sampled the ovaries on different days PAE and conducted RT-qPCR. We observed a high level of *LmDDX6* expression in the first two days PAE, with a declining trend afterwards (Figure 3b). These expression data suggest that *LmDDX6* plays a role in ovary development and oocyte maturation.

### 3.4. Knockdown of LmDDX6 Leads to Oocyte Abortion

**Figure 4:**
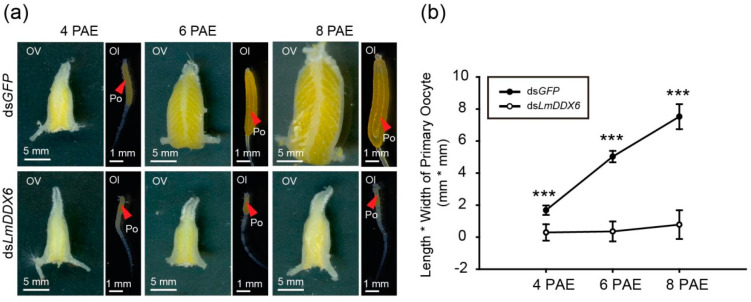
Effects of ds*LmDDX6* treatment on locust ovary development and oocyte maturation. (**a**) Morphology of the ovaries (Ov) and ovarioles (Ol) in adult female locusts after injection of ds*LmDDX6* or ds*GFP*. Ol, ovariole; Ov, ovary; Po, primary oocyte. PAE, days post adult eclosion. Scale bars: Ov, 5 mm; Ol, 1 mm. (**b**). The length × width index of primary oocytes from locusts injected with ds*LmDDX6* or ds*GFP* at 4–8 days PAE. Data are presented as the mean ± standard error of the mean (*n* = 8–13). Statistical significance of differences is indicated as follows: *** *p* < 0.001.

To elucidate the function of *LmDDX6*, we injected ds*LmDDX6* and ds*GFP* into female locusts within several hours PAE. Then, we dissected the injected locusts on different days PAE and observed the ovaries carefully. An obvious difference between ds*LmDDX6*-treated and control ds*GFP*-treated locusts first appeared at 4 days PAE. The size of the ovary was slightly smaller in the ds*LmDDX6*-injected locusts than in the control, ds*GFP*-injected, locusts. Strikingly, primary oocytes were smaller in ds*LmDDX6*-treated locusts (Figure 4a,b). Furthermore, this difference dramatically increased at 6 and 8 days PAE. The sizes of the ovary and primary oocytes in ds*GFP*-treated locusts increased greatly, with the latter reaching the maturation size of ~7.6 mm^2^ at 8 days PAE under our experimental conditions. In contrast, in the ds*LmDDX6*-treated locusts, the ovary and primary oocytes did not change much from 4 to 8 days PAE, with only a slight increase from 0.5 to 0.9 mm^2^ for the primary oocyte size (Figure 4a,b).

### 3.5. Downregulation of Vg Expression by LmDDX6 Knockdown

**Figure 5:**
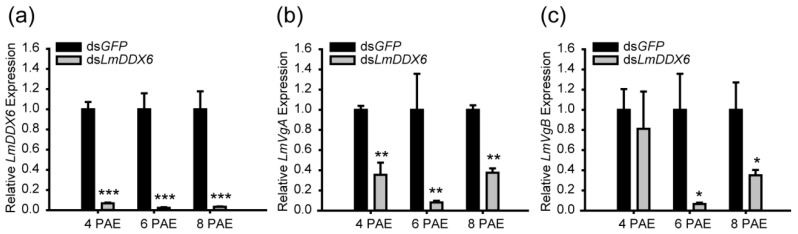
Effect of *LmDDX6* depletion on the expression of the vitellogenin (*Vg*) gene. (**a**) *LmDDX6* RNA interference efficiency in the fat body of ds*LmDDX6*-injected adult females compared with that in ds*GFP*-injected controls. (**b**,**c**) Relative expression levels of *LmVgA* (**b**) and *LmVgB.* (**c**) The mRNA in the fat body of female adult locusts after *LmDDX6* knockdown compared with the corresponding levels in the ds*GFP*-injected group. Data in all panels are presented as the mean ± standard error of the mean (*n* = 8–12). Statistical significance of differences is indicated as follows: * *p* < 0.05; ** *p* < 0.01; *** *p* < 0.001.

Vitellogenin is synthesized in the fat body and is essential for oocyte development. The phenotype of the ovaries and oocytes in the ds*LmDDX6*-treated locusts might be ascribed to the abnormal expression of *LmVg*. To investigate the expression profile of *LmVg* in the locusts, we first examined the silencing efficiency of *LmDDX6* in the fat body and found that it was high: 93.3%, 97.7%, and 96.7% at 4, 6, and 8 days PAE, respectively (Figure 5a). In locusts, two vitellogenin genes, A and B, have been described [36]. Therefore, we performed RT-qPCR for both *LmVgA* and *LmVgB* in the ds*GFP*- and ds*LmDDX6*-treated locusts at 4, 6, and 8 days PAE. As expected, the expression level of *LmVgA* significantly decreased by 64.5%, 91.9%, and 62.4%, respectively (Figure 5b). Similarly, the expression level of *LmVgB* at 6 and 8 days PAE was downregulated by 93.5% and 65%, whereas at 4 days PAE, no significant changes were detected between the ds*GFP*- and ds*LmDDX6*-treated locusts (Figure 5c).

The Vg receptor (VgR) is highly expressed in oocytes and is indispensable for the entry of Vg from the hemolymph into the oocyte. To this end, we examined *LmDDX6* silencing efficiency in the ovary and found a slight reduction (4.6%) at 4 days PAE and decreases by 39.4% and 35.7% at 6 days and 8 days PAE, respectively. We then checked *LmVgR* expression in the ds*GFP*- and ds*LmDDX6*-treated locusts. RT-qPCR results indicated that there was no significant difference in *LmVgR* expression between ds*GFP*- and ds*LmDDX6*-treated locusts at 4, 6, and 8 days PAE, respectively (Figure S3). This finding could have two explanations. First, due to the weak reduction of *LmDDX6* expression in the ovaries of ds*LmDDX6*-treated locusts, the remaining amount of *LmDDX6* expression could be sufficient to maintain the normal expression of *LmVgR*. Secondly, it is possible that LmDDX6 indeed has no effect on the expression of *LmVgR*.

### 3.6. Knockdown of LmDDX6 Affects JH Receptor Met Expression and Its Downstream Target Genes

**Figure 6:**
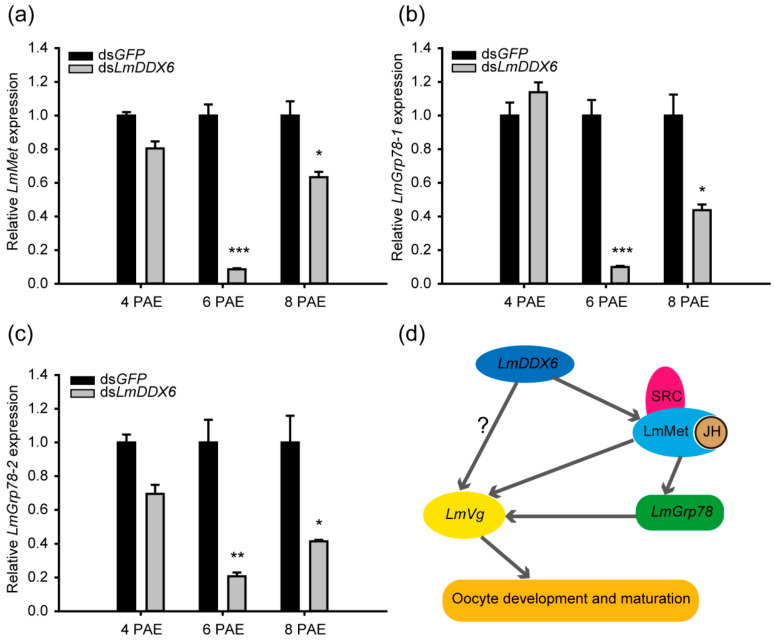
Sensitivity of *Met*, *Grp78-1*, and *Grp78-2* mRNA expression levels to *LmDDX6* knockdown in the fat body of female adult locusts. (**a**–**c**) Effect of *LmDDX6* knockdown on mRNA levels of *LmMet* (**a**), *LmGrp78-1* (**b**), and *LmGrp78-2* (**c**) in the fat body on days 4–8 PAE compared to the corresponding levels in ds*GFP*-injected locusts (negative control). Data are presented as the mean ± standard error of the mean (*n* = 8–12). Statistical significance of differences is indicated as follows: * *p* < 0.05; ** *p* < 0.01; *** *p* < 0.001. (**d**) A proposed model for the role of LmDDX6 in locust oocyte development and maturation. LmDDX6 regulates the expression of *LmVg* directly or indirectly via the JH signaling pathway and, thus, promotes vitellogenesis and oocyte maturation in the locust.

JH is a well-known regulator of vitellogenin synthesis during oocyte development and maturation [18]. To understand the relationship between *LmDDX6* expression and JH signaling pathway activity in vitellogenin synthesis, we chose to examine mRNA levels of the JH receptor Met and its downstream target genes *Grp78-1* and *Grp78-2* [37]. Indeed, the expression levels of these three genes were strongly downregulated at 6 d PAE in the ds*LmDDX6*-treated locusts with reductions by 91.4%, 90.1%, and 79.2%, respectively. However, these reductions in expression levels slightly recovered by 8 days PAE. Furthermore, no significant differences in expression levels of these genes were detected at 4 days PAE between the ds*GFP*- and ds*LmDDX6*-treated locusts (Figure 6a–c). These data clearly indicate a role for *LmDDX6* in the regulation of the JH signaling pathway.

Based on these data, we propose a model for the function of *LmDDX6* in ovary development and oocyte maturation (Figure 6d). *LmDDX6* is expressed in the fat body and its protein product may directly regulate *LmVg* expression there. Alternatively or in parallel, LmDDX6 may alter the expression of the JH receptor gene *LmMet* and its downstream genes *LmGrp78-1* and *LmGrp78-2* and thereby indirectly affect the expression of *LmVg*. In either case, downregulation of *LmVg* expression blocks oocyte development and maturation.

## 4. Discussion

DDX6 and its orthologs comprise one of the evolutionarily earliest families of DDX proteins. Most members of this subfamily are composed of 400–600 amino acids. In addition to the helicase core of 350–400 amino acids, some have elongated N-terminal regions, for example, orthologs in the vertebrates, whereas others have long C-terminal regions, e.g., yeast DHH1 and Ste13 [30]. The sequences of the DDX6 orthologs show high similarity even in their N- and C-terminal regions. To clarify the motifs that might be unique to the DDX6 subfamily, we conducted a MEME motif analysis among various orthologs from the yeast to the mammals and plants. Intriguingly, five such motifs, each containing 10 residues, were identified in this study (Figure 1). Two motifs (consensus TKGNEFEDYC and KRELLMGIFE) are located at the end of the N-terminal region and are closely connected to the Q-motif, which is essential for the ATPase activity of the DDX family members [22]. The other three motifs are in the helicase core, namely, in the DEXDc domain (PYEINLMEEL), in the HELICc domain (YSCYYIHAKM), and in the linker region between the two domains (KVHCLNTLFS). These motifs may determine ATP binding affinity or specificity of the mRNA and other factors associated with DDX6 and its orthologs. To this end, information on the detailed structure and residue mutation analysis as well as related biochemical data are needed.

DDX6 sequence is closely related to that of the translational initiation factor eIF4A, which contains just the helicase core without the longer N- and C-terminal sequences [30,38]. In *S. cerevisiae*, two genes, *TIF1* and *TIF2*, encode the same product, eIF4A [39]. Orthologs of eIF4A are also found in bacteria, e.g., in Acidimicrobiaceae and Magnetococcales (Supplemental File S7). Curiously, to see whether we could find a direct ortholog of DDX6 in bacteria, we performed a BLAST search using the LmDDX6 sequence in the bacterial database in NCBI. Interestingly, we found only one ortholog in *M. arborescens*, but not in any other bacterial species. However, in the constructed phylogenetic tree, this ortholog was found to be closely related to that of *B. mori* (Figure S2), raising the question of the origin of this sequence. When we reblasted this sequence in NCBI, we found that it had 100% identity to the DDX6 ortholog in *P. polytes*, a species from Lepidoptera. It was reported that Lepidopteran species have large gut bacterial community and *M. arborescens* had been found in the midgut of *Spodoptera litura* [40]. Therefore, it is possible that this sequence was a contamination from *P. polytes* during sequencing of the *M. arborescens* genome. In this scenario, DDX6 appears in unicellular eukaryotes and is retained by all multicellular eukaryotes.

Many studies have demonstrated that DDX6 and its orthologs are involved in the assembly of messenger ribonucleoprotein (mRNP), RNA storage, translational repression, and mRNA decay [30]. Xp54, an DDX6 ortholog of Xenopus, is an integral component of mRNP particles in oocytes: It changes the conformation of the mRNP complex by displacing one subset of proteins to enable recruitment of the next one and thereby is involved in mRNP remodeling [41,42]. Me31B, Cgh-1, and DHH1, the DDX6 orthologs in *Drosophila*, *Caenorhabditis*, and *Saccharomyces*, respectively, are the core components of the processing body (PB) involved in mRNA decay, translational repression, and miRNA-mediated gene silencing [43,44,45,46,47]. The mRNA decay in the PB involves the central complex CCR4-CAF1-NOT, the decapping factor DCP1/2, and exonuclease Xrn1 [48]. DDX6 orthologs interact with the factors in the CCR4-CAF1-NOT complex, and this interaction seems to be conserved in some species [49]. Translational repression relies on other factors, such as the repressor protein RAP55 in the vertebrates and CAR-1 in *C. elegans* [50,51]. DDX6 orthologs bind to non-translational mRNA during oogenesis and early embryo development, and, thus, temporarily mask these mRNAs. Later, some of these mRNAs may be reused in later development [42]. In addition, DDX6 interacts directly with AGO1 and AGO2, which are involved in miRNA-mediated gene silencing [52].

Yeast Ste13 is necessary for sexual reproduction, whereas DHH1 regulates cell cycle [53,54]. Me31B, as a component of *Drosophila* germ granules, plays an essential role in germline development [55]. Moreover, DDX6 in mice has been shown to function in gametogenesis and early embryogenesis [56]. Similarly, the DDX6 ortholog Cgh-1 in *C. elegans* is required for gametogenesis and protection from physiological germline apoptosis [57]. However, the function of DDX6 in locusts has not yet been investigated.

We found that knockdown of *LmDDX6* arrested locust oocyte development and maturation, indicating an essential role for LmDDX6 in female locust reproduction. Furthermore, mRNA expression levels of *LmVg* and JH receptor *Met*, as well as of *Grp78-1* and *Grp78-2*, two downstream genes of the JH receptor complex, were significantly reduced in the fat body of the ds*LmDDX6*-injected locusts (Figure 5). Therefore, *LmDDX6* likely regulates ovary development and oocyte maturation by affecting vitellogenesis, at least partially, via the maintenance of the JH signaling pathway activity (Figure 6d). To determine whether LmDDX6 is also involved in mRNA decay, translational repression, and miRNA-mediated gene silencing, as are other DDX6 orthologs [30], we plan to isolate various components of the CCR4-NOT complex and the decapping factors Dcp1 and Dcp2 from *L. migratoria*. In order to understand better the mechanism whereby LmDDX6 controls the key processes of female locust reproduction, it will be necessary to establish whether LmDDX6 directly interacts with those factors, including LmAGO1.

## 5. Conclusions

In this study, we characterized LmDDX6, the DDX6 ortholog in *L. migratoria*, and found that it possesses five unique motifs to the DDX6 subfamily. LmDDX6 is closely related to its orthologs in *Apis dorsata* and *Zootermopsis nevadensis*. In adult female, *LmDDX6* is highly expressed in ovary, muscle, and fat body. Knockdown of *LmDDX6* elicits reduced expression levels of JH receptor *Met* and its downstream targets *Grp78-1* and *Grp78-2*, downregulates *LmVg* expression, and impairs ovary development and oocyte maturation. As such, LmDDX6 is a key player in female locust reproduction and thus could be a novel target for locust biological control.

## Figures and Tables

**Table 1:** List of the genes analyzed in the phylogenetic tree.

Gene Symbol	Full-Length (aa)	N-Termini (aa)	C-Termini (aa)	Protein ID	Species
*LmDDX6*	449	78	58	QOS47384.1	*Locusta migratoria*
*Me31B*	459	76	69	NP_523533.2	*Drosophila melanogaster*
*BmDDX6*	440	74	52	XP_012545299.1	*Bombyx mori*
*TcDDX6*	441	71	56	XP_015834522.1	*Tribolium castaneum*
*CfDDX6*	443	73	56	XP_026461540.1	*Ctenocephalides felis*
*AdDDX6*	444	73	57	XP_006610567.1	*Apis dorsata*
*FoDDX6*	440	67	59	XP_026291730.1	*Frankliniella occidentalis*
*CdDDX6*	450	57	79	CAB3359348.1	*Cloeon dipterum*
*MpDDX6*	446	75	57	XP_022182727.1	*Myzus persicae*
*OcDDX6*	463	86	63	ODM96281.1	*Orchesella cincta*
*ZnDDX6*	429	78	57	XP_021926685.1	*Zootermopsis nevadensis*
*HsDDX6*	483	114	54	NP_001244120.1	*Homo sapiens*
*BtDDX6*	483	114	54	NP_001137339.1	*Bos taurus*
*MmDDX6*	483	114	54	NP_001104296.1	*Mus musculus*
*GgDDX6*	483	114	54	NP_001006319.2	*Gallus gallus*
*XtDDX6*	481	113	53	NP_001072584.1	*Xenopus tropicalis*
*DrDDX6*	484	115	54	XP_684923.1	*Danio rerio*
*Cgh-1*	430	61	55	NP_498646.1	*Caenorhabditis elegans*
*DHH1*	506	64	128	NP_010121.1	*Saccharomyces cerevisiae S288C*
*CgDDX6*	447	69	64	XP_011429888.1	*Crassostrea gigas*
*DjDDX6*	503	67	122	BAF57607.1	*Dugesia japonica*
*CtDDX6*	458	86	58	ELT97926.1	*Capitella teleta*
*BpDDX6*	470	61	95	RNA08982.1	*Brachionus plicatilis*
*AqDDX6*	444	63	67	XP_003386052.1	*Amphimedon queenslandica*
*MbDDX6*	400	33	53	XP_001749654.1	*Monosiga brevicollis MX1*
*CrDDX6*	405	49	42	XP_001692202.1	*Chlamydomonas reinhardtii*
*MpoDDX6*	515	159	42	PTQ47051.1	*Marchantia polymorpha*
*PpDDX6*	448	92	42	XP_024367950.1	*Physcomitrium patens*
*SmRH8*	460	104	42	XP_002987276.2	*Selaginella moellendorffii*
*AcDDX6*	443	87	42	MBC9844858.1	*Adiantum capillus-veneris*
*PsDDX6*	477	121	42	ABR16163.1	*Picea sitchensis*
*AtRH6*	528	172	42	AAK63966.1	*Arabidopsis thaliana*
*AtRH8*	505	149	42	NP_191975.2	*Arabidopsis thaliana*
*AtRH12*	498	142	42	CAA09203.1	*Arabidopsis thaliana*
*OsRH6*	498	142	42	XP_015636229.1	*Oryza sativa*
*OsRH8*	508	152	42	XP_015627069.1	*Oryza sativa*
*OsRH12*	521	165	42	XP_015614831.1	*Oryza sativa*
*MaDDX6*	426	60	52	WP_162815294.1	*Microbacterium arborescens*

## Data Availability

All data produced from this study are included in this published paper.

## Supplementary Materials

The following are available online at https://www.mdpi.com/2075-4450/12/1/70/s1. Figure S1: Motif pattern analysis of multiple DDX6 sequences from diverse species. Figure S2: Effect of *LmDDX6* RNAi on the expression of locust vitellogenin receptor gene. Figure S3: Phylogenetic tree of DDX6 orthologs. Table S1: Primers used in this study. Supplemental File S1: The 48 sequences used for Figure 1 and Figure S1. Supplemental File S2: The 27 sequences of DDX6 from algae. Supplemental File S3: The 191 sequences of DDX6 from insects. Supplemental File S4: The 500 sequences of DDX6 from fungi. Supplemental File S5: The 500 sequences of DDX6 from vertebrates. Supplemental File S6: The 500 sequences of DXX6 from plants. Supplemental File S7: Translation initiation factor from yeast and bacterium.
