# Supplementary material for: DDX6 Is Essential for Oocyte Development and Maturation in Locusta migratoria
Source: Insects. 2021 Jan 14;12(1):70. doi: 10.3390/insects12010070 (PMC7830464; doi:10.3390/insects12010070)
Supplement: Supplementary file 1 [file insects-12-00070-s001.zip › Supplementary material/Figure S1.pdf]

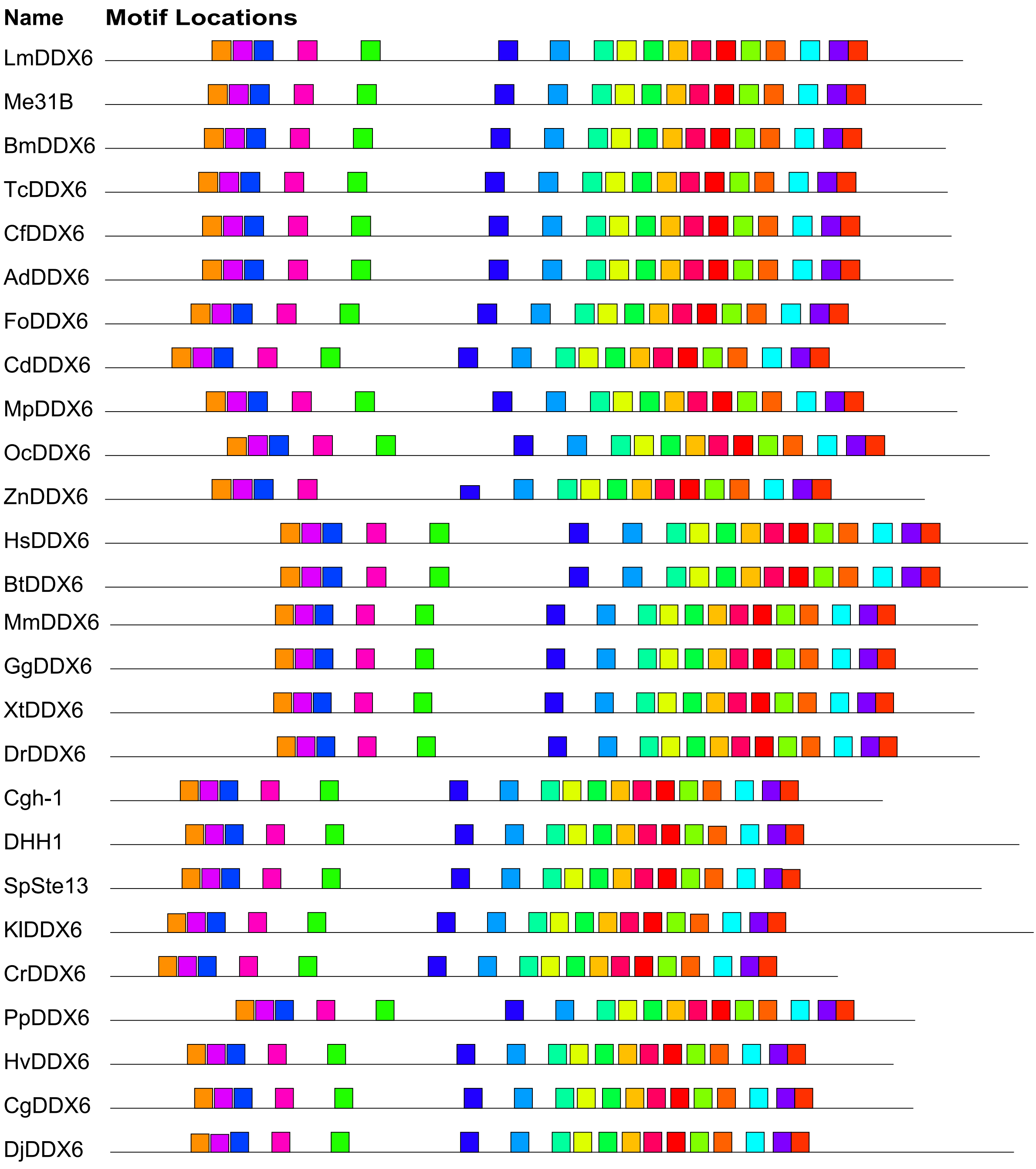

Figure S1. Motif pattern analysis of multiple DDX6 sequences from diverse species. Forty-eight sequences were used for the MEME analysis (http://meme-suite.org/tools/meme) (Supplemental file 1). The parameters were as follows: minimum width = 10, maximum width = 10, and the maximum number of motifs to find = 18.

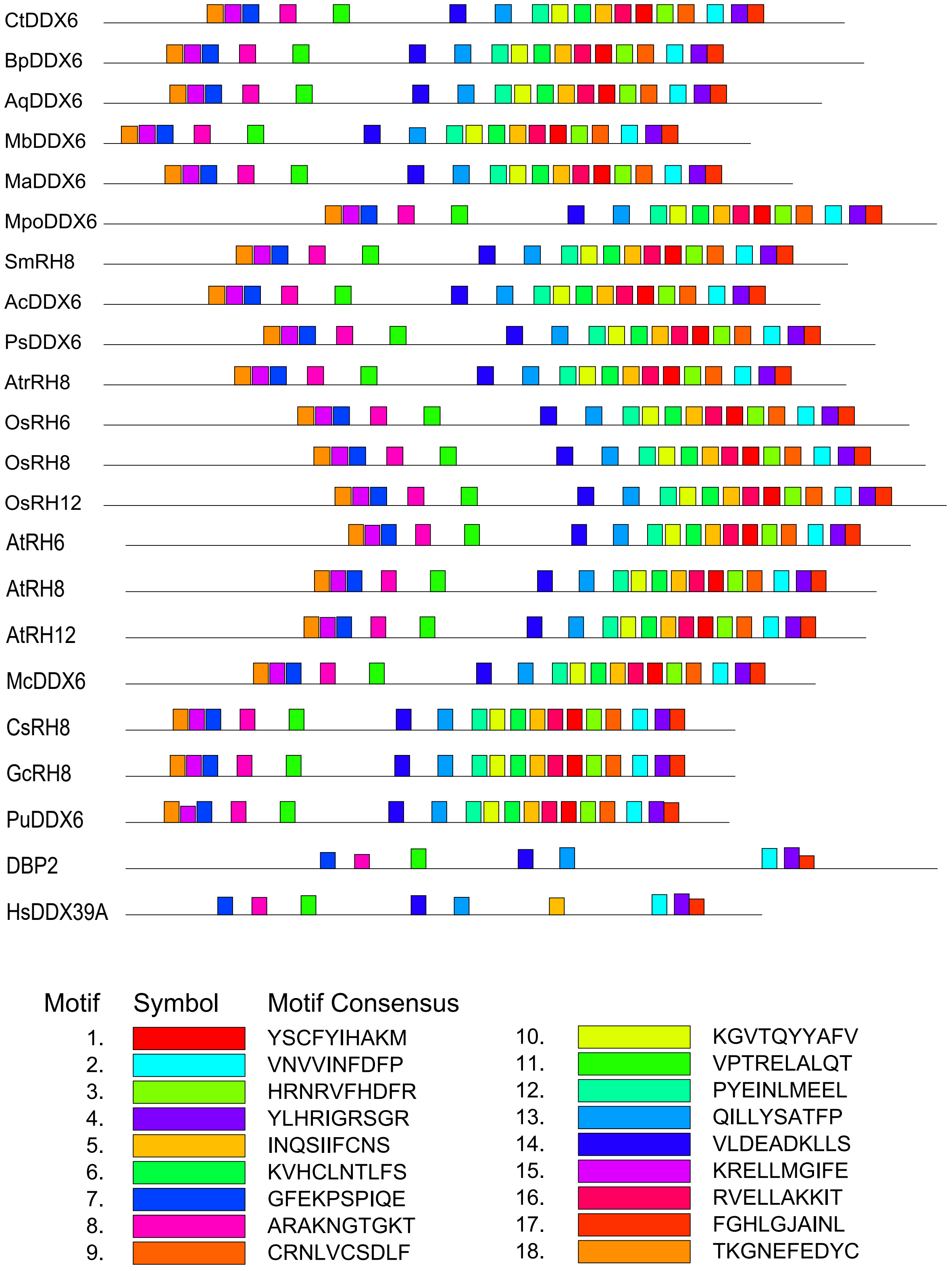

CtDDX6
BpDDX6
AqDDX6
MbDDX6
MaDDX6
MpoDDX6
SmRH8
AcDDX6
PsDDX6
AtrRH8
OsRH6
OsRH8
OsRH12
AtRH6
AtRH8
AtRH12
McDDX6
CsRH8
GcRH8
PuDDX6
DBP2
HsDDX39A
Motif
Symbol
Motif Consensus
1. YSCFYIHAKM
2. VNVVINFDFP
3. HRNRVFHDFR
4. YLHRIGRSGR
5. INQSIIFCNS
6. KVHCLNTLFS
7. GFEKPSPIQE
8. ARAKNGTGKT
9. CRNLVCSDLF
10. KGVTQYYAFV
11. VPTRELALQT
12. PYEINLMEEL
13. QILLYSATFP
14. VLDEADKLLS
15. KRELLMGIFE
16. RVELLAKKIT
17. FGHLGJAINL
18. TKGNEFEDYC
