# Supplementary material for: DDX6 Is Essential for Oocyte Development and Maturation in Locusta migratoria
Source: Insects. 2021 Jan 14;12(1):70. doi: 10.3390/insects12010070 (PMC7830464; doi:10.3390/insects12010070)
Supplement: Supplementary file 1 [file insects-12-00070-s001.zip › Supplementary material/Figure S2.pdf]

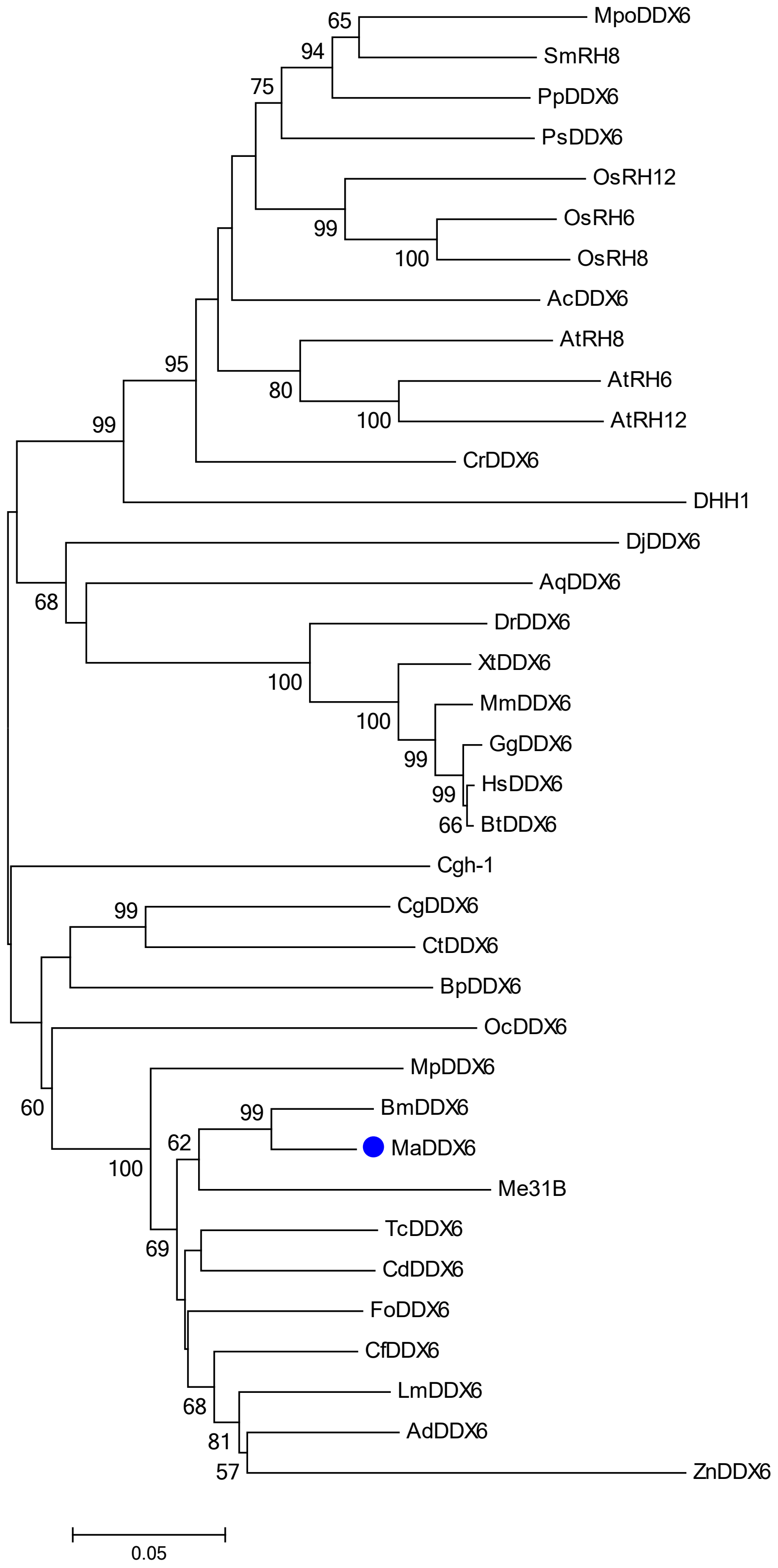

Figure S2. Phylogenetic tree of DDX6 orthologs. DDX6 protein sequences from different species were obtained from NCBI (National Center for Biotechnology Information) and a multiple sequence alignment was performed by using ClustalW software. The phylogenetic tree was generated by MEGA 6 by using the neighbor-joining method with 1,000 repetitions. The protein accession numbers are shown in Table 1. The filled blue circle indicates the DDX6 ortholog from Microbacterium arborescen.
