# Supplementary material for: DDX6 Is Essential for Oocyte Development and Maturation in Locusta migratoria
Source: Insects. 2021 Jan 14;12(1):70. doi: 10.3390/insects12010070 (PMC7830464; doi:10.3390/insects12010070)
Supplement: Supplementary file 1 [file insects-12-00070-s001.zip › Supplementary material/Figure S3.pdf]

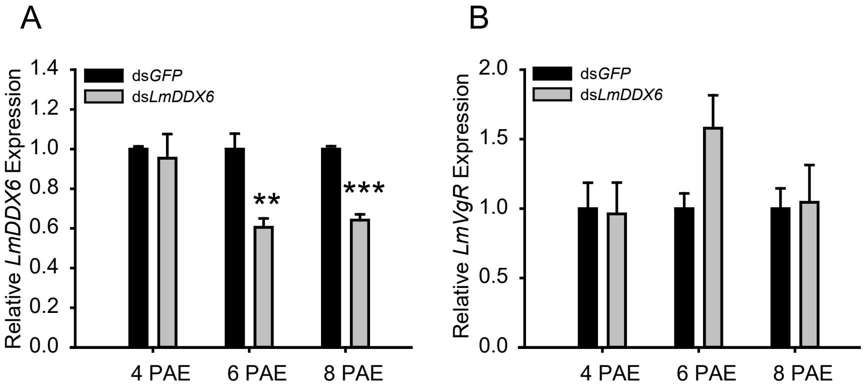

Figure S3. Effect of LmDDX6 RNAi on the expression of locust vitellogenin receptor gene (LmVgR). (a) Efficiency of LmDDX6 RNAi in the ovary after the treatment with dsLmDDX6 compared to the level in the dsGFP-injected group (b) Relative expression of LmVgR in the ovary of female adult at 4-8 days PAE. In both panels, data are presented as the mean ± standard error of the mean (n = 8 − 12). Statistical significance of differences is presented as follows: **$P < 0.01$; ***$P < 0.001$.
