# Supplementary material for: DDX6 Is Essential for Oocyte Development and Maturation in Locusta migratoria
Source: Insects. 2021 Jan 14;12(1):70. doi: 10.3390/insects12010070 (PMC7830464; doi:10.3390/insects12010070)
Supplement: Supplementary file 1 [file insects-12-00070-s001.zip › Supplementary material/Supplemental file 1.docx]

48 Sequences used for figure 1 and supplemental figure 1

>LmDDX6

MMTETHVNSNHVSLLPPVNSINNQKMDMDVTDDKGWKSKLKIPPKDRRIQTSDVTDTRGNEFEEFCLKRELLMGIFEKGWEKPSPIQEASIPIALSGKDVLARAKNGTGKTGAYSIPVLEQVDPKKDCIQALIIVPTRELALQTSQICIELAKHMKVKVMVTTGGTNLRDDIMRIYQTVHVVIATPGRILDLMDKHVANMEHCRMLVLDEADKLLSQDFKMLDHVIAQLPSERQILLYSATFPLSVKQFMEKHLRKPYEINLMEELTLKGVTQYYAFVQERQKVHCLNTLFSKLQINQSIIFCNSTQRVELLAKKITELGYCCYYIHAKMAQAHRNRVFHDFRAGLCRNLVCSDLFTRGIDVQAVNVVINFDFPKMAETYLHRIGRSGRFGHLGIAINLITYEDRFALHRIEQELGTEIKPIPKVIDPSLYVATKIEDAQGIDESNVSK

>Me31B

MMTEKLNSGHTNLTSKGIINDLQIAGNTSDDMGWKSKLKLPPKDNRFKTTDVTDTRGNEFEEFCLKRELLMGIFEKGWERPSPIQEAAIPIALSGKDVLARAKNGTGKTGAYCIPVLEQIDPTKDYIQALVMVPTRELALQTSQICIELAKHLDIRVMVTTGGTILKDDILRIYQKVQLIIATPGRILDLMDKKVADMSHCRILVLDEADKLLSLDFQGMLDHVILKLPKDPQILLFSATFPLTVKNFMEKHLREPYEINLMEELTLKGVTQYYAFVQERQKVHCLNTLFSKLQINQSIIFCNSTQRVELLAKKITELGYCCYYIHAKMAQAHRNRVFHDFRQGLCRNLVCSDLFTRGIDVQAVNVVINFDFPRMAETYLHRIGRSGRFGHLGIAINLITYEDRFDLHRIEKELGTEIKPIPKVIDPALYVANVGASVGDTCNNSDLNNSANEEGNVSK

>BmDDX6

MMTENRISSSNHVGNSISQTKGEVDKSIDDVGWKSKLKIPPKDRRIKIDVTDTRGNEFEEFCLKRELLMGIFEKGWEKPSPIQEASIPIALSGKDVLARAKNGTGKTGAYCIPVLEQVDPKKDTIQALIVVPTRELALQTSQICIELAKHTDIRVMVTTGGTNLRDDIMRIYQNVQVIIATPGRMIDLMDKQVAKMDQCRMLVLDEADKLLSQDFKGMLDMVISRLPKERQILLFSATFPLSVKQFMEKHLKEPYEINLMEELTLKGVTQYYAFVQERQKVHCLNTLFSKLQINQSIIFCNSTQRVELLAKKITELGYCCYYIHARMAQAHRNRVFHDFRAGLCRNLVCSDLFTRGIDVQAVNVVINFDFPRMAETYLHRIGRSGRFGHLGIAINLITYDDRFALHRIEQELGTEIKPIPKVIDPALYVARPEDEDLGDK

>TcDDX6

MMTDTLNSNHVMALSGKVDIDTKMDDMGWKAKLKIPPKDRRIQTSDVTDTRGNEFEEFCLKRELLMGIFEKGWEKPSPIQEASIPIALSGKDILARAKNGTGKTGAYSIPVLEQIDPKKDCIQALIIVPTRELALQTSQICIELAKHLDVRVMVTTGGTNLRDDIMRIYQKVQVIIATPGRILDLMEKGVAVMDQCKILVLDEADKLLSQDFKGMLDTVIKNLPQERQILLFSATFPLTVEQFMRKHLRDPYEINLMEELTLKGVTQYYAFVQERQKVHCLNTLFSKLQINQSIIFCNSTQRVELLAKKITELGYCCYYIHAKMAQAHRNRVFHDFRAGLCRNLVCSDLFTRGIDVQAVNVVINFDFPKMAETYLHRIGRSGRFGHLGIAINLITYDDRFALHRIEQELGTEIKPIPKVIDPKLYVAKLIDDEDTPEDNAK

>CfDDX6

MIAETHLSSIHMNNLSQNNKGDLEKMDDLGWKSKLKIPPKDRRIKTSDVTDTRGNEFEEFCLKRELLMGIFEKGWEKPSPIQEASIPIALSGKDILARAKNGTGKTGAYCIPVLEQVDPKKDCIQALVIVPTRELALQTSQICIELAKHMEIRVMVTTGGTNLRDDIMRIYQKVQVIIATPGRILDLMDKQVANTEHCRMLVLDEADKLLSQDFKGMLDHVISRLPRERQILLFSATFPLSVKQFMEKHLKEPYEINLMEELTLKGVTQYYAFVQERQKVHCLNTLFSKLQINQSIIFCNSTQRVELLAKKITELGYCCYYIHAKMAQAHRNRVFHDFRSGLCRNLVCSDLFTRGIDVQAVNVVINFDFPKMAETYLHRIGRSGRFGHLGIAINLITYEDRFALHRIESELGTEIKPIPKVIDPSLYVAKLEGDVPIEENVSQ

>AdDDX6

MMTETHINSNHVLNSGLNTKSEIDKMDDVGWKAKLKIPPKDKRIKTSDVTDTRGNEFEEFCLKRELLMGIFEKGWEKPSPIQEASIPIALSGKDILARAKNGTGKTGAYSIPVLEQVDPRKDVIQALVLVPTRELALQTSQICIELAKHMEIKVMVTTGGTDLRDDIMRIYQSVQVIIATPGRILDLMDKNVANMDHCKTLVLDEADKLLSQDFKGMLDHVISRLPHERQILLYSATFPLTVKQFMEKHLRDPYEINLMEELTLKGVTQYYAFVQERQKVHCLNTLFSKLQITQSIIFCNSTQRVELLAKKITDLGYCCYYIHAKMAQAHRNRVFHDFRAGLCRNLVSSDLFTRGIDVQAVNVVINFDFPKMAETYLHRIGRSGRFGHLGIAINLITYEDRFNLHRIEQELGTEIKPIPKVIDPSLYVARPEDNNSMEEGNVSK

>FoDDX6

MTEVHSNHVIGGISMNKDLDDGIGWKSKLKIPPKDQRIKTSDVTDTRGNEFEEFCLKRELLMGIFEKGWEKPSPIQEASIPIALSGKDVLARAKNGTGKTGAYSIPVLEQVDPKKDCIQALVIVPTRELALQTSQICIELAKHMDVKVMVTTGGTNLRDDIMRIYQKVHIIIATPGRILDLMDKNVANMENCRILVLDEADKLLSQDFKGMLDHVISRLPKERQILLYSATFPLTVKQFMEKHLKEPYEINLMEELTLKGVTQYYAFVQERQKVHCLNTLFSKLQINQSIIFCNSTQRVELLAKKITELGYCCYYIHAKMAQAHRNRVFHDFRNGLCRNLVCSDLFTRGIDVQAVNVVINFDFPKMAETYLHRIGRSGRFGHLGIAINLITFDDRFALHRIEQELGTEIKPIPKVIDPALYVAKSLDDSHAIEEANNVSK

>CdDDX6

MGSKLDSDKMDDIGWKSKLKIPPKDKRIKTSDVTDTRGNEFEEFCLKRELLMGIFEKGWEKPSPIQEASIPIALTGKDVLARAKNGTGKTGAYCIPVLENIDSKKETIQALVIVPTRELALQTSQICIELSKHMHIKVMVTTGGTNLRDDIMRIFQKVQVVIATPGRILDLMEKKVANMDNCKVLVLDEADKLLSQDFKGMLDTVIAKLPKERQILLYSATFPLTVKNFMDKHLKSPYEINLMEELTLKGVTQYYAFVQERQKVHCLNTLFSKLQINQSIIFCNSTQRVELLAKKITELGYCCYYIHAKMAQAHRNRVFHDFRAGLCRNLVCSDLFTRGIDVQAVNVVINFDFPKMAETYLHRIGRSGRFGHLGIAINLITYEDRFALHRIEQELGTEIKPIPKVIDPSLYVAKFDEHGDHEDGNTNVAIVISANLVEKSKEGLIEEKKS

>MpDDX6

MLATNHNNSGINLSMPSKQLNRISFEGIDDAGWKAKLKLPPQDHRIKTSDVTSTKGNDFEEFCLKRELLMGIFEKGWEKPSPIQEASIPIALSGKDILARAKNGTGKTGAYSIPVLEQVDPKLDVIQALVIVPTRELALQTSQICIELAKHLDIRVMVTTGGTNLKDDILRIYQRVHVIIATPGRILDLLDKSIAKVDHCRILVLDEADKLLSQDFKGMLDHIISRLPSERQILLYSATFPLTVKQFMDKHLRSPYEINLMEELTLKGVTQYYAFVQEKQKVHCLNTLFSKLQINQSIIFCNSTQRVELLAKKITDLGYCCYYIHAKMAQAHRNRVFHDFRKGSCRNLVCSDLFTRGIDVQAVNVVINFDFPKMAETYLHRIGRSGRFGHLGIAINLITYDDRFALHRIEQELGTEIKPIPKVIDPRLYVARPEDVDINEEMDLSK

>OcDDX6

MSLVDMINSGSKVSGSPNTASLLDKKPDMMGIGMGGGQQQTGDWKKQLKMPPKDRRVKTSDVTDREGKEFEDFCLSRELLMGIFEMGWEKPSPIQEASIPMALSGRDILARAKNGTGKTGAYSIPILERVDPKIDSIQALIIVPTRELALQTSQICTDLSKHLKLKVMVTTGGTGLKDDIIRVYQKVHVIIATPGRIIDLMEKNVCKMDQCKMLVLDEADKLLSQDFNKMLDKLISFLPLKRQILLFSATFPVTVEDFMKKHSIDPYKINLMDELTLKGVTQYYAFVQERQKVHCLNTLFSKLEINQSIIFCNSTQRVELLAKKITELGYACYYIHARMNQADRNRVFHDFRKGHCRNLVCSDLFTRGIDIQAVNVVINFDFPKMSETYLHRIGRSGRFGHLGIAINLITYDDRFTLQKIENELGTEIKPIPKEIDKALYVASYQQEGAHEEEEHNGGQNGVK

>ZnDDX6

MMTETHINSNHVAVMPGLNSINTRKDEGDKVDDIGWKAKLKIPPKDRRVQTSDVTDTRGNEFEEFCLKRELLMGIFEKGWEKPSPIQEASIPIALSGKDVLARAKNGTGKTGAYSIPVLEQVDPKKDCIQGKLSRLFDKRDRHLCTGVHLSVEYGTIISGRDWRSIRVLVERLGGNPIVPLDMPIYFYLKADKLLSQDFKGMLDHVISRLPKERQILLFSATFPLTVKQFMEKHLREPYEINLMEELTLKGVTQYYAFVQERQKVHCLNTLFSKLQINQSIIFCNSTQRVELLAKKITELGYCCYYIHAKMVQAHRNRVFHDFRAGLCRNLVCSDLFTRGIDVQAVNVVINFDFPKMAETYLHRIGRSGRFGHLGIAINLITYEDRFALHRIEQELGTEIKPIPKVIDPSLYVAKLEDSQGIEEANISK

>HsDDX6

MSTARTENPVIMGLSSQNGQLRGPVKPTGGPGGGGTQTQQQMNQLKNTNTINNGTQQQAQSMTTTIKPGDDWKKTLKLPPKDLRIKTSDVTSTKGNEFEDYCLKRELLMGIFEMGWEKPSPIQEESIPIALSGRDILARAKNGTGKSGAYLIPLLERLDLKKDNIQAMVIVPTRELALQVSQICIQVSKHMGGAKVMATTGGTNLRDDIMRLDDTVHVVIATPGRILDLIKKGVAKVDHVQMIVLDEADKLLSQDFVQIMEDIILTLPKNRQILLYSATFPLSVQKFMNSHLQKPYEINLMEELTLKGVTQYYAYVTERQKVHCLNTLFSRLQINQSIIFCNSSQRVELLAKKISQLGYSCFYIHAKMRQEHRNRVFHDFRNGLCRNLVCTDLFTRGIDIQAVNVVINFDFPKLAETYLHRIGRSGRFGHLGLAINLITYDDRFNLKSIEEQLGTEIKPIPSNIDKSLYVAEYHSEPVEDEKP

>BtDDX6

MSTARTENPVIMGLSSQNGQLRGPVKPSGGPGGGGTQTQQQMNQLKNTNTINNGTQQQAQSMTTTIKPGDDWKKTLKLPPKDLRIKTSDVTSTKGNEFEDYCLKRELLMGIFEMGWEKPSPIQEESIPIALSGRDILARAKNGTGKSGAYLIPLLERLDLKKDNIQAMVIVPTRELALQVSQICIQVSKHMGGAKVMATTGGTNLRDDIMRLDDTVHVVIATPGRILDLIKKGVAKVDHVQMIVLDEADKLLSQDFVQIMEDIILTLPKNRQILLYSATFPLSVQKFMNSHLQKPYEINLMEELTLKGVTQYYAYVTERQKVHCLNTLFSRLQINQSIIFCNSSQRVELLAKKISQLGYSCFYIHAKMRQEHRNRVFHDFRNGLCRNLVCTDLFTRGIDIQAVNVVINFDFPKLAETYLHRIGRSGRFGHLGLAINLITYDDRFNLKSIEEQLGTEIKPIPSNIDKSLYVAEYHSEPVEDEKA

>MmDDX6

MSTARTENPVIMGLSSQNGQLRGPVKASAGPGGGGTQPQPQLNQLKNTSTINNGTPQQAQSMAATIKPGDDWKKTLKLPPKDLRIKTSDVTSTKGNEFEDYCLKRELLMGIFEMGWEKPSPIQEESIPIALSGRDILARAKNGTGKSGAYLIPLLERLDLKKDNIQAMVIVPTRELALQVSQICIQVSKHMGGAKVMATTGGTNLRDDIMRLDDTVHVVIATPGRILDLIKKGVAKVDHVQMIVLDEADKLLSQDFVQIMEDIILTLPKNRQILLYSATFPLSVQKFMNSHLQKPYEINLMEELTLKGVTQYYAYVTERQKVHCLNTLFSRLQINQSIIFCNSSQRVELLAKKISQLGYSCFYIHAKMRQEHRNRVFHDFRNGLCRNLVCTDLFTRGIDIQAVNVVINFDFPKLAETYLHRIGRSGRFGHLGLAINLITYDDRFNLKSIEEQLGTEIKPIPSNIDKSLYVAEYHSEPAEDEKP

>GgDDX6

MSTARTENPVIMGLSSQNGQLRGPVKPSGGPGGGGTQTQQQMNQLKNANTINNGTQQQAQSMTTTIKPGDDWKKTLKLPPKDLRIKTSDVTSTKGNEFEDYCLKRELLMGIFEMGWEKPSPIQEESIPIALSGRDILARAKNGTGKSGAYLIPLLERLDLKKDNIQAMVIVPTRELALQVSQICIQVSKHMGGAKVMATTGGTNLRDDIMRLDDTVHVVIATPGRILDLIKKGVAKVEHVQMIVLDEANKLLSQDFVQIMEDIILTLPKNRQILLYSATFPLSVQKFMNSHLQKPYEINLMEELTLKGVTQYYAYVTERQKVHCLNTLFSRLQINQSIIFCNSSQRVELLAKKISQLGYSCFYIHAKMRQEHRNRVFHDFRNGLCRNLVCTDLFTRGIDIQAVNVVINFDFPKLAETYLHRIGRSGRFGHLGLAINLITYDDRFNLKSIEEQLGTEIKPIPSNIDKSLYVAEYHSEPVEDEKQ

>XtDDX6

MSTARTENPVLMGMSSQNGQLRGPLKPSAGPGGGGTQTQQINQLKNASTINSGSQQQAQSMSSVIKPGDDWKKTLKLPPKDLRIKTSDVTSTKGNEFEDYCLKRELLMGIFEMGWEKPSPIQEESIPIALSGRDILARAKNGTGKSGAYLIPLLERLDLKKDCIQAMVIVPTRELALQVSQICIQVSKHMGGVKVMATTGGTNLRDDIMRLDDTVHVVIATPGRILDLIKKGVAKVDHIQMIVLDEADKLLSQDFVQIMEDIIITLPKNRQILLYSATFPLSVQKFMTSHLQKPYEINLMEELTLKGVTQYYAYVTERQKVHCLNTLFSRLQINQSIIFCNSSQRVELLAKKISQLGYSCFYIHAKMRQEHRNRVFHDFRNGLCRNLVCTDLFTRGIDIQAVNVVINFDFPKLAETYLHRIGRSGRFGHLGLAINLITYDDRFNLKSIEEQLGTEIKPIPSSIDKSLYVAEYHSESGEDKP

>DrDDX6

MSTARMENPVILGLSNQNGQMRGSVKPAGGPGGGGGGSQTTQPAQVKASSTVNNGNSQPVPTANTIIKPGDDWKKNLKLPPKDLRMKTSDVTATKGNEFEDYCLKRELLMGIFEMGWEKPSPIQEESIPIALSGRDILARAKNGTGKSGAYLIPLLERIDLKKDSIQAVVIVPTRELALQVSQICIQVSKHMGGVKVMATTGGTNLRDDIMRLDETVHVVIATPGRILDLIKKGVAKVGQVQMIVLDEADKLLSQDFVQMMEEILSSLSKQRQILLYSATFPLSVQKFMNSHLQKPYEINLMEELTLKGVTQYYAYVTERQKVHCLNTLFSRLQINQSIIFCNSSQRVELLAKKISQLGYSCFYIHAKMRQEHRNRVFHDFRNGLCRNLVCTDLFTRGIDIQAVNVVINFDFPKLGETYLHRIGRSGRFGHLGLAINLITYDDRFNLKGIEEQLGTEIKPIPSSIDKSLYVAEYHSESGEEVKL

>Cgh-1

MSGAEQQQIVPANNGDENWKAGLNLPAKDRRFKTADVTDTKGVEFEDFCLGRDLLMGIFEKGWEKPSPIQEASIGVALTGQDILARAKNGTGKTGAYCIPVIEKIQPALKAIQAMVIVPTRELALQTSQICVELSKHIQLKVMVTTGGTDLRDDIMRLNGTVHLVIATPGRILDLMEKGVAKMEHCKTLVLDEADKLLSQDFQGILDRLINFLPKERQVMLYSATFPNTVTSFMQKHMHKPYEINLMEELTLLGVTQYYAFVQEKQKVHCLNTLFRKLQINQSIIFCNSTQRVELLAKKITEIGYSCYYIHSKMAQNHRNRVFHDFRQGNCRNLVCSDLLTRGIDIQAVNVVINFDFPRNAETYLHRIGRSGRFGHLGVAINLITYEDRHTLRRIEQELRTRIEPIPKTVDPKLYVADQQLVDAADETTA

>DHH1

MGSINNNFNTNNNSNTDLDRDWKTALNIPKKDTRPQTDDVLNTKGNTFEDFYLKRELLMGIFEAGFEKPSPIQEEAIPVAITGRDILARAKNGTGKTAAFVIPTLEKVKPKLNKIQALIMVPTRELALQTSQVVRTLGKHCGISCMVTTGGTNLRDDILRLNETVHILVGTPGRVLDLASRKVADLSDCSLFIMDEADKMLSRDFKTIIEQILSFLPPTHQSLLFSATFPLTVKEFMVKHLHKPYEINLMEELTLKGITQYYAFVEERQKLHCLNTLFSKLQINQAIIFCNSTNRVELLAKKITDLGYSCYYSHARMKQQERNKVFHEFRQGKVRTLVCSDLLTRGIDIQAVNVVINFDFPKTAETYLHRIGRSGRFGHLGLAINLINWNDRFNLYKIEQELGTEIAAIPATIDKSLYVAENDETVPVPFPIEQQSYHQQAIPQQQLPSQQQFAIPPQQHHPQFMVPPSHQQQQAYPPPQMPSQQGYPPQQEHFMAMPPGQSQPQY

>SpSte13

MAESLIQKLENANLNDRESFKGQMKAQPVDMRPKTEDVTKTRGTEFEDYYLKRELLMGIFEAGFERPSPIQEESIPIALSGRDILARAKNGTGKTAAFVIPSLEKVDTKKSKIQTLILVPTRELALQTSQVCKTLGKHMNVKVMVTTGGTTLRDDIIRLNDTVHIVVGTPGRVLDLAGKGVADFSECTTFVMDEADKLLSPEFTPIIEQLLSYFPKNRQISLYSATFPLIVKNFMDKHLNKPYEINLMDELTLRGVTQYYAFVDESQKVHCLNTLFSKLQINQSIIFCNSTNRVELLAKKITELGYSCFYSHAKMLQSHRNRVFHNFRNGVCRNLVCSDLLTRGIDIQAVNVVINFDFPKNAETYLHRIGRSGRFGHRGLAISFISWADRFNLYRIENELGTEIQPIPPSIDPSLYVFPNGDYQIPRPLTASADQVLAAQQAKGQEGYHNRPNNNRGGHPRGGGNRGGYRQSNRQPRYRGQQKAD

>KlDDX6

MGSDTSESNNDWKTQLNIPKKDTRPQTDDVLNTKGRSFEDFYLKRELLMGIFEAGFEKPSPIQEEAIPVAIAGKDILARAKNGTGKTAAFVIPTLEKVKPKLNKIQALIMVPTRELALQTSQVVRTLGKHCGISCMVTTGGTNLRDDIMRLNEPVHILVGTPGRVLDLASRRVTDLSECHLFIMDEADKMLSRDFKVLAEQILGFLPERRQLLLFSATFPVTVKEFMVKHLKNPHEINLMDELTLKGISQFYAFVEEKQKLHCLNTLFSKLQINQAIIFCNSTNRVELLAKKITELGFSCYYSHARMKQSERNKVFHEFRQGKVRTLVCSDLLTRGIDIQAVNVVINFDFPKTAETYLHRIGRSGRFGHLGLAINLINWNDRFNLYKIEQELNTEIAPIPSQIDKSLYVAEDSSAVPIPFPLESLPITANAPQQPANAEPLPPQQTQVQFHAPPQQQQQQQQQQQQQQYQQFPNQQQQQYGQPLMPQNYQQQAYPPQPFPSKGFPQQQYTQAPQ

>CrDDX6

MATNEDWKQRLNLPPKDARVRTEDVTNTKGNEFEDYFLKRELLMGIFEKGFEKPSPIQEESIPIALAGRDILARAKNGTGKTAAFCIPVVERVDPTRPVIQALLLVPTRELALQTAQVCKELSKYLSIEVMVTTGGTSLKDDIMRLYQTTHIVVATPGRVVDLASKGVARLNECRMLVMDEADKLLSPEFQPVVEQLIGYLPDDRQIMLYSATFPVTVKAFKEKFLRKPYIINLMEELTLKGVTQFYAFVEEKQKVHCLNTLFSKLRINQSIIFCNSVNRVELLAKKITELGYSCYYIHAKMLQSHRNRVFHDFRNGHCRNLVSSDLFTRGIDIQSVNVVINFDFPKNAETYLHRVGRSGRFGHLGLAVNLITYDDRINLFKIEQELGTEIKPIPAQIEEKLYCI

>PpDDX6

MMATSRAGPAPTAPKASSSSNVSRHNSSNSGNILSSNNNSSVNNNNSQDWKAQLKLPPPDARYKTEDVTATKGNEFEDYFLKRELLMGIYEKGFERPSPIQEESIPIALTGSDILARAKNGTGKTAAFCIPAIEKIDQNKNAVQVLLLVPTRELALQTSQVCKELAKHLNIQIMVTTGGTSLRDDIMRLYQPVHLLVGTPGRVLDLANKGVCNLKECTMLVMDEADKLLSPEFQPLVEQLIGFLPENRQILLYSATFPVTVKSFKDRFLRKPYVINLMDELTLKGITQYYAFVEERQKVHCLNTLFSKLQINQSIIFCNSVNRVELLAKKITELGYSCFYIHAKMLQSHRNRVFHDFRNGACRNLVSSDLFTRGIDIQAVNVVINFDFPKNSETYLHRVGRSGRFGHLGLAVNLITYEDRFNLYRIEQELGTEIKPIPPQIDRGIYCR

>HvDDX6

MAATATRNGLDASKTDDVKAADWKSNLKIPPKDQRIKTADVTATKGNEFEDYCLKRELLMGIFEKGFEKPSPIQEQSIPIALAGRDIMARAKNGTGKTGAYLIPLLERVDSTKDYIQALVLVPTRELALQTSQICKDLSNHLGTKVMVTLGGTSLKDDIMRLYQTVHVVVATPGRILDLMKKGVADMSKCQILVMDEADKLLSMDFKKLLDSLIQMLPENRQVLLYSATFPYSVKEFKDKYLSKPYEINLMDELTLKGITQYYAFVEEKQKVHCLNTLFSKLQVNQSIIFCNSVQRVELLARKITQLGYSCFYIHSRMQQSHRNRVFHDFRSGQCRNLVCSDLFTRGIDIQAVNVVINFDFPKNSETYLHRIGRSGRFGHLGIALNLITYDDRFTLYQVEQELGTEIKPIPPSIDKSLYVAEFQIEQTNNENKDEE

>CgDDX6

MASATIEQNSGNHTNSSDAKTGDSTGWKSKLNLPPKDLRVRTSDVTNTKGNEFEDFCLKRELLMGIFEKGWEKPSPIQEASIPIALTGRDVLARAKNGTGKTGAYTIPILEKADPSRDEVQSLVIVPTRELALQTSQICIEISKHLGLKVMVTTGGTNLKDDIMRLYEPVHVIIATPGRILDLMNKNLVKIGKCGMLVLDEADKLLSQDFKGMLDHIIAHLPPDRQILLYSATFPLTVEQFMRKHLNNPYEINLMDELTLKGVTQYYAFVQEKQKVHCLNTLFSKLQINQSIIFCNSTQRVELLAKKITELGYSCFYIHAKMNQQHRNRVFHDFRQGLCRNLVCSDLFTRGIDIQAVNVVINFDFPKHAETYLHRIGRSGRFGHLGVAINLITYDDRFALHKIESELGTEIKPIPKNIDKSLYVAEFHMMNDHEEGGDTHSQRGGGH

>DjDDX6

MNRNANMNIQGGDSISNVQGDQTLWKNYLNIPEKDNRIKTVDVTATQGNSFDDFCLKRDILKGIYEKGWEMPSPIQEASIPISLIGRDILARAKNGTGKTGAYSIPLLEKIDPSINQIQGIILVPTRELALQTSQICTELAKHTETKIMVTTGGTSLKDDIVRLQQTVHIILATPGRVNDLIKRDIVSTNFCKILVIDEADKLLSADFEEVIDYIISKLPTQRQIMLYSATFPQSVQQFLEKHMRDPYEINLMDELTLKGISQYYAYVQERQKVHCLNTLFSRLQINQSIIFCNTAQRVELLAKKITDLGYSCYYIHAKMNQVYRNRVFHEFRNGNCRNLVCTDLFTRGIDIPSVNVVINFDFPKYAETYLHRIGRSGRYGHLGVAVNLITYDDRFALKNIETQLNTEIKAIPKHIDKRLYVAEYQNENELDTRVRDALARGEGQLLDIENTNEITPPVTNATTVMTTNAKSIQNIKPVTTQSARNDYNVYSQRQPPQQNMRH

>CtDDX6

MATTVEKASKGPLPHNNNHTNNAAADIKAAAAAAASNNETAAGWKTQLKLPPKDNRIQTSDVTSTKGNEFEDYCLRRELLMGIFEKGWEKPSPIQEASIPIALTGRDILARAKNGTGKTGAYAIPLLERVDTSKDYLQGLVMVPTRELALQTSQICIELSKHLGVKCMVTTGGTNLKDDIMRLYQTVHLIIATPGRILDLMNKGLVKTHNCKMLILDEADKLLSQDFKGMLDNIIAHLPRDRQILLYSATFPLTVEQFMRKHLENPYEINLMDELTLKGVTQYYAFVQERQKVHCLNTLFSKLQINQSIIFCNSTQRVELLAKKITELGYSCFYIHARMNQQHRNRVFHDFRQGLCRNLVCSDLFTRGIDIQAVNVVINFDFPKHAETYLHRIGRSGRYGHLGVAINLITYEDRFSLHRIESELGTEIKPIPKIIDKALYVAEYGQLPEDDDEEQAAK

>BpDDX6

MTMDTQNVANEIKENEAGWKTKLSLPAKDRRVKTTDVTATKGHEFEDYCLKRELLMGIFEKGWENPSPIQEASIPIALTGRDILARAKNGTGKTGAYLIPILERIDTTKDKIQALIIVPTRELALQTSQICIEVSKHMGCKVMATTGGTNLKEDIMRLQQTVHVVIATPGRILDLMKKGLAVMDNCNMLVMDEADKLLSQDFKNMLDSVISYLQPDRQILLYSATFPCTVDAFIKKHMHNPYEINLMEELTLKGITQYYAYVQEKQKVHCLNTLFSKLQINQSIIFCNSTQRVELLAKKITELGYSCFYIHAKMRQDHRNRIFHDFRNGACRNLVCSDLFTRGIDIQAVNVVINFDFPKMSETYLHRIGRSGRFGHLGIAINLITYDDRFALHKIEQELGTEIKPIPKVIDPHLYVAEYQTEGSFNAAELKAIDNREYSQENQQQQQQQQQHQMLQQQQQQMHQLVQAQP

>AqDDX6

MAAPAAISSATSHSATENGDWKGKLAIPPKDKRKQTEDVARRKGNEFEDYCLKRDLLMGIFEKGYEAPSPIQEESIPIALAGRDILARAKNGTGKTGAYLIPLLEKIDTDKTHIQSLVIVPTRELALQTSQLCIELGKHMNARVMVTTGGTSLRDDIMRLDETVHIIVATPGRILDLIEKGVAKMGACQILVLDEADKLLSMDYQKTLDRIISRLPSGRQVFLFSATFPISIQGFMDTHMRNPYKINLMDELTLKGVTQYYAYLEERQKVHCLNTLFSKLQINQSIIFCNSVQRVELLAKKITQLGYSCFFMHSKMAQHHRNRVFHDFRNGECRNLVCTDLLTRGIDIQAVNVVINFDFPKTSETYLHRIGRSGRFGHLGLAINLITHDDRFNLFSTEQQLKTEIKPVPKEIDKRLYVAEYQVLNEAPPQTSSSKSEGAKPLKT

>MbDDX6

MSESYTIDVTATRGTEFEDFGLKRELLLGIFEKGFENPSPVQEECIPNALMRRDILARAKNGTGKTGAYLIPTLQLVDPALACIQALILVPTRELALQTASIAKELGKHLNLEIMTTTGGTNTRDDILRLGQTVHVVVATGGRILDLIERGVAQMARCNILVFDEADKLLSEDSLDTVQKIISHLPEKRQTMLLSATYPVAVQGFTKRYLKNPHVINLMETLTLKGVTQFYAFLEEKDKVRCLNTLFGKLQINQSIIFCSSHSRVELLAKKITKLGYSCLYIHSRMEQSHRNTVFDAFRKGKSRHLVCTDLFTRGIDIQAVNVVVNFDFPREAETYLHRIGRSGRYGHLGLAINLVTAGNREALLRIERELGTAIAPMPSVVDQSLYVDSSVSDATAQSS

>MaDDX6

MNNQTKGDVDKTVEDKGWKSKLKIPPKDRRIKTSDVTDTRGNEFEEFCLKRELLMGIFEKGWEKPSPIQEAAIPVALSGKDVLARAKNGTGKTGAYCIPVLEQVDPKKDAIQALVVVPTRELALQTSQICIELAKHTDIRVMVTTGGTNLRDDIMRIYQNVQVIIATPGRMIDLMDKQVAKMDQCRMLVLDEADKLLSQDFKGMLDMVICRLPKERQILLFSATFPLSVKQFMEKHLREPYEINLMEELTLKGVTQYYAFVQERQKVHCLNTLFSKLQINQSIIFCNSTQRVELLAKKITELGYCCYYIHARMAQAHRNRVFHDFRAGLCRNLVCSDLFTRGIDVQAVNVVINFDFPRMAETYLHRIGRSGRFGHLGIAINLITYEDRYTLHRIELELNTEIKPIPKVIDPALYVARADEDDSAEK

>MpoDDX6

MVYSRGAQQHYQPRPLGGGGSHGRGQSGGSYQPYQPHTQRAIHQNNHQQQLQQQQQQQQQQQQQQQQQQQNQQQSQLSGQHLALSQAQANVSTSAAPSADGGQGGHLAQGNSSLQDWKAQLKLPPTDSRYRTEDVTATKGNEFEDYFLKRELLMGIYEKGFERPSPIQEESIPIALTGSDILARAKNGTGKTAAFCIPAIEKIDQNKNAIQVLILVPTRELALQTSQVCKELAKHLHIQVMVTTGGTSLKDDIMRLYQPVHLLVGTPGRVLDLARKGVCNLGECTMLVMDEADKLLSPEFQPLVEQLIAFLPDNRQTLLYSATFPVTVKSFKDRFLRKPYVINLMDELTLKGITQFYAFVEERQKVHCLSTLFSKLQINQSIIFCNSVNRVELLAKKITELGYSCFYVHAKMLQSHRNRVFHDFRNGACRNLVSSDLFTRGIDIQAVNVVINFDFPKNSETYLHRVGRSGRFGHLGLAVNLITYEDRFNLYRIEQELGTEIKPIPPQVDQTIYCR

>SmRH8

MQHYQPRLARPQGRVSQPFAQQQQQSQQQNPPLQNSNALAGGPAGDADGNAKVLASSSLQEWKAQLKTPPADARYRTEDVTATKGNEFEDYFLKRELLMGIYEKGFERPSPIQEESIPIALTGSDILARAKNGTGKTAAFCIPAIEKIDPNKNAIQVLILVPTRELALQTSQVCKELAKHLKIETMVTTGGTSLKDDIMRLYQPVHLLVGTPGRVLDLANKGVCKLKDCSMMVMDEADKLLSPEIQPLVERLLSFLPESRQVLLFSATFPVTVKQFKEKFLRKPYVINLMDELTLKGITQYYAFVEERQKVHCLNTLFSKLQINQSIIFCNSVNRVELLAKKITELGYSCFYIHAKMLQSHRNRVFHDFRNGACRNLVSSDLFTRGIDIQAVNVVINFDFPKNSETYLHRVGRSGRFGHLGLAVNLITYEDRFNLYRIEQELGTEIKPIPPQIDQAIYCR

>AcDDX6

MSTNRNYQYVRRQPTVGAPTNGQSSSRINTPVSSSLATFGNSYDWKSQIKRPPADTRYKTEDVTATKGNEFEDYFLKRELLMGIFEKGFEKPSPIQEESIPIALTGSDILARAKNGTGKTAAFCIPVIEKTDPSKNFIQALILVPTRELAFQTSQVCKELAKYLNIEVMVTTGGTSLKDDIMRLYQPVHILVGTPGRVLDLANKGVCKLRGCSMLVMDEADKLLSPESLPSIIQVISYLPQNRQVLLFSATFPVTVKSFKDKFLQKPYIINLMDELTLKGITQYYAFVEERQKVHCLNTLFSKLQINQSIIFCNSVNRVELLAKKITELGYSCFYIHAKMVQSHRNKVFHDFRNGACRNLVCSDVFTRGIDIQAVNVVINFDFPKNSETYLHRVGRSGRFGHLGLAVNLITYEDRVNFYNIERELGTEIKTIPADIDQAIYCQ

>PsDDX6

MNSRGRYGQGAAVANGRVGQVNSYPKSVQQNFAQRNHQQWVSRVQIQAEKDAAAAKRGNPSEEEKRVQSGDVDPSVQDWKSQLKIPPPDSRYKTEDVTATKGNEFEDYFLKRELLMGIYEKGFERPSPIQEESIPIALTGSDILARAKNGTGKTAAFCIPALEKIDPKKNSIQVLLLVPTRELALQTSQVCKELAKHLKIQIMVTTGGTSLKDDIMRLYQPVHILVATPGRVLDLTKKGVCNLKDCAMLVMDEADKLLSPEFQPLVEQLIGFLPENRQILLYSATFPVTVKSFKDKYLRKPYVINLMDELTLKGITQFYAFVEERQKVHCLNTLFSKLQINQSIIFCNSVNRVELLAKKITELGYSCFYIHAKMLQSHRNRVFHDFRNGACRNLVCSDLFTRGIDIQAVNVVINFDFPKNSETYLHRVGRSGRFGHLGLAVNLITYEDRFNLYKIEQELGTEIQQIPPQIDQTVYCR

>AtrRH8

MNNRRGYLPSYGGTPQRLPQQIVQRGPLQNQQQQQQWLRRDQVEEEKRVTSEDAASSSQDWKAQLKIPPKDSRYKTEDVTATKGNEFEDYFLKRELLMGIYEKGFERPSPIQEESIPIALTGSDILARAKNGTGKTAAFCIPALEKIDQNNNVIQVIILVPTRELALQTAQVCKELGKHLKIQIMVTTGGTSLKDDIMRLYQPVHLLVGTPGRVLDLAKKGICNLKDCTMLVMDEADKLLSQEFQPSVEQLIGFLPPNRQILLFSATFPVTVKEFKDKFLRKSYIINLMDELTLKGITQYYAFVEERQKVHCLNTLFSKLQINQSIIFCNSVNRVELLAKKITELGYSCFYIHAKMLQSHRNRVFHDFRNGACRNLVCSDLFTRGIDIQAVNVVINFDFPRNSETYLHRVGRSGRFGHLGLAVNLITYEDRFSLYRIEQELGTEIKPIPPQIDQAIYCK

>OsRH6

MDPRARYPPGIGNGRGGNPNYYGRGPPPSQHQQHQHQHQQPPHPHHHQYVQRQPQPQQTPHNSQHQQWLRRNQIAAEAAGASEQKAPPVADGIDSSSQDWKAQLKLPPQDTRYRTEDVTATKGNEFEDYFLKRELLMGIYEKGFERPSPIQEESIPIALTGSDILARAKNGTGKTAAFCIPALEKIDQDKNAIQVVILVPTRELALQTSQVCKELGKHLKIQVMVTTGGTSLKDDIVRLYQPVHLLVGTPGRILDLTKKGVCVLKNCSMLVMDEADKLLSPEFQPSIQELIRYLPSNRQILMFSATFPVTVKEFKDKYLPKPYVINLMDELTLKGITQFYAFVEERQKVHCLNTLFSKLQINQSIIFCNSVNRVELLAKKITELGYSCFYIHAKMLQDHRNRVFHDFRNGACRNLVCTDLFTRGIDIQAVNVVINFDFPKSAETYLHRVGRSGRFGHLGLAVNLITYEDRFNLYRIEQELGTEIKPIPPQIDRAIYCQ

>OsRH8

MDPRARYPPGIGNGRGGNPNYYNRGPPLQQQHNHHQQQQTSAPHHQQYVQRQPQQHHHHNHHQQHQQQQQQWLRRNQIAREAAGTDRNSEPKAVAQSPAVDGIDSSSQDWKAQLKLPPQDTRYRTEDVTATKGNEFEDYFLKRELLMGIYEKGFERPSPIQEESIPIALTGSDILARAKNGTGKTAAFCIPALEKIDQEKNAIQVVILVPTRELALQTSQVCKELGKHLKIQVMVTTGGTSLKDDIIRLYQPVHLLVGTPGRILDLTKKGICILKDCSMLIMDEADKLLSPEFQPSVEQLIRYLPASRQILMFSATFPVTVKEFKDKYLPKPYVINLMDELTLKGITQFYAFVEERQKVHCLNTLFSKLQINQSIIFCNSVNRVELLAKKITELGYSCFYIHAKMLQDHRNRVFHDFRNGACRNLVCTDLFTRGIDIQAVNVVINFDFPKTAETYLHRVGRSGRFGHLGLAVNLITYEDRFNLYRIEQELGTEIKPIPPQIDQAIYCQ

>OsRH12

MHHPRARYPPGYTSGGGGGGGGGGGGGRGNGGGGFGGGGGGGGGNHGYYGRGPQPQPQQQHYHHQAQQLHQHQQQQQHAQRNSSSQQQQWLRRDQATAAAASGEVAARTAAQLEAVDSSSEDWKAQLNLPAPDTRYRTEDVTATKGNEFEDYFLKRELLMGIYEKGFERPSPIQEESIPIALTGSDILARAKNGTGKTAAFCIPALEKIDPEKNAIQVVILVPTRELALQTSQVCKELGKYLNIQVMVSTGGTSLKDDIMRLYQPVHLLVGTPGRILDLTRKGICVLKDCSMLVMDEADKLLAPEFQPSIEQLIHFLPANRQLLMFSATFPVTVKDFKEKYLPRPYVINLMDELTLKGITQYYAFVEERQKVHCLNTLFSKLQINQSIIFCNSVNRVELLAKKITELGYSCFYIHAKMLQDHRNRVFHDFRNGACRNLVCTDLFTRGIDIQAVNVVINFDFPKTSETYLHRVGRSGRFGHLGLAVNLITYEDRFNLYRIEQELGTEIKTIPPQIDLAVYCQ

>AtRH6

MNNNNNNRGRFPPGIGAAGPGPDPNFQSRNPNPPQPQQYLQSRTPFPQQPQPQPPQYLQSQSDAQQYVQRGYPQQIQQQQQLQQQQQQQQQQQEQQWSRRAQLPGDPSYIDEVEKTVQSEAISDSNNEDWKATLKLPPRDNRYQTEDVTATKGNEFEDYLLKRDLLRGIYEKGFEKPSPIQEESIPIALTGSDILARAKNGTGKTGAFCIPTLEKIDPENNVIQAVILVPTRELALQTSQVCKELSKYLKIEVMVTTGGTSLRDDIMRLYQPVHLLVGTPGRILDLAKKGVCVLKDCAMLVMDEADKLLSVEFQPSIEELIQFLPESRQILMFSATFPVTVKSFKDRYLKKPYIINLMDQLTLMGVTQYYAFVEERQKVHCLNTLFSKLQINQSIIFCNSVNRVELLAKKITELGYSCFYIHAKMVQDHRNRVFHDFRNGACRNLVCTDLFTRGIDIQAVNVVINFDFPRTSESYLHRVGRSGRFGHLGLAVNLVTYEDRFKMYQTEQELGTEIKPIPSLIDKAIYCQ

>AtRH8

MNNRGRYPPGIGAGRGAFNPNPNYQSRSGYQQHPPPQYVQRGNYAQNHQQQFQQAPSQPHQYQQQQQQQQQWLRRGQIPGGNSNGDAVVEVEKTVQSEVIDPNSEDWKARLKLPAPDTRYRTEDVTATKGNEFEDYFLKRELLMGIYEKGFERPSPIQEESIPIALTGRDILARAKNGTGKTAAFCIPVLEKIDQDNNVIQAVIIVPTRELALQTSQVCKELGKHLKIQVMVTTGGTSLKDDIMRLYQPVHLLVGTPGRILDLTKKGVCVLKDCSVLVMDEADKLLSQEFQPSVEHLISFLPESRQILMFSATFPVTVKDFKDRFLTNPYVINLMDELTLKGITQFYAFVEERQKIHCLNTLFSKLQINQSIIFCNSVNRVELLAKKITELGYSCFYIHAKMLQDHRNRVFHDFRNGACRNLVCTDLFTRGIDIQAVNVVINFDFPKNAETYLHRVGRSGRFGHLGLAVNLITYEDRFNLYRIEQELGTEIKQIPPHIDQAIYCQ

>AtRH12

MNTNRGRYPPGVGTGRGAPPNPNYHQSYRQQQPPQDQQYVQRGYSQNPQQMQLQQQHQQQQQQQQWSKRPQLPENASNANEVVQQTTQPEASSDANGQNWKATLRLPPPDTRYQTADVTATKGNEFENYFLKRDLLKGIYEKGFEKPSPIQEESIPIALTGSDILARAKNGTGKTGAFCIPVLEKIDPNNNVIQAMILVPTRELALQTSQVCKELSKYLNIQVMVTTGGTSLRDDIMRLHQPVHLLVGTPGRILDLTKKGVCVLKDCAMLVMDEADKLLSAEFQPSLEELIQFLPQNRQFLMFSATFPVTVKAFKDRHLRKPYVINLMDQLTLMGVTQYYAFVEERQKVHCLNTLFSKLQINQSIIFCNSVNRVELLAKKITELGYSCFYIHAKMVQDHRNRVFHEFRNGACRNLVCTDLFTRGIDIQAVNVVINFDFPRTSESYLHRVGRSGRFGHLGLAVNLVTYEDRFKMYQTEQELGTEIKPIPSNIDQAIYCQ

>McDDX6

MAYQMPPQGYGMPPQGYPQQGYPPQGYQQMPPQGYQQMPPQGYPQQGYPPQQMMQPPPQQQQQGSWKDQLALPPRDERYRTEDVTATKGNEFEDYFLKRELLMGIFEKGFERPSPIQEESIPIALTGRDILARAKNGTGKTAAFTIPVLEKTDTSKNVIQAVLLVPTRELALQTSQVCKELGKHLNVQVMVTTGGTSLKDDIMRLHQPVHIVVATPGRLVDLASKGVAKLNQCTMLAMDEADKLLSPEFQPVIAQLIDFLPRNRQILLYSATFPVTVKSFKEKYLRKPFVINLMEELTLKGITQYYAFVEEKQKVHCLNTLFSKLQINQSIIFCNSVNRVELLAKKITELGYSCFYIHAKMMQSHRNRVFHDFRNGACRNLVSSDLFTRGIDIQSVNVVINFDFPKNSETYLHRVGRSGRFGHLGISVNLITYDDRFNLFRIEKELGTEIQQIPPTIDPAVYCR

>CsRH8

MSAVAAPGADDWKSKLALPAKDNRYKTEDVTATKGNSFEDYFLKRELLMGIYEKGFEAPSPIQEESIPIALTGRDILARAKNGTGKTAAFCIPVLEKVDTSKNEVQALLLVPTRELALQTSQVAKELGKHMGVEVMVSTGGTSLRDDIVRLGATVHVVVATPGRILDLAQKGVAKLDKCGVCVMDEADKLLSPEFQPVVEQLISFLPQNRQICLYSATFPVTVKQFKDKFLRKPYIINLMEELTLKGVTQYYAFVEERQKVHCLNTLFAKLSINQSIIFCNSVNRVELLAKKITELGYSCFYIHAKMLQSHRNRVFHDFRNGNCRNLVSSDLFTRGIDIQAVNVVINFDFPKNSETYLHRVGRSGRFGHLGIAINLITYEDRFNLYKIEQELGTEIKPIPPVIEKSLYCA

>GcRH8

MAAAPAPDDWRSLVKPPPKDTRFRTSDVTATKGNEFEDYYLKRELLMGIYEMGFEQPSPIQEAAIPVSLAGRDILARAKNGTGKTGAYLIPILERVDLKQTVASALVLVPTRELALQTAQVCKRLARHIDGLQVMVSTGGTSLKDDIMRFYQPVHVLVATPGRVLDMTRKNVCKLDRCSMLVMDEADKLLSPEFSHVLEQIISNFMPPDRQISLFSATFPITVKGFRDRYLRKPYEINLMDELTLQGITQYYAFVEESKKVHCLNTLFSKLQINQSIIFCNSVNRVELLARKITDLGYSCFYIHARMPQEQRNRVFHDFRSGACRNLVSSDLFTRGIDIQAVNVVINFDFPKSSETYLHRVGRSGRFGHRGLAISLITYEDRFNLYRIEQELDTEISPIPPLVDKALYCV

>PuDDX6

MIVAGQPQGRPTRPRDDRIKTEDVTATKGNEFADYYLKQDLLLGIFDEGFEKPSPIQEEAIPVALVGRDILARAKNGTGKTGAFLIPILERVDVSQDTPGAVVLVPTRELALQTAQVAKKLGRHLKGLEVMVSTGGTALKDDIMRLYQPVHVLVATPGRVLDLAEKGVAKLSGCKLLVLDEADKLLSREFTPLVEAIITRHLPKERQIMLFSATFPVAVKGFRDRFQRKAHEINLMDELTLRGITQFYAFVEESKKVHCLNTLFSKLQINQSIIFCNSVTRVELLAKKITELGYSCFYIHAKMPQSHRNRVFHDFRSGACRNLVSSDLFTRGIDIQAVNVVINFDFPKSSETYLHRVGRSGRFGHRGLAISMITFEDRFNLYRIEQELGTEIGPIPPSVDKDLYCA

>DBP2

MTYGGRDQQYNKTNYKSRGGDFRGGRNSDRNSYNDRPQGGNYRGGFGGRSNYNQPQELIKPNWDEELPKLPTFEKNFYVEHESVRDRSDSEIAQFRKENEMTISGHDIPKPITTFDEAGFPDYVLNEVKAEGFDKPTGIQCQGWPMALSGRDMVGIAATGSGKTLSYCLPGIVHINAQPLLAPGDGPIVLVLAPTRELAVQIQTECSKFGHSSRIRNTCVYGGVPKSQQIRDLSRGSEIVIATPGRLIDMLEIGKTNLKRVTYLVLDEADRMLDMGFEPQIRKIVDQIRPDRQTLMWSATWPKEVKQLAADYLNDPIQVQVGSLELSASHNITQIVEVVSDFEKRDRLNKYLETASQDNEYKTLIFASTKRMCDDITKYLREDGWPALAIHGDKDQRERDWVLQEFRNGRSPIMVATDVAARGIDVKGINYVINYDMPGNIEDYVHRIGRTGRAGATGTAISFFTEQNKGLGAKLISIMREANQNIPPELLKYDRRSYGGGHPRYGGGRGGRGGYGRRGGYGGGRGGYGGNRQRDGGWGNRGRSNY

>HsDDX39A

MAEQDVENDLLDYDEEEEPQAPQESTPAPPKKDIKGSYVSIHSSGFRDFLLKPELLRAIVDCGFEHPSEVQHECIPQAILGMDVLCQAKSGMGKTAVFVLATLQQIEPVNGQVTVLVMCHTRELAFQISKEYERFSKYMPSVKVSVFFGGLSIKKDEEVLKKNCPHVVVGTPGRILALVRNRSFSLKNVKHFVLDECDKMLEQLDMRRDVQEIFRLTPHEKQCMMFSATLSKDIRPVCRKFMQDPMEVFVDDETKLTLHGLQQYYVKLKDSEKNRKLFDLLDVLEFNQVIIFVKSVQRCMALAQLLVEQNFPAIAIHRGMAQEERLSRYQQFKDFQRRILVATNLFGRGMDIERVNIVFNYDMPEDSDTYLHRVARAGRFGTKGLAITFVSDENDAKILNDVQDRFEVNVAELPEEIDISTYIEQSR
