# Supplementary material for: DDX6 Is Essential for Oocyte Development and Maturation in Locusta migratoria
Source: Insects. 2021 Jan 14;12(1):70. doi: 10.3390/insects12010070 (PMC7830464; doi:10.3390/insects12010070)
Supplement: Supplementary file 1 [file insects-12-00070-s001.zip › Supplementary material/Supplemental file 2.docx]

**27 sequences of DDX6 from algae**

>XP_005646927.1 cytoplasmic DExD/H-box RNA helicase [Coccomyxa subellipsoidea C-169]

MAQPAQAMEAPPAPQNWKSALKLPPKDARIRTEDVTNTKGNEFEDYVLKRELLMGIYEKGFEKPSPIQEESIPIALTGRD

ILARAKNGTGKTAAFTIPLLERIDTSKNEIQAMILVPTRELALQTSQVCKELGKHLGVEVMVTTGGTSLRDDIMRLHAVV

HIMVATPGRILDLSSKGVAKLNKCKMLVMDEADKLLSPEFQPIIEQLISFLAAERQICLYSATFPVTVKQFKEKFLKKPY

IINLMEELTLKGITQYYAFVEEKQKVHCLNTLFSKLQINQSIIFCNSVNRVELLAKKITELGYSCFYIHAKMLQSHRNRV

FHDFRNGNCRNLVSSDLFTRGIDIQAVNVVINFDFPKNSETYLHRVGRSGRFGHLGLAVNLITYEDRFNLFRIEQELGTE

IKPIPPVIEKTLYCV

>XP_002951166.1 DEAD-box RNA helicase, ATP-dependent, cytoplasmic [Volvox carteri f. nagariensis]

MATSGEDWKTRLNLPPKDARVRTEDVTATKGNEFEDYFLKRELLMGIFEKGFEKPSPIQEESIPIALAGRDILARAKNGT

GKTAAFCIPVLERVDPTRSVIQALLLVPTRELALQTAQVCKELAKYLNVEVMVTTGGTSLKDDIMRLYQTTHIVVATPGR

VVDLAGKGVARLNECKMLVMDEADKLLSPEFQPVVEQLIGFLPEDRQIMLYSATFPVTVKAFKEKFLRKPYIINLMEELT

LKGVTQYYAFVEEKQKVHCLNTLFSKLRINQSIIFCNSVNRVELLAKKITELGYSCYYIHAKMLQSHRNRVFHDFRNGHC

RNLVSSDLFTRGIDIQSVNVVINFDFPKNAETYLHRVGRSGRFGHLGLAVNLITYDDRINLFKIEQELGTEIKPIPAQIE

EKLYCI

>KAA6423055.1 DEAD-box ATP-dependent RNA helicase [Trebouxia sp. A1-2]

MAQPAQSDADWKKSLVLPPQDKRKQTEDVTATKGNDFEDYFLKRELLQGIYEKGFEKPSPIQEESIPIALTGRDILARAK

NGTGKTAAFSIPILERIDVTKNVIQAMILVPTRELALQTSQVCKELGKHLGVEVMVTTGGTSLRDDILRLHNVVHIIVAT

PGRILDLSDKGVAKLDRCKMLAMDEADKLLSPEFQPIIEQLIGFLPQDKQILLYSATFPVTVKQFRDKFLRKPYVVNLME

ELTLKGVTQYYAFVEERQKVHCLNTLFSKLQINQSIIFCNSVNRVELLSKKITELGYSCFYIHAKMLQSHRNRVFHDFRN

GNCRNLVSSDLFTRGIDIQAVNVVINFDFPKNSETYLHRVGRSGRFGHLGLAVNLITYEDRFNLYRIEQELGTEIKPIPP

IIEKDLYCS

>KXZ50469.1 hypothetical protein GPECTOR_16g643 [Gonium pectorale]

MAATQSGTEDWKSRLALPPKDARVRTEDVTATKGNEFEDYFLKRELLMGIFEKGFEKPSPIQEESIPIALAGRDILARAK

NGTGKTAAFCIPVLERVDPTRPVIQALLLVPTRELALQTAQVCKELAKYLNIEVMVTTGGTSLKDDIMRLYQTTHIVVAT

PGRVVDLAGKGVARLNECKMLVMDEADKLLSPEFQPVVEQLISFLPEDRQIMLYSATFPVTVKAFKEKFLKKPYIINLME

ELTLKGVTQYYAFVEEKQKVHCLNTLFSKLRINQSIIFCNSVNRVELLAKKITELGYSCYYIHAKMLQSHRNRVFHDFRN

GHCRNLVSSDLFTRGIDIQSVNVVINFDFPKNAETYLHRVGRSGRFGHLGLAVNLITYEDRINLFKIEQELGTEIKPIPA

QIEEKLYCI

>GBF97454.1 DEAD-box ATP-dependent RNA helicase [Raphidocelis subcapitata]

MAQAAAPQATEEWKQQLKIPPRDTRIRTADVTATKGHDFEDYFLKRELLMGIYEKGFEKPSPIQEESIPIALTGRDILAR

AKNGTGKTAAFSIPVLEKVDPTKNEVQAMLLVPTRELALQTAQVCKELGKHLAVDVMVTTGGTSLKDDIMRLYQPVHVVV

ATPGRILDLANKGVCKLNNCRCLVMDEADKLLSPEFQPLIEQLISFLPEDRQIMLFSATFPVTVKQFKEKFLRKPYIINL

MDELTLKGVTQYYAFVEERQKVHCLNTLFSKLRINQSIIFCNSVNRVELLAKKITELGYSCFYIHAKMLQSHRNRVFHDF

RNGLCRNLVSSDLFTRGIDIQSVNVVINFDFPKNSETYLHRVGRSGRFGHLGLAVNLITYDDRINLYRIEQELGTEIRPI

PPHIEEKLYCA

>PNH09115.1 DEAD-box ATP-dependent RNA helicase 8 [Tetrabaena socialis]

MAAQTEEWKNRLALPPKDNRVRTEDVTATKGNEFEDYFLKRELLMGIFEKGFEKPSPIQEESIPIALAGRDILARAKNGT

GKTAAFCIPVLERVDHTRPVIQALLLVPTRELALQTAQVCKELAKYLSIEVMVTTGGTSLKDDIMRLYQTTHIVVATPGR

VVDLAGKGVARLNECRMLVMDEADKLLSPEFQPVVEQLISFLPDDRQIMLYSATFPVTVKAFKEKFLRKPYIINLMEELT

LKGVTQYYAFVEEKQKVHCLNTLFSKLRINQSIIFCNSVNRVELLAKKITELGYSCYYIHAKMLQSHRNRVFHDFRNGHC

RNLVSSDLFTRGIDIQSVNVVINFDFPKNAETYLHRVGRSGRFGHLGLAVNLITYEDRINLFKIEQELGTEIKPIPAQIE

EKLYCI

>XP_001692202.1 cytoplasmic DExD/H-box RNA helicase [Chlamydomonas reinhardtii]

MATNEDWKQRLNLPPKDARVRTEDVTNTKGNEFEDYFLKRELLMGIFEKGFEKPSPIQEESIPIALAGRDILARAKNGTG

KTAAFCIPVVERVDPTRPVIQALLLVPTRELALQTAQVCKELSKYLSIEVMVTTGGTSLKDDIMRLYQTTHIVVATPGRV

VDLASKGVARLNECRMLVMDEADKLLSPEFQPVVEQLIGYLPDDRQIMLYSATFPVTVKAFKEKFLRKPYIINLMEELTL

KGVTQFYAFVEEKQKVHCLNTLFSKLRINQSIIFCNSVNRVELLAKKITELGYSCYYIHAKMLQSHRNRVFHDFRNGHCR

NLVSSDLFTRGIDIQSVNVVINFDFPKNAETYLHRVGRSGRFGHLGLAVNLITYDDRINLFKIEQELGTEIKPIPAQIEE

KLYCI

>PSC67841.1 hypothetical protein C2E20_8521 [Micractinium conductrix]

MAAAPQQAGEDWKSRLALPPKDSRFKTEDVTATKGNSFEDYFLKRELLMGIYEKGFENPSPIQEESIPIALTGRDILARA

KNGTGKTAAFCIPVLEKVDTSKNEVQALLLVPTRELALQTSQVCKELGKHMGVEVMVSTGGTSLRDDIVRLGATVHVIVA

TPGRILDLAQKGVAKLDKCSVCVMDEADKLLSPEFQPVVEQLIAFLSQNRQICLYSATFPVTVKQFKDKFLRKPYIINLM

EELTLKGVSQYYAFVEERQKVHCLNTLFAKLSINQSIIFCNSVNRVELLAKKITELGYSCFYIHAKMLQSHRNRVFHDFR

NGNCRNLVSSDLFTRGIDIQAVNVVINFDFPKNSETYLHRVGRSGRFGHLGIAINLITYEDRFNLYKIEQELGTEIKPIP

PIIEKGLYCA

>PRW59022.1 DEAD-box ATP-dependent RNA helicase 8 [Chlorella sorokiniana]

MSAVAAPGADDWKSKLALPAKDNRYKTEDVTATKGNSFEDYFLKRELLMGIYEKGFEAPSPIQEESIPIALTGRDILARA

KNGTGKTAAFCIPVLEKVDTSKNEVQALLLVPTRELALQTSQVAKELGKHMGVEVMVSTGGTSLRDDIVRLGATVHVVVA

TPGRILDLAQKGVAKLDKCGVCVMDEADKLLSPEFQPVVEQLISFLPQNRQICLYSATFPVTVKQFKDKFLRKPYIINLM

EELTLKGVTQYYAFVEERQKVHCLNTLFAKLSINQSIIFCNSVNRVELLAKKITELGYSCFYIHAKMLQSHRNRVFHDFR

NGNCRNLVSSDLFTRGIDIQAVNVVINFDFPKNSETYLHRVGRSGRFGHLGIAINLITYEDRFNLYKIEQELGTEIKPIP

PVIEKSLYCA

>XP_002508100.1 DEAD/DEAH box helicase [Micromonas commoda]

MAYQMPPQGYGMPPQGYPQQGYPPQGYQQMPPQGYQQMPPQGYPQQGYPPQQMMQPPPQQQQQGSWKDQLALPPRDERYR

TEDVTATKGNEFEDYFLKRELLMGIFEKGFERPSPIQEESIPIALTGRDILARAKNGTGKTAAFTIPVLEKTDTSKNVIQ

AVLLVPTRELALQTSQVCKELGKHLNVQVMVTTGGTSLKDDIMRLHQPVHIVVATPGRLVDLASKGVAKLNQCTMLAMDE

ADKLLSPEFQPVIAQLIDFLPRNRQILLYSATFPVTVKSFKEKYLRKPFVINLMEELTLKGITQYYAFVEEKQKVHCLNT

LFSKLQINQSIIFCNSVNRVELLAKKITELGYSCFYIHAKMMQSHRNRVFHDFRNGACRNLVSSDLFTRGIDIQSVNVVI

NFDFPKNSETYLHRVGRSGRFGHLGISVNLITYDDRFNLFRIEKELGTEIQQIPPTIDPAVYCR

>GFH17930.1 DEAD-box ATP-dependent RNA helicase [Haematococcus lacustris]

MVEDAGEHWKEKLNLPAKDLRVRTLDVTNTKGNDFEDYFLKRQLLMGIFEKGFDKPSPIQEESIPIALAGRDVLARAKNG

TGKTAAFAVPVLEKIDTSKNEIQAMILVPTRELALQTAQVCKELGKYLAVDVMVTTGGTSLKDDIMRLNQTIHIVVATPG

RILDLASKGIAKLNNCRVLVMDEADKLLSFEFQPIIEQLINFLPGDRQIMLFSATFPVTVKDFKEKFLKKPYIINLMDEL

TLKGLSQYYAFVEEKQKVHCLNTLFSKLRINQSIIFCNSVNRVELLAKKITELGYSCFYIHAKMLQSHRNRVFHDFRNGS

CRNLVSSDLFTRGIDIQSVNVVINFDFPKNAETYLHRVGRSGRFGHLGLAVNLITYEDRLNLYRIEQELGTEIKPIPSSI

DERLYCV

>GAX73138.1 hypothetical protein CEUSTIGMA_g591.t1 [Chlamydomonas eustigma]

MAQSSTAVAQRGEDAEWKGKLALPPKDTRIRTEDVTATKGCQFEDYFLKRELLMGIFEKGFEAPSPIQEEAIPIALAGRD

ILARAKNGTGKTAAFCIPVLERIDPSKNEIQALLLVPTRELALQTAQVCKELGKYLGIDVVVTTGGTSLKDDIMRFHSSV

HVVVATPGRILDLAQKNVAKLGTCKVLVMDEADKLLSEDFQKIVEELISQLPEERQIMLFSATFPVTVKQFKEKFLKKQY

VINLMDELTLKGVTQYYAFVEEKQKVHCLNTLFSKLSINQSIIFCNSVNRVELLAKKITELGYSCFYIHAKMLQSHRNRV

FHDFRNGHCRNLVSSDLFTRGIDIQSVNVVINFDFPKNSETYLHRVGRSGRFGHLGLAVNLITYDDRLNLYKIEQELGTE

IKAIPPQIEERLYCS

>XP_003079824.1 Helicase, C-terminal [Ostreococcus tauri]

MQGYGYGHPPQGYAQAPAPAQQQNGNWKDTLAIPAKDTRYRTEDVTATKGNEFEDYFLKRELLMGIFEKGFERPSPIQEE

SIPIALTGRDILARAKNGTGKTAAFSIPILEKVDTSKNIIQAVILVPTRELALQTSQVCKELGKHLGVAVMVTTGGTSLK

DDIMRLHQPVHVVVATPGRLVDLASKGVAKLGQATMLAMDEADKLLSPEFQPVIGQVINFMAKNRQILLYSATFPVTVKD

FKDKYLRKPYVINLMEELTLKGITQYYAFVEERQKVHCLNTLFSKLQINQSIIFCNSVTRVELLARKITELGYSCFYIHA

KMIQAHRNRVFHDFRNGACRNLVSSDLFTRGIDIQSVNVVINFDFPKNSETYLHRVGRSGRFGHLGIAVNLITYDDRMNL

FRIEQELGTEIQQIPSQIDPSKYCR

>ALM54987.1 DEAD box RNA helicase CiRH12, partial [Chlamydomonas sp. ICE-L]

SRNKLAAPPRDTRTRTEDVTNTKGNDFEDYFLKRELLMGIFEKGFEKPSPIQEESIPIALAGRDILARAKNGTGKTAAFS

IPVIEKIDTSKNEIQALLLVPTRELALQTAQVCKELGKYMGVEVMVTTGGTSLKDDIMRLYQTIHIIVATPGRILDLGNK

GVAKLGNCKVLVMDEADKLLSIEFEPIVEQLINFLHPERQIMLFSATFPVTVKQFKERWLKKPYIINLMEDLTLKGITQY

YAFVEEKQKVHCLNTLFSKLRINQSIIFCNSVNRVELLAKKITELGYSCFYIHAKMLQSHRNRVFHDFRNGLCRNLVSSD

LFTRGIDIQSVNVVINFDFPKNSETYLHRVGRSGRFGHLGLAVNLITFDDRMNLYRIEQELGTEIKPIPPSIEERLYCT

>XP_001418212.1 predicted protein [Ostreococcus lucimarinus CCE9901]

MGWNERTRRAPPPQPQQNGNWKDTLALPPRDERYRTEDVTATKGNEFEDYFLKRELLMGIFEKGFERPSPIQEESIPIAL

TGRDILARAKNGTGKTAAFTIPILEKVDTSKNIIQAVILVPTRELALQTSQVAKELGKHLGVAVMVTTGGTSLKDDIMRL

HQPVHIVVATPGRLVDLASKSVAKLGQATMLAMDEADKLLSPEFQPVIAQVINFMAKNRQILLYSATFPVTVKDFKDKYL

RKPYVINLMEELTLKGITQYYAFVEEKQKVHCLNTLFSKLQINQSIIFCNSVTRVELLARKITELGYSCFYIHAKMIQAH

RNRVFHDFRNGACRNLVSSDLFTRGIDIQSVNVVINFDFPKNSETYLHRVGRSGRFGHLGIAVNLITYDDRMNLFRIEQE

LGTEIQQIPSQIDPSKYCR

>QDZ22222.1 DEAD-box ATP-dependent RNA helicase [Chloropicon primus]

MAHNMIPPQVAMTPPPQPQTYANASSQQQQQQQQQQQQAAALYQQPMGAMQQQHPMYAMPMGYNPAMMMPNYYMQQGMQR

GMQMPYGMQPPPGSNPAAQAGYQQQQMGHQQGMPPPQAPQQQQQQQQGAANPGAVVPSNGNGEGWKNGLRLPPKDDRVRT

SDVTNTKGCEFEDFYLKRELLMGIYEKGFDKPSPIQEESIPIALTGRDVLARAKNGTGKTASFIIPILERVKTTKNCIQA

LILVPTRELALQTSQVCKEIGKNLTGCQIMVSTGGTTLKDDIMRLHNPVHIVVATPGRILDLAEKGVARLGNCEMFVLDE

ADKLLSVEFEPVIEKLIRMCSETRQIMLFSATFPVTVKAFKEKWLKRPYVINKMDELTLKGVTQYYAFVEEKQKLHCLNT

VFSKLDVNQSIIFCNSVNRVELLAKKITEMGYSCFYIHARMLQSHRNRVFHDFRNGACRNLVSTDLFTRGIDVQAVNVVI

NFDFPKTSETYLHRVGRSGRFGHLGLAINLITYEDRHNLFRIEKELGTEIKSIPPQIEKSLYCA

>KAF6260087.1 cytoplasmic DExD/H-box RNA helicase [Scenedesmus sp. NREL 46B-D3]

MAQAAPNAAAAEGGDWKARLNLPPKDTRIRTELLMGIYEKGFEKPSPIQEESIPIALTGRDILARAKNGTGKTAAFSIPV

LEKIDTTKAEIQAMLLVPTRELALQTAQVCRELGKHTNVEVMVTTGGTSLKDDIMRLYQTIHVIVATPGRILDLSNKGVC

KLHNCRILVMDEADKLLSPEFQPLIEQLISFLPEDRQIMLFSATFPVTVKQFKEKFLRKPYIINLMDELTLKGVTQYYAF

VEERQKVHCLNTLFSKLRINQSIIFCNSVNRVELLAKKITELGYSCFYIHAKMLQSHRNRVFHDFRNGLCRNLVSSDLFT

RGIDIQSVNVVINFDFPKNSETYLHRVGRSGRFGHLGLAVNLITYDDRINLYKIEQELGTEIKPIPPHIEEKLYCM

>GFH25665.1 DEAD box RNA helicase CiRH12, partial [Haematococcus lacustris]

PSDPYRGKRALSILDGSAHFTAFANAATLLELSPQDVTNTKGNDFEDYFLKRQLLMGIFEKGFEKPSPIQEESIPIALAG

RDVLARAKNGTGKTAAFSIPMLERVDTSKNEIQGKCRLCWSRAKQHDWLSNDAATHMLAPLCAAMVLVPTRELALQTAQV

CKELGKYLEVEVMVTTGGTSLKDDIMRLYQTIHIVVATPGRILDLASKGIAKLNKCKVLIMDEADKLLSFEFQPIIEQLI

NFLPEERQIMLYSATFPVTVKDFKEKFLKKPYIINLMDELTLKGLSQYYAFVEEKQKVHCLNTLFSKLRINQSIIFCNSV

NRVELLAKKITELGYSCFYIHAKMLQSHRNRVFHDFRNGLCRNLVSSDLFTRGIDIQSVNVVINFDFPKNAETYLHRVGR

SGRFGHLGLAVNLITYEDRLNLYRIEQELGTEIKPIPAAIDERLYCV

>KAF5832381.1 DEAD-box RNA helicase, ATP-dependent, cytoplasmic [Dunaliella salina]

MADDDWKQKLHLPAKDTRTRTEDVTATKGNDFEDYFLKRELLMGIFEKGFEKPSPIQEESIPIALVGRDVLARAKNGTGK

TAAFCIPVLEKVDTTRNAIQALILVPTRELALQTAQVCKEMGKYLNVEVMVTTGGTSLKDDIMRLYSTIHIVVATPGRIL

DLANKNVAKLNDCRVLCMDEADKLLSPEFEPIIEQLIGFLPRDRQIMLYSATFPVTVKHFKERWLNKPYIVNLMDELTLK

GITQYYAFVEEKQKVHCLNTLFSKLRINQSIIFCNSVNRVELLAKKITELGYSCFYIHAKMLQSHRNRVFHDFRNGLCRN

LVSSDLFTRGIDIQSVNVVINFDFPKNAETYLHRVGRSGRFGHLGLAVNLITYEDRLNL

>KDD74992.1 DEAD/DEAH box helicase [Helicosporidium sp. ATCC 50920]

MGRRSELLMGIYEKGFENPSPIQEESIPIALTGRDILARAKNGTGKTAAFCIPVLERVDTARNEIQALLLVPTRELALQT

SQVCKELGKHLGVEVMVSTGGTSLRDDIVRLGATVHVVVATPGRILDLAQKGVAKLGSATMVVMDEADKLLSQDFEPVVE

ALLGLLPEQRQICLYSATFPVTVKAFKDRYLRRPYIINLMEELTLKGITQYYAFVEERQKVHCLNTLFAKLSINQSIIFC

NSVHRVELLAKKVTELGYSCFYIHAKMVQSHRNRVFHDFRNGNCRNLVSSDLFTRGIDIQAVNVVINFDFPKSSETYLHR

VGRSGRFGHLGLAVNLITYDDRFNLYRIEQELGTEIKPIPPVIEKSLYCV

>XP_003062040.1 predicted protein [Micromonas pusilla CCMP1545]

MGIFEKGFERPSPIQEESIPIALTGRDILARAKNGTGKTAAFTIPVLEKVDVSKKIIQAILLVPTRELALQTSQVAKELG

KHLDVQVMVTTGGTSLKDDIMRLHQPVHIVVATPGRLVDLASKNVAKLNNATMLVMDEADKLLSPEFQPVIAQLIDFLPR

NRQILLYSATFPVTVKEFKDRYLRKPYVINLMEELTLKGITQYYAFVEEKQKVHCLNTLFSKLQINQSIIFCNSVNRVEL

LAKKITELGYSCFYIHAKMLQSHRNRVFHDFRNGACRNLVSSDLFTRGIDIQSVNVVINFDFPKNSETYLHRVGRSGRFG

HLGISVNLITYDDRFNLFRIEQELGTEIQQIPPVIDPSVYCR

>XP_007513343.1 predicted protein [Bathycoccus prasinos]

MGIFEKGFERPSPIQEESIPIALTGRDILARAKNGTGKTAAFTIPILEKTDATKNIIQAVILVPTRELALQTAQVCKELG

KHLGTQVMVTTGGTSLKDDIMRLHQTVHVVVATPGRLVDLAGKGVAKLSNVTMLAMDEADKLLAPEFEPVIAQVIDFCAK

NRQILLYSATFPVTVKSFKDKWLRKPYVINLMEELTLKGITQYYAFVEERQKVHCLNTLFSKLQINQSMIFCNSVNRVEL

LARKITELGYSCFYIHAKMQQGDRNKVFHDFRAGSCRNLVSSDLFTRGIDIQSVNVVINFDFPKSGETYLHRVGRSGRFG

HLGIAVNLVTYEDRFNLFRIEQELGTEIQQIPQNIDPAKYCR

>RMZ52831.1 hypothetical protein APUTEX25_000950, partial [Auxenochlorella protothecoides]

MSPPPPRSPRELLMGIYEKGFEAPSPIQEESIPVALAGRDILARAKNGTGKTAAFCIPVLERIDTSRNEIQALLLVPTRE

LALQTSQVCKELGRHMGVEVMVATGGTNLREDIMRLGATVHVVVATPGRILDLASKGVARLGAAGVVVMDEADKLLSPEF

QPVIESLLGFMPPQRQVCLYSATFPVTVKAFKDRFLRKPYIINLMEELTLKGITQYYAFVEERQKVHCLNTLFNKLQINQ

SIIFCNSVNRVELLAKKITELGYSCFYIHAKMLQSHRNRVFHDFRNGNCRNLVSSDLFTRGIDIQAVNVVINFDFPKNAE

TYLHRVGRSGRFGHLGLAINLITYDDRFSLCVERNRGRG

>XP_013896709.1 ATP-dependent RNA helicase DDX6/DHH1 [Monoraphidium neglectum]

MAVAPASEDWKQSLKIPEKDLRIRTADVTATKGHEFEDYFLKRELLMGIYEKGFEKPSPIQEESIPIALTGRDILARAKN

GTGKTAAFSIPVLEKIDPTKNEVQAMLLVPTRELALQTAQVCKELGKHLAVDVMVTTGGTSLKDDIMRLYQPVHVVVATP

GRILDLASKGVCKLNNCRVLVMDEADKLLSPEFQPLIEQLISFLPDDRQIMLFSATFPVTVKQFKEKFLRKPYIINLMDE

LTLKGVTQYYAFVEERQKVHCLNTLFSKLRINQSIIFCNSAHRNRVFHDFRNGLCRNLVSSDLFTRGIDIQSVNVVINFD

FPKNSETYLHRELGTEIKPIPPHIEEKLYCA

>XP_005849570.1 hypothetical protein CHLNCDRAFT_56077 [Chlorella variabilis]

MAAATAAAAPPADSDWKKSLALPPKDARYKTEDVTATKGNSFEDYFLKRELLMGIYEKGFENPSPIQEESIPIALTGRDI

LARAKNGTGKTAAFCIPVLEKVDTSKNEVQALLLVPTRELALQTSQVAKELGKHMAVEVMVSTGGTSLRDDIVRLGATVH

VIVATPGRILDLAQKGVAKLDKCAVCVMDEADKLLSPEFQPVVEQLIGFLSQNRQICLYSATFPVTVKQFKDKFLRKPYI

INLMEELTLKGVSQYYAFVEERQKVHCLNTLFAKLSINQSIIFCNSVNRVVVARWMGGDTARMPPAPHIAPPNPHPHIHQ

AVNVVINFDFPKNSETYLHRVGRSGRFGHLGIAINLITYEDRFNLYKIEQELGTEIKPIPPVIEKGLYCA

>GFH26097.1 DEAD-box ATP-dependent RNA helicase, partial [Haematococcus lacustris]

MATVVEEAGEHWKEKLNLPAKDLRVRTLDVTNTKGNDFEDYFLKRQLLMGIFEKGFDKPSPIQEESIPIALAGRDVLARA

KNGTGKTAAFAVPVLEKIDTSKNEIQAMILVPTRELALQTAQVCKELGKYLAVEVMVTTGGTSLKDDIMRLHQTIHIVVA

TPGRILDLASKGIAKLNNCRVLVMDEADKLLSFEFQPIIEQLINFLPGDRQIMLFSATFPVTVKDFKEKFLKKPYIINLM

DELTLKGLSQYYAFVEEKQKVHCLNTLFSKLRINQSIIFCNSVNRVELLAKKITELGYSCFYIHAKMLQSHRNRVFHDFR

NG

>XP_013901052.1 ATP-dependent RNA helicase DDX6/DHH1 [Monoraphidium neglectum]

MAALQHGGRLDVTATKGHEFEDYFLKRELLMGIYEKGFEKPSPIQEESIPIALTGRDILARAKNGTGKTAAFSIPVLEKI

DPTKNEVQAMLLVPTRELALQTAQVCKELGKHLAVDIMVTTGGTSLKDDIMRLYQPVHVVVATPGRILDLASKGVCKLNN

CRVLVMDEADKLLSPEFQPLIEQLISFLPDGRQIMLFSATFPVTVKQFKEKFLRKPYIINLMDELTLKGVTQYYAFVEER

QKVHCLNTLFSKCVCV
