# Supplementary material for: DDX6 Is Essential for Oocyte Development and Maturation in Locusta migratoria
Source: Insects. 2021 Jan 14;12(1):70. doi: 10.3390/insects12010070 (PMC7830464; doi:10.3390/insects12010070)
Supplement: Supplementary file 1 [file insects-12-00070-s001.zip › Supplementary material/Supplemental file 3.docx]

**191 sequences of DDX6 from insects**

>XP_023723833.1 ATP-dependent RNA helicase me31b [Cryptotermes secundus]

MMTETHINSNHVAVMPGLNSISSQKGGVDKIDDIGWKAKLKIPPKDRRVQTSDVTDTRGNEFEEFCLKRELLMGIFEKGW

EKPSPIQEASIPIALSGKDVLARAKNGTGKTGAYSIPVLEQVDPKKDCIQALVIVPTRELALQTSQICIELAKHMDIKVM

VTTGGTNLRDDIMRIYQKVQVIIATPGRILDLMDKNVAIMDSCRILVLDEADKLLSQDFKGMLDHVISRLPKERQILLFS

ATFPLTVKQFMEKHLREPYEINLMEELTLKGVTQYYAFVQERQKVHCLNTLFSKLQINQSIIFCNSTQRVELLAKKITEL

GYCCYYIHAKMAQAHRNRVFHDFRAGLCRNLVCSDLFTRGIDVQAVNVVINFDFPKMAETYLHRIGRSGRFGHLGIAINL

ITYEDRFALHRIEQELGTEIKPIPKVIDPSLYVAKLEDTQGIEEGNVSK

>QBB01435.1 putative putative ATP-dependent RNA helicase me31b isoform X1, partial [Cotesia chilonis]

KCVIEMMTETHINSNHMLNSSLGSKSDLEKMDDVGWKSKLRIPPRDKRIKTSDVTNTRGNEFEEFCLKRELLMGIFEKGW

EKPSPIQEASIPIALSGKDILARAKNGTGKTGAYSIPVLEQVDPRKDIIQALVIVPTRELALQTSQICIELAKHMDVKVM

VTTGGTNLRDDIMRIYQKVHVIIATPGRILDLMDKNVANMDHCRILVLDEADKLLSQDFKGMLDHVISRLPSERQILLYS

ATFPLTVKQFMEKHLRDPYEINLMEELTLKGVTQYYAFVQERQKVHCLNTLFSKLQINQSIIFCNSTQRVELLAKKITDL

GYCCYYIHAKMAQAHRNRVFHDFRAGLCRNLVSSDLFTRGIDVQAVNVVINFDFPKMAETYLHRIGRSGRFGHLGIAINL

ITYEDRFNLHRIEQELGTEIKPIPKVIDPSLYVARPEDNTSLDEGNVSK

>XP_015509618.1 PREDICTED: putative ATP-dependent RNA helicase me31b [Neodiprion lecontei]

MMTETHINSNHMLNSGLGPKGDLEKMDDIGWKAKLKIPPKDKRIKTSDVTDTRGNEFEEFCLKRELLMGIFEKGWEKPSP

IQEASIPIALSGKDVLARAKNGTGKTGAYSIPVLEQVDPRKDVIQALVIVPTRELALQTSQICIELAKHMDVKVMVTTGG

TNLRDDIMRIYQKVQVIIATPGRILDLMDKNVANMDHCRILVLDEADKLLSQDFKGMLDHVISRLPQERQILLYSATFPL

TVKQFMEKHLRDPYEINLMEELTLKGVTQYYAFVQERQKVHCLNTLFSKLQITQSIIFCNSTQRVELLAKKITDLGYCCY

YIHAKMAQAHRNRVFHDFRAGLCRNLVCSDLFTRGIDVQAVNVVINFDFPKMAETYLHRIGRSGRFGHLGIAINLITYED

RFALHRIEQELGTEIKPIPKVIDPSLYVARLEENSIMEEGNISK

>XP_011335980.1 putative ATP-dependent RNA helicase me31b [Ooceraea biroi]

MMTETHLNSNHMLNSGLSNKSEIDKMDDVGWKAKLKIPPKDKRIKTSDVTDTRGNEFEEFCLKRELLMGIFEKGWEKPSP

IQEASIPIALSGKDILARAKNGTGKTGAYSIPVLEQVDPRKDVIQALVIVPTRELALQTSQICIELAKHMDIKVMVTTGG

TNLRDDIMRIYQKVQVIIATPGRILDLMDKNVANMEHCKILVLDEADKLLSQDFKGMLDHVISRLPHERQILLYSATFPL

TVKQFMEKHLRDPYEINLMEELTLKGVTQYYAFVQERQKVHCLNTLFSKLQITQSIIFCNSTQRVELLAKKITDLGYCCY

YIHAKMAQAHRNRVFHDFRAGLCRNLVSSDLFTRGIDVQAVNVVINFDFPKMAETYLHRIGRSGRFGHLGIAINLITYED

RFNLHRIEQELGTEIKPIPKVIDPSLYVARPEDSNGMEEGNVSK

>XP_017763833.1 PREDICTED: putative ATP-dependent RNA helicase me31b [Eufriesea mexicana]

MMTETHINSNHVLNSGLNTKSDIEKMDDVGWKAKLKIPPKDKRIKTSDVTDTRGNEFEEFCLKRELLMGIFEKGWEKPSP

IQEASIPIALSGKDILARAKNGTGKTGAYSIPVLEQVDPQKDVIQALVLVPTRELALQTSQICIELAKHMDVKVMVTTGG

TDLRDDIMRIYQTVQVIIATPGRILDLIDKNVANMEHCKTLVLDEADKLLSQDFKGMLDHVISRLPRERQILLYSATFPL

TVKQFMEKHLRDPYEINLMEELTLKGVTQYYAFVQERQKVHCLNTLFSKLQITQSIIFCNSTQRVELLAKKITDLGYCCY

YIHAKMAQAHRNRVFHDFRAGLCRNLVSSDLFTRGIDVQAVNVVINFDFPKMAETYLHRIGRSGRFGHLGIAINLITYED

RFNLHRIEQELGTEIKPIPKVIDPSLYVARSEDNNSMEEGNVSK

>XP_012266451.1 putative ATP-dependent RNA helicase me31b [Athalia rosae]

MMTENHINSNHMLNSGLGPKADLEKMDDIGWKAKLKIPPKDKRIKTSDVTDTRGNEFEEFCLKRELLMGIFEKGWEKPSP

IQEASIPIALSGKDVLARAKNGTGKTGAYSIPVLEQIDPRKDVIQALVIVPTRELALQTSQICIELAKHMDIKVMVTTGG

TNLRDDIMRIYQKVQAIIATPGRILDLMDKNVANMDHCRILVLDEADKLLSQDFKGMLDHVISRLPQERQILLYSATFPL

TVKQFMEKHLRDPYEINLMEELTLKGVTQYYAFVQERQKVHCLNTLFSKLQITQSIIFCNSTQRVELLAKKITDLGYCCY

YIHAKMAQAHRNRVFHDFRAGLCRNLVCSDLFTRGIDVQAVNVVINFDFPKMAETYLHRIGRSGRFGHLGIAINLITYED

RFALHRIEQELGTEIKPIPKVIDPSLYVARAEESSILEEGNVSK

>XP_008552788.1 PREDICTED: putative ATP-dependent RNA helicase me31b [Microplitis demolitor]

MMTETHINSNHMLNSGLGSKSELEKMDDVGWKSKLRIPPRDKRIKTSDVTNTRGNEFEEFCLKRELLMGIFEKGWEKPSP

IQEASIPIALSGKDILARAKNGTGKTGAYSIPVLEQVDPRKDVIQALVIVPTRELALQTSQICIELAKHMDVKVMVTTGG

TNLRDDIMRIYQKVHVIIATPGRILDLMDKNVATMDHCRILVLDEADKLLSQDFKGMLDHVISRLPKERQILLYSATFPL

TVKQFMEKHLRDPYEINLMEELTLKGVTQYYAFVQERQKVHCLNTLFSKLQINQSIIFCNSTQRVELLAKKITDLGYCCY

YIHAKMAQAHRNRVFHDFRAGLCRNLVSSDLFTRGIDVQAVNVVINFDFPKMAETYLHRIGRSGRFGHLGIAINLITYED

RFNLHRIEQELGTEIKPIPKVIDPSLYVARPEDNTSLDEGNVSK

>XP_001602897.1 ATP-dependent RNA helicase me31b [Nasonia vitripennis]

MMTETHMNTNHVLSSGLGLKTDLEKMDDSGWKSKLKIPPKDRRIKTSDVTDTRGNEFEEFCLKRELLMGIFEKGWEKPSP

IQEASIPIALSGKDVLARAKNGTGKTGAYSIPVLEQVDPKKDVIQALIIVPTRELALQTSQILIELAKHMDIKVMVTTGG

TNLRDDIMRIYQKVQVIIATPGRILDLMDKNVANMDHCRILVLDEADKLLSQDFKGMLDHVISRLPHERQILLYSATFPL

TVKQFMEKHLRDPYEINLMEELTLKGVTQYYAFVQERQKVHCLNTLFSKLQINQSIIFCNTTQRVELLAKKITDLGYCCY

YIHAKMAQAHRNRVFHDFRAGLCRNLVCSDLFTRGIDVQAVNVVINFDFPKMAETYLHRIGRSGRFGHLGIAINLITYED

RFNLHRIEQELGTEIKPIPKVIDPSLYVARPDDNNSLEDSSNVSK

>XP_012532762.1 ATP-dependent RNA helicase me31b [Monomorium pharaonis]

MMTETHMNSNHMLNSGLSTKSEIDKMDDVGWKAKLKIPPKDKRIKTSDVTDTRGNEFEEFCLKRELLMGIFEKGWEKPSP

IQEASIPIALSGKDILARAKNGTGKTGAYSIPVLEQVDPRKDVIQALVIVPTRELALQTSQICIELAKHMDIKVMVTTGG

TNLRDDIMRIYQKVQVIIATPGRILDLMDKNVANMEHCKILVLDEADKLLSQDFKGMLDHVISRLPHERQILLYSATFPL

TVKQFMEKHLRDPYEINLMEELTLKGVTQYYAFVQERQKVHCLNTLFSKLQITQSIIFCNSTQRVELLAKKITDLGYCCY

YIHAKMAQAHRNRVFHDFRAGLCRNLVSSDLFTRGIDVQAVNVVINFDFPKMAETYLHRIGRSGRFGHLGIAINLITYED

RFNLHRIEQELGTEIKPIPKVIDPSLYVARSEDNNSMEEGNVSK

>XP_014477511.1 PREDICTED: putative ATP-dependent RNA helicase me31b [Dinoponera quadriceps]

MMTETHINSNHMLNSGLSTKSEIDKMDDVGWKAKLKIPPKDKRIKTSDVTDTRGNEFEEFCLKRELLMGIFEKGWEKPSP

IQEASIPIALSGKDILARAKNGTGKTGAYSIPVLEQVDPRKDVIQALVIVPTRELALQTSQICIELAKHMDIKVMVTTGG

TNLRDDIMRIYQKVQVIIATPGRILDLMDKNVANMEHCKILVLDEADKLLSQDFKGMLDHVISRLPHERQILLYSATFPL

TVKQFMEKHLRDPYEINLMEELTLKGVTQYYAFVQERQKVHCLNTLFSKLQITQSIIFCNSTQRVELLAKKITDLGYCCY

YIHAKMAQAHRNRVFHDFRAGLCRNLVSSDLFTRGIDVQAVNVVINFDFPKMAETYLHRIGRSGRFGHLGIAINLITYED

RFNLHRIEQELGTEIKPIPKVIDPSLYVARPEDNNSMEEGNVSK

>XP_003704663.1 PREDICTED: putative ATP-dependent RNA helicase me31b [Megachile rotundata]

MMTETHINSNHVLNSGLNTKSEIDKMDDVGWKAKLKIPPKDKRIKTSDVTDTRGNEFEEFCLKRELLMGIFEKGWEKPSP

IQEASIPIALSGKDILARAKNGTGKTGAYSIPVLEQVDPRKDVIQALVIVPTRELALQTSQICIELAKHMDIKVMVTTGG

TDLRDDIMRIYQKVQVIIATPGRILDLMDKNVANMDHCKILVLDEADKLLSQDFKGMLDHVISRLPHERQILLYSATFPL

TVKQFMEKHLRDPYEINLMEELTLKGVTQYYAFVQERQKVHCLNTLFSKLQITQSIIFCNSTQRVELLAKKITDLGYCCY

YIHAKMAQAHRNRVFHDFRAGLCRNLVSSDLFTRGIDVQAVNVVINFDFPKMAETYLHRIGRSGRFGHLGIAINLITYED

RFNLHRIEQELGTEIKPIPKVIDPSLYVARPEDNNSMEEGNVSK

>XP_015601276.1 putative ATP-dependent RNA helicase me31b [Cephus cinctus]

MMTETHINSNHMLNSGLGTKADLEKTDDVGWKAKLKIPPKDKRIKTSDVTDTRGNEFEEFCLKRELLMGIFEKGWEKPSP

IQEASIPIALSGKDILARAKNGTGKTGAYSIPVLEQVDPQIDVIQALVIVPTRELALQTSQICIELAKHMDVKVMVTTGG

TNLRDDIMRIYQKVHVIIATPGRILDLMDKNVANMDNCKILVLDEADKLLSQDFRGMLDHVISRLPKERQILLYSATFPL

TVKQFMEKHLNEPYEINLMEELTLKGVTQYYAFVQERQKVHCLNTLFSKLQITQSIIFCNSTQRVELLAKKITDLGYCCY

YIHARMAQAHRNRVFHDFRAGLCRNLVCSDLFTRGIDVQAVNVVINFDFPKMAETYLHRIGRSGRFGHLGIAINLITYED

RFALHRIEQELGTEIKPIPKVIDPSLYVTRPEDNNSMEEGNVSK

>XP_003690677.1 ATP-dependent RNA helicase me31b [Apis florea]

MMTETHINSNHVLNSGLNTKSEIDKMDDVGWKAKLKIPPKDKRIKTSDVTDTRGNEFEEFCLKRELLMGIFEKGWEKPSP

IQEASIPIALSGKDILARAKNGTGKTGAYSIPVLEQVDPRKDVIQALVLVPTRELALQTSQICIELAKHMEIKVMVTTGG

TDLRDDIMRIYQSVQVIIATPGRILDLMDKNVANMDHCKTLVLDEADKLLSQDFKGMLDHVISRLPHERQILLYSATFPL

TVKQFMEKHLRDPYEINLMEELTLKGVTQYYAFVQERQKVHCLNTLFSKLQITQSIIFCNSTQRVELLAKKITDLGYCCY

YIHAKMAQAHRNRVFHDFRAGLCRNLVSSDLFTRGIDVQAVNVVINFDFPKMAETYLHRIGRSGRFGHLGIAINLITYED

RFNLHRIEQELGTEIKPIPKVIDPSLYVARPEDNNSMEEGNVSK

>XP_003402776.1 putative ATP-dependent RNA helicase me31b [Bombus terrestris]

MMTETHINSNHVLNSGLNTKSEIDKMDDVGWKAKLKIPPKDKRIKTSDVTDTRGNEFEEFCLKRELLMGIFEKGWEKPSP

IQEASIPIALSGKDILARAKNGTGKTGAYSIPVLEQVDPRKDVIQALVLVPTRELALQTSQICIELAKHMDIKVMVTTGG

TDLRDDIMRIYQTVQVIIATPGRILDLMDKNVANMDHCKTLVLDEADKLLSQDFKGMLDHVISRLPHERQILLYSATFPL

TVKQFMEKHLRDPYEINLMEELTLKGVTQYYAFVQERQKVHCLNTLFSKLQITQSIIFCNSTQRVELLAKKITDLGYCCY

YIHAKMAQAHRNRVFHDFRAGLCRNLVSSDLFTRGIDVQAVNVVINFDFPKMAETYLHRIGRSGRFGHLGIAINLITYED

RFNLHRIEQELGTEIKPIPKVIDPSLYVARPEDNNSMEEGNVSK

>XP_018406244.1 PREDICTED: putative ATP-dependent RNA helicase me31b [Cyphomyrmex costatus]

MMTETHMNSNHMLNSGLSNKSEIDKMDDVGWKAKLKIPPKDKRIKTSDVTDTRGNEFEEFCLKRELLMGIFEKGWEKPSP

IQEASIPIALSGKDILARAKNGTGKTGAYSIPVLEQVDPRKDVIQALVIVPTRELALQTSQICIELAKHMDIKVMVTTGG

TNLRDDIMRIYQKVQVIIATPGRILDLMDKNVANMEHCKILVLDEADKLLSQDFKGMLDHVISRLPHERQILLYSATFPL

TVKQFMEKHLRDPYEINLMEELTLKGVTQYYAFVQERQKVHCLNTLFSKLQITQSIIFCNSTQRVELLAKKITDLGYCCY

YIHAKMAQAHRNRVFHDFRAGLCRNLVSSDLFTRGIDVQAVNVVINFDFPKMAETYLHRIGRSGRFGHLGIAINLITYED

RFNLHRIEQELGTEIKPIPKVIDPSLYVARSEDNNSMEEVNVSK

>OXU24906.1 hypothetical protein TSAR_006904 [Trichomalopsis sarcophagae]

MMTETHMNTNHVLSSGLGLKTDLEKMDDSGWKSKLKIPPKDRRIKTSDVTDTRGNEFEEFCLKRELLMGIFEKGWEKPSP

IQEASIPIALSGKDVLARAKNGTGKTGAYSIPVLEQVDPKKDVIQALIIVPTRELALQTSQILIELAKHMDIKVMVTTGG

TNLRDDIMRIYQKVQVIIATPGRILDLMDKNVANMDHCRILVLDEADKLLSQDFKGMLDHVISRLPHERQILLYSATFPL

TVKQFMEKHLRDPYEINLMEELTLKGVTQYYAFVQERQKVHCLNTLFSKLQINQSIIFCNTTQRVELLAKKITDLGYCCY

YIHAKMAQAHRNRVFHDFRAGLCRNLVCSDLFTRGIDVQAVNVVINFDFPKMAETYLHRIGRSGRFGHLGIAINLITYED

RFNLHRIEQELGTEIKPIPKVIDPSLYVARPDDNNSLEDGSNVSK

>XP_029036826.1 ATP-dependent RNA helicase me31b [Osmia bicornis bicornis]

MMTETHINSNHVLNSGLNTKSEIDKMDDVGWKAKLKIPPKDKRIKTSDVTDTRGNEFEEFCLKRELLMGIFEKGWEKPSP

IQEASIPIALSGKDILARAKNGTGKTGAYSIPVLEQVDPRKDVIQALVIVPTRELALQTSQICIELAKHMDIKVMVTTGG

TDLRDDIMRIYQKVQVIIATPGRILDLMDKKVANMDHCKILVLDEADKLLSQDFKGMLDHVISRLPHERQILLYSATFPL

TVKQFMEKHLRDPYEINLMEELTLKGVTQYYAFVQERQKVHCLNTLFSKLQITQSIIFCNSTQRVELLAKKITDLGYCCY

YIHAKMAQAHRNRVFHDFRAGLCRNLVSSDLFTRGIDVQAVNVVINFDFPKMAETYLHRIGRSGRFGHLGIAINLITYED

RFNLHRIEQELGTEIKPIPKVIDPSLYVARPEDNNSMEEGNVSK

>OAD52719.1 Putative ATP-dependent RNA helicase me31b [Eufriesea mexicana]

MMTETHINSNHVLNSGLNTKSDIEKMDDVGWKAKLKIPPKDKRIKTSDVTDTRGNEFEEFCLKRELLMGIFEKGWEKPSP

IQEASIPIALSGKDILARAKNGTGKTGAYSIPVLEQVDPQKDVIQALVLVPTRELALQTSQICIELAKHMDVKVMVTTGG

TDLRDDIMRIYQTVQVIIATPGRILDLIDKNVANMEHCKTLVLDEADKLLSQDFKGMLDHVISRLPRERQILLYSATFPL

TVKQFMEKHLRDPYEINLMEELTLKGVTQYYAFVQERQKVHCLNTLFSKLQITQSIIFCNSTQRVELLAKKITDLGYCCY

YIHAKMAQAHRNRVFHDFRAGLCRNLVNIDLFTRGIDVQAVNVVINFDFPKMAETYLHRIGRSGRFGHLGIAINLITYED

RFNLHRIEQELGTEIKPIPKVIDPSLYVARSEDNNSMEEGNVSK

>XP_011694219.1 PREDICTED: putative ATP-dependent RNA helicase me31b [Wasmannia auropunctata]

MMTETHMNSNHMLNSGLSNKSELDKMDDVGWKAKLKIPPKDKRIKTSDVTDTRGNEFEEFCLKRELLMGIFEKGWEKPSP

IQEASIPIALSGKDILARAKNGTGKTGAYSIPVLEQVDPRKDVIQALVIVPTRELALQTSQICIELAKHMEIKVMVTTGG

TNLRDDIMRIYQKVQVIIATPGRILDLMDKNVANMEHCKILVLDEADKLLSQDFKGMLDHVISRLPNERQILLYSATFPL

TVKQFMEKHLRDPYEINLMEELTLKGVTQYYAFVQERQKVHCLNTLFSKLQITQSIIFCNSTQRVELLAKKITDLGYCCY

YIHAKMAQAHRNRVFHDFRAGLCRNLVSSDLFTRGIDVQAVNVVINFDFPKMAETYLHRIGRSGRFGHLGIAINLITYED

RFNLHRIEQELGTEIKPIPKVIDPSLYVAKPEDNSMEEGNVSK

>XP_011501061.1 PREDICTED: putative ATP-dependent RNA helicase me31b [Ceratosolen solmsi marchali]

MMTESHINSNHVLNSGLGIKTDLEKMDDVGWKAKLKIPPKDKRIKTSDVTDTRGNEFEEFCLKRELLMGIFEKGWEKPSP

IQEASIPIALSGKDVLARAKNGTGKTGAYSIPVLEQVDPKKDVIQALIIVPTRELALQTSQILIELAKHMDVKVMVTTGG

TNLRDDIMRIYQKVQAIIATPGRILDLMDKNVANMDHCRILVLDEADKLLSQDFKGMLDHVISRLPQERQILLYSATFPL

TVKQFMEKHLRDPYEINLMEELTLKGVTQYYAFVQERQKVHCLNTLFSKLQINQSIIFCNTTQRVELLAKKITDLGYCCY

YIHAKMAQAHRNRVFHDFRAGLCRNLVCSDLFTRGIDVQAVNVVINFDFPKMAETYLHRIGRSGRFGHLGIAINLITYED

RFNLHRIEHELGTEIKPIPKVIDPSLYVARPDDNNSLEEGNVSK

>XP_014604326.1 PREDICTED: putative ATP-dependent RNA helicase me31b isoform X1 [Polistes canadensis]

MMTETHMNSNHMLNSALNAKTELEKMDDVGWKAKLKIPPKDKRIKTSDVTDTRGNEFEEFCLKRELLMGIFEKGWEKPSP

IQEASIPIALSGKDILARAKNGTGKTGAYSIPVLEQVDPRKDVIQALVIVPTRELALQTSQICIELAKHMDIKVMVTTGG

TNLRDDIMRIYQKVQVIIATPGRILDLMDKNVANMDHCKILVLDEADKLLSQDFKGMLDHVISKLPHERQILLYSATFPL

TVKQFMEKHLRDPYEINLMEELTLKGVTQYYAFVQERQKVHCLNTLFSKLQITQSIIFCNSTQRVELLAKKITDLGYCCY

YIHAKMAQAHRNRVFHDFRAGLCRNLVSSDLFTRGIDVQAVNVVINFDFPKMAETYLHRIGRSGRFGHLGIAINLITYED

RFNLHRIEQELGTEIKPIPKVIDPSLYVIRPEDNNSMEEGNVSK

>XP_011166764.1 putative ATP-dependent RNA helicase me31b [Solenopsis invicta]

MMTETHMNSNHMLNSGLSNKSEIEKMDDVGWKAKLKIPPKDKRIKTSDVTDTRGNEFEEFCLKRELLMGIFEKGWEKPSP

IQEASIPIALSGKDILARAKNGTGKTGAYSIPVLEQVDPRKDVIQALVIVPTRELALQTSQICIELAKHMDIKVMVTTGG

TNLRDDIMRIYQKVQVIIATPGRILDLMDKNVANMEHCKILVLDEADKLLSQDFKGMLDHVISRLPHERQILLYSATFPL

TVKQFMEKHLRDPYEINLMEELTLKGVTQYYAFVQERQKVHCLNTLFSKLQITQSIIFCNSTQRVELLAKKITDLGYCCY

YIHAKMAQAHRNRVFHDFRAGLCRNLVSSDLFTRGIDVQAVNVVINFDFPKMAETYLHRIGRSGRFGHLGIAINLITYED

RFNLHRIEQELGTEIKPIPKVIDPSLYVARPEDNNSMEEGNVSK

>XP_018311392.1 PREDICTED: putative ATP-dependent RNA helicase me31b isoform X1 [Trachymyrmex zeteki]

MMTETHMNSNHMLNSGLSNKSEIDKMDDVGWKAKLNIPPKDKRIKTSDVTDTRGNEFEEFCLKRELLMGIFEKGWEKPSP

IQEASIPIALSGKDILARAKNGTGKTGAYSIPVLEQVDPRKDVIQALVIVPTRELALQTSQICIELAKHMDIKVMVTTGG

TNLRDDIMRIYQKVQVIIATPGRILDLMDKNVANMEHCKILVLDEADKLLSQDFKGMLDHVISRLPHERQILLYSATFPL

TVKQFMEKHLRDPYEINLMEELTLKGVTQYYAFVQERQKVHCLNTLFSKLQITQSIIFCNSTQRVELLAKKITDLGYCCY

YIHAKMAQAHRNRVFHDFRAGLCRNLVSSDLFTRGIDVQAVNVVINFDFPKMAETYLHRIGRSGRFGHLGIAINLITYED

RFNLHRIEQELGTEIKPIPKVIDPSLYVARPEDNNSMEEGNVSK

>XP_011863849.1 PREDICTED: putative ATP-dependent RNA helicase me31b [Vollenhovia emeryi]

MMTETHMNSNHMLNSGLSNKSEIEKMDDVGWKAKLKIPPKDKRIKTSDVTDTRGNEFEEFCLKRELLMGIFEKGWEKPSP

IQEASIPIALSGKDILARAKNGTGKTGAYSIPVLEQVDPRKDVIQALVIVPTRELALQTSQICIELAKHMDIKVMVTTGG

TNLRDDIMRIYQKVQVIIATPGRILDLMDKNVANMEHCKILVLDEADKLLSQDFKGMLDHVISRLPQERQILLYSATFPL

TVKQFMEKHLKDPYEINLMEELTLKGVTQYYAFVQERQKVHCLNTLFSKLQITQSIIFCNSTQRVELLAKKITDLGYCCY

YIHAKMAQAHRNRVFHDFRAGLCRNLVSSDLFTRGIDVQAVNVVINFDFPKMAETYLHRIGRSGRFGHLGIAINLITYED

RFNLHRIEQELGTEIKPIPKVIDPSLYVARPEDNNSMEEGNVSK

>XP_011062102.1 PREDICTED: putative ATP-dependent RNA helicase me31b [Acromyrmex echinatior]

MMTETHMNSNHMLNSGLSNKSEIDKMDDVGWKAKLKIPPKDKRIKTSDVTDTRGNEFEEFCLKRELLMGIFEKGWEKPSP

IQEASIPIALSGKDILARAKNGTGKTGAYSIPVLEQVDPRKDVIQALVIVPTRELALQTSQICIELAKHMDIKVMVTTGG

TNLRDDIMRIYQKVQVIIATPGRILDLMEKNVANMEHCKILVLDEADKLLSQDFKGMLDHVISRLPHERQILLYSATFPL

TVKQFMEKHLRDPYEINLMEELTLKGVTQYYAFVQERQKVHCLNTLFSKLQITQSIIFCNSTQRVELLAKKITDLGYCCY

YIHAKMAQAHRNRVFHDFRAGLCRNLVSSDLFTRGIDVQAVNVVINFDFPKMAETYLHRIGRSGRFGHLGIAINLITYED

RFNLHRIEQELGTEIKPIPKVIDPSLYVARPEDNNSMEEGNVSK

>XP_015186768.1 PREDICTED: putative ATP-dependent RNA helicase me31b [Polistes dominula]

MMTETHMNSNHMLNSALNTKTEIEKMDDVGWKAKLKIPPKDKRIKTSDVTDTRGNEFEEFCLKRELLMGIFEKGWEKPSP

IQEASIPIALSGKDILARAKNGTGKTGAYSIPVLEQVDPRKDVIQALVIVPTRELALQTSQICIELAKHMDIKVMVTTGG

TNLRDDIMRIYQKVQVIIATPGRILDLMDKNVANMDHCKILVLDEADKLLSQDFKGMLDHVISKLPHERQILLYSATFPL

TVKQFMEKHLRDPYEINLMEELTLKGVTQYYAFVQERQKVHCLNTLFSKLQITQSIIFCNSTQRVELLAKKITDLGYCCY

YIHAKMAQAHRNRVFHDFRAGLCRNLVSSDLFTRGIDVQAVNVVINFDFPKMAETYLHRIGRSGRFGHLGIAINLITYED

RFNLHRIEQELGTEIKPIPKVIDPSLYVIRPEDNNSMEEGNVSK

>KAE8750197.1 hypothetical protein FOCC_FOCC003004 [Frankliniella occidentalis]

MPSSYSDLKVKRLPLSGNCLYTMTEVHSNHVIGGISMNKDLDDGIGWKSKLKIPPKDQRIKTSDVTDTRGNEFEEFCLKR

ELLMGIFEKGWEKPSPIQEASIPIALSGKDVLARAKNGTGKTGAYSIPVLEQVDPKKDCIQALVIVPTRELALQTSQICI

ELAKHMDVKVMVTTGGTNLRDDIMRIYQKVHIIIATPGRILDLMDKNVANMENCRILVLDEADKLLSQDFKGMLDHVISR

LPKERQILLYSATFPLTVKQFMEKHLKEPYEINLMEELTLKGVTQYYAFVQERQKVHCLNTLFSKLQINQSIIFCNSTQR

VELLAKKITELGYCCYYIHAKMAQAHRNRVFHDFRNGLCRNLVCSDLFTRGIDVQAVNVVINFDFPKMAETYLHRIGRSG

RFGHLGIAINLITFDDRFALHRIEQELGTEIKPIPKVIDPALYVAKSLDDSHAIEEANNVSK

>XP_012218710.1 PREDICTED: LOW QUALITY PROTEIN: putative ATP-dependent RNA helicase me31b [Linepithema humile]

MMTETHMNSNHNMLNSGLSNKPEIDKMDDVGWKAKLKIPPKDKRIKTSDVTDTRGNEFEEFCLKRELLMGIFEKGWEKPS

PIQEASIPIALSGKDILARAKNGTGKTGAYSIPVLEQVDPRKDVIQALVIVPTRELALQTSQICIELAKHMDIKVMVTTG

GTNLRDDIMRIYQKVQVIIATPGRILDLMDKNVANMEHCKILVLDEADKLLSQDFKGMLDHVISRLPQERQILLYSATFP

LTVKQFMEKHLRDPYEINLMEELTLKGVTQYYAFVQERQKVHCLNTLFSKLQITQSIIFCNSTQRVELLAKKXTDLGYCC

YYIHAKMAQAHRNRVFHDFRAGLCRNLVSSDLFTRGIDVQAVNVVINFDFPKMAETYLHRIGRSGRFGHLGIAINLITYE

DRFNLHRIEQELGTEIKPIPKVIDPSLYVARPEDNNSMEEGNVSK

>XP_034942755.1 ATP-dependent RNA helicase me31b [Chelonus insularis]

MMTETHINSNHMLNSGLGSKPDLEKMDDVGWKAKLKIPPQDKRIKTSDVTNTRGNEFEEFCLKRELLMGIFEKGWEKPSP

IQEASIPIALSGKDILARAKNGTGKTGAYSIPVLEQVDPKKDVIQALVIVPTRELALQTSQICIELAKHMDIRVMVTTGG

TNLREDIMRIYQKVQVIIATPGRILDLMDKNVANMDHCRILVLDEADKLLSQDFKGMLDHVISRLPLERQILLYSATFPL

TVKQFMEKHLRDPYEINLMEELTLKGVTQYYAFVQERQKVHCLNTLFSKLQINQSIIFCNSTQRVELLAKKITDLGYCCY

YIHAKMAQAHRNRVFHDFRAGLCRNLVSSDLFTRGIDVQAVNVVINFDFPKMAETYLHRIGRSGRFGHLGIAINLITYED

RFNLHRIEQELGTEIKPIPKVIDPSLYVARPEDNTSMEEGNVSK

>XP_026291730.1 putative ATP-dependent RNA helicase me31b [Frankliniella occidentalis]

MTEVHSNHVIGGISMNKDLDDGIGWKSKLKIPPKDQRIKTSDVTDTRGNEFEEFCLKRELLMGIFEKGWEKPSPIQEASI

PIALSGKDVLARAKNGTGKTGAYSIPVLEQVDPKKDCIQALVIVPTRELALQTSQICIELAKHMDVKVMVTTGGTNLRDD

IMRIYQKVHIIIATPGRILDLMDKNVANMENCRILVLDEADKLLSQDFKGMLDHVISRLPKERQILLYSATFPLTVKQFM

EKHLKEPYEINLMEELTLKGVTQYYAFVQERQKVHCLNTLFSKLQINQSIIFCNSTQRVELLAKKITELGYCCYYIHAKM

AQAHRNRVFHDFRNGLCRNLVCSDLFTRGIDVQAVNVVINFDFPKMAETYLHRIGRSGRFGHLGIAINLITFDDRFALHR

IEQELGTEIKPIPKVIDPALYVAKSLDDSHAIEEANNVSK

>XP_034250163.1 ATP-dependent RNA helicase me31b [Thrips palmi]

MTEIVANHVLGGMNMNNQKVDLDDGIGWKSKLKIPPKDQRIKTSDVTDTRGNEFEEFCLKRELLMGIFEKGWEKPSPIQE

ASIPIALSGKDVLARAKNGTGKTGAYSIPVLEQVDPKKDCIQALVIVPTRELALQTSQICIELAKHMDVKVMVTTGGTNL

RDDIMRIYQKVHVIIATPGRILDLMDKKVANMDSCRILVLDEADKLLSQDFKGMLDHVISRLPQERQILLYSATFPLTVK

QFMEKHLKEPYEINLMEELTLKGVTQYYAFVQERQKVHCLNTLFSKLQINQSIIFCNSTQRVELLAKKITELGYCCYYIH

AKMAQAHRNRVFHDFRNGLCRNLVCSDLFTRGIDVQAVNVVINFDFPKMAETYLHRIGRSGRFGHLGIAINLITFDDRFA

LHRIEQELGTEIKPIPKVIDPALYVAKTLDDCHAIEEADSSK

>XP_011315526.1 PREDICTED: putative ATP-dependent RNA helicase me31b [Fopius arisanus]

MMTETHMNSNHMLNSGLSSKSDLEKMDDVGWKAKLKIPPKDKRIKTSDVTDTRGNDFEEFCLKRELLMGIFEKGWEKPSP

IQEASIPIALSGKDILARAKNGTGKTGAYSIPVLEQVDAKKDVIQALVIVPTRELALQTSQICIELAKHMEVKVMVTTGG

TNLRDDIMRIYQKVQVIIATPGRILDLMDKNVANMNHCRILVLDEADKLLSQDFKGMLDHVISRLPQERQILLYSATFPL

TVKQFMEKHLRDPYEINLMEELTLKGVTQYYAFVQERQKVHCLNTLFSKLQITQSIIFCNSTQRVELLAKKITDLGYCCY

YIHAKMAQAHRNRVFHDFRAGLCRNLVSSDLFTRGIDVQAVNVVINFDFPKMAETYLHRIGRSGRFGHLGIAINLITYED

RFNLHRIESELGTEIKPIPKVIDPSLYVARPEDNNSMEEGNVSK

>XP_020287769.1 putative ATP-dependent RNA helicase me31b isoform X2 [Pseudomyrmex gracilis]

MMTETHMNSNHMLNSGLSNKSEIDKMDDVGWKAKLKIPPKDKRIKTSDVTDTRGNEFEEFCLKRELLMGIFEKGWEKPSP

IQEASIPIALSGKDILARAKNGTGKTGAYSIPVLEQVDPKKDVIQALVIVPTRELALQTSQICIELAKHMDIKVMVTTGG

TNLRDDIMRIYQKVQVIIATPGRILDLMDKHVANMGECKILVLDEADKLLSQDFKGMLDHVISRLPPQRQILLYSATFPL

TVKQFMEKHLRDPYEINLMEELTLKGVTQYYAFVQERQKVHCLNTLFSKLQITQSIIFCNSTQRVELLAKKITDLGYCCY

YIHAKMAQAHRNRVFHDFRAGLCRNLVSSDLFTRGIDVQAVNVVINFDFPKMAETYLHRIGRSGRFGHLGIAINLITYED

RFNLHRIEQELGTEIKPIPKVIDPSLYVARPEDNNSMEEGNVSK

>XP_035730236.1 ATP-dependent RNA helicase me31b-like [Vespa mandarinia]

MITETHMNSNHMLNSALNTKTELEKMDDVGWKAKLKIPPKDKRIKTSDVTDTRGNEFEEFCLKRELLMGIFEKGWEKPSP

IQEASIPIALSGKDILARAKNGTGKTGAYSIPVLEQVDPRKDVIQALVIVPTRELALQTSQICIELAKHMDIKVMVTTGG

TNLRDDIMRIYQKVQVIIATPGRILDLMDKNVANMDHCKILVLDEADKLLSQDFKGMLDHVISKLPHERQILLYSATFPL

TVKQFMEKHLRDPYEINLMEELTLKGVTQYYAFVQERQKVHCLNTLFSKLQITQSIIFCNSTQRVELLAKKITDLGYCCY

YIHAKMAQAHRNRVFHDFRAGLCRNLVSSDLFTRGIDVQAVNVVINFDFPKMAETYLHRIGRSGRFGHLGIAINLITYED

RFNLHRIEQELGTEIKPIPKVIDPSLYVIRPEDNNSMEEGNVSK

>XP_029163119.1 ATP-dependent RNA helicase me31b [Nylanderia fulva]

MMIETHINSNHMLNSGLSNKSEIEKMDDVGWKAKLKIPPKDKRIKTSDVTDTRGNEFEEFCLKRELLMGIFEKGWEKPSP

IQEASIPIALSGKDILARAKNGTGKTGAYSIPVLEQVDPRKEVIQALVIVPTRELALQTSQICIELAKHMDIKVMVTTGG

TNLRDDIMRIYQKVQVIIATPGRILDLMDKNVANMEHCKILVLDEADKLLSQDFKGMLDHVISRLPHERQILLYSATFPL

TVKQFMEKHLRDPYEINLMEELTLKGVTQYYAFVQERQKVHCLNTLFSKLQITQSIIFCNSTQRVELLAKKITDLGYCCY

YIHAKMAQAHRNRVFHDFRAGLCRNLVSSDLFTRGIDVQAVNVVINFDFPKMAETYLHRIGRSGRFGHLGIAINLITYED

RFNLHRIEQELGTEIKPIPKVIDPSLYVARPEDNNSMEEGNVSK

>XP_011265969.1 putative ATP-dependent RNA helicase me31b [Camponotus floridanus]

MMIETHINSNHMLNSGLSNKSEIDKMDDVGWKAKLKIPPKDKRIKTSDVTDTRGNEFEEFCLKRELLMGIFEKGWEKPSP

IQEASIPIALSGKDILARAKNGTGKTGAYSIPVLEQVDPRREVIQALVIVPTRELALQTSQICIELAKHMDIKVMVTTGG

TNLRDDIMRIYQKVQVIIATPGRILDLMDKNVANMEHCKILVLDEADKLLSQDFKGMLDHVISRLPHERQILLYSATFPL

TVKQFMEKHLRDPYEINLMEELTLKGVTQYYAFVQERQKVHCLNTLFSKLQITQSIIFCNSTQRVELLAKKITDLGYCCY

YIHAKMAQAHRNRVFHDFRAGLCRNLVSSDLFTRGIDVQAVNVVINFDFPKMAETYLHRIGRSGRFGHLGIAINLITYED

RFNLHRIEQELGTEIKPIPKVIDPSLYVARPEDNNSMEEGNVSK

>XP_020287768.1 putative ATP-dependent RNA helicase me31b isoform X1 [Pseudomyrmex gracilis]

MKNVSGYGRQQHCARNVSNCWETKIFWRNGKESIQYIHYNSSKMMTETHMNSNHMLNSGLSNKSEIDKMDDVGWKAKLKI

PPKDKRIKTSDVTDTRGNEFEEFCLKRELLMGIFEKGWEKPSPIQEASIPIALSGKDILARAKNGTGKTGAYSIPVLEQV

DPKKDVIQALVIVPTRELALQTSQICIELAKHMDIKVMVTTGGTNLRDDIMRIYQKVQVIIATPGRILDLMDKHVANMGE

CKILVLDEADKLLSQDFKGMLDHVISRLPPQRQILLYSATFPLTVKQFMEKHLRDPYEINLMEELTLKGVTQYYAFVQER

QKVHCLNTLFSKLQITQSIIFCNSTQRVELLAKKITDLGYCCYYIHAKMAQAHRNRVFHDFRAGLCRNLVSSDLFTRGID

VQAVNVVINFDFPKMAETYLHRIGRSGRFGHLGIAINLITYEDRFNLHRIEQELGTEIKPIPKVIDPSLYVARPEDNNSM

EEGNVSK

>EZA56181.1 Putative ATP-dependent RNA helicase me31b [Ooceraea biroi]

MMTETHLNSNHMLNSGLSNKSEIDKMDDVGWKAKLKIPPKDKRIKTSDVTDTRGNEFEEFCLKRELLMGIFEKGWEKPSP

IQEASIPIALSGKDILARAKNGTGKTGAYSIPVLEQVDPRKDVIQALVIVPTRELALQTSQICIELAKHMDIKVMVTTGG

TNLRDDIMRIYQKVQVIIATPGRILDLMDKNVANMEHCKILVLDEADKLLSQDFKGMLDHVISRLPHERQILLYSATFPL

TVKQFMEKHLRDPYEINLMEELTLKGVTQYYAFVQERQKVHCLNTLFSKYFIHVLILQLQITQSIIFCNSTQRVELLAKK

ITDLGYCCYYIHAKMAQAHRNRVFHDFRAGLCRNLVSSDLFTRGIDVQAVNVVINFDFPKMAETYLHRIGRSGRFGHLGI

AINLITYEDRFNLHRIEQELGTEIKPIPKVIDPSLYVARPEDSNGMEEGNVSK

>XP_026461540.1 putative ATP-dependent RNA helicase me31b [Ctenocephalides felis]

MIAETHLSSIHMNNLSQNNKGDLEKMDDLGWKSKLKIPPKDRRIKTSDVTDTRGNEFEEFCLKRELLMGIFEKGWEKPSP

IQEASIPIALSGKDILARAKNGTGKTGAYCIPVLEQVDPKKDCIQALVIVPTRELALQTSQICIELAKHMEIRVMVTTGG

TNLRDDIMRIYQKVQVIIATPGRILDLMDKQVANTEHCRMLVLDEADKLLSQDFKGMLDHVISRLPRERQILLFSATFPL

SVKQFMEKHLKEPYEINLMEELTLKGVTQYYAFVQERQKVHCLNTLFSKLQINQSIIFCNSTQRVELLAKKITELGYCCY

YIHAKMAQAHRNRVFHDFRSGLCRNLVCSDLFTRGIDVQAVNVVINFDFPKMAETYLHRIGRSGRFGHLGIAINLITYED

RFALHRIESELGTEIKPIPKVIDPSLYVAKLEGDVPIEENVSQ

>XP_015435825.1 PREDICTED: putative ATP-dependent RNA helicase me31b [Dufourea novaeangliae]

MMTETHINSNHVLNSGLAPKSEIDKMDDVGWKAKLKIPPKDKRIKTSDVTDTRGNEFEEFCLKRELLMGIFEKGWEKPSP

IQEASIPIALSGKDILARAKNGTGKTGAYSIPVLEQVDSRKDVIQALVIVPTRELALQTSQICIELAKHMDIKVMVTTGG

TNLRDDIMRIYQKVQVIIATPGRILDLMDKNVANMDHCKILVLDEADKLLSQDFKGMLDHVISRLPHERQILLYSATFPL

TVKQFMEKHLRDPYEINLMEELTLKGVTQYYAFVQERQKVHCLNTLFSKLQITQSIIFCNSTQRVELLAKKITDLGYCCY

YIHAKMAQAHRNRVFHDFRAGLCRNLVSSDLFTRGIDVQAVNVVINFDFPKMAETYLHRIGRSGRFGHLGIAINLITYED

RFNLHRIEQELGTEIKPIPKVIDPNLYVARPEDNNSMEEGNVSK

>XP_029668949.1 ATP-dependent RNA helicase me31b [Formica exsecta]

MMIETHINSNHMLNSGLSNKSEIDKMDDVGWKAKLKIPPKDKRIKTSDVTDTRGNEFEEFCLKRELLMGIFEKGWEKPSP

IQEASIPIALSGKDILARAKNGTGKTGAYSIPVLEQVDPRREVIQALVIVPTRELALQTSQICIELAKHMDIKVMVTTGG

TNLRDDIMRIYQKVQVIIATPGRILDLMDKNVANMEQCKILVLDEADKLLSQDFKGMLDHVISRLPHERQILLYSATFPL

TVKQFMEKHLRDPYEINLMEELTLKGVTQYYAFVQERQKVHCLNTLFSKLQITQSIIFCNSTQRVELLAKKITDLGYCCY

YIHAKMAQAHRNRVFHDFRAGLCRNLVSSDLFTRGIDVQAVNVVINFDFPKMAETYLHRIGRSGRFGHLGIAINLITYED

RFNLHRIEQELGTEIKPIPKVIDPSLYVARPEDNNSMEEGNVSK

>EFN61728.1 Putative ATP-dependent RNA helicase me31b, partial [Camponotus floridanus]

MIETHINSNHMLNSGLSNKSEIDKMDDVGWKAKLKIPPKDKRIKTSDVTDTRGNEFEEFCLKRELLMGIFEKGWEKPSPI

QEASIPIALSGKDILARAKNGTGKTGAYSIPVLEQVDPRREVIQALVIVPTRELALQTSQICIELAKHMDIKVMVTTGGT

NLRDDIMRIYQKVQVIIATPGRILDLMDKNVANMEHCKILVLDEADKLLSQDFKGMLDHVISRLPHERQILLYSATFPLT

VKQFMEKHLRDPYEINLMEELTLKGVTQYYAFVQERQKVHCLNTLFSKLQITQSIIFCNSTQRVELLAKKITDLGYCCYY

IHAKMAQAHRNRVFHDFRAGLCRNLVSSDLFTRGIDVQAVNVVINFDFPKMAETYLHRIGRSGRFGHLGIAINLITYEDR

FNLHRIEQELGTEIKPIPKVIDPSLYVARPEDNNSMEEGNVSK

>PBC31773.1 Putative ATP-dependent RNA helicase me31b [Apis cerana cerana]

MMTETHINSNHVLNSGLNTKSEIDKMDDVGWKAKLKIPPKDKRIKTSDVTDTRGNEFEEFCLKRELLMGIFEKGWEKPSP

IQEASIPIALSGKDILARAKNGTGKTGAYSIPVLEQVDPRKDVIQALVLVPTRELALQTSQICIELAKHMEIKVMVTTGG

TDLRDDIMRIYQSVQVIIATPGRILDLMDKNVANMDHCKTLVLDEADKLLSQDFKGMLDHVISRLPHERQILLYSATFPL

TVKQFMEKHLRDPYEINLMEELTLKGVTQYYAFVQERQKVHCLNTLFSKLQITQSIIFCNSTQRVELLAKKITDLGYCCY

YIHAKMAQAHRNRVFHDFRAGLCRNLVSSDLFTRGIDVQAVNVVINFDFPKMAETYLHRIGRSGRFGHLGIAINLITYED

RFNLHRIEQELGTEIKPIPKVIDPSLYVARPEDNNSMEEVKQRKYELLVNKAY

>XP_014222340.1 putative ATP-dependent RNA helicase me31b isoform X1 [Trichogramma pretiosum]

MMTETVMNSNHVLNSSLALKADMEKLDDVGWKAKLKIPPKDKRIKTSDVTDTRGNEFDEFCLKRELLMGIFEKGWEKPSP

IQEASIPIALSGKDVLARAKNGTGKTGAYSIPVLEQVDPKRDVIQALIIVPTRELALQTSQILIELAKHMDVKVMVTTGG

TNLRDDIMRIYQKVQVIIATPGRILDLMDKNVANMEYCKILVLDEADKLLSQDFKGMLDHVISRLPQERQILLYSATFPL

TVKQFMEKHLKDPYEINLMEELTLKGVTQYYAFVQERQKVHCLNTLFSKLQINQSIIFCNTTQRVELLAKKITDLGYCCY

YIHAKMAQAHRNRVFHDFRAGLCRNLVCSDLFTRGIDVQAVNVVINFDFPKMAETYLHRIGRSGRFGHLGIAINLITYED

RFNLHRIEQELGTEIKPIPKVIDPCLYVARPEDNSILEEGNVSK

>XP_015126615.1 ATP-dependent RNA helicase me31b [Diachasma alloeum]

MMTETHINSNHMLNSGLTSKSDHEKMDDVGWKAKLKIPPKDKRIKTSDVTDTRGNDFEEFCLKRELLMGIFEKGWEKPSP

IQEASIPIALSGKDILARAKNGTGKTGAYSIPVLEQVDAKRDVIQALVIVPTRELALQTSQICIELAKHMDVKVMVTTGG

TNLRDDIMRIYQKVQVIIATPGRILDLMDKNVANMDHCRILVLDEADKLLSQDFKGMLDHVISRLPQERQILLYSATFPL

TVKQFMEKHLRDPYEINLMEELTLKGVTQYYAFVQERQKVHCLNTLFSKLQITQSIIFCNSTQRVELLAKKITDLGYCCY

YIHAKMAQAHRNRVFHDFRAGLCRNLVSSDLFTRGIDVQAVNVVINFDFPKMAETYLHRIGRSGRFGHLGIAINLITYED

RFNLHRIESELGTEIKPIPKVIDPSLYVARPDDSNSMEEGNVSK

>XP_012272161.1 putative ATP-dependent RNA helicase me31b [Orussus abietinus]

MMTETHINANHMLNSGLNAKAELEKLDDVGWKAKLRIPPKDKRIKTSDVTDTRGNEFEEFCLKRELLMGIFEKGWEKPSP

IQEASIPIALSGKDILARAKNGTGKTGAYSIPVLEQVDPRKEVIQALVIVPTRELALQTSQICIELARHMDIKVMVTTGG

TNLRDDIMRIYQKVQVIIATPGRILDLMDKNVAHMDQCRILVLDEADKLLSQDFKGMLDHVISKLPRERQILLYSATFPL

TVKQFMEKHLKDPYEINLMDELTLKGVTQYYAFVQERQKVHCLNTLFSKLQITQSIIFCNSTQRVELLAKKITDLGYCCY

YIHAKMAQAHRNRVFHDFRAGLCRNLVSSDLFTRGIDVQAVNVVINFDFPKMAETYLHRIGRSGRFGHLGIAINLITYED

RFNLHRIEQELGTEIKPIPKVIDPSLYVARPEDNNSMDEGNVSK

>XP_017877105.1 putative ATP-dependent RNA helicase me31b [Ceratina calcarata]

MMTEHINSNHVLNSGLNSKSDIDKMDDVGWKAKLKIPPKDKRVKTSDVTDTRGNEFEEFCLKRELLMGIFEKGWEKPSPI

QEASIPIALSGKDILARAKNGTGKTGAYSIPVLEQVDPRKDVIQALVIVPTRELALQTSQICIELAKHMDIKVMVTTGGT

DLRDDIMRIYQKVQVIIATPGRILDLMDKNVAQMDHCKILVLDEADKLLSQDFKGMLDHVISRLPHSRQILLYSATFPLT

VKQFMEKHLRDPYEINLMEELTLKGVTQYYAFVQERQKVHCLNTLFSKLQITQSIIFCNSTQRVELLAKKITDLGYCCYY

IHAKMAQAHRNRVFHDFRAGLCRNLVSSDLFTRGIDVQAVNVVINFDFPKMAETYLHRIGRSGRFGHLGIAINLITYEDR

FNLHRIEQELGTEIKPIPKVIDPSLYVARPEDNNSMEEGNVSK

>KAF7390367.1 hypothetical protein [Vespula pensylvanica]

MITETHMNSNHMLNSALNTKTELEKMDDVGWKAKLKIPPKDKRIKTSDVTDTRGNEFEEFCLKRELLMGIFEKGWEKPSP

IQEASIPIALSGKDILARAKNGTGKTGAYSIPVLEQVDPRKDVIQALVIVPTRELALQTSQICIELAKHMDIKVMVTTGG

TNLRDDIMRIYQKVQVIIATPGRILDLMDKNVANMDHCKILVLDEADKLLSQDFKGMLDHVISKLPHERQILLYSATFPL

TVKQFMEKHLRDPYEINLMEELTLKGVTQYYAFVQERQKVHCLNTLFSKLQITQSIIFCNSTQRVELLAKKITDLGYCCY

YIHAKMAQAHRNRVFHDFRAGLCRNLVSSDLFTRGIDVQAVNVVINFDFPKMAETYLHRIGRSGRFGHLGIAINLITYED

RFNLHRIEQELGTEIKPIPKVIDPSLYVIRPEDNNSMEEGNNDDAAAADDYDDDDDDDDDDDDDDDDDDDDDDDDDNGGD

GDGDGGDNDDYDDEEEEEEEEEEEEEENQIVVQNLMETFTKIN

>KAF4518826.1 hypothetical protein B566_EDAN008155 [Ephemera danica]

MLLHPLRIAVVFLNQQTTRISDTSALKSVRFLGHPPSQPPSIFFKMMTETHISASNHKSVLPGLMNSQLKSIDSEKMDDH

GWKAKLKIPPKDKRVKTSDVTDTKGNEFEEFCLKRELLMGIFEKGWEKPSPIQEASIPIALTGKDILARAKNGTGKTGAY

CIPVLEQVDPKKEVIQALVIVPTRELALQTSQICIELAKHMDVKVMVTTGGTNLRDDIMRIFQKVQVVIATPGRILDLME

KKVANMDHCRILVLDEADKLLSQDFKGMLDHVISRLPKERQILLYSATFPLTVKNFMVKHLKEPYEINLMEELTLKGVTQ

YYAFVQERQKVHCLNTLFSKLQINQSIIFCNSTQRVELLAKKITELGYCCYYIHAKMAQAHRNRVFHDFRAGLCRNLVCS

DLFTRGIDVQAVNVVINFDFPKMAETYLHRIGRSGRFGHLGIAINLITYEDRFALHRIEQELGTEIKPIPKVIDPSLYVA

KFDDLQQAIEDSSNTTNASS

>XP_033323558.1 ATP-dependent RNA helicase me31b [Megalopta genalis]

MMTEAHINSNHVLNSGLTPKSDIDKMDDVGWKAKLKIPPKDKRIKTSDVTDTRGNEFEEFCLKRELLMGIFEKGWEKPSP

IQEASIPIALSGKDILARAKNGTGKTGAYSIPVLEQVDPRKDVIQALVIVPTRELALQTSQICIELAKHMDIKVMVTTGG

TILRDDIMRIYQKVQVIIATPGRILDLMDKNVAHMGHCKILVLDEADKLLSQDFKGMLDHVISRLPHERQILLYSATFPL

TVKQFMEKHLRDPYEINLMEELTLKGVTQYYAFVQERQKVHCLNTLFSKLQITQSIIFCNSTQRVELLAKKITDLGYCCY

YIHAKMAQAHRNRVFHDFRAGLCRNLVSSDLFTRGIDVQAVNVVINFDFPKMAETYLHRIGRSGRFGHLGIAINLITYED

RFNLHRIEQELGTEIKPIPKVIDPNLYVARPEDNNSMEEGNVSK

>XP_011139072.1 putative ATP-dependent RNA helicase me31b [Harpegnathos saltator]

MMTETHINSNHMLNSGLSTKSEIDKMDDVGWKAKLKIPPKDKRIKTSDVTDTRGNEFEEFCLKRELLMGIFEKGWEKPSP

IQEASIPIALSSKDILARAKNGTGKTGAYSIPVLEQVDPKKDVIQALVIVPTRELALQTSQICIELAKHMDIKVMVTTGG

TNLRDDIMRIYQKVQVIIATPGRILDLMDKNVANMEHCKILVLDEADKLLSQDFKGMLDHVISRLPHERQILLYSATFPL

TVKQFMEKHLRDPYEINLMEELTLKGVTQYYAFVQERQKVHCLNTLFSKLQITQSIIFCNSTQRVELLAKKITDLGYCCY

YIHAKMAQAHRNRVFHDFRAGLCRNLVSSDLFTRGIDVQAVNVVINFDFPKMAETYLHRIGRSGRFGHLGIAINLITYED

RFNLHRIEQELGTEIKPIPKVIDPSLYVAKPVCND

>KAF7379367.1 hypothetical protein [Vespula vulgaris]

MITETHMNSNHMLNSALNTKTELEKMDDVGWKAKLKIPPKDKRIKTSDVTDTRGNEFEEFCLKRELLMGIFEKGWEKPSP

IQEASIPIALSGKDILARAKNGTGKTGAYSIPVLEQVDPRKDVIQALVIVPTRELALQTSQICIELAKHMDIKVMVTTGG

TNLRDDIMRIYQKVQVIIATPGRILDLMDKNVANMDHCKILVLDEADKLLSQDFKGMLDHVISKLPHERQILLYSATFPL

TVKQFMEKHLRDPYEINLMEELTLKGVTQYYAFVQERQKVHCLNTLFSKLQITQSIIFCNSTQRVELLAKKITDLGYCCY

YIHAKMAQAHRNRVFHDFRAGLCRNLVSSDLFTRGIDVQAVNVVINFDFPKMAETYLHRIGRSGRFGHLGIAINLITYED

RFNLHRIEQELGTEIKPIPKVIDPSLYVIRPEDNNSMEEE

>XP_033225353.1 ATP-dependent RNA helicase me31b [Belonocnema treatae]

MMTETHINSNHMLNSGLGPKTDLAKMDDVGWKAKLKIPPKDIRMKTSDVTDTRGNEFEEFCLKRELLMGIFEKGWEKPSP

IQEASIPIALSGKDILARAKNGTGKTGAYSIPVLEQVDPRKDVIQALVIVPTRELALQTSQILIELAKHMDVKVMVTTGG

TNLRDDIMRIYQKVQVIIATPGRILDLIDQNVANMDHCRILVLDEADKLLSQDFKGMLDHVISRLPQRRQILLYSATFPL

TVKQFMEKHLKDPYEINLMEELTLKGVTQYYAFVQERQKVHCLNTLFSKLQINQSIIFCNTTQRVELLAKKITDLGYCCY

YIHAKMAQAHRNRVFHDFRAGLCRNLVCSDLFTRGIDVQAVNVVINFDFPKMAETYLHRIGRSGRFGHLGIAINLITYED

RFNLHRIEQELGTEIKPIPKVIDPSLYVSRPEDNSCLEEGNVSK

>KOX76536.1 Putative ATP-dependent RNA helicase me31b [Melipona quadrifasciata]

MMTEAHINSNHVLNSGLNTKSEIDKMDDVGWKAKLKIPPKDKRIKTSDVTDTRGNEFEEFCLKRELLMGIFEKGWEKPSP

IQEASIPIALSGKDILARAKNGTGKTGAYSIPVLEQVDPRKDVIQALVLVPTRELALQTSQICIELAKHMDIKVMVTTGG

TDLRDDIMRIYQTVQVIIATPGRILDLMDKNVANMDHCKTLVLDEADKLLSQDFKGMLDHVISRLPHERQILLYSATFPL

TVKQFMEKHLRDPYEINLMEELTLKGVTQYYAFVQERQKVHCLNTLFSKLQITQSIIFCNSTQRVELLAKKITDLGCCCY

YIHAKMAQAHRNRVFHDFRAGLCRNLVSSDLFTRGIDVQAVNVVINFDFPKMAETYLRRIGRSGRFGHLGIAINLITYED

RFNLHRIEQELGTEIKPIPKVTDPSLYVARPEDNNSMEEGNVSKQKHTLVRIHVRKIFCNPCSLFTMLVPSTIRAETIKK

MKKIKKKLRELIKMKKGWKGSVQRWGYLCPRVGSGNDPYYSALVGWESARAVWKWWGSGGEFLRGEGGLEGLDRGLNGAG

SGKG

>XP_022114286.1 putative ATP-dependent RNA helicase me31b [Pieris rapae]

MMTENRISAINHVGNSINNQKGDVDKTVDDMGWKSKLKLPPKDRRMKTSDVTDTRGNEFEEFCLKREILMGIFEKGWEKP

SPIQEASIPIALSGKDVLARAKNGTGKTGAYCIPVIEQVDPKKDCIQALIVVPTRELALQTSQICIELAKHTDIRVMVTT

GGTNLRDDIMRIYQNVQVIIATPGRIIDLMDKQVAKMDQCRMLVLDEADKLLSQDFKGMLDLVISRLPKERQILLFSATF

PLSVKQFMEKHLREPYEINLMEELTLKGVTQYYAFVQERQKVHCLNTLFSKLQINQSIIFCNSTQRVELLAKKITELGYC

CYYIHARMAQAHRNRVFHDFRAGLCRNLVCSDLFTRGIDVQAVNVVINFDFPRMAETYLHRIGRSGRFGHLGIAINLITY

EDRFALHRIEQELGTEIKPIPKVIDPALYVARVSEDDGADK

>RZF36041.1 hypothetical protein LSTR_LSTR005857 [Laodelphax striatellus]

MMTETHVTSNHIALQGKNQMNNTASGKLDMENEIGWKSKLRLPPPDRRIKTSDVTDTKGNEFEEYCLKRDLLMGIFEKGW

EKPSPIQEASIPIALTGKDVLARAKNGTGKTGAYSIPVLEQVNPEKDHIQALVIVPTRELALQTSQICIELAKHMNVRVM

VTTGGTNLRDDILRIYQKVHVIIATPGRILDLMDKNVANMDNCKILLLDEADKLLSQDFKGMLDHVISRLPQERQILLYS

ATFPLTVKQFMEKHLKDPYEINLMEELTLKGVTQYYAFVQERQKVHCLNTLFSKLQINQSIIFCNSTQRVELLAKKITEL

GYCCYYIHAKMAQAHRNRVFHDFRAGLCRNLVCSDLFTRGIDVQAVNVVINFDFPKMAETYLHRIGRSGRFGHLGIALNL

ITYEDRFALHRIEQELGTEIKPIPKVIDPDLYVAKLDDSIEDANVSK

>XP_026315629.1 putative ATP-dependent RNA helicase me31b [Hyposmocoma kahamanoa]

MMTENRISSSNHVGNSMNNQKGDVDKNIDDIGWKSKLKIPPKDRRIKTSDVTDTRGNEFEEFCLKRELLMGIFEKGWEKP

SPIQEASIPIALSGKDVLARAKNGTGKTGAYCIPVLEQVDPKKDCIQALIVVPTRELALQTSQICIELAKHTDIRVMVTT

GGTNLRDDIMRIYQNVQVIIATPGRMIDLMDKQVAKMDQCRMLVLDEADKLLSQDFKGMLDMVISRLPKERQILLFSATF

PLSVKQFMEKHLREPYEINLMEELTLKGVTQYYAFVQERQKVHCLNTLFSKLQINQSIIFCNSTQRVELLAKKITELGYC

CYYIHARMAQAHRNRVFHDFRAGLCRNLVCSDLFTRGIDVQAVNVVINFDFPRMAETYLHRIGRSGRFGHLGIAINLITY

EDRFALHRIEQELGTEIKPIPKVIDPALYVSRPDDDDTADK

>VVC89928.1 unnamed protein product [Leptidea sinapis]

MMTESRISSSNHVGNSMNNQKGELDKTLEDIGWKSKLKVPPKDRRIKTSDVTDTRGNEFEEFCLKREILMGIFEKGWEKP

SPIQEASIPIALSGKDVLARAKNGTGKTGAYCIPVLEQIDPKKDCIQALVVVPTRELALQTSQICIELAKHTDIRVMVTT

GGTNLRDDIMRIYQNVQVIIATPGRMIDLMDKQVAKMEHCRILVLDEADKLLSQDFKGMLDMVINRLPKERQILLFSATF

PLSVKQFMEKHLREPYEINLMEELTLKGVTQYYAFVQERQKVHCLNTLFSKLQINQSIIFCNSTQRVELLAKKITELGYC

CYYIHARMAQAHRNRVFHDFRAGLCRNLVCSDLFTRGIDVQAVNVVINFDFPRMAETYLHRIGRSGRFGHLGIAINLITY

EDRFALHRIEQELGTEIKPIPKVIDPALYVARPEDDNGDK

>XP_011563976.1 PREDICTED: putative ATP-dependent RNA helicase me31b [Plutella xylostella]

MMTERISSSNHVGNSINNQSKGDVDKAVDDIGWKSKLKIPPKDRRIKTSDVTDTRGNEFEEFCLKRELLMGIFEKGWEKP

SPIQEASIPIALSGKDVLARAKNGTGKTGAYCIPVLEQVDPKKDCIQALIVVPTRELALQTSQICIELAKHTDIRVMVTT

GGTNLRDDIMRIYQNVQVIIATPGRMIDLMDKQVAKMDQCRMLVLDEADKLLSQDFKGMLDMVICRLPKERQILLFSATF

PLSVKQFMEKHLREPYEINLMEELTLKGVTQYYAFVQERQKVHCLNTLFSKLQINQSIIFCNSTQRVELLAKKITELGYC

CYYIHARMAQAHRNRVFHDFRAGLCRNLVCSDLFTRGIDVQAVNVVINFDFPRMAETYLHRIGRSGRFGHLGIAINLITY

EDRFALHRIEQELGTEIKPIPKVIDPALYVARPSDDCSDDK

>XP_014204182.1 putative ATP-dependent RNA helicase me31b [Copidosoma floridanum]

MMSTESHMNSNHHVLSSALSLKSDLEKMDDVGWKAKLKIPPKDKRIKTSDVTDTRGNEFEEFCLKRELLMGIFEKGWEKP

SPIQEASIPIALSGKDVLARAKNGTGKTGAYSIPVLEQVDATKDVIQSLIIVPTRELALQTSQILIQLAKHMDVKVMVST

GGTNLRDDIMRIYQKVHVIIATPGRILDLMDKNVADMEHCRILVLDEADKLLSQDFKGMLDHVISRLPQERQILLYSATF

PLTVKQFMEKHLRDPYEINLMEELTLKGVTQYYAFVQERQKVHCLNTLFSKLQINQSIIFCNTTQRVELLAKKITDLGYC

CYYIHAKMAQAHRNRVFHDFRNGLCRNLVCSDLFTRGIDVQAVNVVINFDFPKMAETYLHRIGRSGRFGHLGIAINLITY

EDRFNLHRIEQELGTEIKPIPKVIDPCLYVSRQDENNSLEESNVSK

>KAF3421629.1 hypothetical protein E2986_09645 [Frieseomelitta varia]

MMTEAHINSNHVLNSGLSTKSEIDKMDDVGWKAKLKIPPKDKRIKTSDVTDTRGNEFEEFCLKRELLMGIFEKGWEKPSP

IQEASIPIALSGKDILARAKNGTGKTGAYSIPVLEQVDPRKDVIQALVLVPTRELALQTSQICIELAKHMDIKVMVTTGG

TDLRDDIMRIYQTVQVIIATPGRILDLMDKNVANMDHCKTLVLDEADKLLSQDFKGMLDHTYHVLVFLYTFRLPHERQIL

LYSATFPLTVKQFMEKHLRDPYEINLMEELTLKGVTQYYAFVQERQKVHCLNTLFSKLQITQSIIFCNSTQRVELLAKKI

TDLGYCCYYIHAKMAQAHRNRVFHDFRAGLCRNLVSSDLFTRGIDVQAVNVVINFDFPKMAETYLHRIGRSGRFGHLGIA

INLITYEDRFNLHRIEQELGTEIKPIPKVIDPSLYVARPEDNNSMEEGNVSK

>XP_031843584.1 ATP-dependent RNA helicase me31b [Nomia melanderi]

MMTEAHINSNHVLNSGLTSKSEIDKMDDVGWKAKLKIPPKDKRIKTSDVTDTRGNEFEEFCLKRELLMGIFEKGWEKPSP

IQEASIPIALSGKDILARAKNGTGKTGAYSIPVLEQVDPQKGVIQALVIVPTRELALQTSQICMQLAKHMDIKIMVSTGG

TNLRDDIMRIYQKAQVIIATPGRILDLMDKSVAHMDHCKILVLDEADKLLSQDFKGMLDHVISRLPHERQILLYSATFPL

TVKQFMEKHLRDPYEINLMEELTLKGVTQYYAFVQERQKVHCLNTLFSKLQITQSIIFCNSTQRVELLAKKITDLGYCCY

YIHAKMAQAHRNRVFHDFRAGVCRNLVSSDLFTRGIDVQAVNVVINFDFPKMAETYLHRIGRSGRFGHLGIAINLITYED

RFNLHRIEQELGTEIKPIPKVIDPNLYVARPKDNNSMEEVNVSK

>XP_002427795.1 DEAD box ATP-dependent RNA helicase, putative [Pediculus humanus corporis]

MAIHIPCVITNHITKMMTETHISNIMMPALNSIASGKVEVEKPDEGWKSKLKRPPPDRRVQTSDVTDRKGNDFEEFCLKR

ELLMGIFEKGWEKPSPIQEASIPIALSGKDILARAKNGTGKTGAYTIPVLELVDPKKDVIQALIIVPTRELALQTSQICI

ELAKHMETKVMVTTGGTNLRDDIMRIYQKVQVVIATPGRILDLMEKNVANMDECKTLVLDEADKLLSQDFQGMLDHVISR

LPKDRQILLYSATFPVTVKNFMEKHLHEPYEVNLMEELTLKGVTQYYAFVQERQKVHCLNTLFSKLQINQSIIFCNSTQR

VELLAKKITELGYCCYYIHAKMAQAHRNRVFHDFRNGLCRNLVCSDLFTRGIDVQAVNVVINFDFPKMAETYLHRIGRSG

RFGHLGIAINLITYEDRLALHRIEQELGTEIKPIPKVIDPSLYVAKLEDTQGIEEVNVSK

>XP_022914613.1 putative ATP-dependent RNA helicase me31b [Onthophagus taurus]

MMTESIAANNHVMPVQTKLDMEKVDDIGWKSKLKIPPKDKRIKTSDVTDTRGNEFEEFCLKRELLMGIFEKGWEKPSPIQ

EASIPIALSGKDVLARAKNGTGKTGAYSIPVLEQIDPKKDCIQALIIVPTRELALQTSQICIELAKHMHPVRVMVTTGGT

NLRDDIMRIYQKVQVIIATPGRILDLMDKQVANMDQCRMLVLDEADKLLSQDFKGMLDHVISRLPKERQILLFSATFPLT

VKQFMEKHLRDPYEINLMEELTLKGVTQYYAFVQERQKVHCLNTLFSKLQINQSIIFCNSTQRVELLAKKITELGYCCYY

IHAKMAQAHRNRVFHDFRAGLCRNLVSSDLFTRGIDVQAVNVVINFDFPKIAETYLHRIGRSGRFGHLGVAINLITYEDR

YGLHRIEQELGTEIKPIPKVIDPALYVPKIDDDDSMESNQAQ

>KMQ93867.1 atp-dependent rna helicase me31b [Lasius niger]

MLNSGLSNKSEIDKMDDVGWKAKLKIPPKDKRIKTSDVTDTRGNEFEEFCLKRELLMGIFEKGWEKPSPIQEASIPIALS

GKDILARAKNGTGKTGAYSIPVLEQVDPRKEVIQALVIVPTRELALQTSQICIELAKHMDIKVMVTTGGTNLRDDIMRIY

QKVQVIIATPGRILDLMDKNVANMEHCKILVLDEADKLLSQDFKGMLDHVISRLPHERQILLYSATFPLTVKQFMEKHLR

DPYEINLMEELTLKGVTQYYAFVQERQKVHCLNTLFSKLQITQSIIFCNSTQRVELLAKKITDLGYCCYYIHAKMAQAHR

NRVFHDFRAGLCRNLVSSDLFTRGIDVQAVNVVINFDFPKMAETYLHRIGRSGRFGHLGIAINLITYEDRFNLHRIEQEL

GTEIKPIPKVIDPSLYVARPEDNNSMEEGNVSK

>KRT83187.1 helicase [Oryctes borbonicus]

MMTESITTNNHVMPTQTKLDIDNVDDIGWKAKLKIPPKDKRIKTSDVTSTRGNEFEEFCLKRELLMGIFEKGWEKPSPIQ

EASIPIALSGKDILARAKNGTGKTGAYSIPVLEQIDPEEDAIQALIIVPTRELALQTSQICIELAKHMNVRVMVTTGGTN

LRDDIMRIYQKVQVIIATPGRILDLMDKGVANMEHCRTLVLDEADKLLSQDFKGMLDHVISRLPKDRQIFLFSATFPLTV

KQFMEKHLKNPYEINLMEELTLKGVTQYYAFVQERQKVHCLNTLFSKLQINQSIIFCNSTQRVELLAKKITDLGYCCYYI

HAKMAQSHRNRVFHDFRAGLCRNLVCSDLFTRGIDVQAVNVVINFDFPKMAETYLHRIGRSGRFGHLGIAINLITYEDRF

ALHRIEQELGTEIKPIPKVIDPALYVPKLEDDETIEQTQTQ

>XP_026752361.1 ATP-dependent RNA helicase me31b [Galleria mellonella]

MMTENRISSSNHVGNSLSNQSKGDVDKTIDDIGWKSKLKIPPKDRRIKTSDVTDTRGNEFEEFCLKRELLMGIFEKGWEK

PSPIQEASIPIALSGKDVLARAKNGTGKTGAYCIPVLEQVDPKKDAIQALIVVPTRELALQTSQICIELAKHTDIRVMVT

TGGTNLRDDIMRIYQNVQVIIATPGRMIDLMDKQVAKMDQCRMLVLDEADKLLSQDFKGMLDMVISRLPKERQILLFSAT

FPLSVKQFMEKHLREPYEINLMEELTLKGVTQYYAFVQERQKVHCLNTLFSKLQINQSIIFCNSTQRVELLAKKITELGY

CCYYIHARMAQAHRNRVFHDFRAGLCRNLVCSDLFTRGIDVQAVNVVINFDFPRMAETYLHRIGRSGRFGHLGIAINLIT

YEDRFALHRIEQELGTEIKPIPKVIDPALYVARPEDDDSADK

>RVE52672.1 hypothetical protein evm_002791 [Chilo suppressalis]

MMTENRISSSNHVVNSMSNPNKGDVDKTIDDIGWKSKLKIPPKDRRIKTSDVTDTRGNEFEEFCLKRELLMGIFEKGWEK

PSPIQEASIPIALSGKDVLARAKNGTGKTGAYCIPVLEQVDPKKDCIQALIVVPTRELALQTSQICIELAKHTDIRVMVT

TGGTNLRDDIMRIYQNVQVIIATPGRMIDLMDKKVAKMEQCRMLVLDEADKLLSQDFKGMLDMVISRLPKERQILLFSAT

FPLSVKQFMEKHLREPYEINLMEELTLKGVTQYYAFVQERQKVHCLNTLFSKLQINQSIIFCNSTQRVELLAKKITELGY

CCYYIHARMAQAHRNRVFHDFRAGLCRNLVCSDLFTRGIDVQAVNVVINFDFPRMAETYLHRIGRSGRFGHLGIAINLIT

YEDRFALHRIEQELGTEIKPIPKTIDPALYVSRPDDDDADK

>XP_028162601.1 putative ATP-dependent RNA helicase me31b [Ostrinia furnacalis]

MMTENRISSSNHVGNSMSNQNKGDVDKTIDDIGWKSKLKIPPKDRRIKTSDVTDTRGNEFEEFCLKRELLMGIFEKGWEK

PSPIQEASIPIALSGKDVLARAKNGTGKTGAYCIPVLEQVDPKKDAIQALIVVPTRELALQTSQICIELAKHTDIRVMVT

TGGTNLRDDIMRIYQNVQVIIATPGRMIDLMDKQVAKMDQCRMLVLDEADKLLSQDFKGMLDMVISRLPKERQILLFSAT

FPLSVKQFMEKHLREPYEINLMEELTLKGVTQYYAFVQERQKVHCLNTLFSKLQINQSIIFCNSTQRVELLAKKITELGY

CCYYIHARMAQAHRNRVFHDFRAGLCRNLVCSDLFTRGIDVQAVNVVINFDFPRMAETYLHRIGRSGRFGHLGIAINLIT

YEDRFALHRIEQELGTEIKPIPKVIDPALYVARPDDDDAADK

>XP_022187879.1 putative ATP-dependent RNA helicase me31b [Nilaparvata lugens]

MMTESHLTNHIALQGKNQMNNTAPGKLDMENEVGWKSKLRLPPPDRRIKTSDVTDTKGNEFEEYCLKRDLLMGIFEKGWE

KPSPIQEASIPIALSGKDVLARAKNGTGKTGAYSIPVLEQVDASKDHIQALVIVPTRELALQTSQICIELAKHMNVRVMV

TTGGTNLRDDILRIYQKVHVIIATPGRILDLMDKNVANMDNCKILVLDEADKLLSQDFKGMLDHVISRLPHERQILLYSA

TFPLTVKQFMEKHLKEPYEINLMEELTLKGVTQYYAFVQERQKVHCLNTLFSKLQINQSIIFCNSTQRVELLAKKITELG

YCCYYIHAKMAQAHRNRVFHDFRAGLCRNLVCSDLFTRGIDVQAVNVVINFDFPKMAETYLHRIGRSGRFGHLGIALNLI

TYEDRFALHRIEQELGTEIKPIPKVIDPDLYVAKLDESIEDANVSK

>XP_021183710.1 putative ATP-dependent RNA helicase me31b [Helicoverpa armigera]

MMTENRISSSNHVGNSMTSQNKGDVDKSIDDIGWKSKLKIPPKDRRIKTSDVTDTRGNEFEEFCLKRELLMGIFEKGWEK

PSPIQEASIPIALSGKDVLARAKNGTGKTGAYCIPVLEQVDPKKDAIQALIVVPTRELALQTSQICIELAKHTDIRVMVT

TGGTNLRDDIMRIYQNVQVIIATPGRMIDLMDKQVAKMDQCRMLVLDEADKLLSQDFKGMLDMVISRLPKERQILLFSAT

FPLSVKQFMEKHLREPYEINLMEELTLKGVTQYYAFVQERQKVHCLNTLFSKLQINQSIIFCNSTQRVELLAKKITELGY

CCYYIHARMAQAHRNRVFHDFRAGLCRNLVCSDLFTRGIDVQAVNVVINFDFPRMAETYLHRIGRSGRFGHLGIAINLIT

YEDRFALHRIEQELGTEIKPIPKVIDPALYVARPDDDDSADK

>KAF2885053.1 hypothetical protein ILUMI_21108 [Ignelater luminosus]

MMTDTLANNHLVSLQTKLDDKMDDIGWKAKLKIPPKDRRIKTSDVTDTRGNEFEEFCLKRELLMGIFEKGWEKPSPIQEA

SIPIALSGKDVLARAKNGTGKTGAYCIPVLEQVDPDKDYIQALIIVPTRELALQTSQICIELAKHMRVRVMVTTGGTNLR

DDIMRIYQKVQVIIATPGRILDLMDKQVAKMEQCRILVLDEADKLLSQDFKGMLDHVISRLPDERQILLFSATFPLTVKQ

FMEKHLRDPYEINLMEELTLKGVTQYYAFVQERQKVHCLNTLFSKLQINQSIIFCNSTQRVELLAKKITELGYCCYYIHA

KMAQAHRNRVFHDFRAGLCRNLVCSDLFTRGIDVQAVNVVINFDFPKMAETYLHRIGRSGRFGHLGIAINLITYDDRFAL

HRIEHELGTEIKPIPKVIDPSLYVANADDELLEDANK

>XP_026732383.1 putative ATP-dependent RNA helicase me31b [Trichoplusia ni]

MMTENRISSSNHVGNSMSSQNKGDVDKSIDDIGWKSKLKIPPKDRRIKTSDVTDTRGNEFEEFCLKRELLMGIFEKGWEK

PSPIQEASIPIALSGKDVLARAKNGTGKTGAYCIPVLEQVDPKKDAIQALIVVPTRELALQTSQICIELAKHTDIRVMVT

TGGTNLRDDIMRIYQNVQVIIATPGRMIDLMDKQVAKMDQCRMLVLDEADKLLSQDFKGMLDMVISRLPKERQILLFSAT

FPLSVKQFMEKHLREPYEINLMEELTLKGVTQYYAFVQERQKVHCLNTLFSKLQINQSIIFCNSTQRVELLAKKITELGY

CCYYIHARMAQAHRNRVFHDFRAGLCRNLVCSDLFTRGIDVQAVNVVINFDFPRMAETYLHRIGRSGRFGHLGIAINLIT

YEDRFALHRIEQELGTEIKPIPKVIDPALYVSRPDDDDSADK

>XP_026485419.1 putative ATP-dependent RNA helicase me31b [Vanessa tameamea]

MMTENRISSSNHVGNMSSQKGDVDKTVEDIGWKSKLKLPPKDRRIKTSDVTDTRGNEFEEFCLKREILMGIFEKGWEKPS

PIQEASIPIALSGKDVLARAKNGTGKTGAYCIPVLEQVDPKKDTIQALVVVPTRELALQTSQICIELAKHTDIRVMVTTG

GTNLRDDIMRIYQNVQVIIATPGRMIDLMDKQVAKMENCRMLVLDEADKLLSQDFKGMLDTVISRLPKERQILLFSATFP

LSVKQFMEKHLKEPYEINLMEELTLKGVTQYYAFVQERQKVHCLNTLFSKLQINQSIIFCNSTQRVELLAKKITELGYCC

YYIHARMAQAHRNRVFHDFRAGLCRNLVCSDLFTRGIDVQAVNVVINFDFPRMAETYLHRIGRSGRFGHLGIAINLITYD

DRFALHRIEQELGTEIKPIPKVIDPALYVARADEDDVGK

>XP_030035898.1 ATP-dependent RNA helicase me31b [Manduca sexta]

MMTENRISSSNHVGNSMSNQNKGDVDKSIDDIGWKAKLKIPPKDRRIKTSDVTDTRGNEFEEFCLKRELLMGIFEKGWEK

PSPIQEASIPIALSGKDILARAKNGTGKTGAYCIPVLEQVDPKKDAIQALIVVPTRELALQTSQICIELAKHTDIRVMVT

TGGTNLRDDIMRIYQNVQVIIATPGRMIDLMDKQVAKMDQCRMLVLDEADKLLSQDFKGMLDMVISRLPKERQILLFSAT

FPLSVKQFMEKHLREPYEINLMEELTLKGVTQYYAFVQERQKVHCLNTLFSKLQINQSIIFCNSTQRVELLAKKITELGY

CCYYIHARMAQAHRNRVFHDFRAGLCRNLVCSDLFTRGIDVQAVNVVINFDFPRMAETYLHRIGRSGRFGHLGIAINLIT

YDDRFALHRIEQELGTEIKPIPKVIDPALYVARPEDDDSADK

>XP_017779942.1 PREDICTED: putative ATP-dependent RNA helicase me31b [Nicrophorus vespilloides]

MMTDTLNSNNLVLSSMPNKGDIEKMDDIGWKAKLKIPPKDKRVKTTDVTDTRGNEFEEFCLKRELLMGIFEKGWEKPSPI

QEASIPIALSGKDVLARAKNGTGKTGAYCIPVLEQIDPKKDCIQALIIVPTRELALQTSQICIELAKHMNIRVMVTTGGT

NLRDDIMRIYQKVQVIIATPGRILDLMDKQVAKMDQCRILVLDEADKLLSSDFKGMLDHVISRLPRERQILLFSATFPLT

VKQFMEKHLRDPYEINLMEELTLKGVTQYYAFVQERQKVHCLNTLFSKLNINQSIIFCNSTQRVELLAKKITELGYCCYY

IHAKMAQAHRNRVFHDFRAGLCRNLVCSDLFTRGIDVQAVNVVINFDFPKMAETYLHRIGRSGRFGHLGIAINLITYEDR

FALHRIEQELGTEIKPIPKVIDPSLYVPSMDDDRLQEEISK

>XP_019869316.1 PREDICTED: putative ATP-dependent RNA helicase me31b [Aethina tumida]

MMTDTISSNNHVLPLSNKADMDKMDDLGWKAKLKIPPKDKRIQTSDVTDTRGNEFEEFCLKRELLMGIFEKGWEKPSPIQ

EASIPIALSGKDILARAKNGTGKTGAYSIPVLEQIDPKKDCIQALIIVPTRELALQTSQICIELAKHLDVRVMVTTGGTN

LRDDIMRIYQKVQVIIATPGRILDLMEKRVAVMDQCKILVLDEADKLLSQDFKGMLDTVIKNLPHERQILLFSATFPLTV

EQFMRKHLRDPYEINLMEELTLKGVTQYYAFVQERQKVHCLNTLFSKLQINQSIIFCNSTQRVELLAKKITELGYCCYYI

HAKMAQAHRNRVFHDFRNGLCRNLVCSDLFTRGIDVQAVNVVINFDFPKMAETYLHRIGRSGRFGHLGIAINLITYDDRF

ALHRIEQELGTEIKPIPKVIDPKLYVAKMLDDEETQQTDGTQK

>XP_015834522.1 PREDICTED: putative ATP-dependent RNA helicase me31b [Tribolium castaneum]

MMTDTLNSNHVMALSGKVDIDTKMDDMGWKAKLKIPPKDRRIQTSDVTDTRGNEFEEFCLKRELLMGIFEKGWEKPSPIQ

EASIPIALSGKDILARAKNGTGKTGAYSIPVLEQIDPKKDCIQALIIVPTRELALQTSQICIELAKHLDVRVMVTTGGTN

LRDDIMRIYQKVQVIIATPGRILDLMEKGVAVMDQCKILVLDEADKLLSQDFKGMLDTVIKNLPQERQILLFSATFPLTV

EQFMRKHLRDPYEINLMEELTLKGVTQYYAFVQERQKVHCLNTLFSKLQINQSIIFCNSTQRVELLAKKITELGYCCYYI

HAKMAQAHRNRVFHDFRAGLCRNLVCSDLFTRGIDVQAVNVVINFDFPKMAETYLHRIGRSGRFGHLGIAINLITYDDRF

ALHRIEQELGTEIKPIPKVIDPKLYVAKLIDDEDTPEDNAK

>XP_018325443.1 putative ATP-dependent RNA helicase me31b [Agrilus planipennis]

MMTDSLSTNNHLSSLSTKIDDKTDDIGWKAKLKIPPKDRRIKTSDVTETRGNEFEEFCLKRELLMGIFEKGWEKPSPIQE

ASIPIALSGKDILARAKNGTGKTGGYSIPVLEQVDTTKDCIQALIIVPTRELALQTSQICIELAKHMNVRIMVTTGGTNL

RDDIMRIYQKVHVIIATPGRILDLMDKGVPNMDLCRILVLDEADKLLSQDFKGMLDHVISKLPKERQILLFSATFPLTVK

QFMEKHLRNPYEINLMEELTLKGVTQYYAFVQERQKVHCLNTLFSKLQINQSIIFCNSTQRVELLAKKITELGYCCYYIH

AKMAQAHRNRVFHDFRNGLCRNLVCSDLFTRGIDVQAVNVVINFDFPKMAETYLHRIGRSGRFGHLGIAINLITYDDRFA

LHRIEQELGTEIKPIPKVIDPALYVTNMEDEPHEEK

>XP_028032435.1 putative ATP-dependent RNA helicase me31b [Bombyx mandarina]

MMTENRISSSNHVGNSISQTKGEVDKSIDDVGWKSKLKIPPKDRRIKTSDVTDTRGNEFEEFCLKRELLMGIFEKGWEKP

SPIQEASIPIALSGKDVLARAKNGTGKTGAYCIPVLEQVDPKKDTIQALIVVPTRELALQTSQICIELAKHTDIRVMVTT

GGTNLRDDIMRIYQNVQVIIATPGRMIDLMDKQVAKMDQCRMLVLDEADKLLSQDFKGMLDMVISRLPKERQILLFSATF

PLSVKQFMEKHLKEPYEINLMEELTLKGVTQYYAFVQERQKVHCLNTLFSKLQINQSIIFCNSTQRVELLAKKITELGYC

CYYIHARMAQAHRNRVFHDFRAGLCRNLVCSDLFTRGIDVQAVNVVINFDFPRMAETYLHRIGRSGRFGHLGIAINLITY

DDRFALHRIEQELGTEIKPIPKVIDPALYVARPEDEDLGDK

>XP_026469178.1 LOW QUALITY PROTEIN: putative ATP-dependent RNA helicase me31b [Ctenocephalides felis]

MDDLGWKSKLKIPPKDRRIKTSDVTDTRGNEFEEFCLKRELLMGIFEKGWEKPSPIQEASIPIALSGKDILARAKNGTGK

TGAYCIPVLEQVDPKKDCIQALVIVPTRELALQTSQICIELAKHMEIRVMVTTGGTNLRDDIMRIYQKVQVIIATPGRIL

DLMDKQVANTEHCRMLVLDEADKLLSQDFKGMLDHVISRLPRERQILLFSATFPLSVKQFMEKHLKEPYEINLMEELTLK

GVTQYYAFVQERQKVHCLNTLFSKLQINQSIIFCNSTQRVELLAKKITELGYCCYYIHAKMAQAHRNRVFHDFRSGLCRN

LVCSDLFTRGIDVQAVNVVINFDFPKXAENYLHRIGRSGRFGHLGIAINLITYEDRFALHRIESEXGTEIKPIPKVIDPS

LYVAKLEGDVPIEENVSQ

>XP_013142670.1 PREDICTED: putative ATP-dependent RNA helicase me31b [Papilio polytes]

MMTENRISSSNHVGNSMNNQTKGDVDKTVEDKGWKSKLKIPPKDRRIKTSDVTDTRGNEFEEFCLKRELLMGIFEKGWEK

PSPIQEAAIPVALSGKDVLARAKNGTGKTGAYCIPVLEQVDPKKDAIQALVVVPTRELALQTSQICIELAKHTDIRVMVT

TGGTNLRDDIMRIYQNVQVIIATPGRMIDLMDKQVAKMDQCRMLVLDEADKLLSQDFKGMLDMVICRLPKERQILLFSAT

FPLSVKQFMEKHLREPYEINLMEELTLKGVTQYYAFVQERQKVHCLNTLFSKLQINQSIIFCNSTQRVELLAKKITELGY

CCYYIHARMAQAHRNRVFHDFRAGLCRNLVCSDLFTRGIDVQAVNVVINFDFPRMAETYLHRIGRSGRFGHLGIAINLIT

YEDRYTLHRIELELNTEIKPIPKVIDPALYVARADEDDSAEK

>XP_034827236.1 ATP-dependent RNA helicase me31b [Aphantopus hyperantus]

MMTENRISSSNHVGNISSQKGDVDKTVDDIGWKAKLKLPPKDRRIKTSDVTDTRGNEFEEFCLKREILMGIFEKGWEKPS

PIQEASIPIALSGKDVLARAKNGTGKTGAYCIPVLEQVDAKKDVIQALIVVPTRELALQTSQICIELAKHTDIRVMVTTG

GTNLRDDIMRIYQNVQVIIATPGRIIDLMDKQVAKMDQCRMLVLDEADKLLSQDFKGMLDTVISRLPKERQILLFSATFP

LSVKQFMEKHLKEPYEINLMEELTLKGVTQYYAFVQERQKVHCLNTLFSKLQINQSIIFCNSTQRVELLAKKITELGYCC

YYIHARMAQAHRNRVFHDFRAGLCRNLVCSDLFTRGIDVQAVNVVINFDFPRMAETYLHRIGRSGRFGHLGIAINLITYE

DRFALHRIEQELGTEIKPIPKVIDPALYVARVADDDADK

>XP_023936543.1 putative ATP-dependent RNA helicase me31b [Bicyclus anynana]

MMTENRISSSNHVGNMSNQKGDVNDKTVDDIGWKAKLKLPPKDRRIKTSDVTDTRGNEFEEFCLKREILMGIFEKGWEKP

SPIQEASIPIALSGKDVLARAKNGTGKTGAYCIPVLEQVDPKKDVIQALIVVPTRELALQTSQICIELAKHTDIRVMVTT

GGTNLRDDIMRIYQNVQVIIATPGRIIDLMDKQVAKMDQCRMLVLDEADKLLSQDFKGMLDTVISRLPKERQILLFSATF

PLSVKQFMEKHLREPYEINLMEELTLKGVTQYYAFVQERQKVHCLNTLFSKLQINQSIIFCNSTQRVELLAKKITELGYC

CYYIHARMAQAHRNRVFHDFRAGLCRNLVCSDLFTRGIDVQAVNVVINFDFPRMAETYLHRIGRSGRFGHLGIAINLITY

EDRFALHRIEQELGTEIKPIPKVIDPALYVSRADDDDGDK

>XP_018572609.1 putative ATP-dependent RNA helicase me31b [Anoplophora glabripennis]

MMTDTLSSNNHVMVLPNKLDMDNKMDDLGWKSKLKIPPKDKRIQTSDVTDTRGNEFEEFCLKRELLMGIFEKGWEKPSPI

QEAAIPIALSGKDVLARAKNGTGKTGAYSIPVLEQIDPKKDCIQALIIVPTRELALQTSQICIELAKHLEVRVMVTTGGT

NLRDDIMRIYQKVQVIIATPGRILDLMEKGVAVMDQCKILVLDEADKLLSQDFKGMLDTVIKNLPQERQILLFSATFPLT

VEQFMRKHLRDPYEINLMEELTLKGVTQYYAFVQERQKVHCLNTLFSKLQINQSIIFCNSTQRVELLAKKITELGYCCYY

IHAKMAQAHRNRVFHDFRAGLCRNLVCSDLFTRGIDVQAVNVVINFDFPKMAETYLHRIGRSGRFGHLGIAINLITYDDR

FTLHRIEQELGTEIKPIPKVIDPKLYVSKSGDEEELEEVAK

>XP_014222343.1 putative ATP-dependent RNA helicase me31b isoform X2 [Trichogramma pretiosum]

MEKLDDVGWKAKLKIPPKDKRIKTSDVTDTRGNEFDEFCLKRELLMGIFEKGWEKPSPIQEASIPIALSGKDVLARAKNG

TGKTGAYSIPVLEQVDPKRDVIQALIIVPTRELALQTSQILIELAKHMDVKVMVTTGGTNLRDDIMRIYQKVQVIIATPG

RILDLMDKNVANMEYCKILVLDEADKLLSQDFKGMLDHVISRLPQERQILLYSATFPLTVKQFMEKHLKDPYEINLMEEL

TLKGVTQYYAFVQERQKVHCLNTLFSKLQINQSIIFCNTTQRVELLAKKITDLGYCCYYIHAKMAQAHRNRVFHDFRAGL

CRNLVCSDLFTRGIDVQAVNVVINFDFPKMAETYLHRIGRSGRFGHLGIAINLITYEDRFNLHRIEQELGTEIKPIPKVI

DPCLYVARPEDNSILEEGNVSK

>CAB3223627.1 unnamed protein product [Arctia plantaginis]

MMTENRISSSNYVGNSMTSQNKGDVDKSIDDIGWKAKLKIPPKDRRIKTSDVTDTRGNEFEEFCLKRELLMGIFEKGWEK

PSPIQEASIPIALSGKDVLARAKNGTGKTGAYCIPVLEQVDPKKDAIQALVVVPTRELALQTSQICIELAKHTDIRVMVT

TGGTNLRDDIMRIYQNVQVIIATPGRMIDLMDKQVAKMDQCRMLVLDEADKLLSQDFKGMLDMVISRLPKERQILLFSAT

FPLSVKQFMEKHLREPYEINLMEELTLKGVTQYYAFVQERQKVHCLNTLFSKLQINQSIIFCNSTQRVELLAKKITELGY

CCYYIHARMAQAHRNRVFHDFRAGLCRNLVCSDLFTRGIDVQAVNVVINFDFPRMAETYLHRIGRSGRFGHLGIAINLIT

YEDRFALHRIEQELGTEIKPIPKVIDPALYVSRPDDDDSADK

>KAF5296967.1 hypothetical protein FQR65_LT10119 [Abscondita terminalis]

MMTDTLTTNNHVVPLQTKLDDKLDDIGWKSKLKVPPKDKRIKTSDVTDTRGNEFEEFCLKRELLMGIFEKGWEKPSPIQE

ASIPIALSGKDVLARAKNGTGKTGAYCIPVLEQVDPDKDYIQALILVPTRELALQTSQICIELAKHMRVRVMVTTGGTNL

RDDIMRIYQKVQVIIATPGRILDLMDKQVAKMDQCRILVLDEADKLLSQDFKDMLHHLIARLPDERQILLFSATFPLTVK

QFMEKHLRDPYEINLMEELTLKGVTQYYAFVQERQKVHCLNTLFSKLQINQSIIFCNSTQRVELLAKKITELGYCCYYIH

AKMAQAHRNRVFHDFRAGLCRNLVCSDLFTRGIDVQAVNVVINFDFPKMAETYLHRIGRSGRFGHLGIAINLITYDDRFA

LHRIEQELGTEIKPIPKMIDPSLYVANSEDELLEDSNK

>XP_032514876.1 ATP-dependent RNA helicase me31b [Danaus plexippus plexippus]

MMTENRISSSNHVGNMSSQKGEVDKTVEDIGWKAKLKLPPKDRRLKTSDVTDTRGNEFEEFCLKREILMGIFEKGWEKPS

PIQEASIPIALSGKDVLARAKNGTGKTGAYCIPVLEQVDPKKDAIQALIVVPTRELALQTSQICIELAKHTDIRVMVTTG

GTNLRDDIMRIYQNVQVIIATPGRMIDLMDKQVAKMDQCRMLVLDEADKLLSQDFKGMLDTVISRLPKERQILLFSATFP

LNVKKFMEKHLREPYEINLMEELTLKGVTQYYAFVQERQKVHCLNTLFSKLQINQSIIFCNSTQRVELLAKKITELGYCC

YYIHARMAQAHRNRVFHDFRAGLCRNLVCSDLFTRGIDVQAVNVVINFDFPRMAETYLHRIGRSGRFGHLGIAINLITYE

DRFALHRIEQELGTEIKPIPKVIDPALYVSRPDEDDSADK

>XP_012545299.1 putative ATP-dependent RNA helicase me31b [Bombyx mori]

MMTENRISSSNHVGNSISQTKGEVDKSIDDVGWKSKLKIPPKDRRIKIDVTDTRGNEFEEFCLKRELLMGIFEKGWEKPS

PIQEASIPIALSGKDVLARAKNGTGKTGAYCIPVLEQVDPKKDTIQALIVVPTRELALQTSQICIELAKHTDIRVMVTTG

GTNLRDDIMRIYQNVQVIIATPGRMIDLMDKQVAKMDQCRMLVLDEADKLLSQDFKGMLDMVISRLPKERQILLFSATFP

LSVKQFMEKHLKEPYEINLMEELTLKGVTQYYAFVQERQKVHCLNTLFSKLQINQSIIFCNSTQRVELLAKKITELGYCC

YYIHARMAQAHRNRVFHDFRAGLCRNLVCSDLFTRGIDVQAVNVVINFDFPRMAETYLHRIGRSGRFGHLGIAINLITYD

DRFALHRIEQELGTEIKPIPKVIDPALYVARPEDEDLGDK

>XP_001659287.1 putative ATP-dependent RNA helicase me31b [Aedes aegypti]

MMTETLNSNNHLSQKGENKMDDMGWKAKLKLPPKDNRIKTSDVTDTRGNEFEEFCLKRELLMGIFEKGWEKPSPIQEAAI

PIALVGKDILARAKNGTGKTGAYSIPVLEQIDPTKDYIQALIIVPTRELALQTSQICIELAKHMHIRVMVTTGGTNLKDD

IMRIYQKVQVIIATPGRILDLMDKEVANMANCRMLVLDEADKLLSQDFKGMLDHVIMKLPKERQILLFSATFPLSVKNFM

EKHLREPYEINLMEELTLKGVTQYYAFVQERQKVHCLNTLFSKLQINQSIIFCNSTQRVELLAKKITELGYCCYYIHAKM

QQAHRNRVFHDFRSGLCRNLVCSDLFTRGIDVQAVNVVINFDFPKMAETYLHRIGRSGRFGHLGIAINLITYEDRFDLHR

IEKELGTEIKPIPKVIDPALYVPRPEDQNSTQEEQNISK

>XP_019561697.1 ATP-dependent RNA helicase me31b [Aedes albopictus]

MMTETLNSNNHLSQKGENKMDDMGWKAKLKLPPKDNRVKTSDVTDTRGNEFEEFCLKRELLMGIFEKGWEKPSPIQEAAI

PIALVGKDILARAKNGTGKTGAYSIPVLEQIDPTKDYIQALIIVPTRELALQTSQICIELAKHMHIRVMVTTGGTNLKDD

IMRIYQKVQVIIATPGRILDLMDKEVANMANCRMLVLDEADKLLSQDFKGMLDHVIMKLPKERQILLFSATFPLSVKNFM

EKHLREPYEINLMEELTLKGVTQYYAFVQERQKVHCLNTLFSKLQINQSIIFCNSTQRVELLAKKITELGYCCYYIHAKM

QQAHRNRVFHDFRSGLCRNLVCSDLFTRGIDVQAVNVVINFDFPKMAETYLHRIGRSGRFGHLGIAINLITYEDRFDLHR

IEKELGTEIKPIPKVIDPALYVPRPEDQNSTQEEQNISK

>XP_035782856.1 ATP-dependent RNA helicase me31b [Anopheles albimanus]

MMTETLNSNNHLSQKGENNKVGDMGWKAKLKIPPKDTRIKTSDVTDTRGNEFEEFCLKRPLLMGIFEKGWEKPSPIQEAA

IPIALVGKDILARAKNGTGKTGAYSIPVLEQVDPTKDYIQALIIVPTRELALQTSQICIELAKHMNIRVMVTTGGTNLKD

DIMRIYQKVQVIIATPGRILDLMDKEVANMSQCRMLVLDEADKLLSQDFKGMLDHVIMRLPKERQILLFSATFPLSVKNF

MEKHLRDPYEINLMEELTLKGVTQYYAFVQERQKVHCLNTLFSKLQINQSIIFCNSTQRVELLAKKITELGYCCYYIHAR

MQQAHRNRVFHDFRSGLCRNLVCSDLFTRGIDVQAVNVVINFDFPKMAETYLHRIGRSGRFGHLGIAINLITYEDRFDLH

RIEKELGTEIKPIPKVIDPALYVPRPDDPNSAQEEQNVSK

>XP_017793232.1 PREDICTED: putative ATP-dependent RNA helicase me31b [Habropoda laboriosa]

MMTETHINSNHVLNSGLNTKSEIEKMDDVGWKAKLKIPPKDKRIKTSDVTDTRGNEFEEFCLKRELLMGIFEKGWEKPSP

IQEASIPIALSGKDILARAKNGTGKTGAYSIPVLEQVDPRQDVIQALVIVPTRELALQTSQICIELAKHMDIKVMVTTGG

TDLRDDIMRIYQKVHVIIATPGRILDLMDKSVANMDHCKILVLDEADKLLSQDFKGMLDHVISRLPHERQILLYSATFPL

TVKQFMEKHLRDPYEINLMEELTLKGVTQYYAFEFLFFKLQITQSIIFCNSTQRVELLAKKITDLGYCCYYIHAKMAQAH

RNRVFHDFRAGLCRNLVSSDLFTRGIDVQAVNVVINFDFPKMAETYLHRIGRSGRFGHLGIAINLITYEDRFNLHRIEQE

LGTEIKPIPKVIDPSLYVARPEDNNSMEEANVSK

>XP_031346771.1 ATP-dependent RNA helicase me31b [Photinus pyralis]

MMTDTLTTNNHVVPLQTKLDDKLDDIGWKSKLKVPPKDKRIKTSDVTDTRGNEFEEFCLKRELLMGIFEKGWEKPSPIQE

ASIPIALSGKDVLARAKNGTGKTGAYCIPVLEQVDPDKDYIQALILVPTRELALQTSQICIELAKHLRVRVMVTTGGTNL

RDDIMRIYQKVQVIIATPGRILDLMDKQVAKMEQCRILVLDEADKLLSQDFKDMLHHLIGRLPEERQILLFSATFPLTVK

LFMEKHLRDPYEINLMEELTLKGVTQYYAFVQERQKVHCLNTLFSKLQINQSIIFCNSTQRVELLAKKITELGYCCYYIH

AKMAQAHRNRVFHDFRAGLCRNLVCSDLFTRGIDVQAVNVVINFDFPKMAETYLHRIGRSGRFGHLGIAINLITYDDRFA

LHRIEQELGTEIKPIPKMIDPSLYVANSEDELLEDGNK

>XP_035914743.1 ATP-dependent RNA helicase me31b [Anopheles stephensi]

MMTETLNSNNHLSQKGENNKVGDMGWKAKLKIPPKDTRVKTSDVTDTRGNEFEEFCLKRPLLMGIFEKGWEKPSPIQEAA

IPIALVGKDILARAKNGTGKTGAYSIPVLEQVDPTKDYIQALIIVPTRELALQTSQICIELAKHMNIRVMVTTGGTNLKD

DIMRIYQKVQVIIATPGRILDLMDKEVANMSQCRMLVLDEADKLLSQDFKGMLDHVIMRLPKERQILLFSATFPLSVKNF

MEKHLRDPYEINLMEELTLKGVTQYYAFVQERQKVHCLNTLFSKLQINQSIIFCNSTQRVELLAKKITELGYCCYYIHAR

MQQAHRNRVFHDFRSGLCRNLVCSDLFTRGIDVQAVNVVINFDFPKMAETYLHRIGRSGRFGHLGIAINLITYEDRFDLH

RIEKELGTEIKPIPKVIDPALYVPRPDDPNSAQEEQNVSK

>CAB3359348.1 Hypothetical predicted protein [Cloeon dipterum]

MGSKLDSDKMDDIGWKSKLKIPPKDKRIKTSDVTDTRGNEFEEFCLKRELLMGIFEKGWEKPSPIQEASIPIALTGKDVL

ARAKNGTGKTGAYCIPVLENIDSKKETIQALVIVPTRELALQTSQICIELSKHMHIKVMVTTGGTNLRDDIMRIFQKVQV

VIATPGRILDLMEKKVANMDNCKVLVLDEADKLLSQDFKGMLDTVIAKLPKERQILLYSATFPLTVKNFMDKHLKSPYEI

NLMEELTLKGVTQYYAFVQERQKVHCLNTLFSKLQINQSIIFCNSTQRVELLAKKITELGYCCYYIHAKMAQAHRNRVFH

DFRAGLCRNLVCSDLFTRGIDVQAVNVVINFDFPKMAETYLHRIGRSGRFGHLGIAINLITYEDRFALHRIEQELGTEIK

PIPKVIDPSLYVAKFDEHGDHEDGNTNVAIVISANLVEKSKEGLIEEKKS

>KFB47731.1 DEAD box ATP-dependent RNA helicase [Anopheles sinensis]

MMTETLNSNNHLSQKGENNKVGDMGWKAKLKIPPKDTRIKTSDVTDTRGNEFEEFCLKRPLLMGIFEKGWEKPSPIQEAA

IPIALVGKDILARAKNGTGKTGAYSIPVLEQVDPTRDYIQALIIVPTRELALQTSQICIELAKHMHIRVMVTTGGTNLKD

DIMRIYQKVQVIIATPGRILDLMDKEVANMSQCRMLVLDEADKLLSQDFKGMLDHVIMRLPKERQILLFSATFPLSVKNF

MEKHLRDPYEINLMEELTLKGVTQYYAFVQERQKVHCLNTLFSKLQINQSIIFCNSTQRVELLAKKITELGYCCYYIHAR

MQQAHRNRVFHDFRSGLCRNLVCSDLFTRGIDVQAVNVVINFDFPKMAETYLHRIGRSGRFGHLGIAINLITYEDRFDLH

RIEKELGTEIKPIPKVIDPALYVPRPDDQNSTQEEQNVSK

>XP_014273138.1 putative ATP-dependent RNA helicase me31b [Halyomorpha halys]

MMTESQFATKPLAASPLIPSKPIKKLEPEESWKAKLKIPPTDRRKKTSDVTDTKGNEFEEFCLKRELLMGIFEKGWEKPS

PIQEASIPIALSGRNILARAKNGTGKTGAYSIPVLEQVDPGLDQIQALVIVPTRELALQTSQICIELAKHMNIKVMVTTG

GTNLRDDIMRIFQKVQVIIATPGRILDLMDKKVAHMDHCRILVLDEADKLLSQDFKGMLDHVISRLPKDRQILLYSATFP

LTVKQFMDKHLKDPYEINLMEELTLKGVTQYYAFVQERQKVHCLNTLFSKLQINQSIIFCNSTQRVELLAKKITELGYCC

YYIHAKMAQAHRNRVFHDFRSGLCRNLVCSDLFTRGIDVQAVNVVINFDFPKMAETYLHRIGRSGRFGHLGIAINLITYE

DRFALHRIEQELGTEIKPIPKVIDPSLYVAKLEDGIEDGNYK

>XP_025424726.1 putative ATP-dependent RNA helicase me31b [Sipha flava]

MIATTNHNSSGINLSMPSKQMNRISFEGIDDAGWKAKLKLPPQDKRIKTSDVTSTKGNDFEEFCLKRELLMGIFEKGWEK

PSPIQEASIPIALSGKDILARAKNGTGKTGAYSIPVLEQVDPKLDVIQALVIVPTRELALQTSQICIELAKHLDIRVMVT

TGGTNLKDDILRIYQRVHVIIATPGRILDLLDKSIAKVDHCRILVLDEADKLLSQDFKGMLDHIISRLPSERQILLYSAT

FPLTVKQFMDKHLRSPYEINLMEELTLKGVTQYYAFVQEKQKVHCLNTLFSKLQINQSIIFCNSTQRVELLAKKITDLGY

CCYYIHAKMAQAHRNRVFHDFRKGSCRNLVCSDLFTRGIDVQAVNVVINFDFPKMAETYLHRIGRSGRFGHLGIAINLIT

YDDRFALHRIEQELGTEIKPIPKVIDPRLYVARPEDVDISEEMELSK

>XP_028150016.1 putative ATP-dependent RNA helicase me31b [Diabrotica virgifera virgifera]

MTETIKSNNNHLMGLPNTVDYDNKVDDLGWKAKLKIPPKDRRIQTSDVTDTRGNEFEEFCLKRELLMGIFEKGWEKPSPI

QEASIPIALSGKDVLARAKNGTGKTGAYSIPVLEQIDPKKDCIQALIIVPTRELALQTSQICIELAKYLEVRVMVTTGGT

NLRDDIMRIYQKVQVIIATPGRILDLMEKGVAVMDQCKILVLDEADKLLSQDFKGMLDTVIKNLPQERQILLFSATFPLT

VEQFMRKHLRDPYEINLMEELTLKGVTQYYAFVQERQKVHCLNTLFSKLQINQSIIFCNSTQRVELLAKKITELGYSCYY

IHAKMAQAHRNRVFHDFRSGLCRNLVCSDLFTRGIDVQAVNVVINFDFPKMAETYLHRIGRSGRFGHLGIAINLITYDDR

FSLHRIEQELGTEIKPIPKVIDPKLYVPKVDDEEIETGK

>GFG37431.1 hypothetical protein Cfor_07164, partial [Coptotermes formosanus]

EVEKIDDIGWKAKLKIPPKDRRVQTSDVTDTRGNEFEEFCLKRELLMGIFEKGWEKPSPIQEASIPIALSGKDVLARAKN

GTGKTGAYSIPVLEQVDPKKDSIQALVIVPTRELALQTSQICIELAKHMDVKVMVTTGGTNLRDDIMRIYQKVQVIIATP

GRILDLMDKNVAIMDSCRMLVLDEADKLLSQDFKGMLDHVISRLPKERQILLFSATFPLTVKQFMEKHLREPYEINLMEE

LTLKGVTQYYAFVQERQKVHCLNTLFSKLQINQSIIFCNSTQRVELLAKKITELGYCCYYIHAKMAQAHRNRVFHDFRAG

LCRNLVCSDLFTRGIDVQAVNVVINFDFPKMAETYLHRIGRSGRFGHLGIAINLITYEDRFALHRIEQELGTEIKPIPK

>VVC38603.1 RNA helicase, DEAD-box type, Q motif,Helicase, C-terminal,ATP-dependent RNA helicase DEAD-box, conserved [Cinara cedri]

MIATNHSSTGLSLGMPIKQMNRISFEGIDDAGWKAKLKLPPQDKRIKTSDVTSTKGNDFEEFCLKRELLMGIFEKGWEKP

SPIQEASIPIALSGKDILARAKNGTGKTGAYSIPVLEQVDPKLDVIQALVIVPTRELALQTSQICIELAKHLDIRVMVTT

GGTNLKDDILRIYQRVHVIIATPGRILDLLDKSIAKVDHCRILVLDEADKLLSQDFKGMLDHIISKLPSERQILLYSATF

PLTVKQFMDKHLRSPYEINLMEELTLKGVTQYYAFVQEKQKVHCLNTLFSKLQINQSIIFCNSTQRVELLAKKITDLGYC

CYYIHAKMAQAHRNRVFHDFRKGSCRNLVCSDLFTRGIDVQAVNVVINFDFPKMAETYLHRIGRSGRFGHLGIAINLITY

DDRFALHRIEQELGTEIKPIPKVIDPRLYVARPEDVDINEEMDLSK

>KAF7284573.1 hypothetical protein GWI33_021954 [Rhynchophorus ferrugineus]

MITDRINTNHLTLNKVEFDSKMENKVDSKIDDLGWKSKLKIPPKDRRIQTSDVTNTRGNEFEEFCLKRELLMGIFEKGWE

KPSPIQEVAIPITLTGRDILARAKNGTGKTGAYSIPVLEQINPTKDCIQALIIVPTRELALQTSQICIELAKHLQIRIMV

TTGGTNLRDDIMRIYQKVQVIIATPGRILDLMEKGVATMDQCKILVLDEADKLLSQDFKGMLDTVIKNLPQERQILLFSA

TFPLTVEQFMKKHLKDPYEINLMEELTLKGVTQYYAFVQERQKVHCLNTLFSKLQINQSIIFCNSTQRVELLAKKITELG

YCCYYIHAKMAQAHRNRVFHDFRAGLCRNLVCSDLFTRGIDVQAVNVVINFDFPKMAETYLHRIGRSGRFGHLGIAINLI

TYDDRFALHRIEQELGTEIKPIPKIIDPKLYVAKLEDDDGEENSK

>CRK86372.1 CLUMA_CG000042, isoform A [Clunio marinus]

MNRKDLKNQVRNNNHNNNNNKMMTETMNSNNHLSQADPGWKSKLQIPPKDNRIKTSDVTDTRGNEFEEFCVKRELLMGVF

EMGWEKPSPIQEAAIPIALGGKDILARAKNGTGKTGAYCIPILEQVDEKRDCIQALVIVPTRELALQTSQICIELAKHLN

IKVMVTTGGTNLKDDIMRLYQKVQVVIATPGRILDLMDKNVANMENCKVLVLDEADKLLSQDFKGMLDHVIQKLPRERQI

LLFSATFPLTVKQFMDKHLRNPYEINLMEELTLKGVTQYYAFVQERQKVHCLNTLFSKLQINQSIIFCNSTQRVELLAKK

ITELGYCCYYIHAKMAQVHRNRVFHDFRSGLCRNLVCSDLFTRGIDVQAVNVVINFDFPKMAETYLHRIGRSGRFGHLGI

AINLITYEDRYDLHRIEKELGTEIKPIPKVIDPALYVASKLPEDQEDQNANK

>XP_023018290.1 putative ATP-dependent RNA helicase me31b [Leptinotarsa decemlineata]

MMMTDTLKSNNHIMGLSTKLDLDNKMDDLGWKSKLKIPPKDKRFQTSDVTDTRGNEFEEFCLKRELLMGIFEKGWEKPSP

IQEASIPIALSGKDVLARAKNGTGKTGAYCIPVLEQIDPKKDCIQALIIVPTRELALQTSQICIELAKYLEVRVMVTTGG

TNLRDDIMRIYQKVQVIIATPGRILDLMEKRVAVMDQCKILVLDEADKLLSQDFKGMLDVVIKNLPHERQILLFSATFPL

TVEQFMRKHLRDPYEINLMDELTLIGVTQYYAFVQERQKVHCLNTLFSKLQINQSIIFCNSTQRVELLAKKITELGYSCY

YIHAKMAQAHRNRVFHDFRSGLCRNLVCSDLFTRGIDVQAVNVVINFDFPKMAETYLHRIGRSGRFGHLGIAINLITYDD

RFSLHRIEQELGTEIKPIPKVIDPKLYVAKMLEEEEIESSK

>XP_022182727.1 putative ATP-dependent RNA helicase me31b isoform X1 [Myzus persicae]

MLATNHNNSGINLSMPSKQLNRISFEGIDDAGWKAKLKLPPQDHRIKTSDVTSTKGNDFEEFCLKRELLMGIFEKGWEKP

SPIQEASIPIALSGKDILARAKNGTGKTGAYSIPVLEQVDPKLDVIQALVIVPTRELALQTSQICIELAKHLDIRVMVTT

GGTNLKDDILRIYQRVHVIIATPGRILDLLDKSIAKVDHCRILVLDEADKLLSQDFKGMLDHIISRLPSERQILLYSATF

PLTVKQFMDKHLRSPYEINLMEELTLKGVTQYYAFVQEKQKVHCLNTLFSKLQINQSIIFCNSTQRVELLAKKITDLGYC

CYYIHAKMAQAHRNRVFHDFRKGSCRNLVCSDLFTRGIDVQAVNVVINFDFPKMAETYLHRIGRSGRFGHLGIAINLITY

DDRFALHRIEQELGTEIKPIPKVIDPRLYVARPEDVDINEEMDLSK

>XP_001951330.2 ATP-dependent RNA helicase me31b isoform X1 [Acyrthosiphon pisum]

MLATNHNNSGINLNMPSKQLNRISFEGIDDAGWKAKLKLPPQDHRIKTSDVTSTKGNDFEEFCLKRELLMGIFEKGWEKP

SPIQEASIPIALSGKDILARAKNGTGKTGAYSIPVLEQVDPKLDVIQALVIVPTRELALQTSQICIELAKHLDIRVMVTT

GGTNLKDDILRIYQRVHVIIATPGRILDLLDKSIAKVDHCRILVLDEADKLLSQDFKGMLDHIISRLPSERQILLYSATF

PLTVKQFMDKHLRSPYEINLMEELTLKGVTQYYAFVQEKQKVHCLNTLFSKLQINQSIIFCNSTQRVELLAKKITDLGYC

CYYIHAKMAQAHRNRVFHDFRKGSCRNLVCSDLFTRGIDVQAVNVVINFDFPKMAETYLHRIGRSGRFGHLGIAINLITY

DDRFALHRIEQELGTEIKPIPKVIDPRLYVARPEDVDINEEMDLSK

>VVC38602.1 Hypothetical protein CINCED_3A019670 [Cinara cedri]

MIATNHSSTGLSLGMPIKQMNRIFEGIDDAGWKAKLKLPPQDKRIKTSDVTSTKGNDFEEFCLKRELLMGIFEKGWEKPS

PIQEASIPIALSGKDILARAKNGTGKTGAYSIPVLEQVDPKLDVIQALVIVPTRELALQTSQICIELAKHLDIRVMVTTG

GTNLKDDILRIYQRVHVIIATPGRILDLLDKSIAKVDHCRILVLDEADKLLSQDFKGMLDHIISKLPSERQILLYSATFP

LTVKQFMDKHLRSPYEINLMEELTLKGVTQYYAFVQEKQKVHCLNTLFSKLQINQSIIFCNSTQRVELLAKKITDLGYCC

YYIHAKMAQAHRNRVFHDFRKGSCRNLVCSDLFTRGIDVQAVNVVINFDFPKMAETYLHRIGRSGRFGHLGIAINLITYD

DRFALHRIEQELGTEIKPIPKVIDPRLYVARPEDVDINEEMDLSK

>XP_018898446.1 PREDICTED: putative ATP-dependent RNA helicase me31b [Bemisia tabaci]

MIMMENHIGSNHALSAKNSSTKFDADKDESWKSKLKIPPPDRRIKTSDVTDTRGIEFSEFCLKRELLMGIFEKGWEKPSP

IQEATIPIALTGKDILARAKNGTGKTGAYCIPVLEQINPKLDHIQALIMVPTRELALQTSQICMELSKHMDIKVMVTTGG

TNLKDDIMRIYDKVHVIIATPGRILDLMDKHVANMEHCKILVLDEADKLLSQDFQGMLDHVISKLPQERQIFLFSATFPL

TVKNFMDKHLRNPYEVNLMEELTLKGVTQYYAFVQERQKVHCLNTLFSKLQINQSIIFCNSTQRVELLAKKITDLGYCCY

YIHAKMAQAHRNRVFHDFRKGVCRNLVCSDLFTRGIDVQAVNVVINFDFPKMAETYLHRIGRSGRFGHLGIAINLITYED

RFNLHRIEQELGTEIKPIPKQIDPSLYVARTPENDEQDVSK

>XP_019757099.1 PREDICTED: putative ATP-dependent RNA helicase me31b isoform X1 [Dendroctonus ponderosae]

MMTDRINMNHLTLNSKMDADVKMELKDDLGWKAKLKIPAKDHRIQTTDVTDTRGNEFEEYCLKRELLMGIFEKGWEKPSP

IQEAAIPIALTGKDVLARAKNGTGKTGAYSIPVLEQIDPTKDCIQALVIVPTRELALQTSQICIELAKHLLIRIMVTTGG

TNLRDDIMRIYQKVQVIIATPGRILDLMEKGVAQMDQCKILVLDEADKLLSQDFKGMLDVVIKNLPEKRQVLLFSATFPL

TVEQFMRKHLRSPYEINLMEELTLKGVTQYYAFVQERQKVHCLNTLFSKLQINQSIIFCNSTQRVELLAKKITELGYCCY

YIHAKMAQTHRNRVFHDFRSGLCRNLVCSDLFTRGIDVQAVNVVINFDFPKMAETYLHRIGRSGRFGHLGIAINLITYED

RFALHRIEQELGTEIKPIPKIIDPRLYVAKLGEEDPEDLVK

>XP_319893.3 AGAP009135-PA, partial [Anopheles gambiae str. PEST]

IYSLFSENNKVGDMGWKAKLKIPPKDTRVKTSDVTDTRGNEFEEFCLKRPLLMGIFEKGWEKPSPIQEAAIPIALVGKDI

LARAKNGTGKTGAYSIPVLEQVDPTKDYIQALIIVPTRELALQTSQICIELAKHMNIRVMVTTGGTNLKDDIMRIYQKVQ

VIIATPGRILDLMDKEVANMSQCRMLVLDEADKLLSQDFKGMLDHVIMRLPKERQILLFSATFPLSVKNFMEKHLRDPYE

INLMEELTLKGVTQYYAFVQERQKVHCLNTLFSKLQINQSIIFCNSTQRVELLAKKITELGYCCYYIHARMQQAHRNRVF

HDFRSGLCRNLVCSDLFTRGIDVQAVNVVINFDFPKMAETYLHRIGRSGRFGHLGIAINLITYEDRFDLHRIEKELGTEI

KPIPKVIDPALYVPRPDDPNSTQEEQNVSK

>XP_022182728.1 putative ATP-dependent RNA helicase me31b isoform X2 [Myzus persicae]

MLATNHNNSGINLSMPSKQLNRIFEGIDDAGWKAKLKLPPQDHRIKTSDVTSTKGNDFEEFCLKRELLMGIFEKGWEKPS

PIQEASIPIALSGKDILARAKNGTGKTGAYSIPVLEQVDPKLDVIQALVIVPTRELALQTSQICIELAKHLDIRVMVTTG

GTNLKDDILRIYQRVHVIIATPGRILDLLDKSIAKVDHCRILVLDEADKLLSQDFKGMLDHIISRLPSERQILLYSATFP

LTVKQFMDKHLRSPYEINLMEELTLKGVTQYYAFVQEKQKVHCLNTLFSKLQINQSIIFCNSTQRVELLAKKITDLGYCC

YYIHAKMAQAHRNRVFHDFRKGSCRNLVCSDLFTRGIDVQAVNVVINFDFPKMAETYLHRIGRSGRFGHLGIAINLITYD

DRFALHRIEQELGTEIKPIPKVIDPRLYVARPEDVDINEEMDLSK

>XP_008187802.1 ATP-dependent RNA helicase me31b isoform X2 [Acyrthosiphon pisum]

MLATNHNNSGINLNMPSKQLNRIFEGIDDAGWKAKLKLPPQDHRIKTSDVTSTKGNDFEEFCLKRELLMGIFEKGWEKPS

PIQEASIPIALSGKDILARAKNGTGKTGAYSIPVLEQVDPKLDVIQALVIVPTRELALQTSQICIELAKHLDIRVMVTTG

GTNLKDDILRIYQRVHVIIATPGRILDLLDKSIAKVDHCRILVLDEADKLLSQDFKGMLDHIISRLPSERQILLYSATFP

LTVKQFMDKHLRSPYEINLMEELTLKGVTQYYAFVQEKQKVHCLNTLFSKLQINQSIIFCNSTQRVELLAKKITDLGYCC

YYIHAKMAQAHRNRVFHDFRKGSCRNLVCSDLFTRGIDVQAVNVVINFDFPKMAETYLHRIGRSGRFGHLGIAINLITYD

DRFALHRIEQELGTEIKPIPKVIDPRLYVARPEDVDINEEMDLSK

>XP_014250284.1 putative ATP-dependent RNA helicase me31b [Cimex lectularius]

MMTELSTNHIGANRMPSKKLGDMEDGWKAKLKLPPPDRRKKTSDVTDTKGNEFEEFCLKRELLMGIFEKGWEKPSPIQEA

SIPIALSGRNILARAKNGTGKTGAYSIPVLEQVDPTVEKIQALVIVPTRELALQTSQICIELAKHMDVKVMVTTGGTNLR

DDIMRLYQKVHVVIATPGRILDLMDKNVANMDNCRMLVLDEADKLLSQDFKGMLDLVICRLPKDRQILLYSATFPLTVKQ

FMDKHLEHPYEINLMEELTLKGVTQYYAFVQERQKVHCLNTLFSKLQITQSIIFCNSTQRVELLAKKITELGYCCYYIHA

KMAQAHRNRVFHDFRSGLCRNLVCSDLFTRGIDVQAVNVVINFDFPRMAETYLHRIGRSGRFGHLGIAINLITFEDRLSL

HRIEQELGTEIKPIPKVIDPSLYVAKLEDGIEETNNANK

>XP_030753547.1 ATP-dependent RNA helicase me31b [Sitophilus oryzae]

MITDRINMNHLTLNKLSLDAKMDSKVDSKLDDLGWKSKLKIPPKDRRIQTSDVTDTKGNEFEEFCLNRQLLMGIFEKGWE

KPSPIQEAAIPIALSGKDILARAKNGTGKTGAYSIPVLELIDPTKDYIQALVIVPTRELALQTSQICIELAKHLQIRIMV

TTGGTNLRDDIMRIYQKVQVIIATPGRILDLMEKEVAQMSNCKILVLDEADKLLSQDFKGMLDTVIKNLPRERQILLFSA

TFPLTVEQFMKKHLHEPYEINLMEELTLKGVTQYYAFVQERQKVHCLNTLFSKLQINQSIIFCNSTQRVELLAKKITELG

YCCYYIHAKMAQAHRNRVFHDFRAGLCRNLVCSDLFTRGIDVQAVNVVINFDFPKMAETYLHRIGRSGRFGHLGIAINLI

TYDDRFALHRIEQELGTEIKPIPKIIDPKLYVAKMSIDDDIEENSK

>XP_031624940.1 ATP-dependent RNA helicase me31b [Contarinia nasturtii]

MMTENMSKNHLANHALPKSDGKLAMPTGNELDITGWKSKLKVPPKDNRIKTSDVTDTRGNEFEEFCLKRELLMGIFEKGW

EKPSPIQEAAIPVALGGKDILARAKNGTGKTGAYSIPVLEQIDSTKDCIQALIIVPTRELALQTSQICIELAKHLNIRVM

VTTGGTVLKDDIMRIYQKVQLIIATPGRILDLIDKEVANMSQCRMLVLDEADKLLSQDFKGMLDHVIMKLPKELQILLFS

ATFPLSVKNFMDKHLRDPYEINLMEELTLKGVTQYYAFVQERQKVHCLNTLFSKLQINQSIIFCNSTQRVELLAKKITEL

GYCCYYIHAKMAQAHRNRVFHDFRQGLCRNLVCSDLFTRGIDVQAVNVVINFDFPKMAETYLHRIGRSGRFGHLGIAINL

ITYEDRFNLHRIEKELGTEIKPIPKVIDPALYVASAPNDSLSSNNESIVSK

>XP_015376864.1 PREDICTED: putative ATP-dependent RNA helicase me31b isoform X1 [Diuraphis noxia]

MLAANHNNSGINLNMPSKQLNRISFEGIDDAGWKAKLKLPPQDHRIKTSDVTSTKGNDFEEFCLKRELLMGIFEKGWEKP

SPIQEASIPIALSGKDILARAKNGTGKTGAYSIPVLEQVDPKLDVIQALVIVPTRELALQTSQICIELAKHLDIRVMVTT

GGTNLKDDILRIYQRVHVIIATPGRILDLLDKSIAKVDNCRILVLDEADKLLSQDFKGMLDHIISRLPAERQILLYSATF

PLTVKQFMDKHLHSPYEINLMEELTLKGVTQYYAFVQEKQKVHCLNTLFSKLQINQSIIFCNSTQRVELLAKKITDLGYC

CYYIHAKMAQAHRNRVFHDFRKGSCRNLVCSDLFTRGIDVQAVNVVINFDFPKMAETYLHRIGRSGRFGHLGIAINLITY

DDRFALHRIEQELGTEIKPIPKVIDPRLYVARPEDVDINEEMDLSK

>XP_020807187.1 putative ATP-dependent RNA helicase me31b [Drosophila serrata]

MRTMMTEKLNSGHTILTSKGHINDLQIAGNTSDDMGWKSKLKLPPKDNRFKTTDVTDTRGNEFEEFCLKRELLMGIFEKG

WERPSPIQEAAIPIALSGKDVLARAKNGTGKTGAYCIPVLEQIDPKKDYIQALVMVPTRELALQTSQICIELAKHLDIRV

MVTTGGTILKDDILRIYQKVQLIIATPGRILDLMDKKVADMSHCRILVLDEADKLLSLDFQGMLDHVILKLPKDPQILLF

SATFPLTVKNFMEKHLREPYEINLMEELTLKGVTQYYAFVQERQKVHCLNTLFSKLQINQSIIFCNSTQRVELLAKKITE

LGYCCYYIHAKMAQAHRNRVFHDFRQGLCRNLVCSDLFTRGIDVQAVNVVINFDFPRMAETYLHRIGRSGRFGHLGIAIN

LITYEDRFDLHRIEKELGTEIKPIPKVIDPALYVANVGAATAGETGNNSDLNNSANEEGNVSK

>XP_002014399.1 putative ATP-dependent RNA helicase me31b [Drosophila persimilis]

MMTEKISSGHTNLSNKGIINDQKILANISDDMGWKSKLKLPPKDNRFKTTDVTDTRGNEFEEFCLKRELLMGIFEKGWER

PSPIQEAAIPIALSGKDVLARAKNGTGKTGAYCIPVLEQIDPTKDYIQALVMVPTRELALQTSQICIELAKHLDIRVMVT

TGGTILKDDILRIYQKVQLIIATPGRILDLMDKKVADMSHCRILVLDEADKLLSLDFQGMLDHVILKLPKDPQILLFSAT

FPLTVKNFMEKHLREPYEINLMEELTLKGVTQYYAFVQERQKVHCLNTLFSKLQINQSIIFCNSTQRVELLAKKITELGY

CCYYIHAKMAQAHRNRVFHDFRQGLCRNLVCSDLFTRGIDVQAVNVVINFDFPRMAETYLHRIGRSGRFGHLGIAINLIT

YEDRFDLHRIEKELGTEIKPIPKVIDPALYVANVSGSSGEICNNSDLNTSANEEGNVSK

>XP_015376866.1 PREDICTED: putative ATP-dependent RNA helicase me31b isoform X2 [Diuraphis noxia]

MLAANHNNSGINLNMPSKQLNRIFEGIDDAGWKAKLKLPPQDHRIKTSDVTSTKGNDFEEFCLKRELLMGIFEKGWEKPS

PIQEASIPIALSGKDILARAKNGTGKTGAYSIPVLEQVDPKLDVIQALVIVPTRELALQTSQICIELAKHLDIRVMVTTG

GTNLKDDILRIYQRVHVIIATPGRILDLLDKSIAKVDNCRILVLDEADKLLSQDFKGMLDHIISRLPAERQILLYSATFP

LTVKQFMDKHLHSPYEINLMEELTLKGVTQYYAFVQEKQKVHCLNTLFSKLQINQSIIFCNSTQRVELLAKKITDLGYCC

YYIHAKMAQAHRNRVFHDFRKGSCRNLVCSDLFTRGIDVQAVNVVINFDFPKMAETYLHRIGRSGRFGHLGIAINLITYD

DRFALHRIEQELGTEIKPIPKVIDPRLYVARPEDVDINEEMDLSK

>XP_017152719.1 ATP-dependent RNA helicase me31b [Drosophila miranda]

MMTEKISSGHTNLSNKGIINDQKILANISDDMGWKSKLKLPPKDNRFKTTDVTDTRGNEFEEFCLKRELLMGIFEKGWER

PSPIQEAAIPIALSGKDVLARAKNGTGKTGAYCIPVLEQIDPTKDYIQALVMVPTRELALQTSQICIELAKHLDIRVMVT

TGGTILKDDILRIYQKVQLIIATPGRILDLMDKKVADMSHCRILVLDEADKLLSLDFQGMLDHVILKLPKDPQILLFSAT

FPLTVKNFMEKHLREPYEINLMEELTLKGVTQYYAFVQERQKVHCLNTLFSKLQINQSIIFCNSTQRVELLAKKITELGY

CCYYIHAKMAQAHRNRVFHDFRQGLCRNLVCSDLFTRGIDVQAVNVVINFDFPRMAETYLHRIGRSGRFGHLGIAINLIT

YEDRFDLHRIEKELGTEIKPIPKVIDPALYVANVSGSSGEICNNSDLNTSANEECNVSK

>TMW48608.1 hypothetical protein DOY81_006311 [Sarcophaga bullata]

MMTEKVNSAHGNMVIKSINKEHQMVTKIGDDMGWKSKLKIPPKDNRFKTSDVTDTRGNEFEEFCLKRELLMGIFEKGWER

PSPIQEAAIPIALSGKDVLARAKNGTGKTGAYCIPVLEQIDPSKDYIQALIMVPTRELALQTSQICIELAKHLDIRVMVT

TGGTILKDDILRLYQKVQLIIATPGRILDLMDKKVANMSHCKILVLDEADKLLSLDFQGMLDHVIFKLPKDPQILLFSAT

FPLTVKNFMEKHLREPYEINLMEELTLKGVTQYYAFVQERQKVHCLNTLFSKLQINQSIIFCNSTQRVELLAKKITELGY

CCYYIHAKMAQAHRNRVFHDFRQGLCRNLVCSDLFTRGIDVQAVNVVINFDFPRMAETYLHRIGRSGRFGHLGIAINLIT

YEDRFDLHRIEKELGTEIKPIPKVIDPALYVANLALNSGECSNNDLNNSCTEEGNISK

>XP_019757103.1 PREDICTED: putative ATP-dependent RNA helicase me31b isoform X2 [Dendroctonus ponderosae]

MELKDDLGWKAKLKIPAKDHRIQTTDVTDTRGNEFEEYCLKRELLMGIFEKGWEKPSPIQEAAIPIALTGKDVLARAKNG

TGKTGAYSIPVLEQIDPTKDCIQALVIVPTRELALQTSQICIELAKHLLIRIMVTTGGTNLRDDIMRIYQKVQVIIATPG

RILDLMEKGVAQMDQCKILVLDEADKLLSQDFKGMLDVVIKNLPEKRQVLLFSATFPLTVEQFMRKHLRSPYEINLMEEL

TLKGVTQYYAFVQERQKVHCLNTLFSKLQINQSIIFCNSTQRVELLAKKITELGYCCYYIHAKMAQTHRNRVFHDFRSGL

CRNLVCSDLFTRGIDVQAVNVVINFDFPKMAETYLHRIGRSGRFGHLGIAINLITYEDRFALHRIEQELGTEIKPIPKII

DPRLYVAKLGEEDPEDLVK

>GBP63278.1 Putative ATP-dependent RNA helicase me31b [Eumeta japonica]

MMTESRISSSNHVGNSVSNQSKGDVDKTVDDIGWKSKLKIPPKDRRIKTSDVTDTRGNEFEEFCLKRELLMGIFEKGWEK

PSPIQEASIPIALSGKDILARAKNGTGKTGAYCIPVLEQVDPKKDAIQALIVVPTRELALQTSQICIELAKHTDIRVMVT

TGGTNLRDDIMRIYQNVQVIIATPGRMIDLMDKQVAKMDQCRMLVLDEADKLLSQDFKGMLDMVIARLPKERQILLFSAT

FPLSVKQFMEKHLREPYEINLMEELTLKGVTQYYAFVQERQKVHCLNTLFSKRVELLAKKITELGYCCYYIHARMAQAHR

NRVFHDFRAGLCRNLVCSDLFTRGIDVQAVNVVINFDFPRMAETYLHRIGRSGRFGHLGIAINLITYEDRFTLHRIEQEL

GTEIKPIPKVIDPALYVARSEDEEQAQARGDK

>XP_017075685.1 PREDICTED: putative ATP-dependent RNA helicase me31b [Drosophila eugracilis]

MMTEKLNSGHTNLTSKGLINDLQIAGNTSDDMGWKSKLKLPPKDNRFKTTDVTDTRGNEFEEFCLKRELLMGIFEKGWER

PSPIQEAAIPIALSGKDVLARAKNGTGKTGAYCIPVLEQIDPTKDYIQALVMVPTRELALQTSQICIELAKHLDIRVMVT

TGGTILKDDILRIYQKVQLIIATPGRILDLMDKKVADMSHCRILVLDEADKLLSLDFQGMLDHVILKLPKDPQILLFSAT

FPLTVKNFMEKHLREPYEINLMEELTLKGVTQYYAFVQERQKVHCLNTLFSKLQINQSIIFCNSTQRVELLAKKITELGY

CCYYIHAKMAQAHRNRVFHDFRQGLCRNLVCSDLFTRGIDVQAVNVVINFDFPRMAETYLHRIGRSGRFGHLGIAINLIT

YEDRFDLHRIEKELGTEIKPIPKVIDPALYVANVGASVGDTINNSDLNNSANEEGNVSK

>XP_023298132.1 putative ATP-dependent RNA helicase me31b [Lucilia cuprina]

MMTEKINSAHGNMIIKSINKEHQHQLVTNIGDDMGWKSKLKIPPKDNRFKTSDVTDTRGNEFEEFCLKRELLMGIFEKGW

ERPSPIQEAAIPIALSGKDVLARAKNGTGKTGAYCIPVLEQIDPSKDYIQALIMVPTRELALQTSQICIELAKHLDIRVM

VTTGGTILKDDILRIYQKVQLIIATPGRILDLMDKKVADMSHCKILVLDEADKLLSLDFQGMLDHVILKLPKDPQILLFS

ATFPLTVKNFMEKHLREPYEINLMEELTLKGVTQYYAFVQERQKVHCLNTLFSKLQINQSIIFCNSTQRVELLAKKITEL

GYCCYYIHAKMAQAHRNRVFHDFRQGLCRNLVCSDLFTRGIDVQAVNVVINFDFPRMAETYLHRIGRSGRFGHLGIAINL

ITYEDRFDLHRIEKELGTEIKPIPKVIDPALYVANIALNSGESSNNDLNNSCTEEGNVSK

>XP_017025708.1 PREDICTED: putative ATP-dependent RNA helicase me31b [Drosophila kikkawai]

MRTMMTEKLNSGHTNLTSKGLINDLQIAGNTSDDMGWKSKLKLPPKDNRFKTTDVTDTRGNEFEEFCLKRELLMGIFEKG

WERPSPIQEAAIPIALSGKDVLARAKNGTGKTGAYCIPVLEQIDPTKDYIQALVMVPTRELALQTSQICIELAKHLDIRV

MVTTGGTILKDDILRIYQKVQLIIATPGRILDLMDKKVADMSHCRILVLDEADKLLSLDFQGMLDHVILKLPKDPQILLF

SATFPLTVKNFMEKHLREPYEINLMEELTLKGVTQYYAFVQERQKVHCLNTLFSKLQINQSIIFCNSTQRVELLAKKITE

LGYCCYYIHAKMAQAHRNRVFHDFRQGLCRNLVCSDLFTRGIDVQAVNVVINFDFPRMAETYLHRIGRSGRFGHLGIAIN

LITYEDRFDLHRIEKELGTEIKPIPKVIDPALYVANVGAATSGETCNNSDLNNSANEEGNVSK

>XP_002036453.1 ATP-dependent RNA helicase me31b [Drosophila sechellia]

MMTEKLNSGHTNLTSKGIINDLQIAGNTSDDMGWKSKLKLPPKDNRFKTTDVTDTRGNEFEEFCLKRELLMGIFEKGWER

PSPIQEAAIPIALSGKDVLARAKNGTGKTGAYCIPVLEQIDPTKDKIQALVMVPTRELALQTSQICIELAKHLDIRVMVT

TGGTILKDDILRIYQKVQLIIATPGRILDLMDKKVADMSHCRILVLDEADKLLSLDFQGMLDHVILKLPKDPQILLFSAT

FPLTVKNFMEKHLREPYEINLMEELTLKGVTQYYAFVQERQKVHCLNTLFSKLQINQSIIFCNSTQRVELLAKKITELGY

CCYYIHAKMAQAHRNRVFHDFRQGLCRNLVCSDLFTRGIDVQAVNVVINFDFPRMAETYLHRIGRSGRFGHLGIAINLIT

YEDRFDLHRIEKELGTEIKPIPKVIDPALYVANVGASVGDTCNNSDLNNSANEEGNVSK

>XP_001969421.1 putative ATP-dependent RNA helicase me31b isoform X1 [Drosophila erecta]

MMTEKLNSGHTNLTSKGLINDLQIAGNTSDDMGWKSKLKLPPKDNRFKTTDVTDTRGNEFEEFCLKRELLMGIFEKGWER

PSPIQEAAIPIALSGKDVLARAKNGTGKTGAYCIPVLEQIDPTKDYIQALVMVPTRELALQTSQICIELAKHLDIRVMVT

TGGTILKDDILRIYQKVQLIIATPGRILDLMDKKVADMSHCRILVLDEADKLLSLDFQGMLDHVILKLPKDPQILLFSAT

FPLTVKNFMEKHLREPYEINLMEELTLKGVTQYYAFVQERQKVHCLNTLFSKLQINQSIIFCNSTQRVELLAKKITELGY

CCYYIHAKMAQAHRNRVFHDFRQGLCRNLVCSDLFTRGIDVQAVNVVINFDFPRMAETYLHRIGRSGRFGHLGIAINLIT

YEDRFDLHRIEKELGTEIKPIPKVIDPALYVANVGASVGDTCNNSDLNNSANEEGNVSK

>XP_017005722.1 PREDICTED: putative ATP-dependent RNA helicase me31b [Drosophila takahashii]

MMTEKLNSGHTNLTSKGLINDLQIAGNTSDDMGWKSKLKLPPKDNRFKTTDVTDTRGNEFEEFCLKRELLMGIFEKGWER

PSPIQEAAIPIALSGKDVLARAKNGTGKTGAYCIPVLEQIDPTKDYIQALVMVPTRELALQTSQICIELAKHLDIRVMVT

TGGTILKDDILRIYQKVQLIIATPGRILDLMDKKVADMSHCRILVLDEADKLLSLDFQGMLDHVILKLPKDPQILLFSAT

FPLTVKNFMEKHLREPYEINLMEELTLKGVTQYYAFVQERQKVHCLNTLFSKLQINQSIIFCNSTQRVELLAKKITELGY

CCYYIHAKMAQAHRNRVFHDFRQGLCRNLVCSDLFTRGIDVQAVNVVINFDFPRMAETYLHRIGRSGRFGHLGIAINLIT

YEDRFDLHRIEKELGTEIKPIPKVIDPALYVANIGASVGDTCNNSDLNNSANEEGNVSK

>VVC38605.1 Hypothetical protein CINCED_3A019670 [Cinara cedri]

MIATNHSSTGLSLGMPIKQMNRISFEGIDDAGWKAKLKLPPQDKRIKTSDVTSTKGNDFEEFCLKRELLMGIFEKGWEKP

SPIQEASIPIALSGKDILARAKNGTGKTGAYSIPVLEQVDPKLDVIQALVIVPTRELALQTSQICIELAKHLDIRVMVTT

GGTNLKDDILRIYQRVHVIIATPGRILDLLDKSIAKVDHCRILVLDEADKLLSQDFKGMLDHIISKLPSERQILLYSATF

PLTVKQFMDKHLRSPYEINLMEELTLKGVTQYYAFVQEKQKVHCLNTLFSKLQINQSIIFCNSTQRVELLAKKITDLGYC

CYYIHAKMAQAHRNRVFHDFRKGSCRNLVCSDLFTRGIDVQAVNVVINFDFPKMAETYLHRIGRSGRFGHLGIAINLITY

DDRFALHRIEQELGTEIKPIPKVSNFSNIMYL

>XP_034129071.1 ATP-dependent RNA helicase me31b [Drosophila guanche]

MMTEKISSGHTNLSNKGIINDHKILGNISDDMGWKSKLKLPPKDNRFKTTDVTDTRGNEFEEFCLKRELLMGIFEKGWER

PSPIQEAAIPIALSGKDVLARAKNGTGKTGAYCIPVLEQIDPTKDYIQALVMVPTRELALQTSQICIELAKHLDIRVMVT

TGGTILKDDILRIYQKVQLIIATPGRILDLMDKKVADMSHCRILVLDEADKLLSLDFQGMLDHVILKLPKDPQILLFSAT

FPLTVKNFMEKHLREPYEINLMEELTLKGVTQYYAFVQERQKVHCLNTLFSKLQINQSIIFCNSTQRVELLAKKITELGY

CCYYIHAKMAQAHRNRVFHDFRQGLCRNLVCSDLFTRGIDVQAVNVVINFDFPRMAETYLHRIGRSGRFGHLGIAINLIT

YEDRFDLHRIEKELGTEIKPIPKVIDPALYVANVGGSSGEICNNSDLNTSANEEGNVSK

>XP_017042909.1 PREDICTED: putative ATP-dependent RNA helicase me31b [Drosophila ficusphila]

MMTEKLNSGHTNLTSKGLINDLQIAGNTSDDMGWKSKLKLPPKDNRFKTTDVTDTRGNEFEEFCLKRELLMGIFEKGWER

PSPIQEAAIPIALSGKDVLARAKNGTGKTGAYCIPVLEQIDPTKDHIQALVMVPTRELALQTSQICIELAKHLDIRVMVT

TGGTILKDDILRIYQKVQLIIATPGRILDLMDKKVADMSHCRILVLDEADKLLSLDFQGMLDHVILKLPKDPQILLFSAT

FPLTVKNFMEKHLREPYEINLMEELTLKGVTQYYAFVQERQKVHCLNTLFSKLQINQSIIFCNSTQRVELLAKKITELGY

CCYYIHAKMAQAHRNRVFHDFRQGLCRNLVCSDLFTRGIDVQAVNVVINFDFPRMAETYLHRIGRSGRFGHLGIAINLIT

YEDRFDLHRIEKELGTEIKPIPKVIDPALYVANVGASVGDTCNNSDLNNSANEEGNVSK

>NP_523533.2 maternal expression at 31B, isoform A [Drosophila melanogaster]

MMTEKLNSGHTNLTSKGIINDLQIAGNTSDDMGWKSKLKLPPKDNRFKTTDVTDTRGNEFEEFCLKRELLMGIFEKGWER

PSPIQEAAIPIALSGKDVLARAKNGTGKTGAYCIPVLEQIDPTKDYIQALVMVPTRELALQTSQICIELAKHLDIRVMVT

TGGTILKDDILRIYQKVQLIIATPGRILDLMDKKVADMSHCRILVLDEADKLLSLDFQGMLDHVILKLPKDPQILLFSAT

FPLTVKNFMEKHLREPYEINLMEELTLKGVTQYYAFVQERQKVHCLNTLFSKLQINQSIIFCNSTQRVELLAKKITELGY

CCYYIHAKMAQAHRNRVFHDFRQGLCRNLVCSDLFTRGIDVQAVNVVINFDFPRMAETYLHRIGRSGRFGHLGIAINLIT

YEDRFDLHRIEKELGTEIKPIPKVIDPALYVANVGASVGDTCNNSDLNNSANEEGNVSK

>XP_016982514.1 PREDICTED: putative ATP-dependent RNA helicase me31b [Drosophila rhopaloa]

MMTEKLNSGHTNLTSKGLINDLQIAGNTSDDMGWKSKLKLPPKDNRFKTTDVTDTRGNEFEEFCLKRELLMGIFEKGWER

PSPIQEAAIPIALSGKDVLARAKNGTGKTGAYCIPVLEQIDPTKDYIQALVMVPTRELALQTSQICIELAKHLDIRVMVT

TGGTILKDDILRIYQKVQLIIATPGRILDLMDKKVADMSHCRILVLDEADKLLSLDFQGMLDHVILKLPKDPQILLFSAT

FPLTVKNFMEKHLREPYEINLMEELTLKGVTQYYAFVQERQKVHCLNTLFSKLQINQSIIFCNSTQRVELLAKKITELGY

CCYYIHAKMAQAHRNRVFHDFRQGLCRNLVCSDLFTRGIDVQAVNVVINFDFPRMAETYLHRIGRSGRFGHLGIAINLIT

YEDRFDLHRIEKELGTEIKPIPKVIDPALYVANIGASVGDHNSDLNNSANEEGNVSK

>XP_022228396.1 putative ATP-dependent RNA helicase me31b [Drosophila obscura]

MMTEKLSSGHTNLSNKGIINDHKVLGNISDDMGWKSKLKLPPKDNRFKTTDVTDTRGNEFEEFCLKRELLMGIFEKGWER

PSPIQEAAIPIALSGKDVLARAKNGTGKTGAYCIPVLEQIDPTKDYIQALVMVPTRELALQTSQICIELAKHLDIRVMVT

TGGTILKDDILRIYQKVQLIIATPGRILDLMDKKVADMSHCRILVLDEADKLLSLDFQGMLDHVILKLPKDPQILLFSAT

FPLTVKNFMEKHLREPYEINLMEELTLKGVTQYYAFVQERQKVHCLNTLFSKLQINQSIIFCNSTQRVELLAKKITELGY

CCYYIHAKMAQAHRNRVFHDFRQGLCRNLVCSDLFTRGIDVQAVNVVINFDFPRMAETYLHRIGRSGRFGHLGIAINLIT

YEDRFDLHRIEKELGTEIKPIPKVIDPALYVANVGGSSGEICNNSDLNTSANEEGNVSK

>XP_011195447.1 ATP-dependent RNA helicase me31b [Zeugodacus cucurbitae]

MITENTTNMHASLGSKSAMKALQMVKNPQDDMGWKSKLKIPPKDNRFKTSDVTDTRGNEFEEFCLKRELLMGIFEKGWER

PSPIQEAAIPIALSGKDVLARAKNGTGKTGAYCIPVLEQIDPRKDYIQALVIVPTRELALQTSQICIELAKHLDIRVMVT

TGGTILKDDILRIYQKVQLIIATPGRILDLMDKKVADMSHCKILVLDEADKLLSLDFQGMLDHVIMKLPKDPQILLFSAT

FPLSVKNFMEKHLREPYEINLMEELTLKGVTQYYAFVQERQKVHCLNTLFSKLQINQSIIFCNSTQRVELLAKKITELGY

CCYYIHAKMAQAHRNRVFHDFRQGLCRNLVCSDLFTRGIDVQAVNVVINFDFPRMAETYLHRIGRSGRFGHLGIAINLIT

YEDRFDLHRIEKELGTEIKPIPKVIDPALYVANAAATSTETNNDLNNSVNEEGNISK

>XP_011197588.1 ATP-dependent RNA helicase me31b [Bactrocera dorsalis]

MITENTTNMHASLGSKSAMKALQMVKNPQDDMGWKSKLKIPPKDNRFKTSDVTDTRGNEFEEFCLKRELLMGIFEKGWER

PSPIQEAAIPIALSGKDVLARAKNGTGKTGAYCIPVLEQIDPRKDYIQALVIVPTRELALQTSQICIELAKHLDIRVMVT

TGGTILKDDILRIYQKVQLIIATPGRILDLMDKKVADMSHCKILVLDEADKLLSLDFQGMLDHVIMKLPKDPQILLFSAT

FPLSVKNFMEKHLREPYEINLMEELTLKGVTQYYAFVQERQKVHCLNTLFSKLQINQSIIFCNSTQRVELLAKKITELGY

CCYYIHAKMAQAHRNRVFHDFRQGLCRNLVCSDLFTRGIDVQAVNVVINFDFPRMAETYLHRIGRSGRFGHLGIAINLIT

YEDRFDLHRIEKELGTEIKPIPKVIDPALYVANAAATSTETNNDLNNSANEEGNISK

>XP_018791325.1 PREDICTED: putative ATP-dependent RNA helicase me31b [Bactrocera latifrons]

MITENTTNMHANLGSKSAMKALQMVKNPQDDMGWKSKLKIPPKDNRFKTSDVTDTRGNEFEEFCLKRELLMGIFEKGWER

PSPIQEAAIPIALSGKDVLARAKNGTGKTGAYCIPVLEQIDPRKDYIQALVIVPTRELALQTSQICIELAKHLDIRVMVT

TGGTILKDDILRIYQKVQLIIATPGRILDLMDKKVADMSHCKILVLDEADKLLSLDFQGMLDHVIMKLPKDPQILLFSAT

FPLSVKNFMEKHLREPYEINLMEELTLKGVTQYYAFVQERQKVHCLNTLFSKLQINQSIIFCNSTQRVELLAKKITELGY

CCYYIHAKMAQAHRNRVFHDFRQGLCRNLVCSDLFTRGIDVQAVNVVINFDFPRMAETYLHRIGRSGRFGHLGIAINLIT

YEDRFDLHRIEKELGTEIKPIPKVIDPALYVANAAATSTETNNDLNNSANEEGNISK

>XP_004524166.1 putative ATP-dependent RNA helicase me31b [Ceratitis capitata]

MITENTTNMHANLGSKSAMKALQMVKNPQDDMGWKSKLKIPPKDNRFKTSDVTDTRGNEFEEFCLKRELLMGIFEKGWER

PSPIQEAAIPIALSGKDVLARAKNGTGKTGAYCIPVLEQIDPTKDYIQALVIVPTRELALQTSQICIELAKHLDIRVMVT

TGGTILKDDILRIYQKVQLIIATPGRILDLMDKKVADMSHCKILVLDEADKLLSLDFQGMLDHVIMKLPKDPQILLFSAT

FPLSVKNFMEKHLREPYEINLMEELTLKGVTQYYAFVQERQKVHCLNTLFSKLQINQSIIFCNSTQRVELLAKKITELGY

CCYYIHAKMAQAHRNRVFHDFRQGLCRNLVCSDLFTRGIDVQAVNVVINFDFPRMAETYLHRIGRSGRFGHLGIAINLIT

YEDRFDLHRIEKELGTEIKPIPKVIDPALYVANAAATPTETNNDLNNSANEEGNISK

>XP_036217390.1 ATP-dependent RNA helicase me31b isoform X2 [Bactrocera oleae]

MHASLGSKSAMKALQMVKNPQDDMGWKSKLKIPPKDNRFKTSDVTDTRGNEFEEFCLKRELLMGIFEKGWERPSPIQEAA

IPIALSGKDVLARAKNGTGKTGAYCIPVLEQIDPRKDYIQALVIVPTRELALQTSQICIELAKHLDIRVMVTTGGTILKD

DILRIYQKVQLIIATPGRILDLMDKKVADMSHCKILVLDEADKLLSLDFQGMLDHVIMKLPKDPQILLFSATFPLSVKNF

MEKHLREPYEINLMEELTLKGVTQYYAFVQERQKVHCLNTLFSKLQINQSIIFCNSTQRVELLAKKITELGYCCYYIHAK

MAQAHRNRVFHDFRQGLCRNLVCSDLFTRGIDVQAVNVVINFDFPRMAETYLHRIGRSGRFGHLGIAINLITYEDRFDLH

RIEKELGTEIKPIPKVIDPALYVANAAATSTETNNDLNNSANEEGNISK

>KOB69570.1 putative DEAD box ATP-dependent RNA helicase [Operophtera brumata]

MMTENRISSSNHVGNSLTSQKGDVDKTIDDIGWKSKLKIPPKDRRIKTSDVTDTRGNEFEEFCLKRELLMGIFEKGWEKP

SPIQEAAIPIALSGKDVLARAKNGTGKTGAYCIPVLEQVDPKKDYIQALVVVPTRELALQTSQICIELAKHTDIRVMVTT

GVQVIIATPGRMIDLMDKQVAKMDQCRMLVLDEADKLLSQDFKGMLDMVISRLPKERQILLFSATFPLSVKQFMEKHLKE

PYEINLMEELTLKGVTQYYAFVQERQKVHCLNTLFSKLQINQSIIFCNSTQRVELLAKKITELGYCCYYIHARMAQAHRN

RVFHDFRAGLCRNLVCSDLFTRGIDVQAVNVVINFEFPRMAETYLHRIGRSGRFGHLGIAINLITYEDRFALHRIEQELG

TEIKPIPKVIDPALYVARPDDGGDDLGDK

>XP_023167930.1 ATP-dependent RNA helicase me31b [Drosophila hydei]

MMTEKIVTGHPSLLNKGMLSDQQIASNLGDDMGWKSKLKLPPKDNRFKTTDVTDTRGNEFEEFCLKRELLMGIFEKGWER

PSPIQEAAIPIALTGKDVLARAKNGTGKTGAYCIPVLEQIDPTKDYIQALVMVPTRELALQTSQICIELAKHLDIRVMVT

TGGTILKDDILRIYQKVQLIIATPGRILDLMDKKVADMSHCKILVLDEADKLLSLDFQGMLDHVILKLPKDPQILLFSAT

FPLTVKNFMEKHLREPYEINLMEELTLKGVTQYYAFVQERQKVHCLNTLFSKLQINQSIIFCNSTQRVELLAKKITELGY

CCYYIHAKMAQAHRNRVFHDFRQGLCRNLVCSDLFTRGIDVQAVNVVINFDFPRMAETYLHRIGRSGRFGHLGIAINLIT

YEDRFDLHRIEKELGTEIKPIPKVIDPALYVANVSGSSGETCNNSDLNNSTNEEGNVSK

>KOC62357.1 Putative ATP-dependent RNA helicase me31b [Habropoda laboriosa]

MDDVGWKAKLKIPPKDKRIKTSDVTDTRGNEFEEFCLKRELLMGIFEKGWEKPSPIQEASIPIALSGKDILARAKNGTGK

TGAYSIPVLEQVDPRQDVIQALVIVPTRELALQTSQICIELAKHMDIKVMVTTGGTDLRDDIMRIYQKVHVIIATPGRIL

DLMDKSVANMDHCKILVLDEADKLLSQDFKGMLDHVISRLPHERQILLYSATFPLTVKQFMEKHLRDPYEINLMEELTLK

GVTQYYAFEFLFFKLQITQSIIFCNSTQRVELLAKKITDLGYCCYYIHAKMAQAHRNRVFHDFRAGLCRNLVSSDLFTRG

IDVQAVNVVINFDFPKMAETYLHRIGRSGRFGHLGIAINLITYEDRFNLHRIEQELGTEIKPIPKVIDPSLYVARPEDNN

SMEEANVSK

>XP_002075549.3 putative ATP-dependent RNA helicase me31b [Drosophila willistoni]

MSQRTMMTEKVTSGHTNLLNKGVINDHQIISNDDMGWKSKLKLPPKDNRFKTTDVTDTRGNEFEEFCLKRELLMGIFEKG

WERPSPIQEAAIPIALSGKDVLARAKNGTGKTGAYCIPVLEQIDPTKDYIQALVMVPTRELALQTSQICIELAKHLDIRV

MVTTGGTILKDDILRIYQKVQLIIATPGRILDLMDKKVADMSHCKILVLDEADKLLSLDFQGMLDHVILKLPKDPQILLF

SATFPLTVKNFMEKHLREPYEINLMEELTLKGVTQYYAFVQERQKVHCLNTLFSKLQINQSIIFCNSTQRVELLAKKITE

LGYCCYYIHAKMAQAHRNRVFHDFRQGLCRNLVCSDLFTRGIDVQAVNVVINFDFPRMAETYLHRIGRSGRFGHLGIAIN

LITYEDRFDLHRIEKELGTEIKPIPKVIDPALYVANSGVSSGDTCNNSDLNNSTTEEGNVSK

>XP_017465604.1 PREDICTED: putative ATP-dependent RNA helicase me31b [Rhagoletis zephyria]

MITENTTNMHASLGSKSAIKALQMVKNPQDDMGWKSKLKIPPKDNRFKTSDVTDTRGNEFEEFCLKRELLMGIFEKGWER

PSPIQEAAIPIALSGKDVLARAKNGTGKTGAYCIPVLEQIDHTKDYIQALVIVPTRELALQTSQICIELAKHLDIRVMVT

TGGTILKDDILRIYQKVQLIIATPGRILDLMDKKVADMSHCKILVLDEADKLLSLDFQGMLDHVIMKLPKDPQILLFSAT

FPLSVKNFMEKHLREPYEINLMEELTLKGVTQYYAFVQERQKVHCLNTLFSKLQINQSIIFCNSTQRVELLAKKITELGY

CCYYIHAKMAQAHRNRVFHDFRQGLCRNLVCSDLFTRGIDVQAVNVVINFDFPRMAETYLHRIGRSGRFGHLGIAINLIT

YEDRFDLHRIEKELGTEIKPIPKVIDPALYVANAAATSTETNNDLNNSANEEGNISK

>XP_034473003.1 ATP-dependent RNA helicase me31b [Drosophila innubila]

MMTEKIVPGHSSLVNKGMLSDHQIASNLSDDMGWKSKLKLPPKDNRFKTTDVTDTRGNEFEEFCLKRELLMGIFEKGWER

PSPIQEAAIPIALSGKDVLARAKNGTGKTGAYCIPVLEQIDPTKDYIQALVMVPTRELALQTSQICIELAKHLDIRVMVT

TGGTILKDDILRIYQKVQLIIATPGRILDLMDKKVADMSHCKILVLDEADKLLSLDFQGMLDHVILKLPKDPQILLFSAT

FPLTVKNFMEKHLREPYEINLMEELTLKGVTQYYAFVQERQKVHCLNTLFSKLQINQSIIFCNSTQRVELLAKKITELGY

CCYYIHAKMAQAHRNRVFHDFRQGLCRNLVCSDLFTRGIDVQAVNVVINFDFPRMAETYLHRIGRSGRFGHLGIAINLIT

YEDRFDLHRIEKELGTEIKPIPKVIDPALYVANVGGVSSAETCNNSDLNNSTNEEGNVSK

>XP_030382942.1 ATP-dependent RNA helicase me31b [Scaptodrosophila lebanonensis]

MMTEKVSSGHANMVTKGIIHDHKIVSNHNDDMGWKSKLKLPPKDNRFKTTDVTDTRGNEFEEFCLKRELLMGIFEKGWER

PSPIQEAAIPIALSGKDVLARAKNGTGKTGAYCIPVLEQIDPTKDFIQALVMVPTRELALQTSQICIELAKHLDIRVMVT

TGGTILKDDILRIYQKVQLIIATPGRILDLMDKKVADMSHCKILVLDEADKLLSLDFQGMLDHVILKLPKDPQILLFSAT

FPLTVKNFMEKHLREPYEINLMEELTLKGVTQYYAFVQERQKVHCLNTLFSKLQINQSIIFCNSTQRVELLAKKITELGY

CCYYIHAKMAQAHRNRVFHDFRQGLCRNLVCSDLFTRGIDVQAVNVVINFDFPRMAETYLHRIGRSGRFGHLGIAINLIT

YEDRFDLHRIEKELGTEIKPIPKIIDPALYVANISGCSGEACSNSDLNTSTNEEGNVSK

>KAF6207557.1 hypothetical protein [Apolygus lucorum]

MADLLNSHNSGMVNRLPNNKKPEGEDGWKSKLKVPPTDKRQKTSDVTDTKGNEFEEFCLSRELLMGIFEKGWEKPSPIQE

ASIPVALTGRNILARAKNGTGKTGAYSIPVLEQIDVNLDQIQAMIIVPTRELALQTSQICIELAKHLQIKVMVTTGGTNL

RDDIMRLFQKVQVVIATPGRILDLMDKKVAVVDSCKILVLDEADKLLSQDFKGMLDLVISKLPQDRQILLYSATFPLTVK

QFMDKHLKDPYEINLMEELTLKGVTQYYAFVQERQKVHCLNTLFSKLQITQSIIFCNSTQRVELLAKKITELGYCCYYIH

AKMAQAHRNRVFHDFRSGLCRNLVCSDLFTRGIDVQAVNVVINFDFPKMAETYLHRIGRSGRFGHLGIAINLITYEDRFA

LHRIEQELGTEIKPIPKVIDPELYVAKLDDGIEEANTNNK

>XP_002052405.1 ATP-dependent RNA helicase me31b [Drosophila virilis]

MMTEKIVQGHPSLVNKGILSDHQIATNLSDDMGWKSKLKLPPKDNRFKTTDVTDTRGNEFEEFCLKRELLMGIFEKGWER

PSPIQEAAIPIALSGKDVLARAKNGTGKTGAYCIPVLEQIDPTKDYIQALVMVPTRELALQTSQICIELAKHLDIRVMVT

TGGTILKDDILRIYQKVQLIIATPGRILDLMDKKVADMSHCKILVLDEADKLLSLDFQGMLDHVILKLPKDPQILLFSAT

FPLTVKNFMEKHLREPYEINLMEELTLKGVTQYYAFVQERQKVHCLNTLFSKLQINQSIIFCNSTQRVELLAKKITELGY

CCYYIHAKMAQAHRNRVFHDFRQGLCRNLVCSDLFTRGIDVQAVNVVINFDFPRMAETYLHRIGRSGRFGHLGIAINLIT

YEDRFDLHRIEKELGTEIKPIPKVIDPALYVANVSGSSGETCNNSDLNNSTNEEGNVSK

>XP_013116698.1 PREDICTED: putative ATP-dependent RNA helicase me31b [Stomoxys calcitrans]

MMTEKLNSPLADIINKSLNKVANIGDDMGWKSKLKIPPKDNRFKTSDVTDTRGNEFEEFCLKRKLLMGIFEKGWERPSPI

QEAAIPIALSGKDILARAKNGTGKTGAYCIPVLEQIDPSKDYIQALIMVPTRELALQTSQICIELAKHLDIRVMVTTGGT

ILKDDILRLYQKVQLIIATPGRILDLMDKRVADMSHCKILVLDEADKLLSLDFQGMLDHVILKLPKDPQILLFSATFPLT

VKNFMEKHLREPYEINLMEELTLKGVTQYYAFVQERQKVHCLNTLFSKLQINQSIIFCNSTQRVELLAKKITELGYCCYY

IHAKMAQAHRNRVFHDFRQGLCRNLVCSDLFTRGIDVQAVNVVINFDFPRMAETYLHRIGRSGRFGHLGIAINLITYEDR

FDLHRIEKELGTEIKPIPKVIDPALYVANVAVNSGETSNNDLNNSCIEEGNVSK

>XP_036324427.1 ATP-dependent RNA helicase me31b [Rhagoletis pomonella]

MITENTTNMHASLGSKSAIKALQLVKNPQDDMGWKSKLKIPPKDNRFKTSDVTDTRGNEFEEFCLKRELLMGIFEKGWER

PSPIQEAAIPIALSGKDVLARAKNGTGKTGAYCIPVLEQIDHTKDYIQALVIVPTRELALQTSQICIELAKHLDIRVMVT

TGGTILKDDILRIYQKVQLIIATPGRILDLMDKKVADMSHCKILVLDEADKLLSLDFQGMLDHVIMKLPKDPQILLFSAT

FPLSVKNFMEKHLREPYEINLMEELTLKGVTQYYAFVQERQKVHCLNTLFSKLQINQSIIFCNSTQRVELLAKKITELGY

CCYYIHAKMAQAHRNRVFHDFRQGLCRNLVCSDLFTRGIDVQAVNVVINFDFPRMAETYLHRIGRSGRFGHLGIAINLIT

YEDRFDLHRIEKELGTEIKPIPKVIDPALYVANAAATSTETNNDLNNSANEEGNISK

>XP_034099718.1 ATP-dependent RNA helicase me31b [Drosophila albomicans]

MMTEKIVPGHSSLINKGILSDHQIASNLSDDMGWKSKLKLPPKDNRFKTTDVTDTRGNEFEEFCLKRELLMGIFEKGWER

PSPIQEAAIPIALSGKDVLARAKNGTGKTGAYCIPVLEQIDPTKDYIQALVMVPTRELALQTSQICIELAKHLDIRVMVT

TGGTILKDDILRIYQKVQLIIATPGRILDLMDKKVADMSHCNILVLDEADKLLSLDFQGMLDHVILKLPKDPQILLFSAT

FPLTVKNFMEKHLREPYEINLMEELTLKGVTQYYAFVQERQKVHCLNTLFSKLQINQSIIFCNSTQRVELLAKKITELGY

CCYYIHAKMAQAHRNRVFHDFRQGLCRNLVCSDLFTRGIDVQAVNVVINFDFPRMAETYLHRIGRSGRFGHLGIAINLIT

YEDRFDLHRIEKELGTEIKPIPKVIDPALYVANVGGSSGEVCNNSDLNNSTNEEGNVSK

>XP_002004122.1 ATP-dependent RNA helicase me31b [Drosophila mojavensis]

MMTEKIVPGHPSLINKGMLSDHQIASNLGDDMGWKSKLKLPPKDNRFKTTDVTDTRGNEFEEFCLKRELLMGIFEKGWER

PSPIQEAAIPIALSGKDVLARAKNGTGKTGAYCIPVLEQIDPTKDYIQALVMVPTRELALQTSQICIELAKHLDIRVMVT

TGGTILKDDILRIYQKVQLIIATPGRILDLMDKKVADMSHCKILVLDEADKLLSLDFQGMLDHVILKLPKDPQILLFSAT

FPLTVKNFMEKHLREPYEINLMEELTLKGVTQYYAFVQERQKVHCLNTLFSKLQINQSIIFCNSTQRVELLAKKITELGY

CCYYIHAKMAQAHRNRVFHDFRQGLCRNLVCSDLFTRGIDVQAVNVVINFDFPRMAETYLHRIGRSGRFGHLGIAINLIT

YEDRFDLHRIEKELGTEIKPIPKVIDPALYVANISGSSGETCNNSDLNNSTNEEGNVSK

>XP_005191779.2 PREDICTED: putative ATP-dependent RNA helicase me31b [Musca domestica]

MMTEKINSPLADIINKSNLNKVTNIGDDMGWKSKLKIPPKDNRFKTSDVTDTRGNEFEEFCLKRKLLMGIFEKDWERPSP

IQEAAIPIALSGKDILARAKNGTGKTGAYCIPVLEQIDPSKDYIQALIMVPTRELALQTSQICIELAKHLDIRVMVTTGG

TILKDDILRLYQKVQLIIATPGRILDLMDKRVADMSHCKILVLDEADKLLSLDFQGMLDHVILKLPKDPQILLFSATFPL

TVKNFMEKHLREPYEINLMEELTLKGVTQYYAFVQERQKVHCLNTLFSKLQINQSIIFCNSTQRVELLAKKITELGYCCY

YIHAKMAQAHRNRVFHDFRQGLCRNLVCSDLFTRGIDVQAVNVVINFDFPRMAETYLHRIGRSGRFGHLGIAINLITYED

RFDLHRIEKELGTEIKPIPKVIDPALYVANVAVNSGESSNNDLNNSCIEEGNVSK

>XP_017095390.1 PREDICTED: putative ATP-dependent RNA helicase me31b [Drosophila bipectinata]

MMTEKLSSGRTNLTSKGIINDLQISGNTSDDDMGWKSKLKLPPKDNRFKTTDVTDTRGNEFEEFCLKRELLMGIFEKGWE

RPSPIQEAAIPIALSGKDVLARAKNGTGKTGAYCIPVLEQIDPTKDYIQALVMVPTRELALQTSQICIELAKHLDIRVMV

TTGGTILKDDILRIYQKVQLIIATPGRILDLMDKKVADMSHCRILVLDEADKLLSLDFQGMLDHVILKLPKDPQILLFSA

TFPLTVKNFMEKHIREPYEINLMEELTLKGVTQYYAFVQERQKVHCLNTLFSKLQINQSIIFCNSTQRVELLAKKITELG

YCCYYIHAKMAQAHRNRVFHDFRQGLCRNLVCSDLFTRGIDVQAVNVVINFDFPRMAETYLHRIGRSGRFGHLGIAINLI

TYEDRFDLHRIEKELGTEIKPIPKVIDPALYVANVGASVGDTCNNSDLNNSANEEGNVSK

>XP_001963062.1 ATP-dependent RNA helicase me31b [Drosophila ananassae]

MMTEKLSSGRTNLTSKGIINDLQISGNTSDDDMGWKSKLKLPPKDNRFKTTDVTDTRGNEFEEFCLKRELLMGIFEKGWE

RPSPIQEAAIPIALSGKDVLARAKNGTGKTGAYCIPVLEQIDPTKDYIQALVMVPTRELALQTSQICIELAKHLDIRVMV

TTGGTILKDDILRIYQKVQLIIATPGRILDLMDKKVADMSHCRILVLDEADKLLSLDFQGMLDHVILKLPKDPQILLFSA

TFPLTVKNFMEKHIREPYEINLMEELTLKGVTQYYAFVQERQKVHCLNTLFSKLQINQSIIFCNSTQRVELLAKKITELG

YCCYYIHAKMAQAHRNRVFHDFRQGLCRNLVCSDLFTRGIDVQAVNVVINFDFPRMAETYLHRIGRSGRFGHLGIAINLI

TYEDRFDLHRIEKELGTEIKPIPKVIDPALYVANVGASTGDTCNNSDLNNSANEEGNVSK

>ALC38298.1 me31B [Drosophila busckii]

MMTEKMAAGHSSLVNKGILSDHPIATNISEDMGWKSKLKLPPKDNRFKTTDVTDTRGNEFEEFCLKRPLLMGIFEKGWER

PSPIQEAAIPIALSGKDVLARAKNGTGKTGAYCIPVLEQIDPTKDYIQALVMVPTRELALQTSQICIELAKHLDIRVMVT

TGGTILKDDILRIYQKVQLIIATPGRILDLMDKKVADMSHCKILVLDEADKLLSLDFQGMLDHVILKLPKDPQILLFSAT

FPLTVKNFMEKHLREPYEINLMEELTLKGVTQYYAFVQERQKVHCLNTLFSKLQINQSIIFCNSTQRVELLAKKITELGY

CCYYIHAKMAQAHRNRVFHDFRQGLCRNLVCSDLFTRGIDVQAVNVVINFDFPRMAETYLHRIGRSGRFGHLGIAINLIT

YEDRFDLHRIEKELGTEIKPIPKVIDPALYVANVGGSAGEAANNSDLNNSTNEEGNVSK

>XP_017841041.2 ATP-dependent RNA helicase me31b [Drosophila busckii]

MMTEKMAAGHSSLVNKGILSDHPIATNISEDMGWKSKLKLPPKDNRFKTTDVTDTRGNEFEEFCLKRPLLMGIFEKGWER

PSPIQEAAIPIALSGKDVLARAKNGTGKTGAYCIPVLEQIDPTKDYIQALVMVPTRELALQTSQICIELAKHLDIRVMVT

TGGTILKDDILRIYQKVQLIIATPGRILDLMDKKVADMSHCKILVLDEADKLLSLDFQGMLDHVILKLPKDPQILLFSAT

FPLTVKNFMEKHLREPYEINLMEELTLKGVTQYYAFVQERQKVHCLNTLFSKLQINQSIIFCNSTQRVELLAKKITELGY

CCYYIHAKMAQAHRNRVFHDFRQGLCRNLVCSDLFTRGIDVQAVNVVINFDFPRMAETYLHRIGRSGRFGHLGIAINLIT

YEDRFDLHRIEKELGTEIKPIPKVIDPALYVANVGGSAGEAANNSDLNNSK

>AAA28603.1 RNA helicase [Drosophila melanogaster]

MMTEKLNSGHTNLTSKGIINDLQIAGNTSDDMGWKSKLNCRQRTTRFKTTDVTDTRGNEFEEFCLKRELLMGIFEKGWER

PSPIQEAAIPIALSGKDVLARAKNGTGKTGAYCIPVLEQIDPTKDYIQALVMVPTRELALQTSQICIELAKHLDIRVMVT

TGGTILKDDILRIYQKVQLIIATPGRILDLMDKKVADMSHCRILVLDEADKLLSLDFQGMLDHVILKLPKDPQILLFSAT

FPLTVKNFMEKHLREPYEINLMEELTLKGVTQYYAFVQERQKVHCLNTLFSKLQINQSIIFCNSTQRVELLAKKITELGY

CCYYIHAKMAQAHRNRVFHDFRQGLCRNLVCSDLFTRGIDVQAVNVVINFDFPRMAETYLHRIGRSGRFGHLGIAINLIT

YEDRFDLHRIEKELGTEIKPIPKVIDPALYVANVGASVGDTCNNSDLNNSANEEGNVSK

>NP_723539.1 maternal expression at 31B, isoform B [Drosophila melanogaster]

MGWKSKLKLPPKDNRFKTTDVTDTRGNEFEEFCLKRELLMGIFEKGWERPSPIQEAAIPIALSGKDVLARAKNGTGKTGA

YCIPVLEQIDPTKDYIQALVMVPTRELALQTSQICIELAKHLDIRVMVTTGGTILKDDILRIYQKVQLIIATPGRILDLM

DKKVADMSHCRILVLDEADKLLSLDFQGMLDHVILKLPKDPQILLFSATFPLTVKNFMEKHLREPYEINLMEELTLKGVT

QYYAFVQERQKVHCLNTLFSKLQINQSIIFCNSTQRVELLAKKITELGYCCYYIHAKMAQAHRNRVFHDFRQGLCRNLVC

SDLFTRGIDVQAVNVVINFDFPRMAETYLHRIGRSGRFGHLGIAINLITYEDRFDLHRIEKELGTEIKPIPKVIDPALYV

ANVGASVGDTCNNSDLNNSANEEGNVSK

>EDW86535.2 uncharacterized protein Dwil_GK21209 [Drosophila willistoni]

MGWKSKLKLPPKDNRFKTTDVTDTRGNEFEEFCLKRELLMGIFEKGWERPSPIQEAAIPIALSGKDVLARAKNGTGKTGA

YCIPVLEQIDPTKDYIQALVMVPTRELALQTSQICIELAKHLDIRVMVTTGGTILKDDILRIYQKVQLIIATPGRILDLM

DKKVADMSHCKILVLDEADKLLSLDFQGMLDHVILKLPKDPQILLFSATFPLTVKNFMEKHLREPYEINLMEELTLKGVT

QYYAFVQERQKVHCLNTLFSKLQINQSIIFCNSTQRVELLAKKITELGYCCYYIHAKMAQAHRNRVFHDFRQGLCRNLVC

SDLFTRGIDVQAVNVVINFDFPRMAETYLHRIGRSGRFGHLGIAINLITYEDRFDLHRIEKELGTEIKPIPKVIDPALYV

ANSGVSSGDTCNNSDLNNSTTEEGNVSK

>KAF5304652.1 hypothetical protein FQR65_LT18846 [Abscondita terminalis]

MMTDTLTTNNHVVPLQTKLDDKLDDIGWKSKLKVPPKDKRIKTSDVTDTRGNEFEEFCLKRELLMGIFEKGWEKPSPIQE

ASIPIALSGKDVLARAKNGTGKTGAYCIPVLEQVDPDKDYIQALILVPTRELALQTSQICIELAKHMRVRVMVTTGGTNL

RDDIMRIYQKVQPKMDQCRILVLDEADKLLSQDFKDMLHHLIARLPDERQILLFSATFPLTVKQFMEKHLRDPYEINLME

ELTLKGVTQYYAFVQERQKVHCLNTLFSKLQINQSIIFCNSTQRVELLAKKITELGYCCYYIHAKMAQAHRNRVFHDFRA

GLCRNLVCSDLFTRGIDVQAVNVVINFDFPKMAETYLHRIGRSGRFGHLGIAINLITYDDRFALHRIEQELGTEIKPIPK

MIDPSLYVANSEDELLEDSNK

>VEN37828.1 unnamed protein product [Callosobruchus maculatus]

MMTETIKSNNHMVALHKVDMDNKADDLGWKAKLKIPPKDRRIQTSDVTDTKGNEFEEFCLKRDLLMGIFEKGWERPSPIQ

EASIPIALSGKDVLARAKNGTGKTGAYCIPVLEQIDPKKDCIQALIIVPTRELALQTSQICIELAKHMDVRVMVTTGGTN

LRDDIMRIYQKVQVIIATPGRILDLMEKGVAVMDHCKILVLDEADKLLSQDFKGMLDTVIKNLPQERQILLFSATFPLTV

EQFMRKHLRDPYEINLMEELTLKGVTQYYAFVQERQKVHCLNTLFSKLQINQSIIFCNSTQRVELLAKKITELGYCCYYI

HAKMAQAHRNRVFHDFRAGLCRNLVCSDLFTRGIDVQAVNVVINFDFPKMAETYLHRIGRSGRFGHLGIAINLITYDDRC

MW

>XP_017847225.2 ATP-dependent RNA helicase me31b-like [Drosophila busckii]

MGWKSKLKLPPKDNRFKTTDVTDTRGNEFDEFCLKRPLLMGIFEIGWESPSPIQEAAIPSALRGKDVLARAKNGTEKTGA

YCIPVLEQIDPTKDYIQALVMVPTRELALQTSQICIELAKHLDIRVMVTTRGTILKDDILRIYQKVQLIIATPGRILDLM

DKKVADMSHCKILVLDEADKLLSLDFQGMLDHVILKLPKDPQILLFSATFPLTVKNFMEKHLREPYEISLVEELTLKGVT

QYYAFVQERHKVHCLNTLFSKLQINQSIIFCNSTQRVELLAKKITELGYCCYYIHAKMAQAHRNRVFHDFRQGLCRNLVC

SDLFTRGIDVQAVNVVINFDFPRMAETYLHRIGRSGRFGHLGIAINLITYEDRFDLHRVEKELGTEIKPIPKVIDPALYV

ANVGGSAEEAANNSDLNNSTI

>XP_018311394.1 PREDICTED: putative ATP-dependent RNA helicase me31b isoform X2 [Trachymyrmex zeteki]

MGIFEKGWEKPSPIQEASIPIALSGKDILARAKNGTGKTGAYSIPVLEQVDPRKDVIQALVIVPTRELALQTSQICIELA

KHMDIKVMVTTGGTNLRDDIMRIYQKVQVIIATPGRILDLMDKNVANMEHCKILVLDEADKLLSQDFKGMLDHVISRLPH

ERQILLYSATFPLTVKQFMEKHLRDPYEINLMEELTLKGVTQYYAFVQERQKVHCLNTLFSKLQITQSIIFCNSTQRVEL

LAKKITDLGYCCYYIHAKMAQAHRNRVFHDFRAGLCRNLVSSDLFTRGIDVQAVNVVINFDFPKMAETYLHRIGRSGRFG

HLGIAINLITYEDRFNLHRIEQELGTEIKPIPKVIDPSLYVARPEDNNSMEEGNVSK

>XP_018352978.1 PREDICTED: putative ATP-dependent RNA helicase me31b isoform X2 [Trachymyrmex septentrionalis]

MGIFEKGWEKPSPIQEASIPIALSGKDILARAKNGTGKTGAYSIPVLEQVDPRKDVIQALVIVPTRELALQTSQICIELA

KHMDIKVMVTTGGTNLRDDIMRIYQKVQVIIATPGRILDLMEKNVANMEHCKILVLDEADKLLSQDFKGMLDHVISRLPH

ERQILLYSATFPLTVKQFMEKHLRDPYEINLMEELTLKGVTQYYAFVQERQKVHCLNTLFSKLQITQSIIFCNSTQRVEL

LAKKITDLGYCCYYIHAKMAQAHRNRVFHDFRAGLCRNLVSSDLFTRGIDVQAVNVVINFDFPKMAETYLHRIGRSGRFG

HLGIAINLITYEDRFNLHRIEQELGTEIKPIPKVIDPSLYVARPEDNNSMEEGNVSK

>EDV98851.1 GH13374 [Drosophila grimshawi]

MMTEKIVPGHSSLVNKGILNDHQIANNLSDDMGWKSKLKLPPKDNRFKTTDVTDTRGNEFEEFCLKRELLMGIFEKGWER

PSPIQEAAIPIALSGKDVLARAKNGTGKTGAYCIPVLEQIDPTKDYIQALVMVPTRELALQTSQICIELAKHLDIRVMVT

TGGTILKDDILRIYQKVQLIIATPGRILDLMDKKVADMSHCKILVLDEADKLLSLDFQGMLDHVILKLPKDPQILLFSAT

FPLTVKNFMEKHLREPYEINLMEELTLKGVTQYYAFVQERQKVHCLNTLFSKLQINQSIIFCNSTQRVELLAKKITELGY

CCYYIHAKMAQAHRNRVFHDFRQGLCRNLVCSDLFTRGIDVQAVNVVINFDFPRMAETYLHRIGRSGRFGHLGIAINLIT

YEDRFDLHRXXXXXXXXXXXXXXXXXXXXNPDQTTYTCSSVSYICTKRMSSCMIKLGRQ

>XP_001992926.2 ATP-dependent RNA helicase me31b, partial [Drosophila grimshawi]

MMTEKIVPGHSSLVNKGILNDHQIANNLSDDMGWKSKLKLPPKDNRFKTTDVTDTRGNEFEEFCLKRELLMGIFEKGWER

PSPIQEAAIPIALSGKDVLARAKNGTGKTGAYCIPVLEQIDPTKDYIQALVMVPTRELALQTSQICIELAKHLDIRVMVT

TGGTILKDDILRIYQKVQLIIATPGRILDLMDKKVADMSHCKILVLDEADKLLSLDFQGMLDHVILKLPKDPQILLFSAT

FPLTVKNFMEKHLREPYEINLMEELTLKGVTQYYAFVQERQKVHCLNTLFSKLQINQSIIFCNSTQRVELLAKKITELGY

CCYYIHAKMAQAHRNRVFHDFRQGLCRNLVCSDLFTRGIDVQAVNVVINFDFPRMAETYLHRIGRSGRFGHLGIAINLIT

YEDRFDLHR

>XP_001842559.1 DEAD-box ATP-dependent RNA helicase 8 [Culex quinquefasciatus]

MMTETLNSNNHLGQKGEKVDDMGWKAKLKLPPKDTRIKTSDVTDTRGNEFEEFCLKRELLMGIFEKGWEKPSPIQEAAIP

IALVGKDILARAKNGTGKTGAYSIPVLEQIDPTKDYIQALIIVPTRELALQTSQICIELAKHMHIRVMVTTGGTNLKDDI

MRIYQKVQVIIATPGRILDLMDKEVANMANCRMLVLDEADKLLSQDFKGMLDHVIMKLPKERQILLFSATFPLSVKNFME

KHLREPYEINLMEELTLKGVTQYYAFVQERQKVHCLNTLFSKLQINQSIIFCNSTQRVELLAKKITELGYCCYYIHAKMQ

QAHRNRVFHDFRSGLCRNLVCSDLFTRGIDVQAVNVVINFDFPKMAETYLHRIGRSGRFGHLDLHSIHANNSHRHLEALA

LPSFGDPFLRWLTEREVLQLYRQAPP

>XP_008480260.1 putative ATP-dependent RNA helicase me31b [Diaphorina citri]

MGIFEKGWEKPSPIQEASIPIALTGKDILARAKNGTGKTGAYSIPVLEQIDPSKDYIQALIIVPTRELALQTSQICIELA

KHLNVKVMVTTGGTNLRDDIMRIYQKVHLIIATPGRILDLMDKQVANMDHCKILVLDEADKLLSQDFKGMLDHVISILPH

ERQILLYSATFPLTVKNFMEKHLKDPYEINLMEELTLKGVTQYYAFVQERQKVHCLNTLFSKLQINQSIIFCNSTQRVEL

LAKKITELGYCCYYIHARMAQAHRNRVFHDFRSGLCRNLVCSDLFTRGIDVQAVNVVINFDFPKMAETYLHRIGRSGRYG

HLGIAINLITYEDRYALHRIEKELGTEIKSIPKVIDPALYVARPEDMDRDNSTGASEENNVSK

>XP_021926685.1 putative ATP-dependent RNA helicase me31b [Zootermopsis nevadensis]

MMTETHINSNHVAVMPGLNSINTRKDEGDKVDDIGWKAKLKIPPKDRRVQTSDVTDTRGNEFEEFCLKRELLMGIFEKGW

EKPSPIQEASIPIALSGKDVLARAKNGTGKTGAYSIPVLEQVDPKKDCIQGKLSRLFDKRDRHLCTGVHLSVEYGTIISG

RDWRSIRVLVERLGGNPIVPLDMPIYFYLKADKLLSQDFKGMLDHVISRLPKERQILLFSATFPLTVKQFMEKHLREPYE

INLMEELTLKGVTQYYAFVQERQKVHCLNTLFSKLQINQSIIFCNSTQRVELLAKKITELGYCCYYIHAKMVQAHRNRVF

HDFRAGLCRNLVCSDLFTRGIDVQAVNVVINFDFPKMAETYLHRIGRSGRFGHLGIAINLITYEDRFALHRIEQELGTEI

KPIPKVIDPSLYVAKLEDSQGIEEANISK

>XP_017491194.1 PREDICTED: putative ATP-dependent RNA helicase me31b, partial [Rhagoletis zephyria]

RTQGKIITQNCNEKNHLRDDWKAKLKIPPKDNRVKTSDVTSTRGNDFEEYCLKRELLMGIFEQGWEKPSPIQESAIPVAL

LGNDVLARAKNGTGKTGAYTIPMLEKIDVNNNAIQALVVVPTRELAMQTSNICMELSRHMGVRVMVTTGGTNLKDDIMRI

YDNVHVVIATPGRILDLVEKKVASLNNCKYFVLDEADKLLGQDFKGMLDTLISYLPEERQILLFSATFPITVEQFMRKHL

KNPHEINLMEELTLKGVTQYYAFVQERQKVHCLNTLFSKLQINQSIIFCNSTQRVELLAKKIAELGYSCYYIHARMSQPH

RNRVFHDFRSGLCRNLVCSDLFTRGIDIQAVNVVINFDFPKMAETYLHRIGRSGRFGHLGIAINLITYEDRHLLHKIEAE

LNTSISPIPKVIDPQLYVAEAQEANHQDK

>VVC38604.1 Hypothetical protein CINCED_3A019670 [Cinara cedri]

MGIFEKGWEKPSPIQEASIPIALSGKDILARAKNGTGKTGAYSIPVLEQVDPKLDVIQALVIVPTRELALQTSQICIELA

KHLDIRVMVTTGGTNLKDDILRIYQRVHVIIATPGRILDLLDKSIAKVDHCRILVLDEADKLLSQDFKGMLDHIISKLPS

ERQILLYSATFPLTVKQFMDKHLRSPYEINLMEELTLKGVTQYYAFVQEKQKVHCLNTLFSKLQINQSIIFCNSTQRVEL

LAKKITDLGYCCYYIHAKMAQAHRNRVFHDFRKGSCRNLVCSDLFTRGIDVQAVNVVINFDFPKMAETYLHRIGRSGRFG

HLGIAINLITYDDRFALHRIEQELGTEIKPIPKVIDPRLYVARPEDVDINEEMDLSK

>ODM96281.1 putative ATP-dependent RNA helicase me31b [Orchesella cincta]

MSLVDMINSGSKVSGSPNTASLLDKKPDMMGIGMGGGQQQTGDWKKQLKMPPKDRRVKTSDVTDREGKEFEDFCLSRELL

MGIFEMGWEKPSPIQEASIPMALSGRDILARAKNGTGKTGAYSIPILERVDPKIDSIQALIIVPTRELALQTSQICTDLS

KHLKLKVMVTTGGTGLKDDIIRVYQKVHVIIATPGRIIDLMEKNVCKMDQCKMLVLDEADKLLSQDFNKMLDKLISFLPL

KRQILLFSATFPVTVEDFMKKHSIDPYKINLMDELTLKGVTQYYAFVQERQKVHCLNTLFSKLEINQSIIFCNSTQRVEL

LAKKITELGYACYYIHARMNQADRNRVFHDFRKGHCRNLVCSDLFTRGIDIQAVNVVINFDFPKMSETYLHRIGRSGRFG

HLGIAINLITYDDRFTLQKIENELGTEIKPIPKEIDKALYVASYQQEGAHEEEEHNGGQNGVK

>XP_021956660.1 ATP-dependent RNA helicase cgh-1 [Folsomia candida]

MSTAVVTNNGVVDADLAAALKAKMMLSSENNGGGGGGDWKAQLNLPPKDNRMRTSDVTDREGKEFEDFCLTRELLMGIFE

MGWEKPSPIQEASIPMALSGRDILARAKNGTGKTGAYSIPLLERIDPTLDAIQGLIVVPTRELALQVSQICTDLAKHLQV

KVMVTTGGTGLKDDIIRVYQKVHVIIATPGRIIDLMEKNVCKMDQCKMLVLDEADKLLSQDFNRMLDKLISFLPNKRQIL

LFSATFPITVESFMKKHAIDPYKINLMDELTLKGVTQYYAFVQERQKVHCLNTLFSKLEINQSIIFCNSTQRVELLAKKI

TDLGYACYYIHARMNQADRNRVFHDFRKGHCRNLVCSDLFTRGIDIQAVNVVINFDFPKMSETYLHRIGRSGRYGHLGIA

INLITYEDRFNLQKIEAELGTEIKPIPKEIDKALYVASFHQDGEEHGEGNNKEQNHAK

>XP_030246413.1 ATP-dependent RNA helicase me31b [Drosophila navojoa]

MMTEKIVPGHPSLLNKGMLSDHQTANNLADDMGWKSKLKLPPKDNRFKTTDVTDTRGNEFEEFCLKRELLMGIFEKGWER

PSPIQEAAIPIALSGKDVLARAKNGTGKTGAYCIPVLEQIDPTKDYIQALVMVPTRELALQTSQICIELAKHLDIRVMVT

TGGTILKDDILRIYQKVQLIIATPGRILDLMDKKVADMSHCKILVLDEADKLLSLDFQGMLDHVILKLPKDPQILLFSAT

FPLTVKNFMEKHLREPYEINLMEELTLKGVTQYYAFVQERQKVHCLNTLFSKLQINQSIIFCNSTQRVELLAKKITELGY

CCYYIHAKMAQAHRNRVFHDFRQGLCRNLVCSDLFTRGIDVQAVNVVINFDFPRMAETYLHRIGRSGRFGHLGK

>XP_017858962.1 PREDICTED: putative ATP-dependent RNA helicase me31b [Drosophila arizonae]

MMTEKIVPGHPSLINKGMLSDHQIASNLGDDMGWKSKLKLPPKDNRFKTTDVTDTRGNEFEEFCLKRELLMGIFEKGWER

PSPIQEAAIPIALSGKDVLARAKNGTGKTGAYCIPVLEQIDPTKDYIQALVMVPTRELALQTSQICIELAKHLDIRVMVT

TGGTILKDDILRIYQKVQLIIATPGRILDLMDKKVADMSHCKILVLDEADKLLSLDFQGMLDHVILKLPKDPQILLFSAT

FPLTVKNFMEKHLREPYEINLMEELTLKGVTQYYAFVQERQKVHCLNTLFSKLQINQSIIFCNSTQRVELLAKKITELGY

CCYYIHAKMAQAHRNRVFHDFRQGLCRNLVCSDLFTRGIDVQAVNVVINFDFPRMAETYLHRIGRSGRFGHLGK

>ERL89335.1 hypothetical protein D910_06707 [Dendroctonus ponderosae]

MLINKEAFTDLQEKYQKVIKLLLEDSKSDPENEPYLSKYSAKQILIGMKANIENLLRNHPAEGPENLKLTGMYSTVFLYL

GMVAVDTEEISIGEKHLEKCREIIEKLEALPEAVMIALNMYNQFGILWSQREPEKSKIYLEKAQQLYTSFKASGEAPINV

SEIFDPNLEKHDEELAFNNMEKIHTLTLYYLAQIFGKLKEDFKSAVYCHITLQRQLEMDDYEPIDWALNSATLSQFFMEK

CGFKQARHHLAASSYILSKYKIEDLNAATDINEEYEAKLETFNHRHADVARCWVKYGIFLLGKSKDRLLAHTEDIDEKCS

MVSDLSRMKLSGDAQVTIEDLQNLTFRGIDVGSYESQITDQFILQFSDAKIVFLQTQEWIKLAEEYYTLETLASDYIEIV

QDHTQLYLNLLFFEDNPDNQAKLHKRRIDLLENVVSKVNPQYYMNYCRQIWFELGHSYTSILDIKSDKLRESKEKPKPQA

LAKINILVDKCITHYTSFQSSFKVNIAELEKVQGDVEKPFLQTYFRVAALYGRYITMDRNVQLKNVEQQYEHYKFLTDYC

KKHPNAAELMPVELNICKEMVMDADVKMELKDDLGWKAKLKIPAKDHRIQTTDVTDTRGNEFEEYCLKRELLMGIFEKGW

EKPSPIQEAAIPIALTGKDVLARAKNGTGGTNLRDDIMRIYQKVQVIIATPGRILDLMEKGVAQMDQCKILVLDEADKLL

SQDFKGMLDVVIKNLPEKRQVLLFSATFPLTVEQFMRKHLRSPYEINLMEELTLKGVTQYYAFVQERQKVHCLNTLFSKL

QINQSIIFCNSTQRVELLAKKITELGYCCYYIHAKMAQTHRNRVFHDFRSGLCRNLVCSDLFTRGIDVQAVNVVINFDFP

KMAETYLHRIGRSGRFGHLGIAINLITYEDRFALHRIEQELGTEIKPIPKIIDPRLYVAKLGEEDPEDLVK

>KDR15838.1 Putative ATP-dependent RNA helicase me31b, partial [Zootermopsis nevadensis]

DVTDTRGNEFEEFCLKRELLMGIFEKGWEKPSPIQEASIPIALSGKDVLARAKNGTGKTGAYSIPVLEQVDPKKDCIQGK

LSRLFDKRDRHLCTGVHLSVEYATTPDMLNRADAIIREDRRITTRQLAQQLSVSNGSVIAIIQALAYSKADKLLSQDFKG

MLDHVISRLPKERQILLFSATFPLTVKQFMEKHLREPYEINLMEELTLKGVTQYYAFVQERQKVHCLNTLFSKLQINQSI

IFCNSTQRVELLAKKITELGYCCYYIHAKMVQAHRNRVFHDFRAGLCRNLVCSDLFTRGIDVQAVNVVINFDFPKMAETY

LHRIGRSGRFGHLGIAINLITYEDRFALHRIEQELGTEIKPIPKV

>PSN45934.1 putative ATP-dependent RNA helicase me31b, partial [Blattella germanica]

DLVTVTDDKGWKAKLKIPPKDKRVQTSDVTDTRGCEFEEFCLKRELLMGIFEKGWEKPSPIQEASIPIALSGKDILARAK

NGTGKTGAYSIPVLEKIDPEKDYIQALVIVPTRELALQTSQICIELSKHVGTKVMVTTGGTNLKDDIMRIYQKVQVIIAT

PGRILDLMDKNVADMQYCKILVLDEADKLLSQDFKGMLDHVISRLPKERQILLFSATFPLTVKQFMDKHLQEPYEINLME

ELTLKGVTQYYAFVQERQKLQINQSIIFCNSTQRVELLAKKITDLGYCCYYIHAKMAQAHRNRVFHDFRNGECRNLVCSG

ENL

>TGZ38081.1 putative ATP-dependent RNA helicase me31b [Temnothorax longispinosus]

MPRCLIKSMARYHKTDNSSEETEFPWLPPSTDLKRKSHTKDATSKTSNIWTCSGLPIVTRYSFHKSNGATFDAELNPAGQ

NCGNTHHDAASIDGHVNDNAGKVSTILLDGVPRFSSKAERSATQTSEAGKVAGVINPPVVINGCETQTLSQALYLLPKQK

HESVNAAEGKTASAREGAQHWKRNKTMHYCPYCRKSFDRPWVLKGHLRLHTGERPFECPVCHKSFADRSKYTLQDVTDTR

GNEFEEFCLKRELLMGIFEKGWEKPSPIQEASIPIALSGKDILARAKNGTGKTGAYSIPVLEQVDPRKDVIQALVIVPTR

ELALQTSQICIELAKHMDIKVMVTTGGTNLRDDIMRIYQKVQVIIATPGRILDLMDKNVANMEHCKILVLDEADKLLSQD

FKGMLDHVISRLPHERQILLYSATFPLTVKQFMEKHLRDPYEINLMEELTLKGVTQYYAFVQERQKVHCLNTLFSKVSSD

LFTRGIDVQAVNVVINFDFPKMAETYLHRIGRSGRFGHLGIAINLITYEDRFNLHRIEQELGTEIKPIPKVIDPSLYVAR

PEDNNSMEEGNVSK

>XP_013193669.1 PREDICTED: putative ATP-dependent RNA helicase me31b [Amyelois transitella]

MMTENRISSSNHVGNSLNNQKGDVDKTIDEVGWKSKLKIPPKDRRIKTSDVTDTRGNEFEEFCLKRELLMGIFEKGWEKP

SPIQEASIPIALSGKDVLARAKNGTGKTGAYCIPVLEQVDPKKDAIQALIVVPTRELALQTSQICIELAKHTDIRVMVTT

GGTNLKDDIMRIYQNVQVIIATPGRMIDLMDKQVAKMDQCRMLVLDEADKLLSQDFKGMLDKVIMRLPKERQILLFSATF

PLSVKQFMEKHLREPYEINLMEELTLKGVTQYYAFVQERQKVHCLNTLFSKVNINCIMWIYLCMLSYQHIVWTLFHDK

>KAF0755072.1 putative ATP-dependent RNA helicase me31b isoform X1 [Aphis craccivora]

MLATNHNNSGINLNMPSKQLNRISFEGIDDAGWKAKLKLPPQDHRIKTSDVTSTKGNDFEEFCLKRELLMGIFEKGWEKP

SPIQEASIPIALSGKDILARAKNGTGKTGAYSIPVLEQVDPKLDVIQALVIVPTRELALQTSQICIELAKHLDIRVMVTT

GGTNLKDDILRIYQRVRLEYLVYSDQQETTSGWRPIFVCSHAFPPLNSPKHWLPTLSPLCHCPPICMHEGVVLSQGPQYM

HVIIATPGRILDLLDKSIAKVDHCRILVLDEADKLLSQDFKGMLDHIISRLPSERQILLYSATFPLTVKQFMDKHLRSPY

EINLMEELTLKGVTQYYAFVQEKQKVHCLNTLFSKLQINQSIIFCNSTQRVELLAKKITDLGYCCYYIHAKMAQAHRNRV

FHDFRKGSCRNLVCSDLFTRGIDVQAVNVVINFDFPKMAETYLHRIGRSGRFGHLGIAINLITYDDRFALHRIEQELGTE

IKPIPKVIDPRLYVARPEDVDINEEMDLSK

>XP_026289677.1 putative ATP-dependent RNA helicase me31b [Frankliniella occidentalis]

MVXNVANMENCRILVLDEADKLLSQDFKGMLDHVISRLPKERQILLYSATFPLTVKQFMEKHLKEPYEINLMEELTLKGV

TQYYAFVQERQKVHCLNTLFSKLQINQSIIFCNSTQRVELLAKKITELGYCCYYIHAKMAQAHRNRVFHDFRNGLCRNLV

CSDLFTRGIDVQAVNVVINFDFPKMAETYLHRIGRSGRFGHLGIAINLITFDDRFALHRIEQELGTEIKPIPKVIDPALY

VAKSLDDSHAIEEANNVSK

>6S8R_A Chain A, ATP-dependent RNA helicase me31b [Drosophila melanogaster]

GPHMELTLKGVTQYYAFVQERQKVHCLNTLFSKLQINQSIIFCNSTQRVELLAKKITELGYCCYYIHAKMAQAHRNRVFH

DFRQGLCRNLVCSDLFTRGIDVQAVNVVINFDFPRMAETYLHRIGRSGRFGHLGIAINLITYEDRFDLHRIEKELGTEIK

PIPKVIDPALYV

>XP_017464136.1 PREDICTED: ATP-dependent RNA helicase dhh1-like, partial [Rhagoletis zephyria]

LIVKDFKEKHMRNPHEINLMDELTLRGITQYYAFVEERQKVHCLNTLFSKLQINQSIIFCNSTNRVELLAKKVTELGYSC

FFSHXXXXXGHRNRVFHDFRSGVCRNLVCSDLLTRGIDIQAVNVVINFDFPKNSETYLHRIGRSGRYGHLGLAINLITYE

DRFSLYKIEQELGTEIQPIPSQIDRSLYVAPNASNEDGQIQQSQQQNQQ

>XP_025075483.1 putative ATP-dependent RNA helicase me31b [Pogonomyrmex barbatus]

MVPVDPRKDVIQALVIVPTRELALQTSQICIELAKHMDIKVMVTTGGTNLRDDIMRIYQKVQVIIATPGRILDLMDKNVA

NMEHCKILVLDEADKLLSQDFKGMLDHVISRLPHERQILLYSATFPLTVKQFMEKHLRDPYEINLMEELTLKGVTQYYAF

VQERQKVHCLNTLFSKHDGAPRENSGVRGASVVAAVVGDVSGGGGPVVAVVVSSLVVVVRLQSIRRGGGTEELLVGKRRE

>VEN37829.1 unnamed protein product [Callosobruchus maculatus]

MMTETIKSNNHMVALHKVDMDNKADDLGWKAKLKIPPKDRRIQTSDVTDTKGNEFEEFCLKRDLLMGIFEKGWERPSPIQ

EASIPIALSGKDVLARAKNGTGKTGAYCIPVLEQIDPKKDCIQALIIVPTRELALQTSQICIELAKHMDVRVMVTTGGTN

LRDDIMRIYQKVQE

>XP_011647542.1 putative ATP-dependent RNA helicase me31b, partial [Pogonomyrmex barbatus]

LQITQSIIFCNSTQRVELLAKKITDLGYCCYYIHAKMAQAHRNRVFHDFRAGLCRNLVSSDLFTRGIDVQAVNVVINFDF

PKMAETYLHRIGRSGRFGHLGIAINLITYEDRFNLHRIEQELGTEIKPIPKVIDPSLYVARPEDNNSMEEGNVSK
