# Supplementary material for: DDX6 Is Essential for Oocyte Development and Maturation in Locusta migratoria
Source: Insects. 2021 Jan 14;12(1):70. doi: 10.3390/insects12010070 (PMC7830464; doi:10.3390/insects12010070)
Supplement: Supplementary file 1 [file insects-12-00070-s001.zip › Supplementary material/Supplemental file 4.docx]

**500 sequences of DDX6 from fungi**

>ORY79665.1 DEAD-domain-containing protein [Neocallimastix californiae]

MSVNINALSQQLQNTNISDDKSWKSQLKLPPKDLRPKTEDVTATKGNEFEDYYLKRKLLMGIFEAGFEKPSPIQEESIPI

ALAGRDILARAKNGTGKTAAFVIPALEKVDNKKDHIQAAVLVPTRELALQTSQVFKTLGKHLNVKIMVTTGGTTLKDDIL

RLGETVHILVGTPGRMLDLAGKGVANLSQCQVLIMDEADKLLSPEFQPIVEQLISHFPENRQILLFSATFPIIVKNFKDK

FLKEPYEINLMDELTLRGVTQYYAYVEERQKVHCLNTLFSKLQINQSIIFCNSTSRVELLAKKITELGYSCFYIHAKMLQ

SHRNRVFHDFRNGKCRNLVCSDLLTRGIDIQAVNVVINFDFPKNAETYLHRIGRSGRFGHLGLAINLITYDDRFNLYRIE

QELGTEIQSIPPVIDKNLYVAPSV

>ORX55327.1 DEAD-domain-containing protein [Piromyces finnis]

MSVNINALSQQLQNTTISDSDKSWKSQLKLPPKDLRPKTEDVTATKGNEFEDYYLKRKLLMGIFEAGFEKPSPIQEESIP

IALAGRDILARAKNGTGKTAAFVIPALEKVDNKKDYIQAAVLVPTRELALQTSQVFKTLGKHLNVKIMVTTGGTTLKDDI

LRLGETVHILVGTPGRMLDLAGKGVANLSQCGVLIMDEADKLLSPEFQPIVEQLISHFPENRQILLYSATFPIIVKNFKD

KFLKEPYEINLMDELTLRGVTQYYAYVEERQKVHCLNTLFSKLQINQSIIFCNSTSRVELLAKKITELGYSCFYIHAKML

QSHRNRVFHDFRNGKCRNLVCSDLLTRGIDIQAVNVVINFDFPKNAETYLHRIGRSGRFGHLGLAINLITYDDRFNLYRI

EQELGTEIQSIPPVIDKNLYVAPSV

>ORX78599.1 DEAD-domain-containing protein [Anaeromyces robustus]

MSVNINALSQQLQNTTISDSDKSWKSQLKLPPKDLRPKTEDVTATKGNEFEDYYLKRKLLMGIFEAGFEKPSPIQEESIP

IALAGRDILARAKNGTGKTAAFVIPALEKVDNKKDHIQAAVLVPTRELALQTSQVFKTLGKHLNVKIMVTTGGTTLKDDI

LRLGETVHILVGTPGRMLDLAGKGVANLSQCQVLIMDEADKLLSPEFQPIVEQLISHFPANRQILLFSATFPIIVKNFKD

KFLKEPYEINLMDELTLRGVTQYYAYVEERQKVHCLNTLFSKLQINQSIIFCNSTSRVELLAKKITELGYSCFYIHAKML

QSHRNRVFHDFRNGKCRNLVCSDLLTRGIDIQAVNVVINFDFPKNAETYLHRIGRSGRFGHLGLAINLITYDDRFNLYRI

EQELGTEIQSIPPVIDKNLYVAPSV

>OUM57267.1 hypothetical protein PIROE2DRAFT_65100 [Piromyces sp. E2]

MSVNINALSQQLQNTTISDSDKSWKSQLKLPPKDLRPKTEDVTATKGNEFEDYYLKRKLLMGIFEAGFEKPSPIQEESIP

IALAGRDILARAKNGTGKTAAFVIPALEKVDNKKDHIQAVVLVPTRELALQTSQVFKTLGKHLNVKIMVTTGGTTLKDDI

LRLGETVHILVGTPGRMLDLAGKGVANLSQCGVLIMDEADKLLSPEFQPIVEQLISHFPENRQILLYSATFPIIVKNFKD

KFLKEPYEINLMDELTLRGVTQYYAYVEERQKVHCLNTLFSKLQINQSIIFCNSTSRVELLAKKITELGYSCFYIHAKML

QSHRNRVFHDFRNGKCRNLVCSDLLTRGIDIQAVNVVINFDFPKNAETYLHRIGRSGRFGHLGLAINLITYDDRFNLYRI

EQELGTEIQSIPPVIDKNLYVAPSV

>ORY74224.1 DEAD-domain-containing protein [Neocallimastix californiae]

MSTNINALSQQLQNTTISDSDKSWKSQLKLPPKDLRPKTEDVTATKGNEFEDYYLKRTLLMGIFEAGFEKPSPIQEESIP

IALAGRDILARAKNGTGKTAAFVIPALEKVDNKKDYIQAAVLVPTRELALQTSQVFKTLGKHLNVKIMVTTGGTTLKDDI

LRLGETVHILVGTPGRMLDLAGKGVANLSQCQVLIMDEADKLLSPEFQPIVEQLISHFPENRQILLFSATFPIIVKNFKD

KFLKEPYEINLMDELTLRGVTQYYAYVEERQKVHCLNTLFSKLQINQSIIFCNSTSRVELLAKKITELGYSCFYIHAKML

QSHRNRVFHDFRNGKCRNLVCSDLLTRGIDIQAVNVVINFDFPKNAETYLHRIGRSGRFGHLGLAINLITYDDRFNLYRI

EQELGTEIQSIPPVIDKNLYVAPSV

>EJU02445.1 DEAD-domain-containing protein [Dacryopinax primogenitus]

MAEGSSKSHTSISTEDDWKAGLKAPPKDQRPQTEDVTATQGHSFEDYYLKRELLMGLFEMGFEKPSPIQEAAIPVALTKR

DILARAKNGTGKTGAFVIPTLQQVDVEKNHIQALILVPTRELALQTSQICKQLGKHMGIKVMVTTGGTTLRDDIMRLGET

VHVLVGTPGRILDLSSKGVADLAKCPIFVMDEADKLLSAEFTPVMEQLLSHLPEDRQVMLFSATFPLSVSQFKEDHMKRP

YEINLMEELTLRGVTQYYVFLEEKQKVHCLNTLFSKLQINQSIIFCNSTNRVELLAKKITELGYSCFFSHAKMLQQHRNR

VFHDFRNGVCRNLVCSDLLTRGIDIQAVNVVINFDFPKTSETYLHRIGRSGRFGHLGLAINLITYEDRFNLYRIEQELGT

EIQPIPPDIDKSLYVAPVNDDEEADRERQRAQALAARRANANNNANRGGGAASRTQQFANAPRQGAQSQQRPPQSQAPSQ

PQTQTQPPRQPNYAQISQSVPQQPIQYQQHAVAPPTQGGYPINSLPQQYIPQPVAHSGGMHPAQQRY

>KFH64831.1 ATP-dependent RNA helicase dhh1 [Mortierella verticillata NRRL 6337]

MAANQQQHMAHENDSWKKQLNLPTKDSRPQTEDVTATKGNEFEDYFLKRELLMGIFEAGFERPSPIQEESIPIALAGRDI

LARAKNGTGKTAAFVIPSLEKVNLKKNKIQAMLLVPTRELALQTAQVCKTLGKHMGVQVMVTTGGTTLKDDIMRLGETVH

IVVGTPGRILDLAGKGVANFSECTTFVMDEADKLLSPEFSPIVEQLLTYFPKERQIMLYSATFPLIVKTFKDKHLVKPYE

INLMDELTLRGVTQYYAFVEERQKVHCLNTLFSKLQINQSIIFCNSTNRVELLAKKITELGYSCFYSHAKMLQSHRNRVF

HDFRNGVCRNLVCSDLLTRGIDIQAVNVVINFDFPKNAETYLHRIGRSGRFGHLGLAINLITYEDRFNLYKIEQELGTEI

QAIPPVIDKRLYVAPSALDAQQEMQNQHEEQQKLLHQKQQLQQQQLQQQQQQQHQNQFQHQQQMNHHHHQQMQYHQYQQH

PQQMNHQRPPQQRR

>PJF18548.1 ATP-dependent RNA helicase dhh1 [Paramicrosporidium saccamoebae]

MNPKTSTIAYTKRGAPSINGRPAAPSSIDRQSRPGVPAAPTTQSDDWKNQLHAPPKDHRPQTEDVLATKGNEFEDYFLKR

ELLMGIFEMGYERPSPIQEESIPIALAGRDILARAKNGTGKTAAFIIPTLEKIDPNKNAIQALLLVPTRELALQTAHVCK

ELAKHMGKVQVMVTTGGTLLQDDIVRLYNPVHIIVATPGRVLDLASRGIANMKECNLMVMDEADKLLSPEFQPIVDEIIR

LLPRQRQILMYSATFPMIVKEFKDKHLNKPYEVNLMDELTLKGVTQYYAYVEERQKVHCLNTLFSKLQINQSIIFCNSTN

RVELLAKKITELGYSCFYIHAKMPQADRNRVFHDFRNGKCRNLVSTDLFTRGIDIQAVNVVINFDFPKNAETHLHRIGRS

GRFGHLGVAVNLVTYEDRFNLYRIEQELGTEIAPIPPTIDTKLYVA

>RHZ79004.1 hypothetical protein Glove_152g89 [Diversispora epigaea]

MPMANNLANQQPHAHLNHENDSWKKSLRLPPKDNRPQTEDVTATKGNEFEDYFLKRELLMGIFEAGFERPSPIQEESIPI

ALTGRDILARAKNGTGKTAAFVIPALEKINVKKQKIQALLLVPTRELALQTSQVCKMLGKHMGVQVMVTTGGTTLKDDIL

RLSEIVHIVVGTPGRILDLAGKGVADFSDCPTFVMDEADKLLSPEFSPIVEQLLTYFPKDRQIMLYSATFPLIVKSFKDK

YLVKPYEINLMDELTLRGVTQYYAFVEERQKVHCLNTLFSKLQINQSIIFCNSTNRVELLAKKITELGYSCFYSHAKMLQ

SHRNRVFHDFRNGVCRNLVCSDLLTRGIDIQAVNVVINFDFPKNAETYLHRIGRSGRFGHLGLAINLITYEDRFNLYKIE

QELGTEIQPIPPVIDKRLYVAPTALDDAQQQQQQQQQQQQQQQQQQQQQQNNPNIRNPNSRNNGSNNLRNSNTGRSGMSV

KAAS

>XP_019022565.1 ATP-dependent RNA helicase DHH1 [Saitoella complicata NRRL Y-17804]

MSAEQLSQQVASMNISDESNWKNQLSLPAKDTRPQTEDVTATKGNEFEDFYLKRELLMGIYEAGFEKPSPIQEESIPIAL

TGRDILARAKNGTGKTAAFTIPALEKVNTKSTKIQALILVPTRELALQTSQVCKTLGKHMGVNVMVTTGGTTLKDDIIRL

ADPVHILVGTPGRILDLAGKGVADFSECPTFIMDEADKLLSPEFTPVIEQLLAYFPKDRQVMLYSATFPLVVKSFMDKHL

NKPYEINLMDELTLRGVTQYYAFVEEKQKVHCLNTLFSKLQINQSIIFCNSTNRVELLAKKITELGYSCFYSHAKMLQNH

RNRVFHDFRNGACRNLVCSDLLTRGIDIQAVNVVINFDFPKNAETYLHRIGRSGRFGHLGIAINLINWEDRFNLYKIEQE

LGTEIQPIPPTIDKRLYVAPNAMDTPTQVPRPMPPAGAQQQQHQPTRSLYQQPGQYQQRQQYQRQNHNSARGPGPQSNGA

AMGRNPRYGQNQPRGNGYPVEAEPSGSVPAQ

>KFH66615.1 ATP-dependent RNA helicase dhh1 [Mortierella verticillata NRRL 6337]

MAANQQQHMAHENDSWKKQLNLPTKDSRPQTEDVTATKGNEFEDYFLKRELLMGIFEAGFERPSPIQEESIPIALAGRDI

LARAKNGTGKTAAFVIPSLEKVNLKKNKIQALLLVPTRELALQTAQVCKTLGKHMGVQVMVTTGGTTLKDDIMRLGETVH

IVVGTPGRILDLAGKGVADFSECSTFVMDEADKLLSPEFSPIVEQLLTYFPKERQIMLYSATFPLIVKTFKDKHLVKPYE

INLMDELTLRGVTQYYAFVEERQKVHCLNTLFSKLQINQSIIFCNSTNRVELLAKKITELGYSCFYSHAKMLQSHRNRVF

HDFRNGVCRNLVCSDLLTRGIDIQAVNVVINFDFPKNAETYLHRIGRSGRFGHLGLAINLITYEDRFNLYKIEQELGTEI

QAIPPVIDKRLYVAPSSLDSQQMEHEEQQKRIQQQQQQQQMQHFQQQQMHHHLQQQQYQHPQFQQQRVQHQQQRR

>KZO94215.1 DEAD-domain-containing protein [Calocera viscosa TUFC12733]

MAEGSSKVQSPVSTEENWKAGLKAPPKDTRPQTEDVTATEGHSFEDYYLQRELLMGLFEMGFENPSPIQEAAIPVALTKR

DILARAKNGTGKTGAFVIPTLQQVDVTKKYIQALILVPTRELALQTSQICKQLGKHMGVKVMVTTGGTTLKDDIMRLAED

VHVLVGTPGRILDLASKGIADLSKCPIFVMDEADKLLSAEFTPVMEQLLSHLPEDRQVMLFSATFPMSVAQFKEDHMKRP

YEINLMEELTLRGVTQYYVFLEEKQKVHCLNTLFSKLQINQSIIFCNSTNRVELLAKKITELGYSCFFSHAKMLQQHRNR

VFHDFRNGVCRNLVCSDLLTRGIDIQAVNVVINFDFPKTSETYLHRIGRSGRFGHLGLAINLITYEDRFNLYRIEQELGT

EIQPIPPDIDKSLYVAPFNEDEEADRERQRAQALARRNNANVNRGGSAASRTQQFANAPRAQGQGQPRPQAQAPQQPQAR

QPNYAQISQSGAYQQQPQQPAPQPVQYQQYPPPPQGGYPINPPPQQYMAPPVAHNGIHPAQQRY

>XP_016606024.1 ATP-dependent RNA helicase dhh1 [Spizellomyces punctatus DAOM BR117]

MSVNGLAMQLQNTHISNHDNNDWKRSLKLPAKDNRPQTEDVTATKGNDFEDYFLKRELLMGIFEAGFEKPSPIQEEAIPI

ALAGRDILARAKNGTGKTAAFIIPALEKVNPSKNHIQALILVPTRELALQTSQVCRNLGKHMGIQVMVTTGGTTLKDDIL

RLGQTVHILVATPGRVLDLAGKGVADLRECSMLIMDEADKLLSPEFQPIIEQLISFCRADRQILLFSATFPMIVKNFKDK

YLTKPYEINLMDELTLRGVTQYYAYVEERQKVHCLNTLFSKLQINQSIIFCNSTSRVELLAKKITELGYSCFYIHAKMLQ

SHRNRVFHDFRNGKTRHLVCSDLLTRGIDIQAVNVVINFDFPKNAETYLHRIGRSGRFGHLGLAINLITYEDRFNLYRIE

QELGTEIAPIPPVIDKSLYVAP

>XP_025187233.1 eukaryotic translation initiation factor 4A-like protein [Rhizophagus irregularis DAOM 181602=DAOM 197198]

MAGILPNHQQPHHLNHENDSWKKNLRLPPKDHRPQTEDVTATKGNEFEDYFLKRELLMGIFEAGFERPSPIQEESIPIAL

TGRDILARAKNGTGKTAAFVIPALEKINVKKPKIQALLLVPTRELALQTSQVCKTLGKHMGVQVMVTTGGTTLKDDILRL

SEIVHIVVGTPGRILDLAGKGVADFSECPTFVMDEADKLLSPEFSPIVEQLITYFPKDRQIMLYSATFPLIVKSFKDKYL

VKPYEINLMDELTLRGVTQYYAFVEERQKVHCLNTLFSKLQINQSIIFCNSTNRVELLAKKITELGYSCFYSHAKMLQSH

RNRVFHDFRNGVCRNLVCSDLLTRGIDIQAVNVVINFDFPKNAETYLHRIGRSGRFGHLGLAINLITYDDRFNLYKIEQE

LGTEIQPIPPVIDKRLYVAPSALDDAQTQQIQNNQNNQNINSQNNQNIQNIQNNRSQSHQNHQSHQNSRSSGGMRNTNAR

GGGMGVKAAS

>RGB44106.1 DEAD-domain-containing protein [Rhizophagus diaphanus] [Rhizophagus sp. MUCL 43196]

MAGILPNHQQPHHLNHENDSWKKNLRLPPKDHRPQTEDVTATKGNEFEDYFLKRELLMGIFEAGFERPSPIQEESIPIAL

TGRDILARAKNGTGKTAAFVIPALEKINVKKPKIQALLLVPTRELALQTSQVCKTLGKHMGVQVMVTTGGTTLKDDILRL

SEIVHIVVGTPGRILDLAGKGVADFSECPTFVMDEADKLLSPEFSPIVEQLITYFPKDRQIMLYSATFPLIVKSFKDKYL

VKPYEINLMDELTLRGVTQYYAFVEERQKVHCLNTLFSKLQINQSIIFCNSTNRVELLAKKITELGYSCFYSHAKMLQSH

RNRVFHDFRNGVCRNLVCSDLLTRGIDIQAVNVVINFDFPKNAETYLHRIGRSGRFGHLGLAINLITYDDRFNLYKIEQE

LGTEIQPIPPVIDKRLYVAPSALDDAQTQQIQNNQNNQNINSQNNQNSQNIQNNRSQSHQNHQSHQNSRSSGGGMGVKAA

S

>KAF0520877.1 DEAD-domain-containing protein [Gigaspora margarita]

MANNLANQQSHHHLNHENDSWKKNLRLPPKDNRPQTEDVTNTKGNEFEDYFLKRELLMGIFEAGFERPSPIQEESIPIAL

TGRDILARAKNGTGKTAAFVIPALEKINVKKHKIQALLLVPTRELALQTSQVCKTLGKHMGVQVMVTTGGTTLKDDILRL

SEIVHIVVGTPGRILDLAGKGVADFNECPTFVMDEADKLLSPEFSPIVEQLLTYFPKDRQIMLYSATFPLIVKSFKDKYL

VKPYEINLMDELTLRGVTQYYAFVEERQKVHCLNTLFSKLQINQSIIFCNSTNRVELLAKKITELGYSCFYSHAKMLQSH

RNRVFHDFRNGVCRNLVCSDLLTRGIDIQAVNVVINFDFPKNAETYLHRIGRSGRFGHLGLAINLITYEDRFNLYKIEQE

LGTEIQPIPPVIDKRLYVAPSALDDVQSQQQHQNNQNIRNQNSRNSGGMRHPNSGRSGMGVKAAS

>KZT60936.1 DEAD-domain-containing protein [Calocera cornea HHB12733]

MARRTDEDWKAGLKAPPKDSRPQTEDVTATEGHSFEDYYLKRELLMGLFEMGFENPSPIQEAAIPVALTKRDILARAKNG

TGKTGAFVIPTLQQIDVSKNHIQALILVPTRELALQTSQICKTLGKHMGVKVMVTTGGTTLKDDIMRLSEAVHVLVGTPG

RILDLASKGIADLSKCPIFVMDEADKLLSAEFTPVMEQLLSHLPEDRQVMLFSATFPMSVAQFKEDHMNRPYEINLMEEL

TLRGVTQYYVFLEEKQKVHCLNTLFSKLQINQSIIFCNSTNRVELLAKKITELGYSCFFSHAKMLQQHRNRVFHDFRNGV

CRNLVCSDLLTRGIDIQAVNVVINFDFPKTSETYLHRIGRSGRFGHLGLAINLITYEDRFNLYRIEQELGTEIQPIPPDI

DKSLYVAPFNEDEEADRERQRAQALARRNNANSTNRGASGASRTQQSNAPRSQGQPRAQAQAPPQAQPRQPNYAQISQSA

PYPQQQPQQPQQPMQYQQYAPPPPQGAYPMNAPPQQYMTAPVAHNGIHPGQQRY

>GAO48187.1 hypothetical protein G7K_2367-t1 [Saitoella complicata NRRL Y-17804]

MSAEQLSQQVASMNISDESNWKNQLSLPAKDTRPQTEDVTATKGNEFEDFYLKRELLMGIYEAGFEKPSPIQEESIPIAL

TGRDILARAKNGTGKTAAFTIPALEKVNTKSTKIQALILVPTRELALQTSQVCKTLGKHMGVNVMVTTGGTTLKDDIIRL

ADPVHILVGTPGRILDLAGKGVADFSECPTFIMDEADKLLSPEFTPVIEQLLAYFPKDRQVMLYSATFPLVVKSFMDKHL

NKPYEINLMDELTLRGVTQYYAFVEEKQKVHCLNTLFSKLQINQSIIFCNSTNRVELLAKKITELGYSCFYSHAKMLQNH

RNRVFHDFRNGACRNLVCSDLLTRGIDIQAVNVVINFDFPKNAETYLHRIGRSGRFGHLGIAINLINWEDRFNLYKIEQE

LGTEIQPIPPTIDKRLYVAPNAMDTPTQVPRPMPPAGAQQQQHQPTRSLYQQPGQYQQRQQYQRQNHNSARGPGPQSNGA

AMGRNPSAVKQKCVCKYVEIGRRCGLLNGGLYGACRCLFGGEEVGSRFLCRCPCHVLFHGLSENALFGWTGTAESTRPEG

SLVALDVVKIQTVCGHY

>OAQ34265.1 DEAD-domain-containing protein [Mortierella elongata AG-77]

MAANQQQQLAHDNDSWKKQLNLPTKDTRPQTEDVTATKGNEFEDYFLKRELLMGIFEAGFERPSPIQEESIPIALAGRDI

LARAKNGTGKTAAFLIPTLEKVNVKKNTVQALLLVPTRELALQTAQVCKTLGKHMGVQVMVTTGGTTLKDDIMRLGETVH

IIVGTPGRILDLAGKGVANFSECTTFVMDEADKLLSPEFTPIVEQLLTYFPKERQVMLYSATFPMIVKTFKDKHMVKPYE

INLMDELTLRGVTQYYAFVEERQKVHCLNTLFSKLQINQSIIFCNSTNRVELLAKKITELGYSCFYSHAKMLQSHRNRVF

HDFRNGVCRNLVCSDLLTRGIDIQAVNVVINFDFPKNAETYLHRIGRSGRFGHLGLAINLITYEDRFNLYKIEQELGTEI

QAIPPVIDTRLYVAPSALDDQQQNDHQEQQRLLHQKQQLQQQMHQQQYQQQMLPQQHYPQHQMHPNHQQFQQHHQQRAQQ

YQRR

>GBC02982.1 hypothetical protein RclHR1_04910015 [Rhizophagus clarus]

MAGILPNHQQPHHLNHENDSWKKNLRLPPKDHRPQTEDVTATKGNEFEDYFLKRELLMGIFEAGFERPSPIQEESIPIAL

TGRDILARAKNGTGKTAAFVIPALEKINVKKPKIQALLLVPTRELALQTSQVCKTLGKHMGVQVMVTTGGTTLKDDILRL

SEIVHIVVGTPGRILDLAGKGVADFSECPTFVMDEADKLLSPEFSPIVEQLITYFPKDRQIMLYSATFPLIVKSFKDKYL

VKPYEINLMDELTLRGVTQYYAFVEERQKVHCLNTLFSKLQINQSIIFCNSTNRVELLAKKITELGYSCFYSHAKMLQSH

RNRVFHDFRNGVCRNLVCSDLLTRGIDIQAVNVVINFDFPKNAETYLHRIGRSGRFGHLGLAINLITYDDRFNLYKIEQE

LGTEIQPIPPVIDKRLYVAPSALDDAQTQQQIQNNQNNQNSQNSNHQNNPHSQNSQNNRSQSHQNHQSHQNSRSSSGMRN

TNARGGGMGVKAAS

>ORY92726.1 P-loop containing nucleoside triphosphate hydrolase protein [Leucosporidium creatinivorum]

MSRPQGSSSSNPSPSTSNSNNASASSSSAPAPPQPNNWKQQLSLPAKDNRPQTEDVTATKGNEFEDYFLKRELLMGIFEA

GFERPSPIQEEAIPIALTGRDILARAKNGTGKTAAFIIPTLERVNPKVNKIQALLLVPTRELALQTSQVCKTLGKHTQAQ

IMVTTGGTTLRDDIMRLSETVHILVGTPGRILDLAGKGIADLSECPMFVMDEADKLLSPEFTPVIEQLLSFMPKERQVML

FSATFPLIVKDFKDKWMQKPYEINLMDELTLRGVTQYYAFLEERQKVHCLNTLFSKLQINQSIIFCNSTNRVELLAKKIT

ELGYSCFYSHAKMLQQHRNRVFHDFRNGVCRNLVCSDLLTRGIDIQAVNVVINFDFPKNAETYLHRIGRSGRYGHLGLAI

NLITYEDRFDLYRIEQELGTEIQPIPAVIDRSLYVAPGASDEPGQNSRPAGVTQVPAPMPQQSQAPPPQQQYSQRPPHQQ

NSNNPQQPIPQYNNQQSYNNQQQQAQGYGGGGYGQQQQGRPQQQQGQQGQRNGQPQQNGQRGYPQQGYPQQGRQ

>PKC70917.1 DEAD-domain-containing protein [Rhizophagus irregularis]

MAGILPNHQQPHHLNHENDSWKKNLRLPPKDHRPQTEDVTATKGNEFEDYFLKRELLMGIFEAGFERPSPIQEESIPIAL

TGRDILARAKNGTGKTAAFVIPALKKINVKKPKIQALLLVPTRELALQTSQVCKTLGKHMGVQVMVTTGGTTLKDDILRL

SEIVHIVVGTPGRILDLAGKGVADFSECPTFVMDEADKLLSPEFSPIVEQLITYFPKDRQIMLYSATFPLIVKSFKDKYL

VKPYEINLMDELTLRGVTQYYAFVEERQKVHCLNTLFSKLQINQSIIFCNSTNRVELLAKKITELGYSCFYSHAKMLQSH

RNRVFHDFRNGVCRNLVCSDLLTRGIDIQAVNVVINFDFPKNAETYLHRIGRSGRFGHLGLAINLITYDDRFNLYKIEQE

LGTEIQPIPPVIDKRLYVAPSALDDAQTQQIQNNQNNQNINSQNNQNIQNIQNNRSQSHQNHQSHQNSRSSGGMRNTNAR

GGGMGVKAAS

>CEP17217.1 hypothetical protein [Parasitella parasitica]

MASQQQLSHENDNWKKDLALPQKDTRPQTEDVTATKGNEFEDYFLKRELLMGIFEAGFERPSPIQEEAIPIALTGRDILA

RAKNGTGKTAAFVIPTLEKINNKKDRIQALLLVPTRELALQTAQVCKNLGKHLNIQVMVTTGGTTLKDDIMRLGETVHVV

VGTPGRILDLASKGVADFSEASTFVMDEADKLLSPEFTPIIDQLISHFPKDRQIMLFSATFPMIVKSFKDKYLVKPYEIN

LMDELTLRGVTQYYAYVEEKQKVHCLNTLFSKLQINQSIIFCNSTNRVELLAKKITELGYSCFYSHAKMLQSHRNRVFHD

FRNGVCRNLVCSDLLTRGIDIQAVNVVINFDFPKNAETYLHRIGRSGRFGHLGLAINLITYEDRFNLYKIERELGTEIQP

IPPVIDKSLYVAPNALEDAQVQQPNRQLAIATRQQQQQRDQTQYNNNSDSNPQQQNHHQNGYQQQYRGNYNNSGRGGGRR

QQQQQRGSSSRS

>RIA97337.1 P-loop containing nucleoside triphosphate hydrolase protein [Glomus cerebriforme]

MAGILPNHHQQPHHLNHENDSWKKNLRLPPKDHRPQTEDVTATKGNEFEDYFLKRELLMGIFEAGFERPSPIQEESIPIA

LTGRDILARAKNGTGKTAAFVIPALEKINVKKQKIQALLLVPTRELALQTSQVCKNLGKHMGVQVMVTTGGTTLKDDILR

LSEIVHIVVGTPGRILDLAGKGVADFSECPTFVMDEADKLLSPEFSPIVEQLITYFPKDRQIMLYSATFPLIVKSFKDKY

LVKPYEINLMDELTLRGVTQYYAFVEERQKVHCLNTLFSKLQINQSIIFCNSTNRVELLAKKITELGYSCFYSHAKMLQS

HRNRVFHDFRNGVCRNLVCSDLLTRGIDIQAVNVVINFDFPKNAETYLHRIGRSGRFGHLGLAINLITYDDRFNLYKIEQ

ELGTEIQPIPPVIDKRLYVAPSALDDAQTQQQIQNNQTNQNSQNSRSQSHQSHQSHQNPRSSGGMRNTNARGGGMGVKAA

S

>OZJ04488.1 ATP-dependent RNA helicase dhh1 [Bifiguratus adelaidae]

MLNNTTAPALNHENDKWKQSLHLPPKDSRPQTEDVTNTKGNEFEDYFLKRELLMGIFEAGFERPSPIQEEAIPIALTGRD

ILARAKNGTGKTAAFVIPSLEKVNVKKPKIQALLLVPTRELALQTSQVCKNLGKHMDVQVMVTTGGTTLKDDILRLQETV

HIVVGTPGRILDLASKGIADFSECPTFVMDEADKLLSPEFSPIVEQLIQYFPKDRQIMLFSATFPLLVKNFKDKYLVKPY

EINLMDELTLRGVTQYYAFVEERQKVHCLNTLFSKLQINQSIIFCNSTNRVELLAKKITELGYSCFYSHAKMLQSHRNRV

FHDFRNGVCRNLVCSDLLTRGIDIQAVNVVINFDFPKNAETYLHRIGRSGRFGHLGLAINLITYEDRFNLYKIEQELGTE

IQPIPPVIDKKLYVAPSAIDDPQQQQQQPQQTKQQQMQRGYPNQQFQQPYPPHHRNNYHHHNPHHQNPRRA

>OAQ31408.1 DEAD-domain-containing protein [Mortierella elongata AG-77]

MAADQQPMAHENDSWKKQLNLPTKDERPQTEDVTATKGNEFEDYFLKRELLMGIFEAGFERPSPIQEESIPIALAGRDIL

ARAKNGTGKTAAFVIPSLEKINLKKNNVQALLLVPTRELALQTAQVCKTLGKHMGVQVMVTTGGTTLKDDIMRLGETVHI

IVGTPGRILDLAGKGVANFSECTTFVMDEADKLLSPEFTPIVEQLLTYFPKERQIMLYSATFPMIVKTFKDKHMIKPYEI

NLMDELTLRGVTQYYAFVEERQKVHCLNTLFSKLQINQSIIFCNSTNRVELLAKKITELGYSCFYSHAKMLQSHRNRVFH

DFRNGVCRNLVCSDLLTRGIDIQAVNVVINFDFPKNAETYLHRIGRSGRFGHLGLAINLITYEDRFNLYKIEQELGTEIQ

AIPPVIDKRLYVAPSALDQPENEQQQQQQQPQKRLHNNTSISSNRWPTICTNSNNK

>ORX99239.1 DEAD-domain-containing protein [Basidiobolus meristosporus CBS 931.73]

MASQNNFNGQYPSHDNDNWKKTLALPPKDSRPQTEDVTATKGNEFEDYFLKRELLMGIFEAGFERPSPIQEEAIPIALTG

RDVLARAKNGTGKTAAFVIPSLEKVNTKLSKIQALLLVPTRELALQTSQVCKTLGKHMGVQVMVTTGGTTLKDDIMRLQE

NVHVLVGTPGRILDLAGKGVADFSECPTFVMDEADKLLSPEFSPVVEQLLTFFPKDRQIMLFSATFPIIVKHFKDRHMTK

PYEINLMDELTLRGVTQYYAFVEERQKVHCLNTLFSKLQINQSIIFCNSTNRVELLAKKITQLGYSCFYSHAKMLQSHRN

RVFHDFRNGVCRNLVCSDLLTRGIDIQAVNVVINFDFPKNAETYLHRIGRSGRFGHLGLAINLITYEDRFNLYKIEQELG

TEIQPIPPVIDKRLYVAPSALEDASEAQQQQQQQQQSANKQPQQAPPVQPQFYQQQQQQQQQQQFQRQQLPPHAYQQHPP

QYPQFQQQPPPMSQQPYAPQPPAQFQGQQGRMNNMGYSQQNQQPPHDGRSGQGMNGGGLRAFS

>TPX39825.1 hypothetical protein SeLEV6574_g06965 [Synchytrium endobioticum]

MAAVENGANALASQLANTHISSHDVPGWKKNLKLPTADTRPQTEDVTATKGNEFEDYFLKRELLMGIFEAGFERPSPIQE

ESIPIALTGRDILARAKNGTGKTAAFVIPVLEKVNVHKKVIQALILVPTRELALQTSQVIKTIGKHLNVQVMVTTGGTTL

KDDILRLGQTVHVLVATPGRVLDLAGKGVCDLKEANMLVMDEADKLLSPEFQPIIEQVISFFPDNRQILLFSATFPLIVK

SFNDKFLNKPYEINLMEELTLKGITQYYAYVEERQKVHCLNTLFSKLQINQSIIFCNSTSRVELLAKKITELGYSCYYIH

AKMLQSHRNRVFHDFRNGKTRHLVCSDLLTRGIDIQAVNVVINFDFPKNAETYLHRIGRSGRFGHFGLAINLITYDDRFN

LYRIEQELGTEIAPIPPTIDPALYVAP

>KAF5103922.1 hypothetical protein DV451_001150 [Geotrichum candidum]

MSGESDWKSTLNIPAKDSRPQTEDVTATKGNGFEDFYLKRELLMGIFEAGFEKPSPIQEESIPLALAGRDILARAKNGTG

KTASFVIPALQQVNPKLNKIQALILVPTRELALQTSQVCKTLGKHLNLKIMVTTGGTTLKDDIVRLNDTVHILVATPGRI

LDLASKGVADLSECPMFIMDEADKLLSPEFTPVIEQLLEYFPSDRQSLLFSATFPLVVKSFMDKHLKRPYEINLMDELTL

KGITQYYAFVEEKQKLHCLNTLFSKLQINQSIIFCNSTNRVELLARKITELGYSCFYSHAKMVQANRNRVFHEFRNGGCR

NLVCSDLLTRGIDIQAVNVVINFDFPKNAETYLHRIGRSGRFGHLGLAINLISWNDRFNLYKIEQELGTEIKPIPAHIDA

SLYVADAVESIPRPFPMQELPKANTHSVQPRYHHHNGNQGGGGRFSNNGNGSEGYPNNNQSGGYQNNNYNQSQHPALPPP

QQQQQQPYYDNGSGYPQHPNQFNNHQQPNGYPPQPPPQQGYSNGQYQMH

>ORZ19723.1 ATP-dependent RNA helicase DHH1 [Absidia repens]

MAAPYNNQQLSHENDNWKKAMSLPPKDTRPQTEDVTATKGNEFEDYFLKRELLMGIFEAGFERPSPIQEEAIPIALTGRD

ILARAKNGTGKTAAFVIPTLEKINNKKSKIQALLLVPTRELALQTSQVCKTLGKHLGLQIMVTTGGTTLRDDIMRLSETV

HVVVGTPGRILDLASKGVADFSEASTFVMDEADKLLSPEFTPIIDQLISFFPKERQIMLFSATFPIIVKHFKDRYLVKPY

EINLMDELTLRGVTQYYAFVEEKQKVHCLNTLFSKLQINQSIIFCNSTNRVELLAKKITELGYSCFYSHAKMLQSHRNRV

FHDFRNGVCRNLVCSDLLTRGIDIQAVNVVINFDFPKNAETYLHRIGRSGRFGHLGLAINLITYEDRFNLYKIERELGTE

IQPIPPTIDKQLYVAPNALDDSQVQRPDREAAIATRQQQQQRESQPQNQNQQQQPQVQSNYYQQPQQQYQQQRQQHYYQN

GRGRGGYHGRGGYNGRGRPRQQYQQGQQPHQQMPTN

>XP_018297169.1 hypothetical protein PHYBLDRAFT_184658 [Phycomyces blakesleeanus NRRL 1555(-)]

MSMPSSSNQQLSHENDSWKKTMVLPPKDTRPQTEDVTATKGNEFEDYFLKRELLMGIFEAGFERPSPIQEEAIPIALTGR

DILARAKNGTGKTAAFVIPTLEKINNKKSKIQALLLVPTRELALQTSQVCKNLGKHLGVQVMVTTGGTTLKDDIMRLSES

VHIVVGTPGRILDLASKGVADFSEASTFVMDEADKLLSPEFTPIIDQLISFFPKDRQIMLFSATFPIIVKHFKEKYLVKP

YEINLMDELTLRGVTQYYAYVEEKQKVHCLNTLFSKLQINQSIIFCNSTNRVELLAKKITELGYSCFYSHAKMLQSHRNR

VFHDFRNGVCRNLVCSDLLTRGIDIQAVNVVINFDFPKNAETYLHRIGRSGRFGHLGLAINLITYEDRFNLYKIERELGT

EIQPIPPVIDKRLYVAPSALEDSEGQADNYHFSNSNRGGRFRTQQPNGGGNSRSRIPDPLP

>ESK90139.1 atp-dependent rna helicase dhh1 [Moniliophthora roreri MCA 2997]

MSQIATSSTATSPTGVRDNSWKTGLRPPPKDMRPQTEDVTKTKGVEFEDMSLRRELLMGIFEAGFERPSPIQEEAIPIAL

TKRDVLARAKNGTGKTAAFVIPSLQQIDPTKSRIQALLLVPTRELALQTSQVCKILGKHMGIQVMVTTGGTTLKDDIMRL

SETVHVLVGTPGRILDLAGKGVADLSECPVFVMDEADKLLSPEFAPVMEQLLAYLPENRQVMLFSATFPMIVKDFKDKHM

RQPYEINLMDELTLRGVTQYYAYVEERQKVHCLNTLFSKLQINQSIIFCNSTNRVELLAKKVTELGYSCFYSHAKMLQSH

RNRVFHDFRNGVSRNLVCSDLLTRGIDIQAVNVVINFDFPKNSETYLHRIGRSGRFGHLGLAINLVTYEDRFNLYRIEQE

LGTEIQPIPQIIDKGLYVAPSATEEEKAVQKQVQQQQQQQQQQQQRAQQEQVVYQSNGAPQSRGTPQGAYRGGVPAGAR

>XP_021878255.1 P-loop containing nucleoside triphosphate hydrolase protein [Lobosporangium transversale]

MATNQQQHMAHENDSWKKQLSLPAKDDRPQTEDVTATKGNEFEDYFLKRELLMGIFEAGFERPSPIQEESIPIALAGRDI

LARAKNGTGKTAAFVIPSLEKINLKKNKVQALLLVPTRELALQTAQVCKTLGKHMGVQVMVTTGGTTLKDDIMRLGETVH

IIVGTPGRILDLAGKGVANFSECTTFVMDEADKLLSPEFTPIVEQLLTYFPKDRQIMLYSATFPMIVKTFKDKHMVKPYE

INLMDELTLRGVTQYYAFVEERQKVHCLNTLFSKLQINQSIIFCNSTNRVELLAKKITELGYSCFYSHAKMLQSHRNRVF

HDFRNGVCRNLVCSDLLTRGIDIQAVNVVINFDFPKNAETYLHRIGRSGRFGHLGLAINLITYDDRFNLYKIEQELGTEI

QAIPPVIDKRLYVAPSALDAVEHEDQQKRAQHQQQHQQQHQQQQQHQQQHQQYHQQHQQQHHQMAHHQYQQQQFQQRPQQ

QQRR

>TRM63647.1 P-loop containing nucleoside triphosphate hydrolase protein [Auriculariopsis ampla]

MSQATSSSATPTNNDWKAGLRAPPKDMRPQTEDVTATKGTEFEDMYLRRELLMGIFEAGFEKPSPIQEEAIPKALQRRDV

LARAKNGTGKTAAFVIPTLQQVDPTENKIQALLLVPTRELALQTSQVCKTLGKHMGIQVMVTTGGTTLKDDILRLSETVH

VLVGTPGRILDLAGKNVADLSHCPVFVMDEADKLLSPEFAPVMEQLLSYLPADRQVMLFSATFPMIVKDFKEKHMKTPYE

INLMEELTLRGVTQYYAYVEERQKVHCLNTLFSKLQINQSIIFCNSTNRVELLAKKITELGYSCFYSHAKMLQSHRNRVF

HDFRNGQCRNLVCSDLLTRGIDIQAVNVVINFDFPKNSETYLHRIGRSGRFGHLGLAINLVTYEDRFNLYKIEQELGTEI

SPIPPAIDRGLYVAPSAGGDDQQQKRAQAGANGAQRNGAPQQQRAPQRAPIPAGSGAAR

>TPX57116.1 hypothetical protein PhCBS80983_g04070 [Powellomyces hirtus]

MSANGLAMQLQNTHISSNDHDWKKTLALPTKDTRPQTEDVTATKGNDFEDYFLKRELLMGIFEAGFEKPSPIQEESIPIA

LAGRDILARAKNGTGKTAAFIIPALEKVNLTKSRIQTLILVPTRELALQTSQVCRNLGKHMGVQVMVTTGGTTLKDDILR

LGQTVHILVATPGRVLDLAGKGIADLSECTMLIMDEADKLLSPEFQPIIEQLIAFCKPSRQILLYSATFPMIVKSFKDKY

LNKPYEINLMDELTLRGVTQYYAYVEERQKVHCLNTLFSKLQINQSIIFCNSTSRVELLAKKITELGYSCFYIHAKMLQS

HRNRVFHDFRNGKTRHLVCSDLLTRGIDIQAVNVVINFDFPKNAETYLHRIGRSGRFGHLGLAINLITYEDRFNLYRIEQ

ELGTEIAPIPPVIDKSLYVAP

>EPB84122.1 ATP-dependent RNA helicase DHH1 [Mucor circinelloides 1006PhL]

MASQQQLSHENDNWKKSLALPQKDMRPQTEDVTATKGNEFEDYFLKRELLMGIFEAGFERPSPIQEEAIPIALTGRDILA

RAKNGTGKTAAFVIPTLEKINNKKAKIQALLLVPTRELALQTAQVCKNLGKHLNIQVMVTTGGTTLKDDIMRLSEPVHVV

VGTPGRILDLASKNVADFSEASTFVMDEADKLLSPEFTPIIDQLISHFPKDRQIMLFSATFPMIVKSFKEKYLVKPYEIN

LMDELTLRGVTQYYAYVEEKQKVHCLNTLFSKLQINQSIIFCNSTNRVELLAKKITELGYSCFYSHAKMLQSHRNRVFHD

FRNGVCRNLVCSDLLTRGIDIQAVNVVINFDFPKNAETYLHRIGRSGRFGHLGLAINLITYEDRFNLYKIERELGTEIQP

IPPVIDKNLYVAPNALEDAQVQQPNRQLAIATRQQQQQRDQTQYNNNNSPNNYQQQQQQQQQQQNHHQNGYQQQYRGNYN

NGSRGGGGGGRRQQHQNQQQSQQQPQRGQSSRS

>XP_023468699.1 DEAD-domain-containing protein [Rhizopus microsporus ATCC 52813]

MMNNSMTTPQQLSNHDNNEWKQNLMLPKKDTRPQTEDVTATKGNEFEDYFLKRELLMGIFEAGFERPSPIQEEAIPIALA

GRDVLARAKNGTGKTAAFVIPTLEKINNKISKIQALLLVPTRELALQTAQVCKTLGKHLNIQVMVTTGGTTLKDDILRLG

ETVHIVVGTPGRILDLASKGVADFSEATTFVMDEADKLLSPEFSPIIDQLIAFFPSNRQIMLFSATFPMIVKNFKDKHLT

KPYEINLMDELTLRGVTQYYAFVEEKQKVHCLNTLFSKLQINQSIIFCNSTNRVELLAKKITELGYSCFYSHAKMLQSHR

NRVFHDFRNGVCRNLVCSDLLTRGIDIQAVNVVINFDFPKNAETYLHRIGRSGRFGHLGLAINLITYEDRFNLYKIEREL

GTEIQPIPPVIDKTLYVAPNALDNAEVQQPNRQLAMATRQQQQQRVSQPEQQQQQQQQQFQQFQQFQQFQQLQQLQQPQQ

QQQHYQRRNYHQSSRGGYNHYRGGRQHMGSRGH

>KIJ61858.1 hypothetical protein HYDPIDRAFT_137104 [Hydnomerulius pinastri MD-312]

MSQSARPSSSNSPANHNDSAWKSGLRPPPKDLRPQTEDVTATKGTEFEDMFLRRELLMGIFEAGFEKPSPIQEEAIPIAL

TKRDILARAKNGTGKTAAFVIPSLQQIDVTKNKIQALLLVPTRELALQTSQVCKILGKHMGIQVMVTTGGTTLKDDIMRL

SEVVHVLVGTPGRILDLAGKNVADLSECPVFVMDEADKLLSPEFTPVMEQLLSFLPAERQVMLFSATFPLIVKDFKDKHM

NTPYEINLMEELTLRGVTQYYAYVEERQKVHCLNTLFSKLQINQSIIFCNSTNRVELLAKKVTELGYSCFYSHAKMLQSH

RNRVFHDFRNGVCRNLVCSDLLTRGIDIQAVNVVINFDFPKNSETYLHRIGRSGRFGHLGLAINLVTYEDRFNLYRIEQE

LGTEIQPIPQTIDKGLYVAPSATEEHAPQKPQQQPQQQQQRQPGQQGQATHNGRQNGGPVPPQGQPRGNYANQAAYRGAP

TAR

>OCH86771.1 DEAD-domain-containing protein [Obba rivulosa]

MAQPQQTRPSSSTSGSQSDAWKAGLRPPPKDSRPQTEDVTATRGTEFEDMFLRRELLMGIFEAGFEKPSPIQEEAIPIAL

AKRDVLARAKNGTGKTAAFVIPTLQQVDVTRNKIQALLLVPTRELALQTSQVCKILGKHMGVQVMVTTGGTTLKDDILRL

SETVHVLVGTPGRILDLAGKNVADLSECPVFVMDEADKLLSPEFAPVMEQLLSYLPKERQVMLFSATFPMIVKDFKDKHM

RSPHEINLMDELTLRGVTQYYAYVEERQKVHCLNTLFSKLQINQSIIFCNSTNRVELLAKKVTELGYSCFYSHAKMLQSH

RNRVFHDFRNGVCRNLVCSDLLTRGIDIQAVNVVINFDFPKNSETYLHRIGRSGRFGHLGLAINLVTYEDRFNLYKIEQE

LGTEIQPIPQTIDKGLYVAPSSQDEPQEQKPAPARNAQNQQQQQAQAQPQQRPPVQAQAASAQVVYQSGPQAPRQNGTAV

PTQAQPQRAAYQAAYRNGGVPVAR

>XP_016273562.1 ATP-dependent rna helicase dhh1 [Rhodotorula toruloides NP11]

MSAPQASSSSAPQQQPEDWKKQLNLPTKDTRPQTEDVTATKGNEFEDYFLKRELLMGIFEAGFERPSPIQEEAIPIALTG

RDILARAKNGTGKTAAFIIPVLERVNPKIPKIQALLLVPTRELALQTSQVCKTLGKHTGAQVMVTTGGTTLRDDILRLGE

TVHILVGTPGRILDLAGKGIADLSQCPMFVMDEADKLLSPEFTPVIEQLLSFMPKERQVMLFSATFPLIVKDFKDKWMRK

PYEINLMDELTLRGVTQYYAFLEERQKVHCLNTLFSKLQINQSIIFCNSTNRVELLAKKITELGYSCFYSHARMLQQHRN

RVFHDFRNGVCRNLVCSDLLTRGIDIQAVNVVINFDFPKNAETYLHRIGRSGRYGHLGLAINLITYDDRFNLYRIEQELG

TEIQPIPPVIDRSLYVAPGTEEEVKGAQEAAKAGQKVAVPAGVGGHGMQAPQVRGKDRSLPPLPLSSSSSSNPRHSSSTS

SRLTRCLSSSSSLSSSTTARMRLGQARTARLSLPTSSSSTASSSSTASLSSTTSSSSRHSCSSRLRRWRRASSDERSATT

RERR

>GAN04011.1 eukaryotic translation initiation factor 4A-like protein [Mucor ambiguus]

MASQQQLSHENDNWKKSLALPQKDMRPQTEDVTATKGNEFEDYFLKRELLMGIFEAGFERPSPIQEEAIPIALTGRDILA

RAKNGTGKTAAFVIPTLEKINNKKAKIQALLLVPTRELALQTAQVCKNLGKHLNIQVMVTTGGTTLKDDIMRLSEPVHVV

VGTPGRILDLASKNVADFSEASTFVMDEADKLLSPEFTPIIDQLISHFPKDRQIMLFSATFPMIVKSFKEKYLVKPYEIN

LMDELTLRGVTQYYAYVEEKQKVHCLNTLFSKLQINQSIIFCNSTNRVELLAKKITELGYSCFYSHAKMLQSHRNRVFHD

FRNGVCRNLVCSDLLTRGIDIQAVNVVINFDFPKNAETYLHRIGRSGRFGHLGLAINLITYEDRFNLYKIERELGTEIQP

IPPVIDKNLYVAPNALEDAQVQQPNRQLAIATRQQQQQRDQTQYNNNNSHNNYQQQQQHQQQQQQQQPQQQQQQQVYHQN

GHQQQQYRGNYNNGSRGGGGGGGRRQQHQNQQQRGQSSRS

>ORE18052.1 ATP-dependent RNA helicase DHH1 [Rhizopus microsporus]

MMNNSMTTPQQLSNHDNNEWKQNLMLPKKDTRPQTEDVTATKGNEFEDYFLKRELLMGIFEAGFERPSPIQEEAIPIALA

GRDVLARAKNGTGKTAAFVIPTLEKINNKISKIQALLLVPTRELALQTAQVCKTLGKHLNIQVMVTTGGTTLKDDILRLG

ETVHIVVGTPGRILDLASKGVADFSEATTFVMDEADKLLSPEFSPIIDQLIAFFPSNRQIMLFSATFPMIVKNFKDKHLT

KPYEINLMDELTLRGVTQYYAFVEEKQKVHCLNTLFSKLQINQSIIFCNSTNRVELLAKKITELGYSCFYSHAKMLQSHR

NRVFHDFRNGVCRNLVCSDLLTRGIDIQAVNVVINFDFPKNAETYLHRIGRSGRFGHLGLAINLITYEDRFNLYKIEREL

GTEIQPIPPVIDKTLYVAPNALDNAEVQQPNRQLAMATRQQQQQRVSQPEQQQQQQQQQQQQQQQQQQQQQQQQFQQFQQ

NYHQSSRGGYNHYRGGRQHMGSRGH

>ORE09160.1 ATP-dependent RNA helicase DHH1 [Rhizopus microsporus var. microsporus]

MMNNSMTTPQQLSNHDNNEWKQNLMLPKKDTRPQTEDVTATKGNEFEDYFLKRELLMGIFEAGFERPSPIQEEAIPIALA

GRDVLARAKNGTGKTAAFVIPTLEKINNKISKIQALLLVPTRELALQTAQVCKTLGKHLNIQVMVTTGGTTLKDDILRLG

ETVHIVVGTPGRILDLASKGVADFSEATTFVMDEADKLLSPEFSPIIDQLIAFFPSNRQIMLFSATFPMIVKNFKDKHLT

KPYEINLMDELTLRGVTQYYAFVEEKQKVHCLNTLFSKLQINQSIIFCNSTNRVELLAKKITELGYSCFYSHAKMLQSHR

NRVFHDFRNGVCRNLVCSDLLTRGIDIQAVNVVINFDFPKNAETYLHRIGRSGRFGHLGLAINLITYEDRFNLYKIEREL

GTEIQPIPPVIDKTLYVAPNALDNAEVQQPNRQLAMATRQQQQQRVSQPEQQQQQQQQQQQQQQQQQQFQQFQQNYHQSS

RGGYNHYRGGRQHMGSRGH

>RKP38145.1 hypothetical protein BJ085DRAFT_16548 [Dimargaris cristalligena]

MASSAYANEPESWKNKLSLPPKDTRPQTEDVTATKGNEFEDYYLKRELLMGIFEAGFERPSPIQEESIPIALAGRDILAR

AKNGTGKTAAFTIPVLEKVNTQKPKIQALILVPTRELALQTSQVCKNLGKHMAVEVMVSTGGTVLKEDIMRLKETVHIVV

GTPGRILDLAGKGICDLSECTTFVMDEADKLLSPEFTPVIENLLSYFSSKRQIMLYSATFPMVVKNFKDKHMVKPYEINL

MDELTLRGVTQYYAFVEEKQKVHCLNTLFSKLQINQSIIFCNSTNRVELLAKKITELGYSCFYSHARMRQQDRNRVFHDF

RQGVCRNLVCSDLLTRGIDIQAVNVVINFDFPKNAETYLHRIGRSGRFGHLGLAINLITYDDRFNLYKIEQELGTEIKPI

PPVIDKRLYVAPSAIDDSQQQHQQSRKTAAEVAAASQGTIMSGQPQQHHSQQGPHPNSSYNNNNNGNQSRGYQSGYGGSR

YQNGPQQHPGSSNGGRNYSNNGGQYRQNNGPRPDHNRQGGSVHSNAPRN

>CEG82997.1 Putative ATP-dependent RNA helicase DHH1 [Rhizopus microsporus]

MMNNSMTTPQQSSNHDNNEWKQNLMLPKKDTRPQTEDVTATKGNEFEDYFLKRELLMGIFEAGFERPSPIQEEAIPIALA

GRDVLARAKNGTGKTAAFVIPTLEKINNKISKIQALLLVPTRELALQTAQVCKTLGKHLNIQVMVTTGGTTLKDDILRLG

ETVHIVVGTPGRILDLASKGVADFSEATTFVMDEADKLLSPEFSPIIDQLIAFFPSNRQIMLFSATFPMIVKNFKDKHLT

KPYEINLMDELTLRGVTQYYAFVEEKQKVHCLNTLFSKLQINQSIIFCNSTNRVELLAKKITELGYSCFYSHAKMLQSHR

NRVFHDFRNGVCRNLVCSDLLTRGIDIQAVNVVINFDFPKNAETYLHRIGRSGRFGHLGLAINLITYEDRFNLYKIEREL

GTEIQPIPPVIDKTLYVAPNALDNAEVQQPNRQLAMATRQQQQQRVSQPEQQQQQQQQQQQQQQQQQQFQQFQQFQQFQQ

LQQLQQPQQQQQQHYQRRNYHQSSRGGYNHYRGGRQHMGSRGH

>KAF1800340.1 hypothetical protein FB192DRAFT_1141200 [Mucor lusitanicus]

MASQQQLSHENDNWKKSLALPQKDMRPQTEDVTATKGNEFEDYFLKRELLMGIFEAGFERPSPIQEEAIPIALTGRDILA

RAKNGTGKTAAFVIPTLEKINNKKAKIQALLLVPTRELALQTAQVCKNLGKHLNIQVMVTTGGTTLKDDIMRLSEPVHVV

VGTPGRILDLASKNVADFSEASTFVMDEADKLLSPEFTPIIDQLISHFPKDRQIMLFSATFPMIVKSFKEKYLVKPYEIN

LMDELTLRGVTQYYAYVEEKQKVHCLNTLFSKLQINQSIIFCNSTNRVELLAKKITELGYSCFYSHAKMLQSHRNRVFHD

FRNGVCRNLVCSDLLTRGIDIQAVNVVINFDFPKNAETYLHRIGRSGRFGHLGLAINLITYEDRFNLYKIERELGTEIQP

IPPVIDKNLYVAPNALEDAQVQQPNRQLAIATRQQQQQRDQTQYNNNNSHNNYQQHQQPQQQPQQQQQQQQVYHQNGHQQ

QQQYRGNYNNGSRGGGGGGRRQQHQNQQQQQQRGQSSRS

>RCH87294.1 DExD/H-box ATP-dependent RNA helicase dhh1 [Rhizopus azygosporus]

MMNNSMTTPQQLSNHDNNEWKQNLMLPKKDTRPQTEDVTATKGNEFEDYFLKRELLMGIFEAGFERPSPIQEEAIPIALA

GRDVLARAKNGTGKTAAFVIPTLEKINNKISKIQALLLVPTRELALQTAQVCKTLGKHLNIQVMVTTGGTTLKDDILRLG

ETVHIVVGTPGRILDLASKGVADFSEATTFVMDEADKLLSPEFSPIIDQLIAFFPSNRQIMLFSATFPMIVKNFKDKHLT

KPYEINLMDELTLRGVTQYYAFVEEKQKVHCLNTLFSKLQINQSIIFCNSTNRVELLAKKITELGYSCFYSHAKMLQSHR

NRVFHDFRNGVCRNLVCSDLLTRGIDIQAVNVVINFDFPKNAETYLHRIGRSGRFGHLGLAINLITYEDRFNLYKIEREL

GTEIQPIPPVIDKTLYVAPNALDNAEVQQPNRQLAMATRQQQQQRVSQPEQQQQQQQQQQQQQQQQQQQQQQQQFQQFQQ

FQQFQQLQQLQQPQQQQQQQHYQRRNYHQSSRGGYNHYRGGRQHMGSRGH

>CEI93304.1 Putative ATP-dependent RNA helicase DHH1 [Rhizopus microsporus]

MMNNSMTTPQQLSNHDNNEWKQNLMLPKKDTRPQTEDVTATKGNEFEDYFLKRELLMGIFEAGFERPSPIQEEAIPIALA

GRDVLARAKNGTGKTAAFVIPTLEKINNKISKIQALLLVPTRELALQTAQVCKTLGKHLNIQVMVTTGGTTLKDDILRLG

ETVHIVVGTPGRILDLASKGVADFSEATTFVMDEADKLLSPEFSPIIDQLIAFFPSNRQIMLFSATFPMIVKNFKDKHLT

KPYEINLMDELTLRGVTQYYAFVEEKQKVHCLNTLFSKLQINQSIIFCNSTNRVELLAKKITELGYSCFYSHAKMLQSHR

NRVFHDFRNGVCRNLVCSDLLTRGIDIQAVNVVINFDFPKNAETYLHRIGRSGRFGHLGLAINLITYEDRFNLYKIEREL

GTEIQPIPPVIDKTLYVAPNALDNAEVQQPNRQLAMATRQQQQQRVSQPEQQQQQQQQQQQQQQQQQQQQQQFQQFQQFQ

QFQQLQQLQQPQQQQQQQHYQRRNYHQSSRGGYNHYRGGRQHMGSRGH

>KNE73009.1 ATP-dependent RNA helicase dhh1 [Allomyces macrogynus ATCC 38327]

MSDQYDADPAAQHEWKQQLHLPPKDARPQTDDVLATKGNEFEDYYLKRELLMGIFEAGFERPSPIQEEAIPIALAGRDIL

ARAKNGTGKTAAFVIPTLEKVNPQKHKIQALLLVPTRELALQTSQVCKTLGKHLGLQVMVTTGGTGLKDDIIRLQDTVHI

LVGTPGRLLDLAGKGVAQLDECTTFVMDEADKLLSPEFQPVIEQLLGYLPEDRQIMLFSATFPVLVKDFRDKWLNAPYEI

NLMDDLTLKGVTQYYAFVEERQKVHCLNTLFSKLQINQSIIFCNSTNRVELLAKKITDLGYSCFYIHARMPQAHRNRVFH

DFRQGVCRNLVCSDLLTRGIDIQAVNVVINFDFPKNAETYLHRIGRSGRFGHLGLAINLITYEDRFNLYRIETELGTEIQ

PIPPTVDKHLYVAPALQDAAPQQPQAATELPQQPQADAHHHAHHHHHHRNGGGDDIVSLEAILGPFLCNLPMVLESLKVT

ASIPDNDNEDASILIGRRDSYHQLPSRHAHLVLIPSTIKASEVIMDLFDSFNELCDGDMVDSCTDHRLALALAHFSALAT

LTLRRMNLNARNLPALARIALSRLVHLTHLDLSSNSNFDDMAISSLAPALARLVQLHTLDLSWTGVPDDGIAMLARAALS

RLVALRVLNLRRLLHFTDAECIMDGLLESVQLVDMRGCMVGNKLSLLDAPVGRPGRTVRTGSGMRKGPEWQIERVSAVDE

SGQHVVHQRVTSKRIEPL

>CEG71520.1 Putative ATP-dependent RNA helicase DHH1 [Rhizopus microsporus]

MMNNSMTAPQQLSNHDNNEWKQNLMLPKKDTRPQTEDVTATKGNEFEDYFLKRELLMGIFEAGFERPSPIQEEAIPIALA

GRDVLARAKNGTGKTAAFVIPTLEKINNKISKIQALLLVPTRELALQTAQVCKTLGKHLNIQVMVTTGGTTLKDDILRLG

ETVHIVVGTPGRILDLASKGVADFSEATTFVMDEADKLLSPEFSPIIDQLIAFFPSNRQIMLFSATFPMIVKNFKDKHLT

KPYEINLMDELTLRGVTQYYAFVEEKQKVHCLNTLFSKLQINQSIIFCNSTNRVELLAKKITELGYSCFYSHAKMLQSHR

NRVFHDFRNGVCRNLVCSDLLTRGIDIQAVNVVINFDFPKNAETYLHRIGRSGRFGHLGLAINLITYEDRFNLYKIEREL

GTEIQPIPPVIDKTLYVAPNALDNAEVQQPNRQLAMATRQQQQQRVSQPEQQQQQQQQQQQQQQQQQQQQQQFQQFQQFQ

QFQQLQQLQQPQQQQQQHYQRRNYHQSSRGGYNHYRGGRQHMGSRGH

>CEJ02025.1 Putative ATP-dependent RNA helicase DHH1 [Rhizopus microsporus]

MMNNSMTAPQQLSNHDNNEWKQNLMLPKKDTRPQTEDVTATKGNEFEDYFLKRELLMGIFEAGFERPSPIQEEAIPIALA

GRDVLARAKNGTGKTAAFVIPTLEKINNKISKIQALLLVPTRELALQTAQVCKTLGKHLNIQVMVTTGGTTLKDDILRLG

ETVHIVVGTPGRILDLASKGVADFSEATTFVMDEADKLLSPEFSPIIDQLIAFFPSNRQIMLFSATFPMIVKNFKDKHLT

KPYEINLMDELTLRGVTQYYAFVEEKQKVHCLNTLFSKLQINQSIIFCNSTNRVELLAKKITELGYSCFYSHAKMLQSHR

NRVFHDFRNGVCRNLVCSDLLTRGIDIQAVNVVINFDFPKNAETYLHRIGRSGRFGHLGLAINLITYEDRFNLYKIEREL

GTEIQPIPPVIDKTLYVAPNALDNAEVQQPNRQLAMATRQQQQQRVSQPEQQQQQQQQQQQQQQQQFQQFQQFQQFQQLQ

QLQQPQQQQQQHYQRRNYHQSSRGGYNHYRGGRQHMGSRGH

>TIC06416.1 alpha/beta-hydrolase [Wallemia mellicola]

MMDPLSLSVNLSPVVLRTLVRHLIKKRNKEDKREVDDPNDEYAFDQTFKLTRAFLDIAMNHPVEDLQNLGSTYVPPPFWV

HNVPVTVPMSICNDAAEHLIKAFGGEEPMRAAVGGTRWWQVRPTAGLPAEWICMKSDRKLATRHNKLKVREQPKDPLTPQ

FFQDIEGLPRTILYVHGGAYYWGSINTHRYLIWRMARKMRGKAFAVSYRLSPQYGFPCALHDVLSAYLYLLYPPEGAPHK

AVDPANLIFAGLPLPAGSIHISPWCDLSHSFPSVLQNYETDVIPKYGFVHKPSTLWPPPSEDLLQRTREHIKSSTLRSTS

PLKGLGTAASHKAEKGRRKEETDAAINAEHGRERMDIPREVPTVDINGKTVELKGQIQMYATNEQVGHPYVSPVLAHLGG

LCPLFIMASDGELLRDEAIYVAHKAARPDEFEYKEDAKRRLPSMNGIEGRYGPTKVHLQAYDKMCHDLPLFSMVTPAKYA

YRAMAAFCFHVTKDKAAQPSPAIKPAHNGMTMSDFELDNPLEMPPTPSSGLASPTKLPEAKSPRRKFSLLGLRNEGAGSS

DMSSPVTSNGSQDNAPSSDAERPIKAGPGTAGHIAVYDEATAFKDGNMIRERVSMGGVTRPMEPPEELEYLRMSVNEVGV

FKPDAVKRYIEGMESWNTKFKHAAKNVERQREKSLRESHIHAEKMMKENDDIFDIPEVTWRWQWALENEKPPPASIVARR

DTQEARRLVELVDKVDNKHASKTVNLWTLIVHLLSPQKSKYGVNDEDEVGPWTRDIEELKKKYQTDEKGEVIPEEVSADE

ENNFNNKKEAWMKSQEYRQESEWKNQIRAPPKDLRPQTEDVTNTKGGEWEDFGLRRELLMGIFEAGFEKPSPIQEEAIPS

AIEGRDILARAKNGTGKTASFVIPSLEKINVQKPKIQALLLVPTRELALQTSQVCKTLGKHLGIQVMVTTGGTTLRDDIM

RLADPVHILVGTPGRILDLASKGVANLEECPTFVMDEADKLLSPEFTPVMEQLLGHLPSSRQVMLFSATFPLIVKDFKEK

HMRNPHEINLMDELTLRGITQYYAFVEERQKVHCLNTLFSKLQINQSIIFCNSTNRVELLAKKVTELGYSCFFSHAKMLQ

SHRNRVFHDFRSGVCRNLVCSDLLTRGIDIQAVNVVINFDFPKNSETYLHRIGRSGRYGHLGLAINLITYEDRFSLYKIE

QELGTEIQPIPSQIDRSLYVAPNASNEDGQIQQSQQQNQQQKGLPTLPPTQQVMNAYAQPPPQPTFNQNQQQNNHHQYQQ

RSHRGGYNNNGYYGNYRGRGGPPRGGVPRA

>XP_031023680.1 uncharacterized protein SmJEL517_g04482 [Synchytrium microbalum]

MAAVENGAGQLANQLANTHISSHDGSDWKKSLKLPTPDTRPQTEDVTNTKGNEFEDYFLKRELLMGIFEAGFERPSPIQE

ESIPIALTGRDILARAKNGTGKTAAFVIPVLEKVNVQKKAIQALILVPTRELALQTSQVIKTIGKHMNVQVMVTTGGTTL

KDDILRLGQTVHILVATPGRVLDLAGKGICDLKEASMLVMDEADKLLSPEFQPIIEQIISFHPEKRQILLFSATFPLIVK

SFKDKFLEKPYEINLMEELTLKGITQFYAYVEERQKVHCLNTLFSKLQINQSIIFCNSTSRVELLAKKITELGYSCYYIH

AKMLQSHRNRVFHDFRNGKTRHLVCSDLLTRGIDIQAVNVVINFDFPKNAETYLHRIGRSGRFGHFGLAINLITYDDRFN

LYRIEQELGTEIAPIPPTIDPALYVAP

>KAF5345927.1 hypothetical protein D9758_011459 [Tetrapyrgos nigripes]

MSQAPTPSSSSSDNWKAGLRPPPKDYRPQTEDVTATKGVEFEDMRLRRELLMGIFEAGFENPSPIQEEAIPIALTKRDVL

ARAKNGTGKTAAFVIPSLQQVDPLKNKIQALLLVPTRELALQTSQVCKILGKHMGIQIMVTTGGTTLKDDIMRLSETVHV

LVGTPGRILDLAGKNVADLSECPVFVMDEADKLLSPEFAPVMEQLLAYLPENRQVMLFSATFPMIVKDFKDKHMRSPYEI

NLMDELTLRGVTQYYAYVEERQKVHCLNTLFSKLQINQSIIFCNSTNRVELLAKKVTELGYSCFYSHAKMLQSHRNRVFH

DFRNGVCRNLVCSDLLTRGIDIQAVNVVINFDFPKNSETYLHRIGRSGRFGHLGLAINLVTYEDRFNLYRIEQELGTEIQ

PIPQIIDKGLYVAPSAGVGPTEEEKKLQQQQQQVQARQQQLLQQQQKLLAQQQAQAAQQMQGQGQVVYQSNGQQPRSNGT

PAGYRGGVATAAR

>CDR46917.1 RHTO0S13e03356g1_1 [Rhodotorula toruloides]

MSAPQASSSSAPQQQPEDWKKQLNLPTKDTRPQTEDVTATKGNEFEDYFLKRELLMGIFEAGFERPSPIQEEAIPIALTG

RDILARAKNGTGKTAAFIIPVLERVNPKIPKIQALLLVPTRELALQTSQVCKTLGKHTGAQVMVTTGGTTLRDDILRLGE

TVHILVGTPGRILDLAGKGIADLSQCPMFVMDEADKLLSPEFTPVIEQLLSFMPKERQVMLFSATFPLIVKDFKDKWMRK

PYEINLMDELTLRGVTQYYAFLEERQKVHCLNTLFSKLQINQSIIFCNSTNRVELLAKKITELGYSCFYSHARMLQQHRN

RVFHDFRNGVCRNLVCSDLLTRGIDIQAVNVVINFDFPKNAETYLHRIGRSGRYGHLGLAINLITYDDRFNLYRIEQELG

TEIQPIPPVIDRSLYVAPGTEEEVKGAQEAAKAGQKVAVPAGVGGHGMQAPQVRQGPQPPAAAPQQQQQQQPAPQQQYQQ

PPHQMPQQQQQPQQQYYGQNAPRPGQNGAPLPPHLQQQYGQQQQYGQPQQYNQQQQQAFMQQQAQAMASRQQR

>ORZ25810.1 ATP-dependent RNA helicase DHH1 [Absidia repens]

MSVPYNNQQLSHENDNWKKTMTLPPKDTRPQTEDVTATKGNEFEDYFLKRELLMGIFEAGFERPSPIQEEAIPIALTGRD

ILARAKNGTGKTAAFVIPTLEKINNKKSKIQALLLVPTRELALQTSQVCKTLGKHLGIQIMVTTGGTTLKDDILRLSDTV

HVVVGTPGRILDLASKGVADFSEASTFVMDEADKLLSPEFTPIIDQLISFFPKSRQIMLFSATFPMIVKHFKDKYLVKPY

EINLMDELTLRGVTQYYAFVEEKQKVHCLNTLFSKLQINQSIIFCNSTNRVELLAKKITELGYSCFYSHAKMLQSHRNRV

FHDFRNGVCRNLVCSDLLTRGIDIQAVNVVINFDFPKNAETYLHRIGRSGRFGHLGLAINLITYEDRFNLYKIERELGTE

IQPIPPTIDKQLYVAPNALDEAQVQQPNREAAIATRQQQQQRESQQPQGQQSHHYQNQQGPYLQQQQQFHQQPQQHYQQQ

RQQQFYQNGRGRGGYNGRGGYNGRGRPRQSYQHQQQQQQQQQQQMPMN

>XP_018734553.1 DExD/H-box ATP-dependent RNA helicase DHH1 [Sugiyamaella lignohabitans]

MSDAWKSQLNIPEKDHRPQTEDVTATKGNGFEDFYLKRELLMGIFEAGFEKPSPIQEEAIPIALAGRDILARAKNGTGKT

AAFTIPALQQVNPKLGKIQALILVPTRELALQTSQVCRTLGKHLGINVMVTTGGTTLKDDIIRLNESVHVLVGTPGRVLD

LAGKNVVDFSECPMFIMDEADKLLSPDFTPIIEQLLAHFPPDRQILLFSATFPLLVKSFMDKHLNKPYEINLMDELTLRG

ITQYYAFVEEKQKLHCLNTLFSKLSINQSIIFCNSTNRVELLARKITELGYSCYYSHAKMIQAHRNRVFHEFRNGTCRNL

VCSDLLTRGIDIQAVNVVINFDFPKNAETYLHRIGRSGRFGHLGLAINLINWNDRFNLYQIEQELGTEIKPIPSHIDRSL

YVAQNTDAIPKPFPLKELPKSNQRSHEKYQQQTDLNFQPRPQHHNQHHHHQQQQGQQHLQQNQNQNPNQIPTQNQHPSQA

FSGQPQPPQGVPPQGYPQFPQGYPPQPGYPPQGFPQQPPVPPNGQFYAQPPPALPQ

>ORX45686.1 ATP-dependent RNA helicase DHH1 [Hesseltinella vesiculosa]

MSLQPLSHEHDDWKKTLALPPKDTRPQTEDVTATKGNEFEDYFLKRELLMGIFEAGFERPSPIQEEAIPIALTGRDILAR

AKNGTGKTAAFVIPTLEKINNKKSKVQALILVPTRELALQTSQVCKTLGKHLGIQIMVTTGGTTLKDDIMRLAETVHVVV

GTPGRILDLASKGVADFSEASTFVMDEADKLLSPEFTPIIDQLISFFPKDRQIMLFSATFPMIVKTFKDKYLVKPYEINL

MDELTLRGVTQYYAFVEEKQKVHCLNTLFSKLQINQSIIFCNSTNRVELLAKKITELGYSCFYSHAKMLQSHRNRVFHDF

RNGVCRNLVCSDLLTRGIDIQAVNVVINFDFPKNAETYLHRIGRSGRFGHLGLAINLITYEDRFNLYKIERELGTEIQPI

PPSIDRQLYVAPNALDDAQVQQPNREAAIATRQQQQQREARDQTSQQIPQHQSQQQQQQHQQHHYHYHDQSNGRGRGGYR

GRGGYRGRGGPRQQQQQQQRPRPLP

>ORY06446.1 DEAD-domain-containing protein [Basidiobolus meristosporus CBS 931.73]

MSSQNNLNGHYPSHDNDNWKKSLALPPKDNRPQTEDVTATKGNEFEDYFLKRELLMGIFEAGFERPSPIQEEAIPIALTG

RDILARAKNGTGKTAAFVIPSLEKVNIKIPKIQALLLVPTRELALQTSQVCKTLGKHMDIQVMVTTGGTTLKDDIMRLQE

TVHVLVGTPGRILDLAGKGVADFSECPTFVMDEADKLLSPEFSPVVEQLLTFFPKDRQIMLFSATFPIIVKHFKDRHMTK

PYEINLMDELTLRGVTQYYAFVEERQKVHCLNTLFSKLQINQSIIFCNSTNRVELLAKKITQLGYSCFYSHARMLQSHRN

RVFHDFRNGVCRNLVCSDLLTRGIDIQAVNVVVNFDFPKNAETYLHRIGRSGRFGHLGIAINLITYEDRFNLYKIEQELG

TEIQPIPPVIDKRLYVAPSALEDTPEAQQAQQSQQPQPQPQPTQNPVFQQPQHPFQHQQLPPHAYQQHPPQHPQFQQQLQ

YPQAPTQYQLQQGRINNNGYPQQSPQHEHDGRSGSGFNGGLRAFS

>OBZ89928.1 ATP-dependent RNA helicase DHH1 [Choanephora cucurbitarum]

MASQQQLSHENDNWKKTLNLPKKDTRPQTEDVTATKGNEFEDYFLKRELLMGIFEAGFEHPSPIQEEAIPIALTGRDILA

RAKNGTGKTAAFVIPTLEKINNKKAKIQALLLVPTRELALQTAQVCKTLGKHLNIQVMVTTGGTTLKDDIMRLGETVHVV

VGTPGRILDLASKNVADFSEANTFVMDEADKLLSPEFTPIIDQLISYFPKDRQIMLFSATFPMIVKSFKDKYLVKPYEIN

LMDELTLRGVTQYYAYVEEKQKVHCLNTLFSKLQINQSIIFCNSTNRVELLAKKITELGYSCFYSHAKMLQSHRNRVFHD

FRNGVCRNLVCSDLLTRGIDIQAVNVVINFDFPKNAETYLHRIGRSGRFGHLGLAINLITYEDRFNLYKIERELGTEIQP

IPPVIDKTLYVAPNALEDAQVQQPNRQQAIATRQQQQQREFHVQQQQQQQPNNYPQQYQQQMNGQQHYHQGNGQYQQQAS

LPPSQQQQPQQQHQHQQQQQQAQQHYQGNGGQRGYHNHHHHHGRGGRRNHNMQRGGHQSSRS

>KAF5122887.1 hypothetical protein DV452_000466 [Geotrichum candidum]

MSGESDWKSTLNIPAKDSRPQTEDVTATKGNGFEDFYLKRELLMGIFEAGFEKPSPIQEESIPLALAGRDILARAKNGTG

KTASFVIPALQQVNSKLNKIQALILVPTRELALQTSQVCKTLGKHLNLKIMVTTGGTTLKDDIVRLNDTVHILVATPGRI

LDLASKGVADLSECPMFIMDEADKLLSPEFTPVIEQLLEYFPSDRQSLLFSATFPLVVKSFMDKHLKRPYEINLMDELTL

KGITQYYAFVEEKQKLHCLNTLFSKLQINQSIIFCNSTNRVELLARKITELGYSCFYSHAKMVQANRNRVFHEFRNGGCR

NLVCSDLLTRGIDIQAVNVVINFDFPKNAETYLHRIGRSGRFGHLGLAINLISWNDRFNLYKIEQELGTEIKPIPAHIDA

SLYVADAVESIPRPFPMQELPKANTHSVQPRYHHHNGNQGGGGRFSNNGNGSEGYPNNNQSGGYQNNNYNQSQHPALPPP

QQQQQQPYYDNGSGYPQHPNQFNNHQQPNGYPPQPPPQQGYSNGQYQMH

>KNE68395.1 ATP-dependent RNA helicase dhh1 [Allomyces macrogynus ATCC 38327]

MSDQYDADPAVQQEWKHQLHLPPKDARPQTDDVLATKGNEFEDYYLKRELLMGIFEAGFERPSPIQEEAIPIALAGRDIL

ARAKNGTGKTAAFVIPTLEKINPQKHKIQALLLVPTRELALQTSQVCKTLGKHLGLQVMVTTGGTGLKDDIIRLQDTVHI

LVGTPGRLLDLAGKGVAQLDECTTFVMDEADKLLSPEFQPVIEQLLGYLPEDRQIMLFSATFPVLVKDFRDKWLNAPYEI

NLMDDLTLKGVTQYYAFVEERQKVHCLNTLFSKLQINQSIIFCNSTNRVELLAKKITDLGYSCFYIHARMPQAHRNRVFH

DFRQGVCRNLVCSDLLTRGIDIQAVNVVINFDFPKNAETYLHRIGRSGRFGHLGLAINLITYEDRFNLYRIETELGTEIQ

PIPPTVDKHLYVAPALQDAAPQQPQAATELPQQPQADHAHHGHHHHHHRNGHGGGGGGGGAPRATAYGSGYREHREQVPR

GGGGGTGGGGHHQHRQHHRQMQKEQAAHRGVPITSRIRRRPRGTQRVATSSKSRGTAVLRAIEQVAHRPRVR

>EGU12280.1 ATP-dependent RNA helicase dhh1 [Rhodotorula toruloides ATCC 204091]

MSAPQASSSAPQQQPEDWKKQLNLPTRDTRPQTEDVTATKGNEFEDYFLKRELLMGIFEAGFERPSPIQEEAIPIALTGR

DILARAKNGTGKTAAFIIPVLERVNPKIPKIQALLLVPTRELALQTSQVCKTLGKHTGAQVMVTTGGTTLRDDILRLGDT

VHILVGTPGRILDLAGKGIADLSQCPMFVMDEADKLLSPEFTPVIEQLLSFMPKERQVMLFSATFPLIVKDFKDKWMRKP

YEINLMDELTLRGVTQYYAFLEERQKVHCLNTLFSKLQINQSIIFCNSTNRVELLAKKITELGYSCFYSHARMLQQHRNR

VFHDFRNGVCRNLVCSDLLTRGIDIQAVNVVINFDFPKNAETYLHRIGRSGRYGHLGLAINLITYDDRFNLYRIEQELGT

EIQPIPPVIDRSLYVAPGTEEEVKGAQEAAKAGQKVARPAGVGGHGMQAPQVRQGPQPPATAPQQQQQQQPAPQQYQQPP

HQMPQQQQQQQQQQSQQQYYGQNALRPGQNGAPLPPHLQQQYGQQQQYGQPQQYSQQQQQQAFMLQQAQAMASRQQR

>XP_031855194.1 uncharacterized protein SAPINGB_P004588 [Saprochaete ingens]

MASNPTTSQDASSAAQNLNNSDWRSALNIPAKDTRPQTEDVTNTKGNGFEDFYLKRELLMGIFEAGFEKPSPIQEEAIPI

ALAGRDILARAKNGTGKTAAFVIPALQQVNTKINKIQALILVPTRELALQTSQVCKTLGKHLGIQVMATTGGTNLKDDIL

RLNETVHVLVGTPGRVLDFAGKQVIDFSECPMFIMDEADKLLSPEFTPVVEQLLGYFPASRQILLFSATFPLVVKSFMDK

HLNKPYEINLMDELTLRGITQYYAFVEEKQKLHCLNTLFSKLQINQSIIFCNSTNRVELLARKITELGYSCYYSHAKMQQ

QNRNRVFHEFRNGNCRNLVCSDLLTRGIDIQAVNVVINFDFPKNAETYLHRIGRSGRFGHLGLAINLISWNDRFSLYKIE

QELGTEIKPIPAHIDTSLYVADAVENIPKPFPITELPRDSSTSRVNGNNNQRYNNNRNQQDNQIYQQQQQFGQIPPQQYQ

YMPQQYAPLQQQQPQQQQQQLQQQHQQQQQQPQQQQQQIPAGYYNNTQGNNYPNYPPRPAFMQAPPPQQAAYYTQQR

>OJA17858.1 hypothetical protein AZE42_05899 [Rhizopogon vesiculosus]

MSQSARPSTSTSTSSSATPNDPSWRAGLRLPPKDLRPQTEDVTNTKGTEFEDMFLRRELLMGIFEEGYERPSPIQEEAIP

IALTKRDILARAKNGTGKTAAFVIPSLQQIDIGKNKIQALLLVPTRELALQTSQVCKKLGKHMGIQVMVTTGGTTLKDDI

MRLQETVHVLVGTPGRILDLAGKKVADLSECPVFVMDEADKLLSPEFTPVMEQLLTFLPADRQVMLFSATFPMMVKDFKD

KRMRNPYEINLMEELTLRGVTQYYAYVEERQKVHCLNTLFSKLQINQSIIFCNSTNRVELLAKKVTDLGYSCFYSHAKML

QSHRNRVFHDFRNGVCRNLVCSDLLTRGIDIQAVNVVINFDFPKNSETYLHRIGRSGRFGHLGLAINLVTYEDRFNLYRI

EQELGTEIQPIPQTIDKGLYVAPTAAEEPAPQKPQSQSQLQQTQQQQRQQVPSQQAPSNGRQNGAAPAPAPAPSQGRGGH

ANQAAYRNGAPPR

>GEM07293.1 ATP-dependent RNA helicase dhh1 [Rhodotorula toruloides]

MSQPQASSSSQQPQQGQDQQPEDWKKQLNLPQKDTRPQTEDVTATKGNEFEDYFLKRELLMGIFEAGFERPSPIQEEAIP

IALTGRDILARAKNGTGKTAAFIIPVLERVNPKIPKIQALLLVPTRELALQTSQVCKTLGKHTGAEVMVTTGGTTLRDDI

LRLGETVHILVGTPGRILDLAGKGIADLSQCPMFVMDEADKLLSPEFTPVIEQLLSFMPKERQVMLFSATFPLIVKDFKD

KWMRKPYEINLMDELTLRGVTQYYAFLEERQKVHCLNTLFSKLQINQSIIFCNSTNRVELLAKKITELGYSCFYSHARML

QQHRNRVFHDFRNGVCRNLVCSDLLTRGIDIQAVNVVINFDFPKNAETYLHRIGRSGRYGHLGLAINLITYDDRFNLYRI

EQELGTEIQPIPPVIDRSLYVAPGTEEEVKGAQEAAKAGQKVARPAGVGGHGMQTPQVRQGQPPLPAAAPQQQQQQQPAA

QQYQQPPRQISQQPPQQPYYGQNASRPGQNGAPLPPHLQQQHGQQQQQQQYGQQQQYGQPQQYNQQQQAFMQQQAQAMAS

RQQR

>EPZ36415.1 putative ATP-dependent RNA helicase ddx6 [Rozella allomycis CSF55]

MSNSVQNESNWKAKLQAPVKDLRPKTEDVTATKGNEFEDYYLKRELLMGIYEMGYEHPSPIQEESIPIALTGRDILARAK

NGTGKTAAFIIPVLEKVNVSKNEIQALLLVPTRELALQTAQVCKQLGKHMNVQVMVSTGGTNLKEDILRLNNTVHICVAT

PGRILDLAQKNVADFTKCNVVVMDEADKLLSPEFQPIVEQLINFLPKSRQILLYSATFPVTVKQFKEKYLVKPYEINLME

ELTLKGITQYYAFVEERQKVHCLNTLFSKLAINQSIIFCNSANRVELLAKKITELGYSCFYIHSRMPQAERNRVFHDFRS

GHCRNLVCTDLFTRGIDIQSVNVVINFDFPKNSETYLHRIGRSGRFGHLGLAVNLITFDDRFNLYKIEQELGTEIMPIPS

VIDKMLYAA

>THU82298.1 DEAD-domain-containing protein [Dendrothele bispora CBS 962.96]

MSQAQTPASSSSDNWKTGLRPPPKDFRPQTEDVTATKGVEFEDMKLRRELLMGIFEAGFEKPSPIQEEAIPIALTKRDVL

ARAKNGTGKTAAFVIPSLQQVDPLKNKIQALLLVPTRELALQTSQVCKILGKHMGIQVMVTTGGTTLKDDIMRLSETVHV

LVGTPGRILDLAGKSVADLSECPVFVMDEADKLLSPEFAPVMEQLLAYLPESRQVMLFSATFPMIVKDFKEKHMMSPYEI

NLMDELTLRGVTQYYAYVEERQKVHCLNTLFSKLQINQSIIFCNSTNRVELLAKKVTELGYSCFYSHAKMLQSHRNRVFH

DFRNGVCRNLVCSDLLTRGIDIQAVNVVINFDFPKNSETYLHRIGRSGRFGHLGLAINLVTYEDRFNLYRIEQELGTEIQ

PIPQNIDKGLYVAPSAGVGPTEDERKIQQQVQQARQQQQQLAQQQKLLAQQQAAAVAQMQQQQGQVVYQSNGQQPRSNGT

PTGYRGGVAAAAR

>XP_006957352.1 DEAD-domain-containing protein [Wallemia mellicola CBS 633.66]

MASTSQTQESEWKNQIRAPPKDLRPQTEDVTNTKGGEWEDFGLRRELLMGIFEAGFEKPSPIQEEAIPSAIEGRDILARA

KNGTGKTASFVIPSLEKINVQKPKIQALLLVPTRELALQTSQVCKTLGKHLGIQVMVTTGGTTLRDDIMRLADPVHILVG

TPGRILDLASKGVANLEECPTFVMDEADKLLSPEFTPVMEQLLGHLPSSRQVMLFSATFPLIVKDFKEKHMRNPHEINLM

DELTLRGITQYYAFVEERQKVHCLNTLFSKLQINQSIIFCNSTNRVELLAKKVTELGYSCFFSHAKMLQSHRNRVFHDFR

SGVCRNLVCSDLLTRGIDIQAVNVVINFDFPKNSETYLHRIGRSGRYGHLGLAINLITYEDRFSLYKIEQELGTEIQPIP

SQIDRSLYVAPNASNEDGQIQQSQQQNQQQKGLPTLPPTQQVMNAYAQPPPQPTFNQNQQQNNHHQYQQRSHRGGYNNNG

YYGNYRGRGGPPRGGVPRA

>TIC17271.1 alpha/beta-hydrolase [Wallemia mellicola]

MMDPLSLSVNLSPVVLRTLVRHLIKKRNKEDKREVDDPNDEYAFDQTFKLTRAFLDIAMNHPVEDLQNLGSTYVPPPFWV

HNVPVTVPMSICNDAAEHLIKAFGGEEPMRAAVGGTRWWQVRPTAGLPAEWICMKSDRKLATRHNKLKVREQPKDPLTPQ

FFQDIEGLPRTILYVHGGAYYWGSINTHRYLIWRMARKMRGKAFAVSYRLSPQYGFPCALHDVLSAYLYLLYPPEGAPHK

AVDPANLIFAGLPLPAGSIHISPWCDLSHSFPSVLQNYETDVIPKYGFVHKPSTLWPPPSEDLLQRTREHIKSSTLRSTS

PLKGLGTAASHKAEKGRRKEETDAAINAEHGRERMDIPREVPTVDINGKTVELKGQIQMYATNEQVGHPYVSPVLAHLGG

LCPLFIMASDGELLRDEAIYVAHKAARPDEFEYKEDAKRRLPSMNGIEGRYGPTKVHLQAYDKMCHDLPLFSMVTPAKYA

YRAMAAFCFHVTKDKAAQPSPAIKPAHNGMTMSDFELDNPLEMPPTPSSGLASPTKLPEAKSPRRKFSLLGLRNEGAGSS

DMSSPVTSNGSQDNAPSSDAERPIKAGPGTAGHIAVYDEATAFKDGNMIRERVSMGGVTRPMEPPEELEYLRMSVNEVGV

FKPDAVKRYIEGMESWNTKFKHAAKNVERQREKSLRESHIHAEKMMKENDDIFDIPEVTWRWQWALENEKPPPASIVARR

DTQEARRLVELVDKVDNKHASKTVNLWTLIVHLLSPQKSKYGVNDEDEVGPWTRDIEELKKKYQTDEKGEVIPEEVSADE

ENNFDNKKEAWMKSQEYRQESEWKNQIRAPPKDLRPQTEDVTNTKGGEWEDFGLRRELLMGIFEAGFEKPSPIQEEAIPS

AIEGRDILARAKNGTGKTASFVIPSLEKINVQKPKIQALLLVPTRELALQTSQVCKTLGKHLGIQVMVTTGGTTLRDDIM

RLADPVHILVGTPGRILDLASKGVANLEECPTFVMDEADKLLSPEFTPVMEQLLGHLPSSRQVMLFSATFPLIVKDFKEK

HMRNPHEINLMDELTLRGITQYYAFVEERQKVHCLNTLFSKLQINQSIIFCNSTNRVELLAKKVTELGYSCFFSHAKMLQ

SHRNRVFHDFRSGVCRNLVCSDLLTRGIDIQAVNVVINFDFPKNSETYLHRIGRSGRYGHLGLAINLITYEDRFSLYKIE

QELGTEIQPIPSQIDRSLYVAPNASNEDGQIQQSQQQNQQQKGLPTLPPTQQVMNAYAQPPPQPTFNQNQQQNNHHQYQQ

RSHRGGYNNNGYYGNYRGRGGPPRGGVPRA

>TIB79700.1 alpha/beta-hydrolase [Wallemia mellicola]

MMDPLSLSVNLSPVVLRTLVRHLIKKRNKEDKREVDDPNDEYAFDQTFKLTRAFLDIAMNHPVEDLQNLGSTYVPPPFWV

HNVPVTVPMSICNDAAEHLIKAFGGEEPMRAAVGGTRWWQVRPTAGLPAEWICMKSDRKLATRHNKLKVREQPKDPLTPQ

FFQDIEGLPRTILYVHGGAYYWGSINTHRYLIWRMARKMRGKAFAVSYRLSPQYGFPCALHDVLSAYLYLLYPPEGAPHK

AVDPANLIFAGLPLPAGSIHISPWCDLSHSFPSVLQNYETDVIPKYGFVHKPSTLWPPPSEDLLQRTREHIKSSTLRSTS

PLKGLGTAASHKAEKGRRKEETNAAINAEHGRERMDIPREVPTVDINGKTVELKGQIQMYATNEQVGHPYVSPVLAHLGG

LCPLFIMASDGELLRDEAIYVAHKAARPDEFEYKEDAKRRLPSMNGIEGRYGPTKVHLQAYDKMCHDLPLFSMVTPAKYA

YRAMAAFCFHVTKDKAAQPSPAIKPAHNGMTMSDFELDNPLEMPPTPSSGLASPTKLPEAKSPRRKFSLLGLRNEGAGSS

DMSSPVTSNGSQDNAPSSDAERPIKAGPGTAGHIAVYDEATAFKDGNMIRERVSMGGVTRPMEPPEELEYLRMSVNEVGV

FKPDAVKRYIEGMESWNTKFKHAAKNVERQREKSLRESHIHAEKMMKENDDIFDIPEVTWRWQWALENEKPPPASIVARR

DTQEARRLVELVDKVDNKHASKTVNLWTLIVHLLSPQKSKYGVNDEDEVGPWTRDIEELKKKYQTDEKGEVIPEEVSADE

ENNFDNKKEAWMKSQEYRQESEWKNQIRAPPKDLRPQTEDVTNTKGGEWEDFGLRRELLMGIFEAGFEKPSPIQEEAIPS

AIEGRDILARAKNGTGKTASFVIPSLEKINVQKPKIQALLLVPTRELALQTSQVCKTLGKHLGIQVMVTTGGTTLRDDIM

RLADPVHILVGTPGRILDLASKGVANLEECPTFVMDEADKLLSPEFTPVMEQLLGHLPSSRQVMLFSATFPLIVKDFKEK

HMRNPHEINLMDELTLRGITQYYAFVEERQKVHCLNTLFSKLQINQSIIFCNSTNRVELLAKKVTELGYSCFFSHAKMLQ

SHRNRVFHDFRSGVCRNLVCSDLLTRGIDIQAVNVVINFDFPKNSETYLHRIGRSGRYGHLGLAINLITYEDRFSLYKIE

QELGTEIQPIPSQIDRSLYVAPNASNEDGQIQQSQQQNQQQKGLPTLPPTQQVMNAYAQPPPQPTFNQNQQQNNHHQYQQ

RSHRGGYNNNGYYGNYRGRGGPPRGGVPRA

>SCV74050.1 BQ2448_6482 [Microbotryum intermedium]

MSASTRTTRPNQQQTRPQQPQQPQPQPSASSSSSSSLTNNTTAVATQGTTPADWKAKLNLPAKDHRPQTEDVTATKGNEF

EDYFLKRELLMGIFEAGFERPSPIQEEAIPIALTGRDILARAKNGTGKTAAFIIPTLERVNPKINKIQALLLVPTRELAL

QTSQVCKTLGKHTGAQIMVTTGGTTLRDDILRLGEPVHILVGTPGRILDLAGKGIADLSACPMFVMDEADKLLSPEFTPV

IEQLLKLMPKERQVMLFSATFPLIVKDFKDKWMTKPYEINLMDELTLRGVTQYYAFLEERQKVHCLNTLFSKLQINQSII

FCNSTNRVELLAKKITELGYSCFYSHARMLQAHRNRVFHDFRNGVCRNLVCSDLLTRGIDIQAVNVVINFDFPKNAETYL

HRIGRSGRYGHLGLAINLITYDDRFDLYRIEQELGTEIQPIPPVIDRSLYVAPGTEEETTQRPNQAQAQQHQHQQQQQQQ

QQRQAYPQQQPQQQQRSYQGQGQQGQRGQQQQQQRPQQQQPQGEVYGGQQQNGQRRQ

>XP_007313871.1 hypothetical protein SERLADRAFT_354128 [Serpula lacrymans var. lacrymans S7.9]

MSQSTRPSSSNSAATNHNDSSWKSGLRPPPKDFRPQTEDVTATKGLEFEDMYLRRELLMGIFEAGFEKPSPIQEEAIPTA

LTKRDILARAKNGTGKTAAFVIPSLQQIDTSRNKIQALLLVPTRELALQTSQVCKILGKHMGVQVMVTTGGTTLKDDIMR

LSETVHVLVGTPGRILDLAGKNVADLSECPVFVMDEADKLLSPEFTPVMEQLLSFLPEERQVMLFSATFPMIVKDFKDKY

MKSPYEINLMEELTLRGVTQYYAYVEERQKVHCLNTLFSKLQINQSIIFCNSTNRVELLAKKVTELGYSCFYSHAKMLQS

HRNRVFHDFRNGVCRNLVCSDLLTRGIDIQAVNVVINFDFPKNSETYLHRIGRSGRFGHLGLAINLVTYDDRFNLYRIEQ

ELGTEIQPIPQTIDKGLYVAPSSSEETTTQKATQQTQQRQQTSAQQAQAAYQSNGQQSRSNGPAPPPQGQQPPRTYGGGY

RGGAPGPR

>KAF4618589.1 hypothetical protein D9613_010095 [Agrocybe pediades]

MSQPAPSSSSTPANGNDSSWKSGLRPPPKDIRPQTEDVTATKGLEFEDMSLKRELLMGIFEAGFEKPSPIQEEAIPIALS

KRDVLARAKNGTGKTAAFVIPSLQQIDLSKPKIQALLLVPTRELALQTSQVCKILGKHMGLQVMVTTGGTTLKDDILRLS

ETVHVLVGTPGRILDLAGKGVADLSECPVFVMDEADKLLSPEFAPVMEQLLSFLPSSRQVMLFSATFPMIVKDFKEKHMQ

SPYEINLMDELTLRGVTQYYAYVEERQKVHCLNTLFSKLQINQSIIFCNSTNRVELLAKKVTELGYSCFYSHAKMLQSHR

NRVFHDFRNGVCRNLVCSDLLTRGIDIQAVNVVINFDFPKNSETYLHRIGRSGRFGHLGLAINLVTYEDRFNLYKIEQEL

GTEIQPIPQTIDRGLYVAPSGGEESQVQRPPQQQQQARQQVAAAPVQRNGQQPQPHPVQAIPQQQQQQQQQQYAVRQNGV

PPQQQQPRGQPAYRGGVPVAR

>KZT25797.1 eukaryotic translation initiation factor 4A-like protein [Neolentinus lepideus HHB14362 ss-1]

MAQQSRPSSSASPNSTANDWKAGLRPPPKDSRPQTEDVTATKGLEFEDMYLRRELLMGIFEAGFERPSPIQEEAIPVALT

KRDVLARAKNGTGKTAAFVIPSLQQIDVNKNKIQALLLVPTRELALQTSQVCKILGKHMGVQVMVTTGGTTLKDDIMRLS

EDVHVLVGTPGRVLDLAGKSVADLSECPVFVMDEADKLLSPEFTPVVEQLLSYLPKERQVMLFSATFPLIVKDFKDKHMS

SPYEINLMDELTLRGVTQYYAFVEERQKVHCLNTLFSKLQINQSIIFCNSTNRVELLAKKITELGYSCFYSHAKMLQSHR

NRVFHDFRNGVCRNLVCSDLLTRGIDIQAVNVVINFDFPKNSETYLHRIGRSGRFGHLGLAINLVTYEDRFSLYKIEQEL

GTEIQPIPSSIDKRLYVAPGAIAEGSDQSQKASAQKATANGQQQPQAQQSRPQPVQQGPVRPPQPNPGSNQVLYQSSSAQ

PQQARPNGVSMAQQQQRPYPSGPAPAGYRGGVAVPR

>KIJ15927.1 hypothetical protein PAXINDRAFT_168904 [Paxillus involutus ATCC 200175]

MSQSARPSSSNSPANHNDSSWKSSLRPPPKDVRPQTEDVTATKGTEFEDMFLRRELLMGIFEAGFEKPSPIQEEAIPIAL

TKRDILARAKNGTGKTAAFVIPSLQQIDVNKNKIQALLLVPTRELALQTAQVCKILGKHLGVQVMVTTGGTTLKDDIMRL

SEVVHVLVGTPGRILDLAGKNVADLSECPVFVMDEADKLLSPEFTPVMEQLLSFLPADRQVMLFSATFPLIVKDFKDKHM

RSPYEINLMEELTLRGVTQYYAYVEERQKVHCLNTLFSKLQINQSIIFCNSTNRVELLAKKVTELGYSCFYSHAKMLQSH

RNRVFHDFRNGVCRNLVCSDLLTRGIDIQAVNVVINFDFPKNSETYLHRIGRSGRFGHLGLAINLVTYEDRFNLYRIEQE

LGTEIQSIPQTIDKGLYVAPSSAEEPQKPQAQPQPQQQQRQQGPAHQGQSTHNGRQNGGQAPQGQPRGGYANQPAYRGNP

TSR

>OAX36647.1 DEAD-domain-containing protein [Rhizopogon vinicolor AM-OR11-026]

MSQSTRPSTSTSTSSSATPNDPSWRAGLRLPPKDLRPQTEDVTNTKGTEFEDMFLRRELLMGIFEEGYERPSPIQEEAIP

VALTKRDILARAKNGTGKTAAFVIPSLQQIDIGKNKIQALLLVPTRELALQTSQVCKKLGKHMGIQVMVTTGGTTLKDDI

MRLQETVHVLVGTPGRILDLAGKKVADLSECPVFVMDEADKLLSPEFTPVMEQLLTFLPADRQVMLFSATFPMMVKDFKD

KRMRNPYEINLMEELTLRGVTQYYAYVEERQKVHCLNTLFSKLQINQSIIFCNSTNRVELLAKKVTDLGYSCFYSHAKML

QSHRNRVFHDFRNGVCRNLVCSDLLTRGIDIQAVNVVINFDFPKNSETYLHRIGRSGRFGHLGLAINLVTYEDRFNLYRI

EQELGTEIQPIPQTIDKGLYVAPTATEEPAPQKPQSQSQLQQTQQQQRQQTPSQQAPSNGRQNGAAPAPSQGRGGHANQA

AYRNGAPAR

>TDL25197.1 DEAD-domain-containing protein [Rickenella mellea]

MAQAATPSSSSSNNKDWKANLRAPPKDDRPQTEDVTATKGTEFEDMYLRRELLMGIFEAGFERPSPIQEEAIPIALTKRD

ILARAKNGTGKTAAFVIPALQQIDVAKNKIQALLLVPTRELALQTSQVCKILGKHMGVHVMVTTGGTTLKDDILRLSETV

HVLVGTPGRILDLAGKGVADLSECPVFVMDEADKLLSPEFTPVMEQLLSYLPDSRQVMLFSATFPMIVKDFKEKHMDAPH

EINLMDELTLRGVTQYYAFVEERQKVHCLNTLFSKLQINQSIIFCNSTNRVELLAKKVTELGYSCFYSHAKMLQSHRNRV

FHDFRNGVCRNLVCSDLLTRGIDIQAVNVVINFDFPKNSETYLHRIGRSGRFGHLGLAINLITYEDRFNLYRIEQELGTE

IQPIPQQIDKNLYVAPSAIDEPAGQKTAPVPAQTQAPSQSQSKEQEAAAQQQQRQLLAAQARAAAVNPSLAQQQGQVLYQ

SNGQQSRQNGPTQAVQQQQQPTAQRVGQQQQPYRGTPPTPGVIPRGGARQPVAR

>CDO74578.1 hypothetical protein BN946_scf184583.g5 [Trametes cinnabarina]

MAQQARPSSSTSNSDAWKSGLRPPPKDYRPQTEDVTATKGVEFEDMYLRRELLMGIYEAGFEKPSPIQEEAIPIALTKRD

ILARAKNGTGKTAAFVIPSLQQIDVNKNKIQALLLVPTRELALQTSQVCKTLGKHMGVQVMVTTGGTTLKDDIMRLSETV

HVLVGTPGRILDLAGKNVADLSECPVFVMDEADKLLSPEFAPVMEQLLSYLPKDRQVMLFSATFPMIVKDFKDKHMKSPY

EINLMDELTLRGVTQYYAYVEERQKVHCLNTLFSKLQINQSIIFCNSTNRVELLAKKVTELGYSCFYSHAKMLQSHRNRV

FHDFRNGVCRNLVCSDLLTRGIDIQAVNVVINFDFPKNSETYLHRIGRSGRFGHLGLAINLVTYEDRFNLYKIEQELGTE

IQPIPQTIDKGLYVAPTSPPEENEQQQQQQQKAKQPQPQPQRQALPPAVSPAQQRQGPAQVVYQSNPQPPRANGNGIPQQ

VQYQNGYRGVPVAR

>OMH83480.1 ATP-dependent RNA helicase dhh1 [Zancudomyces culisetae]

MASTKTRTVGEGEQEWKQSLKVPEKDLRFQTEDVTATKGNEFEDYYLKRELLMGIFEAGFEKPSPIQEESIPIALTGRDI

LARAKNGTGKTAAFIIPMLERINTANTHVQSLILVPTRELALQTSQVCKTLGKHMQINVMVTTGGTTLKDDIMRLSETVH

VLVGTPGRVLDLADKGVVNLKTAEMFIMDEADKLLSQEFTPVIEKLLAHFQAKRQILLYSATFPLMVKEFKDKFMVKPYE

INLMDELTLKGITQYYAFVEEKQKVHCLNTLFSKLHINQSIIFCNSTNRVELLARKITELGYSCFYSHAKMLQQHRNRVF

HDFRNGACRNLVCSDLLTRGIDIQAVNVVINFDFPKNAETYLHRIGRSGRFGHLGLAINLVTYEDRFNLYRIEQELGTEI

LPIPAVIDKRLYVAPSDSQDAPQKPLKNPQSNPPVQYQPRRHNPHQSNPRYTNGPPGMRDGNSYRPAHPSQNPENAPPGY

HSTSSYSQPHRNRYYRSNKKPTP

>OAD02367.1 hypothetical protein MUCCIDRAFT_88745 [Mucor lusitanicus CBS 277.49]

MSLNNNNLSHENDNWKKSLALPAKDMRPQTEDVTATKGNEFEDYFLKRELLMGIFEAGFERPSPIQEEAIPIALTGRDIL

ARAKNGTGKTAAFVIPTLEKINNKKNKIQALLLVPTRELALQTSQVCKNLGKHLGLQIMVTTGGTTLKDDIMRLSEPVHV

VVGTPGRILDLASKGVADFSEASTFVMDEADKLLSPEFTPIIEQLIHHFPKDRQIMLFSATFPLIVKTFKEKFLVKPYEI

NLMDELTLRGVTQYYAYVEEKQKVHCLNTLFSKLQINQSIIFCNSTNRVELLAKKITELGYSCFYSHARMLQSHRNRVFH

DFRNGVCRNLVCSDLLTRGIDIQAVNVVINFDFPKNAETYLHRIGRSGRFGHLGLAINLITYEDRFNLYKIERELGTEIQ

PIPPVIDKSLYVAPNALEDAQVQQPDRQQALATRQQQQQQSNEEQQQPHYYQNNNNGRANNWRGRGRGGGGGSGDYNTSV

Q

>CEQ42427.1 SPOSA6832_04243 [Sporidiobolus salmonicolor]

MSQPPSASPSVASTPAPQPEDWKKSLNLPQKDTRPQTEDVTATKGNEFEDYFLKRELLMGIFEAGFERPSPIQEEAIPIA

LTGRDILARAKNGTGKTAAFIIPTLERVNPKLNHIQALLLVPTRELALQTSQVCKTLGKHTGVQVMVTTGGTTLRDDILR

LGETVHVLVGTPGRILDLAGKGVADLSHCPMFVMDEADKLLSPEFTPVIEQLLAFMPKERQVMLFSATFPLIVKDFKDKW

MSKPYEINLMDELTLRGVTQYYAFLEERQKVHCLNTLFSKLQINQSIIFCNSTNRVELLAKKITELGYSCFYSHAKMLQQ

HRNRVFHDFRNGVCRNLVCSGATSLSILSLRFESRADLPASLADLLTRGIDIQAVNVVINFDFPKNAETYLHRIGRSGRY

GHLGLAINLITYEDRFNLYRIEQELGTEIQPIPAVIDRSLYVAPGMEEESQPQQKQGAASAEGQKARPSGVGGHGMAAPQ

QRQLPPPASSQRPPQLQQSAPAPAPASQGEDEEAE

>EMD35619.1 hypothetical protein CERSUDRAFT_96734 [Gelatoporia subvermispora B]

MAQPQQTRPSSSTSSSPNDTWKASLRPPPKDSRPQTEDVTATKGTEFEDMFLRRELLMGIFEAGFEKPSPIQEEAIPVAL

AKRDVLARAKNGTGKTAAFVIPTLQQVDVNKNKIQALLLVPTRELALQTAQVCKILGKHMGAQVMVTTGGTTLKDDILRL

SEAVHVLVGTPGRILDLAGKNVADLSECPVFVMDEADKLLSPEFAPVMEQLLSYLPKERQVMLFSATFPMIVKDFKEKHM

RSPHEINLMDELTLRGVTQYYAYVEERQKVHCLNTLFSKLQINQSIIFCNSTNRVELLAKKVTELGYSCFYSHAKMLQSH

RNRVFHDFRNGVCRNLVCSDLLTRGIDIQAVNVVINFDFPKNSETYLHRIGRSGRFGHLGLAINLVTYEDRFNLYKIEQE

LGTEIQPIPQTIDKGLYVAPASQDEPQEQKPAPARPAQNQAQQQPQAQRTPGQATQAATAQVVYQSGPQAPRQNGTAVPQ

QAQTQRQAYQAAYRGGGVPVAR

>TIB71390.1 hypothetical protein E3Q24_02352, partial [Wallemia mellicola]

VVLRTLVRHLIKKRNKEDKREVDDPNDEYAFDQTFKLTRAFLDIAMNHPVEDLQNLGSTYVPPPFWVHNVPVTVPMSICN

DAAEHLIKAFGGEEPMRAAVGGTRWWQVRPTAGLPAEWICMKSDRKLATRHNKLKVREQPKDPLTPQFFQDIEGLPRTIL

YVHGGAYYWGSINTHRYLIWRMARKMRGKAFAVSYRLSPQYGFPCALHDVLSAYLYLLYPPEGAPHKAVDPANLIFAGLP

LPAGSIHISPWCDLSHSFPSVLQNYETDVIPKYGFVHKPSTLWPPPSEDLLQRTREHIKSSTLRSTSPLKGLGTAASHKA

EKGRRKEETNAAINAEHGRERMDIPREVPTVDINGKTVELKGQIQMYATNEQVGHPYVSPVLAHLGGLCPLFIMASDGEL

LRDEAIYVAHKAARPDEFEYKEDAKRRLPSMNGIEGRYGPTKVHLQAYDKMCHDLPLFSMVTPAKYAYRAMAAFCFHVTK

DKAAQPSPAIKPAHNGMTMSDFELDNPLEMPPTPSSGLASPTKLPEAKSPRRKFSLLGLRNEGAGSSDMSSPVTSNGSQD

NAPSSDAERPIKAGPGTAGHIAVYDEATAFKDGNMIRERVSMGGVTRPMEPPEELEYLRMSVNEVGVFKPDAVKRYIEGM

ESWNTKFKHAAKNVERQREKSLRESHIHAEKMMKENDDIFDIPEVTWRWQWALENEKPPPASIVARRDTQEARRLVELVD

KVDNKHASKTVNLWTLIVHLLSPQKSKYGVNDEDEVGPWTRDIEELKKKYQTDEKGEVIPEEVSADEENNFDNKKEAWMK

SQEYRQESEWKNQIRAPPKDLRPQTEDVTNTKGGEWEDFGLRRELLMGIFEAGFEKPSPIQEEAIPSAIEGRDILARAKN

GTGKTASFVIPSLEKINVQKPKIQALLLVPTRELALQTSQVCKTLGKHLGIQVMVTTGGTTLRDDIMRLADPVHILVGTP

GRILDLASKGVANLEECPTFVMDEADKLLSPEFTPVMEQLLGHLPSSRQVMLFSATFPLIVKDFKEKHMRNPHEINLMDE

LTLRGITQYYAFVEERQKVHCLNTLFSKLQINQSIIFCNSTNRVELLAKKVTELGYSCFFSHAKMLQSHRNRVFHDFRSG

VCRNLVCSDLLTRGIDIQAVNVVINFDFPKNSETYLHRIGRSGRYGHLGLAINLITYEDRFSLYKIEQELGTEIQPIPSQ

IDRSLYVAPNASNEDGQIQQSQQQNQQQKGLPTLPPTQQVMNAYAQPPPQPTFNQNQQQNNHHQYQQRSHRGGYNNNGYY

GNYRGRGGPPRGGVPRA

>KAF5096746.1 hypothetical protein D0Z03_001562 [Galactomyces reessii]

MSDSDWKSNLNLPARDTRPQTEDVTATKGNGFEDFYLKRELLMGIFEAGFEKPSPIQEESIPIALAGRDILARAKNGTGK

TASFVIPALQQVNPKLSKIQALILVPTRELALQTSQVCKTLGKHLNINVMVTTGGTTLKDDIVRLNDAVHVLVGTPGRVL

DLAGKNVVDFSECPMFIMDEADKLLSPEFTPIIEQCLAYFPSSRQILLFSATFPLVVKSFMDKHLNKPYEINLMDELTLR

GITQYYAFVEEKQKLHCLNTLFSKLQINQSIIFCNSTNRVELLARKITELNYSCFYSHAKMLQSHRNRVFHEFRNGNCRN

LVCSDLLTRGIDIQAVNVVINFDFPKNAETYLHRIGRSGRFGHLGLAINLISWNDRFNLYKIEQELGTEIKPIPSTIDTS

LYVADSVESIPRPFPMQELPKTSSHQNRYQNNNNNNNQYQQQPQYPPQLQYQQQPYQQQPQFQQQPYQQQYRQQMRPPGF

NGNGNFPPQQQFQQRPGGY

>KZS87242.1 DEAD-domain-containing protein [Sistotremastrum niveocremeum HHB9708]

MAQPQASSSSSTPAESWKTGLRAPPTDARPQTEDVTATKGTEFEDMFLRRELLMGLFEAGFERPSPIQEEAIPVALAKRD

ILARAKNGTGKTAAFVVPALQQIDVTKQKIQALLLVPTRELALQTSQVCKTLGKHMGIQVMVTTGGTTLKDDILRLSDAV

HVLVGTPGRILDLVGKGVADLSECPVFVMDEADKLLSPEFTPVMEQLLSYLPQERQVMLFSATFPMIVKDFKDKHMRSPY

EINLMDELTLRGVTQYYAFVEERQKVHCLNTLFSKLQINQSIIFCNSTNRVELLAKKVTELGYSCFYSHAKMLQSHRNRV

FHDFRNGVCRNLVCSDLLTRGIDIQAVNVVINFDFPKSSETYLHRIGRSGRFGHLGLAINLITYEDRFNLYRIEQELGTE

IQPIPQNIDKTLYVAPAVIDEPVGQKNFRAGQGTDGSEKPQTNPNVAIPPPQRQPSIPPPGPVYTSNGQPPRMNGISPQI

LQAQAAQQMAQAARMQPSQQQAYINSNAGGYNGNVGRGGVPVNARGRGAVPRQVAR

>KAA8909245.1 hypothetical protein TRICI_004557 [Trichomonascus ciferrii]

MTTALSNQLENTSISSDSTQDWKSELNLPKKDSRPQTDDVTKTKGNGFEDFYLKRELLMGIFEAGFEKPSPIQEEAIPIA

LAGRDVLARAKNGTGKTAAFVIPALQQINPKIPKIQSLMLVPTRELALQTSQVCRTLGKHLGIKVMVTTGGTTLKDDIIR

LNDTVHILVGTPGRVLDLAGKNVVDFSECPMFIMDEADKLLSPEFTPIIEQLLAHFASDRQILLFSATFPLVVKSFMDKH

LNRPYEINLMDELTLRGITQYYAFVEEKQKLHCLNTLFSKLSINQSIIFCNSTNRVELLARKITELGYSCYYSHAKMLQS

HRNKVFHEFRNGNCRNLVCSDLLTRGIDIQAVNVVINFDFPKNAETYLHRIGRSGRFGHLGLAINLINWNDRFNLYKIEQ

ELGTEIKPIPAQIDKSLYVAEATESIPKPFPITDMPQTSQRVHRYHQGHRNNNRQQQQNRQPQQQPQPQVPQQYGYPHAG

AMPPPPPQGMMPPPHPQMGMPPNNGYYPPPPPPQTQ

>TIC45361.1 alpha/beta-hydrolase, partial [Wallemia mellicola]

VVLRTLVRHLIKKRNKEDKREVDDPNDEYAFDQTFKLTRAFLDIAMNHPVEDLQNLGSTYVPPPFWVHNVPVTVPMSICN

DAAEHLIKAFGGEEPMRAAVGGTRWWQVRPTAGLPAEWICMKSDRKLATRHNKLKVREQPKDPLTPQFFQDIEGLPRTIL

YVHGGAYYWGSINTHRYLIWRMARKMRGKAFAVSYRLSPQYGFPCALHDVLSAYLYLLYPPEGAPHKAVDPANLIFAGLP

LPAGSIHISPWCDLSHSFPSVLQNYETDVIPKYGFVHKPSTLWPPPSEDLLQRTREHIKSSTLRSTSPLKGLGTAASHKA

EKGRRKEETDAAINAEHGRERMDIPREVPTVDINGKTVELKGQIQMYATNEQVGHPYVSPVLAHLGGLCPLFIMASDGEL

LRDEAIYVAHKAARPDEFEYKEDAKRRLPSMNGIEGRYGPTKVHLQAYDKMCHDLPLFSMVTPAKYAYRAMAAFCFHVTK

DKAAQPSPAIKPAHNGMTMSDFELDNPLEMPPTPSSGLASPTKLPEAKSPRRKFSLLGLRNEGAGSSDMSSPVTSNGSQD

NAPSSDAERPIKAGPGTAGHIAVYDEATAFKDGNMIRERVSMGGVTRPMEPPEELEYLRMSVNEVGVFKPDAVKRYIEGM

ESWNTKFKHAAKNVERQREKSLRESHIHAEKMMKENDDIFDIPEVTWRWQWALENEKPPPASIVARRDTQEARRLVELVD

KVDNKHASKTVNLWTLIVHLLSPQKSKYGVNDEDEVGPWTRDIEELKKKYQTDEKGEVIPEEVSADEENNFDNKKEAWMK

SQEYRQESEWKNQIRAPPKDLRPQTEDVTNTKGGEWEDFGLRRELLMGIFEAGFEKPSPIQEEAIPSAIEGRDILARAKN

GTGKTASFVIPSLEKINVQKPKIQALLLVPTRELALQTSQVCKTLGKHLGIQVMVTTGGTTLRDDIMRLADPVHILVGTP

GRILDLASKGVANLEECPTFVMDEADKLLSPEFTPVMEQLLGHLPSSRQVMLFSATFPLIVKDFKEKHMRNPHEINLMDE

LTLRGITQYYAFVEERQKVHCLNTLFSKLQINQSIIFCNSTNRVELLAKKVTELGYSCFFSHAKMLQSHRNRVFHDFRSG

VCRNLVCSDLLTRGIDIQAVNVVINFDFPKNSETYLHRIGRSGRYGHLGLAINLITYEDRFSLYKIEQELGTEIQPIPSQ

IDRSLYVAPNASNEDGQIQQSQQQNQQQKGLPTLPPTQQVMNAYAQPPPQPTFNQNQQQNNHHQYQQRSHRGGYNNNGYY

GNYRGRGGPPRGGVPRA

>OSC97750.1 DEAD-domain-containing protein [Trametes coccinea BRFM310]

MAQQARPSSSTSNSDAWKSGLRPPPKDYRPQTEDVTATKGVEFEDMYLRRELLMGIYEAGFEKPSPIQEEAIPIALTKRD

ILARAKNGTGKTAAFVIPSLQQIDVNKNKIQALLLVPTRELALQTSQVCKTLGKHMGVQVMVTTGGTTLKDDIMRLSETV

HVLVGTPGRILDLAGKNVADLSECPVFVMDEADKLLSPEFAPVMEQLLSYLPKDRQVMLFSATFPMIVKDFKDKHMKSPY

EINLMDELTLRGVTQYYAYVEERQKVHCLNTLFSKLQINQSIIFCNSTNRVELLAKKVTELGYSCFYSHAKMLQSHRNRV

FHDFRNGVCRNLVCSDLLTRGIDIQAVNVVINFDFPKNSETYLHRIGRSGRFGHLGLAINLVTYEDRFNLYKIEQELGTE

IQPIPQTIDKGLYVAPTSPPDETEQQQQQQQQQKGKQAQGQLQPQPQRQALPQAASPAQQRQGPAQVVYQSNPQPPRANG

NGIPQQAQYQNGYRGVPVAR

>OLY83564.1 ATP-dependent RNA helicase dhh1 [Smittium mucronatum]

MTDTNSTTAIESTNDWKAQLDLPPKDTRLQTEDVTNTKGNEFEDYYLKRELLMGIFEAGFEKPSPIQEESIPIALTGRDI

LARAKNGTGKTAAFVIPALEKINPASPSLQTLILVPTRELALQTSQVCKTIGKHLGINIMVTTGGTTLKDDILRLSETVH

ILVGTPGRVLDLASKNVIDFSKAETFIMDEADKLLSPEFTPVIEQLLGFFPPKRQILLYSATFPLVVKTFKDKHMVKPYE

INLMDELTLRGVTQYYAFVEEKQKVHCLNTLFSKLQINQSIIFCNSTNRVELLARKITELGYSCFYSHARMLQNHRNRVF

HDFRNGSCRNLVCSDLLTRGIDIQAVNVVINFDFPRNAETYLHRIGRSGRFGHLGLAINLVTYEDRFNLYKIEQELGTEI

QPIPPIIDKRLYVAPSAIDEYPNQPMQQEQRQQQHLQHQQMQQRKVNNNFNRNYNNNNNNYSNQRQYHNNNSENYNDNAN

PNADGSNQYQNNNGANRGQYQNNGNRNQRQPNYNNDDQNQKNSNPQNKSNFNGPPGYQNQNRNSRQNENSQNRNYRPFRQ

NNQNNQNSNNQ

>KII87922.1 hypothetical protein PLICRDRAFT_54962 [Plicaturopsis crispa FD-325 SS-3]

MSQQQSSSSSTPASSTPTSLEPWKAGLRAPPKDARPQTEDVTATKGIEFEDMFLRRELLMGIFEAGFEKPSPIQEEAIPT

ALTKRDVLARAKNGTGKTAAFVIPALQQVDVQKNKIQALLLVPTRELALQTSQVCKALGKHMGVQVMVTTGGTTLKDDIL

RLSETVHVLVGTPGRILDLAGKSVADLSECPVFVMDEADKLLSPEFAPVMEQLLSFCPSDRQVMLFSATFPMIVKDFKDK

HMNQPYEINLMDELTLRGVTQYYAYVEERQKVHCLNTLFSKLQINQSIIFCNSTNRVELLAKKVTELGYSCFYSHAKMLQ

AHRNRVFHDFRNGVCRNLVCSDLLTRGIDIQAVNVVINFDFPKNSETYLHRIGRSGRFGHLGLAINLVTYEDRFNLYRIE

QELGTEIMPIPQQIDKGLYVAPGTADESGGQKPGVQGQTSQQQQLQRQQGPARPPQQQQPAQGQIVYQSNGRASANGAPL

QIQGLAARTQARTGAQVAR

>KAF3880324.1 DEAD-domain-containing protein [Suillus brevipes Sb2]

MSQSARPSTSTSTASSATPNDASWRAGLRPPPKDLRPQTEDVTNTKGTEFEDMFLRRELLMGIFEAGFERPSPIQEEAIP

VALTKRDILARAKNGTGKTAAFVIPSLQQIDVGKNKIQALLLVPTRELALQTSQVCKILGKHMGIQVMVTTGGTTLKDDI

MRLSETVHVLVGTPGRILDLAGKNVADLSECPVFVMDEADKLLSPEFTPVMEQLLSFLPTDRQVMLFSATFPMMVKDFKD

KRMRTPYEINLMEELTLRGVTQYYAYVEERQKVHCLNTLFSKLQINQSIIFCNSTNRVELLAKKVTDLGYSCFYSHAKML

QSHRNRVFHDFRNGVCRNLVCSDLLTRGIDIQAVNVVINFDFPKNSETYLHRIGRSGRFGHLGLAINLVTYEDRFNLYRI

EQELGTEIQPIPQAIDKGLYVAPSAAEEPAPQKPQPQPSQQQRQQAPSQQTSNNGRQNGAAPAPSQGQQAQRSSLVNQAV

YRNGTPTR

>XP_018271565.1 uncharacterized protein RHOBADRAFT_36372 [Rhodotorula graminis WP1]

MSAPSTSSSGAKSAPAPGPDDWKKTLNLPKKDARPQTEDVTATKGNEFEDYFLKRELLMGIFEAGFERPSPIQEEAIPIA

LTGRDILARAKNGTGKTAAFIIPTLERVNPKVPKIQALLLVPTRELALQTSQVCKTLGKHTGANIMVTTGGTTLRDDILR

LGEEVHILVGTPGRILDLAGKGIADLSECPMFVMDEADKLLSPEFTPVIEQLLSFMPKERQVMLFSATFPLIVKDFKDKW

MRKPYEINLMDELTLRGVTQYYAFLEERQKVHCLNTLFSKLQINQSIIFCNSTNRVELLAKKITELGYSCFYSHARMLQA

HRNRVFHDFRNGVCRNLVCSDLLTRGIDIQAVNVVINFDFPKNAETYLHRIGRSGRYGHLGLAINLITFDDRFNLYRIEQ

ELGTEIQPIPPVIDRSLYVAPGTEDEVKEAQAAAAQGQKSRPAGVGGHGMQAPQQRQVAAAPAPVAAAAAPQPQAQAPSA

PAPAQQQPQQQQQRGQQQQQPAYQQAPPPQQQPYYGGYPQQQRAPQGYGAPQGQQQQIPPHLMAQYQAQQQAMLAQQQQY

LAQQQQQQGGAARR

>CED82342.1 atp-dependent rna helicase dhh1 [Phaffia rhodozyma]

MASSSSTSQSSEWKSKLNLPQRDARPQTEDVLNTKGGDFEDYNLKRELLMGIFEAGFEKPSPIQEEAIPVALTGRDILAR

AKNGTGKTAAFTIPSLQRVNPKRPKIQALLLVPTRELALQTSQVCKTLGKHLGINVMVTTGGTTLKDDIMRLAEPVHVLV

GTPGRILDLAGKGIADLSECPTFVMDEADKLLSPEFQPVMEQLLSFCPPERQVMLFSATFPLIVKDFKDKHMVQPHEINL

MEELTLRGVTQYYAFVEERQKVHCLNTLFSKLQINQSIIFCNSTNRVELLAKKITELGYSCFYSHAKMLQAHRNRVFHDF

RQGVCRNLVCSDLLTRGIDIQAVNVVINFDFPKTAETYLHRIGRSGRFGHLGLAINLLTYEDRFNLYRIEQELGTEIQPV

PSVIDARLYVAPSAIQMDQWTEGSIERIAATSSSSNFTASTP

>XP_024337664.1 hypothetical protein POSPLADRAFT_1074739 [Postia placenta MAD-698-R-SB12]

MAQQQQARPSSSTANSDAWKSGLRPPPKDDRPQTEDVTATRGLEFDEMFLRRELLMGIYEAGFEKPSPIQEEAIPIALTK

RDVLARAKNGTGKTAAFVIPSLQQVDITKNKIQALLLVPTRELALQTSQVCKILGKHMGVQVMVTTGGTTLKDDIIRLSE

TVHVLVGTPGRILDLAGKNVADLSECPVFVMDEADKLLSPEFAPVMEQLLSYLPKDRQVMLFSATFPLIVKDFKDKHMKS

PYEINLMDELTLRGVTQYYAYVEERQKVHCLNTLFSKLQINQSIIFCNSTNRVELLAKKVTELGYSCFYSHAKMLQSHRN

RVFHDFRNGVCRNLVCSDLLTRGIDIQAVNVVINFDFPKNSETYLHRIGRSGRFGHLGLAINLVTYEDRFNLYKIEQELG

TEIQPIPQYIDKGLYVAPGATEEAPEQPQPQQKPRANQAQPQQPRQSAPQSQPVPGQVVYSSAPQQARVNGNGTAVPQAQ

QPPRAAYNGQAAYRGGVPVAR

>XP_018740460.1 uncharacterized protein MSY001_1904 [Malassezia sympodialis ATCC 42132]

MATTAPGNDPPPVQRPPRDLRPQTEDVTATKGNDFEDFFLKRELLMGIFEAGFERPSPIQEESIPIALSGRDILARAKNG

TGKTAAYVIPSLERINPKKEKIQAVLLVPTRELALQTSQVAKTLGKHLGVEIMVSTGGTTLKDDILRLGQTVHMLVGTPG

RILDLASKGIADLSQCTTFVMDEADKLLSPEFTPVMEQLLGLLPKERQVMLFSATFPLIVKDFKDKHMVKPYEINLMDEL

TLRGVTQYYAFVEERQKVHCLNTLFSKLQINQSIIFCNSTNRVELLAKKITELGYSCFYSHAKMLQAHRNRVFHDFRQGA

CRNLVCSDLLTRGIDIQAVNVVINFDFPKNAETYLHRIGRSGRFGHMGLAINLITYEDRFNLYRIEQELGTEVQPIPATI

DKRLYVAPSLIEEAEQREREAKATGKPTQGPGVMQQGLPSIPPTQAAMHAVPMEHNGNFTNTVPHRRGGRRNRRGGGGNG

NGSKNNGNGSQSQTS

>PIL23281.1 transporter [Ganoderma sinense ZZ0214-1]

MSQQARPSSSTSHNENWKTGLRPPPKDYRPQTEDVTATKGVEFEDMFLRRELLMGIFEAGFEKPSPIQEEAIPVALTKRD

VLARAKNGTGKTAAFVIPSLQQVDVSKNKIQALLLVPTRELALQTAQVCKTLGKHMGVQVMVTTGGTTLKDDIMRLSETV

HVLVGTPGRILDLAGKGVADLSECPVFVMDEADKLLSAEFAPVMEQLLSYLPKDRQVMLFSATFPIIVKDFREKHMKSPY

EINLMEELTLRGVTQYYAYVEERQKVHCLNTLFSKLQINQSIIFCNSTNRVELLAKKVTELGYSCFYSHAKMLQSHRNRV

FHDFRNGVCRNLVCSDLLTRGIDIQAVNVVINFDLPKNAETYLHRIGRSGRFGHLGLAINLVTYEDRFNLYKIEQELGTE

IQPIPQQIDKGLYVAPSGPSEDNVDQKGKAVVQQPQPQPQPAQRQGQPGPQGRQQVVYQSGPQQRVNGGAVPPYPNGYRG

VPVAR

>XP_007331967.1 hypothetical protein AGABI1DRAFT_115242 [Agaricus bisporus var. burnettii JB137-S8]

MSQPSSSSTPHDSAWKTGLRPPPRDSRPQTEDVTATKGLEFEDMSIRRELLMGIFEAGFEKPSPIQEEAIPFALTKRDIL

ARAKNGTGKTAAFVIPSLQQIDINKHKIQALLLVPTRELALQTSQVCKTLGKHMGIQVMVTTGGTTLKDDILRLSESVHV

LVGTPGRILDLAGKGVADLSECPVFVMDEADKLLSPEFAPVMEQLLSYLPSERQVMLFSATFPMIVKTFKDKHMRSPYEI

NLMDELTLRGVTQYYAYVEERQKVHCLNTLFSKLQINQSIIFCNSTNRVELLAKKVTELGYSCFYSHAKMLQSHRNRVFH

DFRNGVCRNLVCSDLLTRGIDIQAVNVVINFDFPKNSETYLHRIGRSGRFGHLGLAINLVTYEDRFNLYKIEQELGTEIQ

PIPQTIDRTLYVAPGSSEEQHQKPQPQSQQPQQRQAAPSPSPAAAAAPRAGQPSLPAPQSISQQPAFNGQASARANGGLP

PQAQQMRGQPTYRGGVPVAR

>TFK67341.1 DEAD-domain-containing protein [Pluteus cervinus]

MSQVPSTSSSTPSNNDWKAGLRAPPKDFRPQTEDVTATKGLEFEDMFLRRELLMGIFEAGFEKPSPIQEEAIPIALAKRD

VLARAKNGTGKTAAFVIPALQQIDVNKPKIQALLLVPTRELALQTSQVCKILGKHMGVQVMVTTGGTTLKDDILRLSESV

HVLVGTPGRILDLAGKNVADLSECPVFVMDEADKLLSAEFAPVMEQLLSYLPSGRQVMLFSATFPLIVKDFKEKHMSSPY

EINLMDELTLRGVTQYYAYVEERQKVHCLNTLFSKLQINQSIIFCNSTNRVELLAKKVTELGYSCFYSHAKMLQSHRNRV

FHDFRNGVCRNLVCSDLLTRGIDIQAVNVVINFDFPKNSETYLHRIGRSGRFGHLGLAINLVTYEDRFNLYKIEQELGTE

IQPIPQIIDKGLYVAPNATEEASAPQQKQTPQQQQQQRQAQAVASAAAPRAPAPTQGQVVYQSNGQHPRQNGGTPTGMTQ

AQLKAQAAYRGGQAPAAR

>KAF1806741.1 ATP-dependent RNA helicase DHH1 [Mucor lusitanicus]

MSLNNNNLSHENDNWKKSLALPAKDMRPQTEDVTATKGNEFEDYFLKRELLMGIFEAGFERPSPIQEEAIPIALTGRDIL

ARAKNGTGKTAAFVIPTLEKINNKKNKIQALLLVPTRELALQTSQVCKNLGKHLGLQIMVTTGGTTLKDDIMRLSEPVHV

VVGTPGRILDLASKGVADFSEASTFVMDEADKLLSPEFTPIIEQLIHHFPKDRQIMLFSATFPLIVKTFKEKFLVKPYEI

NLMDELTLRGVTQYYAYVEEKQKVHCLNTLFSKLQINQSIIFCNSTNRVELLAKKITELGYSCFYSHARMLQSHRNRVFH

DFRNGVCRNLVCSDLLTRGIDIQAVNVVINFDFPKNAETYLHRIGRSGRFGHLGLAINLITYEDRFNLYKIERELGTEIQ

PIPPVIDKSLYVAPNALEDAQVQQPDRQQALATRQQQQQQSNEEQQQPHYYQNNNNGRANNWRGRGRGGGGGSGGGGARG

REGNGGYQTRNI

>XP_017993843.1 atp-dependent rna helicase dhh1 [Malassezia pachydermatis]

MASTAPGSDGLPSVQRPPKDTRPQTEDVTATKGNDFEDFFLKRELLMGIFEAGFEHPSPIQEEAIPIALSGRDILARAKN

GTGKTGAYVIPSLERINTKKNKIQAVLLVPTRELALQTSQVAKTLGKHLGVEIMVSTGGTTLKDDILRLGQTVHMLVGTP

GRILDLASKGIADLSQCTTFVMDEADKLLSPEFTPVMEQLLALLPKERQVMLFSATFPLIVKDFKDKHMVKPYEINLMDE

LTLRGVTQYYAFVEERQKVHCLNTLFSKLQINQSIIFCNSTNRVELLAKKITELGYSCFYSHAKMLQAHRNRVFHDFRNG

ACRNLVCSDLLTRGIDIQAVNVVINFDFPKNAETYLHRIGRSGRFGHMGLAINLITYEDRFNLYRIEQELGTEVQPIPAT

IDKRLYVAPSLIEEAEKREKEAAASGKPAQGPGIMQQGLPTIPPTQAAMHAVPVEQNGNFTNTVPQRRGGGRRNRRGGGN

NNNQQQTQKETPSST

>SGY46055.1 BQ5605_C001g00376 [Microbotryum silenes-dioicae]

MSASTRNSRPNPQQSTRPQQQPSASSSSSSSNTTTTTAVATQGPTPADWKAKLNLPAKDHRPQTEDVTATKGNEFEDYFL

KRELLMGIFEAGFERPSPIQEEAIPIALTGRDILARAKNGTGKTAAFIIPTLERVNPKIHKIQALLLVPTRELALQTSQV

CKTLGKHTGAQIMVTTGGTTLRDDILRLGEPVHILVGTPGRILDLAGKGIADLSACPMFVMDEADKLLSPEFTPVIEQLL

KLMPKERQVMLFSATFPLIVKDFKDKWMTKPYEINLMDELTLRGVTQYYAFLEERQKVHCLNTLFSKLQINQSIIFCNST

NRVELLAKKITELGYSCFYSHARMLQAHRNRVFHDFRNGVCRNLVCSDLLTRGIDIQAVNVVINFDFPKNAETYLHRIGR

SGRYGHLGLAINLITYDDRFDLYRIEQELGTEIQPIPPVIDRSLYVAPGTEEETTQRPNQAQAQQSQQQQQQQRQAYPQQ

QQQGQQGQQQQRSYQKQGQQGQGGQQQQRPQQGQQPQGGGYGGQQQNGQRRQ

>KAF5113885.1 hypothetical protein DV454_003270 [Geotrichum candidum]

MSDSDWKSNLNLPARDTRPQTEDVTATKGNGFEDFYLKRELLMGIFEAGFEKPSPIQEESIPIALAGRDILARAKNGTGK

TASFVIPALQQVNPKLAKIQALILVPTRELALQTSQVCKTLGKHLNINVMVTTGGTTLKDDIIRLNDAVHVLVGTPGRVL

DLAGKNVVDFSECPMFIMDEADKLLSPEFTPIIEQCLAYFPSSRQILLFSATFPLVVKSFMDKHLNKPYEINLMDELTLR

GITQYYAFVEEKQKLHCLNTLFSKLQINQSIIFCNSTNRVELLARKITELNYSCFYSHAKMLQSHRNRVFHEFRNGNCRN

LVCSDLLTRGIDIQAVNVVINFDFPKNAETYLHRIGRSGRFGHLGLAINLISWNDRFNLYKIEQELGTEIKPIPSTIDTS

LYVADSAENIPRPFPMQELPKTSSHQNRYQNNNNNNNNQYQQQQQYQQQSQYPQQQQYQQQPQYQQQPYQQQQFQQQPYQ

QQQQQPYRQQIPPPGFNGGNGNFPPQQQFQQRPSGY

>KIK99181.1 hypothetical protein PAXRUDRAFT_23846 [Paxillus rubicundulus Ve08.2h10]

MSQSARPSSSNSLANRNDSSWKSSLRPPPKDIRPQTEDVTATKGTEFEDMFLRRELLMGIFEAGFEKPSPIQEEAIPIAL

TKRDILARAKNGTGKTAAFVIPSLQQIDVNKNKIQALLLVPTRELALQTAQVCKILGKHLGVQVMVTTGGTTLKDDIMRL

SEVVHVLVGTPGRILDLAGKNVADLNECPVFVMDEADKLLSPEFTPVMEQLLSFLPADRQVMLFSATFPLIVKDFKDKHM

KSPYEINLMEELTLRGVTQYYAYVEERQKVHCLNTLFSKLQINQSIIFCNSTNRVELLAKKVTELGYSCFYSHAKMLQSH

RNRVFHDFRNGVCRNLVCSDLLTRGIDIQAVNVVINFDFPKNSETYLHRIGRSGRFGHLGLAINLVTYEDRFNLYRIEQE

LGTEIQSIPQTIDKGLYVAPSSVEEPQKPLAQPQPQQQQRQQPPAHQGQSTHNGRQNGGQVPQGQPRGSYANQPAYRGNP

TAR

>SCZ99239.1 BZ3500_MvSof-1268-A1-R1_Chr3-1g05897 [Microbotryum saponariae]

MSASTRNPRPNPQQATRLQQQQQPSASSSSSSSNTTTTTAVATQGTTPADWKAKLNLPAKDHRPQTEDVTATKGNEFEDY

FLKRELLMGIFEAGFERPSPIQEEAIPIALTGRDILARAKNGTGKTAAFIIPTLERVNPKINKIQALLLVPTRELALQTS

QVCKTLGKHTGAQIMVTTGGTTLRDDILRLGEPVHILVGTPGRILDLAGKGIADLSACPMFVMDEADKLLSPEFTPVIEQ

LLKLMPKERQVMLFSATFPLIVKDFKDKWMTKPYEINLMDELTLRGVTQYYAFLEERQKVHCLNTLFSKLQINQSIIFCN

STNRVELLAKKITELGYSCFYSHARMLQAHRNRVFHDFRNGVCRNLVCSDLLTRGIDIQAVNVVINFDFPKNAETYLHRI

GRSGRYGHLGLAINLITYDDRFDLYRIEQELGTEIQPIPPVIDRSLYVAPGTEEETIQRPNQAQAQQNQQQQQQQGQQGQ

PQQRSYQGQGQQSQGGQQQQRPQQGQQQQGGGYGGQQQNGQRRQ

>KIY47370.1 eukaryotic translation initiation factor 4A-like protein [Fistulina hepatica ATCC 64428]

MAQTPSSSASNDAWKTGLRAPPKDVRPQTEDVTNTKGIEFEDMFLRRELLVGIFEAGFEKPSPIQEEAIPVALTKRDILA

RAKNGTGKTAAFVIPCLQQIDPNKNKIQALLLVPTRELALQTSQVCKMLGKHMRINVMVTTGGTTLKDDIMRLSETVHVL

VGTPGRILDLAGKNVADLSECPVFVMDEADKLLSPEFSPVMEQLLAYLPEDRQVMLFSATFPMIVKDFKDKHMTTPHEIN

LMEELTLRGVTQYYAYVEERQKVHCLNTLFSKLQINQSIIFCNSTNRVELLAKKITELGYSCFYSHAKMLQSHRNRVFHD

FRNGVCRNLVCSDLLTRGIDIQAVNVVINFDFPRNSETYLHRIGRSGRYGHYGLAINLVTYEDRFSLYKIEQELGTEIAP

IPQNIDRGLYVAPNVPDEAPKPQAKPQQQQQQQQQQQQQQQQQQPIQSQPNGTPRNGAPQAQRVSASYRAVPGASAPVGQ

R

>KAF5103773.1 hypothetical protein DV451_001254 [Geotrichum candidum]

MSDSDWKSNLNLPARDTRPQTEDVTATKGNGFEDFYLKRELLMGIFEAGFEKPSPIQEESIPIALAGRDILARAKNGTGK

TASFVIPALQQVNPKLAKIQALILVPTRELALQTSQVCKTLGKHLNINVMVTTGGTTLKDDIIRLNDAVHVLVGTPGRVL

DLAGKNVVDFSECPMFIMDEADKLLSPEFTPIIEQCLAYFPSSRQILLFSATFPLVVKSFMDKHLNKPYEINLMDELTLR

GITQYYAFVEEKQKLHCLNTLFSKLQINQSIIFCNSTNRVELLARKITELNYSCFYSHAKMLQSHRNRVFHEFRNGNCRN

LVCSDLLTRGIDIQAVNVVINFDFPKNAETYLHRIGRSGRFGHLGLAINLISWNDRFNLYKIEQELGTEIKPIPSTIDTS

LYVADSAENIPRPFPMQELPKTSSHQNRYQNNNNNNNQYQQQQQYQQQSQYPQQQQYQQQPQYQQQPYQQQQFQQQPYQQ

QQQPYRQQIPPPGFNGGNGNFPPQQQFQQRPSGY

>KAF7729108.1 DExD/H-box ATP-dependent RNA helicase dhh1 [Apophysomyces ossiformis]

MSASINEQHLSHEHDNWKKNIVLPPKDKRPQTEDVTATKGNEFEDYFLKRELLMGIFEAGFERPSPIQEEAIPIALTGRD

ILARAKNGTGKTAAFVIPTLEKINNKKPKIQALLLVPTRELALQTSQVCKTLGKHLGIQVMVTTGGTTLKDDIMRLGEPV

HIVVGTPGRILDLASKNVADFSEATTFVMDEADKLLSPEFTPVIDQLIHFFPKDRQIMLFSATFPMIVKHFKDKYLVKPY

EINLMDELTLRGVTQYYAYVEEKQKVHCLNTLFSKLQINQSIIFCNSTNRVELLAKKITDLGYSCFYSHAKMLQSHRNRV

FHDFRNGVCRNLVCSDLLTRGIDIQAVNVVINFDFPKNAETYLHRIGRSGRFGHLGLAINLITYEDRFNLYKIERELGTE

IQPIPPVIDKNLYVAPSSLEDAQVQKPNRQAAESVRLQQQQQEYQPQYQPQYQSQRQHHHQQQHHHYHQQQRQEQRPHYD

QHGDANRGQRNGGRPPHQNSAGRPRPRPKHAA

>KAF5102873.1 hypothetical protein D0Z00_000131 [Geotrichum candidum]

MSDSDWKSNLNLPARDTRPQTEDVTATKGNGFEDFYLKRELLMGIFEAGFEKPSPIQEESIPIALAGRDILARAKNGTGK

TASFVIPALQQVNPKLAKIQALILVPTRELALQTSQVCKTLGKHLNINVMVTTGGTTLKDDIVRLNDAVHVLVGTPGRVL

DLAGKNVVDFSECPMFIMDEADKLLSPEFTPIIEQCLAYFPSSRQILLFSATFPLVVKSFMDKHLNKPYEINLMDELTLR

GITQYYAFVEEKQKLHCLNTLFSKLQINQSIIFCNSTNRVELLARKITELNYSCFYSHAKMLQSHRNRVFHEFRNGNCRN

LVCSDLLTRGIDIQAVNVVINFDFPKNAETYLHRIGRSGRFGHLGLAINLISWNDRFNLYKIEQELGTEIKPIPSTIDTS

LYVADSAENIPRPFPMQELPKTSSHQNRYQNNNNNNQYQQQQQQYQQQPQYPQQQQYQQQPQYQQQPYQQQQFQQQQYQQ

QQYRQQIPPPGFNGGNGNFPPQQQFQQRPGGY

>PBK63248.1 DEAD-domain-containing protein [Armillaria solidipes]

MSQASNAVAASSSNNNESWKSGLRPPPKDVRPQTEDVTATKGLEFEDMFLRRELLMGIFETGFEKPSPIQEEAIPIALTK

RDVLARAKNGTGKTAAFVIPSLQQVDISKPKIQALLLVPTRELALQTSQVCKHLGKHMGVNVMVTTGGTTLKDDIMRLAE

AVHVLVGTPGRILDLAGKNVADLSECPVFVMDEADKLLSPEFSPVMEQLLSYLPEERQVMLFSATFPMIVKDFKDKHMRT

PYEINLMDELTLRGVTQYYAYVEERQKVHCLNTLFSKLQINQSIIFCNSTNRVELLAKKVTDLGYSCFYSHAKMLQSHRN

RVFHDFRNGVCRNLVCSDLLTRGIDIQAVNVVINFDFPKNSETYLHRIGRSGRFGHLGLAINLVTYEDRFNLYRIEQELG

TEIQPIPQAIDRGLYVAPAAAEENNKQQTKQNGTAVAQTQQQTRPVYQGGR

>RXW19232.1 hypothetical protein EST38_g6627 [Psathyrella aberdarensis]

MAQPTPTSSSSTASADNSWKASLNQPKKDFRPQTEDVTATKGLEFEDMFLRRELLMGIFEAGFEKPSPIQEEAIPIALTK

RDVLARAKNGTGKTAAFVIPTLQQVDVNKPKIQALLLVPTRELALQTAQVCKILGKHMGVQVMVTTGGTTLKDDIMRLSD

TVHVLVGTPGRILDLAGKGVADLSECPVFVMDEADKLLSPEFAPVMEQLLAFLPQERQVMLFSATFPLIVKDFKDKHMRS

PYEINLMDELTLRGVTQYYAFVEERQKVHCLNTLFSKLQINQSIIFCNSTNRVELLAKKITELGYSCFYSHAKMLQSHRN

RVFHDFRSGICRNLVCSDLLTRGIDIQAVNVVINFDFPKNSETYLHRIGRSGRFGHLGLAINLVTYEDRFNLYKIEQELG

TEIQPIPQTIDRSLYVDPNAAEEEKQKPFQHPVLPQQQILQQQAQLAQQQQQQQARAAATPSPAIPPQQNRNIPQQQPVY

PTQGVPQQVRQNGQAPRGQPAYRGAAPVAR

>KDE02539.1 ATP-dependent RNA helicase dhh1 [Microbotryum lychnidis-dioicae p1A1 Lamole]

MSASTRNSRPNPQQATRPQQQPSASSSSSSSNTTTTTAVATQGPTPADWKAKLNLPAKDHRPQTEDVTATKGNEFEDYFL

KRELLMGIFEAGFERPSPIQEEAIPIALTGRDILARAKNGTGKTAAFIIPTLERVNPKIHKIQALLLVPTRELALQTSQV

CKTLGKHTGAQIMVTTGGTTLRDDILRLGEPVHILVGTPGRILDLAGKGIADLSACPMFVMDEADKLLSPEFTPVIEQLL

KLMPKERQVMLFSATFPLIVKDFKDKWMTKPYEINLMDELTLRGVTQYYAFLEERQKVHCLNTLFSKLQINQSIIFCNST

NRVELLAKKITELGYSCFYSHARMLQAHRNRVFHDFRNGVCRNLVCSDLLTRGIDIQAVNVVINFDFPKNAETYLHRIGR

SGRYGHLGLAINLITYDDRFDLYRIEQELGTEIQPIPPVIDRSLYVAPGTEEETTQRPNQAQAQQSQQQQQQQRQAYPQQ

QQQGQQGQQQQRSYQKQGQQGQGGQQQQRPQQGQQPQGGGYGGQQQNGQRRQ

>PFH48713.1 hypothetical protein AMATHDRAFT_149316 [Amanita thiersii Skay4041]

MSHTGPSTSSTCVLLLSLSPQSSLLPPHHTSHRPSNDNWKTGLRPPPKDLRPQTEDVTATRGLEFEDMYLRRELLMGIFE

AGFEKPSPIQEEAIPIALTKRDILARAKNGTGKTAAFVIPALQQIDVNKSKIQALLLVPTRELALQTSQVCKILGKHVGV

QVMVTTGGTTLKDDILRLSETVHVLVGTPGRILDLAGKGVADLSECPVFVMDEADKLLSPEFAPVMEQLLSYLPEDRQVM

LFSATFPMIVKDFKDKHMRSPYEINLMDELTLRGVTQYYAYVEERQKVHCLNTLFSKLQINQSIIFCNSTQRVELLAKKV

TELGYSCFYSHAKMLQSHRNRVFHDFRNGVCRNLVCSDLLTRGIDIQAVNVVINFDFPKNSETYLHRIGRSGRFGHLGLA

INLVTYEDRFNLYRIEQELGTEIQPIPQNIDRSLYVAPSLPEAAPPQQQQQAQQQQQQAQAPQRVQQTAAPQPIKNVQPV

YVSNGSQQQVRPNGVPPNVPTPQMRGTPVYRGGVPVAR

>XP_007869359.1 eukaryotic translation initiation factor 4A-like protein [Gloeophyllum trabeum ATCC 11539]

MAQQSRPSSSASPASSANDWKAGLRPPPKDARPQTEDVTATKGLEFEDMYLRRELLMGIFEAGFERPSPIQEEAIPVALT

KRDVLARAKNGTGKTAAFVIPALQQIDVNKNKIQALLLVPTRELALQTSQVCKILGKHMGVQVMVTTGGTTLKDDIMRLS

ETVHVLVGTPGRVLDLTGKNVADLSECPVFVMDEADKLLSPEFTPVVEQLLSYMPKERQVMLFSATFPLIVKDFKDKHMS

SPYEINLMDELTLRGVTQYYAFVEERQKVHCLNTLFSKLQINQSIIFCNSTNRVELLAKKITELGYSCFYSHAKMLQSHR

NRVFHDFRNGVCRNLVCSDLLTRGIDIQAVNVVINFDFPKNSETYLHRIGRSGRFGHLGLAINLVTYEDRFNLYKIEQEL

GTEIQPIPSTIDKRLYVAPGAAEEQQKEKASTANGQQQPRPQQHQQGPARPPQQAPNQVLYQSGAPPQQQVRPNGTSLGQ

PQHIMQQRAYPGGQVPVPTGYRGGVAVPR

>KTB39544.1 hypothetical protein WG66_7898 [Moniliophthora roreri]

MSQIATSSTATSPTGVRDNSWKTGLRPPPKDMRPQTEDVTKTKGVEFEDMSLRRELLMGIFEAGFERPSPIQEEAIPIAL

TKRDVLARAKNGTGKTAAFVIPSLQQIDPTKSRIQALLLVPTRELALQTSQVCKILGKHMGIQVMVTTGGTTLKDDIMRL

SETVHVLVGTPGRILDLAGKGVADLSECPVFVMDEADKLLSPEFAPVMEQLLAYLPENRQVMLFSATFPMIVKDFKASSA

LELQRDKHMRQPYEINLMDELTLRGVTQYYAYVEERQKVHCLNTLFSKLQINQSIIFCNSTNRVELLAKKVTELGYSCFY

SHAKMLQSHRNRVFHDFRNGVSRNLVCSDLLTRGIDIQAVNVVINFDFPKNSETYLHRIGRSGRFGHLGLAINLVTYEDR

FNLYRIEQELGTEIQPIPQIIDKGLYVAPSATEEEKAVQKQVQQQQQQQQQQQQQQRAQQEQVVYQSNGAPQSRGTPQGA

YRGGVPAGAR

>ODQ67748.1 putative DEAD-box RNA helicase Dhh1/Vad1 [Nadsonia fulvescens var. elongata DSM 6958]

MSTNQLESMHNSQEPNNWKNQLNIPNKDTRIQTEDVTNTKGNSFEDFYLKRELLMGIFEAGFEKPSPIQEESIPIALAGR

DILARAKNGTGKTAAFIIPALQKVNPKINKTQALILVPTRELALQTSQVCRTLGKHLGLNIMVTTGGTTLRDDILRLNET

VHILVATPGRVLDLAGKGVADFSECPMFVMDEADKLLSPEFTPIIEQLLAYFPTERQTLLFSATFPLLVKSFMDKHLNKP

YEINLMDELTLRGITQYYAFVDERQKLHCLNTLFSKLSINQSIIFCNSTSRVELLAKKITELGYSCFYSHAKMQQGHRNR

VFHEFRNGNCRNLVCSDLLTRGIDIQAVNVVINFDFPKNAETYLHRIGRSGRFGHLGLAINLINWNDRYNLYKIEQELGT

EILPIPASIDKSLYCADNNDSIPRPFPMAELPKTTTYPNNNGSNGGFRQNNQQYNGNQPPQNQPQLQAQPFYGNNPNVNG

GNPNIPYQQNGPLPQQQQQYGQYYQAQQPPHQQQQPSHQQSPQQASQQQQQQQQQPQGYFPPVMQQQPPTQAPQNY

>RDB29951.1 ATP-dependent RNA helicase dhh1 [Hypsizygus marmoreus]

MSQTVPSTSSTYAFSPHSPSSLAQSNGGWPQHQDQRRANTPSFIPSSSQPSSESWKAGLRPPPKDLRPQTEDVTATKGLE

FEDMFLRRELLMGIFEAGFEKPSPIQEEAIPIALTKRDVLARAKNGTGKTAAFVIPALQQVDVSKNKIQALLLVPTRELA

LQTAQVCKTLGKHMGVQVMVTTGGTTLKDDILRLSEEVHVLVGTPGRILDLAGKGVADLSECPVFVMDEADKLLSPEFAP

VMEQLLGYLPKERQVMLFSATFPMIVKDFKDKHMKSPYEINLMDELTLRGVTQYYAYVEERQKVHCLNTLFSKLQINQSI

IFCNSTNRVELLAKKVTELGYSCFYSHAKMLQSHRNRVFHDFRNGVCRNLVCSDLLTRGIDIQAVNVVINFDFPKNSETY

LHRIGRSGRFGHLGLAINLVTYEDRFNLYRIEQELGTEIQPIPQTIDKGLYVAPSSAEEPAGQKPSQATQQQQREQLQQR

QQGSTGTGQARLEIQQQQVVYQSSVQQTVVRQNGVVAAQPRGQPAAYRGAVPVAR

>TFL01643.1 P-loop containing nucleoside triphosphate hydrolase protein [Pterula gracilis]

MATAAPSSSSTPQKDWKAGLQAPPKDFRPRTEDVTGTKGTEFEDMFLRRELLMGIFEAGFEKPSPIQEEAIPVALTKRDV

LARAKNGTGKTAAFVIPTLQQVDVATKKIQALLLVPTRELALQTSQVCKILGKHMGVQVMVTTGGTTLKDDILRLSETVH

VLVGTPGRILDLAGKNVADLSECPVFVMDEADKLLSHEFAPVMEQLLSFLPKDRQVMLFSATFPIIVKDFKEKHMKDPYE

INLMEELTLRGVTQYYAYVEERQKVHCLNTLFSKLQINQSIIFCNSTNRVELLAKKVTELGYSCFYSHAKMLQGHRNRVF

HDFRNGVCRNLVCSDLLTRGIDIQAVNVVINFDFPKNSETYLHRIGRSGRFGHLGLAINLVTYEDRFNLYRIEQELGTEI

QPIPQQIDMHLYVAPSGSEQPPPQQQQQQQQQRQIAGAAQPNGPQPAQNGSAPGSQSRAAPPPQSAQASYQGQR

>SAL99522.1 hypothetical protein [Absidia glauca]

MSAPYNNQQLRYVVNDALAHENDDWKKTMTLPAKDTRPQTEDVTATKGNEFEDYFLKRELLMGIFEAGFERPSPIQEEAI

PIALTGRDILARAKNGTGKTAAFVIPTLEKVNNKKSKIQALLLVPTRELALQTSQVCKTLGKHLGVQIMVTTGGTTLKDD

IMRLSETVHVVVGTPGRILDLASKGVADFSEASTFVMDEADKLLSPEFTPIIDQLISFFPRDRQIMLFSATFPMIVKHFK

DKYLVKPYEINLMDELTLRGVTQYYAFVEEKQKVHCLNTLFSKLQINQSIIFCNSTNRVELLAKKITELGYSCFYSHAKM

LQSHRNRVFHDFRNGVCRNLVCSDLLTRGIDIQAVNVVINFDFPKNAETYLHRIGRSGRFGHLGLAINLITYEDRFNLYK

IERELGTEIQAIPPTIDKQLYVAPNALDDSQVQRPDREAAIATRQQQQQRESHPQDQQQQLQQGQYQNQNQQLVYHQQQQ

HQQQHYQQQRQQHYYQNGGRGRGGGYNGRGGYQGRGRPRQQQQQQQQQPYQQMPMN

>CDH58577.1 atp-dependent rna helicase dhh1 [Lichtheimia corymbifera JMRC:FSU:9682]

MSAQTSIHTMSHDEDEWKKSLQLPTRDNRPQTEDVTKTKGNEFEDYFLKRELLMGIFEAGFERPSPIQEESIPIALTGRD

ILARAKNGTGKTAAFVIPTLEKVNTKKSQIQALILVPTRELALQTSQVCKKLGKHLGVHVMVTTGGTTLKDDIMRLSEPV

HIVVGTPGRILDLASKNVADFSEASTFVMDEADKLLSPEFTPVIERLLAYFPNERQIMLFSATFPMVVKSFKEKYLVKPY

EINLMEDLTLKGVTQFYAYVEEKQKVHCLNTLFSKLQINQSIIFCNSTSRVELLAKKITELGYSCFYSHARMLQSHRNRV

FHDFRNGVCRNLVCSDLLTRGIDIQAVNVVINFDFPKNAETYLHRIGRSGRYGHLGLAINLITYEDRFNLYKIERELGTE

ITPIPAQIDKSLYVAPNALEENMVQQPQPKRMDQDGIQEQQQQHQQHENNHHHHFQQHGGRGGWGNRGRGGYWSHRGRGA

WSNRGRGGGGGANNNHRVRRVPKPLV

>KIL61043.1 hypothetical protein M378DRAFT_187630 [Amanita muscaria Koide BX008]

MSQTGPSTSASPVNDNWKAGLRPPQKDYRPQTEDVTATKGLEFEDMFLRRELLMGIFEAGFEKPSPIQEEAIPIALTKRD

VLARAKNGTGKTAAFIIPSLQQIDINRNKIQALLLVPTRELALQTSQVCKTLGKHMGVQVMVTTGGTTLKDDILRLSESV

HVLVGTPGRILDLASKGVADLSECPVFVMDEADKLLSPEFAPVMEQLLSYLPGERQVMLFSATFPMIVKDFKEKHMRSPH

EINLMDELTLRGVTQYYAYVEERQKVHCLNTLFSKLQINQSIIFCNSTQRVELLAKKVTELGYSCFYSHAKMLQAHRNRV

FHDFRNGVCRNLVCSDLLTRGIDIQAVNVVINFDFPKNSETYLHRIGRSGRYGHLGLAINLVTYEDRFNLYRIEQELGTE

IQPIPQSIDKSLYVAPSMPEETIPRPISTSQQQQQQQAQQRAQVQVQAQIKNAQSVYAANGSQQAQLRPPNGVPPNVATH

PQARGSPLYRGGVPPVAR

>KAF6744143.1 eukaryotic translation initiation factor 4A-like protein [Coprinellus angulatus]

MAQPASASSSSSTPAPNEEWKTGLKAPRKDMRPQTEDVTATKGLEFEDMFLRRELLMGIFEAGFEKPSPIQEEAIPIALT

KRDVLARAKNGTGKTAAFVIPTLQQVDVARNKIQALLLVPTRELALQTSQVCKILGKHMGVQVMVTTGGTTLKDDIMRLS

ETVHVLVGTPGRILDLAGKGVADLSECPVFVMDEADKLLSPEFAPVMEQLLAFLPQERQVMLFSATFPMIVKDFKDKHMT

TPYEINLMDELTLRGVTQYYAFVEERQKVHCLNTLFSKLQINQSIIFCNSTNRVELLAKKITELGYSCFYSHAKMLQSHR

NRVFHDFRNGVCRNLVCSDLLTRGIDIQAVNVVINFDFPKNSETYLHRIGRSGRFGHLGLAINLVTYEDRFNLYKIEQEL

GTEIQPIPQSIDRSLYVAPEVGEEERQRPFQNPVQPVPQQVQQQQVQAQQVQQQRVAPHQLQGQRQRNGRQQMMMMRQNG

QVPRGQGAYRGGAPVAR

>XP_007771542.1 DEAD-domain-containing protein [Coniophora puteana RWD-64-598 SS2]

MAHQQRPAPSTTNDTAWKSGLRPPPKDFRPQTEDVTATKGMDFEDMFLRRDLLMGIFEAGFEKPSPIQEEAIPIALTKRD

VLARAKNGTGKTAAFVIPSLQQVDITKNKIQALLLVPTRELALQTSQVCKTLGKHMGVQVMVTTGGTTLKDDIMRLGEVV

HVLVGTPGRILDLAGKNVADLSECPVFVMDEADKLLSPEFTPVMEQLLAYLPKERQVMLFSATFPMIVKDFKGKHMRNPY

EINLMEELTLRGVTQYYAYLEEKQKVHCLNTLFSKLQINQSIIFCNSTNRVELLAKKVTELGYSCFYSHAKMLQSHRNRV

FHDFRHGAFRNLVCSDLLTRGIDIQAVNVVINFDFPKNAETYLHRIGRSGRFGHLGIAINLVTYEDRFNLYRVEQELGTE

IMPIPSVIDRGLYVAPGATDEPQQKTQQQQSQQRNGAPAYGQAQAQQQNGRQAQQQAQGQAQRR

>TNY20915.1 DEAD-domain-containing protein [Rhodotorula diobovata]

MSAPSSSSAGPAPGPEDWKKTLNLPKKDIRPQTEDVTATKGNEFEDYFLKRELLMGIFEAGFERPSPIQEEAIPIALTGR

DILARAKNGTGKTAAFIIPTLERVNPKVPKIQALLLVPTRELALQTSQVCKTLGKHTGANIMVTTGGTTLRDDILRLGEE

VHILVGTPGRILDLAGKGIADLSECPMFVMDEADKLLSPEFTPVIEQLLSFMPKERQVMLFSATFPLIVKDFKDKWMRKP

YEINLMDELTLRGVTQYYAFLEERQKVHCLNTLFSKLQINQSIIFCNSTNRVELLAKKITELGYSCFYSHARMLQAHRNR

VFHDFRNGVCRNLVCSDLLTRGIDIQAVNVVINFDFPKNAETYLHRIGRSGRYGHLGLAINLITFDDRFNLYRIEQELGT

EIQPIPPVIDRSLYVAPGTEDEVKEAQAVAAQGQKSRPAGVGGHGMQAPQQRQAVPAPAAPAPAPQQQAPPPQQQQPQQP

YYGAGVGGYPQQQQQQPNGARPPPPPQQGFMQPQGSGQQQQQIPPHLLAQYQAQQQQAMMAQQQQQYLAQQQRQQQQQGR

R

>KKY21595.1 putative dna repair protein rad18 [Phaeomoniella chlamydospora]

MAPLKRGIDSLSNNESQSDAASLNGYESSDAGSDILQQAARKRAKLSKRHQADEDTPADDEIVNDDSDETDDSNQQTTQT

TQQLGNLPADNGIFRSIQVINFMCHKNFEFTFGPLINFICGKNGSGKSAVLTALTLCLGGKASSTNRGQSLKNFIKQGED

SASIIVKIKNEGIGAYLPDEYGKTIIVERHFNKSGTSGFKIKSEKGRIVSTRKGDLDEICEFFVFQIDNPMNVLSQDLAR

QFIGSSSPQEKYKFYMRGIQLEQLDQDYTILQEAIANMEKRLELREDDIKLLEERKEKARLKLQMSDRHESLRDRIRNYR

RQMAWAQVEEQERIVESFDDQLQTADHRIAEAEAETTQFEEAFEAAHNAASRAGAAQEDVGREIQQAEEEKKEVKSAHDA

AKHELSDFQGQQRAIRDALKAADTSIAEREREIANEKQRLEDLHGGSHGRRLAELDRLKDDAESARDAHKDHQNARAELD

DDLETAETAMSEKRKPIERQREEIEKAEGLLQTMMRNHGAQNNAFHERMPILLREIERESSFAEKPVGPVGNHIRLLKPE

WSSQLEKAFGATLSSFIVTSKRDMEILSKIMKRVKCELQILIGSSRAIDTRPHEPEESFDTVFRALAIDNDLVKNQLIIQ

HGIEQTLLIADLAQASRVLYDGPRPRNVKRCYCIHQYEKRKGFHLTYTRDGRGSQDPIGEFRGRPRMKTDIEIQINLQRD

AIQGLKRQLSRLEEEFRGAGSQVETCKQAITRHKREERELQIASQRADDAVDELKEEIEKDSVEDGRLEVLEQSLEEAKE

EKTVNEGSYQDSVNAIDQAKQKLLVERNRLAGMDARLEELRSKQRRVDAEAVRLAQDRKNALGEKNAAFSRIDDAKEDRA

RLNRRRQEVVDRVADYTEKASQISGRVNVDPGETPTSLDKKLEKLENDLRRMQHQSGGTREELAIEAARTHEAYQTALES

VLGLKRLGQKLNQSLAQRKKRWRLFRGYITRRAKSQFQAFLSERSFRGKILTNHEKKLLDIQVEPDITKAKSSGREAKTL

SGGEKSFSQICLLLSIWEAMGSRIRCLDEFDVFMDSVNRRMSIELLMSAARRSAGRQFILISPGAKEDFRMAPDVHVQEL

RPPERGQTTLELSDGTSDSDWKSQLNLPTKDSRVQTEDVTATKGLEFEDFYIKRDLLMGIFEAGFEKPSPIQEETIPVAL

TGRDILARAKNGTGKTAAFIIPTLERINPKNPKTQALILVPTRELALQTSQVCKTLGKHLGLNVMVTTGGTGLKDDIIRL

GDTVHIIVGTPGRILDLASKGVADLSECPIFVMDEADKLLSPEFTPVIEQLLNFHPKDRQVMLFSATFPLIVKSFKDKHM

RNPYEINLMDELTLRGITQYYAFVEEKQKVHCLNTLFSKLQINQSIIFCNSTNRVELLAKKITELGYSCFYSHAKMLQSN

RNRVFHDFRNGVCRNLVCSDLLTRGIDIQAVNVVINFDFPKNAETYLHRIGRSGRYGHRGLAINLINWEDRFNLYRIEQE

LGTEIQPIPQTIDKGLYVYDNPENIPRPPSSAPPRRPSQSTIQPGQVPANGEQQQPRRQGYQGGRGNYQHRSGSYRGGRG

QGRGRGGGIDSARSNGQSTGQVTQA

>PBK81442.1 DEAD-domain-containing protein [Armillaria gallica]

MSQASSAVAASSSNNNESWKSGLRPPPKDVRPQTEDVTATKGLEFEDMFLRRELLMGIFETGFEKPSPIQEEAIPIALTK

RDVLARAKNGTGKTAAFVIPSLQQVDILKPKIQALLLVPTRELALQTSQVCKHLGKHMGVNVMVTTGGTTLKDDIMRLSE

AVHVLVGTPGRILDLAGKNVADLSECPVFVMDEADKLLSPEFSPVMEQLLSYLPEERQVMLFSATFPMIVKDFKDKHMRT

PYEINLMDELTLRGVTQYYAYVEERQKVHCLNTLFSKLQINQSIIFCNSTNRVELLAKKVTDLGYSCFYSHAKMLQSHRN

RVFHDFRNGVCRNLVCSDLLTRGIDIQAVNVVINFDFPKNSETYLHRIGRSGRFGHLGLAINLVTYEDRFNLYRIEQELG

TEIQPIPQTIDRGLYVAPAAAEENNKQQTKQNGIAVAQTQQQTRPVYQGGR

>KAF7316432.1 hypothetical protein [Mycena indigotica]

MSHPPSSNPATSPPAAGESWKNGLRLPPKDLRPQTSDVTATKGTEFEDMFLRRELLMGIFEAGFEKPSPIQEEAIPIALT

KRDVLARAKNGTGKTAAFVIPSLQQVDVAKSKIQALLLVPTRELALQTSQVCKILGKHMGIQVMVTTGGTTLKDDIIRLS

ETVHVLVGTPGRILDLAGKNVADLSECPVFVMDEADKLLSPEFAPVMEQLLAYLPEKRQVMLFSATFPMIVKDFKEKHMV

SPYEINLMDELTLRGVTQYYAYVEERQKVHCLNTLFSKLQINQSIIFCNSTNRVELLAKKVTELGYSCFYSHAKMLQSHR

NRVFHDFRNGVCRNLVCSDLLTRGIDIQAVNVVINFDFPKNSETYLHRIGRSGRFGHLGLAINLVTYEDRFNLYRIEQEL

GTEIGPIPQTIDRGLYVDPGAIQEEGQPRQQQQQQQPQHPQHPVAGPARPPQQPNHHQQQQALLQQHAAALIQQQQQQQQ

QVLYQSNGNAPPVQRQPPHVGRSNGVPQGMHGGYRGGVPGGR

>XP_007301961.1 DEAD-domain-containing protein [Stereum hirsutum FP-91666 SS1]

MSQPATQQQATPSTSSSQNGNWRAGLRPPPRDDRPQTEDVKALKGIEFEDMFLRRELLMGIFEAGFERPSPIQEEAIPIA

LAKRDVLARAKNGTGKTAAFVIPSLQQVDVSRNKIQALLLVPTRELALQTSQVCKILGKHMGVQVMVTTGGTTLKDDIMR

LAETVHVLVGTPGRILDLAGKNVADLSECPVFVMDEADKLLSPEFSPVMEQLLAYLPKERQVMLFSATFPLIVKDFKDKH

MKSPYEINLMEELTLRGVTQYYAFVEERQKVHCLNTLFSKLQINQSIIFCNSTNRVELLAKKVTELGYSCFYSHAKMLQS

HRNRVFHDFRNGVCRNLVCSDLLTRGIDIQAVNVVINFDFPKNSETYLHRIGRSGRFGHLGLAINLVTYEDRFNLYKIEQ

ELGTEIQPIPQTIDKGLYVAPGATADETSRAGQKTGQPQAQQQQQPQPTAGPPQQQQQQGVLSPQQVAAQRAQAAAQAQA

QAQARQAAQQQQQQQAVYQTNGQAGRPNGQAVQAAGNRQSYPAGANAGYRGQVPVTQR

>VDB91318.1 unnamed protein product [Peniophora sp. CBMAI 1063]

MAQPAPSTSGSADGWRANLRPPPRDERPQTEDVQNIKGIEFEDMKLRRELLMGIFEAGFERPSPIQEEAIPAALSKRDIL

ARAKNGTGKTASFVVPSLEQVDVSKNKIQALLLVPTRELALQTSQVCKNLGKHMGVQVMVTTGGTTLKDDILRLSETVHV

LVGTPGRILDLAGKGVADLSECPVFVMDEADKLLSPEFAPVMEQLLAFLPKTRQVMLFSATFPIIVKDFKEKHMKNPYEI

NLMEELTLRGVTQYYAYVEERQKVHCLNTLFSKLQINQSIIFCNSTNRVELLAKKVTELGYSCFYSHAKMLQSHRNRVFH

DFRNGVCRNLVCSDLLTRGIDIQAVNVVINFDFPKNSETYLHRIGRSGRFGHLGLAINLVTYEDRFNLYKIEQELGTEIQ

PIPQVIDKGLYVAPSAQDEPAGQKPRQQQQTQQQQQVKQTVYQTNGAAAAPAPARNGGTPAGAR

>KDQ58786.1 hypothetical protein JAAARDRAFT_34642 [Jaapia argillacea MUCL 33604]

MSQQQSRASSSASASTQNDWKNGLRPPPKDFRPQTEDVTATKGIEFEDMYIRRELLMGIFEAGFEKPSPIQEEAIPIALT

KRDVLARAKNGTGKTAAFVIPSLQQIDVNKHKIQALILVPTRELALQTSQVCKVLGKHMGVQVMVTTGGTTLKDDILRLS

EVVHVLVATPGRVLDLAGKGVADLSECPVFVMDEADKLLSPEFTPVIEQLLGYLPKDRQVMLFSATFPMIVKDFKEKHMS

SPYEINLMEELTLRGVTQYYAFVEERQKVHCLNTLFSKLQINQSIIFCNSTNRVELLAKKITELGYSCFYSHAKMLQSHR

NRVFHDFRNGVCRNLVCSDLLTRGIDIQAVNVVINFDFPKNSETYLHRIGRSGRFGHLGLAINLVTYEDRFSLYKIEQEL

GTEIQPIPQFIDKALYVAPSGGSGEEQQPQQPKPQQSASATQKQLQQQSGGVVQQKQPQTQPPRQQQAPPPPPPLTVAAQ

QAQAGQVLYQSTIPPQQQPQQAMRPNGMAQVQPQLAAQRAYAGGPGPAGPGYNRGGVAVPR

>KAF7318873.1 hypothetical protein [Mycena chlorophos]

MAHPASSSNLANSAPASGESWKNGLRLPPKDLRPQTTDVTATKGTEFEDMFLRRELLMGIFEAGFEKPSPIQEEAIPIAL

TKRDILARAKNGTGKTAAFVIPSLQQVDVSKNKIQALLLVPTRELALQTSQVCKILGKHMGVQVMVTTGGTTLKDDIIRL

SETVHVLVGTPGRILDLAGKNVADLSECPVFVMDEADKLLSPEFAPVMEQLLAYLPDKRQVMLFSATFPMIVKDFKDKHM

VSPYEINLMDELTLRGVTQYYAYVEERQKVHCLNTLFSKLQINQSIIFCNSTNRVELLAKKVTELGYSCFYSHAKMLQSH

RNRVFHDFRNGVCRNLVCSDLLTRGIDIQAVNVVINFDFPKNSETYLHRIGRSGRFGHLGLAINLVTYEDRFNLYRIEQE

LGTEIGPIPQVIDRGLYVDPGAVQEEGGQQRNNNNAQQQQQQQAPVAGPARPPPQQQQHHHQQQQQQQMLQQQAAAMIQQ

QHQQQQQVLYQSNGAPPAQRQGPNVVMRTTGLPPPQQGIPQMQYRGGVPGGGR

>CEL57930.1 ATP-dependent RNA helicase DDX6/DHH1 [Rhizoctonia solani AG-1 IB]

MASATNNSSASNDDWKSGLRAPPKDDRPQTEDVTATKGLEFEGMGLRRELLMGIFEAGFERPSPIQEEAIPIALTRRDIL

ARAKNGTGKTAAFTIPSLQQVDPAKPKIQAMLLTPTRELALQTAQVCKNLGKHMGINVMVTTGGTTLKDDIIRLSEAVHV

LVGTPGRILDLAGKQVADLSQCRVFVMDEADKLLSPEFTPVMEQLLSFVPSDRQVMLFSATFPMIVKQFKDKHMKTPHEI

NLMDELTLRGVTQYYAFVEERQKVHCLNTLFAKLQINQSIIFCNSTNRVELLAKKVTELGYSCFYSHAKMVQSARNRVFH

DFRNGVCRNLVCSDLLTRGIDIQAVNVVINFDFPKHSETYLHRIGRSGRFGHLGLAINLVTYEDRFNLYRIEQELGTEIQ

PIPTEINKSLYVAPSVIDEPAGQVPSNRGSAPESNNRSGTNGQSVDGQQPRNGGTPMGQGRGQPRGGHRGGPNGRGAAPQ

R

>KIM70603.1 hypothetical protein SCLCIDRAFT_156787 [Scleroderma citrinum Foug A]

MSQTTRPSSSNSLSAANHKDLTWKAGLRPPLKDTRPQTEDVTATKGLEFEDMYLRRELLMGIFEAGFEKPSPIQEEAIPV

ALTNRDILARAKNGTGKTAAFVIPALQQIDINKPKIQALLLVPTRELALQTSQVCKILGKHMGVQVMVTTGGTTLKDDIM

RLSETVHVLVGTPGRILDLAGKSIADLSECPIFVMDEADKLLSPEFTPVMEQLLSFLPNDRQVMLFSATFPLLVKDFKEK

HMNSPYEINLMEELTLRGVTQYYAYVEERQKVHCLNTLFSKLQINQSIIFCNSTNRVELLAKKITELGYSCFYSHAKMLQ

SHRNRVFHDFRNGVCRNLVCSDLLTRGIDIQAVNVVINFDFPKNSETYLHRIGRSGRFGHLGLAINLVTYEDRFNLYRIE

QELGTEIQPIPQYIDKSLYVAPSAIEEPAPQKGQAQQQQQQRQHTSAQQSHTPQGNGRPNGVQPPAQPAQSQARASYPAQ

PTYRATPVAR

>KNE99579.1 ATP-dependent RNA helicase dhh1 [Puccinia striiformis f. sp. tritici PST-78]

MAASTSSASDLSHDPDWKSKLNLPQKDLRPQTEDVTKTKGIEFEDLYLRRDLLMGIFEAGFEKPSPIQEEAIPIALAGRD

ILARAKNGTGKTGAFVIPTLEKTNVRVNRIQALILVPTRELALQTSQVCKTLGKHTGIQIMVTTGGTTLKDDILRLQEAV

HVVVGTPGRILDLASKGVADLSQCKTFVMDEADKLLSPEFTVVIEQLLSFLHKERQVMLFSATFPMIVKDFKDKHMIKPY

EINLMEELTLQGVTQYYAFLEERQKVHCLNTLFSKLQINQAIIFCNSTNRVELLARKITDIGYSCFYSHAKMLQQHRNRV

FHDFRNGVCRNLVCSDLLTRGIDIQAVNVVINFDFPKNAETYLHRIGRSGRFGHRGIAINLITYENRFDLYRIEQELGTE

IQPIPAVIDKALYVAPGIEDTPSSAPTHANPSAAASNANQANNSNQSQQSNQTHRNGRQANHQPTDQSHVPQPHDKPTLP

PQQLTFQKAAPIDSLSHATPTEYANGAPPSNNSRMNHKSTRGGGGPHRGRGGGAHRAPSGIAPAA

>PLW16706.1 hypothetical protein PCASD_18473 [Puccinia coronata var. avenae f. sp. avenae]

MAASSSSASDLSHDPDWKSKLSLPQKDLRPQTEDVTKTKGIEFEDLYLRRDLLMGIFEAGFEKPSPIQEEAIPIALAGRD

ILARAKNGTGKTGAFVIPTLEKTNVRINRIQALILVPTRELALQTSQVCKTLGKHTGIQIMVTTGGTTLKDDILRLQEAV

HVVVGTPGRILDLASKGVADLSQCKTFVMDEADKLLSPEFTVVIEQLLSFLHKERQVMLFSATFPMIVKDFKDKHMIKPY

EINLMEELTLQGVTQYYAFLEERQKVHCLNTLFSKLQINQAIIFCNSTNRVELLARKITDIGYSCFYSHAKMLQQHRNRV

FHDFRNGVCRNLVCSDLLTRGIDIQAVNVVINFDFPKNAETYLHRIGRSGRFGHRGIAINLITYENRFDLYRIEQELGTE

IQPIPAVIDKALYVAPGIEDTPSSTPTQPNSSVSNASQTAHSNQGQQSNQANRNGRQTNQQHSDHSHVPQPHDKPTLPPQ

QLTFQKAAPIDSLSHATPTEYANGAPPPNSSRMNHKSTRGGSGGPHRGRGGAGGTHRAPSGIAPAA

>GAT48069.1 predicted protein [Mycena chlorophos]

MAHPASSSNPANSTPASGESWKNGLRLPPKDLRPQTTDVTATKGTEFEDMFLRRELLMGIFEAGFEKPSPIQEEAIPIAL

TKRDILARAKNGTGKTAAFVIPSLQQVDVSKNKIQALLLVPTRELALQTSQVCKILGKHMGVQVMVTTGGTTLKDDIIRL

SETVHVLVGTPGRILDLAGKNVADLSECPVFVMDEADKLLSPEFAPVMEQLLAYLPDKRQVMLFSATFPMIVKDFKDKHM

VSPYEINLMDELTLRGVTQYYAYVEERQKVHCLNTLFSKLQINQSIIFCNSTNRVELLAKKVTELGYSCFYSHAKMLQSH

RNRVFHDFRNGVCRNLVCSDLLTRGIDIQAVNVVINFDFPKNSETYLHRIGRSGRFGHLGLAINLVTYEDRFNLYRIEQE

LGTEIGPIPQVIDRGLYVDPGAVQEEGGQQRNNNNNNNNNAQQQQQQQAPVAGPARPPQQQQHHHQQQQQQMLQQQAAAM

IQQQHQQQQQVLYQSNGAPPAQRQGPNVVMRTTGLPPPQQGIPQMQYRGGVPGGGR

>XP_006463363.1 hypothetical protein AGABI2DRAFT_194203 [Agaricus bisporus var. bisporus H97]

MSQPPPSSSASSSSTPHDSAWKTGLRPPPRDSRPQTEDVTATKGLEFEDMSIRRELLMGIFEAGFEKPSPIQEEAIPFAL

TKRDILARAKNGTGKTAAFVIPSLQQIDINKHKIQALLLVPTRELALQTSQVCKTLGKHMGIQVMVTTGGTTLKDDILRL

SESVHVLVGTPGRILDLAGKGVADLSECPVFVMDEADKLLSPEFAPVMEQLLSYLPSERQVMLFSATFPMIVKTFKDKHM

RSPYEINLMDELTLRGVTQYYAYVEERQKVHCLNTLFSKLQINQSIIFCNSTNRVELLAKKVTELGYSCFYSHAKMLQSH

RNRVFHDFRNGVCRNLVCSDLLTRGIDIQAVNVVINFDFPKNSETYLHRIGRSGRFGHLGLAINLVTYEDRFNLYKIEQE

LGTEIQPIPQTIDRTLYVAPGSSEEQHQKPQPQSQQPQQRQAAPSPSPAAAAAPRAGQPSLPAPQSISQQPAFNGQASAR

ANGGLPPQAQQMRGQPTYRGGVPVAR

>XP_003027845.1 uncharacterized protein SCHCODRAFT_70659 [Schizophyllum commune H4-8]

MSQATSSSTTPSNNDWKSGLRAPPKDLRPQTEDVLATKGTEFENMYLRRELLMGIFEAGFEKPSPIQEEAIPKALQRRDI

LARAKNGTGKTAAFVIPTLQQVDPTKNKIQALLLVPTRELALQTSQVCKILGKHMGIQVMVTTGGTTLKDDILRLNETVH

VLVGTPGRILDLAGKNVADLSQCPVFVMDEADKLLSPEFAPVMEQLLSYLPNDRQVMLFSATFPMIVKDFKEKHMNNPYE

INLMEELTLRGVTQYYAYVEERQKVHCLNTLFSKLQINQSIIFCNSTNRVELLAKKITELGYSCFYSHAKMLQSHRNRVF

HDFRNGQCRNLVCSDLLTRGIDIQAVNVVINFDFPKNSETYLHRIGRSGRFGHLGLAINLVTYEDRFNLYKIEQELGTEI

MPIPQTIDRGLYVAPTGASEEQQQQQKRAQAANGVQRNGTPQQRPPQRAPVPAGSGAAR

>RKO86773.1 P-loop containing nucleoside triphosphate hydrolase protein [Blyttiomyces helicus]

MAAAYMITNHENPDWKRTLNLPAKDTRPQTEDVTATKGNEFEDYFLKRELLMGIFEAGFERPSPIQEESIPIALAGRDIL

ARAKNGTGKTAAFTIPVLEKTNITKNEIQALLLVPTRELALQTSQVCKNLGKHMGVQVMVTTGGTTLKDDIMRLGQTVHI

LVGTPGRVLDLAGKGVADLSKCTTLVMDEADKLLSPEFQPIIEQLISFLKNERQILLYSATFPMIVKSFTDRYLTKPYEI

NLMEDALTLRGITQFYAYVEERQKVHCLNTLFSKLQINQSIIFCNSTSRVELLAKKITELGYSCFYIHAKMLQAHRNRVF

HDFRNGKTRHLVCSDLLTRGIDIQAVNVVINFDFPKNAETYLHRIGRSGRFGHLGLAINLITYDDRFNLYKIEQELGTEI

APIPPIIDKSLYVAP

>XP_007404630.1 uncharacterized protein MELLADRAFT_46659 [Melampsora larici-populina 98AG31]

MATSTNPHPGDQDWKSKLNLPQKDLRPQTEDVTKTKGVEFEDLYLRRDLLMGIFEAGFERPSPIQEEAIPIALAGRDILA

RAKNGTGKTGAFVIPSLEKTNVKLNRIQALILVPTRELALQTSQVCKTLGKHTGIQIMVTTGGTTLKDDILRLQETVHIV

VGTPGRILDLAGKGVADLSQCKTFVMDEADKLLSPEFTVVIEQLLSFLHKERQVMLFSATFPMIVKDFKDKHMVKPYEIN

LMEELTLQGVTQYYAFLEERQKVHCLNTLFSKLQINQAIIFCNSTNRVELLARKITDIGYSCFYSHAKMLQQHRNRVFHD

FRNGVCRNLVCSDLLTRGIDIQAVNVVINFDFPKNAETYLHRIGRSGRFGHRGIAINLITYENRFDLYRIEQELGTEIQP

IPAVIDKALYVAPGIEDTPAPTSKRQQQSASSTNAAQHANANTNNRGPQDSRNNRQANQSQPSSNPSQPHDKPTLPPQQL

TFQKAAPIDSLSHATPTEYASGLPPSHNRNNHHKVGRGGGGGHRGRGSGSGHRHPSGATPVA

>XP_018290498.1 hypothetical protein PHYBLDRAFT_134291 [Phycomyces blakesleeanus NRRL 1555(-)]

MSHPSTSQPLSHETDDWKKSITLPAKDTRPQTEDVTATKGNEFEDYFLKRELLMGIFEAGFERPSPIQEEAIPIALTGRD

VLARAKNGTGKTAAFVIPTLEKINVKKPKVQALLLVPTRELALQTSQVCKNLGKHLDVQVMVTTGGTTLKDDIMRLSEPV

HVIVGTPGRLLDLASKGVADFSEATTFVMDEADKLLSPEFTPIIEQLISFFPKDRQIMLFSATFPLVVKHFKEKFLIKPY

EINLMDELTLRGVTQYYAYVEEKQKVHCLNTLFSKLQINQSIIFCNSTNRVELLAKKITELGYSCFYSHAKMLQSHRNRV

FHDFRNGVCRNLVCSDLLTRGIDIQAVNVVINFDFPKNAETYLHRIGRSGRFGHLGLAINLITYEDRFNLYKIERELGTE

IQPIPPIIDKRLYVAPSSLEDAQVQQPDVEAAAATARQQRQQAGLPHEEYGRQQGHWRQNGRNNPNGGGGRRVSDGGGGG

GGGGGGGGGVNGNGGRRAPNPMSTARPKPKPKPF

>OMJ16213.1 ATP-dependent RNA helicase dhh1 [Smittium culicis]

MADSKNNSSLDNSNDWKSQLDLPPKDTRLQTEDVTNTKGNEFEDYYLKRELLMGIFEAGFEKPSPIQEESIPIALTGRDI

LARAKNGTGKTAAFVIPALEKINPSSPSLQTLILVPTRELALQTSQVCKTIGKHLGINIMVTTGGTTLKDDIMRLSETVH

ILVGTPGRVLDLACKNVIDFSKAETFIMDEADKLLSPEFTPVIEQLLGFFPPRRQILLYSATFPLVVKTFKDKNMVKPYE

INLMDELTLRGVTQYYAFVEEKQKVHCLNTLFSKLQINQSIIFCNSTNRVELLARKITELGYSCFYSHARMLQNHRNRVF

HDFRNGSCRNLVCSDLLTRGIDIQAVNVVINFDFPRNAETYLHRIGRSGRFGHLGLAINLVTYEDRFNLYKIEQELGTEI

QPIPPIIDKRLYVAPSAIDEYPNQLLQQEQRQQQHLQHQQMQQRKMNPNFNRNYNNNNNNNNNNSYPNQRQYPNNNQNNG

QGFNNGQSLNNGNQYNNRNNYQNNRNRQNNNNAEQNPDQNQKNNQNSNKSNYNAPPGYQNRNQNDSNRNYRQYRQNNNQN

NNV

>KAF2234193.1 ATP-dependent RNA helicase DHH1 [Viridothelium virens]

MAVETDITNQLQATKLDDTSDTNWRQGLKAPPKDTRVQTEDVTNTKGLEFEDFYIKRELMMGIFEAGYEKPSPIQEAAIP

VALTGRDILARAKNGTGKTAAYVIPTLERINNKNTKIQALILVPTRELALQTSQVFRTLGKHIGVKVMVTTGGTGLKDDI

IRLNEAVHIVVGTPGRILDLASKQVADLSACQTFVMDEADKLLSPEFTPVIEQLLGFHPKDRQVMLFSATFPLVVKSFKD

KHMKSPYEINLMDELTLRGITQYYAFVEEKQKVHCLNTLFSKLQINQSIIFCNSTQRVELLAKKITELGYSCFYSHAKMQ

QNDRNRVFHDFRNGACRNLVCSDLLTRGIDIQAVNVVINFDFPKNAETYLHRIGRSGRFGHLGLAINLINWDDRFNLYKI

EQELGTEIQAIPPVIEKSLYVYESPENIPRPPSNAPQRQIQKSTTTQNSNRQNYNRNQRGGAHFQGQRRGPPPGQPSQNR

QNPVPQQRAAGSAAQA

>XP_001832628.1 ATP-dependent RNA helicase dhh1 [Coprinopsis cinerea okayama7#130]

MAQVAPASSSTSSTSDNWKTGLKPPPKDLRPQTEDVTATKGLEFEDMRLGRELLMGIFEAGFEKPSPIQEEAIPVALTKQ

DILARAKNGTGKTAAFVIPSLQQIDVAKPKIQALLLVPTRELALQTAQVCKILGKHMGVQVMVTTGGTTLKDDIIRLSDT

VHVLVGTPGRILDLAGKNVADLSECPVFVMDEADKLLSPEFAPVMEQLLSFLPSERQVMLFSATFPMIVKDFKDKHMRSP

YEINLMEELTLRGVTQYYAYVEERQKVHCLNTLFSKLQINQSIIFCNSTNRVELLAKKITELGYSCFYSHAKMLQSHRNR

VFHDFRNGVCRNLVCSDLLTRGIDIQAVNVVINFDFPKNSETYLHRIGRSGRFGHLGLAINLVTYEDRFNLYKIEQELGT

EIQPIPQSIDRSLYVAPNATEDQKQKPFQHQSQQQQQMQQRQVAGPAPPRNGQVPAQQHPQQQQQQQQQQQQQVAYNQGM

QQRQNGQPPRVQQQQQQQPAYRGGVPVGR

>TIB18941.1 hypothetical protein E3P92_00289 [Wallemia ichthyophaga]

MMDPFSLGVNLSPVVLKTLVRHLIKKRNNNTNKSETPSTPNDEYAFDQTFKLTRAFLDIAMNHPVEDLQSLGSTYIPPPF

WVHNVAVTVPMSIINNAAEYLIKAFGGEQPMRAAVGGTRWWQVRPTPGLPSEWICMKSDVKLATRNNKPNKPREQPKDSF

TPQFFKDIEGLPRTILFVHGGAYYWGSINTHRYLIWRMARKMRGKAFAVSYRLSPQYGFPCALQDVLSAYLYLLYPPEGA

PHKAVDPSNLIFAGDSAGGGLLLSLLLIIRDLGLPLPAGSIHISPWCDLSHSFPSVLSNYETDVIPKYGFVHKPSTLWPP

PSEELLQRTRQHIKSSTQRSKSPLRGVGSAASRQAEEGRIEEDAINEAAGMTNERGHEKLEPPRDVPTVDLDGRTVELKG

QIQMYATNEQVGHPYVSPVLAHLGGLCPLFIMASDGELLRDEGIYIAHKAARPDDFEYKEDAKRRLPSMNGIEGRFGPTK

VHLQVYDKMCHDLPLFSMVTPAKYAYRAMAAFCFQVTKDTTVQPSPALQGSDIPNTMSDLELDSAAESPSPLRKLDSPAE

APSPSTKLDSPADVPSPSRKIDNPAIALLPPPPSPGKPKRKFSLLGLKNNEGAGATSIDMEKAATVSSQTGTPASDAERP

IKSGPGTAGNMAVYDESMAFKERNMIRERVSLVGQIRPMESSENMDILRIPADEVGVFKPAAVKRYLEGKNKWMHKFKHT

AKNVERQREKSLKESNIHAEKKIKDSDNIFDTPQVTWRWQWALENERPPPASIVARRDTQEARKLVQIVDNADSKQNSRT

LSLWTLVMHLMSPQKSKASTNDDGDDVAPWTKDIEKLKRKDGKKDEGQTVSESDSPEEEEINIDKRKEQWLKNHVKDEYW

LWLSDDTLDKRSGKWIVESAFHIYHHEWLQLRKRRRQEISNTAHKAHPKTSAESEWKNQLRVPQKDLRPQTEDVTKTKGG

EWEDFGLRRELLMGIFEAGFEKPSPIQEEAIPSAIAGRDILARAKNGTGKTASFVIPSLEKINVQKPKIQALLLVPTREL

ALQTSQVCKSLGKHLGIQVMVTTGGTTLRDDIMRLSDPVHILVGTPGRILDLAGKGVANLNECPTFVMDEADKLLSPEFT

PVMEQLLSHLPSSRQVMLFSATFPLIVKDFKEKHMKDPHEINLMDELTLRGITQYYAFVDERQKVHCLNTLFSKLQINQS

IIFCNSTNRVELLAKKVTELGYSCFFSHAKMLQSHRNRVFHDFRSGVCRNLVCSDLLTRGIDIQAVNVVINFDFPKNSET

YLHRIGRSGRYGHLGLAINLITYEDRFNLYKIEQELGTEIQPIPSQIDRSLYVAPNASDDDGQIQQSQQQQKGLPTLPPT

QQVMNAYSQPPPQPTFNQSNQNHQYQQRSNRGGYNSGYYGGSGFRGRGGGPPRGGVPRA

>PVU95272.1 hypothetical protein BB561_001915 [Smittium simulii]

MAQINTESQFSNDQDWKRNLALPPKDTRFQTEDVTATKGNEFEDYYLKRELLMGIFEAGFEKPSPIQEESIPIALTGRDI

LARAKNGTGKTAAFTIPALEKIKTDSSYVQCLILVPTRELALQTSQVCKTLGKHLGINIMATTGGTTLKDDIMRLSETVH

IIVGTPGRILDLANKGVVKLNNTETFIMDEADKLLSPEFTPVIEQLISFLPKKRQILLYSATFPIAVKSFKDKHMIKPYE

INLMDELTLRGVTQYYAFVEEKQKVHCLNTLFSKLQINQSIIFCNSTNRVELLARKITELGYSCFYSHARMLQPDRNRVF

HDFRNGTCRNLVCTDLLTRGIDIQAVNVVINFDFPRNAETYLHRIGRSGRFGHLGIAINLITYEDRFNIYKIEQELGTEI

QPIPSTIDKRLYVAPSAVDDYPNQLLHQHQKNLQLQQQQQMQQHSYKKSSGNHYNYKNNNYRPNNYRSNYNRNNNPNNNS

NDNNVNGNNDNNNDNTNKNQFNNSSNRTSNQNEPNGHRNRQNNNRNYNPNSSYNKQ

>TIA89622.1 hypothetical protein E3P97_02921 [Wallemia ichthyophaga]

MMDPFSLGVNLSPVVLKTLVRHLIKKRYNNTNKSETPSTPNDEYAFDQTFKLTRAFLDIAMNHPVEDLQSLGSSYIPPPF

WVHNVAVTVPMSIINNAAEYLIKAFGGEQPMRAAVGGTRWWQVRPTPGLPSEWICMKSDVKLATRNNKPNKPREQPKDSF

TPQFFKDIEGLPRTILFVHGGAYYWGSINTHRYLIWRMARKMRGKAFAVSYRLSPQYGFPCALQDVLSAYLYLLYPPEGA

PHKAVDPSNLIFAGDSAGGGLLLSLLLIIRDLGLPLPAGSIHISPWCDLSHSFPSVLSNYETDVIPKYGFVHKPSTLWPP

PSEELLQRTRQHIKSSTQRSKSPLRGVGSAASRKAEEGRIEEDAINEAAGMTNERGHEKLEPPRDVPTVDLDGRTVELKG

QIQMYATNEQVGHPYVSPVLAHLGGLCPLFIMASDGELLRDEGIYIAHKAARPDDFEYKEDAKRRLPSMNGIEGRFGPTK

VHLQVYDKMCHDLPLFSMVTPAKYAYRAMAAFCFQVTKDTTVQPSPALQGSDIPNTMSDLELDSAAESPSPLRKLDSPAE

APSPSTKLDSPADVPSPSRKIDNPAIALSPPPPSPGKPKRKFSLLGLKNNEGAGATSIDMEKAATVSSQTGTPASDAERP

IKSGPGTAGNMAVYDESMAFKERNMIRERVSLVGQIRPMESSENMDILRIPADEVGVFKPAAVKRYLEGKNKWMHKFKHT

AKNVERQREKSLKESNIHAEKKIKDSDNIFDTPQVTWRWQWALENERPPPASIVARRDTQEARKLVQIVDNADSKQNSRT

LSLWTLVMHLMSPQKSKASTNDDGDDVAPWTKDIEKLKRKDGKKDEAQTVSESDSPEEEEINIDKRKEQWLKNHVKDEYW

LWLSDDTLDKRSGKWIVESAFHIYHHEWLQLRKRRRQEISNTAHKAHPKTSAESEWKNQLRVPQKDLRPQTEDVTKTKGG

EWEDFGLRRELLMGIFEAGFEKPSPIQEEAIPSAIAGRDILARAKNGTGKTASFVIPSLEKINVQKPKIQALLLVPTREL

ALQTSQVCKSLGKHLGIQVMVTTGGTTLRDDIMRLSDPVHILVGTPGRILDLAGKGVANLNECPTFVMDEADKLLSPEFT

PVMEQLLSHLPSSRQVMLFSATFPLIVKDFKEKHMKDPHEINLMDELTLRGITQYYAFVDERQKVHCLNTLFSKLQINQS

IIFCNSTNRVELLAKKVTELGYSCFFSHAKMLQSHRNRVFHDFRSGVCRNLVCSDLLTRGIDIQAVNVVINFDFPKNSET

YLHRIGRSGRYGHLGLAINLITYEDRFNLYKIEQELGTEIQPIPSQIDRSLYVAPNASDDDGQIQQSQQQKGLPTLPPTQ

QVMNAYSQPPPQPTFNQSNQNHQYQQRSNRGGYNSGYYGGSGFRGRGGGPPRGGVPRA

>TIB65366.1 hypothetical protein E3P77_02705 [Wallemia ichthyophaga]

MMDPFSLGVNLSPVVLKTLVRHLIKKRNNNTNNSETPSTPNDEYAFDQTFKLTRAFLDIAMNHPVEDLQSLGSTYIPPPF

WVHNVAVTVPMSIINNAAEYLIKAFGGEQPMRAAVGGTRWWQVRPTPGLPSEWICMKSDVKLATRNNKPNKPREQPKDSF

TPQFFKDIEGLPRTILFVHGGAYYWGSINTHRYLIWRMARKMRGKAFAVSYRLSPQYGFPCALQDVLSAYLYLLYPPEGA

PHKAVDPSNLIFAGDSAGGGLLLSLLLIIRDLGLPLPAGSIHISPWCDLSHSFPSVLSNYETDVIPKYGFVHKPSTLWPP

PSEELLQRTRQHIKSSTQRSKSPLRGVGSAASRKAEEGRIEEDAINEAAGMTNERGHEKLEPPRDVPTVDLDGRTVELKG

QIQMYATNEQVGHPYVSPVLAHLGGLCPLFIMASDGELLRDEGIYIAHKAARPDDFEYKEDAKRRLPSMNGIEGRFGPTK

VHLQVYDKMCHDLPLFSMVTPAKYAYRAMAAFCFQVTKDTTVQPSPALQGSDIPNTMSDLELDSAAESPSPLRKLDSPAE

APSPSTKLDSPADVPSPSRKIDNPAIALLPPPPSPGKPKRKFSLLGLKNNEGAGATSIDMEKAATVSSQTGTPASDAERP

IKSGPGTAGNMAVYDESMAFKERNMIRERVSLVGQIRPMESSENMDILRIPADEVGVFKPAAVKRYLEGKNKWMHKFKHT

AKNVERQREKSLKESNIHAEKKIKDSDNIFDTPQVTWRWQWALENERPPPASIVARRDTQEARKLVQIVDNADSKQNSRT

LSLWTLVMHLMSPQKSKASTNDDGDDVAPWTKDIEKLKRKDGKKDEGQTVSESDSPEEEEINIDKRKEQWLKNHVKDEYW

LWLSDDTLDKRSGKWIVESAFHIYHHEWLQLRKRRRQEISNTAHKAHPKTSAESEWKNQLRVPQKDLRPQTEDVTKTKGG

EWEDFGLRRELLMGIFEAGFEKPSPIQEEAIPSAIAGRDILARAKNGTGKTASFVIPSLEKINVQKPKIQALLLVPTREL

ALQTSQVCKSLGKHLGIQVMVTTGGTTLRDDIMRLSDPVHILVGTPGRILDLAGKGVANLNECPTFVMDEADKLLSPEFT

PVMEQLLSHLPSSRQVMLFSATFPLIVKDFKEKHMKDPHEINLMDELTLRGITQYYAFVDERQKVHCLNTLFSKLQINQS

IIFCNSTNRVELLAKKVTELGYSCFFSHAKMLQSHRNRVFHDFRSGVCRNLVCSDLLTRGIDIQAVNVVINFDFPKNSET

YLHRIGRSGRYGHLGLAINLITYEDRFNLYKIEQELGTEIQPIPSQIDRSLYVAPNASDDDGQIQQSQQQQKGLPTLPPT

QQVMNAYSQPPPQPTFNQSNQNHQYQQRSNRGGYNSGYYGGSGFRGRGGGPPRGGVPRA

>GAA99335.1 hypothetical protein E5Q_06030 [Mixia osmundae IAM 14324]

MADVAMETMQPAPAPAAVKESAPPAAAEPRRVRFNVGSKYQVREIIGEGAYGVVCSAYHKPTGTRVAIKKIQPFDHTMFA

LRTLRELKLLKYFQEQNVSENIISIIDIIKPSTIDSFKEVYIIQELMETDLHRVIRTQDLSMSHIEYLTYQCLRALKACH

SADIIHRDLKPSNLLLNSNCDLKVCDFGLARSIQTAQPAANSSTGFMTEYVATRWYRAPEIMLTFKQYTKAIDIWSVGCI

VAEMLSGRPLFPGRDYHHQLTLILDVLGTPPLDEFYAINSRRSRDYLRALPFRKKRSFESLFPNAPPEAIDFLSRSLTFS

PTKRMTVEEALAHPFMSAYHDPEDEPSAPPLPPHFFHFDHVKDEITKDALKQLLYDERRRKTDRLHQLQRERHTSPTKHR

VRATPDDTSTPSDCQGRPAAVIMASASTSRAAANPEGQWKEKLNLPAKDNRPQTEDVLRTKGNEFEDYFLKRELLMGIFE

AGFEKPSPIQEEAIPIALAGRDILARAKNGTGKTGSFVIPALEKVNPKVNKIQALILVPTRELALQTSQVCKTLGKHLGV

QIMVTTGGTSLKDDILRLQETVHIVVGTPGRVLDLASKGIADVSACPTFVMDEADKLLSPEFTLVIEQILSLLPKDRQVM

LFSATFPLLVKDFKDKHMTKPYEINLMDELTLRGVTQYYAFLEERQKVHCLNTLFSKLQINQSIIFTNSTSRVELLAKKI

TELGYSCFYSHAKMLQADRNRVFHDFRSGKCRNLVCSDLLTRGIDVPSVNVVINFDFPKQSESYLHRIGRSGRFGHLGLA

INLITYEDRFNLYRIEQELGTEILPIPPTIDRSLYVAPGSGDHPAERKPQQPRNASQQQPQQTAQRVQGSSQPSYASNHA

PNSTNRPIIPPQQSMVQAQNPINEISHAKPTHYANGAPPNGRQRAGAGRGQQPDGVSRSSAARESATTIKEAPKDKIIIY

RQQLRQAVSIRSGIRCVNSGSNREDLAGRSHLCSHRSAQPLWPITMLPESAMARGRRHHPRASFNACSLSSMLGVEATLI

ILQ

>OMJ28924.1 ATP-dependent RNA helicase DHH1 [Smittium culicis]

MADSKNNSSLDNSNDWKSQLDLPPKDTRLQTEDVTNTKGNEFEDYYLKRELLMGIFEAGFEKPSPIQEESIPIALTGRDI

LARAKNGTGKTAAFVIPALEKINPSSPSLQTLILVPTRELALQTSQVCKTIGKHLGINIMVTTGGTTLKDDIMRLSETVH

ILVGTPGRVLDLACKSVIDFSKAETFIMDEADKLLSPEFTPVIEQLLGFFPPRRQILLYSATFPLVVKTFKDKNMVKPYE

INLMDELTLRGVTQYYAFVEEKQKVHCLNTLFSKLQINQSIIFCNSTNRVELLARKITELGYSCFYSHARMLQNHRNRVF

HDFRNGSCRNLVCSDLLTRGIDIQAVNVVINFDFPRNAETYLHRIGRSGRFGHLGLAINLVTYEDRFNLYKIEQELGTEI

QPIPPIIDKRLYVAPSAIDEYPNQLLQQEQRQQQHLQHQQMQQRKMNPNFNRNYNNNNNNNNNNYPNQRQYPNNNQNNGQ

GYNNGQNMNNGNQYNNRNNYQNNRNQQSNNNAEQNPDQNQNQNQKNNQNSNKNNFNAPPGYQNRNQNDSNRNYRQYRQNN

NQNSNV

>TIB37551.1 hypothetical protein E3P84_00170 [Wallemia ichthyophaga]

MMDPFSLGVNLSPVVLKTLVRHLIKKRNNNTNKSETPSTPNDEYAFDQTFKLTRAFLDIAMNHPVEDLQSLGSTYIPPPF

WVHNVAVTVPMSIINNAAEYLIKAFGGEQPMRAAVGGTRWWQVRPTPGLPSEWICMKSDVKLATRNNKPNKPREQPKDSF

TPQFFKDIEGLPRTILFVHGGAYYWGSINTHRYLIWRMARKMRGKAFAVSYRLSPQYGFPCALQDVLSAYLYLLYPPEGA

PHKAVDPSNLIFAGDSAGGGLLLSLLLIIRDLGLPLPAGSIHISPWCDLSHSFPSVLSNYETDVIPKYGFVHKPSTLWPP

PSEELLQRTRQHIKSSTQRSKSPLRGVGSAASRKAEEGRIEEDAINEAAGMTNERGHEKLEPPRDVPTVDLDGRTVELKG

QIQMYATNEQVGHPYVSPVLAHLGGLCPLFIMASDGELLRDEGIYIAHKAARPDDFEYKEDAKRRLPSMNGIEGRFGPTK

VHLQVYDKMCHDLPLFSMVTPAKYAYRAMAAFCFQVTKDTTVQPSPALQGSDIPNTMSDLELDSAAESPSPLRKLDSPAE

APSPSTKLDSPADVPSPSRKIDNPAIALLPPPPSPGKPKRKFSLLGLKNNEGAGATSIDMEKAATVSSQTGTPASDAERP

IKSGPGTAGNMAVYDESMAFKERNMIRERVSLVGQIRPMESSENMDILRMPADEVGVFKPAAVKRYLEGKNKWMHKFKHT

AKNVERQREKSLKESNIHAEKKIKDSDNIFDTPQVTWRWQWALENERPPPASIVARRDTQEARKLVQIVDNADSKQNSRT

LSLWTLVMHLMSPQKSKASTNDDGDDVAPWTKDIEKLKRKDGKKDEGQTVSESDSPEEEEINIDKRKEQWLKNHVKDEYW

LWLSDDTLDKRSGKWIVESAFHIYHHEWLQLRKRRRQEISNTAHKAHPKTSAESEWKNQLRVPQKDLRPQTEDVTKTKGG

EWEDFGLRRELLMGIFEAGFEKPSPIQEEAIPSAIAGRDILARAKNGTGKTASFVIPSLEKINVQKPKIQALLLVPTREL

ALQTSQVCKSLGKHLGIQVMVTTGGTTLRDDIMRLSDPVHILVGTPGRILDLAGKGVANLNECPTFVMDEADKLLSPEFT

PVMEQLLSHLPSSRQVMLFSATFPLIVKDFKEKHMKDPHEINLMDELTLRGITQYYAFVDERQKVHCLNTLFSKLQINQS

IIFCNSTNRVELLAKKVTELGYSCFFSHAKMLQSHRNRVFHDFRSGVCRNLVCSDLLTRGIDIQAVNVVINFDFPKNSET

YLHRIGRSGRYGHLGLAINLITYEDRFNLYKIEQELGTEIQPIPSQIDRSLYVAPNASDDDGQIQQSQQQQKGLPTLPPT

QQVMNAYSQPPPQPTFNQSNQNHQYQQRSNRGGYNSGYYGGSGFRGRGGGPPRGGVPRA

>KXS19389.1 DEAD-domain-containing protein [Gonapodya prolifera JEL478]

MASSSAVGPMISATNGLAHQLQNTHISDPPANWKSTLALPPPDLRPKTEDVTRTKGNEFEDYQLKRELLMGIFEAGFERP

SPIQEESIPIALLGKDILARAKNGTGKTAAFTIPILEKVNTRVNKVQAVILVPTRELALQTSQVAKTLGKHLPNLQVMVT

TGGTTLKDDILRLGETVHILVGTPGRLLDLASKNVANLSTASILVMDEADKLLSPEFQPIIEQLISFMPKQRQILLYSAT

FPMIVKAFKDKFLSKPYEINLMDELTLKGVTQYYAYVDERQKVHCLNTLFSKLQINQSIIFCNSTSRVELLARKITELGY

SCYYIHAKMVQAHRNRVFHDFRQGKCRNLVCSDLLTRGIDIQAVNVVINFDFPKNAETYLHRIGRSGRFGHLGLAINLIT

YDDRFNLYKIEQELGTEIQPIPPVIDKQLYT

>TIB03833.1 hypothetical protein E3P95_00355 [Wallemia ichthyophaga]

MMDPFSLGVNLSPVVLKTLVRHLIKKRNNNTNKSETPSTPNDEYAFDQTFKLTRAFLDIAMNHPVEDLQSLGSTYIPPPF

WVHNVAVTVPMSIINNAAEYLIKAFGGEQPMRAAVGGTRWWQVRPTPGLPSEWICMKSDVKLATRNNKPNKPREQPKDSF

TPQFFKDIEGLPRTILFVHGGAYYWGSINTHRYLIWRMARKMRGKAFAVSYRLSPQYGFPCALQDVLSAYLYLLYPPEGA

PHKAVDPSNLIFAGDSAGGGLLLSLLLIIRDLGLPLPAGSIHISPWCDLSHSFPSVLSNYETDVIPKYGFVHKPSTLWPP

PSEELLQRTRQHIKSSTQRSKSPLRGVGSAASRKAEEGRIEEDAINEAAGMTNERGHEKLEPPRDVPTVDLDGRTVELKG

QIQMYATNEQVGHPYVSPVLAHLGGLCPLFIMASDGELLRDEGIYIAHKAARPDDFEYKEDAKRRLPSMNGIEGRFGPTK

VHLQVYDKMCHDLPLFSMVTPAKYAYRAMAAFCFQVTKDTTVQPSPALQGSDIPNTMSDLELDSAAESPSPLRKLDSPAE

APSPSTKLDSPADVPSPSRKIDNPAIALLPPPPSPGKPKRKFSLLGLKNNEGAGATSIDMEKAATVSSQTGTPASDAERP

IKSGPGTAGNMAVYDESMAFKERNMIRERVSLVGQIRPMESSENMDILRIPADEVGVFKPAAVKRYLEGKNKWMHKFKHT

AKNVERQREKSLKESNIHAEKKIKDSDNIFDTPQVTWRWQWALENERPPPASIVARRDTQEARKLVQIVDNADSKQNSRT

LSLWTLVMHLMSPQKSKASTNDDGDDVAPWTKDIEKLKRKDGKKDEGQTVSESDSPEEEEINIDKRKEQWLKNHVKDEYW

LWLSDDTLDKRSGKWIVESAFHIYHHEWLQLRKRRRQEISNTAHKAHPKTSAESEWKNQLRVPQKDLRPQTEDVTKTKGG

EWEDFGLRRELLMGIFEAGFEKPSPIQEEAIPSAIAGRDILARAKNGTGKTASFVIPSLEKINVQKPKIQALLLVPTREL

ALQTSQVCKSLGKHLGIQVMVTTGGTTLRDDIMRLSDPVHILVGTPGRILDLAGKGVANLNECPTFVMDEADKLLSPEFT

PVMEQLLSHLPSSRQVMLFSATFPLIVKDFKEKHMKDPHEINLMDELTLRGITQYYAFVDERQKVHCLNTLFSKLQINQS

IIFCNSTNRVELLAKKVTELGYSCFFSHAKMLQSHRNRVFHDFRSGVCRNLVCSDLLTRGIDIQAVNVVINFDFPKNSET

YLHRIGRSGRYGHLGLAINLITYEDRFNLYKIEQELGTEIQPIPSQIDRSLYVAPNASDDDGQIQQSQQQQKGLPTLPPT

QQVMNAYSQPPPQPTFNQSNQNHQYQQRSNRGGYNSGYYGGSGFRGRGGGPPRGGVPRA

>KAF7789667.1 hypothetical protein EIP86_000613 [Pleurotus ostreatoroseus]

MSQQARPSSSNAGESWKSGLSLPPKDNRPQTDDVLATKGLEFEDMKLRRELLMGIFEAGFERPSPIQEEAIPIALTKRDV

LARAKNGTGKTAAFVIPSLQQVDVEKNKIQALLLVPTRELALQTAQVCKILGKHMGIQVMVTTGGTTLKDDIIRLSETVH

VLVGTPGRILDLVGKNVADLSECPVFVMDEADKLLSPEFAPVMEQLLSYMPNDRQVMLFSATFPLIVKDFKDKHMSSPYE

INLMDELTLRGVTQYYAYVEERQKVHCLNTLFSKLQINQSIIFCNSTNRVELLAKKVTELGYSCFYSHAKMLQSHRNRVF

HDFRKGVCRNLVCSDLLTRGIDIQAVNVVINFDFPKNSETYLHRIGRSGRFGHLGLAINLVTYEDRFNLYRIEQELGTEI

QPIPQTIDKGLYVAPGAINDSNEQQKKAQSQAQAQSQPPAPAQAQQRQSIPAQPVPTSGSAQQVVYQSATAPQVRPNGAP

VAASPAGARNGPYPQAGYRAGVPVAR

>TIA80751.1 hypothetical protein E3P98_02481 [Wallemia ichthyophaga]

MMDPFSLGVNLSPVVLKTLVRHLIKKRNNNTNKSETPSTPNDEYAFDQTFKLTRAFLDIAMNHPVEDLQSLGSTYIPPPF

WVHNVAVTVPMSIINNAAEYLIKAFGGEQPMRAAVGGTRWWQVRPTPGLPSEWICMKSDVKLATRNNKPNKPREQPKDSF

TPQFFKDIEGLPRTILFVHGGAYYWGSINTHRYLIWRMARKMRGKAFAVSYRLSPQYGFPCALQDVLSAYLYLLYPPEGA

PHKAVDPSNLIFAGDSAGGGLLLSLLLIIRDLGLPLPAGSIHISPWCDLSHSFPSVLSNYETDVIPKYGFVHKPSTLWPP

PSEELLQRTRQHIKSSTQRSKSPLRGVGSAASRKAEEGRIEEDAINEAAGMTNERGHEKLEPPRDVPTVDLDGRTVELKG

QIQMYATNEQVGHPYVSPVLAHLGGLCPLFIMASDGELLRDEGIYIAHKAARPDEFEYKEDAKRRLPSMNGIEGRFGPTK

VHLQVYDKMCHDLPLFSMVTPAKYAYRAMAAFCFQVTKDTTVQPSPALQGSDIPNTMSDLELDSAAESPSPLRKLDSPAE

APSPSTKLDSPADVPSPSRKIDNPAIALLPPPPSPGKPKRKFSLLGLKNNEGAGATSIDMEKAATVSSQTGTPASDAERP

IKSGPGTAGNMAVYDESMAFKERNMIRERVSLVGQIRPMESSENMDILRMPADEVGVFKPAAVKRYLEGKNKWMHKFKHT

AKNVERQREKSLKESNIHAEKKIKDSDNIFDTPQVTWRWQWALENERPPPASIVARRDTQEARKLVQIVDNADSKQNSRT

LSLWTLVMHLMSPQKSKASTNDDGDDVAPWTKDIEKLKRKDGKKDEGQTVSESDSPEEEEINIDKRKEQWLKNHVKDEYW

LWLSDDTLDKRSGKWIVESAFHIYHHEWLQLRKRRRQEISNTAHKAHPKTSAESEWKNQLRVPQKDLRPQTEDVTKTKGG

EWEDFGLRRELLMGIFEAGFEKPSPIQEEAIPSAIAGRDILARAKNGTGKTASFVIPSLEKINVQKPKIQALLLVPTREL

ALQTSQVCKSLGKHLGIQVMVTTGGTTLRDDIMRLSDPVHILVGTPGRILDLAGKGVANLNECPTFVMDEADKLLSPEFT

PVMEQLLSHLPSSRQVMLFSATFPLIVKDFKEKHMKDPHEINLMDELTLRGITQYYAFVDERQKVHCLNTLFSKLQINQS

IIFCNSTNRVELLAKKVTELGYSCFFSHAKMLQSHRNRVFHDFRSGVCRNLVCSDLLTRGIDIQAVNVVINFDFPKNSET

YLHRIGRSGRYGHLGLAINLITYEDRFNLYKIEQELGTEIQPIPSQIDRSLYVAPNASDDDGQIQQSQQQQKGLPTLPPT

QQVMNAYSQPPPQPTFNQSNQNHQYQQRSNRGGYNSGYYGGSGFRGRGGGPPRGGVPRA

>RKP07575.1 P-loop containing nucleoside triphosphate hydrolase protein [Thamnocephalis sphaerospora]

MLPQHPPAGGDQESWKTQLNLPAKDTRPQTEDVTNTKGVEFEELYLKRELLMGIFEAGFERPSPIQEESIPIALTGRDIL

ARAKNGTGKTAAFVIPSLEKVQPRKSKIQALLLVPTRELALQTSQVCKTLGKHLNIEVMVTTGGTTLKDDIMRLAQPVHI

LVGTPGRILDLASKGVADFSECPTFVMDEADKLLSPEFSPVVERLLGFFPKDRQIMLYSATFPIIVKSFKDKYMVKPYEI

NLMDELTLRGVTQYYAFVEERQKVHCLNTLFSKLQINQSIIFCNSTNRVELLAKKITELGYSCFYSHAKMPQQNRNRVFH

DFRQGSSRNLVCSDLLTRGIDIQAVNVVINFDFPKNAETYLHRIGRSGRFGHLGLAINLITYDDRFNLYKIEQELGTEIQ

PIPPVIDKRLYVAPNLLEEAAGQPRVTPQQQAEQQQLLQQQFQQQRHYQRQLHQHHHHHQQQQQQSHGPHAYGTSNHHHH

QQAHHQQQPQSHHRNQQRRRNNNRHQHGGGGGGGGGGNANGQAAGGVRNA

>EIE83902.1 ATP-dependent RNA helicase DHH1 [Rhizopus delemar RA 99-880]

MSTNQTSRDNENWKNTLALPQKDNRPQTEDVTATKGNEFEDYFLKRELLMGIFEAGFEKPSPIQEEAIPLALAGRDILAR

AKNGTGKTAAFIIPTLEKINNSLSKIQALILVPTRELALQTSQVCKTLGKHLNIQVMVTTGGTTLKDDIMRLSEPVHVVV

GTPGRILDLASKGVADFSTANTFVMDEADKLLSPEFTPIIEQLLGYFPNNRQIMLFSATFPMVVKTFKDKFLIKPYEINL

MDELTLRGVTQYYAYVEEKQKVHCLNTLFSKLQINQSIIFCNSTNRVELLAKKITELGYSCFYSHAKMLQSHRNRVFHDF

RNGVCRNLVCSDLLTRGIDIQAVNVVINFDFPKNAETYLHRIGRSGRFGHLGLAINLITYEDRFNLYKIERELGTEIQPI

PPVIDKNLYVAPNALEDAQVQQPNRQQANATRQHQQNQEQQGQQSIMSDSQVQLMQAQQRMQSQMIPQQQEEQQQQQQQQ

QRQTQHHSHYNHHKSNNWRGRGRGRGRGGNHHQSNRPAPKPLF

>XP_014568574.1 hypothetical protein L969DRAFT_47510 [Mixia osmundae IAM 14324]

MADVAMETMQPAPAPAAVKESAPPAAAEPRRVRFNVGSKYQVREIIGEGAYGVVCSAYHKPTGTRVAIKKIQPFDHTTPS

LIEDGRRTLRELKLLKYFQEQNVSENIISIIDIIKPSTIDSFKEVYIIQELMETDLHRVIRTQDLSMSHIEYLTYQCLRA

LKACHSADIIHRDLKPSNLLLNSNCDLKVCDFGLARSIQTAQPAANSSTGFMTEYVATRWYRAPEIMLTFKQYTKAIDIW

SVGCIVAEMLSGRPLFPGRDYHHQLTLILDVLGTPPLDEFYAINSRRSRDYLRALPFRKKRSFESLFPNAPPEAIDFLSR

SLTFSPTKRMTVEEALAHPFMSAYHDPEDEPSAPPLPPHFFHFDHVKDEITKDALKQLLYDERRRKTDRLHQLQRERHTS

PTKHRVRATPDDTSTPSDCQGRPAAVIMASASTSRAAANPEGQWKEKLNLPAKDNRPQTEDVLRTKGNEFEDYFLKRELL

MGIFEAGFEKPSPIQEEAIPIALAGRDILARAKNGTGKTGSFVIPALEKVNPKVNKIQALILVPTRELALQTSQVCKTLG

KHLGVQIMVTTGGTSLKDDILRLQETVHIVVGTPGRVLDLASKGIADVSACPTFVMDEADKLLSPEFTLVIEQILSLLPK

DRQVMLFSATFPLLVKDFKDKHMTKPYEINLMDELTLRGVTQYYAFLEERQKVHCLNTLFSKLQINQSIIFTNSTSRVEL

LAKKITELGYSCFYSHAKMLQADRNRVFHDFRSGKCRNLVCSDLLTRGIDVPSVNVVINFDFPKQSESYLHRIGRSGRFG

HLGLAINLITYEDRFNLYRIEQELGTEILPIPPTIDRSLYVAPGSGDHPAERKPQQPRNASQQQPQQTAQRVQGSSQPSY

ASNHAPNSTNRPIIPPQQSMVQAQNPINEISHAKPTHYANGAPPNGRQRAGAGRGQQPDGVSRSSAARESATTIKEAPKD

KIIIYRQQLRQAVSIRSGIRCVNSGSNREDLAGRSHLCSHRSAQPLWPITMLPESAMARGRRHHPRASFNACSLSSMLGV

EATLIILQ

>KAF5106618.1 hypothetical protein DV453_003767 [Geotrichum candidum]

MSDSDWKLNLNLPARDTRPQTEDVTATKGNGFEDFYLKRELLMGIFEAGFEKPSPIQEESIPIALAGRDILARAKNGTGK

TASFVIPALQQVNPKLAKIQALILVPTRELALQTSQVCKTLGKHLNINVMVTTGGTTLKDDIIRLNDAVHVLVGTPGRVL

DLAGKNVVDFSECPMFIMDEADKLLSPEFTPIIEQCLAYFPSSRQILLFSATFPLVVKSFMDKHLNKPYEINLMDELTLR

GITQYYAFVEEKQKLHCLNTLFSKLQINQSIIFCNSTNRVELLARKITELNYSCFYSHAKMLQSHRNRVFHEFRNGNCRN

LVCSDLLTRGIDIQAVNVVINFDFPKNAETYLHRIGRSGRFGHLGLAINLISWNDRFNLYKIEQELGTEIKPIPSTIDTS

LYVADSAENIPRPFPMQELPKTSSHQNRYQNNNNNNNQYQQQQQYQQQSQYPQQQQYQQQPQYQQQPYQQQQFQQQPYQQ

QQQPYRQQIPPPGFNGGNGNFPPQQQFQQRPSGY

>TIB00850.1 hypothetical protein E3P96_02548 [Wallemia ichthyophaga]

MMDPFSLGVNLSPVVLKTLVRHLIKKRYNNTNKSETPSTPNDEYAFDQTFKLTRAFLDIAMNHPVEDLQSLGSTYIPPPF

WVHNVAVTVPMSIINNAAEYLIKAFGGEQPMRAAVGGTRWWQVRPTPGLPSEWICMKSDVKLATRNNKPNKPREQPKDSF

TPQFFKDIEGLPRTILFVHGGAYYWGSINTHRYLIWRMARKMRGKAFAVSYRLSPQYGFPCALQDVLSAYLYLLYPPEGA

PHKAVDPSNLIFAGDSAGGGLLLSLLLIIRDLGLPLPAGSIHISPWCDLSHSFPSVLSNYETDVIPKYGFVHKPSTLWPP

PSEELLQRTRQHIKSSTQRSKSPLRGVGSAASRKAEEGRIEEDAINEAAGMTNERGHEKLEPPRDVPTVDLDGRTVELKG

QIQMYATNEQVGHPYVSPVLAHLGGLCPLFIMASDGELLRDEGIYIAHKAARPDDFEYKEDAKRRLPSMNGIEGRFGPTK

VHLQVYDKMCHDLPLFSMVTPAKYAYRAMAAFCFQVTKDTTVQPSPALQGSDIPNTMSDLELDSAAESPSPLRKLDSPAE

APSPSTKLDSPADVPSPSRKIDNPAIALLPPPPSPGKPKRKFSLLGLKNNEGAGATSIDMEKAATVSSQTGTPASDAERP

IKSGPGTAGNMAVYDESMAFKERNMIRERVSLVGQIRPMESSENMDILRIPADEVGVFKPAAVKRYLEGKNKWMHKFKHT

AKNVERQREKSLKESNIHAEKKIKDSDNIFDTPQVTWRWQWALENERPPPASIVARRDTQEARKLVQIVDNADSKQNSRT

LSLWTLVMHLMSPQKSKANTNDDGDDVAPWTKDIEKLKRKDGKKDEAQTVSESDSPEEEEINIDKRKEQWLKNHVKDEYW

LWLSDDTLDKRSGKWIVESAFHIYHHEWLQLRKRRRQEISNTAHKAHPKTSAESEWKNQLRVPQKDLRPQTEDVTKTKGG

EWEDFGLRRELLMGIFEAGFEKPSPIQEEAIPSAIAGRDILARAKNGTGKTASFVIPSLEKINVQKPKIQALLLVPTREL

ALQTSQVCKSLGKHLGIQVMVTTGGTTLRDDIMRLSDPVHILVGTPGRILDLAGKGVANLNECPTFVMDEADKLLSPEFT

PVMEQLLSHLPSSRQVMLFSATFPLIVKDFKEKHMKDPHEINLMDELTLRGITQYYAFVDERQKVHCLNTLFSKLQINQS

IIFCNSTNRVELLAKKVTELGYSCFFSHAKMLQSHRNRVFHDFRSGVCRNLVCSDLLTRGIDIQAVNVVINFDFPKNSET

YLHRIGRSGRYGHLGLAINLITYEDRFNLYKIEQELGTEIQPIPSQIDRSLYVAPNASDDDGQIQQSQQQKGLPTLPPTQ

QVMNAYSQPPPQPTFNQSNQNHQYQQRSNRGGYNSGYYGGSGFRGRGGGPPRGGVPRA

>TFK25287.1 eukaryotic translation initiation factor 4A-like protein [Coprinopsis marcescibilis]

MAQVAPASSSSSTSTTEHWRNGLQAPPKDVRPQTEDVTATKGMEFEDMKLRRELLMGIFEAGFEKPSPIQEEAIPTALTS

RDILARAKNGTGKTAAFVIPSLEQVDVGKPKIQALLLVPTRELALQTAQVCKILGKHMGVQVMVTTGGTTLKDDIIRLSE

SVHVLVGTPGRILDLAGKGVADLSECPVFVMDEADKLLSPEFAPVMEQLLSFLPQERQVMLFSATFPMIVKDFKDKHMRS

PYEINLMDELTLRGVTQYYAYVEERQKVHCLNTLFSKLQINQSIIFCNSTNRVELLAKKITELGYSCFYSHAKMLQSHRN

RVFHDFRNGVCRNLVCSDLLTRGIDIQAVNVVINFDFPKNSETYLHRIGRSGRFGHLGLAINLVTYEDRFNLYKIEQELG

TEIQPIPQSIDRSLYVAPNATDEQKQKPFQHQSQQQKQIAAVSAQQQQQQQQQQQRNGQISQQQAYAAQLRQNGQPQPPR

IQQQQQQQQQAYRGGVPVAR

>TIB31577.1 hypothetical protein E3P86_03307 [Wallemia ichthyophaga]

MMDPFSLGVNLSPVVLKTLVRHLIKKRNNNTNKSETPSTPNDEYAFDQTFKLTRAFLDIAMNHPVEDLQSLGSSYIPPPF

WVHNVAVTVPMSIINNAAEYLIKAFGGEQPMRAAVGGTRWWQVRPTPGLPSEWICMKSDVKLATRNNKPNKPREQPKDSF

TPQFFKDIEGLPRTILFVHGGAYYWGSINTHRYLIWRMARKMRGKAFAVSYRLSPQYGFPCALQDVLSAYLYLLYPPEGA

PHKAVDPSNLIFAGDSAGGGLLLSLLLIIRDLGLPLPAGSIHISPWCDLSHSFPSVLSNYETDVIPKYGFVHKPSTLWPP

PSEELLQRTRQHIKSSTQRSKSPLRGVGSAASRKAEEGRIEEDAINEAAGMTNERGHEKLEPPRDVPTVDLDGRTLELKG

QIQMYATNEQVGHPYVSPVLAHLGGLCPLFIMASDGELLRDEGIYIAHKAARPDDFEYKEDAKRRLPSMNGIEGRFGPTK

VHLQVYDKMCHDLPLFSMVTPAKYAYRAMAAFCFQVTKDTTVQPSPALQGSDIPNTMSDLELDSAAESPSPLRKLDSPAE

APSPSTKLDSPADVPSPSRKIDNPAIALSPPPPSPGKPKRKFSLLGLKNNEGAGATSIDMEKAATVSSQTGTPASDAERP

IKSGPGTAGNMAVYDESMAFKERNMIRERVSLVGQIRPMESSENMDILRIPADEVGVFKPAAVKRYLEGKNKWMHKFKHT

AKNVERQREKSLKESNIHAEKKIKDSDNIFDTPQVTWRWQWALENERPPPASIVARRDTQEARKLVQIVDNADSKQNSRT

LSLWTLVMHLMSPQKSKASTNDDGDDVAPWTKDIEKLKRKDGKKDEGQTVSESDSPEEEEINIDKRKEQWLKNHVKDEYW

LWLSDDTLDKRSGKWIVESAFHIYHHEWLQLRKRRRQEISNTAHKAHPKTSAESEWKNQLRVPQKDLRPQTEDVTKTKGG

EWEDFGLRRELLMGIFEAGFEKPSPIQEEAIPSAIAGRDILARAKNGTGKTASFVIPSLEKINVQKPKIQALLLVPTREL

ALQTSQVCKSLGKHLGIQVMVTTGGTTLRDDIMRLSDPVHILVGTPGRILDLAGKGVANLNECPTFVMDEADKLLSPEFT

PVMEQLLSHLPSSRQVMLFSATFPLIVKDFKEKHMKDPHEINLMDELTLRGITQYYAFVDERQKVHCLNTLFSKLQINQS

IIFCNSTNRVELLAKKVTELGYSCFFSHAKMLQSHRNRVFHDFRSGVCRNLVCSDLLTRGIDIQAVNVVINFDFPKNSET

YLHRIGRSGRYGHLGLAINLITYEDRFNLYKIEQELGTEIQPIPSQIDRSLYVAPNASDDDGQIQQSQQQQKGLPTLPPT

QQVMNAYSQPPPQPTFNQSNQNHQYQQRSNRGGYNSGYYGGSGFRGRGGGPPRGGVPRA

>KDQ06910.1 hypothetical protein BOTBODRAFT_60364 [Botryobasidium botryosum FD-172 SS1]

MTSQAQTATAAGTSDSWKAGLRAPPKDDRPQTEDVTATKGLEFEDMFLRRELLMGIFEAGFERPSPIQEEAIPAALSKRD

ILARAKNGTGKTAAFVIPSLQHVDVTKNKIQALLLVPTRELALQTSQVCKTLGKHMGINVMVTTGGTTLKDDILRLSEAV

HVLVGTPGRILDLSGKGVADLSECPVFVMDEADKLLSPEFAPVMEQLLSYLPTPRQVMLFSATFPMIVKEFKDKHMDSPY

EINLMEELTLRGVTQYYAFVEERQKVHCLNTLFSKLQINQSIIFCNSTNRVELLAKKVTELGYSCFYSHAKMLQSHRNRV

FHDFRNGACRNLVCSDLLTRGIDIQAVNVVINFDFPKNSETYLHRIGRSGRFGHLGLAINLITYEDRFNLYRIEQELGTE

IQPIPQVIDKNLYVAPSAIGEPVGQKTAQRSQQQQQPQAQQQRTAQGQNGSAAPAVTRTPQPQQAQQQQQQQQPNRNGQL

SHQQQLQLQQQQQQAQWQQQQQQLQAQNAAAVAAAAANQQQQQPRYQQQPQGHGSSSPAPRGGYRGGATGPVRGGAPTRG

MR

>ORY91167.1 ATP-dependent RNA helicase DHH1 [Syncephalastrum racemosum]

MSHENDKWKEDLNLPTKDSRPQTEDVTATKGNEFEDYFLKRELLMGIFEAGFERPSPIQEEAIPIALTGRDILARAKNGT

GKTAAFVIPTLEKINNKKNKIQALILVPTRELALQTSQVCKNLGKHLDIQVMVTTGGTTLKDDIMRLSETVHIVVGTPGR

ILDLAGKGVADFSEAHTFVMDEADKLLSPEFTPIMDQLIQFFPPTRQIMLFSATFPMIVKTFKEKHLKRPYEINLMDELT

LRGVTQFYAYVEEKQKVHCLNTLFSKLQINQSIIFCNSTNRVELLAKKITDLGYSCFYSHARMLQSHRNRVFHDFRNGVC

RNLVCSDLLTRGIDIQAVNVVINFDFPKNAETYLHRIGRSGRFGHLGLAINLITYEDRFNLYKIERELGTEIQPIPPVIN

KDLYVAPNSLETNMIQQPNREAAEATRQQQQQQDADQQHQQHQGRRHHHRGGRGGGGGGGGGNRGGYRRPYKPKAAQPN

>TIA70154.1 hypothetical protein E3P91_03222 [Wallemia ichthyophaga]

MMDPFSLGVNLSPVVLKTLVRHLIKKRNNNTNKSETPSTPNDEYAFDQTFKLTRAFLDIAMNHPVEDLQSLGSTYIPPPF

WVHNVAVTVPMSIINNAAEYLIKAFGGEQPMRAAVGGTRWWQVRPTPGLPSEWICMKSDVKLATRNNKPNKPREQPKDSF

TPQFFKDIEGLPRTILFVHGGAYYWGSINTHRYLIWRMARKMRGKAFAVSYRLSPQYGFPCALQDVLSAYLYLLYPPEGA

PHKAVDPSNLIFAGDSAGGGLLLSLLLIIRDLGLPLPAGSIHISPWCDLSHSFPSVLSNYETDVIPKYGFVHKPSTLWPP

PSEELLQRTRQHIKSSTQRSKSPLRGVGSAASRKAEEGRIEEDAINEAAGMTNERGHEKLEPPRDVPTVDLDGRTVELKG

QIQMYATNEQVGHPYVSPVLAHLGGLCPLFIMASDGELLRDEGIYIAHKAARPDEFEYKEDAKRRLPSMNGIEGRFGPTK

VHLQVYDKMCHDLPLFSMVTPAKYAYRAMAAFCFQVTKDTTVQPSPALQGSDIPNTMSDLELDSAAESPSPLRKLDSPAE

APSPSTKLDSPADVPSPSRKIDNPAIALLPPPPSPGKPKRKFSLLGLKNNEGAGATSIDMEKAATLSSQTGTPASDAERP

IKSGPGTAGNMAVYDESMAFKERNMIRERVSLVGQIRPMESSENMDILRMPADEVGVFKPAAVKRYLEGKNKWMHKFKHT

AKNVERQREKSLKESNIHAEKKIKDSDNIFDTPQVTWRWQWALENERPPPASIVARRDTQEARKLVQIVDNADSKQNSRT

LSLWTLVMHLMSPQKSKASTNDDGDDVAPWTKDIEKLKRKDGKKDEGQTVSESDSPEEEEINIDKRKEQWLKNHVKDEYW

LWLSDDTLDKRSGKWIVESAFHIYHHEWLQLRKRRRQEISNTAHKAHPKTSAESEWKNQLRVPQKDLRPQTEDVTKTKGG

EWEDFGLRRELLMGIFEAGFEKPSPIQEEAIPSAIAGRDILARAKNGTGKTASFVIPSLEKINVQKPKIQALLLVPTREL

ALQTSQVCKSLGKHLGIQVMVTTGGTTLRDDIMRLSDPVHILVGTPGRILDLAGKGVANLNECPTFVMDEADKLLSPEFT

PVMEQLLSHLPSSRQVMLFSATFPLIVKDFKEKHMKDPHEINLMDELTLRGITQYYAFVDERQKVHCLNTLFSKLQINQS

IIFCNSTNRVELLAKKVTELGYSCFFSHAKMLQSHRNRVFHDFRSGVCRNLVCSDLLTRGIDIQAVNVVINFDFPKNSET

YLHRIGRSGRYGHLGLAINLITYEDRFNLYKIEQELGTEIQPIPSQIDRSLYVAPNASDDDGQIQQSQQQQKGLPTLPPT

QQVMNAYSQPPPQPTFNQSNQNHQYQQRSNRGGYNSGYYGGSGFRGRGGGPPRGGVPRA

>KWU43124.1 DEAD-domain-containing protein [Rhodotorula sp. JG-1b]

MSAPASTSSPSAPPPTGAEAPAPASAPAPAPQPQDWRKQLNLPQKDTRPQTEDVTNTKGNDFEDYFLKRELLMGIFEAGF

ERPSPIQEEAIPIALTGRDVLARAKNGTGKTAAFIIPTLERVNPKIPKIQALLLVPTRELALQTSQVCKTLGKHTGINVM

VTTGGTTLRDDIMRLGEAVHVLVGTPGRILDLAGKGIADLSECPIFVMDEADKLLSPEFTPVIEQLLSFMPKERQVMLFS

ATFPIIVKDFKDKWMRKPYEINLMDELTLRGVTQYYAFLEERQKVHCLNTLFSKLQINQSIIFCNSTNRVELLAKKITEL

GYSCFYSHARMLQAHRNRVFHDFRNGVCRNLVCSDLLTRGIDIQAVNVVINFDFPKSAETYLHRIGRSGRYGHLGLAINL

ITSDDRYNLYRIEQELGTEIQPIPPVIDRSLYVAPGTEEESKDAQAAAAKGQRPAGQQPQYQPQQQQQQQQQQTSQQQQQ

QQPPQMAAAPVSIPQYGGGGYGGPPPPQQNGGGPMPPHQQQQQIPPHLLQQYYQQQQQVAMYAAQQQQGQQQLRR

>KAA1095116.1 DExD/H-box ATP-dependent RNA helicase dhh1 [Puccinia graminis f. sp. tritici]

MAASSSSASDMSHDPDWKSKLSLPQKDLRPQTEDVTKTKGIEFEDLYLRRDLLMGIFEAGFEKPSPIQEEAIPIALAGRD

ILARAKNGTGKTGAFVIPTLEKTNVRINRIQALILVPTRELALQTSQVCKTLGKHTGIQIMVTTGGTTLKDDILRLQEAV

HVVVGTPGRILDLASKGVADLSQCKTFVMDEADKLLSPEFTVVIEQLLSFLHKDRQVMLFSATFPMIVKDFKDKHMIKPY

EINLMEELTLQGVTQYYAFLEERQKVHCLNTLFSKLQINQAIIFCNSTNRVELLARKITDIGYSCFYSHAKMLQQHRNRV

FHDFRNGVCRNLVCSDLLTRGIDIQAVNVVINFDFPKNAETYLHRIGRSGRFGHRGIAINLITYENRFDLYRIEQELGTE

IQPIPAVIDKALYVAPGIEDTPSSTPTHGGSSVSTSNAHQATHPNQSQQSNQAHRNGRQTNHQPSDQSHVPQPHDKPTLP

PQQLTFQKAAPIDSLSHATPTEYANGAPPPNSSRVNNHKSTRGGSGGPHRGRGGGAHRAPSGIAPAA

>CEG81286.1 Putative Atp-dependent rna helicase dhh1 [Rhizopus microsporus]

MSSSSQLNHEIENWKTALTLPPKDSRPQTEDVTATKGNEFEDYFLKRELLMGIFEAGFERPSPIQEEAIPLALAGRDILA

RAKNGTGKTAAFVIPTLEKITNTPKIQALILVPTRELALQTSQVCKTLGKHLNIQVMVTTGGTTLKDDIMRLSETVHIVV

GTPGRILDLASKGVADFSAAHTFVMDEADKLLSPEFKPIIEQLLDYFPKNKQIMLFSATFPMIVKNFKDKYLVKPYEINL

MDELTLRGVTQYYAYVEEKQKVHCLNTLFSKLQINQSIIFCNSTNRVELLAKKITELGYSCFYSHAKMLQSHRNRVFHDF

RNGVCRNLVCSDLLTRGIDIQAVNVVINFDFPKNAETYLHRIGRSGRFGHLGLAINLITYEDRFNLYKIERELGTEIQPI

PPVIDKSLYVAPNALEDAQIQQPNKQQANATRQHQQSTEQQDQTQQPLQGQQQQQQQRQHYSHQQRHPHYHNRSNNWRGR

GRGGGHHHSNRSTQKQQPYQQ

>KZT71909.1 eukaryotic translation initiation factor 4A-like protein [Daedalea quercina L-15889]

MAQQSRPSSSTSDGWKASLRPPPKDSRPQTEDVTNTKGIDFEDMYLRRELLMGIFEAGFERPSPIQEEAIPIALAKRDVL

ARAKNGTGKTAAFVIPSLQQIEVNKNKIQALLLVPTRELALQTAHVCKVLGKHMGAQVMVTTGGTTLKDDIIRLSEAVHV

LVGTPGRILDLAGKNVADLSECPVFVMDEADKLLSPEFAPVMEQLLSYLPKDRQVMLFSATFPMIVKDFKEKHMKQPYEI

NLMDELTLRGVTQYYAYVEERQKVHCLNTLFSKLQINQSIIFCNSTNRVELLAKKVTELGYSCFYSHAKMLQSHRNRVFH

DFRNGVCRNLVCSDLLTRGIDIQAVNVVINFDFPKNSETYLHRIGRSGRFGHLGLAINLVTYEDRFNLYKIEQELGTEIQ

PIPQIIDKGLYVAPNASDDAEQQQSQKTRAAQAQQTPQQRQVAPQPALPQQATVQPQVVYSSSPRPVAANGNGVPPPAQR

APYRGVPVAR

>KAF5314628.1 hypothetical protein D9611_007143 [Coprinellus angulatus]

MAQPASASSSSSTPAPNEEWKTGLKAPRKDLRPQTEDVTATKGLEFEDMFLRRELLMGIFEAGFEKPSPIQEEAIPIALT

KRDVLARAKNGTGKTAAFVIPTLQQVDVARNKIQALLLVPTRELALQTSQVCKILGKHMGVQVMVTTGGTTLKDDIMRLS

ETVHVLVGTPGRILDLAGKGVADLSECPVFVMDEADKLLSPEFAPVMEQLLAFLPQERQVMLFSATFPMIVKDFKDKHMT

TPYEINLMDELTLRGVTQYYAFVEERQKVHCLNTLFSKLQINQSIIFCNSTNRVELLAKKITELGYSCFYSHAKMLQSHR

NRVFHDFRNGVCRNLVCSDLLTRGIDIQAVNVVINFDFPKNSETYLHRIGRSGRFGHLGLAINLVTYEDRFNLYKIEQEL

GTEIQPIPQSIDRSLYVAPEVGEEERQRPFQNPLPVQQMQQQSVQPVPQQVQQVQVQQVQQQVQAQQAQQVQQQRVAPHQ

LQGQQQQRNGGYPVQVQQQQQQMMMRQNGQVPRGQGAYRGGAPVAR

>GAN00930.1 conserved hypothetical protein [Mucor ambiguus]

MSLNNNLSHENDNWKKSLALPAKDVRPQTEDVTATKGNEFEDYFLKRELLMGIFEAGFERPSPIQEEAIPIALTGRDILA

RAKNGTGKTAAFVIPTLEKINNKRNKIQALLLVPTRELALQTSQVCKNLGKHLGLQIMVTTGGTTLKDDIMRLSEPVHVV

VGTPGRILDLASKGVADFSEASTFVMDEADKLLSPEFTPIIEQLIHHFPKDRQIMLFSATFPLIVKTFKEKFLVKPYEIN

LMDELTLRGVTQYYAYVEEKQKVHCLNTLFSKLQINQSIIFCNSTNRVELLAKKITELGYSCFYSHARMLQSHRNRVFHD

FRNGVCRNLVCSDLLTRGIDIQAVNVVINFDFPKNAETYLHRIGRSGRFGHLGLAINLITYEDRFNLYKIERELGTEIQP

IPPVIDKSLYVAPNALEDAQVQQPDRQQALATRQQQQQQSNEEQQQPHYYQNNNGRSNNWRGRGRGGGGGGEGYHHQYNN

RPKQQHPSFQD

>POY74810.1 hypothetical protein BMF94_2083 [Rhodotorula taiwanensis]

MSAPSAASASASAPQPQDWKKQLNLPQKDTRPQTEDVTATKGNDFEDYFLKRELLMGIFEAGFERPSPIQEEAIPIALTG

RDVLARAKNGTGKTAAFIIPTLERVNPKIPKIQALLLVPTRELALQTSQVCKTLGKHTGINVMVTTGGTTLRDDIMRLGE

AVHVLVGTPGRILDLAGKGIADLSECPIFVMDEADKLLSPEFTPVIEQLLSFMAKERQVMLFSATFPIIVKDFKDKWMRK

PYEINLMDELTLRGVTQYYAFLEERQKVHCLNTLFSKLQINQSIIFCNSTNRVELLAKKITELGYSCFYSHARMLQAHRN

RVFHDFRNGVCRNLVCSDLLTRGIDIQAVNVVINFDFPKSAETYLHRIGRSGRYGHLGLAINLITYDDRYNLYRIEQELG

TEIQPIPPVIDRSLYVAPGTEEEPASTANSNSSQAAGGKVASRPAGVGGHGMQQPQQRQLAAAPQQAAPQQSQPQQQQQQ

SAALQSAAPVSLPTQQPQSQPRPPQMNGMQMPQQRFVPPPPHGQPAPQIPPHMMQQYIQQQQTAYYAQQQQQQQQQR

>EPB90373.1 ATP-dependent RNA helicase dhh1 [Mucor circinelloides 1006PhL]

MSLNNNLSHENDNWKKSLALPAKDVRPQTEDVTATKGNEFEDYFLKRELLMGIFEAGFERPSPIQEEAIPIALTGRDILA

RAKNGTGKTAAFVIPTLEKINNKKNKIQALLLVPTRELALQTSQVCKNLGKHLGLQIMVTTGGTTLKDDIMRLSEPVHVV

VGTPGRILDLASKGVADFSEASTFVMDEADKLLSPEFTPIIEQLINHFPKDRQIMLFSATFPLIVKTFKDKFLVKPYEIN

LMDELTLRGVTQYYAYVEEKQKVHCLNTLFSKLQINQSIIFCNSTNRVELLAKKITELGYSCFYSHARMLQSHRNRVFHD

FRNGVCRNLVCSDLLTRGIDIQAVNVVINFDFPKNAETYLHRIGRSGRFGHLGLAINLITYEDRFNLYKIERELGTEIQP

IPPVIDKSLYVAPNALEDAQVQQPDRQQALATRQQQQQQSNEEQQQPHYYQNNSNSRSNNWRGRGRGGSGGGGGGGGNGY

HHQYQNRPKQQQQQQSFQD

>OLL22173.1 ATP-dependent RNA helicase dhh1 [Neolecta irregularis DAH-3]

MPKFPLTYIFRKIPTMSADLTSKMGSVNLGGSDWKSSLKVPLKDTRPQTMDVTNTKGLEFEDLYLRRELLMGIFEHGFEK

PSPIQEEAIPVALTGRDILARAKNGTGKTGAYVIPSLEKVDADQEYIQALLLVPTRELALQTAQVCKSLSKHLNLNVMVT

TGGTTLKDDIIRLEETVHVVVGTPGRILDLAKKGLAKFDQCSTFIMDEADKLLSPEFTPVIEELLAFFPKNRQIMLFSAT

FPVTVKDFMDKYLRNPYEINLMDELTLCGVTQYYAFVEEKQKVHCLNTLFSKLQINQSIIFCNSTNRVELLAKKITELGY

SCFYSHAKMIQNHRNRVFHDFREGGCRNLVCSDLLTRGIDIQAVNVVINFDFPKNAETYLHRIGRSGRFGHLGLAINLIN

WEDRFNLYKIEQELGTEIKAIPPVIDKSLYVAPSAMSLSAGQEKQSRQQQLQQQRNMLDNRHSGRQQQQSQTYQRQPHNQ

QQNYTASANHRGQSSRGRTNARSPSNQPSNGYNRPDGGARVRAQ

>KAE8226104.1 hypothetical protein CF319_g1247 [Tilletia indica]

MSTSATSSSQSSLQPGPGQSDAQWRQQVMAHIPKDDRPQTQDVTATKGNDFEDYFLKRELLMGIFEAGFEKPSPIQEEAI

PIALTGRDILARAKNGTGKTAAYVIPTLERLNTKKNKIQAVLLVPTRELALQTSQVAKTLGKHLGVEIMVTTGGTTLKDD

ILRLSQVVHVLVGTPGRILDLASKGVADLSQCTTFVMDEADKLLSPEFTPVMEQLLSLLPQERQVMLFSATFPLIVKDFK

DKHMVKPYEINLMDELTLRGVTQYYAFVEERQKVHCLNTLFSKLQINQSIIFCNSTNRVELLAKKITELGYSCFYSHAKM

LQAHRNRVFHDFRNGACRNLVCSDLLTRGIDIQAVNVVINFDFPKNAETYLHRIGRSGRFGHLGLAINLITYEDRFNLYR

IEQELGTEIQPIPASIDKRLYVAPGLINAVEQQALKNGANGSSRGQGGNNGKQSGNGSQLGGGGIQAGRPVIPPTQAAMH

ANAIPQNESFTQSVPGRYRGGGQQQRAGGQQGQPSA

>KAF7764004.1 hypothetical protein Agabi119p4_8541 [Agaricus bisporus var. burnettii]

MSESELREYDKLLDEADWDIYYWCTEEREPPARWATSAVLGRLREHARNEGRVVFFCSQLHTPSTLTMSQPSSASSSSTP

HDSAWKTGLRPPPRDSRPQTEDVTATKGLEFEDMSIRRELLMGIFEAGFEKPSPIQEEAIPFALTKRDILARAKNGTGKT

AAFVIPSLQQIDINKHKIQALLLVPTRELALQTSQVCKTLGKHMGIQVMVTTGGTTLKDDILRLSESVHVLVGTPGRILD

LAGKGVADLSECPVFVMDEADKLLSPEFAPVMEQLLSYLPSERQVMLFSATFPMIVKTFKDKHMRSPYEINLMDELTLRG

VTQYYAYVEERQKVHCLNTLFSKLQINQSIIFCNSTNRVELLAKKVTELGYSCFYSHAKMLQSHRNRVFHDFRNGVCRNL

VCSDLLTRGIDIQAVNVVINFDFPKNSETYLHRIGRSGRFGHLGLAINLVTYEDRFNLYKIEQELGTEIQPIPQTIDRTL

YVAPGSSEEQHQKPQPQSQQPQQRQAAPSPSPAAAAAPRAGQPSLPAPQSISQQPAFNGQASARANGGLPPQAQQMRGQP

TYRGGVPVAR

>CUA76911.1 ATP-dependent RNA helicase DDX6/DHH1 [Rhizoctonia solani]

MASTTNTPSASNGDWKAGLRAPPKDDRPQTEDVTATKGLEFEDMALRRELLMGIFEAGFERPSPIQEEAIPVALTRRDIL

ARAKNGTGKTAAFTIPSLQHVDPAKPKIQAMLLTPTRELALQTAQVCKTLGKHMGINVMVTTGGTTLKDDIIRLSEAVHV

LVGTPGRILDLAGKQVADLSSCRVFVMDEADKLLSPEFTPVMEQLLSFVPADRQVMLFSATFPMIVKQFKDKHMKNPHEI

NLMDELTLRGVTQYYAFVEERQKVHCLNTLFAKLQINQSIIFCNSTNRVELLAKKVTELGYSCFYSHAKMVQSARNRVFH

DFRNGVCRNLVCSDLLTRGIDIQAVNVVINFDFPKHSETYLHRIGRSGRFGHLGLAINLITYEDRFNLYRIEQELGTEIQ

PIPAEINKSLYVAPSAIDEPAGQVPSNRSSAPESNSRSGTNGQATDGQQPRNGGTPTGQGRGQPRGGQRGGGANGRGAAP

QR

>XP_016235192.1 ATP-dependent RNA helicase DHH1 [Exophiala spinifera]

MTDALASQLNKTSLNDGADESNWKDSLKIPNKDTRQQTEDVTATKGLDFEDFYIKRELMMGIFEAGFEKPSPIQEETIPV

ALTGRDILARAKNGTGKTAAFVIPTLERINPKNPKTQALLLVPTRELALQTSQVCKLLGKHLGINVMVTTGGTGLKDDII

RLGETVHIIVGTPGRILDLASKGVADLSECPIFVMDEADKLLSPEFTVVIEQLLAFLPKDRQVMLFSATFPMMVKTFKDK

HMRNPYEINLMDELTLRGITQYYAFVEEKQKVHCLNTLFSKLQINQSIIFCNSTNRVELLAKKITELGYSCFYSHAKMLQ

QNRNKVFHDFRAGVCRNLVCSDLLTRGIDIQAVNVVINFDFPKNAETYLHRIGRSGRFGHLGLAINLINWDDRFNLYKIE

QELGTEIQPIPPSIDKSLYVYDSPENIPRPMPPPAPASKANNAPMVSANASVGPNSNGQGQGRRQYNQSNGYGQQYNNQG

RGGYRGRGRGQGGRGRGGYAQQAAAPVGQTAQ

>TKA54277.1 ATP-dependent RNA helicase dhh1 [Rhodotorula sp. CCFEE 5036]

MSAPASTSSPSAPPPATGANAPAPAPAPAPQPQDWRKQLNLPQKDTRPQTEDVTNTKGNDFEDYFLKRELLMGIFEAGFE

RPSPIQEEAIPIALTGRDVLARAKNGTGKTAAFIIPTLERVNPKIPKIQALLLVPTRELALQTSQVCKTLGKHTGINVMV

TTGGTTLRDDIMRLGEAVHVLVGTPGRILDLAGKGIADLSECPIFVMDEADKLLSPEFTPVIEQLLSFMPKERQVMLFSA

TFPIIVKDFKDKWMRKPYEINLMDELTLRGVTQYYAFLEERQKVHCLNTLFSKLQINQSIIFCNSTNRVELLAKKITELG

YSCFYSHARMLQAHRNRVFHDFRNGVCRNLVCSDLLTRGIDIQAVNVVINFDFPKSAETYLHRIGRSGRYGHLGLAINLI

TADDRYNLYRIEQELGTEIQPIPPVIDRSLYVAPGTEEESKDAQAVAAKGQRPAGVGGHGMQAPQQRQVAPAPAPQSSAA

APAPAVAPQPQQPQYQPQQPQQQQQPQQQPQQQPQQPPQMAAAPVSIPQYGGGGYGGPPPPPQQNGGGPMPPHQQIPPHL

LQQYYQQQQQAAMYAAQQQQGQQQQLRR

>KIO14251.1 hypothetical protein M404DRAFT_12103 [Pisolithus tinctorius Marx 270]

MSQATRPSSSNSLSTANSNDLSWKAGLRPPPKDLRPQTKDVTATKGLEFEDMYLRRELLMGIFEAGFERPSPIQEEAIPI

ALTKRDVLARAKNGTGKTAAFVIPALQQIDINKPKIQALLLVPTRELALQTSQVCKILGKHMGIQVMATTGGTTLKDDIM

RLAEPVHVLVGTPGRILDLAGKSVADLSECPVFVMDEADKLLSPEFTPVMEQLLSFLPTDRQVMLFSATFPLIVKDFKDK

HMNSPYEINLMEELTLRGVTQYYAYVEERQKVHCLNTLFSKLQINQSIIFCNSTNRVELLAKKITELGYSCFYSHAKMLQ

SHRNRVFHDFRNGVCRNLVCSDLLTRGIDIQAVNVVINFDFPKNSETYLHRIGRSGRFGHFGLAINLVTYEDRFNLYRIE

QELGTEIQPIPQHIDKSLYVAPGGLEEPEPQKQPVQQQPQQRQQAPGQQVQNHQGNGRPNGMQVLPQAAQPQQRVPYPAQ

PGYRATPVAR

>KZV65327.1 DEAD-domain-containing protein [Peniophora sp. CONT]

MIKRGYSADGWRANLRPPPRDERPQTEDVQNIKGIEFEDMKLRRELLMGIFEAGFERPSPIQEEAIPAALSKRDILARAK

NGTGKTASFVVPSLEQVDVSKNKIQALLLVPTRELALQTSQVCKNLGKHMGVQVMVTTGGTTLKDDILRLSETVHVLVGT

PGRILDLAGKGVADLSECPVFVMDEADKLLSPEFAPVMEQLLAFLPKTRQVMLFSATFPIIVKDFKEKHMKNPYEINLME

ELTLRGVTQYYAYVEERQKVHCLNTLFSKLQINQSIIFCNSTNRVELLAKKVTELGYSCFYSHAKMLQSHRNRVFHDFRN

GVCRNLVCSDLLTRGIDIQAVNVVINFDFPKNSETYLHRIGRSGRFGHLGLAINLVTYEDRFNLYKIEQELGTEIQPIPQ

SIDKGLYVAPSAQDEPAGQKPRQQQQTQQQQQVKQTVYQTNGGAAAPAPARNGGAPAAQR

>TPX57814.1 hypothetical protein SpCBS45565_g08144 [Spizellomyces sp. 'palustris']

MSVNGLAMQLQNTHISNHDNNDWKRSLKLPTKDTRPQTEDVTATKGNDFEDYFLKRELLMGIFEAGFEKPSPIQEEAIPI

ALAGRDILARAKNGTGKTAAFIIPALEKVNPSKNHIQALILVPTRELALQTSQVCRSLGKHMGIQVMVTTGGTTLKDDIL

RLGQTATPGRVLDLAGKGVADLRECSMLIMDEADKLLSPEFQPIIEQLISFCRADRQILLFSATFPMIVKNFKDKYLTKP

YEINLMDELTLRGVTQYYAYVEERQKVHCLNTLFSKLQINQSIIFCNSTSRVELLAKKITELGYSCFYIHAKMLQSHRNR

VFHDFRNGKTRHLVCSDLLTRGIDIQAVNVVINFDFPKNAETYLHRIGRSGRFGHLGLAINLITYEDRFNLYRIEQELGT

EIAPIPPVIDKSLYVAP

>THH29408.1 hypothetical protein EUX98_g4781 [Antrodiella citrinella]

MAQAQQQARPSSSTPRINNTNNASADNAWKANLNVPVKDDRPQTEDVTATKGLEFEDMQLRRELLMGIFEAGFERPSPIQ

EEAIPIALTKRDVLARAKNGTGKTAAFVIPSLQQIDINKRKIQALLLVPTRELALQTAQVCKILGKHMGLNVMVTTGGTT

LKDDIMRLAEAVHVLVGTPGRILDLAGKNVADLSECPVFVMDEADKLLSPEFAPVMEQLLSYLPKDRQVMLFSATFPLIV

KDFKDKHMESPYEINLMDELTLRGVTQYYAYVEERQKVHCLNTLFSKLQINQSIIFCNSTNRVELLAKKVTELGYSCFYS

HAKMLQSHRNRVFHDFRQGVCRNLVCSDLLTRGIDIQAVNVVINFDFPKNSETYLHRIGRSGRFGHLGLAINLVTYEDRF

NLYKIEQELGTEIQPIPQQIDKGLYVAPSAIQEDQPQKPKLAQQPQPQPQPAPAPAPAPSQQQRQTATPSNAAPSQPQQQ

QQVVYQSAGGPMRQNGAPVSTNGQPPRGYQGGYRGGVPVAR

>KIJ98785.1 hypothetical protein K443DRAFT_194552 [Laccaria amethystina LaAM-08-1]

MSQPATSSSSTPSITDSWKSTLRPPPKDIRPQTEDVTATKGTEFEDMFLRRELLMGIFEAGFEKPSPIQEEAIPIALAKR

DILARAKNGTGKTAAFVIPSLQQVDISKNKIQALLLVPTRELALQTSQVCKTLGKHIGVQVMVTTGGTTLKDDILRLSET

VHVLVGTPGRILDLAGKNVADLTECPVFVMDEADKLLSPEFAPVMEQLLSYLPTERQVMLFSATFPMIVKDFKDKHMKSP

YEINLMDELTLRGVTQYYAYVEERQKVHCLNTLFSKLQINQSIIFCNSTNRVELLAKKVTELGYSCFYSHAKMLQSHRNR

VFHDFRNGVCRNLVCSDLLTRGIDIQAVNVVINFDFPKNSETYLHRIGRSGRFGHLGLAINLVTYEDRFNLYKIEQELGT

EIQPIPQNIDRGLYVAPSGSEEHEKAQQQQPKAPQPQQRQGQVMYSSGQPQQGRQQQNGAVPPHHPRGQHSYRGGVPVVR

>KAE8215730.1 hypothetical protein CF327_g986 [Tilletia walkeri]

MSTSATSSSQSSLQPGPGQSDAQWRQQVMAHIPKDDRPQTQDVTATKGNDFEDYFLKRELLMGIFEAGFEKPSPIQEEAI

PIALTGRDILARAKNGTGKTAAYVIPTLERLNTKKNKIQAVLLVPTRELALQTSQVAKTLGKHLGVEIMVTTGGTTLKDD

ILRLSQVVHVLVGTPGRILDLASKGVADLSQCTTFVMDEADKLLSPEFTPVMEQLLSLLPQERQVMLFSATFPLIVKDFK

DKHMVKPYEINLMDELTLRGVTQYYAFVEERQKVHCLNTLFSKLQINQSIIFCNSTNRVELLAKKITELGYSCFYSHAKM

LQAHRNRVFHDFRNGACRNLVCSDLLTRGIDIQAVNVVINFDFPKNAETYLHRIGRSGRFGHLGLAINLITYEDRFNLYR

IEQELGTEIQPIPASIDKRLYVAPGLINAVEQQALKNGANGSSRGQGGNNGEQSGNGSQLGGGGIQAGRPVIPPTQAAMH

ANAIPQNESFTQSVPGRYRGGGQQQRAGGQQGQPSA

>XP_002174323.1 ATP-dependent RNA helicase Ste13 [Schizosaccharomyces japonicus yFS275]

MTQNLVQQLAGTTLNDSAESWKSQLQTPGVDLRPKTEDVTNTRGTEFEDYYLKRELLMGIFEAGFERPSPIQEESIPIAL

AGRDILARAKNGTGKTAAFVIPSLEKVDTKKSKIQTLILVPTRELALQTSQVCKTLGKHMNVNVMVTTGGTTLRDDIIRL

NEPVHIVVGTPGRVLDLAGKGVADFSECSNFVMDEADKLLSPEFTPIIEQLLTHFPKNRQISLYSATFPLLVKSFMDKHL

NKPYEINLMDELTLRGVTQYYAFVDERQKVHCLNTLFSKLQINQSIIFCNSTNRVELLAKKITELGYSCFYSHAKMLQSH

RNRVFHNFRNGVCRNLVCSDLLTRGIDIQAVNVVINFDFPKNAETYLHRIGRSGRFGHRGLAISFISWEDRFNLYKIETE

LGTEIQPIPATIDPSLYVFPDGEYQVPRPLSVSQFPNTNLNPNQPIESQPRNYGNNSRTRRGRNYYRGNGQQRSGNSGGK

QQQRKPTYPDGNVAITQH

>KAF7725646.1 DExD/H-box ATP-dependent RNA helicase dhh1 [Apophysomyces ossiformis]

MSQPAITQQLSHENDNWKKSITLPPKDNRPQTEDVTATKGNEFEDYFLKRELLMGIFEAGFERPSPIQEEAIPIALTGRD

ILARAKNGTGKTAAFVIPTLEKINNKKSKVQALLLVPTRELALQTSQVCKNLGKHLGIQVMVTTGGTTLKDDIMRLSDAV

HIIVGTPGRILDLASKGVADFSEASTFVMDEADKLLSPEFTPIIEQLIQFFPKDRQIMLFSATFPMVVKHFKDKFLVKPY

EINLMDELTLRGVTQYYAYVEEKQKVHCLNTLFSKLQINQSIIFCNSTNRVELLAKKITELGYSCFYSHAKMLQSHRNRV

FHDFRNGVCRNLVCSDLLTRGIDIQAVNVVINFDFPKNAETYLHRIGRSGRFGHLGLAINLITYEDRFNLYKIERELGTE

IQAIPPVIDKNLYVAPSALEDAQVQQPDRQAAAAARQHQQQQEQRPQEEQHRSHMNGRQRGGGQNGGGGRRGHHHNHGGG

GRPRHAQQQKTRQQM

>XP_009268120.1 ATP-dependent RNA helicase dhh1 [Wallemia ichthyophaga EXF-994]

MASTSQTAESEWKNQLRVPQKDLRPQTEDVTKTKGGEWEDFGLRRELLMGIFEAGFEKPSPIQEEAIPSAIAGRDILARA

KNGTGKTASFVIPSLEKINVQKPKIQALLLVPTRELALQTSQVCKSLGKHLGIQVMVTTGGTTLRDDIMRLSDPVHILVG

TPGRILDLAGKGVANLNECPTFVMDEADKLLSPEFTPVMEQLLSHLPSSRQVMLFSATFPLIVKDFKEKHMKDPHEINLM

DELTLRGITQYYAFVDERQKVHCLNTLFSKLQINQSIIFCNSTNRVELLAKKVTELGYSCFFSHAKMLQSHRNRVFHDFR

SGVCRNLVCSDLLTRGIDIQAVNVVINFDFPKNSETYLHRIGRSGRYGHLGLAINLITYEDRFNLYKIEQELGTEIQPIP

SQIDRSLYVAPNASDDDGQIQQSQQQQKGLPTLPPTQQVMNAYSQPPPQPTFNQSNQNHQYQQRSNRGGYNSGYYGGSGF

RGRGGGPPRGGVPRA

>KIM91499.1 hypothetical protein PILCRDRAFT_669 [Piloderma croceum F 1598]

MSQSTRPSSSSSGPAESWKTGLRPPPKDIRPQTEDVTATKGTEFEDMFLGRELLMGIFEAGFEKPSPIQEEAIPIALSKR

DILARAKNGTGKTAAFVIPSLQQIDVSKSKIQALLLVPTRELALQTSQVCKILGKHMGIQVMVTTGGTTLKDDILRLSDA

VHVLVGTPGRILDLAGKNVADLSECPVFVMDEADKLLSPEFAPVMEQLLAFMPEERQVMLFSATFPMIVKDFKDKHMNSP

YEINLMDELTLRGVTQYYAYVEERQKVHCLNTLFSKLQINQSIIFCNSTNRVELLAKKVTELGYSCFYSHAKMLQSHRNR

VFHDFRNGVCRNLVCSDLLTRGIDIQAVNVVINFDFPKNSETYLHRIGRSGRFGHLGLAINLVTYEDRFNLYRIEQELGT

EIQPIPQTIDKGLYVAPSAAEEPAGQKPSTSSKQTQQQQQQQPRQTSASQPRGQQQQGQGQAVSQTNGQQGRQNGQASRG

GHAGGRGGASSNR

>EPS95261.1 hypothetical protein FOMPIDRAFT_101552 [Fomitopsis pinicola FP-58527 SS1]

MAQQARPSSSTSDGWKAGLRPPPKDSRPQTEDVTNTKGIEFEDMYLRRELLMGVFEAGFERPSPIQEEAIPIALAKRDVL

ARAKNGTGKTAAFVIPSLQQIDVNKNKIQALLLVPTRELALQTAHVCKVLGKHMGAQIMVTTGGTTLKDDIIRLSETVHV

LVGTPGRILDLAGKNVADLSECPVFVMDEADKLLSPEFAPVMEQLLSYLPKGRQVMLFSATFPMIVKDFKDKHMKQPYEI

NLMDELTLRGVTQYYAYVEERQKVHCLNTLFSKLQINQSIIFCNSTNRVELLAKKVTELGYSCFYSHAKMLQSHRNRVFH

DFRNGVCRNLVCSDLLTRGIDIQAVNVVINFDFPKNSETYLHRIGRSGRFGHLGLAINLVTYEDRFNLYKIEQELGTEIQ

PIPQVIDKGLYVAPTSVDEPEQQQQQQKARAQAQHAPQQRQAAPQPTLPQQQAGAQPQVVYSSSPRPAANGNGVAPTAQR

AQYRGVPVAR

>TIB60458.1 hypothetical protein E3P78_03144 [Wallemia ichthyophaga]

MMDPFSLGVNLSPVVLKTLVRHLIKKRNNNTNKSETPSTPNDEYAFDQTFKLTRAFLDIAMNHPVEDLQSLGSTYIPPPF

WVHNVAVTVPMSIINNAAEYLIKAFGGEQPMRAAVGGTRWWQVRPTPGLPSEWICMKSDVKLATRNNKPNKPREQPKDSF

TPQFFKDIEGLPRTILFVHGGAYYWGSINTHRYLIWRMARKMRGKAFAVSYRLSPQYGFPCALQDVLSAYLYLLYPPEGA

PHKAVDPSNLIFAGDSAGGGLLLSLLLIIRDLGLPLPAGSIHISPWCDLSHSFPSVLSNYETDVIPKYGFVHKPSTLWPP

PSEELLQRTRQHIKSSTQRSKSPLRGVGSAASRKAEEGRIEEDAINEAAGMTNERGHEKLEPPRDVPTVDLDGRTVELKG

QIQMYATNEQVGHPYVSPVLAHLGGLCPLFIMASDGELLRDEGIYMAHKAARPDDFEYKEDAKRRLPSMNGIEGRFGPTK

VHLQVYDKMCHDLPLFSMVTPAKYAYRAMAAFCFQVTKDTTVQPSPALQGSDIPNTMSDLELDSAAESPSPLRKLDSPAE

APSPSTKLDSPADVPSPSRKIDNPAIALLPPPPSPGKPKRKFSLLGLKNNEGAGATSIDMEKAATVSSQTGTPASDAERP

IKSGPGTAGNMAVYDESMAFKERNMIRERVSLVGQIRPMESSENMDILRIPADEVGVFKPAAVKRYLEGKNKWMHKFKHT

AKNVERQREKSLKESNIHAEKKIKDSDNIFDTPQVTWRWQWALENERPPPASIVARRDTQEARKLVQIVDNADSKQNSRT

LSLWTLVMHLMSPQKSKASTNDDGDDVAPWTKDIEKLKRKDGKKDEGQTVSESDSPEEEEINIDKRKEQWLKNHVKDEYW

LWLSDDTLDKRSGKWIVESAFHIYHHEWLQLRKRRRQEISNTAHKAHPKTSAESEWKNQLRVPQKDLRPQTEDVTKTKGG

EWEDFGLRRELLMGIFEAGFEKPSPIQEEAIPSAIAGRDILARAKNGTGKTASFVIPSLEKINVQKPKIQALLLVPTREL

ALQTSQVCKSLGKHLGIQVMVTTGGTTLRDDIMRLSDPVHILVGTPGRILDLAGKGVANLNECPTFVMDEADKLLSPEFT

PVMEQLLSHLPSSRQVMLFSATFPLIVKDFKEKHMTDPHEINLMDELTLRGITQYYAFVDERQKVHCLNTLFSKLQINQS

IIFCNSTNRVELLAKKVTELGYSCFFSHAKMLQSHRNRVFHDFRSGVCRNLVCSDLLTRGIDIQAVNVVINFDFPKNSET

YLHRIGRSGRYGHLGLAINLITYEDRFNLYKIEQELGTEIQPIPSQIDRSLYVAPNASDDDGQIQQSQQEQKGLPTLPPT

QQVMNAYSQPPPQPTFNQSNQNHQYQQRSNRGGYNSGYYGGSGFRGRGGGPPRGGVPRA

>KIY70674.1 DEAD-domain-containing protein [Cylindrobasidium torrendii FP15055 ss-10]

MNPASSSSTPAPNSRPPSSHPSAANKTPSEWKQQLHAPPKDMRPQTEDVTATKGTEFEDMFLRRELLMGIYEAGFERPSP

IQEEAIPVALTGRDVLARAKNGTGKTAAFVIPSLEKVDISKSKIQALLLVPTRELALQTSQVCKMLSKHMGINVMVTTGG

TTLKDDIMRLSDAVHVLVGTPGRILDLASKGVADISQCPVFVMDEADKLLSPEFSPVIEQLLSFMPNNRQVMLFSATFPL

IVKDFKDKHMKTPHEINLMDELTLRGVTQYYAYVEERQKVHCLNTLFSKLQINQSIIFCNSTNRVELLAKKITDLGYSCF

YSHAKMLQSHRNRVFHDFRQGHCRNLVCSDLLTRGIDIQAVNVVINFDFPKNSETYLHRIGRSGRFGHLGIAINLVTYED

RFSLYRIEQELGTEIQPIPASIDRSLYVAPQGGDEQAQQPHQQQVANGTAVARR

>KAE8199086.1 hypothetical protein CF336_g1371 [Tilletia laevis]

MSSSTSQQQSSQSSLQPQPGQSDAQWRQQVMAHIPKDDRPQTQDVTATKGNDFEDYFLKRELLMGIFEAGFEKPSPIQEE

AIPIALTGRDILARAKNGTGKTAAYVIPTLERLNTKKNKIQSVLLVPTRELALQTSQVAKTLGKHLGVEIMVTTGGTTLK

DDILRLSQVVHVLVGTPGRILDLASKGVADLSQCTTFVMDEADKLLSPEFTPVMEQLLSLLPQERQVMLFSATFPLIVKD

FKDKHMVKPYEINLMDELTLRGVTQYYAFVEERQKVHCLNTLFSKLQINQSIIFCNSTNRVELLAKKITELGYSCFYSHA

KMLQAHRNRVFHDFRNGACRNLVCSDLLTRGIDIQAVNVVINFDFPKNAETYLHRIGRSGRFGHLGLAINLITYEDRFNL

YRIEQELGTEIQPIPASIDKRLYVAPGLINAVEQHQLKNGANGGNSRGQGSSGEQSGNGAAGGGGIQAGRPVIPPTQAAM

HANAIPQNESFTQSVPGRYRGGGQQRAGGQQGQSSA

>ODQ73725.1 hypothetical protein LIPSTDRAFT_51663, partial [Lipomyces starkeyi NRRL Y-11557]

WKSQINRPKKDIRPQTEDVTDTKGSEFEDYYLKRELLMGIFEAGFEKPSPIQEESIPVALAGRDILARAKNGTGKTAAFV

IPALEKVNPKSDKIQALILVPTRELALQTSQVCKTLGKHLSINVMVTTGGTTLKDDIVRLHDTVHIMVGTPGRILDLAGK

GVADFSECPMFIMDEADKLLSPEFTPIIEQLLSHFHPSRQILLFSATFPLVVKSFMDKYLTKPYEINLMDELTLRGITQY

YAFVEEKQKVHCLNTLFSKLQINQSIIFCNSTNRVELLAKKITELGYSCFYSHARMLQAHRNRVFHDFRNGVCRNLVCSD

LLTRGIDIQAVNVVINFDFPKNAETYLHRIGRSGRFGHLGLAINLINWDDRFNLYKIEQELGTEIAPIPHTIDKRLYVYD

SPETIPRPQIPQQPQPPQQQQNHQQQLQQQQQQQQQQQQQQQRQQRTPQQRSHGGHPRQQGPRLQNRRN

>TFK38080.1 P-loop containing nucleoside triphosphate hydrolase protein [Crucibulum laeve]

MSHAPSTSSTPSNNNESWKSGLRPPPKDSRPQTEDVTATKGMEFEDMFLRRELLMGIFEAGFEKPSPIQEEAIPIALTKR

DILARAKNGTGKTAAFVIPSLQQVDVNKPKIQALLLVPTRELALQTAQVCKILGKHMGAQVMVTTGGTTLKDDILRLSEN

VHVLVGTPGRILDLAGKGVADLSECPVFVMDEADKLLSPEFAPVMEQLLAYLPTERQVMLFSATFPMIVKDFKDKHMNSP

YEINLMDELTLRGVTQYYAYVEERQKVHCLNTLFSKLQINQSIIFCNSTNRVELLAKKVTELGYSCFYSHAKMLQSHRNR

VFHDFRNGVCRNLVCSDLLTRGIDIQAVNVVINFDFPKNSETYLHRIGRSGRFGHLGLAINLVTYEDRFNLYKIEQELGT

EIQPIPQTIDRGLYVAPSAPEETPAQKQAMRQQQQQQVQAAQRNGQPHVQPQQQQQRANGQQQQQQRGQGGYRGGAPPVA

R

>KAF5381300.1 hypothetical protein D9615_008316 [Tricholomella constricta]

MNKPSAAQVDRKERAERMAYERSVSGIQHTNHTTSGYEIPGRPVVPDGLYPVDYDDLCNLSEGRADILMDCFGLEPTFSG

ILYDDLRDKVLAIATFMGAQMRDEWWAEFHEWNTEREKELEEEAARKYQSECEGREDCEVGPPPPPISFPSARRDGVQCS

AHGWCSRLAVVVVVVVTGLVVMVMVGSRALREPFWVMFVDCPSSGVLQGSTNLTPEHFEHAQSQPHLEPLSSLVAFIAGK

SILKPPSSHTAVMSQTGPSTSSAPAHNESWKASLRPPPKDIRPQTEDVTATKGLEFEDMFLRRELLMGIFEAGFEKPSPI

QEEAIPIALTKRDILARAKNGTGKTAAFVIPSLQQVDVNKNKIQALLLVPTRELALQTSQVCKTLGKHMGVQVMVTTGGT

TLKDDILRLSETVHVLVGTPGRILDLAGKGVADLSECPVFVMDEADKLLSPEFAPVMEQLLSYLAAERQVMLFSATFPMI

VKDFKDKHMKSPYEINLMDELTLRGVTQYYAYVEERQKVHCLNTLFSKLQINQSIIFCNSTNRVELLAKKVTELGYSCFY

SHAKMLQSHRNRVFHDFRNGVCRNLVCSDLLTRGIDIQAVNVVINFDFPKNSETYLHRIGRSGRFGHLGLAINLVTYEDR

FNLYRIEQELGTEIQPIPQTIDKGLYVAPSTPEETASQKQQQQQRQQGTTPQPRAPQQTQSQQVVYQSNGQQPQSVRQNG

AAALPRGQGGYRGAVPVAR

>PWW80580.1 putative ATP dependent RNA helicase [Tuber magnatum]

MATALADQLEATSLGGGQDPNWSSKLKAPKKDLRPQTEDVTATKGLEFEDLYIKRELLMGIFEAGFEKPSPIQEETIPVA

LTGRDMLARAKNGTGKTAAFVIPALERVNPKSTKTQALILVPTRELALQTSQVCKSLGKHLGVNVMVTTGGTGLKDDIIR

LNEPVHILVGTPGRILDLAGKAVADFSECPTFIMDEADKLLSPEFTPIIEQLLAYFPSDRQIMLFSATFPLVVKSFMDKH

LNKPYEINLMDELTLRGVTQYYAFVEEKQKVHCLNTLFSKLQINQSIIFCNSTNRVELLAKKITELGYSCFYSHAKMLQN

HRNRVFHDFRGGVCRNLVCSDLLTRGIDIQAVNVVINFDFPKNAETYLHRIGRSGRFGHLGLAINLINWDDRFNLYKIEQ

ELGTEIFPIPSVIDKKLYVYDSPENIPRPPPTPQQPRQETREHNQRQSTQHQNQNSQQQYQSRQNQHHTENPGNPQQQRP

MNHRQQQGPRNYQNSGPHRGPXWAGTRAESAPPQI

>PCH35393.1 DEAD-domain-containing protein [Wolfiporia cocos MD-104 SS10]

MAQQARPSSSTSRSDAWKAGLRPPPKDGRPQTEDVTATKGVEFEDMFIRRELLMGIFEAGFERPSPIQEEAIPMALTKRD

ILARAKNGTGKTAAFVIPSLQQIDITKNKIQALLLVPTRELALQTSQVCKNLGKHMGVQVMVTTGGTTLKDDIIRLSETV

HVLVGTPGRILDLAGKNVADLSECPVFVMDEADKLLSPEFAPVMEQLLSFMPKDRQVMLFSATFPMIVKDFKDKHMRSPY

EINLMDELTLRGVTQYYAYVEERQKVHCLNTLFSKLQINQSIIFCNSTNRVELLAKKVTELGYSCFYSHAKMLQSHRNRV

FHDFRNGVCRNLVCSDLLTRGIDIQAVNVVINFDFPKNSETYLHRIGRSGRFGHLGLAINLVTYEDRFNLYKIEQELGTE

IQPIPQHIDKGLYVAPGATEESSEQKTRPAQGQGQAQPQRQQTPAQSATPQPTQAQVVYTSAPAARPNGNAAPQAQQPQR

AAYQASTYRGGVPVAR

>XP_007367461.1 eukaryotic translation initiation factor 4A-like protein [Dichomitus squalens LYAD-421 SS1]

MAQQPRPSSSTSHTENWKTGLRPPPKDYRPQTEDVTATKGVEFEDMFLRRELLMGIFEAGFERPSPIQEEAIPIALTKRD

VLARAKNGTGKTAAFVIPSLQQVDVSKNKIQALLLVPTRELALQTAQVCKTLGKHMGIQVMVTTGGTTLKDDIIRLSEAV

HVLVGTPGRILDLAGKGVADLSECPVFVMDEADKLLSPEFAPVMEQLLSYLPKTRQVMLFSATFPMIVKDFKEKHMKSPY

EINLMEELTLRGVTQYYAYVEERQKVHCLNTLFSKLQINQSIIFCNSTNRVELLAKKVTELGYSCFYSHAKMLQSHRNRV

FHDFRNGVCRNLVCSDLLTRGIDIQAVNVVINFDFPKNAETYLHRIGRSGRFGHLGLAINLVTYEDRFNLYKIEQELGTE

IQPIPQQIDKGLYVAPTGPTEDNTEQQQKGKAVAQQPQPQPQPAQRQAHQPVPQVQTRQQASPQVMYQSGPPQQRVNGGP

VPQQQPYPNGYRGVPVAR

>XP_001881258.1 predicted protein [Laccaria bicolor S238N-H82]

MDSWKSTLRPPPKDIRPQTEDVTATKGIEFEDMFLRRELLMGIFEAGFEKPSPIQEEAIPIALTKRDILARAKNGTGKTA

AFVIPSLQQIDITKNKIQALLLVPTRELALQTSQVCKTLGKHMGVQVMVTTGGTTLKDDILRLSESVHVLVGTPGRILDL

AGKNVADLTECPVFVMDEADKLLSPEFAPVMEQLLSYLPTERQVMLFSATFPMIVKDFKDKHMNSPYEINLMDELTLRGV

TQYYAYVEERQKVHCLNTLFSKLQINQSIIFCNSTNRVELLAKKVTELGYSCFYSHAKMLQSHRNRVFHDFRNGVCRNLV

CSDLLTRGIDIQAVNVVINFDFPKNSETYLHRIGRSGRFGHLGLAINLVTYEDRFNLYKIEQELGTEIQPIPQNIDRGLY

VAPSGSEEQEKAQQQQQQPKAAQPQQRQANVLNSNSSRVKRR

>XP_012177496.1 predicted protein [Fibroporia radiculosa]

MAQQARPSSSTSDAWKADLRPPPKDDRPQTEDVTATKGIEFEDMFLRRELLMGIFEAGFEKPSPIQEEAIPIALTKRDVL

ARAKNGTGKTAAFVIPSLQQIDVNKNKIQALLLVPTRELALQTSQVCKILGKHMGIQVMVTTGGTTLKDDIMRLSETVHV

LVGTPGRILDLAGKNVADLSECPVFVMDEADKLLSPEFAPVMEQLLSYISKDRQVMLFSATFPLIVKDFKDKYMRSPYEI

NLMDELTLRGVTQYYAYVEERQKVHCLNTLFSKLQINQSIIFCNSTNRVELLAKKVTELGYSCFYSHAKMLQSHRNRVFH

DFRNGVCRNLVCSDLLTRGIDIQAVNVVINFDFPKNSETYLHRIGRSGRFGHLGLAINLVTYEDRFNLYKIEQELGTEIQ

PIPQQIDKGLYVAPGTTEEPTEQKVRAVQNQRQPTPAQIASTTQQSSPAQVVYSSNPQPPRPQNGSAIPSTQARAPYQAY

RGGVPVAR

>XP_023471347.1 DEAD-domain-containing protein [Rhizopus microsporus ATCC 52813]

MSSSSQLNHETENWKTALTLPPKDSRPQTEDVTATKGNEFEDYFLKRELLMGIFEAGFERPSPIQEEAIPLALAGRDILA

RAKNGTGKTAAFVIPTLEKITNTPKIQALILVPTRELALQTSQVCKTLGKHLNIQVMVTTGGTTLKDDIMRLSETVHIVV

GTPGRILDLASKGVADFSAAHTFVMDEADKLLSPEFTPIIEQLLDYFPKNKQIMLFSATFPMIVKNFKDKYLVKPYEINL

MDELTLRGVTQYYAYVEEKQKVHCLNTLFSKLQINQSIIFCNSTNRVELLAKKITELGYSCFYSHAKMLQSHRNRVFHDF

RNGVCRNLVCSDLLTRGIDIQAVNVVINFDFPKNAETYLHRIGRSGRFGHLGLAINLITYEDRFNLYKIERELGTEIQPI

PPVIDKSLYVAPNALEDAQIQQPNKQQANATRQHQQSTEQQDQTQQQVVNGQQPLQGQQQQQQQQRQYYSHQQRHPHYHN

RSNNWRGRGRGGGHHHSNRSTQKQQPYQQ

>PPQ66800.1 hypothetical protein CVT26_009647 [Gymnopilus dilepis]

MQLSTADNPLTCALPQNLHNPPAARAIERFAINGNAIVTGGAGGLGLEAARALLEHGAKGLAIFDLPEALAKHHDRIRAM

HHDFQQVKVLEVPVDVSNEEEVMKAVEKTVGELGGDVRVLACFAGIARGGDDREMSVETWRRVQEVNTMGAWICARTVAK

EMIKQNSGGSIIFAASIAAHRAVYPLHQTVYNVSKAGLLQLTQSLAAEWARYGIRVNSVSPGFMDTAMTQGEGPAPGVRV

WEERCPMGRQGAVHEIAGPVVMLASEAGRPSNTNSDSWKSGLRPPPKDVRPQTEDVTKTKGLEWEDMYLRRELLMGIFEA

GFERPSPIQEEAIPIALSKRDILARAKNGTGKTAAFVIPSLQQIDINKPKIQALLLVPTRELALQTSQVCKILGKHMGLQ

VMVTTGGTTLKDDILRLSETVHVLVGTPGRILDLAGKGVADLSECPVFVMDEADKLLSAEFAPVMEQLLSFLPADRQVML

FSATFPLIVKDFKEKHMKSPYEINLMDELTLRGVTQYYAYVEERQKVHCLNTLFSKLQINQSIIFCNSTNRVELLAKKVT

ELGYSCFYSHAKMLQSHRNRVFHDFRNGVCRNLVCSDLLTRGIDIQAVNVVINFDFPKNSETYLHRIGRSGRFGHLGLAI

NLVTYEDRFNLYKIEQELGTEIQPIPQNIDRGLYVAPGGGEESQVQRAPQKPVQQQQGQGQQQQRNGPAPHPQLPQAPQQ

QQLTQLQQQQLQSLTPQQRQQLLLQQQMMHQQQQQQLLAQQAAQAQAQGQVPRGQGVYRGGPGVPVARKRAKERRQSTCR

PIFLVTCLLFALISGHWLSAVIELYKAFIRPRDVGYCVAPYAKTPAEIAFLDLKNPYNIAGSVFFVLTLVFGDWFLVYRL

FIIWARNVFIIIPPVMMSTGDLVAYIFSRADEPYYVVAKPWITALYAVSVACNIYSTSMITLKLISPGAQVHFRGVRRKV

VEVLVQSAALYCLLAILILSFSLASTNLLFITTSLSVAVHQTNPVVPFLRLVSAQGINFCLIVIRTHNQPSDTVCVVETG

TDEIQFTETDVSDAHSQA

>TFK49147.1 eukaryotic translation initiation factor 4A-like protein [Heliocybe sulcata]

MAQQSRPSSSASPNSTANDWKAGLRPPPKDSRPQTEDVTATKGLEFEDMYLRRELLMGIFEAGFERPSPIQEEAIPVALT

KRDVLARAKNGTGKTAAFVIPSLQQIDVNKNKIQALLLVPTRELALQTSQVCKILGKHMGVQVMVTTGGTTLKDDIMRLS

EVVHVLVGTPGRVLDLAGKGVADLSECPVFVMDEADKLLSPEFTPVVEQLLSYLSKERQVMLFSATFPLIVKDFKDKHMS

SPYEINLMDELTLRGVTQYYAYVEERQKVHCLNTLFSKLQINQSIIFCNSTNRVELLAKKITELGYSCFYSHAKMLQSHR

NRVFHDFRNGVCRNLVCSDLLTRGIDIQAVNVVINFDFPKNSETYLHRIGRSGRFGHLGLAINLVTYEDRFSLYKIEQEL

GTEIQPIPPSIDKRLYVAPGASADGSEQNQKSSANGQQQQAQQPRPQAVQQGPARPPQQSPAPSQVLYQSNAAQSQQARP

NGAGSAPQQQRAYPAGQVPTPAGYRGGVAVPR

>RPD69405.1 eukaryotic translation initiation factor 4A-like protein [Lentinus tigrinus ALCF2SS1-7]

MSQQARPSSSTSQAESWKAGLRPPPKDYRPQTEDVTATKGVEFEDMFIRRELLMGIFEAGFERPSPIQEEAIPIALTKRD

VLARAKNGTGKTAAFVIPSLQQVDVTKNKIQALLLVPTRELALQTAQVCKTLGKHMGVQVMVTTGGTTLKDDIMRLSETV

HVLVGTPGRILDLAGKNVADLSECPVFVMDEADKLLSPEFAPVMEQLLSYLPKDRQVMLFSATFPMIVKDFKDKHMKSPY

EINLMDELTLRGVTQYYAYVEERQKVHCLNTLFSKLQINQSIIFCNSTNRVELLAKKVTELGYSCFYSHAKMLQSHRNRV

FHDFRNGVCRNLVCSDLLTRGIDIQAVNVVINFDFPKNSETYLHRIGRSGRFGHLGLAINLVTYEDRFNLYKIEKELGTE

IQPIPQQIDKGLYVAPTGAAEDEQQPQQQQQQQQKAKAAPQPQPQPQPAPAAAPANGPRPGPAQAAASAQARQGPAQVVY

QSGPQQRINGAAIPQQAAFQNGYRGVPVQR

>RPD61436.1 eukaryotic translation initiation factor 4A-like protein [Lentinus tigrinus ALCF2SS1-6]

MSQQARPSSSTSQAESWKAGLRPPPKDYRPQTEDVTATKGVEFEDMFIRRELLMGIFEAGFERPSPIQEEAIPIALTKRD

VLARAKNGTGKTAAFVIPSLQQVDVTKNKIQALLLVPTRELALQTAQVCKTLGKHMGVQVMVTTGGTTLKDDIMRLSETV

HVLVGTPGRILDLAGKNVADLSECPVFVMDEADKLLSPEFAPVMEQLLSYLPKDRQVMLFSATFPMIVKDFKDKHMKSPY

EINLMDELTLRGVTQYYAYVEERQKVHCLNTLFSKLQINQSIIFCNSTNRVELLAKKVTELGYSCFYSHAKMLQSHRNRV

FHDFRNGVCRNLVCSDLLTRGIDIQAVNVVINFDFPKNSETYLHRIGRSGRFGHLGLAINLVTYEDRFNLYKIEKELGTE

IQPIPQQIDKGLYVAPTGAAEDEQQPQQQQQQQQKAKAAPQSQPQPQPQPAPAAAPANGPRPGPAQAAASAQARQGPAQV

VYQSGPQQRINGAAIPQQAAFQNGYRGVPVQR

>KZP33755.1 DEAD-domain-containing protein, partial [Fibularhizoctonia sp. CBS 109695]

MAQPARPSSSSSGPSDSWKSGLRPPPKDIRPQTEDVTATKGIEFEDMFLGRELLMGIFEAGFERPSPIQEEAIPVALTKR

DILARAKNGTGKTAAFVIPSLQQIDVSKNKIQALLLVPTRELALQTSQVCKILGKHMGAQVMVTTGGTTLKDDILRLSET

VHVLVGTPGRILDLAGKNVADLSECPVFVMDEADKLLSPEFAPVMEQLLSFLPEERQVMLFSATFPMIVKDFKDTHMRSP

YEINLMDELTLRGVTQYYAYVEERQKVHCLNTLFSKLQINQSIIFCNSTNRVELLAKKVTELGYSCFYSHAKMLQSHRNR

VFHDFRNGVCRNLVCSDLLTRGIDIQAVNVVINFDFPKNSETYLHRIGRSGRFGHLGLAINLVTYEDRFNLYRIEQELGT

EIQPIPATIDKGLYVAPGASAPEESTVAQKQAAAAKQVQAQAHQQPRQATPSQAQPRAAQPSAQGQVVYQSNGQPGRGAN

GQAARGGRGAA

>KAF4583520.1 DExD/H-box ATP-dependent RNA helicase dhh1 [Pleurotus pulmonarius]

MAQTATSSSSPANANDNWKATLRPPPRDSRPQTEDVTATKGTEFEDMFLRRELLMGIFEAGFERPSPIQEEAIPIALTHR

DILARAKNGTGKTAAFVIPSLQQVDVSKPKIQALLLVPTRELALQTSQVCKILGKHMGVQVMVTTGGTTLKDDILRLSES

VHVLVGTPGRILDLAGKNVADLSECPVFVMDEADKLLSPEFTPVMEQLLSFLPKERQVMLFSATFPMIVKDFKDKYMNKP

YEINLMDELTLRGVTQYYAYVEERQKVHCLNTLFSKLQINQSIIFCNSTNRVELLAKKVTELGYSCFYSHAKMLQSHRNR

VFHDFRNGVCRNLVCSDLLTRGIDIQAVNVVINFDLPKSSETYLHRIGRSGRFGHLGLAINLVTYEDRFNLYKIEQELGT

EIQPIPQVIDKGLYVAPGLGAEEQQAAQQQQAQQQARALAQQRQQQVQQVAGVAGPARPPQQQQPQQQGQIVYQSNGQPP

ARQNGTSRGGTPNPNPYRGTPVPAAQSARS

>TPX75899.1 hypothetical protein CcCBS67573_g02825 [Chytriomyces confervae]

MAMELAKVIGDAHPKTILCSSFNPFRREIYTGGEDATIRVWEAESGKLLNVLTEHVGWVTNLLFCKELKVLFSASIDGFI

IAWGPSGKVLQKIATGSPIYCMAYNSRRQKLLAGYNKRIRVFQMISPEDSHLSSEVLERKAVSCSEHSDVVSCLESLVIR

YDRKIVIYDIPHHGDLKMTASKTISNAHDAAISCMVFGKDADNSWLITGSFDRIVKLWSLDGNLLQRFDGFGDTITSICY

VLPTQTLWITANSQTPIVFDPRSGINVSDFIGTSSEEIHQKKNISSFKQITFVPELNEVVGVTNRRAIVVWKYNPAASIT

VLSGHSDSIECLTFTMKEPLLIFSGSSDGLIRKWERLQLNTFMYSQENLHLPKEERHEEEITVNSFSKNPEERRKRQTAL

HRQIVEKLDRWKRGLEEDMNKQDNQIELMSSDAVRAFKRNQTKLKTELQISKTFETDVKTGSAALKTSRQGVFSLFYYEE

MDLLMSGYEDSRIRIWGYNEEAVKYSPDDGEKEAVDQAGLSSENVTNRVSGMSLKHTFNDHKDSVTGISCFHRDGRHWMI

STGWDRRICIYDLKQLRLHDVFRNQQKGYGKEELAADGIIFDLEYSPDRNEFGYASADKSAYIRKFSSKGDEMLLQAVLI

GHEAEVTKIKWNRKHQQWVTGSEDRTIRIWAAEGLPLLNVINNDSPVSALCIDAINGCIIAGSQDKCVRVFDPEKKDEVA

QKNVGHMDEIRAIIHIPVRNQYVTSSLDNTVRIWNAYMKKGQRRVAMSNSKASLSLNANGAAFDAEEDGHMYSELNPLMV

TKLANSAAAAAALRPTGRVTVEDNSEATQGKVLEDELRTTLSDLEFALTAEKTKRIYLKTTRIMNNGVAALTTQLQNTQL

AADDWKKNATAPPKDARPQTSDVTATKGHEFADYFLKRELLMGLYEAGFERPTPIQEESIPIAMTGRDILARAKNGTGKT

ASFIIPVLEKSDPTKNYIQALILVPTRELALQTSQVCKTIGKHCGLEVMVTTGGTTLKDDILRLGQNVHILVATPGRVLD

LAGKGICDLSKCTMMIMDEADKLLSPEFQPVIEQIIGFHPKERQILLFSATFPIVVKAFKDKFLAKPYEINLMDELTLKG

VTQFYAFMEEKQKVHCLNTLFSKLQVNQSIIFCNSTSRVELLAKKITELGYSCFYIHAKMLQAHRNRVFHDFRSGRTRHL

VCSDLLTRGIDIQAVNVVINFDFPKNAETYLHRIGRSGRFGHLGLAINLITYEDRFNLYRIEQELGTEIAPIPPVIDKSL

YVAPGI

>TFK92374.1 DEAD-domain-containing protein [Polyporus arcularius HHB13444]

MSQQARPSSSTSHADSWKAGLRPPPKDYRPQTEDVTATKGVEFEDMYIRRELLMGIFEAGFERPSPIQEEAIPIALTKRD

VLARAKNGTGKTAAFVIPSLQQIDVNKNKIQALLLVPTRELALQTAQVCKTLGKHMGVQVMVTTGGTTLKDDIMRLSEVV

HVLVGTPGRILDLAGKGVADLSECPVFVMDEADKLLSPEFAPVMEQLLSYLPKDRQVMLFSATFPMIVKDFKEKHMKSPY

EINLMDELTLRGVTQYYAYVEERQKVHCLNTLFSKLQINQSIIFCNSTNRVELLAKKVTELGYSCFYSHAKMLQSHRNRV

FHDFRNGVCRNLVCSDLLTRGIDIQAVNVVINFDFPKNSETYLHRIGRSGRFGHLGLAINLVTYEDRFNLYKIEQELGTE

IQPIPQQIDKGLYVAPTGAAEEEPQQQQQPQQQQKAKAGPQPQPQPAPAAPNGPRPGPAQAAASAQARQGPPQVVYQSSP

QQRLNGGAAIPQQQAAYQNGYRGVPVQR

>RCH90092.1 DExD/H-box ATP-dependent RNA helicase dhh1 [Rhizopus azygosporus]

MSSSSQLNHETENWKTALTLPPKDSRPQTEDVTATKGNEFEDYFLKRELLMGIFEAGFERPSPIQEEAIPLALAGRDILA

RAKNGTGKTAAFVIPTLEKITNTPKIQALILVPTRELALQTSQVCKTLGKHLNIQVMVTTGGTTLKDDIMRLSETVHIVV

GTPGRILDLASKGVADFSAAHTFVMDEADKLLSPEFTPIIEQLLDYFPKNKQIMLFSATFPMIVKNFKDKYLVKPYEINL

MDELTLRGVTQYYAYVEEKQKVHCLNTLFSKLQINQSIIFCNSTNRVELLAKKITELGYSCFYSHAKMLQSHRNRVFHDF

RNGVCRNLVCSDLLTRGIDIQAVNVVINFDFPKNAETYLHRIGRSGRFGHLGLAINLITYEDRFNLYKIERELGTEIQPI

PPVIDKSLYVAPNALEDAQIQQPNKQQANATRQHQQSTEQQDQTQQQVINGQQPLQGQQQQQQQRQHYSHQQRHPHYHNR

SNNWRGRGRGGGHHHSNRSTQKQQPYQQ

>NP_596324.1 ATP-dependent RNA helicase Ste13 [Schizosaccharomyces pombe]

MAESLIQKLENANLNDRESFKGQMKAQPVDMRPKTEDVTKTRGTEFEDYYLKRELLMGIFEAGFERPSPIQEESIPIALS

GRDILARAKNGTGKTAAFVIPSLEKVDTKKSKIQTLILVPTRELALQTSQVCKTLGKHMNVKVMVTTGGTTLRDDIIRLN

DTVHIVVGTPGRVLDLAGKGVADFSECTTFVMDEADKLLSPEFTPIIEQLLSYFPKNRQISLYSATFPLIVKNFMDKHLN

KPYEINLMDELTLRGVTQYYAFVDESQKVHCLNTLFSKLQINQSIIFCNSTNRVELLAKKITELGYSCFYSHAKMLQSHR

NRVFHNFRNGVCRNLVCSDLLTRGIDIQAVNVVINFDFPKNAETYLHRIGRSGRFGHRGLAISFISWADRFNLYRIENEL

GTEIQPIPPSIDPSLYVFPNGDYQIPRPLTASADQVLAAQQAKGQEGYHNRPNNNRGGHPRGGGNRGGYRQSNRQPRYRG

QQKAD

>OAD06286.1 hypothetical protein MUCCIDRAFT_118649, partial [Mucor lusitanicus CBS 277.49]

PQKDMRPQTEDVTATKGNEFEDYFLKRELLMGIFEAGFERPSPIQEEAIPIALTGRDILARAKNGTGKTAAFVIPTLEKI

NNKKAKIQALLLVPTRELALQTAQVCKNLGKHLNIQVMVTTGGTTLKDDIMRLSEPVHVVVGTPGRILDLASKNVADFSE

ASTFVMDEADKLLSPEFTPIIDQLISHFPKDRQIMLFSATFPMIVKSFKEKYLVKPYEINLMDELTLRGVTQYYAYVEEK

QKVHCLNTLFSKLQINQSIIFCNSTNRVELLAKKITELGYSCFYSHAKMLQSHRNRVFHDFRNGVCRNLVCSDLLTRGID

IQAVNVVINFDFPKNAETYLHRIGRSGRFGHLGLAINLITYEDRFNLYKIERELGTEIQPIPPVIDKNLYVAPNALEDAQ

VQQPNRQ

>XP_001730716.1 hypothetical protein MGL_2170 [Malassezia globosa CBS 7966]

MANTAPGMESQRPQKDTRPQTEDVTATKGNDFEDFFLKRELLMGIFEAGFEHPSPIQEEAIPIALSGRDVLARAKNGTGK

TAAYVIPTLERVNTKKNKIQAVLLVPTRELALQTSQVAKTLGKHLGIEIMVSTGGTTLKDDILRLGQTVHILVGTPGRIL

DLASKGIADLSQCTTYVMDEADKLLSPEFTPVMEQLLGLLPKERQVMLFSATFPLIVKDFKDKHMVKPYEINLMDELTLR

GVTQYYAFVEERQKVHCLNTLFSKLQINQSIIFCNSTNRVELLAKKITELGYSCFYSHAKMLQAHRNRVFHDFRNGACRN

LVCSDLLTRGIDIQAVNVVINFDFPKNAETYLHRIGRSGRFGHMGLAINLITYEDRFNLYRIEQELGTEVQPIPATIDKR

LYVAPSLIQEAENRERDAQEQGKPTQGPSIMQQGLPAIPPTQAAMHAVPVSQNANFTNTVPNRRGGRRNNRRGNGNRNPN

SRIHNQSTAGNEGHSEGAAPPV

>CEG63343.1 Putative Atp-dependent rna helicase dhh1 [Rhizopus microsporus]

MSSSSQLNHETENWKTALTLPPKDSRPQTEDVTATKGNEFEDYFLKRELLMGIFEAGFERPSPIQEEAIPLALAGRDILA

RAKNGTGKTAAFVIPTLEKITNTPKIQALILVPTRELALQTSQVCKTLGKHLNIQVMVTTGGTTLKDDIMRLSETVHIVV

GTPGRILDLASKGVADFSAAHTFVMDEADKLLSPEFTPIIEQLLDYFPKNKQIMLFSATFPMIVKNFKDKYLIKPYEINL

MDELTLRGVTQYYAYVEEKQKVHCLNTLFSKLQINQSIIFCNSTNRVELLAKKITELGYSCFYSHAKMLQSHRNRVFHDF

RNGVCRNLVCSDLLTRGIDIQAVNVVINFDFPKNAETYLHRIGRSGRFGHLGLAINLITYEDRFNLYKIERELGTEIQPI

PPVIDKSLYVAPNALEDAQIQQPNKQQANATRQHQQSTEQQDQTQQQVINGQQPLQGQQQQQQQRQHYSHQQRHPHYHNR

SNNWRGRGRGGGYHHSNRSTQKQQPYQQ

>KAF3912808.1 hypothetical protein AA313_de0201266 [Arthrobotrys entomopaga]

MAASLAEKLESTTLNDLDWRSQLKTPVKDNRPQTEDVTATKGLDFEELYIKRELLMGIFEAGFEKPSPIQEETIPVALTG

RDILARAKNGTGKTAAFVIPALERINAKSPKTQALILVPTRELALQTSQVCKTLGKHLGINVMVTTGGTGLRDDIVRLNE

AVHVLVGTPGRILDLAGKGVADFSECPTFIMDEADKLLSPEFTPIIEQLLAYFPGDRQIMLFSATFPLVVKSFMDKHLKQ

PYEINLMDELTLRGVTQYYAFVEEKQKVHCLNTLFSKLQINQSIIFCNSTNRVELLAKKITELGYSCFYSHAKMLQNHRN

RVFHDFRNGVCRNLVCSDLLTRGIDIQAVNVVINFDFPKNAETYLHRIGRSGRFGHLGLAINLINWDDRFNLYKIEQELG

TEIQPIPAQIDKKLYVYDSPESIPRPPAAQPFVRSAPSAANSAQQPVSSSRANTTEPSNSSQTNQPQGMRQNKPRGNHSM

NQGYRGGRGSRGNYGHHQVYGRRNDNGQNTDGAARSSQNA

>KIJ36696.1 hypothetical protein M422DRAFT_232268 [Sphaerobolus stellatus SS14]

MSQQSRPSTSNSDWKAGLRTPQKDTRPQTEDVTNTKGLDFEQLGLRRELLMGIFEAGFERPSPIQEEAIPIALTRRDVLA

RAKNGTGKTAAFIIPALQGIDVSKPKIQALILVPTRELALQTSQVCKTLGKHMGAQVMVTTGGTTLKDDILRLSEPVHVL

VGTPGRILDLAGKSVADLSECPVFVMDEADKLLSPEFAPVMEQLLSYLPTENRQVMLFSATFPMIVKDFKEKHMRNPYEI

NLMDELTLKGVTQYYAFVEERQKVHCLNTLFSKLQINQSIIFCNSTNRVELLAKKVTELGYSCFYSHAKMLQSHRNRVFH

DFRNGVCRNLVCSDLLTRGIDIQAVNVVINFDFPKNSETYLHRIGRSGRFGHLGLAINLITYEDRFNLYRIEQELGTEIQ

PIPTQIDKSLYVAPVEVDEPASSQPKAADEERRKQEQAHAQQAQQAQALQAQQQAQQQAQQQAQLQAQAQLQAQQLQAQM

QAQAQLQAQARAQAQAQAQYMQQQRMLQQQQQQQQGNGIPAGAARSNGIPPPQQMAYGRGPAR

>CEP14076.1 hypothetical protein [Parasitella parasitica]

MSSNLSHENDNWKKSLVLPAKDVRPQTEDVTATKGNEFEDYYLKRELLMGIFEAGFERPSPIQEEAIPIALTGRDILARA

KNGTGKTAAFVIPTLEKINNKKNKIQALLLVPTRELALQTSQVCKNLGKHLGLQIMVTTGGTTLKDDIMRLNEPVHVVVG

TPGRILDLASKGVADFSEASTFVMDEADKLLSPEFTPIIEQLIQHFPQDRQIMLFSATFPLIVKTFKDKFLVKPYEINLM

DELTLRGVTQYYAYVEEKQKVHCLNTLFSKLQINQSIIFCNSTNRVELLAKKITELGYSCFYSHARMLQSHRNRVFHDFR

NGVCRNLVCSDLLTRGIDIQAVNVVINFDFPKNAETYLHRIGRSGRFGHLGLAINLITYEDRFNLYKIEQELNTEIQPIP

PVIDKRLYVAPSALEDAQVQQPDRQQALATRQQQQQQGSEEQQQQQQQQPQHHNYNNNRPSNWRGRGRGGGGGGNGHNRS

KQPH

>KAB5590786.1 ATP-dependent RNA helicase [Ceratobasidium theobromae]

MHNLLLYALGLARPAKKDELASQTHVDLYDYGLSGPQGADIVMQQIRRTPFVTHINLGHNPLGDFGLSIIVDYLHREGRD

LPIEELSLNNCDISDSGLGIISRYIWGNRTLRRLYLMGNNISGSKSPIEAFADSLNHSRIQTLVLTNNERLSDRFLVRFL

GNLDAPYLRELQLSRIGLTQSSLPVLKKFLTSPSCYGLRSLHLNANSLSNRGVHNLVDDLLTGNTTLCGMEAFANSSPGS

TVVGSNDSISDEAFLEETLGALKFVLARNSDHLRKAECEAKALLVVARTLLLTQSHPKAPSGGSVFPWRKLMPELQHYIL

RHLHTTLSDAQHTRVCIYASSKATLPSLRPIPRYAKEYIEDYLIAVGCKILLPPRTPPGYIVAIEIATTNNLLHRSKLGS

HPSGESPLVLGRFAAGSIDSNGDWKAGLRAPPKDDRPQTEDVTATKGLEFEDMALRRELLMGIFEAGFEHPSPIQEEAIP

IALTRRDILARAKNGTGKTAAFTIPSLQQVDPTKPKIQAMLLTPTRELALQTAQVCKNLGKHMGINVMVTTGGTTLKDDI

IRLSEAVHVLVGTPGRILDLAGKQVADLSSCRVFVMDEADKLLSPEFTPVMEQLLSFVPGDRQVMLFSATFPMIVKQFKD

KHMKNPHEINLMDELTLRGVTQYYAFVEERQKVHCLNTLFAKLQINQSIIFCNSTNRVELLAKKVTELGYSCFYSHAKMV

QSARNRVFHDFRNGVCRNLVCSDLLTRGIDIQAVNVVINFDFPKHSETYLHRIGRSGRFGHLGLAINLITYEDRFNLYRI

EQELGTEIQPIPAEINKSLYVAPSAMDEPAGQAPSRSDAAESGANGQATHSQSRSQQRGGAPQAQRGVGRGAAPQR

>KAF4564800.1 DExD/H-box ATP-dependent RNA helicase dhh1 [Pleurotus pulmonarius]

MAQTATSSSSPANANDNWKATLRPPPRDSRPQTEDVTATKGTEFEDMFLRRELLMGIFEAGFERPSPIQEEAIPIALTHR

DILARAKNGTGKTAAFVIPSLQQVDVSKPKIQALLLVPTRELALQTSQVCKILGKHMGVQVMVTTGGTTLKDDILRLSES

VHVLVGTPGRILDLAGKNVADLSECPVFVMDEADKLLSPEFTPVMEQLLSFLPKERQVMLFSATFPMIVKDFKDKYMNKP

YEINLMDELTLRGVTQYYAYVEERQKVHCLNTLFSKLQINQSIIFCNSTNRVELLAKKVTELGYSCFYSHAKMLQSHRNR

VFHDFRNGVCRNLVCSDLLTRGIDIQAVNVVINFDLPKSSETYLHRIGRSGRFGHLGLAINLVTYEDRFNLYKIEQELGT

EIQPIPQVIDKGLYVAPGLGAEEQQQAQQQQAQQQARALAQQRQQQVQQAAGVAGPARPPQQQQPQQQGQIVYQSNGQPP

ARQNGTSRGGTPNPNPYRGTPVPAAQSARS

>KAF7426067.1 DExD/H-box ATP-dependent RNA helicase dhh1 [Pleurotus ostreatus]

MAQTATSSSSPANASDNWKATLRPPPRDSRPQTEDVTATKGTEFEDMFLRRELLMGIFEAGFERPSPIQEEAIPIALTHR

DILARAKNGTGKTAAFVIPSLQQVDVSKPKIQALLLVPTRELALQTSQVCKILGKHMGVQVMVTTGGTTLKDDILRLSES

VHVLVGTPGRILDLAGKNVADLSECPVFVMDEADKLLSPEFTPVMEQLLSFLPKERQVMLFSATFPMIVKDFKDKYMNKP

YEINLMDELTLRGVTQYYAYVEERQKVHCLNTLFSKLQINQSIIFCNSTNRVELLAKKVTELGYSCFYSHAKMLQSHRNR

VFHDFRNGVCRNLVCSDLLTRGIDIQAVNVVINFDLPKSSETYLHRIGRSGRFGHLGLAINLVTYEDRFNLYKIEQELGT

EIQPIPQVIDKGLYVAPGLGAEEQQQAQQQQAQQQARALAQQRQQQVQQAAGVAGPARPPQQQQQQPQGQIVYQSNGQPP

ARQNGTSRGGTPNPNPYRGTPVPAAQSARS

>XP_008042303.1 DEAD-domain-containing protein [Trametes versicolor FP-101664 SS1]

MAQQARPSSSTSHSDTWKAGLRPPPKDYRPQTEDVTATKGVEFEDMYLRRELLMGIYEAGFERPSPIQEEAIPIALTKRD

VLARAKNGTGKTAAFVIPSLQQVDVNKNKIQALLLVPTRELALQTAQVCKNLGKHMGVQVMVTTGGTTLKDDIMRLSEEV

HVLVGTPGRILDLAGKGVADLSECPVFVMDEADKLLSPEFAPVMEQLLSYLPKDRQVMLFSATFPMIVKDFKDKHMKSPY

EINLMDELTLRGVTQYYAYVEERQKVHCLNTLFSKLQINQSIIFCNSTNRVELLAKKVTELGYSCFYSHAKMLQSHRNRV

FHDFRNGVCRNLVCSDLLTRGIDIQAVNVVINFDFPKNSETYLHRIGRSGRFGHLGLAINLVTYEDRFNLYKIEQELGTE

IQPIPQQIDKGLYVAPSGASEENGEQQPQQPQKGKGPAQPQPQPQQGHRQAHPQAASPAPGRAGPAQVVYQSQPQAPRAN

GSGAPQQSGPQYQNGYRGVPVAR

>PPQ86614.1 hypothetical protein CVT25_006798 [Psilocybe cyanescens]

MSTAATSSSSTPANNDSSWKTGLRPPPKDIRPQTEDVTATKGTEFEDMFLNRELLMGIFEAGFEKPSPIQEEAIPIALAK

RDILARAKNGTGKTAAFVIPSLQQIDLTKPKIQALLLVPTRELALQTSQVCKILGKHMGLQVMVTTGGTTLKDDILRLSE

AVHVLVGTPGRILDLAGKGVADLSECPVFVMDEADKLLSPEFAPVMEQLLSFLPTDRQVMLFSATFPMIVKDFKEKHMRN

PYEINLMDELTLRGVTQYYAYVEERQKVHCLNTLFSKLQINQSIIFCNSTNRVELLAKKVTELGYSCFYSHAKMLQSHRN

RVFHDFRNGVCRNLVCSDLLTRGIDIQAVNVVINFDFPKNSETYLHRIGRSGRFGHLGLAINLVTYEDRFNLYKVEQELG

TEIQPIPQTIDRGLYVAPSGSEESQVQRPSQQQQQQQQARQQISPAPALGPAPAQRNGQQQQQQQQQQQMPARQNGAPPQ

QPRGQPAYRGGVPVGR

>RPB08547.1 putative DEAD-box RNA helicase Dhh1/Vad1 [Morchella conica CCBAS932]

MATALAEQLESTSLEGQDPDWKSKLKAPKKDLRPQTEDVTATKGLDFEDLYIKRELLMGIFEAGFEKPSPIQEETIPVAL

TGRDILARAKNGTGKTAAFVIPALERVNPKSTKTQALILVPTRELALQTSQVCKTLGKHLGINVMVTTGGTGLKDDIIRL

NEAVHILVGTPGRILDLAGKGVADFSECPTFIMDEADKLLSPEFTPIIEQLLAYFPNDRQIMLFSATFPLVVKSFMDKHL

NKPYEINLMDELTLRGVTQYYAFVEEKQKVHCLNTLFSKLQINQSIIFCNSTNRVELLAKKITELGYSCFYSHAKMLQNH

RNRVFHDFRNGVCRNLVCSDLLTRGIDIQAVNVVINFDFPKNAETYLHRIGRSGRFGHLGLAINLINWEDRFNLYKIEQE

LGTEIQPIPPTIDKKLYVYDSPESIPRPPSTPQPTRQNQGDNQNPQQLQQRNANHAAHNRAPNHGQGRGRGFQHQQGPNQ

GQYNNNNQGFNQQQNRRPGPRYNQNQGQPGGRPNGFGPPDAGPQVRAQ

>OJT14878.1 ATP-dependent RNA helicase dhh1 [Trametes pubescens]

MAQQARPSSSTSHSDTWKAGLRPPPKDYRPQTEDVTATKGVEFEDMYLRRELLMGIYEAGFERPSPIQEEAIPIALTKRD

VLARAKNGTGKTAAFVIPSLQQVDVNKNKIQALLLVPTRELALQTAQVCKNLGKHMGVQVMVTTGGTTLKDDIMRLSEEV

HVLVGTPGRILDLAGKGVADLSECPVFVMDEADKLLSPEFAPVMEQLLSYLPKDRQVMLFSATFPMIVKDFKDKHMKSPY

EINLMDELTLRGVTQYYAYVEERQKVHCLNTLFSKLQINQSIIFCNSTNRVELLAKKVTELGYSCFYSHAKMLQSHRNRV

FHDFRNGVCRNLVCSDLLTRGIDIQAVNVVINFDFPKNSETYLHRIGRSGRFGHLGLAINLVTYEDRFNLYKIEQELGTE

IQPIPQQIDKGLYVAPSGASEENGEQQPQQPQKGKGPAQPQQPQQGHRQAHPQAASPAPGRAGPAQVVYQSQPQAPRANG

SGAPQQSGPQYQNGYRGVPVAR

>OCB86642.1 DEAD-domain-containing protein [Sanghuangporus baumii]

MSTVTSPSASSSTGTPSGQDWRAGLKAPPKDSRPKTEDVTATKGVEFEDMFLRRELLMGIFEAGFERPSPIQEEAIPVAL

TKRDILARAKNGTGKTAAFVIPSLQQLDVTKNRIQALVLVPTRELALQTAQVCKNLGKHLGANVMVTTGGTTLKDDIMRL

SEPVHVLVGTPGRILDLAGKGVADLSECPVFVMDEADKLLSPEFTPVMEQLLSYLPKERQVMLFSATFPIIVKDFKNKHM

DSPYEINLMEELTLRGVTQYYAFVEERQKVHCLNTLFSKLQINQSIIFCNSTNRVELLAKKITDLGYSCFYSHAKMLQSH

RNRVFHDFRNGVCRNLVCSDLLTRGIDIQAVNVVINFDFPKNSETYLHRIGRSGRFGHLGLAINLITYEDRFNLYKIEQE

LGTEIQPIPQQIDRSLYVAPAGGLDESTPSPAPAASSQPQQQSQAQTQKQPQQQPQRQTPQAQVQAPQQVLYQSNGQVPH

PPQQGVPPQLQQRPVANGPMPAQLMQQQQQYAAVMRNAQYPAHGPGGAGPGMVPGPGGPPRVGAQQQPVRR

>RDX42471.1 DEAD-domain-containing protein [Polyporus brumalis]

MSQQARPSSSTSHADSWKAGLRPPPKDYRPQTEDVTATKGVEFEDMYIRRELLMGIFEAGFERPSPIQEEAIPIALTKRD

VLARAKNGTGKTAAFVIPSLQQIDVNKNKIQALLLVPTRELALQTAQVCKTLGKHMGVQVMVTTGGTTLKDDIMRLSEVV

HVLVGTPGRILDLAGKGVADLSECPVFVMDEADKLLSPEFAPVMEQLLSYLPKDRQVMLFSATFPMIVKDFKEKHMKSPY

EINLMDELTLRGVTQYYAYVEERQKVHCLNTLFSKLQINQSIIFCNSTNRVELLAKKVTELGYSCFYSHAKMLQSHRNRV

FHDFRNGVCRNLVCSDLLTRGIDIQAVNVVINFDFPKNSETYLHRIGRSGRFGHLGLAINLVTYEDRFNLYKIEQELGTE

IQPIPQQIDKGLYVAPTGAAEEEPQQQQQQQQQKAKAAPQPQPQPAPAAPNGPRPGPAQAAASAQARQGPPQVVYQSSPQ

QRLNGGAAIPQQQAAYQNGYRGVPVQR

>SJX63582.1 probable ATP-dependent RNA helicase DHH1 [Sporisorium reilianum f. sp. reilianum]

MSTSGPAPGQSEAAWKQQITSKISKDERPQTEDVLNTKGNEFEDYFLKRELLMGIFEAGFERPSPIQEEAIPIALTGRDI

LARAKNGTGKTAAYVIPSLEKLNAKKNKIQAVLLVPTRELALQTSQVAKTLGKHLGVEVMVTTGGTTLRDDILRLGQTVH

MLVGTPGRILDLAGKGVADLSQCTTFVMDEADKLLSPEFTPVMEQLLSFLPKERQVMLFSATFPLIVKDFKDRNMVKPYE

INLMDELTLRGVTQYYAFVEERQKVHCLNTLFSKLQINQSIIFCNSTNRVELLAKKITELGYSCFYSHAKMLQAHRNRVF

HDFRNGACRNLVCSDLLTRGIDIQAVNVVINFDFPKNAETYLHRIGRSGRFGHLGLAINLITYEDRFNLYRIEQELGTEI

QPIPSNIDKRLYVAPSLIQEAEGKNQNGNRQEGRPVIPPGQQAMHAAAIPQNQNFSNTVPHRHRGGGGGGGGQRGGGGRG

GPQGQGGVPAQ

>XP_011389806.1 putative DExD/H-box ATP-dependent RNA helicase DHH1 [Ustilago maydis 521]

MSASGPAPGQSEAAWKQQITSRLSKDERPQTEDVLNTKGNEFEDYFLKRELLMGIFEAGFERPSPIQEEAIPIALTGRDI

LARAKNGTGKTAAYVIPSLEKLNTKKNKIQAVLLVPTRELALQTSQVAKTLGKHLGVEVMVTTGGTTLRDDILRLGQTVH

LLVGTPGRILDLAGKGVADLSQCTTFVMDEADKLLSPEFTPVMEQLLSFLPKERQVMLFSATFPLIVKDFKDRNMVKPYE

INLMDELTLRGVTQYYAFVEERQKVHCLNTLFSKLQINQSIIFCNSTNRVELLAKKITELGYSCFYSHAKMLQAHRNRVF

HDFRNGACRNLVCSDLLTRGIDIQAVNVVINFDFPKNAETYLHRIGRSGRFGHLGLAINLITYEDRFNLYRIEQELGTEI

QPIPSNIDKRLYVAPSLIQEAEGNNQNGNKQEGRPVIPPGQQAMHAAAIPLNQNFTNTVPHRHRSGGGGGRGGGGGGRGG

PHGGVPAQ

>CDS11083.1 Putative ATP-dependent RNA helicase DHH1 [Lichtheimia ramosa]

MSHEEDEWKKSLQLPTRDNRPQTEDVTKTKGNEFEDYFLKRELLMGIFEAGFERPSPIQEESIPIALTGRDILARAKNGT

GKTAAFVIPTLEKVNTKKSQIQALILVPTRELALQTSQVCKTLGKHLGVHVMVTTGGTTLKDDIMRLSEPVHIVVGTPGR

ILDLASKNVADFSEASTFVMDEADKLLSPEFTPVIERLLAYFPQERQIMLFSATFPMVVKSFKDKYLVKPYEINLMEDLT

LKGVTQFYAYVEEKQKVHCLNTLFSKLQINQSIIFCNSTSRVELLAKKITELGYSCFYSHARMLQSHRNRVFHDFRNGVC

RNLVCSDLLTRGIDIQAVNVVINFDFPKNAETYLHRIGRSGRYGHLGLAINLITYEDRFNLYKIERELGTEITPIPAQID

KSLYVAPNALEENMVQQPQPKPLDNNGIQQQQQQQQSDNHHHPHFQHGGGRGGWNGNRGRGGYWSHRGRGTWSNRGRGGA

NNNNHRVKRVPKPLV

>XP_009158872.1 ATP-dependent RNA helicase dhh1 [Exophiala dermatitidis NIH/UT8656]

MTDSLANQLNKTSLNDGAEDPNWKDSLNLPAKDSRQQTEDVTATKGLEFEDFYIKRELMMGIFEAGFEKPSPIQEETIPV

ALTGRDILARAKNGTGKTAAFVIPTLERINPKNPKTQALILVPTRELALQTSQVCKTLGKHLGINVMVTTGGTGLKDDII

RLGEPVHIIVGTPGRILDLASKGVADLSECPIFVMDEADKLLSPEFTVVIEQLLSFLPKDRQVMLFSATFPMIVKSFKDK

HMRNPYEINLMDELTLRGITQYYAFVEEKQKVHCLNTLFSKLQINQSIIFCNSTNRVELLAKKITELGYSCFYSHAKMLQ

QNRNKVFHDFRAGVCRNLVCSDLLTRGIDIQAVNVVINFDFPKNAETYLHRIGRSGRFGHLGLAINLINWDDRFNLYKIE

QELGTEIQPIPPSIDKSLYVYENPESIPRPMPPPQPAKALPSNAQYNANSNGQPQFRRQQNQSNGYGQDYNNQGRGGYRG

RGRGQGPRGRGGYSQQTAAPVGQTAQ

>RPA76092.1 DEAD-domain-containing protein [Ascobolus immersus RN42]

MKRLTNASSSDEEWKGKLNIPAKDHRPQTEDVTQRKGLEFEDLYIKREILMGVFEAGFEKPSPIQEETIPVALTGRDILA

RAKNGTGKTAAFVIPALERVNPKSNKTQVLILVPTRELALQTSQVCKILGKHLGINVMVTTGGTTLKDDIIRLHDPVHIL

VGTPGRILDLAGKSVADFSECPIFIMDEADKLLSPEFTPIIEQLLAYFPADRQIMLFSATFPLVVKSFMDKHLKQPYEIN

LMDELTLRGITQYYAFVEEKQKVHCLNTLFSKLQINQSIIFCNSTNRVELLAKKITELGYSCFYSHARMAQASRNRVFHD

FRNGVCRNLVCSDLLTRGIDIQAVNVVINFDFPKNAETYLHRIGRSGRFGHLGLAINLISWEDRFNLYRIEQELGTEIQP

IPKEIDKKLYVYDSPETIPRPPKLQIAQTPTAPNHQQATQPRPQQQQNQNQGHSQGQNQPPRQQQQGGPQGGPQGGQRQS

NYQQPRPRGPPVNGSQQGSQGFPRQQQQGGRGGPRPNGQQPQQQQQQPARQH

>CBQ73743.1 probable ATP-dependent RNA helicase DHH1 [Sporisorium reilianum SRZ2]

MSASGPAPGQSEAAWKQQITSKISKDERPQTEDVLNTKGNEFEDYFLKRELLMGIFEAGFERPSPIQEEAIPIALTGRDI

LARAKNGTGKTAAYVIPSLEKLNTKKNKIQAVLLVPTRELALQTSQVAKTLGKHLGVEVMVTTGGTTLRDDILRLGQTVH

MLVGTPGRILDLAGKGVADLSQCTTFVMDEADKLLSPEFTPVMEQLLSFLPKERQVMLFSATFPLIVKDFKDRNMVKPYE

INLMDELTLRGVTQYYAFVEERQKVHCLNTLFSKLQINQSIIFCNSTNRVELLAKKITELGYSCFYSHAKMLQAHRNRVF

HDFRNGACRNLVCSDLLTRGIDIQAVNVVINFDFPKNAETYLHRIGRSGRFGHLGLAINLITYEDRFNLYRIEQELGTEI

QPIPSNIDKRLYVAPSLIQEAEGKNQNGNMQEGRPVIPPGQQAMHAAAIPQNQNFSNTVPHRHRGGGGGGGGQRGGGGRG

GPQGQGGVPAQ

>RPA95063.1 DEAD-domain-containing protein [Choiromyces venosus 120613-1]

MATALADQLEATSLGGGRDPNWKSKLKAPKKDLRPQTEDVTATKGLDFEDLYIKRELLMGIFEAGFEKPSPIQEETIPVA

LTGRDILARAKNGTGKTAAFVIPALERVNPKSTKTQALILVPTRELALQTSQVCKTLGKHLGINVMVTTGGTGLKDDIIR

LNEAVHILVGTPGRILDLAGKTVADFSECPTFIMDEADKLLSPEFTPIIEQLLAYFPSDRQIMLFSATFPLVVKSFMDKH

LNKPYEINLMDELTLRGVTQYYAFVEEKQKVHCLNTLFSKLQINQSIIFCNSTNRVELLAKKITELGYSCFYSHAKMLQN

HRNRVFHDFRNGVCRNLVCSDLLTRGIDIQAVNVVINFDFPKNAETYLHRIGRSGRFGHLGLAINLINWDDRFNLYKIEQ

ELGTEIIPIPPVIDKKLYVYDSPESIPRPPPMPQQPRQETREQNQRQNTQHQNQNSQQQYQSRQNQHHTGNPGNPVNPQQ

QQQQQQQQQQRSMNHRQQQQGPRNYPNSGPHRGPFQEPGTGRGQGQNRRPPRYDDRQGRSNGFGPGDSGPSVRAQ

>KJA23157.1 hypothetical protein HYPSUDRAFT_138278 [Hypholoma sublateritium FD-334 SS-4]

MLKTGGSINRPALTNDSWKAGLQRPKKDNRPQTEDVTATKGTEFEDMSLKRELLMGIFEAGFEKPSPIQEEAIPVALAKR

DILARAKNGTGKTAAFVIPSLQQVDLSKNKIQALLLVPTRELALQTSQVCKILGKHMGLQVMVTTGGTTLKDDILRLSET

VHVLVGTPGRILDLAGKGVADLSECPVFVMDEADKLLSPEFAPVMEQLLSFLPDDRQVMLFSATFPMIVKDFKEKHMKSP

YEINLMDELTLRGVTQYYAYVEERQKVHCLNTLFSKLQINQSIIFCNSTNRVELLAKKVTELGYSCFYSHAKMLQSHRNR

VFHDFRNGVCRNLVCSDLLTRGIDIQAVNVVINFDFPKNSETYLHRIGRSGRFGHLGLAINLVTYEDRFNLYKIEQELGT

EIQPIPQSIDRGLYVAPSGGEESQVQRAPQQQQLQQQPHPQPAPQQRQQAQGASSHRNGHQQQVPQQQQQQQLPQYQNGA

PAAQYRAPVYR

>XP_007731184.1 ATP-dependent RNA helicase DHH1 [Capronia epimyces CBS 606.96]

MPDALAAQLNKASLNDGAEDPNWRDSLKLPTKDTRQQTEDVTATKGLEFEDFYIKRELMMGIFEAGFEKPSPIQEETIPV

ALTGRDILARAKNGTGKTAAFVIPTLERINPKNPKTQALILVPTRELALQTSQVCKMLGKHLGINVMVTTGGTGLKDDII

RLGEPVHIIVGTPGRILDLASKGVADLSECPIFVMDEADKLLSPEFTIVIEQLLSFLPKDRQVMLFSATFPMIVKSFKDK

HMRNPYEINLMDELTLRGITQYYAFVEEKQKVHCLNTLFSKLQINQSIIFCNSTNRVELLAKKITELGYSCFYSHAKMLQ

QNRNKVFHDFRAGVCRNLVCSDLLTRGIDIQAVNVVINFDFPKNAETYLHRIGRSGRFGHLGLAINLINWEDRFNLYKIE

QELGTEIQPIPPSIDKSLYVYENPENIPRSMPPPSQTTRSLPSNAPANGQNQARRPQNQSNGYGQQPNNQGRGGYRGRGR

SQGQGQGQGPRGGRGGYPHQTAAPVGQTAQ

>KDQ31787.1 hypothetical protein PLEOSDRAFT_22618, partial [Pleurotus ostreatus PC15]

ANASDNWKATLRPPPRDSRPQTEDVTATKGTEFEDMFLRRELLMGIFEAGFERPSPIQEEAIPIALTHRDILARAKNGTG

KTAAFVIPSLQQVDVSKPKIQALLLVPTRELALQTSQVCKILGKHMGVQVMVTTGGTTLKDDILRLSESVHVLVGTPGRI

LDLAGKNVADLSECPVFVMDEADKLLSPEFTPVMEQLLSFLPKERQVMLFSATFPMIVKDFKDKYMNKPYEINLMDELTL

RGVTQYYAYVEERQKVHCLNTLFSKLQINQSIIFCNSTNRVELLAKKVTELGYSCFYSHAKMLQSHRNRVFHDFRNGVCR

NLVCSDLLTRGIDIQAVNVVINFDLPKSSETYLHRIGRSGRFGHLGLAINLVTYEDRFNLYKIEQELGTEIQPIPQVIDK

GLYVAPGLGAEEQQQAQQQQAQQQARALAQQRQQQVQQAAGVAASSRGRGRGRGQGGSNITLHAFPTRTRSATNTLG

>CDR88560.1 probable ATP-dependent RNA helicase DHH1 [Sporisorium scitamineum]

MSASGPAPGQSEAAWKQQITSKLSKDERPQTEDVLNTKGNEFEDYFLKRELLMGIFEAGFERPSPIQEEAIPIALTGRDI

LARAKNGTGKTAAYVIPSLEKLNTKKNKIQAVLLVPTRELALQTSQVAKTLGKHLGVEVMVTTGGTTLRDDILRLGQTVH

MLVGTPGRILDLAGKGVADLSQCTTFVMDEADKLLSPEFTPVMEQLLSFLPKERQVMLFSATFPLIVKGFKDRNMVKPYE

INLMDELTLRGVTQYYAFVEERQKVHCLNTLFSKLQINQSIIFCNSTNRVELLAKKITELGYSCFYSHAKMLQAHRNRVF

HDFRNGACRNLVCSDLLTRGIDIQAVNVVINFDFPKNAETYLHRIGRSGRFGHLGLAINLITYEDRFNLYRIEQELGTEI

QPIPSNIDKRLYVAPSLIQEAEGKNQNGNRQEGRPVIPPGQQAMHAAAIPQNQNFSNTVPHRHRGGGGGGQRGGGRGGPQ

GQGGVPAQ

>SAM03852.1 hypothetical protein [Absidia glauca]

MSGPYNNQQLSHENDNWKKTMTLPPKDTRPQTEDVTATKGNEFEDYFLKRELLMGIFEAGFERPSPIQEEAIPIALTGRD

ILARAKNGTGKTAAFVIPTLEKVNNKKSKIQALLLVPTRELALQTSQVCKTLGKHLGVQIMVTTGGTTLKDDILRLSETV

HVVVGTPGRILDLASKGVADFSEASTFVMDEADKLLSPEFTPIIDQLISFFPSERQIMLFSATFPMIVKHFKDKYLVKPY

EINLMDELTLRGVTQYYAFVEEKQKVHCLNTLFSKLQINQSIIFCNSTNRVELLAKKITELGYSCFYSHAKMLQSHRNRV

FHDFRNGVCRNLVCSGKRRDGSGIGRNRKMLTRITLYWRLDLLTRGIDIQAVNVVINFDFPKNAETYLHRIGRSGRFGHL

GLAINLITYEDRFNLYKIERELGTEIQPIPPTIDKQLYVAPNALDEAQVQQPNREAAIATRQQQQQRESQPQQQQQQQQQ

QQQQQPQQQQQYHQAQQPHYQQNQHQQQQPQQRQNYFQNGRGRGGYNGGRGGYNGRGRPRQSYQQQQQQQQPHVPMN

>XP_007719915.1 ATP-dependent RNA helicase DHH1 [Capronia coronata CBS 617.96]

MTDALAAQLNKASLNDGAEDPNWKDSLKLPTKDNRQQTEDVTATKGLDFEDFYIKRELMMGIFEAGFEKPSPIQEETIPV

ALTGRDILARAKNGTGKTAAFVIPTLERINPKNPKTQALILVPTRELALQTSQVCKMLGKHLGINVMVTTGGTGLKDDII

RLGEPVHIIVGTPGRILDLASKGVADLSECPIFVMDEADKLLSPEFTVVIEQLLSFLPKDRQVMLFSATFPMIVKTFKDK

HMRNPYEINLMDELTLRGITQYYAFVEEKQKVHCLNTLFSKLQINQSIIFCNSTNRVELLAKKITELGYSCFYSHAKMLQ

QNRNKVFHDFRAGVCRNLVCSDLLTRGIDIQAVNVVINFDFPKNAETYLHRIGRSGRFGHLGLAINLINWEDRFNLYKIE

QELGTEIQPIPPSIDKSLYVYENPESIPRPMPPAGQPKSLPSNAPANANTNGQNQGRRPQNQSNGYGQQYNSQGRGGYRG

RGRGQGARGRGGYPQQTAAPVGQTAQ

>XP_027482532.1 ATP-dependent RNA helicase DDX6/DHH1 [Malassezia restricta]

MTSVAPGMEVRRLPKDTRPQTEDVTVTKGNDFEDFFLKRELLMGIFEAGFEHPSPIQEEAIPIALSGRDILARAKNGTGK

TAAYVIPTLERVNTKKNKIQAVLLVPTRELALQTSQVAKTLGKHLGVEIMVSTGGTTLKDDILRLGQTVHILVGTPGRIL

DLASKGIADLSQCTTYVMDEADKLLSPEFTPVMEQLLGLMPKERQVMLFSATFPLIVKDFKDKHMVKPYEINLMDELTLR

GVTQYYAFVEERQKVHCLNTLFSKLQINQSIIFCNSTNRVELLAKKITELGYSCFYSHAKMLQAHRNRVFHDFRNGACRN

LVCSDLLTRGIDIQAVNVVINFDFPKNAETYLHRIGRSGRFGHMGLAINLITYEDRFNLYRIEQELGTEVQPIPATIDKR

LYVAPSLIEEAENRERDAKAQGKPTQGPGIMQQGLPSIPPTQAAMHAVPMNQNANFTNTVPHRRGGRRNRRGGHAGHGNA

NGAANGAQGGA

>CDW97717.1 hypothetical protein [Sporisorium scitamineum]

MSASGPAPGQSEAAWKQQITSKLSKDERPQTEDVLNTKGNEFEDYFLKRELLMGIFEAGFERPSPIQEEAIPIALTGRDI

LARAKNGTGKTAAYVIPSLEKLNTKKNKIQAVLLVPTRELALQTSQVAKTLGKHLGVEVMVTTGGTTLRDDILRLGQTVH

MLVGTPGRILDLAGKGVADLSQCTTFVMDEADKLLSPEFTPVMEQLLSFLPKERQVMLFSATFPLIVKDFKDRNMVKPYE

INLMDELTLRGVTQYYAFVEERQKVHCLNTLFSKLQINQSIIFCNSTNRVELLAKKITELGYSCFYSHAKMLQAHRNRVF

HDFRNGACRNLVCSDLLTRGIDIQAVNVVINFDFPKNAETYLHRIGRSGRFGHLGLAINLITYEDRFNLYRIEQELGTEI

QPIPSNIDKRLYVAPSLIQEAEGKNQNGNRQEGRPVIPPGQQAMHAAAIPQNQNFSNTVPHRHRGGGGGGQRGGGRGGPQ

GQGGVPAQ

>XP_013020865.1 ATP-dependent RNA helicase Ste13 [Schizosaccharomyces cryophilus OY26]

MTENLIQKLENATLNEQDWKAQLQATNVDKRPKTEDVTKTRGTEFEDYYLKRELLMGIFEAGFERPSPIQEESIPIALSG

RDILARAKNGTGKTAAFVIPSLEKVDTKKNKIQTLILVPTRELALQTSQVCKTLGKHMNIKVMVTTGGTTLRDDILRLND

PVHIVVGTPGRVLDLAGKGVANFSECMTFVMDEADKLLSPEFTPIIEQLLSYFPKNRQISLYSATFPLIVKAFMDKYLSK

PYEINLMDELTLRGITQYYAFVDESQKVHCLNTLFSKLQINQSIIFCNSTNRVELLAKKITELGYSCFYSHAKMLQSHRN

RVFHNFRNGVCRNLVCSDLLTRGIDIQAVNVVVNFDFPKNAETYLHRIGRSGRFGHRGLAISFISWADRFNLYRIENELG

TEIQPIPSSIDPSLYVFPNGNYQVPRPLAGTAEQALAAQQARGQEFNHNRNNYRGHSGRGNGGGYRGGYGPRRNPRYGGS

NPKSLS

>XP_025597481.1 putative ATP-dependent RNA helicase DHH1 [Tilletiopsis washingtonensis]

MASSTASDANWRAEIAARVEKDARPQTDDVLATKGNDFEDYFLKRSLLMGIFEAGFERPSPIQEEAIPIALTGRDILARA

KNGTGKTAAYIIPTLERLRPERTAIQAVLLVPTRELALQTSQVAKTLGKHMGIQIMVTTGGTTLRDDILRLSQTVHVLVG

TPGRVLDLASKGIADLSQCGTFVMDEADKLLSPEFTPVMEQLLGLLPRERQVMLFSATFPLIVKDFKDKHMVKPYEINLM

DELTLRGVTQYYAFVEERQKVHCLNTLFSRLQINQSIIFCNSTNRVELLAKKITELGYSCFYSHAKMLQAHRNRVFHDFR

NGACRNLVCSDLLTRGIDIQAVNVVINFDFPKNAETYLHRIGRSGRFGHLGLAINLITYEDRFNLYRIEQELGTEIQPIP

ATIDKRLYVAPALIDAAEEEEAKRAGAPRQKQHQNGAPVIPPTQRAMHQAIIPQSQDFTQSVAGGTRGRGGGQPQQQQSQ

Q

>EIE81845.1 ATP-dependent RNA helicase DHH1 [Rhizopus delemar RA 99-880]

MNNSSATPQQQPSHDTENWKQNLALPRKDTRPQTEDVTATKGNEFEDYFLKRELLMGIFEAGFERPSPIQEEAIPIALAN

RDILARAKNGTGKTAAFVIPTLEKINNKVSKIQALLLVPTRELALQTAQVCKTLGKHLNIQVMVTTGGTTLKDDIMRLSE

IVHVVVGTPGRILDLASKGVADFSQANTFVMDEADKLLSPEFTPVIDQLISYFPKNRQIMLFSATFPMIVKSFKDKHLTK

PYEINLMDELTLRGVTQYYAYVEEKHKVHCLNTLFSKLQINQSIIFCNSTNRVELLAKKITELGYSCFYSHAKMLQSHRN

RVFHDFRNGVCRNLVCSDLLTRGIDIQAVNVVINFDFPKNAETYLHRIGRSGRFGHLGLAINLITYEDRFNLYKIERELG

TEIQPIPPVIDKQLYVAPNALEETEVQQPNRQLALAMRQQQQQREVQPEQQQQQQQQQQQQQQQQQQRRNYGQSSRGGYY

HQRGGRHYTGPRGQ

>AYO41413.1 ATP-dependent RNA helicase dhh1 [Malassezia restricta CBS 7877]

MEVRRLPKDTRPQTEDVTVTKGNDFEDFFLKRELLMGIFEAGFEHPSPIQEEAIPIALSGRDILARAKNGTGKTAAYVIP

TLERVNTKKNKIQAVLLVPTRELALQTSQVAKTLGKHLGVEIMVSTGGTTLKDDILRLGQTVHILVGTPGRILDLASKGI

ADLSQCTTYVMDEADKLLSPEFTPVMEQLLGLMPKERQVMLFSATFPLIVKDFKDKHMVKPYEINLMDELTLRGVTQYYA

FVEERQKVHCLNTLFSKLQINQSIIFCNSTNRVELLAKKITELGYSCFYSHAKMLQAHRNRVFHDFRNGACRNLVCSDLL

TRGIDIQAVNVVINFDFPKNAETYLHRIGRSGRFGHMGLAINLITYEDRFNLYRIEQELGTEVQPIPATIDKRLYVAPSL

IEEAENRERDAKAQGKPTQGPGIMQQGLPSIPPTQAAMHAVPMNQNANFTNTVPHRRGGRRNRRGGHAGHGNANGAANGA

QGGA

>EPS40311.1 hypothetical protein H072_5868 [Dactylellina haptotyla CBS 200.50]

MASSLAEKLESTTLSDADWRAQLKTPVKDNRPQTEDVTATKGLDFEELYIKRELLMGIFEAGFEKPSPIQEETIPVALTG

RDILARAKNGTGKTAAFVIPALERVNAKSPKTQALILVPTRELALQTSQVCKTLGKHLGINVMVTTGGTGLKDDIIRLNE

AVHVLVGTPGRILDLAGKGVADFSECPTFIMDEADKLLSPEFTPIIEQLLAYFPSDRQIMLFSATFPLVVKSFMDKHLKQ

PYEINLMDELTLRGVTQYYAFVEEKQKVHCLNTLFSKLQINQSIIFCNSTNRVELLAKKITELGYSCFYSHAKMLQNHRN

RVFHDFRNGVCRNLVCSDLLTRGIDIQAVNVVINFDFPKNAETYLHRIGRSGRFGHLGLAINLINWDDRFNLYKIEQELG

TEIQPIPAQIDKKLYVYDSPESIPRPPATQPSVRPALSLPGSAQQPTTNRMTAGDSHNPSQTNQPQTIRPNKPRGNHGMN

QGFRGGRGSQRNYGHHQGYGRRNDTGQNSETMPRTSQNA

>PKI84057.1 Dhh1p [Malassezia vespertilionis]

MAITAPFQRPPKDLRPQTEDVTATKGNDFEDYFLKRELLMGIFEAGFERPSPIQEESIPIALSGRDILARAKNGTGKTAA

YVIPALERINTKKDKIQAVLLVPTRELALQTSQVAKTLGKHLSVEIMVSTGGTSLRDDIMRLDQPVQMLVGTPGRILDLA

GKGIADLSQCTAFVMDEADKLLSPEFTPVVEQLLSHLPKERQVMLFSATFPLIVKDFKDKHMVKPYEVNLMDELTLRGVT

QYYAFVEERQKVHCLNTLFSKLQVNQSIIFCNSTNRVELLAKKITELGYSCFYSHAKMLQAHRNRVFHDFRQGATRNLVC

SDLLTRGIDIQAVNVVINFDFPKNAETYLHRIGRSGRFGHMGLAINLITYEDRFNLYRIEQELGTEVQPIPATIDKRLYV

APSLIEEAEQREREAAASDAPVPKGPQAMQQGLPSIPPTQAAMHAVPITQNDNFTKSVQHRRGRRGGNREKGGQGGEGRQ

MA

>XP_007384334.1 DEAD-domain-containing protein [Punctularia strigosozonata HHB-11173 SS5]

MAQATTPASSSSSNWKSSIRAPPKDLRPQTEDVTATKGTEFEDMFLRRELLMGIFEAGFERPSPIQEEAIPVALTKRDVL

ARAKNGTGKTAAFVIPALQQIDISRPKIQALLLVPTRELALQTAQVCKALGKHMGAQVMVTTGGTTLKDDIMRLHETVHV

LVGTPGRILDLAGKNVADLSECPVFVMDEADKLLSPEFAPVMEQLLAFLPRDRQVMLFSATFPMIVKDFKDKHMKQPYEI

NLMDELTLRGVTQYYAFLEERQKVHCLNTLFSKLQINQSIIFCNSTNRVELLAKKITELGYSCFYSHAKMLQSHRNRVFH

DFRNGVCRNLVCSDLLTRGIDIQAVNVVINFDFPKNAETYLHRIGRSGRFGHLGLAINLVTYEDRFNLYKIEQELGTEIQ

PIPAQIDRSLYVAPSAPDEDPMSPSVSSTTNSAQQQQQQQQQAKARAAAAQALPQPQVVYQSGPPQGRLNGSPAPGLIQQ

QQVVGQGVARGYSAQAGFRGVPAAR

>XP_029737338.1 hypothetical protein EX895_005515 [Sporisorium graminicola]

MSASGPAPGQSEVAWKQQITAKLSKDERPQTEDVLNTKGNEFEDYFLKRELLMGIFEAGFERPSPIQEEAIPIALTGRDI

LARAKNGTGKTAAYVIPSLEKLNTKKNKIQAVLLVPTRELALQTSQVAKTLGKHLGVEVMVTTGGTTLRDDILRLGQTVH

MLVGTPGRILDLAGKGVADLSQCTTFVMDEADKLLSPEFTPVMEQLLSFLPKERQVMLFSATFPLIVKDFKDRNMVKPYE

INLMDELTLRGVTQYYAFVEERQKVHCLNTLFSKLQINQSIIFCNSTNRVELLAKKITELGYSCFYSHAKMLQAHRNRVF

HDFRNGACRNLVCSDLLTRGIDIQAVNVVINFDFPKNAETYLHRIGRSGRFGHLGLAINLITYEDRFNLYRIEQELGTEI

QPIPSNIDKRLYVAPSLIQEAEGKSQNGNRQEGRPVIPPGQQAMHAAAIPQNQNFSNTVPHRHRGGGGGGQRGGGGRGGP

QGHGGVPAQ

>SPO26808.1 probable ATP-dependent RNA helicase DHH1 [Ustilago trichophora]

MSASGPAPGQSEAAWKQQITSQIAKDERPQTEDVLNTKGNEFEDYFLKRELLMGIFEAGFERPSPIQEEAIPIALTGRDI

LARAKNGTGKTAAYVIPSLEKLNTKKNKIQAVLLVPTRELALQTSQVAKTLGKHLGVEVMVTTGGTTLRDDILRLGQTVH

MLVGTPGRILDLAGKGVADLSQCTTFVMDEADKLLSPEFTPVMEQLLSFLPKERQVMLFSATFPLIVKDFKDRNMVKPYE

INLMDELTLRGVTQYYAFVEERQKVHCLNTLFSKLQINQSIIFCNSTNRVELLAKKITELGYSCFYSHAKMLQAHRNRVF

HDFRNGACRNLVCSDLLTRGIDIQAVNVVINFDFPKNAETYLHRIGRSGRFGHLGLAINLITYEDRFNLYRIEQELGTEI

QPIPSNIDKRLYVAPSLIQEAEGKNQNGNRQEGRPVIPPGQQAMHAAAIPQNQNFSNTVPHRHRGGGGGGQRGGAGRGPQ

GQGQGGVPAQ

>SPO26532.1 probable ATP-dependent RNA helicase DHH1 [Ustilago trichophora]

MSASGPAPGQSEAAWKQQITSQIAKDERPQTEDVLNTKGNEFEDYFLKRELLMGIFEAGFERPSPIQEEAIPIALTGRDI

LARAKNGTGKTAAYVIPSLEKLNTKKNKIQAVLLVPTRELALQTSQVAKTLGKHLGVEVMVTTGGTTLRDDILRLGQTVH

MLVGTPGRILDLAGKGVADLSQCTTFVMDEADKLLSPEFTPVMEQLLSFLPKERQVMLFSATFPLIVKDFKDRNMVKPYE

INLMDELTLRGVTQYYAFVEERQKVHCLNTLFSKLQINQSIIFCNSTNRVELLAKKITELGYSCFYSHAKMLQAHRNRVF

HDFRNGACRNLVCSDLLTRGIDIQAVNVVINFDFPKNAETYLHRIGRSGRFGHLGLAINLITYEDRFNLYRIEQELGTEI

QPIPSNIDKRLYVAPSLIQEAEGKNQNGNRQEGRPVIPPGQQAMHAAAIPQNQNFSNTVPHRHRGGGGGGQRGGGGRGPQ

GQGQSQGQGGVPAQ

>XP_025350463.1 putative ATP-dependent RNA helicase DHH1 [Pseudomicrostroma glucosiphilum]

MSTSGPAPGQSEAQWKAEITAKITRDERPQTDDVLKTKGNDFEDYFLKRELLMGIFEAGFEKPSPIQEEAIPIALTGRDI

LARAKNGTGKTAAYVIPSLERLNPKKNKIQAVLLVPTRELALQTSQVAKTLGKHLGVEIMVTTGGTTLKDDILRLNQTVH

VLVGTPGRILDLAGKGLADLSECGTFVMDEADKLLSPEFTPVMEQLLGLLPKERQVMLFSATFPLIVKDFKDKHMVKPYE

INLMDELTLRGVTQYYAFVEERQKVHCLNTLFSRLQINQSIIFCNSTNRVELLAKKITELGYSCFYSHAKMLQAHRNRVF

HDFRNGACRNLVCSDLLTRGIDIQAVNVVINFDFPKNAETYLHRIGRSGRFGHLGLAINLITYEDRFNLYRIEQELGTEI

QPIPSQIDKKLYVAPSLIQSAEQQEQQQQQRGNQKRQNGQANQAGMPVIPPTQAAMHANAIPQSQNFTQSVAHRGQRRGP

GGGGGAGAPPSAQA

>XP_013018037.1 ATP-dependent RNA helicase Ste13 [Schizosaccharomyces octosporus yFS286]

MTENLIQKLENTTLNEQDWKAQLQANNIDKRPKTEDVTKTRGTEFEDYYLKRELLMGIFEAGFERPSPIQEESIPIALSG

RDILARAKNGTGKTAAFVIPSLEKVDTKKNKIQTLILVPTRELALQTSQVCKNLGKHMNAKVMVTTGGTTLRDDILRLND

PVHIVVGTPGRVLDLAGKGVADFSECMTFVMDEADKLLSPEFTPIIEQLLSYFPKNRQISLYSATFPLIVKAFMDKHLNK

PYEINLMDELTLRGITQYYAFVDESQKVHCLNTLFSKLQINQSIIFCNSTNRVELLAKKITELGYSCFYSHAKMLQSHRN

RVFHNFRNGVCRNLVCSDLLTRGIDIQAVNVVVNFDFPKNAETYLHRIGRSGRFGHRGLAISFISWADRFNLYRIENELG

TEIQPIPSSIDPSLYVFPNGNYQVPRPLTGTAEQTLAAQQARGQEYNNNRNNNRNHSGRGNGGGYRGGYGARRNPRYGGP

NPKSLS

>KIM47033.1 hypothetical protein M413DRAFT_16541 [Hebeloma cylindrosporum h7]

MSQTATSSSSTPSNNDSSWKSGLRPPPKDIRPQTEDVTATKGLEFEDMFLKRELLMGIFEAGFERPSPIQEEAIPIALAK

RDILARAKNGTGKTAAFVIPSLQQIDVNKPKIQALLLVPTRELALQTSQVCKILGKHMGLQVMVTTGGTTLKDDILRLNE

TVHVLVGTPGRILDLAGKGVADLSECPVFVMDEADKLLSPEFAPVMEQLLAFLPSDRQVMLFSATFPIIVKDFKDKHMKS

PYEINLMDELTLRGVTQYFAYVEERQKVHCLNTLFSKLQINQSIIFCNSTNRVELLAKKVTELGYSCFYSHAKMLQSHRN

RVFHDFRNGVCRNLVCSDLLTRGIDIQAVNVVINFDFPKNSETYLHRIGRSGRFGHLGLAINLVTYEDRFNLYKIEQELG

TEIQPIPQTIEKGLYVAPSGGEESQVQRPPQQAVAGAQPQPQQQAQQQRQQQGPSAPAQQQRNGQQPPQQQQSAQILQQQ

AAAAAARQQQNGAVPTQQPRGQPAYRGGVPVSR

>CDH55774.1 atp-dependent rna helicase dhh1 [Lichtheimia corymbifera JMRC:FSU:9682]

MSHEHDTWKQSLQLPAKDIRPQTEDVTATKGNEFEDYFLKRELLMGIFEAGFERPSPIQEEAIPIALTGRDILARAKNGT

GKTAAFIIPTLEKINNKKPKIQALIVVPTRELALQTSQVCKTLGKHLGIQVMVSTGGTTLKDDIMRLGETVHIVVGTPGR

ILDLGSRGIADFSEANTFVMDEADKLLSPEFAPVIDRLLKLFPRDRQIMLFSATFPMMVKTFKDKHLNKPYEINLMEELT

LRGITQFYAYVEERQKVHCLNTLFSKLQINQSIIFCNSTQRVELLAKKITDLGYSCFYSHAKMLQSHRNRVFHDFRNGVC

RNLVCSDLLTRGIDIQAVNVVINFDFPKNAETYLHRIGRSGRYGHFGLAINLITYEDRFNLYKIERELGTQIAPIPATID

KRLYVAPNALDSAQIQMPNREVAMATRQQQQEQLQRGGQQQQHHHQQQQQNRRAY

>OAV93892.1 ATP-dependent RNA helicase DHH1 [Puccinia triticina 1-1 BBBD Race 1]

MAASSSSASDLSLDPDWKSKLSLPQKDLRPQTEDVTKTKGIEFEDLYLRRDLLMGIFEAGFEKPSPIQEEAIPIALAGRD

ILARAKNGTGKTGAFVIPTLEKTNVRINRIQALILVPTRELALQTSQVCKTLGKHTGIQIMVTTGGTTLKDDILRLQEAV

HVVVGTPGRILDLASKGVADLSQCKTFVMDEADKLLSPEFTVVIEQLLSFLHKERQVMLFSATFPMIVKDFKDKHMIKPY

EINLMEELTLQGVTQYYAFLEERQKVHCLNTLFSKLQINQAIIFCNSTNRVELLARKITDIGYSCFYSHAKMLQQHRNRV

FHDFRNGVCRNLVCSDLLTRGIDIQAVNVVINFDFPKNAETYLHRIGRSGRFGHRGIAINLITYENRFDLYRIEQELGTE

IQPIPAVIDKALYVAPGIEDTPSSTPTHGSSSAPSSNANQAGHQHQQSNQAHRNGRQTNQQPSDQSHVPQPHDKPTLPPQ

QLTFQKAAPIDSLSHATPTEYANGAPPPNSSRVNHKSTRGGSGGPHRGRGGGAHRAPSGIAPAA

>KDR80281.1 hypothetical protein GALMADRAFT_136789 [Galerina marginata CBS 339.88]

MSQTATSSSSTPASNDSSWKTGLRPPPKDIRPQTEDVTATKGLEFEDMFLNRELLMGIFEAGFEKPSPIQEEAIPIALAK

RDILARAKNGTGKTAAFVIPSLQQIDLTRPKIQALLLVPTRELALQTSQVCKILGKHMGLQVMVTTGGTTLKDDILRLSE

MVHVLVGTPGRILDLAGKGVADLSECPVFVMDEADKLLSPEFAPVMEQLLSFLPTDRQVMLFSATFPMIVKDFKEKHMRS

PYEINLMDELTLRGVTQYYAYVEERQKVHCLNTLFSKLQINQSIIFCNSTNRVELLAKKVTELGYSCFYSHAKMLQSHRN

RVFHDFRGGVCRNLVCSDLLTRGIDIQAVNVVINFDFPKNSETYLHRIGRSGRFGHLGLAINLVTYEDRFNLYKIEQELG

TEIQPIPQTIDRGLYVAPSGGEETQVQRPAQQPMQQGQQQQQQQQRPQASAAAPRNGQHPQQPQITQQQQPVRQNGAPPQ

QPRGQPSYRGGVPAAR

>TIA89786.1 hypothetical protein E3P99_01871 [Wallemia hederae]

MASTSQTPESEWRNQLKVPPKDLRPQTEQAAQLTSAQQDVTNTKGAEWEDFGLRRELLMGIFEAGFEKPSPIQEEALPSA

IQGRDVLARAKNGTGKTASFVIPSLERINVQKPKIQALLLVPTRELALQTSQVCKTLGKHLGIQVMVTTGGTTLRDDIMR

LSEPVHILVGTPGRILDLAGKGVANLNECPTFVMDEADKLLSPEFTPVMEQLLSHLPTSRQVMLFSATFPLIVKDFKEKH

MNNPHEINLMDELTLRGITQYYAFVEERQKVHCLNTLFSKLQINQSIIFCNSTNRVELLAKKVTELGYSCFFSHAKMLQS

HRNRVFHDFRSGVCRNLVCSDLLTRGIDIQAVNVVINFDFPKNSETYLHRIGRSGRYGHLGLAINLITYEDRFNLYKIEQ

ELGTEIQPIPSQIDRSLYVAPSASDDDGQIQQSQQQQQQQQQKGLPTLPPTQQVMNAYSQPPPQPTFNQSNQNHHQYQQR

SNRGGYNSGYYGGSGYRGRGGGPPRGGVPRA

>XP_014657000.1 eukaryotic translation initiation factor 4A-like protein [Moesziomyces antarcticus]

MSASGPAPGQSEAAWKQQITNKVAKDERPQTEDVLNTKGNEFEDYFLKRELLMGIFEAGFERPSPIQEEAIPIALTGRDI

LARAKNGTGKTAAYVIPSLEKLNTKKNKIQAVLLVPTRELALQTSQVAKTLGKHLGVEVMVTTGGTTLRDDILRLGQTVH

MLVGTPGRILDLAGKGVADLSQCTTFVMDEADKLLSPEFTPVMEQLLSFLPKERQVMLFSATFPLIVKDFKDRNMVKPYE

INLMDELTLRGVTQYYAFVEERQKVHCLNTLFSKLQINQSIIFCNSTNRVELLAKKITELGYSCFYSHAKMLQSHRNRVF

HDFRNGACRNLVCSDLLTRGIDIQAVNVVINFDFPKNAETYLHRIGRSGRFGHLGLAINLITYEDRFNLYRIEQELGTEI

QPIPSNIDKRLYVAPSLIQEAEGKNQNGNRQEGRPVIPPGQQAMHAAAIPQNQNFSNTVPHRHRGGRGGGGGGGGGRQHH

GQGSVPAQ

>PAV15228.1 eukaryotic translation initiation factor 4A [Pyrrhoderma noxium]

MSTVTTPSASSSTGTQQDWRSGLRAPPKDDRPQTEDVTATKGMEFEDMFLRRELLMGIFEAGFERPSPIQEEAIPVALTK

RDILARAKNGTGKTAAFVIPSLQQIDVSKPKIQALLLVPTRELALQTAQVCKILGKHLGANVMVTTGGTTLKDDIMRLSE

SVHVLVGTPGRILDLAGKGVADLSECPVFVMDEADKLLSPEFTPVMEQLLSYLPSSRQVMLFSATFPMIVKDFKDKHMDS

PYEINLMDELTLRGVTQYYAFVEERQKVHCLNTLFSKLQINQSIIFCNSTNRVELLAKKVTELGYSCFYSHAKMLQSHRN

RVFHDFRQGVCRNLVCSDLLTRGIDIQAVNVVINFDFPKNSETYLHRIGRSGRFGHLGLAINLITYDDRFNLYKIEQELG

TEIQPIPQQIDKSLYVAPGAMSEPVGQPATLQPEQQQQQQQQQQHQQGAQIQQKIQQHQQQIARGPVPVQLPQMQPPQQQ

QPQQQQQILYQSNGQQPQQVRAAIPNGPAAQLAAAAARGQQYQPYMAGPGMGGGNRGGAPQSVRR

>KEP55787.1 ATP-dependent RNA helicase dhh1 [Rhizoctonia solani 123E]

MASATGTPSASNGDWKTGLRAPPKDDRPQTEDVTATKGIEFEDMPLRRELLMGIFEAGFEHPSPIQEEAIPVALTRRDIL

ARAKNGTGKTAAFTIPSLQHVDPTKPKIQAMLLTPTRELALQTAQVCKNLGKHMGINVMVTTGGTTLKDDIIRLSEAVHV

LVGTPGRILDLAGKQVADLSSCRVFVMDEADKLLSPEFTPVMEQLLSFVPADRQVMLFSATFPMIVKQFKDKHMKNPHEI

NLMDELTLRGVTQYYAFVEERQKVHCLNTLFAKLQINQSIIFCNSTNRVELLAKKVTELGYSCFYSHAKMVQSARNRVFH

DFRNGVCRNLVCSDLLTRGIDIQAVNVVINFDFPKHSETYLHRIGRSGRFGHLGLAINLITYEDRFNLYRIEQELGTEIQ

PIPAEINKSLYVAPSAIDEPAGQVPNSRSSAPESNSRSGTNGQAADAQQPRNGGTHAGQGRGQPRGGQQGPRGGGANGRG

AAPQR

>GAC75080.1 ATP-dependent RNA helicase [Moesziomyces antarcticus T-34]

MSASGPAPGQSEAAWKQQITNKVAKDERPQTEDVLNTKGNEFEDYFLKRELLMGIFEAGFERPSPIQEEAIPIALTGRDI

LARAKNGTGKTAAYVIPSLEKLNTKKNKIQAVLLVPTRELALQTSQVAKTLGKHLGVEVMVTTGGTTLRDDILRLGQTVH

MLVGTPGRILDLAGKGVADLSQCTTFVMDEADKLLSPEFTPVMEQLLSFLPKERQVMLFSATFPLIVKDFKDRNMVKPYE

INLMDELTLRGVTQYYAFVEERQKVHCLNTLFSKLQINQSIIFCNSTNRVELLAKKITELGYSCFYSHAKMLQSHRNRVF

HDFRNGACRNLVCSDLLTRGIDIQAVNVVINFDFPKNAETYLHRIGRSGRFGHLGLAINLITYEDRFNLYRIEQELGTEI

QPIPSNIDKRLYVAPSLIQEAEGKSQNGNRQEGRPVIPPGQQAMHAAAIPQNQNFSNAVPHRHRGGRGGGGGGGGGRQHH

GQGSVPAQ

>ELU41871.1 ATP dependent RNA helicase (Dhh1), putative [Rhizoctonia solani AG-1 IA]

MASATNNSSASNEDWKSGLRAPPKDDRPQTEDVTATKGLEFEDMGLRRELLMGIFEAGFERPSPIQEEAIPVALTRRDIL

ARAKNGTGKTAAFTIPSLQQVDPTKPKIQAMLLTPTRELALQTAQVCKNLGKHMGINVMVTTGGTTLKDDIIRLSEAVHV

LVGTPGRILDLAGKQVADLSQCRVFVMDEADKLLSPEFTPVMEQLLSFVPSDRQVMLFSATFPMIVKQFKVNDKHMKSPH

EINLMDELTLRGVTQYYAFVEERQKVHCLNTLFAKLQINQSIIFCNSTNRVELLAKKVTELGYSCFYSHAKMVQSARNRV

FHDFRNGVCRNLVCSDLLTRGIDIQAVNVVINFDFPKHSETYLHRIGRSGRFGHLGLAINLITYDDRFNLYRIEQELGTE

IQPIPAEINKSLYVAPSAIDEPAGQVPSNRAP

>ORE13345.1 ATP-dependent RNA helicase DHH1 [Rhizopus microsporus]

MSSSSQLNHETENWKTALTLPPKDSRPQTEDVTATKGNEFEDYFLKRELLMGIFEAGFERPSPIQEEAIPLALAGRDILA

RAKNGTGKTAAFVIPTLEKITNIPKIQALILVPTRELALQTSQVCKTLGKHLNIQVMVTTGGTTLKDDIMRLSETVHIVV

GTPGRILDLASKGVADFSAAHTFVMDEADKLLSPEFTPIIEQLLDYFPKNKQIMLFSATFPMIVKNFKDKYLVKPYEINL

MDELTLRGVTQYYAYVEEKQKVHCLNTLFSKLQINQSIIFCNSTNRVELLAKKITELGYSCFYSHAKMLQSHRNRVFHDF

RNGVCRNLVCSDLLTRGIDIQAVNVVINFDFPKNAETYLHRIGRSGRFGHLGLAINLITYEDRFNLYKIERELGTEIQPI

PPVIDKRLYVAPNALEDAQIQQPNKQQANATRQHQQSTEQQDQTQQQVTNGQQPLQGQQQQQQQRQHYSHQQRHPHYHNR

SNNWRGRGRGGGHHHSNRSTQKQQPYQQ

>ETS59938.1 hypothetical protein PaG_05919 [Moesziomyces aphidis DSM 70725]

MSASGPAPGQSEAAWKQQITNKVAKDERPQTEDVLNTKGNEFEDYFLKRELLMGIFEAGFERPSPIQEEAIPIALTGRDI

LARAKNGTGKTAAYVIPSLEKLNTKKNKIQAVLLVPTRELALQTSQVAKTLGKHLGVEVMVTTGGTTLRDDILRLGQTVH

MLVGTPGRILDLAGKGVADLSQCTTFVMDEADKLLSPEFTPVMEQLLSFLPKERQVMLFSATFPLIVKDFKDRNMVKPYE

INLMDELTLRGVTQYYAFVEERQKVHCLNTLFSKLQINQSIIFCNSTNRVELLAKKITELGYSCFYSHAKMLQSHRNRVF

HDFRNGACRNLVCSDLLTRGIDIQAVNVVINFDFPKNAETYLHRIGRSGRFGHLGLAINLITYEDRFNLYRIEQELGTEI

QPIPSNIDKRLYVAPSLIQEAEGKSQNGNRQEGRPVIPPGQQAMHAAAIPQNQNFSNTVPHRHRGGRGGGGGGGGGRQHH

GQGSVPAQ

>KZT01228.1 DEAD-domain-containing protein [Laetiporus sulphureus 93-53]

MSQQARPSSSTPSSDAWKSGLRPPPKDDRPQTEDVTATKGIEWDDMHLRRDLLMGIFEAGFERPSPIQEEAIPIALTKRD

ILARAKNGTGKTAAFVIPALQQVDINKNKIQALLLVPTRELALQTSQVCKILGKHMGVQVMVTTGGTTLKDDIIRLSETV

HVLVGTPGRILDLSGKNVADLSECPVFVMDEADKLLSPEFAPVMEQLLSFMPSDRQVMLFSATFPMIVKDFKDKHMNSPY

EINLMDELTLRGVTQYYAYVEERQKVHCLNTLFSKLQINQSIIFCNSTNRVELLAKKVTELGYSCFYSHAKMLQSHRNRV

FHDFRNGVCRNLVCSDLLTRGIDIQAVNVVINFDFPKNSETYLHRIGRSGRFGHLGLAINLVTYEDRFNLYKIEQELGTE

IQPIPQYIDKGLYVAPGAISAEESSKQHKGKNSQSQAHTPTQSMPAPQPTIPAQLVYSSTQPAKQNGTTTPQPQAARAAY

QAQPAHRGAVPVAR

>XP_013321755.1 ATP-dependent RNA helicase dhh1 [Exophiala xenobiotica]

MTDALASQLNKASLNDGADESNWRDSLKLPNKDTRQQTEDVTATKGLEFEDFYIKRELMMGIFEAGFEKPSPIQEETIPV

ALTGRDILARAKNGTGKTAAFVIPTLERINPKNPKTQALLLVPTRELALQTSQVCKTLGKHLGINVMVTTGGTGLKDDII

RLGETVHIIVGTPGRILDLASKGVADLSECPIFVMDEADKLLSPEFTVVIEQLLSFLPKDRQVMLFSATFPMMVKTFKDK

HMHNPYEINLMDELTLRGITQYYAFVEEKQKVHCLNTLFSKLQINQSIIFCNSTNRVELLAKKITELGYSCFYSHAKMLQ

QNRNKVFHDFRAGVCRNLVCSDLLTRGIDIQAVNVVINFDFPKNAETYLHRIGRSGRFGHLGLAINLINWDDRFNLYKIE

QELGTEIQPIPPSIDKSLYVYDSPENIPRPMPPPAQASRANNAPVMAANAFAGANMNGQAQGRRPYNQSNQSNGYGQQYN

IQGRGGYRGRGRGQGQGGRGRGGHPHQAAAPIGQTAQ

>KAF3940704.1 hypothetical protein ABW19_dt0202359 [Dactylella cylindrospora]

MASSLADQLESTTLNDADWRSQLKAPVKDNRPQTEDVTATKGLDFEELYIKRELLMGIFEAGFEKPSPIQEETIPVALTG

RDILARAKNGTGKTAAFVIPALERVNAKISKTQALILVPTRELALQTSQVCKTLGKHLGINVMVTTGGTGLKDDIIRLNE

AVHVLVGTPGRILDLAGKGVADFSECPTFIMDEADKLLSPEFTPIIEQLLAYFPSDRQIMLFSATFPLVVKSFMDKHLKQ

PYEINLMDELTLRGVTQYYAFVEEKQKVHCLNTLFSKLQINQSIIFCNSTNRVELLAKKITELGYSCFYSHAKMLQNHRN

RVFHDFRNGVCRNLVCSDLLTRGIDIQAVNVVINFDFPKNAETYLHRIGRSGRFGHLGLAINLINWDDRFNLYKIEQELG

TEIQPIPAQIDKKLYVYDSPETIPRPPPAQPSARPPAVINNPSQSSIARTAPQDNHGNTQNNPQQNPRQGKTRGSHNIGQ

GFRGGRGGQRNYGHHQGYARRNDGGQSSENTARNS

>KAF5310704.1 hypothetical protein D9619_008133 [Psilocybe cf. subviscida]

MSQPQSSSSTPANADWKANLQLPKKDFRPQTEDVTATKGTEFEDMNLRRELLMGIFEAGFEKPSPIQEEAIPIALTKRDV

LARAKNGTGKTAAFVIPSLQQVDVNKNKIQALLLVPTRELALQTSQVCKILGKHMGLQVMVTTGGTTLKDDILRLSEVVH

VLVGTPGRILDLAGKGVADLSECPVFVMDEADKLLSPEFAPVMEQLLSFLPTERQVMLFSATFPMIVKDFKDKHMHNPYE

INLMDELTLRGVTQYYAYVEERQKVHCLNTLFSKLQINQSIIFCNSTNRVELLAKKVTELGYSCFYSHAKMLQSHRNRVF

HDFRNGVCRNLVCSDLLTRGIDIQAVNVVINFDFPKNSETYLHRIGRSGRFGHLGLAINLVTYEDRFNLYKIEQELGTEI

MPIPQTIDRGLYVAPGGGDDEGEVQQQRPPQQAPQPAPQAQQQQRQQLPPQMQQQQVQRNGQPIPQQQYVQQQQQYAQQP

QYQNGAPQAQRGPAPVYRQGPPVGR

>KIV81870.1 ATP-dependent RNA helicase dhh1 [Exophiala sideris]

MTDSLASQLNQTSLNDGAGDSNWKDSLKIPTKDTRHQTEDVTATKGLEFEDFYIKRELMMGIFEAGFEKPSPIQEETIPV

ALTGRDVLARAKNGTGKTAAFVIPTLERINPKNPKTQALLLVPTRELALQTSQVCKTLGKHLGINVMVTTGGTGLKDDII

RLGEAVHIIVGTPGRILDLASKGVADLSECPIFVMDEADKLLSPEFTVVIEQLLSFLPKDRQVMLFSATFPMMVKTFKDK

HMRNPYEINLMDELTLRGITQYYAFVEEKQKVHCLNTLFSKLQINQSIIFCNSTNRVELLAKKITELGYSCFYSHAKMLQ

QNRNKVFHDFRAGVSRNLVCSDLLTRGIDIQAVNVVINFDFPKNAETYLHRIGRSGRFGHLGLAINLINWDDRFNLYKIE

QELGTEIQPIPPSIDKSLYVYDSPENIPRPMPPPAQAQQGNSNPGTNAYSNGQAQGGRRSYNQSNGYGQQYNQGRGGYRG

RGRGQGGRGRGGQFQQSTPPVGQAAQ

>KAF2220032.1 P-loop containing nucleoside triphosphate hydrolase protein [Elsinoe ampelina]

MASELANQLSGTSLNDNSAQGDDWKAGLKKPNKDGRQQTEDVTATKGLEFEDFYIKRELMMGIFEAGFEKPSPIQEEAIP

VALTGRDVLARAKNGTGKTGSFVIPTLERVNPKNSKIQALLLVPTRELALQTSQVCKTLGKHLGINVMVTTGGTSLRDDI

IRLNETVHILVGTPGRILDLAAKGIADLSDCQTFVMDEADKLLSPEFTSQIDQLIAFHPKDRQIMLFSATFPVIVKDFKD

KHMNDPHEINLMDELTLRGITQYYAFVEEKQKVHCLNTLFSRLQINQSIIFCNSTQRVELLAKRVTELGYSCYYSHARMM

QHARNRVFHDFRAGHCRNLVCSDLLTRGIDIQAVNVVINFDFPKNAETYLHRIGRSGRFGHLGLAINLISWEDRFNLYRI

EQELGTEIQPIPSNIDKSLYVYETPENIPRPISGAPQQRQPQGPSDNVRGRGGFQQRGMSRGRGGYQGQGGQGGQGQGQG

QGRGAPRDPTQQRPQNGFAPRGQAQPRAPPAAAQS

>XP_025375601.1 putative ATP-dependent RNA helicase DHH1 [Acaromyces ingoldii]

MSASGPAPGQTEAQWRQEIQQRVVRDERPQTDDVLKTKGNDFEDYFLKRELLMGIFEAGFERPSPIQEEAIPIALTGRDI

LARAKNGTGKTAAYVIPSLERLSTKKTHIQAVLLVPTRELALQTSQVCKTLGKHTGVEVMVTTGGTTLRDDILRLSQTVH

VLVGTPGRILDLAGKNIADLSQCTTFVMDEADKLLSPEFTPVIEQLLSFLPKERQVMLFSATFPLIVKDFKDRHMVKPYE

INLMDELTLRGVTQYYAFVEERQKVHCLNTLFSRLQINQSIIFCNSTNRVELLAKKITELGYSCFYSHAKMLQAHRNRVF

HDFRNGACRNLVCSDLLTRGIDIQAVNVVINFDFPKNAETYLHRIGRSGRFGHLGLAINLITYEDRFNLYRIEQELGTEI

QPIPSTIDKRLYVAPSLITEDEVNKQNNSGKASANGGSNNGNGNGAQQQQQRQHQSGAPVIPPTQAKFTQTTIPQSQDFT

QSIAGRNRGPRQPQQARQA

>ORZ02853.1 P-loop containing nucleoside triphosphate hydrolase protein [Syncephalastrum racemosum]

MSASTIISQSFSHDDDEWKANLKLPKKDARPQTEDVTQTKGNEFEDYFLKRELLMGIFEAGFERPSPIQEEAIPIALTGR

DILARAKNGTGKTAAFVIPTLEKIQTKKNHIQALILVPTRELALQTSQVCKNLGKHLDINVMVTTGGTTLKDDIIRLSEP

VHVVVGTPGRILDLASKGVADFSEASTFVMDEADKLLSPEFTPVIERLIECFPEKRQIMLFSATFPIVVRDFRGKHLVRP

YEINLMDELTLRGVTQYYAYVEEKQKVHCLNTLFSKLQINQSIIFCNSTNRVELLAKKITELGYSCFYSHARMLQDHRNR

VFHDFRQGVCRNLVCSDLLTRGIDIQAVNVVINFDFPKNAETYLHRIGRSGRYGHFGLAINLITYEDRFNLYKIERELGT

EITPIPPVIDKRLYVAPNALDDTTDLRKANMLPPNGSASSAHSRPPVQQQQYQYRQSQQQQNGRPHRSNNGRSRGNGTNH

RGRRNNNNSNGNFNPRADF

>PPQ99334.1 hypothetical protein CVT24_009148 [Panaeolus cyanescens]

MSQPASSSSTPANGNDSSWKTGLRPPPKDIRPQTEDVTATKGLEFEDMKLHRELLMGIFEAGFERPSPIQEEAIPIALTR

RDVLARAKNGTGKTAAFVIPSLQQVDVTKPKIQALLLVPTRELALQTAQVCKILGKHMGLQVMVTTGGTTLKDDILRLSE

IVHVLVGTPGRILDLAGKGVADLSECPVFVMDEADKLLSPEFAPVMEQLLSFLPAERQVMLFSATFPMIVKDFKDKHMRS

PHEINLMDELTLRGVTQYYAYVEERQKVHCLNTLFSKLQINQSIIFCNSTNRVELLAKKVTELGYSCFYSHAKMLQSHRN

RVFHDFRNGVCRNLVCSDLLTRGIDIQAVNVVINFDFPKNSETYLHRIGRSGRFGHLGLAINLVTYEDRFNLYKIEQELG

TEIQPIPQTIDRGLYVAPSGGEDSQVQRAPQQPQRQQPQQQQGQAAQQQKPQPTGTPVQRAGPPHAAPNQQQYIQQQQMA

RPGGQQPAYRGGVPVAR

>XP_025368461.1 putative ATP-dependent RNA helicase DHH1 [Ceraceosorus guamensis]

MATSSASSAAQDAQWRAEVAARVEKDGRPQTEDVTATKGNDFEDYFLKRPLLMGIFEAGFERPSPIQEEAIPIALTGRDI

LARAKNGTGKTAAYVIPSLERLKPEKSAIQAVLLVPTRELALQTSQVVKTLGKHMGIQVMVTTGGTTLRDDILRLSQTVH

ILVGTPGRILDLASKGIADLTQCSTFVMDEADKLLSPEFTPVMEQLLGLLPRERQVMLFSATFPLIVKDFKDKHMVKPYE

INLMDELTLRGVTQYYAFVEERQKVHCLNTLFSRLQINQSIIFCNSTNRVELLAKKITELGYSCFYSHAKMLQAHRNRVF

HDFRNGACRNLVCSDLLTRGIDIQAVNVVINFDFPKNAETYLHRIGRSGRFGHLGLAINLITYEDRFNLYRIEQELGTEI

APIPPTIDKRLYVAPSLITEDDGKNANGAKAHQNGAPVIPPTQRAMHQAIIPQSQDFTQSSGGRRGGGGGGHASASAAAG

AAPSQPAA

>CDS12947.1 Putative ATP-dependent RNA helicase DHH1 [Lichtheimia ramosa]

MSHEHDTWKQSLQLPAKDIRPQTEDVTATKGNEFEDYFLKRELLMGIFEAGFERPSPIQEEAIPIALTGRDILARAKNGT

GKTAAFIIPTLEKINNKKPKIQALIVVPTRELALQTSQVCKTLGKHLGIQVMVSTGGTTLKDDIMRLGETVHIVVGTPGR

ILDLGSRGLADFSEAHTFVMDEADKLLSPEFAPVIDRLLKLFPRDRQIMLFSATFPMMVKTFKDKHLNKPYEINLMEELT

LRGITQFYAYVEERQKVHCLNTLFSKLQINQSIIFCNSTQRVELLAKKITDLGYSCFYSHAKMLQSHRNRVFHDFRNGVC

RNLVCSDLLTRGIDIQAVNVVINFDFPKNAETYLHRIGRSGRYGHFGLAINLITYEDRFNLYKIERELGTQIAPIPATID

KRLYVAPNALDSAQIQMPNREVAMATRQQQQEQLQRGGQQQHQQQQNRRAY

>KIK36386.1 hypothetical protein CY34DRAFT_811305 [Suillus luteus UH-Slu-Lm8-n1]

MSQSTRPSTSTSTASSTTLNDASWRAGLRPPPKDLRPQTEDVTNTKGTEFEDMFLRRELLMGIFEAGFARPSPIQEEAIP

VALTKRDILARAKNGTGKTAAFVIPSLQQIDVGKNKIQALLLVPTRELALQTSQVCKIPGKHTGIQVMVTTGGTTLKDDY

MRLSETVHVLVGTPGRILDLAGKNVADLSECPVFVMDEADKLLSPEFTPVMEQLLSFLPTDRQVMLFSATFPMMVKDFKD

KRMRTPYEINLMEELTLRGVTQYYAYVEERQKVHCLNTLFSKLQINQSIIFCNSTNRVELLAKKVTDLGYSCFYSHAKML

QSHRNRVFHDFRNGVCRNLVCSDLLTRGIDIQAVNVVINFDFPKNSETYLHRIGRSGRFGHLGLAINLVTYEDRFNLYRI

EQELGTEIQPIPQAIDKGLYVAPSAAEEPAPQKPQSQPQPQPQPSQQQRQQAPSQQTSNNGRQNGAAPASSQVQQAQRSG

LVNQAVFRNGTPTR

>OAL71665.1 ATP-dependent RNA helicase DHH1 [Trichophyton violaceum]

MTDALAAQLNNTKLGDSSTDNKWKEQLKIPAKDTRVQTEDVTATKGLEFEDFYIKRDLMMGIFEAGFEKPSPIQEETIPV

ALTGRDILARAKNGTGKTAAFVIPTLERTNPKIAKTQALILVPTRELALQTSQVCKTLGKHLGINVMVTTGGTGLQDDII

RLNDTVHIIVGTPGRILDLASKGVADLSECTTFVMDEADKLLSPEFTPVIEQLLTFHPKDRQVMLFSATFPIIVKTFKDK

HMRNPYEINLMDELTLRGITQYYAFVEERQKVHCLNTLFSKLQINQSIIFCNSTNRVELLAKKITELGYSCFYSHARMLQ

HNRNRVFHDFRNGVCRNLVCSDLLTRGIDIQAVNVVINFDFPKNAETYLHRIGRSGRFGHLGLAINLINWDDRYNLYKIE

QELGTEIQPIPPSIDKKLYVYETPNTIPRPISNKPSEEQKPASAESQPRKQTTNSSNGHGQYSSNRGHYGRGSYRGSRGS

GQRSNNPDSSRAAGNPQANAASSGTPGS

>KIP07293.1 hypothetical protein PHLGIDRAFT_105879 [Phlebiopsis gigantea 11061_1 CR5-6]

MSQQTHPSSSTSGDVAWKSGLQPPPKDMRPQTEDVTATKGMEFEDMHLRRELLMGIFEAGFERPSPIQEEAIPIALTKRD

VLARAKNGTGKTAAFVIPSLQQIDINKNKIQALLLVPTRELALQTAQVCKILGKHMNLQVMVTTGGTTLKDDIIRLSEAV

HVLVGTPGRILDLAGKNIADLSECPVFVMDEADKLLSPEFSPVMEQLLSYMPKDRQVMLFSATFPMIVKDFKDKHMESPY

EINLMDELTLRGVTQYYAYVEERQKVHCLNTLFSKLQINQSIIFCNSTNRVELLAKKVTELGYSCFYSHAKMLQSHRNRV

FHDFRKGVCRNLVCSDLLTRGIDIQAVNVVINFDLPKNSETYLHRIGRSGRFGHLGLAINLVTYEDRFNLYRIEQELGTE

IQPIPQTIDKGLYVAPGATHEPEQQQQLQKAKQSQPQPQPQAAPQSQIQQRGTTSSQGPNSQVVYQSATPQGRPNGNHTP

QQQARGHPQGPYRGGVPVAR

>RPB25439.1 ATP-dependent RNA helicase DHH1 [Terfezia boudieri ATCC MYA-4762]

MSAALAEQLENTSISGQDGDWKANLKAPAKDLRPQTEDVTATKGLEFEDLYIKRELLMGIFEAGFDKPSPIQEETIPVAL

TGRDILARAKNGTGKTAAFVIPALERVNPKSTKTQALILVPTRELALQTSQVCKTLGKHLGINVMVTTGGTGLKDDIIRL

NDAVHILVGTPGRILDLAGKGVADFSECPIFIMDEADKLLSPEFTPIIEQLLAYFPVDRQTMLFSATFPLVVKSFMDKHL

RQPYEINLMDELTLRGVTQYYAFVEEKQKVHCLNTLFSKLQINQSIIFCNSTNRVELLAKKITELGYSCFYSHAKMLQNH

RNRVFHDFRNGVCRNLVCSDLLTRGIDIQAVNVVINFDFPKNAETYLHRIGRSGRFGHLGLAINLINWEDRFNLYKIEQE

LGTEIQPIPPTIDKKLYVYDSPETIPKPPPPTTAEKKEAEQRQQSRPSNTSNSHHNTPQRQQNYNSSTGSYQFPRQPQNS

QQKPRPGLGPSSQAQGVPPPTQQVPPQGHYPNPNSAAQNHQNRRPPRYNNQSQQGRPNGFNAHEGGPSVRAM

>TEB34265.1 DEAD-domain-containing protein [Coprinellus micaceus]

MAQPVSAASSSANSEDWKTSLKLPKKDVRPQTEDVTATKGLEFEDMYLRRELLMGIFEAGFEKPSPIQEEAIPIALTKRD

VLARAKNGTGKTAAFVIPSLQQVDITKPKIQALLLVPTRELALQTSQVCKILGKHMGIQVMVTTGGTTLKDDILRLSENV

HVLVGTPGRILDLAGKGVADLSECPVFVMDEADKLLSPEFAPVMEQLLSFLPQERQVMLFSATFPMIVKDFKDKHMNSPY

EINLMDELTLRGVTQYYAYVEERQKVHCLNTLFSKLQINQSIIFCNSTNRVELLAKKITELGYSCFYSHAKMLQSHRNRV

FHDFRNGVCRNLVCSDLLTRGIDIQAVNVVINFDFPKNSETYLHRIGRSGRFGHLGLAINLVTYEDRFNLYKIEQELGTE

IQPIPQSIDRSLYVAPDASEDERQKPFQNPLSQTQQIQQQRAIAPASTPPAPALAPHQQPRNGQQQPPYPTQQGVPQQQQ

PRQNGQPPRGQVAYRGGAPAAR

>XP_016289922.1 ATP-dependent RNA helicase [Kalmanozyma brasiliensis GHG001]

MSSSGPAPGQSEAAWKQQITSKLSKDERPQTEDVLNTKGNEFEDYFLKRELLMGIFEAGFERPSPIQEEAIPIALTGRDI

LARAKNGTGKTAAYVIPSLEKLNTKKNKIQAVLLVPTRELALQTSQVAKTLGKHLGVEVMVTTGGTTLRDDILRLGQTVH

MLVGTPGRILDLAGKGVADLSQCTTFVMDEADKLLSPEFTPVMEQLLSFLPKERQVMLFSATFPLIVKDFKDRNMVKPYE

INLMDELTLRGVTQYYAFVEERQKVHCLNTLFSKLQINQSIIFCNSTNRVELLAKKITELGYSCFYSHAKMLQAHRNRVF

HDFRNGACRNLVCSDLLTRGIDIQAVNVVINFDFPKNAETYLHRIGRSGRFGHLGLAINLITYEDRFNLYRIEQELGTEI

QPIPSNIDKRLYVAPSLIQEAENKNQNGNRQEGRPVIPPGQQAMHASAIPQNQNFSNTVPHRHRGGGRGGGGGGRGGRGG

HNGGAPAQ

>XP_003237974.1 ATP-dependent RNA helicase DHH1 [Trichophyton rubrum CBS 118892]

MTDALAAQLNNTKLGDSSTDNKWKEQLKIPAKDTRVQTEDVTATKGLEFEDFYIKRDLMMGIFEAGFEKPSPIQEETIPV

ALTGRDILARAKNGTGKTAAFVIPTLERTNPKIAKTQALILVPTRELALQTSQVCKTLGKHLGINVMVTTGGTGLQDDII

RLNDTVHIIVGTPGRILDLASKGVADLSECTTFVMDEADKLLSPEFTPVIEQLLTFHPKDRQVMLFSATFPIIVKTFKDK

HMRNPYEINLMDELTLRGITQYYAFVEERQKVHCLNTLFSKLQINQSIIFCNSTNRVELLAKKITELGYSCFYSHARMLQ

HNRNRVFHDFRNGVCRNLVCSDLLTRGIDIQAVNVVINFDFPKNAETYLHRIGRSGRFGHLGLAINLINWDDRYNLYKIE

QELGTEIQPIPPSIDKKLYVYETPNTIPRPISNKPSEEQKPASAESQPRKQTTNPSNGHGQYSSNRGHYGRGSYRGSRGS

GQRSNNPDSSRAAGNPQANAASSGTPGS

>XP_013339766.1 hypothetical protein AUEXF2481DRAFT_70100 [Aureobasidium subglaciale EXF-2481]

MADQLANQLEAASLNESSQQDNNWRSGLKVPAKDGRVQTEDVTATKGLEFEDFYLKRELLMGIFESGFEKPSPIQEEAIP

VALTGRDVLARAKNGTGKTAAFIIPTLERVNPKNSKIQALILVPTRELALQTSQVCKTLGKHLGVNVMVTTGGTGLKDDI

IRLNEAVHVLIGTPGRILDLASRGVADLSTAQTFVMDEADKLLSPEFTSTIDQLLSFHPPDRQVMLFSATFPVIVKEFKD

KHMKDPHEINLMDELTLRGITQYYAFVEEKQKVHCLNTLFSRLQINQSIIFCNSTTRVELLAKKVTELGYSCFYSHARML

QQNRNRVFHDFRAGHCRNLVCSDLLTRGIDIQAVNVVINFDFPKNAETYLHRIGRSGRFGHLGLAINLINWEDRFNLYRI

EQELGTEIKPIPQHIDKGLYVYDTPENIPRPITSAPQLQQQPQERRQHHQQGYRGGRGGNYQGQQGQRRGPPRNQNPSQG

GQGGQGGFQQQQAQYNQQAFPTQQQQAPSYPQGGFAQPSTFPTPQSFPQPQGQQPGR

>XP_505690.1 YALI0F21032p [Yarrowia lipolytica CLIB122]

MSEWKESLNVPKKDTRHKTEDVTATKGTGFEDFFLKRELLMGIFEAGFENPSPIQEEAIPIALAGRDILARAKNGTGKTA

AFVIPALQQVNPKVNKIQALIMVPTRELALQTSQVCKTLGKHLGIKVMVTTGGTNLRDDIMRLEDTVHVLVGTPGRVLDL

AGKGVADLSESPMFIMDEADKLLSPDFTPIIEQVLHFFPEDRQILLFSATFPLTVKAFMDRNLHKPYEINLMDELTLRGI

TQYYAFVDEKQKLHCLNTLFSKLDINQSIIFCNSTVRVELLARKITELGYSCYYSHAKMIQSHRNRVFHEFRNGTCRNLV

CSDLLTRGIDIQAVNVVINFDFPKNAETYLHRIGRSGRFGHLGIAINLINWNDRYNLYKIEQELGTEIKPIPAQIDKNLY

VAESSENIPRPFPIADMPRGKESKRNEQFQPQQNQNQQQQQDGQNQHSLGPVPPQGPPQGIPGPGYGYPPPQGFPQGMPP

QGMPPQGAGGYMPPTPYGYQQQGFPPQPPQGFNNGQPQQPSQ

>DAA72877.1 TPA_exp: Uncharacterized protein A8136_5321 [Trichophyton benhamiae CBS 112371]

MTDALAAQLNNTKLGDSSTDNKWKEQLKIPAKDTRVQTEDVTATKGLEFEDFYIKRDLMMGIFEAGFEKPSPIQEETIPV

ALTGRDILARAKNGTGKTAAFVIPTLERTNPKIAKTQALILVPTRELALQTSQVCKTLGKHLGINVMVTTGGTGLQDDII

RLNDTVHIIVGTPGRILDLASKGVADLSECTTFVMDEADKLLSPEFTPVIEQLLTFHPKDRQVMLFSATFPIIVKTFKDK

HMRNPYEINLMDELTLRGITQYYAFVEERQKVHCLNTLFSKLQINQSIIFCNSTNRVELLAKKITELGYSCFYSHARMLQ

HNRNRVFHDFRNGVCRNLVCSDLLTRGIDIQAVNVVINFDFPKNAETYLHRIGRSGRFGHLGLAINLINWDDRYNLYKIE

QELGTEIQPIPPSIDKKLYVYETPNTIPRPISNNPSEEQKSAGQESQPRRQTTNPSNGHGQYSSNRGHYSRGSYRGSRGS

GQRSNNPDSSRAAGNSQANAANSGTPGS

>PVF93514.1 putative ATP-dependent RNA helicase DHH1 [Serendipita vermifera 'subsp. bescii']

MSTTAFNANAPKGDATWRDGLKAPPKDTRVKTQDVMTTSNVEWEDMFLRRELLMGIFEEGFEKPSPIQESAIPIALSKRD

ILARAKNGTGKTAAFVIPSLQQVNTKSPKVQALLLVPTRELALQTSQVAKNLGKHIEGLQVMVSTGGTTLKDDILRLNEP

VHVLVGTPGRILDLASKGVADLSECGVFVMDEADKLLSPEFTPIMESLLNHLPEERQVMLFSATFPMIVKDFKEKHMRKP

HEINLMEELTLKGVTQYYAFVEERQKVHCLNTLFSKLQINQSIIFCNSTNRVELLAKKITELGYSCFYSHAKMLQSHRNR

VFHDFRNGVCRNLVCSDLLTRGIDIQAVNVVINFDFPKNAETYLHRIGRSGRFGHLGLAINLLTYEDRFNLYTIEQELGT

EIAPIPPVIDRSLYVAGSAPPASLAPPSNANGRAGSGTSTPSNEAARRRNTPTPQLQQQSQYNPTDYSGNAAMIARAQGP

YQQPVNVQMQAQMQQQQLQQQALQAQVLQAQALQAQQYQASNGAENVRQYSNHHQGQGERGHGRGRGRGGRGGRGGANGN

VA

>SAM82584.1 probable ATP-dependent RNA helicase DHH1 [Ustilago bromivora]

MSLAGPASGQSEAAWKQQIASKIAKDERPQTEDVLNTKGNEFEDYFLKRELLMGIFEAGFERPSPIQEEAIPIALTGRDI

LARAKNGTGKTAAYVIPSLEKLNTKKNKIQAVLLVPTRELALQTSQVAKTLGKHLGVEVMVTTGGTTLRDDILRLGQTVH

VLVGTPGRILDLAGKGVADLSQCHTFVMDEADKLLSPEFTPVMEQLLSFLPKERQVMLFSATFPLIVKDFKDRNMVKPYE

INLMDELTLRGVTQYYAFVEERQKVHCLNTLFSKLQINQSIIFCNSTNRVELLAKKITELGYSCFYSHAKMLQSHRNRVF

HDFRNGACRNLVCSDLLTRGIDIQAVNVVINFDFPKNAETYLHRIGRSGRFGHLGLAINLITYEDRFNLYRIEQELGTEI

QPIPSNIDKRLYVAPSLIQEGEKGGQNGNRQEGRPVIPPGQQAMHASAIPQNQNFSNSVPHRHRGGGPRGGGGGAGRGQG

GATPAQ

>CEH14760.1 atp-dependent rna helicase dhh1 [Ceraceosorus bombacis]

MATSSASSAAQDAQWRAEIAARVEKDGRPQTEDVTATKGNDFEDYFLKRPLLMGIFEAGFERPSPIQEEAIPIALTGRDI

LARAKNGTGKTAAYVIPSLERLKPEKSAIQAVLLVPTRELALQTSQVVKTLGKHMGIQVMVTTGGTTLRDDILRLSQTVH

ILVGTPGRILDLASKGIADLTQCSTFVMDEADKLLSPEFTPVMEQLLGLLPRERQVMLFSATFPLIVKDFKDKHMVKPYE

INLMDELTLRGVTQYYAFVEERQKVHCLNTLFSRLQINQSIIFCNSTNRVELLAKKITELGYSCFYSHAKMLQAHRNRVF

HDFRNGACRNLVCSDLLTRGIDIQAVNVVINFDFPKNAETYLHRIGRSGRFGHLGLAINLITYEDRFNLYRIEQELGTEI

APIPPTIDKRLYVAPSLITEDDGKNANGAKAHQNGAPVIPPTQRAMHQAIIPQSQDFTQSSGDLCTPTLSPIAFSALSHL

RRMHQHPTVTPVWSINAFPTYLESRLHPSKPSRFRGIERGHRSPNSKAIDDRQSVYVY

>KAF4556189.1 ATP-dependent RNA helicase dhh1 [Elsinoe fawcettii]

MASELASQLQNTSLNESEAQGDDWRAGLKKPNKDGRQQTEDVTATKGLEFEDFYIKRELMMGIFEAGFEKPSPIQEEAIP

VALTGRDVLARAKNGTGKTGSFVIPTLERVNPKNSKIQALLLVPTRELALQTSQVCKTLGKHLGINVMVTTGGTSLRDDI

IRLNETVHILVGTPGRILDLAAKGIADLSACQTFVMDEADKLLSPEFTSQIDQLIAFHPKDRQIMLFSATFPVIVKDFKD

KHMNDPHEINLMDELTLRGITQYYAFVEEKQKVHCLNTLFSRLQINQSIIFCNSTQRVELLAKRVTELGYSCYYSHARMM

QHARNRVFHDFRAGHCRNLVCSDLLTRGIDIQAVNVVINFDFPKNAETYLHRIGRSGRFGHLGLAINLISWEDRFNLYRI

EQELGTEIKPIPSNIDKSLYVYDTPENIPRPISGAPQQRQPQGPSDNVRGRGGFQQRGMNRGRGGYQNQGQGQGRGAPRD

PNQQRPQNGFAPRGQQQPRAPPAAAQS

>CCX07757.1 Similar to ATP-dependent RNA helicase dhh1; acc. no. A1CJ18 [Pyronema omphalodes CBS 100304]

MATSLAEQVENMNLTGDEWKRELKIPVKDNRHQTEDVTNTKGLDFEDLYIKRGLLMGIFEAGFEKPSPIQEETIPVALTG

RDILARAKNGTGKTAAFVIPALERVNAKSPKTQALILVPTRELALQTSQVCKTLGKHLGVNVMVTTGGTGLKDDIIRLNE

AVHILVGTPGRILDLAGKGVADFTECPTFIMDEADKLLSPEFTPIIEQLLAYFPKDRQIMLFSATFPLVVKSFMDKHLNQ

PYEINLMDELTLRGVTQYYAFVEEKQKVHCLNTLFSKLQINQSIIFCNSTNRVELLAKKITELGYSCFYSHAKMLQNHRN

RVFHDFRNGVCRNLVCSDLLTRGIDIQAVNVVINFDFPKNAETYLHRIGRSGRFGHLGLAINLINWDDRFNLYKIEQELG

TEIQPIPAIIDKALYVYDSPETIPRPPPAPQQNTRPNQHQQPGQNQPGNNQYQPRNGNSSPRGQGQGQGQGQGPANQNRP

RQYPQSNNQPQYQSQNVAPTQQQQQQGQHQQAIQGRRPPQQRYNNQQQQRPNGYTADSGPVPPRGQ

>KXN89345.1 ATP-dependent RNA helicase dhh1 [Leucoagaricus sp. SymC.cos]

MFSERTAHWWKRCSLFVATQLALNQLSSPPLCYHQFSVLHITCISLTHPARVCSDIAQSPVPPEDTFSPAPFAEQHLMSQ

PAPSSSSTASRDPRTPSNTPHNSSWKAGLRPPPKDLRPQTEDVTATKGLEFEEMSLRRELLMGIFEAGFEKPSPIQEEAI

PIALTKRDVLARAKNGTGKTAAFVIPSLQQIDIDKPKIQALLLVPTRELALQTSQVCKTLGKHMGLQVMVTTGGTTLKDD

ILRLSETVHVLVGTPGRILDLAGKGVADLSECPVFVMDEADKLLSPEFAPVMEQLLSFLPADRQVMLFSATFPMIVKDFK

DKHMRSPYEINLMDELTLRGVTQYYAFVEERQKVHCLNTLFSKLQINQSIIFCNSTNRVELLAKKVTELGYSCFYSHAKM

LQSHRNRVFHDFRNGVSRNLVCSDLLTRGIDIQAVNVVINFDFPKNSETYLHRIGRSGRFGHLGLAINLVTYEDRFNLYK

IEQELGTEIQPIPQNIDRGLYTAPAPAPVPVQAQAQRTGPPAPPQIPQQLQYQPQPVYNGQIPMRPNGGLPPQAQPMRAQ

PAYRGGVPVGR

>RFU24220.1 hypothetical protein B7463_g12115, partial [Scytalidium lignicola]

MADSLANKLESTKLTDNGSSDGWKADLKIPAKDTRQQTEDVTATKGLEFEDFGLKRDLLMGIFEAGFEKPSPIQEEAIPV

ALTGRDILARAKNGTGKTAAFVIPALEKINPKSSKIQALILVPTRELALQTSQVCKTLGKHLGVNVMVTTGGTTLRDDIV

RLQDPVHIIVGTPGRILDLAGKNVADLSECPMFIMDEADKLLSPEFTPVIEQLLQFHPKDRQVMLFSATFPITVKSFSDK

NMDSPYEINLMDELTLRGITQYYAFVEEKQKVHCLNTLFSKLQINQSIIFCNSTNRVELLAKKITELGYSCFYSHARMLQ

ANRNRVFHDFRNGVCRNLVCSDLLTRGIDIQAVNVVINFDFPKNAETYLHRIGRSGRFGHLGLAINLINWEDRFNLYNIE

RDLGTEIQPIPATIDKSLYVYDTPENIPRPISNFQTNRQPTGRDPRDQQTSQQPQQQQNNQRGGHGGYSNGRPNGAGQAQ

AQRQPQGQRQGQGFSRGGGRFSDQSGRRGGGHGSQGQRQSVPDGQRGGGRGQVAAQ

>KAF1982015.1 DEAD-domain-containing protein [Aulographum hederae CBS 113979]

MTTDITNRLQNPTLNDTAQPASSDWRAGLTAPTKDSRQQTEDVTATKGLEFEEFFIKRELMMGIFEAGFEKPSPIQEEAI

PVALTGRDILARAKNGTGKTAAYVIPLLERINPKSPKIQALILVPTRELALQTSQVCKMLGKHLGINVMVSTGGTGLRDD

IIRLSENVHVVVGTPGRILDLAGKGVANLETCQTFVMDEADKLLSPEFTQVIEQLLAFHPKDRQMMLFSATFPLVVKDFK

DKHMNSPYEINLMEELTLRGITQYYAFVEEKQKVHCLNTLFNKLQINQSIIFCNSTNRVELLAKKITELGYSCFYSHARM

LQQNRNRVFHDFRNGVCRNLVCSDLLTRGIDIQAVNVVINFDFPKNAETYLHRIGRSGRFGHLGLAINLISWEDRFNLYR

IEQELGTEVLPIPSTIEKSLYVYESPESIPRPVSNAGPQQPRDNSNPQGRDPSRQNWQRNQRGGGNFQGQRRPPPRDPNN

QQQQGQYRPNPNSNGQQRATRPPPQQ

>GBF66219.1 ATP-dependent RNA helicase [Trichophyton mentagrophytes]

MTDALTAQLNNTKLGDSSTDNKWKEQLKIPAKDTRVQTEDVTATKGLEFEDFYIKRDLMMGIFEAGFEKPSPIQEETIPV

ALTGRDILARAKNGTGKTAAFVIPTLERTNPKIAKTQALILVPTRELALQTSQVCKTLGKHLGINVMVTTGGTGLQDDII

RLNDTVHIIVGTPGRILDLASKGVADLSECTTFVMDEADKLLSPEFTPVIEQLLTFHPKDRQVMLFSATFPIIVKTFKDK

HMRNPYEINLMDELTLRGITQYYAFVEERQKVHCLNTLFSKLQINQSIIFCNSTNRVELLAKKITELGYSCFYSHARMLQ

HNRNRVFHDFRNGVCRNLVCSDLLTRGIDIQAVNVVINFDFPKNAETYLHRIGRSGRFGHLGLAINLINWDDRYNLYKIE

QELGTEIQPIPPSIDKKLYVYETPNTIPRPISNKPSEEQKPAGQESQPRRQTTNPSNGHGQYSSNRGHYGRGSYRGSRGS

GQRSNNPDSSRAAGNPQANAANSGTPGS

>XP_013332352.1 RNA helicase [Rasamsonia emersonii CBS 393.64]

MADALASQLNNTKLGEASMDNSWKEQLKIPAKDTRVQTEDVTATKGLEFEDFYIKRELMMGIFEAGFEKPSPIQEETIPV

ALTGRDILARAKNGTGKTAAFVIPTLERINPKSTKTQALILVPTRELALQTSQVCKTLGKHLGINVMVTTGGTGLMDDII

RLNDAVHIIVGTPGRVLDLASKGVADLSECPTFVMDEADKLLSPEFTPVIEQLLSFHPKDRQVMLFSATFPLIVKSFKDK

HMRNPYEINLMDELTLRGITQYYAFVEEKQKVHCLNTLFSKLQINQSIIFCNSTNRVELLAKKITELGYSCFYSHARMPQ

QNRNRVFHDFRNGVCRNLVCSDLLTRGIDIQAVNVVINFDFPKNAETYLHRIGRSGRFGHLGLAINLINWDDRFNLYKIE

QELGTEIQPIPQTIDKKLYVYESPETIPRPIASAPQPRQASAVDNDKGNGEQQPRRHGNHANGGHYNSNRGRGSYRGARG

HGQRRGGHSDGHKSMGAPAQAGSKPQPMPAS

>XP_016263525.1 ATP-dependent RNA helicase DHH1 [Exophiala oligosperma]

MTDALASQLNKTSLNDGADESNWKESLKIPNKDTRQQTEDVTATKGLDFEDFYIKRQLMMGIFEAGFEKPSPIQEETIPV

ALTGRDILARAKNGTGKTAAFVIPTLERINPKNPKTQALLLVPTRELALQTSQVCKLLGKHLGINVMVTTGGTGLKDDII

RLGETVHIIVGTPGRILDLASKGVADLSECPIFVMDEADKLLSPEFTVVIEQLLGFLPRDRQVMLFSATFPMMVKTFKDK

HMRNPYEINLMDELTLRGITQFYAFVEEKQKVHCLNTLFSKLQINQSIIFCNSTNRVELLAKKITELGYSCFYSHAKMLQ

QNRNKVFHDFRAGVCRNLVCSDLLTRGIDIQAVNVVINFDFPKNAETYLHRIGRSGRFGHLGLAINLINWDDRFNLYKIE

QELGTEIQPIPQSIDKKLYVYDSPENIPRPMPPPAPASRANNAPMMSANASMSPNANGQGQGRRQYNQSNGYGQQYNNQG

RSGYRGRGRGQGGRGRGGYAHQAAAPVGQTAQ

>THZ16828.1 DEAD-domain-containing protein [Aureobasidium pullulans]

MADQLASQLEAASLNESNQADGNWRSGLKVPAKDGRVQTEDVTATKGLEFEDFYLKRELLMGIFESGFEKPSPIQEEAIP

VALTGRDVLARAKNGTGKTAAFIIPTLERVNPKNSKIQALILVPTRELALQTSQVCKTLGKHLGVNVMVTTGGTGLKDDI

IRLNEAVHVLIGTPGRILDLASRGVADLSTAQTFVMDEADKLLSPEFTSTIDQLLSFHPPDRQVMLFSATFPVIVKEFKD

KHMKDPHEINLMDELTLRGITQYYAFVEEKQKVHCLNTLFSRLQINQSIIFCNSTTRVELLAKKVTELGYSCFYSHARML

QQNRNRVFHDFRAGHCRNLVCSDLLTRGIDIQAVNVVINFDFPKNAETYLHRIGRSGRFGHLGLAINLINWEDRFNLYRI

EQELGTEIKPIPQHIDKGLYVYDTPENIPRPITSAPQLQQQPQERRQHHQPGYRGGRGGNYQGQQGQRRGPPRNQYPSQG

GQGGGFQQQQAQYNQQAFPTQQAPQQAPSYPQGGFSQPSAFPTPQSFPQPQGQQPSR

>CDI54154.1 probable ATP-dependent RNA helicase DHH1, partial [Melanopsichium pennsylvanicum 4]

MSASGPAPGQSEAQWKQQITSQVTKDERPQTEDVLNTKGNEFEDYFLKRELLMGIFEAGFERPSPIQEEAIPIALTGRDI

LARAKNGTGKTAAYVIPSLEKLNTKKNKIQAVLLVPTRELALQTSQVAKTLGKHLGVEVMVTTGGTTLKDDILRLGQTVH

MLVGTPGRILDLAGKGVADLSQCTTFVMDEADKLLSPEFTPVMEQLLSFLPKERQVMLFSATFPLIVKDFKDRNMVKPYE

INLMDELTLRGVTQYYAFVEERQKVHCLNTLFSKLQINQSIIFCNSTNRVELLAKKITELGYSCFYSHAKMLQAHRNRVF

HDFRNGACRNLVCSDLLTRGIDIQAVNVVINFDFPKNAETYLHRIGRSGRFGHLGLAINLITYEDRFNLYRIEQELGTEI

QPIPSNIDKRLYVAPSLIQEAEGKGQNGNRQEGRPVIPPGQQAMHAAAIPQNQNFSNTVPHRHRGSGGGGQRGGGRGGGG

REGPQDHGQVSAQ

>KDB22915.1 ATP-dependent RNA helicase dhh1 [Trichophyton interdigitale MR816]

MTDALAAQLNNTKLGDSSTDNKWKEQLKIPAKDTRVQTEDVTATKGLEFEDFYIKRDLMMGIFEAGFEKPSPIQEETIPV

ALTGRDILARAKNGTGKTAAFVIPTLERTNPKIAKTQALILVPTRELALQTSQVCKTLGKHLGINVMVTTGGTGLQDDII

RLNDTVHIIVGTPGRILDLASKGVADLSECTTFVMDEADKLLSPEFTPVIEQLLTFHPKDRQVMLFSATFPIIVKTFKDK

HMRNPYEINLMDELTLRGITQYYAFVEERQKVHCLNTLFSKLQINQSIIFCNSTNRVELLAKKITELGYSCFYSHARMLQ

HNRNRVFHDFRNGVCRNLVCSDLLTRGIDIQAVNVVINFDFPKNAETYLHRIGRSGRFGHLGLAINLINWDDRYNLYKIE

QELGTEIQPIPPSIDKKLYVYETPNTIPRPISNKPSEEQKPAGQESQPRRQTTNPSNGHGQYSSNRGHYGRGSYRGSRGS

GQRSNNPDSSRAAGNPQANAANSGTPGS

>KAF3894528.1 ATP-dependent RNA helicase [Trichophyton interdigitale]

MTDALAAQLNNTKLGDSSTDNKWKEQLKIPAKDTRVQTEDVTATKGLEFEDFYIKRDLMMGIFEAGFEKPSPIQEETIPV

ALTGRDILARAKNGTGKTAAFVIPTLERTNPKIAKTQALILVPTRELALQTSQVCKTLGKHLGINVMVTTGGTGLQDDII

RLNDTVHIIVGTPGRILDLASKGVADLSECTTFVMDEADKLLSPEFTPVIEQLLTFHPKDRQVMLFSATFPIIVKTFKDK

HMRNPYEINLMDELTLRGITQYYAFVEERQKVHCLNTLFSKLQINQSIIFCNSTNRVELLAKKITELGYSCFYSHARMLQ

HNRNRVFHDFRNGVCRNLVCSDLLTRGIDIQAVNVVINFDFPKNAETYLHRIGRSGRFGHLGLAINLINWDDRYNLYKIE

QELGTEIQPIPPSIDKKLYVYETPNTIPRPISNKPSEEQKPVGQESQPRRQTTNPSNGHGQYSSNRGHYGRGSYRGSRGS

GQRSNNPDSSRAAGNPQANAANSGTPGS

>XP_007875348.1 ATP-dependent RNA helicase dhh1 [Pneumocystis murina B123]

MSSIRDSGSVAVDSTKMYDDWKKTLNLSVKDTRPQTEDVTKTKGNEFEDFYLKRELLMGIFEAGFERPSPIQEESIPIAL

TGRDILARAKNGTGKTAAFVIPALEKLNPKKPKIQALILVPTRELALQTSHVCKTLGKHMGINVMVTTGGTSLQQDIIRL

NDPVHVIVGTPGRILDLAGKGVAEFSECPTFIMDEADKLLSIEFTPVIEQLLAYFPKDRQIMLYSATFPLVVKSFMDKHL

SKPYEINLMDELTLRGVTQYYAFVEEKQKVHCLNTLFSKLQINQSIIFCNSTNRVELLAKKITELGYSCFYSHAKMLQNH

RNRVFHDFRNGVCRNLVCSDLLTRGIDIQAVNVVINFDFPKNSETYLHRIGRSGRFGHLGLAINLINWDDRFNLYKIEQE

LSTEIQPIPSIIDKRLYVSPSALDEPVSNKAPPKQHTAISQSNGNSSNQSNNEQSYVRQHSQRGRGGNRARFHGGRYSNR

ANGHIISSNDNIVRD

>TCD65119.1 DExD/H-box ATP-dependent RNA helicase dhh1 [Steccherinum ochraceum]

MSQAQQQARPSSSTPRSNNGDNAWKSQLNVPAKDDRPQTDDVLATKGLEFEDMQLRRELLMGIFEAGFERPSPIQEEAIP

IALTKRDVLARAKNGTGKTAAFVIPSLQQIDINKRKIQALLLVPTRELALQTAQVCKILGKHMGLNVMVTTGGTTLKDDI

MRLSEAVHVLVGTPGRILDLAGKNVADLSECPVFVMDEADKLLSPEFAPVMEQLLSYLPKDRQVMLFSATFPLIVKDFKD

KHMESPYEINLMDELTLRGVTQYYAYVEERQKVHCLNTLFSKLQINQSIIFCNSTNRVELLAKKVTELGYSCFYSHAKML

QSHRNRVFHDFRQGVCRNLVCSDLLTRGIDIQAVNVVINFDLPKNSETYLHRIGRSGRFGHLGLAINLVTYEDRFNLYKI

EQELGTEIQPIPQIIDKGLYVAPSAIAEGEQQKQKQTQQQQQAQPQPQPQPAPAPSAQQQQQQRQQQAASPQQVVYQSTA

GSQGRPNGAPVGANGQPPRGYQGAYRGGVPVAR

>EZF29913.1 ATP-dependent RNA helicase dhh1 [Trichophyton interdigitale H6]

MTDALAAQLNNTKLGDSSTDNKWKEQLKIPAKDTRVQTEDVTATKGLEFEDFYIKRDLMMGIFEAGFEKPSPIQEETIPV

ALTGRDILARAKNGTGKTAAFVIPTLERTNPKIAKTQALILVPTRELALQTSQVCKTLGKHLGINVMVTTGGTGLQDDII

RLNDTVHIIVGTPGRILDLASKGVADLSECTTFVMDEADKLLSPEFTPVIEQLLTFHPKDRQVMLFSATFPIIVKTFKDK

HMRNPYEINLMDELTLRGITQYYAFVEERQKVHCLNTLFSKLQINQSIIFCNSTNRVELLAKKITELGYSCFYSHARMLQ

HNRNRVFHDFRNGVCRNLVCSDLLTRGIDIQAVNVVINFDFPKNAETYLHRIGRSGRFGHLGLAINLINWDDRYNLYKIE

QELGTEIQPIPPSIDKKLYVYETPNTIPRPISNKPSEEQKPAGQESQPRRQTANPSNGHGQYSSNRGHYGRGSYRGSRGS

GQRSNNPDSSRAAGNPQANAANSGTPGS

>THW07808.1 DEAD-domain-containing protein [Aureobasidium pullulans]

MPIPAPKLPRNHTLTRSPLSESNQADGNWRSGLKVPAKDGRVQTEDVTATKGLEFEDFYLKRELLMGIFESGFEKPSPIQ

EEAIPVALTGRDVLARAKNGTGKTAAFIIPTLERVNPKNSKIQALILVPTRELALQTSQVCKTLGKHLGVNVMVTTGGTG

LKDDIIRLNEAVHVLIGTPGRILDLASRGVADLSTAQTFVMDEADKLLSPEFTSTIDQLLSFHPPDRQVMLFSATFPVIV

KEFKDKHMKDPHEINLMDELTLRGITQYYAFVEEKQKVHCLNTLFSRLQINQSIIFCNSTTRVELLAKKVTELGYSCFYS

HARMLQQNRNRVFHDFRAGHCRNLVCSDLLTRGIDIQAVNVVINFDFPKNAETYLHRIGRSGRFGHLGLAINLINWEDRF

NLYRIEQELGTEIKPIPQHIDKGLYVYDTPENIPRPITSAPQLQQQPQERRQHHQQGYRGGRGGNYQGQQGQRRGPPRNQ

YPSQGGQGGGFQQQQAQYNQQAFPTQQAPQQAPSYPQGGFSQPSAFPTPQSFPQPQGQQPSR

>XP_007272025.1 DEAD-domain-containing protein [Fomitiporia mediterranea MF3/22]

MSGVTSPSASSSTGTGSGQDWRAGLRAPPKDNRPQTEDVTATKGMEFEDMFLRRELLMGIFEAGFERPSPIQEEAIPVAL

SKRDVLARAKNGTGKTAAFVIPTLQQLDVSKNRIQALLLVPTRELALQTAQVCKILGKHLGANVMVTTGGTTLKDDIMRL

SEPVHVLVGTPGRILDLASKGVADLSECPVFVMDEADKLLSPEFTPVMEQLLSFLPESRQVMLFSATFPMIVKDFKDKHM

DSPYEINLMDELTLRGVTQYYAFVEERQKVHCLNTLFSKLQINQSIIFCNSTNRVELLAKKVTELGYSCFYSHAKMLQSH

RNRVFHDFRNGVCRNLVCSDLLTRGIDIQAVNVVINFDFPKNSETYLHRIGRSGRYGHLGLAINLITYEDRFNLYKIEQE

LGTEIQPIPAQIDRSLYVAPAGGDEPSNQAEQQLQQQVQKQQQQQQQQQLQKQQAVRQAMSAQVAPQPQQIIYQSNGQAP

QSQQRVVPNGPMPAQLQQQQLAAAAARNQQYQAYAQVQPGVAAPRPGAQPVRR

>EJD39556.1 DEAD-domain-containing protein [Auricularia subglabra TFB-10046 SS5]

MAQNQASSSSNSNDWKTALNLPKKDLRPQTEDVTNTKGIEFEEMYLRRELLMGIFEAGFERPSPIQEEAIPVALARRDIL

ARAKNGTGKTAAFVIPTLQQVDVSKPKIQGVLLVPTRELALQTSQVCKILGKHMGVQVMVTTGGTTLKDDIMRLSEVVHL

LVGTPGRILDLAGKGVADLSECNVFVMDEADKLLSPEFSPVMEQLLEYLPSNRQVMLFSATFPIIVKDFKDKHMKNPHEI

NLMEELTLRGVTQYYAYVEERQKVHCLNTLFSKLQINQSIIFCNSTNRVELLARKVTELGYSCFFSHAKMLQAHRNRVFH

DFRNGVCRNLVCSDLLTRGIDIQAVNVVINFDFPKNSETYLHRIGRSGRFGHLGLAINLVTYEDRFNLFRVEQELGTEIQ

PIPQTIDKSLYVAPPGADEPGPTRKQQKQGQQGQQTPQSRGQTPAQRAGQPAQLTRQQPTFQQQQILNGYQQQQQQQQQT

PVTR

>RVD81715.1 hypothetical protein DFL_009565 [Arthrobotrys flagrans]

MASSLTEKLESTTLSDADWRAQLKAPVKDLRPQTEDVTATKGLDFEELYIKRELLMGIFEAGFEKPSPIQEETIPVALTG

RDILARAKNGTGKTAAFVIPALERVNSKSPKTQALILVPTRELALQTSQVCKTLGKHLGINVMVTTGGTVLKDDIIRLNE

AVHVLVGTPGRILDLAGKGVADFSECPTFIMDEADKLLSPEFTPIIEQLLAYFPSDRQIMLFSATFPLVVKSFMDKHLKQ

PYEINLMDELTLRGVTQYYAFVEEKQKVHCLNTLFSKLQINQSIIFCNSTNRVELLAKKITELGYSCFYSHAKMLQNHRN

RVFHDFRNGVCRNLVCSDLLTRGIDIQAVNVVINFDFPKNAETYLHRIGRSGRFGHLGLAINLINWDDRFNLYKIEQELG

TEIQPIPAQIDKKLYVYDSPESIPRPPAAQNSIRPAPSPATANPVSQSTNRPDARSSPHPTQLQAVRNNKTRGNHSMGQN

FRGGRGPQRGYGHHQSYGRRNDNGHGAEGALPNSHNA

>THX05565.1 DEAD-domain-containing protein [Aureobasidium pullulans]

MADQLASQLEAASLNESNQVDGNWRSGLKVPAKDGRVQTEDVTATKGLEFEDFYLKRELLMGIFESGFEKPSPIQEEAIP

VALTGRDVLARAKNGTGKTAAFIIPTLERVNPKNSKIQALILVPTRELALQTSQVCKTLGKHLGVNVMVTTGGTGLKDDI

IRLNEAVHVLIGTPGRILDLASRGVADLSTAQTFVMDEADKLLSPEFTSTIDQLLSFHPPDRQVMLFSATFPVIVKEFKD

KHMKDPHEINLMDELTLRGITQYYAFVEEKQKVHCLNTLFSRLQINQSIIFCNSTTRVELLAKKVTELGYSCFYSHARML

QQNRNRVFHDFRAGHCRNLVCSDLLTRGIDIQAVNVVINFDFPKNAETYLHRIGRSGRFGHLGLAINLINWEDRFNLYRI

EQELGTEIKPIPQHIDKGLYVYDTPENIPRPITSAPQLQQQPQERRQHHQQGYRGGRGGNYQGQQGQRRGPPRNQYPSQG

GQGGGFQQQQAQYNQQAFPTQQAPQQAPSYPQGGFSQPSAFPTPQSFPQPQGQQPSR

>CUS07673.1 unnamed protein product [Tuber aestivum]

MATALADQLEATSLGGGQDPNWCSKLKAPKKDLRPQTEDVTATKGLDFEDLYIKRELLMGIFEAGFEKPSPIQEETIPVA

LTGRDMLARAKNGTGKTAAFVIPALERVNPKSTKTQALILVPTRELALQTSQVCKSLGKHLGINVMVTTGGTGLKDDIIR

LNEPVHILVGTPGRILDLAGKTVADFSECPTFIMDEADKLLSPEFTPIIEQLLAYFPSDRQIMLFSATFPLVVKSFMDKH

LNKPYEINLMDELTLRGVTQYYAFVEEKQKVHCLNTLFSKLQINQSIIFCNSTNRVELLAKKITELGYSCFYSHAKMLQN

HRNRVFHDFRGGVCRNLVCSDLLTRGIDIQAVNVVINFDFPKNAETYLHRIGRSGRFGHLGLAINLINWDDRFNLYKIEQ

ELGTEIFPIPPVIDKKLYVYDSPESIPRPPPAPQQPRQETREHNQRQSTQHQNQSSQQQYQSRQNQHHAETPGNPQQQQQ

QRPMSHRQQGPRNYQNSGPHRGPFQEPGGAGRGQGQNRRPPRYDDRQGRSNGFGPGDPGPTSRAQ

>XP_016228234.1 ATP-dependent RNA helicase DHH1 [Exophiala mesophila]

MADALASQLNKTSLTDGPDDSSWKQGLKIPTKDGRQQTEDVLATKGLEFEDFYIKRELMMGIFEAGFEKPSPIQEETIPV

ALTGRDILARAKNGTGKTAAFVIPTLERINPKNPKTQALLLVPTRELALQTSQVCKTLGKHLGINVMVTTGGTGLKDDIL

RLGDTVHIIIATPGRILDLASKGIADLSECSIFVMDEADKLLSPEFTVVIEQLLSFHPKDRQVMLFSATFPMIVKHFKDK

HMRNPYEINLMDELTLRGITQYYAFVEEKQKVHCLNTLFSKLQINQSIIFCNSTNRVELLAKKITELGYSCFYSHAKMLQ

QNRNKVFHDFRAGVSRNLVCSDLLTRGIDIQAVNVVINFDFPKNAETYLHRIGRSGRFGHLGLAINLINWDDRFNLYKIE

QELGTEIQPIPPSIDKSLYVYDNPENIPRSMPPPAQARPSYSSTNAASTSHNGQVQSQAQGQRRPQGQSNGFPQQFNNQG

RGGYRGRGRGQGTRGGGRGGHQPALPVGQTTQ

>THW21388.1 DEAD-domain-containing protein [Aureobasidium pullulans]

MADQLASQLEAASLNESNQADGNWRSGLKVPAKDGRVQTEDVTATKGLEFEDFYLKRELLMGIFESGFEKPSPIQEEAIP

VALTGRDVLARAKNGTGKTAAFIIPTLERVNPKNSKIQALILVPTRELALQTSQVCKTLGKHLGVNVMVTTGGTGLKDDI

IRLNEAVHVLIGTPGRILDLASRGVADLSTAQTFVMDEADKLLSPEFTSTIDQLLSFHPPDRQVMLFSATFPVIVKEFKD

KHMKDPHEINLMDELTLRGITQYYAFVEEKQKVHCLNTLFSRLQINQSIIFCNSTTRVELLAKKVTELGYSCFYSHARML

QQNRNRVFHDFRAGHCRNLVCSDLLTRGIDIQAVNVVINFDFPKNAETYLHRIGRSGRFGHLGLAINLINWEDRFNLYRI

EQELGTEIKPIPQHIDKGLYVYDTPENIPRPITSAPQLQQQPQERRQHHQQGYRGGRGGNYQGQQGQRRGPPRNQYPSQG

GQGGGFQQQQAQYNQQAFPTQQAPQQAPSYPQGGFSQPSAFPTPQSFPQPQGQQPSR

>THV86220.1 DEAD-domain-containing protein [Aureobasidium pullulans]

MPIPAPKLTRNHTLTRSPLSESNQADGNWRSGLKVPAKDGRVQTEDVTATKGLEFEDFYLKRELLMGIFESGFEKPSPIQ

EEAIPVALTGRDVLARAKNGTGKTAAFIIPTLERVNPKNSKIQALILVPTRELALQTSQVCKTLGKHLGVNVMVTTGGTG

LKDDIIRLNEAVHVLIGTPGRILDLASRGVADLSTAQTFVMDEADKLLSPEFTSTIDQLLSFHPPDRQVMLFSATFPVIV

KEFKDKHMKDPHEINLMDELTLRGITQYYAFVEEKQKVHCLNTLFSRLQINQSIIFCNSTTRVELLAKKVTELGYSCFYS

HARMLQQNRNRVFHDFRAGHCRNLVCSDLLTRGIDIQAVNVVINFDFPKNAETYLHRIGRSGRFGHLGLAINLINWEDRF

SLYRIEQELGTEIKPIPQHIDKGLYVYDTPENIPRPITSAPQLQQQPQERRQHHQQGYRGGRGGNYQGQQGQRRGPPRNQ

YPSQGGQGGGFQQQQAQYNQQAFPTQQAPQQAPQQAPSYPQGGFSQPSAFPTPQSFPQPQGQQPSR

>OBW67679.1 Pentafunctional AROM polypeptide [Aureobasidium pullulans]

MPIPASKLTRNHTLTRSPLSESNQADGNWRSGLKVPAKDGRVQTEDVTATKGLEFEDFYLKRELLMGIFESGFEKPSPIQ

EEAIPVALTGRDVLARAKNGTGKTAAFIIPTLERVNPKNSKIQALILVPTRELALQTSQVCKTLGKHLGVNVMVTTGGTG

LKDDIIRLNEAVHVLIGTPGRILDLASRGVADLSTAQTFVMDEADKLLSPEFTSTIDQLLSFHPPDRQVMLFSATFPVIV

KEFKDKHMKDPHEINLMDELTLRGITQYYAFVEEKQKVHCLNTLFSRLQINQSIIFCNSTTRVELLAKKVTELGYSCFYS

HARMLQQNRNRVFHDFRAGHCRNLVCSDLLTRGIDIQAVNVVINFDFPKNAETYLHRIGRSGRFGHLGLAINLINWEDRF

NLYRIEQELGTEIKPIPQHIDKGLYVYDTPENIPRPITSAPQLQQQPQERRQHHQQGYRGGRGGNYQGQQGQRRGPPRNQ

YPSQGGQGGGFQQQQAQYNQQAFPTQQAPQQAPSYPQGGFSQPSAFPTPQSFPQPQGQQPSR

>RVX74898.1 ATP-dependent RNA helicase DHH1 [Exophiala mesophila]

MAEALASQLNKTSLTDGPDDSSWKQGLKIPTKDGRQQTEDVLATKGLEFEDFYIKRELMMGIFEAGFEKPSPIQEETIPV

ALTGRDILARAKNGTGKTAAFVIPTLERINPKNPKTQALLLVPTRELALQTSQVCKTLGKHLGINVMVTTGGTGLKDDIL

RLGDTVHIIIATPGRILDLASKGIADLSECSIFVMDEADKLLSPEFTVVIEQLLSFHPKDRQVMLFSATFPMIVKHFKDK

HMRNPYEINLMDELTLRGITQYYAFVEEKQKVHCLNTLFSKLQINQSIIFCNSTNRVELLAKKITELGYSCFYSHAKMLQ

QNRNKVFHDFRAGVSRNLVCSDLLTRGIDIQAVNVVINFDFPKNAETYLHRIGRSGRFGHLGLAINLINWDDRFNLYKIE

QELGTEIQPIPPSIDKSLYVYDNPENIPRSMPPPAQARQSYSATNAASTSHNGQVQSQAQGQRRPQGQSNGFPQQFNNQG

RGGYRGRGRGQGTRGGGRGGHQPALPVGQTTQ

>CAA7264364.1 unnamed protein product [Agrocybe aegerita]

MSQATTSSSSTPANNDVSWKAGLRPPPKDIRPQTEDVTATKGLEFEDMHLRRELLMGIFEAGFEKPSPIQEEAIPIALTK

RDVLARAKNGTGKTAAFVIPSLQQIDISKPKIQALLLVPTRELALQTSQVCKILGKHMGLQVMVTTGGTTLKDDILRLSE

TVHVLVGTPGRILDLAGKGVADLSECPVFVMDEADKLLSPEFAPVMEQLLSFLPADRQVMLFSATFPMIVKDFKDKHMKT

PHEINLMDELTLRGVTQYYAYVEERQKVHCLNTLFSKLQINQSIIFCNSTNRVELLAKKVTELGYSCFYSHAKMLQSHRN

RVFHDFRNGVCRNLVCSDLLTRGIDIQAVNVVINFDFPKNSETYLHRIGRSGRFGHLGLAINLVTYEDRFNLYKIEQELG

TEIQPIPQNIDRGLYVAPSGGEESQVQRPHQQPVPPQQQQQQARQQQAAAAIQRNGPATQQVQQIQPQVAQQYAARQNGV

PQQQPPRIQPAYRGGVPVAR

>KAA6409064.1 ATP-dependent RNA helicase dhh1 [Lasallia pustulata]

MASSLADQLNDTKINDGSPDAGWKVKLKLPTKDARPQTEDVTATKGLEFEDFYIKRELMMGIFEAGFEKPSPIQEETIPV

ALTGRDILARAKNGTGKTAAFVIPTLERINPKSTKTQALILVPTRELALQTSQVCKTLGKHLDINVMVTTGGTGIKEDIL

RLNDPVHIIVGTPGRILDLASKGVADLTECPTFVMDEADKLLSPEFTPVIEQLLSFPPKDRQVMLFSATFPLIVKSFKDK

HMTNPYEINLMDELTLRGITQYYAFVEEKQKVHCLNTLFSKLQINQSIIFCNSTNRVELLAKKITELGYSCFYSHARMQQ

HNRNRVFHDFRNGVCRNLVCSDLLTRGIDIQAVNVVINFDFPKNAETYLHRIGRSGRFGHLGLAINLINWDDRFNLYRIE

QELGTEIQPIPQAIDKKLYVYDSPDTIPRPVSNSVPQRQDSGRAQHRGDGSQQISKVPSNTINGTGAISGRTAGAGRSQL

QQQPGSYQRSQRTQGQSYGSRRGPPKEQGDQGRQNGFPPQQAAKKPAIQAS

>RCH80432.1 DExD/H-box ATP-dependent RNA helicase dhh1, partial [Rhizopus stolonifer]

HGTEKWKQNLALPKKDTRPQTEDVTATKGNEFEDYFLKRELLMGIFEAGFERPSPIQEEAIPIALVNRDILARAKNGTGK

TAAFVIPTLEKINNKVSKIQALLLVPTRELALQTAQVCKKLGKHLNIQVMVTTGGTTLKDDIMRLSEPVHLVVGTPGRIL

DLASKGVADFSQANTFVMDEADKLLSPEFTPVIDQLISFFPKNRQIMLFSATFPIVVKTFKEKHLANPYEINLMDELTLR

GVTQYYAYVEEKQKVHCLNTLFSKLQINQSIIFCNSTNRVELLAKKITELGYSCFYSHAKMLQSHRNRVFHDFRNGVCRN

LVCSDLLTRGIDIQAVNVVINFDFPKNAETYLHRIGRSGRFGHFGLAINLITYEDRFNLYKIERELGTEIQPIPPVIDKQ

LYVAPNALEDTEVQRPNRQLALATRQQQQQREHQLEQQQYQQQRRNYGQTFLGQKEGYYQAGSRDQ

>XP_009549682.1 hypothetical protein HETIRDRAFT_67389 [Heterobasidion irregulare TC 32-1]

MSQQARPSSSSSPNDNWRAGLRPPPRDDRPQTEDVMATKGLEFEDMFLRRELLMGIFEAGFEKPSPIQEEAIPMALTKRD

ILARAKNGTGKTAAFVIPSLQQIDINKNKIQALLLVPTRELALQTSQVCKILGKHMGIQVMVTTGGTTLKDDILRLSETV

HVLVGTPGRILDLTGKNVADLSECPVFVMDEADKLLSPEFSPVMEQLLAYLPKDRQVMLFSATFPLIVKDFKDKHMNSPH

EINLMDELTLRGVTQYYAFVEERQKVHCLNTLFSKLQINQSIIFCNSTNRVELLAKKVTELGYSCFYSHAKMLQSHRNRV

FHDFRNGVCRNLVCSDLLTRGIDIQAVNVVINFDFPKNSETYLHRIGRSGRFGHLGLAINLVTYEDRFNLYKIEQELGTE

IQPIPHQIDKGLYVAPSAVEEPTGQKPSRSTQSEQQSQSQLQQPQSQLQPPRSQATNGTAQPAGGARPYPTGAPAGAYRG

QVPVAQR

>KAF3270986.1 DExD/H-box ATP-dependent RNA helicase dhh1 [Arthrobotrys oligospora]

MASSLSEKLESTTLSDADWRAQLTAPVKDLRPQTEDVTATKGLDFEELYIKRELLMGIFEAGFEKPSPIQEETIPVALTG

RDILARAKNGTGKTAAFVIPALERVNSKSPKTQALILVPTRELALQTSQVCKTLGKHLGINVMVTTGGTVLKDDIIRLNE

AVHVLVGTPGRILDLAGKGVADFSECPTFIMDEADKLLSPEFTPIIEQLLAYFPSDRQIMLFSATFPLVVKSFMDKHLKQ

PYEINLMDELTLRGVTQYYAFVEEKQKVHCLNTLFSKLQINQSIIFCNSTNRVELLAKKITELGYSCFYSHAKMLQNHRN

RVFHDFRNGVCRNLVCSDLLTRGIDIQAVNVVINFDFPKNAETYLHRIGRSGRFGHLGLAINLINWDDRFNLYKIEQELG

TEIQPIPAQIDKKLYVYDSPESIPRPPAAQSSIRSAPSPATTNPISQNTNRPDARSGPHLAQPQAVRHNKTRGNHSTGQS

FRGGRGSQRGYGHHQSYGRRNDNGHGTESILPSSHNA

>KZV82419.1 DEAD-domain-containing protein [Exidia glandulosa HHB12029]

MAQNQASSSNSTEWKAALNLPKKDLRPQTEDVTATKGTEFEDMFLRRELLMGIFEAGFERPSPIQEEAIPIALARRDVLA

RAKNGTGKTAAFVIPTLQQVDVTKPKIQACLLVPTRELALQTSQVCKILGKHMGVQVMVTTGGTTLKDDIMRLAEVVHVL

VGTPGRILDLAGKGVADLSECPVFVMDEADKLLSPEFAPVMEQLLAYLPPTRQVMLFSATFPIIVKDFKDKHMKNPHEIN

LMDELTLRGVTQYYAYVEERQKVHCLNTLFSKLQINQSIIFCNSTNRVELLARKVTELGYSCFFSHAKMLQAHRNRVFHD

FRNGVCRNLVCSDLLTRGIDIQAVNVVINFDFPKNSETYLHRIGRSGRFGHLGLAINLVTYEDRFNLFRVEQELGTEIQP

IPQTIDKSLYVAPAGAEEPTGAVKKGQNRNPQQPGSRQQQTPQQSRGQTPAVAGPPRPPQQQPTYQQQQILNGAQAQAQY

QQPVAR

>XP_014172819.1 ATP dependent RNA helicase [Grosmannia clavigera kw1407]

MADGLVEKLKSTTLNDDRKSDDWKSTLNIPTKDSRQQTEDVTNTKGLEFEEFGLKRDLLMGIFEAGFEKPSPIQEESIPV

ALTGRDILARAKNGTGKTAAFVIPALEKINPKVSKIQCLILVPTRELAMQTSQVCKSLGKHLNINVMVTTGGTGLRDDIV

RLQEAVHIVVGTPGRILDLASKNVADLSECPMFIMDEADKLLSAEFTPVIEQLLQFHPKDRQVMLFSATFPLSVKDFSDK

NMVSPYEINLMDELTLRGITQYYAFVEERQKVHCLNTLFSKLQINQSIIFCNSTNRVELLAKKITELGYSCFYSHAKMAQ

QARNRVFHDFRNGVCRNLVCSDLLTRGIDIQAVNVVINFDFPKNAETYLHRIGRSGRYGHLGLAINLINWDDRLNLYNIE

RDLGTEIQPIPATIDKSLYVYENPESIPRPIATLAKPASFPAASGSGSVGQLQTNSQSRASQAQPTLPQAQGDWQQGGFS

GSTQRGGYQARGQSSGSRGGRGRNGYHGGRGHYGGRGGRGGQGAPPAQQA

>THW78045.1 DEAD-domain-containing protein [Aureobasidium pullulans]

MPIPAPKLTRNHTLTRSPLSESNQADGNWRSGLKVPAKDGRVQTEDVTATKGLEFEDFYLKRELLMGIFESGFEKPSPIQ

EEAIPVALTGRDVLARAKNGTGKTAAFIIPTLERVNPKNSKIQALILVPTRELALQTSQVCKTLGKHLGVNVMVTTGGTG

LKDDIIRLNEAVHVLIGTPGRILDLASRGVADLSTAQTFVMDEADKLLSPEFTSTIDQLLSFHPPDRQVMLFSATFPVIV

KEFKDKHMKDPHEINLMDELTLRGITQYYAFVEEKQKVHCLNTLFSRLQINQSIIFCNSTTRVELLAKKVTELGYSCFYS

HARMLQQNRNRVFHDFRAGHCRNLVCSDLLTRGIDIQAVNVVINFDFPKNAETYLHRIGRSGRFGHLGLAINLINWEDRF

NLYRIEQELGTEIKPIPQHIDKGLYVYDTPENIPRPITSAPQLQQQPQERRQHHQQGYRGGRGGNYQGQQGQRRGPPRNQ

YPSQGGQGGGFQQQQAQYNQQAFPTQQAPQQAPSYPQGGFSQPSAFPTPQSFPQPQGQQPSR

>THX20364.1 DEAD-domain-containing protein [Aureobasidium pullulans]

MADQLASQLEAASLNESNQADGNWRSGLKVPAKDGRVQTEDVTATKGLEFEDFYLKRELLMGIFESGFEKPSPIQEEAIP

VALTGRDVLARAKNGTGKTAAFIIPTLERVNPKNSKIQALILVPTRELALQTSQVCKTLGKHLGVNVMVTTGGTGLKDDI

IRLNEAVHVLIGTPGRILDLASRGVADLSTAQTFVMDEADKLLSPEFTSTIDQLLSFHPPDRQVMLFSATFPVIVKEFKD

KHMKDPHEINLMDELTLRGITQYYAFVEEKQKVHCLNTLFSRLQINQSIIFCNSTTRVELLAKKVTELGYSCFYSHARML

QQNRNRVFHDFRAGHCRNLVCSDLLTRGIDIQAVNVVINFDFPKNAETYLHRIGRSGRFGHLGLAINLINWEDRFNLYRI

EQELGTEIKPIPQHIDKGLYVYDTPENIPRPITSAPQLQQQPQERRQHHQQGYRGGRGGNYQGQQGQRRGPPRNQYPSQG

GQGGGFQQQQAQYNQQAFPTQQAPQQAQQAPSYPQGGFSQPSAFPTPQSFPQPQGQQPSR

>KAF3081079.1 DExD/H-box ATP-dependent RNA helicase dhh1 [Arthrobotrys oligospora]

MASSLSEKLESTTLSDADWRAQLTAPVKDLRPQTEDVTATKGLDFEELYIKRELLMGIFEAGFEKPSPIQEETIPVALTG

RDILARAKNGTGKTAAFVIPALERVNSKSPKTQALILVPTRELALQTSQVCKTLGKHLGINVMVTTGGTVLKDDIIRLNE

AVHVLVGTPGRILDLAGKGVADFSECPTFIMDEADKLLSPEFTPIIEQLLAYFPSDRQIMLFSATFPLVVKSFMDKHLKQ

PYEINLMDELTLRGVTQYYAFVEEKQKVHCLNTLFSKLQINQSIIFCNSTNRVELLAKKITELGYSCFYSHAKMLQNHRN

RVFHDFRNGVCRNLVCSDLLTRGIDIQAVNVVINFDFPKNAETYLHRIGRSGRFGHLGLAINLINWDDRFNLYKIEQELG

TEIQPIPAQIDKKLYVYDSPESIPRPPAAQNSIRSAPSPATTNPISQNTNRPDARSGPHPAQPQAVRHNKTRGNHSTGQS

FRGGRGSQRGYGHHQSYGRRNDNGHGTESILPSSHSA

>XP_013268274.1 ATP-dependent RNA helicase dhh1 [Rhinocladiella mackenziei CBS 650.93]

MTDALAAQLNKASLNDGADDSNWKQALKIPTKDTRQQTEDVLATKGLEFEDFYIKRELMMGIFEAGFEKPSPIQEETIPV

ALTGRDILARAKNGTGKTAAFVIPTLERINPKNPKTQALILVPTRELALQTSQVCKTLGKHLGINVMVTTGGTGLKDDII

RLGETVHIIVGTPGRIADLAGKGVADLSECPIFVMDEADKLLSPEFTDVIEQLLSFHPKDRQVMLFSATFPMIVKSFKDK

HMRNPYEINLMDELTLRGITQYYAFVEEKQKVHCLNTLFSKLQINQSIIFCNSTNRVELLAKKITELGYSCFYSHAKMLQ

QNRNKVFHDFRAGVCRNLVCSDLLTRGIDIQAVNVVINFDFPKNAETYLHRIGRSGRFGHLGLAINLIGWDDRFNLYKIE

QELGTEIQPIPPSIDKSLYVYDSPENIPRSMPPPAQVAKPNPANAAANANAATANGQYQGRRPQNQSNGYGQQYNSGRGG

YRGRGRGQGARGRGGHPHQAAAPVSQTAQ

>SLM40172.1 atp-dependent rna helicase dhh1 [Lasallia pustulata]

MASSLADQLNDTKINDGSPDAGWKVKLKIPTKDARPQTEDVTATKGLEFEDFYIKRELMMGIFEAGFEKPSPIQEETIPV

ALTGRDILARAKNGTGKTAAFVIPTLERINPKSTKTQALILVPTRELALQTSQVCKTLGKHLDINVMVTTGGTGIKEDIL

RLNDPVHIIVGTPGRILDLASKGVADLTECPTFVMDEADKLLSPEFTPVIEQLLSFPPKDRQVMLFSATFPLIVKSFKDK

HMTNPYEINLMDELTLRGITQYYAFVEEKQKVHCLNTLFSKLQINQSIIFCNSTNRVELLAKKITELGYSCFYSHARMLQ

HNRNRVFHDFRNGVCRNLVCSDLLTRGIDIQAVNVVINFDFPKNAETYLHRIGRSGRFGHLGLAINLINWDDRFNLYKIE

QELGTEIQPIPQAIDKKLYVYDSPDTIPRPVSNSVPQRQDSGRAQHRGDGSQQISKVPSNTINGTGAISGRTAGAGRSQL

QQQPGSYQRSQRTQGQSYGSRRGPPKEQGDQGRQNGFPPQQAAKKPAIQAS

>KAF2099992.1 ATP-dependent RNA helicase DHH1 [Rhizodiscina lignyota]

MATDITNQLAASKLTYSDTNAKDGNDWKQGLKQPSKDTRVQTDDVLGTKGLEFEDFYIKRELMMGIFEAGFEKPSPIQEE

AIPVALTGRDILARAKNGTGKTAAYVIPTLERINPKSTKTQALILVPTRELALQTSQVCKTLGKHLGLNVMVSTGGTGLK

DDIIRLSETVHIIVGTPGRILDLASKQVADLSGCQTFVMDEADKLLSPEFTPSVEQLLAFHPKDRQVMLFSATFPISVKS

FKDQHMKTPYEINLMDELTLRGITQYYAFVEEKQKVHCLNTLFNKLQINQSIIFCNSTNRVELLAKKITELGYSCFYSHA

RMMQHARNRVFHDFRNGVCRNLVCSDLLTRGIDIQAVNVVINFDFPKNAETYLHRIGRSGRFGHLGLAINLINWEDRFNL

YRIEQELGTEIQPIPQVIEKKLYVYDTPETIPRPINNPSQEQQNQDASITRQLSGSRGGFAQRGQRGGGQYQQRRGPPRD

PNQHTQGQRNGQNGFAGQGRQPKPAPIQQ

>KEQ62464.1 DEAD-domain-containing protein [Aureobasidium melanogenum CBS 110374]

MADQLANQLEAASLNESSQADGNWRSGLKVPAKDGRVQTEDVTATKGLEFEDFYLKRELLMGIFESGFEKPSPIQEEAIP

VALTGRDVLARAKNGTGKTAAFIIPTLERVNPKNSKIQALILVPTRELALQTSQVCKTLGKHLGVNVMVTTGGTGLKDDI

IRLNEAVHILIGTPGRILDLASRGVADLSTAQTFVMDEADKLLSPEFTSTIDQLLSFHPPDRQVMLFSATFPVIVKEFKD

KHMKDPHEINLMDELTLRGITQYYAFVEEKQKVHCLNTLFSRLQINQSIIFCNSTTRVELLAKKVTELGYSCFYSHARML

QHNRNRVFHDFRAGHCRNLVCSDLLTRGIDIQAVNVVINFDFPKNAETYLHRIGRSGRFGHLGLAINLINWEDRFNLYRI

EQELGTEIKPIPQHIDKGLYVYDTPENIPRPITSAPQLQQQPQERRQHHQQGYRGGRGGNYQGQQGQRRGPPRNQYPSQG

GQGGQGGYQQQQAQYNQQAFGAPPQQQQQQSSYPQGGFAQPNAFPTPQSFPQPQGQQPSR

>XP_011118775.1 hypothetical protein AOL_s00007g573 [Arthrobotrys oligospora ATCC 24927]

MASSLSEKLESTTLSDADWRAQLTAPVKDLRPQTEDVTATKGLDFEELYIKRELLMGIFEAGFEKPSPIQEETIPVALTG

RDILARAKNGTGKTAAFVIPALERVNSKSPKTQALILVPTRELALQTSQVCKTLGKHLGINVMVTTGGTVLKDDIIRLNE

AVHVLVGTPGRILDLAGKGVADFSECPTFIMDEADKLLSPEFTPIIEQLLAYFPSDRQIMLFSATFPLVVKSFMDKHLKQ

PYEINLMDELTLRGVTQYYAFVEEKQKVHCLNTLFSKLQINQSIIFCNSTNRVELLAKKITELGYSCFYSHAKMLQNHRN

RVFHDFRNGVCRNLVCSDLLTRGIDIQAVNVVINFDFPKNAETYLHRIGRSGRFGHLGLAINLINWDDRFNLYKIEQELG

TEIQPIPAQIDKKLYVYDSPESIPRPPAAQSSIRSAPSPATTNPISQNTNRPDARSGPHPAQPQAVRHNKTRGNHSTGQS

FRGGRGSQRGYGHHQSYGRRNDNGHGTESILPSSHNA

>CCF52042.1 probable ATP-dependent RNA helicase DHH1 [Ustilago hordei]

MSSAGPASGQSEAAWKQQIASKIAKDERPQTEDVLNTKGNEFEDYFLKRELLMGIFEAGFERPSPIQEEAIPIALTGRDI

LARAKNGTGKTAAYVIPSLEKLNTKKNKIQAVLLVPTRELALQTSQVAKTLGKHLGVEVMVTTGGTTLRDDILRLGQTVH

MLVGTPGRILDLAGKGVADLSQCHTFVMDEADKLLSPEFTPVMEQLLSFLPKERQVMLFSATFPLIVKDFKDRNMVKPYE

INLMDELTLRGVTQYYAFVEERQKVHCLNTLFSKLQINQSIIFCNSTNRVELLAKKITELGYSCFYSHAKMLQSHRNRVF

HDFRNGACRNLVCSDLLTRGIDIQAVNVVINFDFPKNAETYLHRIGRSGRFGHLGLAINLITYEDRFNLYRIEQELGTEI

QPIPSNIDKRLYVAPSLIQEGEKGGQNGNRQEGRPVIPPGQQAMHASAIPQNQNFSNSVPHRHRGGGSRGGGGGTGRGQG

APPAQ

>THH09892.1 hypothetical protein EW146_g8546 [Bondarzewia mesenterica]

MSQQARPSSSSSRNDNWRAGLRPPPRDDRPQTEDIKATKGLEFEDMFLRRELLMGIFEAGFEKPSPIQEEAIPIALTKRD

ILARAKNGTGKTAAFVIPALQQIDINKNKIQALLLVPTRELALQTSQVCKILGKHMGVQVMVTTGGTTLKDDILRLSEMV

HVLVGTPGRILDLTGKGVADLSECPVFVMDEADKLLSPEFSPVMEQLLTYLPKDRQVMLFSATFPLIVKDFKDKHMNTPY

EINLMDELTLRGVTQYYAFVEERQKVHCLNTLFSKLQINQSIIFCNSTNRVELLAKKVTELGYSCFYSHAKMLQSHRNRV

FHDFRNGVCRNLVCSDLLTRGIDIQAVNVVINFDFPKNSETYLHRIGRSGRFGHLGLAINLVTYEDRFNLYKIEQELGTE

IQPIPQQIDKGLYVAPSAAADEPAVQKPSRTQQTEQQTQRQQAAAQVQAQTQARQAALQQQQQQQGQVVYSSGAQAVRPN

GTAQAQAGAGARTYPGAGAGAGGATGAYRGQVPVAQR

>THV86802.1 DEAD-domain-containing protein [Aureobasidium pullulans]

MPIPVPKPTWNHTLTRSPLSESNQADGNWRSGLKVPAKDGRVQTEDVTATKGLEFEDFYLKRELLMGIFESGFEKPSPIQ

EEAIPVALTGRDVLARAKNGTGKTAAFIIPTLERVNPKNSKIQALILVPTRELALQTSQVCKTLGKHLGVNVMVTTGGTG

LKDDIIRLNEAVHVLIGTPGRILDLASRGVADLSTAQTFVMDEADKLLSPEFTSTIDQLLSFHPPDRQVMLFSATFPVIV

KEFKDKHMKDPHEINLMDELTLRGITQYYAFVEEKQKVHCLNTLFSRLQINQSIIFCNSTTRVELLAKKVTELGYSCFYS

HARMLQQNRNRVFHDFRAGHCRNLVCSDLLTRGIDIQAVNVVINFDFPKNAETYLHRIGRSGRFGHLGLAINLINWEDRF

NLYRIEQELGTEIKPIPQHIDKGLYVYDTPENIPRPITSAPQLQQQPQERRQHHQQGYRGGRGGNYQGQQGQRRGPPRNQ

YPSQGGQGGGFQQQQAQYNQQAFPTQQAPQQAPSYPQGGFSQPSAFPTPQSFPQPQGQQPSR

>THW26377.1 DEAD-domain-containing protein [Aureobasidium pullulans]

MADQLASQLEAASLNESNQADGNWRSGLKVPAKDGRVQTEDVTATKGLEFEDFYLKRELLMGIFESGFEKPSPIQEEAIP

VALTGRDVLARAKNGTGKTAAFIIPTLERVNPKNSKIQALILVPTRELALQTSQVCKTLGKHLGVNVMVTTGGTGLKDDI

IRLNEAVHVLIGTPGRILDLASRGVADLSTAQTFVMDEADKLLSPEFTSTIDQLLSFHPPDRQVMLFSATFPVIVKEFKD

KHMKDPHEINLMDELTLRGITQYYAFVEEKQKVHCLNTLFSRLQINQSIIFCNSTTRVELLAKKVTELGYSCFYSHARML

QQNRNRVFHDFRAGHCRNLVCSDLLTRGIDIQAVNVVINFDFPKNAETYLHRIGRSGRFGHLGLAINLINWEDRFNLYRI

EQELGTEIKPIPQHIDKGLYVYDTPENIPRPITSAPQLQQQPQERRQHHQQGYRGGRGGNYQGQQGQRRGPPRNQYPSQG

GQGGGFQQQQAQYNQQAFPTQQAPQQAPQQAPQQAPQQAPSYPQGGFSQPSAFPTPQSFPQPQGQQPSR

>PWN47299.1 putative ATP-dependent RNA helicase DHH1 [Violaceomyces palustris]

MSASGPAPGQSEASWKAEILSKVRKDDRPQTEDVLNTKGNDFEDYFLKRELLMGIFEAGFERPSPIQEEAIPIALTGRDI

LARAKNGTGKTAAYVIPSLERLNTKKNKIQAVLLVPTRELALQTSQVAKTLGKHMGVEVMVTTGGTTLRDDILRLGQTVH

LLVGTPGRILDLAGKGVADLSQCNTFVMDEADKLLSPEFTPVMEQLLSFLPKERQVMLFSATFPMIVKDFKDKNMVKPYE

INLMDELTLRGVTQYYAFVEERQKVHCLNTLFSKLQINQSIIFCNSTNRVELLAKKITELGYSCFYSHAKMLQSHRNRVF

HDFRNGACRNLVCSDLLTRGIDIQAVNVVINFDFPKNAETYLHRIGRSGRFGHLGLAINLITYEDRFNLYRIEQELGTEI

QPIPSNIDKRLYVAPALIQEGEEKAKAAAHHQNGGARQDGRPVIPPGQAAMHATTIPQSQDFTQSVPHRHRGGGHGGQQQ

AA

>THW81582.1 DEAD-domain-containing protein [Aureobasidium pullulans]

MADQLASQLEAASLNESNQADGNWRSGLKVPAKDGRVQTEDVTATKGLEFEDFYLKRELLMGIFESGFEKPSPIQEEAIP

VALTGRDVLARAKNGTGKTAAFIIPTLERVNPKNSKIQALILVPTRELALQTSQVCKTLGKHLGVNVMVTTGGTGLKDDI

IRLNEAVHVLIGTPGRILDLASRGVADLSTAQTFVMDEADKLLSPEFTSTIDQLLSFHPPDRQVMLFSATFPVIVKEFKD

KHMKDPHEINLMDELTLRGITQYYAFVEEKQKVHCLNTLFSRLQINQSIIFCNSTTRVELLAKKVTELGYSCFYSHARML

QQNRNRVFHDFRAGHCRNLVCSDLLTRGIDIQAVNVVINFDFPKNAETYLHRIGRSGRFGHLGLAINLINWEDRFNLYRI

EQELGTEIKPIPQHIDKGLYVYDTPENIPRPITSAPQLQQQPQERRQHHQQGYRGGRGGNYQGQQGQRRGPPRNQYPSQG

GQGGGFQQQQAQYNQQAFPTQQAPQQAPQQAPSYPQGGFSQPSAFPTPQSFPQPQGQQPSR

>THY34846.1 DEAD-domain-containing protein [Aureobasidium pullulans]

MRSESNQADGNWRSGLKVPAKDGRVQTEDVTATKGLEFEDFYLKRELLMGIFESGFEKPSPIQEEAIPVALTGRDVLARA

KNGTGKTAAFIIPTLERVNPKNSKIQALILVPTRELALQTSQVCKTLGKHLGVNVMVTTGGTGLKDDIIRLNEAVHVLIG

TPGRILDLASRGVADLSTAQTFVMDEADKLLSPEFTSTIDQLLSFHPPDRQVMLFSATFPVIVKEFKDKHMKDPHEINLM

DELTLRGITQYYAFVEEKQKVHCLNTLFSRLQINQSIIFCNSTTRVELLAKKVTELGYSCFYSHARMLQQNRNRVFHDFR

AGHCRNLVCSDLLTRGIDIQAVNVVINFDFPKNAETYLHRIGRSGRFGHLGLAINLINWEDRFNLYRIEQELGTEIKPIP

QHIDKGLYVYDTPENIPRPITSAPQLQQQPQERRQHHQQGYRGGRGGNYQGQQGQRRGPPRNQYPSQGGQGGGFQQQQAQ

YNQQAFPTQQAPQQAPQQAPSYPQGGFSQPSAFPTPQSFPQPQGQQPSR

>XP_016614033.1 ATP-dependent RNA helicase dhh1 [Cladophialophora bantiana CBS 173.52]

MTDALAAQLNKASLTDGADEANWKDSLNLPAKDTRQQTEDVTATKGLEFEDFYIKRELMMGIFEAGFEKPSPIQEETIPV

ALTGRDILARAKNGTGKTAAFVIPTLERINPKNPKTQALILVPTRELALQTSQVCKTLGKHLGINVMVTTGGTGLKDDII

RLGETVHIIVGTPGRILDLASKGVADLSECPIFVMDEADKLLSPEFTVVIEQLLSFHPKDRQVMLFSATFPMIVKTFKDK

HMRNPYEINLMDELTLRGITQYYAFVEEKQKVHCLNTLFSKLQINQSIIFCNSTNRVELLAKKITELGYSCFYSHAKMLQ

QNRNKVFHDFRAGVCRNLVCSDLLTRGIDIQAVNVVINFDFPKNAETYLHRIGRSGRFGHRGLAINLINWDDRFNLYKIE

QELGTEIQPIPPSIDKSLYVYDNPETIPRAMPPPAQIPKITASNATPTANINATNANGQYQQARRPQNQSNGYGQQYNNQ

GRGGYRGRGRGQGARGRGGHPQQPTAAPVGQIAH

>KAF3161163.1 DExD/H-box ATP-dependent RNA helicase dhh1 [Arthrobotrys oligospora]

MASSLSEKLESTTLSDADWRAQLTAPVKDLRPQTEDVTATKGLDFEELYIKRELLMGIFEAGFEKPSPIQEETIPVALTG

RDILARAKNGTGKTAAFVIPALERVNSKSPKTQALILVPTRELALQTSQVCKTLGKHLGINVMVTTGGTVLKDDIIRLNE

AVHVLVGTPGRILDLAGKGVADFSECPTFIMDEADKLLSPEFTPIIEQLLAYFPSDRQIMLFSATFPLVVKSFMDKHLKQ

PYEINLMDELTLRGVTQYYAFVEEKQKVHCLNTLFSKLQINQSIIFCNSTNRVELLAKKITELGYSCFYSHAKMLQNHRN

RVFHDFRNGVCRNLVCSDLLTRGIDIQAVNVVINFDFPKNAETYLHRIGRSGRFGHLGLAINLINWDDRFNLYKIEQELG

TEIQPIPAQIDKKLYVYDSPESIPRPPAAQNSIRSAPSPATTNPISQNTNRPDARSGPHPAQPQAVRHNKTRGNHSTGQS

FRGGRGSQRGYGHHQSYGRRNDNGHGTESILPSSHNA

>TIA34670.1 DEAD-domain-containing protein [Aureobasidium pullulans]

MADQLASQLEAASLNESNQADGNWRSGLKVPAKDGRVQTEDVTATKGLEFEDFYLKRELLMGIFESGFEKPSPIQEEAIP

VALTGRDVLARAKNGTGKTAAFIIPTLERVNPKNSKIQALILVPTRELALQTSQVCKTLGKHLGVNVMVTTGGTGLKDDI

IRLNEAVHVLIGTPGRILDLASRGVADLSTAQTFVMDEADKLLSPEFTSTIDQLLSFHPPDRQVMLFSATFPVIVKEFKD

KHMKDPHEINLMDELTLRGITQYYAFVEEKQKVHCLNTLFSRLQINQSIIFCNSTTRVELLAKKVTELGYSCFYSHARML

QQNRNRVFHDFRAGHCRNLVCSDLLTRGIDIQAVNVVINFDFPKNAETYLHRIGRSGRFGHLGLAINLINWEDRFNLYRI

EQELGTEIKPIPQHIDKGLYVYDTPENIPRPITSAPQLQQQPQERRQHHQQGYRGGRGGNYQGQQGQRRGPPRNQYPSQG

GQGGGSQQQQAQYNQQAFPTQQAPQQAPQQAPQQAPSYPQGGFSQPSAFPTPQSFPQPQGQQPSR

>PWZ00313.1 putative ATP-dependent RNA helicase DHH1 [Testicularia cyperi]

MSASGPAPGQTEAQWKQQITSKVGKDERPQTEDVLNTKGNEFEDYFLKRELLMGIFEAGFERPSPIQEEAIPIALTGRDI

LARAKNGTGKTAAYVIPSLEKLNTKKNKIQAVLLVPTRELALQTSQVAKTLGKHLGVEVMVTTGGTTLRDDILRLGQTVH

MLVGTPGRILDLAGKGVADLSQCTTFVMDEADKLLSPEFTPVMEQLLSFLPKERQVMLFSATFPLIVKDFKDKNMVKPYE

INLMDELTLRGVTQYYAFVEERQKVHCLNTLFSKLQINQSIIFCNSTNRVELLAKKITELGYSCFYSHAKMLQSHRNRVF

HDFRNGACRNLVCSDLLTRGIDIQAVNVVINFDFPKNAETYLHRIGRSGRFGHLGLAINLITYEDRFNLYRIEQELGTEI

QPIPSNIDKRLYVAPSLIQESESRNGGQGNGQNGNRQEGRPVIPPGQQAMHAAAIPQNQNFSNSVPHRHHRGGGGGGGGR

GGGHHQQQQQQQQQQQHYQQGAPAH

>TIA70056.1 DEAD-domain-containing protein [Aureobasidium pullulans]

MPIPAPKLTRNHMLTKSPLSESNQADSNWRSGLKVPAKDGRVQTEDVTATKGLEFEDFYLKRELLMGIFESGFEKPSPIQ

EEAIPVALTGRDVLARAKNGTGKTAAFIIPTLERVNPKNSKIQALILVPTRELALQTSQVCKTLGKHLGVNVMVTTGGTG

LKDDIIRLNEAVHVLIGTPGRILDLASRGVADLSTAQTFVMDEADKLLSPEFTSTIDQLLSFHPPDRQVMLFSATFPVIV

KEFKDKHMKDPHEINLMDELTLRGITQYYAFVEEKQKVHCLNTLFSRLQINQSIIFCNSTTRVELLAKKVTELGYSCFYS

HARMLQQNRNRVFHDFRAGHCRNLVCSDLLTRGIDIQAVNVVINFDFPKNAETYLHRIGRSGRFGHLGLAINLINWEDRF

NLYRIEQELGTEIKPIPQHIDKGLYVYDTPENIPRPITSAPQLQQQPQERRQHHQQGYRGGRGGNYQGQQGQRRGPPRNQ

YPSQGGQGGGFQQQQAQYNQQAFPTQQAPQQAPQQAPSYPQGGFSQPSAFPTPQSFPQPQGQQPSR

>XP_013423804.1 DEAD-domain-containing protein [Aureobasidium namibiae CBS 147.97]

MADQLANQLEAASLNESNQADGNWRSGLKVPAKDGRVQTEDVTATKGLEFEDFYLKRELLMGIFESGFEKPSPIQEEAIP

VALTGRDVLARAKNGTGKTAAFIIPTLERVNPKNSKIQALILVPTRELALQTSQVCKTLGKHLGVNVMVTTGGTGLKDDI

IRLNEAVHVLIGTPGRILDLASRGVADLSTAQTFVMDEADKLLSPEFTSTIDQLLSFHPPDRQVMLFSATFPVIVKEFKD

KHMKDPHEINLMDELTLRGITQYYAFVEEKQKVHCLNTLFSRLQINQSIIFCNSTTRVELLAKKVTELGYSCFYSHARML

QHNRNRVFHDFRAGHCRNLVCSDLLTRGIDIQAVNVVINFDFPKNAETYLHRIGRSGRFGHLGLAINLINWEDRFNLYRI

EQELGTEIKPIPQHIDKGLYVYDTPENIPRPINSAPQLQQQPQERRQHHQQGYRGGRGGNYEGQQGHQGQRRGPPRNQYP

SQGGQGGGFQQQQAQYNQQAFGAPSQQQQAPSYPQGGFSQPNAFPTPQSFPQPQGQ

>XP_007741840.1 ATP-dependent RNA helicase DHH1 [Cladophialophora psammophila CBS 110553]

MTDALAAQLNKASLTDGADEANWKDSLNLPAKDTRQQTEDVTATKGLEFEDFYIKRELMMGIFEAGFEKPSPIQEETIPV

ALTGRDILARAKNGTGKTAAFVIPTLERINPKNPKTQALILVPTRELALQTSQVCKTLGKHLGINVMVTTGGTGLKDDII

RLGETVHIIVGTPGRILDLASKGVADLSECPIFVMDEADKLLSPEFTVVIEQLLSFQPKDRQVMLFSATFPMIVKAFKDK

HMRNPYEINLMDELTLRGITQYYAFVEEKQKVHCLNTLFSKLQINQSIIFCNSTNRVELLAKKITELGYSCFYSHAKMLQ

QNRNKVFHDFRAGVCRNLVCSDLLTRGIDIQAVNVVINFDFPKNAETYLHRIGRSGRFGHRGLAINLINWDDRFNLYKIE

QELGTEIQPIPPSIDKSLYVYDNPETIPRAMPPPAQTTKITASNATPTANTNATNANGQYQQARRPQNQSNGYGQQYNNQ

GRGGYRGRGRGQGSRGRGGHPQQPTAAPVGQIAH

>XP_018230037.1 ATP-dependent RNA helicase dhh1 [Pneumocystis jirovecii RU7]

MSSLRGPDSVVLDSSKTYDDWKKTLNLSTKDTRPQTEDVTKTKGNEFEDFYLKRELLMGIFEAGFERPSPIQEESIPIAL

TGRDILARAKNGTGKTAAFIIPALEKLNSKKPKTQALILVPTRELALQTSHVCKTLGKHMGINVMVTTGGTSLQQDIIRL

HDPVHVIVGTPGRILDLAGKGVAEFSECFTFIMDEADKLLSLEFTPVIEQLLAYFPKDRQIMLYSATFPLVVKSFMDKHL

SKPYEINLMDELTLRGVTQYYAFVEEKQKVHCLNTLFSKLQINQSIIFCNSTNRVELLAKKITELGYSCFYSHAKMLQNH

RNRVFHDFRNGVCRNLVCSDLLTRGIDIQAVNVVINFDFPKNSETYLHRIGRSGRFGHLGLAINLINWEDRFNLYKIEQE

LATEIQPIPSIIDKRLYVSPSALDEPVNNRPRPKQRSVAPQSNGNANSQLNNEQSYIRQHPLRGRGGNRARSHGRRYSDR

VNGHTVLSNDNITRD

>RMZ89069.1 hypothetical protein DV736_g3701, partial [Chaetothyriales sp. CBS 134916]

MASQLADQLGKTTLTDPSPDPDWKKNLKARPKDEREQTEDVTATKGLDFEDFYIKRELMMGIFEAGFEKPSPIQEETIPV

ALTGRDILARAKNGTGKTAAFVIPTLEKVNPKNPKIQALLLVPTRELALQTSQVCKTLGKHLGLEVMVTTGGTGLKDDII

RLQSTVHILVGTPGRILDLTSKGVADLSECSIFVMDEADKLLSPEFTVVIEQLLNFHPKDRQVMLFSATFPLVVKDFKDK

HMRDPYEINLMDELTLRGITQYYAFVEEKQKVHCLNTLFSKLQINQSIIFCNSTNRVELLAKKITELGYSCFYSHARMMQ

QHRNKVFHDFRAGVSRNLVCSDLLTRGIDIQAVNVVINFDFPKNAETYLHRIGRSGRFGHLGLAINLITWEDRLNLYRIE

QELGTEIQPIPQSIDKSLYVYENPDNIPRPLPTAQPTTLPGGQNDPARKPNGYGQHVRGHFNRGVGGPRGRGRGGGRGGR

GGGQFVQQFQNGVPAQ

>ORZ34332.1 P-loop containing nucleoside triphosphate hydrolase protein [Catenaria anguillulae PL171]

MSRSSQSTRSSKINNGDSAGPASSSSRRDDTQLNGALANMSLQDTSESAGAAADDMTSPTSEADWKRSLHRPKKDNRPQT

EDVLATKGNEFEDYYLKRELLMGIFEAGFEKPSPIQEEAIPLALAGRDILARAKNGTGKTAAFVIPTLEKVNTAKAKIQA

VILVPTRELALQTSQVCKTLGKHLGIQVMVSTGGTLLKEDIMRLQDTVHILVGTPGRLLDLAGKGVAQLDECTTFVMDEA

DKLLSTEFEPVIESLLGFLPKDRQLMLFSATFPITVKTFKDKWLHDPYEINLMDDLTLKGVTQYYAFVEERQKVHCLNTL

FSKLQINQSIIFCNSTNRVELLAKKITELGYSCFFIHARMPQAHRNRVFHDFRAGVCRNLVCSDLLTRGIDIQAVNVVIN

FDFPKNTETYLHRIGRSGRYGHLGVAINLLTYEDRFNLYRIETELGTEISPIPSTIDKSLYVAPDMLDEEPQKPEKDPVQ

QPAMPPAHHSGGYRTYDNRDRDGGGRRYHGANAAGGGGGSGGARDGPKSPTSGTYHAPRREVQQPRRDA

>XP_029759559.1 DEAD-domain-containing protein [Aureobasidium pullulans EXF-150]

MADQLASQLEAASLNESNQADGNWRSGLKVPAKDGRVQTEDVTATKGLEFEDFYLKRELLMGIFESGFEKPSPIQEEAIP

VALTGRDVLARAKNGTGKTAAFIIPTLERVNPKNSKIQALILVPTRELALQTSQVCKTLGKHLGVNVMVTTGGTGLKDDI

IRLNEAVHVLIGTPGRILDLASRGVADLSTAQTFVMDEADKLLSPEFTSTIDQLLSFHPPDRQVMLFSATFPVIVKEFKD

KHMKDPHEINLMDELTLRGITQYYAFVEEKQKVHCLNTLFSRLQINQSIIFCNSTTRVELLAKKVTELGYSCFYSHARML

QQNRNRVFHDFRAGHCRNLVCSDLLTRGIDIQAVNVVINFDFPKNAETYLHRIGRSGRFGHLGLAINLINWDDRFNLYRI

EQELGTEIKPIPQHIDKGLYVYDTPENIPRPITSAPQLQQQPQERRQHHQQGYRGGRGGNYQGQQGQRRGPPRNQYPSQG

GQGGGFQQQQAQYNQQAFPTQQAPQQAPSYPQGGFSQPSAFPTPQSFPQPQGQQPSR

>THX37585.1 DEAD-domain-containing protein [Aureobasidium pullulans]

MYDGNWRSGLKVPAKDGRVQTEDVTATKGLEFEDFYLKRELLMGIFESGFEKPSPIQEEAIPVALTGRDVLARAKNGTGK

TAAFIIPTLERVNPKNSKIQALILVPTRELALQTSQVCKTLGKHLGVNVMVTTGGTGLKDDIIRLNEAVHVLIGTPGRIL

DLASRGVADLSTAQTFVMDEADKLLSPEFTSTIDQLLSFHPPDRQVMLFSATFPVIVKEFKDKHMKDPHEINLMDELTLR

GITQYYAFVEEKQKVHCLNTLFSRLQINQSIIFCNSTTRVELLAKKVTELGYSCFYSHARMLQQNRNRVFHDFRAGHCRN

LVCSDLLTRGIDIQAVNVVINFDFPKNAETYLHRIGRSGRFGHLGLAINLINWEDRFNLYRIEQELGTEIKPIPQHIDKG

LYVYDTPENIPRPITSAPQLQQQPQERRQHHQQGYRGGRGGNYQGQQGQRRGPPRNQYPSQGGQGGGFQQQQAQYNQQAF

PTQQAPQQAPSYPQGGFSQPSAFPTPQSFPQPQGQQPSR

>PIA19476.1 DEAD-domain-containing protein [Coemansia reversa NRRL 1564]

MAEAHLPNNSDDWKNALSLPAKDTRPQTEDVMATKGNEFEDYYLKRELLMGIFEAGFERPSPIQEESIPIAVAGRDILAR

AKNGTGKTAAFVIPALEKVNTAVANIQCLILVPTRELALQTSQVCKMLGKHMGVNVMVTTGGTTLKDDIIRLSETVHILV

GTPGRVLDLAGKGVADFSQASAFIMDEADKLLSPEFTPVIEQLLSFFPPSRQILLYSATFPLVVKGFKDKHMNKPYEINL

MEELTLRGVTQYYAFVEERQKVHCLNTLFSKLQINQSIIFCNSTNRVELLAKKITDLGYSCFYSHARMIQSHRNRVFHDF

RNGACRNLVCSDLLTRGIDIQAVNVVINFDFPKNAETYLHRIGRSGRFGHLGLAINLITSEDRFNLYKIEQELGTEIQPI

PQVIDKRLYVAPSAMDEPPQQRQQPTGTHGPAPPPGLIQGSVSRGHLQ

>XP_001248331.1 ATP-dependent RNA helicase DHH1 [Coccidioides immitis RS]

MAEALASQLNKAKLGDNGAETKWKEQLKLPPKDTRTQTEDVTATKGLEFEDFYIKRELMMGIFEAGFEKPSPIQEETIPV

ALTGRDILARAKNGTGKTAAFVIPTLERTNPKISKTQALILVPTRELALQTSQVCKTLGKHLGINVMVTTGGTGLQDDII

RLSDTVHIIVGTPGRILDLASKGVADLSECTTFVMDEADKLLSPEFTPVIEQLLSFHPKDRQVMLFSATFPMIVKSFKDK

HMRNPYEINLMDELTLRGITQYYAFVEERQKVHCLNTLFSKLQINQSIIFCNSTNRVELLAKKITELGYSCFYSHARMLQ

QNRNRVFHDFRNGVCRNLVCSDLLTRGIDIQAVNVVINFDFPKNAETYLHRIGRSGRFGHLGLAINLINWDDRYNLYKIE

QELGTEIQPIPPSIDKKLYVYDTPETIPRPIANASTERNPPAQLAQSSDNQNHRQAHHISGGHGQQTANRGHSLRGSYRG

GRAQGHRGGHPENNRTNPMSSRSNMPTSTTAS

>TPX25158.1 DExD/H-box ATP-dependent RNA helicase dhh1 [Coccidioides immitis]

MAEALASQLNKAKLGDNGAETKWKEQLKLPPKDTRTQTEDVTATKGLEFEDFYIKRELMMGIFEAGFEKPSPIQEETIPV

ALTGRDILARAKNGTGKTAAFVIPTLERTNPKISKTQALILVPTRELALQTSQVCKTLGKHLGINVMVTTGGTGLQDDII

RLSDTVHIIVGTPGRILDLASKGVADLSECTTFVMDEADKLLSPEFTPVIEQLLSFHPKDRQVMLFSATFPMIVKSFKDK

HMRNPYEINLMDELTLRGITQYYAFVEERQKVHCLNTLFSKLQINQSIIFCNSTNRVELLAKKITELGYSCFYSHARMLQ

QNRNRVFHDFRNGVCRNLVCSDLLTRGIDIQAVNVVINFDFPKNAETYLHRIGRSGRFGHLGLAINLINWDDRYNLYKIE

QELGTEIQPIPPSIDKKLYVYDTPETIPRPIANASTERNPPAQLAQSSDNQNHRQAHHISRGHGQQTANRGHSLRGSYRG

SRAQGHRGGHPENNRTNPMSSRSNMPTSTTAS

>THZ58605.1 DEAD-domain-containing protein [Aureobasidium pullulans]

MYDGNWRSGLKVPAKDGRVQTEDVTATKGLEFEDFYLKRELLMGIFESGFEKPSPIQEEAIPVALTGRDVLARAKNGTGK

TAAFIIPTLERVNPKNSKIQALILVPTRELALQTSQVCKTLGKHLGVNVMVTTGGTGLKDDIIRLNEAVHVLIGTPGRIL

DLASRGVADLSTAQTFVMDEADKLLSPEFTSTIDQLLSFHPPDRQVMLFSATFPVIVKEFKDKHMKDPHEINLMDELTLR

GITQYYAFVEEKQKVHCLNTLFSRLQINQSIIFCNSTTRVELLAKKVTELGYSCFYSHARMLQQNRNRVFHDFRAGHCRN

LVCSDLLTRGIDIQAVNVVINFDFPKNAETYLHRIGRSGRFGHLGLAINLINWEDRFNLYRIEQELGTEIKPIPQHIDKG

LYVYDTPENIPRPITSAPQLQQQPQERRQHHQQGYRGGRGGNYQGQQGQRRGPPRNQYPSQGGQGGGFQQQQAQYNQQAF

PTQQAPQQAPQQAPSYPQGGFSQPSAFPTPQSFPQPQGQQPSR

>KAF3203569.1 DExD/H-box ATP-dependent RNA helicase dhh1 [Arthrobotrys oligospora]

MASSLSEKLESVTLSDADWRAQLTAPVKDLRPQTEDVTATKGLDFEELYIKRELLMGIFEAGFEKPSPIQEETIPVALTG

RDILARAKNGTGKTAAFVIPALERVNSKSPKTQALILVPTRELALQTSQVCKTLGKHLGINVMVTTGGTVLKDDIIRLNE

AVHVLVGTPGRILDLAGKGVADFSECPTFIMDEADKLLSPEFTPIIEQLLAYFPSDRQIMLFSATFPLVVKSFMDKHLKQ

PYEINLMDELTLRGVTQYYAFVEEKQKVHCLNTLFSKLQINQSIIFCNSTNRVELLAKKITELGYSCFYSHAKMLQNHRN

RVFHDFRNGVCRNLVCSDLLTRGIDIQAVNVVINFDFPKNAETYLHRIGRSGRFGHLGLAINLINWDDRFNLYKIEQELG

TEIQPIPAQIDKKLYVYDSPESIPRPPAAQNSIRSAPSPATTNPISQNTNRSDARSGTHPAQPQAVRHNKTRGNHSTGQS

FRGGRGSQRGYGHHQSYGRRNDNGHGTEGILPSSHNA

>XP_007672754.1 uncharacterized protein BAUCODRAFT_371348 [Baudoinia panamericana UAMH 10762]

MASELANKLEATTLNESSTNNDWRAGLKAPPKDGRVQTEDVTATKGLEFEDFYLKRELLMGIFEAGFEKPSPIQEETIPV

ALTGRDILARAKNGTGKTAAFVIPTLERINPKLEKTQALILVPTRELALQTSQVCKTLGKHLGINVMVTTGGTGLKDDII

RLNEVVHIIVGTPGRILDLASKGVADLSAAKTFVMDEADKLLSPEFTVTIEQLLQFHPADRQVMLFSATFPIVVKSFMDK

HMRDPHEINLMDELTLRGITQYYAFVEEKQKVHCLNTLFSRLQINQSIIFCNSTTRVELLAKKITELGYSCFYSHAKMLQ

QHRNRVFHDFRNGAMRNLVCSDLLTRGIDIQAVNVVINFDFPKNAETYLHRIGRSGRFGHLGLAINLINWDDRFNLYRIE

QELGTEIQPIPPQIDKSLYVYENAESIPRPINTAPAQAEQRQQHDTRRQQGNGADLANPANRTGGYRGGRGGGGRGGGGY

RGPPRDPNQQGQNGGYQGQQRQGGYQQRPPPQQQAMPQSAAHA

>XP_022463732.1 hypothetical protein KNAG_0C03820 [Kazachstania naganishii CBS 8797]

MPSTMENQDWKSQLNIPKKDSRPQTDDVLKTKGNTFEDFYLKRELLMGIFEAGFEKPSPIQEEAIPVAITGRDILARAKN

GTGKTAAFVIPTLEKVKPKLNKIQALIMVPTRELALQTSQVVRSLGKHCGVSCMVTTGGTNLRDDILRLNDTIHILVGTP

GRVLDLASRKIADLSECGLFVMDEADKMLSRDFKTIIEQILAFLPKNHQSLLFSATFPLTVKEFMEKHLNKPYEINLMEE

LTLKGITQYYAFVEERQKLHCLNTLFSKLQINQAIIFCNSTNRVELLAKKITDLGYSCYYSHARMKQQERNRVFHEFRQG

KVRTLVCSDLLTRGIDIQAVNVVINFDFPKTAETYLHRIGRSGRFGHLGLAINLINWNDRFNLYKIEQELGTEIAAIPST

IDKSLYVADDATNVPVPFPIEQQNLAHQNGQNSQQQTATSMANIQIPPQQNHPQFAAQFNVPPQQQQLQQQQLLQHQQLQ

QQQQLQQQQPNVYQGLPPSTHQMGYPPMQQPQF

>SPO40618.1 probable ATP-dependent RNA helicase DHH1 [Anthracocystis flocculosa]

MSSSGPAPGQSEASWKQEILGKLRKDDRPQTEDVLNTKGNDFEDYFLKRELLMGIFEAGFERPSPIQEEAIPIALTGRDI

LARAKNGTGKTAAYVIPSLEKLNTKKNVIQAVLLVPTRELALQTSQVAKTLGKHLGVEVMVTTGGTTLRDDILRLGQTVH

LLVGTPGRILDLAGKGVADLSQCTTFVMDEADKLLSPEFTPVMEQLLSFLPKERQVMLFSATFPMIVKDFKDKNMVKPYE

INLMDELTLRGVTQYYAFVEERQKVHCLNTLFSKLQINQSIIFCNSTNRVELLAKKITELGYSCFYSHAKMLQSHRNRVF

HDFRNGACRNLVCSDLLTRGIDIQAVNVVINFDFPKNAETYLHRIGRSGRFGHLGLAINLITYEDRFNLYRIEQELGTEI

QPIPSNIDKRLYVAPSLIQNEGDERRQGQNGGGNGNGAGANGRQQEGRPVIPPGQAAMHAASIPQSQDFTQTVPHRHRGG

GGHGGHGGGGGGGHGGGGHVQAA

>EFW16869.1 ATP-dependent RNA helicase dhh1 [Coccidioides posadasii str. Silveira]

MAEALASQLNKAKLGDNGAETKWKEQLKLPPKDTRTQTEDVTATKGLEFEDFYIKRELMMGIFEAGFEKPSPIQEETIPV

ALTGRDILARAKNGTGKTAAFVIPTLERTNPKISKTQALILVPTRELALQTSQVCKTLGKHLGINVMVTTGGTGLQDDII

RLSDTVHIIVGTPGRILDLASKGVADLSECTTFVMDEADKLLSPEFTPVIEQLLSFHPKDRQVMLFSATFPMIVKSFKDK

HMRNPYEINLMDELTLRGITQYYAFVEERQKVHCLNTLFSKLQINQSIIFCNSTNRVELLAKKITELGYSCFYSHARMLQ

QNRNRVFHDFRNGVCRNLVCSDLLTRGIDIQAVNVVINFDFPKNAETYLHRIGRSGRFGHLGLAINLINWDDRYNLYKIE

QELGTEIQPIPPSIDKKLYVYDTPETIPRPIANASTERNPPAQLAQSSDNQNHRQAHHIGGGHGQQTANRGHSLRGSYRG

GRAQGHRGGHPENNRTNPMSSRSNMPTSTTAS

>KFY81981.1 hypothetical protein V498_08673 [Pseudogymnoascus sp. VKM F-4517 (FW-2822)]

MADALAGKLESTTLGSTPNDADWKKKLKIPAKDNRHQTEDVTATKGLEFEEFSIKRDLLMGIFEAGFEKPSPIQEEAIPV

ALTGRDILARAKNGTGKTAAFVIPALERINPKSSKIQCLILVPTRELALQTSQVCKTLGKHLGVNVMVTTGGTGLRDDIV

RLAEPVHIVVGTPGRILDLAGKNVADLSECPMFIMDEADKLLSPEFTPVIEQLLQFHPKDRQIMLFSATFPRSVQAFSVK

NMDQPYEINLMDELTLRGITQYYAFVEEKQKVHCLNTLFSKLQINQSIIFCNSTNRVELLAKKITELGYSCFYSHARMLQ

ANRNRVFHDFRNGVCRNLVCSDLLTRGIDIQAVNVVINFDFPKNAETYLHRIGRSGRFGHLGLAINLINWDDRFNLYNIE

KELGTEIQPIPAMIDKNLYVYDSPESIPRPISNPAPRQQAPREQLQIQQRGQAPQNYSNGRQGPSGQAQSQNQAQAQAQA

QAQAQAQAQAQAQAQAQAQAQAQAQAQAQAQAQAQAQAQAQAQAQAQAQAQAQAQAQAQAQAQAQAQAQAQAQAQAQAQA

QAQAQAQAQAQAQAQAQAQAQAQAQAQAQAQAQAQAQAQAQAQAQAQAQAQAQAQAQAQAQAQAQLQQGQSQGQRQPSNQ

PQGQQRSQNFSRGNARVGAQPGRGRGFDGARGGRGQAPPPS

>XP_004181296.1 hypothetical protein TBLA_0F02360 [Tetrapisispora blattae CBS 6284]

MSTVQQQLDTNDWKSKLNIPKKDTRPQTEDVLNTKGNSFEDFYLKRELLMGIFEAGFEKPSPIQEESIPIAIAGRDILAR

AKNGTGKTAAFVIPTLEKVKPKINKIQALIMVPTRELALQTSQVVRTLGKHCGISCMVTTGGTNLRDDIMRLNETIHVLV

GTPGRVLDLASRKVADLSECSLFIMDEADKMLSRDFKTIIEQILIFLPKHHQSLLFSATFPLTVKEFMVKHLTKPYEINL

MDELTLKGITQYYAFVEERQKLHCLNTLFSKLQINQAIIFCNSTNRVELLAKKITDLGYSCYYSHARMKQQERNRVFHEF

RQGKVRTLVCSDLLTRGIDIQAVNVVINFDFPKTAETYLHRIGRSGRFGHLGLAINLINWNDRFNLYKIEQELGTEIAAI

PSTIDKSLYVANDNSAVPIPFPITNDQHADNQNQMPNQMPNQMPPQQFNPQFNPQQQMMNMQMPPPPAQQQNFKSTSILR

AIIYMLPPPTFSPFLRIFQFDLQFFFYLFLSFYIYFFYLQILFFLSLSLCSLFYFISFSIHTFLYLVIFSHYYFLLDHFF

LFSIHKKKINPSHSYHFLTPSPATN

>RYC62147.1 hypothetical protein CHU98_g4074 [Xylaria longipes]

MTESITKKLQASTLNDATDNSDWKSNLNIPARDSRHQTEDVTNTKGLEWEEFSLKRDLLMGIFEAGYEKPSPIQEEAIPV

ALTGRDILARAKNGTGKTAAFVVPTLERINPKVNKIQCLILVPTRELAMQTSQVCKTLGKHLGVNVMVTTGGTTLRDDIV

RLQDAVHIVVGTPGRILDLAGKGVADLSECPMFIMDEADKLLSIEFTPVIEQLMQFHPKDRQVMLFSATFPLSVKDFSDK

NMSKPYEINLMDELTLRGITQYYAFVEEKSKVHCLNTLFSKLQINQSIIFCNSTNRVELLAKKITELGYSCFYSHAKMAQ

HARNRVFHDFRNGVCRNLVCSDLLTRGIDIQAVNVVINFDFPKNAETYLHRIGRSGRYGHLGLAINLINWEDRYNLYNIE

KDLGTEIQPIPSTIDKALYVYDNPDNIPRPVNTYATKQATTHKLQQGETSTQAAQQNNLYPPQGRGQGDFRPQAGRGGHF

NDSQPQPRQYQNNRGGHRGGRGRGRGFQHFGGRGGGRGQAQPPPQA

>ESZ89682.1 ATP-dependent RNA helicase DHH1 [Sclerotinia borealis F-4128]

MADALATKLAATQINDNANPEDWKAGLNIPVKDTRHQTEDVTATKGLEFEEFSIKRDLLMGIFEAGFEKPSPIQEEAIPV

ALTGRDILARAKNGTGKTAAFAIPALERINPKSNKVQCLILVPTRELALQTSQVCKTLGQHLGVNVMVTTGGTTLRDDIM

RLSEAVHIIVGTPGRILDLAGKNVADLSECSMFIMDEADKLLSPEFTIVIEQLLQFHPKDRQIMLFSATFPMTVKDFSDK

NMNSPYEINLMDELTLRGITQYYAFVEEKDKVHCLNTLFSKLQINQSIIFCNSTNRVELLAKKITELGYSCFYSHARMIQ

ANRNRVFHDFRNGVCRNLVCSDLLTRGIDIQAVNVVINFDFPKNAETYLHRIGRSGRFGHLGLAINLISWEDRFNLYNIE

QELGTEIAAIPQTIERSLYVYETPENIPRPVSNFQTNARQPSGRDQQQPTQHSGNQRSGGQNNFNNGRPNGSGQIHSQGQ

GQGPRQIQGQRPNYSRGRGGFGGGRGGGQGPRQNGFDNSRGGRGQGPPAQ

>KAA1472530.1 eukaryotic translation initiation factor 4A-like protein [Dentipellis sp. KUC8613]

MSQSTRPSSSSSHNDNWRAGLRPPPRDERPKTEDVKNIKGLEFEDMYLRRELLMGIFEAGFETPSPIQEEAIPIALTKRD

ILARAKNGTGKTAAFVIPSLQQVDVSKNKIQALLLVPTRELALQTSQVCKTLGKHMGVQVMVTTGGTTLKDDILRLSEEV

HVLVGTPGRILDLAGKNVADLSECPVFVMDEADKLLSPEFSPVMEQLLSYLPKDRQVMLFSATFPLIVKDFKNKHMNSPY

EINLMDELTLRGVTQYYAYVEERQKVHCLNTLFSKLQINQSIIFCNSTNRVELLAKKVTELGYSCFYSHAKMLQSHRNRV

FHDFRNGVCRNLVCSDLLTRGIDIQAVNVVINFDFPKNSETYLHRIGRSGRFGHLGLAINLVTYEDRFNLYKIEQELGTE

IQPIPQQIDKGLYVAPSAQDEPTPQKPARTQEQQAAQQRQQQQQTAAQVQGPSRPPAQQVVYQSNGQPGRPNGAPGAVQG

QARPYPAAPAGAYQGQVPVAQR

>CCJ28576.1 unnamed protein product [Pneumocystis jirovecii]

MSSLRGPDSVVLDSSKTYDDWKKTLNLSTKDTRPQTEDVTKTKGNEFEDFYLKRELLMGIFEAGFERPSPIQEESIPIAL

TGRDILARAKNGTGKTAAFIIPALEKLNSKKPKTQALILVPTRELALQTSHVCKTLGKHMGINVMVTTGGTSLQQDIIRL

HDPVHVIVGTPGRILDLAGKGVAEFSECFTFIMDEADKLLSLEFTPVIEQLLAYFPKDRQIMLYSATFPLVVKSFMDKHL

SKPYEINLMDELTLRGVTQYYAFVEEKQKVHCLNTLFSKLQINQSIIFCNSTNRVELLAKKITELGYSCFYSHAKMLQNH

RNRVFHDFRNGVCRNLVCSDLLTRGIDIQAVNVVINFDFPKNSETYLHRIGRSGRFGHLGLAINLINWEDRFNLYKIEQE

LATEIQPIPSIIDKRLYVSPSALDEPVNNRPRPKQRSVAPQSNGNANSQLNNEQSYIRQHPLRGRGGNRARSHGRRYSDR

VNGHTFNCKVRIDIDIFLDL

>XP_003065260.1 ATP-dependent RNA helicase, putative [Coccidioides posadasii C735 delta SOWgp]

MAEALASQLNKAKDNGAETKWKEQLKLPPKDTRTQTEDVTATKGLEFEDFYIKRELMMGIFEAGFEKPSPIQEETIPVAL

TGRDILARAKNGTGKTAAFVIPTLERTNPKISKTQALILVPTRELALQTSQVCKTLGKHLGINVMVTTGGTGLQDDIIRL

SDTVHIIVGTPGRILDLASKGVADLSECTTFVMDEADKLLSPEFTPVIEQLLSFHPKDRQVMLFSATFPMIVKSFKDKHM

RNPYEINLMDELTLRGITQYYAFVEERQKVHCLNTLFSKLQINQSIIFCNSTNRVELLAKKITELGYSCFYSHARMLQQN

RNRVFHDFRNGVCRNLVCSDLLTRGIDIQAVNVVINFDFPKNAETYLHRIGRSGRFGHLGLAINLINWDDRYNLYKIEQE

LGTEIQPIPPSIDKKLYVYDTPETIPRPIANASTERNPPAQLAQSSDNQNHRQAHHIGGGHGQQTANRGHSLRGSYRGGR

AQGHRGGHPENNRTNPMSSRSNMPTSTTAS

>CDF91248.1 ZYBA0S10-03026g1_1 [Zygosaccharomyces bailii CLIB 213]

MSKEDWSSQLKIPAKDTRPQTDDVLNTKGNTFEDFCLKRELLMGIFEAGFEKPSPIQEESIPVAIAGRDILARAKNGTGK

TAAFVIPTLEKVKPKINKIQALIMVPTRELALQTSQVVRTLGKHCGISCMVTTGGTNLRDDILRLNETVHVLVGTPGRVL

DLASRKVADLSDCSLFVMDEADKMLSRDFKTIIEQILIFLPPQHQSLLFSATFPITVKEFMVKHLHKPYEINLMEELTLK

GITQYYAFVEERQKLHCLNTLFSKLQINQAIIFCNSTNRVELLAKKITDLGYSCYYSHARMRQQERNKVFHDFRQGKVRT

LVCSDLLTRGIDIQAVNVVINFDFPKTAETYLHRIGRSGRFGHLGLAINLINWNDRFNLYKIEQELGTEIAAIPATIDKS

LYVAEDNSNIPTPYPIQHQQPQSQPKPQPQYASPPQGFTAQGVPQQLSPHVMAPHGLAAQNLSPHGLTQQGLPPQGPAQG

LPQGTSPQTLAQGLPQGIMAQGIPPPYAAQPHF

>XP_033461951.1 DEAD-domain-containing protein [Dissoconium aciculare CBS 342.82]

MASELSNKLEATSLNDSTPNAGDYRAGLKAPPKDNRHQTEDVTATKGIEFEEFYLKRELLMGIFEAGFEKPSPIQEETIP

VALTGRDVLARAKNGTGKTAAFVIPTLERVNAKVDKIQALLLVPTRELALQTSQVCKTLGKHLGINVMVTTGGTGLRDDI

MRLNEPVHIVVGTPGRILDLASKGVADLSHAQTFVMDEADKLLSPEFTVTIEQLLQFHPKDRQVMLFSATFPVVVKDFKD

KHMNDPHEINLMDELTLRGITQYYAFVEEKQKVHCLNTLFSRLQINQSIIFCNSTTRVELLAKKITELGYSCFYSHAKML

QHHRNRVFHDFRNGAMRNLVCSDLLTRGIDIQAVNVVINFDFPKNAETYLHRIGRSGRFGHLGLAINLINWEDRFNLYRI

EQELGTEIQPIPAAIDKSLYVYDSPESIPRPINTAPPNQSGQPANGRQQGSGQDLLNPANRTGSGHRGGRGGYAGQPRRG

GFAPRGGPSQAPGPPAAHA

>SSD62142.1 probable ATP-dependent RNA helicase DHH1 [Saccharomycodes ludwigii]

MDSSDWKSSLTLPKKDIRPQTDDVTKTKGNSFEDFYLKRELLMGIFEAGFENPSPIQEESIPIAIAGRDILARAKNGTGK

TAAFVIPTLEKLKPKVNKIQALILVPTRELALQTSQVVRTLGKHLNISCMVSTGGTNLRDDILRLHEPVHVLVGTPGRVL

DLASRKVADLSECHMFIMDEADKMLSRDFKTLVEQILTFFPAKHQSLLFSATFPLTVKYFMDQHLEKPYEINLMDELTLK

GITQYYAFVEERQKLHCLNTLFSKLQINQAIIFCNSTNRVELLAKKITDLGYSCYYSHARMSQQERNTVFHEFRQGKVRT

LVCSDLLTRGIDIQAVNVVINFDFPKTAETYLHRIGRSGRFGHLGLAINLINWNDRFSLYKIEQELGTEIQPIPPTIDKS

LYVAEDSTAIPKPFATPATINGPQTSIAQQLPSQQQQYQQQQYQQQQYQQQQPQQQQPQPQQFNLPPQQGNPQFAMQYPV

QQPQQQFNHYPQQPQQSAPFNGYQQPQQQF

>XP_002584013.1 hypothetical protein UREG_06980 [Uncinocarpus reesii 1704]

MADSLASQLNQTRLGFVPRENGAEVKWKEQLKLPPKDTRTQTEDVTATKGLEFEDFYIKRELMMGIFEAGFEKPSPIQEE

TIPVALTGRDILARAKNGTGKTAAFVIPTLERTNPKISKTQALILVPTRELALQTSQVCKTLGKHLGINVMVTTGGTGLQ

DDIIRLNDTVHIIVGTPGRILDLASKGVADLSECATFVMDEADKLLSPEFTPVIEQLLSFHPKDRQVMLFSATFPMIVKS

FKDKHMRNPYEINLMDELTLRGITQYYAFVEERQKVHCLNTLFSKLQINQSIIFCNSTNRVELLAKKITELGYSCFYSHA

RMFQQNRNRVFHDFRNGVCRNLVCSDLLTRGIDIQAVNVVINFDFPKNAETYLHRIGRSGRFGHLGLAINLIGWEDRYNL

YKIEQELGTEIQPIPPSIDKKLYVYDTPETIPRPIANAPSGRNTLSTQQATNSESQHRRQGHPGSGHGQQAANRGQQSRG

SYRGGRGQGHRANHPESNRMNPGLPASTAPTS

>CRG83272.1 ATP-dependent RNA helicase DDX6/DHH1 [Talaromyces islandicus]

MADTLATQLSNTKLGDVNSDGAWKDTLKIPAKDTRTQTEDVTATKGLEFEDFYIKRELMMGIFEAGFEKPSPIQEETIPV

ALTGRDILARAKNGTGKTAAFIIPTLERINPKNTKTQALILVPTRELALQTSQVCKTLGKHLGINVMVTTGGTGLMDDII

RLNDAVHIIVGTPGRVLDLASKGVADLSECPTFVMDEADKLLSPEFTPVIEQLLSFHPKDRQVMLFSATFPMIVKSFKDK

HMRNPYEINLMDELTLRGITQYYAFVEEKQKVHCLNTLFSKLQINQSIIFCNSTNRVELLAKKITELGYSCFYSHARMLQ

QNRNRVFHDFRNGVCRNLVCSDLLTRGIDIQAVNVVINFDFPKNAETYLHRIGRSGRFGHLGLAINLINWEDRFNLYKIE

QELGTEIQPIPQAIDKKLYVYESPDTIPRPIASAPQPRQQEAEFDQQNRRHGNYHPAGNFGGPNRGRGHYRGRGQGQRRG

GNHHDNHRSMGGHPHDTKPQPTPAS

>ODH46787.1 ATP-dependent RNA helicase DHH1 [Paracoccidioides brasiliensis]

MADALASQLSSAKLGENNHSEKWKEHLKAPPKDTRTQTEDVTATKGLEFEDFYIKRELMMGIFEAGFEKPSPIQEETIPV

ALTGRDILARAKNGTGKTAAFVIPTLERTNPKNSKTQALILVPTRELALQTSQVCKTLGKHLGINVMVTTGGTGLQDDII

RLNEAVHIIVGTPGRILDLASKGVADLSECSTFVMDEADKLLSPEFTPVIEQLLSFHPKDRQIMLFSATFPLIVKSFKDK

HMRNPYEINLMDELTLRGITQYYAFVEEKQKVHCLNTLFSKLQINQSIIFCNSTNRVELLAKKITELGYSCFYSHAKMLQ

HNRNRVFHDFRNGVCRNLVCSDLLTRGIDIQAVNVVINFDFPKNAETYLHRIGRSGRFGHLGLAINLINWEDRYNLYKIE

QELGTEIQPIPQSIDKKLYVYDTPDTIPRPITSTPTQPPKQQQQQQQQQASAPAQYPNNPEGHSRRQGHSQYPSHRGRGS

YRGGRGQGHRGGHAENHRQNGTGPRPSAAASAPVS

>RMZ82303.1 hypothetical protein DV738_g1811, partial [Chaetothyriales sp. CBS 135597]

MASQLADQLDKTTLTDRAPDADWKKNLKARPKDEREQTEDVTATKGLEFEDFYIKRELMMGIFEAGFEKPSPIQEETIPV

ALTGRDILARAKNGTGKTAAFVIPTLEKVNPKNPKIQALLLVPTRELALQTSQVCKTLGKHLGLEVMVTTGGIGLKDDII

RLQSTVHILVGTPGRILDLTSKGVADVSECSIFVMDEADKLLSPEFTVVIEQLLNFHPKDRQVMLFSATFPLVVKDFKDK

HMRDPYEINLMDELTLRGITQYYAFVEEKQKVHCLNTLFSKLQINQSIIFCNSTNRVELLAKKITELGYSCFYSHARMLQ

QHRNKVFHDFRAGVSRNLVCSDLLTRGIDIQAVNVVINFDFPKNAETYLHRIGRSGRFGHLGLAINLITWEDRLNLYRIE

QELGTEIQPIPQSIDKSLYVYENPENIPRPLPTSQPTAPTGGQNDGGRKPNGYGQHVRGRFNRGVGGPRGGHGHGRGGRG

GGQFAQQVQNGVPAQ

>XP_010761262.1 ATP-dependent RNA helicase DHH1 [Paracoccidioides brasiliensis Pb18]

MADALASQLSSAKLGENNHSEKWKEHLKAPPKDTRTQTEDVTATKGLEFEDFYIKRELMMGIFEAGFEKPSPIQEETIPV

ALTGRDILARAKNGTGKTAAFVIPTLERTNPKNSKTQALILVPTRELALQTSQVCKTLGKHLGINVMVTTGGTGLQDDII

RLNEAVHIIVGTPGRILDLASKGVADLSECSTFVMDEADKLLSPEFTPVIEQLLSFHPKDRQIMLFSATFPLIVKSFKDK

HMRNPYEINLMDELTLRGITQYYAFVEEKQKVHCLNTLFSKLQINQSIIFCNSTNRVELLAKKITELGYSCFYSHAKMLQ

HNRNRVFHDFRNGVCRNLVCSDLLTRGIDIQAVNVVINFDFPKNAETYLHRIGRSGRFGHLGLAINLINWEDRYNLYKIE

QELGTEIQPIPQSIDKKLYVYDTPDTIPRPITSTPTQPPKQQQQQQQQQASAPAQYPNNPEGHSRRQGHSQYPSHRGRGS

YRGGRGQGHRGGHAENHRQNGTGPRPNAAASAPVS

>PIG83439.1 ATP-dependent RNA helicase dhh1 [Aspergillus arachidicola]

MAEALASQLNNTTLGEASSDTRWKDQLKAPAKDARPQTEDVTATKGLEFEDFYIKRELMMGIFEAGFEKPSPIQEETIPV

ALTGRDILARAKNGTGKTAAFVIPTLERINPKSTKTQALILVPTRELALQTSHVCKTLGKHLGINVMVTTGGTGLMDDII

RLNDAVHILVGTPGRVLDLASKGVADLSECPTFVMDEADKLLSPEFTPVIEQLLSFHPKDRQVMLFSATFPLIVKSFKDK

HMRNPYEINLMDELTLRGITQYYAFVEEKQKVHCLNTLFSKLQINQSIIFCNSTNRVELLAKKITELGYSCFYSHARMLQ

QHRNRVFHDFRNGVCRNLVCSDLLTRGIDIQAVNVVINFDFPKNAETYLHRIGRSGRFGHLGLAINLINWDDRFNLYKIE

QELGTEIQPIPQNIDKKLYVYESPETIPRPIANASQAQLATSGNQTQNMGERRNNNHSNGGHYQFGRGRGSYRGGRSQGQ

RRNMQNEMNKFGTSQNQQQSGKSQPAQPSSLRSIIAGSTAGAIEIAITYPAEFAKTRSQLNRKLPDGKKLPWPPFGKQWY

AGCTTLIIGNSLKAGIRFVAFDRFKSLLQDENGKISGPRTVIAGFGAGFTESLLAVTPFESIKTQLSVAFKPSLATIDDR

KSANPRMRGFLHGSKLIFQERGIRGFFQGFVPTTARQAANSATRFSSYTMLKQLAESYVAPGEKLGTASTFAIGGMAGFI

TVYVTQPLDTVKTRMQSLEASKNYKNSFVCAARIFKDEGLFTFWSGAVPRLARLILSGGIVFTMYEKTMDALDSLDSKRQ

YI

>GHJ86005.1 hypothetical protein NliqN6_2407 [Naganishia liquefaciens]

MTSVNAAANSSTNGADWKKRLQAPPKDERPQTEDVTATKGNVFEDFGLKRELLMGIFEAGFEKPSPIQEEAIPVALSGKD

ILARAKNGTGKTAAFVIPALQQINPLLPHVQAIILVPTRELALQTSQVCKTLSKHMGLEIMVTTGGTTLKDDIMRLEQTV

HIMVATPGRLLDLAQKGIADLKKCKTFIMDEADKLLSKEFTIVVEQLLAMCAPDRQVMLFSATFPLVVKDFRDRNMHDAH

EINLMEELTLKGVTQYYAFVEERQKVQCLNTLFSKLQINQSIIFCNSTNRVELLAKKITELGYSCFYSHAKMLQAHRNRV

FHDFRAGATRNLVCSDLLTRGIDIQAVNVVINFDFPKTAESYLHRIGRSGRFGHLGLAVSLVTVEDRHNLYRIEQELGTQ

IAAIPSNIDPKLYVAPYGQADEGEQAQATQQPVPSKAISNHGAQQAQLPPSGNINQMRAPQQRMQAPQNGSINSPQPSQH

TPQMPNGMSAQGYLQQFAGPPRAGPPPGAY

>AQZ18442.1 DHH1 (YDL160C) [Zygosaccharomyces parabailii]

MSKEDWSSQLKIPAKDTRPQTDDVLNTKGNTFEDFCLKRELLMGIFEAGFEKPSPIQEESIPVAIAGRDILARAKNGTGK

TAAFVIPTLEKVKPKINKIQALIMVPTRELALQTSQVVRTLGKHCGISCMVTTGGTNLRDDILRLNETVHVLVGTPGRVL

DLASRKVADLSDCSLFVMDEADKMLSRDFKTIIEQILIFLPPQHQSLLFSATFPITVKEFMVKHLHKPYEINLMEELTLK

GITQYYAFVEERQKLHCLNTLFSKLQINQAIIFCNSTNRVELLAKKITDLGYSCYYSHARMRQQERNKVFHDFRQGKVRT

LVCSDLLTRGIDIQAVNVVINFDFPKTAETYLHRIGRSGRFGHLGLAINLINWNDRFNLYKIEQELGTEIAAIPATIDKS

LYVAEDNSNIPTPYPIQHQQPQSQPKPQPQYASPPQGFTAQGVPQQLSPHVMAPHGLAAQNLSPHGLTQQGLPPQGPAQG

LPQGTSPQTLAQGLPQGIMAQGIPPPCAAQPHF

>KFY26492.1 hypothetical protein V493_04059 [Pseudogymnoascus sp. VKM F-4281 (FW-2241)]

MADALAGKLESTTLGSSQNDADWKKKLKIPAKDNRQQTEDVTATKGLEFEEFSIKRDLLMGIFEAGFEKPSPIQEEAIPV

ALTGRDILARAKNGTGKTAAFVIPALERINPKSSKIQCLILVPTRELALQTSQVCKTLGKHLGVNVMVTTGGTGLRDDIV

RLAEPVHIVVGTPGRILDLAGKSVADLSECPMFIMDEADKLLSPEFTPVIEQLLQFHPKDRQIMLFSATFPRSVQAFSVK

NMDQPYEINLMDELTLRGITQYYAFVEEKQKVHCLNTLFSKLQINQSIIFCNSTNRVELLAKKITELGYSCFYSHARMLQ

ANRNRVFHDFRNGVCRNLVCSDLLTRGIDIQAVNVVINFDFPKNAETYLHRIGRSGRFGHLGLAINLINWDDRFNLYNIE

KELGTEIQPIPAMIDKNLYVYDSPESIPRPISNPAPRQQAPREQLQIQQRGQAPANYSNGRQGPSSQAQPQNQAQAQAQA

QAQAQAQAQAQAQAQAQAQAQAQAQAQAQAQAQAQAQAQAQAQAQAQAQAQAQAQAQAQAQAQAQAQAQAQAQAQAQAQA

QAQAQAQAQAQAQAQAQAQAQAQAQAQAQAQAQAQAQAQAQAQAQAQAQAQAQAQAQAQAQAQAQAQAQAQAQAQAQAQA

QAQAQAQAQAQAQAQAQAQAQAQAQAQLQQGQSQAQRQPSNQPQGQRNQNFSRGNARVGTQPGRGRGFDGARGGRGQVPP

PS

>RMD41015.1 hypothetical protein DV735_g4138, partial [Chaetothyriales sp. CBS 134920]

MASQLADQLGKTTLTDRAPDADWKKKLKARPKDEREQTEDVTATKGLDFEDFYIKRELMMGIFEAGFEKPSPIQEETIPV

ALTGRDILARAKNGTGKTAAFVIPTLEKVNPKNPKIQALLLVPTRELALQTSQVCKTLGKHLGLEVMVTTGGIGLKDDII

RLQSPVHILVGTPGRILDLTSKGVADVSECSIFVMDEADKLLSPEFTVVIEQLLNFHPKDRQVMLFSATFPLVVKDFKDK

HMRDPYEINLMDELTLRGITQYYAFVEEKQKVHCLNTLFSKLQINQSIIFCNSTNRVELLAKKITELGYSCFYSHARMLQ

QHRNKVFHDFRAGVSRNLVCSDLLTRGIDIQAVNVVINFDFPKNAETYLHRIGRSGRFGHLGLAINLITWEDRLNLYRIE

QELGTEIQPIPQSIDKSLYVYDKPENIPRPLPTSQPTAPAGGQNEGGRRANGYGQHVRGRFNRGVGGPRGGHGHGRGGRG

GGQFVQQVQNGVPAQ

>XP_007756258.1 ATP-dependent RNA helicase DHH1 [Cladophialophora yegresii CBS 114405]

MTDALASQLNKTSLNDGADDSTWKDSLKLPAKDTRQQTEDVTATKGLEFEDFYIKRELMMGIFEAGFEKPSPIQEETIPV

ALTGRDILARAKNGTGKTAAFVIPTLERINPKNPKTQALILVPTRELALQTSQVCKTLGKHLGINVMVTTGGTGLKDDIL

RLGETVHIIVGTPGRILDLASKGVADLSECPIFVMDEADKLLSPEFTVVIEQLLSFHPKDRQVMLFSATFPMIVKTFKDK

HMRNPYEINLMDELTLRGITQYYAFVEEKQKVHCLNTLFSKLQINQSIIFCNSTNRVELLAKKITELGYSCFYSHAKMLQ

QNRNKVFHDFRAGVSRNLVCSDLLTRGIDIQAVNVVINFDFPKNAETYLHRIGRSGRFGHRGLAINLINWDDRYNLYKIE

QELGTEIQPIPPSIDKSLYVYDNPENIPRALPPPAPAARPNTSTTPTNQHATNANGQYPQFRKPQNQFNGFPQQYNNNNN

QGRGGYRGRGRGQGARGRGGHPPQAAAVGQTAQ

>KAF2175563.1 ATP-dependent RNA helicase DHH1 [Zopfia rhizophila CBS 207.26]

MTTDITNQLAATKISDAASSPDANWKDGLKIPSKDARPQTEDVTATKGLEFEDFFIKRELMMGIFEAGFEKPSPIQEETI

PVALTGRDILARAKNGTGKTAAFVIPTLERVNPKSNKTQALILVPTRELALQTSQVCKTLGKHLGINVMVSTGGTGLKDD

IIRLSDPVHIIVGTPGRILDLAGKGVADLSACQTFVMDEADKLLSPEFTPVVEQLLGFHPKDRQVMLFSATFPIVVKSFK

DKHMNSPYEINLMDELTLRGITQYYAFVEEKQKVHCLNTLFNKLQINQSIIFCNSTNRVELLAKKITELGYSCFYSHARM

LQHNRNRVFHDFRNGVCRNLVCSDLLTRGIDIQAVNVVINFDFPKNAETYLHRIGRSGRFGHLGLAINLINWEDRFNLYR

IEQELGTEIQPIPQVIEKKLYVYESPENIPRPISNPHRNQGQGNNQEQDGAGNVSRSNSTQVNNRQNYNSNQRGGGQFQG

QRRGPPQNQQNRQNGHNPQRNPRPQPAGPA

>XP_003173227.1 ATP-dependent RNA helicase DHH1 [Nannizzia gypsea CBS 118893]

MTDALATQLNDTKLGDASTDNKWKEQLKIPAKDTRVQTEDVTATKGLEFEDFYIKRDLMMGIFEAGFEKPSPIQEETIPV

ALTGRDILARAKNGTGKTAAFVIPTLERTNPKIAKTQALILVPTRELALQTSQVCKTLGKHLGINVMVTTGGTGLQDDII

RLNDTVHIIVGTPGRILDLASKGVADLSECTTFVMDEADKLLSPEFTPVIEQLLTFHPKDRQVMLFSATFPIIVKTFKDK

HMRNPYEINLMDELTLRGITQYYAFVEERQKVHCLNTLFSKLQINQSIIFCNSTNRVELLAKKITELGYSCFYSHARMLQ

HNRNRVFHDFRNGVCRNLVCSDLLTRGIDIQAVNVVINFDFPKNAETYLHRIGRSGRFGHLGLAINLINWDDRYNLYKIE

QELGTEIQPIPPSIDKKLYVYDTPNTIPRPISNNPPEQHKPTSQENHPRRQSNNPSNGHGQYNSNRGHYSRGSYRGGRGS

SQRSNNHENSRAAGNPQASAANSGAPAS

>OBT64112.1 ATP-dependent RNA helicase DDX6/DHH1 [Pseudogymnoascus sp. 23342-1-I1]

MADALAGKLESTTLGSSQNDADWKKKLKIPAKDNRQQTEDVTATKGLEFEEFSIKRDLLMGIFEAGFEKPSPIQEEAIPV

ALTGRDILARAKNGTGKTAAFVIPALERINPKSSKIQCLILVPTRELALQTSQVCKTLGKHLGVNVMVTTGGTGLRDDIV

RLAEPVHIVVGTPGRILDLAGKSVADLSECPMFIMDEADKLLSPEFTPVIEQLLQFHPKDRQIMLFSATFPRSVQAFSVK

NMDQPYEINLMDELTLRGITQYYAFVEEKQKVHCLNTLFSKLQINQSIIFCNSTNRVELLAKKITELGYSCFYSHARMLQ

ANRNRVFHDFRNGVCRNLVCSDLLTRGIDIQAVNVVINFDFPKNAETYLHRIGRSGRFGHLGLAINLINWDDRFNLYNIE

KELGTEIQPIPAMIDKNLYVYDSPESIPRPISNPAPRQQAPREQLQIQQRGQAPANYSNGRQGPSSQAQPQNQAQAQAQA

QAQAQAQAQLQQGQSQGQRQPSNQPQGQQRNQNFSRGNARVGAQPGRGRGFDGARGGRGQAPPPS

>PGH00473.1 ATP-dependent RNA helicase dhh1 [Blastomyces parvus]

MADALASQLNSTKLGDTHSEKWRENLKAPPKDTRTQTEDVTATKGLEFEDFYIKRELMMGIFEAGFEKPSPIQEETIPVA

LTGRDILARAKNGTGKTAAFVIPTLERTNPKNSKTQALILVPTRELALQTSQVCKTLGKHLGINVMVTTGGTGLQDDIIR

LNEAVHIIVGTPGRILDLASKGVADLSECSTFVMDEADKLLSPEFTPVIEQLLSFHPKDRQVMLFSATFPMIVKSFKDKH

MRNPYEINLMDELTLRGITQYYAFVEEKQKVHCLNTLFSKLQINQSIIFCNSTNRVELLAKKITELGYSCFYSHAKMLQH

NRNRVFHDFRNGVCRNLVCSDLLTRGIDIQAVNVVINFDFPKNAETYLHRIGRSGRFGHLGLAINLINWEDRYNLYKIEQ

ELGTEIQPIPQSIDKKLYVYDTPDTIPRPISNAPQPQQQPASAHAQHINNPEGHNRRQGQHQYSSNRGRGSYRGGRGQGH

RGGHGENHRQNGTAPRPNPAASAPAS

>KFY83924.1 hypothetical protein V500_09773 [Pseudogymnoascus sp. VKM F-4518 (FW-2643)]

MADALAGKLESTTLGSIQNDPDWKKKLKIPAKDNRHQTEDVTATKGLEFEEFSIKRDLLMGIFEAGFEKPSPIQEEAIPV

ALTGRDILARAKNGTGKTAAFVIPALERINPKSSKIQCLILVPTRELALQTSQVCKTLGKHLGVNVMVTTGGTGLRDDIV

RLAEPVHIVVGTPGRILDLAGKSVADLSECPMFIMDEADKLLSPEFTPVIEQLLQFHPKDRQIMLFSATFPRSVQAFSVK

NMDQPYEINLMDELTLRGITQYYAFVEEKQKVHCLNTLFSKLQINQSIIFCNSTNRVELLAKKITELGYSCFYSHARMLQ

ANRNRVFHDFRNGVCRNLVCSDLLTRGIDIQAVNVVINFDFPKNAETYLHRIGRSGRFGHLGLAINLINWDDRFNLYNIE

KELGTEIQPIPAMIDKNLYVYDSPESIPRPISNPAPRQQAPREQLQIQQRGQAPANYSNGRQGPSGQAQPQSQAQAQAQA

QAQAQAQAQAQLQQGQSQGQRQPSNQPQGGQRNQNFSRGNARVGAQPGRGRGFDGARGGRGQAPPPS

>XP_008720081.1 ATP-dependent RNA helicase dhh1 [Cyphellophora europaea CBS 101466]

MADQLAAQLDKTTIKEGQDDASWKTSLKAPVKDGREQTEDVTATKGLDFEEFYIKRELMMGIFEAGFEKPSPIQEETIPV

ALTGRDVLARAKNGTGKTAAFVIPTLERINPKNPKTQALILVPTRELALQTSQVCKTLGKHLGINVMVTTGGTGLKDDII

RLGDAVHIIVGTPGRILDLSSKGVADLSECSIFVMDEADKLLSPEFTVVIEQLLNFHPKDRQVMLFSATFPIIVKDFKDK

HMRNPYEINLMDELTLRGITQYYAFVEEKQKVHCLNTLFSKLQINQSIIFCNSTNRVELLAKKITELGYSCFYSHARMMQ

HHRNKVFHDFRAGVSRNLVCSDLLTRGIDIQAVNVVINFDFPKNAETYLHRIGRSGRFGHLGLAINLITWEDRLNLYKIE

QELGTEIQPIPPSIDKSLYVYENPENIPRPIHAAPPPRPSQLNGQNGTQQGRRNFNGYNQGYGQRGNYNQGRGGYRGRGA

PRGGRGGQQQIPQNAAPVS

>THV74680.1 DEAD-domain-containing protein [Aureobasidium pullulans]

MKYGNWRSGLKVPAKDGRVQTEDVTATKGLEFEDFYLKRELLMGIFESGFEKPSPIQEEAIPVALTGRDVLARAKNGTGK

TAAFIIPTLERVNPKNSKIQALILVPTRELALQTSQVCKTLGKHLGVNVMVTTGGTGLKDDIIRLNEAVHVLIGTPGRIL

DLASRGVADLSTAQTFVMDEADKLLSPEFTSTIDQLLSFHPPDRQVMLFSATFPVIVKEFKDKHMKDPHEINLMDELTLR

GITQYYAFVEEKQKVHCLNTLFSRLQINQSIIFCNSTTRVELLAKKVTELGYSCFYSHARMLQQNRNRVFHDFRAGHCRN

LVCSDLLTRGIDIQAVNVVINFDFPKNAETYLHRIGRSGRFGHLGLAINLINWEDRFNLYRIEQELGTEIKPIPQHIDKG

LYVYDTPENIPRPITSAPQLQQQPQERRQHHQQGYRGGRGGNYQGQQGQRRGPPRNQYPSQGGQGGGFQQQQAQYNQQAF

PTQQAPQQAPSYPQGGFSQPSAFPTPQSFPQPQGQQPSR

>RMZ85399.1 hypothetical protein DV737_g710, partial [Chaetothyriales sp. CBS 132003]

MMASQLADQLGKTTLTDPSPDSDWKKNLKARPKDEREQTEDVTATKGLDFEDFYIKRGLMMGIFEAGFEKPSPIQEETIP

VALTGRDILARAKNGTGKTAAFVIPTLEKVNPKNPKIQALLLVPTRELALQTSQVCKTLGKHLGLEVMVTTGGTGLKDDI

IRLQSTVHILVGTPGRILDLTSKGVADLSECSIFVMDEADKLLSPEFTVVIEQLLNFHPKDRQVMLFSATFPLVVKDFKD

KHMRDPYEINLMDELTLRGITQYYAFVEEKQKVHCLNTLFSKLQINQSIIFCNSTNRVELLAKKITELGYSCFYSHARMI

QQHRNKVFHDFRAGVSRNLVCSDLLTRGIDIQAVNVVINFDFPKNAETYLHRIGRSGRFGHLGLAINLITWEDRLNLYRI

EQELGTEIQPIPQSIDKSLYVYENPENIPRPLPTAQPTALAGGQNDPGRKPNGYGQHVRGHFNRGTGGPRGRGRGQGRGG

RGGGQFVQQFQNGVPAQ

>TFY68713.1 hypothetical protein EVG20_g3451 [Dentipellis fragilis]

MRAQRSLKRSVRNPPPNFEFVVVAVLYLSTLPSASAPSSKQAFHPLFACPAIFTPPLTYSRPPQIEFATQRVLAFTKANR

ITSLHLVAFSRPPCTPNTPVPTPSHPRRPAFHDVAINSPLLILFVSLALDFDNLFSHLCCPFVASHNGNWRAGLRPPPRD

ERPKTEDVKNIKGLEFEDMYLRRELLMGIFEAGFETPSPIQEEAIPIALTKRDILARAKNGTGKTAAFVIPSLQQVDVSK

NKIQALLLVPTRELALQTSQVCKTLGKHMGVQVMVTTGGTTLKDDILRLSEEVHVLVGTPGRILDLAGKNVADLSECPVF

VMDEADKLLSPEFSPVMEQLLSYLPKDRQVMLFSATFPLIVKDFKNKHMNSPYEINLMDELTLRGVTQYYAYVEERQKVH

CLNTLFSKLQINQSIIFCNSTNRVELLAKKVTELGYSCFYSHAKMLQSHRNRVFHDFRNGVCRNLVCSDLLTRGIDIQAV

NVVINFDFPKNSETYLHRIGRSGRFGHLGLAINLVTYEDRFNLYKIEQELGTEILPIPQQIDKGLYVAPSAQDEPAPQKP

ARTQDQQAAQRQQQQQTAAQVQGPSRPPAQQVVYQSNGQPGRPNGAPAAVQGQARSYHAGPAGAYQGQVPVAQR

>XP_018692009.1 ATP-dependent RNA helicase dhh1 [Fonsecaea erecta]

MTDALAAQLNKASLNDGADESNWKDSLNLPAKDTRQQTEDVTATKGLDFEDFYIKRELMMGIFEAGFEKPSPIQEETIPV

ALTGRDILARAKNGTGKTAAFVIPTLERINPKNPKTQALILVPTRELALQTSQVCKTLGKHLGINVMVTTGGTGLKDDII

RLGETVHIIVGTPGRILDLASKGVADLSECPIFVMDEADKLLSPEFTVVIEQLLSFHPKDRQVMLFSATFPMIVKSFKDK

HMRNPYEINLMDELTLRGITQYYAFVEEKQKVHCLNTLFSKLQINQSIIFCNSTNRVELLAKKITELGYSCFYSHAKMLQ

QNRNKVFHDFRAGVCRNLVCSDLLTRGIDIQAVNVVINFDFPKNAETYLHRIGRSGRFGHRGLAINLINWDDRFNLYKIE

QELGTEIQPIPPSIDKSLYVYDNPETIPRAMPPPAQPPKTNPSNVNAAANSTANNNATHTNGQYQQVRRPQNQSNGYGQQ

YNNQGRGGYRGRGRGQGGRGRGGHPQQPTAAPVGQIAH

>XP_033386789.1 ATP-dependent RNA helicase dhh1 [Aaosphaeria arxii CBS 175.79]

MTSEITNQLAATKLSEPSASADWKSNLKLPAKDTREQTEDVTATKGLEFEDFFIKRELMMGIFEAGFEKPSPIQEETIPV

ALTGRDILARAKNGTGKTAAFIIPTLERVNPKNNKTQALILVPTRELALQTSQVCKTLGKHLGLNVMVSTGGTGLKDDII

RLSDPVHIIVGTPGRILDLAGKGVADLSSCQTFVMDEADKLLSPEFTPVVEQLLAHHPKDRQVMLFSATFPIVVKSFKEK

HMNSPYEINLMDELTLRGITQYYAFVEEKQKVHCLNTLFNKLQINQSIIFCNSTNRVELLAKKITELGYSCFYSHARMLQ

HNRNRVFHDFRNGVCRNLVCSDLLTRGIDIQAVNVVINFDFPKNAETYLHRIGRSGRFGHLGLAINLINWDDRFNLYRIE

QELGTEIQPIPQVIEKNLYVYDSPENIPRPVSNTQRPPQGQIQDQDGANAARAGPGQNNRQNFNRPQRGGAQFQQGQRRG

PPQNQQPRGQNGQQPPRNNRPQPAGPA

>OBT72067.1 ATP-dependent RNA helicase DDX6/DHH1 [Pseudogymnoascus sp. 05NY08]

MADALAGKLESTTLGSSQNDPDWKKKLKIPAKDNRHQTEDVTATKGLEFEEFSIKRDLLMGIFEAGFEKPSPIQEEAIPV

ALTGRDILARAKNGTGKTAAFVIPALERINPKSSKIQCLILVPTRELALQTSQVCKTLGKHLGVNVMVTTGGTGLRDDIV

RLAEPVHIVVGTPGRILDLAGKSVADLSECPMFVMDEADKLLSPEFTPVIEQLLQFHPKDRQIMLFSATFPRSVQAFSVK

NMDQPYEINLMDELTLRGITQYYAFVEEKQKVHCLNTLFSKLQINQSIIFCNSTNRVELLAKKITELGYSCFYSHARMLQ

ANRNRVFHDFRNGVCRNLVCSDLLTRGIDIQAVNVVINFDFPKNAETYLHRIGRSGRFGHLGLAINLINWDDRFNLYNIE

KELGTEIQPIPAMIDKNLYVYDSPESIPRPISNPAPRQQPPREQLQIQQRSGQAPANYSNGRQGPSGQAQPQGQAQAQPQ

PQLQQGQSQGQRQPTNQPQGQRNQNFSRGNARVGAQPGRGRGFDGARGGRGQAPPPS

>XP_013278776.1 ATP-dependent RNA helicase dhh1 [Fonsecaea pedrosoi CBS 271.37]

MTDALAAQLNKASLNDGADESNWKDSLNLPAKDTRQQTEDVTATKGLDFEDFYIKRELMMGIFEAGFEKPSPIQEETIPV

ALTGRDILARAKNGTGKTAAFVIPTLERINPKNPKTQALILVPTRELALQTSQVCKTLGKHLGINVMVTTGGTGLKDDII

RLGETVHIIVGTPGRILDLASKGVADLSECPIFVMDEADKLLSPEFTVVIEQLLSFHPKDRQVMLFSATFPMIVKAFKDK

HMRNPYEINLMDELTLRGITQYYAFVEEKQKVHCLNTLFSKLQINQSIIFCNSTNRVELLAKKITELGYSCFYSHAKMLQ

QNRNKVFHDFRAGVCRNLVCSDLLTRGIDIQAVNVVINFDFPKNAETYLHRIGRSGRFGHRGLAINLINWDDRFNLYKIE

QELGTEIQPIPPSIDKSLYVYENPESIPRAMPPPAQTQKANTSNANANANNTNNNATNSNGQYQQARRPQNQSNGYGQQY

NNQGRGGYRGRGRGQGARGRGGHPQQSTAAPVGQIAH

>OBT83580.1 ATP-dependent RNA helicase DDX6/DHH1 [Pseudogymnoascus sp. 03VT05]

MADALAGKLESTTLGSSQNDPDWKKKLKIPAKDNRHQTEDVTATKGLEFEEFSIKRDLLMGIFEAGFEKPSPIQEEAIPV

ALTGRDILARAKNGTGKTAAFVIPALERINPKSSKIQCLILVPTRELALQTSQVCKTLGKHLGVNVMVTTGGTGLRDDIV

RLAEPVHIVVGTPGRILDLAGKSVADLSECPMFVMDEADKLLSPEFTPVIEQLLQFHPKDRQIMLFSATFPRSVQAFSVK

NMDQPYEINLMDELTLRGITQYYAFVEEKQKVHCLNTLFSKLQINQSIIFCNSTNRVELLAKKITELGYSCFYSHARMLQ

ANRNRVFHDFRNGVCRNLVCSDLLTRGIDIQAVNVVINFDFPKNAETYLHRIGRSGRFGHLGLAINLINWDDRFNLYNIE

KELGTEIQPIPAMIDKNLYVYDSPESIPRPISNPAPRQQPPREQLQIQQRSGQAPPNYSNGRQGPSGQAQPQGQAQAQAQ

PQLQQGQSQGQRQPTNQPQGQRNQNFSRGNARVGAQPGRGRGFDGARGGRGQAPPPS

>XP_025580635.1 DEAD-domain-containing protein [Aspergillus ibericus CBS 121593]

MTDALASQLNNTTIGDASSDTKWKEQLNVPAKDARPQTEDVTATKGLEFEDFYIKRELMMGIFEAGFEKPSPIQEETIPV

ALTGRDILARAKNGTGKTAAFVIPTLERINPKSTKTQALILVPTRELALQTSHVCKTLGKHLGINVMVTTGGTGLMDDII

RLNDAVHILVGTPGRVLDLASKGVADLSECPTFVMDEADKLLSPEFTPVIEQLLSFHPKDRQVMLFSATFPLIVKSFKDK

HMRNPYEINLMDELTLRGITQYYAFVEEKQKVHCLNTLFSKLQINQSIIFCNSTNRVELLAKKITELGYSCFYSHARMLQ

QHRNRVFHDFRNGVCRNLVCSDLLTRGIDIQAVNVVINFDFPKNAETYLHRIGRSGRFGHLGLAINLINWEDRFNLYKIE

QELGTEIQPIPQNIDKKLYVYDSPDTIPRPIANPSQPPITTTAVNTNAGERRHNHHMNGGSYQYGRGRGSYRGGRGQGQR

RSMQNETKFSTQGQSSGKSHPAQVS

>SMN18278.1 similar to Saccharomyces cerevisiae YDL160C DHH1 Cytoplasmic DExD/H-box helicase [Kazachstania saulgeensis]

MTSTSMDNEDWKSKLNIPQRDTRPQTEDVLGTKGNTFEDFGLKRELLMGIFEAGFEKPSPIQEESIPVAITGRDILARAK

NGTGKTAAFVIPTLEQIKPKVNKIQALIMVPTRELALQTSQVVRTLGKHCNISCMVTTGGTNLRDDILRLNDIVHVVVGT

PGRILDLASRKIADLSNCSLFVMDEADKMLSRDFKTIIEQILIFLPKNHQSLLFSATFPLSVKEFMVKHLNKPYEINLME

ELTLKGITQYYAFVEERQKLHCLNTLFSKLQINQAIIFCNSTNRVELLAKKITDLGYSCYYSHARMKQQERNKVFHEFRQ

GKVRTLVCSDLLTRGIDIQAVNVVINFDFPKTAETYLHRIGRSGRFGHLGLAINLINWNDRFNLYKIEQELGTEIAAIPA

TIDKSLYVAENNDNVPIPVPIEQQSFHNNNNNIVIPPQQQQFAHFNNNQLPPQQYPQQHQQQHHQQPQPQQQQQQQGIPP

QQFQPQFHAQQYPPQMQMQNQGNYPPGVGPQY

>KLJ13226.1 ATP-dependent RNA helicase DHH1 [Blastomyces silverae]

MADALASQLNSTKLGDTHSEKWRENLKAPPKDTRTQTEDVTATKGLEFEDFYIKRELMMGIFEAGFEKPSPIQEETIPVA

LTGRDILARAKNGTGKTAAFVIPTLERTNPKNSKTQALILVPTRELALQTSQVCKTLGKHLGINVMVTTGGTGLQDDIIR

LNEAVHIIVGTPGRILDLASKGVADLSECSTFVMDEADKLLSPEFTPVIEQLLSFHPKDRQVMLFSATFPMIVKSFKDKH

MRNPYEINLMDELTLRGITQYYAFVEEKQKVHCLNTLFSKLQINQSIIFCNSTNRVELLAKKITELGYSCFYSHAKMLQH

NRNRVFHDFRNGVCRNLVCSDLLTRGIDIQAVNVVINFDFPKNAETYLHRIGRSGRFGHLGLAINLINWEDRYNLYKIEQ

ELGTEIQPIPQSIDKKLYVYDTPDTIPRPISNAPQHHHQQPASTIAQPPNNPEGHNRRQGQHQYSSNRGRGSYRGGRGQG

HRGGHGENHRQNGTAPRPNAAASAPTS

>OCT47697.1 ATP-dependent RNA helicase DHH1 [Cladophialophora carrionii]

MTDALAAQLNKTSLNDGAEDPNWKDSLKLPAKDTRQQTEDVTATKGLEFEDFYIKRELMMGIFEAGFEKPSPIQEETIPV

ALTGRDILARAKNGTGKTAAFVIPTLERINPKNPKTQALILVPTRELALQTSQVCKTLGKHLGINVMVTTGGTGLKDDIL

RLGETVHIIVGTPGRILDLASKGVADLSECPIFVMDEADKLLSPEFTVVIEQLLSFHPKDRQVMLFSATFPMIVKSFKDK

HMRNPYEINLMDELTLRGITQYYAFVEEKQKVHCLNTLFSKLQINQSIIFCNSTNRVELLAKKITELGYSCFYSHAKMLQ

QNRNKVFHDFRAGVSRNLVCSDLLTRGIDIQAVNVVINFDFPKNAETYLHRIGRSGRFGHRGLAINLINWDDRYNLYKIE

QELGTEIQPIPPSIDKSLYVYDNPENIPRALPPPAPAAKPNTSTAPANQHATNANGQYPQFRKSQNQFNGFPQQYNNNNN

QGRGGYRGRGRGQGARGRGGHPQQAAAVGQTAQ

>EEH07818.1 ATP-dependent RNA helicase DHH1 [Histoplasma capsulatum G186AR]

MADALASQLNSTKLGESHSEKWRENLKVPPKDTRTQTEDVTATKGLEFEDFYIKRELMMGIFEAGFEKPSPIQEETIPVA

LTGRDILARAKNGTGKTAAFVIPTLERTNPKNSKTQALILVPTRELALQTSQVCKTLGKHLGINVMVTTGGTGLQDDIIR

LNEAVHIIVGTPGRILDLASKGVADLSDCSTFVMDEADKLLSPEFTPVIEQLLSFHPKDRQVMLFSATFPLIVKSFKDKH

MRNPYEINLMDELTLRGITQYYAFVEEKQKVHCLNTLFSKLQINQSIIFCNSTNRVELLAKKITELGYSCFYSHAKMLQH

NRNRVFHDFRNGVCRNLVCSDLLTRGIDIQAVNVVINFDFPKNAETYLHRIGRSGRFGHLGLAINLINWEDRYNLYKIEQ

ELGTEIQPIPQSIDKKLYVYDTPDTIPRPTSNVGHQQQQQQQHQHTSEPPQHPNNPGNHNRRHGQHQYASNRGRGSYRGG

RGQGHRGGYGENHRQNCTAPRPSAAASAPAS

>XP_003680310.1 hypothetical protein TDEL_0C02100 [Torulaspora delbrueckii]

MSDDQSWKAQLNLPKRDTRPQTDDVLNTKGNSFEDFYLKRELLMGIFEAGFEKPSPIQEESIPVAIAGRDILARAKNGTG

KTAAFVIPALEKVKPKLNKIQALIMVPTRELALQTSQVVRTLGKHCGVSCMVTTGGTNLKDDIMRLNETVHILVGTPGRV

LDLASRKLADLSECSLFIMDEADKMLSRDFKTIIEQILIFLPQSHQSLLFSATFPLTVKEFMVKHLHKPYEINLMEELTL

KGITQYYAFVEERQKLHCLNTLFSKLQINQAIIFCNSTNRVELLAKKITDLGYSCYYSHARMRQQERNRVFHEFRQGKVR

TLVCSDLLTRGIDIQAVNVVINFDFPKTAETYLHRIGRSGRFGHLGLAINLINWNDRFNLYKIEQELGTEIAAIPATIDK

SLYVADDASAVPVPFPIQQQHQQSQQQVHQQQYMPPQQQQQAMPPQHGYPMQGIPQQGIPPQGIPPPQMQAHFAAQPQY

>RDW73806.1 hypothetical protein BP5796_07248 [Coleophoma crateriformis]

MAEALANQLESTKLSDGASPDDWKSGLKIPAKDTRQQTEDVTATKGLEFEDFSIKRDLLMGIFEAGFEKPSPIQEEAIPV

ALTGRDILARAKNGTGKTAAFTIPALERINPKSSKIQALILVPTRELALQTSQVCKTLGKHLGINVMVTTGGTTLRDDIV

RLQDPVHVIVGTPGRILDLAGKNVADLSECPMFIMDEADKLLSPEFTPVIEQLLQFHPKDRQVMLFSATFPITVKNFSDK

NMTDPYEINLMDELTLRGITQYYAFVEEKSKVHCLNTLFSKLQINQSIIFCNSTNRVELLAKKITELGYSCFYSHARMLQ

ANRNRVFHDFRNGVCRNLVCSDLLTRGIDIQAVNVVINFDFPKNAETYLHRIGRSGRFGHLGLAINLINWDDRFNLYNIE

RDLGTEIQPIPQTIDKNLYVYDSPENIPRPVSNFQTNRQSADREQQRRLEQQPSGQRGGGQANVANNGRSNGANREQGQR

QPQGQRQGQGFGQRGGGRGGRHYDGQRGGRGQGMPAQ

>KAF2685503.1 ATP-dependent RNA helicase-like protein dhh1 [Lentithecium fluviatile CBS 122367]

MTSEITNQLAATKLSDASPSGDANWKDGLKIPSKDTRVQTEDVTATKGLEFEDFFIKRELMMGIFEAGFEKPSPIQEETI

PVALTGRDILARAKNGTGKTAAFVIPTLERVNPKTPKTQALILVPTRELALQTSQVCKMLGKHLGINVMVSTGGTGLKDD

IIRLSEPVHIIVGTPGRILDLAGKGVADLSACQTFVMDEADKLLSPEFTPVIEQLLGFHPKDRQVMLFSATFPIVVKSFK

DKHMNQPYEINLMDELTLRGITQYYAFVEEKQKVHCLNTLFNKLQINQSIIFCNSTNRVELLAKKITELGYSCFYSHARM

LQHNRNRVFHDFRNGVCRNLVCSDLLTRGIDIQAVNVVINFDFPKNAETYLHRIGRSGRFGHLGLAINLINWEDRFNLYR

IEQELGTEIQPIPQVIEKKLYVYETPENIPRPISNPAQGQEQGQGQDGSVQRAHSGQNYRGNYNNRRGSGGQYQGPRRGG

APQGQQQNRQNGGNPQRNHRPQPQPANPV

>XP_002789230.1 ATP-dependent RNA helicase DHH1 [Paracoccidioides lutzii Pb01]

MADALASQLSSVKLGENHHSEKWKEHLKAPPKDTRTQTEDVTATKGLEFEDFYIKRELMMGIFEAGFEKPSPIQEETIPV

ALTGRDILARAKNGTGKTAAFVIPTLERTNPKNSKTQALILVPTRELALQTSQVCKTLGKHLGINVMVTTGGTGLQDDII

RLNEAVHIIVGTPGRILDLASKGVADLSECSTFVMDEADKLLSPEFTPVIEQLLSFHPKDRQIMLFSATFPLIVKSFKDK

HMRNPYEINLMDELTLRGITQYYAFVEEKQKVHCLNTLFSKLQINQSIIFCNSTNRVELLAKKITELGYSCFYSHAKMLQ

HNRNRVFHDFRNGVCRNLVCSDLLTRGIDIQAVNVVINFDFPKNAETYLHRIGRSGRFGHLGLAINLINWEDRYNLYKIE

QELGTEIQPIPQSIDKKLYVYDTPDTIPRPITSTPTQPPKQQHQQQPQQPSAPAQYPNNPEGHSRRQGHSQYPSHRGRGS

YRGGRGQGHRGGHAENHRQNGTGPRPNAAASAPAS

>KFY49563.1 hypothetical protein V496_09912 [Pseudogymnoascus sp. VKM F-4515 (FW-2607)]

MADALAGKLESTTLGSTPNDADWKKKLKIPAKDNRHQTEDVTATKGLEFEEFSIKRDLLMGIFEAGFEKPSPIQEEAIPV

ALTGRDILARAKNGTGKTAAFVIPALERINPKSSKIQCLILVPTRELALQTSQVCKTLGKHLGVNVMVTTGGTGLRDDIV

RLAEPVHIVVGTPGRILDLAGKNVADLSECPMFIMDEADKLLSPEFTPVIEQLLQFHPKDRQIMLFSATFPRSVQAFSVK

NMDQPYEINLMDELTLRGITQYYAFVEEKQKVHCLNTLFSKLQINQSIIFCNSTNRVELLAKKITELGYSCFYSHARMLQ

ANRNRVFHDFRNGVCRNLVCSDLLTRGIDIQAVNVVINFDFPKNAETYLHRIGRSGRFGHLGLAINLINWDDRFNLYNIE

KELGTEIQPIPAMIDKNLYVYDSPESIPRPISNPAPRQQAPREQLQIQQRGQAPQNYSNGRQGPSGQAQSQNQAQAQAQA

QAQAQAQAQAQAQAQAQAQAQAQLQQGQSQGQRQPSNQPQGQQRSQNFSRGNARVGAQPGRGRGFDGARGGRGQAPPPS

>PVU87756.1 hypothetical protein BB559_005900 [Furculomyces boomerangus]

MSEVKTKSSSNNQQDWKNQLSLPAKDTRVQTEDVTATKGNEFEDYYLKRELLMGIFEAGFEKPSPIQEESIPIALTGRDI

LARAKNGTGKTAAFIIPALEKINTGSPDVQCLILVPTRELALQTSQVCKTIGKHMNINVMVTTGGTNLKDDIMRLSENVH

IIVGTPGRVLDLANKNVINFSKAETFIMDEADKLLSPEFTPVIEQLLGFFPKKRQVLLYSATFPLVVKSFKDKHMVKPYE

INLMDELTLKGVTQYYAYVEEKQKVHCLNTLFSKLQINQSIIFCNSTNRVELLARKITELGYSCFYSHARMLQHHRNRVF

HDFRNGSCRNLVCSDLLTRGIDIQAVNVVINFDFPRNAETYLHRIGRSGRFGHLGIAINLITNEDRFNLYKIEQELGTEI

LPIPPVIDKRLNINDHNSYNKNQPPPGLNRPRNQNNNTNEGPPGYHGNQRRNNQRQGGYNQNGENYSQQTGK

>OQV03430.1 hypothetical protein CLAIMM_08476 [Cladophialophora immunda]

MTDALAAQLNKASLNDGADESNWKDALNLPAKDTRQQTEDVTATKGLDFEDFYIKRELMMGIFEAGFEKPSPIQEETIPV

ALTGRDILARAKNGTGKTAAFVIPTLERINPKNPKTQALILVPTRELALQTSQVCKTLGKHLGINVMVTTGGTGLKDDII

RLGETVHIIVGTPGRILDLASKGVADLSECPIFVMDEADKLLSPEFTVVIEQLLSFHPKDRQVMLFSATFPMIVKAFKDK

HMRNPYEINLMDELTLRGITQYYAFVEEKQKVHCLNTLFSKLQINQSIIFCNSTNRVELLAKKITELGYSCFYSHAKMLQ

QNRNKVFHDFRAGVCRNLVCSDLLTRGIDIQAVNVVINFDFPKNAETYLHRIGRSGRFGHRGLAINLINWDDRFNLYKIE

QELGTEIQPIPPSIDKSLYVYDNPETIPRAMPPPAQAQKAHASNANATANTTANNNATNTNGQYQQARRPQNQSNGYGQQ

YNNQGRGGYRGRGRGQGARGRGGHPQQPTAAPVGQIAH

>KFZ09561.1 hypothetical protein V502_08669 [Pseudogymnoascus sp. VKM F-4520 (FW-2644)]

MADALAGKLESTTLGSSQNDPDWKKKLKIPAKDNRHQTEDVTATKGLEFEEFSIKRDLLMGIFEAGFEKPSPIQEEAIPV

ALTGRDILARAKNGTGKTAAFVIPALERINPKSSKIQCLILVPTRELALQTSQVCKTLGKHLGVNVMVTTGGTGLRDDIV

RLAEPVHIVVGTPGRILDLAGKSVADLSECPMFIMDEADKLLSPEFTPVIEQLLQFHPKDRQIMLFSATFPRSVQAFSVK

NMDQPYEINLMDELTLRGITQYYAFVEEKQKVHCLNTLFSKLQINQSIIFCNSTNRVELLAKKITELGYSCFYSHARMLQ

ANRNRVFHDFRNGVCRNLVCSDLLTRGIDIQAVNVVINFDFPKNAETYLHRIGRSGRFGHLGLAINLINWDDRFNLYNIE

KELGTEIQPIPAMIDKNLYVYDSPESIPRPISNPAPRQQAPREQLQIQQRGQAPANYSNGRQGPSGQAQPQSQAQAQAQA

QAQAQAQAQAQAQLQQGQSQGQRQPSNQPQGGQRNQNFSRGNARVGAQPGRGRGFDGARGGRGQAPPPS

>XP_024322528.1 DExD/H-box ATP-dependent RNA helicase dhh1 [Pseudogymnoascus destructans]

MADALAGKLESTTLGSSQNDPDWKKKLKIPAKDNRQQTEDVTATKGLEFEEFSIKRDLLMGIFEAGFEKPSPIQEEAIPV

ALTGRDILARAKNGTGKTAAFVIPALERINPKSSKIQCLILVPTRELALQTSQVCKTLGKHLGVNVMVTTGGTGLRDDIV

RLAEPVHIVVGTPGRILDLAGKSVADLSECPMFIMDEADKLLSPEFTPVIEQLLQFHPKDRQIMLFSATFPRSVQAFSVK

NMDQPYEINLMDELTLRGITQYYAFVEEKQKVHCLNTLFSKLQINQSIIFCNSTNRVELLAKKITELGYSCFYSHARMLQ

ANRNRVFHDFRNGVCRNLVCSDLLTRGIDIQAVNVVINFDFPKNAETYLHRIGRSGRFGHLGLAINLINWDDRFNLYNIE

KELGTEIQPIPAMIDKNLYVYDSPESIPRPISNPAPRQQPPREQLQIQQRGGQALANYSNGRQGPPGEAQPQAQAQAQAQ

LQQGQSQGQRQPSNQPQGQQRNQNFSRGNARVGAQPGRGRGFDGARGGRGQAPPPS

>XP_016248542.1 ATP-dependent RNA helicase dhh1 [Cladophialophora immunda]

MTDALAAQLNKASLNDGADESNWKDALNLPAKDTRQQTEDVTATKGLDFEDFYIKRELMMGIFEAGFEKPSPIQEETIPV

ALTGRDILARAKNGTGKTAAFVIPTLERINPKNPKTQALILVPTRELALQTSQVCKTLGKHLGINVMVTTGGTGLKDDII

RLGETVHIIVGTPGRILDLASKGVADLSECPIFVMDEADKLLSPEFTVVIEQLLSFHPKDRQVMLFSATFPMIVKAFKDK

HMRNPYEINLMDELTLRGITQYYAFVEEKQKVHCLNTLFSKLQINQSIIFCNSTNRVELLAKKITELGYSCFYSHAKMLQ

QNRNKVFHDFRAGVCRNLVCSDLLTRGIDIQAVNVVINFDFPKNAETYLHRIGRSGRFGHRGLAINLINWDDRFNLYKIE

QELGTEIQPIPPSIDKSLYVYDNPETIPRAMPPPAQAQKAHASNVNATANTTANNNATNTNGQYQQARRPQNQSNGYGQQ

YNNQGRGGYRGRGRGQGARGRGGHPQQLTAAPVGQIAH

>XP_033588440.1 P-loop containing nucleoside triphosphate hydrolase protein [Neohortaea acidophila]

MASELANKLEATSLTDNQTNNDWKAGLKQPAKDGRVQTEDVTATKGLEFEDFYLKRELLMGIFEAGFEKPSPIQEETIPV

ALTGRDVLARAKNGTGKTAAFVIPTLERINPKLDKTQALLLVPTRELALQTSQVCKTLGKHLGINVMVTTGGTGLKDDIM

RLNETVHIIVGTPGRILDLASKGVADLSAAQTFVMDEADKLLSPEFTVTIEQLLQFHPKDRQVMLFSATFPIVVKSFKDK

HMRDPHEINLMDELTLRGITQYYAFVEEKQKVHCLNTLFSRLQINQSIIFCNSTTRVELLAKKITELGYSCFYSHAKMLQ

QHRNRVFHDFRNGAMRNLVCSDLLTRGIDIQAVNVVINFDFPKNAETYLHRIGRSGRFGHLGLAINLINWEDRFNLYRIE

QELGTEIQPIPSSIDKKLYVYDAPENIPRPVNTAPQRGPPQPPGRQQGNGEDLQNPANRTGGFRGGRGGGRGGGGFRGPP

RDPNQQQPQHSNGFQGNQRQGGPWQQRPPQQQQQGPPPAAHA

>PYI10495.1 DEAD-domain-containing protein [Aspergillus sclerotiicarbonarius CBS 121057]

MADALASQLNNTTLGDASSDAKWKEQLNVPAKDARPQTEDVTATKGLEFEDFYIKRELMMGIFEAGFEKPSPIQEETIPV

ALTGRDILARAKNGTGKTAAFVIPTLERINPKSTKTQALILVPTRELALQTSHVCKTLGKHLGINVMVTTGGTGLMDDII

RLNDAVHILVGTPGRVLDLASKGVADLSECPTFVMDEADKLLSPEFTPVIEQLLSFHPKDRQVMLFSATFPLIVKSFKDK

HMRNPYEINLMDELTLRGITQYYAFVEEKQKVHCLNTLFSKLQINQSIIFCNSTNRVELLAKKITELGYSCFYSHARMLQ

QHRNRVFHDFRNGVCRNLVCSDLLTRGIDIQAVNVVINFDFPKNAETYLHRIGRSGRFGHLGLAINLINWEDRFNLYKIE

QELGTEIQPIPQNIDKKLYVYDSPDTIPRPIANPSQPPITTTAVNSNTGERRHNHHMNGGQYQYGRGRGSYRGGRGQGQR

RSMQNEAKFGTQGQSSGKSHPAQVS

>PGH31112.1 ATP-dependent RNA helicase dhh1 [Emmonsia crescens]

MADALASQLNSTKLGENHSEKWRENLKAPPKDTRTQTEDVTATKGLEFEDFYIKRELMMGIFEAGFEKPSPIQEETIPVA

LTGRDILARAKNGTGKTAAFVIPTLERTNPKNSKTQALILVPTRELALQTSQVCKTLGKHLGVNVMVTTGGTGLQDDIIR

LNEAVHIIVGTPGRILDLASKGVADLSECSTFVMDEADKLLSPEFTPVIEQLLSFHPKDRQVMLFSATFPMIVKSFKDKH

MRNPYEINLMDELTLRGITQYYAFVEEKQKVHCLNTLFSKLQINQSIIFCNSTNRVELLAKKITELGYSCFYSHAKMLQH

NRNRVFHDFRNGVCRNLVCSDLLTRGIDIQAVNVVINFDFPKNAETYLHRIGRSGRFGHLGLAINLINWEDRYNLYKIEQ

ELGTEIQPIPQSIDKKLYVYDTPDTIPRPISNAPPHQHQQQQQQASAPAQHPNNPDSHNRRQGQHQYSSNRGRGSYRGGR

GQGHRGGHGENHRQNGTAPRPNAAASAPAS

>XP_028488471.1 ATP-dependent RNA helicase DHH1 [Byssochlamys spectabilis]

MDSAWREKLNVPPKDNRTQTEDVTATKGLEFEDFYIKRELMMGIFEAGFEKPSPIQEETIPVALTGRDILARAKNGTGKT

AAFVIPTLERINPKSTKTQALILVPTRELALQTSQVCKTLGKHLGVNVMVTTGGTGLMDDIIRLNDPVHIIVGTPGRVLD

LASKGVADLSECHTFVMDEADKLLSPEFTPVIEQLLSFHPKDRQIMLFSATFPLIVKSFKDKHMRNPYEINLMDELTLRG

ITQYYAFVEEKQKVHCLNTLFSKLQINQSIIFCNSTNRVELLAKKITELGYSCFYSHARMLQHNRNRVFHDFRNGVCRNL

VCSDLLTRGIDIQAVNVVINFDFPKNAETYLHRIGRSGRFGHLGLAINLINWDDRFNLYKIEQELGTEIQPIPQSIDKKL

YVYESPDTIPRPISSLPQNQPRQLTQGQPGSNGEPHQRRNNHHTNGGHYNSNRGRGSYRGGRGQGQRRGGQYDGNKSGVP

PQAGGKSQPLPAS

>XP_008727205.1 ATP-dependent RNA helicase DHH1 [Cladophialophora carrionii CBS 160.54]

MTDALASQLNKTSLNDGAEDPNWKDSLKLPAKDTRQQTEDVTATKGLEFEDFYIKRELMMGIFEAGFEKPSPIQEETIPV

ALTGRDILARAKNGTGKTAAFVIPTLERINPKNPKTQALILVPTRELALQTSQVCKTLGKHLGINVMVTTGGTGLKDDIL

RLGETVHIIVGTPGRILDLASKGVADLSECPIFVMDEADKLLSPEFTVVIEQLLSFHPKDRQVMLFSATFPMIVKAFKDK

HMRNPYEINLMDELTLRGITQYYAFVEEKQKVHCLNTLFSKLQINQSIIFCNSTNRVELLAKKITELGYSCFYSHAKMLQ

QNRNKVFHDFRAGVSRNLVCSDLLTRGIDIQAVNVVINFDFPKNAETYLHRIGRSGRFGHRGLAINLINWDDRYNLYKIE

QELGTEIQPIPPSIDKSLYVYDNPENIPRALPPPAPAAKPNTSTAPTNQHATNANGQYPQFRKSQNQFNGFPQQYNNNNN

QGRGGYRGRGRGQGARGRGGHPQQAAAVGQTAQ

>XP_002152684.1 DEAD-box RNA helicase Dhh1/Vad1, putative [Talaromyces marneffei ATCC 18224]

MVDAVTSQLNNTKLGETDGSWKDKLKIPAKDNRTQTEDVTATKGLEFEDFYIKRELMMGIFEAGFEKPSPIQEETIPVAL

TGRDILARAKNGTGKTAAFIIPTLERINPKSTKTQALILVPTRELALQTSQVCKTLGKHLGINVMVTTGGTGLMDDIIRL

NDTVHIIVGTPGRVLDLASKGVADLSECPTFVMDEADKLLSPEFTPVIEQLLSFHPKDRQVMLFSATFPMIVKSFKDKHM

RNPYEINLMDELTLRGITQYYAFVEEKQKVHCLNTLFSKLQINQSIIFCNSTNRVELLAKKITELGYSCFYSHARMLQHN

RNRVFHDFRNGVCRNLVCSDLLTRGIDIQAVNVVINFDFPKNAETYLHRIGRSGRFGHLGLAINLINWDDRFNLYKIEQE

LGTEIQPIPQSIDKKLYVYDSPDTIPRPIASAPQPDETQQREQQGYRQGNHHPGGRYNNNAGRGRGGYRGRGGQGQRRGG

YQNPHGHDGGKPQPTPAS

>XP_027614718.1 predicted protein [Sparassis crispa]

MSQPARPSSSTSDAWKASLRPPPRDDRPQTEDVTATKGMDFEDMFLRRELLMGIFEAGFEKPSPIQEEAIPIALAKRDVL

ARAKNGTGKTAAFVIPSLQQIDVNKNKIQALLLVPTRELALQTAQVCKILGKHMGVQVMVTTGGTTLKDDILRLSEVVHV

LVGTPGRILDLAGKNVADLSECPIFVMDEADKLLSPEFAPVMEQLLSYMAKDRQVMLFSATFPMIVKDFKDKHMRSPYEI

NLMDELTLRGVTQYYAYVEERQKVHCLNTLFSKLQINQSIIFCNSTNRVELLAKKVTELGYSCFYSHAKMLQSHRNRVFH

DFRNGVCRNLVCSDLLTRGIDIQAVNVVINFDFPKNAETYLHRIGRSGRYGHLGLAINLVTYEDRFNLYKIEQELGTEIQ

PIPQAIDKTLYVAPSAIEEPAEQKSARTTQAPVQAQGQSQQKQQAPQQPQSQAQLPAQVVYNSAPLRQNGVAVPQQSRPV

FQTPFRGSVPRVRGITRLLSAGVFTTMSSANYGRLPLHALSRRSSADSLSSSTSPDPSYYPTRRRRWYSSLWNLHRPRTV

LMLFKIFLGCAVLVLLGGYILWEPHIELAFYWRGWLKQEINTVVPLAGCFRPENVSPRYNLSAALHGQKKTEVHAGLPLR

LGVDCYDFAGTIEPEPHDGVVLLPDERVHYHTYWRTDLAPFGERQEWMLKSFFATQNTNTARLVLWSNGDLSDNAILQRW

LRRFPDAFVLQVVNYEDLARGTVLHGSELLRVKDTKAWIDGDLVRLLVLWAYGGVWVDMDSLLTRDLAPLLEHEFVIQWD

CYDKIYVPFNGALMRFHEHSPYLCEAFNIMVNSRPPREGSTDWGALLYLKLWRRLTAESIPPFKVLPWCFTDARSCRFDN

RLPDPFEQDPINGQWTVGYGREEGGGLDRALAKVFSVHLHNQWRKPFPLGGWLDRLLLRRYDARLSTSFGARESEEL

>OJD19859.1 ATP-dependent RNA helicase DHH1 [Emergomyces pasteurianus Ep9510]

MADALASQLNSSKLGENHTEKWRENLKAPPKDTRTQTEDVTATKGLDFEDFYIRRELMMGIFEAGFEKPSPIQEETIPVA

LTGRDILARAKNGTGKTAAFVIPTLERTNPKNSKTQALILVPTRELALQTSQVCKTLGKHLGINVMVTTGGTGLQDDIIR

LNESVHIIVGTPGRILDLASKGVADLSECSTFVMDEADKLLSPEFTPVIEQLLSFHPKDRQVMLFSATFPMIVKSFKDKH

MRNPYEINLMDELTLRGITQYYAFVEEKQKVHCLNTLFSKLQINQSIIFCNSTNRVELLAKKITELGYSCFYSHAKMLQH

NRNRVFHDFRNGVCRNLVCSDLLTRGIDIQAVNVVINFDFPKNAETYLHRIGRSGRFGHLGLAINLINWEDRYNLYKIEQ

ELGTEIQPIPQSIDKKLYVYDTPDTIPRPISNALPRQQQQQQQQQQLQQASTSTNPEGHNRRQGQHQYSSNRGRGSYRGG

RGHGHRGGHGENHSQNGTAPRSNAAASASAS

>KFZ04720.1 hypothetical protein V501_09030 [Pseudogymnoascus sp. VKM F-4519 (FW-2642)]

MADALAGKLESTTLGSSQNDPDWKKKLKIPAKDNRHQTEDVTATKGLEFEEFSIKRDLLMGIFEAGFEKPSPIQEEAIPV

ALTGRDILARAKNGTGKTAAFVIPALERINPKSSKIQCLILVPTRELALQTSQVCKTLGKHLGVNVMVTTGGTGLRDDIV

RLAEPVHIVVGTPGRILDLAGKSVADLSECPMFIMDEADKLLSPEFTPVIEQLLQFHPKDRQIMLFSATFPRSVQAFSVK

NMDQPYEINLMDELTLRGITQYYAFVEEKQKVHCLNTLFSKLQINQSIIFCNSTNRVELLAKKITELGYSCFYSHARMLQ

ANRNRVFHDFRNGVCRNLVCSDLLTRGIDIQAVNVVINFDFPKNAETYLHRIGRSGRFGHLGLAINLINWDDRFNLYNIE

KELGTEIQPIPAMIDKNLYVYDSPESIPRPISNPAPRQQPPREQLQIQQRGGQAPANYTNGRQGPSTQAQPQGQAPAQAQ

AQLQQGQPQGQRQPTNQPQGQQRNQNFSRGNARVGAQPGRGRGFDGARGGRGQAPPPS

>XP_001395078.1 ATP-dependent RNA helicase dhh1 [Aspergillus niger CBS 513.88]

MTDALASQLNNTTLGDASSDAKWKEQLNVPAKDARPQTEDVTATKGLEFEDFYIKRELMMGIFEAGFEKPSPIQEETIPV

ALTGRDILARAKNGTGKTAAFVIPTLERINPKSTKTQALILVPTRELALQTSHVCKTLGKHLGINVMVTTGGTGLMDDII

RLNDAVHILVGTPGRVLDLASKGVADLSECPTFVMDEADKLLSPEFTPVIEQLLSFHPKDRQVMLFSATFPLIVKSFKDK

HMRNPYEINLMDELTLRGITQYYAFVEEKQKVHCLNTLFSKLQINQSIIFCNSTNRVELLAKKITELGYSCFYSHARMLQ

QHRNRVFHDFRNGVCRNLVCSDLLTRGIDIQAVNVVINFDFPKNAETYLHRIGRSGRFGHLGLAINLINWEDRFNLYKIE

QELGTEIQPIPQNIDKKLYVYDSPDTIPRPIANPSQPQITAQAANANIGERRHNHHMNGGQYQYGRGRGSYRGGRGQGQR

RNMQNETKFGTQGQSSGKSHPTQVS

>OAL30963.1 ATP-dependent RNA helicase dhh1 [Fonsecaea multimorphosa]

MTDALAAQLNKASLNDGADESNWKDSLNLPAKDTRHQTEDVTATKGLDFEDFYIKRELMMGIFEAGFEKPSPIQEETIPV

ALTGRDILARAKNGTGKTAAFVIPTLERINPKNPKTQALILVPTRELALQTSQVCKTLGKHLGINVMVTTGGTGLKDDII

RLGETVHIIVGTPGRILDLASKGVADLSECPIFVMDEADKLLSPEFTVVIEQLLSFHPKDRQVMLFSATFPMIVKSFKDK

HMRNPYEINLMDELTLRGITQYYAFVEEKQKVHCLNTLFSKLQINQSIIFCNSTNRVELLAKKITELGYSCFYSHAKMLQ

QNRNKVFHDFRAGVCRNLVCSDLLTRGIDIQAVNVVINFDFPKNAETYLHRIGRSGRFGHRGLAINLINWDDRFNLYKIE

QELGTEIQPIPPSIDKSLYVYDNPELIPRAMPPPAQTARSNASNANATANTSANNNATNTNGQYQQDVGDTVGEAEVKVL

EAAAVILSSQQQHLWVKSLTSLRFGL

>XP_002620701.1 ATP-dependent RNA helicase DHH1 [Blastomyces gilchristii SLH14081]

MADALASQLNSTKLGDNHSEKWRENLKAPPKDTRTQTEDVTATKGLEFEDFYIKRELMMGIFEAGFEKPSPIQEETIPVA

LTGRDILARAKNGTGKTAAFVIPTLERTNPKNSKTQALILVPTRELALQTSQVCKTLGKHLGINVMVTTGGTGLQDDIIR

LNEAVHIIVGTPGRILDLASKGVADLSECSTFVMDEADKLLSPEFTPVIEQLLSFHPKDRQVMLFSATFPMIVKSFKDKH

MRNPYEINLMDELTLRGITQYYAFVEEKQKVHCLNTLFSKLQINQSIIFCNSTNRVELLAKKITELGYSCFYSHAKMLQH

NRNRVFHDFRNGVCRNLVCSDLLTRGIDIQAVNVVINFDFPKNAETYLHRIGRSGRFGHLGLAINLINWEDRYNLYKIEQ

ELGTEIQPIPQSIDKKLYVYDTPDTIPRPISNAPQHHSQQPASAIAQRPNSPEGHNRRQGQHQYSSNRGRGSYRGGRGQG

HRGGHGENHRQNGTAPRPNAAASAPAS

>AQZ12093.1 DHH1 (YDL160C) [Zygosaccharomyces parabailii]

MSKEDWSSQLKVPAKDTRPQTDDVLNTKGNTFEDFCLKRELLMGIFEAGFEKPSPIQEESIPVAIAGRDILARAKNGTGK

TAAFVIPTLEKVKPKINKIQALIMVPTRELALQTSQVVRTLGKHCGISCMVTTGGTNLRDDILRLNETVHVLVGTPGRVL

DLASRKVADLSDCSLFVMDEADKMLSRDFKTIIEQILIFLPPQHQSLLFSATFPITVKEFMVKHLHKPYEINLMEELTLK

GITQYYAFVEERQKLHCLNTLFSKLQINQAIIFCNSTNRVELLAKKITDLGYSCYYSHARMRQQERNKVFHDFRQGKVRT

LVCSDLLTRGIDIQAVNVVINFDFPKTAETYLHRIGRSGRFGHLGLAINLINWNDRFNLYKIEQELGTEIAAIPATIDKS

LYVAEDNSNIPTPYPIQHHQPQSQPKPQPQYASPPQGFTAQGLHQQLSPHVMAPHGLASQNLSPHGLTQQGLPPQGPAQG

LPQGTSPQTLPQGLPQGIMAQGIPPHYAAQPHF

>OCL02082.1 DEAD-domain-containing protein [Glonium stellatum]

MTSDITNQLAATKLSEPSSDASWKDGLKIPSKDARPQTEDVTATKGLEFEDFYIKRELMMGIFEAGFEKPSPIQEETIPV

ALTGRDILARAKNGTGKTAAFVIPTLERINPKSAKTQALILVPTRELALQTSQVCKMLGKHLGINVMVTTGGTGLKDDII

RLSEPVHIIVGTPGRILDLASKGVADLSACATFVMDEADKLLSPEFTPVIEQLLAFHPKDRQVMLFSATFPIVVKSFKDK

HMNSPYEINLMDELTLRGITQYYAFVEEKQKVHCLNTLFNKLQINQSIIFCNSTNRVELLAKKITELGYSCFYSHARMLQ

HNRNRVFHDFRNGVCRNLVCSDLLTRGIDIQAVNVVINFDFPKNAETYLHRIGRSGRFGHLGLAINLINWEDRFNLYRIE

QELGTEIQPIPQVIEKKLYVYESPETIPRPISNNPSQRNQSQAPAQSQAQAQAQVEVQTQDQDNSATVSRSNSSQSNNRQ

NYQRGQRGGGQYQGQRRGQPQHQQNRQNGHNPQRNARPPPAAPA

>KMP02113.1 ATP-dependent RNA helicase dhh1 [Coccidioides immitis RMSCC 2394]

MAEALASQLNKAKLGDNGAETKWKEQLKLPPKDTRTQTEDVTATKGLEFEDFYIKRELMMGIFEAGFEKPSPIQEETIPV

ALTGRDILARAKNGTGKTAAFVIPTLERTNPKISKTQALILVPTRELALQTSQVCKTLGKHLGINVMVTTGGTGLQDDII

RLSDTVHIIVGTPGRILDLASKGVADLSECTTFVMDEADKLLSPEFTPVIEQLLSFHPKDRQVMLFSATFPMIVKSFKDK

HMRNPYEINLMDELTLRGITQYYAFVEERQKVHCLNTLFSKLQINQSIIFCNSTNRVELLAKKITELGYSCFYSHARMLQ

QNRNRVFHDFRNGVCRNLVCSDLLTRGIDIQAVNVVINFDFPKNAETYLHRIGRSGRFGHLGLAINLINWDDRYNLYKIE

QELGTEIQPIPPSIDKKLYVYDTPETIPRPIANASTERNPPAQLAQSSDNQNHRQAHHISGGHGQQTANRGHSLRGSYRG

GRAQGHRGGHPENNLTAIWMLFGGSSPTQSFDSFTLEFSHFLFPFWTTFSAVAYITSPGLVPRSPCSYFNGRFTNMYHTA

HVSTRF

>XP_018127036.1 ATP-dependent RNA helicase DDX6/DHH1 [Pseudogymnoascus verrucosus]

MADALAGKLESTTLGSSQNDPDWKKKLKIPAKDNRHQTEDVTATKGLEFEEFSIKRDLLMGIFEAGFEKPSPIQEEAIPV

ALTGRDILARAKNGTGKTAAFVIPALERINPKSSKIQCLILVPTRELALQTSQVCKTLGKHLGVNVMVTTGGTGLRDDIV

RLAEPVHIVVGTPGRILDLAGKSVADLSECPMFIMDEADKLLSPEFTPVIEQLLQFHPKDRQIMLFSATFPRSVQAFSVK

NMDQPYEINLMDELTLRGITQYYAFVEEKQKVHCLNTLFSKLQINQSIIFCNSTNRVELLAKKITELGYSCFYSHARMLQ

ANRNRVFHDFRNGVCRNLVCSDLLTRGIDIQAVNVVINFDFPKNAETYLHRIGRSGRFGHLGLAINLINWDDRFNLYNIE

KELGTEIQPIPAMIDKNLYVYDSPESIPRPISNPAPRQQPPREQLQIQQRGGQAPANYSNGRQGPSSQAQPQGQAQAQAP

AQLQQGQPQGQRQPTNQPQGQRNQNFSRGNARVGAQPGRGRGFDGARGGRGQAPPPS

>XP_007882290.1 hypothetical protein PFL1_06556 [Anthracocystis flocculosa PF-1]

MSSSGPAPGQSEASWKQEILGKLRKDDRPQTEDVLNTKGNDFEDYFLKRELLMGIFEAGFERPSPIQEEAIPIALTGRDI

LARAKNGTGKTAAYVIPSLEKLNTKKNVIQAVLLVPTRELALQTSQVAKTLGKHLGVEVMVTTGGTTLRDDILRLGQTVH

LLVGTPGRILDLAGKGVADLSQCTTFVMDEADKLLSPEFTPVMEQLLSFLPKERQVMLFSATFPMIVKDFKDKNMVKPYE

INLMDELTLRGVTQYYAFVEERQKVHCLNTLFSKLQINQSIIFCNSTNRVELLAKKITELGYSCFYSHAKMLQSHRNRVF

HDFRNGACRNLVCSDLLTRGIDIQAVNVVINFDFPKNAETYLHRIGRSGRFGHLGLAINLITYEDRFNLYRIEQELGTEI

QPIPSNIDKRLYVAPSLIQNEGDERRQGQNGGGNGNGAGANGRQQEGRPVIPPGQAAMHAASIPQSQDLPRRCLV

>KKZ64812.1 ATP-dependent RNA helicase DHH1 [Emmonsia crescens UAMH 3008]

MADALASQLNSTKIGENHSEKWRENLKAPPKDTRTQTEDVTATKGLEFEDFYIKRELMMGIFEAGFEKPSPIQEETIPVA

LTGRDILARAKNGTGKTAAFVIPTLERTNPKNSKTQALILVPTRELALQTSQVCKTLGKHLGVNVMVTTGGTGLQDDIIR

LNEAVHIIVGTPGRILDLASKGVADLSECSTFVMDEADKLLSPEFTPVIEQLLSFHPKDRQVMLFSATFPMIVKSFKDKH

MRNPYEINLMDELTLRGITQYYAFVEEKQKVHCLNTLFSKLQINQSIIFCNSTNRVELLAKKITELGYSCFYSHAKMLQH

NRNRVFHDFRNGVCRNLVCSDLLTRGIDIQAVNVVINFDFPKNAETYLHRIGRSGRFGHLGLAINLINWEDRYNLYKIEQ

ELGTEIQPIPQSIDKKLYVYDTPDTIPRPISNAPPHQQQQQQVSAPAQHPNNPDSHNRRQGQHQYSSNRGRGSYRGGRGQ

GHRGGHGENHRQNGTAPRPNAAASAPAS

>KAF2000459.1 ATP-dependent RNA helicase-like protein dhh1 [Amniculicola lignicola CBS 123094]

MTTDITNQLAATKLRCVHSSEAAYNWREGLKIPSKDARPQTEDVTATKGLEFEDFLIKRELMMGIFEAGFEKPSPIQEET

IPVALTGRDILARAKNGTGKTAAFVIPTLERVNPKNNKTQALILVPTRELALQTSQVCKTLGKHLGVNVMVSTGGTGLKD

DIIRLSESVHIIVGTPGRILDLAGKGVADLSACETFVMDEADKLLSPEFTPVIEQLLAFHPKDRQVMLFSATFPIVVKSF

KDKHMNSPYEINLMDELTLRGITQYYAFVEEKQKVHCLNTLFTKLQINQSIIFCNSTNRVELLAKKITELGYSCFYSHAR

MLQHNRNRVFHDFRNGLCRNLVCSDLLTRGIDIQAVNVVINFDFPKNAETYLHRIGRSGRFGHLGLAINLINWEDRFNLY

RIEQELGTEIQPIPTVIEKNLYVYDSPENIPRPISGPPRPQGQIEDGSGNNGRPNPNQTNNRQNFQRNQRGGGQYQGQRR

GPPPNGQPNRQNGQNPQRNPRPQPPAA

>EER42776.1 ATP-dependent RNA helicase DHH1 [Histoplasma capsulatum H143]

MADALASQLNSTKLGESHSEKWRENLKMPPKDTRTQTEDVTATKGLEFEDFYIKRELMMGIFEAGFEKPSPIQEETIPVA

LTGRDILARAKNGTGKTAAFVIPTLERTNPKNSKTQALILVPTRELALQTSQVCKTLGKHLGINVMVTTGGTGLQDDIIR

LNEAVHIIVGTPGRILDLASKGVADLSDCSTFVMDEADKLLSPEFTPVIEQLLSFHPKDRQVMLFSATFPLIVKSFKDKH

MRNPYEINLMDELTLRGITQYYAFVEEKQKVHCLNTLFSKLQINQSIIFCNSTNRVELLAKKITELGYSCFYSHAKMLQH

NRNRVFHDFRNGVCRNLVCSDLLTRGIDIQAVNVVINFDFPKNAETYLHRIGRSGRFGHLGLAINLINWEDRYNLYKIEQ

ELGTEIQPIPQSIDKKLYVYDTPDTIPRPTSNVGHQQQQQQQQQQHQHTSEPPQHPNNPGNHNRRHGQHQYASNRGRGSY

RGGRGQGHRGGYGENHRQNCTAPRPSAAASAPAS

>OOQ86870.1 ATP-dependent RNA helicase dhh1 [Penicillium brasilianum]

MADALASQLKNTTLGRDGTSEVRLQDTLKLPPKDARPQTEDVTATKGLEFEDFYIKRELMMGIFEAGFEKPSPIQEETIP

VALTGRDILARAKNGTGKTAAFVIPTLERINPKSTKTQALILVPTRELALQTSQVCKTLGKHLGINVMVTTGGTGLMDDI

IRLNDAVHILVGTPGRVLDLASKGVADLSECPTFVMDEADKLLSPEFTPVIEQLMSFHPKDRQVMLFSATFPLIVKSFKD

KHMRNPYEINLMDELTLRGITQYYAFVEEKQKVHCLNTLFSKLQINQSIIFCNSTNRVELLAKKITELGYSCFYSHARML

QQHRNRVFHDFRNGVCRNLVCSDLLTRGIDIQAVNVVINFDFPKNAETYLHRIGRSGRFGHLGLAINLINWEDRFNLYKI

EQELGTEIQPIPQIIDKKLYVYDSPENIPRPISNPAQPMLSGPQHPSNGDRQPRRQHNHQNNGQYYNHSRGRGGSYRGGR

GQGPRRGPQQHDPNKSALAPSQVGPKPPAPVS

>OOF98484.1 hypothetical protein ASPCADRAFT_161929 [Aspergillus carbonarius ITEM 5010]

MTDALASQLNNTTLGDASSDTKWKEQLNVPAKDARPQTEDVTATKGLEFEDFYIKRELMMGIFEAGFEKPSPIQEETIPV

ALTGRDILARAKNGTGKTAAFVIPTLERINPKSTKTQALILVPTRELALQTSHVCKTLGKHLGINVMVTTGGTGLMDDII

RLNDAVHILVGTPGRVLDLASKGVADLSECPTFVMDEADKLLSPEFTPVIEQLLSFHPKDRQVMLFSATFPLIVKSFKDK

HMRNPYEINLMDELTLRGITQYYAFVEEKQKVHCLNTLFSKLQINQSIIFCNSTNRVELLAKKITELGYSCFYSHARMLQ

QHRNRVFHDFRNGVCRNLVCSDLLTRGIDIQAVNVVINFDFPKNAETYLHRIGRSGRFGHLGLAINLINWEDRFNLYKIE

QELGTEIQPIPQNIDKKLYVYDSPDTIPRPIANPSQPPITNSTVNLNTGERRHNHHMNGGQYQYGRGRGSYRGGRGQGQR

RNMQSEAKFGSQSQSSGKSHPTQVS

>KAF4307909.1 ATP-dependent RNA helicase dhh1 [Botryosphaeria dothidea]

MAADITNQLASASLNESSADWKNSLKAPPKDNRVQTEDVTATKGLEFEDFYIKRELMMGIFEAGFEKPSPIQEETIPVAL

TGRDILARAKNGTGKTAAFVIPTLERINPKSTKTQALILVPTRELALQTSQVCKTLGKHLGINVMVTTGGTGLKDDIIRL

SEPVHIIVGTPGRILDLASKGVADLSACPTFVMDEADKLLSPEFTPVIEQLLAFHPKDRQVMLFSATFPIVVKSFKDKHM

NQPYEINLMDELTLRGITQYYAFVEEKQKVHCLNTLFNKLQINQSIIFCNSTNRVELLAKKITELGYSCFYSHAKMLQHA

RNRVFHDFRNGVCRNLVCSDLLTRGIDIQAVNVVINFDFPKNAETYLHRIGRSGRFGHLGLAINLINWDDRFNLYKIEQE

LGTEIQPIPQTIEKKLYVYESPESIPRPISNAPANRGPLQQASIQGQLQDQDANPNASRGNAQHGGRGGYRGQRGGFQGQ

RRGGGQRNGFNGQRAPRPQA

>KIO31446.1 hypothetical protein M407DRAFT_5062 [Tulasnella calospora MUT 4182]

MSTATESPTSALTNNGDWKSQLNIPQKDDRPQTEDVTATKGLEWEDMQLRRELLMGIFEAGFEKPSPIQEEAIPVALTTR

DVLARAKNGTGKTAAFIIPSLQLIDTAIPKVQVMLLVPTRELALQTSQVCKLLGKHLGVNVMVTTGGTTLKDDIMRLSEA

VHVLVGTPGRILDLAGKGVADLSKCTIFVMDEADKLLSPEFTPVIEELLGFLPENRQVMLFSATFPMIVKDFKLKHMRSP

YEINLMDELTLRGVTQYYAFVEERQKVHCLNTLFSKLQINQSIIFCNSTNRVELLAKKITELGYSCFYSHAKMLQAHRNR

VFHDFRNGACRNLVCSDLLTRGIDIQAVNVVINFDFPKNSETYLHRIGRSGRFGHLGLAINLITYEDRFNLYRIEQELGT

EIQPIPQTIDKNLYVAPSGGGSSEPQTSTGRLGSQALPPKQDTRLTHRAPQQPRLPPQVQPAPGTPTSQAQQTPQQQQQQ

QATQVYATTPNGPPRNGLTPVQQQQMLAAQQAQQAQQQQQLFALQQQQQQQQQQQQQQQQRIAQQYQNAQATAMAQQQQA

AAMIAAARGQGHPARGGRGGFGRGQPQPR

>TKA78384.1 ATP-dependent RNA helicase DHH1 [Friedmanniomyces simplex]

MASELANKLEGTTLNDGATNDDWKASLKQPAKDKRVQTEDVTKTKGLEFEDFYLKRPLLMGIFEAGFEKPSPIQEETIPV

ALTGRDVLARAKNGTGKTAAFVIPTLERINPKLEKTQALILVPTRELALQTSQVCKTLGKHLGVNVMVTTGGTGLKDDII

RLSDTVHIIVGTPGRILDLASKGVADLSAATTFVMDEADKLLSPEFTVTIEQLLQFHPKDRQVMLFSATFPVIVKSFMDK

HMRDPHEINLMDELTLRGITQFYAFVEEKQKVHCLNTLFSRLQINQSIIFCNSTTRVELLAKKITELGYSCFYSHAKMLQ

QHRNRVFHEFRNGAVRNLVCSDLLTRGIDIQAVNVVINFDFPKNAETYLHRIGRSGRFGHLGLAINLISWEDRFNLYRIE

QELGTEIQPIPAAIDKSLYVYDAPDTIPRPINTAPQQQQQRPQQEGRRQQGNGADLQNPANRTGGFRGGRGGGRGGNFRG

PPRDSNQQSQNGFQGQQQRQGGFQQQQHQPQQQPLAPSAAQA

>PKR97999.1 ATP-dependent RNA helicase dhh1 [Cercospora zeina]

MADGLANKLEKTSLNEAADGDWKSGLKAPPKDGRQQTEDVTATKGLDFEEFYLKRELLMGIYEAGFEKPSPIQEETIPVA

LSGRDILARAKNGTGKTAAFVIPTLERINPKLDKIQALLLVPTRELALQTSQVCKTLGKHLGINVMVTTGGTGLRDDIIR

LNDPVHIIVGTPGRILDLAGKGVADLSEAKTFVMDEADKLLSPEFTVTIEQLLKFHPKDRQVMLFSATFPVVVKDFKDKH

MNDPHEINLMDELTLRGITQYYAFVEEKQKVHCLNTLFSRLQINQSIIFCNSTTRVELLAKKITELGYSCFYSHAKMLQQ

HRNRVFHDFRNGAMRNLVCSDLLTRGIDIQAVNVVINFDFPKNAETYLHRIGRSGRFGHLGLAINLINWEDRFNLYRIEQ

ELGTEIQPIPGTIDKKLYVYDAPENIPRPINTAPPPQAQQPAQQAGRQQGSGQDLLNPANRTGGYRGGRGGGYGRGRGGP

RNDQNGFQGQQRPGGFQQRAPPQQQMAPAGPPAAQA

>OBT56443.1 ATP-dependent RNA helicase DDX6/DHH1 [Pseudogymnoascus sp. 24MN13]

MADALAGKLESTTLGSSQNDPDWKKKLKIPVKDNRHQTEDVTATKGLEFEEFSIKRDLLMGIFEAGFEKPSPIQEEAIPV

ALTGRDILARAKNGTGKTAAFVIPALERINPKSSKIQCLILVPTRELALQTSQVCKTLGKHLGVNVMVTTGGTGLRDDIV

RLAEPVHIVVGTPGRILDLAGKSVADLSECPMFIMDEADKLLSPEFTPVIEQLLQFHPKDRQIMLFSATFPRSVQAFSVK

NMDQPYEINLMDELTLRGITQYYAFVEEKQKVHCLNTLFSKLQINQSIIFCNSTNRVELLAKKITELGYSCFYSHARMLQ

ANRNRVFHDFRNGVCRNLVCSDLLTRGIDIQAVNVVINFDFPKNAETYLHRIGRSGRFGHLGLAINLINWDDRFNLYNIE

KELGTEIQPIPAMIDKNLYVYDSPESIPRPISNPAPRQQPPREQLQIQQRGGQAPANYSNGRQGPSSQAQPQGQAQAQAP

AQLQQGQPQGQRQPTNQPQGQRNQNFSRGNARVGAQPGRGRGFDGARGGRGQAPPPS

>KJY02018.1 ATP-dependent RNA helicase dhh1 like protein [Zymoseptoria brevis]

MAAELASKLESTSLNDSSPAGGDWKAGLKAPAKDGRHQTEDVTATKGLEFEEFYLKRELLMGIFEAGFEKPSPIQEETIP

VALSGRDILARAKNGTGKTAAFVIPTLERINPKLDKVQALLLVPTRELALQTSQVCKTLGKHLGINVMVTTGGTGLRDDI

MRLAETVHIIVGTPGRILDLAGKGVADLSEAKTFVMDEADKLLSPEFTVTIEQLLKFHPKDRQVMLFSATFPVAVKEFKD

RHMNDPHEINLMDELTLRGITQYYAFVEEKQKVHCLNTLFSRLNINQSIIFCNSTTRVELLAKKITELGYSCFYSHAKML

QQHRNRVFHDFRNGAMRNLVCSDLLTRGIDIQAVNVVINFDFPKNAETYLHRIGRSGRFGHLGLAINLINWDDRFNLYRI

EQELGTEIQPIPPSIDKSLYVYDSPENIPRPINTAPQQPQSQQAGRQQGNGSDLQDPANRTSGGYRGGRGGGGFQGRGGG

FQGQGQGRGRGGPRNDQQYPQNGAQNGQNGFQGQQQQQRQGGQYPPRPNQQMPPAGPPAAQA

>XP_016636920.1 ATP-dependent RNA helicase dhh1 [Fonsecaea multimorphosa CBS 102226]

MTDALAAQLNKASLNDGADESNWKDSLNLPAKDTRHQTEDVTATKGLDFEDFYIKRELMMGIFEAGFEKPSPIQEETIPV

ALTGRDILARAKNGTGKTAAFVIPTLERINPKNPKTQALILVPTRELALQTSQVCKTLGKHLGINVMVTTGGTGLKDDII

RLGETVHIIVGTPGRILDLASKGVADLSECPIFVMDEADKLLSPEFTVVIEQLLSFHPKDRQVMLFSATFPMIVKSFKDK

HMRNPYEINLMDELTLRGITQYYAFVEEKQKVHCLNTLFSKLQINQSIIFCNSTNRVELLAKKITELGYSCFYSHAKMLQ

QNRNKVFHDFRAGVCRNLVCSDLLTRGIDIQAVNVVINFDFPKNAETYLHRIGRSGRFGHRGLAINLINWDDRFNLYKIE

QELGTEIQPIPPSIDKSLYVYDNPELIPRAMPPPAQTARSNASNANATANTSANNNATNTNGQYQQVRRPQNQSNGYGQQ

YNNQGRGGYRGRGRGQGARGRGGHPQQPAAAPLGQIAH

>XP_033667094.1 uncharacterized protein M409DRAFT_66697 [Zasmidium cellare ATCC 36951]

MASELANKLEATTLDDASASGDWKSALKAPPKDGRQQTEDVTATKGLEFEEFYLKRELLMGIYEAGFEKPSPIQEETIPV

ALSGRDVLARAKNGTGKTAAFVIPTLERINPKVDKIQALLLVPTRELALQTSQVCKTLGKHLGINVMVTTGGTGLREDII

RLSDPVHIVVGTPGRILDLAGKGVADLSEVKTFVMDEADKLLSPEFTVTIEQLLKFHPKDRQVMLFSATFPVVVKDFKDK

HMSDPHEINLMDELTLRGITQYYAFVEEKQKVHCLNTLFSRLQINQSIIFCNSTTRVELLAKKITELGYSCFYSHAKMLQ

HHRNRVFHDFRNGAMRNLVCSDLLTRGIDIQAVNVVINFDFPKNAETYLHRIGRSGRFGHLGLAINLINWEDRFNLYRIE

QELGTEIKPIPPSIDKKLYVYDTPENIPRPISSAPAQRQQQGQQGRQQGNGQDLQNPANRTTGGYRGGRGGGFGAGRGRG

GPRNDQNGFQGQQRQGGYQQRPNQQQMAPAGPPAAQA

>OJD23626.1 ATP-dependent RNA helicase dhh1 [Blastomyces percursus]

MADALASQLNSTKLGDNHSEKWRENLKAPPKDTRTQTEDVTATKGLEFEDFYIKRELMMGIFEAGFEKPSPIQEETIPVA

LTGRDILARAKNGTGKTAAFVIPTLERTNPKNSKTQALILIPTRELALQTSQVCKTLGKHLGINVMVTTGGTGLQDDIIR

LNEAVHIIVGTPGRILDLASKGVADLSECSTFVMDEADKLLSPEFTPVIEQLLSFHPKDRQVMLFSATFPMIVKSFKDKH

MRNPYEINLMDELTLRGITQYYAFVEEKQKVHCLNTLFSKLQINQSIIFCNSTNRVELLAKKITELGYSCFYSHAKMLQH

NRNRVFHDFRNGVCRNLVCSDLLTRGIDIQAVNVVINFDFPKNAETYLHRIGRSGRFGHLGLAINLINWEDRYNLYKIEQ

ELGTEIQPIPQSIDKKLYVYDTPDTIPRPISNAPQQQPASAIAQTPNNPEGHNRRQGQHQYSSNRGRGSYRGGRGQGHRG

SHGESHRQNGTAPRPNAAASAPAS

>KAF2211935.1 hypothetical protein CERZMDRAFT_97855 [Cercospora zeae-maydis SCOH1-5]

MADGLANKLEKTSLNEAADGDWKSGLKAPPKDGRQQTEDVTATKGLDFEEFYLKRELLMGIYEAGFEKPSPIQEETIPVA

LSGRDILARAKNGTGKTAAFVIPTLERINPKLDKIQALLLVPTRELALQTSQVCKTLGKHLGINVMVTTGGTGLRDDIIR

LNDPVHIVVGTPGRILDLAGKGVADLSEAKTFVMDEADKLLSPEFTVTIEQLLKFHPKDRQVMLFSATFPVVVKDFKDKH

MNDPHEINLMDELTLRGITQYYAFVEEKQKVHCLNTLFSRLQINQSIIFCNSTTRVELLAKKITELGYSCFYSHAKMLQQ

HRNRVFHDFRNGAMRNLVCSDLLTRGIDIQAVNVVINFDFPKNAETYLHRIGRSGRFGHLGLAINLINWEDRFNLYRIEQ

ELGTEIQPIPGTIDKKLYVYDAPENIPRPINTAPPQQQQQQQQQQQQAQQAGRQQGSGQDLLNPANRTGGYRGGRGGGYG

RGRGGPRNDQNGLQGQQRQGGFQQRAPPQQQMAPAGPPAAQA

>XP_020123520.1 ATP-dependent RNA helicase dhh1 [Talaromyces atroroseus]

MVDAVTSQLSNTKLGETEGTWKDQLKIPAKDNRTQTEDVTATKGLEFEDFYIKRELMMGIFEAGFEKPSPIQEETIPVAL

TGRDILARAKNGTGKTAAFIIPTLERINPKSRKTQALILVPTRELALQTSQVCKTLGKHLGINVMVTTGGTGLMDDIIRL

NDTVHIIVGTPGRVLDLASKGVADLSECPTFVMDEADKLLSPEFTPVIEQLLSFHPKDRQVMLFSATFPMIVKSFKDKHM

RNPYEINLMDELTLRGITQYYAFVEEKQKVHCLNTLFSKLQINQSIIFCNSTNRVELLAKKITELGYSCFYSHARMLQHN

RNRVFHDFRNGVCRNLVCSDLLTRGIDIQAVNVVINFDFPKNAETYLHRIGRSGRFGHLGLAINLINWEDRFNLYKIEQE

LGTEIQPIPQSIDKKLYVYDSPDTIPRPITSGPQGQQPEGYRGAEHQAYRQQPGGRYNNAGRGRGAYRGRGGQGQRRGGG

HDNHRPVNPHAHDGTKPQPTPAS

>QLQ79161.1 hypothetical protein HG537_0B05090 [Torulaspora globosa]

MSDDQSWKSQLNIPKRDTRPQTEDVLNTKGNTFEDFYLKRELLMGIFEAGFEKPSPIQEEAIPVAIAGRDVLARAKNGTG

KTAAFVIPALERVKPKLNKIQALIMVPTRELALQTSQVVRTLGKHCGVSCMVTTGGTNLKDDILRLNETVHILVGTPGRV

LDLASRKLADLSECSLFIMDEADKMLSRDFKTIIEQILIFLPQSYQSLLFSATFPLTVKEFMVKHLNKPYEINLMEELTL

KGITQYYAFVEERQKLHCLNTLFSKLQINQAIIFCNSTNRVELLAKKITDLGYSCYYSHARMKQQERNRVFHEFRQGKVR

TLVCSDLLTRGIDIQAVNVVINFDFPKTAETYLHRIGRSGRFGHLGLAINLINWNDRFNLYKIEQELGTEIAAIPATIDK

SLYVAEDTSAVPVPFPIQQKQPQTTQFSQPQQYLPQPQQNHQIHPQAHPLQTMPQPQRGISPQDIPPPQMQGHFAAQPQY

>XP_002486388.1 DEAD-box RNA helicase Dhh1/Vad1, putative [Talaromyces stipitatus ATCC 10500]

MVDAVTSQLSNTKLGETEGSWKDQLKIPAKDNRTQTEDVTATKGLEFEDFYIKRELMMGIFEAGFEKPSPIQEETIPVAL

TGRDILARAKNGTGKTAAFIIPTLERINPKSTKTQALILVPTRELALQTSQVCKTLGKHLGINVMVTTGGTGLMDDIIRL

NDTVHIIVGTPGRVLDLASKGVADLSECPTFVMDEADKLLSPEFTPVIEQLLSFHPKDRQVMLFSATFPMIVKSFKDKHM

RNPYEINLMDELTLRGITQYYAFVEEKQKVHCLNTLFSKLQINQSIIFCNSTNRVELLAKKITELGYSCFYSHARMLQHN

RNRVFHDFRNGVCRNLVCSDLLTRGIDIQAVNVVINFDFPKNAETYLHRIGRSGRFGHLGLAINLINWDDRFNLYKIEQE

LGTEIQPIPQSIDKKLYVYDSPDTIPRPIASGPQPGTEGQQRSEKQGYRQGNHHPGGRYSNNNSSRGRGNYRGRGGQGQR

RGGGYQNAHAHDGTKPQPTPAS

>KAF2641300.1 ATP-dependent RNA helicase DHH1 [Massarina eburnea CBS 473.64]

MADQIATQLAATKLSESSPSGTLNWKENLNIPTKDTRVQTEDVTATKGLEFEDFFIKRELMMGIFEAGFEKPSPIQEETI

PVALTGRDILARAKNGTGKTAAFVIPTLERVNPKSNKTQALILVPTRELALQTSQVCKLLGKHLGINVMVSTGGTGLKDD

IIRLSESVHIIVGTPGRILDLAGKGVADLSACQTFVMDEADKLLSPEFTPVVEQLLSFHPKDRQVMLFSATFPIVVKSFK

DKHMNSPYEINLMDELTLRGITQYYAFVEEKQKVHCLNTLFNKLQINQSIIFCNSTNRVELLAKKITELGYSCFYSHARM

LQHNRNRVFHDFRNGVCRNLVCSDLLTRGIDIQAVNVVINFDFPKNAETYLHRIGRSGRFGHLGLAINLINWDDRFNLYR

IEQELGTEIQPIPQVIEKNLYVYDAPESIPRPMNSAVQRPTQGQDQENAVARSNPNQGQGQTQNNRGNYNRNQRGGRQFQ

SQVRGPQGPPPQGQGPNPSRQNGQNPQRNARPQPANP

>ORY12747.1 ATP-dependent RNA helicase DHH1 [Clohesyomyces aquaticus]

MTSEITNQLAATQLGGEAPASDANWKDGLKVPSKDARPQTEDVTATKGLEFEDFFIKRELMMGIFEAGFEKPSPIQEETI

PVALTGRDILARAKNGTGKTAAFVIPTLERINPKSNKTQALILVPTRELALQTSQVCKMLGKHLDINVMVSTGGTGLKDD

IIRLADPVHIIVGTPGRILDLAGKGVADLSACQTFVMDEADKLLSPEFTPVVEQLLGFHPKERQVMLFSATFPIVVKSFK

DKHMNQPYEINLMDELTLRGITQYYAFVEEKQKVHCLNTLFNKLQINQSIIFCNSTNRVELLAKKITELGYSCFYSHAKM

LQHNRNRVFHDFRNGVCRNLVCSDLLTRGIDIQAVNVVINFDFPKNAETYLHRIGRSGRFGHLGLAINLINWEDRFNLYR

IEQELGTEIQPIPQVIEKKLYVYESPETIPRPISNPPRPGQPQDQDGSVARSNSSQQNSRQNYQRGGRGGGGQFQRRGGA

QNQQNRQNGQYPQRNSRPQPAGPA

>RAO72982.1 hypothetical protein BHQ10_008994 [Talaromyces amestolkiae]

MVDAVTSQLSNTKLGETDGSWKDQLKIPAKDNRTQTEDVTATKGLEFEDFYIKRELMMGIFEAGFEKPSPIQEETIPVAL

TGRDILARAKNGTGKTAAFIIPTLERINPKSTKTQALILVPTRELALQTSQVCKTLGKHLGINVMVTTGGTGLMDDIIRL

NDTVHIIVGTPGRVLDLASKGVADLSECPTFVMDEADKLLSPEFTPVIEQLLSFHPKDRQVMLFSATFPMIVKSFKDKHM

RNPYEINLMDELTLRGITQYYAFVEEKQKVHCLNTLFSKLQINQSIIFCNSTNRVELLAKKITELGYSCFYSHARMLQHN

RNRVFHDFRNGVCRNLVCSDLLTRGIDIQAVNVVINFDFPKNAETYLHRIGRSGRFGHLGLAINLINWEDRFNLYKIEQE

LGTEIQPIPQSIDKKLYVYDSPDTIPRPIASAPQPGSEGQQREHHGYRQGGHHPGGRYNNHNTGRGRGGYRGRGGQGQRR

GGYQNPHGHDGSKPQPTPAS

>PGH16094.1 ATP-dependent RNA helicase DHH1 [Polytolypa hystricis UAMH7299]

MADALASQLNNTKLGEGNADNRWKEHLKAPVKDTRVQTEDVTATKGLEFEDFYIKRELMMGIFEAGFEKPSPIQEETIPV

ALTGRDILARAKNGTGKTAAFVIPTLERTNPKNSKTQALILVPTRELALQTSQVCKTLGKHLGINVMVTTGGTGLQDDII

RLNDAVHIIVGTPGRILDLASKGVADLSECSTFVMDEADKLLSPEFTPVIEQLLSFHPKDRQVMLFSATFPMIVKSFKDK

HMRNPYEINLMDELTLRGITQYYAFVEEKQKVHCLNTLFSKLQINQSIIFCNSTNRVELLAKKITELGYSCFYSHARMLQ

HNRNRVFHDFRNGVCRNLVCSDLLTRGIDIQAVNVVINFDFPKNAETYLHRIGRSGRFGHLGLAINLINWEDRYNLYKIE

QELGTEIQPIPQSIDKKLYVYDTPETIPRPIANAPPQRQNPGGQQPHANATENQERQQAHQHPRHGQYGSNRGHQTRGGY

RGGRGQGHRGGQGDPNRQNGPRQNPSAPTATA

>RAR04713.1 atp-dependent rna helicase dhh1 [Stemphylium lycopersici]

MTSEITNQLAATQLTDATPSGDANWKAGLNAPAKDARPQTEDVTATKGLEFEDFFIKRELMMGIFEAGFEKPSPIQEETI

PVALTGRDILARAKNGTGKTAAFVIPTLERVNPKSPKTQALILVPTRELALQTSQVCKQLGKHLGINVMVSTGGTGLKDD

IIRLSDPVHIIVGTPGRILDLAGKGVADLSACQTFVMDEADKLLSPEFTPVVEQLLGFHPKDRQVMLFSATFPIVVKSFK

DKHMNSPYEINLMDELTLRGITQYYAFVEEKQKVHCLNTLFNKLQINQSIIFCNSTNRVELLAKKITELGYSCFYSHARM

LQHNRNRVFHDFRNGVCRNLVCSDLLTRGIDIQAVNVVINFDFPKNAETYLHRIGRSGRFGHLGLAINLINWEDRFNLYR

IEQELGTEIQPIPQVIEKNLYVYESPESIPRPMSNQPRAPAQIQQQEQEGAARGNPNARGGFRGGRGGGGGGGGGQFQGQ

RRGPPQNAQNRQQNGQNPQRNPRPQPAGPPQAS

>TKA47489.1 ATP-dependent RNA helicase DHH1 [Friedmanniomyces endolithicus]

MASELASKLEATTLNEGATNDNWKASLKQPVKDKRVQTEDVTKTKGLEFEDFYLKRPLLMGIFEAGFEKPSPIQEETIPV

ALTGRDVLARAKNGTGKTAAFVIPTLERINPKLEKTQALILVPTRELALQTSQVCKTLGKHLGVNVMVTTGGTGLKDDII

RLSDTVHIIVGTPGRILDLASKGVADLSAATTFVMDEADKLLSPEFTVTIEQLLQFHPKDRQVMLFSATFPVIVKSFMDK

HMRDPHEINLMDELTLRGITQFYAFVEEKQKVHCLNTLFSRLQINQSIIFCNSTTRVELLAKKITELGYSCFYSHAKMLQ

QHRNRVFHEFRNGAVRNLVCSDLLTRGIDIQAVNVVINFDFPKNAETYLHRIGRSGRFGHLGLAINLISWEDRFNLYRIE

QELGTEIQPIPAAIDKSLYVYDAPENIPRPVNTASQPQQRPQQEGRRQQGNGADLQNPANRTGGYRGGRGGGRGGGFRGP

PREPGQQNQNGSQGQQRPSGFQQQPPPQALTPSAAQA

>KNG47632.1 atp-dependent rna helicase dhh1 [Stemphylium lycopersici]

MTSEITNQLAATQLTDATPSGDANWKAGLNAPAKDARPQTEDVTATKGLEFEDFFIKRELMMGIFEAGFEKPSPIQEETI

PVALTGRDILARAKNGTGKTAAFVIPTLERVNPKSPKTQALILVPTRELALQTSQVCKQLGKHLGINVMVSTGGTGLKDD

IIRLSDPVHIIVGTPGRILDLAGKGVADLSACQTFVMDEADKLLSPEFTPVVEQLLGFHPKDRQVMLFSATFPIVVKSFK

DKHMNSPYEINLMDELTLRGITQYYAFVEEKQKVHCLNTLFNKLQINQSIIFCNSTNRVELLAKKITELGYSCFYSHARM

LQHNRNRVFHDFRNGVCRNLVCSDLLTRGIDIQAVNVVINFDFPKNAETYLHRIGRSGRFGHLGLAINLINWEDRFNLYR

IEQELGTEIQPIPQVIEKNLYVYESPESIPRPMSNQPRAPAQIQQQEQEGAARGNPNARGGFRGGRGGGGGGGGQFQGQR

RGPPQNAQNRQQNGQNPQRNPRPQPAGPPQAS

>OCK82431.1 DEAD-domain-containing protein [Lepidopterella palustris CBS 459.81]

MTSEITNKLAATRLGDSSSPDANWKDGLKTPSKDARPQTEDVTATKGLEFEDFYIKRELMMGIFEAGFEKPSPIQEETIP

VALTGRDILARAKNGTGKTAAFVIPTLEKVNPKSPKTQALILVPTRELALQTSQVCKILGKHLGMNVMVTTGGTGLKDDI

IRLSEPVHIIVGTPGRILDLASKGVADLSGCHTFVMDEADKLLSPEFTPVIEQLLAFHPKDRQVMLFSATFPIVVKSFKD

KHMNSPYEINLMDELTLRGITQYYAFVEEKQKVHCLNTLFNKLQINQSIIFCNSTNRVELLAKKITELGYSCFYSHARML

QHNRNRVFHDFRNGVCRNLVCSDLLTRGIDIQAVNVVINFDFPKNAETYLHRIGRSGRFGHLGLAINLINWEDRFNLYRI

EQELGTEIQPIPQVIEKKLYVYESPENIPRPISNPPGQRNQGQGQGQGQNQNQEGTGNTAGSNSTQPNRQNFQRNQRGGS

QFQGQRRGPPPTQQNRQNGHNAQRNTRPPPAAPA

>TKA44122.1 ATP-dependent RNA helicase DHH1 [Friedmanniomyces endolithicus]

MASELASKLEGTTLNDGATNDDWKASLKQPVKDKRVQTEDVTKTKGLEFEDFYLKRPLLMGIFEAGFEKPSPIQEETIPV

ALTGRDVLARAKNGTGKTAAFVIPTLERINPKLEKTQALILVPTRELALQTSQVCKTLGKHLGVNVMVTTGGTGLKDDII

RLSDTVHIIVGTPGRILDLASKGVADLSAATTFVMDEADKLLSPEFTVTIEQLLQFHPKDRQVMLFSATFPVIVKSFMDK

HMRDPHEINLMDELTLRGITQFYAFVEEKQKVHCLNTLFSRLQINQSIIFCNSTTRVELLAKKITELGYSCFYSHAKMLQ

QHRNRVFHEFRNGAVRNLVCSDLLTRGIDIQAVNVVINFDFPKNAETYLHRIGRSGRFGHLGLAINLISWEDRFNLYRIE

QELGTEIQPIPAAIDKSLYVYDAPENIPRPVNTASQPQQRPQQEGRRQQGNGADLQNPANRTGGYRGGRGGGRGGGFRGP

PREPGQQNQNGFQGQQRQSGFQQQPQPQALAPSAAQA

>XP_025394407.1 DEAD-domain-containing protein [Aspergillus heteromorphus CBS 117.55]

MADALASQLNNTTLGDANPDAKWKDQLNVPAKDARPQTEDVTATKGLEFEDFYIKRELMMGIFEAGFEKPSPIQEETIPV

ALTGRDILARAKNGTGKTAAFVIPTLERINPKSTKTQALILVPTRELALQTSHVCKTLGKHLGINVMVTTGGTGLMDDII

RLNDAVHILVGTPGRVLDLASKGVADLSECPTFVMDEADKLLSPEFTPVIEQLLSFHPKDRQVMLFSATFPLIVKSFKDK

HMRNPYEINLMDELTLRGITQYYAFVEEKQKVHCLNTLFSKLQINQSIIFCNSTNRVELLAKKITELGYSCFYSHARMLQ

QHRNRVFHDFRNGVCRNLVCSDLLTRGIDIQAVNVVINFDFPKNAETYLHRIGRSGRFGHLGLAINLINWEDRFNLYKIE

QELGTEIQPIPQNIDKKLYVYDSPDTIPRPISNPSQPHTTTAPNPNTGERRHNHHANSGQYQYGRSRGTYRGGRGQGQRR

SIQNETKFGAPQGQSSSKSHPTQVL

>XP_003716556.1 ATP-dependent RNA helicase DHH1 [Pyricularia oryzae 70-15]

MADVLADQLRSATLSDATNNEDWRRNLNIPARDNRQQTEDVTNTKGLEFENFGLKRDLLMGIFEAGFEKPSPIQEESIPV

ALTGRDILARAKNGTGKTAAFVVPALETINPKVSKIQCLILVPTRELAMQTSQVCKTLGKHLGINVMVTTGGTTLRDDIL

RLQDPVHIVVGTPGRILDLAGKNVADLSECPMFIMDEADKLLSIEFTPVIEQLLQFHPKDRQVMLFSATFPISVKEFSDK

NMTNPYEINLMDELTLRGITQYYAFVEEKQKVHCLNTLFSKLQINQSIIFCNSTNRVELLAKKITELGYSCFYSHAKMQQ

QARNRVFHDFRNGVCRNLVCSDLLTRGIDIQAVNVVINFDFPKNAETYLHRIGRSGRYGHLGLAINLISWEDRFNLYNIE

RDLGTEIQPIPSTIDKSLYVYDNPETIPRPINIPAQPSSAGNAQSAAPNQGPPPQQQRPHQGQENWQNQNGRHNGSSQQQ

QPRGPHQNRGRGGGRGRGGFQGQGQGQRNYNNNYRGGRGGHNQGQHQQQMPNQQS

>GAD99861.1 ATP-dependent RNA helicase DHH1 [Byssochlamys spectabilis No. 5]

MTDALASQLSNTKLGDSNMDAAWKEKLNVPAKDNRAQTEDVTATKGLEFEDFYIKRELMMGIFEAGFEKPSPIQEETIPV

ALTGRDILARAKNGTGKTAAFVIPTLERINPKSTKTQALILVPTRELALQTSQVCKTLGKHLGINVMVTTGGTGLMDDII

RLNDPVHIIVGTPGRVLDLASKGVADLSECHTFVMDEADKLLSPEFTPVIEQLLSFHPKDRQIMLFSATFPLIVKSFKDK

HMRNPYEINLMDELTLRGITQYYAFVEEKQKVHCLNTLFSKLQINQSIIFCNSTNRVELLAKKITELGYSCFYSHARMLQ

HNRNRVFHDFRNGVCRNLVCSDLLTRGIDIQAVNVVINFDFPKNAETYLHRIGRSGRFGHLGLAINLINWDDRFNLYKIE

QELGTEIQPIPQSIDKKLYVYESPDTIPRPISNLPQNQPRQLTQGQPGSNGEPHQRRNHHTNGGHYNSNRGRGSYRGGRG

QGQRRGGQHDGNKTGVPSQAGGKPQPLQAS

>GES65585.1 hypothetical protein ATETN484_0012049000 [Aspergillus terreus]

MADALASQLNNTSLGDANSDAKWKNQLKTPAKDARPQTEDVTATKGLEFEDFYIKRELMMGIFEAGFEKPSPIQEETIPV

ALTGRDILARAKNGTGKTAAFVIPTLERINPKSTKTQALILVPTRELALQTSHVCKTLGKHLGINVMVTTGGTGLMDDII

RLNDAVHILVGTPGRVLDLASKGVADLSECPTFVMDEADKLLSPEFTPVIEQLLSFHPKDRQVMLFSATFPLIVKSFKDK

HMRNPYEINLMDELTLRGITQYYAFVEEKQKVHCLNTLFSKLQINQSIIFCNSTNRVELLAKKITELGYSCFYSHARMLQ

QHRNRVFHDFRNGVCRNLVCSDLLTRGIDIQAVNVVINFDFPKNAETYLHRIGRSGRFGHLGLAINLINWDDRFNLYKIE

QELGTEIQPIPQNIDKKLYVYDSPDTIPRPISNPSQPPQATSMPSGQHIGDRRHNNHPNGGHYQYNRGRGSYRGGRGQGP

RRNMQHDQNRYNASQGQHQSGKSQAAPVS

>QBZ60772.1 hypothetical protein PoMZ_07714 [Pyricularia oryzae]

MADVLADQLRSATLSDATNNEDWRRNLNIPARDNRQQTEDVTNTKGLEFENFGLKRDLLMGIFEAGFEKPSPIQEESIPV

ALTGRDILARAKNGTGKTAAFVVPALETINPKVSKIQCLILVPTRELAMQTSQVCKTLGKHLGINVMVTTGGTTLRDDIL

RLQDPVHIVVGTPGRILDLAGKNVADLSECPMFIMDEADKLLSIEFTPVIEQLLQFHPKDRQVMLFSATFPISVKEFSDK

NMTNPYEINLMDELTLRGITQYYAFVEEKQKVHCLNTLFSKLQINQSIIFCNSTNRVELLAKKITELGYSCFYSHAKMQQ

QARNRVFHDFRNGVCRNLVCSDLLTRGIDIQAVNVVINFDFPKNAETYLHRIGRSGRYGHLGLAINLISWEDRFNLYNIE

RDLGTEIQPIPSTIDKSLYVYDNPETIPRPINIPAQPSSAGNAQSAAPNQGPPPQQQRPHQGQENWQNQNGRHNGSNQQQ

QPRGPHQNRGRGGGRGRGGFQGQGQGQRNYNNNYRGGRGGHNQGQHQQQMPNQQS

>ORY79504.1 P-loop containing nucleoside triphosphate hydrolase protein [Protomyces lactucaedebilis]

MDSVNRQMQQTSISSSNDDWKSKLNVPAKDTRPQTEDVTATKGMEFADMHLKRELLMGIFEAGFERPSPIQEEAIPVALT

GRDILARAKNGTGKTAAFCIPSLEKVDVKLNKIQALLLTPTRELALQTAQVCKILGKHMGINVMVTTGGTSLTQDIMRLN

DPIHVVVGTPGRILDLANKGIAKFDQCPTFVMDEADKLLSPEFTPVVEQLLAHCPKDRQISLFSATFPITVKDFMDRNLN

KPFEINLMDELTLRGVTQYYAFVEEKQKVHCLNTLFSKLQINQSIIFCNSTNRVELLAKKITELGYSCFYSHAKMLQAHR

NRVFHDFRNGVSRNLVCSDLLTRGIDIQAVNVVINFDFPKNAETYLHRIGRSGRFGHLGLAINLVSWEDRFNLYKIEQEL

GTEIQPIPNTIDKQLYVAPSAINSGEGAQKPTKQQPLQHGVPQTAEQAQQQQERRQQQQQQQQQQQRGGQQGQQQQFGQG

QQGQVHGQRGGQRNGYAQQGGQRGGRGGGQGQGQARPQ

>XP_016638709.1 ATP-dependent RNA helicase DHH1 [Scedosporium apiospermum]

MTDALADKLNTIQIGYASNLSPPALQRHPPLSVDSLTSPSDGTNDDWKKALKIPAKDGRKQTEDVTNTKGLEFEDFGLKR

DLLMGIFEAGFEKPSPIQEEAIPVALTGRDILARAKNGTGKTAAFVIPALEKINPKVSKIQCLILVPTRELAMQTSHVCK

SLGKHLGINVMVTTGGTGLRDDIIRLQDPVHIVVGTPGRILDLAGKNVADLSECPMFIMDEADKLLSQEFTPVIEQLLQF

HPKDRQVMLFSATFPLSVKDFSDKNMVSPYEINLMDELTLRGITQYYAFVDEKSKVHCLNTLFSRLQINQSIIFCNSTNR

VELLAKKITELGYSCFYSHAKMAQQARNRVFHDFRNGVCRNLVCSDLLTRGIDIQAVNVVINFDFPKNAETYLHRIGRSG

RYGHLGLAINLINWDDRFNLYNIERDLGTEIQPIPQTIDKSLYVYENPETIPRPISTLKPGVNSTAKPNAPQNFNGQSQD

QAQPAQQRSQHTHHQQQQPAGPAPNVNWQTQNLTQNGPSHYQSNRGRGRGRGYHGQGRGRGSYNGGYGRGRGQGPAIQQS

>KAE8336517.1 ATP-dependent RNA helicase dhh1 [Aspergillus arachidicola]

MAEALASQLNNTTLGEASSDTRWKDQLKAPAKDARPQTEDVTATKGLEFEDFYIKRELMMGIFEAGFEKPSPIQEETIPV

ALTGRDILARAKNGTGKTAAFVIPTLERINPKSTKTQALILVPTRELALQTSHVCKTLGKHLGINVMVTTGGTGLMDDII

RLNDAVHILVGTPGRVLDLASKGVADLSECPTFVMDEADKLLSPEFTPVIEQLLSFHPKDRQVMLFSATFPLIVKSFKDK

HMRNPYEINLMDELTLRGITQYYAFVEEKQKVHCLNTLFSKLQINQSIIFCNSTNRVELLAKKITELGYSCFYSHARMLQ

QHRNRVFHDFRNGVCRNLVCSDLLTRGIDIQAVNVVINFDFPKNAETYLHRIGRSGRFGHLGLAINLINWDDRFNLYKIE

QELGTEIQPIPQNIDKKLYVYESPETIPRPIANASQAQLATSGNQTQNMGERRNNNHSNGGHYQFGRGRGSYRGGRSQGQ

RRNMQNEMNKFGTSQNQQQSGKSQPAQVSPN

>XP_025444907.1 DEAD-domain-containing protein [Aspergillus brunneoviolaceus CBS 621.78]

MADALASQMKSTTLGDTSSDAKWKEQLNVPAKDARPQTEDVTATKGLEFEDFYIKRELMMGIFEAGFEKPSPIQEETIPV

ALTGRDILARAKNGTGKTAAFVIPTLERINPKSTKTQALILVPTRELALQTSHVCKTLGKHLGINVMVTTGGTGLMDDII

RLNDAVHILVGTPGRVLDLASKGVADLSECPTFVMDEADKLLSPEFTPVIEQLLSFHPKDRQVMLFSATFPLIVKSFKDK

HMRNPYEINLMDELTLRGITQYYAFVEEKQKVHCLNTLFSKLQINQSIIFCNSTNRVELLAKKITELGYSCFYSHARMLQ

QHRNRVFHDFRNGVCRNLVCSDLLTRGIDIQAVNVVINFDFPKNAETYLHRIGRSGRFGHLGLAINLINWDDRFNLYKIE

QELGTEIQPIPQNIDKKLYVYDSPDTIPRPIANPSQPHINTTVTNATAADRRPTHHANGGHYQFHRGRGSYRGGRGQGQR

RSIHNDAKQFSASQGQTGGKTHTTPVS

>QLL31648.1 hypothetical protein HG536_0B05130 [Torulaspora globosa]

MSEDQSWKSQLNIPKKDTRPQTEDVLNTKGNTFEDFYLKRELLMGIFEAGFEKPSPIQEEAIPVAIAGRDVLARAKNGTG

KTAAFVIPALERVKPKLNKIQALIMVPTRELALQTSQVVRTLGKHCGVSCMVTTGGTNLKDDILRLNETVHILVGTPGRV

LDLASRKLADLSECSLFIMDEADKMLSRDFKTIIEQILIFLPQSYQSLLFSATFPLTVKEFMVKHLNKPYEINLMEELTL

KGITQYYAFVEERQKLHCLNTLFSKLQINQAIIFCNSTNRVELLAKKITDLGYSCYYSHARMKQQERNRVFHEFRQGKVR

TLVCSDLLTRGIDIQAVNVVINFDFPKTAETYLHRIGRSGRFGHLGLAINLINWNDRFNLYKIEQELGTEIAAIPATIDK

SLYVAEDTSAVPVPFPIQQQQTVQLSQPQQYVPPQQQNHQIHPQVHPLQTMSQPQRGISPQDIPPPQMQGHFAAQPQY

>OTA53176.1 DEAD-domain-containing protein [Hypoxylon sp. EC38]

MADALAAKLRASTLNDTPQNDDWKKQLNIPARDSRQQTEDVTNTKGLEWEDFNLKRDLLMGIFEAGFEKPSPIQEESIPV

ALTGRDILARAKNGTGKTAAFVVPALERINPKVNKIQCLILVPTRELAMQTSQVCKTLGKHLGVNVMVTTGGTTLRDDIV

RLQDPVHIVVGTPGRILDLAGKNVADLSECPMFIMDEADKLLSIEFTPVIEQLLQFHPKDRQIMLFSATFPLSVKDFSDK

NMNRPYEINLMDELTLRGITQYYAFVEEKQKVHCLNTLFSKLQINQSIIFCNSTNRVELLAKKITELGYSCFYSHAKMAQ

HARNRVFHDFRNGVCRNLVCSDLLTRGIDIQAVNVVINFDFPKNAETYLHRIGRSGRYGHLGLAINLINWDDRFNLYNIE

KDLGTEIQPIPATIDKSLYVYENPESIPRPINTYTTKPMNSGALPQSNDQSSQQANNSQPSQVRGQGPQGGRGGHFSDSQ

SPSYRSDRGSNRGGGRGGNRGRGGRGYNNYSRGGGRGQSQQPQ

>XP_030988179.1 uncharacterized protein PgNI_00651 [Pyricularia grisea]

MADVLADQLRSATLSDATNNDDWRRNLNIPARDNRQQTEDVTNTKGLEFENFGLKRDLLMGIFEAGFEKPSPIQEESIPV

ALTGRDILARAKNGTGKTAAFVVPALEKINPKVSKIQCLILVPTRELAMQTSQVCKTLGKHLGINVMVTTGGTTLRDDIL

RLQDPVHIVVGTPGRILDLAGKNVADLSECPMFIMDEADKLLSIEFTPVIEQLLQFHPKDRQVMLFSATFPISVKEFSDK

NMTDPYEINLMDELTLRGITQYYAFVEEKQKVHCLNTLFSKLQINQSIIFCNSTNRVELLAKKITELGYSCFYSHAKMQQ

QARNRVFHDFRNGVCRNLVCSDLLTRGIDIQAVNVVINFDFPKNAETYLHRIGRSGRYGHLGLAINLISWEDRFNLYNIE

RDLGTEIQPIPSTIDKSLYVYENPETIPRPINIPAQPSSVGNAQSAAPNQGPPPQQQRPHQGQENWQNQNGRHNGANQQQ

QPRGPHQNRGRGGGRGRGGFQGQGQGQRNYNNNYRGGRGGHNQGQHQQQMPNQQS

>XP_023450874.1 ATP-dependent RNA helicase dhh1 [Cercospora beticola]

MADGLANKLEKTSLNEAAEGDWKSGLKAPPKDGRQQTEDVTATKGLDFEEFYLKRELLMGIYEAGFEKPSPIQEETIPVA

LSGRDILARAKNGTGKTAAFVIPTLERINPKLDKIQALLLVPTRELALQTSQVCKTLGKHLGINVMVTTGGTGLRDDIIR

LNDPVHIVVGTPGRILDLAGKGVADLSEAKTFVMDEADKLLSPEFTVTIEQLLKFHPKDRQVMLFSATFPVVVKDFKDKH

MNDPHEINLMDELTLRGITQYYAFVEEKQKVHCLNTLFSRLQINQSIIFCNSTTRVELLAKKITELGYSCFYSHAKMLQQ

HRNRVFHDFRNGAMRNLVCSDLLTRGIDIQAVNVVINFDFPKNAETYLHRIGRSGRFGHLGLAINLINWEDRFNLYRIEQ

ELGTEIQPIPGTIDKKLYVYDAPENIPRPINTAPPQQQQQQGQQAGRQQGSGQDLLNPANRTGGYRGGRGGGYGRGRGGP

RNDQNGFQGQQRQGGFQQRPPPQQQMAPAGPPLAQA

>GAP90605.1 putative ATP-dependent RNA helicase DHH1 [Rosellinia necatrix]

MSDSIASSLQASTINDADDNRVWKQSLSIPPRDNRQQTEDVTNTKGIEWEEFGLKRDLLMGIFEAGYEKPSPIQEEAIPV

ALTGRDILARAKNGTGKTAAFVVPTLERINPKVNKIQCLILVPTRELAMQTSQVCKTLGKHLGVNVMVTTGGTTLRDDIV

RLQDAVHIVVGTPGRILDLAGKGVADLSECPMFIMDEADKLLSIEFTPVIEQLLQFHPKDRQVMLFSATFPLSVKDFSDK

NMSKPYEINLMDELTLRGITQYYAFVEEKSKVHCLNTLFSKLQINQSIIFCNSTNRVELLAKKITELGYSCFYSHAKMAQ

HARNRVFHDFRNGVCRNLVCSDLLTRGIDIQAVNVVINFDFPKNAETYLHRIGRSGRYGHLGLAINLINWEDRYNLYNIE

KDLGTEIQAIPSTIDKALYVYDNPENIPRPVNTYTTKPVPANKFQTQQGEASTPTSTSGPQQNNPPTPQGRGQGDFRTQT

GRGGQFIDSQPRQYQNRGGQRGRGGRGRGFPQFNARGGGRGQPQTQPPQ

>PCH02268.1 Hypothetical protein PENO1_038600 [Penicillium sp. 'occitanis']

MVDAVTSQLSNTKLGETDGSWKDQLKIPAKDNRTQTEDVTATKGLEFEDFYIKRELMMGIFEAGFEKPSPIQEETIPVAL

TGRDILARAKNGTGKTAAFIIPTLERINPKSTKTQALILVPTRELALQTSQVCKTLGKHLGINVMVTTGGTGLMDDIIRL

NDTVHIIVGTPGRVLDLASKGVADLSECPTFVMDEADKLLSPEFTPVIEQLLSFHPKDRQVMLFSATFPMIVKSFKDKHM

RNPYEINLMDELTLRGITQYYAFVEEKQKVHCLNTLFSKLQINQSIIFCNSTNRVELLAKKITELGYSCFYSHARMLQHN

RNRVFHDFRNGVCRNLVCSDLLTRGIDIQAVNVVINFDFPKNAETYLHRIGRSGRFGHLGLAINLINWDDRFNLYKIEQE

LGTEIQPIPQSIDKKLYVYDSPDTIPRPIASAPQPGTEGQQREQQGYRQGGHHPGGRYNNNAGRGRGGFRGRGGQGQRRG

GYQTHGHDGSKPQPTPAS

>PYH95202.1 DEAD-domain-containing protein [Aspergillus ellipticus CBS 707.79]

MADALASQLNSATLGDASSDAKWKDQLNVPAKDARPQTEDVTATKGLEFEDFYIKRELMMGIFEAGFEKPSPIQEETIPV

ALTGRDILARAKNGTGKTAAFVIPTLERINPKSTKTQALILVPTRELALQTSHVCKTLGKHLGINVMVTTGGTGLMDDII

RLNDAVHILVGTPGRVLDLASKGVADLSECPTFVMDEADKLLSPEFTPVIEQLLSFHPKDRQVMLFSATFPLIVKAFKDK

HMRNPYEINLMDELTLRGITQYYAFVEEKQKVHCLNTLFSKLQINQSIIFCNSTNRVELLAKKITELGYSCFYSHARMLQ

QHRNRVFHDFRNGVCRNLVCSDLLTRGIDIQAVNVVINFDFPKNAETYLHRIGRSGRFGHLGLAINLINWEDRFNLYKIE

QELGTEIQPIPQNIDKKLYVYDSPDTIPRPISNPSQPHAAAAAPNPNPNTGERRHNHHTNSSGQYQYGRGRGSYRGGRGQ

GQRRSIQNETKFSGPPGQSGGKSYPTQVS

>KFY10046.1 hypothetical protein V492_05235 [Pseudogymnoascus sp. VKM F-4246]

MADALAGKLESTTLGDGQNNSDWKKNLKIPAKDNRQQTEDVTATKGLEFEEFSIKRDLLMGIFEAGFEKPSPIQEEAIPV

ALTGRDILARAKNGTGKTAAFVIPALERINPKSSKIQCLILVPTRELALQTSQVCKTLGKHLGVNVMVTTGGTGLRDDIV

RLAEPVHIVVGTPGRILDLAGKSVADLSECPMFIMDEADKLLSPEFTPVIEQLLQFHPKDRQIMLFSATFPRSVQAFSVK

NMDQPYEINLMDELTLRGITQYYAFVEEKQKVHCLNTLFSKLQINQSIIFCNSTNRVELLAKKITELGYSCFYSHARMLQ

ANRNRVFHDFRNGVCRNLVCSDLLTRGIDIQAVNVVINFDFPKNAETYLHRIGRSGRFGHLGLAINLINWDDRFNLYNIE

KELGTEIQPIPAMIDKNLYVYDSPESIPRPISNPAPRQQQQAQREQLQIQQRGQAPANYSNGRQGPSGQAQPQGQAQAQL

QQGQTQGQRQPSNQPQGQRNQNFSRGNARVGAQPGRGRGFDGARGGRGQAPPPS

>KUL83360.1 hypothetical protein ZTR_11239 [Talaromyces verruculosus]

MVDAVTSQLSNTKLGETDGSWKDQLKIPAKDNRTQTEDVTATKGLEFEDFYIKRELMMGIFEAGFEKPSPIQEETIPVAL

TGRDILARAKNGTGKTAAFIIPTLERINPKSTKTQALILVPTRELALQTSQVCKTLGKHLGINVMVTTGGTGLMDDIIRL

NDTVHIIVGTPGRVLDLASKGVADLSECPTFVMDEADKLLSPEFTPVIEQLLSFHPKDRQVMLFSATFPMIVKSFKDKHM

RNPYEINLMDELTLRGITQYYAFVEEKQKVHCLNTLFSKLQINQSIIFCNSTNRVELLAKKITELGYSCFYSHARMLQHN

RNRVFHDFRNGVCRNLVCSDLLTRGIDIQAVNVVINFDFPKNAETYLHRIGRSGRFGHLGLAINLINWDDRFNLYKIEQE

LGTEIQPIPQSIDKKLYVYDSPDTIPRPIASAPQPGTEGQQHEQQGYRQGGHHPGGRYNNNAGRGRGGFRGRGGQGQRRG

GYQTHGHDGSKPQPTPAS

>KFY29593.1 hypothetical protein V494_08638 [Pseudogymnoascus sp. VKM F-4513 (FW-928)]

MADALAGKLESTTLGDGQNNSDWKKNLKIPAKDNRQQTEDVTATKGLEFEEFSIKRDLLMGIFEAGFEKPSPIQEEAIPV

ALTGRDILARAKNGTGKTAAFVIPALERINPKSSKIQCLILVPTRELALQTSQVCKTLGKHLGVNVMVTTGGTGLRDDIV

RLAEPVHIVVGTPGRILDLAGKSVADLSECPMFIMDEADKLLSPEFTPVIEQLLQFHPKDRQIMLFSATFPRSVQAFSVK

NMDQPYEINLMDELTLRGITQYYAFVEEKQKVHCLNTLFSKLQINQSIIFCNSTNRVELLAKKITELGYSCFYSHARMLQ

ANRNRVFHDFRNGVCRNLVCSDLLTRGIDIQAVNVVINFDFPKNAETYLHRIGRSGRFGHLGLAINLINWDDRFNLYNIE

KELGTEIQPIPAMIDKNLYVYDSPESIPRPISNPAPRQQQQAQREQLQIQQRGQAPANYSNGRQGPSVQAQPQGQAQAQL

QQGQTQGQRQPSNQPQGQRNQNFSRGNARVGAQPGRGRGFDGARGGRGQTPPPS

>XP_007916727.1 putative atp-dependent rna helicase dhh1 protein [Phaeoacremonium minimum UCRPA7]

MADSLVDQLKASSLSGGGSDEWKKTLNIPAKDSRQQTEDVTNTKGMEFEEFGLKRDLLMGIFEAGFEKPSPIQEESIPVA

LTGRDILARAKNGTGKTAAFVIPALEKINPKVSKIQCLILVPTRELAMQTSQVCKTLGKHLGINVMVTTGGTGLRDDIVR

LQDPVHIVVGTPGRILDLAGKNVADLSECPMFIMDEADKLLSIEFTPVIEQLLKFHPKDRQVMLFSATFPLSVKDFSDKN

MVSPYEINLMDELTLRGITQYYAFVEEKQKVHCLNTLFSKLQINQSIIFCNSTNRVELLAKKITELGYSCFYSHAKMAQQ

ARNRVFHDFRNGVCRNLVCSDLLTRGIDIQAVNVVINFDFPKNAETYLHRIGRSGRYGHLGLAINLINWDDRFNLYNIER

DLGTEIQPIPQTIDKSLYVYENPETIPRPISNLNRGANASPAAQLPGLPQGQASSSQPRQPPNDWQGQNGRQNGQRQYQG

GRGQGGSRGGQGRGRGGGYQGQRQNYNGQRGGRGQGQPAPQV

>XP_007782284.1 ATP-dependent RNA helicase DHH1 [Coniosporium apollinis CBS 100218]

MASDITNQLAITTLNDPQPSDANWKDGLKIPSKDSRVQTEDVTATKGLEFEDFYIKRELMMGIFEAGFEKPSPIQEETIP

VALTGRDILARAKNGTGKTAAFVIPTLERINPKSTKTQALILVPTRELALQTSQVCKTLGKHLGINVMVTTGGTGLKDDI

IRLGETVHIIVGTPGRILDLASKGVADLSACQTFIMDEADKLLSPEFTPVIEQLLAFHPKDRQVMLFSATFPIVVKSFKD

KHMNSPYEINLMDELTLRGITQYYAFVEEKQKVHCLNTLFNKLQINQSIIFCNSTNRVELLAKKITELGYSCFYSHARML

QHARNRVFHDFRNGACRNLVCSDLLTRGIDIQAVNVVINFDFPKNAETYLHRIGRSGRFGHLGLAINLINWDDRFNLYKI

EQELGTEIQPIPQTIEKKLYVYESPENIPRPISSAPAQNQQQEEDGARRSNQRQGDNRQGFQQNQNQNRRGGRYQGQPRG

PPRNQNQQGQLNGFNGQRTPRPAPPPQT

>EJT43708.1 DHH1-like protein [Saccharomyces kudriavzevii IFO 1802]

MSSINKNFITNNNSNTDLDLDWKTALNIPKKDTRPQTDDVLNTKGNTFEDFYLKRELLMGIFEAGFEKPSPIQEEAIPVA

ITGRDILARAKNGTGKTAAFVIPTLERVKPKLNKIQALIMVPTRELALQTSQVVRTLGKHCGISCMVTTGGTNLRDDILR

LNETVHILVGTPGRVLDLASRKVADLSDCSLFIMDEADKMLSRDFKTIIEQILSFLPTTHQSLLFSATFPLTVKEFMVKH

LHKPYEINLMEELTLKGITQYYAFVEERQKLHCLNTLFSKLQINQAIIFCNSTNRVELLAKKITDLGYSCYYSHARMKQQ

ERNKVFHEFRQGKVRTLVCSDLLTRGIDIQAVNVVINFDFPKTAETYLHRIGRSGRFGHLGLAINLINWNDRFNLYKIEQ

ELGTEIAAIPATIDKSLYVAENDETVPVPFPIEQRSYQPQAASQQQLPPQQQFAIPPQQHHPQFMVPPPQHQQQQVYPPS

QIPPQQGYPPQQEHFMAMPPGQPQPQF

>TLD33013.1 hypothetical protein PspLS_01084 [Pyricularia sp. CBS 133598]

MADVLADQLRSATLSDATNNEDWRRNLNIPARDNRQQTEDVTNTKGLEFENFGLKRDLLMGIFEAGFEKPSPIQEESIPV

ALTGRDILARAKNGTGKTAAFVVPALEKINPKVSKIQCLILVPTRELAMQTSQVCKTLGKHLGINVMVTTGGTTLRDDIL

RLQDPVHIVVGTPGRILDLAGKNVADLSECPMFIMDEADKLLSIEFTPVIEQLLQFHPKDRQVMLFSATFPISVKEFSDK

NMTDPYEINLMDELTLRGITQYYAFVEEKQKVHCLNTLFSKLQINQSIIFCNSTNRVELLAKKITELGYSCFYSHAKMQQ

QARNRVFHDFRNGVCRNLVCSDLLTRGIDIQAVNVVINFDFPKNAETYLHRIGRSGRYGHLGLAINLISWEDRFNLYNIE

RDLGTEIQPIPSTIDKSLYVYDNPETIPRPINIPAQPSSAGNAQGSVPNQGPPPQQQRPHQGQENWQNQNGRHNGANQQQ

QPRGPHQNRGRGGGRGRGGFQGQGQGQRNYNNNYRGGRGGHNQGQHQQQMPNQQS

>KKK15663.1 ATP-dependent RNA helicase dhh1 [Aspergillus rambellii]

MADALAAQLNNTSLGDANSDAKWKEQLKLPAKDARPQTEDVTATKGLEFEDFYIKRELMMGIFEAGFEKPSPIQEETIPV

ALTGRDILARAKNGTGKTAAFVIPTLERINPKSIKTQALILVPTRELALQTSHVCKTLGKHLGINVMVTTGGTGLMDDII

RLNDAVHILVGTPGRVLDLASKGVADLSECPTFVMDEADKLLSPEFTPVIEQLLSFHPKDRQVMLFSATFPLIVKSFKDK

HMRNPYEINLMDELTLRGITQYYAFVEEKQKVHCLNTLFSKLQINQSIIFCNSTNRVELLAKKITELGYSCFYSHARMLQ

QHRNRVFHDFRNGVCRNLVCSDLLTRGIDIQAVNVVINFDFPKNAETYLHRIGRSGRFGHLGLAINLINWDDRFNLYKIE

QELGTEIQPIPQNIDKKLYVYDSPDTIPRPISNPSQPHQAPAATLNMNPGDRRHHNHPNGGQYQSNRGRGSYRGGRGQGQ

RRNIQNDGGRFSASQGQPSGKPQPATQVS

>XP_031903556.1 ATP-dependent RNA helicase dhh1 [Aspergillus alliaceus]

MADALASQLNNTTLGEASSDARWKDQLKAPAKDARPQTEDVTATKGLEFEDFYIKRELMMGIFEAGFEKPSPIQEETIPV

ALTGRDILARAKNGTGKTAAFVIPTLERINPKSTKTQALILVPTRELALQTSHVCKTLGKHLGINVMVTTGGTGLMDDII

RLNDAVHILVGTPGRVLDLASKGVADLSECPTFVMDEADKLLSPEFTPVIEQLLSFHPKDRQVMLFSATFPLIVKSFKDK

HMRNPYEINLMDELTLRGITQYYAFVEEKQKVHCLNTLFSKLQINQSIIFCNSTNRVELLAKKITELGYSCFYSHARMLQ

QHRNRVFHDFRNGVCRNLVCSDLLTRGIDIQAVNVVINFDFPKNAETYLHRIGRSGRFGHLGLAINLINWDDRFNLYKIE

QELGTEIQPIPQNIDKKLYVYESPETIPRPIANSSQTQLATNANLTQNPGERRHNNHLNGGHYQYGRTRGSYRGGRGQGQ

RRNIQNEMNKFGGSQNQQQSGKTQPAQVS

>XP_020056948.1 uncharacterized protein ASPACDRAFT_59467 [Aspergillus aculeatus ATCC 16872]

MADALASQMKSTSLGDTSSDAKWKEQLNVPAKDARPQTEDVTATKGLEFEDFYIKRELMMGIFEAGFEKPSPIQEETIPV

ALTGRDILARAKNGTGKTAAFVIPTLERINPKSTKTQALILVPTRELALQTSHVCKTLGKHLGINVMVTTGGTGLMDDII

RLNDAVHILVGTPGRVLDLASKGVADLSECPTFVMDEADKLLSPEFTPVIEQLLSFHPKDRQVMLFSATFPLIVKSFKDK

HMRNPYEINLMDELTLRGITQYYAFVEEKQKVHCLNTLFSKLQINQSIIFCNSTNRVELLAKKITELGYSCFYSHARMLQ

QHRNRVFHDFRNGVCRNLVCSDLLTRGIDIQAVNVVINFDFPKNAETYLHRIGRSGRFGHLGLAINLINWDDRFNLYKIE

QELGTEIQPIPQNIDKKLYVYDSPDTIPRPIANPSQPQINTTVTNAPAADRRPTHHANGGHYQFHRGRGSYRGGRGQGQR

RSIHNDAKQFSASQGQTGGKNHTTPVS

>PNS21280.1 ATP-dependent RNA helicase dhh1 [Sphaceloma murrayae]

MASELATQLGNTSLNDNGASSEDWKAGLKKPGKDGRQQTEDVTATKGLEFEDFYIKRELMMGIFEAGFEKPSPIQEETIP

VALTGRDILARAKNGTGKTASFVIPTLERVNPKNSKIQALLLVPTRELALQTSQVCKTLGKHLGINVMVTTGGTSLRDDI

IRLNETVHILVGTPGRILDLAAKGIADLSACQTFVMDEADKLLSPEFTSQIDQLLAFHPKDRQVMLFSATFPVIVKDFKD

KHMNDPHEINLMDELTLRGITQYYAFVEEKQKVHCLNTLFSRLQINQSIIFCNSTTRVELLAKKITELGYSCFYSHARML

QHARNRVFHDFRAGHCRNLVCSDLLTRGIDIQAVNVVINFDFPKNAETYLHRIGRSGRFGHLGLAINLINWEDRFNLYRI

EQELGTEIAPIPATIDKNLYVYDNPENIPRPISGAPQQRQPQGPSDNIRGRGGFQSRGGNRGRGGFQGQGRGPPRDQNDQ

RPQNGYAPRGPAQSRAPPAAAQS

>OBT48061.1 ATP-dependent RNA helicase DDX6/DHH1 [Pseudogymnoascus sp. WSF 3629]

MADALAGKLESTTLGSSTSSQNDPDWKKKLKIPVKDNRHQTEDVTATKGLEFEEFSIKRDLLMGIFEAGFEKPSPIQEEA

IPVALTGRDILARAKNGTGKTAAFVIPALERINPKSSKIQCLILVPTRELALQTSQVCKTLGKHLGVNVMVTTGGTGLRD

DIVRLAEPVHIVVGTPGRILDLAGKSVADLSECPMFIMDEADKLLSPEFTPVIEQLLQFHPKDRQIMLFSATFPRSVQAF

SVKNMDQPYEINLMDELTLRGITQYYAFVEEKQKVHCLNTLFSKLQINQSIIFCNSTNRVELLAKKITELGYSCFYSHAR

MLQANRNRVFHDFRNGVCRNLVCSDLLTRGIDIQAVNVVINFDFPKNAETYLHRIGRSGRFGHLGLAINLINWDDRFNLY

NIEKELGTEIQPIPAMIDKNLYVYDSPESIPRPISNPAPRQQPPREQLQIQQRGGQAPANYSNGRQGPSGQAQPQGQQAQ

AQAQAQAQLQQQQQQQQQQGQTQGQRQPSNQPQGQQRNQNFSRGNARVGAQPGRGRGFDGARGGRGQAPPPS

>XP_035350583.1 uncharacterized protein TRUGW13939_11584 [Talaromyces rugulosus]

MADTLATQLSNTKLGDANPEGAWKDTLKIPAKDTRTQTEDVTATKGLEFEDFYIKRELMMGIFEAGFEKPSPIQEETIPV

ALTGRDILARAKNGTGKTAAFIIPTLERINPKNTKTQALILVPTRELALQTSQVCKTLGKHLGINVMVTTGGTGLMDDII

RLNDAVHIIVGTPGRVLDLASKGVADLSECPTFVMDEADKLLSPEFTPVIEQLLSFHPKDRQVMLFSATFPMIVKSFKDK

HMRNPYEINLMDELTLRGITQYYAFVEEKQKVHCLNTLFSKLQINQSIIFCNSTNRVELLAKKITELGYSCFYSHARMLQ

QNRNRVFHDFRNGVCRNLVCSDLLTRGIDIQAVNVVINFDFPKNAETYLHRIGRSGRFGHLGLAINLINWEDRFNLYKIE

QELGTEIQPIPQAIDKKLYVYESPDTIPRPIASASQPRPAEAEYDQQNRRHGNYHTAGHFGAPNRGRGHYRGRGQGQRRG

GNHHDNHRSMGGYPHDNKPQPTPAS

>SMQ55317.1 unnamed protein product [Zymoseptoria tritici ST99CH_3D7]

MAAELASKLETTSLNDSSPAGGDWKAGLKAPAKDGRHQTEDVTATKGLEFEEFYLKRELLMGIFEAGFEKPSPIQEETIP

VALSGRDILARAKNGTGKTAAFVIPTLERINPKLDKVQALLLVPTRELALQTSQVCKTLGKHLGINVMVTTGGTGLRDDI

MRLAETVHIIVGTPGRILDLAGKGVADLSEAKTFVMDEADKLLSPEFTVTIEQLLKFHPKDRQVMLFSATFPVVVKEFKD

RHMNDPHEINLMDELTLRGITQYYAFVEEKQKVHCLNTLFSRLNINQSIIFCNSTTRVELLAKKITELGYSCFYSHAKML

QQHRNRVFHDFRNGAMRNLVCSDLLTRGIDIQAVNVVINFDFPKNAETYLHRIGRSGRFGHLGLAINLINWDDRFNLYRI

EQELGTEIQPIPPSIDKSLYVYDSPENIPRPINTAPQQPQSQQAGRQQGNGSDLQDPANRTSGGYRGGRGGGGFQGRGGG

FQGQGQGRGRGGPRNDQQYPQNGAQNGQNGFQGEQQQRQGGQYPARPNQQMPPAGPPAAQA

>KAE8375323.1 ATP-dependent RNA helicase dhh1 [Aspergillus bertholletiae]

MAEALASQLNNTTLGEASSDTRWKDQLKAPAKDARPQTEDVTATKGLEFEDFYIKRELMMGIFEAGFEKPSPIQEETIPV

ALTGRDILARAKNGTGKTAAFVIPTLERINPKSTKTQALILVPTRELALQTSHVCKTLGKHLGINVMVTTGGTGLMDDII

RLNDAVHILVGTPGRVLDLASKGVADLSECPTFVMDEADKLLSPEFTPVIEQLLSFHPKDRQVMLFSATFPLIVKSFKDK

HMRNPYEINLMDELTLRGITQYYAFVEEKQKVHCLNTLFSKLQINQSIIFCNSTNRVELLAKKITELGYSCFYSHARMLQ

QHRNRVFHDFRNGVCRNLVCSDLLTRGIDIQAVNVVINFDFPKNAETYLHRIGRSGRFGHLGLAINLINWDDRFNLYKIE

QELGTEIQPIPQNIDKKLYVYESPETIPRPIANASQTQLATSGNQNQNLGERRYNNHSNGGHYQFGRGRGSYRGGRGQGQ

RRNIQNEMNKFGASQSQSQVSPN

>XP_016757185.1 ATP-dependent RNA helicase DHH1 [Sphaerulina musiva SO2202]

MASELANKLGQTTLNDAETVGGDWRSGLKAPPKDGRQQTEDVTATKGLDFEEFYLKRELLMGIYEAGFEKPSPIQEETIP

VALSGRDILARAKNGTGKTAAFVIPTLERINPKVDKIQALLLVPTRELALQTSQVCKTLGKHLGINVMVTTGGTGLRDDI

IRLNEPVHIVVGTPGRILDLAGKGVADLSEAKTFVMDEADKLLSPEFTVTIEQLLKFHPKDRQVMLFSATFPVVVKEFKD

RHMNDPHEINLMDELTLRGITQYYAFVEEKQKVHCLNTLFSRLQINQSIIFCNSTTRVELLAKKITELGYSCFYSHAKML

QQHRNRVFHDFRNGAMRNLVCSDLLTRGIDIQAVNVVINFDFPKNAETYLHRIGRSGRFGHLGLAINLINWEDRFNLYRI

EQELGTEIQPIPGTIDKKLYVYDAPENIPRPINTAPQQQQSQQPGRQQGSGQDLLNPANRTGGYRGGRGGGGGGGHGRGR

GGPRNDQNGFQGQQRPGGFQQRPPQQQQMASAGPPVAQA

>KFX86920.1 hypothetical protein V490_08726 [Pseudogymnoascus sp. VKM F-3557]

MTDALAGKLESTTLGSTPNDADWKKKLKIPAKDNRQQTEDVTATKGLEFEEFSIKRDLLMGIFEAGFEKPSPIQEEAIPV

ALTGRDILARAKNGTGKTAAFVIPALERINTKSSKIQCLILVPTRELALQTSQVCKTLGKHLGVNVMVTTGGTGLRDDIV

RLAEPVHIVVGTPGRILDLAGKNVADLSECPMFIMDEADKLLSPEFTPVIEQLLQFHPKDRQIMLFSATFPRSVQAFSVK

NMDQPYEINLMDELTLRGITQYYAFVEEKQKVHCLNTLFSKLQINQSIIFCNSTNRVELLAKKITELGYSCFYSHARMLQ

ANRNRVFHDFRNGVCRNLVCSDLLTRGIDIQAVNVVINFDFPKNAETYLHRIGRSGRFGHLGLAINLINWDDRFNLYNIE

KELGTEIQPIPAMIDKNLYVYDSPESIPRPISNPAPRQQAQREQLQIQQRGQAPAANYSNGRQGPSAQAQPQNQAQAQAQ

AQAQAQLQQGQSQGQRQPTNQPQGQRNQNFSRGNARVGAQPGRGRGFEGARGGRGQAPPPS

>KAB8269652.1 ATP-dependent RNA helicase dhh1 [Aspergillus minisclerotigenes]

MAEALASQLNNTTLGEASSDTRWKDQLKAPAKDARPQTEDVTATKGLEFEDFYIKRELMMGIFEAGFEKPSPIQEETIPV

ALTGRDILARAKNGTGKTAAFVIPTLERINPKSTKTQALILVPTRELALQTSHVCKTLGKHLGINVMVTTGGTGLMDDII

RLNDAVHILVGTPGRVLDLASKGVADLSECPTFVMDEADKLLSPEFTPVIEQLLSFHPKDRQVMLFSATFPLIVKSFKDK

HMRNPYEINLMDELTLRGITQYYAFVEEKQKVHCLNTLFSKLQINQSIIFCNSTNRVELLAKKITELGYSCFYSHARMLQ

QHRNRVFHDFRNGVCRNLVCSDLLTRGIDIQAVNVVINFDFPKNAETYLHRIGRSGRFGHLGLAINLINWDDRFNLYKIE

QELGTEIQPIPQNIDKKLYVYESPETIPRPIANASQAQLATSGNQSQNMGERRHNNHSNGGHYQFGRGRGSYRGGRSQGQ

RRNMQNEMNKFGTSQNQQQSGKSQPAQVSPN

>XP_003848580.1 uncharacterized protein MYCGRDRAFT_101609 [Zymoseptoria tritici IPO323]

MAAELASKLETTSLNDSSPAGGDWKAGLKAPAKDGRHQTEDVTATKGLEFEEFYLKRELLMGIFEAGFEKPSPIQEETIP

VALSGRDILARAKNGTGKTAAFVIPTLERINPKLDKVQALLLVPTRELALQTSQVCKTLGKHLGINVMVTTGGTGLRDDI

MRLAETVHIIVGTPGRILDLAGKGVADLSEAKTFVMDEADKLLSPEFTVTIEQLLKFHPKDRQVMLFSATFPVVVKEFKD

RHMNDPHEINLMDELTLRGITQYYAFVEEKQKVHCLNTLFSRLNINQSIIFCNSTTRVELLAKKITELGYSCFYSHAKML

QQHRNRVFHDFRNGAMRNLVCSDLLTRGIDIQAVNVVINFDFPKNAETYLHRIGRSGRFGHLGLAINLINWDDRFNLYRI

EQELGTEIQPIPPSIDKSLYVYDSPENIPRPINTAPQQPQSQQAGRQQGNGSDLQDPANRTSGGYRGGRGGGGFQGQQQQ

RQGGQYPARPNQQMPPAGPPAAQA

>KIW69375.1 ATP-dependent RNA helicase DHH1 [Phialophora americana]

MTDALAAQLNKTSLNDGADESTWKDSLKLPAKDTRQQTEDVTATKGLEFEDFYIKRELMMGIFEAGFEKPSPIQEETIPV

ALTGRDILARAKNGTGKTAAFVIPTLERINPKNPKTQALILVPTRELALQTSQVCKTLGKHLGINVMVTTGGTGLKDDIL

RLGEPVHIIVGTPGRILDLASKGVADLSECPIFVMDEADKLLSPEFTVVIEQLLSFHPKDRQVMLFSATFPMIVKSFKDK

HMRNPYEINLMDELTLRGITQYYAFVEEKQKVHCLNTLFSKLQINQSIIFCNSTNRVELLAKKITELGYSCFYSHAKMLQ

QNRNKVFHDFRAGVSRNLVCSDLLTRGIDIQAVNVVINFDFPKNAETYLHRIGRSGRFGHRGLAINLINWDDRYNLYKIE

QELGTEIQPIPPSIDKSLYVYDNPENIPRALPPPAPAGKPNTSTAPANQQATNANGQYPQYRRSQNQFNGFQQQYNNNQG

RGGYRGRGRGQGARGRGGHPQQAAAAAVGQTAQ

>PPJ55586.1 hypothetical protein CBER1_03722 [Cercospora berteroae]

MADGLANKLEKTSLNEAAEGDWKSGLKAPPKDGRQQTEDVTATKGLDFEEFYLKRELLMGIYEAGFEKPSPIQEETIPVA

LAGRDVLARAKNGTGKTAAFVIPTLERINPKLDKIQALLLVPTRELALQTSQVCKTLGKHLGINVMVTTGGTGLRDDIIR

LNDPVHIVVGTPGRILDLAGKGVADLSEAKTFVMDEADKLLSPEFTVTIEQLLKFHPKDRQVMLFSATFPVVVKDFKDKH

MNDPHEINLMDELTLRGITQYYAFVEEKQKVHCLNTLFSRLQINQSIIFCNSTTRVELLAKKITELGYSCFYSHAKMLQQ

HRNRVFHDFRNGAMRNLVCSDLLTRGIDIQAVNVVINFDFPKNAETYLHRIGRSGRFGHLGLAINLINWEDRFNLYRIEQ

ELGTEIQPIPGTIDKKLYVYDAPENIPRPINTAPPQQQQLQQGQQAGRQQGSGQDLLNPANRTGGYRGGRGGSYGRGRGG

PRNDQNGFQGQQRQGGFQQRPPPQQQMAPAGPPLAQA

>KAF2769009.1 DEAD-domain-containing protein [Teratosphaeria nubilosa]

MASELANKLEATTLSEKPTDNGDWKSGLNKPAKDGRQQTEDVTATKGLDFEDFYLKRELLMGIFEAGFEKPSPIQEETIP

VALTGRDILARAKNGTGKTAAFVIPTLERINPKLDKTQALILVPTRELALQTSQVMKTLGKHLGLNIMVTTGGTGLKDDI

MRLNDTVHVIVGTPGRILDLASKGVADLSAANTFVMDEADKLLSPEFTSTIEQLLAFHPKDRQVMLFSATFPVIVKSFME

KHMRDPHEINLMDELTLRGITQYYAFVEEKQKVHCLNTLFSRLQINQSIIFCNSTTRVELLAKKITELGYSCYYSHAKML

QQHRNRVFHDFRNGAMRNLVCSDLLTRGIDIQAVNVVINFDFPKNAETYLHRIGRSGRFGHLGLAINLINWEDRFNLYRI

EQELQTEIQPIPASIDKKLYVYDSPENIPRPINTAPQGQRPQIEGRKQQGNGADLQNPANRTSGYRGGRGGGRGGGYRQQ

RQGGYPQRPPPAQQTPAQA

>KFX98410.1 hypothetical protein O988_04359 [Pseudogymnoascus sp. VKM F-3808]

MTDALAGKLESTTLGSTPNDADWKKKLKIPAKDNRQQTEDVTATKGLEFEEFSIKRDLLMGIFEAGFEKPSPIQEEAIPV

ALTGRDILARAKNGTGKTAAFVIPALERINTKSSKIQCLILVPTRELALQTSQVCKTLGKHLGVNVMVTTGGTGLRDDIV

RLAEPVHIVVGTPGRILDLAGKNVADLSECPMFIMDEADKLLSPEFTPVIEQLLQFHPKDRQIMLFSATFPRSVQAFSVK

NMDQPYEINLMDELTLRGITQYYAFVEEKQKVHCLNTLFSKLQINQSIIFCNSTNRVELLAKKITELGYSCFYSHARMLQ

ANRNRVFHDFRNGVCRNLVCSDLLTRGIDIQAVNVVINFDFPKNAETYLHRIGRSGRFGHLGLAINLINWDDRFNLYNIE

KELGTEIQPIPAMIDKNLYVYDSPESIPRPISNPAPRQQAQREQLQIQQRGQAPAANYSNGRQGPSGQAQPQNQAQAQAQ

AQAQAQLQQGQSQGQRQPTNQPQGQRNQNFSRGNARVGAQPGRGRGFDGARGGRGQAPPPS

>KFY37430.1 hypothetical protein V495_07181 [Pseudogymnoascus sp. VKM F-4514 (FW-929)]

MTDALAGKLESTTLGSTPNDADWKKKLKIPAKDNRQQTEDVTATKGLEFEEFSIKRDLLMGIFEAGFEKPSPIQEEAIPV

ALTGRDILARAKNGTGKTAAFVIPALERINTKSSKIQCLILVPTRELALQTSQVCKTLGKHLGVNVMVTTGGTGLRDDIV

RLAEPVHIVVGTPGRILDLAGKNVADLSECPMFIMDEADKLLSPEFTPVIEQLLQFHPKDRQIMLFSATFPRSVQAFSVK

NMDQPYEINLMDELTLRGITQYYAFVEEKQKVHCLNTLFSKLQINQSIIFCNSTNRVELLAKKITELGYSCFYSHARMLQ

ANRNRVFHDFRNGVCRNLVCSDLLTRGIDIQAVNVVINFDFPKNAETYLHRIGRSGRFGHLGLAINLINWDDRFNLYNIE

KELGTEIQPIPAMIDKNLYVYDSPESIPRPISNPAPRQQAQREQLQIQQRGQAPAANYSNGRQGPSGQAQPQNQAQAQLQ

QGQSQGQRQPTNQPQGQRNQNFSRGNARVGAQPGRGRGFEGARGGRGQAPPSS

>XP_001646418.1 hypothetical protein Kpol_2001p67 [Vanderwaltozyma polyspora DSM 70294]

MSTQIEDQDWKSKLNIPKKDTRPQTEDVLSTKGNTFEDFYLKRELLMGIFEAGFEKPSPIQEESIPVALAGRDILARAKN

GTGKTAAFVIPTLEKVKSKHNKIQALIMVPTRELALQTSQVVRTLGKHCGISCMVTTGGTNLRDDILRLNETVHILVGTP

GRVLDLASRKVADLSECNLFIMDEADKMLSRDFKSIIEQILVFLPKNHQSLLFSATFPLSVKEFMVNHLHKPYEINLMDE

LTLKGITQYYAFVEEKQKLHCLNTLFSKLQINQAIIFCNSTNRVELLAKKITDLGYSCYYSHARMKQQERNRVFHEFRQG

KVRTLVCSDLLTRGIDIQAVNVVINFDFPKTAETYLHRIGRSGRFGHLGLAINLINWNDRFNLYKIEQELGTEIQAIPAT

IDKSLYVANDNSAVPVPFPIEQSHVQPSMQQQHMPLPPQQPIPNQQFGGMPQQYSPQQQQYQQYPPQGMAMPPQGIQMQQ

QPQF

>SMR60527.1 unnamed protein product [Zymoseptoria tritici ST99CH_1E4]

MAAELASKLEATSLNDSSPAGGDWKAGLKAPAKDGRHQTEDVTATKGLEFEEFYLKRELLMGIFEAGFEKPSPIQEETIP

VALSGRDILARAKNGTGKTAAFVIPTLERINPKLDKVQALLLVPTRELALQTSQVCKTLGKHLGINVMVTTGGTGLRDDI

MRLAETVHIIVGTPGRILDLAGKGVADLSEAKTFVMDEADKLLSPEFTVTIEQLLKFHPKDRQVMLFSATFPVVVKEFKD

RHMNDPHEINLMDELTLRGITQYYAFVEEKQKVHCLNTLFSRLNINQSIIFCNSTTRVELLAKKITELGYSCFYSHAKML

QQHRNRVFHDFRNGAMRNLVCSDLLTRGIDIQAVNVVINFDFPKNAETYLHRIGRSGRFGHLGLAINLINWDDRFNLYRI

EQELGTEIQPIPPSIDKSLYVYDSPENIPRPINTAPQQPQSQQAGRQQGNGSDLQDPANRTSGGYRGGRGGGGFQGRGGG

FQGQGQGRGRGGPRNDQQYPQNGAQNGQNGFQGQQQQRQGGQYPARPNQQMPPAGPPAAQA

>XP_033543541.1 ATP-dependent RNA helicase DHH1 [Lindgomyces ingoldianus]

MTSEITNQLAATQLSGEAPASDANWKDGLKLPSKDVRPQTEDVTATKGLEFEDFFIKRELMMGIFEAGFEKPSPIQEETI

PVALTGRDILARAKNGTGKTAAFVIPTLERVNPKSNKTQALILVPTRELALQTSQVCKTLGKHLNLNVMVSTGGTGLKDD

IIRLADPVHIIVGTPGRILDLAGKGVADLSACQTFVMDEADKLLSPEFTPVVEQLLGFHPKDRQVMLFSATFPIVVKAFK

DKHMNQPYEINLMDELTLRGITQYYAFVEEKQKVHCLNTLFNKLQINQSIIFCNSTNRVELLAKKITELGYSCFYSHAKM

LQHNRNRVFHDFRNGVCRNLVCSDLLTRGIDIQAVNVVINFDFPKNAETYLHRIGRSGRFGHLGLAINLINWEDRFNLYR

IEQELGTEIQPIPQVIEKNLYVYESPDTIPRPISNPQRPGQKQEQDGSGNTVRSNPAQYNNRQNHNRNQRGGGGQFQRRG

PAQNQQTRQNGQHPQRNPRPQPAGPA

>XP_035366274.1 ATP-dependent RNA helicase dhh1 [Lasiodiplodia theobromae]

MAADITNQLAAASLNESSPDWKNGLKAPPKDNRVQTEDVTATKGLEFEDFYIKRELMMGIFEAGFEKPSPIQEETIPVAL

TGRDILARAKNGTGKTAAFVIPTLERINPKSTKTQALILVPTRELALQTSQVCKTLGKHLGINVMVTTGGTGLKDDIIRL

SEPVHIIVGTPGRILDLASKGVADLSACPTFVMDEADKLLSPEFTPVIEQLLAFHPKDRQVMLFSATFPIVVKSFKDKHM

NQPYEINLMDELTLRGITQYYAFVEEKQKVHCLNTLFNKLQINQSIIFCNSTNRVELLAKKITELGYSCFYSHAKMLQHA

RNRVFHDFRNGVCRNLVCSDLLTRGIDIQAVNVVINFDFPKNAETYLHRIGRSGRFGHLGLAINLINWDDRFNLYKIEQE

LGTEIQPIPQTIEKKLYVYESPESIPRPISNAPANRPPPQQPSQPQQFPQPQQGQPQDQDPNAPRGNAQHGGRGGYRGPR

GGFQGQRRGGGQRNGYNGQRAPRPQA

>KAF2500075.1 DEAD-domain-containing protein [Lophium mytilinum]

MTTDITNQLGAVKLGEPASETSWKDGLKIPSKDARPQTEDVTATKGLEFEDFYIKRELMMGIFEAGFEKPSPIQEEAIPV

ALTGRDILARAKNGTGKTAAFVIPTLERINPKSTQVQALILVPTRELALQTSQVCKMLGKHLGLNVMVTTGGTGLKDDII

RLQETVHIIIGTPGRILDLASKGVAVLSGCQTFVMDEADKLLSPEFTPVIEQLLAFHPKDRQVMLFSATFPIVVKHFKDK

HMNSPYEINLMDELTLRGITQYYAFVEEKQKVHCLNTLFNKLQINQSIIFCNSTNRVELLAKKITELGYSCFYSHARMLQ

HNRNRVFHDFRNGVCRNLVCSDLLTRGIDIQAVNVVINFDFPKNAETYLHRIGRSGRFGHLGLAINLINWDDRFNLYRIE

QELGTEIQPIPQTIEKKLYVYESPENIPRPISNPPAPRTQGSGQTQDQDDSNVPRSNSQANNRQNFQRNQRGGAQFQGQR

RGGPPSNQQNRQNGHNAQRTARPPPAAPA

>KAF7118315.1 hypothetical protein CNMCM5793_007827 [Aspergillus hiratsukae]

MADTLANQLNNTSLGEANSEMRWKEQLNMPAKDARPQTEDVTATKGLEFEDFYIKRELMMGIFEAGFEKPSPIQEETIPV

ALTGRDILARAKNGTGKTAAFVIPTLERINPKSTKTQALILVPTRELALQTSQVCKTLGKHLGINVMVTTGGTGLMDDII

RLNDAVHILVGTPGRVLDLASKGVADLSECPTFVMDEADKLLSPEFTPVIEQLLSFHPKDRQVMLFSATFPLIVKSFKDK

HMRNPYEINLMDELTLRGITQYYAFVEEKQKVHCLNTLFSKLQINQSIIFCNSTNRVELLAKKITELGYSCFYSHARMLQ

QHRNRVFHDFRNGVCRNLVCSDLLTRGIDIQAVNVVINFDFPKNAETYLHRIGRSGRFGHLGLAINLINWDDRFNLYKIE

QELGTEIQPIPQNIDKKLYVYDSPETIPRPISNPSQPRQIANTPANTSTGDRRHHNHPNSGQYQFNRGRGSYRGRGQGQR

RSGQTETNKFGHPQGQHSGKTTTAPVS

>KFY25729.1 hypothetical protein V491_01616 [Pseudogymnoascus sp. VKM F-3775]

MTDALAGKLESTTLSDSQNSDWKKNLKIPAKDNRQQTEDVTATKGLEFEEFSIKRDLLMGIFEAGFEKPSPIQEEAIPVA

LTGRDILARAKNGTGKTAAFVIPALERINPKSSKIQCLILVPTRELALQTSQVCKTLGKHLGVNVMVTTGGTGLRDDIVR

LAEPVHIVVGTPGRILDLAGKSVADLSECPMFIMDEADKLLSPEFTPVIEQLLQFHPKDRQIMLFSATFPRSVQAFSVKN

MDQPYEINLMDELTLRGITQYYAFVEEKQKVHCLNTLFSKLQINQSIIFCNSTNRVELLAKKITELGYSCFYSHARMLQA

NRNRVFHDFRNGVCRNLVCSDLLTRGIDIQAVNVVINFDFPKNAETYLHRIGRSGRFGHLGLAINLINWDDRFNLYNIEK

ELGTEIQPIPAMIDKNLYVYDSPESIPRPISNPAPRPQAQREQLQIQQRGGQAPANYSNGRQGPSGQAQPQNQAQAQAQA

QAQAQAQAQAQLQQGQSQGQRQPSNQPQGQRNQNFSRGNARVGAQSGRGRGFDGARGGRGQAPPPS
