# Supplementary material for: DDX6 Is Essential for Oocyte Development and Maturation in Locusta migratoria
Source: Insects. 2021 Jan 14;12(1):70. doi: 10.3390/insects12010070 (PMC7830464; doi:10.3390/insects12010070)
Supplement: Supplementary file 1 [file insects-12-00070-s001.zip › Supplementary material/Supplemental file 5.docx]

**500 sequences of DDX6 from vertebrates**

>XP_006642205.1 PREDICTED: probable ATP-dependent RNA helicase DDX6 [Lepisosteus oculatus]

MSTARTENPVILGLSSQNGQIRGPVKPAGGPGGGGGGPPSQPSGQMKTSSAINNGSSQLLPPANTAIKPGDDWKKNLKLP

PKDMRMKTSDVTATKGNEFEDYCLKRELLMGIFEMGWEKPSPIQEESIPIALSGRDILARAKNGTGKSGAYLIPLLERID

LKRDCIQAVVIVPTRELALQVSQICIQVSKHMGGVKVMATTGGTNLRDDIMRLDETVHVVIATPGRILDLIKKGVAKVGQ

VQMIVLDEADKLLSQDFVQMMEEILSTLPKNRQILLYSATFPLSVQKFMNAHLQKPYEINLMEELTLKGVTQYYAYVTER

QKVHCLNTLFSRLQINQSIIFCNSSQRVELLAKKISQLGYSCFYIHAKMRQEHRNRVFHDFRNGLCRNLVCTDLFTRGID

IQAVNVVINFDFPKLAETYLHRIGRSGRFGHLGLAINLITYDDRFNLKGIEEQLGTEIKPIPGSIDKSLYVAEYHSESGE

EAKL

>XP_018430586.1 PREDICTED: probable ATP-dependent RNA helicase DDX6 [Nanorana parkeri]

MSTTRTENPVLMGLSSQNGQLRGPVKQSQGPGNGGTPTLQINQLKNASTINNGSQQQAQSMGSGIKPGDDWKKTLKLPPK

DLRIKTSDVTSTKGNEFEDYCLKRELLMGIFEMGWEKPSPIQEESIPIALSGRDILARAKNGTGKSGAYLIPLLERLDLK

KDCIQAMVIVPTRELALQVSQICIQVSKHLGGVKVMATTGGTNLRDDIMRLDDTVHVVIATPGRILDLIKKGVAKVDHIQ

MIVLDEADKLLSQDFVQIMEDIISTLPKNRQILLYSATFPLSVQKFMSSHLQKPYEINLMEELTLKGVTQYYAYVTERQK

VHCLNTLFSRLQINQSIIFCNSSQRVELLAKKISQLGYSCFYIHAKMRQEHRNRVFHDFRNGLCRNLVCTDLFTRGIDIQ

AVNVVINFDFPKLAETYLHRIGRSGRFGHLGLAINLITYDDRFNLKSIEEQLGTEIKPIPSSIDKSLYVAEYHSESGEEK

R

>XP_035411543.1 probable ATP-dependent RNA helicase DDX6 isoform X1 [Cygnus atratus]

MSTARTENPVIMGLSSQNGQLRGPVKPSGGPGGGGAQTQQQMNQLKNANTINNGTQQQAQSMTTAIKPGDDWKKTLKLPP

KDLRIKTSDVTSTKGNEFEDYCLKRELLMGIFEMGWEKPSPIQEESIPIALSGRDILARAKNGTGKSGAYLIPLLERLDL

KKDNIQAMVIVPTRELALQVSQICIQVSKHMGGAKVMATTGGTNLRDDIMRLDDTVHVVIATPGRILDLIKKGVAKVEHV

QMIVLDEADKLLSQDFVQIMEDIILTLPKNRQILLYSATFPLSVQKFMNSHLQKPYEINLMEELTLKGVTQYYAYVTERQ

KVHCLNTLFSRLQINQSIIFCNSSQRVELLAKKISQLGYSCFYIHAKMRQEHRNRVFHDFRNGLCRNLVCTDLFTRGIDI

QAVNVVINFDFPKLAETYLHRIGRSGRFGHLGLAINLITYDDRFNLKSIEEQLGTEIKPIPSNIDKSLYVAEYHSEPVED

EKQ

>XP_027555052.1 probable ATP-dependent RNA helicase DDX6 [Neopelma chrysocephalum]

MSTARTENPVIMGLSSQNGQLRGPVKPSGGPGGGGTQTQQQMNQLKNANTINNGTQAQSMTTAIKPGDDWKKTLKLPPKD

LRIKTSDVTSTKGNEFEDYCLKRELLMGIFEMGWEKPSPIQEESIPIALSGRDILARAKNGTGKSGAYLIPLLERLDLKK

DNIQAMVIVPTRELALQVSQICIQVSKHMGGAKVMATTGGTNLRDDIMRLDDTVHVVIATPGRILDLIKKGVAKVEHVQM

IVLDEADKLLSQDFVQIMEDIILTLPKNRQILLYSATFPLSVQKFMNSHLQKPYEINLMEELTLKGVTQYYAYVTERQKV

HCLNTLFSRLQINQSIIFCNSSQRVELLAKKISQLGYSCFYIHAKMRQEHRNRVFHDFRNGLCRNLVCTDLFTRGIDIQA

VNVVINFDFPKLAETYLHRIGRSGRFGHLGLAINLITYDDRFNLKSIEEQLGTEIKPIPSNIDKSLYVAEYHSEPVEDEK

Q

>XP_009906613.1 PREDICTED: probable ATP-dependent RNA helicase DDX6 [Picoides pubescens]

MSTARTENPVIMGLSSQNGQLRGPVKPSGGPGGGGPPTQQQMNQLKNANTINNGTQQQAQSMTTAIKPGDDWKKTLKLPP

KDLRIKTSDVTSTKGNEFEDYCLKRELLMGIFEMGWEKPSPIQEESIPIALSGRDILARAKNGTGKSGAYLIPLLERLDL

KKDNIQAMVIVPTRELALQVSQICIQVSKHMGGAKVMATTGGTNLRDDIMRLDDTVHVVIATPGRILDLIKKGVAKVEHV

QMIVLDEADKLLSQDFVQIMEDIILTLPKNRQILLYSATFPLSVQKFMNSHLQKPYEINLMEELTLKGVTQYYAYVTERQ

KVHCLNTLFSRLQINQSIIFCNSSQRVELLAKKISQLGYSCFYIHAKMRQEHRNRVFHDFRNGLCRNLVCTDLFTRGIDI

QAVNVVINFDFPKLAETYLHRIGRSGRFGHLGLAINLITYDDRFNLKSIEEQLGTEIKPIPSNIDKSLYVAEYHSEPVED

EKQ

>XP_009096031.1 probable ATP-dependent RNA helicase DDX6 [Serinus canaria]

MSTARTENPVIMGLSSQNGQLRGPVKPSGGPGGGGTQTQQQMNQLKNANTINNGTQQQAQSMTTTIKPGDDWKKTLKLPP

KDLRIKTSDVTSTKGNEFEDYCLKRELLMGIFEMGWEKPSPIQEESIPIALSGRDILARAKNGTGKSGAYLIPLLERLDL

KKDNIQAMVIVPTRELALQVSQICIQVSKHMGGAKVMATTGGTNLRDDIMRLDDTVHVVIATPGRILDLIKKGVAKVEHV

QMIVLDEADKLLSQDFVQIMEDIILTLPKNRQILLYSATFPLSVQKFMNSHLQKPYEINLMEELTLKGVTQYYAYVTERQ

KVHCLNTLFSRLQINQSIIFCNSSQRVELLAKKISQLGYSCFYIHAKMRQEHRNRVFHDFRNGLCRNLVCTDLFTRGIDI

QAVNVVINFDFPKLAETYLHRIGRSGRFGHLGLAINLITYDDRFNLKSIEEQLGTEIKPIPSNIDKSLYVAEYHSEPVEE

EKQ

>XP_010073394.1 PREDICTED: probable ATP-dependent RNA helicase DDX6 [Pterocles gutturalis]

MSTARTENPVIMGLSSQNGQLRGPVKPSGGPGGGGTQTQQQMNQLKNTNTINNGTQQQAQSMTTTIKPGDDWKKTLKLPP

KDLRIKTSDVTSTKGNEFEDYCLKRELLMGIFEMGWEKPSPIQEESIPIALSGRDILARAKNGTGKSGAYLIPLLERLDL

KKDNIQAMVIVPTRELALQVSQICIQVSKHMGGAKVMATTGGTNLRDDIMRLDDTVHVVIATPGRILDLIKKGVAKVEHV

QMIVLDEADKLLSQDFVQIMEDIILTLPKNRQILLYSATFPLSVQKFMNSHLQKPYEINLMEELTLKGVTQYYAYVTERQ

KVHCLNTLFSRLQINQSIIFCNSSQRVELLAKKISQLGYSCFYIHAKMRQEHRNRVFHDFRNGLCRNLVCTDLFTRGIDI

QAVNVVINFDFPKLAETYLHRIGRSGRFGHLGLAINLITYDDRFNLKSIEEQLGTEIKPIPSNIDKSLYVAEYHSEPVED

EKQ

>XP_032633047.1 probable ATP-dependent RNA helicase DDX6 [Chelonoidis abingdonii]

MSTARTENPVIMGLSSQNGQLRGPVKPSGGPGGGGTQIQQQMNQLKNTNTINNGTQQQAQSMTTAIKPGDDWKKTLKLPP

KDLRIKTSDVTSTKGNEFEDYCLKRELLMGIFEMGWEKPSPIQEESIPIALSGRDILARAKNGTGKSGAYLIPLLERLDL

KKDNIQAMVIVPTRELALQVSQICIQVSKHMGGAKVMATTGGTNLRDDIMRLDDTVHVVIATPGRILDLIKKGVAKVEHV

QMIVLDEADKLLSQDFVQIMEDIILTLPKNRQILLYSATFPLSVQKFMNSHLQKPYEINLMEELTLKGVTQYYAYVTERQ

KVHCLNTLFSRLQINQSIIFCNSSQRVELLAKKISQLGYSCFYIHAKMRQEHRNRVFHDFRNGLCRNLVCTDLFTRGIDI

QAVNVVINFDFPKLAETYLHRIGRSGRFGHLGLAINLITYDDRFNLKSIEEQLGTEIKPIPSNIDKSLYVAEYHTEPVDD

EKP

>NP_001268976.1 probable ATP-dependent RNA helicase DDX6 [Gallus gallus]

MSTARTENPVIMGLSSQNGQLRGPVKPSGGPGGGGTQTQQQMNQLKNANTINNGTQQQAQSMTTTIKPGDDWKKTLKLPP

KDLRIKTSDVTSTKGNEFEDYCLKRELLMGIFEMGWEKPSPIQEESIPIALSGRDILARAKNGTGKSGAYLIPLLERLDL

KKDNIQAMVIVPTRELALQVSQICIQVSKHMGGAKVMATTGGTNLRDDIMRLDDTVHVVIATPGRILDLIKKGVAKVEHV

QMIVLDEADKLLSQDFVQIMEDIILTLPKNRQILLYSATFPLSVQKFMNSHLQKPYEINLMEELTLKGVTQYYAYVTERQ

KVHCLNTLFSRLQINQSIIFCNSSQRVELLAKKISQLGYSCFYIHAKMRQEHRNRVFHDFRNGLCRNLVCTDLFTRGIDI

QAVNVVINFDFPKLAETYLHRIGRSGRFGHLGLAINLITYDDRFNLKSIEEQLGTEIKPIPSNIDKSLYVAEYHSEPVED

EKQ

>NXM30017.1 DDX6 helicase [Oxyruncus cristatus]

MSTARTENPVIMGLSSQNGQLRGPVKPSGGPGGGGTQTQQQMNQLKNVNTINNGTQQQAQSMTTAIKPGDDWKKTLKLPP

KDLRIKTSDVTSTKGNEFEDYCLKRELLMGIFEMGWEKPSPIQEESIPIALSGRDILARAKNGTGKSGAYLIPLLERLDL

KKDNIQAMVIVPTRELALQVSQICIQVSKHMGGAKVMATTGGTNLRDDIMRLDDTVHVVIATPGRILDLIKKGVAKVEHV

QMIVLDEANKLLSQDFVQIMEDIILTLPKNRQILLYSATFPLSVQKFMNSHLQKPYEINLMEELTLKGVTQYYAYVTERQ

KVHCLNTLFSRLQINQSIIFCNSSQRVELLAKKISQLGYSCFYIHAKMRQEHRNRVFHDFRNGLCRNLVCTDLFTRGIDI

QAVNVVINFDFPKLAETYLHRIGRSGRFGHLGLAINLITYDDRFNLKSIEEQLGTEIKPIPSNIDKSLYVAEYHSEPVED

EKQ

>XP_007523665.1 PREDICTED: probable ATP-dependent RNA helicase DDX6 [Erinaceus europaeus]

MSTARTENPVIMGLSSQNGQLRGPVKPSGGPGGGGTQTQQQMSQLKNTNTINNGTQQQAQSMTTTIKPGDDWKKTLKLPP

KDLRIKTSDVTSTKGNEFEDYCLKRELLMGIFEMGWEKPSPIQEESIPIALSGRDILARAKNGTGKSGAYLIPLLERLDL

KKDNIQAMVIVPTRELALQVSQICIQVSKHMGGAKVMATTGGTNLRDDIMRLDDTVHVVIATPGRILDLIKKGVAKVDHV

QMIVLDEADKLLSQDFVQIMEDIILTLPKNRQILLYSATFPLSVQKFMNSHLQKPYEINLMEELTLKGVTQYYAYVTERQ

KVHCLNTLFSRLQINQSIIFCNSSQRVELLAKKISQLGYSCFYIHAKMRQEHRNRVFHDFRNGLCRNLVCTDLFTRGIDI

QAVNVVINFDFPKLAETYLHRIGRSGRFGHLGLAINLITYDDRFNLKSIEEQLGTEIKPIPSNIDKSLYVAEYHSEPVED

EKP

>XP_007062450.1 probable ATP-dependent RNA helicase DDX6 [Chelonia mydas]

MSTARTENPVIMGLSSQNGQLRGPVKPSGGPGGGGTQTQQQMNQLKNTNTINNGTQQQAQSMTTAIKPGDDWKKTLKLPP

KDLRIKTSDVTSTKGNEFEDYCLKRELLMGIFEMGWEKPSPIQEESIPIALSGRDILARAKNGTGKSGAYLIPLLERLDL

KKDNIQAMVIVPTRELALQVSQICIQVSKHMGGAKVMATTGGTNLRDDIMRLDDTVHVVIATPGRILDLIKKGVAKVEHV

QMIVLDEADKLLSQDFVQIMEDIILTLPKNRQILLYSATFPLSVQKFMNSHLQKPYEINLMEELTLKGVTQYYAYVTERQ

KVHCLNTLFSRLQINQSIIFCNSSQRVELLAKKISQLGYSCFYIHAKMRQEHRNRVFHDFRNGLCRNLVCTDLFTRGIDI

QAVNVVINFDFPKLAETYLHRIGRSGRFGHLGLAINLITYDDRFNLKSIEEQLGTEIKPIPSNIDKSLYVAEYHTEPVDD

EKP

>XP_011950936.2 probable ATP-dependent RNA helicase DDX6 [Ovis aries]

MSTARTENPVIMGLSSQNGQLRGPVKPSGGPGGGGAQTQQQMNQLKNTNAINNGTQQQAQSMTTTIKPGDDWKKTLKLPP

KDLRIKTSDVTSTKGNEFEDYCLKRELLMGIFEMGWEKPSPIQEESIPIALSGRDILARAKNGTGKSGAYLIPLLERLDL

KKDNIQAMVIVPTRELALQVSQICIQVSKHMGGAKVMATTGGTNLRDDIMRLDDTVHVVIATPGRILDLIKKGVAKVDHV

QMIVLDEADKLLSQDFVQIMEDIILTLPKNRQILLYSATFPLSVQKFMNSHLQKPYEINLMEELTLKGVTQYYAYVTERQ

KVHCLNTLFSRLQINQSIIFCNSSQRVELLAKKISQLGYSCFYIHAKMRQEHRNRVFHDFRNGLCRNLVCTDLFTRGIDI

QAVNVVINFDFPKLAETYLHRIGRSGRFGHLGLAINLITYDDRFNLKSIEEQLGTEIKPIPSNIDKSLYVAEYHSEPVED

EKA

>XP_005058612.1 PREDICTED: probable ATP-dependent RNA helicase DDX6 [Ficedula albicollis]

MSTARTENPVIMGLSSQNGQLRGPVKPSGGPGGGGTQTQQQMNQLKNANTINNGTQQQAQSMTTAIKPGDDWKKTLKLPP

KDLRIKTSDVTSTKGNEFEDYCLKRELLMGIFEMGWEKPSPIQEESIPIALSGRDILARAKNGTGKSGAYLIPLLERLDL

KKDNIQAMVIVPTRELALQVSQICIQVSKHMGGAKVMATTGGTNLRDDIMRLDDTVHVVIATPGRILDLIKKGVAKVEHV

QMIVLDEADKLLSQDFVQIMEDIILTLPKNRQILLYSATFPLSVQKFMNSHLQKPYEINLMEELTLKGVTQYYAYVTERQ

KVHCLNTLFSRLQINQSIIFCNSSQRVELLAKKISQLGYSCFYIHAKMRQEHRNRVFHDFRNGLCRNLVCTDLFTRGIDI

QAVNVVINFDFPKLAETYLHRIGRSGRFGHLGLAINLITYDDRFNLKSIEEQLGTEIKPIPSNIDKSLYVAEYHSEPVED

EKQ

>NWY68222.1 DDX6 helicase [Erithacus rubecula]

MSTARTENPVIMGLSSQNGQLRGPVKPSGGPGGGGTQTQQQMNQLKNANAINNGTQQQAQSMTTTIKPGDDWKKTLKLPP

KDLRIKTSDVTSTKGNEFEDYCLKRELLMGIFEMGWEKPSPIQEESIPIALSGRDILARAKNGTGKSGAYLIPLLERLDL

KKDNIQAMVIVPTRELALQVSQICIQVSKHMGGAKVMATTGGTNLRDDIMRLDDTVHVVIATPGRILDLIKKGVAKVEHV

QMIVLDEADKLLSQDFVQIMEDIILTLPKNRQILLYSATFPLSVQKFMNSHLQKPYEINLMEELTLKGVTQYYAYVTERQ

KVHCLNTLFSRLQINQSIIFCNSSQRVELLAKKISQLGYSCFYIHAKMRQEHRNRVFHDFRNGLCRNLVCTDLFTRGIDI

QAVNVVINFDFPKLAETYLHRIGRSGRFGHLGLAINLITYDDRFNLKSIEEQLGTEIKPIPSNIDKSLYVAEYHSEPVED

EKQ

>XP_006037671.1 probable ATP-dependent RNA helicase DDX6 [Alligator sinensis]

MSTARTENPVIMGLSSQNGQLRGPVKPSGGPGGGGTQTQQQMNQLKNANTINNGTQQQAQSMTTTIKPGDDWKKTLKLPP

KDLRIKTSDVTSTKGNEFEDYCLKRELLMGIFEMGWEKPSPIQEESIPIALSGRDILARAKNGTGKSGAYLIPLLERLDL

KKDNIQAMVIVPTRELALQVSQICIQVSKHMGGAKVMATTGGTNLRDDIMRLDDTVHVVIATPGRILDLIKKGVAKVEHV

QMIVLDEADKLLSQDFVQIMEDIILTLPKNRQILLYSATFPLSVQKFMNSHLQKPYEINLMEELTLKGVTQYYAYVTERQ

KVHCLNTLFSRLQINQSIIFCNSSQRVELLAKKISQLGYSCFYIHAKMRQEHRNRVFHDFRNGLCRNLVCTDLFTRGIDI

QAVNVVINFDFPKLAETYLHRIGRSGRFGHLGLAINLITYDDRFNLKSIEEQLGTEIKPIPSNIDKSLYVAEYHSEPVED

EKP

>XP_006120991.1 probable ATP-dependent RNA helicase DDX6 [Pelodiscus sinensis]

MSTARTENPVIMGLSSQNGQLRGPVKPSAVPGSAGTQTQQQMNQLKNTNAINNGTQQQAQSMTTAIKPGDDWKKTLKLPP

KDLRIKTSDVTSTKGNEFEDYCLKRELLMGIFEMGWEKPSPIQEESIPIALSGRDILARAKNGTGKSGAYLIPLLERLDL

KKDNIQAMVIVPTRELALQVSQICIQVSKHMGGAKVMATTGGTNLRDDIMRLDDTVHVVIATPGRILDLIKKGVAKVEHV

QMIVLDEADKLLSQDFVQIMEDIILTLPKNRQILLYSATFPLSVQKFMNSHLQKPYEINLMEELTLKGVTQYYAYVTERQ

KVHCLNTLFSRLQINQSIIFCNSSQRVELLAKKISQLGYSCFYIHAKMRQEHRNRVFHDFRNGLCRNLVCTDLFTRGIDI

QAVNVVINFDFPKLAETYLHRIGRSGRFGHLGLAINLITYDDRFNLKSIEEQLGTEIKPIPSNIDKSLYVAEYHTEPVDD

EKP

>XP_009579459.1 PREDICTED: probable ATP-dependent RNA helicase DDX6 [Fulmarus glacialis]

MSTARTENPVIMGLSSQNGQLRGPVKPSGGPGGGGTQTQQQMNQLKNANTINNGTQQQAQSMTTPIKPGDDWKKTLKLPP

KDLRIKTSDVTSTKGNEFEDYCLKRELLMGIFEMGWEKPSPIQEESIPIALSGRDILARAKNGTGKSGAYLIPLLERLDL

KKDNIQAMVIVPTRELALQVSQICIQVSKHMGGAKVMATTGGTNLRDDIMRLDDTVHVVIATPGRILDLIKKGVAKVEHV

QMIVLDEADKLLSQDFVQIMEDIILTLPKNRQILLYSATFPLSVQKFMNSHLQKPYEINLMEELTLKGVTQYYAYVTERQ

KVHCLNTLFSRLQINQSIIFCNSSQRVELLAKKISQLGYSCFYIHAKMRQEHRNRVFHDFRNGLCRNLVCTDLFTRGIDI

QAVNVVINFDFPKLAETYLHRIGRSGRFGHLGLAINLITYDDRFNLKSIEEQLGTEIKPIPSNIDKSLYVAEYHSEPVED

EKQ

>XP_008936029.1 PREDICTED: probable ATP-dependent RNA helicase DDX6 [Merops nubicus]

MSTARTENPVIMGLSSQNGQLRGPVKPSGGPGGGGTQTQQQMNQLKNANTINNGTQQQAQSMTAAIKPGDDWKKTLKLPP

KDLRIKTSDVTSTKGNEFEDYCLKRELLMGIFEMGWEKPSPIQEESIPIALSGRDILARAKNGTGKSGAYLIPLLERLDL

KKDNIQAMVIVPTRELALQVSQICIQVSKHMGGAKVMATTGGTNLRDDIMRLDDTVHVVIATPGRILDLIKKGVAKVEHV

QMIVLDEADKLLSQDFVQIMEDIILTLPKNRQILLYSATFPLSVQKFMNSHLQKPYEINLMEELTLKGVTQYYAYVTERQ

KVHCLNTLFSRLQINQSIIFCNSSQRVELLAKKISQLGYSCFYIHAKMRQEHRNRVFHDFRNGLCRNLVCTDLFTRGIDI

QAVNVVINFDFPKLAETYLHRIGRSGRFGHLGLAINLITYDDRFNLKSIEEQLGTEIKPIPSNIDKSLYVAEYHSEPVED

EKQ

>XP_030320492.1 probable ATP-dependent RNA helicase DDX6 [Calypte anna]

MSTARTENPVIMGLSSQNGQLRGPVKPSGGSGGGGTQTQQQMNQLKNANTINNGTQQQAQSMTTAIKPGDDWKKTLKLPP

KDLRIKTSDVTSTKGNEFEDYCLKRELLMGIFEMGWEKPSPIQEESIPIALSGRDILARAKNGTGKSGAYLIPLLERLDL

KKDNIQAMVIVPTRELALQVSQICIQVSKHMGGAKVMATTGGTNLRDDIMRLDDTVHVVIATPGRILDLIKKGVAKVEHV

QMIVLDEADKLLSQDFVQIMEDIILTLPKNRQILLYSATFPLSVQKFMNSHLQKPYEINLMEELTLKGVTQYYAYVTERQ

KVHCLNTLFSRLQINQSIIFCNSSQRVELLAKKISQLGYSCFYIHAKMRQEHRNRVFHDFRNGLCRNLVCTDLFTRGIDI

QAVNVVINFDFPKLAETYLHRIGRSGRFGHLGLAINLITYDDRFNLKSIEEQLGTEIKPIPSNIDKSLYVAEYHSEPVED

EKQ

>XP_017320792.1 PREDICTED: probable ATP-dependent RNA helicase DDX6 [Ictalurus punctatus]

MSAARTENSVILGLTNQNGQKRGSAKPTGGPGGAGGGSQTSQPAQIKASSAVNNGNSLPVPTANTVIKPGDDWKKNLKLP

PKDMRMKTSDVTATKGNEFEDYCLKRELLMGIFEMGWEKPSPIQEESIPIALSGRDILARAKNGTGKSGAYLIPLLERID

LKKDCIQALVIVPTRELALQVSQICIQVSKHMGGVKVMATTGGTNLRDDIMRLDETVHVVIATPGRVLDLIKKGVAKVSQ

VQMIVLDEADKLLSQDFVQMMEEILSFLPKQRQILLYSATFPLSVQKFMNAHLQKPYEINLMEELTLKGVTQYYAYVTER

QKVHCLNTLFSRLQINQSIIFCNSSQRVELLAKKISQLGYSCFYIHAKMRQEHRNRVFHDFRNGLCRNLVCTDLFTRGID

IQAVNVVINFDFPKLGETYLHRIGRSGRFGHLGLAINLITYDDRFNLKGIEEQLGTEIKPIPSSIDKSLYVAEYHSESAE

EVKL

>XP_032820546.1 probable ATP-dependent RNA helicase DDX6 [Petromyzon marinus]

MAATRTENGVAVGMGGQMRSTTGPNMLRVNAPTMMPQSKGPMPMSASALQSASMHGGSQAQNPGAGSGVKPGDDWKAGLK

LPPKDRRVKTSDVTATKGNEFEDYCLKRELLMGIFEKGWEKPSPIQEESIPIALSGRDILARAKNGTGKSGAYLIPLLER

LDMKKPYIQALTIVPTRELALQTSQICIELSKHMGGVKVMATTGGTNLRDDILRLDETVHVIIATPGRILDLINKGLAKV

DAVQMIVLDEADKLLSQDFVQMLDDIIRAMPRNRQILLYSATFPLSVQKFMNRHLQKPYEINLMEELTLKGITQFYAYVN

ERQKVHCLNTLFSKLQINQSIIFCNSTQRVELLAKKITQLGYSCFYIHAKMKQEHRNRVFHDFRRGECRNLVCSDLFTRG

IDIQAVNVVINFDFPKNAETYLHRIGRSGRYGHLGLAINLITYEDRFNLKGIEEQLGTEIKPIPGTIDKSLYVAEFHSEP

DEGGSTQQPAQQQPPSQQQP

>XP_030394164.1 probable ATP-dependent RNA helicase DDX6 [Gopherus evgoodei]

MSTARTENPVIMGLSSQNGQLRGPVKPSGGPGGGGTQTQQQMNQLKNTNTINNGIQQQAQSMTTAIKPGDDWKKTLKLPP

KDLRIKTSDVTSTKGNEFEDYCLKRELLMGIFEMGWEKPSPIQEESIPIALSGRDILARAKNGTGKSGAYLIPLLERLDL

KKDNIQAMVIVPTRELALQVSQICIQVSKHMGGAKVMATTGGTNLRDDIMRLDDTVHVVIATPGRILDLIKKGVAKVEHV

QMIVLDEADKLLSQDFVQIMEDIILTLPKNRQILLYSATFPLSVQKFMNSHLQKPYEINLMEELTLKGVTQYYAYVTERQ

KVHCLNTLFSRLQINQSIIFCNSSQRVELLAKKISQLGYSCFYIHAKMRQEHRNRVFHDFRNGLCRNLVCTDLFTRGIDI

QAVNVVINFDFPKLAETYLHRIGRSGRFGHLGLAINLITYDDRFNLKSIEEQLGTEIKPIPSNIDKSLYVAEYHTEPVDD

EKP

>XP_004427303.1 PREDICTED: probable ATP-dependent RNA helicase DDX6 [Ceratotherium simum simum]

MSTARTENPVIMGLSSQNGQLRGPVKPSGGPGGGGTQTQQQMNQLKTTNTINNGTQQQAQSMTTTIKPGDDWKKTLKLPP

KDLRIKTSDVTSTKGNEFEDYCLKRELLMGIFEMGWEKPSPIQEESIPIALSGRDILARAKNGTGKSGAYLIPLLERLDL

KKDNIQAMVIVPTRELALQVSQICIQVSKHMGGAKVMATTGGTNLRDDIMRLDDTVHVVIATPGRILDLIKKGVAKVDHV

QMIVLDEADKLLSQDFVQIMEDIILTLPKNRQILLYSATFPLSVQKFMNSHLQKPYEINLMEELTLKGVTQYYAYVTERQ

KVHCLNTLFSRLQINQSIIFCNSSQRVELLAKKISQLGYSCFYIHAKMRQEHRNRVFHDFRNGLCRNLVCTDLFTRGIDI

QAVNVVINFDFPKLAETYLHRIGRSGRFGHLGLAINLITYDDRFNLKSIEEQLGTEIKPIPSNIDKSLYVAEYHSEPVED

EKA

>XP_005299846.1 probable ATP-dependent RNA helicase DDX6 [Chrysemys picta bellii]

MSTARTENPVIMGLSSQNGQLRGPVKPSGGPGGGGTQTQQQMNQLKNTNTINNGTQQQAQSMTSAIKPGDDWKKTLKLPP

KDLRIKTSDVTSTKGNEFEDYCLKRELLMGIFEMGWEKPSPIQEESIPIALSGRDILARAKNGTGKSGAYLIPLLERLDL

KKDNIQAMVIVPTRELALQVSQICIQVSKHMGGAKVMATTGGTNLRDDIMRLDDTVHVVIATPGRILDLIKKGVAKVEHV

QMIVLDEADKLLSQDFVQIMEDIILTLPKNRQILLYSATFPLSVQKFMNSHLQKPYEINLMEELTLKGVTQYYAYVTERQ

KVHCLNTLFSRLQINQSIIFCNSSQRVELLAKKISQLGYSCFYIHAKMRQEHRNRVFHDFRNGLCRNLVCTDLFTRGIDI

QAVNVVINFDFPKLAETYLHRIGRSGRFGHLGLAINLITYDDRFNLKSIEEQLGTEIKPIPSNIDKSLYVAEYHTEPVDD

EKP

>XP_009566929.1 PREDICTED: probable ATP-dependent RNA helicase DDX6 [Cuculus canorus]

MSTARTENPVIMGLSSQNGQLRGPVKPSGGPGGGGTQTQPQMNPLKNANTINNGTQQQAQSMTTAIKPGDDWKKTLKLPP

KDLRIKTSDVTSTKGNEFEDYCLKRELLMGIFEMGWEKPSPIQEESIPIALSGRDILARAKNGTGKSGAYLIPLLERLDL

KKDNIQAMVIVPTRELALQVSQICIQVSKHMGGAKVMATTGGTNLRDDIMRLDDTVHVVIATPGRILDLIKKGVAKVEHV

QMIVLDEADKLLSQDFVQIMEDIILTLPKNRQILLYSATFPLSVQKFMNSHLQKPYEINLMEELTLKGVTQYYAYVTERQ

KVHCLNTLFSRLQINQSIIFCNSSQRVELLAKKISQLGYSCFYIHAKMRQEHRNRVFHDFRNGLCRNLVCTDLFTRGIDI

QAVNVVINFDFPKLAETYLHRIGRSGRFGHLGLAINLITYDDRFNLKSIEEQLGTEIKPIPSNIDKSLYVAEYHSEPVED

EKQ

>NXW58280.1 DDX6 helicase [Eurystomus gularis]

MSTARTENPVIMGLSSQNGQLRGPVKPSGGPGGGGTQAQQQMNQLKNANTINNGTQQQAQSMTTAIKPGDDWKKTLKLPP

KDLRIKTSDVTSTKGNEFEDYCLKRELLMGIFEMGWEKPSPIQEESIPIALSGRDILARAKNGTGKSGAYLIPLLERLDL

KKDNIQAMVIVPTRELALQVSQICIQVSKHMGGAKVMATTGGTNLRDDIMRLDDTVHVVIATPGRILDLIKKGVAKVEHV

QMIVLDEANKLLSQDFVQIMEDIILTLPKNRQILLYSATFPLSVQKFMNSHLQKPYEINLMEELTLKGVTQYYAYVTERQ

KVHCLNTLFSRLQINQSIIFCNSSQRVELLAKKISQLGYSCFYIHAKMRQEHRNRVFHDFRNGLCRNLVCTDLFTRGIDI

QAVNVVINFDFPKLAETYLHRIGRSGRFGHLGLAINLITYDDRFNLKSIEEQLGTEIKPIPSNIDKSLYVAEYHSEPVED

EKQ

>XP_021061908.1 probable ATP-dependent RNA helicase DDX6 [Mus pahari]

MSTARTENPVIMGLPSQNGQLRGPVKASAGPGGGGTQPQPHVNQLKNTSTINNGTPQQAQSMAATIKPGDDWKKTLKLPP

KDLRIKTSDVTSTKGNEFEDYCLKRELLMGIFEMGWEKPSPIQEESIPIALSGRDILARAKNGTGKSGAYLIPLLERLDL

KKDNIQAMVIVPTRELALQVSQICIQVSKHMGGAKVMATTGGTNLRDDIMRLDDTVHVVIATPGRILDLIKKGVAKVDHV

QMIVLDEADKLLSQDFVQIMEDIILTLPKNRQILLYSATFPLSVQKFMNSHLQKPYEINLMEELTLKGVTQYYAYVTERQ

KVHCLNTLFSRLQINQSIIFCNSSQRVELLAKKISQLGYSCFYIHAKMRQEHRNRVFHDFRNGLCRNLVCTDLFTRGIDI

QAVNVVINFDFPKLAETYLHRIGRSGRFGHLGLAINLITYDDRFNLKSIEEQLGTEIKPIPSNIDKSLYVAEYHSEPAED

EKP

>NP_001072584.1 probable ATP-dependent RNA helicase ddx6 [Xenopus tropicalis]

MSTARTENPVLMGMSSQNGQLRGPLKPSAGPGGGGTQTQQINQLKNASTINSGSQQQAQSMSSVIKPGDDWKKTLKLPPK

DLRIKTSDVTSTKGNEFEDYCLKRELLMGIFEMGWEKPSPIQEESIPIALSGRDILARAKNGTGKSGAYLIPLLERLDLK

KDCIQAMVIVPTRELALQVSQICIQVSKHMGGVKVMATTGGTNLRDDIMRLDDTVHVVIATPGRILDLIKKGVAKVDHIQ

MIVLDEADKLLSQDFVQIMEDIIITLPKNRQILLYSATFPLSVQKFMTSHLQKPYEINLMEELTLKGVTQYYAYVTERQK

VHCLNTLFSRLQINQSIIFCNSSQRVELLAKKISQLGYSCFYIHAKMRQEHRNRVFHDFRNGLCRNLVCTDLFTRGIDIQ

AVNVVINFDFPKLAETYLHRIGRSGRFGHLGLAINLITYDDRFNLKSIEEQLGTEIKPIPSSIDKSLYVAEYHSESGEDK

P

>XP_014886879.1 PREDICTED: probable ATP-dependent RNA helicase DDX6 [Poecilia latipinna]

MSTTRTENPVILGLSNQNGQLRSSVKPAGAPGGGGGGPQPPQLNQMIKGTINGNSQPAPPTNAVIKPGDDWKKNLKLPPK

DMRIKTSDVTATKGNEFEDYCLKRELLMGIFEMGWEKPSPIQEESIPIALSGRDILARAKNGTGKSGAYLIPLLERIDLK

KDCLQALVIVPTRELALQVSQICIQVSKHMGGVKVMATTGGTNLRDDIMRLDETVHVVIATPGRILDLIKKGVAKVNQVQ

MIVLDEADKLLSQDFVVMMEEMLGFLPKQRQILLYSATFPLSVQKFMNAHLQKPYEINLMEELTLKGVTQYYAYVTERQK

VHCLNTLFSRLQINQSIIFCNSSQRVELLAKKISQLGYSCFYIHAKMRQEHRNRVFHDFRNGLCRNLVCTDLFTRGIDIQ

AVNVVINFDFPKLGETYLHRIGRSGRFGHLGLAINLITYDDRFNLKAIEEQLGTEIKPIPGIIDKSLYVAEYHSETGEEV

KQ

>XP_028722211.1 probable ATP-dependent RNA helicase DDX6 [Peromyscus leucopus]

MSTARTENPVIMGLSSQNGQLRGPVKPSGGPGGGGTPTQQQMNQLKNTNTINNGTQQQAQSMAATIKPGDDWKKTLKLPP

KDLRIKTSDVTSTKGNEFEDYCLKRELLMGIFEMGWEKPSPIQEESIPIALSGRDILARAKNGTGKSGAYLIPLLERLDL

KKDTIQAMVIVPTRELALQVSQICIQVSKHMGGAKVMATTGGTNLRDDIMRLDDTVHVVIATPGRILDLIKKGVAKVDHV

QMIVLDEADKLLSQDFVQIMEDIILTLPKNRQILLYSATFPLSVQKFMNSHLQKPYEINLMEELTLKGVTQYYAYVTERQ

KVHCLNTLFSRLQINQSIIFCNSSQRVELLAKKISQLGYSCFYIHAKMRQEHRNRVFHDFRNGLCRNLVCTDLFTRGIDI

QAVNVVINFDFPKLAETYLHRIGRSGRFGHLGLAINLITYDDRFNLKSIEEQLGTEIKPIPSNIDKSLYVAEYHSEPVED

EKP

>XP_009467328.1 PREDICTED: probable ATP-dependent RNA helicase DDX6 [Nipponia nippon]

MSTARTENPVIMGLSSQNGQLRGPVKPSAGPGGGGTQTQQQMNQLKNANTINNGTQQQAQSMTTAIKPGDDWKKTLKLPP

KDLRIKTSDVTSTKGNEFEDYCLKRELLMGIFEMGWEKPSPIQEESIPIALSGRDILARAKNGTGKSGAYLIPLLERLDL

KKDNIQAMVIVPTRELALQVSQICIQVSKHMGGAKVMATTGGTNLRDDIMRLDDTVHVVIATPGRILDLIKKGVAKVEHV

QMIVLDEADKLLSQDFVQIMEDIILTLPKNRQILLYSATFPLSVQKFMNSHLQKPYEINLMEELTLKGVTQYYAYVTERQ

KVHCLNTLFSRLQINQSIIFCNSSQRVELLAKKISQLGYSCFYIHAKMRQEHRNRVFHDFRNGLCRNLVCTDLFTRGIDI

QAVNVVINFDFPKLAETYLHRIGRSGRFGHLGLAINLITYDDRFNLKSIEEQLGTEIKPIPSNIDKSLYVAEYHSEPVED

EKQ

>XP_015842358.1 PREDICTED: probable ATP-dependent RNA helicase DDX6 [Peromyscus maniculatus bairdii]

MSTARTENPVIMGLSSQNGQLRGPVKPSGGPGGGGTPTQQQMNQLKNTNTINNGTQQQAQSMTATIKPGDDWKKTLKLPP

KDLRIKTSDVTSTKGNEFEDYCLKRELLMGIFEMGWEKPSPIQEESIPIALSGRDILARAKNGTGKSGAYLIPLLERLDL

KKDTIQAMVIVPTRELALQVSQICIQVSKHMGGAKVMATTGGTNLRDDIMRLDDTVHVVIATPGRILDLIKKGVAKVDHV

QMIVLDEADKLLSQDFVQIMEDIILTLPKNRQILLYSATFPLSVQKFMNSHLQKPYEINLMEELTLKGVTQYYAYVTERQ

KVHCLNTLFSRLQINQSIIFCNSSQRVELLAKKISQLGYSCFYIHAKMRQEHRNRVFHDFRNGLCRNLVCTDLFTRGIDI

QAVNVVINFDFPKLAETYLHRIGRSGRFGHLGLAINLITYDDRFNLKSIEEQLGTEIKPIPSNIDKSLYVAEYHSEPVED

EKP

>TFJ97697.1 putative ATP-dependent RNA helicase DDX6 [Platysternon megacephalum]

MSTARTENPVIMGLSSQNGQLRGPVKPSGGPGGGGTQTQQQMNQLKNTNTINNGTQQQAQSMTTAIKPGDDWKKTLKLPP

KDLRIKTSDVTSTKGNEFEDYCLKRELLMGIFEMGWEKPSPIQEESIPIALSGRDILARAKNGTGKSGAYLIPLLERLDL

KKDNIQAMVIVPTRELALQVSQICIQVSKHMGGAKVMATTGGTNLRDDIMRLDDTVHVVIATPGRILDLIKKGVAKVEHV

QMIVLDEADKLLSQDFVQIMEDIILTLPKNRQILLYSATFPLSVQKFMNSHLQKPYEINLMEELTLKGVTQYYAYVTERQ

KVHCLNTLFSRLQINQSIIFCNSSQRVELLAKKISQLGYSCFYIHAKMRQEHRNRVFHDFRNGLCRNLVCTDLFTRGIDI

QAVNVVINFDFPKLAETYLHRIGRSGRFGHLGLAINLITYDDRFNLKSIEEQLGTEIKPIPSNIDKSLYVAEYHTEPIDD

EKP

>NP_001244120.1 probable ATP-dependent RNA helicase DDX6 [Homo sapiens]

MSTARTENPVIMGLSSQNGQLRGPVKPTGGPGGGGTQTQQQMNQLKNTNTINNGTQQQAQSMTTTIKPGDDWKKTLKLPP

KDLRIKTSDVTSTKGNEFEDYCLKRELLMGIFEMGWEKPSPIQEESIPIALSGRDILARAKNGTGKSGAYLIPLLERLDL

KKDNIQAMVIVPTRELALQVSQICIQVSKHMGGAKVMATTGGTNLRDDIMRLDDTVHVVIATPGRILDLIKKGVAKVDHV

QMIVLDEADKLLSQDFVQIMEDIILTLPKNRQILLYSATFPLSVQKFMNSHLQKPYEINLMEELTLKGVTQYYAYVTERQ

KVHCLNTLFSRLQINQSIIFCNSSQRVELLAKKISQLGYSCFYIHAKMRQEHRNRVFHDFRNGLCRNLVCTDLFTRGIDI

QAVNVVINFDFPKLAETYLHRIGRSGRFGHLGLAINLITYDDRFNLKSIEEQLGTEIKPIPSNIDKSLYVAEYHSEPVED

EKP

>XP_021140859.1 probable ATP-dependent RNA helicase DDX6 [Columba livia]

MSTARTENPVIMGLSSQNGQLRGPVKPSGGPGGGGTPTQQQMNQLKNANTINNGTQQQAQSMTTALKPGDDWKKTLKLPP

KDLRIKTSDVTSTKGNEFEDYCLKRELLMGIFEMGWEKPSPIQEESIPIALSGRDILARAKNGTGKSGAYLIPLLERLDL

KKDNIQAMVIVPTRELALQVSQICIQVSKHMGGAKVMATTGGTNLRDDIMRLDDTVHVVIATPGRILDLIKKGVAKVEHV

QMIVLDEADKLLSQDFVQIMEDIILTLPKNRQILLYSATFPLSVQKFMNSHLQKPYEINLMEELTLKGVTQYYAYVTERQ

KVHCLNTLFSRLQINQSIIFCNSSQRVELLAKKISQLGYSCFYIHAKMRQEHRNRVFHDFRNGLCRNLVCTDLFTRGIDI

QAVNVVINFDFPKLAETYLHRIGRSGRFGHLGLAINLITYDDRFNLKSIEEQLGTEIKPIPSNIDKSLYVAEYHSEPVED

EKQ

>XP_004667445.1 PREDICTED: probable ATP-dependent RNA helicase DDX6 [Jaculus jaculus]

MSTARTENPVIMGLSSQNGQLRGPVKPSGGPGGGGTQTQQQMNQLKTTNTINNGTQQQAQSMTTTIKPGDDWKKTLKLPP

KDLRIKTSDVTSTKGNEFEDYCLKRELLMGIFEMGWEKPSPIQEESIPIALSGRDILARAKNGTGKSGAYLIPLLERLDL

KKDNIQAMVIVPTRELALQVSQICIQVSKHMGGAKVMATTGGTNLRDDIMRLDDTVHVVIATPGRILDLIKKGVAKVDHV

QMIVLDEADKLLSQDFVQIMEDIILTLPKNRQILLYSATFPLSVQKFMNSHLQKPYEINLMEELTLKGVTQYYAYVTERQ

KVHCLNTLFSRLQINQSIIFCNSSQRVELLAKKISQLGYSCFYIHAKMRQEHRNRVFHDFRNGLCRNLVCTDLFTRGIDI

QAVNVVINFDFPKLAETYLHRIGRSGRFGHLGLAINLITYDDRFNLKSIEEQLGTEIKPIPSNIDKSLYVAEYHSEPVED

EKP

>NP_001137339.1 probable ATP-dependent RNA helicase DDX6 [Bos taurus]

MSTARTENPVIMGLSSQNGQLRGPVKPSGGPGGGGTQTQQQMNQLKNTNTINNGTQQQAQSMTTTIKPGDDWKKTLKLPP

KDLRIKTSDVTSTKGNEFEDYCLKRELLMGIFEMGWEKPSPIQEESIPIALSGRDILARAKNGTGKSGAYLIPLLERLDL

KKDNIQAMVIVPTRELALQVSQICIQVSKHMGGAKVMATTGGTNLRDDIMRLDDTVHVVIATPGRILDLIKKGVAKVDHV

QMIVLDEADKLLSQDFVQIMEDIILTLPKNRQILLYSATFPLSVQKFMNSHLQKPYEINLMEELTLKGVTQYYAYVTERQ

KVHCLNTLFSRLQINQSIIFCNSSQRVELLAKKISQLGYSCFYIHAKMRQEHRNRVFHDFRNGLCRNLVCTDLFTRGIDI

QAVNVVINFDFPKLAETYLHRIGRSGRFGHLGLAINLITYDDRFNLKSIEEQLGTEIKPIPSNIDKSLYVAEYHSEPVED

EKA

>NXD87150.1 DDX6 helicase [Halcyon senegalensis]

MSTARTENPVIMGLSSQNGQLRGPVKPSGGPGGGGTQTQQQMNQLKNANTINNGTQQQAQSMTTSIKPGDDWKKTLKLPP

KDLRIKTSDVTSTKGNEFEDYCLKRELLMGIFEMGWEKPSPIQEESIPIALSGRDILARAKNGTGKSGAYLIPLLERLDL

KKDNIQAMVIVPTRELALQVSQICIQVSKHMGGAKVMATTGGTNLRDDIMRLDDTVHVVIATPGRILDLIKKGVAKVEHV

QMIVLDEANKLLSQDFVQIMEDIILTLPKNRQILLYSATFPLSVQKFMNSHLQKPYEINLMEELTLKGVTQYYAYVTERQ

KVHCLNTLFSRLQINQSIIFCNSSQRVELLAKKISQLGYSCFYIHAKMRQEHRNRVFHDFRNGLCRNLVCTDLFTRGIDI

QAVNVVINFDFPKLAETYLHRIGRSGRFGHLGLAINLITYDDRFNLKSIEEQLGTEIKPIPSNIDKSLYVAEYHSEPVED

EKQ

>XP_008824161.1 probable ATP-dependent RNA helicase DDX6 [Nannospalax galili]

MSTARTENPVIMGLSSQNGQLRGPVKPSGGPGGGGTQTQQQMNQLKNTNTINNGTQQAQSMTTTIKPGDDWKKTLKLPPK

DLRIKTSDVTSTKGNEFEDYCLKRELLMGIFEMGWEKPSPIQEESIPIALSGRDILARAKNGTGKSGAYLIPLLERLDLK

KDNIQAMVIVPTRELALQVSQICIQVSKHMGGAKVMATTGGTNLRDDIMRLDDTVHVVIATPGRILDLIKKGVAKVDHVQ

MIVLDEADKLLSQDFVQIMEDIILTLPKNRQILLYSATFPLSVQKFMNSHLQKPYEINLMEELTLKGVTQYYAYVTERQK

VHCLNTLFSRLQINQSIIFCNSSQRVELLAKKISQLGYSCFYIHAKMRQEHRNRVFHDFRNGLCRNLVCTDLFTRGIDIQ

AVNVVINFDFPKLAETYLHRIGRSGRFGHLGLAINLITYDDRFNLKSIEEQLGTEIKPIPSNIDKSLYVAEYHSEPVEDE

KP

>XP_036113660.1 probable ATP-dependent RNA helicase DDX6 [Molossus molossus]

MSTARTENPVIMGLSSQNGQLRGPVKPSGGPGGGGTQTQQQMNQLKNTNTINNGTQQQAQSMTTTIKPGDDWKKALKLPP

KDLRIKTSDVTSTKGNEFEDYCLKRELLMGIFEMGWEKPSPIQEESIPIALSGRDILARAKNGTGKSGAYLIPLLERLDL

KKDNIQAMVIVPTRELALQVSQICIQVSKHMGGAKVMATTGGTNLRDDIMRLDDTVHVVIATPGRILDLIKKGVAKVDHV

QMIVLDEADKLLSQDFVQIMEDIILTLPKNRQILLYSATFPLSVQKFMNSHLQKPYEINLMEELTLKGVTQYYAYVTERQ

KVHCLNTLFSRLQINQSIIFCNSSQRVELLAKKISQLGYSCFYIHAKMRQEHRNRVFHDFRNGLCRNLVCTDLFTRGIDI

QAVNVVINFDFPKLAETYLHRIGRSGRFGHLGLAINLITYDDRFNLKSIEEQLGTEIKPIPSNIDKSLYVAEYHSEPVED

DKP

>XP_017915190.1 PREDICTED: probable ATP-dependent RNA helicase DDX6 [Capra hircus]

MSTARTENPVIMGLSSQNGQLRGPVKPSGGPGGGGTQTQQQMNQLKNTNAINNGTQQQAQSMTTTIKPGDDWKKTLKLPP

KDLRIKTSDVTSTKGNEFEDYCLKRELLMGIFEMGWEKPSPIQEESIPIALSGRDILARAKNGTGKSGAYLIPLLERLDL

KKDNIQAMVIVPTRELALQVSQICIQVSKHMGGAKVMATTGGTNLRDDIMRLDDTVHVVIATPGRILDLIKKGVAKVDHV

QMIVLDEADKLLSQDFVQIMEDIILTLPKNRQILLYSATFPLSVQKFMNSHLQKPYEINLMEELTLKGVTQYYAYVTERQ

KVHCLNTLFSRLQINQSIIFCNSSQRVELLAKKISQLGYSCFYIHAKMRQEHRNRVFHDFRNGLCRNLVCTDLFTRGIDI

QAVNVVINFDFPKLAETYLHRIGRSGRFGHLGLAINLITYDDRFNLKSIEEQLGTEIKPIPSNIDKSLYVAEYHSEPVED

EKA

>XP_005328396.1 probable ATP-dependent RNA helicase DDX6 [Ictidomys tridecemlineatus]

MSTARTENPVIMGLSSQNGQLRGPVKPSGGPGGGGTQSQQQMNQLKNTNTINNGTQQQAQSMTTTIKPGDDWKKTLKLPP

KDLRIKTSDVTSTKGNEFEDYCLKRELLMGIFEMGWEKPSPIQEESIPIALSGRDILARAKNGTGKSGAYLIPLLERLDL

KKDNIQAMVIVPTRELALQVSQICIQVSKHMGGAKVMATTGGTNLRDDIMRLDDTVHVVIATPGRILDLIKKGVAKVDHV

QMIVLDEADKLLSQDFVQIMEDIILTLPKNRQILLYSATFPLSVQKFMNSHLQKPYEINLMEELTLKGVTQYYAYVTERQ

KVHCLNTLFSRLQINQSIIFCNSSQRVELLAKKISQLGYSCFYIHAKMRQEHRNRVFHDFRNGLCRNLVCTDLFTRGIDI

QAVNVVINFDFPKLAETYLHRIGRSGRFGHLGLAINLITYDDRFNLKSIEEQLGTEIKPIPSNIDKSLYVAEYHSEPVED

EKP

>KAB0370576.1 hypothetical protein FD755_016985 [Muntiacus reevesi]

MSTARTENPVIMGLSSQNGQLRGPVKPSGGPGGGGAQTQQQMNQLKNTNTINNGTQQQAQSMTTTIKPGDDWKKTLKLPP

KDLRIKTSDVTSTKGNEFEDYCLKRELLMGIFEMGWEKPSPIQEESIPIALSGRDILARAKNGTGKSGAYLIPLLERLDL

KKDNIQAMVIVPTRELALQVSQICIQVSKHMGGAKVMATTGGTNLRDDIMRLDDTVHVVIATPGRILDLIKKGVAKVDHV

QMIVLDEADKLLSQDFVQIMEDIILTLPKNRQILLYSATFPLSVQKFMNSHLQKPYEINLMEELTLKGVTQYYAYVTERQ

KVHCLNTLFSRLQINQSIIFCNSSQRVELLAKKISQLGYSCFYIHAKMRQEHRNRVFHDFRNGLCRNLVCTDLFTRGIDI

QAVNVVINFDFPKLAETYLHRIGRSGRFGHLGLAINLITYDDRFNLKSIEEQLGTEIKPIPSNIDKSLYVAEYHSEPVED

EKP

>KAB0347134.1 hypothetical protein FD754_011991 [Muntiacus muntjak]

MSTARTENPVIMGLSSQNGQLRGPVKPSGGPGGGGAQTQQQMNQLKNTNTINNGTQQQAQSMTATIKPGDDWKKTLKLPP

KDLRIKTSDVTSTKGNEFEDYCLKRELLMGIFEMGWEKPSPIQEESIPIALSGRDILARAKNGTGKSGAYLIPLLERLDL

KKDNIQAMVIVPTRELALQVSQICIQVSKHMGGAKVMATTGGTNLRDDIMRLDDTVHVVIATPGRILDLIKKGVAKVDHV

QMIVLDEADKLLSQDFVQIMEDIILTLPKNRQILLYSATFPLSVQKFMNSHLQKPYEINLMEELTLKGVTQYYAYVTERQ

KVHCLNTLFSRLQINQSIIFCNSSQRVELLAKKISQLGYSCFYIHAKMRQEHRNRVFHDFRNGLCRNLVCTDLFTRGIDI

QAVNVVINFDFPKLAETYLHRIGRSGRFGHLGLAINLITYDDRFNLKSIEEQLGTEIKPIPSNIDKSLYVAEYHSEPVED

EKP

>XP_024426491.1 probable ATP-dependent RNA helicase DDX6 [Desmodus rotundus]

MSTARTENPVIMGLSSQNGQLRGPVKPSGGPGGGGTQTQQQINQLKNTNTINNGTQQQAQSMTTTIKPGDDWKKTLKLPP

KDLRIKTSDVTSTKGNEFEDYCLKRELLMGIFEMGWEKPSPIQEESIPIALSGRDILARAKNGTGKSGAYLIPLLERLDL

KKDNIQAMVIVPTRELALQVSQICIQVSKHMGGAKVMATTGGTNLRDDIMRLDDTVHVVIATPGRILDLIKKGVAKVDHV

QMIVLDEADKLLSQDFVQIMEDIILTLPKNRQILLYSATFPLSVQKFMNSHLQKPYEINLMEELTLKGVTQYYAYVTERQ

KVHCLNTLFSRLQINQSIIFCNSSQRVELLAKKISQLGYSCFYIHAKMRQEHRNRVFHDFRNGLCRNLVCTDLFTRGIDI

QAVNVVINFDFPKLAETYLHRIGRSGRFGHLGLAINLITYDDRFNLKSIEEQLGTEIKPIPSNIDKSLYVAEYHSEPVED

DKP

>XP_034024382.1 probable ATP-dependent RNA helicase ddx6 [Thalassophryne amazonica]

MSVARTENPVILGLSSQNGQLRGSVKPAGAPGGNVPTQQLHQMKGTINNGGSQLAPSANAVIKPGDDWKRSLKLPPKDMR

MKTSDVTATKGNEFEDYCLKRELLMGIFEMGWEKPSPIQEESIPIALSGRDILARAKNGTGKSGAYLIPLLERIDLKKDS

IQALVIVPTRELALQVSQICIQVSKHMGGVKVMATTGGTNLRDDIMRLDETVHVVIATPGRILDLIKKGVAKVNQVQMIV

LDEADKLLSQDFVAMMEEILGFLPKQRQILLYSATFPLSVQKFMNAHLQKPYEINLMEELTLKGVTQYYAYVTERQKVHC

LNTLFSRLQINQSIIFCNSSQRVELLAKKISQLGYSCFYIHAKMRQEHRNRVFHDFRNGLCRNLVCTDLFTRGIDIQAVN

VVINFDFPKLGETYLHRIGRSGRFGHLGLAINLITYDDRFNLKGIEEQLGTEIKPIPGIIDKSLYVAEYHSESGEVKQ

>XP_004712835.1 probable ATP-dependent RNA helicase DDX6 [Echinops telfairi]

MSTARTENPVIMGLSSQNGQLRGPVKPSSGPGGGGTQTQQQINQLKNTNTINNGTQQQAQSMTTAIKPGDDWKKTLKLPP

KDLRIKTSDVTSTKGNEFEDYCLKRELLMGIFEMGWEKPSPIQEESIPIALSGRDILARAKNGTGKSGAYLIPLLERLDL

KKDNIQAMVIVPTRELALQVSQICIQVSKHMGGAKVMATTGGTNLRDDIMRLDDTVHVVIATPGRILDLIKKGVAKVDHV

QMIVLDEADKLLSQDFVQIMEDIILTLPKNRQILLYSATFPLSVQKFMNSHLQKPYEINLMEELTLKGVTQYYAYVTERQ

KVHCLNTLFSRLQINQSIIFCNSSQRVELLAKKISQLGYSCFYIHAKMRQEHRNRVFHDFRNGLCRNLVCTDLFTRGIDI

QAVNVVINFDFPKLAETYLHRIGRSGRFGHLGLAINLITYDDRFNLKSIEEQLGTEIKPIPSNIDKSLYVAEYHSEPVED

EKP

>XP_003129962.1 probable ATP-dependent RNA helicase DDX6 isoform X1 [Sus scrofa]

MSTARTENPVIMGLSSQNGQLRGPVKPSGGPGGGGTQTQQQMNQLKNTNTINNGTQQQAQSMTATIKPGDDWKKTLKLPP

KDLRIKTSDVTSTKGNEFEDYCLKRELLMGIFEMGWEKPSPIQEESIPIALSGRDILARAKNGTGKSGAYLIPLLERLDL

KKDNIQAMVIVPTRELALQVSQICIQVSKHMGGAKVMATTGGTNLRDDIMRLDDTVHVVIATPGRILDLIKKGVAKVDHV

QMIVLDEADKLLSQDFVQIMEDIILTLPKNRQILLYSATFPLSVQKFMNSHLQKPYEINLMEELTLKGVTQYYAYVTERQ

KVHCLNTLFSRLQINQSIIFCNSSQRVELLAKKISQLGYSCFYIHAKMRQEHRNRVFHDFRNGLCRNLVCTDLFTRGIDI

QAVNVVINFDFPKLAETYLHRIGRSGRFGHLGLAINLITYDDRFNLKSIEEQLGTEIKPIPSNIDKSLYVAEYHSEPVED

EKP

>XP_026252106.1 probable ATP-dependent RNA helicase DDX6 [Urocitellus parryii]

MSTARTENPVIMGLSSQNGQLRGPMKPSGGPGGGGTQSQQQMNQLKNTNTINNGTQQQAQSMTTTIKPGDDWKKTLKLPP

KDLRIKTSDVTSTKGNEFEDYCLKRELLMGIFEMGWEKPSPIQEESIPIALSGRDILARAKNGTGKSGAYLIPLLERLDL

KKDNIQAMVIVPTRELALQVSQICIQVSKHMGGAKVMATTGGTNLRDDIMRLDDTVHVVIATPGRILDLIKKGVAKVDHV

QMIVLDEADKLLSQDFVQIMEDIILTLPKNRQILLYSATFPLSVQKFMNSHLQKPYEINLMEELTLKGVTQYYAYVTERQ

KVHCLNTLFSRLQINQSIIFCNSSQRVELLAKKISQLGYSCFYIHAKMRQEHRNRVFHDFRNGLCRNLVCTDLFTRGIDI

QAVNVVINFDFPKLAETYLHRIGRSGRFGHLGLAINLITYDDRFNLKSIEEQLGTEIKPIPSNIDKSLYVAEYHSEPVED

EKP

>XP_012723762.1 probable ATP-dependent RNA helicase ddx6 [Fundulus heteroclitus]

MSTTRTENPVILGLSNQNGQLRGSVKPTGAPGGGGGGLQQPQLNQMLKGTINGNSQPAPPTNAVIKPGDDWKKNLKLPPK

DMRIKTSDVTATKGNEFEDYCLKRELLMGIFEMGWEKPSPIQEESIPIALSGRDILARAKNGTGKSGAYLIPLLERIDLK

KDCLQALVIVPTRELALQVSQICIQVSKHMGGVKVMATTGGTNLRDDIMRLDETVHVVIATPGRILDLIKKGVAKVNQVQ

MVVLDEADKLLSQDFVVMMEEMLGFLPKQRQILLYSATFPLSVQKFMNAHLQKPYEINLMEELTLKGVTQYYAYVTERQK

VHCLNTLFSRLQINQSIIFCNSSQRVELLAKKISQLGYSCFYIHAKMRQEHRNRVFHDFRNGLCRNLVCTDLFTRGIDIQ

AVNVVINFDFPKLGETYLHRIGRSGRFGHLGLAINLITYDDRFNLKAIEEQLGTEIKPIPGIIDKSLYVAEYHSESGEEV

KQ

>XP_016063179.1 PREDICTED: probable ATP-dependent RNA helicase DDX6 [Miniopterus natalensis]

MSTARTENPVIMGLSSQNGQLRGPVKPSGGPGGGGTQTQQQMNQLKNTNTINNGTQQQAQSMTTTIKPGDDWKKTLKLPP

KDLRIKTSDVTSTKGNEFEDYCLKRELLMGIFEMGWEKPSPIQEESIPIALSGRDILARAKNGTGKSGAYLIPLLERLDL

KKDNIQAMVIVPTRELALQVSQICIQVSKHMGGAKVMATTGGTNLRDDIMRLDDTVHVVIATPGRILDLIKKGVAKVDHV

QMIVLDEADKLLSQDFVQIMEDIILTLPKNRQILLYSATFPLSVQKFMNSHLQKPYEINLMEELTLKGVTQYYAYVTERQ

KVHCLNTLFSRLQINQSIIFCNSSQRVELLAKKISQLGYSCFYIHAKMRQEHRNRVFHDFRNGLCRNLVCTDLFTRGIDI

QAVNVVINFDFPKLAETYLHRIGRSGRFGHLGLAINLITYDDRFNLKSIEEQLGTEIKPIPSNIDKSLYVAEYHSEPVED

DKP

>EOA96312.1 Putative ATP-dependent RNA helicase DDX6, partial [Anas platyrhynchos]

MSTARTENPVIMGLSSQNGQLRGPVKPSGGPGGGGTQTQQQMNQLKNANTINNGTQQQAQSMTTTIKPGDDWKKTLKLPP

KDLRIKTSDVTSTKGNEFEDYCLKRELLMGIFEMGWEKPSPIQEESIPIALSGRDILARAKNGTGKSGAYLIPLLERLDL

KKDNIQAMVIVPTRELALQVSQICIQVSKHMGGAKVMATTGGTNLRDDIMRLDDTVHVVIATPGRILDLIKKGVAKVEHV

QMIVLDEANKLLSQDFVQIMEDIILTLPKNRQILLYSATFPLSVQKFMNSHLQKPYEINLMEELTLKGVTQYYAYVTERQ

KVHCLNTLFSRLQINQSIIFCNSSQRVELLAKKISQLGYSCFYIHAKMRQEHRNRVFHDFRNGLCRNLVCTDLFTRGIDI

QAVNVVINFDFPKLAETYLHRIGRSGRFGHLGLAINLITYDDRFNLKSIEEQLGTEIKPIPSNIDKSLYVAEYHSEPVED

EKQ

>KFV08299.1 putative ATP-dependent RNA helicase DDX6 [Pterocles gutturalis]

MSTARTENPVIMGLSSQNGQLRGPVKPSGGPGGGGTQTQQQMNQLKNTNTINNGTQQQAQSMTTTIKPGDDWKKTLKLPP

KDLRIKTSDVTSTKGNEFEDYCLKRELLMGIFEMGWEKPSPIQEESIPIALSGRDILARAKNGTGKSGAYLIPLLERLDL

KKDNIQAMVIVPTRELALQVSQICIQVSKHMGGAKVMATTGGTNLRDDIMRLDDTVHVVIATPGRILDLIKKGVAKVEHV

QMIVLDEANKLLSQDFVQIMEDIILTLPKNRQILLYSATFPLSVQKFMNSHLQKPYEINLMEELTLKGVTQYYAYVTERQ

KVHCLNTLFSRLQINQSIIFCNSSQRVELLAKKISQLGYSCFYIHAKMRQEHRNRVFHDFRNGLCRNLVCTDLFTRGIDI

QAVNVVINFDFPKLAETYLHRIGRSGRFGHLGLAINLITYDDRFNLKSIEEQLGTEIKPIPSNIDKSLYVAEYHSEPVED

EKQ

>NXN89783.1 DDX6 helicase [Bombycilla garrulus]

MSTARTENPVIMGLSSQNGQLRGPVKPSGGPGVGGTQTQQQMNQLKNANTINNGTQQQAQSMTTAIKPGDDWKKTLKLPP

KDLRIKTSDVTSTKGNEFEDYCLKRELLMGIFEMGWEKPSPIQEESIPIALSGRDILARAKNGTGKSGAYLIPLLERLDL

KKDNIQAMVIVPTRELALQVSQICIQVSKHMGGAKVMATTGGTNLRDDIMRLDDTVHVVIATPGRILDLIKKGVAKVEHV

QMIVLDEANKLLSQDFVQIMEDIILTLPKNRQILLYSATFPLSVQKFMNSHLQKPYEINLMEELTLKGVTQYYAYVTERQ

KVHCLNTLFSRLQINQSIIFCNSSQRVELLAKKISQLGYSCFYIHAKMRQEHRNRVFHDFRNGLCRNLVCTDLFTRGIDI

QAVNVVINFDFPKLAETYLHRIGRSGRFGHLGLAINLITYDDRFNLKSIEEQLGTEIKPIPSNIDKSLYVAEYHSEPVEE

EKQ

>NWS14864.1 DDX6 helicase [Pachyramphus minor]

MSTARTENPVIMGLSSQNGQLRGPVKPSGGPGGGGTQTQQQMNQLKNTNTINNGTQQQAQSMTTAIKPGDDWKKTLKLPP

KDLRIKTSDVTSTKGNEFEDYCLKRELLMGIFEMGWEKPSPIQEESIPIALSGRDILARAKNGTGKSGAYLIPLLERLDL

KKDNIQAMVIVPTRELALQVSQICIQVSKHMGGAKVMATTGGTNLRDDIMRLDDTVHVVIATPGRILDLIKKGVAKVEHV

QMIVLDEANKLLSQDFVQIMEDIILTLPKNRQILLYSATFPLSVQKFMNSHLQKPYEINLMEELTLKGVTQYYAYVTERQ

KVHCLNTLFSRLQINQSIIFCNSSQRVELLAKKISQLGYSCFYIHAKMRQEHRNRVFHDFRNGLCRNLVCTDLFTRGIDI

QAVNVVINFDFPKLAETYLHRIGRSGRFGHLGLAINLITYDDRFNLKSIEEQLGTEIKPIPSNIDKSLYVAEYHSEPVED

EKQ

>NWY89510.1 DDX6 helicase [Loxia curvirostra]

MSTARTENPVIMGLSSQNGQLRGPVKPSGGPGGGGTQTQQQMNQLKNANTINNGTQQQAQSMTTTIKPGDDWKKTLKLPP

KDLRIKTSDVTSTKGNEFEDYCLKRELLMGIFEMGWEKPSPIQEESIPIALSGRDILARAKNGTGKSGAYLIPLLERLDL

KKDNIQAMVIVPTRELALQVSQICIQVSKHMGGAKVMATTGGTNLRDDIMRLDDTVHVVIATPGRILDLIKKGVAKVEHV

QMIVLDEANKLLSQDFVQIMEDIILTLPKNRQILLYSATFPLSVQKFMNSHLQKPYEINLMEELTLKGVTQYYAYVTERQ

KVHCLNTLFSRLQINQSIIFCNSSQRVELLAKKISQLGYSCFYIHAKMRQEHRNRVFHDFRNGLCRNLVCTDLFTRGIDI

QAVNVVINFDFPKLAETYLHRIGRSGRFGHLGLAINLITYDDRFNLKSIEEQLGTEIKPIPSNIDKSLYVAEYHSEPVEE

EKQ

>NWH81858.1 DDX6 helicase [Piaya cayana]

MSTARTENPVIMGLSSQNGQLRGPVKPSGGPGGGGTQTQPQLNPLKTANTINNGTQQQAQSMTTAIKPGDDWKKTLKLPP

KDLRIKTSDVTSTKGNEFEDYCLKRELLMGIFEMGWEKPSPIQEESIPIALSGRDILARAKNGTGKSGAYLIPLLERLDL

KKDNIQAMVIVPTRELALQVSQICIQVSKHMGGAKVMATTGGTNLRDDIMRLDDTVHVVIATPGRILDLIKKGVAKVEHV

QMIVLDEANKLLSQDFVQIMEDIILTLPKNRQILLYSATFPLSVQKFMNSHLQKPYEINLMEELTLKGVTQYYAYVTERQ

KVHCLNTLFSRLQINQSIIFCNSSQRVELLAKKISQLGYSCFYIHAKMRQEHRNRVFHDFRNGLCRNLVCTDLFTRGIDI

QAVNVVINFDFPKLAETYLHRIGRSGRFGHLGLAINLITYDDRFNLKSIEEQLGTEIKPIPSNIDKSLYVAEYHSEPVED

EKQ

>XP_007552379.1 PREDICTED: probable ATP-dependent RNA helicase DDX6 [Poecilia formosa]

MSTTRTENPVILGLSNQNGQLRSSVKPAGAPGGGGGGPQPTQLNQMIKGTINGNSQPAPPTNAVIKPGDDWKKNLKLPPK

DMRIKTSDVTATKGNEFEDYCLKRELLMGIFEMGWEKPSPIQEESIPIALSGRDILARAKNGTGKSGAYLIPLLERIDLK

KDCLQALVIVPTRELALQVSQICIQVSKHMGGVKVMATTGGTNLRDDIMRLDETVHVVIATPGRILDLIKKGVAKVNQVQ

MIVLDEADKLLSQDFVVMMEEMLGFLPKQRQILLYSATFPLSVQKFMNAHLQKPYEINLMEELTLKGVTQYYAYVTERQK

VHCLNTLFSRLQINQSIIFCNSSQRVELLAKKISQLGYSCFYIHAKMRQEHRNRVFHDFRNGLCRNLVCTDLFTRGIDIQ

AVNVVINFDFPKLGETYLHRIGRSGRFGHLGLAINLITYDDRFNLKAIEEQLGTEIKPIPGIIDKSLYVAEYHSETGEEV

KQ

>XP_010007469.1 PREDICTED: probable ATP-dependent RNA helicase DDX6 [Nestor notabilis]

MSTARTENPVIMGLSSQNGQLRGPVKPSGGPGGGGTQTQQQMNQLKNTNTINNGTQQQAQIMTTAIKPGDDWKKTLKLPP

KDLRIKTSDVTSTKGNEFEDYCLKRELLMGIFEMGWEKPSPIQEESIPIALSGRDILARAKNGTGKSGAYLIPLLERLDL

KKDNIQAMVIVPTRELALQVSQICIQVSKHMGGAKVMATTGGTNLRDDIMRLDDTVHVVIATPGRILDLIKKGVAKVEHV

QMIVLDEADKLLSQDFVQIMEDIILTLPKNRQILLYSATFPLSVQKFMNSHLQKPYEINLMEELTLKGVTQYYAYVTERQ

KVHCLNTLFSRLQINQSIIFCNSSQRVELLAKKISQLGYSCFYIHAKMRQEHRNRVFHDFRNGLCRNLVCTDLFTRGIDI

QAVNVVINFDFPKLAETYLHRIGRSGRFGHLGLAINLITYDDRFNLKSIEEQLGTEIKPIPSNIDKSLYVAEYHSEPVED

EKQ

>XP_006912638.1 probable ATP-dependent RNA helicase DDX6 [Pteropus alecto]

MSTARTENPVIMGLSSQNGQLRGPVKPSGGPGGGGTQTQQQMNQLKNTNTINNGTQQQGQSMTTTIKPGDDWKKTLKLPP

KDLRIKTSDVTSTKGNEFEDYCLKRELLMGIFEMGWEKPSPIQEESIPIALSGRDILARAKNGTGKSGAYLIPLLERLDL

KKDNIQAMVIVPTRELALQVSQICIQVSKHMGGAKVMATTGGTNLRDDIMRLDDTVHVVIATPGRILDLIKKGVAKVDHV

QMIVLDEADKLLSQDFVQIMEDIILTLPKNRQILLYSATFPLSVQKFMNSHLQKPYEINLMEELTLKGVTQYYAYVTERQ

KVHCLNTLFSRLQINQSIIFCNSSQRVELLAKKISQLGYSCFYIHAKMRQEHRNRVFHDFRNGLCRNLVCTDLFTRGIDI

QAVNVVINFDFPKLAETYLHRIGRSGRFGHLGLAINLITYDDRFNLKSIEEQLGTEIKPIPSNIDKSLYVAEYHSEPVED

EKP

>XP_001370626.1 PREDICTED: probable ATP-dependent RNA helicase DDX6 [Monodelphis domestica]

MSTARTENPVIMGLSSQNGQLRGPVKPSGGPGGGGTQTQQQMNQLKNTNTINNGTQQQAQSMTTTIKPGDDWKKTLKLPP

KDLRIKTSDVTSTKGNEFEDYCLKRELLMGIFEMGWEKPSPIQEESIPIALSGRDILARAKNGTGKSGAYLIPLLERLDL

KKDNIQAMVIVPTRELALQVSQICIQVSKHMGGAKVMATTGGTNLRDDIMRLDDTVHVVIATPGRILDLIKKGVAKVDHV

QMIVLDEADKLLSQDFVQIMEDIILTLPKNRQILLYSATFPLSVQKFMNSHLQKPYEINLMEELTLKGVTQYYAYVTERQ

KVHCLNTLFSRLQINQSIIFCNSSQRVELLAKKISQLGYSCFYIHAKMRQEHRNRVFHDFRNGLCRNLVCTDLFTRGIDI

QAVNVVINFDFPKLAETYLHRIGRSGRFGHLGLAINLITYDDRFNLKSIEEQLGTEIKPIPSNIDKSLYVAEYHSEPVED

EKP

>KAF4072131.1 hypothetical protein AMELA_G00270840 [Ameiurus melas]

MSAARTENPVILGLTNQNGQKRGSVKPTGGPGGCGSQTQPAQIKASSTVNNGNSLPVPTANTIKHDLCFVRPGDDWKKNL

KLPPKDMRMKTSDVTATKGNEFEDYCLKRELLMGIFEMGWEKPSPIQEESIPIALSGRDILARAKNGTGKSGAYLIPLLE

RIDLKKDCIQALVIVPTRELALQVSQICIQVSKHMGGVKVMATTGGTNLRDDIMRLDETVHVVIATPGRVLDLIKKGVAK

VGQVQMIVLDEADKLLSQDFVQMMEEILSFLPKQRQILLYSATFPLSVQKFMNAHLQKPYEINLMEELTLKGVTQYYAYV

TERQKVHCLNTLFSRLQINQSIIFCNSSQRVELLAKKISQLGYSCFYIHAKMRQEHRNRVFHDFRNGLCRNLVCTDLFTR

GIDIQAVNVVINFDFPKLGETYLHRIGRSGRFGHLGLAINLITYDDRFNLKGIEEQLGTEIKPIPSSIDKSLYVAEYHSE

SAEEVKL

>NWY77870.1 DDX6 helicase [Rhegmatorhina hoffmannsi]

MSTARTENPVIMGLSSQNGQLRGPVKPSGGPGGGGTTSQQQMNQLKNANTINNGTQQQAQSMSTAIKPGDDWKKTLKLPP

KDLRIKTSDVTSTKGNEFEDYCLKRELLMGIFEMGWEKPSPIQEESIPIALSGRDILARAKNGTGKSGAYLIPLLERLDL

KKDNIQAMVIVPTRELALQVSQICIQVSKHMGGAKVMATTGGTNLRDDIMRLDDTVHVVIATPGRILDLIKKGVAKVEHV

QMIVLDEANKLLSQDFVQIMEDIILTLPKNRQILLYSATFPLSVQKFMNSHLQKPYEINLMEELTLKGVTQYYAYVTERQ

KVHCLNTLFSRLQINQSIIFCNSSQRVELLAKKISQLGYSCFYIHAKMRQEHRNRVFHDFRNGLCRNLVCTDLFTRGIDI

QAVNVVINFDFPKLAETYLHRIGRSGRFGHLGLAINLITYDDRFNLKSIEEQLGTEIKPIPSNIDKSLYVAEYHSEPVED

EKQ

>NWV94028.1 DDX6 helicase [Machaerirhynchus nigripectus]

MSTARTENPVIMGLSSQNGQLRGPVKPSGGPGGGGTQTQQQMNQLKNANAINNGTQQQAQSMTTTIKPGDDWKKTLKLPP

KDLRIKTSDVTSTKGNEFEDYCLKRELLMGIFEMGWEKPSPIQEESIPIALSGRDILARAKNGTGKSGAYLIPLLERLDL

KKDNIQAMVIVPTRELALQVSQICIQVSKHMGGAKVMATTGGTNLRDDIMRLDDTVHVVIATPGRILDLIKKGVAKVEHV

QMIVLDEANKLLSQDFVQIMEDIILTLPKNRQILLYSATFPLSVQKFMNSHLQKPYEINLMEELTLKGVTQYYAYVTERQ

KVHCLNTLFSRLQINQSIIFCNSSQRVELLAKKISQLGYSCFYIHAKMRQEHRNRVFHDFRNGLCRNLVCTDLFTRGIDI

QAVNVVINFDFPKLAETYLHRIGRSGRFGHLGLAINLITYDDRFNLKSIEEQLGTEIKPIPSNIDKSLYVAEYHSEPVED

EKQ

>XP_005856840.1 PREDICTED: probable ATP-dependent RNA helicase DDX6 [Myotis brandtii]

MSTARTENPVIMGLSSQNGQLRGPVKPSGGPGGGGTPTQQQMNQLKNTNTINNGTQQQAQSMTTTIKPGDDWKKTLKLPP

KDLRIKTSDVTSTKGNEFEDYCLKRELLMGIFEMGWEKPSPIQEESIPIALSGRDILARAKNGTGKSGAYLIPLLERLDL

KKDNIQAMVIVPTRELALQVSQICIQVSKHMGGAKVMATTGGTNLRDDIMRLDDTVHVVIATPGRILDLIKKGVAKVDHV

QMIVLDEADKLLSQDFVQIMEDIILTLPKNRQILLYSATFPLSVQKFMNSHLQKPYEINLMEELTLKGVTQYYAYVTERQ

KVHCLNTLFSRLQINQSIIFCNSSQRVELLAKKISQLGYSCFYIHAKMRQEHRNRVFHDFRNGLCRNLVCTDLFTRGIDI

QAVNVVINFDFPKLAETYLHRIGRSGRFGHLGLAINLITYDDRFNLKSIEEQLGTEIKPIPSNIDKSLYVAEYHSEPVED

DKP

>KFV74826.1 putative ATP-dependent RNA helicase DDX6 [Picoides pubescens]

MSTARTENPVIMGLSSQNGQLRGPVKPSGGPGGGGPPTQQQMNQLKNANTINNGTQQQAQSMTTAIKPGDDWKKTLKLPP

KDLRIKTSDVTSTKGNEFEDYCLKRELLMGIFEMGWEKPSPIQEESIPIALSGRDILARAKNGTGKSGAYLIPLLERLDL

KKDNIQAMVIVPTRELALQVSQICIQVSKHMGGAKVMATTGGTNLRDDIMRLDDTVHVVIATPGRILDLIKKGVAKVEHV

QMIVLDEANKLLSQDFVQIMEDIILTLPKNRQILLYSATFPLSVQKFMNSHLQKPYEINLMEELTLKGVTQYYAYVTERQ

KVHCLNTLFSRLQINQSIIFCNSSQRVELLAKKISQLGYSCFYIHAKMRQEHRNRVFHDFRNGLCRNLVCTDLFTRGIDI

QAVNVVINFDFPKLAETYLHRIGRSGRFGHLGLAINLITYDDRFNLKSIEEQLGTEIKPIPSNIDKSLYVAEYHSEPVED

EKQ

>XP_004385709.1 probable ATP-dependent RNA helicase DDX6 isoform X2 [Trichechus manatus latirostris]

MSTARTENPVIMGLSSQNGQLRGPVKPSGGPGGGGTQTQQQMNQLKNTNTINNGTQQQAQSMTTAIKPGDDWKKTLKLPP

KDLRIKTSDVTSTKGNEFEDYCLKRELLMGIFEMGWEKPSPIQEESIPIALSGRDILARAKNGTGKSGAYLIPLLERLDL

KKDNIQAMVIVPTRELALQVSQICIQVSKHMGGAKVMATTGGTNLRDDIMRLDDTVHVVIATPGRILDLIKKGVAKVDHV

QMIVLDEADKLLSQDFVQIMEDIILTLPKNRQILLYSATFPLSVQKFMNSHLQKPYEINLMEELTLKGVTQYYAYVTERQ

KVHCLNTLFSRLQINQSIIFCNSSQRVELLAKKISQLGYSCFYIHAKMRQEHRNRVFHDFRNGLCRNLVCTDLFTRGIDI

QAVNVVINFDFPKLAETYLHRIGRSGRFGHLGLAINLITYDDRFNLKSIEEQLGTEIKPIPSNIDKSLYVAEYHSEPVED

EKP

>NWI99922.1 DDX6 helicase [Crypturellus undulatus]

MSTARTENPVIMGLSSQNGQLRGPVKPSGGPGGGGTQTQQQMNQLKNANTINNGTQQQAQSMTTTIKPGDDWKKTLKLPP

KDLRIKTSDVTSTKGNEFEDYCLKRELLMGIFEMGWEKPSPIQEESIPIALSGRDILARAKNGTGKSGAYLIPLLERLDL

KKDNIQAMVIVPTRELALQVSQICIQVSKHMGGAKVMATTGGTNLRDDIMRLDDTVHVVIATPGRILDLIKKGVAKVEHV

QMIVLDEANKLLSQDFVQIMEDIILTLPKNRQILLYSATFPLSVQKFMNSHLQKPYEINLMEELTLKGVTQYYAYVTERQ

KVHCLNTLFSRLQINQSIIFCNSSQRVELLAKKISQLGYSCFYIHAKMRQEHRNRVFHDFRNGLCRNLVCTDLFTRGIDI

QAVNVVINFDFPKLAETYLHRIGRSGRFGHLGLAINLITYDDRFNLKSIEEQLGTEIKPIPSNIDKSLYVAEYHSEPVED

EKP

>XP_030361453.1 probable ATP-dependent RNA helicase DDX6 isoform X1 [Strigops habroptila]

MSTARTENPVIMGLSSQNGQLRGPVKPSGGPGGGGTQTQQQMNQLKNANTINNGTQQQAQIMTTAIKPGDDWKKTLKLPP

KDLRIKTSDVTSTKGNEFEDYCLKRELLMGIFEMGWEKPSPIQEESIPIALSGRDILARAKNGTGKSGAYLIPLLERLDL

KKDNIQAMVIVPTRELALQVSQICIQVSKHMGGAKVMATTGGTNLRDDIMRLDDTVHVVIATPGRILDLIKKGVAKVEHV

QMIVLDEADKLLSQDFVQIMEDIILTLPKNRQILLYSATFPLSVQKFMNSHLQKPYEINLMEELTLKGVTQYYAYVTERQ

KVHCLNTLFSRLQINQSIIFCNSSQRVELLAKKISQLGYSCFYIHAKMRQEHRNRVFHDFRNGLCRNLVCTDLFTRGIDI

QAVNVVINFDFPKLAETYLHRIGRSGRFGHLGLAINLITYDDRFNLKSIEEQLGTEIKPIPSNIDKSLYVAEYHSEPVED

EKQ

>NXG22217.1 DDX6 helicase [Grallaria varia]

MSTARTENPVIMGLSSQNGQLRGPVKPSGGPGGGGTQTQQQMNQLKTTNTINNGTQQQAQSMTTAIKPGDDWKKTLKLPP

KDLRIKTSDVTSTKGNEFEDYCLKRELLMGIFEMGWEKPSPIQEESIPIALSGRDILARAKNGTGKSGAYLIPLLERLDL

KKDNIQAMVIVPTRELALQVSQICIQVSKHMGGAKVMATTGGTNLRDDIMRLDDTVHVVIATPGRILDLIKKGVAKVEHV

QMIVLDEANKLLSQDFVQIMEDIILTLPKNRQILLYSATFPLSVQKFMNSHLQKPYEINLMEELTLKGVTQYYAYVTERQ

KVHCLNTLFSRLQINQSIIFCNSSQRVELLAKKISQLGYSCFYIHAKMRQEHRNRVFHDFRNGLCRNLVCTDLFTRGIDI

QAVNVVINFDFPKLAETYLHRIGRSGRFGHLGLAINLITYDDRFNLKSIEEQLGTEIKPIPSNIDKSLYVAEYHSEPVED

EKQ

>XP_006890900.1 PREDICTED: probable ATP-dependent RNA helicase DDX6 [Elephantulus edwardii]

MSTARTENPVIMGLSSQNGQLRGPVKPSGGPGGGGTQTQQQMNQLKNANAINNGTQQQAQSMTTTIKPGDDWKKTLKLPP

KDLRIKTSDVTSTKGNEFEDYCLKRELLMGIFEMGWEKPSPIQEESIPIALSGRDILARAKNGTGKSGAYLIPLLERLDL

KKDNIQAMVIVPTRELALQVSQICIQVSKHMGGAKVMATTGGTNLRDDIMRLDDTVHVVIATPGRILDLIKKGVAKVDHV

QMIVLDEADKLLSQDFVQIMEDIILTLPKNRQILLYSATFPLSVQKFMNSHLQKPYEINLMEELTLKGVTQYYAYVTERQ

KVHCLNTLFSRLQINQSIIFCNSSQRVELLAKKISQLGYSCFYIHAKMRQEHRNRVFHDFRNGLCRNLVCTDLFTRGIDI

QAVNVVINFDFPKLAETYLHRIGRSGRFGHLGLAINLITYDDRFNLKSIEEQLGTEIKPIPSNIDKSLYVAEYHSEPVED

EKP

>XP_010007187.1 PREDICTED: probable ATP-dependent RNA helicase DDX6 isoform X2 [Chaetura pelagica]

MSTARTENPVIMGLSSQNGQLRGPVKPSGGPGGGGTQTQQQMNQLKNANTINNGTQQQAQSMTTAIKPGDDWKKTLKLPP

KDLRIKTSDVTSTKGNEFEDYCLKRELLMGIFEMGWEKPSPIQEESIPIALSGRDILARAKNGTGKSGAYLIPLLERLDL

KKDNIQAMVIVPTRELALQVSQICIQVSKHMGGAKVMATTGGTNLRDDIMRLDDTVHVVIATPGRILDLIKKGVAKVEHV

QMIVLDEANKLLSQDFVQIMEDIILTLPKNRQILLYSATFPLSVQKFMNSHLQKPYEINLMEELTLKGVTQYYAYVTERQ

KVHCLNTLFSRLQINQSIIFCNSSQRVELLAKKISQLGYSCFYIHAKMRQEHRNRVFHDFRNGLCRNLVCTDLFTRGIDI

QAVNVVINFDFPKLAETYLHRIGRSGRFGHLGLAINLITYDDRFNLKSIEEQLGTEIKPIPSNIDKSLYVAEYHSEPVED

EKQ

>NXO71909.1 DDX6 helicase [Phainopepla nitens]

MSTARTENPVIMGLSSQNGQLRGPVKPSGGPGGGGTQTQQQMNQLKNANTINNGTQQQAQSMTTAIKPGDDWKKTLKLPP

KDLRIKTSDVTSTKGNEFEDYCLKRELLMGIFEMGWEKPSPIQEESIPIALSGRDILARAKNGTGKSGAYLIPLLERLDL

KKDNIQAMVIVPTRELALQVSQICIQVSKHMGGAKVMATTGGTNLRDDIMRLDDTVHVVIATPGRILDLIKKGVAKVEHV

QMIVLDEANKLLSQDFVQIMEDIILTLPKNRQILLYSATFPLSVQKFMNSHLQKPYEINLMEELTLKGVTQYYAYVTERQ

KVHCLNTLFSRLQINQSIIFCNSSQRVELLAKKISQLGYSCFYIHAKMRQEHRNRVFHDFRNGLCRNLVCTDLFTRGIDI

QAVNVVINFDFPKLAETYLHRIGRSGRFGHLGLAINLITYDDRFNLKSIEEQLGTEIKPIPSNIDKSLYVAEYHSEPVEE

EKQ

>XP_027900781.1 probable ATP-dependent RNA helicase DDX6 [Xiphophorus couchianus]

MSTIRTENPVILGLSNQNGQLRSSVKPGGAPGGGGGGPQPPQLNQMIKGAINGNSQSAPPTNAVIKPGDDWKKNLKLPPK

DMRIKTSDVTATKGNEFEDYCLKRELLMGIFEMGWEKPSPIQEESIPIALSGRDILARAKNGTGKSGAYLIPLLERIDLK

KDCLQALVIVPTRELALQVSQISIQVSKHMGGVKVMATTGGTNLRDDIMRLDETVHVVIATPGRILDLIKKGVAKVNQVQ

MIVLDEADKLLSQDFVVMMEEMLGFLPKKRQILLYSATFPLSVQKFMNAHLQKPYEINLMEELTLKGVTQYYAYVTERQK

VHCLNTLFSRLQINQSIIFCNSSQRVELLAKKISQLGYSCFYIHAKMRQEHRNRVFHDFRNGLCRNLVCTDLFTRGIDIQ

AVNVVINFDFPKLGETYLHRIGRSGRFGHLGLAINLITYDDRFNLKAIEEQLGTEIKPIPGIIDKSLYVAEYHSESGEEV

KQ

>XP_006833954.1 PREDICTED: probable ATP-dependent RNA helicase DDX6 isoform X1 [Chrysochloris asiatica]

MSTARTENPVIMGLSSQNGQLRGPVKPSGGPGGGGTQTQQQLNQLKNTNTINNGTQQQAQSMTTTIKPGDDWKKTLKLPP

KDLRIKTSDVTSTKGNEFEDYCLKRELLMGIFEMGWEKPSPIQEESIPIALSGRDILARAKNGTGKSGAYLIPLLERLDL

KKDNIQAMVIVPTRELALQVSQICIQVSKHMGGAKVMATTGGTNLRDDIMRLDDTVHVVIATPGRILDLIKKGVAKVDHV

QMIVLDEADKLLSQDFVQIMEDIILTLPKNRQILLYSATFPLSVQKFMNSHLQKPYEINLMEELTLKGVTQYYAYVTERQ

KVHCLNTLFSRLQINQSIIFCNSSQRVELLAKKISQLGYSCFYIHAKMRQEHRNRVFHDFRNGLCRNLVCTDLFTRGIDI

QAVNVVINFDFPKLAETYLHRIGRSGRFGHLGLAINLITYDDRFNLKSIEEQLGTEIKPIPSNIDKSLYVAEYHSEPVED

EKP

>NXC17464.1 DDX6 helicase [Corythaeola cristata]

MSTARTENPVIMGLSSQNGQLRGPVKPSGGPGGGGTPTQQQMNQLKNANAINNGTQQQAQSMTTAIKPGDDWKKTLKLPP

KDLRIKTSDVTSTKGNEFEDYCLKRELLMGIFEMGWEKPSPIQEESIPIALSGRDILARAKNGTGKSGAYLIPLLERLDL

KKDNIQAMVIVPTRELALQVSQICIQVSKHMGGAKVMATTGGTNLRDDIMRLDDTVHVVIATPGRILDLIKKGVAKVEHV

QMIVLDEANKLLSQDFVQIMEDIILTLPKNRQILLYSATFPLSVQKFMNSHLQKPYEINLMEELTLKGVTQYYAYVTERQ

KVHCLNTLFSRLQINQSIIFCNSSQRVELLAKKISQLGYSCFYIHAKMRQEHRNRVFHDFRNGLCRNLVCTDLFTRGIDI

QAVNVVINFDFPKLAETYLHRIGRSGRFGHLGLAINLITYDDRFNLKSIEEQLGTEIKPIPSNIDKSLYVAEYHSEPVED

EKQ

>KAF3823643.1 hypothetical protein GH733_007111 [Mirounga leonina]

MTIVMSTARTENPVIMGLSSQNGQLRGPVKPSGGPGGGGTQTQQQMNQLKNTNTINNGTQQQAQSMTTTIKPGDDWKKTL

KLPPKDLRIKTSDVTSTKGNEFEDYCLKRELLMGIFEMGWEKPSPIQEESIPIALSGRDILARAKNGTGKSGAYLIPLLE

RLDLKKDNIQAMVIVPTRELALQVSQICIQVSKHMGGAKVMATTGGTNLRDDIMRLDDTVHVVIATPGRILDLIKKGVAK

VDHVQMIVLDEADKLLSQDFVQIMEDIILTLPKNRQILLYSATFPLSVQKFMNSHLQKPYEINLMEELTLKGVTQYYAYV

TERQKVHCLNTLFSRLQINQSIIFCNSSQRVELLAKKISQLGYSCFYIHAKMRQEHRNRVFHDFRNGLCRNLVCTDLFTR

GIDIQAVNVVINFDFPKLAETYLHRIGRSGRFGHLGLAINLITYDDRFNLKSIEEQLGTEIKPIPSNIDKSLYVAEYHSE

PVEDEKP

>NXG09482.1 DDX6 helicase [Sakesphorus luctuosus]

MSTARTENPVIMGLSSQNGQLRGPVKPSGGPGGGGTQSQQQMNQLKNANTINNGTQQQAQSMTTAIKPGDDWKKTLKLPP

KDLRIKTSDVTSTKGNEFEDYCLKRELLMGIFEMGWEKPSPIQEESIPIALSGRDILARAKNGTGKSGAYLIPLLERLDL

KKDNIQAMVIVPTRELALQVSQICIQVSKHMGGAKVMATTGGTNLRDDIMRLDDTVHVVIATPGRILDLIKKGVAKVEHV

QMIVLDEANKLLSQDFVQIMEDIILTLPKNRQILLYSATFPLSVQKFMNSHLQKPYEINLMEELTLKGVTQYYAYVTERQ

KVHCLNTLFSRLQINQSIIFCNSSQRVELLAKKISQLGYSCFYIHAKMRQEHRNRVFHDFRNGLCRNLVCTDLFTRGIDI

QAVNVVINFDFPKLAETYLHRIGRSGRFGHLGLAINLITYDDRFNLKSIEEQLGTEIKPIPSNIDKSLYVAEYHSEPVED

EKQ

>XP_029374077.1 probable ATP-dependent RNA helicase DDX6 [Echeneis naucrates]

MSTARTENPVILGMSNQNGQLRGSVKPAGAPGGGGGGPQQQQLNQMKGTINNGNTQPAPTTNAVIKPGDDWKKNLKLPPK

DMRMKTSDVTATKGNEFEDYCLKRELLMGIFEMGWEKPSPIQEESIPIALSGRDILARAKNGTGKSGAYLIPLLERIDLK

KDCIQALVIVPTRELALQVSQICIQVSKHMGGVKVMATTGGTNLRDDIMRLDETVHVVIATPGRILDLIKKGVAKVNQVQ

MIVLDEADKLLSQDFVVMMEEILGFLPKQRQILLYSATFPLSVQKFMNSHLQKPYEINLMEELTLKGVTQYYAYVTERQK

VHCLNTLFSRLQINQSIIFCNSSQRVELLAKKISQLGYSCFYIHAKMRQEHRNRVFHDFRNGLCRNLVCTDLFTRGIDIQ

AVNVVINFDFPKLGETYLHRIGRSGRFGHLGLAINLITYDDRFNLKGIEEQLGTEIKPIPGIIDKSLYVAEYHSESGEEV

KP

>KFQ27323.1 putative ATP-dependent RNA helicase DDX6 [Merops nubicus]

MSTARTENPVIMGLSSQNGQLRGPVKPSGGPGGGGTQTQQQMNQLKNANTINNGTQQQAQSMTAAIKPGDDWKKTLKLPP

KDLRIKTSDVTSTKGNEFEDYCLKRELLMGIFEMGWEKPSPIQEESIPIALSGRDILARAKNGTGKSGAYLIPLLERLDL

KKDNIQAMVIVPTRELALQVSQICIQVSKHMGGAKVMATTGGTNLRDDIMRLDDTVHVVIATPGRILDLIKKGVAKVEHV

QMIVLDEANKLLSQDFVQIMEDIILTLPKNRQILLYSATFPLSVQKFMNSHLQKPYEINLMEELTLKGVTQYYAYVTERQ

KVHCLNTLFSRLQINQSIIFCNSSQRVELLAKKISQLGYSCFYIHAKMRQEHRNRVFHDFRNGLCRNLVCTDLFTRGIDI

QAVNVVINFDFPKLAETYLHRIGRSGRFGHLGLAINLITYDDRFNLKSIEEQLGTEIKPIPSNIDKSLYVAEYHSEPVED

EKQ

>KFP05525.1 putative ATP-dependent RNA helicase DDX6 [Calypte anna]

MSTARTENPVIMGLSSQNGQLRGPVKPSGGSGGGGTQTQQQMNQLKNANTINNGTQQQAQSMTTAIKPGDDWKKTLKLPP

KDLRIKTSDVTSTKGNEFEDYCLKRELLMGIFEMGWEKPSPIQEESIPIALSGRDILARAKNGTGKSGAYLIPLLERLDL

KKDNIQAMVIVPTRELALQVSQICIQVSKHMGGAKVMATTGGTNLRDDIMRLDDTVHVVIATPGRILDLIKKGVAKVEHV

QMIVLDEANKLLSQDFVQIMEDIILTLPKNRQILLYSATFPLSVQKFMNSHLQKPYEINLMEELTLKGVTQYYAYVTERQ

KVHCLNTLFSRLQINQSIIFCNSSQRVELLAKKISQLGYSCFYIHAKMRQEHRNRVFHDFRNGLCRNLVCTDLFTRGIDI

QAVNVVINFDFPKLAETYLHRIGRSGRFGHLGLAINLITYDDRFNLKSIEEQLGTEIKPIPSNIDKSLYVAEYHSEPVED

EKQ

>XP_015239666.1 PREDICTED: probable ATP-dependent RNA helicase DDX6 [Cyprinodon variegatus]

MSTTRTENPVILGLSNQNGQLRGSVKPTGAPGGGLQQPQINQMIKGTINGNSQPAPPTNAVIKPGDDWKKNLKVPPKDMR

IKTSDVTATKGNEFEDYCLKRELLMGIFEMGWEKPSPIQEESIPIALSGRDILARAKNGTGKSGAYLIPLLERIDLKKDC

LQALVIVPTRELALQVSQICIQVSKHMGGVKVMATTGGTNLRDDIMRLDETVHVVIATPGRILDLIKKGVAKVNQVQMVV

LDEADKLLSQDFVIMMEEMLGFLPKQRQILLYSATFPLSVQKFMNAHLQKPYEINLMEELTLKGVTQYYAYVTERQKVHC

LNTLFSRLQINQSIIFCNSSQRVELLAKKISQLGYSCFYIHAKMRQEHRNRVFHDFRNGLCRNLVCTDLFTRGIDIQAVN

VVINFDFPKLGETYLHRIGRSGRFGHLGLAINLITYDDRFNLKAIEEQLGTEIKPIPGIIDKSLYVAEYHSESGEEVKQ

>NXU58375.1 DDX6 helicase [Turnix velox]

MSTARTENPVIMGLSSQNGQLRGPVKPSGGPGGGGTQTQQQMNQLKNANTINNGTQQQAQNMTTAIKPGDDWKKTLKLPP

KDLRIKTSDVTSTKGNEFEDYCLKRELLMGIFEMGWEKPSPIQEESIPIALSGRDILARAKNGTGKSGAYLIPLLERLDL

KKDNIQAMVIVPTRELALQVSQICIQVSKHMGGAKVMATTGGTNLRDDIMRLDDTVHVVIATPGRILDLIKKGVAKVEHV

QMIVLDEANKLLSQDFVQIMEDIILTLPKNRQILLYSATFPLSVQKFMNSHLQKPYEINLMEELTLKGVTQYYAYVTERQ

KVHCLNTLFSRLQINQSIIFCNSSQRVELLAKKISQLGYSCFYIHAKMRQEHRNRVFHDFRNGLCRNLVCTDLFTRGIDI

QAVNVVINFDFPKLAETYLHRIGRSGRFGHLGLAINLITYDDRFNLKSIEEQLGTEIKPIPSNIDKSLYVAEYHSEPVED

EKQ

>XP_018620200.1 probable ATP-dependent RNA helicase DDX6 [Scleropages formosus]

MSTARTENPVILGLPSQNGQIRGSVKPAGGPGGGGGGPQLQPSSQIKASSTINNGSSQPMPTANTVIKPGDDWKKNLKIP

PKDLRMKTSDVTATKGNEFEDYCLKRELLMGIFEMGWEKPSPIQEESIPIALSGRDILARAKNGTGKSGAYLIPLLERID

LKKDCIQALVIVPTRELALQVSQICIQVSKHMGGVKVMATTGGTNLRDDIMRLDETVHVVIATPGRILDLIKKGVAKVDQ

VQMIVLDEADKLLSQDFVQMMEEILSFLPKQRQILLYSATFPLSVQKFMTSHLQKPYEINLMEELTLKGVTQYYAYVTER

QKVHCLNTLFSRLQINQSIIFCNSSQRVELLAKKISQLGYSCFYIHAKMRQEHRNRVFHDFRNGLCRNLVCTDLFTRGID

IQAVNVVINFDFPKLGETYLHRIGRSGRFGHLGLAINLITYDDRFNLKGIEEQLGTEIKPIPGNIDKSLYVAEYHSESGE

ELKL

>XP_005796898.1 probable ATP-dependent RNA helicase DDX6 [Xiphophorus maculatus]

MSTTRTENPVILGLSNQNGQLRSSVKPGGAPGGGGGGPQPPQLNQMIKGAINGNSQSAPPTNAVIKPGDDWKKNLKLPPK

DMRIKTSDVTATKGNEFEDYCLKRELLMGIFEMGWEKPSPIQEESIPIALSGRDILARAKNGTGKSGAYLIPLLERIDLK

KDCLQALVIVPTRELALQVSQISIQVSKHMGGVKVMATTGGTNLRDDIMRLDETVHVVIATPGRILDLIKKGVAKVNQVQ

MIVLDEADKLLSQDFVVMMEEMLGFLPKKRQILLYSATFPLSVQKFMNAHLQKPYEINLMEELTLKGVTQYYAYVTERQK

VHCLNTLFSRLQINQSIIFCNSSQRVELLAKKISQLGYSCFYIHAKMRQEHRNRVFHDFRNGLCRNLVCTDLFTRGIDIQ

AVNVVINFDFPKLGETYLHRIGRSGRFGHLGLAINLITYDDRFNLKAIEEQLGTEIKPIPGIIDKSLYVAEYHSESGEEV

KQ

>NWH48779.1 DDX6 helicase [Fregata magnificens]

MSTARTENPVIMGLSSQNGQLRGPVKPSGGPGGGGTQTQQQMNQLKNANAINNGTQQQAQSMTTAIKPGDDWKKTLKLPP

KDLRIKTSDVTSTKGNEFEDYCLKRELLMGIFEMGWEKPSPIQEESIPIALSGRDILARAKNGTGKSGAYLIPLLERLDL

KKDNIQAMVIVPTRELALQVSQICIQVSKHMGGAKVMATTGGTNLRDDIMRLDDTVHVVIATPGRILDLIKKGVAKVEHV

QMIVLDEANKLLSQDFVQIMEDIILTLPKNRQILLYSATFPLSVQKFMNSHLQKPYEINLMEELTLKGVTQYYAYVTERQ

KVHCLNTLFSRLQINQSIIFCNSSQRVELLAKKISQLGYSCFYIHAKMRQEHRNRVFHDFRNGLCRNLVCTDLFTRGIDI

QAVNVVINFDFPKLAETYLHRIGRSGRFGHLGLAINLITYDDRFNLKSIEEQLGTEIKPIPSNIDKSLYVAEYHSEPVED

EKQ

>KAF7699188.1 hypothetical protein HF521_003930 [Silurus meridionalis]

MSTARTENPVILGLTNQNGQKRATAKPTGGPGGGGGGPQTQPAQIKSSSTVNNGNSLPVPTANTVIKPGDDWKKNLKLPP

KDMRMRTSDVTATKGNEFEDYCLKRELLMGIFEMGWEKPSPIQEESIPIALSGRDILARAKNGTGKSGAYLIPLLERIDL

KKDCIQALVIVPTRELALQVSQICIQVSKHMGGVKVMATTGGTNLRDDIMRLDETVHVVIATPGRVLDLIKKGVAKVGQV

QMIVLDEADKLLSQDFVQMMEEILSFLPKQRQILLYSATFPLSVQKFMNSHLQKPYEINLMEELTLKGVTQYYAYVTERQ

KVHCLNTLFSRLQINQSIIFCNSSQRVELLAKKISQLGYSCFYIHAKMRQEHRNRVFHDFRNGLCRNLVCTDLFTRGIDI

QAVNVVINFDFPKLGETYLHRIGRSGRFGHLGLAINLITYDDRFNLKGIEEQLGTEIKPIPSSIDKSLYVAEYHSESAEE

VKL

>XP_030621640.1 probable ATP-dependent RNA helicase DDX6 [Chanos chanos]

MSTARTENPVILGLSNQNGQLRGSVKPAGGPGGGGGGPQTTQPTQIKASSTINNGSSQPVPTANTVIKPGDDWKKNLKLP

PKDMRMKTSDVTATKGNEFEDYCLKRELLMGIFEMGWEKPSPIQEESIPIALSGRDILARAKNGTGKSGAYLIPLLERID

LKKDCIQALVIVPTRELALQVSQICIQVSKHMGGVKVMATTGGTNLRDDIMRLDETVHVVIATPGRILDLIKKGVAKVSQ

VQMIVLDEADKLLSQDFVQMMEEILSFLPKQRQILLYSATFPLSVQKFMNAHLQKPYEINLMEELTLKGVTQYYAYVTER

QKVHCLNTLFSRLQINQSIIFCNSSQRVELLAKKISQLGYSCFYIHAKMRQEHRNRVFHDFRNGLCRNLVCTDLFTRGID

IQAVNVVINFDFPKLGETYLHRIGRSGRFGHLGLAINLITYDDRFNLKGIEEQLGTEIKPIPSSIDKSLYVAEYHSESGE

EVKA

>XP_002722747.1 PREDICTED: probable ATP-dependent RNA helicase DDX6 [Oryctolagus cuniculus]

MSTARTENPVIMGLSSQNGQLRGPVKPSGGPGGGGTQTQQQMNQLKTTNTINNGTQQQAQSLTTTIKPGDDWKKTLKLPP

KDLRIKTSDVTSTKGNEFEDYCLKRELLMGIFEMGWEKPSPIQEESIPIALSGRDILARAKNGTGKSGAYLIPLLERLDL

KKDNIQAMVIVPTRELALQVSQICIQVSKHMGGAKVMATTGGTNLRDDIMRLDDTVHVVIATPGRILDLIKKGVAKVDHV

QMIVLDEADKLLSQDFVQIMEDIILTLPKNRQILLYSATFPLSVQKFMNSHLQKPYEINLMEELTLKGVTQYYAYVTERQ

KVHCLNTLFSRLQINQSIIFCNSSQRVELLAKKISQLGYSCFYIHAKMRQEHRNRVFHDFRNGLCRNLVCTDLFTRGIDI

QAVNVVINFDFPKLAETYLHRIGRSGRFGHLGLAINLITYDDRFNLKSIEEQLGTEIKPIPSNIDKSLYVAEYHSEPVED

EKP

>XP_026876878.1 probable ATP-dependent RNA helicase ddx6 [Electrophorus electricus]

MSTARTENPVILGLSNQNGQMRGSVKPPGGPGGGGGGAPTTQPTQIKAPSTINNGNSQPVSTANTVIKPGDDWKKNLKLP

PKDMRMKTSDVTATKGNEFEDYCLKRELLMGIFEMGWEKPSPIQEESIPIALSGRDILARAKNGTGKSGAYLIPLLERID

LKKDCIQALVIVPTRELALQVSQICIQVSKHMGGVKVMATTGGTNLRDDIMRLDETVHVVIATPGRILDLIKKGVAKVGQ

VQMIVLDEADKLLSQDFVQMMEEILSFLPKQRQILLYSATFPLSVQKFMNSHLQKPYEINLMEELTLKGVTQYYAYVTER

QKVHCLNTLFSRLQINQSIIFCNSSQRVELLAKKISQLGYSCFYIHAKMRQEHRNRVFHDFRNGLCRNLVCTDLFTRGID

IQAVNVVINFDFPKLGETYLHRIGRSGRFGHLGLAINLITYDDRFNLKGIEEQLGTEIKPIPSSIDKSLYVAEYHSESSE

EVKQ

>NXT70731.1 DDX6 helicase [Chaetops frenatus]

MSTARTENPVIMGLSSQNGQLRGPVKPSGGPGGGGTQTQQQMNQLKNANTINNGTQQQAQSMTTAIKPGDDWKKTLKLPP

KDLRIKTSDVTSTKGNEFEDYCLKRELLMGIFEMGWEKPSPIQEESIPIALSGRDILARAKNGTGKSGAYLIPLLERLDL

KKDNIQAMVIVPTRELALQVSQICIQVSKHMGGAKVMATTGGTNLRDDIMRLDDTVHVVIATPGRILDLIKKGVAKVEHV

QMIVLDEANKLLSQDFVQIMEDIILTLPKNRQILLYSATFPLSVQKFMNSHLQKPYEINLMEELTLKGVTQYYAYVTERQ

KVHCLNTLFSRLQINQSIIFCNSSQRVELLAKKISQLGYSCFYIHAKMRQEHRNRVFHDFRNGLCRNLVCTDLFTRGIDI

QAVNVVINFDFPKLAETYLHRIGRSGRFGHLGLAINLITYDDRFNLKSIEEQLGTEIKPIPSNIDKSLYVAEYHSEPGED

EKQ

>NWW99051.1 DDX6 helicase [Caloenas nicobarica]

MSTARTENPVIMGLSSQNGQLRGPVKPSGGPGGGGTQTQQQMNQLKNANTINNGTQQQAQSMTTTLKPGDDWKKTLKLPP

KDLRIKTSDVTSTKGNEFEDYCLKRELLMGIFEMGWEKPSPIQEESIPIALSGRDILARAKNGTGKSGAYLIPLLERLDL

KKDNIQAMVIVPTRELALQVSQICIQVSKHMGGAKVMATTGGTNLRDDIMRLDDTVHVVIATPGRILDLIKKGVAKVEHV

QMIVLDEANKLLSQDFVQIMEDIILTLPKNRQILLYSATFPLSVQKFMNSHLQKPYEINLMEELTLKGVTQYYAYVTERQ

KVHCLNTLFSRLQINQSIIFCNSSQRVELLAKKISQLGYSCFYIHAKMRQEHRNRVFHDFRNGLCRNLVCTDLFTRGIDI

QAVNVVINFDFPKLAETYLHRIGRSGRFGHLGLAINLITYDDRFNLKSIEEQLGTEIKPIPSNIDKSLYVAEYHSEPVED

EKQ

>KFO81891.1 putative ATP-dependent RNA helicase DDX6 [Cuculus canorus]

MSTARTENPVIMGLSSQNGQLRGPVKPSGGPGGGGTQTQPQMNPLKNANTINNGTQQQAQSMTTAIKPGDDWKKTLKLPP

KDLRIKTSDVTSTKGNEFEDYCLKRELLMGIFEMGWEKPSPIQEESIPIALSGRDILARAKNGTGKSGAYLIPLLERLDL

KKDNIQAMVIVPTRELALQVSQICIQVSKHMGGAKVMATTGGTNLRDDIMRLDDTVHVVIATPGRILDLIKKGVAKVEHV

QMIVLDEANKLLSQDFVQIMEDIILTLPKNRQILLYSATFPLSVQKFMNSHLQKPYEINLMEELTLKGVTQYYAYVTERQ

KVHCLNTLFSRLQINQSIIFCNSSQRVELLAKKISQLGYSCFYIHAKMRQEHRNRVFHDFRNGLCRNLVCTDLFTRGIDI

QAVNVVINFDFPKLAETYLHRIGRSGRFGHLGLAINLITYDDRFNLKSIEEQLGTEIKPIPSNIDKSLYVAEYHSEPVED

EKQ

>NXA77141.1 DDX6 helicase [Thryothorus ludovicianus]

MSTARTENPVIMGLSSQNGQLRGPVKPSGGPGGGGTQTQQQMNQLKNANTINNGTQQQAQSMTTALKPGDDWKKTLKLPP

KDLRIKTSDVTSTKGNEFEDYCLKRELLMGIFEMGWEKPSPIQEESIPIALSGRDILARAKNGTGKSGAYLIPLLERLDL

KKDNIQAMVIVPTRELALQVSQICIQVSKHMGGAKVMATTGGTNLRDDIMRLDDTVHVVIATPGRILDLIKKGVAKVEHV

QMIVLDEANKLLSQDFVQIMEDIILTLPKNRQILLYSATFPLSVQKFMNSHLQKPYEINLMEELTLKGVTQYYAYVTERQ

KVHCLNTLFSRLQINQSIIFCNSSQRVELLAKKISQLGYSCFYIHAKMRQEHRNRVFHDFRNGLCRNLVCTDLFTRGIDI

QAVNVVINFDFPKLAETYLHRIGRSGRFGHLGLAINLITYDDRFNLKSIEEQLGTEIKPIPSNIDKSLYVAEYHSEPVED

EK

>KFR01415.1 putative ATP-dependent RNA helicase DDX6 [Nipponia nippon]

MSTARTENPVIMGLSSQNGQLRGPVKPSAGPGGGGTQTQQQMNQLKNANTINNGTQQQAQSMTTAIKPGDDWKKTLKLPP

KDLRIKTSDVTSTKGNEFEDYCLKRELLMGIFEMGWEKPSPIQEESIPIALSGRDILARAKNGTGKSGAYLIPLLERLDL

KKDNIQAMVIVPTRELALQVSQICIQVSKHMGGAKVMATTGGTNLRDDIMRLDDTVHVVIATPGRILDLIKKGVAKVEHV

QMIVLDEANKLLSQDFVQIMEDIILTLPKNRQILLYSATFPLSVQKFMNSHLQKPYEINLMEELTLKGVTQYYAYVTERQ

KVHCLNTLFSRLQINQSIIFCNSSQRVELLAKKISQLGYSCFYIHAKMRQEHRNRVFHDFRNGLCRNLVCTDLFTRGIDI

QAVNVVINFDFPKLAETYLHRIGRSGRFGHLGLAINLITYDDRFNLKSIEEQLGTEIKPIPSNIDKSLYVAEYHSEPVED

EKQ

>XP_004585195.1 probable ATP-dependent RNA helicase DDX6 [Ochotona princeps]

MSTARTENPVIMGLSSQNGQLRAPVKPSGGPGGGGTQTQQQMNQLKTTNTINNGTQQQAQSLTTTIKPGDDWKKTLKLPP

KDLRIKTSDVTSTKGNEFEDYCLKRELLMGIFEMGWEKPSPIQEESIPIALSGRDILARAKNGTGKSGAYLIPLLERLDL

KKDNIQAMVIVPTRELALQVSQICIQVSKHMGGAKVMATTGGTNLRDDIMRLDDTVHVVIATPGRILDLIKKGVAKVDHV

QMIVLDEADKLLSQDFVQIMEDIILTLPKNRQILLYSATFPLSVQKFMNSHLQKPYEINLMEELTLKGVTQYYAYVTERQ

KVHCLNTLFSRLQINQSIIFCNSSQRVELLAKKISQLGYSCFYIHAKMRQEHRNRVFHDFRNGLCRNLVCTDLFTRGIDI

QAVNVVINFDFPKLAETYLHRIGRSGRFGHLGLAINLITYDDRFNLKSIEEQLGTEIKPIPSNIDKSLYVAEYHSEPVED

EKP

>XP_033925357.1 probable ATP-dependent RNA helicase DDX6 isoform X2 [Melopsittacus undulatus]

MSTARTENPVIMGLSSQNGQLRGPVKPSGGPGGGGTQTQQQMNQLKNANTLNNGTQQQAQIMTSTIKPGDDWKKTLKLPP

KDLRIKTSDVTSTKGNEFEDYCLKRELLMGIFEMGWEKPSPIQEESIPIALSGRDILARAKNGTGKSGAYLIPLLERLDL

KKDNIQAMVIVPTRELALQVSQICIQVSKHMGGAKVMATTGGTNLRDDIMRLDDTVHVVIATPGRILDLIKKGVAKVEHV

QMIVLDEADKLLSQDFVQIMEDIILTLPKNRQILLYSATFPLSVQKFMNSHLQKPYEINLMEELTLKGVTQYYAYVTERQ

KVHCLNTLFSRLQINQSIIFCNSSQRVELLAKKISQLGYSCFYIHAKMRQEHRNRVFHDFRNGLCRNLVCTDLFTRGIDI

QAVNVVINFDFPKLAETYLHRIGRSGRFGHLGLAINLITYDDRFNLKSIEEQLGTEIKPIPSNIDKSLYVAEYHSEPVED

EKQ

>NXO27345.1 DDX6 helicase [Cisticola juncidis]

MSTARTENPVIMGLSSQNGQLRGPVKPSGGPGGGGTQTQQQMNQMKNANTINNGTQQQAQSMTTAIKPGDDWKKTLKLPP

KDLRIKTSDVTSTKGNEFEDYCLKRELLMGIFEMGWEKPSPIQEESIPIALSGRDILARAKNGTGKSGAYLIPLLERLDL

KKDNIQAMVIVPTRELALQVSQICIQVSKHMGGAKVMATTGGTNLRDDIMRLDDTVHVVIATPGRILDLIKKGVAKVEHV

QMIVLDEANKLLSQDFVQIMEDIILTLPKNRQILLYSATFPLSVQKFMNSHLQKPYEINLMEELTLKGVTQYYAYVTERQ

KVHCLNTLFSRLQINQSIIFCNSSQRVELLAKKISQLGYSCFYIHAKMRQEHRNRVFHDFRNGLCRNLVCTDLFTRGIDI

QAVNVVINFDFPKLAETYLHRIGRSGRFGHLGLAINLITYDDRFNLKSIEEQLGTEIKPIPSNIDKSLYVAEYHSEPVED

EKQ

>KFW07901.1 putative ATP-dependent RNA helicase DDX6 [Fulmarus glacialis]

MSTARTENPVIMGLSSQNGQLRGPVKPSGGPGGGGTQTQQQMNQLKNANTINNGTQQQAQSMTTPIKPGDDWKKTLKLPP

KDLRIKTSDVTSTKGNEFEDYCLKRELLMGIFEMGWEKPSPIQEESIPIALSGRDILARAKNGTGKSGAYLIPLLERLDL

KKDNIQAMVIVPTRELALQVSQICIQVSKHMGGAKVMATTGGTNLRDDIMRLDDTVHVVIATPGRILDLIKKGVAKVEHV

QMIVLDEANKLLSQDFVQIMEDIILTLPKNRQILLYSATFPLSVQKFMNSHLQKPYEINLMEELTLKGVTQYYAYVTERQ

KVHCLNTLFSRLQINQSIIFCNSSQRVELLAKKISQLGYSCFYIHAKMRQEHRNRVFHDFRNGLCRNLVCTDLFTRGIDI

QAVNVVINFDFPKLAETYLHRIGRSGRFGHLGLAINLITYDDRFNLKSIEEQLGTEIKPIPSNIDKSLYVAEYHSEPVED

EKQ

>XP_008424655.1 PREDICTED: probable ATP-dependent RNA helicase DDX6 [Poecilia reticulata]

MSTTRTENPVILGLSNQNGQLRSSVKPAGAPGGGGGGPQPPQLNQMIKGTINGNSQPAPPTNAVIKPGDDWKKNLKLPPK

DTRIKTSDVTATKGNEFEDYCLKRELLMGIFEMGWEKPSPIQEESIPIALSGRDILARAKNGTGKSGAYLIPLLERIDLK

RDCLQALVIVPTRELALQVSQICIQVSKHMGGVKVMATTGGTNLRDDIMRLDETVHVVIATPGRILDLIKKGVAKVNQVQ

MVVLDEADKLLSQDFVVMMEEMLGFLPKQRQILLYSATFPLSVQKFMNAHLQKPYEINLMEELTLKGVTQYYAYVTERQK

VHCLNTLFSRLQINQSIIFCNSSQRVELLAKKISQLGYSCFYIHAKMRQEHRNRVFHDFRNGLCRNLVCTDLFTRGIDIQ

AVNVVINFDFPKLGETYLHRIGRSGRFGHLGLAINLITYDDRFNLKAIEEQLGTEIKPIPGIIDKSLYVAEYHSESGEEV

KQ

>XP_022617241.1 probable ATP-dependent RNA helicase DDX6 [Seriola dumerili]

MSTARTENPVILGLSNQNGQLRGSVKPAGAPGGGGGGPQQQQLNQMKGTINNGNSQPAPTTNAVIKPGDDWKKNLKLPPK

DMRMKTSDVTATKGNEFEDYCLKRELLMGIFEMGWEKPSPIQEESIPIALSGRDILARAKNGTGKSGAYLIPLLERIDLK

KDCIQALVIVPTRELALQVSQICIQVSKHMGGVKVMATTGGTNLRDDIMRLDETVHVVIATPGRILDLIKKGVAKVNQVQ

MIVLDEADKLLSQDFVVMMEEILGFLPKQRQILLYSATFPLSVQKFMNSHLQKPYEINLMEELTLKGVTQYYAYVTERQK

VHCLNTLFSRLQINQSIIFCNSSQRVELLAKKISQLGYSCFYIHAKMRQEHRNRVFHDFRNGLCRNLVCTDLFTRGIDIQ

AVNVVINFDFPKLGETYLHRIGRSGRFGHLGLAINLITYDDRFNLKGIEEQLGTEIKPIPGIIDKSLYVAEYHSESGEEV

KP

>XP_023797079.1 probable ATP-dependent RNA helicase DDX6 [Cyanistes caeruleus]

MSTARTENPVIMGLSSQNGQLRGPVKPSGGPGGGGTQTQQQMNQLKNASTINNGTQQQAQSMTTAIKPGDDWKKTLKLPP

KDLRIKTSDVTSTKGNEFEDYCLKRELLMGIFEMGWEKPSPIQEESIPIALSGRDILARAKNGTGKSGAYLIPLLERLDL

KKDNIQAMVIVPTRELALQVSQICIQVSKHMGGAKVMATTGGTNLRDDIMRLDDTVHVVIATPGRILDLIKKGVAKVEHV

QMIVLDEADKLLSQDFVQIMEDIILTLPKNRQILLYSATFPLSVQKFMNSHLQKPYEINLMEELTLKGVTQYYAYVTERQ

KVHCLNTLFSRLQINQSIIFCNSSQRVELLAKKISQLGYSCFYIHAKMRQEHRNRVFHDFRNGLCRNLVCTDLFTRGIDI

QAVNVVINFDFPKLAETYLHRIGRSGRFGHLGLAINLITYDDRFNLKSIEEQLGTEIKPIPSNIDKSLYVAEYHSEPVED

EKQ

>XP_004636497.1 probable ATP-dependent RNA helicase DDX6 [Octodon degus]

MSTARTENPVIMGLSSQNGQLRGPVKPSGGPGGGGTQTQQQMNQLKNTNTINNGTQQQAQSMTTTIKPGDDWKKTLKLPP

KDLRIKTSDVTSTKGNEFEDYCLKRELLMGIFEMGWEKPSPIQEESIPIALSGRDILARAKNGTGKSGAYLIPLLERLDL

KKDNIQAMVIVPTRELALQVSQICIQVSKHMGGAKVMATTGGTNLRDDIMRLDDTVHVIIATPGRILDLIKKGVAKVDHV

QMIVLDEADKLLSQDFVQIMEDIILTLPKNRQILLYSATFPLSVQKFMNSHLQKPYEINLMEELTLKGVTQYYAYVTERQ

KVHCLNTLFSRLQINQSIIFCNSSQRVELLAKKISQLGYSCFYIHAKMRQEHRNRVFHDFRNGLCRNLVCTDLFTRGIDI

QAVNVVINFDFPKLAETYLHRIGRSGRFGHLGLAINLITYDDRFNLKSIEEQLGTEIKPIPSNIDKSLYVAEYHSEPVED

EKP

>KAF7235394.1 putative ATP-dependent RNA helicase DDX6 [Varanus komodoensis]

MSTARTENPVIMGLSSQNGQLRGPVKPTGGPGGGGTQAQQQINQLKHPNTINNGTQQQAQSMASTIKPGDDWKKTLKLPP

KDLRIKTSDVTSTKGNEFEDYCLKRELLMGIFEMGWEKPSPIQEESIPIALSGRDILARAKNGTGKSGAYLIPLLERLDL

KKDNIQAMVIVPTRELALQVSQICIQVSKHMGGAKVMATTGGTNLRDDIMRLDDTVHVVIATPGRILDLIKKGVAKVDHV

QMIVLDEADKLLSQDFVQIMEDIILTLPKNRQILLYSATFPLSVQKFMNSHLQKPYEINLMEELTLKGVTQYYAYVTERQ

KVHCLNTLFSRLQINQSIIFCNSSQRVELLAKKISQLGYSCFYIHAKMRQEHRNRVFHDFRNGLCRNLVCTDLFTRGIDI

QAVNVVINFDFPKLAETYLHRIGRSGRFGHLGLAINLITYDDRFNLKSIEEQLGTEIKPIPSNIDKSLYVAEYHSEPVDD

EKP

>KAF3693260.1 putative ATP-dependent RNA helicase DDX6 [Channa argus]

MSTARTENPVILGLSNQNGQLRSSVKPAGAPGGGGGGTPPQQQQQQQQLNQMKGTINNGNSQPAPTTNAVIKPGDDWKKN

LKLPPKDMRMKTSDVTATKGNEFEDYCLKRELLMGIFEMGWEKPSPIQEESIPIALSGRDILARAKNGTGKSGAYLIPLL

ERIDLKKDCIQAMVIVPTRELALQVSQICIQVSKHMGGVKVMATTGGTNLRDDIMRLDETVHVVIATPGRILDLIKKGVA

KVNQVQMIVLDEADKLLSQDFVVMMEEILGFLPKQRQILLYSATFPLSVQKFMNSHLQKPYEINLMEELTLKGVTQYYAY

VTERQKVHCLNTLFSRLQINQSIIFCNSSQRVELLAKKISQLGYSCFYIHAKMRQEHRNRVFHDFRNGLCRNLVCTDLFT

RGIDIQAVNVVINFDFPKLGETYLHRIGRSGRFGHLGLAINLITYDDRFNLKGIEEQLGTEIKPIPGIIDKSLYVAEYHS

ESGEEVKP

>XP_028563388.1 probable ATP-dependent RNA helicase DDX6 [Podarcis muralis]

MSTARTENPVIMGLSSQNGQLRGPVKPTGGPGGGGTQAQQQMNQLKHPNTINNGTQQQAQSMASTIKPGDDWKKTLKLPP

KDLRIKTSDVTSTKGNEFEDYCLKRELLMGIFEMGWEKPSPIQEESIPIALSGRDILARAKNGTGKSGAYLIPLLERLDL

KKDNIQAMVIVPTRELALQVSQICIQVSKHMGGAKVMATTGGTNLRDDIMRLDDTVHVVIATPGRILDLIKKGVAKVDHV

QMIVLDEADKLLSQDFVQIMEDIILTLPKNRQILLYSATFPLSVQKFMNSHLQKPYEINLMEELTLKGVTQYYAYVTERQ

KVHCLNTLFSRLQINQSIIFCNSSQRVELLAKKISQLGYSCFYIHAKMRQEHRNRVFHDFRNGLCRNLVCTDLFTRGIDI

QAVNVVINFDFPKLAETYLHRIGRSGRFGHLGLAINLITYDDRFNLKSIEEQLGTEIKPIPSNIDKSLYVAEYHSEPVED

EKP

>XP_022058462.1 probable ATP-dependent RNA helicase DDX6 [Acanthochromis polyacanthus]

MSTARTENPVILGLSNQNGQIRGSVKPAGAPGGGGGGPQQQQFNQMKGTINNGNSQPAPTTNAVIKPGDDWKKNLKLPPK

DMRMKTSDVTATKGNEFEDYCLKRELLMGIFEMGWEKPSPIQEESIPIALSGRDILARAKNGTGKSGAYLIPLLERIDLK

KDCIQALVIVPTRELALQVSQICIQVSKHMGGVKVMATTGGTNLRDDIMRLDETVHVVIATPGRILDLIKKGVAKVNQVQ

MIVLDEADKLLSQDFVVMMEEILGFLPKQRQILLYSATFPLSVQKFMNAHLQKPYEINLMEELTLKGVTQYYAYVTERQK

VHCLNTLFSRLQINQSIIFCNSSQRVELLAKKISQLGYSCFYIHAKMRQEHRNRVFHDFRNGLCRNLVCTDLFTRGIDIQ

AVNVVINFDFPKLGETYLHRIGRSGRFGHLGLAINLITYDDRFNLKGIEEQLGTEIKPIPGIIDKSLYVAEYHSESGEEV

KP

>XP_009321665.1 PREDICTED: probable ATP-dependent RNA helicase DDX6 [Pygoscelis adeliae]

MSTARTENPVIMGLSSQNGQLRGPVKPSGGPGGGGTQTQQQMNQLKNANTINNGTQQQAQSMTTAIKPGDDWKKTLKLPP

KDLRIKTSDVTSTKGNEFEDYCLKRELLMGIFEMGWEKPSPIQEESIPIALSGRDILARAKNGTGKSGAYLIPLLERLDL

KKDNIQAMVIVPTRELALQVSQICIQVSKHMGGAKVMATTGGTNLRDDIMRLDDTVHVVIATPGRILDLIKKGVAKVEHV

QMIVLDEADKLLSQDFVQIMEDIILTLPKNRQILLYSATFPLSVQKFMNSHLQKPYEINLMEELTLKGVTQYYAYVTERQ

KVHCLNTLFSRLQINQSIIFCNSSQRVELLAKKISQLGYSCFYIHAKMRQEHRNRVFHDFRNGLCRNLVCTDLFTRGIDI

QAVNVVINFDFPKLAETYLHRIGRSARFGHLGLAINLITYDDRFNLKSIEEQLGTEIKPIPSNIDKSLYVAEYHSEPVED

EKQ

>BAA04482.1 RCK [Homo sapiens]

MGLSSQNGQLRGPVKPTGGPGGGGTQTQQQMNQLKNTNTINNGTQQQAQSMTTTIKPGDDWKKTLKLPPKDLRIKTSDVT

STKGNEFEDYCLKRELLMGIFEMGWEKPSPIQEESIPIALSGRDILARAKNGTGKSGAYLIPLLERLDLKKDNIQAMVIV

PTRELALQVSQICIQVSKHMGGAKVMATTGGTNLRDDIMRLDDTVHVVIATPGRILDLIKKGVAKVDHVQMIVLDEADKL

LSQDFVQIMEDIILTLPKNRQILLYSATFPLSVQKFMNSHLQKPYEINLMEELTLKGVTQYYAYVTERQKVHCLNTLFSR

LQINQSIIFCNSSQRVELLAKKISQLGYSCFYIHAKMRQEHRNRVFHDFRNGLCRNLVCTDLFTRGIDIQAVNVVINFDF

PKLAETYLHRIGRSGRFGHLGLAINLITYDDRFNLKSIEEQLGTEIKPIPSNIDKSLYVAEYHSEPVEDEKP

>XP_026536781.1 probable ATP-dependent RNA helicase DDX6 [Notechis scutatus]

MSTARTENPVIMGLSSQNGQLRGPVKPGGGPGSGGTQAQQQINQLKHPNTINNGTQQQAQSMASTIKPGDDWKKTLKLPP

KDLRIKTSDVTSTKGNEFEDYCLKRELLMGIFEMGWEKPSPIQEESIPIALSGRDILARAKNGTGKSGAYLIPLLERLDL

KKDNIQAMVIVPTRELALQVSQICIQVSKHMGGAKVMATTGGTNLRDDIMRLDDTVHVVIATPGRILDLIKKGVAKVDHI

QMIVLDEADKLLSQDFVQIMEDIILTLPKNRQILLYSATFPLSVQKFMNSHLQKPYEINLMEELTLKGVTQYYAYVTERQ

KVHCLNTLFSRLQINQSIIFCNSSQRVELLAKKISQLGYSCFYIHAKMRQEHRNRVFHDFRNGLCRNLVCTDLFTRGIDI

QAVNVVINFDFPKLAETYLHRIGRSGRFGHLGLAINLITYDDRFNLKSIEEQLGTEIKPIPSNIDKSLYVAEYHSETVED

DKP

>NWQ79586.1 DDX6 helicase [Columbina picui]

MSTARTENPVIMGLSSQNGQLRGPVKPSGGPGGGGTQTQQQMNQLKNANTINNGTQQQAQSMTTALKPGDDWKKTLKLPP

KDLRIKTSDVTSTKGNEFEDYCLKRELLMGIFEMGWEKPSPIQEESIPIALSGRDILARAKNGTGKSGAYLIPLLERLDL

KKDNIQAMVIVPTRELALQVSQICIQVSKHMGGAKVMATTGGTNLRDDIMRLDDTVHVVIATPGRILDLIKKGVAKVEHV

QMIVLDEANKLLSQDFVQIMEDIILTLPKNRQILLYSATFPLSVQKFMNSHLQKPYEINLMEELTLKGVTQYYAYVTERQ

KVHCLNTLFSRLQINQSIIFCNSSQRVELLAKKISQLGYSCFYIHAKMRQEHRNRVFHDFRNGLCRNLVCTDLFTRGIDI

QAVNVVINFDFPKLAETYLHRIGRSGRFGHLGLAINLITYDDRFNLKSIEEQLGTEIKPIPSNIDKSLYVAEYHSEPVED

EKQ

>NXH14681.1 DDX6 helicase [Bucco capensis]

MSTARTENPVIMGLSSQNGQLRGPVKPSGGPGGGGTQTQQQMNQLKNANTINNGTQQQAQSMTTAIKPGDDWKKTLKLPP

KDLRIKTSDVTSTKGNEFEDYCLKRELLMGIFEMGWEKPSPIQEESIPIALSGRDILARAKNGTGKSGAYLIPLLERLDL

KRDNIQAMVIVPTRELALQVSQICIQVSKHMGGAKVMATTGGTNLRDDIMRLDDTVHVVIATPGRILDLIKKGVAKVEHV

QMIVLDEANKLLSQDFVQIMEDIILTLPKNRQILLYSATFPLSVQKFMNSHLQKPYEINLMEELTLKGVTQYYAYVTERQ

KVHCLNTLFSRLQINQSIIFCNSSQRVELLAKKISQLGYSCFYIHAKMRQEHRNRVFHDFRNGLCRNLVCTDLFTRGIDI

QAVNVVINFDFPKLAETYLHRIGRSGRFGHLGLAINLITYDDRFNLKSIEEQLGTEIKPIPSNIDKSLYVAEYHSEPVED

EKQ

>NWI55129.1 DDX6 helicase [Calyptomena viridis]

MSTARTENPVIMGLSSQNGQLRGPVKPSGGPGGGGTQTQQQMNQLKNANTINNGTQQQAQSLSTAIKPGDDWKKTLKLPP

KDLRIKTSDVTSTKGNEFEDYCLKRELLMGIFEMGWEKPSPIQEESIPIALSGRDILARAKNGTGKSGAYLIPLLERLDL

KKDNIQAMVIVPTRELALQVSQICIQVSKHMGGAKVMATTGGTNLRDDIMRLDDTVHVVIATPGRILDLIKKGVAKVEHV

QMIVLDEANKLLSQDFVQIMEDIILTLPKNRQILLYSATFPLSVQKFMNSHLQKPYEINLMEELTLKGVTQYYAYVTERQ

KVHCLNTLFSRLQINQSIIFCNSSQRVELLAKKISQLGYSCFYIHAKMRQEHRNRVFHDFRNGLCRNLVCTDLFTRGIDI

QAVNVVINFDFPKLAETYLHRIGRSGRFGHLGLAINLITYDDRFNLKSIEEQLGTEIKPIPSNIDKSLYVAEYHSEPVED

EKQ

>XP_026227438.1 probable ATP-dependent RNA helicase ddx6 [Anabas testudineus]

MSTARTENPVILGLSNQNGQLRGSVKPAGAPGGGGGGPQQQLNQMKGTINNGNSQPAPTTNAVIKPGDDWKKNLKLPPKD

MRMKTSDVTATKGNEFEDYCLKRELLMGIFEMGWEKPSPIQEESIPIALSGRDILARAKNGTGKSGAYLIPLLERIDLKK

DCIQALVIVPTRELALQVSQICIQVSKHMGGVKVMATTGGTNLRDDIMRLDETVHVVIATPGRILDLIKKGVAKVNQVQM

IVLDEADKLLSQDFVVMMEEILGFLPKQRQILLYSATFPLSVQKFMNAHLQKPYEINLMEELTLKGVTQYYAYVTERQKV

HCLNTLFSRLQINQSIIFCNSSQRVELLAKKISQLGYSCFYIHAKMRQEHRNRVFHDFRNGLCRNLVCTDLFTRGIDIQA

VNVVINFDFPKLGETYLHRIGRSGRFGHLGLAINLITYDDRFNLKGIEEQLGTEIKPIPGIIDKSLYVAEYHSESGEEVK

P

>XP_003229776.2 PREDICTED: probable ATP-dependent RNA helicase DDX6 isoform X1 [Anolis carolinensis]

MSTARTENPVIMGLASQNGQLRGPVKPSGGPGGGGGTQGQPQINPLKHPNAINNGTQQQAQSMASTIKPGDDWKKTLKLP

PKDLRIKTSDVTSTKGNEFEDYCLKRELLMGIFEMGWEKPSPIQEESIPIALSGRDILARAKNGTGKSGAYLIPLLERLD

LKKDYIQAMVIVPTRELALQVSQICIQVSKHMGGAKVMATTGGTNLRDDIMRLDDTVHVVIATPGRILDLIKKGVAKVDH

IQMIVLDEADKLLSQDFVQIMEDIILTLPKNRQILLYSATFPLSVQKFMNSHLQKPYEINLMEELTLKGVTQYYAYVTER

QKVHCLNTLFSRLQINQSIIFCNSSQRVELLAKKISQLGYSCFYIHAKMRQEHRNRVFHDFRNGLCRNLVCTDLFTRGID

IQAVNVVINFDFPKLAETYLHRIGRSGRFGHLGLAINLITYDDRFNLKSIEEQLGTEIKPIPSNIDKSLYVAEYHSEPGE

DGKALRLNTQAKRAL

>NWR49346.1 DDX6 helicase [Regulus satrapa]

MSTARTENPVIMGLSSQNGQLRGPVKPSGGPGGGGTATQQQMNQLKNASTINNGTQQQAQSMTAAIKPGDDWKKTLKLPP

KDLRIKTSDVTSTKGNEFEDYCLKRELLMGIFEMGWEKPSPIQEESIPIALSGRDILARAKNGTGKSGAYLIPLLERLDL

KKDNIQAMVIVPTRELALQVSQICIQVSKHMGGAKVMATTGGTNLRDDIMRLDDTVHVVIATPGRILDLIKKGVAKVEHV

QMIVLDEANKLLSQDFVQIMEDIILTLPKNRQILLYSATFPLSVQKFMNSHLQKPYEINLMEELTLKGVTQYYAYVTERQ

KVHCLNTLFSRLQINQSIIFCNSSQRVELLAKKISQLGYSCFYIHAKMRQEHRNRVFHDFRNGLCRNLVCTDLFTRGIDI

QAVNVVINFDFPKLAETYLHRIGRSGRFGHLGLAINLITYDDRFNLKSIEEQLGTEIKPIPSNIDKSLYVAEYHSEPVED

EKQ

>XP_008287986.1 PREDICTED: probable ATP-dependent RNA helicase DDX6 [Stegastes partitus]

MSTARTENPVILGLSNQNGQLRGSVKPAGAPGGGGGGPQQQQFNQMKGTINNGNSQPAPTTNAVIKPGDDWKKNLKLPPK

DMRMKTSDVTATKGNEFEDYCLKRELLMGIFEMGWEKPSPIQEESIPIALSGRDILARAKNGTGKSGAYLIPLLERIDLK

KDCIQALVIVPTRELALQVSQICIQVSKHMGGVKVMATTGGTNLRDDIMRLDETVHVVIATPGRILDLIKKGVAKVNQVQ

MIVLDEADKLLSQDFVVMMEEILGFLPKQRQILLYSATFPLSVQKFMNAHLQKPYEINLMEELTLKGVTQYYAYVTERQK

VHCLNTLFSRLQINQSIIFCNSSQRVELLAKKISQLGYSCFYIHAKMRQEHRNRVFHDFRNGLCRNLVCTDLFTRGIDIQ

AVNVVINFDFPKLGETYLHRIGRSGRFGHLGLAINLITYDDRFNLKGIEEQLGTEIKPIPGIIDKSLYVAEYHSESGEEV

KP

>XP_034281070.1 probable ATP-dependent RNA helicase DDX6 [Pantherophis guttatus]

MSTARTENPVIMGLSSQNGQLRGPVKPGGGPGSGGTQVQQQINQLKHPNTINNGTQQQAQSMASTIKPGDDWKKTLKLPP

KDLRIKTSDVTSTKGNEFEDYCLKRELLMGIFEMGWEKPSPIQEESIPIALSGRDILARAKNGTGKSGAYLIPLLERLDL

KKDNIQAMVIVPTRELALQVSQICIQVSKHMGGAKVMATTGGTNLRDDIMRLDDTVHVVIATPGRILDLIKKGVAKVDHI

QMIVLDEADKLLSQDFVQIMEDIILTLPKNRQILLYSATFPLSVQKFMNSHLQKPYEINLMEELTLKGVTQYYAYVTERQ

KVHCLNTLFSRLQINQSIIFCNSSQRVELLAKKISQLGYSCFYIHAKMRQEHRNRVFHDFRNGLCRNLVCTDLFTRGIDI

QAVNVVINFDFPKLAETYLHRIGRSGRFGHLGLAINLITYDDRFNLKSIEEQLGTEIKPIPSNIDKSLYVAEYHSEPVED

DKP

>NXK78609.1 DDX6 helicase [Amazona guildingii]

MSTARTENPVIMGLSSQNGQLRGPVKPSGGPGGGGTQTQQQMNQLKNANTINNGTQQQAQIMTTTIKPGDDWKKTLKLPP

KDLRIKTSDVTSTKGNEFEDYCLKRELLMGIFEMGWEKPSPIQEESIPIALSGRDILARAKNGTGKSGAYLIPLLERLDL

KKDNIQAMVIVPTRELALQVSQICIQVSKHMGGAKVMATTGGTNLRDDIMRLDDTVHVVIATPGRILDLIKKGVAKVEHV

QMIVLDEANKLLSQDFVQIMEDIILTLPKNRQILLYSATFPLSVQKFMNSHLQKPYEINLMEELTLKGVTQYYAYVTERQ

KVHCLNTLFSRLQINQSIIFCNSSQRVELLAKKISQLGYSCFYIHAKMRQEHRNRVFHDFRNGLCRNLVCTDLFTRGIDI

QAVNVVINFDFPKLAETYLHRIGRSGRFGHLGLAINLITYDDRFNLKSIEEQLGTEIKPIPSNIDKSLYVAEYHSEPVED

EKQ

>XP_030899158.1 probable ATP-dependent RNA helicase DDX6 isoform X1 [Melopsittacus undulatus]

MSTARTENPVIMGLSSQNGQLRGPVKPSGGPGGGGTQTQQQMNQLKNANTLNNGTQQQAQIMTSTIKPGDDWKKTLKLPP

KDLRIKTSDVTSTKGNEFEDYCLKRELLMGIFEMGWEKPSPIQEESIPIALSGRDILARAKNGTGKSGAYLIPLLERLDL

KKDNIQAMVIVPTRELALQVSQICIQVSKHMGGAKVMATTGGTNLRDDIMRLDDTVHVVIATPGRILDLIKKGVAKVEHV

QMIVLDEADKLLSQDFVQIMEDIILTLPKNRQILLYSATFPLSVQKFMNSHLQKPYEINLMEELTLKGVTQYYAYVTERQ

KVHCLNTLFSRLQINQSIIFCNSSQRVELLAKKISQLGYSCFYIHAKMRQEHRNRVFHDFRNGLCRNLVCTDLFTRGIDI

QAVNVVINFDFPKLAETYLHRIGRSGRFGHLGLAINLITYDDRFNLKSIEEQLGTEIKPIPSNIDKSLYVAEYHSEPVED

EKQL

>XP_026769948.1 probable ATP-dependent RNA helicase ddx6 [Pangasianodon hypophthalmus]

MSTARTENPVILGLTNQNGQKRGSAKPTGGPGGGGGGGGPQTTQPAQIKASSTVNNGNSLPVPTANTVIKPGDDWKKNLK

LPPKDMRMKTSDVTATKGNEFEDYCLKRELLMGIFEMGWEKPSPIQEESIPIALSGRDILARAKNGTGKSGAYLIPLLER

IDLKKDCIQALVIVPTRELALQVSQICIQVSKHMGGVKVMATTGGTNLRDDIMRLDETVHVVIATPGRVLDLIKKGVAKV

GQVQMIVLDEADKLLSQDFVQMMEEILSFLPKQRQILLYSATFPLSVQKFMNAHLQKPYEINLMEELTLKGVTQYYAYVT

ERQKVHCLNTLFSRLQINQSIIFCNSSQRVELLAKKISQLGYSCFYIHAKMRQEHRNRVFHDFRNGLCRNLVCTDLFTRG

IDIQAVNVVINFDFPKLGETYLHRIGRSGRFGHLGLAINLITYDDRFNLKGIEEQLGTEIKPIPSSIDKSLYVAEYHSES

AEEVKL

>XP_008576061.1 PREDICTED: probable ATP-dependent RNA helicase DDX6 [Galeopterus variegatus]

MSTARTENPVIMGLSSQNGQLRGPVKPSGGPGGGGTQTQQQMNQLKNTNTMNNGTQQQAQSMTTTIKPGDDWKKTLKLPP

KDLRIKTSDVTSTKGNEFEDYCLKRELLMGIFEMGWEKPSPIQEESIPIALSGRDILARAKNGTGKSGAYLIPLLERLDL

KKDNIQAMVIVPTRELALQVSQICIQVSKHMGGAKVMATTGGTNLRDDIMRLDDTVHVVIATPGRILDLIKKGVAKVDHV

QMIVLDEADKLLSQDFVQIMEDIILTLPKNRQILLYSATFPLSVQKFMNSHLQKPYEINLMEELTLKGVTQYYAYVTERQ

KVHCLNTLFSRLQINQSIIFCNSSQRVELLAKKISQLGYSCFYIHAKMRQEHRNRVFHDFRNGLCRNLVCTDLFTRGIDI

QAVNVVINFDFPKLAETYLHRIGRSGRFGHLGLAINLITYDDRFNLKSIEEQLGTEIKPIPSNIDKSLYVAEYHSEPVED

EKP

>XP_003418301.1 probable ATP-dependent RNA helicase DDX6 [Loxodonta africana]

MSTARTENPVIMGLSSQNGQLRGPVKPSGGPGGGGTQTQQQMNQLKNTNTVNNGTQQQAQSLTTTIKPGDDWKKTLKLPP

KDLRIKTSDVTSTKGNEFEDYCLKRELLMGIFEMGWEKPSPIQEESIPIALSGRDILARAKNGTGKSGAYLIPLLERLDL

KKDNIQAMVIVPTRELALQVSQICIQVSKHMGGAKVMATTGGTNLRDDIMRLDDTVHVVIATPGRILDLIKKGVAKVDHV

QMIVLDEADKLLSQDFVQIMEDIILTLPKNRQILLYSATFPLSVQKFMNSHLQKPYEINLMEELTLKGVTQYYAYVTERQ

KVHCLNTLFSRLQINQSIIFCNSSQRVELLAKKISQLGYSCFYIHAKMRQEHRNRVFHDFRNGLCRNLVCTDLFTRGIDI

QAVNVVINFDFPKLAETYLHRIGRSGRFGHLGLAINLITYDDRFNLKSIEEQLGTEIKPIPSNIDKSLYVAEYHSEPAED

EKA

>XP_007259595.2 probable ATP-dependent RNA helicase DDX6 [Astyanax mexicanus]

MSTARTENPVILGLSNQNGQMRGSVKPTGGPGGGGGGPQTTQPTQIKASSTINNGNSQPVPTVNTVIKPGDDWKKNLKLP

PKDMRMKTSDVTATKGNEFEDYCLKRELLMGIFEMGWEKPSPIQEESIPIALSGRDILARAKNGTGKSGAYLIPLLERID

LKKDCIQALVIVPTRELALQVSQICIQVSKHMGGVKVMATTGGTNLRDDIMRLDETVHVVIATPGRILDLIKKGVAKVGQ

VQMIVLDEADKLLSQDFVQMMEEILSFLAKQRQILLYSATFPLSVQKFMNSHLQKPYEINLMEELTLKGVTQYYAYVTER

QKVHCLNTLFSRLQINQSIIFCNSSQRVELLAKKISQLGYSCFYIHAKMRQEHRNRVFHDFRNGLCRNLVCTDLFTRGID

IQAVNVVINFDFPKLGETYLHRIGRSGRFGHLGLAINLITYDDRFNLKGIEEQLGTEIKPIPSSIDKSLYVAEYHSESSE

EVKL

>XP_009962053.2 probable ATP-dependent RNA helicase DDX6 [Tyto alba alba]

MSTARTENPVIMGLSSQNGQLRGPVKPSGGPGGGGTQTQQQMNQLKNANTINNGTQQQAQSMTTAIKPGDDWKKTLKLPP

KDLRIKTSDVTSTKGNEFEDYCLKRELLMGIFEMGWEKPSPIQEESIPIALSGRDILARAKNGTGKSGAYLIPLLERLDL

KKDNIQAMVIVPTRELALQVSQICIQVSKHMGGAKVMATTGGTNLRDDIMRLDDTVHGVIATPGRILDLIKKGVAKVEHV

QMIVLDEADKLLSQDFVQIMEDIILTLPKNRQILLYSATFPLSVQKFMNSHLQKPYEINLMEELTLKGVTQYYAYVTERQ

KVHCLNTLFSRLQINQSIIFCNSSQRVELLAKKISQLGYSCFYIHAKMRQEHRNRVFHDFRNGLCRNLVCTDLFTRGIDI

QAVNVVINFDFPKLAETYLHRIGRSGRFGHLGLAINLITYDDRFNLKSIEEQLGTEIKPIPSNIDKSLYVAEYHSEPVED

EKQ

>XP_023139343.1 probable ATP-dependent RNA helicase ddx6 [Amphiprion ocellaris]

MSTARTENPVILGLSNQNGQPRGSVKPAGAPGGGGGGPQQQQFNQMKGTINNGNSQPAPTTNAVIKPGDDWKKNLKLPPK

DMRMKTSDVTATKGNEFEDYCLKRELLMGIFEMGWEKPSPIQEESIPIALSGRDILARAKNGTGKSGAYLIPLLERIDLK

KDCIQALVIVPTRELALQVSQICIQVSKHMGGVKVMATTGGTNLRDDIMRLDETVHVVIATPGRILDLIKKGVAKVNQVQ

MIVLDEADKLLSQDFVVMMEEILGFLPKQRQILLYSATFPLSVQKFMNAHLQKPYEINLMEELTLKGVTQYYAYVTERQK

VHCLNTLFSRLQINQSIIFCNSSQRVELLAKKISQLGYSCFYIHAKMRQEHRNRVFHDFRNGLCRNLVCTDLFTRGIDIQ

AVNVVINFDFPKLGETYLHRIGRSGRFGHLGLAINLITYDDRFNLKGIEEQLGTEIKPIPGIIDKSLYVAEYHSESGEEV

KP

>KFQ45799.1 putative ATP-dependent RNA helicase DDX6 [Nestor notabilis]

MSTARTENPVIMGLSSQNGQLRGPVKPSGGPGGGGTQTQQQMNQLKNTNTINNGTQQQAQIMTTAIKPGDDWKKTLKLPP

KDLRIKTSDVTSTKGNEFEDYCLKRELLMGIFEMGWEKPSPIQEESIPIALSGRDILARAKNGTGKSGAYLIPLLERLDL

KKDNIQAMVIVPTRELALQVSQICIQVSKHMGGAKVMATTGGTNLRDDIMRLDDTVHVVIATPGRILDLIKKGVAKVEHV

QMIVLDEANKLLSQDFVQIMEDIILTLPKNRQILLYSATFPLSVQKFMNSHLQKPYEINLMEELTLKGVTQYYAYVTERQ

KVHCLNTLFSRLQINQSIIFCNSSQRVELLAKKISQLGYSCFYIHAKMRQEHRNRVFHDFRNGLCRNLVCTDLFTRGIDI

QAVNVVINFDFPKLAETYLHRIGRSGRFGHLGLAINLITYDDRFNLKSIEEQLGTEIKPIPSNIDKSLYVAEYHSEPVED

EKQ

>XP_028930707.1 probable ATP-dependent RNA helicase DDX6 isoform X2 [Ornithorhynchus anatinus]

MSTARTENPVIMGLSSQNGQLRGPVKPSGGPGGGGTPTQQQMNQLKNTNTINNGTQQQAQSLTTTIKPGDDWKKTLKLPP

KDLRIKTSDVTSTKGNEFEDYCLKRELLMGIFEMGWEKPSPIQEESIPIALSGRDILARAKNGTGKSGAYLIPLLERLDL

KKDNIQAMVIVPTRELALQVSQICIQVSKHMGGAKVMATTGGTNLRDDIMRLDDTVHVVIATPGRILDLIKKGVAKVDHV

QMIVLDEADKLLSQDFVQIMEDIILTLPKNRQILLYSATFPLSVQKFMNSHLQKPYEINLMEELTLKGVTQYYAYVTERQ

KVHCLNTLFSRLQINQSIIFCNSSQRVELLAKKISQLGYSCFYIHAKMRQEHRNRVFHDFRNGLCRNLVCTDLFTRGIDI

QAVNVVINFDFPKLAETYLHRIGRSGRFGHLGLAINLITYDDRFNLKSIEEQLGTEIKPIPSNIDKSLYVAEYHSEPVED

EKP

>XP_026173747.1 probable ATP-dependent RNA helicase ddx6 [Mastacembelus armatus]

MSTARTENPVILGLSSQNGQLRGSVKPAGAPGGGGGGPQQQQQQQQQQQLNQIKGTINNGSSQLAPATNAVIKPGDDWKK

NLKLPPKDMRMKTSDVTATKGNEFEDYCLKRELLMGIFEMGWEKPSPIQEESIPIALSGRDILARAKNGTGKSGAYLIPL

LERIDLKKDCIQALVIVPTRELALQVSQICIQVSKHMGGVKVMATTGGTNLRDDIMRLDETVHVVIATPGRILDLIKKGV

AKVNQVQMIVLDEADKLLSQDFVVMMEEILGFLSKQRQILLYSATFPLSVQKFMNAHLQKPYEINLMEELTLKGVTQYYA

YVTERQKVHCLNTLFSRLQINQSIIFCNSSQRVELLAKKISQLGYSCFYIHAKMRQEHRNRVFHDFRNGLCRNLVCTDLF

TRGIDIQAVNVVINFDFPKLGETYLHRIGRSGRFGHLGLAINLITYDDRFNLKGIEEQLGTEIKPIPGIIDKSLYVAEYH

SESGEEVKP

>XP_015742882.1 probable ATP-dependent RNA helicase DDX6 [Python bivittatus]

MSTARTENPVIMGLSSQNGQLRGPVKPGGGPGSGGTQAQQQINQLKHPNTINNGTQQQAQSMASTIKPGDDWKKTLKLPP

KDLRIKTSDVTSTKGNEFEDYCLKRELLMGIFEMGWEKPSPIQEESIPIALSGRDILARAKNGTGKSGAYLIPLLERLDL

KKDNIQAMVIVPTRELALQVSQICIQVSKHMGGAKVMATTGGTNLRDDIMRLDDTVHVVIATPGRILDLIKKGVAKVDHI

QMIVLDEADKLLSQDFVQIMEDIILTLPKNRQILLYSATFPLSVQKFMNSHLQKPYEINLMEELTLKGVTQYYAYVTERQ

KVHCLNTLFSRLQINQSIIFCNSSQRVELLAKKISQLGYSCFYIHAKMRQEHRNRVFHDFRNGLCRNLVCTDLFTRGIDI

QAVNVVINFDFPKLAETYLHRIGRSGRFGHLGLAINLITYDDRFNLKSIEEQLGTEIKPIPSNIDKSLYVAEYHSEPVED

DKP

>XP_015806763.1 PREDICTED: probable ATP-dependent RNA helicase DDX6 [Nothobranchius furzeri]

MSSNRTENPVILGLSNQNGQLRGSVKPAGAPGGGGGGPQQQQLNQMKGTINGNSQPAPPTNAIIKPGDDWKKNLKLPPKD

TRIKTSDVTATKGNEFEDYCLKRELLMGIFEMGWEKPSPIQEESIPIALSGRDILARAKNGTGKSGAYLIPLLERIDLKR

DCLQALVIVPTRELALQVSQICIQVSKHMGGVKVMATTGGTNLRDDIMRLDETVHVVIATPGRILDLIKKGVAKVGQVQM

IVLDEADKLLSQDFVVMMEEMLGFLPRQRQILLYSATFPLSVQKFMNAHLQKPYEINLMEELTLKGVTQYYAYVTERQKV

HCLNTLFSRLQINQSIIFCNSSQRVELLAKKISQLGYSCFYIHAKMRQEHRNRVFHDFRNGLCRNLVCTDLFTRGIDIQA

VNVVINFDFPKLGETYLHRIGRSGRFGHLGLAINLITYDDRFNLKGIEEQLGTEIKPIPGIIDKSLYVAEYHSEGGEEVK

Q

>XP_013911318.1 PREDICTED: probable ATP-dependent RNA helicase DDX6 [Thamnophis sirtalis]

MSTARTENPVIMGLSSQNGQLRGPVKPGGGPGSGGTQAQQQINQLKHPNTINNGTQQQAQSMASTIKPGDDWKKTLKLPP

KDLRIKTSDVTSTKGNEFEDYCLKRELLMGIFEMGWEKPSPIQEESIPIALSGRDILARAKNGTGKSGAYLIPLLERLDL

KKDKIQAMVIVPTRELALQVSQICIQVSKHMGGAKVMATTGGTNLRDDIMRLDDTVHVVIATPGRILDLIKKGVAKVDHI

QMIVLDEADKLLSQDFVQIMEDIILTLPKNRQILLYSATFPLSVQKFMNSHLQKPYEINLMEELTLKGVTQYYAYVTERQ

KVHCLNTLFSRLQINQSIIFCNSSQRVELLAKKISQLGYSCFYIHAKMRQEHRNRVFHDFRNGLCRNLVCTDLFTRGIDI

QAVNVVINFDFPKLAETYLHRIGRSGRFGHLGLAINLITYDDRFNLKSIEEQLGTEIKPIPSNIDKSLYVAEYHSEPVED

DKP

>XP_010627615.1 probable ATP-dependent RNA helicase DDX6 [Fukomys damarensis]

MSTARTENPVIMGLSSQNGQLRGPVKPSGGPGGGGTQTQQQMNQLKNTNTINNGTQQQAQSLTTTIKPGDDWKKTLKLPP

KDLRIKTSDVTSTKGNEFEDYCLKRELLMGIFEMGWEKPSPIQEESIPIALSGRDILARAKNGTGKSGAYLIPLLERLDL

KKDNIQAMVIVPTRELALQVSQICIQVSKHMGGAKVMATTGGTNLRDDIMRLDDTVHVVIATPGRILDLIKKGVAKVDHV

QMIVLDEADKLLSQDFVQIMEDIILTLPKNRQILLYSATFPLSVQKFMNSHLQKPYEINLMEELTLKGVTQYYAYVTERQ

KVHCLNTLFSRLQINQSIIFCNSSQRVELLAKKISQLGYSCFYIHAKMRQEHRNRVFHDFRNGLCRNLVCTDLFTRGIDI

QAVNVVINFDFPKLAETYLHRIGRSGRFGHLGLAINLITYDDRFNLKSIEEQLGTEIKPIPSNIDKSLYVAEYHSEPVED

EKP

>XP_028274675.1 probable ATP-dependent RNA helicase DDX6 [Parambassis ranga]

MSTARTENPVILGLSNQNGQLRSSVKPAGAPGGGGGGPQQQQFNQMKGTINNGNSQPAPTTNAVIKPGDDWKKNLKLPPK

DMRMKTSDVTATKGNEFEDYCLKRELLMGIFEMGWEKPSPIQEESIPIALSGRDILARAKNGTGKSGAYLIPLLERIDLK

KDCIQALVVVPTRELALQVSQICIQVSKHMGGVKVMATTGGTNLRDDIMRLDETVHVVIATPGRILDLIKKGVAKVNQVQ

MIVLDEADKLLSQDFVVMMEEILGFLPKQRQILLYSATFPLSVQKFMNAHLQKPYEINLMEELTLKGVTQYYAYVTERQK

VHCLNTLFSRLQINQSIIFCNSSQRVELLAKKISQLGYSCFYIHAKMRQEHRNRVFHDFRNGLCRNLVCTDLFTRGIDIQ

AVNVVINFDFPKLGETYLHRIGRSGRFGHLGLAINLITYDDRFNLKGIEEQLGTEIKPIPGIIDKSLYVAEYHSESGEEV

KP

>NXE39129.1 DDX6 helicase [Ptilorrhoa leucosticta]

MSTARTENPVIMGLSSQNGQLRGPVKPSGGPGGGGTQTQQQMNQLKNANVINNGTQQQAQSMTTTIKPGDDWKKTLKLPP

KDLRIKTSDVTSTKGNEFEDYCLKRELLMGIFEMGWEKPSPIQEESIPIALSGRDILARAKNGTGKSGAYLIPLLERLDL

KKDNIQAMVIVPTRELALQVSQICIQVSKHMGGAKVMATTGGTNLRDDIMRLDDTVHVVIATPGRILDLIKKGVAKVEHV

QMIVLDEANKLLSQDFVQIMEDIILTLPKNRQILLYSATFPLSVQKFMNSHLQKPYEINLMEELTLKGVTQYYAYVTERQ

KVHCLNTLFSRLQINQSIIFCNSSQRVELLAKKISQLGYSCFYIHAKMRQEHRNRVFHDFRNGLCRNLVCTDLFTRGIDI

QAVNVVINFDFPKLAETYLHRIGRSGRFGHLGLAINLITYDDRFNLKSIEEQLGTEIKPIPSNIDKSLYVAEYHSEPVED

EKQ

>NXE15101.1 DDX6 helicase [Lophotis ruficrista]

MSTARTENPVIMGLSSQNGQLRGPVKPSGGPGGGGTQTQQQMNQLKNANTINNGTQQQAQSLTTAIKPGDDWKKTLKLPP

KDLRIKTSDVTSTKGNEFEDYCLKRELLMGIFEMGWEKPSPIQEESIPIALSGRDILARAKNGTGKSGAYLIPLLERLDL

KKDNIQAMVIVPTRELALQVSQICIQVSKHMGGAKVMATTGGTNLRDDIMRLDDTVHVVIATPGRILDLIKKGVAKVEHV

QMIVLDEANKLLSQDFVQIMEDIILTLPKNRQILLYSATFPLSVQKFMNSHLQKPYEINLMEELTLKGVTQYYAYVTERQ

KVHCLNTLFSRLQINQSIIFCNSSQRVELLAKKISQLGYSCFYIHAKMRQEHRNRVFHDFRNGLCRNLVCTDLFTRGIDI

QAVNVVINFDFPKLAETYLHRIGRSGRFGHLGLAINLITYDDRFNLKSIEEQLGTEIKPIPSNIDKSLYVAEYHSEPVED

EKQ

>NWY43601.1 DDX6 helicase [Sylvia atricapilla]

MSTARTENPVIMGLSSQNGQLRGPVKPSGGPGGGGTQTQQQMNQLKNANTMNNGTQQQAQSMTTAIKPGDDWKKTLKLPP

KDLRIKTSDVTSTKGNEFEDYCLKRELLMGIFEMGWEKPSPIQEESIPIALSGRDILARAKNGTGKSGAYLIPLLERLDL

KKDNIQAMVIVPTRELALQVSQICIQVSKHMGGAKVMATTGGTNLRDDIMRLDDTVHVVIATPGRILDLIKKGVAKVEHV

QMIVLDEANKLLSQDFVQIMEDIILTLPKNRQILLYSATFPLSVQKFMNSHLQKPYEINLMEELTLKGVTQYYAYVTERQ

KVHCLNTLFSRLQINQSIIFCNSSQRVELLAKKISQLGYSCFYIHAKMRQEHRNRVFHDFRNGLCRNLVCTDLFTRGIDI

QAVNVVINFDFPKLAETYLHRIGRSGRFGHLGLAINLITYDDRFNLKSIEEQLGTEIKPIPSNIDKSLYVAEYHSEPVED

EKQ

>CAG09056.1 unnamed protein product [Tetraodon nigroviridis]

MSTARTENPVILGLSNQNGQLRGSVKPAGAPGGGGGGPQQLQINQMKGAINNGNSQPAPTTNAVIKPGDDWKKNLKLPPK

DLRMKTSDVTATKGNEFEDYCLKRELLMGIFEMGWEKPSPIQEESIPIALSGRDILARAKNGTGKSGAYLIPLLERIDLK

RDCIQALVIVPTRELALQVSQICIQVSKHMGGVKVMATTGGTNLRDDIMRLDETVHVVIATPGRILDLIKKGVAKVSQVQ

MIVLDEADKLLSQDFVGMMEEILGFLSKQRQILLYSATFPLSVQKFMTSHLQKPYEINLMEELTLKGVTQYYAYVTERQK

VHCLNTLFSRLQINQSIIFCNSSQRVELLAKKISQLGYSCFYIHAKMRQEHRNRVFHDFRNGLCRNLVCTDLFTRGIDIQ

AVNVVINFDFPKLGETYLHRIGRSGRFGHLGLAINLITYDDRFNLKGIEEQLGTEIKPIPGIIDKSLYVAEYHSESGEEV

KP

>XP_007909384.1 PREDICTED: probable ATP-dependent RNA helicase DDX6 isoform X2 [Callorhinchus milii]

MTTARTENPIIMGLSTQNGQLRGPLKPSTGPGGMPPQQTTQTNQQLQQLKHASTLNGTQQQAHTTSTTIKPGDDWKKSLK

LPPKDRRMKTSDVTATKGNEFEDYCLKRELLMGIFEMGWEKPSPIQEESIPIALSGRDILARAKNGTGKSGAYLIPLLER

LDLKRDCIQAMVIVPTRELALQVSQICIQVSKHMGGVKVMATTGGTNLRDDIMRLDETVHVIIATPGRILDLIKKGLAKV

DNIQMIVLDEADKLLSQDFVQMMEDIISTLPRNRQILLYSATFPLSVQKFMNSHLQKPYEINLMEELTLKGVTQYYAYVT

ERQKVHCLNTLFSRLQINQSIIFCNSSQRVELLAKKISQLGYSCFYIHAKMRQEHRNRVFHDFRNGMCRNLVCTDLFTRG

IDIQAVNVVINFDFPKIAETYLHRIGRSGRFGHLGLAINLITYDDRFNLKAIEEQLGTEIKPIPGSIDKSLYVAEYHSEP

DGEDKQ

>NWS48521.1 DDX6 helicase [Probosciger aterrimus]

MSTARTENPVIMGLSSQNGQLRGPVKPSGGPGGGGTQTQQQMNQLKNANTINNGTQQQAQIMTTAIKPGDDWKKTLKLPP

KDLRIKTSDVTSTKGNEFEDYCLKRELLMGIFEMGWEKPSPIQEESIPIALSGRDILARAKNGTGKSGAYLIPLLERLDL

KKDNIQAMVIVPTRELALQVSQICIQVSKHMGGAKVMATTGGTNLRDDIMRLDDTVHVVIATPGRILDLIKKGVAKVEHV

QMIVLDEANKLLSQDFVQIMEDIILTLPKNRQILLYSATFPLSVQKFMNSHLQKPYEINLMEELTLKGVTQYYAYVTERQ

KVHCLNTLFSRLQINQSIIFCNSSQRVELLAKKISQLGYSCFYIHAKMRQEHRNRVFHDFRNGLCRNLVCTDLFTRGIDI

QAVNVVINFDFPKLAETYLHRIGRSGRFGHLGLAINLITYDDRFNLKSIEEQLGTEIKPIPSNIDKSLYVAEYHSEPVED

EKQ

>NXY15646.1 DDX6 helicase [Atrichornis clamosus]

MSTARTENPVIMGLSSQNGQLRGPVKPSGGPGGGGTQTQQQMNQLKNASTINNGTQQQAQSMTTTIKPGDDWKKTLKLPP

KDLRIKTSDVTSTKGNEFEDYCLKRELLMGIFEMGWEKPSPIQEESIPIALSGRDILARAKNGTGKSGAYLIPLLERLDL

KKDNIQAMVIVPTRELALQVSQICIQVSKHMGGAKVMATTGGTNLRDDIMRLDDTVHVVIATPGRILDLIKKGVAKVEHV

QMIVLDEANKLLSQDFVQIMEDIILTLPKNRQILLYSATFPLSVQKFMNSHLQKPYEINLMEELTLKGVTQYYAYVTERQ

KVHCLNTLFSRLQINQSIIFCNSSQRVELLAKKISQLGYSCFYIHAKMRQEHRNRVFHDFRNGLCRNLVCTDLFTRGIDI

QAVNVVINFDFPKLAETYLHRIGRSGRFGHLGLAINLITYDDRFNLKSIEEQLGTEIKPIPSNIDKSLYVAEYHSEPVED

EKQ

>XP_028639522.1 probable ATP-dependent RNA helicase DDX6 [Grammomys surdaster]

MSTARTENPVIMGLSSQNGQLRGPVKASAGPGGGGTQPQPQMNQLKNTSTINNGTQQQAQSMAATIKPGDDWKKTLKLPP

KDLRIKTSDVTSTKGNEFEDYCLKRELLMGIFEMGWEKPSPIQEESIPIALSGRDILARAKNGTGKSGAYLIPLLERLDL

KKDNIQAMVIVPTRELALQVSQICIQVSKHMGGAKVMATTGGTNLRDDIMRLDDTVHVVIATPGRILDLIKKGVAKVDHV

QMIVLDEADKLLSQDFVQIMEDIILTLPKNRQILLYSATFPLSVQKFMNSHLQKPYEINLMEELTLKGVTQYYAYVTERQ

KVHCLNTLFSRLQINQSIIFCNSSQRVELLAKKISQLGYSCFYIHAKMRQEHRNRVFHDFRNGLCRNLVCTDLFTRGIDI

QAVNVVINFDFPKLAETYLHRIGRSGRFGHLGLAINLITYDDRFNLKSIEEQLGTEIKPIPSNIDKSLYVAEYHSEPAED

EKP

>XP_031201275.1 probable ATP-dependent RNA helicase DDX6 [Mastomys coucha]

MSTARTENPVLMGLSSQNGQLRGPVKASAGPGGGGSQPQPQPMNQLKNTSTINNGTQQQAQSMAATIKPGDDWKKTLKLP

PKDLRIKTSDVTSTKGNEFEDYCLKRELLMGIFEMGWEKPSPIQEESIPIALSGRDILARAKNGTGKSGAYLIPLLERLD

LKKDNIQAMVIVPTRELALQVSQICIQVSKHMGGAKVMATTGGTNLRDDIMRLDDTVHVVIATPGRILDLIKKGVAKVDH

VQMIVLDEADKLLSQDFVQIMEDIILTLPKNRQILLYSATFPLSVQKFMNSHLQKPYEINLMEELTLKGVTQYYAYVTER

QKVHCLNTLFSRLQINQSIIFCNSSQRVELLAKKISQLGYSCFYIHAKMRQEHRNRVFHDFRNGLCRNLVCTDLFTRGID

IQAVNVVINFDFPKLAETYLHRIGRSGRFGHLGLAINLITYDDRFNLKSIEEQLGTEIKPIPSNIDKSLYVAEYHSEPAE

DEKP

>XP_021493584.1 probable ATP-dependent RNA helicase DDX6 [Meriones unguiculatus]

MSTARTENPVIMGLSSQNGQLRGPVKASGGPGGGGTQTQQQMNQLKNTSTINNGTQQQAQSMAATIKPGDDWKKTLKLPP

KDLRIKTSDVTSTKGNEFEDYCLKRELLMGIFEMGWEKPSPIQEESIPIALSGRDILARAKNGTGKSGAYLIPLLERLDL

KKDNIQAMVIVPTRELALQVSQICIQVSKHMGGAKVMATTGGTNLRDDIMRLDDTVHVVIATPGRILDLIKKGVAKVDHV

QMIVLDEADKLLSQDFVQIMEDIILTLPKNRQILLYSATFPLSVQKFMNSHLQKPYEINLMEELTLKGVTQYYAYVTERQ

KVHCLNTLFSRLQINQSIIFCNSSQRVELLAKKISQLGYSCFYIHAKMRQEHRNRVFHDFRNGLCRNLVCTDLFTRGIDI

QAVNVVINFDFPKLAETYLHRIGRSGRFGHLGLAINLITYDDRFNLKSIEEQLGTEIKPIPSNIDKSLYVAEYHSEPVED

EKP

>XP_004560553.1 probable ATP-dependent RNA helicase DDX6 [Maylandia zebra]

MSTARTENPVILGLPNQNGQLRGSVKPAGAPGGGGGGPQQQLNQMKGTINNGNSQPAPTTNAVIKPGDDWKKNLKLPPKD

MRMKTSDVTATKGNEFEDYCLKRELLMGIFEMGWEKPSPIQEESIPIALSGRDILARAKNGTGKSGAYLIPLLERIDLKK

DCIQALVIVPTRELALQVSQICIQVSKHMGGVKVMATTGGTNLRDDIMRLDETVHVVIATPGRILDLIKKGVAKVGQVQM

IVLDEADKLLSQDFVVMMEEILGFLPKQRQILLYSATFPLSVQKFMNSHLQKPYEINLMEELTLKGVTQYYAYVTERQKV

HCLNTLFSRLQINQSIIFCNSSQRVELLAKKISQLGYSCFYIHAKMRQEHRNRVFHDFRNGLCRNLVCTDLFTRGIDIQA

VNVVINFDFPKLGETYLHRIGRSGRFGHLGLAINLITYDDRFNLKGIEEQLGTEIKPIPGIIDKSLYVAEYHSESGEEVK

P

>NP_001102762.1 probable ATP-dependent RNA helicase DDX6 [Rattus norvegicus]

MSTARTENPVIMGLSSQNGQLRGPVKASAGPGGGGPQTQTQMNQLKNTSTINNGTQQQAQSMAATIKPGDDWKKTLKLPP

KDLRIKTSDVTSTKGNEFEDYCLKRELLMGIFEMGWEKPSPIQEESIPIALSGRDILARAKNGTGKSGAYLIPLLERLDL

KKDNIQAMVIVPTRELALQVSQICIQVSKHMGGAKVMATTGGTNLRDDIMRLDDTVHVVIATPGRILDLIKKGVAKVDHV

QMIVLDEADKLLSQDFVQIMEDIILTLPKNRQILLYSATFPLSVQKFMNSHLQKPYEINLMEELTLKGVTQYYAYVTERQ

KVHCLNTLFSRLQINQSIIFCNSSQRVELLAKKISQLGYSCFYIHAKMRQEHRNRVFHDFRNGLCRNLVCTDLFTRGIDI

QAVNVVINFDFPKLAETYLHRIGRSGRFGHLGLAINLITYDDRFNLKSIEEQLGTEIKPIPSNIDKSLYVAEYHSEPAED

EKP

>XP_015668400.1 probable ATP-dependent RNA helicase DDX6 [Protobothrops mucrosquamatus]

MSTARTENPVIMGLSSQNGQLRGPVKPGGGPGSGGTQAQQQINQLKHPNTINNGTQQQAQSMASTIKPGDDWKKTLKLPP

KDLRIKTSDVTSTKGNEFEDYCLKRELLMGIFEMGWEKPSPIQEESIPIALSGRDILARAKNGTGKSGAYLIPLLERLDL

KKDHIQAMVIVPTRELALQVSQICIQVSKHMGGAKVMATTGGTNLRDDIMRLDDTVHVVIATPGRILDLIKKGVAKVDHI

QMIVLDEADKLLSQDFVQIMEDIILTLPKNRQILLYSATFPLSVQKFMNSHLQKPYEINLMEELTLKGVTQYYAYVTERQ

KVHCLNTLFSRLQINQSIIFCNSSQRVELLAKKISQLGYSCFYIHAKMRQEHRNRVFHDFRNGLCRNLVCTDLFTRGIDI

QAVNVVINFDFPKLAETYLHRIGRSGRFGHLGLAINLITYDDRFNLKSIEEQLGTEIKPIPSNIDKSLYVAEYHSEPVED

DKP

>NP_001104296.1 probable ATP-dependent RNA helicase DDX6 [Mus musculus]

MSTARTENPVIMGLSSQNGQLRGPVKASAGPGGGGTQPQPQLNQLKNTSTINNGTPQQAQSMAATIKPGDDWKKTLKLPP

KDLRIKTSDVTSTKGNEFEDYCLKRELLMGIFEMGWEKPSPIQEESIPIALSGRDILARAKNGTGKSGAYLIPLLERLDL

KKDNIQAMVIVPTRELALQVSQICIQVSKHMGGAKVMATTGGTNLRDDIMRLDDTVHVVIATPGRILDLIKKGVAKVDHV

QMIVLDEADKLLSQDFVQIMEDIILTLPKNRQILLYSATFPLSVQKFMNSHLQKPYEINLMEELTLKGVTQYYAYVTERQ

KVHCLNTLFSRLQINQSIIFCNSSQRVELLAKKISQLGYSCFYIHAKMRQEHRNRVFHDFRNGLCRNLVCTDLFTRGIDI

QAVNVVINFDFPKLAETYLHRIGRSGRFGHLGLAINLITYDDRFNLKSIEEQLGTEIKPIPSNIDKSLYVAEYHSEPAED

EKP

>GCF49779.1 hypothetical protein parPi_0010586 [Paroedura picta]

MSTARTENPVIMGLSSQNGQLRGPVKPSGGPGGGGTQAQQQINQLKHPNTINNGTQQQAQGMPSTLKPGDDWKKTLKLPP

KDLRIKTSDVTSTKGNEFEDYCLKRELLMGIFEMGWEKPSPIQEESIPIALSGRDILARAKNGTGKSGAYLIPLLERLDL

KKDNIQAMVIVPTRELALQVSQICIQVSKHMGGAKVMATTGGTNLRDDIMRLDDTVHVVIATPGRILDLIKKGVAKVDHV

QMIVLDEADKLLSQDFVQIMEDIILTLPKNRQILLYSATFPLSVQKFMNSHLQKPYEINLMEELTLKGVTQYYAYVTERQ

KVHCLNTLFSRLQINQSIIFCNSSQRVELLAKKISQLGYSCFYIHAKMRQEHRNRVFHDFRNGLCRNLVCTDLFTRGIDI

QAVNVVINFDFPKLAETYLHRIGRSGRFGHLGLAINLITYDDRFNLKSIEEQLGTEIKPIPSNIDKSLYVAEYHSEPGEE

EKP

>NWW95832.1 DDX6 helicase [Rhynochetos jubatus]

MSTARTENPVIMGLSSQNGQLRGPVKPSGGPGGGGTQTQQQMNQLKNASAINNGTQQQAQSVTAAMKPGDDWKKTLKLPP

KDLRIKTSDVTSTKGNEFEDYCLKRELLMGIFEMGWEKPSPIQEESIPIALSGRDILARAKNGTGKSGAYLIPLLERLDL

KKDNIQAMVIVPTRELALQVSQICIQVSKHMGGAKVMATTGGTNLRDDIMRLDDTVHVVIATPGRILDLIKKGVAKVEHV

QMIVLDEADKLLSQDFVQIMEDIILTLPKNRQILLYSATFPLSVQKFMNSHLQKPYEINLMEELTLKGVTQYYAYVTERQ

KVHCLNTLFSRLQINQSIIFCNSSQRVELLAKKISQLGYSCFYIHAKMRQEHRNRVFHDFRNGLCRNLVCTDLFTRGIDI

QAVNVVINFDFPKLAETYLHRIGRSGRFGHLGLAINLITYDDRFNLKSIEEQLGTEIKPIPSNIDKSLYVAEYHSEPVEE

EKQ

>NP_001083721.1 ATP-dependent RNA helicase ddx6 [Xenopus laevis]

MSTARTENPVLMGMSSQNGQLRGPLKPSAGPGGGGTQTQQINQLKNASTINSGSQQQAQSMSSIIKPGDDWKKTLKLPPK

DLRIKTSDVTSTKGNEFEDYCLKRELLMGIFEMGWEKPSPIQEESIPIALSGRDILARAKNGTGKTGAYLIPLLERLDLK

KDCIQAMVIVPTRELALQVSQICIQVSKHMGGAKVMATTGGTNLRDDIMRLDDTVHVVIATPGRILDLIKKGVAKVDHIQ

MIVLDEADKLLSQDFMQIMEDIIMTLPKNRQILLYSATFPLSVQKFMTLHLQKPYEINLMEELTLKGVTQYYAYVTERQK

VHCLNTLFSRLQINQSIIFCNSSQRVELLAKKISQLGYSCFYIHAKMRQEHRNRVFHDFRNGLCRNLVCTDLFTRGIDIQ

AVNVVINFDFPKLAETYLHRIGRSGRFGHLGLAINLITYDDRFNLKSIEEQLGTEIKPIPSSIDKNLYVAEYHSESGEDK

P

>XP_006152352.1 probable ATP-dependent RNA helicase DDX6 isoform X2 [Tupaia chinensis]

MSTARTENPVIMGLSSQNGQLRGPVKPSGGPGGGGTQTQQQMNQLKNTNTINNGTQQQAQSVTTTIKPGDDWKKTLKLPP

KDLRIKTSDVTSTKGNEFEDYCLKRELLMGIFEMGWEKPSPIQEESIPIALSGRDILARAKNGTGKSGAYLIPLLERLDL

KKDNIQAMVIVPTRELALQVSQICIQVSKHMGGAKVMATTGGTNLRDDIMRLDDTVHVVIATPGRILDLIKKGVAKVDHV

QMIVLDEADKLLSQDFVQIMEDIILTLPKNRQILLYSATFPLSVQKFMNSHLQKPYEINLMEELTLKGVTQYYAYVTERQ

KVHCLNTLFSRLQINQSIIFCNSSQRVELLAKKISQLGYSCFYIHAKMRQEHRNRVFHDFRNGLCRNLVCTDLFTRGIDI

QAVNVVINFDFPKLAETYLHRIGRSGRFGHLGLAINLITYDDRFNLKSIEEQLGTEIKPIPSNIDKSLYVAEYHSEPVED

EKP

>XP_023662884.1 probable ATP-dependent RNA helicase DDX6 [Paramormyrops kingsleyae]

MSTARTENPVILGLSSQNGQLRGSVKPAGGPSGGGGGAQLQPTSQVKSSSTINNGSSQPMPTANTVIKPGDDWKKNLKLP

PKDMRMKTSDVTATKGNEFEDYCLKRELLMGIFEMGWEKPSPIQEESIPIALSGRDILARAKNGTGKSGAYLIPLLERID

LKKDCIQALVIVPTRELALQVSQICIQVSKHMGGVKVMATTGGTNLRDDIMRLDETVHVVIATPGRILDLIKKGVAKVDQ

VQMIVLDEADKLLSQDFVQMMEEILSFLSKQRQILLYSATFPLSVQKFMGSHLQKPYEINLMEELTLKGVTQYYAYVTER

QKVHCLNTLFSRLQINQSIIFCNSSQRVELLAKKISQLGYSCFYIHAKMRQEHRNRVFHDFRNGLCRNLVCTDLFTRGID

IQAVNVVINFDFPKLGETYLHRIGRSGRFGHLGLAINLITYDDRFNLKGIEEQLGTEIKPIPGSIDKSLYVAEYHSESGE

ELKL

>NWU17094.1 DDX6 helicase [Cephalopterus ornatus]

MSTARTENPVIMGLSSQNGQLRGPVKPSGGPGGGGTQTQQQMNQLKNASTINNGTQQQAQSMTTAIKPGDDWKKTLKLPP

KDLRIKTSDVTSTKGNEFEDYCLKRELLMGIFEMGWEKPSPIQEESIPIALSGRDILARAKNGTGKSGAYLIPLLERLDL

KKDNIQAMVIVPTRELALQVSQICIQVSKHMGGAKVMATTGGTNLRDDIMRLDDTVHVVIATPGRILDLIKKGVAKVEHV

QMIVLDEANKLLSQDFVQIMEDIILTLPKNRQILLYSATFPLSVQKFMNSHLQKPYEINLMEELTLKGVTQYYAYVTERQ

KVHCLNTLFSRLQINQSIIFCNSSQRVELLAKKISQLGYSCFYIHAKMRQEHRNRVFHDFRNGLCRNLVCTDLFTRGIDI

QAVNVVINFDFPKLAETYLHRIGRSGRFGHLGLAINLITYDDRFNLKSIEEQLGTEIKPIPSNIDKSLYVAEYHSEPVED

EKQ

>EDL25604.1 DEAD (Asp-Glu-Ala-Asp) box polypeptide 6, isoform CRA_a, partial [Mus musculus]

QSMSTARTENPVIMGLSSQNGQLRGPVKASAGPGGGGTQPQPQLNQLKNTSTINNGTPQQAQSMAATIKPGDDWKKTLKL

PPKDLRIKTSDVTSTKGNEFEDYCLKRELLMGIFEMGWEKPSPIQEESIPIALSGRDILARAKNGTGKSGAYLIPLLERL

DLKKDNIQAMVIVPTRELALQVSQICIQVSKHMGGAKVMATTGGTNLRDDIMRLDDTVHVVIATPGRILDLIKKGVAKVD

HVQMIVLDEADKLLSQDFVQIMEDIILTLPKNRQILLYSATFPLSVQKFMNSHLQKPYEINLMEELTLKGVTQYYAYVTE

RQKVHCLNTLFSRLQINQSIIFCNSSQRVELLAKKISQLGYSCFYIHAKMRQEHRNRVFHDFRNGLCRNLVCTDLFTRGI

DIQAVNVVINFDFPKLAETYLHRIGRSGRFGHLGLAINLITYDDRFNLKSIEEQLGTEIKPIPSNIDKSLYVAEYHSEPA

EDEKP

>XP_029811857.1 probable ATP-dependent RNA helicase DDX6 [Suricata suricatta]

MSTARTENPVIMGLSSQNGQLRGPVKPSGGPGGGGPQTQQQMNQLKNAGTINNGTQQQAQSMTATIKPGDDWKKTLKLPP

KDLRIKTSDVTSTKGNEFEDYCLKRELLMGIFEMGWEKPSPIQEESIPIALSGRDILARAKNGTGKSGAYLIPLLERLDL

KKDNIQAMVIVPTRELALQVSQICIQVSKHMGGAKVMATTGGTNLRDDIMRLDDTVHVVIATPGRILDLIKKGVAKVDHV

QMIVLDEADKLLSQDFVQIMEDIILTLPKNRQILLYSATFPLSVQKFMNSHLQKPYEINLMEELTLKGVTQYYAYVTERQ

KVHCLNTLFSRLQINQSIIFCNSSQRVELLAKKISQLGYSCFYIHAKMRQEHRNRVFHDFRNGLCRNLVCTDLFTRGIDI

QAVNVVINFDFPKLAETYLHRIGRSGRFGHLGLAINLITYDDRFNLKSIEEQLGTEIKPIPSNIDKSLYVAEYHSEPVED

EKP

>XP_030599451.1 probable ATP-dependent RNA helicase DDX6 [Archocentrus centrarchus]

MSTARTENPVILGLPNQNGQLRGSVKPAGAPGGGGGGPQQQQLNQMKGTINNGNSQPAPTTNAVIKPGDDWKKNLKLPPK

DMRMKTSDVTATKGNEFEDYCLKRELLMGIFEMGWEKPSPIQEESIPIALSGRDILARAKNGTGKSGAYLIPLLERIDLK

KDCIQALVIVPTRELALQVSQICIQVSKHMGGVKVMATTGGTNLRDDIMRLDETVHVVIATPGRILDLIKKGVAKVGQVQ

MIVLDEADKLLSQDFVVMMEEILGFLPKQRQILLYSATFPLSVQKFMNAHLQKPYEINLMEELTLKGVTQYYAYVTERQK

VHCLNTLFSRLQINQSIIFCNSSQRVELLAKKISQLGYSCFYIHAKMRQEHRNRVFHDFRNGLCRNLVCTDLFTRGIDIQ

AVNVVINFDFPKLGETYLHRIGRSGRFGHLGLAINLITYDDRFNLKGIEEQLGTEIKPIPGIIDKSLYVAEYHSESGEEV

KP

>XP_018521005.1 PREDICTED: probable ATP-dependent RNA helicase DDX6 [Lates calcarifer]

MSTARTENPVILGLSNQNGQLRGSVKPAGAPGGGGGGPQQQQLNQMKGTINNGNSQPAPTTNAVIKPGDDWKKNLKLPPK

DMRMKTSDVTATKGNEFEDYCLKRELLMGIFEMGWEKPSPIQEESIPIALSGRDILARAKNGTGKSGAYLIPLLERIDLK

KDCIQAVVIVPTRELALQVSQICIQVSKHMGGVKVMATTGGTNLRDDIMRLDETVHVVIATPGRILDLIKKGVAKVNQVQ

MIVLDEADKLLSQDFVVMMEEILGFLPKQRQILLYSATFPLSVQKFMNAHLQKPYEINLMEELTLKGVTQYYAYVTERQK

VHCLNTLFSRLQINQSIIFCNSSQRVELLAKKISQLGYSCFYIHAKMRQEHRNRVFHDFRNGLCRNLVCTDLFTRGIDIQ

AVNVVINFDFPKLGETYLHRIGRSGRFGHLGLAINLITYDDRFNLKGIEEQLGTEIKPIPGIIDKSLYVAEYHSESGEEV

KP

>XP_025854894.1 probable ATP-dependent RNA helicase DDX6 [Vulpes vulpes]

MSTARTENPVIMGLSSQNGQLRGPVKPSGGPGGGGTQTQQQMNQLKNTNTINNGTQQQAQSMTTTIKPGDDWKKTLKLPP

KDLRIKTSDVTSTKGNEFEDYCLKRELLMGIFEMGWEKPSPIQEESIPIALSGRDILARAKNGTGKSSAYLIPLLERLDL

KKDNIQAMVIVPTRELALQVSQICIQVSKHMGGAKVMATTGGTNLRDDIMRLDDTVHVVIATPGRILDLIKKGVAKADHV

QMIVLDEADKLLSQDFVQIMEDIILMLPKNRQILLYSATFPLSVQKFMNSHLQKPYEINLMEELTLKGVTQYYAYVTERQ

KVHCLNTLFSRLQINQSIIFCNSSQRVELLAKKISQLGYSCFYIHAKMRQEHRNRVFHDFRNGLCRNLVCTDLFTRGIDI

QAVNVVINFDFPKLAETYLHRIGRSGRFGHLGLAINLITYDDRFNLKSIEEQLGTEIKPIPSNIDKSLYVAEYHSEPVED

EKP

>XP_003450212.1 probable ATP-dependent RNA helicase DDX6 [Oreochromis niloticus]

MSTARTENPVILGLPNQNGQLRGSVKPAGAPGGGGGGPQQLNQMKGTINNGNSQPAPTTNAVIKPGDDWKKNLKLPPKDM

RMKTSDVTATKGNEFEDYCLKRELLMGIFEMGWEKPSPIQEESIPIALSGRDILARAKNGTGKSGAYLIPLLERIDLKKD

CIQALVIVPTRELALQVSQICIQVSKHMGGVKVMATTGGTNLRDDIMRLDETVHVVIATPGRILDLIKKGVAKVGQVQMI

VLDEADKLLSQDFVVMMEEILGFLPKQRQILLYSATFPLSVQKFMNSHLQKPYEINLMEELTLKGVTQYYAYVTERQKVH

CLNTLFSRLQINQSIIFCNSSQRVELLAKKISQLGYSCFYIHAKMRQEHRNRVFHDFRNGLCRNLVCTDLFTRGIDIQAV

NVVINFDFPKLGETYLHRIGRSGRFGHLGLAINLITYDDRFNLKGIEEQLGTEIKPIPGIIDKSLYVAEYHSESGEEVKP

>XP_013871325.1 PREDICTED: probable ATP-dependent RNA helicase DDX6 [Austrofundulus limnaeus]

MSTTRTENPVILGLSNQNGQLRGSVKPAGAPGGGGGGPPQLNQMKGKVNGSSQPAPPTNAVIKPGDDWKKNLKLPPKDMR

IKTSDVTATKGNEFEDYCLKRELLMGIFEMGWEKPSPIQEESIPIALSGRDILARAKNGTGKSGAYLIPLLERIDLKKSC

LQALVIVPTRELALQVSQICIQVSKHMGGVKVMATTGGTNLRDDIMRLDETVHVVIATPGRILDLIKKGVAKVNQVQMIV

LDEADKLLSQDFVVMMEEMLGFLPKHRQILLYSATFPLSVQKFMNAHLQKPYEINLMEELTLKGVTQYYAYVTERQKVHC

LNTLFSRLQINQSIIFCNSSQRVELLAKKISQLGYSCFYIHAKMRQEHRNRVFHDFRNGLCRNLVCTDLFTRGIDIQAVN

VVINFDFPKLGETYLHRIGRSGRFGHLGLAINLITYDDRFNLKGIEEQLGTEIKPIPGIIDKSLYVAEYHSESGEEAKQ

>XP_029964188.1 probable ATP-dependent RNA helicase DDX6 [Salarias fasciatus]

MSTARTENPVIMGLSTQNGQLRGSVKPAGAPGGGGGGPQQQPFSQMKGTINNGSSQPAPTTNAVIKPGDDWKKNLKLPPK

DMRMKTSDVTATKGNEFEDYCLKRELLMGIFEMGWEKPSPIQEESIPIALSGRDILARAKNGTGKSGAYLIPLLERIDLK

KDCIQAVVIVPTRELALQVSQICIQVSKHMGGVKVMATTGGTNLRDDIMRLDETVHVIIATPGRILDLIKKGVAKVNQVQ

MIVLDEADKLLSQDFVVMMEEILGFLPKQRQILLYSATFPLSVQKFMNAHLQKPYEINLMEELTLKGVTQYYAYVTERQK

VHCLNTLFSRLQINQSIIFCNSSQRVELLAKKISQLGYSCFYIHAKMRQEHRNRVFHDFRNGLCRNLVCTDLFTRGIDIQ

AVNVVINFDFPKLGETYLHRIGRSGRFGHLGLAINLITYDDRFNLKAIEEQLGTEIKPIPGIIDKSLYVAEYHSESGEEV

KP

>XP_016854329.1 PREDICTED: probable ATP-dependent RNA helicase DDX6 isoform X2 [Anolis carolinensis]

MSTARTENPVIMGLASQNGQLRGPVKPSGGPGGGGGTQGQPQINPLKHPNAINNGTQQQAQSMASTIKPGDDWKKTLKLP

PKDLRIKTSDVTSTKGNEFEDYCLKRELLMGIFEMGWEKPSPIQEESIPIALSGRDILARAKNGTGKSGAYLIPLLERLD

LKKDYIQAMVIVPTRELALQVSQICIQVSKHMGGAKVMATTGGTNLRDDIMRLDDTVHVVIATPGRILDLIKKGVAKVDH

IQMIVLDEADKLLSQDFVQIMEDIILTLPKNRQILLYSATFPLSVQKFMNSHLQKPYEINLMEELTLKGVTQYYAYVTER

QKVHCLNTLFSRLQINQSIIFCNSSQRVELLAKKISQLGYSCFYIHAKMRQEHRNRVFHDFRNGLCRNLVCTDLFTRGID

IQAVNVVINFDFPKLAETYLHRIGRSGRFGHLGLAINLITYDDRFNLKSIEEQLGTEIKPIPSNIDKSLYVAEYHSEPGE

DGKA

>XP_005069498.1 probable ATP-dependent RNA helicase DDX6 [Mesocricetus auratus]

MSTARTENPVIMGLSSQNGQLRGPVKPSGGPGGGGPQTQQQMNQLKNTNTINNGTQQQAQSMTATIKPGDDWKKTLKLPP

KDLRIKTSDVTSTKGNEFEDYCLKRELLMGIFEMGWEKPSPIQEESIPIALSGRDILARAKNGTGKSGAYLIPLLERLDL

KKDNILAMVIVPTRELALQVSQICIQVSKHMGGAKVMATTGGTNLRDDIMRLDDTVHVVIATPGRILDLIKKGVAKVDHV

QMIVLDEADKLLSQDFVQIMEDIILTLPKNRQILLYSATFPLSVQKFMNSHLQKPYEINLMEELTLKGVTQYYAYVTERQ

KVHCLNTLFSRLQINQSIIFCNSSQRVELLAKKISQLGYSCFYIHAKMRQEHRNRVFHDFRNGLCRNLVCTDLFTRGIDI

QAVNVVINFDFPKLAETYLHRIGRSGRFGHLGLAINLITYDDRFNLKSIEEQLGTEIKPIPSNIDKSLYVAEYHSEPVED

EKP

>XP_027300148.1 probable ATP-dependent RNA helicase DDX6 isoform X1 [Anas platyrhynchos]

MSTARTENPVIMGLSSQNGQLRGPVKPSGGPGGGGTQTQQQMNQLKNANTINNGTQQQAQSMTTTIKFFFGRPGDDWKKT

LKLPPKDLRIKTSDVTSTKGNEFEDYCLKRELLMGIFEMGWEKPSPIQEESIPIALSGRDILARAKNGTGKSGAYLIPLL

ERLDLKKDNIQAMVIVPTRELALQVSQICIQVSKHMGGAKVMATTGGTNLRDDIMRLDDTVHVVIATPGRILDLIKKGVA

KVEHVQMIVLDEADKLLSQDFVQIMEDIILTLPKNRQILLYSATFPLSVQKFMNSHLQKPYEINLMEELTLKGVTQYYAY

VTERQKVHCLNTLFSRLQINQSIIFCNSSQRVELLAKKISQLGYSCFYIHAKMRQEHRNRVFHDFRNGLCRNLVCTDLFT

RGIDIQAVNVVINFDFPKLAETYLHRIGRSGRFGHLGLAINLITYDDRFNLKSIEEQLGTEIKPIPSNIDKSLYVAEYHS

EPVEDEKQ

>KFW66637.1 putative ATP-dependent RNA helicase DDX6 [Pygoscelis adeliae]

MSTARTENPVIMGLSSQNGQLRGPVKPSGGPGGGGTQTQQQMNQLKNANTINNGTQQQAQSMTTAIKPGDDWKKTLKLPP

KDLRIKTSDVTSTKGNEFEDYCLKRELLMGIFEMGWEKPSPIQEESIPIALSGRDILARAKNGTGKSGAYLIPLLERLDL

KKDNIQAMVIVPTRELALQVSQICIQVSKHMGGAKVMATTGGTNLRDDIMRLDDTVHVVIATPGRILDLIKKGVAKVEHV

QMIVLDEANKLLSQDFVQIMEDIILTLPKNRQILLYSATFPLSVQKFMNSHLQKPYEINLMEELTLKGVTQYYAYVTERQ

KVHCLNTLFSRLQINQSIIFCNSSQRVELLAKKISQLGYSCFYIHAKMRQEHRNRVFHDFRNGLCRNLVCTDLFTRGIDI

QAVNVVINFDFPKLAETYLHRIGRSARFGHLGLAINLITYDDRFNLKSIEEQLGTEIKPIPSNIDKSLYVAEYHSEPVED

EKQ

>XP_033774211.1 probable ATP-dependent RNA helicase DDX6 [Geotrypetes seraphini]

MSTTRTENPVIMGLTSQNGQLRGPVKPSGGPGSGIQTQQQMNQLKNASTINNGTQQQAQSMPTTIKPGDDWKKTLKLPPK

DLRIKTSDVTSTKGNEFEDYCLKRELLMGIFEMGWEKPSPIQEESIPIALSGRDILARAKNGTGKSGAYLIPLLERLDLK

KDSIQAMVIVPTRELALQVSQICIQVSKHMGGVKVMATTGGTNLRDDIMRLDDTVHVVIATPGRILDLIKKGVAKVDQIQ

MMVLDEADKLLSQDFVQIMEDIILTLPKNRQILLYSATFPLSVQKFMNSHLQKPYEINLMEELTLKGVTQYYAYVTERQK

VHCLNTLFSRLQINQSIIFCNSSQRVELLAKKISQLGYSCFYIHAKMRQEHRNRVFHDFRNGLCRNLVCTDLFTRGIDIQ

AVNVVINFDFPKLAETYLHRIGRSGRFGHLGLAINLITYDDRFNLKSIEEQLGTEIKPIPSNIDKSLYVAEYHSEPVEDE

KP

>XP_033832720.1 probable ATP-dependent RNA helicase ddx6 [Periophthalmus magnuspinnatus]

MSTARTESPVILGLSNQNGQIRGSGKPGGGGGGGGGGGGGPQLNPIKGTVNNGDSLPATTTNAVIKPGDDWKKNLKLPPK

DMRMKTSDVTATKGNEFEDYCLKRELLMGIFEMGWEKPSPIQEESIPIALSGRDILARAKNGTGKSGAYLIPLLERIDLK

KDCIQAFVIVPTRELALQVSQICIQVSKHMGGVKVMATTGGTNLRDDIMRLDETVHVVIATPGRILDLIKKGVAKVNQVQ

MIVLDEADKLLSQDFVVMMEEILSFLPKQRQILLYSATFPLSVQKFMNSHLQKPYEINLMEELTLKGVTQYYAYVTERQK

VHCLNTLFSRLQINQSIIFCNSSQRVELLAKKISQLGYSCFYIHAKMRQEHRNRVFHDFRNGLCRNLVCTDLFTRGIDIQ

AVNVVINFDFPKLGETYLHRIGRSGRFGHLGLAINLITYDDRFNLKGIEEQLGTEIKPIPGIIDKSLYVAEYHSESHEEA

KP

>XP_005988179.1 PREDICTED: probable ATP-dependent RNA helicase DDX6 isoform X1 [Latimeria chalumnae]

MSTARTENPVIMGLSNQNGQLRGPVKPSGGPGGGGTQTQQQQMTNQLKNVSTINNGTQQQAQTLTTTLKPGDDWKKNLKI

PPKDRRIKTSDVTSTKGNEFEDYCLKRELLMGIFEMGWEKPSPIQEESIPIALSGRDILARAKNGTGKSGAYLIPLLERI

DLKKDSIQALVIVPTRELALQVSQICIQVSKHMGGVKVMATTGGTNLRDDIMRLDDTVHVVIATPGRILDLIKKGVAKVD

QIQMIVLDEADKLLSQDFVQIMEEIIFTLPKNRQILLYSATFPLSVQKFMGSHLQKPYEINLMEELTLKGVTQYYAYVTE

RQKVHCLNTLFSRLQINQSIIFCNSSQRVELLAKKISQLGYSCFYIHAKMRQEHRNRVFHDFRNGLCRNLVCTDLFTRGI

DIQAVNVVINFDFPKLAETYLHRIGRSGRFGHLGLAINLITYDDRFNLKSIEEQLGTEIKPIPGNIDKSLYVAEYHSEPG

DGEKH

>NXD02803.1 DDX6 helicase [Certhia familiaris]

MSTARTENPVIMGLSSQNGQLRGPVKPSGGPGGGGTQTQQQMNQLKNASTINNGTQQQAQSMTTALKPGDDWKKTLKLPP

KDLRIKTSDVTSTKGNEFEDYCLKRELLMGIFEMGWEKPSPIQEESIPIALSGRDILARAKNGTGKSGAYLIPLLERLDL

KKDNIQAMVIVPTRELALQVSQICIQVSKHMGGAKVMATTGGTNLRDDIMRLDDTVHVVIATPGRILDLIKKGVAKVEHV

QMIVLDEANKLLSQDFVQIMEDIILTLPKNRQILLYSATFPLSVQKFMNSHLQKPYEINLMEELTLKGVTQYYAYVTERQ

KVHCLNTLFSRLQINQSIIFCNSSQRVELLAKKISQLGYSCFYIHAKMRQEHRNRVFHDFRNGLCRNLVCTDLFTRGIDI

QAVNVVINFDFPKLAETYLHRIGRSGRFGHLGLAINLITYDDRFNLKSIEEQLGTEIKPIPSNIDKSLYVAEYHSEPVED

EKQ

>BAC35670.1 unnamed protein product [Mus musculus]

MSTARTENPVIMGLSSQNGQLRGPVKASAGPGGGGTQPQPQLNQLKNTSTINNGTPQQAQSMAATIKPGDDWKKTLKLPP

KDLRIKTSDVTSTKGNEFEDYCLKRELLMGIFEMGWEKPSPIQEESIPIALSGRDILARAKNGTGKSGAYLIPLLERLDL

KKDNIQAMVIVPTRELALQVSQICIQVSKHMGGAKVMATTGGTNLRDDIMRLDDTVHVVIATPGRILDLIKKGVAKVDHV

RMIVLDEADKLLSQDFVQIMEDIILTLPKNRQILLYSATFPLSVQKFMNSHLQKPYEINLMEELTLKGVTEYYAYVTERQ

KVHCLNTLFSRLQINQSIIFCNSSQRVELLAKKISQLGYSCFYIHAKMRQEHRNRVFHDFRNGLCRNLVCTDLFTRGIDI

QAVNVVMNFDFPKLAETYLHRIGRSGRFGHLGLAINLITYDDRFNLKSIEEQLGTEIKPIPSNIDKSLYVAEYHSEPAED

EKP

>NXH46694.1 DDX6 helicase [Dicaeum eximium]

MSTARTENPVIMGLSSQNGQLRGPVKPSGGPGGGGTQTQQQMNQLKNANTINNGTQQQAQSMTTAIKPGDDWKKTLKLPP

KDLRIKTSDVTSTKGNEFEDYCLKRELLMGIFEMGWEKPSPIQEESIPIALSGRDILARAKNGTGKSGAYLIPLLERLDL

KKDNIQAMVIVPTRELALQVSQICIQVSKHMGGAKVMATTGGTNLRDDIMRLDDTVHVVIATPGRILDLIKKGVAKVEQV

QMIVLDEANKLLSQDFVQIMEDIILTLPKNRQILLYSATFPLSVQKFMNSHLQKPYEINLMEELTLKGVTQYYAYVTERQ

KVHCLNTLFSRLQINQSIIFCNSSQRVELLAKKISQLGYSCFYIHAKMRQEHRNRVFHDFRNGLCRNLVCTDLFTRGIDI

QAVNVVINFDFPKLAETYLHRIGRSGRFGHLGLAINLITYDDRFNLKSIEEQLGTEIKPIPSNIDKSLYVAEYHSEPVED

EKQ

>XP_029428406.1 probable ATP-dependent RNA helicase DDX6 [Rhinatrema bivittatum]

MSTTRTENPVIMGLSSQNGQLRGPVKPSGGPGGGIQTQQQMNQLKNASTINNGTQQQAQSMPTTIKPGDDWKKTLKLPPK

DLRIKTSDVTSTKGNEFEDYCLKRELLMGIFEMGWEKPSPIQEESIPIALSGRDILARAKNGTGKSGAYLIPLLERLDLK

KDSIQAMVIVPTRELALQVSQICIQVSKHMGGVKVMATTGGTNLRDDIMRLDDTVHVVIATPGRILDLIKKGVAKVDQIQ

MIVLDEADKLLSQDFVQIMEDIILTLPKNRQILLYSATFPLSVQKFMNSHLQKPYEINLMEELTLKGVTQYYAYVTERQK

VHCLNTLFSRLQINQSIIFCNSSQRVELLAKKISQLGYSCFYIHAKMRQEHRNRVFHDFRNGLCRNLVCTDLFTRGIDIQ

AVNVVINFDFPKLAETYLHRIGRSGRFGHLGLAINLITYDDRFNLKSIEEQLGTEIKPIPSNIDKSLYVAEYHSEPVEDE

KP

>XP_021028749.1 probable ATP-dependent RNA helicase DDX6 [Mus caroli]

MSTARTENPVIMGLSSQNGXXRGPVKASAGPGGGGTQPQPQMNQLKNTSTINNGTPQQAQSMAATIKPGDDWKKTLKLPP

KDLRIKTSDVTSTKGNEFEDYCLKRELLMGIFEMGWEKPSPIQEESIPIALSGRDILARAKNGTGKSGAYLIPLLERLDL

KKDNIQAMVIVPTRELALQVSQICIQVSKHMGGAKVMATTGGTNLRDDIMRLDDTVHVVIATPGRILDLIKKGVAKVDHV

QMIVLDEADKLLSQDFVQIMEDIILTLPKNRQILLYSATFPLSVQKFMNSHLQKPYEINLMEELTLKGVTQYYAYVTERQ

KVHCLNTLFSRLQINQSIIFCNSSQRVELLAKKISQLGYSCFYIHAKMRQEHRNRVFHDFRNGLCRNLVCTDLFTRGIDI

QAVNVVINFDFPKLAETYLHRIGRSGRFGHLGLAINLITYDDRFNLKSIEEQLGTEIKPIPSNIDKSLYVAEYHSEPAED

EKP

>NXT14103.1 DDX6 helicase [Prunella fulvescens]

MSTARTENPVIMGLSSQNGQLRGPVKPSGGPGGGGTQTQQQMNQLKNANTINNGTQQQAQSMTTAIKPGDDWKKTLKLPP

KDLRIKTSDVTSTKGNEFEDYCLKRELLMGIFEMGWEKPSPIQEESIPIALSGRDILARAKNGTGKSGAYLIPLLERLDL

KKDNIQAMVIVPTRELALQVSQICIQVSKHMGGAKVMATTGGTNLRDDIMRLDDTVHVVIATPGRILDLIKKGVAKVEHV

QMIVLDEANKLLSQDFVQIMEDIILTLPKNRQILLYSATFPLSVQKFMNSHLQKPYEINLMEELTLKGVTQYYAYVTERQ

KVHCLNTLFSRLQINQSIIFCNSSQRVELLAKKISQLGYSCFYIHAKMRQEHRNRVFHDFRNGLCRNLVCTDLFTRGIDI

QAVNVVINFDFPKLAETYLHRIGRSGRFGHLGLDINLITYDDRFNLKSIEEQLGTEIKPIPSNIDKSLYVAEYHSEPVED

EKQ

>XP_029922562.1 probable ATP-dependent RNA helicase DDX6 [Myripristis murdjan]

MSTARTENPVILGLSNQNGQLRGSVKPAGGPGGGGGGPQQQHISQMKGSSTVNNGNPQPAPTSNTVIKPGDDWKKNLKLP

PKDMRMKTSDVTATKGNEFEDYCLKRELLMGIFEMGWEKPSPIQEESIPIALSGRDILARAKNGTGKSGAYLIPLLERID

LKKDCIQALGIVPTRELALQVSQICIQVSKHMGGVKVMATTGGTNLRDDIMRLDETVHVVIATPGRILDLIKKGVAKVNQ

VQMIVLDEADKLLSQDFVVMMEEILGFLPKQRQILLYSATFPLSVQKFMNSHLQKPYEINLMEELTLKGVTQYYAYVTER

QKVHCLNTLFSRLQINQSIIFCNSSQRVELLAKKISQLGYSCFYIHAKMRQEHRNRVFHDFRNGLCRNLVCTDLFTRGID

IQAVNVVINFDFPKLGETYLHRIGRSGRFGHLGLAINLITYDDRFNLKGIEEQLGTEIKPIPGIIDKSLYVAEYHSESGE

EVKL

>XP_028664305.1 probable ATP-dependent RNA helicase DDX6 [Erpetoichthys calabaricus]

MSSTRAENPVILGLSNQNGQLRGPMKPAGAPGGGGGGGCVTLTQQANQIKNSGTINNGNPQSNVPANSSIKPGDDWKKNL

KLPPKDMRMKTSDVTATKGNEFEDYCLKRELLMGIFEMGWEKPSPIQEESIPIALSGRDILARAKNGTGKSGAYLIPLLE

RIDLKKDCIQAVCIVPTRELALQVSQICIQVSKHMGGVKVMATTGGTNLRDDIMRLDETVHVVIATPGRILDLIKKGVAK

VGQVQMIVLDEADKLLSQDFVQMMEEIIGTLPKNRQILLYSATFPLSVQKFMNAHLQKPYEINLMEELTLKGVTQYYAYV

TERQKVHCLNTLFSRLQINQSIIFCNSSQRVELLAKKISQLGYSCFYIHAKMRQEHRNRVFHDFRNGLCRNLVCTDLFTR

GIDIQAVNVVINFDFPKLAETYLHRIGRSGRFGHLGLAINLITYDDRFNLKGIEEQLGTEIKPIPGSIDKSLYVAEYHSE

TGSEEKP

>XP_020649670.1 probable ATP-dependent RNA helicase DDX6 [Pogona vitticeps]

MSTARTENPVIMGLTSQNGQLRGPVKPSSGPGGTGTQAQQQINQLKHPTTINNGTQQQAQSMASTIKPGDDWKKTLKLPP

KDLRIKTSDVTSTKGNEFEDYCLKRELLMGIFEMGWEKPSPIQEESIPIALSGRDILARAKNGTGKSGAYLIPLLERLDL

KKDNIQAMVIVPTRELALQVSQICIQVSKHMGGAKVMATTGGTNLRDDIMRLDDTVHVVIATPGRILDLIKKGVAKVDHV

QMIVLDEADKLLSQDFVQIMEDIILTLPKNRQILLYSATFPLSVQKFMNSHLQKPYEINLMEELTLKGVTQYYAYVTERQ

KVHCLNTLFSRLQINQSIIFCNSSQRVELLAKKISQLGYSCFYIHAKMRQEHRNRVFHDFRNGLCRNLVCTDLFTRGIDI

QAVNVVINFDFPKLAETYLHRIGRSGRFGHLGLAINLITYDDRFNLKSIEEQLGTEIKPIPSNIDKSLYVAEYHSESVEE

EKP

>KAE8295019.1 putative ATP-dependent RNA helicase ddx6 [Larimichthys crocea]

MSTARTENPVILGLSNQNGQLRGSVKPAGAPGGGGGGGGGGGGGGPQQQQLNQMKGAINNGSSQPAPTTNAVIKPGDDWK

KNLKLPPKDMRMKTSDVTATKGNEFEDYCLKRELLMGIFEMGWEKPSPIQEESIPIALSGRDILARAKNGTGKSGAYLIP

LLERIDLKKDCIQALVIVPTRELALQVSQICIQVSKHMGGVKVMATTGGTNLRDDIMRLDETVHVVIATPGRILDLIKKG

VAKVNQVQMIVLDEADKLLSQDFVVMMEEILGFLAKQRQILLYSATFPLSVQKFMNAHLQKPYEINLMEELTLKGVTQYY

AYVTERQKVHCLNTLFSRLQINQSIIFCNSSQRVELLAKKISQLGYSCFYIHAKMRQEHRNRVFHDFRNGLCRNLVCTDL

FTRGIDIQAVNVVINFDFPKLGETYLHRIGRSGRFGHLGLAINLITYDDRFNLKGIEEQLGTEIKPIPGIIDKSLYVAEY

HSESGEEVKP

>XP_018079874.1 PREDICTED: ATP-dependent RNA helicase ddx6 isoform X1 [Xenopus laevis]

MSTARTENPVLMGMSSQNGQLRGPLKPSAGPGGGGTQTQQINQLKNASTINSGSQQQAQSMSSIIKPGDDWKKTLKLPPK

DLRIKTSDVTSTKGNEFEDYCLKRELLMGIFEMGWEKPSPIQEESIPIALSGRDILARAKNGTGKSGAYLIPLLERLDLK

KDCIQAMVIVPTRELALQVSQICIQVSKHMGGAKVMATTGGTNLRDDIMRLDDTVHVVIATPGRILDLIKKGVAKVDHIQ

MIVLDEADKLLSQDFMQIMEDIIMTLPKNRQILLYSATFPLSVQKFMTLHLQKPYEINLMEELTLKGVTQYYAYVTERQK

VHCLNTLFSRLQINQSIIFCNSSQRVELLAKKISQLGYSCFYIHAKMRQEHRNRVFHDFRNGLCRNLVCTDLFTRGIDIQ

AVNVVINFDFPKLAETYLHRIGRSGRFGHLGLAINLITYDDRFNLKSIEEQLGTEIKPIPSSIDKNLYVAEYHSESGEDK

P

>XP_035239749.1 probable ATP-dependent RNA helicase ddx6 [Anguilla anguilla]

MSTARTENPVILGLANQNGQIRGPLKPATAPPGGVGGSPQPQQTGQLNASSTINNGGSQPLPMANTIKVGDDWKKNLKLP

PKDMRMRTSDVTATKGNEFEDYCLKRELLMGIFEMGWEKPSPIQEESIPIALSGRDILARAKNGTGKSGAYLIPLLERID

LKKDCIQAVGIVPTRELALQVSQICIQVSRHMGGVKVMATTGGTNLRDDIMRLDETVHVIIATPGRILDLIKKGVAKVDK

VQMIVLDEADKLLSQDFLQMMEEMLSFLPKQRQILLYSATFPLSVQKFMNAHLQKPYEINLMEELTLKGVTQYYAYVTER

QKVHCLNTLFSRLQINQSIIFCNSSQRVELLAKKISQLGYSCFYIHAKMRQEHRNRVFHDFRNGLCRNLVCTDLFTRGID

IQAVNVVINFDFPKLGETYLHRIGRSGRFGHLGLAINLITYEDRFNLKGIEEQLGTEIKPIPGSIDKSLYVAEYHSENAE

EGKL

>XP_026988410.1 probable ATP-dependent RNA helicase DDX6 [Tachysurus fulvidraco]

MSTARTENPVILGLTNQNGQKRGSAKPTGGPGGGGGGPQTQPTSIKASSTVNNGSSLPLPTANTVIKPGDDWKKNLKLPP

KDMRMKTSDVTATKGNEFEDYCLKRELLMGIFEMGWEKPSPIQEESIPIALSGRDILARAKNGTGKSGAYLIPLLERIDL

KKDCIQALVVVPTRELALQVSQICIQVSKHMGGVKVMATTGGTNLRDDIMRLDETVHVVIATPGRVLDLIKKGVAKVGQV

QMIVLDEADKLLSQDFVQMMEEILSFLSKQRQILLYSATFPLSVQKFMNSHLQKPYEINLMEELTLKGVTQYYAYVTERQ

KVHCLNTLFSRLQINQSIIFCNSSQRVELLAKKISQLGYSCFYIHAKMRQEHRNRVFHDFRNGLCRNLVCTDLFTRGIDI

QAVNVVINFDFPKLGETYLHRIGRSGRFGHLGLAINLITYDDRFNLKGIEEQLGTEIKPIPSSIDKSLYVAEYHSESTED

IKL

>XP_015283539.1 PREDICTED: probable ATP-dependent RNA helicase DDX6 [Gekko japonicus]

MSTARTENPVIMGLSSQNGQLRGPVKPSSGPGGGGTQAQQQINQLKHPNTINNGTQQQAQGMPSTLKPGDDWKKTLKLPP

KDLRIKTSDVTSTKGNEFEDYCLKRELLMGIFEMGWEKPSPIQEESIPIALSGRDILARAKNGTGKSGAYLIPLLERLDL

KKDNIQAMVIVPTRELALQVSQICIQVSKHMGGAKVMATTGGTNLRDDIMRLDDTVHVVIATPGRILDLIKKGVAKVDHV

QMIVLDEADKLLSQDFVQIMEDIILTLPKNRQILLYSATFPLSVQKFMNSHLQKPYEINLMEELTLKGVTQYYAYVTERQ

KVHCLNTLFSRLQINQSIIFCNSSQRVELLAKKISQLGYSCFYIHAKMRQEHRNRVFHDFRNGLCRNLVCTDLFTRGIDI

QAVNVVINFDFPKLAETYLHRIGRSGRFGHLGLAINLITYDDRFNLKSIEEQLGTEIKPIPSNIDKSLYVAEYHSEPVEE

EKP

>XP_032905384.1 probable ATP-dependent RNA helicase DDX6 [Amblyraja radiata]

MTTARTEIPVIMGLSTQNGQLRGPLKSSTVPGGVPPQQVTQTNQQLQQLKNASTLNGTQQQAHTTSSTIKPGDDWKKSLK

LPPKDRRMKTSDVTATKGNEFEDYCLKRELLMGIFEMGWEKPSPIQEASIPIALSGRDILARAKNGTGKSGAYLIPLLER

LDLKRDYIQAMVIVPTRELALQVSQICIQVSKHMGGVKVMATTGGTNLRDDILRLDETVHVVIATPGRILDLIKKGLAKV

DNIQMIVLDEADKLLSQDFVQMMEDIISTLPRTRQILLYSATFPLSVQKFMNSHLQKPYEINLMEELTLKGVTQYYAYVT

ERQKVHCLNTLFSRLQINQSIIFCNSSQRVELLAKKISQLGYSCFYIHAKMRQEHRNRVFHDFRNGMCRNLVCTDLFTRG

IDIQAVNVVINFDFPKIAETYLHRIGRSGRFGHLGLAINLITYDDRFNLKAIEEQLGTEIKPIPGSIDKSLYVAEYHSEP

DGDDKQ

>RVE65163.1 hypothetical protein OJAV_G00132770 [Oryzias javanicus]

MSTTRTENPVILGMSTQNGQLRGSVKPAGAPGGGGGGGGPQQLNQMKGTINGNSQPSPTTNAVVKPGDDWKKSLKLPPKD

TRIKTSDVTATKGNEFEDYCLKRELLMGIFEMGWEKPSPIQEESIPIALSGRDILARAKNGTGKSGAYLIPLLERIDLKR

DCIQALGIVPTRELALQVSQICIQVSKHMGGVKVMATTGGTNLRDDIMRLDETVHVVIATPGRILDLIKKGVAKVNQVQM

IVLDEADKLLSQDFVVMMEEILGFLPKQRQILLYSATFPLSVQKFMNSHLQKPYEINLMEELTLKGVTQYYAYVTERQKV

HCLNTLFSRLQINQSIIFCNSSQRVELLAKKISQLGYSCFYIHAKMRQEHRNRVFHDFRNGLCRNLVCTDLFTRGIDIQA

VNVVINFDFPKLGETYLHRIGRSGRFGHLGLAINLITYDDRFNLKGIEEQLGTEIKPIPGIIDKSLYVAEYHSEGGEEVK

P

>XP_024132542.1 probable ATP-dependent RNA helicase ddx6 [Oryzias melastigma]

MSTTRTENPVILGMSTQNGQLRGSVKPTGAPGGGGGPQQLNQMKGTINGNSQPSPTTNAVVKPGDDWKKSLKLPPKDTRI

KTSDVTATKGNEFEDYCLKRELLMGIFEMGWEKPSPIQEESIPIALSGRDILARAKNGTGKSGAYLIPLLERIDLKRDCI

QALGIVPTRELALQVSQICIQVSKHMGGVKVMATTGGTNLRDDIMRLDETVHVVIATPGRILDLIKKGVAKVNQVQMIVL

DEADKLLSQDFVVMMEEILGFLPKQRQILLYSATFPLSVQKFMNSHLQKPYEINLMEELTLKGVTQYYAYVTERQKVHCL

NTLFSRLQINQSIIFCNSSQRVELLAKKISQLGYSCFYIHAKMRQEHRNRVFHDFRNGLCRNLVCTDLFTRGIDIQAVNV

VINFDFPKLGETYLHRIGRSGRFGHLGLAINLITYDDRFNLKGIEEQLGTEIKPIPGIIDKSLYVAEYHSEGGEEVKP

>TRY94363.1 hypothetical protein DNTS_010040 [Danionella translucida]

MSTARMENPVILGLSNQNGQMRGSVKSAGGPGGGGGLSQTTQPPQVKASSTVNNGNSQPVPTANTIIKPGDDWKKNLKLP

PKDMRMKTSDVTATKGNEFEDYCLKRELLMGIFEMGWEKPSPIQEESIPIALSGRDILARAKNGTGKSGAYLIPLLERID

LKKDSIQAVVIVPTRELALQVSQICIQVSKHMGGVKVMATTGGTNLRDDIMRLDETVHVVIATPGRILDLIKKGVAKVGQ

VQMIVLDEADKLLSQDFVQMMEEILSSLSKQRQILLYSATFPLSVQKFMNSHLQKPYEINLMEELTLKGVTQYYAYVTER

QKVHCLNTLFSRLQINQSIIFCNSSQRVELLAKKISQLGYSCFYIHAKMRQEHRNRVFHDFRNGLCRNLVCTDLFTRGID

IQAVNVVINFDFPKLGETYLHRIGRSGRFGHLGLAINLITYDDRFNLKGIEEQLGTEIKPIPSSIDKSLYVAEYHSESGE

EVKL

>XP_003968632.1 probable ATP-dependent RNA helicase DDX6 [Takifugu rubripes]

MSTARTENPVILGLSNQNGQIRGSVKPAGAPGGGGGGPQQHQVNQMKGAINNGNFQPAPTTNAVIKPGDDWKKNLKLPPK

DMRMKTSDVTATKGNEFEDYCLKRELLMGIFEMGWEKPSPIQEESIPIALSGRDILARAKNGTGKSGAYLIPLLERIDLK

RDCIQAVVIVPTRELALQVSQICIQVSKHMGGVKVMATTGGTNLRDDIMRLDETVHVIIATPGRILDLIKKGVAKVSQVQ

MIVLDEADKLLSQDFVGMMEEMLGFLSKQRQILLYSATFPLSVQKFMTSHLQKPYEINLMEELTLKGVTQYYAYVTERQK

VHCLNTLFSRLQINQSIIFCNSSQRVELLAKKISQLGYSCFYIHAKMRQEHRNRVFHDFRNGLCRNLVCTDLFTRGIDIQ

AVNVVINFDFPKLGETYLHRIGRSGRFGHLGLAINLITYDDRFNLKGIEEQLGTEIKPIPGIIDKSLYVAEYHSESGEEV

KP

>XP_028818035.1 probable ATP-dependent RNA helicase DDX6 [Denticeps clupeoides]

MSTARTENPVILGLPNQNGQIRGSVKPAGAPGGGGGGPQLQQPSQMKPSSTINGSSQPASTANTVIKPGDDWKKNLKLPP

KDMRMKTSDVTATKGNEFEDYCLKRELLMGIFEMGWEKPSPIQEESIPIALSGRDILARAKNGTGKSGAYLIPLLERIDL

KKDCIQALVVVPTRELALQVSQICIQVSKHMGGVKVMATTGGTNLRDDIMRLDETVHVVIATPGRILDLIKKGVAKVSQV

QMIVLDEADKLLSQDFVQMMEEILSFLSKQRQILLYSATFPLSVQKFMNSHLQKPYEINLMEELTLKGVTQYYAYVTERQ

KVHCLNTLFSRLQINQSIIFCNSSQRVELLAKKISQLGYSCFYIHAKMRQEHRNRVFHDFRNGLCRNLVCTDLFTRGIDI

QAVNVVINFDFPKLGETYLHRIGRSGRFGHLGLAINLITYDDRFNLKGIEEQLGTEIKPIPSSIDKSLYVAEYHSESGEE

VKP

>XP_017543428.1 PREDICTED: probable ATP-dependent RNA helicase DDX6 [Pygocentrus nattereri]

MSTARTENPVILGLSNQNGQMRGSVKPAGGPQTTQPTQIKASSTINNGNSQPVPTANTVIKPGDDWKKNLKLPPKDMRMK

TSDVTATKGNEFEDYCLKRELLMGIFEMGWEKPSPIQEESIPIALSGRDILARAKNGTGKSGAYLIPLLERIDLKKDCIQ

ALVIVPTRELALQVSQICIQVSKHMGGVKVMATTGGTNLRDDIMRLDETVHVVIATPGRILDLIKKGVAKVGQVQMIVLD

EADKLLSQDFVQMMEEILSFLPKQRQILLYSATFPLSVQKFMNAHLQKPYEINLMEELTLKGVTQYYAYVTERQKVHCLN

TLFSRLQINQSIIFCNSSQRVELLAKKISQLGYSCFYIHAKMRQEHRNRVFHDFRNGLCRNLVCTDLFTRGIDIQAVNVV

INFDFPKLGETYLHRIGRYGRFGHLGLAINLITYDDRFNLKGIEEQLGTEIKPIPSSIDKSLYVAEYHSESSEEVKP

>XP_035535828.1 probable ATP-dependent RNA helicase ddx6 [Morone saxatilis]

MSTVRTENPVILGLSNQNGQLRGSVKPAGAPGGGGGGGGGGGGPQQQQLNQMKGAINNGNSQPAPTTNAVIKPGDDWKKN

LKLPPKDMRMKTSDVTATKGNEFEDYCLKRELLMGIFEMGWEKPSPIQEESIPIALSGRDILARAKNGTGKSGAYLIPLL

ERIDLKKDCIQALVIVPTRELALQVSQICIQVSKHMGGVKVMATTGGTNLRDDIMRLDETVHVVIATPGRILDLIKKGVA

KVNQVQMIVLDEADKLLSQDFVVMMEEILGFLAKQRQILLYSATFPLSVQKFMNSHLQKPYEINLMEELTLKGVTQYYAY

VTERQKVHCLNTLFSRLQINQSIIFCNSSQRVELLAKKISQLGYSCFYIHAKMRQEHRNRVFHDFRNGLCRNLVCTDLFT

RGIDIQAVNVVINFDFPKLGETYLHRIGRSGRFGHLGLAINLITYDDRFNLKGIEEQLGTEIKPIPGIIDKSLYVAEYHS

ESGEEVKP

>XP_009917607.1 PREDICTED: probable ATP-dependent RNA helicase DDX6 [Haliaeetus albicilla]

MSTARTENPVIMGLSSQNGQLRGPVKPSGGPGGGGTQTQQQMNQLKNANTINNGTQQQAQTMTTAIKPGDDWKKTLKLPP

KDLRIKTSDVTSTKGNEFEDYCLKRELLMGIFEMGWEKPSPIQEESIPIALSGRDILARAKNGTGKSGAYLIPLLERLDL

KKDNIQAMVIVPTRELALQVSQICIQVSKHMGGAKVMATTGGTNLRDDIMRLDDTVHVVIATPGRILDLIKKGVAKVEHV

QMIVLDEADKLLSQDFVQIMEDIILTLPKNRQILLYSATFPLSVQKFMNSHLQKPYEINLMEELTLKGVTQYYAYVTERQ

KVHCLNTLFSRLQINQSIIFCNSSQRVELLAKKISQLGYSCFYIHAKMRQEHRNRVFHDFRNGLCRNLVCTDLFTRGIDI

QAVNVVINFDFPKLAETYLHRIGRSGRFGHLGLAINLITYDDRFNLKSIEEQLGTEIKPIPSNIDKSLYVAEYHSEPVED

EKQ

>XP_034556590.1 probable ATP-dependent RNA helicase ddx6 [Notolabrus celidotus]

MSTARTENPVILGLSNQNGQLRGSVKPAGAPGGGGGGGGGGGGGGGPQQQQLNQMKGAINNGNSQPAPTTNAVIKPGDDW

KKNLKLPPKDMRIKTSDVTATKGNEFEDYCLKRELLMGIFEMGWEKPSPIQEESIPIALSGRDILARAKNGTGKSGAYLI

PLLERIDLKKDCIQALVIVPTRELALQVSQICIQVSKHMGGVKVMATTGGTNLRDDIMRLDETVHVVIATPGRILDLIKK

GVAKVNQVQMIVLDEADKLLSQDFVVMMEEILGFMAKQRQILLYSATFPLSVQKFMNSHLQKPYEINLMEELTLKGVTQY

YAYVTERQKVHCLNTLFSRLQINQSIIFCNSSQRVELLAKKISQLGYSCFYIHAKMRQEHRNRVFHDFRNGLCRNLVCTD

LFTRGIDIQAVNVVINFDFPKLGETYLHRIGRSGRFGHLGLAINLITYDDRFNLKGIEEQLGTEIKPIPGIIDKSLYVAE

YHSESGEEVKP

>XP_029026001.1 probable ATP-dependent RNA helicase DDX6 [Betta splendens]

MSTARTENPVILGLSNQNGQLRGSVKPAGAPGGGGGGPQQQLNQLKGTINNGNSQPAPTTNAVIKPGDDWKKNLKLPPKD

MRMKTSDVTATKGNEFEDYCLKRELLMGIFEMGWEKPSPIQEESIPIALSGRDILARAKNGTGKSGAYLIPLLERIDLKK

DCIQALVIVPTRELALQVSQICIQVSKHMGGVKVMATTGGTNLRDDIMRLDETVHVVIATPGRILDLIKKGVAKVNQVQM

IVLDEADKLLSQDFVVMMEEILGFLSKQRQILLYSATFPLSVQKFMNAHLQKPYEINLMEELTLKGVTQYYAYVTERQKV

HCLNTLFSRLQINQSIIFCNSSQRVELLAKKISQLGYSCFYIHAKMRQEHRNRVFHDFRNGLCRNLVCTDLFTRGIDIQA

VNVVINFDFPKLGETYLHRIGRSGRFGHLGLAINLITYDDRFNLKGIEEQLGTEIKPIPGIIDKSLYVAEYHSESGEEVK

P

>XP_010738007.2 probable ATP-dependent RNA helicase DDX6 [Larimichthys crocea]

MSTARTENPVILGLSNQNGQLRGSVKPAGAPGGGGGGGGGGGGPQQQQLNQMKGAINNGSSQPAPTTNAVIKPGDDWKKN

LKLPPKDMRMKTSDVTATKGNEFEDYCLKRELLMGIFEMGWEKPSPIQEESIPIALSGRDILARAKNGTGKSGAYLIPLL

ERIDLKKDCIQALVIVPTRELALQVSQICIQVSKHMGGVKVMATTGGTNLRDDIMRLDETVHVVIATPGRILDLIKKGVA

KVNQVQMIVLDEADKLLSQDFVVMMEEILGFLAKQRQILLYSATFPLSVQKFMNAHLQKPYEINLMEELTLKGVTQYYAY

VTERQKVHCLNTLFSRLQINQSIIFCNSSQRVELLAKKISQLGYSCFYIHAKMRQEHRNRVFHDFRNGLCRNLVCTDLFT

RGIDIQAVNVVINFDFPKLGETYLHRIGRSGRFGHLGLAINLITYDDRFNLKGIEEQLGTEIKPIPGIIDKSLYVAEYHS

ESGEEVKP

>TKS80706.1 putative ATP-dependent RNA helicase DDX6 [Collichthys lucidus]

MSTARTENPVILGLSNQNGQLRGSVKPAGAPGGGGGGGGPQQQQLNQMKGAINNGSSQPAPTTNAVIKPGDDWKKNLKLP

PKDMRMKTSDVTATKGNEFEDYCLKRELLMGIFEMGWEKPSPIQEESIPIALSGRDILARAKNGTGKSGAYLIPLLERID

LKKDCIQALVIVPTRELALQVSQICIQVSKHMGGVKVMATTGGTNLRDDIMRLDETVHVVIATPGRILDLIKKGVAKVNQ

VQMIVLDEADKLLSQDFVVMMEEILGFLAKQRQILLYSATFPLSVQKFMNAHLQKPYEINLMEELTLKGVTQYYAYVTER

QKVHCLNTLFSRLQINQSIIFCNSSQRVELLAKKISQLGYSCFYIHAKMRQEHRNRVFHDFRNGLCRNLVCTDLFTRGID

IQAVNVVINFDFPKLGETYLHRIGRSGRFGHLGLAINLITYDDRFNLKGIEEQLGTEIKPIPGIIDKSLYVAEYHSESGE

EVKP

>KQK78730.1 putative ATP-dependent RNA helicase DDX6 [Amazona aestiva]

MSTARTENPVIMGLSSQNGQLRGPVXPSGGPGGGGTQTQQQMNQLKNANTINNGTQQQAQIMTTTIKPGDDWKKTLKLPP

KDLRIKTSDVTSTKGNEFEDYCLKRELLMGIFEMGWEKPSPIQEESIPIALSGRDILARAKNGTGKSGAYLIPLLERLDL

KKDNIQAMVIVPTRELALQVSQICIQVSKHMGGAKVMATTGGTNLRDDIMRLDDTVHVVIATPGRILDLIKKGVAKVEHV

QMIVLDEADKLLSXDFVQIMEDIILTLPKNRQILLYSATFPLSVQKFMNSHLQKPYEINLMEELTLKGVTQYYAYVTERQ

KVHCLNTLFSRLQINQSIIFCNSSQRVELLAKKISQLGYSCFYIHAKMRQEHRNRVFHDFRNGLCRNLVCTDLFTRGIDI

QAVNVVIXFDFPKLAETYLHRIGRSGRFGHLGLAINLITYDDRFNLKSIEEQLGTEIKPIPSNIDKSLYVAEYHSEPVED

EKQ

>XP_017271755.1 probable ATP-dependent RNA helicase DDX6 [Kryptolebias marmoratus]

MSTTRTENPVILGLSNQNGQLRGSVKPAGAPGGGGGGPQQQMNQMKGTINGSSQPAPQTNAVIKPGDDWKKNLKLPPKDM

RIRTSDVTATKGNEFEDYCLKRELLMGIFEMGWEKPSPIQEESIPIALSGRDILARAKNGTGKSGAYLIPLLERIDLKKD

CLQALVIVPTRELALQVSQICIQVSKHMGGVKVMATTGGTNLRDDIMRLDETVHVVIATPGRILDLIKKGVAKVNQVQMI

VLDEADKLLSQDFVVMMEEMLGFLSKQRQILLYSATFPLSVQKFMNAHLQKPYEINLMEELTLKGVTQYYAYVTERQKVH

CLNTLFSRLQINQSIIFCNSSQRVELLAKKISQLGYSCFYIHAKMRQEHRNRVFHDFRNGLCRNLVCTDLFTRGIDIQAV

NVVINFDFPKLGETYLHRIGRSGRFGHLGLAINLITYDDRFNLKGIEEQLGTEIKPIPGIIDKSLYVAEYHSESGEEVKQ

>XP_005735978.1 PREDICTED: probable ATP-dependent RNA helicase DDX6 [Pundamilia nyererei]

MSTARTENPVILGLPNQNGQLRGSVKPAGAPGGGGGGPQQQLNQMKGTINNGNSQPAPTTNAVIKPGDDWKKNLKLPPKD

MRMKTSDVTATKGNEFEDYCLKRELLMGIFEMGWEKPSPIQEESIPIALSGRDILARAKNGTGKSGAYLIPLLERIDLKK

DCIQALGIVPTRELALQVSQICIQVSKHMGGVKVMATTGGTNLRDDIMRLDETVHVVIATPGRILDLIKKGVAKVGQVQM

IVLDEADKLLSQDFVVMMEEILGFLPKQRQILLYSATFPLSVQKFMNSHLQKPYEINLMEELTLKGVTQYYAYVTERQKV

HCLNTLFSRLQINQSIIFCNSSQRVELLAKKISQLGYSCFYIHAKMRQEHRNRVFHDFRNGLCRNLVCTDLFTRGIDIQA

VNVVINFDFPKLGETYLHRIGRSGRFGHLGLAINLITYDDRFNLKGIEEQLGTEIKPIPGIIDKSLYVAEYHSESGEEVK

P

>XP_020784204.1 probable ATP-dependent RNA helicase DDX6 [Boleophthalmus pectinirostris]

MSTARTESPVILGLSNQNGQIRGSGKPGGGGGGGPQLNPIKGTINNGETLPATTTNAVIKPGDDWKKNLKLPPKDMRMKT

SDVTATKGNEFEDYCLKRELLMGIFEMGWEKPSPIQEESIPIALSGRDILARAKNGTGKSGAYLIPLLERIDLKKDCIQA

FVIVPTRELALQVSQICIQVSKHMGGVKVMATTGGTNLRDDIMRLDETVHVVIATPGRILDLIKKGVAKVNQVQMIVLDE

ADKLLSQDFVVMMEEILSFLPKQRQILLYSATFPLSVQKFMNSHLQKPYEINLMEELTLKGVTQYYAYVTERQKVHCLNT

LFTQLQINQSIIFCNSSQRVELLAKKISQLGYSCFYIHAKMRQEHRNRVFHDFRNGLCRNLVCTDLFTRGIDIQAVNVVI

NFDFPKLGETYLHRIGRSGRFGHLGLAINLITYDDRFNLKGIEEQLGTEIKPIPGIIDKSLYVAEYHSESHEEAKP

>XP_025913777.1 probable ATP-dependent RNA helicase DDX6 isoform X1 [Apteryx rowi]

MSTARTENPVIMGLSSQNGQLRGPVKPSGGPGGGGTQTQQQMNQLKNANTINNGTQQQAQSMTTTIKPGDDWKKTLKLPP

KDLRIKTSDVTSTKGNEFEDYCLKRELLMGIFEMGWEKPSPIQEESIPIALSGRDILARAKNGTGKSGAYLIPLLERLDL

KKDNIQAMVIVPTRELALQVSQICIQVSKHMGGAKVMATTGGTNLRDDIMRLDDTVHVVIATPGRILDLIKKGVAKVEHV

QMIVLDEADKLLSQDFVQIMEDIILTLPKNRQILLYSATFPLSVQKFMSLFQNSHLQKPYEINLMEELTLKGVTQYYAYV

TERQKVHCLNTLFSRLQINQSIIFCNSSQRVELLAKKISQLGYSCFYIHAKMRQEHRNRVFHDFRNGLCRNLVCTDLFTR

GIDIQAVNVVINFDFPKLAETYLHRIGRSGRFGHLGLAINLITYDDRFNLKSIEEQLGTEIKPIPSNIDKSLYVAEYHSE

PVEDEKP

>XP_030264660.1 probable ATP-dependent RNA helicase ddx6 [Sparus aurata]

MSTVRTENPVILGLSNQNGQLRGSVKPAGTPGGGGGGGGPQQQQLNQIKGAINGNSQPAPTTNAVIKPGDDWKKNLKLPP

KDMRMKTSDVTATKGNEFEDYCLKRELLMGIFEMGWEKPSPIQEESIPIALSGRDILARAKNGTGKSGAYLIPLLERIDL

KKDCIQALVIVPTRELALQVSQICIQVSKHMGGVKVMATTGGTNLRDDIMRLDETVHVVIATPGRILDLIKKGVAKVNQV

QMIVLDEADKLLSQDFVVMMEEILGFLAKQRQILLYSATFPLSVQKFMSAHLQKPYEINLMEELTLKGVTQYYAYVTERQ

KVHCLNTLFSRLQINQSIIFCNSSQRVELLAKKISQLGYSCFYIHAKMRQEHRNRVFHDFRNGLCRNLVCTDLFTRGIDI

QAVNVVINFDFPKLGETYLHRIGRSGRFGHLGLAINLITYDDRFNLKGIEEQLGTEIKPIPGIIDKSLYVAEYHSESGEE

VKP

>XP_033487218.1 probable ATP-dependent RNA helicase ddx6 [Epinephelus lanceolatus]

MSTARTENPLILGLSNQNGQLRGSVKPAGAPGGGGGGPQQQQLNQMKGTINNGNSQPAPTTNAVIKPGDDWKKSLKLPPK

DMRMKTSDVTATKGNEFEDYCLKRELLMGIFEMGWEKPSPIQEESIPIALSGRDILARAKNGTGKSGAYLIPLLERIDLK

RDCIQAVVIVPTRELALQVSQICIQVSKHMGGVKVMATTGGTNLRDDIMRLDETVHVIIATPGRILDLIKKGVAKVNQVQ

MIVLDEADKLLSQDFVVMMEEMLGFLPKQRQILLYSATFPLSVQKFMNAHLQKPYEINLMEELTLKGVTQYYAYVTERQK

VHCLNTLFSRLQINQSIIFCNSSQRVELLAKKISQLGYSCFYIHAKMRQEHRNRVFHDFRNGLCRNLVCTDLFTRGIDIQ

AVNVVINFDFPKLGETYLHRIGRSGRFGHLGLAINLITYDDRFNLKGIEEQLGTEIKPIPGIIDKSLYVAEYHSESGEEV

KP

>XP_028930703.1 probable ATP-dependent RNA helicase DDX6 isoform X1 [Ornithorhynchus anatinus]

MSTARTENPVIMGLSSQNGQLRGPVKPSGGPGGGGTPTQQQMNQLKNTNTINNGTQQQAQSLTTTIKFSSCRPGDDWKKT

LKLPPKDLRIKTSDVTSTKGNEFEDYCLKRELLMGIFEMGWEKPSPIQEESIPIALSGRDILARAKNGTGKSGAYLIPLL

ERLDLKKDNIQAMVIVPTRELALQVSQICIQVSKHMGGAKVMATTGGTNLRDDIMRLDDTVHVVIATPGRILDLIKKGVA

KVDHVQMIVLDEADKLLSQDFVQIMEDIILTLPKNRQILLYSATFPLSVQKFMNSHLQKPYEINLMEELTLKGVTQYYAY

VTERQKVHCLNTLFSRLQINQSIIFCNSSQRVELLAKKISQLGYSCFYIHAKMRQEHRNRVFHDFRNGLCRNLVCTDLFT

RGIDIQAVNVVINFDFPKLAETYLHRIGRSGRFGHLGLAINLITYDDRFNLKSIEEQLGTEIKPIPSNIDKSLYVAEYHS

EPVEDEKP

>XP_026143846.1 probable ATP-dependent RNA helicase ddx6 [Carassius auratus]

MSTARMENPVILGLSNQNGQMRGSVKPAGGPGGGGGVSQTTQPAQVKPSSTVNNGNSQPAPTANTIIKPGDDWKKNLKLP

PKDLRMRTSDVTATKGNEFEDYCLKRELLMGIFEMGWEKPSPIQEESIPIALSGRDILARAKNGTGKSGAYLIPLLERID

LKKDSIQALVIVPTRELALQVSQICIQVSKHMGGVKVMATTGGTNLRDDIMRLDETVHVVIATPGRILDLIKKGVAKVNQ

VQMIVLDEADKLLSQDFVQMMEEILSYLAKQRQILLYSATFPLSVQKFMNSHLQKPYEINLMEELTLKGVTQYYAYVTER

QKVHCLNTLFSRLQINQSIIFCNSSQRVELLAKKISQLGYSCFYIHAKMRQEHRNRVFHDFRNGLCRNLVCTDLFTRGID

IQAVNVVINFDFPKLGETYLHRIGRSGRFGHLGLAINLITYDDRFNLKGIEEQLGTEIKPIPSSIDKSLYVAEYHSESGE

EVKL

>XP_684923.1 probable ATP-dependent RNA helicase DDX6 [Danio rerio]

MSTARMENPVILGLSNQNGQMRGSVKPAGGPGGGGGGSQTTQPAQVKASSTVNNGNSQPVPTANTIIKPGDDWKKNLKLP

PKDLRMKTSDVTATKGNEFEDYCLKRELLMGIFEMGWEKPSPIQEESIPIALSGRDILARAKNGTGKSGAYLIPLLERID

LKKDSIQAVVIVPTRELALQVSQICIQVSKHMGGVKVMATTGGTNLRDDIMRLDETVHVVIATPGRILDLIKKGVAKVGQ

VQMIVLDEADKLLSQDFVQMMEEILSSLSKQRQILLYSATFPLSVQKFMNSHLQKPYEINLMEELTLKGVTQYYAYVTER

QKVHCLNTLFSRLQINQSIIFCNSSQRVELLAKKISQLGYSCFYIHAKMRQEHRNRVFHDFRNGLCRNLVCTDLFTRGID

IQAVNVVINFDFPKLGETYLHRIGRSGRFGHLGLAINLITYDDRFNLKGIEEQLGTEIKPIPSSIDKSLYVAEYHSESGE

EVKL

>XP_023817401.1 probable ATP-dependent RNA helicase DDX6 [Oryzias latipes]

MSTTRTENPVILGMSTQNGQLRGSVKPAGAPGGGGGGPQQLNQMKGTINGNSQPSPTTNAVVKPGDDWKKSLKLPPKDTR

IKTSDVTATKGNEFEDYCLKRELLMGIFEMGWERPSPIQEESIPIALSGRDILARAKNGTGKSGAYLIPLLERIDLKRDC

IQALGIVPTRELALQVSQICIQVSKHMGGVKVMATTGGTNLRDDIMRLDETVHVVIATPGRILDLIKKGVAKVNQVQMIV

LDEADKLLSQDFVVMMEEILGFLPKQRQILLYSATFPLSVQKFMNSHLQKPYEINLMEELTLKGVTQYYAYVTERQKVHC

LNTLFSRLQINQSIIFCNSSQRVELLAKKISQLGYSCFYIHAKMRQEHRNRVFHDFRNGLCRNLVCTDLFTRGIDIQAVN

VVINFDFPKLGETYLHRIGRSGRFGHLGLAINLITYDDRFNLKGIEEQLGTEIKPIPGIIDKSLYVAEYHSEGGEEVKP

>XP_026086989.1 probable ATP-dependent RNA helicase DDX6 [Carassius auratus]

MSTARMENPVILGLSNQNGQMRGSVKPAGGPGGGGSQTTQPAQVKPSSTVNNGNSQPAPTANTIIKPGDDWKKNLKLPPK

DLRMRTSDVTATKGNEFEDYCLKRELLMGIFEMGWEKPSPIQEESIPIALSGRDILARAKNGTGKSGAYLIPLLERIDLK

KDCIQALGIVPTRELALQVSQICIQVSKHMGGVKVMATTGGTNLRDDIMRLDETVHVVIATPGRILDLIKKGVAKVNQVQ

MIVLDEADKLLSQDFVQMMEEILSFLPKQRQILLYSATFPLSVQKFMNTHLQKPYEINLMDELTLKGVTQYYAYVTERQK

VHCLNTLFSRLQINQSIIFCNSSQRVELLAKKISQLGYSCFYIHAKMRQEHRNRVFHDFRNGLCRNLVCTDLFTRGIDIQ

AVNVVINFDFPKLGETYLHRIGRSGRFGHLGLAINLITYDDRFNLKGIEEQLGTEIKPIPSSIDKSLYVAEYHSESGEEV

KL

>OPJ68335.1 putative ATP-dependent RNA helicase DDX6 [Patagioenas fasciata monilis]

MSTARTENPVIMGLSSQNGQLRGPVKPSGGPGGGGTQTQQQMNQLKNANTINNGTQQQAQSMTTALKPGDDWKKTLKLPP

KDLRIKTSDVTSTKGNEFEDYCLKRELLMGIFEMGWEKPSPIQEESIPIALSGRDILARAKNGTGKSGAYLIPLLERLDL

KKDNIQAMVIVPTRELALQVSQICIQVSKHMGGAKVMATTGGTNLRDDIMRLDDTVHVVIATPGRILDLIKKGVAKVEHV

QMIVLDEADKLLSQDFVQIMEDIILTLPKNRQILLYSATFPLSVQKFMNSHLQKPYEINLMEELTLKGVTQYYAYVTERQ

KVHCLNTLFSRLQINQSIIFCNSSQRVELLAKKISQLGYSCFYIHAKMRQEHRNRVFHDFRNGLCRNLVCTDLFTRGIDI

QAVNVVINFDFPKLAETYLHRIGRSGRFGHLGLAINLITYDDRFNLKSIEEQLGTEIKPIPSNIDKSLKHALITHARR

>XP_014350635.1 PREDICTED: probable ATP-dependent RNA helicase DDX6 isoform X2 [Latimeria chalumnae]

MENCKPGDDWKKNLKIPPKDRRIKTSDVTSTKGNEFEDYCLKRELLMGIFEMGWEKPSPIQEESIPIALSGRDILARAKN

GTGKSGAYLIPLLERIDLKKDSIQALVIVPTRELALQVSQICIQVSKHMGGVKVMATTGGTNLRDDIMRLDDTVHVVIAT

PGRILDLIKKGVAKVDQIQMIVLDEADKLLSQDFVQIMEEIIFTLPKNRQILLYSATFPLSVQKFMGSHLQKPYEINLME

ELTLKGVTQYYAYVTERQKVHCLNTLFSRLQINQSIIFCNSSQRVELLAKKISQLGYSCFYIHAKMRQEHRNRVFHDFRN

GLCRNLVCTDLFTRGIDIQAVNVVINFDFPKLAETYLHRIGRSGRFGHLGLAINLITYDDRFNLKSIEEQLGTEIKPIPG

NIDKSLYVAEYHSEPGDGEKH

>KFP96604.1 putative ATP-dependent RNA helicase DDX6 [Haliaeetus albicilla]

MSTARTENPVIMGLSSQNGQLRGPVKPSGGPGGGGTQTQQQMNQLKNANTINNGTQQQAQTMTTAIKPGDDWKKTLKLPP

KDLRIKTSDVTSTKGNEFEDYCLKRELLMGIFEMGWEKPSPIQEESIPIALSGRDILARAKNGTGKSGAYLIPLLERLDL

KKDNIQAMVIVPTRELALQVSQICIQVSKHMGGAKVMATTGGTNLRDDIMRLDDTVHVVIATPGRILDLIKKGVAKVEHV

QMIVLDEANKLLSQDFVQIMEDIILTLPKNRQILLYSATFPLSVQKFMNSHLQKPYEINLMEELTLKGVTQYYAYVTERQ

KVHCLNTLFSRLQINQSIIFCNSSQRVELLAKKISQLGYSCFYIHAKMRQEHRNRVFHDFRNGLCRNLVCTDLFTRGIDI

QAVNVVINFDFPKLAETYLHRIGRSGRFGHLGLAINLITYDDRFNLKSIEEQLGTEIKPIPSNIDKSLYVAEYHSEPVED

EKQ

>NXM75637.1 DDX6 helicase [Serilophus lunatus]

MSTARTEHPVIMGLASPNGQLRGPAKPSGGPGGGGPPAQQHINQLKNSSTINNGTQQQQQQQAQSMTTAMKPGDDWKKTL

KLPPKDLRIKTSDVTSTKGNEFEDYCLKRELLMGIFEMGWEKPSPIQEESIPIALSGRDILARAKNGTGKSGAYLIPLLE

RLDLKKDNIQAMVIVPTRELALQVSQICIQVSKHMGGAKVMATTGGTNLRDDIMRLDDTVHVVIATPGRILDLIKKGVAK

VEHVQMIVLDEANKLLSQDFVQIMEDIILTLPKNRQILLYSATFPLSVQKFMNSHLQKPYEINLMEELTLKGVTQYYAYV

TERQKVHCLNTLFSRLQINQSIIFCNSSQRVELLAKKISQLGYSCFYIHAKMRQEHRNRVFHDFRNGLCRNLVCTDLFTR

GIDIQAVNVVINFDFPKLAETYLHRIGRSGRFGHLGLAINLITYDDRFNLKSIEEQLGTEIKPIPSNIDKSLYVAEYHSE

PVEDEKQ

>XP_034150210.1 probable ATP-dependent RNA helicase ddx6 [Esox lucius]

MSTASTQNPVILGLSKQNGQHRGQVGKPAGSPGGGGGGPQAQKPSQMKQAPSTINNGTGRGSAQLAKAAPPASTVIKPGD

DWKKNLTLPPKDMRMRTSDVTATKGNEFEDYCLKRELLMGIFEMGWEKPSPIQEESIPIALSGRDILARAKNGTGKSGAY

LIPLLERIDLKKDCIQAVGIVPTRELALQVSQICIQVSKHMGGVKVMATTGGTNLRDDIMRLDETVHVVIATPGRILDLI

KKGVAKVNHVQMIVLDEADKLLSQDFVVMMEEILSYLPKQRQILLYSATFPLSVQKFMNSHLSKPYEINLMEELTLKGVT

QYYAYVTERQKVHCLNTLFSRLQINQSIIFCNSSQRVELLAKKISQLGYSCFYIHAKMRQEHRNRVFHDFRNGLCRNLVC

TDLFTRGIDIQAVNVVINFDFPKLGETYLHRIGRSGRFGHLGLAINLITYDDRFNLKGIEEQLGTEIKPIPGSIDKSLYV

AEYHSESGEEGKP

>XP_030076314.1 probable ATP-dependent RNA helicase DDX6 [Microcaecilia unicolor]

MSTTRTENPVIMGLTSQNGQLRGPIKPSGGPGSGIQTQQQMNQLKNASTINNGTQQQAQSLPTTIKPGDDWKKTLKLPPK

DLRIKTSDVTSTKGNEFEDYCLKRELLMGIFEMGWEKPSPIQEESIPIALSGRDILARAKNGTGKSGAYLIPLLERLDLK

KDSIQAMVIVPTRELALQVSQICIQVSKHMGGVKVMATTGGTNLRDDIMRLDDTVHVVIATPGRILDLIKKGVAKVDQIQ

MIVLDEADKLLSQDFVQIMEDIILTLPKNRQILLYSATFPLSVQKFMNSHLQKPYEINLMEELTLKGVTQYYAYVTERQK

VHCLNTLFSRLQINQSIIFCNSSQRVELLAKKISQLGYSCFYIHAKMRQEHRNRVFHDFRNGLCRNLVCTDLFTRGIDIQ

AVNVVINFDFPKLAETYLHRIGRSGRFGHLGLAINLITYDDRFNLKSIEEQLGTEIKPIPSNIDKSLYVAEYHSEPVEDE

KP

>XP_030007779.1 probable ATP-dependent RNA helicase ddx6 [Sphaeramia orbicularis]

MSTAGTENPVILGLSNQNGQVICSAKTAGAPGGGGGGGGGGGGGPQKPQINQMKGTINMGNSVSAPTPNAVIRPGDDWKK

SLKLPPKDMRMKTSDVTATKGNEFEDYCLKRELLMGIFEMGWEKPSPIQEESIPIALSGRDILARAKNGTGKSGAYLIPL

LERIDLKRDCIQALVIVPTRELALQVSQICIQVSKHMGGVKVMATTGGTNLRDDIMRLDETVHVVIATPGRILDLIKKGV

AKVNQVQMIVLDEADKLLSQDFVLMMEEILGFLSKQRQILLYSATFPLSVQKFMTSHLQKPYEINLMEELTLKGVTQYYA

YVTERQKVHCLNTLFSRLQINQSIIFCNSSQRVELLAKKISQLGYSCFYIHAKMRQEHRNRVFHDFRNGLCRNLVCTDLF

TRGIDIQAVNVVINFDFPKLGETYLHRIGRSGRFGHLGLAINLITYDDRFNLKGIEEQLGTEIKPIPGIIDKSLYVAEYH

SESGEEVKP

>TMS07501.1 putative ATP-dependent RNA helicase DDX6 [Larimichthys crocea]

MKGAINNGSSQPAPTTNAVIKPGDDWKKNLKLPPKDMRMKTSDVTATKGNEFEDYCLKRELLMGIFEMGWEKPSPIQEES

IPIALSGRDILARAKNGTGKSGAYLIPLLERIDLKKDCIQALVIVPTRELALQVSQICIQVSKHMGGVKVMATTGGTNLR

DDIMRLDETVHVVIATPGRILDLIKKGVAKVNQVQMIVLDEADKLLSQDFVVMMEEILGFLAKQRQILLYSATFPLSVQK

FMNAHLQKPYEINLMEELTLKGVTQYYAYVTERQKVHCLNTLFSRLQINQSIIFCNSSQRVELLAKKISQLGYSCFYIHA

KMRQEHRNRVFHDFRNGLCRNLVCTDLFTRGIDIQAVNVVINFDFPKLGETYLHRIGRSGRFGHLGLAINLITYDDRFNL

KGIEEQLGTEIKPIPGIIDKSLYVAEYHSESGEEVKP

>EHH23489.1 hypothetical protein EGK_06964 [Macaca mulatta]

MSTARTENPVIMGLSSQNGQLRGPVKPTGGPGGGGTQTQQQMNQLKNTNTINNGTQQQAQSMTTTIKPGDDWKKTLKLPP

KDLRIKTSDVTSTKGNEFEDYCLKRELLMGIFEMGWEKPSPIQEESIPIALSGRDILARAKNGTGKSGAYLIPLLERLDL

KKDNIQAMVIVPTRELALQVSQICIQVSKHMGGAKVMATTGGTNLRDDIMRLDDTVHVVIATPGRILDLIKKGVAKVDHV

QMIVLDEADKLLSQDFVQIMEDIILTLPKNRQILLYSATFPLSVQKFMNSHLQKPYEINLMEELTLKGVTQYYAYVTERQ

KVHCLNTLFSRLQINQSIIFCNSSQRVELLAKKISQLGYSCFYIHAKMRQEHRNRVFHDFRNGLCRNLVCTDLFTRGIDI

QAVNVVINFDFPKLAETYLHRIGRSGRFGHPGLAINLITYDGRFNLKSIEEQLGTEIKPIPSNIDKSLYVAEYHSEPVED

EKP

>XP_018097502.1 PREDICTED: ATP-dependent RNA helicase ddx6-like [Xenopus laevis]

MSTARTENPVLMGMSSQNGQLRGPLKPNAGPGGGGTQTQQINQLKNASTINSGSQQQAQSMSSVIKPGDDWKKTLKLPPK

DLRIKTSDVTSTKGNEFEDYCLKRELLMGIFEMGWEKPSPIQEESIPIALSGRDILARAKNGTGKSGAYLIPLLERLDLK

KDYIQAMVIVPTRELALQVSQICIQVSKHMGGVKVMATTGGTNLRDDIMRLDDTVHVVIATPGRILDLIKKGVAKVDHIQ

MIVLDEADKLLSQDFVQIMEDIIMTLPKNRQILLYSATFPLSVQKFMSLHLQKPYEINLMEELTLKGVTQYYAYVTERQK

VHCLNTLFSRLQINQSIIFCNSSQRVELLAKKISQLGYSCFYIHAKMRQEHRNRVFHDFRNGLCRNLVCTDLFTRGIDIQ

AVNVVINFDFPKLAETYLHRIGRSGRFGHLGLAINLITYDDRFNLKSIEEQLGTEIKPIPSSIDKNLYVAEYHSESGENK

H

>NP_001128971.1 probable ATP-dependent RNA helicase DDX6 [Pongo abelii]

MSTARTENPVIMGLSSQNGQLRGPVKPTGGPGGGGTQTQQQMNQLKNTNTINNGTQQQAQSMTTTIKPGDDWKKTLKLPP

KDLRIKTSDVTSTKGNEFEDYCLKRELLMGIFEMGWEKPSPIQEESIPIALSGRDILARAKNGTGKSGAYLIPLLERLDL

KKDNIQAMVIVPTRELALQVSQICIQVSKHMGGAKVMATTGGTNLRGDIMRLDDTVHVVIATPGRILDLIKKGVAKVDHV

QMIVLDEADKLLSQDFVQIMEDIILTLPKNRQILLYSATFPLSVQKFMNSHLQKPYEINLMEELTLKGVTQYYAYVTERQ

KVHCLNTLFSRLQINQSIIFCNSSQRVELLAKKISQLGYSCFYIHAKMRQEHRNRVFHDFRNGLCRNLVCTDLFTRGIDI

QAVNVVINFDFPKLAETYLHRIGGSGRFGHLGLAINLITYDDRFNLKSIEEQLGTEIKPIPSNIDKSLYVAEYHSEPVED

EKP

>XP_035289190.1 probable ATP-dependent RNA helicase ddx6 [Anguilla anguilla]

MSTVRTENPVILGLSNQNGQLRGSVKPAVGPGGGVGGPALQQASQAKSSSTINNGSSQTMATSNAAIKPGDDWKKNLKLP

PKDMRMRTSDVTATKGNEFEDYCLKRELLMGIFEMGWEKPSPIQEESIPIALSGRDILARAKNGTGKSGAYLIPLLERID

LKKDYIQALVIVPTRELALQVSQICIQVSKHMGGVKVMATTGGTNLRDDIMRLDETVHVIIATPGRILDLIKKGVAKVNH

IQMIVLDEADKLLSQDFVQMMEETLSTMPKYRQILLYSATFPLSVQKFMNAHLQKPYEINLMEELTLKGVTQYYAYVTER

QKVHCLNTLFSRLQINQSIIFCNSSQRVELLAKKISQLGYSCFYIHAKMRQEHRNRVFHDFRNGLCRNLVSTDLFTRGID

IQAVNVVINFDFPRLAETYLHRIGRSGRFGHLGLAINLITYDDRFNLKGIEEQLGTEIKPIPGSIDKSLYVAEYHSESGE

EAKL

>TSR87292.1 putative ATP-dependent RNA helicase DDX6 [Bagarius yarrelli]

MSTARTENPVILGLTNQNGQKRGPAKPTGGPGGGGGGPQSTQPTQIKASSTVNNGNPLPVPTANTVIKPGDDWKKNLKLP

PKDMRMKTSDVTATKGNEFEDYCLKRELLMGIFEMGWEKPSPIQEESIPIALSGRDILARAKNGTGKSGAYLIPLLERID

LKKDCIQALVIVPTRELALQVSQICIQVSKHMGGVKVMATTGGTNLRDDIMRLDETVHVVIATPGRVLDLIKKGVAKVGQ

VQMIVLDEADKLLSQDFVQMMEEILSFLHKQRQILLYSATFPLSVQKFMNSHLQKPYEINLMEELTLKGVTQYYAYVTER

QKVHCLNTLFSRLQINQSIIFCNSSQRVELLAKKISQLGYSCFYIHAKMRQEHRNRVFHDFRNGLCRNLVCTDLFTRGID

IQAVNVVINFDFPKLGETYLHRIGRSGRFGHLGLAINLITYDDRFNLKGIEEQLGTEIKPIPSSIDKSLYVAEYHSESTE

EVKP

>XP_008148317.1 probable ATP-dependent RNA helicase DDX6 isoform X1 [Eptesicus fuscus]

MRASYCCLLFRDLQVLACIAIKDSILSKTSIYLFILESLINCITVSLLIFKSVEVWKRHLCVSYSMSTARTENPVIMGLS

SQNGQLRGPVKPSGGPGGGGTPTQQQMNQLKNSTTINNGTPQQAPSVAAAIKPGDDWKKTLKLPPKDLRIKTSDVTSTKG

NEFEDYCLKRELLMGIFEMGWEKPSPIQEESIPIALSGRDILARAKNGTGKSGAYLIPLLERLDLKKDNIQAMVIVPTRE

LALQVSQICIQVSKHMGGAKVMATTGGTNLRDDIMRLDDTVHVVIATPGRILDLIKKGVAKVDHVQMIVLDEADKLLSQD

FVQIMEDIILTLPKNRQILLYSATFPLSVQKFMNSHLQKPYEINLMEELTLKGVTQYYAYVTERQKVHCLNTLFSRLQIN

QSIIFCNSSQRVELLAKKISQLGYSCFYIHAKMRQEHRNRVFHDFRNGLCRNLVCTDLFTRGIDIQAVNVVINFDFPKLA

ETYLHRIGRSGRFGHLGLAINLITYDDRFNLKSIEEQLGTEIKPIPSNIDKSLYVAEYHSEPVEDDKP

>XP_034346569.1 probable ATP-dependent RNA helicase DDX6 [Arvicanthis niloticus]

MSTARTENPVIMGLSSQNGQLRGPVKASAGPGGGGTQPQPQMNQLKNSSTLNNGTPQQAQSVAATIKPGDDWKKTLKLPP

KDLRIKTSDVTSTKGNEFEDYCLKRELLMGIFEMGWEKPSPIQEESIPIALSGRDILARAKNGTGKSGAYLIPLLERLDL

KKDNIQAMVIVPTRELALQVSQICIQVSKHMGGAKVMATTGGTNLRDDIMRLDDTVHVVIATPGRILDLIKKGVAKVDHV

QMIVLDEADKLLSQDFVQIMEDIILTLPKNRQILLYSATFPLSVQKFMNCHLQKPYEINLMEELTLKGVTQYYAYVTERQ

KVHCLNTLFSRLQINQSIIFCNSSQRVELLAKKISQLGYSCFYIHAKMRQEHRNRVFHDFRNGLCRNLVCTDLFTRGIDI

QAVNVVINFDFPKLAETYLHRIGRSGRFGHLGLAINLITYDDRFNLKSIEEQLGTEIKPIPSNIDKSLYVAEYHSEPAED

EKP

>KAF7655771.1 hypothetical protein LDENG_00051120 [Lucifuga dentata]

MSTVRTENPVILGLSNQNGQLRGSVKPAGGPGGGGGGGPQQQLSQIKGTINNGSPQPVPTTNAVIKPGDDWKKNLKLPPK

DMRMKTSDVTATKGNEFEDYCLKRELLMGIFEMGWEKPSPIQEESIPIALSGRDILARAKNGTGKSGAYLIPLLERIDLK

RDCIQAVGIVPTRELALQVSQICIQVSKHMGGVKVMATTGGTNLRDDIMRLDETVHVVIATPGRILDLIKKGVAKVNQVQ

MIVLDEADKLLSQDFVVMMEEILGFLPKQRQILLYSATFPLSVQKFMNSHLQKPYEINLMEELTLKGVTQYYAYVTERQK

VHCLNTLFSRLQINQSIIFCNSSQRVELLAKKISQLGYSCFYIHAKMRQEHRNRVFHDFRNGLCRNLVCTDLFTRGIDIQ

AVNVVINFDFPKLGETYLHRIGRSGRFGHLGLAINLITYDDRFNLKGIEEQLGTEIKPIPGIIDKSLYVAEYHSESSEEV

KL

>XP_031719467.1 probable ATP-dependent RNA helicase DDX6 [Anarrhichthys ocellatus]

MSTARTEHPVILGLSNQNGQLRGSVKPAGAPGGSGGGPQQQQLNQMKGTINNGNSQLAPATNSVIKPGDDWKKSLKLPPK

DTRMRTSDVTATKGNEFEDYCLKRELLMGIFEMGWEKPSPIQEESIPIALSGRDILARAKNGTGKSGAYLIPLLERIDLK

RDFIQAVVIVPTRELALQVSQICIQVSKHMGGVKVMATTGGTNLRDDIMRLDETVHVIIATPGRILDLIKKGVAKVNQVQ

MVVLDEADKLLSQDFVLMMEDMLSFLPKQRQILLYSATFPISVQKFMNAHLQKPYEINLMEELTLKGVTQYYAYVTERQK

VHCLNTLFSRLQINQSIIFCNSSQRVELLAKKISQLGYSCFYIHAKMRQEHRNRVFHDFRNGLCRNLVCTDLFTRGIDIQ

AVNVVINFDFPKLGETYLHRIGRSGRFGHLGLAINLITYDDRFNLKGIEEQLGTEIKPIPGIIDKSLYVAEYHSESGEEV

KP

>NP_001166415.1 probable ATP-dependent RNA helicase DDX6 [Cavia porcellus]

MGLSSQNGQLRGPVKPSGGPGGGGTQTQQQMNQLKNTNTINNGTQQQAQSMTTTIKPGDDWKKTLKLPPKDLRIKTSDVT

STKGNEFEDYCLKRELLMGIFEMGWEKPSPIQEESIPIALTGRDILARAKNGTGKSGAYLIPLLERLDLKKDNIQAMVIV

PTRELALQVSQICIQVSKHMGGAKVMATTGGTNLRDDIMRLDDTVHVVIATPGRILDLIKKGVAKVDHVQMIVLDEADKL

LSQDFVQIMEDIILTLPKNRQILLYSATFPLSVQKFMNSHLQKPYEINLMEELTLKGVTQYYAYVTERQKVHCLNTLFPR

LQTNQSIIFCNSSQRVELLAKKISQLGYSCFYIHAKMRQEHRNRVFHDFRNGLCRNLVCTDLFTRGIDIQAVNVVINFDF

PKLAETYLHRIGRSGRFGHLGLAINLITYDDRFNLKSIEEQLGTEIKPIPSNIDKSLYVAEYHSEPVEDEKP

>XP_014071054.1 PREDICTED: probable ATP-dependent RNA helicase DDX6 [Salmo salar]

MSTTSTQNPVILGHSNQNGQLRGPVVKPAGGQGSGGGSHQTQQPGQMKQASSTINNGTGSSSTQLAKANPTANTVIKPGD

DWKRNLTLPPKDMRMRTSDVTATKGNEFEDYCLKRELLMGIFEMGWEKPSPIQEESIPIALSGRDILARAKNGTGKSGAY

LIPLLERIDLKKDCIQAMGIVPTRELALQVSQICIQISKHMGGVKVMATTGGTNLRDDIMRLDETVHVVIATPGRILDLI

KKGVAKVNQVQMMVLDEADKLLSQDFVVMMEEVLSYLPRQRQILLYSATFPLSVQKFMNSHLSKPYEINLMEELTLKGVT

QYYAYVTERQKVHCLNTLFSRLQINQSIIFCNSSQRVELLAKKISQLGYSCFYIHAKMRQEHRNRVFHDFRNGLCRNLVC

TDLFTRGIDIQAVNVVINFDFPKLGETYLHRIGRSGRFGHLGLAINLITYDDRFNLKGIEEQLGTEIRPIPGSIDKSLYV

AEYHSENGEEVKL

>XP_033375559.1 probable ATP-dependent RNA helicase DDX6 isoform X2 [Parus major]

MYVRTWDVWTGAVPPAGRGRAHGRKEEAAILERRVAITRHRGSERILYLGEGERGWWRPGDDWKKTLKLPPKDLRIKTSD

VTSTKGNEFEDYCLKRELLMGIFEMGWEKPSPIQEESIPIALSGRDILARAKNGTGKSGAYLIPLLERLDLKKDNIQAMV

IVPTRELALQVSQICIQVSKHMGGAKVMATTGGTNLRDDIMRLDDTVHVVIATPGRILDLIKKGVAKVEHVQMIVLDEAD

KLLSQDFVQIMEDIILTLPKNRQILLYSATFPLSVQKFMNSHLQKPYEINLMEELTLKGVTQYYAYVTERQKVHCLNTLF

SRLQINQSIIFCNSSQRVELLAKKISQLGYSCFYIHAKMRQEHRNRVFHDFRNGLCRNLVCTDLFTRGIDIQAVNVVINF

DFPKLAETYLHRIGRSGRFGHLGLAINLITYDDRFNLKSIEEQLGTEIKPIPSNIDKSLYVAEYHSEPVEDEKQ

>XP_029629801.1 probable ATP-dependent RNA helicase DDX6 [Salmo trutta]

MSTASTQNPVILGHSNQNGQLRGPVGKPAGGQGSGGGSHQTQQPGQMKQASSTINNGTGPSSTQLAKANPTANTVIKPGD

DWKRNLTLPPKDMRMRTSDVTATKGNEFEDYCLKRELLMGIFEMGWEKPSPIQEESIPIALSGRDILARAKNGTGKSGAY

LIPLLERIDLKKDCIQAMGIVPTRELALQVSQICIQISKHMGGVKVMATTGGTNLRDDIMRLDETVHVVIATPGRILDLI

KKGVAKVNQVQMMVLDEADKLLSQDFVVMMEEVLSYLPKQRQILLYSATFPLSVQKFMNSHLSKPYEINLMEELTLKGVT

QYYAYVTERQKVHCLNTLFSRLQINQSIIFCNSSQRVELLAKKISQLGYSCFYIHAKMRQEHRNRVFHDFRNGLCRNLVC

TDLFTRGIDIQAVNVVINFDFPKLGETYLHRIGRSGRFGHLGLAINLITYDDRFNLKGIEEQLGTEIRPIPGSIDKSLYV

AEYHSENGEEVKL

>XP_019742923.1 PREDICTED: probable ATP-dependent RNA helicase DDX6 [Hippocampus comes]

MSTARTENPVILGLSNQNGQLRGSVKPAGAPGGGGGGGGGGGPQQPLNQMKGTINNGNAQTAPTTNAVIKPGDDWKKNLK

LPPKDMRMRTSDVTATKGNEFEDYCLKRELLMGIFEMGWEKPSPIQEESIPIALSGRDILARAKNGTGKSGAYLIPLLER

IDLKRDYIQALVIVPTRELALQVSQICIQVSKHMGGVKVMATTGGTNLRDDIMRLDETVHVVIATPGRILDLIKKGVAKV

SQVQMIVLDEADKLLSQDFVVMMEEILGFLSKQRQILLYSATFPLSVQKFMNSHLQKPYEINLMEELTLKGVTQYYAYVT

ERQKVHCLNTLFSRLQINQSIIFCNSSQRVELLAKKISQLGYSCFYIHAKMRQEHRNRVFHDFRNGLCRNLVCTDLFTRG

IDIQAVNVVINFDFPKLGETYLHRIGRSGRFGHLGLAINLITYDDRFNLKGIEEQLGTEIKPIPGIIDKSLYVAEYHSET

GEEVKP

>KAF5907834.1 putative ATP-dependent RNA helicase DDX6, partial [Clarias magur]

MSTARTENPVILGLTNQNGQKRGSTKPTGGPGGGPQTTQPAQIKASSAVNNGNSLPVPTANTVIKPGDDWKKNLKLPPKD

MRMKTADVTATKGNEFEDYCLKRELLMGIFEMGWEKPSPIQEESIPIALSGRDILARAKNGTGKSGAYLIPLLERIDLKK

DCIQALVIVPTRELALQVSQICIQVSKHMGGVKVMATTGGTNLRDDIMRLDETVHVVIATPGRVLDLIKKGVAKVGQVQM

IVLDEADKLLSQDFVQMMEEILSCLSKQRQILLYSATFPLSVQKFMNSHLQKPYEINLMEELTLKGVTQYYAYVTERQKV

HCLNTLFSRLQINQSIIFCNSSQRVELLAKKISQLGYSCFYIHAKMRQEHRNRVFHDFRNGLCRNLVCTDLFTRGIDIQA

VNVVINFDFPKLGETYLHRIGRSGRFGHLGLAINLITYDDRFNLKGIEEQLGTEIKPIPSSIDKSLYVAEYHSESAEEVK

LEIYCTGELLRQVQMAKLFDDNKVFVDMKLTAEPDIVLDAFSKLTQRFPNGTVPPSDIQVFVNTYFVNSGKEFEPWSPPD

WHDKPKLLAKISDLKLRSWAEELHGLWKSLGRKVTNDVRDNPQMYSLIYCPYPGIVPGGRFTEFYYWPESYTHDAELVEG

LPAEAQEKLWTELKAAAESGWDFSSRWYINNLDNSDSFRDTRASYIVPVDLNALICRNEHVLANFHRILGNEERALVYDN

AVSVRLKAIESVLWDSKKGAWFDYNLLNRTRNYAFYPTNLSPLWARCFSQPEMGHQALQYLRSGQQWDMPNAWPPLQHML

IEGLSQLDSVDSQDLATDLAQKWIHTNWLAYVKYDGMFEKYDVSGDGKPGGGGEYKVQLGFGWTNGVALQLLDQYGDKL

>XP_008307229.1 probable ATP-dependent RNA helicase DDX6 [Cynoglossus semilaevis]

MSTARTENPVILGLSNQNGQLRGSVKPAGAPGGGGGGPQQQQLNQIKGTINNGNPQQAPTTNAVIKPGDDWKKSLKLPPK

DMRMKTSDVTATKGNEFEDYCLKRELLMGIFEMGWEKPSPIQEESIPIALSGRDILARAKNGTGKSGAYLIPLLERIDLK

RDCIQAVVIVPTRELALQVSQICIQVSKHMGGVKVMATTGGTNLRDDIMRLDETVHVIIATPGRILDLIKKGVAKVNQVQ

MFVLDEADKLLSQDFVVMMEELLGFLSKQRQILLYSATFPLSVQKFMNSHLQKPYEINLMEELTLKGVTQYYAYVSERQK

VHCLNTLFSRLQINQSIIFCNSSQRVELLAKKISQLGYSCFYIHAKMRQEHRNRVFHDFRNGLCRNLVCTDLFTRGIDIQ

AVNVVINFDFPKLGETYLHRIGRSGRFGHLGLAINLITYDDRFNLKGIEEQLGTEIKPIPGIIDKSLYVAEYHSESGEEV

KP

>XP_016093146.1 PREDICTED: probable ATP-dependent RNA helicase DDX6 [Sinocyclocheilus grahami]

MSTARMENPVILGLSNQNGQMRGSVKPVGGPGGGGGGSQTTQPAQVKPSSTVNNGNSQPAPTANTIIKPGDDWKKNLKLP

PKDLRMRTSDVTATKGNEFEDYCLKRELLMGIFEMGWEKPSPIQEESIPIALSGRDILARAKNGTGKSGAYLIPLLERID

LKKDSIQALVIVPTRELALQVSQICIQVSKHMGGVKVMATTGGTNLRDDILRLDETVHVVIATPGRILDLIKKGVAKVNQ

VQMIVLDEADKLLSQDFVQMMEELLSYLHKQRQILLYSATFPLSVQKFMNAHLQKPYEINLMEELTLKGVTQYYAYVTER

QKVHCLNTLFSRLQINQSIIFCNSSQRVELLAKKISQLGYSCFYIHAKMRQEHRNRVFHDFRNGLCRNLVCTDLFTRGID

IQAVNVVINFDFPKLGETYLHRIGRSGRFGHLGLAINLITYDDRFNLKGIEEQLGTEIKPIPSSIDKSLYVAEYHSESGE

EVKL

>XP_030236243.1 probable ATP-dependent RNA helicase DDX6 [Gadus morhua]

MSAARTENPIILGLSNQNGQMRGSVKPAGGPGGGGGGGSQHQPSQIKTSCTINNGSSQPTTTANTVIKPGDDWKKNLKLP

PKDTRMRTSDVTATKGNEFEDYCLKRELLMGIFEMGWEKPSPIQEESIPIALSGRDILARAKNGTGKSGAYLIPLLERID

LKRDCIQAVGIVPTRELALQVSQICIQVGKHMGGVKVMATTGGTNLRDDIMRLDETVHVIIATPGRILDLIKKGVAKVNQ

VQMVVLDEADKLLSQDFMAMMEEMLSFLPKQRQILLYSATFPLSVQKFMNAYLQKPYEINLMEELTLKGVTQYYAYVTER

QKVHCLNTLFSRLQINQSIIFCNSSQRVELLAKKISQLGYSCFYIHAKMRQEHRNRVFHDFRNGLCRNLVCTDLFTRGID

IQAVNVVINFDFPKLGETYLHRIGRSGRFGHLGLAINLITYDDRFNLKGIEEQLGTEIKPIPGIIDKSLYVAEYHSEGGE

EGKL

>XP_032361912.1 probable ATP-dependent RNA helicase DDX6 [Etheostoma spectabile]

MSTARTENPVILGLSNQNGQLRGPVKPAGAPGGGGGPLLQQLNQMKGTINNGNSQPAPTANAVIKPGDDWKKSLKLPPKD

MRMKTSDVTATKGNEFEDYCLKRELLMGIFEMGWEKPSPIQEESIPIALSGRDILARAKNGTGKSGAYLIPLLERIDLKR

DCIQAVVIVPTRELALQVSQICIQVSKHMGGVKVMATTGGTNLRDDIMRLDETVHVIIATPGRILDLIKKGVAKVNQVQM

VVLDEADKLLSQDFVLMMEEMLGFLAKQRQILLYSATFPLSVQKFMNAHLQKPYEINLMEELTLKGVTQYYAYVTERQKV

HCLNTLFSRLQINQSIIFCNSSQRVELLAKKISQLGYSCFYIHAKMRQEHRNRVFHDFRNGLCRNLVCTDLFTRGIDIQA

VNVVINFDFPKLGETYLHRIGRSGRFGHLGLAINLITYDDRFNLKGIEEQLGTEIKPIPGIIDKSLYVAEYHSESGEELK

P

>XP_033968890.1 probable ATP-dependent RNA helicase ddx6 [Trematomus bernacchii]

MSTARTENPMILGLSNQNGQLRGSVKTAGAPGGGVGGPQQQLNQMKGTINNGNSLPAPTTNAVIKLGDDWKKNLKLPPKD

TRMRTSDVTATKGNEFEDYCLKRELLMGIFEMGWEKPSPIQEESIPIALSGRDILARAKNGTGKSGAYLIPLLERIDLKR

DCIQAMVIVPTRELALQVSQISIQVSKHMGGVKVMATTGGTNLRDDIMRLDETVHVVIATPGRILDLIKKGVAKVNQVHM

VVLDEADKLLSQDFVSMMEEMLGFLAKQRQILLYSATFPLSVQKFMNAHLQKPYEINLMEELTLKGVTQYYAYVTERQKV

HCLNTLFSRLQINQSIIFCNSSQRVELLAKKISQLGYSCFYIHAKMRQEHRNRVFHDFRNGLCRNLVCTDLFTRGIDIQA

VNVVINFDFPKLGETYLHRIGRSGRFGHLGLAINLITYDDRFNLKGIEEQLGTEIKPIPGIIDKSLYVAEYHSESGEEIK

P

>XP_028428866.1 probable ATP-dependent RNA helicase DDX6 [Perca flavescens]

MSTTRTENPVILGLSNQNGQLRGPVKPAGAPGGGGGGPLLQQLNQMKGTINNGNSQPAPTTNAVIKPGDDWKKNLKLPPK

DMRMKTSDVTATKGNEFEDYCLKRELLMGIFEMGWEKPSPIQEESIPIALSGRDILARAKNGTGKSGAYLIPLLERIDLK

RDCIQAVVIVPTRELALQVSQICIQVSKHMGGVKVMATTGGTNLRDDIMRLDETVHVIIATPGRILDLIKKGVAKVNQVQ

MVVLDEADKLLSQDFVLMMEEMLGFLAKQRQILLYSATFPLSVQKFMNAHLQKPYEINLMEELTLKGVTQYYAYVTERQK

VHCLNTLFSRLQINQSIIFCNSSQRVELLAKKISQLGYSCFYIHAKMRQEHRNRVFHDFRNGLCRNLVCTDLFTRGIDIQ

AVNVVINFDFPKLGETYLHRIGRSGRFGHLGLAINLITYDDRFNLKGIEEQLGTEIKPIPGIIDKSLYVAEYHSESGEEL

KP

>XP_033952683.1 probable ATP-dependent RNA helicase ddx6 [Pseudochaenichthys georgianus]

MNTARTENPMILGLSNQNGQLRGSVKTAGAPGGGVGGPQQQLNQMKGTINNGNSLPAPTTNAVIKLGDDWKKNLKLPPKD

TRMRTSDVTATKGNEFEDYCLKRELLMGIFEMGWEKPSPIQEESIPIALSGRDILARAKNGTGKSGAYLIPLLERIDLKR

DCIQAMVIVPTRELALQVSQISIQVSKHMGGVKVMATTGGTNLRDDIMRLDETVHVVIATPGRILDLIKKGVAKVNQVHM

VVLDEADKLLSQDFVSMMEEMLGFLAKQRQILLYSATFPLSVQKFMNAHLQKPYEINLMEELTLKGVTQYYAYVTERQKV

HCLNTLFSRLQINQSIIFCNSSQRVELLAKKISQLGYSCFYIHAKMRQEHRNRVFHDFRNGLCRNLVCTDLFTRGIDIQA

VNVVINFDFPKLGETYLHRIGRSGRFGHLGLAINLITYDDRFNLKGIEEQLGTEIKPIPGIIDKSLYVAEYHSDSGEEIK

P

>XP_016346150.1 PREDICTED: probable ATP-dependent RNA helicase ddx6 [Sinocyclocheilus anshuiensis]

MSTARMENPVILGLSNQNGQMRGSVKPAGGPGGGGGGSQTTQPAQVKPSSTVNNGNSQPAPTANTIIKPGDDWKKNLKLP

PKDLRMRTSDVTATKGNEFEDYCLKRELLMGIFEMGWEKPSPIQEESIPIALSGRDILARAKNGTGKSGAYLIPLLERID

LKKDSIQALVIVPTRELALQVSQICIQVSKHMGGVKVMATTGGTNLRDDILRLDETVHVVIATPGRILDLIKKGVAKVSQ

VQMIVLDEADKLLSQDFVQMMEELLSYLHKQRQILLYSATFPLSVQKFMNAHLQKPYEINLMEELTLKGVTQYYAYVTER

QKVHCLNTLFSRLQINQSIIFCNSSQRVELLAKKISQLGYSCFYIHAKMRQEHRNRVFHDFRNGLCRNLVCTDLFTRGID

IQAVNVVINFDFPKLGETYLHRIGRSGRFGHLGLAINLITYDDRFNLKGIEEQLGTEIKPIPSSIDKSLYVAEYHSESGE

EVKP

>XP_010771038.1 PREDICTED: probable ATP-dependent RNA helicase DDX6 [Notothenia coriiceps]

MSTARTENPMILGLSNQNGQLRGSVKTAGAPGGGVGGPQQQLIQMKGTINNGNSLPAPTTNAVIKLGDDWKKNLKLPPKD

TRMRTSDVTATKGNEFEDYCLKRELLMGIFEMGWEKPSPIQEESIPIALSGRDILARAKNGTGKSGAYLIPLLERIDLKR

DCIQAMVIVPTRELALQVSQISIQVSKHMGGVKVMATTGGTNLRDDIMRLDETVHVVIATPGRILDLIKKGVAKVNQVHM

VVLDEADKLLSQDFVSMMEEMLGFLAKQRQILLYSATFPLSVQKFMNAHLQKPYEINLMEELTLKGVTQYYAYVTERQKV

HCLNTLFSRLQINQSIIFCNSSQRVELLAKKISQLGYSCFYIHAKMRQEHRNRVFHDFRNGLCRNLVCTDLFTRGIDIQA

VNVVINFDFPKLGETYLHRIGRSGRFGHLGLAINLITYDDRFNLKGIEEQLGTEIKPIPGIIDKSLYVAEYHSESGEEIK

P

>XP_034722464.1 probable ATP-dependent RNA helicase ddx6 [Etheostoma cragini]

MSTARTENPVILGLSNQNGQLRGPVKPAGAPGGGGGGPLLQQLNQMKGTINNGNSQPAPTANAVIKPGDDWKKNLKLPPK

DMRMKTSDVTATKGNEFEDYCLKRELLMGIFEMGWEKPSPIQEESIPIALSGRDILARAKNGTGKSGAYLIPLLERIDLK

RDCIQAVVIVPTRELALQVSQICIQVSKHMGGVKVMATTGGTNLRDDIMRLDETVHVIIATPGRILDLIKKGVAKVNQVQ

MVVLDEADKLLSQDFVLMMEEMLGFLAKQRQILLYSATFPLSVQKFMNAHLQKPYEINLMEELTLKGVTQYYAYVTERQK

VHCLNTLFSRLQINQSIIFCNSSQRVELLAKKISQLGYSCFYIHAKMRQEHRNRVFHDFRNGLCRNLVCTDLFTRGIDIQ

AVNVVINFDFPKLGETYLHRIGRSGRFGHLGLAINLITYDDRFNLKGIEEQLGTEIKPIPGIIDKSLYVAEYHSESGEEL

KP

>XP_034405219.1 probable ATP-dependent RNA helicase DDX6 [Cyclopterus lumpus]

MSTARTETPVILGLSNQNGQLRGSVKPAGAPGGGGGGPQQQLNQMKGTINNGNAQLAPTTNAVIKPGDDWKKSLKLPPKD

TRMKTSDVTSTKGNEFEDYCLKRELLMGIFEMGWEKPSPIQEESIPIALSGRDILARAKNGTGKSGAYLIPLLERIDLKR

DCIQAMVIVPTRELALQVSQISIQVSKHMGGVKVMATTGGTNLRDDIMRLDETVHVIIATPGRILDLIKKGVAKVNQVQM

VVLDEADKLLSQDFVVMMEEMLGFLPKRRQILLYSATFPISVQKFMNAHLQKPYEINLMEELTLKGVTQYYAYVTERQKV

HCLNTLFSRLQINQSIIFCNSSQRVELLAKKISQLGYSCFYIHAKMRQEHRNRVFHDFRNGLCRNLVCTDLFTRGIDIQA

VNVVINFDFPKLGETYLHRIGRSGRFGHLGLAINLITYDDRFNLKGIEEQLRTEIKPIPGIIDKSLYVAEYHSESGEEVK

P

>XP_031142827.1 probable ATP-dependent RNA helicase ddx6 [Sander lucioperca]

MSTARTENPVILGLSNQNGQLRGPVKPAGAPGGGGGPLLQQLNQMKGTINNGNSPPVPTTNAVIKPGDDWKKNLKLPPKD

MRMKTSDVTATKGNEFEDYCLKRELLMGIFEMGWEKPSPIQEESIPIALSGRDILARAKNGTGKSGAYLIPLLERIDLKR

DCIQAVVIVPTRELALQVSQICIQVSKHMGGVKVMATTGGTNLRDDIMRLDETVHVIIATPGRILDLIKKGVAKVNQVQM

VVLDEADKLLSQDFVLMMEEMLGFLAKQRQILLYSATFPLSVQKFMNAHLQKPYEINLMEELTLKGVTQYYAYVTERQKV

HCLNTLFSRLQINQSIIFCNSSQRVELLAKKISQLGYSCFYIHAKMRQEHRNRVFHDFRNGLCRNLVCTDLFTRGIDIQA

VNVVINFDFPKLGETYLHRIGRSGRFGHLGLAINLITYDDRFNLKGIEEQLGTEIKPIPGIIDKSLYVAEYHSESGEELK

P

>NXE56827.1 DDX6 helicase [Casuarius casuarius]

MSTARTENPVIMGLSSQNGQLRGPVKPSGGPGGGGTQTQQQMNQLKNANTINNGTQQQAQSMTTTIKPGDDWKKTLKLPP

KDLRIKTSDVTSTKGNEFEDYCLKRELLMGIFEMGWEKPSPIQEESIPIALSGRDILARAKNGTGKSGAYLIPLLERLDL

KKDNIQAMVIVPTRELALQVSQICIQVSKHMGGAKVMATTGGTNLRDDIMRLDDTVHVVIATPGRILDLIKKGVAKVEHV

QMIVLDEATCTINLIYFLFLLLQADKLLSQDFVQIMEDIILTLPKNRQILLYSATFPLSVQKFMNSHLQKPYEINLMEEL

TLKGVTQYYAYVTERQKVHCLNTLFSRLQINQSIIFCNSSQRVELLAKKISQLGYSCFYIHAKMRQEHRNRVFHDFRNGL

CRNLVCTDLFTRGIDIQAVNVVINFDFPKLAETYLHRIGRSGRFGHLGLAINLITYDDRFNLKSIEEQLGTEIKPIPSNI

DKSLYVAEYHSEPVEDEKP

>XP_028320664.1 probable ATP-dependent RNA helicase DDX6 [Gouania willdenowi]

MSTARTENPVIMGLPSQNGQLRGSVKPAGAPGGGGGPQQPAFNPIKGTINNGNSQPAPSTNAVIKPGDDWKKNLKLPPKD

MRMKTSDVTATKGNEFEDYCLKRELLMGIFEMGWEKPSPIQEESIPIALSGRDILARAKNGTGKSGAYLIPLLERIDLKR

DCIQALGVVPTRELALQVSQICIQVSKHMGGVKVMATTGGTNLRDDIMRLDETVHVVIATPGRILDLIKKGVAKVNQVQM

VVLDEADKLLSQDFVLMMEEMLGFLPKQRQILLYSATFPLSVQKFMNSHLQKPYEINLMEELTLKGVTQYYAYVTERQKV

HCLNTLFSRLQINQSIIFCNSSQRVELLAKKISQLGYSCFYIHAKMRQEHRNRVFHDFRNGLCRNLVSTDLFTRGIDIQA

VNVVINFDFPKLGETYLHRIGRSGRFGHLGLAINLITYDDRFNLKGIEEQLGTEIKPIPGIIDKSLYVAEYHSESGEEVK

P

>XP_035479892.1 probable ATP-dependent RNA helicase ddx6 [Scophthalmus maximus]

MSTVRTENPVILGLSNQNGQLRGSVKPAGAPGGGGGGPQQQLLSQMKGTINNGNSQPAPTTNAVIKPGDDWKKNLKLPPK

DMRMKTSDVTATKGNEFEDYCLKRELLMGIFEMGWEKPSPIQEESIPIALSGRDILARAKNGTGKSGAYLIPLLERIDLK

KDCIQAMVIVPTRELALQVSQICIQVSKRMGGVKVMATTGGTNLRDDILRLDETVHVVIATPGRILDLIKKGVAKVNQVQ

MVVLDEADKLLSQDFVLMMEEMLGFLAKQRQILLYSATFPLSVQKFMNSHLQKPYEINLMEELTLKGVTQYYAYVTERQK

VHCLNTLFSRLQINQSIIFCNSSQRVELLAKKISQLGYSCFYIHAKMRQEHRNRVFHDFRNGLCRNLVCTDLFTRGIDIQ

AVNVVINFDFPKLGETYLHRIGRSGRFGHLGLAINLITYDDRFNLKGIEEQLGTEIKPIPGIIDKSLYVAEYHSESGEEV

KP

>XP_034078924.1 probable ATP-dependent RNA helicase DDX6 [Gymnodraco acuticeps]

MSTARTENPMILGLSNQNGQLRGSVKTAGAPGGGVGGPQQQLNQMKGTINNGNSLPAPTTKLGDDWKKNLKLPPKDTRMR

TSDVTATKGNEFEDYCLKRELLMGIFEMGWEKPSPIQEESIPIALSGRDILARAKNGTGKSGAYLIPLLERIDLKRDCIQ

AMVIVPTRELALQVSQISIQVSKHMGGVKVMATTGGTNLRDDIMRLDETVHVVIATPGRILDLIKKGVAKVNQVHMVVLD

EADKLLSQDFVSMMEEMLGFLAKRRQILLYSATFPLSVQKFMNAHLQKPYEINLMEELTLKGVTQYYAYVTERQKVHCLN

TLFSRLQINQSIIFCNSSQRVELLAKKISQLGYSCFYIHAKMRQEHRNRVFHDFRNGLCRNLVCTDLFTRGIDIQAVNVV

INFDFPKLGETYLHRIGRSGRFGHLGLAINLITYDDRFNLKGIEEQLGTEIKPIPGIIDKSLYVAEYHSESGEEIKP

>PWA28042.1 hypothetical protein CCH79_00012078 [Gambusia affinis]

MSTTRTENPVILGLSNQNGQLRSSVKPGGAPGGGGGGPQPPQLNQMIKGAINGNSQSAPPTNAVIKPGDDWKKNLKLPPK

DMRMKTSDVTATKGNEFEDYCLKRELLMGIFEMGWEKPSPIQEESIPIALSGRDILARAKNGTGKSGAYLIPLLERIDLK

KDCLQALVIVPTRELALQVSQISIQVSKHMGGVKVMATTGGTNLRDDIMRLDETVHVVIATPGRILDLIKKGVAKVNRVQ

MIVLDEADKLLSQDFVVMMEEMLSFLPKQRQILLYSATFPLSVQKFMNAHLQKPYEINLMEELTLKGVTQYYAYVTERQK

VHCLNTLFSRINQSIIFCNSSQRVELLAKKISQLGYSCFYIHAKMRQEHRNRVFHDFRNGLCRNLVCTDLFTRGIDIQAV

NVVINFDFPKLGETYLHRIGRSGRFGHLGLAINLITYDDRFNLKAIEEQLGTEIKPIPGIIDKSLYVAEYHSESGEEGKQ

>XP_019943893.1 PREDICTED: probable ATP-dependent RNA helicase DDX6 [Paralichthys olivaceus]

MSTARTENPVILGLSNQNGQLRGSVKPAGAPGGGGPQQQQQQQQLNQVKGTINNGNSQPAPTTNAVIKPGDDWKKNLKLP

PKDTRMKTSDVTATKGNEFEDYCLKRELLMGIFEMGWEKPSPIQEESIPIALSGRDILARAKNGTGKSGAYLIPLLERID

LKRDCIQAVVIVPTRELALQVSQICIQVSKRMGGVKVMATTGGTNLRDDILRLDETVHVIIATPGRILDLIKKGVAKVNQ

VQMIVLDEADKLLSQDFVVMMEEVLGFLSKQRQILLYSATFPLSVQKFMNSHLQKPYEINLMEELTLKGVTQYYAYVTER

QKVHCLNTLFSRLQINQSIIFCNSSQRVELLAKKISQLGYSCFYIHAKMRQEHRNRVFHDFRNGLCRNLVCTDLFTRGID

IQAVNVVINFDFPKLGETYLHRIGRSGRFGHLGLAINLITYDDRFNLKGIEEQLGTEIKPIPGIIDKSLYVAEYHSESGE

EVKP

>AAB94769.1 putative RNA helicase RCK, partial [Mus musculus]

KASAGPGGGGTQTQQQMNQLKNTSTINNGTPQQAQSMAATIKPGDDWKKTLKLPPKDLRIKTSDVTSTKGNEFEDYCLKR

ELLMGIFEMGWEKPSPIQEESIPIALSGRDILARAKNGTGKSGAYLIPLLERLDLKKDNIQAMVIVPTRELALQVSQICI

QVSKHMGGAKVMATTGPTNLRDDIMRLDDTVHVVIATPGRILDLIKKGVAKVDHVQMIVLDEADKLLSQDFVQIMEDIIL

TLPKNRQILLYSATFPLSVQKFMNSHLQKPYEINLMEELTLKGVTQYYAYVTERQKVHCLNTLFSRLQINQSIIFCNSSQ

RVELLAKKISQLGYSCFYIHAKMRQEHRNRVFHDFENGLCRNLVCTDLFTRGIDIQAVNVVINFDFPKLAETYLHRVGRS

GRFGHLGLAINLITYDDRFNLKSIEEQLGTEIKPIPSNIDKSLYVAEYHSEPV

>KAF4008936.1 hypothetical protein G4228_000111 [Cervus hanglu yarkandensis]

MSTARTENPVIMGLSSQNGQLRGPVKPSGGPGGGGAQTQQQMNQLKNTNTINNGTQQQAQSMTTTIKPGDDWKKTLKLPP

KDLRIKTSDVTSTKGNEFEDYCLKRELLMGIFEMGWEKPSPIQEESIPIALSGRDILARAKNGTGKSGAYLIPLLERLDL

KKDNIQAMVIVPTRELALQVSQICIQVSKHMGGAKVMATTGGTNLRDDIMRLDDTVHVVIATPGRILDLIKKGVAKVDHV

QMIVLDEADKLLSQDFVQIMEDIILTLPKNRQILLYSATFPLSVQKFMNSHLQKPYEINLMEELTLKGVTQYYAYVTERQ

KVHCLNTLFSRLQINQSIIFCNSSQRVELLAKKISQLGYSCFYIHAKMRQEHRNRVFHDFRNGLCRNLVCTDLFTRGIDI

QAVNVVINFDFPKLAETYLHRIGRSGRFGHLGLAINLITYDDRFNLKSIEEQLGTEIKPIPSNIDKSLLQPLPQSQLCPA

CSLYPLGPLLSQDSRILTYQYGIPTSLQKTGQQWDFPNAWDPLQDLVIRGRQGWGLPHLSTTRAEARPPWHWGGEGFGWT

NGVALMLLDRYGDRLSSGVQTAFLEPHCLAAALLLSLLPQ

>XP_016375868.1 PREDICTED: probable ATP-dependent RNA helicase DDX6 [Sinocyclocheilus rhinocerous]

MSTARMENPVILGLSNQNGQMRGSVKPAGGPGGGGGGSQTTLPAQVKPSSTVNNGDSQSAPTANTIIKPGDDWKKNLKLP

PKDLRMRTSDVTATKGNEFEDYCLKRELLMGIFEMGWEKPSPIQEESIPIALSGRDILARAKNGTGKSGAYLIPLLERID

LKKDSIQALVVVPTRELALQVSQICIQVSKHMGGVKVMATTGGTNLRDDIMRLDETVHVVIATPGRILDLIKKGVAKVNQ

VQMIVLDEADKLLSQDFVQMMEELLNCLAKQRQILLYSATFPLSVQKFMNTHLQKPYEINLMDELTLKGVTQYYAYVTER

QKVHCLNTLFSRLQINQSIIFCNSSQRVELLAKKISQLGYSCFYIHAKMRQEHRNRVFHDFRNGLCRNLVCTDLFTRGID

IQAVNVVINFDFPKLGETYLHRIGRSGRFGHLGLAINLITYDDRFNLKGIEEQLGTEIKPIPSSIDKSLYVAEYHSESGE

EVKL

>XP_031651183.1 LOW QUALITY PROTEIN: probable ATP-dependent RNA helicase DDX6 [Oncorhynchus kisutch]

MSTASTQNPVILGHSNQNGQLRGPVVKPAGGQGSGGGSHQTQQPSHMKQASSTINNGTGPSSTQLAKATPTANTVIKPGD

DWKRNLTLPPKDTRMRTSDVTATKGNEFEDYCLKRELLMGIFEMGWEKPSPIQEESIPIALSGRDILARAKNGTGKSGAY

LIPLLERIDLKKDCIQAMGIVPTRELALQVSQICIQISKHMGGVKVMATTGGTNLRDDIMRLDETVHVVIAXPGRILDLI

KKGVAKANQVQMMVLDEADKLLSQDFVVMMEEILSYLPKQRQILLYSATFPLSVQKFMNSHLSKPYEINLMEELTLKGVT

QYYAYVTERQKVHCLNTLFSRLQINQSIIFCNSSQRVELLAKKISQLGYSCFYIHAKMRQEHRNRVFHDFRNGLCRNLVC

TDLFTRGIDIQAVNVVINFDFPKLGETYLHRIGRSGRFGHLGLAINLITYDDRFNLKGIEEQLGTEIRPIPGCIDKSLYV

AEYHSENGEVKL

>XP_007909383.1 PREDICTED: probable ATP-dependent RNA helicase DDX6 isoform X1 [Callorhinchus milii]

MTTARTENPIIMGLSTQNGQLRGPLKPSTGPGGMPPQQTTQTNQQLQQLKHASTLNGTQQQAHTTSTTIKPGDDWKKSLK

LPPKDRRMKTSDVTATKGNEFEDYCLKRELLMGIFEMGWEKPSPIQEESIPIALSGRDILARAKNGTGKSGAYLIPLLER

LDLKRDCIQAMVIVPTRELALQVSQICIQVSKHMGGVKVMATTGGTNLRDDIMRLDETVHVIIATPGRILDLIKKGLAKV

DNIQMIVLDEADKLLSQDFVQMMEDIISTLPRNRQILLYSATFPLSVQKFMMMQDCLAERVPNNSHLQKPYEINLMEELT

LKGVTQYYAYVTERQKVHCLNTLFSRLQINQSIIFCNSSQRVELLAKKISQLGYSCFYIHAKMRQEHRNRVFHDFRNGMC

RNLVCTDLFTRGIDIQAVNVVINFDFPKIAETYLHRIGRSGRFGHLGLAINLITYDDRFNLKAIEEQLGTEIKPIPGSID

KSLYVAEYHSEPDGEDKQ

>XP_030008554.1 LOW QUALITY PROTEIN: probable ATP-dependent RNA helicase ddx6 [Sphaeramia orbicularis]

MSTAGTENPVILGLSNQNGQVICSAKTAGAPGGGGGGGGGGGGGPQKPQINQMKGTINMGNSVSAPTPNAVIRPRDDWKK

SLKLPPKDMRMKTSDVTATKGNEFEDYCLKRELLMGIFEMGWEKPSPIQEESIPIALSGRDILARAKNGTGKSGAYLIPL

LERIDLKRDCIQALVIVPTRELALQVSQICIQVSKHMGGVKVMATTGGTNLRDDIMRLDETVHVVIATPGRILDLIKKGV

AKVNQVQMIVLDEADKLLSQDFVLMMEEILGXSKQRQILLYSATFPLSVQKFMTSHLQKPYEINLMEELTLKGVTQYYAY

VTERQKVHCLNTLFSRLQINQSIIFCNSSQRVELLAKKISQLGYSCFYIHAKMRQEHRNRVFHDFRNGLCRNLVCTDLFT

RGIDIQAVNVVINFDFPKLGETYLHRIGRSGRFGHLGLAINLITYDDRFNLKGIEEQLGTEIKPIPGIIDKSLYVAEYHS

ESGEEVKP

>XP_016318752.1 PREDICTED: probable ATP-dependent RNA helicase ddx6 [Sinocyclocheilus anshuiensis]

MSTARMENPVILGLSNQNGQMRGSVKPAGGPGGGGGGSQTTLPAQVKPSSTVNNGDSQPAPTANTIIKPGDDWKKNLKLP

PKDLRMRTSDVTATKGNEFEDYCLKRELLMGIFEMGWEKPSPIQEESIPIALSGRDILARAKNGTGKSGAYLIPLLERID

LKKDSIQALVVVPTRELALQVSQICIQVSKHMGGVKVMATTGGTNLRDDIMRLDETVHVVIATPGRILDLIKKGVAKVNQ

VQMIVLDEADKLLSQDFVQMMEELLNCLAKQRQILLYSATFPLSVQKFMNTHLQKPYEINLMDELTLKGVTQYYAYVTER

QKVHCLNTLFSRLQINQSIIFCNSSQRVELLAKKISQLGYSCFYIHAKMRQEHRNRVFHDFRNGLCRNLVCTDLFTRGID

IQAVNVVINFDFPKLGETYLHRIGRSGRFGHLGLAINLITYDDRFNLKGIEEQLGTEIKPIPSSIDKSLYVAEYHSESGE

EVKL

>NWI10886.1 DDX6 helicase [Crypturellus soui]

MSTARTENPVIMGLSSQNGQLRGPVKPSGGPGGGGTQTQQQMNQMKNANTINNGTQQQAQSMTTTIKPGDDWKKTLKLPP

KDLRIKTSDVTSTKGNEFEDYCLKRELLMGIFEMGWEKPSPIQEESIPIALSGRDILARAKNGTGKSGAYLIPLLERLDL

KKDNIQAMVIVPTRELALQVSQICIQVSKHMGGAKVMATTGGTNLRDDIMRLDDTVHVVIATPGRILDLIKKGVAKVEHV

QMIADKLLSQDFVQIMEDIILTLPKNRQILLYSATFPLSVQKFMNSHLQKPYEINLMEELTLKGVTQYYAYVTERQKVHC

LNTLFSRLQINQSIIFCNSSQRVELLAKKISQLGYSCFYIHAKMRQEHRNRVFHDFRNGLCRNLVCTDLFTRGIDIQAVN

VVINFDFPKLAETYLHRIGRSGRFGHLGLAINLITYDDRFNLKSIEEQLGTEIKPIPSNIDKSLYVAEYHSEPVEDEKP

>XP_025913782.1 probable ATP-dependent RNA helicase DDX6 isoform X3 [Apteryx rowi]

MILGPGDDWKKTLKLPPKDLRIKTSDVTSTKGNEFEDYCLKRELLMGIFEMGWEKPSPIQEESIPIALSGRDILARAKNG

TGKSGAYLIPLLERLDLKKDNIQAMVIVPTRELALQVSQICIQVSKHMGGAKVMATTGGTNLRDDIMRLDDTVHVVIATP

GRILDLIKKGVAKVEHVQMIVLDEADKLLSQDFVQIMEDIILTLPKNRQILLYSATFPLSVQKFMSLFQNSHLQKPYEIN

LMEELTLKGVTQYYAYVTERQKVHCLNTLFSRLQINQSIIFCNSSQRVELLAKKISQLGYSCFYIHAKMRQEHRNRVFHD

FRNGLCRNLVCTDLFTRGIDIQAVNVVINFDFPKLAETYLHRIGRSGRFGHLGLAINLITYDDRFNLKSIEEQLGTEIKP

IPSNIDKSLYVAEYHSEPVEDEKP

>NXX22926.1 DDX6 helicase [Podargus strigoides]

MSTARTENPVIMGLSSQNGQLRGPVKPSGGPGGGGTQTQQQMNQLKNANTINNGTQQQAQSMTTTIKPGDDWKKTLKLPP

KDLRIKTSDVTSTKGNEFEDYCLKRELLMGIFEMGWEKPSPIQEESIPIALSGRDILARAKNGTGKSGAYLIPLLERLDL

KKDNIQAMVIVPTRELALQVSQICIQVSKHMGGAKVMATTGGTNLRDDIMRLDDTVHVVIATPGRILDLIKKGVAKVEHV

QMIADKLLSQDFVQIMEDIILTLPKNRQILLYSATFPLSVQKFMNSHLQKPYEINLMEELTLKGVTQYYAYVTERQKVHC

LNTLFSRLQINQSIIFCNSSQRVELLAKKISQLGYSCFYIHAKMRQEHRNRVFHDFRNGLCRNLVCTDLFTRGIDIQAVN

VVINFDFPKLAETYLHRIGRSGRFGHLGLAINLITYDDRFNLKSIEEQLGTEIKPIPSNIDKSLYVAEYHSEPVEDEKQ

>EMP33396.1 Putative ATP-dependent RNA helicase DDX6 [Chelonia mydas]

MSTARTENPVIMGLSSQNGQLRGPVKPSGGPGGGGTQTQQQMNQLKNTNTINNGTQQQAQSMTTAIKPGDDWKKTLKLPP

KDLRIKTSDVTSTKGNEFEDYCLKRELLMGIFEMGWEKPSPIQEESIPIALSGRDILARAKNGTGKSGAYLIPLLERLDL

KKDNIQAMVIVPTRELALQVSQICIQVSKHMGGAKVMATTGGTNLRDDIMRLDDTVHVVIATPGRILDLIKKGVAKVEHV

QMIADKLLSQDFVQIMEDIILTLPKNRQILLYSATFPLSVQKFMNSHLQKPYEINLMEELTLKGVTQYYAYVTERQKVHC

LNTLFSRLQINQSIIFCNSSQRVELLAKKISQLGYSCFYIHAKMRQEHRNRVFHDFRNGLCRNLVCTDLFTRGIDIQAVN

VVINFDFPKLAETYLHRIGRSGRFGHLGLAINLITYDDRFNLKSIEEQLGTEIKPIPSNIDKSLYVAEYHTEPVDDEKP

>NXA42906.1 DDX6 helicase [Eudromia elegans]

MSTARTENPVIMGLSSQNGQLRGPVKPSGGPGGGGTQTQQQINQLKNANTINNGTQQQAQSMTTAIKPGDDWKKTLKLPP

KDLRIKTSDVTSTKGNEFEDYCLKRELLMGIFEMGWEKPSPIQEESIPIALSGRDILARAKNGTGKSGAYLIPLLERLDL

KKDNIQAMVIVPTRELALQVSQICIQVSKHMGGAKVMATTGGTNLRDDIMRLDDTVHVVIATPGRILDLIKKGVAKVEHV

QMIADKLLSQDFVQIMEDIILTLPKNRQILLYSATFPLSVQKFMNSHLQKPYEINLMEELTLKGVTQYYAYVTERQKVHC

LNTLFSRLQINQSIIFCNSSQRVELLAKKISQLGYSCFYIHAKMRQEHRNRVFHDFRNGLCRNLVCTDLFTRGIDIQAVN

VVINFDFPKLAETYLHRIGRSGRFGHLGLAINLITYDDRFNLKSIEEQLGTEIKPIPSNIDKSLYVAEYHSEPVEDEKP

>NWX49854.1 DDX6 helicase [Steatornis caripensis]

MSTARTENPVIMGLSSQNGQLRGPVKPSGGPGGGGTQTQQQMNQLKNANTINNGTQQQAQSMTTAIKPGDDWKKTLKLPP

KDLRIKTSDVTSTKGNEFEDYCLKRELLMGIFEMGWEKPSPIQEESIPIALSGRDILARAKNGTGKSGAYLIPLLERLDL

KKDNIQAMVIVPTRELALQVSQICIQVSKHMGGAKVMATTGGTNLRDDIMRLDDTVHVVIATPGRILDLIKKGVAKVEHV

QMIADKLLSQDFVQIMEDIILTLPKNRQILLYSATFPLSVQKFMNSHLQKPYEINLMEELTLKGVTQYYAYVTERQKVHC

LNTLFSRLQINQSIIFCNSSQRVELLAKKISQLGYSCFYIHAKMRQEHRNRVFHDFRNGLCRNLVCTDLFTRGIDIQAVN

VVINFDFPKLAETYLHRIGRSGRFGHLGLAINLITYDDRFNLKSIEEQLGTEIKPIPSNIDKSLYVAEYHSEPVEDEKQ

>NXA57111.1 DDX6 helicase [Nothocercus julius]

MSTARTENPVIMGLSSQNGQLRGPVKPSGGPGGGGTQTQQQMNQLKNANAINNGTQQQAQSMTTTIKPGDDWKKTLKLPP

KDLRIKTSDVTSTKGNEFEDYCLKRELLMGIFEMGWEKPSPIQEESIPIALSGRDILARAKNGTGKSGAYLIPLLERLDL

KKDNIQAMVIVPTRELALQVSQICIQVSKHMGGAKVMATTGGTNLRDDIMRLDDTVHVVIATPGRILDLIKKGVAKVEHV

QMIADKLLSQDFVQIMEDIILTLPKNRQILLYSATFPLSVQKFMNSHLQKPYEINLMEELTLKGVTQYYAYVTERQKVHC

LNTLFSRLQINQSIIFCNSSQRVELLAKKISQLGYSCFYIHAKMRQEHRNRVFHDFRNGLCRNLVCTDLFTRGIDIQAVN

VVINFDFPKLAETYLHRIGRSGRFGHLGLAINLITYDDRFNLKSIEEQLGTEIKPIPSNIDKSLYVAEYHSEPVEDEKP

>XP_016114638.1 PREDICTED: probable ATP-dependent RNA helicase ddx6 [Sinocyclocheilus grahami]

MSTARMENPVILGLSNQNGQMRGSVKPAGGPGGGGGGSQTTLPAQVKPSSTVNNGDSQPAPTANTIIKPGDDWKKNLKLP

PKDLRMRTSDVTATKGNEFEDYCLKRELLMGIFEMGWEKPSPIQEESIPIALSGRDILARAKNGTGKSGAYLIPLLERID

LKKDSIQAVVVVPTRELALQVSQICIQVSKHMGGVKVMATTGGTNLRDDIMRLDETVHVVIATPGRILDLIKKGVAKVNQ

VQMIVLDEADKLLSQDFVQMMEELLNCLAKQRQILLYSATFPLSVQKFMNTHLQKPYEINLMDELTLKGVTQYYAYVTER

QKVHCLNTLFSRLQINQSIIFCNSSQRVELLAKKISQLGYSCFYIHAKMRQEHRNRVFHDFRNGLCRNLVCTDLFTRGID

IQAVNVVINFDFPKLGETYLHRIGRSGRFGHLGLAINLITYDDRFNLKGIEEQLGTEIKPIPSSIDKSLYVAEYHSESGE

EVKL

>KAF0030147.1 hypothetical protein F2P81_016878 [Scophthalmus maximus]

MSTVRTENPVILGLSNQNGQLRGSVKPAGAPGGGGGGPQQQLLSQMKGTINNGNSQPAPTTNAVIKPGDDWKKNLKLPPK

DMRMKTSDVTATKGNEFEDYCLKRELLMGIFEMGWEKPSPIQEESIPIALSGRDILARAKNGTGKSGAYLIPLLERIDLK

KDCIQAMVIVPTRELALQVSQICIQVSKRMGGVKVMATTGGTNLRDDILRLDETVHVVIATPGRILDLIKKGVAKVNQVQ

MVVLDEADKLLSQDFVLMMEEMLGFLAKQRQILLYSATFPLSVQKFMNSHLQKPYEINLMEELTLKGVTQYYAYVTERQK

VHCLNTLFSRLQINQSIIFCNSSQRVELLAKKISQLGYSCFYIHAKMRQEHRNRVFHDFRNGLCRNLVCTDLFTRGIDIQ

AVNVVINFDFPKLGETYLHRIGRSGRFGHLGLAINLITYDDRFNLKGIEEQLGTEIKPIPGIIDKSLYVAEYHTALLAGV

KSGSPAPCDSEIYCTGPILHQVQKAKLFDDDKYFVDMKLKAMPDVVLSAFHNLSSQMPNMTVLPARLQEFLSVNFERAGS

EFEPWTPPDWHDKPKFLGGISDPKLREWAEKIHNLWKSLGRKMRAGVKDHPELYSQMYVPHPFVVPGGRFRELYYWDSYW

VVNGLLLSEMTDTAYGMIQNFLHLVSRYGFVPNGGRIYYERRSQPPFLTLMVESYYQATKDKDFLRGALPALEQEHRFWM

QNRSVAVKVNGSEHVLNRYHVQVGLPRPESYTDDLDLAEGLTDDHKEQLWMDLKAGAESGWDFSSRWYVDGGGGHNNNKN

NNSGSLRDTRTSQILPTDLNALLCLNEKTLASFHRILGDGDSAALYDQAAARRQGAMESVLWDAERGAWFDYNLMTHAKH

SEFYPSNMAPVWAQCYSRPEMGEKAVQYLKASGALRFPNGVPTSLRESGQQWDYPNAWPPLQHMLISGLSKLPSEDAQQL

AFDLAQRWIKTNWLAYIKYDAMFEKYDVNGDGKPGSGGEYDVQLGFGWTNGVVLQLLERYGATLTSGSRQVSPGLLLPLV

ISAALALR

>XP_024261918.1 LOW QUALITY PROTEIN: probable ATP-dependent RNA helicase DDX6 [Oncorhynchus tshawytscha]

MSTASTQNPVILGHSNQNGQLRGPVVKPAGGQGSGGGSHQTQQPSHMKQASSTINNGTGPSSTQLAKATPTANTVIKPGD

DWKRNLTLPPKDTRMRTSDVTAXKGNEFEDYCLKRELLMGIFEMGWXKPSPIQEESIPIALSGRDILARAKNGTGKSGAY

LIPLLERIDLKKDCIQAMGIVPTRELALQVSQICIQISKHMGGVKVMATTGGTNLRDDIMRLDETVHVVIATPGRILDLI

KKGVAKVNQVQMMVLDEADKLLSQDFVVMMEEILSYLPKQRQILLYSATFPLSVQKFMNSHLSKPYEINLMEELTLKGVT

QYYAYVTERQKVHCLNTLFSRLQINQSIIFCNSSQRVELLAKKISQLGYSCFYIHAKMRQEHRNRVFHDFRNGLCRNLVC

TDLFTRGIDIQAVNVVINFDFPKLGETYLHRIGRSGRFGHLGLAINLITYDDRFNLKGIEEQLGTEIRPIPGCIDKSLYV

AEYHSENGEVKL

>GCC34628.1 hypothetical protein [Chiloscyllium punctatum]

MTTARTENPVIMGLSTQNGQLRGPLKPSAGPGGMPSPQVSQTNQQLQQLKNASTLNGTQQQAHTTSSTIKPGDDWKKSLK

LPPKDRRMKTSDVTATKGNEFEDYCLKRELLMGIFEMGWEKPSPIQEESIPIALSGRDILARAKNGTGKSGAYLIPLLER

LDLKRDCIQAMVIVPTRELALQVSQICIQVSKHMGGVKVMATTGGTNLRDDILRLDETVHVVIATPGRILDLIKKGLAKV

DNIQMIVLDEADKLLSQDFVQMMEDIISTLPRSRQILLYSATFPLSVQKFMNSHLQKPYEINLMEELTLKGVTQYYAYVT

ERQKVHCLNTLFSRLQINQSIIFCNSSQRVELLAKKISQLGYSCFYIHAKMRQEHRNRVFHDFRNGMCRNLVCTDLFTRG

IDIQAVNVVINFDFPKIAETYLHRIGRSGRFGHLGLAINLITYDDRFNLKAIEEQLGTEIKPIPGITVQTNRR

>AWO98658.1 Trehalase [Scophthalmus maximus]

MSTVRTENPVILGLSNQNGQLRGSVKPAGAPGGGGGGPQQQLLSQMKGTINNGNSQPAPTTNAVIKPGDDWKKNLKLPPK

DMRMKTSDVTATKGNEFEDYCLKRELLMGIFEMGWEKPSPIQEESIPIALSGRDILARAKNGTGKSGAYLIPLLERIDLK

KDCIQAMVIVPTRELALQVSQICIQVSKRMGGVKVMATTGGTNLRDDILRLDETVHVVIATPGRILDLIKKGVAKVNQVQ

MVVLDEADKLLSQDFVLMMEEMLGFLAKQRQILLYSATFPLSVQKFMNSHLQKPYEINLMEELTLKGVTQYYAYVTERQK

VHCLNTLFSRLQINQSIIFCNSSQRVELLAKKISQLGYSCFYIHAKMRQEHRNRVFHDFRNGLCRNLVCTDLFTRGIDIQ

AVNVVINFDFPKLGETYLHRIGRSGRFGHLGLAINLITYDDRFNLKGIEEQLGTEIKPIPGIIDKSLYVAEYHSESAALL

AGVKSGSPAPCDSEIYCTGPILHQVQKAKLFDDDKYFVDMKLKAMPDVVLSAFHNLSSQMPNMTVLPARLQEFLSVNFER

AGSEFEPWTPPDWHDKPKFLGGISDPKLREWAEKIHNLWKSLGRKMRAGVKDHPELYSQMYVPHPFVVPGGRFRELYYWD

SYWVVNGLLLSEMTDTAYGMIQNFLHLVSRYGFVPNGGRIYYERRSQPPFLTLMVESYYQATKDKDFLRGALPALEQEHR

FWMQNRSVAVKVNGSEHVLNRYHVQVGLPRPESYTDDLDLAEGLTDDHKEQLWMDLKAGAESGWDFSSRWYVDGGGGHNN

NKNNNSGSLRDTRTSQILPTDLNALLCLNEKTLASFHRILGDGDSAALYDQAAARRQGAMESVLWDAERGAWFDYNLMTH

AKHSEFYPSNMAPVWAQCYSRPEMGEKAVQYLKASGALRFPNGVPTSLRESGQQWDYPNAWPPLQHMLISGLSKLPSEDA

QQLAFDLAQRWIKTNWLAYIKYDAMFEKYDVNGDGKPGSGGEYDVQLGFGWTNGVVLQLLERYGATLTSGSRQVSPGLLL

PLVISAALALR

>TNN66655.1 putative ATP-dependent RNA helicase DDX6 [Liparis tanakae]

MSTARTETPVILGLSNQNGQLRGSVKPTGAPGGGGGSGGGGGGGGPQPQLIQMKGTINNGNGPLGPSSNAIKPGDDWKKS

LKLPPKDTRMRTSDVTSTKGNEFEDYCLKRELLMGIFEMGWEKPSPIQEESIPIALSGRDILARAKNGTGKSGAYLIPLL

ERIDLKRDCIQAMVIVPTRELALQVSQISIQVSKHMGGVKVMATTGGTNLRDDIMRLDETVHVIIATPGRILDLIKKGLA

KVNQVQMVVLDEADKLLSQDFVVMMEEMLGFLSKRRQILLYSATFPVSVQKFMNAHLQKPYEINLMEELTLKGVTQYYAY

VTERQKVHCLNTLFSRLQINQSIIFCNSSQRVELLAKKISQLGYSCFYIHAKMRQEHRNRVFHDFRNGLCRNLVCTDLFT

RGIDIQAVNVVINFDFPKLGETYLHRIGRSGRFGHLGLAINLITYDDRFNLKGIEEQLGTEIKPIPGIIDKSLYVAEYHS

ESGEEI

>XP_028679627.1 probable ATP-dependent RNA helicase ddx6 [Erpetoichthys calabaricus]

MATARTENPPSMVMGLRKQNGQLRGQQQKPITPQAGSSGFGSQSTKNTHIVQNVSQTPAVSQPPQNSGIRFGDDWKKALQ

LPPKDMRIKTSDVTATKGNEFEDYCLKRELLMGIFEMGWEKPSPIQEESIPIALSGRDILARAKNGTGKSGAYLIPLLER

LDLKKDCIQAIVMVPTRELALQVSQICIQISKHMGGVKVMATTGGTNLRDDIMRLDETVHVVIATPGRILDLIKKGVAKV

DRVQMMVMDEADKLLSLDFVVLIEEIISYLPKNRQILLYSATFPISVQKFMSKYLQKPYEINLMDELTLKGITQFYAYVT

ERQKVHCLNTLFSRLQINQSIIFCNSTQRVELLAKKITQLGYSCFYIHAKMMQEYRNRVFHDFRNGLCRNLVCTDLFTRG

IDIQAVNVVINFDFPKNAETYLHRIGRSGRFGHLGLAINLITSEDRFNLKSIEDQLVTEIKPIPSTIDKSLYVAEYHSEV

NSQDEGGRSDEQQ

>XP_033852189.1 probable ATP-dependent RNA helicase ddx6 [Acipenser ruthenus]

MSTARMETPVILGLSNQNGQLRGPVKPPGGPGGGGGGVTPTQQTNQIKNASVNNGNPYPAQTPNSTIKPGDDWKKNLQVP

TKDMRMKTSDVTATKGNEFEDYCLKRELLMGIFEMGWEKPSPIQEESIPIALSGRDILARAKNGTGKSGAYLIPLLERID

LKKDCIQALVVVPTRELALQVSQISIQVSKHMGGVKVMATTGGTNLRDDIMRLDETVHVVIATPGRILDLIKKGVAKVSK

VQMIVLDEADKLLSQDFVQIMEEIIGTLAKNRQVLLYSATFPTSVQKFMSTHLQKPYEINLMEELTLKGVTQYYAYVTER

QKVHCLNTLFSRLQINQSIIFCNSSQRVELLAKKISQLGYSCFYIHAKMRQEHRNRVFHDFRNGLCRNLVCTDLFTRGID

IQAVNVVINFDFPKLAETYLHRIGRSGRFGHLGLAINLITYDDRFNLKGIEEQLGTEIKPIPGSIDKSLYVAEYHSETGE

EDKL

>XP_012688203.1 probable ATP-dependent RNA helicase DDX6 [Clupea harengus]

MTTARTESQVILGLSNQNGQLRGSGKTAAGPGGGSPQQPASQVKATSTINNGSSQPAPTNTVIKPGDDWKKSLTLPPKDT

RMRTSDVTATKGNEFEDYCLKRELLMGIFEMGWEKPSPIQEESIPIALSGRDILARAKNGTGKSGAYLIPLLERIDLKKD

CIQALVLVPTRELALQVSQISIQVSKHMGGVKVMATTGGTNLRDDIMRLDETVHVVIATPGRILDLIKKGVAKVSQVQMI

VLDEADKLLSQDFLPMMEETLSFMCKQRQILLYSATFPLSVQKFMNAQLQKPYEINLMEELTLKGVTQYYAYVTERQKVH

CLNTLFSRLQINQSIIFCNSSQRVELLAKKISQLGYSCFYIHAKMRQEHRNRVFHDFRNGLCRNLVCTDLFTRGIDIQAV

NVVINFDFPKLGETYLHRIGRSGRFGHLGLAINLITYDDRFNLKSIEEQLGTEIKPIPSSIDKSLYVAEFHPESGEELKL

>RMB91917.1 hypothetical protein DUI87_31445 [Hirundo rustica rustica]

MSTARTENPVIMGLSSQNGQLRGPVKPSGGPGGGGTQTQQQMNQLKNANTINNGTQQQAQSMTTAIKPGDDWKKTLKLPP

KDLRIKTSDVTSTKGNEFEDYCLKRELLMGIFEMGWEKPSPIQEESIPIALSGRDILARAKNGTGKSGAYLIPLLERLDL

KKDNIQAMVIVPTRELALQVSQICIQVSKHMGGAKVMATTGGTNLRDDIMRLDDTVHVVIATPGRILDLIKKGVAKVEHA

DKLLSQDFVQIMEDIILTLPKNRQILLYSATFPLSVQKFMNSHLQKPYEINLMEELTLKGVTQYYAYVTERQKVHCLNTL

FSRLQINQSIIFCNSSQRVELLAKKISQLGYSCFYIHAKMRQEHRNRVFHDFRNGLCRNLVCTDLFTRGIDIQAVNVVIN

FDFPKLAETYLHRIGRSGRFGHLGLAINLITYDDRFNLKSIEEQLGTEIKPIPSNIDKSLYVAEYHSEPVEDEKQ

>XP_034459921.1 probable ATP-dependent RNA helicase ddx6 [Hippoglossus hippoglossus]

MSTARTENPMILGLSNQNGQLRGSVKPAGAPGGGGGGPQLQQLQLNQVKGTINNGSSQSAPTTNAVIKPGDDWKKNLKLP

PRDTRMKTSDVTATKGNEFEDYCLKRELLMGIFEMGWEKPSPIQEESIPIALSGRDILARAKNGTGKSGAYLIPLLERID

LKRDHIQAVVIVPTRELALQVSQICIQVSKRMGGVKVMATTGGTNLRDDILRLDETVHVIIATPGRILDLIKKGVAKVNQ

VQMIVLDEADKLLSQDFMAMMEEILGFLSKQRQILLYSATFPLSVQKFMNAYLQKPYEINLMEELTLKGVTQYYAYVTER

QKVHCLNTLFSRLQINQSIIFCNSSQRVELLAKKISQLGYSCFYIHAKMRQEHRNRVFHDFRNGLCRNLVCTDLFTRGID

IQAVNVVINFDFPKLGETYLHRIGRSGRFGHLGLAINLITYDDRFNLKGIEEQLGTEIKPIPGIIDKSLYVAEYHSESGE

EVKP

>EAW67413.1 DEAD (Asp-Glu-Ala-Asp) box polypeptide 6, isoform CRA_b [Homo sapiens]

MSTARTENPVIMGLSSQNGQLRGPVKPTGGPGGGGTQTQQQMNQLKNTNTINNGTQQQAQSMTTTIKPGDDWKKTLKLPP

KDLRIKTSDVTSTKGNEFEDYCLKRELLMGIFEMGWEKEESIPIALSGRDILARAKNGTGKSGAYLIPLLERLDLKKDNI

QAMVIVPTRELALQVSQICIQVSKHMGGAKVMATTGGTNLRDDIMRLDDTVHVVIATPGRILDLIKKGVAKVDHVQMIVL

DEADKLLSQDFVQIMEDIILTLPKNRQILLYSATFPLSVQKFMNSHLQKPYEINLMEELTLKGVTQYYAYVTERQKVHCL

NTLFSRLQINQSIIFCNSSQRVELLAKKISQLGYSCFYIHAKMRQEHRNRVFHDFRNGLCRNLVCTDLFTRGIDIQAVNV

VINFDFPKLAETYLHRIGRSGRFGHLGLAINLITYDDRFNLKSIEEQLGTEIKPIPSNIDKSLYVAEYHSEPVEDEKP

>XP_030002603.1 probable ATP-dependent RNA helicase ddx6 [Sphaeramia orbicularis]

MATAKTENVGPVVMGLNKQNGQLRGQTKPASVQPAPTSQGKSLAPPQKAGNAPQEGGGIKFGDDWKKSLKLPPKDNRVKT

SDVTSTKGNEFEDYCLKRELLMGIFEMGWEKPSPIQEESIPIALSGRDILARAKNGTGKSGAYLIPMLERIDLKKDHIQA

IVMVPTRELALQVSQICIQISKHLGGVKVMATTGGTNLRDDIMRLDETVHVVIATPGRILDLIKKGVAKVDRVQMMIMDE

ADKLLSQDFVVLIEDIISFLAKNRQILLYSATFPISVQKFMAKHLQKPYEINLMEELTLKGITQYYAYVTERQKVHCLNT

LFSRLQINQSIIFCNSTQRVELLAKKITQLGYSCFYIHAKMMQEYRNRVFHDFRNGLCRNLVCTDLFTRGIDIQAVNVVI

NFDFPKNAETYLHRIGRSGRFGHLGLAINLITSEDRFNLKAIEDQLVTDIKPIPSSIDKSLYVAEYHCSNADCDVEEVEE

KSGHQQDST

>XP_023595691.1 probable ATP-dependent RNA helicase DDX6 isoform X1 [Trichechus manatus latirostris]

MSTARTENPVIMGLSSQNGQLRGPVKPSGGPGGGGTQTQQQMNQLKNTNTINNGTQQQAQSMTTAIKPGDDWKKTLKLPP

KDLRIKTSDVTSTKGNEFEDYCLKRELLMGIFEMGWEKPSPIQIEVCVLSWNTAEVTLWPSRISRLEAYTTSVHPSLEES

IPIALSGRDILARAKNGTGKSGAYLIPLLERLDLKKDNIQAMVIVPTRELALQVSQICIQVSKHMGGAKVMATTGGTNLR

DDIMRLDDTVHVVIATPGRILDLIKKGVAKVDHVQMIVLDEADKLLSQDFVQIMEDIILTLPKNRQILLYSATFPLSVQK

FMNSHLQKPYEINLMEELTLKGVTQYYAYVTERQKVHCLNTLFSRLQINQSIIFCNSSQRVELLAKKISQLGYSCFYIHA

KMRQEHRNRVFHDFRNGLCRNLVCTDLFTRGIDIQAVNVVINFDFPKLAETYLHRIGRSGRFGHLGLAINLITYDDRFNL

KSIEEQLGTEIKPIPSNIDKSLYVAEYHSEPVEDEKP

>KAF4796439.1 putative ATP-dependent RNA helicase DDX6 [Turdus rufiventris]

MSTARTENPVIMGLSSQNGQLRGPVKPSGGPGGGGTQTQQQMNQLKNASTINNGTQQQAQSMTTAIKPGDDWKKTLKLPP

KDLRIKTSDVTSTKGNEFEDYCLKRELLMGIFEMGWEKPSPIQEESIPIALSGRDILARAKNGTGKSGAYLIPLLERLDL

KKDNIQAMVIVPTRELALQVSQICIQVSKHMGGAKVMATTGGTNLRDDIMRLDDTVHVVIATPGRILDLIKKGVAKVEHA

DKLLSQDFVQIMEDIILTLPKNRQILLYSATFPLSVQKFMNSHLQKPYEINLMEELTLKGVTQYYAYVTERQKVHCLNTL

FSRLQINQSIIFCNSSQRVELLAKKISQLGYSCFYIHAKMRQEHRNRVFHDFRNGLCRNLVCTDLFTRGIDIQAVNVVIN

FDFPKLAETYLHRIGRSGRFGHLGLAINLITYDDRFNLKSIEEQLGTEIKPIPSNIDKSLYVAEYHSEPVEDEKQ

>XP_008329300.1 probable ATP-dependent RNA helicase ddx6 [Cynoglossus semilaevis]

MATARTENVGTMVMGLNKQNGQLRGQTKPASVQPAPMTQAKAMGVPAKVGGAPQDGGGIKFGDDWKKSLKLPPKDNRVKT

SDVTSTKGNEFEDYCLKRELLMGIFEMGWEKPSPIQEESIPIALSGRDILARAKNGTGKSGAYLIPLLERIDLKKDYIQA

LVMVPTRELALQVSQICIQISKHLGGVKVMATTGGTNLRDDIMRLDETVHVVIATPGRILDLIKKGVAKVDRVQMMVMDE

ADKLLSQDFVVLIEDIISFLAKNRQILLYSATFPISVQKFMSKHLQKPYEINLMEELTLKGITQYYAYVTERQKVHCLNT

LFSRLQINQSIIFCNSTQRVELLAKKITQLGYSCFYIHAKMMQEYRNRVFHDFRNGLCRNLVCTDLFTRGIDIQAVNVVI

NFDFPKNAETYLHRIGRSGRFGHLGLAINLITSEDRFNLKAIEDQLVTDIKPIPGSIDKSLYVAEFHTSSGDCEVEEVEE

KAGLQQDSS

>XP_026203995.1 probable ATP-dependent RNA helicase ddx6 [Anabas testudineus]

MATARTEIVGPVVMGLSKQNGQLRGQTKPASVQPAPTTQGKALGPPQKAGNAPQDGGGIKFGDDWKKSLQLPPKDNRVKT

SDVTSTKGNEFEDYCLKRELLMGIFEMGWEKPSPVQEESIPIALSGRDILARAKNGTGKSGAYLIPLLERLDMKKDHIQA

IVMVPTRELALQVSQICIQISKHLGGVKVMATTGGTNLRDDIMRLDETVHVVIATPGRILDLIKKGVAKVDKVQMMVMDE

ADKLLSQDFVVLIEDIIGFLPKNRQILLYSATFPISVQKFMSKHLQKPYEINLMEELTLKGITQFYAYVTERQKVHCLNT

LFSRLQINQSIIFCNSTQRVELLAKKITQLGYSCFYIHAKMMQEYRNRVFHDFRNGLCRNLVCTDLFTRGIDIQAVNVVI

NFDFPKNAETYLHRIGRSGRFGHLGLAINLITSEDRFNLKAIEDQLVTDIKPIPSSIDKSLYVAEYHSTSTDCDVEEAEE

KPSRQQDST

>XP_026225882.1 probable ATP-dependent RNA helicase DDX6 [Anabas testudineus]

MAMARTANPAPMIGLNKPANVQLRGQTKPAGLHGTAQQPSALQKRSSIPQSSGGIMFGDDWKKCLELPPKDNRVKTSDVT

STKGNEFEDYCLKRELLMGIFEMGWEKPSPIQEESIPIALSGRDILARAKNGTGKSGAYLIPMLERIDLKKDHIQALVIV

PTRELALQVSQISIQLSKHLGGVKVMASTGGTNLRDDIMRLDETVHVVIATPGRILDLIKKGVAKVDKTQMMVMDEADKL

LSQDFVVLIEDMISFLPKDRQILLYSATFPISVQTFMSNHLKKPYEINLMEELTLKGITQYYAYVTERQKVHCLNTLFSR

LQINQSIIFCNSTQRVELLAKKITQLGYSCFYIHAKMMQEYRNRVFHDFRNGLCRNLVCTDLFTRGIDIQAVNVVINFDF

PKSAETYLHRIGRSGRFGHLGLAINLITSEDRYNLKTIEDQLGTDIKPIPSSIDKSLYVAEFHSVDPDDAKNKELEAA

>XP_034560473.1 probable ATP-dependent RNA helicase ddx6 [Notolabrus celidotus]

MATARTENVGPVVMGLNKQNGQIRGQTKPAPVQPAPTTQGKALGAPQIAGGAPQEGGGIKFGDDWKKSLKLPPKDNRVKT

SDVTSTKGNEFEDYCLKRELLMGIFEMGWEKPSPIQEESIPIALSGRDILARAKNGTGKSGAYLIPMLERIDLKKDHIQA

LVMVPTRELALQVSQISIQISKHLGGVKVMATTGGTNLRDDIMRLDETVHVVIATPGRILDLIKKGVAKVDKVQMMVMDE

ADKLLSQDFVVLIEDIISFLPKNRQILLYSATFPISVQKFMAKHLQKPYEINLMEELTLKGITQYYAYVTERQKVHCLNT

LFSRLQINQSIIFCNSTQRVELLAKKITQLGYSCFYIHAKMMQEYRNRVFHDFRNGLCRNLVCTDLFTRGIDIQAVNVVI

NFDFPKNAETYLHRIGRSGRFGHLGLAINLITSEDRFNLKAIEDQLVTDIKPIPSSIDKSLYVAEFHTSGGDCDVEEVEE

KPSRQKDST

>XP_020789999.1 probable ATP-dependent RNA helicase DDX6 [Boleophthalmus pectinirostris]

MATARTENVGPVVMGLNKQNGQLRGQTKPSVQSAPAPGKTLSAPQKSSIPQESGGIKFGDDWKKNLQLPPKDHRVRTSDV

TATKGNEFEDYCLKRELLMGIFEMGWEKPSPIQEESIPIALSGRDILARAKNGTGKSGAYLIPMLERIDLKKDHIQALVM

VPTRELALQVSQICIQISKHLGGVKVMATTGGTNLRDDIMRLDETVHIVIATPGRILDLIKKGVAKVDRVQMMIMDEADK

LLSQDFVVLIEDIISFLPKKRQILLYSATFPISVQRFMASHLQKPYEINLMEELTLKGITQYYAYVTERQKVHCLNTLFS

RLQINQSIIFCNSTQRVELLAKKITQLGYSCFYIHAKMMQEYRNRVFHDFRNGLCRNLVCTDLFTRGIDIQAVNVVINFD

FPKNAETYLHRIGRSGRFGHLGLAINLITSEDRFNLKAIEDQLVTDIKPIPSSIDKSLYVAEFHCEGDEGEEKHPQDTT

>XP_028288879.1 probable ATP-dependent RNA helicase ddx6 [Parambassis ranga]

MATARTENVGPVVMGLNKQNGQLRGQTKPASVQQAPTTQGKPLGALQKAGGAPQDGGGIKFGDDWKKSLKLPPKDNRVKT

SDVTSTKGNEFEDYCLKRELLMGIFEMGWEKPSPIQEESIPIALSGRDILARAKNGTGKSGAYLIPLLERIDLKKDHIQA

IVMVPTRELALQVSQICIQISKHLGGVKVMATTGGTNLRDDIMRLDETVHVVIATPGRILDLIKKGVAKVDRVQMMVMDE

ADKLLSQDFVVLIEDIISFLAKNRQILLYSATFPISVQKFMAKHLQKPYEINLMEELTLKGITQYYAYVTERQKVHCLNT

LFSRLQINQSIIFCNSTQRVELLAKKITQLGYSCFYIHAKMMQEYRNRVFHDFRNGLCRNLVCTDLFTRGIDIQAVNVVI

NFDFPKNAETYLHRIGRSGRFGHLGLAINLITSEDRFNLKAIEDQLVTDIKPIPSSIDKSLYVAEFHSANADCDVEEVEE

KPGRQQDST

>XP_031593911.1 probable ATP-dependent RNA helicase ddx6 [Oreochromis aureus]

MATARTENVGPVVMGLNKQNGQLRGQTKPAAVQPAPTTQVKTLGAPQKAGGASQDGGGIKFGDDWKKSLQLPPKDNRVKT

SDVTSTKGNEFEDYCLKRELLMGIFEMGWEKPSPIQEESIPIALSGRDILARAKNGTGKSGAYLIPLLERIDLKKDHIQA

VVMVPTRELALQMSQICIQLSKHLGGVKVMATTGGTNLRDDIMRLDETVHVVIATPGRILDLIKKGVAKVDKVQMMVMDE

ADKLLSQDFVVLIEDIISFLAKNRQILLYSATFPISVQKFMAKHLQKPYEINLMEELTLKGITQYYAYVTERQKVHCLNT

LFSRLQINQSIIFCNSTQRVELLAKKITQLGYSCFYIHAKMMQEYRNRVFHDFRNGLCRNLVCTDLFTRGIDIQAVNVVI

NFDFPKNAETYLHRIGRSGRFGHLGLAINLITSEDRFNLKAIEDQLVTDIKPIPSSIDKSLYVAEYHSTSGDCDVEEVEE

KRQQDST

>NXS18966.1 DDX6 helicase [Mystacornis crossleyi]

MSTARTENPVIMGLSSQNGQLRGPVKPSGGPGGGGTQTQQQMNQLKNANAINNGTQQQAQSMTTAIKPGDDWKKTLKLPP

KDLRIKTSDVTSTKGNEFEDYCLKRELLMGIFEMGWEKPSPIQEESIPIALSGRDILARAKNGTGKSGAYLIPLLERLDL

KKDNIQAMVIVPTRELALQVSQICIQVSKHMGGAKVMATTGGTNLRDDIMRLDDTVHVVIATPGRILDLIKKGVAKVEHV

QMIVLDEANKLLSQDFVQIMEDIILTLPKNRQILLYSATFPLSVQKFMNSHLQKPYEINLMEELTLKGVTQYYAYVTERQ

KVHCLNTLFSRLQINQSIIFCNSSQRVELLAKKISQLGYSCFYIHAKMRQEHRNLVFHDFXNLVCTDLFTRGIDIQAVNV

VINFDFPKLAETYLHRIGRSGRFGHLGLAINLITYDDRFNLKSIEEQLGTEIKPIPSNIDKSLYVAEYHSEPVEDEKQ

>XP_004569268.1 probable ATP-dependent RNA helicase ddx6 [Maylandia zebra]

MATARTESVGPVVMGLNKQNGQLRGQTKPAAVQPAPTTQGKTLGAPQKAGGASQDGGGIKFGDDWKKSLQLPPKDNRVKT

SDVTSTKGNEFEDYCLKRELLMGIFEMGWEKPSPIQEESIPIALSGRDILARAKNGTGKSGAYLIPLLERIDLKKDHIQA

IVMVPTRELALQMSQICIQLSKHLGGVKVMATTGGTNLRDDIMRLDETVHVVIATPGRILDLIKKGVAKVDKVQMMVMDE

ADKLLSQDFVVLIEDIISFLAKNRQILLYSATFPISVQKFMAKHLQKPYEINLMEELTLKGITQYYAYVTERQKVHCLNT

LFSRLQINQSIIFCNSTQRVELLAKKITQLGYSCFYIHAKMMQEYRNRVFHDFRNGLCRNLVCTDLFTRGIDIQAVNVVI

NFDFPKNAETYLHRIGRSGRFGHLGLAINLITSEDRFNLKAIEDQLVTDIKPIPSSIDKSLYVAEYHSTSGDCDVEEVEE

KRQQDST

>XP_013128042.1 probable ATP-dependent RNA helicase ddx6 [Oreochromis niloticus]

MATARTENVGPVVMGLNKQNGQLRGQTKPAAVQPAPTTQGKTLGAPQKAGGASQDGGGIKFGDDWKKSLQLPPKDNRVKT

SDVTSTKGNEFEDYCLKRELLMGIFEMGWEKPSPIQEESIPIALSGRDILARAKNGTGKSGAYLIPLLERIDLKKDHIQA

IVMVPTRELALQMSQICIQLSKHLGGVKVMATTGGTNLRDDIMRLDETVHVVIATPGRILDLIKKGVAKVDKVQMMVMDE

ADKLLSQDFVVLIEDIISFLAKNRQILLYSATFPISVQKFMAKHLQKPYEINLMEELTLKGITQYYAYVTERQKVHCLNT

LFSRLQINQSIIFCNSTQRVELLAKKITQLGYSCFYIHAKMMQEYRNRVFHDFRNGLCRNLVCTDLFTRGIDIQAVNVVI

NFDFPKNAETYLHRIGRSGRFGHLGLAINLITSEDRFNLKAIEDQLVTDIKPIPSSIDKSLYVAEYHSTSGDCDVEEVEE

KRQQDST

>XP_029022384.1 probable ATP-dependent RNA helicase ddx6 [Betta splendens]

MATTRTENVGPVVMGLNKQNGLRGQTKPASAQPAPTTQGKALGAPQKAGSAPQDGGGIKFGDDWKKSLQLPPKDNRVKTS

DVTATKGNEFEDYCLKRELLMGIFEMGWEKPSPIQEESIPIALSGRDILARAKNGTGKSGAYLIPLLERIDLKKDHIQAI

VMVPTRELALQVSQICIQISKHLGGVKVMATTGGTNLRDDIMRLDETVHVVIATPGRILDLIKKGVAKVDKVQMMVMDEA

DKLLSQDFVVLIEDIISFLPKNRQILLYSATFPISVQKFMAKHLQKPYEINLMEELTLKGITQFYAYVTERQKVHCLNTL

FSRLQINQSIIFCNSTQRVELLAKKITQLGYSCFYIHAKMMQEYRNRVFHDFRNGLCRNLVCTDLFTRGIDIQAVNVVIN

FDFPKNAETYLHRIGRSGRFGHLGLAINLITSEDRFNLKAIEDQLVTDIKPIPSSIDKSLYVAEYHSAGADCDVEEVEEK

PGRQQDST

>KAB0406084.1 hypothetical protein E2I00_019205 [Balaenoptera physalus]

MSTARTENPVIMGLSSQNGQLRGPVKPSGGPGGGGTQTQQQMNQLKNTNTINNGTQQQAQSMTTTIKPGDDWKKTLKLPP

KDLRIKTSDVTSTKGNEFEDYCLKRELLMGIFEMGWEKPSPIQEESIPIALSGRDILARAKNGTGKSGAYLIPLLERLDL

KKDNIQAMVIVPTRELALQVSQICIQVSKHMGGAKVMATTGGTNLRDDIMRLDDTVHVVIATPGRILDLIKKGVAKVDHV

QMIVLDEADKLLSQDFVQIMEDIILTLPKNRQILLYSATFPLSVQKFMNSHLQKPYEINLMEELTLKGVTQYYAYVTERQ

KVHCLNTLFSRVSLLGFSSCMLFGKVNEGFCFPSLCHRFVLFTPCAQLQINQSIIFCNSSQRVELLAKKISQLGYSCFYI

HAKMRQEHRNRVFHDFRNGLCRNLVCTDLFTRGIDIQAVNVVINFDFPKLAETYLHRIGRSGRFGHLGLAINLITYDDRF

NLKSIEEQLGTEIKPIPSNIDKSLYVAEYHSEPVEDEKP

>XP_030597771.1 probable ATP-dependent RNA helicase ddx6 [Archocentrus centrarchus]

MATARTENVGPLVMGLNKQNGQLRGQTKPAAVQPAPTTQGKTLGAPQKAGGASQDGGGIKFGDDWKKSLHLPPKDNRVKT

SDVTSTKGNEFEDYCLKRELLMGIFEMGWEKPSPIQEESIPIALSGRDILARAKNGTGKSGAYLIPLLERIDLKKDHIQA

IVMVPTRELALQVSQICIQLSKHLGGVKVMATTGGTNLRDDIMRLDETVHVVIATPGRILDLIKKGVAKVDKVQMMVMDE

ADKLLSQDFVVLIEDIISFLAKNRQILLYSATFPISVQKFMAKHLQKPYEINLMEELTLKGITQYYAYVTERQKVHCLNT

LFSRLQINQSIIFCNSTQRVELLAKKITQLGYSCFYIHAKMMQEYRNRVFHDFRNGLCRNLVCTDLFTRGIDIQAVNVVI

NFDFPKNAETYLHRIGRSGRFGHLGLAINLITSEDRFNLKAIEDQLVTDIKPIPSSIDKSLYVAEYHSASGDCDVEEVEE

KRQQDST

>XP_029927660.1 probable ATP-dependent RNA helicase ddx6 [Myripristis murdjan]

MATARTANPTSVMGLNKPANGQLKGQPKPANLQSIPSAAAQQPAGPQKGSSIPQSSGGIKFGDDWKKCLQLPPKDTRMKT

SDVTSTKGNEFEDYCLKRELLMGIFEMGWEKPSPVQEESIPIALSGRDILARAKNGTGKSGAYLIPLLERIDLKKDHIQA

IVMVPTRELALQMSQISIQLSKHLGGVKVMATTGGTNLRDDIMRLDETVHVIIATPGRILDLVKKGVAKVDKTQMMVMDE

ADKLLSQDFVILIEEIISFLPKDRQILLYSATFPVSVQKFMAKHLKKPYEINLMEELTLKGITQYYAYVTERQKVHCLNT

LFSRLQINQSIIFCNSTQRVELLAKKITQLGYSCFYIHAKMMQEYRNRVFHDFRNGLCRNLVCTDLFTRGIDIQAVNVVI

NFDFPKSAETYLHRIGRSGRFGHLGLAINLITSEDRFNLKGIEEQLVTDIKPIPSSIDKSLYVAEFHSVDPDDDGGDGEA

KQKELGAA

>XP_031151631.1 probable ATP-dependent RNA helicase ddx6 [Sander lucioperca]

MATARTENIGPVVMGLNKQNGQLRGQTKPASVQPAPTTQGKTLGASQIAGGAAQDGGGIKFGDDWKKSLKLPPKDNRVKT

SDVTSTKGNEFEDYCLKRELLMGIFEMGWEKPSPIQEESIPIALSGRDILARAKNGTGKSGAYLIPLLERIDLKKDHIQA

LVMVPTRELALQVSQISIQISKHLGGVKVMATTGGTNLRDDIMRLDETVHVVIATPGRILDLIKKGVAKVDRVQMMVMDE

ADKLLSQDFVVLIEDIISFLAKNRQILLYSATFPISVQKFMAKHLQKPYEINLMEELTLKGITQYYAYVTERQKVHCLNT

LFSRLQINQSIIFCNSTQRVELLAKKITQLGYSCFYIHAKMMQEYRNRVFHDFRNGLCRNLVCTDLFTRGIDIQAVNVVI

NFDFPKNAETYLHRIGRSGRFGHLGLAINLITSEDRFNLKAIEDQLVTDIKPIPSSIDKSLYVAEFHASGADCDVEEIEE

KPARQQDST

>XP_010739702.1 probable ATP-dependent RNA helicase ddx6 [Larimichthys crocea]

MATARTANPASMIGLNKPANGQFRGQTKPAGQQSGLFGTAQQLGPTQKMSSIPQSSGGIKFGDDWKKCLELPPKDTRMKT

SDVTSTKGNEFEDYCLKRELLMGIFEMGWEKPSPIQEESIPIALSGRDILARAKNGTGKSGAYLIPLLERIDLKKDNIQA

IVMVPTRELALQVSQISIQIGKHLGGVKVMATTGGTNLRDDIMRLDEIVHVVIATPGRILDLMKKGVAKVDKVQMMVMDE

ADKLLSQDFVVLIEDIIAFLPRERQILLYSATFPISVQKFMSKHLKKPYEINLMEELTLKGITQYYAYVTERQKVHCLNT

LFSRLQINQSIIFCNSTQRVELLAKKITQLGYSCFYIHAKMMQEYRNRVFHDFRNGLCRNLVCTDLFTRGIDIQAVNVVI

NFDFPKSAETYLHRIGRSGRFGHLGLAINLITSDDRYNLKNIEDQLVTEIKPIPGSIDKSLYVAEFHSVDPDEDGIEGGA

KNKEL

>XP_034747968.1 probable ATP-dependent RNA helicase ddx6 [Etheostoma cragini]

MATARTENIGPVVMGLNKQNGQLRGQTKPASVQQAPTTQGKTLGAPQVAGGAAQDGGGIKFGDDWKKSLKLPPKDNRVKT

SDVTSTKGNEFEDYCLKRELLMGIFEMGWEKPSPIQEESIPIALSGRDILARAKNGTGKSGAYLIPLLERIDLKKDHIQA

LVMVPTRELALQVSQISIQISKHLGGVKVMATTGGTNLRDDIMRLDETVHVVIATPGRILDLIKKGVAKVDRVQMMVMDE

ADKLLSQDFVVLIEDIISFLAKNRQILLYSATFPISVQKFMAKHLQKPYEINLMEELTLKGITQYYAYVTERQKVHCLNT

LFSRLQINQSIIFCNSTQRVELLAKKITQLGYSCFYIHAKMMQEYRNRVFHDFRNGLCRNLVCTDLFTRGIDIQAVNVVI

NFDFPKNAETYLHRIGRSGRFGHLGLAINLITSEDRFNLKAIEDQLVTDIKPIPSSIDKSLYVAEFHASGADCDVEEIEE

TPGRQQDTT

>XP_022613014.1 probable ATP-dependent RNA helicase ddx6 [Seriola dumerili]

MATARTENVGPVVMGLNKQNGQLRAGQTKPASVQPAPTTQGKALGALQKAGGAPQDGGGIKFGDDWKKSLKLPPKDNRVK

TSDVTSTKGNEFEDYCLKRELLMGIFEMGWEKPSPIQEESIPIALSGRDILARAKNGTGKSGAYLIPLLERIDLKKDHIQ

AIVMVPTRELALQVSQICIQISKHLGGVKVMATTGGTNLRDDIMRLDETVHVVIATPGRILDLIKKGVAKVDRAQMMVMD

EADKLLSQDFVVLIEDIISFLAKNRQILLYSATFPISVQKFMAKHLQKPYEINLMEELTLKGITQFYAYVTERQKVHCLN

TLFSRLQINQSIIFCNSTQRVELLAKKITQLGYSCFYIHAKMMQEYRNRVFHDFRNGLCRNLVCTDLFTRGIDIQAVNVV

INFDFPKNAETYLHRIGRSGRFGHLGLAINLITSEDRFNLKAIEDQLVTDIKPIPGSIDKSLYVAEYHSSSADCDVEEVE

EKPGRQQDST

>XP_035527706.1 probable ATP-dependent RNA helicase ddx6 [Morone saxatilis]

MATARTANPAPMIGLNKPNGQFRGQNKPAGQQSGLFATAQHPSAPQKMTMSIPQSSGGIKFGDDWKKCLELPPKDTRMKT

SDVTSTKGNEFEDYCLKRELLMGIFEMGWEKPSPIQEESIPIALSGRDILARAKNGTGKSGAYLIPLLERIDLKKDHIQA

IVMVPTRELALQVSQISIQISKHLGGVKVMATTGGTNLRDDIMRLDEIVHVVIATPGRILDLMKKGVAKVDKAQMMVMDE

ADKLLSQDFVVLIEDIISFLPKERQILLYSATFPISVQKFMNKHLKKPYEINLMEELTLKGITQYYAYVTERQKVHCLNT

LFSRLQINQSIIFCNSTQRVELLAKKITQLGYSCFYIHAKMMQEYRNRVFHDFRNGLCRNLVCTDLFTRGIDIQAVNVVI

NFDFPKNAETYLHRIGRSGRFGHLGLAINLITSDDRYNLKNIEDQLVTEIKPIPGSIDKSLYVAEFHSVDPDDDDIEGGA

MNKEL

>XP_033492761.1 probable ATP-dependent RNA helicase ddx6 [Epinephelus lanceolatus]

MATARTENVGPVVMGLNKQNGQLRGQTKPASVQPAPTTQGKALGAPQIAGGAAQDGGGIKFGDDWKKSLKLPPKDNRVKT

SDVTSTKGNEFEDYCLKRELLMGIFEMGWEKPSPIQEESIPIALSGRDILARAKNGTGKSGAYLIPLLERIDLKKDHIQA

LVMVPTRELALQVSQISIQISKHLGGVKVMATTGGTNLRDDIMRLDETVHVVIATPGRILDLIKKGVAKVDRVQMMVMDE

ADKLLSQDFVVLIEDIISFLAKNRQILLYSATFPISVQKFMAKHLQKPYEINLMEELTLKGITQYYAYVTERQKVHCLNT

LFSRLQINQSIIFCNSTQRVELLAKKITQLGYSCFYIHAKMMQEYRNRVFHDFRNGLCRNLVCTDLFTRGIDIQAVNVVI

NFDFPKNAETYLHRIGRSGRFGHLGLAINLITSEDRFNLKAIEDQLVTDIKPIPSSIDKSLYVAEFHSSSADCDVEEIEE

KPGRQQDST

>XP_026156944.1 probable ATP-dependent RNA helicase ddx6 [Mastacembelus armatus]

MATARTETVGPVVMGLNKQNGQLRGQTTKPASVQTAPTTQGKALGALQKAGSAPQDTGGIKFGDDWKKSLKLPPKDNRVK

TSDVTATKGNEFEDYCLKRELLMGIFEMGWEKPSPIQEESIPIALSGRDILARAKNGTGKSGAYLIPLLERIDLKKDHIQ

AIVMVPTRELALQVSQICIQISKHLGGVKVMATTGGTNLRDDIMRLDETVHVVIATPGRLLDLIKKGVAKVDKVQMMVMD

EADKLLSQDFVVLIEDIISFLAKNRQILLYSATFPISVQKFMAKHLQKPYEINLMEELTLKGITQFYAYVTERQKVHCLN

TLFSRLQINQSIIFCNSTQRVELLAKKITQLGYSCFYIHAKMMQEYRNRVFHDFRNGLCRNLVCTDLFTRGIDIQAVNVV

INFDFPKNAETYLHRIGRSGRFGHLGLAINLITSEDRFNLKAIEDQLVTDIKPIPSSIDKSLYVAEFHSTSADCDVEEAE

EKPGRQQDST

>XP_029298732.1 probable ATP-dependent RNA helicase DDX6 isoform X1 [Cottoperca gobio]

MATARTENVGPVIMGLNKQNGQLRGQTKPASVQPAPTMSKALGAHQIVGGAAQDGGGIKFGDDWKKSLKLPPKDTRVKTS

DVTSTKGNEFEDYCLKRELLMGIFEMGWEKPSPIQEESIPIALSGRDILARAKNGTGKSGAYLIPLLERIDLKKDHIQAL

VMVPTRELALQVSQISIQISKHLGGVKVMATTGGTNLRDDIMRLDETVHVVIATPGRILDLIKKGVAKVDRVQMMVMDEA

DKLLSQDFVVLIEDIISFLAKNRQILLYSATFPISVQKFMAKHLQKPYEINLMEELTLKGITQYYAYVTERQKVHCLNTL

FSRLQINQSIIFCNSTQRVELLAKKITQLGYSCFYIHAKMMQEYRNRVFHDFRNGLCRNLVCTDLFTRGIDIQAVNVVIN

FDFPKNAETYLHRIGRSGRFGHLGLAINLITSEDRFNLKSIEDQLVTDIKPIPSSIDKSLYVAEFHTSGADCDVEEIEEK

PGRQQDGT

>XP_018555309.1 PREDICTED: probable ATP-dependent RNA helicase ddx6 [Lates calcarifer]

MATARTENVGPVVMGLNKQNGQLRGQTKPASVQPAPTTQGKATGAPQKAGGAPQDGGGIKFGDDWKKSLKLPPKDNRVKT

SDVTSTKGNEFEDYCLKRELLMGIFEMGWEKPSPIQEESIPIALSGRDILARAKNGTGKSGAYLIPLLERIDLKKDHIQA

IVMVPTRELALQVSQICIQISKHLGGVKVMATTGGTNLRDDIMRLDETVHVVIATPGRILDLIKKGVAKVDKVQMMVMDE

ADKLLSQDFVVLIEDIISFLAKNRQILLYSATFPISVQKFMAKHLQKPYEINLMEELTLKGITQFYAYVTERQKVHCLNT

LFSRLQINQSIIFCNSTQRVELLAKKITQLGYSCFYIHAKMMQEYRNRVFHDFRNGLCRNLVCTDLFTRGIDIQAVNVVI

NFDFPKNAETYLHRIGRSGRFGHLGLAINLITSEDRFNLKAIEDQLVTDIKPIPSSIDKSLYVAEYHSSSGDCDVEEVEE

KPGRQQDST

>XP_030225484.1 probable ATP-dependent RNA helicase ddx6 [Gadus morhua]

MATARTANPSSMMGLNKPANGQLRGQPKPVGLHPSIPQLGAPQKIGSIPQSSGGIKFGDDWKRSLHLPPKDTRVRTSDVT

STKGNEFEDYCLKRELLMGIFEMGWEKPSPIQEESIPIALSGRDILARAKNGTGKSGAYLIPLLERIDLKKDFIQAIVMV

PTRELALQVSQISIQVAKHLGGVKVMATTGGTNLRDDIMRLDETVHVVIATPGRVLDLIKKGVAKVDRTQMMVMDEADKL

LSQDFVVLIEDIISFLPRERQILLYSATFPTSVQKFMNKHLKKPYEINLMEELTLKGITQYYAYVTERQKVHCLNTLFSR

LQINQSIIFCNSTQRVELLAKKITQLGYSCFYIHAKMMQEYRNRVFHDFRNGLCRNLVCTDLFTRGIDIQAVNVVINFDF

PKNAETYLHRIGRSGRFGHLGLAINLITSEDRFNLKGIEDQLVTDIKPIPGSIDKSLYVAEFHSVNPDDDDDDGGIEGKH

KGLGAI

>XP_032391137.1 probable ATP-dependent RNA helicase ddx6 [Etheostoma spectabile]

MATARTENIGPVVMGLNKQNGQLRGQTKPASVQQAPTTQGKTLGAPQIAGGAAQDGGGIKFGDDWKKSLKLPPKDNRVKT

SDVTSTKGNEFEDYCLKRELLMGIFEMGWEKPSPIQEESIPIALSGRDILARAKNGTGKSGAYLIPLLERIDLKKDHIQA

LVMVPTRELALQVSQISIQISKHLGGVKVMATTGGTNLRDDIMRLDETVHVVIATPGRILDLIKKGVAKVDRVQMMVMDE

ADKLLSQDFVVLIEDIISFLAKNRQILLYSATFPISVQKFMAKHLQKPYEINLMEELTLKGITQYYAYVTERQKVHCLNT

LFSRLQINQSIIFCNSTQRVELLAKKITQLGYSCFYIHAKMMQEYRNRVFHDFRNGLCRNLVCTDLFTRGIDIQAVNVVI

NFDFPKNAETYLHRIGRSGRFGHLGLAINLITSEDRFNLKAIEDQLVTDIKPIPSSIDKSLYVAEFHASGTDCDVEEIEE

TPGRQQDTT

>XP_029369448.1 probable ATP-dependent RNA helicase ddx6 [Echeneis naucrates]

MATARTENAGPVVMGLNKQNGQLRGQTKPASVQPAPTTQGKALGASQKAGGVPQDGGGIKFGDDWKRSLKLPPKDNRVKT

SDVTSTKGNEFEDYCLKRELLMGIFEMGWEKPSPIQEESIPIALSGRDILARAKNGTGKSGAYLIPLLERIDLKKDHIQA

IVMVPTRELALQVSQICIQISKHLGGVKVMATTGGTNLRDDIMRLDETVHVVIATPGRILDLIKKGVAKVDRVQMMVMDE

ADKLLSQDFVVLIEDIISFLAKNRQILLYSATFPISVQKFMAKHLQKPYEINLMEELTLKGITQFYAYVTERQKVHCLNT

LFSRLQINQSIIFCNSTQRVELLAKKITQLGYSCFYIHAKMMQEYRNRVFHDFRNGLCRNLVCTDLFTRGIDIQAVNVVI

NFDFPKNAETYLHRIGRSGRFGHLGLAINLITSEDRFNLKAIEDQLVTDIKPIPSSIDKSLYVAEYHSTSADCDVDEVEE

KPGHQQEST

>XP_023117000.1 probable ATP-dependent RNA helicase ddx6 [Amphiprion ocellaris]

MATARTESVGPVVMGLNKQNGQLRGQTKPAPASPAPTTQGKTLGAPQKAGGGPQDGGGIKFGDDWKKSLKLPPKDNRVKT

SDVTATKGNEFEDYCLKRELLMGIFEMGWEKPSPIQEESIPIALSGRDILARAKNGTGKSGAYLIPLLERIDLKKDHIQA

MVMVPTRELALQVSQICIQISKHLGGVKVMATTGGTNLRDDIMRLDETVHVVIATPGRILDLIKKGVAKVDRVQMMVMDE

ADKLLSQDFVVLIEDIISFLAKNRQILLYSATFPISVQKFMAKHLQKPYEINLMEELTLKGITQFYAYVTERQKVHCLNT

LFSRLQINQSIIFCNSTQRVELLAKKITQLGYSCFYIHAKMMQEYRNRVFHDFRNGLCRNLVCTDLFTRGIDIQAVNVVI

NFDFPKNAETYLHRIGRSGRFGHLGLAINLITSEDRFNLKAIEDQLVTDIKPIPSSIDKSLYVAEFHSGSADCDVEEVEE

KPGRQQDST

>XP_029976128.1 probable ATP-dependent RNA helicase ddx6 [Salarias fasciatus]

MATARTESVGPLVMGLNKQNGQLKGQTKPASVPPAPTTQGKSVGAAQGGGGGVKFGDDWKKSLKLPPKDNRVKTSDVTST

KGNEFEDYCLKRELLMGIFEMGWEKPSPIQEESIPIALSGRDILARAKNGTGKSGAYLIPLLERIDLKKDHIQAMVMVPT

RELALQVSQICIQISKHLGGVKVMATTGGTNLRDDIMRLDETVHVVIATPGRILDLIKKGVAKVDRVQMMVMDEADKLLS

QDFVVLIEDIISFLAKNRQILLYSATFPISVQKFMAKHLQKPYEINLMEELTLKGITQFYAYVTERQKVHCLNTLFSRLQ

INQSIIFCNSTQRVELLAKKITQLGYSCFYIHAKMMQEYRNRVFHDFRNGLCRNLVCTDLFTRGIDIQAVNVVINFDFPK

NAETYLHRIGRSGRFGHLGLAINLITSEDRFNLKAIEDQLVTDIKPIPGSIDKSLYVAEYHSGSADCEVEEVEEKPGRPQ

DT

>XP_020506001.1 probable ATP-dependent RNA helicase ddx6 [Labrus bergylta]

MATAKTENPATMFGLNKPANGQLRGQTKPAGQQSGVLVPLQQPNALQKRTTIPQSSGGIKFGDDWKKCLALPPKDTRMRT

SDVTSTKGNEFEDYCLKRELLMGIFEMGWEKPSPIQEESIPIALSGRDILARAKNGTGKSGAYLIPLLERIDLKKDHIQA

IVMVPTRELALQMSQISIQLSKHLGGVKVMATTGGTNLRDDIMRLDETVHVVIATPGRVLDLIRKGVAKVDKARMMVMDE

ADKLLSQDFVVLIEDIISYLPKDRQILLYSATFPISVQTFMSKHLEKPYEINLMEELTLKGITQYYAYVTERQKVHCLNT

LFSRLQINQSIIFCNSTQRVELLAKKITQLGYSCFYIHAKMMQEYRNRVFHDFRNGLCRNLVCTDLFTRGIDIQAVNVVI

NFDFPKSAETYLHRIGRSGRFGHLGLAINLITSEDRYNLKNIEDQLVTEIKPIPGSIDKSLYVAEFHSVDPDDEGKNGDF

GAI

>XP_028453344.1 probable ATP-dependent RNA helicase ddx6 [Perca flavescens]

MATARTENIGPVVMGLNKQNGQLRGQTKPASVQPAPTTQGKTLGAPQIAGGAAQDGGGIKFGDDWKKSLKLPPKDTRVKT

SDVTSTKGNEFEDYCLKRELLMGIFEMGWEKPSPIQEESIPIALSGRDILARAKNGTGKSGAYLIPLLERIDLKKDHIQA

LVMVPTRELALQVSQISIQISKHLGGVKVMATTGGTNLRDDIMRLDETVHVVIATPGRILDLIKKGVAKVDRVQMMVMDE

ADKLLSQDFVVLIEDIISFLAKNRQILLYSATFPISVQKFMAKHLQKPYEINLMEELTLKGITQYYAYVTERQKVHCLNT

LFSRLQINQSIIFCNSTQRVELLAKKITQLGYSCFYIHAKMMQEYRNRVFHDFRNGLCRNLVCTDLFTRGIDIQAVNVVI

NFDFPKNAETYLHRIGRSGRFGHLGLAINLITSEDRFNLKAIEDQLVTDIKPIPSSIDKSLYVAEFHASGADCDVEEIEE

KPGRKQDST

>TMS17939.1 putative ATP-dependent RNA helicase DDX6 [Larimichthys crocea]

MATARTETVGPVVMGLNKQNGQLRGGPTKPASVQPAPTTQGKSLGAPQIAGGAAQDGGGIKFGDDWKKSLKLPPKDNRVK

TSDVTSTKGNEFEDYCLKRELLMGIFEMGWEKPSPIQEESIPIALSGRDILARAKNGTGKSGAYLIPLLERIDLKKDHIQ

ALVMVPTRELALQVSQISIQISKHLGGVKVMATTGGTNLRDDIMRLDETVHVVIATPGRILDLIKKGVAKVDRVQMMVMD

EADKLLSQDFVVLIEDIISFLAKNRQILLYSATFPISVQKFMAKHLQKPYEINLMEELTLKGITQYYAYVTERQKVHCLN

TLFSRLQINQSIIFCNSTQRVELLAKKITQLGYSCFYIHAKMMQEYRNRVFHDFRNGLCRNLVCTDLFTRGIDIQAVNVV

INFDFPKNAETYLHRIGRSGRFGHLGLAINLITSEDRFNLKAIEDQLVTDIKPIPGSIDKSLYVA

>XP_030267269.1 probable ATP-dependent RNA helicase ddx6 [Sparus aurata]

MATARTENVGPVVMGLNKQNGQLRGQTKPASVQPASTTQGKALGAPQIAGGAAQDGGGIKFGDDWKKSLKLPPKDTRVKT

SDVTSTKGNEFEDYCLKRELLMGIFEMGWEKPSPIQEESIPIALSGRDILARAKNGTGKSGAYLIPLLERIDLKKDHIQA

LVMVPTRELALQVSQISIQISKHLGGVKIMATTGGTNLRDDIMRLDETVHVVIATPGRILDLIKKGVAKVDRVQMMVMDE

ADKLLSQDFVVLIEDIISFLAKNRQILLYSATFPISVQKFMAKHLQKPYEINLMEELTLKGITQYYAYVTERQKVHCLNT

LFSRLQINQSIIFCNSTQRVELLAKKITQLGYSCFYIHAKMMQEYRNRVFHDFRNGLCRNLVCTDLFTRGIDIQAVNVVI

NFDFPKNAETYLHRIGRSGRFGHLGLAINLITSEDRFNLKAIEDQLVTDIKPIPGSIDKSLYVAEFHSSGGDCDVEEVEE

KSGHQQDSA

>XP_017260775.1 probable ATP-dependent RNA helicase ddx6 [Kryptolebias marmoratus]

MATARTESVGPVVMGLNKQNGQLRGQNKPASVQPASTAPGKSLGAPQKAGGANKDGGGIKFGDDWKKSLQLPPKDNRVKT

SDVTATKGNEFEDYCLKRELLMGIFEMGWEKPSPIQEESIPIALSGRDILARAKNGTGKSGAYLIPLLERIDLKKDHIQA

MVMVPTRELALQVSQICIQISKHLGGVKVMATTGGTNLRDDIMRLDETVHVVIATPGRILDLIKKGVAKVDRVQMMVMDE

ADKLLSQDFVVLIEDIISFLAKNRQILLYSATFPISVQKFMVKHLQKPYEINLMEELTLKGITQFYAYVTERQKVHCLNT

LFSRLQINQSIIFCNSTQRVELLAKKITQLGYSCFYIHAKMMQEYRNRVFHDFRNGLCRNLVCTDLFTRGIDIQAVNVVI

NFDFPKNAETYLHRIGRSGRFGHLGLAINLITSEDRFNLKAIEEQLVTDIKPIPSSIDKSLYVAEYHSGGDCEVEEKAER

QQDST

>XP_023995599.1 LOW QUALITY PROTEIN: probable ATP-dependent RNA helicase DDX6 [Salvelinus alpinus]

MSTASTHNPLILGHSSQNGQLRGPVVKVAGGQGSGGGSHQTHQSSQMKQASSTINNGTGPSSTQLAKATPTANTVIKPGD

DWKRNLTLPPKDMRMRTSDVTATKGNEFEDYCLKRELLXGIFEVGWEKPSPIQEESIPIALSGRDILARAKNGTGKSGAY

LIPLLERIDLKKDCIQAMGIVPTRELALQVSQICIQISKHMGGVKVMATTGGTNLRDDIMRLDETVHVVIATPGRILDLI

KKGVAKVNQVQMIVLDEADKLLSQDFVVMMDEVLSYLPKQRQILLYSATFPLSVQKFMNSHLSKPYEINLMEELTLKGVT

QYYAYVTERQKVHCLNTLFSRLQINQSIIXCNSSQRVELLAKDVSQLGYSCFLHPAKMRQEHRNRVFHDFRNGLCRNLVC

TDLFTRGIDIQAVNVVINFDFPKLGETYLHRIGRSGRFGHLGLAINLITYDDRFNLKGIEEQLGTEIRPIPGSIDKSLYV

AEYHSENGEEVKL

>XP_008284012.1 PREDICTED: probable ATP-dependent RNA helicase ddx6 [Stegastes partitus]

MATARTENVGPVVMGLNKQNGQLRGQTKPASVPPAPTTQGKTLGAPQKAGGAPQDGGGIKFGDDWKKSLKLPPKDNRVKT

SDVTATKGNEFEDYCLKRELLMGIFEMGWEKPSPIQEESIPIALSGRDILARAKNGTGKSGAYLIPLLERIDLKKDHIQA

IVMVPTRELALQVSQICIQISKHLGGVKVMATTGGTNLRDDIMRLDETVHVVIATPGRILDLIKKGVAKVDRVQMMVMDE

ADKLLSQDFVVLIEDIISFLAKNRQILLYSATFPISVQKFMAKHLQKPYEINLMEELTLKGITQFYAYVTERQKVHCLNT

LFSRLQINQSIIFCNSTQRVELLAKKITQLGYSCFYIHAKMMQEYRNRVFHDFRNGLCRNLVCTDLFTRGIDIQAVNVVI

NFDFPKNAETYLHRIGRSGRFGHLGLAINLITSEDRFNLKAIEDQLVTDIKPIPSSIDKSLYVAEYHSTGADCDVEEVEE

KPGRQQDST

>XP_030202931.1 probable ATP-dependent RNA helicase ddx6 [Gadus morhua]

MATAKTESTAAAVLMGLNKQNGQLRGQPKPATLLSGPQVQGKAVALQQKTGGPPQEGGGGGGGGIKFGDDWKTSLHLPPK

DTRVRTSDVTSTKGNEFEDYCLKRELLMGIFEMGWEKPSPIQEESIPIALSGRDILARAKNGTGKSGAYLIPLLERIDLK

KDYIQAMVMVPTRELALQVSQICIQISKHMGGVKVMATTGGTNLRDDIMRLDETVHVVIATPGRILDLIKKGVAKVDRVQ

MMVMDEADKLLSQDFVVLIEDIISFLAKSRQILLYSATFPISVQKFMNKHLQKPYEINLMEELTLKGITQYYAYVTERQK

VHCLNTLFSRLQINQSIIFCNSTQRVELLAKKITQLGYSCFYIHAKMMQEYRNRVFHDFRNGLCRNLVSTDLFTRGIDIQ

AVNVVINFDFPKNAETYLHRIGRSGRFGHLGLAINLITSEDRYNLKSIEDQLMTDIKPIPSCIDKSLYVAEFHSSNPDCE

VEEVDERSGRQQD

>XP_035537690.1 probable ATP-dependent RNA helicase ddx6 [Morone saxatilis]

MATARTENVGPVVMGLNKQNGQLRGQTKPASVQPAPTTQGKALGAPQIGGGASQDGGGIKFGDDWKKSLKLPPKDNRVKT

SDVTSTKGNEFEDYCLKRELLMGIFEMGWEKPSPIQEESIPIALSGRDILARAKNGTGKSGAYLIPLLERIDLKKDHIQA

LVMVPTRELALQVSQISIQISKHLGGVKVMATTGGTNLRDDIMRLDETVHVVIATPGRILDLIKKGVAKVDRVQMMVMDE

ADKLLSQDFVVLIEDIISFLAKNRQILLYSATFPISVQKFMAKHLQKPYEINLMEELTLKGITQYYAYVTERQKVHCLNT

LFSRLQINQSIIFCNSTQRVELLAKKITQLGYSCFYIHAKMMQEYRNRVFHDFRNGLCRNLVCTDLFTRGIDIQAVNVVI

NFDFPKNAETYLHRIGRSGRFGHLGLAINLITSEDRFNLKAIEDQLVTDIKPIPGSIDKSLYVAEYHSSGADCDVEEVEE

KPGHHQDST

>XP_028985379.1 probable ATP-dependent RNA helicase DDX6 [Betta splendens]

MATTRTANPAPMFGFNKPANGQLVGQAKSDGPQSGPTGNAQHPSALQKRSSFPQSSGGIKFGDDWKKCLELPAKDNRVKT

SDVTATKGNEFEDYCLKRELLMGIFEMGWEKPSPIQEESIPIALSGRDILARAKNGTGKSGAYLIPMLERIDVKKNHIQA

IVMVPTRELALQVSQISIQLGKHMAGIKVMATTGGTNLRDDIMRLDDTVHVVIATPGRILDLMKKGVAKVDKAQMMVMDE

ADKLLSQDFVVLIEDIISFLPKDRQILLYSATFPISVQKFMSNHLKKPYEINLMEELTLKGITQYYAYVTERQKVHCLNT

LFSKLQINQSIIFCNSTQRVELLAKKITQLGYSCFYIHAKMMQEYRNRVFHDFRNGLCRNLVSTDLFTRGIDIQAVNVVI

NFDFPKSAETYLHRIGRSGRFGHLGLAINLITSDDRYNLKTIEDQLGTDIKPIPGSIDKSLYVAEFHSVDPEEDECDDDK

AKNKELGAA

>KAF7686266.1 hypothetical protein HF521_015628 [Silurus meridionalis]

MATAKMENVGPVVMGLGKQNGQLRGLNSQSGPNVQGNPFARVSTGGQKPAADSQDGPGIRFGEDWKKSLQLPPKDHRVKT

SDVTSTKGNEFEDYCLKRELLMGIFEMGWEKPSPVQEESIPIALSGRDILARAKNGTGKSGAYLIPMLERIDLKKDYIQA

IVMVPTRELALQVSQISIELSKHLGGVKVMATTGGTNLRDDIMRLDETVHVIIATPGRILDLMKKGVAKVDKVQIMVMDE

ADKLLSQDFVILIEDIISFLDRKRQILLYSATFPSSVQKFMMKHLKKPYEINLMEELTLKGITQYYAYVTERQKVHCLNT

LFSRLQINQSIIFCNSTQRVELLAKKITQLGYSCFYIHAKMMQEYRNRVFHDFRNGLCRNLVCTDLFTRGIDIQAVNVVI

NFDFPKTAETYLHRIGRSGRFGHLGLAINLITAEDRFNLKAIEDQLVTDIKPIPGSIDKSLYVAEFHSANDDAEEQETPE

DP

>XP_030248414.1 probable ATP-dependent RNA helicase ddx6 [Sparus aurata]

MATARTANPAPMIGLNKPANGQFRGQTKPAGQLPGLLAPAQQPSGPMRMSSIPQSSGGIKFGDDWKKCLELPPKDTRMRT

SDVTSTKGNEFEDYCLKRELLMGIFEMGWEKPSPIQEESIPIALSGRDILARAKNGTGKSGAYLIPLLERIDLKKDFIQA

IVLVPTRELALQVSQISIQISKHLGGVKVMATTGGTNLRDDIMRLDETVHVVIATPGRILDLIKKGVAKVERAQMIVMDE

ADKLLSQDFVVIIEDIISFFPKERQILLYSATFPISVQKFMSKHLKKPYEINLMEELTLKGITQYYAYVTERQKVHCLNT

LFSRLQINQSIIFCNSTQRVELLAKKITQLGYSCFYIHAKMMQEYRNRVFHDFRNGLCRNLVCTDLFTRGIDIQAVNVVI

NFDFPKSAETYLHRIGRSGRFGHLGLAINLITCDDRYNLKNIEDQLVTEIKPIPGSIDKSLYVAEFHSVDPDDDEDDDGI

EGGAKIKEL

>XP_008302149.1 PREDICTED: probable ATP-dependent RNA helicase ddx6 [Stegastes partitus]

MATARTANPAPMIGLNGQLRGQTKSAAQQAGLHSTVQQLGAPQERTSIPQSSGGIRFGDDWKKLLAVPPKDTRVKTSDVT

STKGNEFEDYCLKRELLMGIFEMGWEKPSPIQEESIPIALSGRDILARAKNGTGKSGAYLIPLLERIDLKKDHIQAIVMV

PTRELALQMSQISIQLSKHLGGVKVMATTGGTNLRDDILRLDETVHVVIATPGRILDLMKKGVAKVDKTQMMVMDEADKL

LSQDFVVLIEDIISFLPKNRQILLYSATFPISVQKFMSKHLQKPYEINLMEELTLKGITQYYAYVTERQKVHCLNTLFSR

LQINQSIIFCNSTQRVELLAKKITQLGYSCFYIHAKMMQEYRNRVFHDFRNGLCRNLVCTDLFTRGIDIQAVNVVINFDF

PKNAETYLHRIGRSGRFGHLGLAINLITSDDRYNLKTIEDQLITDIKPIPSCIDKSLYVAEFHSVDPDDDGDEGGAKNKE

LGAA

>XP_021526869.1 LOW QUALITY PROTEIN: probable ATP-dependent RNA helicase DDX6 [Aotus nancymaae]

MSTARTENPVIMGLSSQNGQLRGPVKPSGGPGGGGTQTQQQMNQLKNTNTINNGTQQQAQSMTTTIKPGDDWKKTLKLPP

KDLRIKTSDVTSTKGNEFEDYCLKRELLMGIFEMGWEKPSPIQEESIPIALSGRDILARAKNGTGKSGAYLIPLLERLDL

KKDNIQAMVIVPTRELALQVSQICIQVSKHMGGAKVMATTGGTNLRDDIMRLDDTVHVVIATPGRILDLIKKGVAKVDHV

QMIVLDEADKLXSQDFVQIREDIILTLPKKQADLTIFRYFPLSVQKFMNSHLQKPYEINLMEELTLKGVTQYYAYVTERQ

KVHCLNTLFSRLQINQSIIFCNSSQRVELLAKKISQLGYSCFYIHAKMRQEHRNRVFHDFRNGLCRNLVCTDLFTRGIDI

QAVNVVINFDFPKLAETYLHRIGRSGRFGHLGLAINLITYDDRFNLKSIEEQLGTEIKPIPSNIDKSLYVAEYHSEPVED

EKP

>XP_034400668.1 probable ATP-dependent RNA helicase ddx6 [Cyclopterus lumpus]

MATARTENVGPIVMGLNKQNGQLRGQTKSASVQPAPTTLGKALGAPQIVGGAAQEGGGIKFGDDWKKSLKLPPKDNRVKT

SDVTSTKGNEFEDYCLKRELLMGIFEMGWEKPSPIQEESIPIALSGRDILARAKNGTGKSGAYLIPLLERIDLKKDHIQA

LVMVPTRELALQVSQISIQISKHLGGVKVMATTGGTNLRDDIMRLDETVHVVIATPGRILDLIKKGVAKVDRVQIMVMDE

ADKLLSQDFVVLIEDIISFLAKNRQILLYSATFPISVQKFMAKHLQKPYEINLMEELTLKGITQYYAYVTERQKVHCLNT

LFSRLQINQSIIFCNSTQRVELLAKKITQLGYSCFYIHAKMMQEYRNRVFHDFRNGLCRNLVCTDLFTRGIDIQAVNVVI

NFDFPKNAETYLHRIGRSGRFGHLGLAINLITSEDRFNLKAIEDQLVTDIKPIPSSIDKSLYVAEFHTSGADCDVEEIEE

KPGRQQDRA

>XP_010790679.1 PREDICTED: probable ATP-dependent RNA helicase ddx6 [Notothenia coriiceps]

MATARTENSGPVIMGLNKQNGQLRGQTKPAPVQPAPTTQGKAPAAPQTEGGAAQDGGGIKFGDDWKKSLKLPPRDDRVRT

SDVTSTKGNEFEDYCLKRELLMGIFEMGWEKPSPIQEESIPIALSGRDILARAKNGTGKSGAYLIPMLERIDLKKDYIQA

LVMVPTRELALQVSQISIQISKHLGGVKVMATTGGTNLRDDIMRLDETVHVVIATPGRILDLIKKGVAKVDRVQMMVMDE

ADKLLSQDFVVLIEDIISFLAKHRQILLYSATFPISVQKFMSKHLQKPYEINLMEELTLKGITQYYAYVTERQKVHCLNT

LFSRLQINQSIIFCNSTQRVELLAKKITQLGYSCFYIHAKMMQEYRNRVFHDFRNGLCRNLVCTDLFTRGIDIQAVNVVI

NFDFPKNAETYLHRIGRSGRFGHLGLAINLITSEDRFNLKSIEDQLVTDIKPIPSSIDKSLYVAEFHTSGGDCDVEEIEE

KPGRQQDGK

>XP_033980500.1 probable ATP-dependent RNA helicase ddx6 [Trematomus bernacchii]

MATARTENSGPVLMGLNKQNGQLRGQTKPAPVQPAPTTQGKAPAAPQTVGGAAQDGGGIKFGDDWKKSLKLPPRDDRVRT

SDVTSTKGNEFEDYCLKRELLMGIFEMGWEKPSPIQEESIPIALSGRDILARAKNGTGKSGAYLIPMLERIDLKKDYIQA

LVMVPTRELALQVSQISIQISKHLGGVKVMATTGGTNLRDDIMRLDETVHVVIATPGRILDLIKKGVAKVDRVQMMVMDE

ADKLLSQDFVVLIEDIISFLAKHRQILLYSATFPISVQKFMSKHLQKPYEINLMEELTLKGITQYYAYVTERQKVHCLNT

LFSRLQINQSIIFCNSTQRVELLAKKITQLGYSCFYIHAKMMQEYRNRVFHDFRNGLCRNLVCTDLFTRGIDIQAVNVVI

NFDFPKNAETYLHRIGRSGRFGHLGLAINLITSEDRFNLKSIEDQLVTDIKPIPSSIDKSLYVAEFHTSGGDCDVEEIEE

KPGRQQDGK

>XP_019109709.1 probable ATP-dependent RNA helicase ddx6 [Larimichthys crocea]

MATARTETVGPVVMGLNKQNGQLRGGPTKPASVQPAPTTQGKSLGAPQIAGGAAQDGGGIKFGDDWKKSLKLPPKDNRVK

TSDVTSTKGNEFEDYCLKRELLMGIFEMGWEKPSPIQEESIPIALSGRDILARAKNGTGKSGAYLIPLLERIDLKKDHIQ

ALVMVPTRELALQVSQISIQISKHLGGVKVMATTGGTNLRDDIMRLDETVHVVIATPGRILDLIKKGVAKVDRVQMMVMD

EADKLLSQDFVVLIEDIISFLAKNRQILLYSATFPISVQKFMAKHLQKPYEINLMEELTLKGITQYYAYVTERQKVHCLN

TLFSRLQINQSIIFCNSTQRVELLAKKITQLGYSCFYIHAKMMQEYRNRVFHDFRNGLCRNLVCTDLFTRGIDIQAVNVV

INFDFPKNAETYLHRIGRSGRFGHLGLAINLITSEDRFNLKAIEDQLVTDIKPIPGSIDKSLYVAEFHSSGADCDVEEVE

EKPGHQHDST

>XP_033949907.1 probable ATP-dependent RNA helicase ddx6 [Pseudochaenichthys georgianus]

MATARTENSGPVIMGLNKQNGQLRGQTKPAPVQPAPTTQGKAPAAPQTAGGAAQDGGGIKFGDDWKKSLKLPPRDDRVRT

SDVTSTKGNEFEDYCLKRELLMGIFEMGWEKPSPIQEESIPIALSGRDILARAKNGTGKSGAYLIPMLERIDLKKDYIQA

LVMVPTRELALQVSQISIQISKHLGGVKVMATTGGTNLRDDIMRLDETVHVVIATPGRILDLIKKGVAKVDRVQMMVMDE

ADKLLSQDFVVLIEDIISFLAKHRQILLYSATFPISVQKFMSKHLQKPYEINLMEELTLKGITQYYAYVTERQKVHCLNT

LFSRLQINQSIIFCNSTQRVELLAKKITQLGYSCFYIHAKMMQEYRNRVFHDFRNGLCRNLVCTDLFTRGIDIQAVNVVI

NFDFPKNAETYLHRIGRSGRFGHLGLAINLITSEDRFNLKSIEDQLVTDIKPIPSSIDKSLYVAEFHTSGGDCDVEEIEE

KPGRQQDGK

>XP_017581072.1 PREDICTED: probable ATP-dependent RNA helicase ddx6 [Pygocentrus nattereri]

MASARTENVGPVVMGINKQNGQFRGQPKPASQSGPIVGSGQSGKVPTGGQKSAGGSQEGPGIRFGDDWKKSLQLPPKDNR

VRTSDVTATKGNEFEDYCLKRELLMGIFEMGWEKPSPIQEESIPIALSGRDILARAKNGTGKSGAYLIPMLERIDLKKDH

IQALVLVPTRELALQVSQISIQLSKHLGGVKVMATTGGTNLRDDIMRLDETVHVVIATPGRILDLIKKGVAKVDKVQMMV

MDEADKLLSQDFVVLIEDIIGFLAKNRQILLYSATFPTSVQKFMAKHLQKPYEINLMEELTLKGITQYYAYVTERQKVHC

LNTLFSRLQINQSIIFCNSTQRVELLAKKITQLGYSCFYIHAKMMQEYRNRVFHDFRNGLCRNLVCTDLFTRGIDIQAVN

VVINFDFPKNAETYLHRIGRSGRFGHLGLAINLITAEDRFNLKAIEDQLVTDIKPIPGSIDKSLYVAEYHTMNSEVEAEE

REQLAGKLQTS

>KAE8282499.1 putative ATP-dependent RNA helicase ddx6 [Larimichthys crocea]

MGLNKQNGQLRGGPTKPASVQPAPTTQGKSLGAPQIAGGAAQDGGGIKFGDDWKKSLKLPPKDNRVKTSDVTSTKGNEFE

DYCLKRELLMGIFEMGWEKPSPIQEESIPIALSGRDILARAKNGTGKSGAYLIPLLERIDLKKDHIQALVMVPTRELALQ

VSQISIQISKHLGGVKVMATTGGTNLRDDIMRLDETVHVVIATPGRILDLIKKGVAKVDRVQMMVMDEADKLLSQDFVVL

IEDIISFLAKNRQILLYSATFPISVQKFMAKHLQKPYEINLMEELTLKGITQYYAYVTERQKVHCLNTLFSRLQINQSII

FCNSTQRVELLAKKITQLGYSCFYIHAKMMQEYRNRVFHDFRNGLCRNLVCTDLFTRGIDIQAVNVVINFDFPKNAETYL

HRIGRSGRFGHLGLAINLITSEDRFNLKAIEDQLVTDIKPIPGSIDKSLYVAEFHSSGADCDVEEVEEKPGHQHDST

>XP_013881103.1 PREDICTED: probable ATP-dependent RNA helicase ddx6 [Austrofundulus limnaeus]

MATTRTESVGPLVLGLNKQNGQLRGLNRPASVPAPPTAPGKSLGALQKPGGANQDGGGGGIKFGDDWKKSLQLPPKDNRV

KTSDVTSTKGNEFEDYCLKRELLMGIFEMGWEKPSPIQEESIPIALSGRDILARAKNGTGKSGAYLIPLLERIDLKKDHI

QAMVMVPTRELALQVSQICIQISKHLGGVKVMATTGGTNLRDDIMRLDETVHVVIATPGRILDLIKKGVAKVDRVQMMVM

DEADKLLSQDFVVLIEDIISFLAKNRQILLYSATFPISVQKFMVKHLQKPYEINLMEELTLKGITQFYAYVTERQKVHCL

NTLFSRLQINQSIIFCNSTQRVELLAKKITQLGYSCFYIHAKMMQEYRNRVFHDFRNGLCRNLVCTDLFTRGIDIQAVNV

VINFDFPKNAETYLHRIGRSGRFGHLGLAINLITSEDRFNLKAIEEQLVTDIKPIPGSIDKSLYVAEFHSAGDDCEVEEV

EEKPEPRQHST

>XP_034091900.1 probable ATP-dependent RNA helicase ddx6 [Gymnodraco acuticeps]

MATARTENSGPVIMGLNKQNGQLRGQTKPAPVQPAPTTQGKAPAAPQTVGGAAQDGGGIKFGDDWKKSLKLPPRDDRVRT

SDVTSTKGNEFEDYCLKRELLMGIFEMGWEKPSPIQEESIPIALSGRDILARAKNGTGKSGAYLIPMLERIDLKKDYIQA

LVMVPTRELALQVSQISIQISKHLGGVKVMATTGGTNLRDDIMRLDETVHVVIATPGRILDLIKKGVAKVDRVQMMVMDE

ADKLLSQDFVVLIEDIISFLAKHRQILLYSATFPISVQKFMSKHLQKPYEINLMEELTLKGITQYYAYVTERQKVHCLNT

LFSRLQINQSIIFCNSTQRVELLAKKITQLGYSCFYIHAKMMQEYRNRVFHDFRNGLCRNLVCTDLFTRGIDIQAVNVVI

NFDFPKNAETYLHRIGRSGRFGHLGLAINLITSEDRFNLKSIEDQLVTDIKPIPSSIDKSLYVAEFHTSGGDCDVEEIEE

KPGRQQDGK

>KAF7652552.1 hypothetical protein LDENG_00095220 [Lucifuga dentata]

MATARTANPASVIGLNKPANGQPKTALQQPVTQQSSIPQKRSSIPQNSWGIKFGDDWKNCLELPPKDNRVKTSDVTSTKG

NEFEDYCLKRELLMGIFEMGWEKPSPVQEESIPIALSGRDILARAKNGTGKSGAYLIPLLERIDLKKDHIQAIVMVPTRE

LALQMSQISIQLSKHLGGVKVMATTGGTNLRDDIMRLDETVHVVIATPGRILDLIKKGVAKVDKTQVMVMDEADKLLSQD

FVVLIEDIIGFLPKDRQILLYSATFPISVQKFMNKHLKKPYEINLMEELTLKGITQYYAYVTERQKVHCLNTLFSRLQIN

QSIIFCNSTQRVELLAKKITQLGYSCFYIHAKMMQEYRNRVFHDFRNGLCRNLVCTDLFTRGIDIQAVNVVINFDFPKSA

ETYLHRIGRSGRFGHLGLAINLITSEDRYNLKSTEEQLVTDIKPIPSSIDKSLYVAEFHSVDPHDDSEVGAKQKEVGAA

>XP_034553628.1 probable ATP-dependent RNA helicase ddx6 [Notolabrus celidotus]

MATTRTANPATMVGLNKPANGQLRGPTMPAEQSGVLESVQQPNPEKRMGIPLSSGGIKFGDDWKNCLELPPKDTRLKTSD

VTSTKGNEFEDYCLKRELLMGIFEMGWEKPSPIQEESIPIALSGRDILARAKNGTGKSGAYLIPLLERIDLKKDHIQAIV

MVPTRELALQVSQISIQLSKHLGGVKVMATTGGTNLRDDIMRLDETVHVVIATPGRILDLMRKGVAKMDRVSVMVMDEAD

KLLSQDFVVLIQDIISFLPKERQILLYSATFPISVQTFMNKNLKKPYEINLMEELTLKGITQYYAYVTERQKVHCLNTLF

SRLQINQSIIFCNSTQRVELLAKKISQLGYSCFYIHAKMMQEYRNRVFHDFRNGLCRNLVCTDLFTRGIDIQAVNVVINF

DFPKSAETYLHRIGRSGRFGHLGLAINLITSEDRYNLKNIEDQLVTEIKPIPGSIDKSLYVAEFHSVDPDDPAGDKCMDS

LAA

>AWP21470.1 putative ATP-dependent RNA helicase DDX6-like [Scophthalmus maximus]

MATARTENVGTVVMGLNKQNGQLRGQMKPASVQPGPTTQGKTMGAPQKAGCGPQDGGGIKFGDDWKKSLKLPPKDTRVKT

SDVTSTKGNEFEDYCLKRELLMGIFEMGWEKPSPIQEESIPIALSGRDILARAKNGTGKSGAYLIPMLERIDLKKDHIQA

MVVVPTRELALQVSQICIQISKHLGGVKVMATTGGTNLRDDILRLDETVHVVIATPGRILDLIKKGVAKVDRVQMMVMDE

ADKLLSQDFVVLIEDIISFLAKGRQILLYSATFPISVQKFMAKHLQKPYEINLMEELTLKGITQFYAYVTERQKVHCLNT

LFSRLQINQSIIFCNSTQRVELLAKKITQLGYSCFYIHAKMMQEYRNRVFHDFRNGLCRNLVCTDLFTRGIDIQAVNVVI

NFDFPKNAETYLHRIGRSGRFGHLGLAINLITSEDRFNLKSIEDQLVTDIKPIPGSIDKSLYVAEYHSGSGDCDVEEVEE

KPGHQQDEVL

>XP_020497901.1 probable ATP-dependent RNA helicase ddx6 [Labrus bergylta]

MATARTENVGPVVMGLNKQNGQLRGQTKPAPVQPASTTQGKALGSAQIAGAALQDGGGGGGGGGGIKFGDDWKKSLKLPP

KDCRVKTSDVTSTKGNEFEDYCLKRELLMGIFEMGWEKPSPIQEESIPIALSGRDILARAKNGTGKSGAYLIPLLERIDL

KKDHIQALVMVPTRELALQVSQISIQISKHLGGVKVMATTGGTNLRDDIMRLDEIVHVVIATPGRILDLIKKGVAKVDRV

QMMVMDEADKLLSQDFVVLIEDIISFLAKNRQILLYSATFPISVQKFMAKHLQKPYEINLMEELTLKGITQYYAYVTERQ

KVHCLNTLFSRLQINQSIIFCNSTQRVELLAKKITQLGYSCFYIHAKMMQEYRNRVFHDFRNGLCRNLVCTDLFTRGIDI

QAVNVVINFDFPKNAETYLHRIGRSGRFGHLGLAINLITSEDRFNLKSIEDQLVTDIKPIPSSIDKSLYVAEFHSSGADC

DVEEVEEKPGRQQDST

>XP_029919657.1 probable ATP-dependent RNA helicase ddx6 [Myripristis murdjan]

MATARTENVGPVVMGLNKQNGQLRGQTKPATIQSGPTTQGKAASTPQKAGSAPQEGGGIKFGDDWKKSLQLPPKDNRVKT

SDVTSTKGNEFEDYCLKRELLMGIFEMGWEKPSPIQEESIPIALSGRDILARAKNGTGKSGAYLIPLLERIDLKKDHIQA

IVMVPTRELALQVSQISIQISKHLGGVKVMATTGGTNLRDDIMRLDETVHVVIATPGRILDLIKKGVAKVDKVQMMVMDE

ADKLLSQDFVVLIEDIIGFLAKNRQILLYSATFPISVQKFMAKHLQKPYEINLMEELTLKGITQYYAYVTERQKVHCLNT

LFSRLQINQSIIFCNSTQRVELLAKKITQLGYSCFYIHAKMMQEYRNRVFHDFRNGLCRNLVCTDLFTRGIDIQAVNVVI

NFDFPKNAETYLHRIGRSGRFGHLGLAINLITSEDRFNLKAIEDQLVTDIKPIPSSIDKSLYVAEFHSTNPDCEVEEVEE

KSGRQQDGS

>RVE66677.1 hypothetical protein OJAV_G00109640 [Oryzias javanicus]

MATARTENVGPVVMGLNKQNGQLRGQNKPPAVQPAPPSQGKGLGALQKAGGAPQDGGGIKFGDDWKKSLLLPPKDNRVKT

SDVTATKGNEFEDYCLKRELLMGIFEMGWEKPSPIQEESIPIALSGRDILARAKNGTGKSGAYLIPLLERIDLKKDHIQA

MVMVPTRELALQVSQICIQLSKHLGGVKVMATTGGTNLRDDIMRLDETVHVVIATPGRILDLIKKGVAKVDRVQMIVMDE

ADKLLSQDFVVLIEDIISFLAKNRQILLYSATFPISVQKFMAKHLQKPYEINLMEELTLKGITQFYAYVTERQKVHCLNT

LFSRLQINQSIIFCNSTQRVELLAKKITQLGYSCFYIHAKMMQEYRNRVFHDFRNGLCRNLVCTDLFTRGIDIQAVNVVI

NFDFPKNAETYLHRIGRSGRFGHLGLAINLITSEDRFNLKAIEEQLVTDIKPIPSSIDKSLYVAEYHCSSGDGDAEDKPE

RQQDST

>XP_026783923.1 probable ATP-dependent RNA helicase ddx6 [Pangasianodon hypophthalmus]

MATAKMENVGAVVMGISKQNGQLRGLSSQSGSTIQSNPFARVSTGGQKPAADSQEGPGIRFGEDWKKSLQLPPKDKRVKT

SDVTATKGNEFEDYCLKRELLMGIFEMGWEKPSPVQEESIPIALSGRDILARAKNGTGKSGAYLIPMLERIDLKKDYIQA

LVMVPTRELALQVSQISIQLSKHLGGVKVMATTGGTNLRDDIMRLDEIVHVVIATPGRILDLIKKGVAKVDKVQIMVMDE

ADKLLSQDFVVLIEDIISFLDKNRQILLYSATFPITVQKFMAKHLRKPYEINLMEELTLKGITQYYAYVTERQKVHCLNT

LFSRLQINQSIIFCNSTQRVELLAKKITQLGYSCFYIHAKMMQEYRNRVFHDFRNGLCRNLVCTDLFTRGIDIQAVNVVI

NFDFPKNAETYLHRIGRSGRFGHLGLAINLITAEDRFNLKAIEDQLVTDIKPIPSNIDKSLYVAEFHSTNDEAEEQETQE

NP

>XP_035476750.1 probable ATP-dependent RNA helicase ddx6 [Scophthalmus maximus]

MATARTENVGTVVMGLNKQNGQLRGQMKPASVQPGPTTQGKTMGAPQKAGCGPQDGGGIKFGDDWKKSLKLPPKDTRVKT

SDVTSTKGNEFEDYCLKRELLMGIFEMGWEKPSPIQEESIPIALSGRDILARAKNGTGKSGAYLIPMLERIDLKKDHIQA

MVVVPTRELALQVSQICIQISKHLGGVKVMATTGGTNLRDDILRLDETVHVVIATPGRILDLIKKGVAKVDRVQMMVMDE

ADKLLSQDFVVLIEDIISFLAKGRQILLYSATFPISVQKFMAKHLQKPYEINLMEELTLKGITQFYAYVTERQKVHCLNT

LFSRLQINQSIIFCNSTQRVELLAKKITQLGYSCFYIHAKMMQEYRNRVFHDFRNGLCRNLVCTDLFTRGIDIQAVNVVI

NFDFPKNAETYLHRIGRSGRFGHLGLAINLITSEDRFNLKSIEDQLVTDIKPIPGSIDKSLYVAEYHSGSGDCDVEEVEE

KPGHQQDGT

>XP_034729134.1 probable ATP-dependent RNA helicase ddx6 [Etheostoma cragini]

MATARTANPAQMIGLNKPANGQLRGQASLLAAAQQPSVPQKRTSIPQSSGGIKFGDDWKKCLELPPKDTRMRTADVTSTK

GNEFEDYCLKRELLMGIFEMGWEKPSPVQEESIPIALSGRDILARAKNGTGKSGAYLIPLLERIDLKKDHIQAIVMVPTR

ELALQVSQISIQLSKHLGGVKVMATTGGTNLRDDIMRLDETVHVVIATPGRILDLIKKGVAKMDKAQLIVMDEADKLLSQ

DFVVLIEDIISFMPKDRQILLYSATFPISVQKFMNKHLKKPYEINLMEELTLKGITQYYAYVTERQKVHCLNTLFSRLQI

NQSIIFCNSTQRVELLAKKITQLGYSCFYIHAKMMQEYRNRVFHDFRNGLCRNLVCTDLFTRGIDIQAVNVVINFDFPKN

AETYLHRIGRSGRFGHLGLAINLITSEDRYNLKNIEDQLVTDIKPIPSSIDKSLYVAEFHSVDPDDDENDYDDHGRAKKK

ELGGI

>XP_003458835.1 probable ATP-dependent RNA helicase ddx6 [Oreochromis niloticus]

MATARTANPAPMAGLNKPANGQLRGQTKTGGQQSELLSNVQHPSAHKRTSIPQSSGGIKFGDDWKKCLELPPKDTRVKTS

DVTSTKGNEFEDYCLKRELLMGIFEMGWEKPSPVQEESIPIALSGRDILARAKNGTGKSGAYLIPLLERIDLKKDHIQAI

VMVPTRELALQMSQISIQLSKHLGGVKIMATTGGTNLRDDIMRLDETVHVVIATPGRILDLIKKGVAKVDKTQMLVMDEA

DKLLSQDFVVLIENIISFMPKDRQILLYSATFPISVQKFMSKHMQKPYEINLMEELTLKGITQYYAYVTERQKVHCLNTL

FSRLQINQSIIFCNSTQRVELLAKKITQLGYSCFYIHAKMMQEYRNRVFHDFRNGLCRNLVCTDLFTRGIDIQAVNVVIN

FDFPKNAETYLHRIGRSGRFGHLGLAINLITSDDRYNLKNIEDQLVTDIKPIPSSIDKSLYVAEFHSVDAEDGGEEKA

>XP_023265125.1 probable ATP-dependent RNA helicase ddx6 [Seriola lalandi dorsalis]

MATARTANPAPMIGLNKAANGQLRGQTKPAGQQSGLLATAQQSSVSQNRSSIPQNSGGIKFGDDWKKCLELPPKDTRLRT

SDVTSTKGNEFEDYCLKRELLMGIFEMGWEKPSPIQEESIPIALSGRDILARAKNGTGKSGAYLIPLLERIDLKKDHIQA

LVMVPTRELALQMSQISIQLSKHLGGVKVMATTGGTNLRDDIMRLDEIVHVVIATPGRILDLIKKGVAKVDKTQMMVMDE

ADKLLSQDFVVLIEDIISFLPKGRQILLYSATFPISVQKFMSKHLKKPYEINLMEELTLKGITQYYAYVTERQKVHCLNT

LFSRLQINQSIIFCNSTQRVELLAKKITQLGYSCFYIHAKMMQEYRNRVFHDFRNGLCRNLVCTDLFTRGIDIQAVNVVI

NFDFPKSAETYLHRIGRSGRFGHLGLAINLITSDDRYNLKTIEDQLITDIKPIPSSIDKSLYVAEFHSVDPDDDDDIGEA

KNKELGTA

>XP_031695498.1 probable ATP-dependent RNA helicase ddx6 [Anarrhichthys ocellatus]

MATARTENAGPVIMGLNKQNGQLRGQTKPASVQPAPTTQGKALGAPQIAGGAAQDGGGIKFGDDWKKSLKLPPRDNRVKT

SDVTSTKGNEFEDYCLKRELLMGIFEMGWEKPSPIQEESIPIALSGRDILARAKNGTGKSGAYLIPLLERIDLKKDYIQA

LVMVPTRELALQVSQISIQISKHLGGVKVMATTGGTNLRDDIMRLDETVHVVIATPGRILDLIKKGVAKVDRVQIMVMDE

ADKLLSQDFVVLIEDIISFLAKGRQILLYSATFPISVQKFMAKHLQKPYEINLMEELTLKGITQYYAYVTERQKVHCLNT

LFSRLQINQSIIFCNSTQRVELLAKKITQLGYSCFYIHAKMMQEYRNRVFHDFRNGLCRNLVCTDLFTRGIDIQAVNVVI

NFDFPKNAETYLHRIGRSGRFGHLGLAINLITSEDRFNLKAIEDQLVTDIKPIPSSIDKSLYVAEFHTSGADCDVEEIEE

KPGRQQDGT

>XP_034409072.1 probable ATP-dependent RNA helicase ddx6 isoform X2 [Cyclopterus lumpus]

MATARTANSAPMIGLNKPANGQLRGQAGLLASAQQPNALQKRTGIPQSSGGIKFGDDWKKCLELPPRDTRMRTSDVTSTK

GNEFEDYCLKRELLMGIFEMGWEKPSPVQEESIPIALSGRDILARAKNGTGKSGAYLIPLLERIDLKKDHIQAIVMVPTR

ELALQMSQISIQLSKHLGGVKVMATTGGTNLRDDIMRLDETVHVVIATPGRILDLIKKGVAKMDKAQLIVMDEADKLLSQ

DFVVLIEDIISFMPRDRQILLYSATFPISVQKFMSKHLKKPYEINLMEELTLKGITQYYAYVTERQKVHCLNTLFSRLQI

NQSIIFCNSTQRVELLAKKITQLGYSCFYIHAKMMQEYRNRVFHDFRNGLCRNLVCTDLFTRGIDIQAVNVVINFDFPKN

AETYLHRIGRSGRFGHLGLAINLITSEDRYNLKNIEDQLVTDIKPIPSCIDKSLYVAEFHSVDPDADDDIGGGDNGGAKS

KELGAI

>XP_006808249.1 probable ATP-dependent RNA helicase ddx6 [Neolamprologus brichardi]

MATARTANPAPMAGLNKPANGQLRGQTKTGGQQSELLSNVQHPSAHKRTSIPQSSGGIKFGDDWKKCLELPPKDTRVKTS

DVTSTKGNEFEDYCLKRELLMGIFEMGWEKPSPVQEESIPIALSGRDILARAKNGTGKSGAYLIPLLERIDLKKDHIQAI

VMVPTRELALQMSQISIQLSKHLGGVKVMATTGGTNLRDDIMRLDETVHVVIATPGRILDLIKKGVAKVDKTQMMVMDEA

DKLLSQDFVVLIENIISFMPKDRQILLYSATFPISVQKFMSKHMKKPYEINLMEELTLKGITQYYAYVTERQKVHCLNTL

FSRLQINQSIIFCNSTQRVELLAKKITQLGYSCFYIHAKMMQEYRNRVFHDFRNGLCRNLVCTDLFTRGIDIQAVNVVIN

FDFPKNAETYLHRIGRSGRFGHLGLAINLITSDDRYNLKNIEDQLVTDIKPIPSSIDKSLYVAEFHSVDAEDGGEETA

>XP_022620374.1 probable ATP-dependent RNA helicase ddx6 [Seriola dumerili]

MATARTANPAPMIGLNKAANGQLRGQTKPAGQSGLLATAQLSSVSQNRSSIPQNSGGIKFGDDWKKCLELPPKDTRLRTS

DVTSTKGNEFEDYCLKRELLMGIFEMGWEKPSPIQEESIPIALSGRDILARAKNGTGKSGAYLIPLLERIDLKKDHIQAL

VMVPTRELALQMSQISIQLSKHLGGVKVMATTGGTNLRDDIMRLDEIVHVVIATPGRILDLIKKGVAKVDKTQMMVMDEA

DKLLSQDFVVLIEDIISFLPKGRQILLYSATFPISVQKFMSKHLKKPYEINLMEELTLKGITQYYAYVTERQKVHCLNTL

FSRLQINQSIIFCNSTQRVELLAKKITQLGYSCFYIHAKMMQEYRNRVFHDFRNGLCRNLVCTDLFTRGIDIQAVNVVIN

FDFPKSAETYLHRIGRSGRFGHLGLAINLITSDDRYNLKTIEDQLITDIKPIPSSIDKSLYVAEFHSVDPDDDDDIGEAK

NKELGTA

>KAF0024456.1 hypothetical protein F2P81_023258 [Scophthalmus maximus]

MATARTENVGTVVMGLNKQNGQLRGQMKPASVQPGPTTQGKTMGAPQKAGCGPQDGGGIKFGDDWKKSLKLPPKDTRVKT

SDVTSTKGNEFEDYCLKRELLMGIFEMGWEKPSPIQEESIPIALSGRDILARAKNGTGKSGAYLIPMLERIDLKKDHIQA

MVVVPTRELALQVSQICIQISKHLGGVKVMATTGGTNLRDDILRLDETVHVVIATPGRILDLIKKGVAKVDRVQMMVMDE

ADKLLSQDFVVLIEDIISFLAKGRQILLYSATFPISVQKFMAKHLQKPYEINLMEELTLKGITQFYAYVTERQKVHCLNT

LFSRLQINQSIIFCNSTQRVELLAKKITQLGYSCFYIHAKMMQEYRNRVFHDFRNGLCRNLVCTDLFTRGIDIQAVNVVI

NFDFPKNAETYLHRIGRSGRFGHLGLAINLITSEDRFNLKSIEDQLVTDIKPIPGSIDKSLYVAEYHSGSGDCDVEEVEE

KPGHQQDELLRSHAASVEPMSGLTARLWENQINSQSMRSQHGYSRCRPHFTQPIAFEQKRYIMNETHRCVSRFFREWSSD

TRVFVIRQRRQRMGSVLAASSPNPPPPAAGGAAAPGLTVPPGFGMPQVSPVIPPTGAASGQEAETPLPNPGAFDECHRKC

KEVFPMQMEGVRVVVNKGLSNHFQVNHTVLLSTTGDSTYRFGATYVGSKQTGPAELFPVIVGDMDNSGSLNAQIIHQITN

RIRSKVAFQTQQNKFVNWQGDAEIRGEDYTATVTLGNPDVLVGSGIVVTHYLQSITPALALGGELVYHRRPGEEGAVMSL

VGRYTGNNYIATLTLGSAGAHASYYHKANDQLQVGVEFEASTRMQDTSVSFGYQLDVPKANLLFKGSVDSNWIVGATLEK

KLLPLPLSLVLCSFLNHRKNKFQCGFGVTIG

>XP_031144776.1 probable ATP-dependent RNA helicase ddx6 [Sander lucioperca]

MATARTANPAQMIGLNKPANGQLRGQAALLAAAQQPSALQKRTSIPQSSGGIKFGDDWKKCLELPPKDTRMRTADVTSTK

GNEFEDYCLKRELLMGIFEMGWEKPSPVQEESIPIALSGRDILARAKNGTGKSGAYLIPLLERIDLKKDHIQAIVMVPTR

ELALQVSQISIQLSKHLGGVKVMATTGGTNLRDDIMRLDETVHVVIATPGRILDLIKKGVAKMDKAQLIVMDEADKLLSQ

DFVVLIEDIISFMPKDRQILLYSATFPISVQKFMNKHLKKPYEINLMEELTLKGITQYYAYVTERQKVHCLNTLFSRLQI

NQSIIFCNSTQRVELLAKKITQLGYSCFYIHAKMMQEYRNRVFHDFRNGLCRNLVCTDLFTRGIDIQAVNVVINFDFPKN

AETYLHRIGRSGRFGHLGLAINLITSEDRYNLKNIEDQLVTDIKPIPSSIDKSLYVAEFHSVDPDDDENDYDDHGGAKNK

ELGGI

>KAF3707203.1 putative ATP-dependent RNA helicase ddx6 [Channa argus]

MATARTENVGPVVMGLNKQNGQLRGQTKPASVQPVPTVQGKASGLPQKAGTGPQDGGAIKFGDDWKKSLQLPPKDNRVKT

SDVTATKGNEFEDYCLKRELLMGIFEMGWEKPSPIQEESIPIALSGRDILARAKNGTGKSGAYLIPLLERIDLKKDHIQA

IVMVPTRELALQVSQISIQISKHLGGVKVMATTGGTNLRDDIMRLDETVHVVIATPGRILDLIKKGVAKVDKVQMMVMDE

ADKLLSQDFLVLIEDIIGFLPRNRQILLYSATFPISVQKFMAKHLQKPYEINLMEELTLKGITQFYAYVTERQKVHCLNT

LFSRLQINQSIIFCNSTQRVELLAKKITQLGYSCFYIHAKMMQEYRNRVFHDFRNGLCRNLVCTDLFTRGIDIQAVNVVI

NFDFPKNAETYLHRIGRSGRFGHLGLAINLITSEDRFNLKAIEDQLVTDIKPIPGSIDKSLYVAEYHSNSGDCGVEEVED

KVGRQHDST

>NXJ10913.1 DDX6 helicase [Odontophorus gujanensis]

MSTARTENPVIMGLSSQNGQLRGPVKPSGGPGGGGTQTQQQMNQLKNANTINNGTQQQAQSMTTTIKPGDDWKKTLKLPP

KDLRIKTSDVTSTKGNEFEDYCLKRELLMGIFEMGWEKPSPIQEESIPIALSGRDILARAKNGTGKSGAYLIPLLERLDL

KKDNIQAMVIVPTRELALQVSQICIQVSKHMGGAKVMATTGGTNLRDDIMRLDDTVHVVIATPGRILDLIKKGVAKVEHV

QMILSLISYFVLLLLQADKLLSQDFVQIMEDIILTLPKNRQILLYSATFPLSVQKFMNSHLQKPYEINLMEELTLKGVTQ

YYAYVTERQKVHCLNTLFSRLQINQSIIFCNSSQRVELLAKKISQLGYSCFYIHAKMRQEHRNRVFHDFRNGLCRNLVCT

DLFTRGIDIQAVNVVINFDFPKLAETYLHRIGRSGRFGHLGLAINLITYDDRFNLKSIEEQLGTEIKPIPSNIDKSLYVA

EYHSEPVEDEKQ

>XP_026040128.1 probable ATP-dependent RNA helicase ddx6 [Astatotilapia calliptera]

MATARTANPAPMAGLNKPANGQLRGQTKTGGQQSELLSNVQHPSAHKRTSIPQSSGGIKFGDDWKKCLELPPKDTRVKTS

DVTSTKGNEFEDYCLKRELLMGIFEMGWEKPSPVQEESIPIALSGRDILARAKNGTGKSGAYLIPLLERIDLKKDHIQAM

VMVPTRELALQMSQISIQLSKHLGGVKIMATTGGTNLRDDIMRLDETVHVVIATPGRILDLIKKGVAKVDKTQMMVMDEA

DKLLSQDFVVLMENIISFMPKDRQILLYSATFPISVQKFMSKHMQKPYEINLMEELTLKGITQYYAYVTERQKVHCLNTL

FSRLQINQSIIFCNSTQRVELLAKKITQLGYSCFYIHAKMMQEYRNRVFHDFRNGLCRNLVCTDLFTRGIDIQAVNVVIN

FDFPKNAETYLHRIGRSGRFGHLGLAINLITSDDRYNLKNIEDQLVTDIKPIPSSIDKSLYVAEFHSVDAEDGEEKA

>XP_033496644.1 probable ATP-dependent RNA helicase DDX6 [Epinephelus lanceolatus]

MATARTANPAAMIGLNKQANGQLRGQPGLLATAQQPSALQKKTSIPQSSGGIKFGDDWKKCLELPPKDTRMKTSDVTSTK

GNEFEDYCLKRELLMGIFEMGWEKPSPVQEESIPIALSGRDILARAKNGTGKSGAYLIPLLERIDLKKDHIQAIVMVPTR

ELALQVSQISIQLSKHLGGVKIMATTGGTNLRDDIMRLDEIVHVVIATPGRILDLIKKGVAKMDRAQMIVMDEADKLLSQ

DFVVLIEDIISFMSKDRQILLYSATFPISVQKFMSKHLRKPYEINLMEELTLKGITQYYAYVTERQKVHCLNTLFSRLQI

NQSIIFCNSTQRVELLAKKITQLGYSCFYIHAKMMQEYRNRVFHDFRNGLCRNLVCTDLFTRGIDIQAVNVVINFDFPKN

AETYLHRIGRSGRFGHLGLAINLITSDDRYNLKNIEDQLVTDIKPIPSSIDKSLYVAEFHSVDPDADGDDEGGALNNELG

AI

>XP_003969279.1 probable ATP-dependent RNA helicase ddx6 [Takifugu rubripes]

MATTRTETVGPVILGLNKQNGQLRGQTKPVSVQSASAAPGKALAPSQTAGGGPQDGGGIKFGDDWKRNLKLPPKDHRVRT

SDVTSTKGNEFEDYCLKRELLMGIFEMGWEKPSPIQEESIPIALSGRDILARAKNGTGKSGAYLIPMLERIDLKKDHIQA

MVLVPTRELALQVSQISIQIAKHLGGVKVMATTGGTNLRDDIMRLDETVHVVIATPGRILDLIKKGVAKVDRVHIMVMDE

ADKLLSQDFVVLVEDIISFLAKNRQILLYSATFPISVQKFMAKHLQKPYEINLMEELTLKGITQYYAYVTERQKVHCLNT

LFSRLQINQSIIFCNSTQRVELLAKKITQLGYSCFYIHAKMMQEYRNRVFHDFRNGLCRNLVCTDLFTRGIDIQAVNVVI

NFDFPKNAETYLHRIGRSGRFGHLGLAINLITSEDRFNLKTIEEQLITDIKPIPGSIDKSLYVAEFHCSSADCEVEEVEE

KPGHQKDSP

>XP_014325647.1 probable ATP-dependent RNA helicase ddx6 [Xiphophorus maculatus]

MATARTENLGPVVMGLNKQNGQLKGQTKPAAGPPASTQGKNPAGSNQDGGGIKFGDDWKKCLQLPPKDNRVKTSDVTATK

GNEFEDYCLKRELLMGIFEMGWEKPSPIQEESIPIALSGRDILARAKNGTGKSGAYLIPLLERIDLKKDHIQAIVMVPTR

ELALQMSQICIQLSKHLGGVKVMATTGGTNLRDDIMRLDETVHVVIATPGRILDLIKKGVAKVDRVQMMVMDEADKLLSQ

DFVVLIEDIISFLAKNRQILLYSATFPISVQKFMVKHLQKPYEINLMEELTLKGITQFYAYVTERQKVHCLNTLFSRLQI

NQSIIFCNSTQRVELLAKKITQLGYSCFYIHAKMMQEYRNRVFHDFRNGLCRNLVCTDLFTRGIDIQAVNVVINFDFPKN

AETYLHRIGRSGRFGHLGLAINLITSEDRFNLKAIEEQLVTDIKPIPSSIDKSLYVAEYHSGPVTDGDEDDAEEKPPRQQ

DST

>XP_004550985.1 probable ATP-dependent RNA helicase ddx6 [Maylandia zebra]

MATARTANPAPMAGLNKPANGQLRGQTKTGGQQSELLSNVQHPSAHKRTSIPQSSGGIKFGDDWKKCLELPPKDTRVKTS

DVTSTKGNEFEDYCLKRELLMGIFEMGWEKPSPVQEESIPIALSGRDILARAKNGTGKSGAYLIPLLERIDLKKDHIQAM

VMVPTRELALQMSQISIQLSKHLGGVKIMATTGGTNLRDDIMRLDETVHVVIATPGRILDLIKKGVAKVDKTQMMVMDEA

DKLLSQDFVVLIENIISFMPKDRQILLYSATFPISVQKFMSKHMQKPYEINLMEELTLKGITQYYAYVTERQKVHCLNTL

FSRLQINQSIIFCNSTQRVELLAKKITQLGYSCFYIHAKMMQEYRNRVFHDFRNGLCRNLVCTDLFTRGIDIQAVNVVIN

FDFPKNAETYLHRIGRSGRFGHLGLAINLITSDDRYNLKNIEDQLVTDIKPIPSSIDKSLYVAEFHSVDAEDGEEKA

>XP_029591774.1 probable ATP-dependent RNA helicase ddx6 isoform X1 [Salmo trutta]

MASARTENLGPIGMGLNKQLRGVPKPASPQSGPLVLGSLLSKAVGAPQKAGAALEGTGIRFGDDWKKSLQLPPKDTRVRT

SDVTSTKGNEFEDYCLKRELLMGIFEMGWEKPSPIQEESIPIALSGRDILARAKNGTGKSGAYLIPMLERIDLKKDYIQA

IVMVPTRELALQVSQISIQISKHLGGVKVMATTGGTNLRDDIMRLDETVHVVIATPGRILDLIKKGVAKVDRVQMMVMDE

ADKLLSQDFVVLIEDIISFLGKGRQILLYSATFPISVQKFMAKHLQKPYEINLMEELTLKGITQYYAYVTERQKVHCLNT

LFSRLQINQSIIFCNSTQRVELLAKKITQLGYSCFYIHAKMMQEYRNRVFHDFRNGLCRNLVCTDLFTRGIDIQAVNVVI

NFDFPKNAETYLHRIGRSGRFGHLGLAINLITSEDRFNLKSIEDQLVTDIKPIPGSIDKSLYVAEFHATNPNCEEELKET

GRQQVEP

>OXB75928.1 hypothetical protein H355_012896 [Colinus virginianus]

MSTARTENPVIMGLSSQNGQLRGPVKPSGGPGGGGTQTQQQMNQLKNANTINNGTQQQAQSMTTTIKPGDDWKKTLKLPP

KDLRIKTSDVTSTKGNEFEDYCLKRELLMGIFEMGWEKPSPIQEESIPIALSGRDILARAKNGTGKSGAYLIPLLERLDL

KKDNIQAMVIVPTRELALQVSQICIQVSKHMGGAKVMATTGGTNLRDDIMRLDDTVHVVIATPGRILDLIKKGVAKVEHA

DKLLSQDFVQIMEDIILTLPKNRQILLYSATFPLSVQKFMNSHLQKPYEINLMEELTLKGVTQYYAYVTERQKVHCLNTL

FSRLQINQSIIFCNSSQRVELLAKKISQLGYSCFYIHAKMRQEHRNRVFHDFRNGLCRNLVCTGLDFMEINILHGLACLS

DLFTRGIDIQAVNVVINFDFPKLAETYLHRIGRSGRFGHLGLAINLITYDDRFNLKSIEEQLGTEIKPIPSNIDKSLYVA

EYHSEPVEDEKQ

>XP_015829536.1 PREDICTED: probable ATP-dependent RNA helicase ddx6 [Nothobranchius furzeri]

MATARTENAGPVVMGLNKQNGQLRGQTKPASVQPASAAQEKSLGAPQKPEWGGIKFGDDWKKSLQLPPKDNRVRTSDVTA

TKGNEFEDYCLKRELLMGIFEMGWEKPSPIQEESIPIALSGRDILARAKNGTGKSGAYLIPLLERIDLKKDHIQAIVMVP

TRELALQMSQISIQLSKHLGGVKVMATTGGTNLRDDIMRLDETVHVVIATPGRILDLIKKGVAKVDRVQMMVMDEADKLL

SQDFVVLIEDIISFLAKNRQILLYSATFPISVQKFMVKHLQKPYEINLMEELTLKGITQFYAYVTERQKVHCLNTLFSRL

QINQSIIFCNSTQRVELLAKKITQLGYSCFYIHAKMMQEYRNRVFHDFRNGLCRNLVCTDLFTRGIDIQAVNVVINFDFP

KNAETYLHRIGRSGRFGHLGLAINLITSEDRFNLKAIEEQLVTDIKPIPSSIDKSLYVAEYHSASGDCEVEEVEEKPERQ

QDST

>XP_007560107.1 PREDICTED: probable ATP-dependent RNA helicase ddx6 [Poecilia formosa]

MATARTENLGPVVMGLNKQNGQLKGQTKPAAGPPASTQGKNPAGSNQDGGGIKFGDDWKKCLQLPPKDNRVKTSDVTATK

GNEFEDYCLKRELLMGIFEMGWEKPSPIQEESIPIALSGRDILARAKNGTGKSGAYLIPLLERIDLKKDHIQAIVMVPTR

ELALQMSQICIQLSKHLGGVKVMATTGGTNLRDDIMRLDETVHVVIATPGRILDLIKKGVAKVDRVQMMVMDEADKLLSQ

DFVVLIEDIISFLAKNRQILLYSATFPISVQKFMVKHLQKPYEINLMEELTLKGITQFYAYVTERQKVHCLNTLFSRLQI

NQSIIFCNSTQRVELLAKKITQLGYSCFYIHAKMMQEYRNRVFHDFRNGLCRNLVCTDLFTRGIDIQAVNVVINFDFPKN

AETYLHRIGRSGRFGHLGLAINLITSEDRFNLKAIEEQLVTDIKPIPSSIDKSLYVAEYHSGPVTDGDEAEEKPPRQQDS

T

>XP_034409071.1 probable ATP-dependent RNA helicase ddx6 isoform X1 [Cyclopterus lumpus]

MLQIEARTANSAPMIGLNKPANGQLRGQAGLLASAQQPNALQKRTGIPQSSGGIKFGDDWKKCLELPPRDTRMRTSDVTS

TKGNEFEDYCLKRELLMGIFEMGWEKPSPVQEESIPIALSGRDILARAKNGTGKSGAYLIPLLERIDLKKDHIQAIVMVP

TRELALQMSQISIQLSKHLGGVKVMATTGGTNLRDDIMRLDETVHVVIATPGRILDLIKKGVAKMDKAQLIVMDEADKLL

SQDFVVLIEDIISFMPRDRQILLYSATFPISVQKFMSKHLKKPYEINLMEELTLKGITQYYAYVTERQKVHCLNTLFSRL

QINQSIIFCNSTQRVELLAKKITQLGYSCFYIHAKMMQEYRNRVFHDFRNGLCRNLVCTDLFTRGIDIQAVNVVINFDFP

KNAETYLHRIGRSGRFGHLGLAINLITSEDRYNLKNIEDQLVTDIKPIPSCIDKSLYVAEFHSVDPDADDDIGGGDNGGA

KSKELGAI

>XP_013997638.1 PREDICTED: probable ATP-dependent RNA helicase ddx6 isoform X1 [Salmo salar]

MASATTENLGPIGMGLNKQNGQLRGVPKPASPQSGPLVLGSLLSKAVGAPQKAGAALEGTGIRFGDDWKKSLQLPPKDTR

VRTSDVTSTKGNEFEDYCLKRELLMGIFEMGWEKPSPIQEESIPIALSGRDILARAKNGTGKSGAYLIPMLERIDLKKDY

IQAIVMVPTRELALQVSQISIQISKHLGGVKVMATTGGTNLRDDIMRLDETVHVVIATPGRILDLIKKGVAKVDRVQMMV

MDEADKLLSQDFVVLIEDIISFLGKGRQILLYSATFPISVQKFMAKHLQKPYEINLMEELTLKGITQYYAYVTERQKVHC

LNTLFSRLQINQSIIFCNSTQRVELLAKKITQLGYSCFYIHAKMMQEYRNRVFHDFRNGLCRNLVCTDLFTRGIDIQAVN

VVINFDFPKNAETYLHRIGRSGRFGHLGLAINLITSEDRFNLKSIEDQLVTDIKPIPGSIDKSLYVAEFHATNPNCEEEL

KETGRQQVEP

>XP_031709306.1 probable ATP-dependent RNA helicase ddx6 [Anarrhichthys ocellatus]

MATARTANPAPMLGLNKPANGQLRGQTGLLPTAQQPNALQKRTSIPQSSGGIKFGDDWKKCLELPPRDTRMRTSDVTSTK

GNEFEDYCLKRELLMGIFEMGWEKPSPVQEESIPIALSGRDILARAKNGTGKSGAYLIPLLERIDLKKDHIQAIVMVPTR

ELALQMSQISIQLSKHLGGVKVMATTGGTNLRDDIMRLDETVHVVIATPGRILDLIKKGVAKMDKAQLIVMDEADKLLSQ

DFVVLIEDIISFMPKDRQILLYSATFPISVQKFMSKHLKKPYEINLMEELTLKGITQYYAYVTERQKVHCLNTLFSRLQI

NQSIIFCNSTQRVELLAKKITQLGYSCFYIHAKMMQEYRNRVFHDFRNGLCRNLVCTDLFTRGIDIQAVNVVINFDFPKN

AETYLHRIGRSGRFGHLGLAINLITSEDRYNLKNIEDQLVTDIKPIPSCIDKSLYVAEFHSVDPDAEDDEDGGAKSKELG

AI

>XP_026867354.1 probable ATP-dependent RNA helicase ddx6 isoform X1 [Electrophorus electricus]

MATARMESVGPVIMGISKQNGQFRGHTKPSSQTGPTGESSQPGKIPAVAQNPAGGSQEGPGIRFGDDWKKRLQLPPKDNR

IKTSDVTATKGNEFEDYCLKRELLMGIFEMGWEKPSPIQEESIPIALSGRDILARAKNGTGKSGAYLIPMLERIDLKKDH

IQAIVMVPTRELALQVSQITIQLSKHLGGVKVMATTGGTNLRDDIMRLDETVHVVIATPGRILDLIKKGVAKVDKVQMMI

MDEADKLLSQDFVVLIEDIISFLAKNRQILLYSATFPISVQKFMVKHLQKPYEINLMEELTLKGITQFYAYVTERQKVHC

LNTLFSRLQINQSIIFCNSTQRVELLAKKITQLGYSCFYIHAKMMQEYRNRVFHDFRNGLCRNLVCTDLFTRGIDIQAVN

VVINFDFPKNAETYLHRIGRSGRFGHLGLAINLITAEDRFNLKVVEDQLVTEIKPIPGNIDKSLYVAEFHSASLNSEAET

QEQETLPLAVKPQAL

>XP_028436311.1 probable ATP-dependent RNA helicase ddx6 [Perca flavescens]

MATARTANPAQMIGLNKPANGQLRGQAALLAAAQQSSALQKRTSIPQSSGGIKFGDDWKKCLELPPKDTRMRTADVTSTK

GNEFEDYCLKRELLMGIFEMGWEKPSPVQEESIPIALSGRDILARAKNGTGKSGAYLIPLLERIDLKKDHIQAIVMVPTR

ELALQVSQISIQLSKHLGGVKVMATTGGTNLRDDIMRLDETVHVVIATPGRILDLIKKGVAKMDKAQLIVMDEADKLLSQ

DFVVLIEDIISFMPKDRQILLYSATFPISVQKFMNKHLKKPYEINLMEELTLKGITQYYAYVTERQKVHCLNTLFSRLQI

NQSIIFCNSTQRVELLAKKITQLGYSCFYIHAKMMQEYRNRVFHDFRNGLCRNLVCTDLFTRGIDIQAVNVVINFDFPKN

AETYLHRIGRSGRFGHLGLAINLITSEDRYNLKNIEDQLVTDIKPIPSSIDKSLYVAEFHSVDPDDDDDENDYDDHGGAK

NKELGGI

>XP_032374621.1 probable ATP-dependent RNA helicase DDX6 [Etheostoma spectabile]

MATARTANPAQMIGLNKPANGQLRGQAALLAAAQQPSAPQKRTSIPQSSGGIKFGDDWKKCLELPPKDTRMRTADVTSTK

GNEFEDYCLKRELLMGIFEMGWEKPSPVQEESIPIALSGRDILARAKNGTGKSGAYLIPLLERIDLKKDHIQAIVMVPTR

ELALQVSQISIQLSKHLGGVKVMATTGGTNLRDDIMRLDETVHVVIATPGRILDLIKKGVAKMDKAQLIVMDEADKLLSQ

DFVVLIEDIISFMPKDRQILLYSATFPISVQKFMNKHLKKPYEINLMEELTLKGITQYYAYVTERQKVHCLNTLFSRLQI

NQSIIFCNSTQRVELLAKKITQLGYSCFYIHAKMMQEYRNRVFHDFRNGLCRNLVCTDLFTRGIDIQAVNVVINFDFPKN

AETYLHRIGRSGRFGHLGLAINLITSEDRYNLKNIEDQLVTDIKPIPSSIDKSLYVAEFHSVDPDDDENDYDDHGRAKKK

ELGGI

>XP_028316375.1 probable ATP-dependent RNA helicase ddx6 [Gouania willdenowi]

MATARTESVSPALMGLNKQNGQLKGLTKAASAQPGATTQGKTSGALPLQKAGSAQEGGGGGIKFGDDWKKSLKLPPKDHR

VRTSDVTATKGNEFEDYCLKRELLMGIFEMGWEKPSPIQEESIPIALSGRDILARAKNGTGKSGAYLIPMLERIDLKKDY

IQAVVMVPTRELALQVSQIAIQISKHLGGVKVMATTGGTNLRDDIMRLDETVHVVIATPGRILDLIKKGVAKVDRVQIMV

MDEADKLLSQDFVVLIEDIISFLAKNRQILLYSATFPISVQKFMAKHLQKPYEINLMEELTLKGISQFYAYVTERQKVHC

LNTLFSRLQINQSIIFCNSTQRVELLAKKITQLGYSCFYIHAKMMQEYRNRVFHDFRNGLCRNLVCTDLFTRGIDIQAVN

VVINFDFPKNAETYLHRIGRSGRFGHLGLAINLITSEDRFNLKAIEDQLVTDIKPIPGSIDKSLYVAEFHTADSEVDEKP

GQLLDST

>XP_020453091.1 probable ATP-dependent RNA helicase ddx6 [Monopterus albus]

MATARTENVGPVVMGLSKQNGQLRGQTKPTSVQPASTTQGKALGAPQKASSAPQDAGGIRFGDDWKKSLKLPPKDNRVKT

SDVTATKGNEFEDYCLKRELLMGIFEMGWEKPSPIQEESIPIALSGRDILARAKNGTGKSGAYLIPLVERIDLKKDHIQA

IVMVPTRELALQVSQICIQISKHLGGVKVMATTGGTILRDDIMRLDETVHVVIATPGRILDLIKKGVAKVDKVQMMVMDE

ADKLLSQDFVVLIEDIIGFLAKNRQILLYSATFPISVQKFMAKHLQKPYEINLMEELTLKGITQFYAYVTERQKVHCLNT

LFSRLQINQSIIFCNSTQRVELLAKKITQLGYSCFYIHAKMMQEYRNRVFHDFRNGLCRNLVCTDLFTRGIDIQAVNVVI

NFDFPKNAETYLHRIGRSGRFGHLGLAINLITSEDRFNLKAIEDQLVTDIKPIPGSIDKSLYVAEYHSVGADSGMEEAEE

KPGRQQDST

>XP_007258341.1 probable ATP-dependent RNA helicase ddx6 [Astyanax mexicanus]

MASARMENAGPVVMGINKQNGQLRGQPKPISQSGPMAVSGQPGKVPVGALKSAASLQEGPGIRFGDDWKKSLQLPPKDNR

VKTSDVTATKGNEFEDYCLKRELLMGIFEMGWEKPSPIQEESIPIALSGRDILARAKNGTGKSGAYLIPMLERIDLKKDH

VQAMVMVPTRELALQVSQISIQLSKHLGGVKVMATTGGTNLRDDIMRLDETVHVIIATPGRILDLIKKGVAKVDKVQMIV

MDEADKLLSQDFVVLIEDIISFLAKNRQILLYSATFPTSVQKFMSKHLQKPYEINLMEELTLKGITQYYAYVTERQKVHC

LNTLFSRLQINQSIIFCNSTQRVELLAKKITQLGYSCFYIHAKMMQEYRNRVFHDFRNGLCRNLVCTDLFTRGIDIQAVN

VVINFDFPKNAETYLHRIGRSGRFGHLGLAINLITAEDRFNLKAIEDQLVTDIKPIPGSIDKSLYVAEYHLGNPNSEAEA

EEREHLAGKPQTS

>XP_029307384.1 probable ATP-dependent RNA helicase ddx6 [Cottoperca gobio]

MATARTANPSPMIGLNKPANGQLRGPTGLLASAQQPSALQKRTGIPQSSGGIKFGDDWKKCLELPPRDTRMRTSDVTSTK

GNEFEDYCLKRELLMGIFEMGWEKPSPVQEESIPIALSGRDILARAKNGTGKSGAYLIPLLERIDLKKDHIQAIVMVPTR

ELALQMSQISIQLSKHLGGVKVMATTGGTNLRDDIMRLDETVHVVIATPGRILDLIKKGVAKMDRAQLIVMDEADKLLSQ

DFVVLIEDIISFMPKDRQILLYSATFPISVQKFMSKHLKKPYEINLMEELTLKGITQYYAYVTERQKVHCLNTLFSRLQI

NQSIIFCNSTQRVELLAKKITQLGYSCFYIHAKMMQEYRNRVFHDFRNGLCRNLVCTDLFTRGIDIQAVNVVINFDFPKN

AETYLHRIGRSGRFGHLGLAINLITSEDRYNLKNIEDQLVTDIKPIPSCIDKSLYVAEFHSVDPDAEDDDIEAGARNKEL

GGI

>XP_014852347.1 PREDICTED: probable ATP-dependent RNA helicase DDX6 [Poecilia mexicana]

MATARTENLGPVVMGLNKQNGQLKGQTKPAAGPPASTQGKNPAGSNQDGGGIKFGDDWKKCLQLPPKDNRVKTSDVTATK

GNEFEDYCLKRELLMGIFEMGWEKPSPIQEESIPIALSGRDILARAKNGTGKSGAYLIPLLERIDLKKDHIQAIVMVPTR

ELALQMSQICIQLSKHLGGVKVMATTGGTNLRDDIMRLDETVHVVIATPGRILDLIKKGVAKVDRVQMMVMDEADKLLSQ

DFVVLIEDIISFLAKNRQILLYSATFPISVQKFMVKHLQKPYEINLMEELTLKGITQFYAYVTERQKVHCLNTLFSRLQI

NQSIIFCNSTQRVELLAKKITQLGYSCFYIHAKMMQEYRNRVFHDFRNGLCRNLVCTDLFTRGIDIQAVNVVINFDFPKN

AETYLHRIGRSGRFGHLGLAINLITSEDRFNLKAIEEQLVTDIKPIPSSIDKSLYVAEYHSGPVGDEDEEKPPRQQDST

>KAF3692143.1 putative ATP-dependent RNA helicase DDX6 [Channa argus]

MASQCVEPMATANPAQMIKLNKPANGQLRGQTRPAGQQSGLLGTAQQNSTIQKRNSIPQSSGGIKFGDDWKKCLELPPKD

NRVKTSDVTSTKGNEFEDYCLKRELLMGIFEMGWEKPSPIQEESIPIALSGRDILARAKNGTGKSGAYLIPLLERIDHKK

DHIQAMVIVPTRELALQLSQISIQLSKHLSGVKIMASTGGTNLRDDIMRLDETVHVVIATPGRLLDLIKKGVAKVDKTQM

MVMDEADKLLSQDFVVLIEDIISFLSKDRQILLYSATFPVNVQKFMSNHLKKPYEINLMEELTLKGITQYYAYVTERQKV

HCLNTLFSRLQINQSIIFCNSTQRVELLAKKITQLGYSCFYIHAKMMQEYRNRVFHDFRNGLCRNLVCTDLFTRGIDIQA

VNVVINFDFPKSAETYLHRIGRSGRFGHLGLAINLITSDDRYNLKTVEDQLGTDIKPIPSSIDKSLYVAEFHCVDPDVED

CNGGAKNKEVGAA

>XP_035278278.1 probable ATP-dependent RNA helicase ddx6 [Anguilla anguilla]

MATARTENPASMVMGMSKPNGQLRGQPKPTSLPSGPTALGSQAGKAPSTPQKGGSALQNSGGIRFGDDWKKCLQLPPKDT

RVRTSDVTATKGNEFEDYCLKRELLMGIFEMGWEKPSPIQEESIPIALSGRDILARAKNGTGKSGAYLIPLLERIDLKKD

FIQAMVMVPTRELALQVSQISIQVAKHLGGVKVMATTGGTNLRDDIMRLDETVHVVIATPGRILDLIKKGVAKVDRLQMM

VMDEADKLLSQDFVVLIEDIISFLPKNRQILLYSATFPISVQKFMAKHLQKPYEINLMDELTLKGITQYYAYVTERQKVH

CLNTLFSRLQINQSIIFCNSTQRVELLAKKITQLGYSCFYIHAKMMQEYRNRVFHDFRNGLCRNLVCTDLFTRGIDIQAV

NVVINFDFPKNAETYLHRIGRSGRFGHLGLAINLITSEDRFNLKAIEDQLVTDIKPIPGSIDKSLYVAEFHSGSPECEGQ

EGDRETTHPLGELHQA

>XP_029587284.1 probable ATP-dependent RNA helicase ddx6 [Salmo trutta]

MAAARTENLGPVVMGLNKQNGQLRGPPKLASPQSGPLVLGSLSDEATGASQKAGAAQEGTGIRFGDDWKKSLQLPPKDTR

VRTSDVTSTKGNEFEDYCLKRELLMGIFEMGWEKPSPIQEESIPIALSGRDILARAKNGTGKSGAYLIPMLERIDLKKDY

IQAIVMVPTRELALQVSQISIQISKHLGGVKVMATTGGTNLRDDIMRLDETVHVVIATPGRILDLIKKGVAKVDRVQMMV

MDEADKLLSQDFVVLIEDIISFLAKGRQILLYSATFPISVQKFMAKHLQKPYEINLMEELTLKGITQYYAYVTERQKVHC

LNTLFSRLQINQSIIFCNSTQRVELLAKKITQLGYSCFYIHAKMMQEYRNRVFHDFRNGLCRNLVCTDLFTRGIDIQAVN

VVINFDFPKNAETYLHRIGRSGRFGHLGLAINLITSEDRFNLKSIEDQLVTDIKPIPGSIDKNLYVAEFHTANLDCEVEE

ELKETGCQQEET

>XP_023831223.1 probable ATP-dependent RNA helicase ddx6 [Salvelinus alpinus]

MASARTENLGPIGMGLNKQNGQLRGVPKPASPQSGPLVLGSLLSKAVGAPQKAGAALEGTGIRYGDDWKKSLQLPPKDTR

VRTSDVTSTKGNEFEDYCLKRELLMGIFEMGWEKPSPIQEESIPIALSGRDILARAKNGTGKSGAYLIPMLERIDLKKDY

IQAIVMVPTRELALQVSQISIQISKHLGGVKVMATTGGTNLRDDIMRLDETVHVVIATPGRILDLIKKGVAKVDRVQMMV

MDEADKLLSQDFVVLIEDIISFLGKGRQILLYSATFPISVQKFMAKHLQKPYEINLMEELTLKGITQYYAYVTERQKVHC

LNTLFSRLQINQSIIFCNSTQRVELLAKKITQLGYSCFYIHAKMMQEYRNRVFHDFRNGLCRNLVCTDLFTRGIDIQAVN

VVINFDFPKNAETYLHRIGRSGRFGHLGLAINLITSEDRFNLKSIEDQLVTDIKPIPGSIDKSLYVAEFHATNPNCEEEL

KETGRQKVEP

>XP_028327439.1 probable ATP-dependent RNA helicase ddx6 [Gouania willdenowi]

MATTTANPSALNGMNKASNGQLRGPPFGPHAAIQQANPPLSKTSFPQSSGGIKFGADWKNSLELPPKDTRVRTTDVTSTK

GNEFEDYCLKRELLMGIFEMGWEKPSPIQEESIPIALSGRDILARAKNGTGKSGAYLIPLLEKIDLKKDHIQAMVLVPTR

ELALQMSQITIQLSKHLGGVKVMATTGGTNLRDDILRLDETVHVVIATPGRILDLMRKGVAKVNQAQMMVMDEADKLLSQ

DFVVLVEDIISFFPKERQILLYSATFPMSVQLFMSKQLQKPYEINLMEELTLKGITQYYAYVTERQKVHCLNTLFSRLQI

NQSIIFCNSTQRVELLAKKITQLGYSCFYIHAKMMQEYRNRVFHDFRNGLCRNLVCTDLFTRGIDIQAVNVVINFDFPRS

AETYLHRIGRSGRFGHLGLAINLISSEDRYNLKTIEDQLVTDIKPIPSSIDKSLYVAEFHSLHTHTHHQEEEEEEERPEI

QEVGLV

>XP_014032535.1 PREDICTED: probable ATP-dependent RNA helicase ddx6 [Salmo salar]

MAAARTENLGPVVMGLNKQNGQLRGPPKLASPQSGPLVLGSLSDEATGASQKAGAAQEGTGIRFGDDWKKSLQLPPKDTR

VRTSDVTSTKGNEFEDYCLKRELLMGIFEMGWEKPSPIQEESIPIALSGRDILARAKNGTGKSGAYLIPMLERIDLKKDY

IQAIVMVPTRELALQVSQISIQISKHLGGVKVMATTGGTNLRDDIMRLDETVHVVIATPGRILDLIKKGVAKVDRVQMMV

MDEADKLLSQDFVVLIEDIISFLAKGRQILLYSATFPISVQKFMAKHLQKPYEINLMEELTLKGITQYYAYVTERQKVHC

LNTLFSRLQINQSIIFCNSTQRVELLAKKITQLGYSCFYIHAKMMQEYRNRVFHDFRNGLCRNLVCTDLFTRGIDIQAVN

VVINFDFPKNAETYLHRIGRSGRFGHLGLAINLITSEDRFNLKSIEDQLVTDIKPIPGSIDKNLYVAEFHTANLDCEVEE

ELKETGCQQEEP

>XP_015247140.1 PREDICTED: probable ATP-dependent RNA helicase ddx6 [Cyprinodon variegatus]

MATARTENLGPVMMGLNKQNGQLRGQTKPAAGPPTSTPAKNPAGSNQEGGGIKFGDDWKKCLQLPPRDNRVKTSDVTATK

GNEFEDYCLKRELLMGIFEMGWEKPSPIQEESIPIALSGRDILARAKNGTGKSGAYLIPLLERIDLKKDHIQAIVMVPTR

ELALQMSQICIQLSKHLGGVKVMATTGGTNLRDDIMRLDETVHVVIATPGRILDLIKKGVAKVDRVQMMVMDEADKLLSQ

DFVVLIEDIISFLAKNRQILLYSATFPISVQKFMVKHLQKPYEINLMEELTLKGITQFYAYVTERQKVHCLNTLFSRLQI

NQSIIFCNSTQRVELLAKKITQLGYSCFYIHAKMMQEYRNRVFHDFRNGLCRNLVCTDLFTRGIDIQAVNVVINFDFPKN

AETYLHRIGRSGRFGHLGLAINLITSEDRFNLKAIEEQLVTDIKPIPSSIDRSLYVAEYHTSSGDCEVEEVEEKPQRQQD

ST

>XP_035649465.1 probable ATP-dependent RNA helicase ddx6 [Oncorhynchus keta]

MASARTENLGPIGMGLNKQNGQLRGVPKPASPQSRPLVLGSLLSKAVGAPQKAGASLEGTGIRFGDDWKKSLQLPPKDTR

VRTSDVTSTKGNEFEDYCLKRELLMGIFEMGWEKPSPIQEESIPIALSGRDILARAKNGTGKSGAYLIPMLERIDLKKDY

IQAIVMVPTRELALQVSQISIQISKHLGGVKVMATTGGTNLRDDIMRLDETVHVVIATPGRILDLIKKGVAKVDRVQMMV

MDEADKLLSQDFVVLIEDIISFLGKGRQILLYSATFPISVQKFMAKHLHKPYEINLMEELTLKGITQYYAYVTERQKVHC

LNTLFSRLQINQSIIFCNSTQRVELLAKKITQLGYSCFYIHAKMMQEYRNRVFHDFRNGLCRNLVCTDLFTRGIDIQAVN

VVINFDFPKNAETYLHRIGRSGRFGHLGLAINLITSEDRFNLKSIEDQLVTDIKPIPGSIDKSLYVAEFHATNPNCEEGL

KETGRQQVEP

>XP_013997641.1 PREDICTED: probable ATP-dependent RNA helicase ddx6 isoform X2 [Salmo salar]

MFGDDWKKSLQLPPKDTRVRTSDVTSTKGNEFEDYCLKRELLMGIFEMGWEKPSPIQEESIPIALSGRDILARAKNGTGK

SGAYLIPMLERIDLKKDYIQAIVMVPTRELALQVSQISIQISKHLGGVKVMATTGGTNLRDDIMRLDETVHVVIATPGRI

LDLIKKGVAKVDRVQMMVMDEADKLLSQDFVVLIEDIISFLGKGRQILLYSATFPISVQKFMAKHLQKPYEINLMEELTL

KGITQYYAYVTERQKVHCLNTLFSRLQINQSIIFCNSTQRVELLAKKITQLGYSCFYIHAKMMQEYRNRVFHDFRNGLCR

NLVCTDLFTRGIDIQAVNVVINFDFPKNAETYLHRIGRSGRFGHLGLAINLITSEDRFNLKSIEDQLVTDIKPIPGSIDK

SLYVAEFHATNPNCEEELKETGRQQVEP

>XP_029513825.1 probable ATP-dependent RNA helicase ddx6 isoform X1 [Oncorhynchus nerka]

MKAEMAAARTENLGPVVMGLNKQNGQLRGPPKPASPQSGPLVLGSLSDEATGASQKAGAAQEGTGIRFGDDWKKSLQLPP

KDTRVRTSDVTSTKGNEFEDYCLKRELLMGIFEMGWEKPSPIQEESIPIALSGRDILARAKNGTGKSGAYLIPMLERIDL

KKDYIQAIVMVPTRELALQVSQISIQISKHLGGVKVMATTGGTNLRDDIMRLDETVHVVIATPGRILDLIKKGVAKVDRV

QMMVMDEADKLLSQDFVVLIEDIISFLAKGRQILLYSATFPISVQKFMAKHLQKPYEINLMEELTLKGITQYYAYVTERQ

KVHCLNTLFSRLQINQSIIFCNSTQRVELLAKKITQLGYSCFYIHAKMMQEYRNRVFHDFRNGLCRNLVCTDLFTRGIDI

QAVNVVINFDFPKNAETYLHRIGRSGRFGHLGLAINLITSEDRFNLKSIEDQLVTDIKPIPGSIDKNLYVAEFHTANLDC

EVEEELKETGCQQEEP

>XP_029519668.1 probable ATP-dependent RNA helicase ddx6 [Oncorhynchus nerka]

MASARTENLGPIGMGLNKQNGQLRGVPKPASPQSGPLVLGSLLSKAVGAPQKAGASLEGTGIRFGDDWKKSLQLPPKDTR

VRTSDVTSTKGNEFEDYCLKRELLMGIFEMGWEKPSPIQEESIPIALSGRDILARAKNGTGKSGAYLIPMLERIDLKKDY

IQAIVMVPTRELALQVSQISIQISKHLGGVKVMATTGGTNLRDDIMRLDETVHVVIATPGRILDLIKKGVAKVDRVQMMV

MDEADKLLSQDFVVLIEDIISFLGKGRQILLYSATFPISVQKFMAKHLHKPYEINLMEELTLKGITQYYAYVTERQKVHC

LNTLFSRLQINQSIIFCNSTQRVELLAKKITQLGYSCFYIHAKMMQEYRNRVFHDFRNGLCRNLVCTDLFTRGIDIQAVN

VVINFDFPKNAETYLHRIGRSGRFGHLGLAINLITSEDRFNLKSIEDQLVTDIKPIPGSIDKSLYVAEFHATNPNCEEGL

KETGRQQVEP

>XP_023119442.1 probable ATP-dependent RNA helicase ddx6 isoform X1 [Amphiprion ocellaris]

MATARTANPAPMIGLNKPANGQLRGQTKPVAQQAGLHSTVQQLSAPQERTSIPQSSRGIRFGDDWKKLLELPPKDNRMKT

SDVTSTKGNEFEDYCLKRELLMGIFEMGWEKPSPIQEESIPIALSGRDILARAKNGTGKSGAYLIPLLERIDLKKDHIQA

IVMVPTRELALQMSQISIQLSKHLGGVKVMATTGGTNLRDDILRLDEIVHVVVATPGRILDLIKKGVAKVDQTQMMVMDE

ADKLLSQDFVALIEDIISFLPKNRQILLYSATFPISVQKFMSKHLQKPYEINLMEELTLKGITQYYAYVTERQKVHCLNT

LFSRLQINQSIIFCNSTQRVELLAKKITQLGYSCFYIHAKMMQEYRNRVFHDFRNGLCRNLVCTDLFTRGIDIQAVNVVI

NFDFPKNAETYLHRIGRSGRFGHLGLAINLITSDDRYNLKTIEDQLITDIKPIPSSIDKSLYVAEFHSVDPDEDGDEGGA

KNKDLGAA

>XP_021427439.1 probable ATP-dependent RNA helicase ddx6 [Oncorhynchus mykiss]

MKAEMAAARTENLGPVVLGLNKQNGQLRGLPKPASPQSGPLVLGSLSDEATGASQKAGAAQEGTGIRFGDDWKKSLQLPP

KDTRVRTSDVTSTKGNEFEDYCLKRELLMGIFEMGWEKPSPIQEESIPIALSGRDILARAKNGTGKSGAYLIPMLERIDL

KKDYIQAIVMVPTRELALQVSQISIQISKHLGGVKVMATTGGTNLRDDIMRLDETVHVVIATPGRILDLIKKGVAKVDRV

QMMVMDEADKLLSQDFVVLIEDIISFLAKGRQILLYSATFPISVQKFMAKHLQKPYEINLMEELTLKGITQYYAYVTERQ

KVHCLNTLFSRLQINQSIIFCNSTQRVELLAKKITQLGYSCFYIHAKMMQEYRNRVFHDFRNGLCRNLVCTDLFTRGIDI

QAVNVVINFDFPKNAETYLHRIGRSGRFGHLGLAINLITSEDRFNLKSIEDQLVTDIKPIPGSIDKNLYVAEFHTANLDC

EVEEELKETGCQQEEP

>XP_018587778.2 probable ATP-dependent RNA helicase ddx6 [Scleropages formosus]

MATARTENPATMVMGLNKQNGQMRGQSKAATVPSATLSVGAQSGKIPSGSQKGMSAPQISSGIRFGDDWKKCLQLPPKDM

RVKTSDVTATKGNEFEDYCLKRELLMGIFEMGWEKPSPIQEESIPIALSGRDILARAKNGTGKSGAYLIPLLERIDLKKD

HIQAMVLVPTRELALQVSQISIQLSKHLGGVKIMATTGGTNLRDDIMRLDETVHVVIATPGRILDLIKKGVAKVDRVQMM

VMDEADKLLSQDFVVLIEEIIGFLAKNRQILLYSATFPISVQKFMSKHLQKPYEINLMDELTLKGITQYYAYVTEKQKVH

CLNTLFSRLQINQSIIFCNSTQRVELLAKKITQLGYSCFYIHAKMMQEYRNRVFHDFRNGLCRNLVCTDLFTRGIDIQAV

NVVINFDFPKNAETYLHRIGRSGRFGHLGLAINLITSEDRFNLKAIEDQLVTDIKPIPGTIDKSLYVAEFHSTNPDGEGE

EGDGEPGRRERHGP

>XP_023688989.1 probable ATP-dependent RNA helicase ddx6 [Paramormyrops kingsleyae]

MATARTENPATVVMGLNKQNGQMRGQPKPAPVSSGPLAVGSQPGKTSSAPQKGASVPQSGSGIKFGDDWKKCLQLPPKDL

RVKTSDVTATKGNEFEDYCLKRELLMGIFEMGWEKPSPIQEESIPIALSGRDILARAKNGTGKSGAYLIPLLERVDLKKD

YIQALVMVPTRELALQVSQISIQISKHLGGVKIMATTGGTNLRDDIMRLDETVHVVIATPGRILDLIKKGVAKVDRVQMM

VMDEADKLLSQDFVVLIEDIIGFLSKNRQILLYSATFPISVQKFMAKHLQKPYEINLMDELTLKGITQYYAYVTERQKVH

CLNTLFSRLQINQSIIFCNSTQRVELLAKKITQLGYSCFYIHAKMMQEYRNRVFHDFRNGLCRNLVCTDLFTRGIDIQAV

NVVINFDFPKNAETYLHRIGRSGRFGHLGLAINLITSEDRFNLKSIEDQLVTDIKPIPSSIDKSLYVAEFHSANPDREGE

EGAGEAGNKP

>XP_024297607.1 probable ATP-dependent RNA helicase ddx6 [Oncorhynchus tshawytscha]

MAAARTENLGPVVMGLNGQLRGPPKPASPQSGPLVLGSLSDEATGASQKAGAAQEGTGIRFGDDWKKSLQLPPKDTRVRT

SDVTSTKGNEFEDYCLKRELLMGIFEMGWEKPSPIQEESIPIALSGRDILARAKNGTGKSGAYLIPMLERIDLKKDYIQA

IVMVPTRELALQVSQISIQISKHLGGVKVMATTGGTNLRDDIMRLDETVHVVIATPGRILDLIKKGVAKVDRVQMMVMDE

ADKLLSQDFVVLIEDIISFLAKGRQILLYSATFPISVQKFMAKHLQKPYEINLMEELTLKGITQYYAYVTERQKVHCLNT

LFSRLQINQSIIFCNSTQRVELLAKKITQLGYSCFYIHAKMMQEYRNRVFHDFRNGLCRNLVCTDLFTRGIDIQAVNVVI

NFDFPKNAETYLHRIGRSGRFGHLGLAINLITSEDRFNLKSIEDQLVTDIKPIPGSIDKNLYVAEFHTANLDCEVEEELK

ETGCQQEEP

>XP_030644685.1 probable ATP-dependent RNA helicase ddx6 [Chanos chanos]

MASARTENAGPVVMGMNKQNGQLRGQPKPASPSGPQAANQLGKAPAGPQKAGSESQTGPGIRFGDDWKKSLQLPPKDNRV

RTSDVTATKGNEFEDYCLKRELLMGIFEMGWEKPSPIQEESIPIALSGRDILARAKNGTGKSGAYLIPMLERIDLKKDHI

QALVLVPTRELALQVSQISIQLSKHLGGVKVMATTGGTNLRDDIMRLDETVHVVIATPGRILDLMKKGVAKVDKVQMMVM

DEADKLLSQDFVVLIEDIIRFLTKNRQILLYSATFPISVQKFMAKHLQKPYEINLMEELTLKGITQYYAYVTERQKVHCL

NTLFSRLQINQSIIFCNSTQRVELLAKKITQLGYSCFYIHAKMMQEYRNRVFHDFRNGLCRNLVSTDLFTRGIDIQAVNV

VINFDFPKNAETYLHRIGRSGRYGHLGLAINLITAEDRFNLKAIEDQLVTDIKPIPSSIDKSLYVAEFHSASPDAEVEAE

EKDPPRPASKPQDP

>XP_024252004.1 probable ATP-dependent RNA helicase ddx6 isoform X1 [Oncorhynchus tshawytscha]

MASARTENLGPIGMGLNKQNGQLRGVPKPASPQSGPLVLGSLLSKTVGAPQKAGASLEGTGIRFGDDWKKSLQLPPKDTR

VRTSDVTSTKGNEFEDYCLKRELLMGIFEMGWEKPSPIQEESIPIALSGRDILARAKNGTGKSGAYLIPMLERIDLKKDY

IQAVVMVPTRELALQVSQISIQISKHLGGVKVMATTGGTNLRDDIMRLDETVHVVIATPGRILDLIKKGVAKVDRVQMMV

MDEADKLLSQDFVVLIEDIISFLGKGRQILLYSATFPISVQKFMAKHLHKPYEINLMEELTLKGITQYYAYVTERQKVHC

LNTLFSRLQINQSIIFCNSTQRVELLAKKITQLGYSCFYIHAKMMQEYRNRVFHDFRNGLCRNLVCTDLFTRGIDIQAVN

VVINFDFPKNAETYLHRIGRSGRFGHLGLAINLITSEDRFNLKSIEDQLVTDIKPIPGSIDKSLYVAEFHATNPNCEEGL

KETGRQQVEP

>XP_031649638.1 probable ATP-dependent RNA helicase ddx6 [Oncorhynchus kisutch]

MAAARTENLGPVVMGLNKQKGQLRGPPKPASPQSGPLVLGSLSDEATGASQKAGAAQEGTGIRFGDDWKKSLQLPPKDTR

VRTSDVTSTKGNEFEDYCLKRELLMGIFEMGWEKPSPIQEESIPIALSGRDILARAKNGTGKSGAYLIPMLERIDLKKDY

IQAIVMVPTRELALQVSQISIQISKHLGGVKVMATTGGTNLRDDIMRLDETVHVVIATPGRILDLIKKGVAKVDRVQMMV

MDEADKLLSQDFVVLIEDIISFLAKGRQILLYSATFPISVQKFMAKHLQKPYEINLMEELTLKGITQYYAYVTERQKVHC

LNTLFSRLQINQSIIFCNSTQRVELLAKKITQLGYSCFYIHAKMMQEYRNRVFHDFRNGLCRNLVCTDLFTRGIDIQAVN

VVINFDFPKNAETYLHRIGRSGRFGHLGLAINLITSEDRFNLKSIEDQLVTDIKPIPGSIDKNLYVAEFHTANLDCEVEE

ELKETGCQQEEP

>XP_010789435.1 PREDICTED: probable ATP-dependent RNA helicase ddx6 [Notothenia coriiceps]

MATARTANPMIGLNKPANGQLKGQMGLLSGVQQSSALQKKTSIPQSSGGIKFGDDWKKCLDLPPKDCRMRTSDVTSTKGN

EFEDYCLKRELLMGIFEMGWEKPSPVQEESIPIALSGRDILARAKNGTGKSGAYLIPLLERIDLKKDHIQAIVMVPTREL

ALQMSQISIQLSKHLGGVKVMATTGGTNLRDDIMRLDETVHVVIATPGRILDLIKKGVAKMDRAQLIVMDEADKLLSQDF

VVLIEDIISFMPKDRQILLYSATFPISVQKFMSKHLSKPYEINLMEELTLKGITQYYAYVTERQKVHCLNTLFSRLQINQ

SIIFCNSTQRVELLAKKITQLGYSCFYIHAKMMQEYRNRVFHDFRNGLCRNLVCTDLFTRGIDIQAVNVVINFDFPKNAE

TYLHRIGRSGRFGHLGLAINLITSEDRYNLKNIEDQLVTDIKPIPSCIDKSLYVAEFHSVDPDAEDDELEIGGRNKELGG

I

>XP_034017368.1 probable ATP-dependent RNA helicase ddx6 [Thalassophryne amazonica]

MATARTETIGPVTVGLTKQNGQLRGQTKPVSVQPASMTQGNPSGLPQKPGSASKDGGIKFGDDWKKCLQLPPKDNRVKTS

DVTATKGNEFEDYCLKRELLMGIFEMGWEKPSPIQEESIPIALSGRDILARAKNGTGKSGAYLIPLLERIDLKKDHIQAM

VIVPTRELALQVSQICIQISKHLGGVKVMATTGGTNLRDDIMRLDETVHVIIATPGRILDLIKKGLAKVDKVQIMVMDEA

DKLLSQDFVVLIEDIISFLAKNRQILLYSATFPISVQKFMTKHLQKPYEINLMEELTLKGITQYYAYVTERQKVHCLNTL

FSRLQINQSIIFCNSTQRVELLAKKITQLGYSCFYIHAKMMQEYRNRVFHDFRNGLCRNLVCTDLFTRGIDIQAVNVVIN

FDFPKNAETYLHRIGRSGRFGHLGLAINLITSEDRFNLKAIEDQLVTDIKPIPGSIDRSLYVAEYHTASGNCDGGEEDDD

KTGHQQDSI

>XP_020356307.1 probable ATP-dependent RNA helicase ddx6 isoform X1 [Oncorhynchus kisutch]

MASARTENLGPICMGLNKQNGQLRGVPKPASPQSGPLVLGSLLSKAVGAPQKAGASLEGTGIRFGDDWKKSLQLPPKDTR

VRTSDVTSTKGNEFEDYCLKRELLMGIFEMGWEKPSPIQEESIPIALSGRDILARAKNGTGKSGAYLIPMLERIDLKKDY

IQAIVMVPTRELALQVSQISIQISKHLGGVKVMATTGGTNLRDDIMRLDETVHVVIATPGRILDLIKKGVAKVDRVQMMV

MDEADKLLSQDFVVLIEDIISFLGKGRQILLYSATFPISVQKFMAKHLHKPYEINLMEELTLKGITQYYAYVTERQKVHC

LNTLFSRLQINQSIIFCNSTQRVELLAKKITQLGYSCFYIHAKMMQEYRNRVFHDFRNGLCRNLVCTDLFTRGIDIQAVN

VVINFDFPKNAETYLHRIGRSGRFGHLGLAINLITSEDRFNLKSIEDQLVTDIKPIPGSIDKSLYVAEFHATNPNCEEGL

KETGRQQVEP

>XP_029513826.1 probable ATP-dependent RNA helicase ddx6 isoform X2 [Oncorhynchus nerka]

MAAARTENLGPVVMGLNKQNGQLRGPPKPASPQSGPLVLGSLSDEATGASQKAGAAQEGTGIRFGDDWKKSLQLPPKDTR

VRTSDVTSTKGNEFEDYCLKRELLMGIFEMGWEKPSPIQEESIPIALSGRDILARAKNGTGKSGAYLIPMLERIDLKKDY

IQAIVMVPTRELALQVSQISIQISKHLGGVKVMATTGGTNLRDDIMRLDETVHVVIATPGRILDLIKKGVAKVDRVQMMV

MDEADKLLSQDFVVLIEDIISFLAKGRQILLYSATFPISVQKFMAKHLQKPYEINLMEELTLKGITQYYAYVTERQKVHC

LNTLFSRLQINQSIIFCNSTQRVELLAKKITQLGYSCFYIHAKMMQEYRNRVFHDFRNGLCRNLVCTDLFTRGIDIQAVN

VVINFDFPKNAETYLHRIGRSGRFGHLGLAINLITSEDRFNLKSIEDQLVTDIKPIPGSIDKNLYVAEFHTANLDCEVEE

ELKETGCQQEEP

>XP_012707030.2 probable ATP-dependent RNA helicase ddx6 [Fundulus heteroclitus]

MATARTENLGPVVMGLNKQNGQLRGQPKPAAGPPASAQGKNPAGGNQDGGGIRFGDDWKKCLQLPPRDTRVKTSDVTATK

GNEFEDYCLKRELLMGIFEMGWEKPSPIQEESIPIALSGRDILARAKNGTGKSGAYLIPLLERIDLKKDHIQAVVMVPTR

ELALQMSQICIQLSKHLGGVKVMATTGGTNLRDDIMRLDETVHVVIATPGRILDLIKKGVAKVDRVQMMVMDEADKLLSQ

DFVVLIEDIISFLAKNRQILLYSATFPISVQKFMVKHLQKPYEINLMEELTLKGITQFYAYVTERQKVHCLNTLFSRLQI

NQSIIFCNSTQRVELLAKKITQLGYSCFYIHAKMMQEYRNRVFHDFRNGLCRNLVCTDLFTRGIDIQAVNVVINFDFPKN

AETYLHRIGRSGRFGHLGLAINLITSEDRFNLKAIEEQLVTDIKPIPSSIDKSLYVAEYHSSSGDCDVEEVEEKPHRPQD

ST

>XP_018527883.1 PREDICTED: probable ATP-dependent RNA helicase ddx6 [Lates calcarifer]

MATARTANPAPMIGLNKAANGQFRGQTKPAGPQSGLLATVQQSGDPQKRSSIPQNSGGIRFGDDWKKCLELPPKDTRLKT

SDVTSTKGNEFEDYCLKRELLMGIFEMGWEKPSPVQEESIPIALSGRDILARAKNGTGKSGAYLIPLLERIDLKKDHIQA

MVLVPTRELALQMSQISIQLSKHLGGVKVMATTGGTNLRDDIMRLDEIVHVVIATPGRILDLIKKGVAKVNKTQMMVMDE

ADKLLSQDFVVLIEDIISFLPKDRQILLYSATFPISVQKFMSKHLQKPYEINLMEELTLKGITQYYAYVTERQKVHCLNT

LFSRLQINQSIIFCNSTQRVELLAKKITQLGYSCFYIHAKMMQEYRNRVFHDFRNGLCRNLVCTDLFTRGIDIQAVNVVI

NFDFPKNAETYLHRIGRSGRFGHLGLAINLITSDDRYNLKTIEDQLVTDIKPIPSSIDKSLYVAEFHSVDPDDDDNDGEL

KNKELGAA

>XP_031581964.1 probable ATP-dependent RNA helicase DDX6 [Oreochromis aureus]

MATARTANPAPMAGLNKPANGQLRGQTKTGGQQPELLSNVQHPSAHKRTSIPQSSGGIKFGDDWKKCLELPPKDTRVKTS

DVTSTKGNEFEDYCLKRELLMGIFEMGWEKPSPVQEESIPIALSGRDILARAKNGTGKSGAYLIPLLERIDLKKDHIQAI

VMVPTRELALQMSQISIQLSKHLGGVKIMATTGGTNLRDDIMRLDETVHVVIATPGRILDLIKKGVAKVDKNQMVVMDEA

DKLLSQDFVVLIENIISFMPKNRQILLYSATFPISVQKFMSKHMQKPYEINLMEELTLKGITQYYAYVTERQKVHCLNTL

FSRLQINQSIIFCNSTQRVELLAKKITQLGYSCFYIHAKMMQEYRNRVFHDFRNGLCRNLVCTDLFTRGIDIQAVNVVIN

FDFPKNAETYLHRIGRSGRFGHLGLAINLITSDDRYNLKNIEDQLVTDIKPIPSSIDKSLYVAEFHSVDAEDGGEEKA

>XP_028280657.1 probable ATP-dependent RNA helicase ddx6 [Parambassis ranga]

MATARTANPASMLGLNKPSNGQLRGQTKPAGQQSGLLSTAQPPSALQNRTSIPQSSGGIKFGDDWKRCLELPPKDNRVKT

SDVTSTKGNEFEDYCLKRELLMGIFEMGWEKPSPIQEESIPIALSGRDILARAKNGTGKSGAYLIPLLERIDLKKDHIQA

LVMVPTRELALQMSQISIQISKHLGGVKVMATTGGTNLRDDILRLDEIVHVVIATPGRILDLIKKGVAKVDKAQMIVMDE

ADKLLSQDFVILIEDIISFMPKNRQILLYSATFPISVQKFMSKHLKKPYEINLMEELTLKGITQYYAYVTERQKVHCLNT

LFSKLQINQSIIFCNSTQRVELLAKKITQLGYSCFYIHAKMMQEYRNRVFHDFRNGLCRNLVCTDLFTRGIDIQAVNVVI

NFDFPKNAETYLHRIGRSGRFGHLGLAINLITSDDRYNLKTIEDQLVTDIKPIPSSIDKSLYVAEFHSVNPDDDEHGDEE

GAKHKE

>XP_005999191.1 PREDICTED: probable ATP-dependent RNA helicase ddx6 [Latimeria chalumnae]

MATARTENPLVVGLSKQNGQVRAQQKVASGPPGIGQLPQNQPFKTPTSGQSLPQKPATQQQGSGMTAIRFGDDWKKSLQL

PPKDMRIRTSDVTATKGNEFEDYCLKRELLMGIFEMGWEKPSPIQEESIPIALSGRDILARAKNGTGKSGAYLIPLLERL

DLKKDCIQAIVMVPTRELALQVSQISIQLGKRMGDLKVMATTGGTNLRDDIMRLDETVHVIIGTPGRLLDLVKKGVAKVD

RTQMIVMDEADKLLSQDFLVILEEIISFLPKNRQILLYSATFPISVQKFMSKYLQKPYEINLMDELTLKGITQFYAYVTE

RQKVHCLNTLFSRLQINQSIIFCNSTQRVELLAKKITQLGYSCFYIHAKMMQEYRNRVFHDFRNGLCRNLVCTDLFTRGI

DIQAVNVVINFDFPKNAETYLHRIGRSGRFGHLGLAINLITSDDRFNLKGIEDQLCTEIKPIPSSIDKSLYVAEYHSQSP

EGEEEGQ

>XP_010899187.1 probable ATP-dependent RNA helicase ddx6 [Esox lucius]

MATPRTENLGPVVMGLNKQNGQLRGPPKSPSPQSGPLHLGSMTGKASGAPQKAGAAQEGTGIRFGDDWKKCLQLPPKDTR

VRTSDVTSTKGNEFEDYCLKRELLMGIFEMGWEKPSPIQEESIPIALSGRDILARAKNGTGKSGAYLIPMLERIDLKKDY

IQAIVMVPTRELALQVSQISIQISKHLGGVKVMATTGGTNLRDDIMRLDETVHVVIATPGRILDLIKKGVAKVDRVQMMV

MDEADKLLSQDFVVLIEDIISFLAKGRQILLYSATFPISVQKFMAKHLQKPYEINLMEELTLKGITQYYAYVTERQKVHC

LNTLFSRLQINQSIIFCNSTQRVELLAKKITQLGYSCFYIHAKMMQEYRNRVFHDFRNGLCRNLVCTDLFTRGIDIQAVN

VVINFDFPKNAETYLHRIGRSGRFGHLGLAINLITSEDRFNLKSIEDQLVTDIKPIPGSIDKSLYVAEFHTANPNCEVDE

ELKETGHQQGEL

>XP_022053718.1 probable ATP-dependent RNA helicase ddx6 [Acanthochromis polyacanthus]

MATTRTANPAAMIGLNKPANGQLRGQTKPGAQQLGLHSTVQQLSAPQERTSIPQSSGSIRFGDDWKKLLELPPKDNRMKT

SDVTSTKGNEFEDYCLKRELLMGIFEMGWEKPSPIQEESIPIALSGRDILARAKNGTGKSGAYLIPLLERIDLKKDHIQA

IVMVPTRELALQMSQISIQLGKHLGGVKIMATTGGTNLRDDILRLDEMVHVVVATPGRILDLIKKGVAKVDQTQMMVMDE

ADKLLSQDFVALIEDIISFLPKNRQILLYSATFPITVQKFMSKHLQKPYEINLMEELTLKGITQYYAYVTERQKVHCLNT

LFARLQINQSIIFCNSTQRVELLAKKITQLGYSCFYIHAKMMQEYRNRVFHDFRNGLCRNLVCTDLFTRGIDIQAVNVVI

NFDFPKNAETYLHRIGRSGRFGHLGLAINLITSEDRYNLKTIEDQLITDIKPIPSSIDKSLYVAEFHSVDPDEDGDEGGA

KNKDLGAA

>KAF4080430.1 hypothetical protein AMELA_G00171140 [Ameiurus melas]

MATAKMESVSAVAMGISKQNGQLRGLSSQSGPSVQSNPFARVSTGGQKPAADSQEGPGIRFGEDWKKSLQLPPKDNRMKT

SDVTATKGNEFEDYCLKRELLMGIFEMGWEKPSPVQEESIPIALSGRDILARAKNGTGKSGAYLIPMLERIDLKKDYIQA

LVMVPTRELALQVSQISIQLSKHLGGVKVMATTGGTNLRDDIMRLDEIVHVVIATPGRILDLIKKGVAKVDKVQIMVMDE

ADKLLSQDFVVLIEDIISFLAKNRQILLYSATFPVTVQKFMAKYLKKPYEINLMEELTLKGITQYYAYVTERQKVHCLNT

LFSRLQINQSIIFCNSTQRVELLAKKITQLGYSCFYIHAKMMQEYRNRVFHDFRNGLCRNLVCTDLFTRGIDIQAVNVVI

NFDFPKNAETYLHRIGRSGRFGHLGLAINLITAEDRFNLKAIEDQLVTDIKPIPSSIDKSLYVAEFHSANDEAEEQETPE

EP

>XP_029959213.1 probable ATP-dependent RNA helicase ddx6 [Salarias fasciatus]

MATARTANPALMIGMNNPTNGQLRGQTKSAGQQSGLFSVGQQPSVTQKQTTIPQSSGGIKFGDDWKKCLELPPKDHRVKT

SDVTSTKGNEFEDYCLKRELLMGIFEMGWEKPSPIQEESIPIALSGRDILARAKNGTGKSGAYLIPLLERIDLKKDHIQA

IVMVPTRELALQMSQISIQLSKHLGGVKVMATTGGTNLRDDIMRLDEIVHVVIATPGRILDLIKKGVAKVDRVQMMVMDE

ADKLLSQDFVILIEDIISFLARDRQILLYSATFPISVQKFMAKHLKKPYEINLMEELTLKGITQYYAYVTERQKVHCLNT

LFSRLQINQSIIFCNSTQRVELLAKKITQLGYSCFYIHAKMMQEYRNRVFHDFRNGLCRNLVCTDLFTRGIDIQAVNVVI

NFDFPKSAETYLHRIGRSGRFGHLGLAINLITSDDRYNLKTIEDQLVTDIKPIPGSIDKSLYVAEFHSVDQEEEEDEREA

RLKALGAA

>XP_017336712.1 PREDICTED: probable ATP-dependent RNA helicase ddx6 [Ictalurus punctatus]

MATAKMESVGAVAMGISKQNGQLRGLSSQSGPSVQSNPFARVSTGGQKPAADSQEGPGIRFGEDWKKSLQLPPKDNRMKT

SDVTATKGNEFEDYCLKRELLMGIFEMGWEKPSPVQEESIPIALSGRDILARAKNGTGKSGAYLIPMLERIDLKKDYIQA

LVMVPTRELALQVSQISIQLSKHLGGVKVMATTGGTNLRDDIMRLDEIVHVVIATPGRILDLIKKGVAKVDKVQIMVMDE

ADKLLSQDFVVLIEDIISFLAKNRQILLYSATFPVTVQKFMAKYLKKPYEINLMEELTLKGITQYYAYVTERQKVHCLNT

LFSRLQINQSIIFCNSTQRVELLAKKITQLGYSCFYIHAKMMQEYRNRVFHDFRNGLCRNLVCTDLFTRGIDIQAVNVVI

NFDFPKNAETYLHRIGRSGRFGHLGLAINLITAEDRFNLKAIEDQLVTDIKPIPSSIDKSLYVAEFHSANDEAEEQETPE

EP

>XP_035650942.1 probable ATP-dependent RNA helicase ddx6 [Oncorhynchus keta]

MAAARTENLGPVVMGLNKQNGQLRGPPKPASPQSGPLVLGSLSDEATGASQKAGAAQEGTGIRFGDDWKKSLQLPPKDTR

VRTSDVTSTKGNEFEDYCLKRELLMGIFEMGWEKPSPIQEESIPIALAGRDILARAKNGTGKSGAYLIPMLERIDLKKDY

IQAIVMVPTRELALQVSQISIQISKHLGGVKVMATTGGTNLRDDIMRLDETVHVVIATPGRILDLIKKGVAKVDRVQMMV

MDEADKLLSQDFVVLIEDIISFLAKGRQILLYSATFPISVQKFMGKHLQKPYEINLMEELTLKGITQYYAYVTERQKVHC

LNTLFSRLQINQSIIFCNSTQRVELLAKKITQLGYSCFYIHAKMMQEYRNRVFHDFRNGLCRNLVCTDLFTRGIDIQAVN

VVINFDFPKNAETYLHRIGRSGRFGHLGLAINLITSEDRFNLKSIEDQLVTDIKPIPGSIDKNLYVAEFHTAGLDCEVEE

ELKETGCQQEEP

>KAF4100628.1 hypothetical protein G5714_018824 [Onychostoma macrolepis]

MATKMENTGPAVMGLNTHNRQLIGQPKAPTQLGPQTVSAQPGKEPMGTQKPANASQEGPGIRFGDDWKKSLQLPDKDNRV

KTSDVTATKGNEFEDYCLKRELLMGIFEMGWEKPSPIQEESIPIALSGRDILARAKNGTGKSGAYLIPLLERIDIKKDHI

QAIVIVPTRELALQVSQISIQISKHLGVKVMATTGGTNLRDDIMRLDEEVHVVIATPGRILDLIKKGIAKVDKVQMMVMD

EADKLLSQDFVVIIEEIIGFLSKNRQILLYSATFPISVQKFMAKHLQKPYEINLMEELTLKGITQYYAYVTERQKVHCLN

TLFSRLQINQSIIFCNSTQRVELLAKKITQLGYSCFYIHAKMMQEYRNRVFHDFRNGLCRNLVCTDLFTRGIDIQAVNVV

INFDFPRNAETYLHRIGRSGRFGHLGLAINLITAEDRFNLKTIEDQLMTDIKPIPGSIDKSLYVAEFHSLNPDCEAEAPQ

PGEGSEAH

>XP_035570041.1 LOW QUALITY PROTEIN: probable ATP-dependent RNA helicase DDX6 [Canis lupus dingo]

MSTARTENPVIMGLSSQNGQLRGPVKPSGGPGGGGTQTQQQMNQLKNTNTINNGTQQQAQSMTTTIKPGDDWKKTLKLPP

KDLRIKTSDVTSTKGNEFEDYCLKWELLMGIFEMGWEKPSPIQEESIPIALSGRDILARAKNGTGKSGAYFIPLLERLDL

KKDNIQAMVIVPTREVALQVSQICIQVSKHMGGAKVMATTGGTNLRDDIMRLDDTVHVVIATPGRILDVIKKGAAKVDHV

QMIVLDEADKLLSQDFVQIMEDIILTLPKNRQILLYSATFPLSVQKFMNSHLRKPCEINLMEELTLKGVTQYYAYVTERQ

KVLCLNTLFSRLQINQSIIFCNSSQRVELLAKISLLGYSCFYIHAKMRQEHRNRVFHDFRNGLCRNLVCTDLFPRGIDIQ

AVNVVINFDFPKLAETYLYRIGRSGRFGHLGLAINLITYDDRFNLKNIEEQLGTEIKPIPSNIDKSLYEAEYHSEPVEDE

KP

>KTG41596.1 hypothetical protein cypCar_00025551 [Cyprinus carpio]

MATARTEIPSSVMPMSKQNGQAKPMTLQSGSHTSSTQSGKTPVMPQKGSSIHQSSGGIRFGDDWKKCLQLPPKDLRVRTT

DVTATKGNEFEDYCLKRELLMGIFEMGWEKPSPIQEESIPIVLSGRDILARAKNGTGKSGAYLIPLLERIDLKKDYVQAI

VFVPTRELALQVSQISINMSKHLGGVKVMATTGGTNLRDDIMRLDETVHVIIATPGRILDLIKKGVAKVDKAQMIVMDEA

DKLLSQDFVVLIEDIISFLPKKRQILLYSATFPISVQKFMTKHLQKPYEINLMDELTLKGITQYYAYVTERQKVHCLNTL

FSRLQINQSIIFCNSTQRVELLAKKITQLGYSCFYIHAKMMQEYRNRVFHDFRNGLCRNLVCTDLFTRGIDIQAVNVVIN

FDFPKNAETYLHRIGRSGRYGHLGLAINLITSEDRFNLKGIEDQLMTDIKPIPSSIDKSLYVADFHTMNPDEEEAAHKAG

ELN

>ROL47794.1 putative ATP-dependent RNA helicase DDX6 [Anabarilius grahami]

MATARTEIPASVMQMTKQNGQSKPMTLQSGSLTSLTQSGKTPMMPQKGRSIPQSSGGIRFGDDWKKCLQLPPKDMRVRTT

DVTATKGNEFEDYCLKRELLMGIFEMGWEKPSPIQEESIPIVLSGRDILARAKNGTGKSGAYLIPLLERIDLKKDHVQAL

VMVPTRELALQVSQISINMSKHLGGVKVMATTGGTNLRDDIMRLDETVHVIIATPGRILDLIKKGVAKVDKAQMVVMDEA

DKLLSQDFVVLIEDIISFLPKNRQILLYSATFPISVQKFMSKHLQKPYEINLMDELTLKGITQYYAYVTERQKVHCLNTL

FSRLQINQSIIFCNSTQRVELLAKKITQLGYSCFYIHAKMMQEYRNRVFHDFRNGLCRNLVCTDLFTRGIDIQAVNVVIN

FDFPKNAETYLHRIGRSGRYGHLGLAINLITSEDRFNLKGIEDQLMTDIKPIPSSIDKSLYVAEFHSMNPDEEEAAHKAG

DLN

>XP_004074359.1 probable ATP-dependent RNA helicase ddx6 [Oryzias latipes]

MATARTENVGPMVMGLNKQNGQLRGQNKPPAVQPAPPGQGKGFGAHQKAGGPPQDGGGIKFGDDWKRSLLLPPKDNRVKT

SDVTATKGNEFEDYCLKRELLMGIFEMGWEKPSPIQEESIPIALSGRDILARAKNGTGKSGAYLIPLLERIDLKKDHIQA

IVMVPTRELALQVSQISIQISKHMGGVKVMATTGGTNLRDDIMRLDEIVHVVIATPGRILDLIKKGVAKVDRVQMIVMDE

ADKLLSQDFVVLIEDIISFLAKNRQILLYSATFPMSVQKFMAKHLQKPYEINLMEELTLKGITQFYAYVTERQKVHCLNT

LFSRLQINQSIIFCNSTQRVELLAKKITQLGYSCFYIHAKMMQEYRNRVFHDFRNGLCRNLVCTDLFTRGIDIQAVNVVI

NFDFPKNAETYLHRIGRSGRFGHLGLAINLITSEDRFNLKAIEEQLVTDIKPIPSSIDKSLYVAEYHSSGADGDGEDKPE

RQQDST

>XP_026083120.1 probable ATP-dependent RNA helicase ddx6 [Carassius auratus]

MATARTEIPSSVMPMSKQNGQSKPMTLQSGSLTSSTQSGKTPVMPQKGSSIHQSSGGIRFGDDWKKCLQLPPKDLRVRTT

DVTATKGNEFEDYCLKRELLMGIFEMGWEKPSPIQEESIPIVLSGRDILARAKNGTGKSGAYLIPLLERIDLKKDYVQAI

VFVPTRELALQVSQISINMSKHLGGVKVMATTGGTNLRDDIMRLDETVHVIIATPGRILDLIKKGVAKVDKAQMIVMDEA

DKLLSQDFVVLIEDIISFLPKKRQILLYSATFPVSVQKFMSKHLQKPYEINLMDELTLKGITQYYAYVTERQKVHCLNTL

FSRLQINQSIIFCNSTQRVELLAKKITQLGYSCFYIHAKMMQEYRNRVFHDFRNGLCRNLVCTDLFTRGIDIQAVNVVIN

FDFPKNAETYLHRIGRSGRYGHLGLAINLITSEDRFNLKGIEDQLMTDIKPIPSSIDKSLYVADFHTMNPDEEEAAHKAG

DLN

>XP_029359731.1 probable ATP-dependent RNA helicase ddx6 [Echeneis naucrates]

MATARTANPAPMIGLNKAANGQLRGQTKPGGEQSGVFSNAQQPSAPEHRSSIPQNSGGIKFGDDWKKCLELPPKDTRMKT

SDVTSTKGNEFEDYCLKRELLMGIFEMGWEKPSPIQEESIPIALSGRDILARAKNGTGKSGAYLIPLLERIDLKKDHIQA

IVMVPTRELALQVSQISIQLGKHLGGVKVMATTGGTNLRDDIMRLDEIVHVIIATPGRILDLIKKGVAKVDKTQIMVMDE

ADKLLSQDFVVLIEDIIGFLPKSRQILLYSATFPISVQKFMSKHLQKPYEINLMEELTLKGITQYYAYVTERQKVHCLNT

LFSRLQINQSIIFCNSTQRVELLAKKITQLGYSCFYIHAKMMQEYRNRVFHDFRNGLCRNLVCTDLFTRGIDIQAVNVVI

NFDFPKSAETYLHRIGRSGRFGHLGLAINLITSDDRYNLKTIEDQLVTDIKPIPSSIDKSLYVAEFHSVDPDDDDGEHKN

KELGSA

>XP_030639290.1 probable ATP-dependent RNA helicase ddx6 [Chanos chanos]

MATARTENPASVMGLTKQNGQLRGQPKPLGLQPGGPIAASTQPGKAPSAPPTGSSIPQSSGGIRFGDDWKKCLQLPPKDM

RVQTSDVTATKGNEFEDYCLKRELLMGIFEMGWEKPSPIQEESIPIALSGRDILARAKNGTGKSGAYLIPLLERIDVKKE

HIQAVVLVPTRELALQVSQISIQISKHLSGMKIMATTGGTNLRDDIMRLDEIVHVVIATPGRILDLIKKGVAKMDKVQTM

VMDEADKLLSQDFVVLIEDIISFLPKNRQILLYSATFPISVQKFMVKHLQKPYEINLMDELTLKGITQYYAYVTERQKVH

CLNTLFSRLQINQSIIFCNSTQRVELLAKKITQLGYSCFYIHAKMMQEYRNRVFHDFRNGLCRNLVCTDLFTRGIDIQAV

NVVINFDFPKNAETYLHRIGRSGRYGHLGLAINLITSEDRFSLKTIEDQLITDIKPIPSSIDKSLYVAEFHSAIPDAEEA

EKETAAKPGDTTAP

>XP_007249635.1 probable ATP-dependent RNA helicase DDX6 [Astyanax mexicanus]

MATARTELFASALGLSKQNGQSRPPGLQSGPVTGMTQPGKALGDSQKGSSIPQTSGGIRFGDDWKKSLQLPPKDTRVKTS

DVTATKGNDFEDYCLKRELLMGIFEMGWEKPSPIQEESIPIALSGRDILARAKNGTGKSGAYLIPLLERIDLKKDHIQAV

VMVPTRELALQMSQICISMSKHLGGVKVMATTGGTNLKDDIMRLDETVHVVIATPGRILDLIKKGVAKVDKVQMMVMDEA

DKLLSQDFVVLIEDIISFLDKNRQILLYSATFPVSVQRFMAKHLRKPYEINLMDELTLKGITQYYAYVTERQKVHCLNTL

FSRLQINQSIIFCNSTQRVELLARKISQLGYSCFYIHAKMMQEYRNRVFHDFRNGLCRNLVCTDLFTRGIDIQAVNVVIN

FDFPKNAETYLHRIGRSGRYGHLGLAINLITSDDRFNLKTIEEQLVTDIKPIPGSIDKSLYVAEFHSTNPDEEEEEEEPR

AEINAP

>XP_012685001.2 probable ATP-dependent RNA helicase ddx6 [Clupea harengus]

MATARTESMGPMVMGLNKQNGQLRGQTKPLSQSGPHSVSTPPGKMTVGGGALKPGGGTQEGGIRFGDDWKKSLLLPPKDT

RVRTSDVTSTKGNEFEDYCLKRELLMGIFEMGWEKPSPIQEESIPIALSGRDILARAKNGTGKSGAYLIPLLERIDLKKD

YIQAVVMVPTRELALQVSQISIQISKHLGGVKVMATTGGTNLRDDILRLDETVHVVIATPGRILDLIKKGVAKVDRVQMM

VMDEADKLLSQDFVVLIEDIISFLAKGRQILLYSATFPISVQKFMSKHLQKPYEINLMEELTLKGITQFYAYVTERQKVH

CLNTLFSRLQINQSIIFCNSTQRVELLAKKITQLGYSCFYIHAKMMQEYRNRVFHDFRNGLCRNLVCTDLFTRGIDIQAV

NVVINFDFPKNAETYLHRIGRSGRFGHLGLAINLITSDDRFNLKSIEEQLVTDIKPIPGSIDKSLYVAEYHSVSPDGEEE

EPPRRPVTTPNQP

>XP_010892439.1 probable ATP-dependent RNA helicase ddx6 [Esox lucius]

MATAKTENPMSIMGLNKTNGQLRGPPKPVGLMSGPLATAPQPQASQKGGSIPQGSGGIKFGDDWKKSLQLPAKDCRVRTS

DVTSTKGNEFEDYCLKRELLMGIFEMGWEKPSPIQEESIPIALSGRDILARAKNGTGKSGAYLIPLLERIDLKKDHIQAL

VMVPTRELALQVSQISIQLSKHLGGVKVMATTGGTNLRDDIMRLDETVHVVIATPGRILDLIKKGVAKVDKVQMMVMDEA

DKLLSQDFVVLIEDIISFLAKGRQILLYSATFPISVQKFMAKHLSKPYEINLMDELTLKGITQYYAYVTERQKVHCLNTL

FSRLQINQSIIFCNSTQRVELLAKKITQLGYSCFYIHAKMMQEYRNRVFHDFRNGLCRNLVCTDLFTRGIDIQAVNVVIN

FDFPKNAETYLHRIGRSGRFGHLGLAINLITSEDRFNLKSIEDQLVTDIKPIPGSIDKSLYVAEFHSADPDAEDKEMSGK

HCELSAA

>XP_016356233.1 PREDICTED: probable ATP-dependent RNA helicase ddx6 [Sinocyclocheilus anshuiensis]

MATARTEIPASVMPMSKQNGQSKPMNLQSGSLASSLQSGKTPVMSQKGCNIPQSSGGIRFGDDWKKCLQLPPKDMRAKTT

DVTATKGNEFEDYCLKRELLMGIFEMGWEKPSPIQEESIPIVLSGRDILARAKNGTGKSGAYLIPLLERIDMKKDYVQAI

VLVPTRELALQMSQISINMSKHLGGVKVMATTGGTNLRDDIMRLDETVHVIIATPGRILDLMKKGVAKVDKAQMIVMDEA

DKLLSQDFVVLIEDIISFLPKNRQVLLYSATFPTSVQKFMTKHLQKPYEINLMDELTLKGITQYYAYVTERQKVHCLNTL

FSRLQINQSIIFCNSTQRVELLAKKITQLGYSCFYIHAKMMQEYRNRVFHDFRNGLCRNLVCTDLFTRGIDIQAVNVVIN

FDFPKNAETYLHRIGRSGRYGHLGLAINLITSEDRFNLKGIEDQLMTDIKPIPSSIDKSLYVAEFHSMNPDEEEAAHKAG

ELN

>XP_016376463.1 PREDICTED: probable ATP-dependent RNA helicase ddx6 [Sinocyclocheilus rhinocerous]

MATARTEIPTSVMPMSKQNGQSKPMNLQSGLLTSSLQSGKTPVMSQKGCNIPQSSGGIRFGDDWKKCLQLPPKDMRAKTT

DVTATKGNEFEDYCLKRELLMGIFEMGWEKPSPIQEESIPIVLSGRDILARAKNGTGKSGAYLIPLLERIDMKKDYVQAI

VLVPTRELALQMSQISINMSKHLGGVKVMATTGGTNLRDDIMRLDETVHVIIATPGRILDLMKKGVAKVDKAQMIVMDEA

DKLLSQDFVVLIEDIISFLPKNRQVLLYSATFPTSVQKFMTKHLQKPYEINLMDELTLKGITQYYAYVTERQKVHCLNTL

FSRLQINQSIIFCNSTQRVELLAKKITQLGYSCFYIHAKMMQEYRNRVFHDFRNGLCRNLVCTDLFTRGIDIQAVNVVIN

FDFPKNAETYLHRIGRSGRYGHLGLAINLITSEDRFNLKGIEDQLMTDIKPIPSSIDKSLYVAEFHSMNPDEEEAAHKAG

ELN

>RXN28671.1 putative ATP-dependent RNA helicase DDX6 [Labeo rohita]

MSTKMENTGPALMGLNTHNRQLIGQPKAPSQLGPQTVSAQPGKEPMGIQKPANASQEGPSIRFGDDWKKSLQLPAKDNRV

KTSDVTATKGNEFEDYCLKRELLMGIFEMGWEKPSPIQEESIPIALSGRDILARAKNGTGKSGAYLIPLLERIDIKKDHI

QAMVIVPTRELALQVSQISIQISKHLGGVKVMATTGGTNLRDDIMRLDETVHVVIATPGRILDLIKKGVAKVDKIHMMVM

DEADKLLSQDFVIIIEDIIGFLAKNRQILLYSATFPISVQKFMAKHLQKPYEINLMEELTLKGITQFYAYVTERQKVHCL

NTLFSRLQINQSIIFCNSTQRVELLAKKITQLGYSCFYIHAKMMQEYRNRVFHDFRNGLCRNLVCTDLFTRGIDIQAVNV

VINFDFPKNAETYLHRIGRSGRFGHLGLAINLITAEDRFNLKTIEDQLMTDIKPIPGSIDKSLYVAEFHSMNPDCEAEAS

QPDGEFEAL

>KAF4103185.1 hypothetical protein G5714_016068 [Onychostoma macrolepis]

MATARTEIPASVMPMSKQNGQSKPMNLQSGSLTSSSQSGKTPLMPQKGSSIPQSSGGIRFGDDWKKCLQLPPKDMRVKTT

DVTATKGNEFEDYCLKRELLMGIFEMGWEKPSPIQEESIPIVLSGRDILARAKNGTGKSGAYLIPLLERIDLKKDYVQAI

VLVPTRELALQVSQISINMSKHLGGVKVMATTGGTNLRDDIMRLDETVHAIIATPGRILDLMKKGVAKVDKAQMIVMDEA

DKLLSQDFVVLIEDIISFLPKNRQILLYSATFPTSVQKFMSKHLQKPYEINLMDELTLKGITQYYAYVTERQKVHCLNTL

FSRLQINQSIIFCNSTQRVELLAKKITQLGYSCFYIHAKMMQEYRNRVFHDFRNGLCRNLVCTDLFTRGIDIQAVNVVIN

FDFPKNAETYLHRIGRSGRYGHLGLAINLITSEDRFNLKGIEDQLMTDIKPIPSSIDKSLYVAEFHSMNPDEEEATHKAG

ELN

>XP_019959974.1 PREDICTED: probable ATP-dependent RNA helicase ddx6 [Paralichthys olivaceus]

MATARTENLGTVVMGLNKQNGQLRGQTKSASVQPAPTTQGKSMGALQKAGCPPQEGGGIKFGDDWKKSLKLPPKDTRVRT

SDVTSTKGNEFEDYCLKRELLMGIFEMGWEKPSPIQEESIPIALSGRDILARAKNGTGKSGAYLIPMLERIDLKKDYIQA

MVMVPTRELALQVSQISIQISKHLGGVKVMATTGGTNLRDDILRLDEIVHVVIATPGRILDLIKKGVAKVDRVQMMVMDE

ADKLLSQDFVVLIEDIISFLAKNRQILLYSATFPISVQKFMAKHLQKPYEINLMEELTLKGITQFYAYVTERQKVHCLNT

LFSRLQINQSIIFCNSTKRVELLAKKITQLGYSCFYIHARMMQEYRNRVFHDFRNGLCRNLVCTDLFTRGIDIQAVNVVI

NFDFPKNAETYLHRIGRSGRFGHLGLAINLITSEDRVNLKAIEEQLVTDIKPIPSSIDKSLYVAEYHSSSGDCDVEEIEE

KPGLQEDSI

>TSK34890.1 putative ATP-dependent RNA helicase DDX6 [Bagarius yarrelli]

MATARTQILTSVMGVTKQNGQARTAALQSGSQPATVLRASGKGSSVPQTSGGIRFGDDWKKSLQLPQKDMRVKTSDVTAT

KGNEFEDYCLKRELLMGIFEMGWEKPSPIQEESIPIALSGRDILARAKNGTGKSGAYLIPLLERIDLKKDHIQAIVLVPT

RELALQVSQICINMSRHMGGVKVMATTGGTNLKDDIMRLDETVHVVIATPGRLLDLMKKGLAKTDKVHMLVMDEADKLLS

QDFVILVEEIIGFLDKNRQILLYSATFPITVQKFMSKHLKKPYEINLMDELTLKGITQYYAYVTERQKVHCLNTLFSRLQ

INQSIIFCNSTQRVELLAKKITQLGYSCFYIHAKMMQEYRNRVFHDFRNGLCRNLVCTDLFTRGIDIQAVNVVINFDFPK

NAETYLHRIGRSGRFGHLGLAINLITSDDRFNLKAIEDQLVTDIKPIPGSIDKTLYVAEFHSINNEEEEDQESELGAP

>XP_001340860.1 probable ATP-dependent RNA helicase ddx6 [Danio rerio]

MATARTEIPASVMPVTKQNGQAKPMSLQTGSFSSSTPSGKTPVMPQKGSSIPQSSGGIRFGDDWKKCLQLPPKDTRVRTT

DVTATKGNEFEDYCLKRELLMGIFEMGWEKPSPIQEESIPIALSGRDILARAKNGTGKSGAYLIPLLERIDLKKDYIQAI

VLVPTRELALQVSQISINMSKHLGGIKVMATTGGTNLRDDIMRLDEIVHVIIATPGRILDLIKKGVAKVDKVQMAVMDEA

DKLLSQDFVVLIEDIISFLPKKRQILLYSATFPISVQKFMTKHLQKPYEINLMDELTLKGITQYYAYVTERQKVHCLNTL

FSRLQINQSIIFCNSTQRVELLAKKITQLGYSCFYIHAKMMQEYRNRVFHDFRNGLCRNLVCTDLFTRGIDIQAVNVVIN

FDFPKNAETYLHRIGRSGRYGHLGLAINLITSEDRFNLKGIEDQLMTDIKPIPSSIDKSLYVAEFHSMNPDEEEAAHKTG

ELN

>XP_019721598.1 PREDICTED: probable ATP-dependent RNA helicase ddx6 [Hippocampus comes]

MATARTENVDPPAMGLNKQNGQLRGQTKPASVQPGSSTQPKVLGATQKAGTASQARGTIKFGDDWKKSLQLPPKDTRVKT

SDVTATKGNEFEDYCLKRELLMGIFEMGWEKPSPVQEESIPIALSGRDILARAKNGTGKSGAYLIPLLERIDLKKDYIQA

MVVVPTRELALQVSQICIQISKHLGGVKVMATTGGTNLRDDIMRLDEIVHVIVATPGRILDLIQKGVARVDRVQMMVMDE

ADKLLSQDFVVLIEDIISFLAKNRQILLYSATFPISVQKFMTNYLQKPYEINLMEELTLKGITQYYAYVTERQKVHCLNT

LFSRLQINQSIIFCNSTQRVELLAKKITQLGYSCFYIHAKMMQEYRNRVFHDFRNGLCRNLVCTDLFTRGIDIQAVNVVI

NFDFPKNAETYLHRIGRSGRFGHFGLAINLITSEDRFNLKTIEDQLVTDIKPIPGSIDKGLYVAEFHSSAGDCEVEEVQE

RSGHQQDHT

>XP_034467324.1 probable ATP-dependent RNA helicase DDX6 [Hippoglossus hippoglossus]

MATARTANPATMMGLNKAANGQFRGQTRPPGQQSGLLAAAQQSSDPMKRTGIPQSSGGIKFGDDWKRCLELPPKDTRLRT

SDVTSTKGNEFEDYCLKRELLMGIFEMGWEKPSPIQEESIPIALSGRDILARAKNGTGKSGAYLIPMLERIDLRKDHIQA

MVMVPTRELALQMSQISIQLSKHLGGVKVMATTGGTNLRDDIMRLEETVHVVIATPGRILDLIKKGVAVVDKLQMMVMDE

ADKLLSQDFVVLIEDIISFLPKNRQILLYSATFPVSVQTFMNKHLQKPYEINLMEELTLKGITQYYAYVTERQKVHCLNT

LFSKLQINQSIIFCNSVQRVELLAKKITQLGYSCFYIHAKMMQEYRNRVFHDFRNGLCRNLVCTDLFTRGIDIQAVNVVI

NFDFPKNAETYLHRIGRSGRFGHLGLAINLITIDDRNNLKTIEDQLVTDIKPIPSCIDKSLYVAEFHSVDPDDDGDGKGK

NNELG

>KAF1388180.1 hypothetical protein PFLUV_G00087530 [Perca fluviatilis]

MATARTANPAQMIGLNKPANGQLRGQAALLAAAQQSSALQKRTSIPQSSGGIKFGDDWKKCLELPPKDTRMRTADVTSTK

GNEFEDYCLKRELLMGIFEMGWEKPSPVQEESIPIALSGRDILARAKNGTGKSGAYLIPLLERIDLKKDHIQAIVMVPTR

ELALQVSQISIQLSKHLGGVKVMATTGGTNLRDDIMRLDETVHVVIATPGRILDLIKKGVAKMDKAQLIVMDEADKLLSQ

DFVVLIEDIISFMPKDRQILLYSATFPISVQKFMNKHLKKPYEINLMEELTLKGITQYYAYVTERQKVHCLNTLFSRLQI

NQSIIFCNSTQRVELLAKKITQLGYSCFYIHAKMMQEYRNRVFHDFRNGLCRNLVCTDLFTRGIDIQAVNVVINFDFPKN

AETYLHRIGRSGRFGHLGLAINLITSEDRYNLKNIEDQLVTDIKPIPSSIDKSLQKQGTGRNLSDWRNAAPGIHDTILAM

HPTRFQVMISVPYQSVDELRKIGGHLSTTDVVVNFAGVELPLDPYCQLCYSLSMGSVLAAAPPSPAPAAAGGSQGVPGLV

SVPPGFTMPSVSSVPPTSGSGQQSADTDSPLPNPGTYEECHRKCKEVFPLQMEGVRLVVNKGLSNHFQVSHTVTLSTLGD

SGYRFGSTYVGSKQTGPAESFPVMVGDMDNTGSLNAQIIHQLTSAVRSKIAIQTQQHKFVNWQCEMECRGEDFTAAVTLG

NPDVLVGSGILLGHYLQSITPALALGGELVYHRRPGEEGAVTSLLGRYTGDNYVATLTLGGAGAHATYYHKASDQLQVGV

EFEASTRMQDTTASFGYQLDVPKANFLFKGTVDSNWVVGATLEKKLVPLPLTLALGAFLNHRKNKFQCGFGVTIG

>XP_026782633.1 probable ATP-dependent RNA helicase DDX6 [Pangasianodon hypophthalmus]

MATARTQILASVMGVTKQNGQPRPADLQSGSQPATVLSASQKGSSIPHTSGGIRFGDDWKKSLQLPQKDMRVKTSDVTAT

KGNEFEDYCLKRELLMGIFEMGWEKPSPIQEESIPIALSGRDILARAKNGTGKSGAYLIPLLERIDLKKGHIQAIVMVPT

RELALQVSQICINMSRHMGGVKVMATTGGTNLKDDIMRLDETVHVVIATPGRLLDLMKKGVAKADKVHMMVMDEADKLLS

QDFVILIEDIIGFLDKNRQILLYSATFPITVQKFMSKHLRKPYEINLMDELTLKGITQYYAYVTERQKVHCLNTLFSRLQ

INQSIIFCNSTQRVELLAKKITQLGYSCFYIHAKMMQEYRNRVFHDFRNGLCRNLVCTDLFTRGIDIQAVNVVINFDFPK

NAETYLHRIGRSGRFGHLGLAINLITSDDRFNLKAIEDQLVTDIKPIPGSIDKSLYVAEFHSINHEEEEEKEAALSAS

>OBS58487.1 hypothetical protein A6R68_10352, partial [Neotoma lepida]

PGDDWKKTLKLPPKDLRIKTSDVTSTKGNEFEDYCLKRELLMGIFEMGWEKPSPIQEESIPIALSGRDILARAKNGTGKS

GAYLIPLLERLDLKKDNIQAMVIVPTRELALQVSQICIQVSKHMGGAKVMATTGGTNLRDDIMRLDDTVHVVIATPGRIL

DLIKKGVAKVDHVQMIVLDEADKLLSQDFVQIMEDIILTLPKNRQILLYSATFPLSVQKFMNSHLQKPYEINLMEELTLK

GVTQYYAYVTERQKVHCLNTLFSRLQINQSIIFCNSSQRVELLAKKISQLGYSCFYIHAKMRQEHRNRVFHDFRNGLCRN

LVCTDLFTRGIDIQAVNVVINFDFPKLAETYLHRIGRSAINLITYDDRFNLKSIEEQLGTEIKPIPSNIDKSLYVAEYHS

EPGWEGRVLCPKRVSASAKRGLSMA

>NXS80452.1 DDX6 helicase [Erpornis zantholeuca]

MSTARTENPVIMGLSSQNGQLRGPVKPSGGPGGGGTQTQQQMNQLKNANTINNGTQQQAQSMTTAIKPGDDWKKTLKLPP

KDLRIKTSDVTSTKGNEFEDYCLKRELLMGIFEMGWEKPSPIQEESIPIALSGRDILARAKNGTGKSGAYLIPLLERLDL

KKDNIQGQLRWISLSCSQVMVSVAIQICIQVSKHMGGAKVMATTGGTNLRDDIMRLDDTVHVVIATPGRILDLIKKGVAK

VEHVQMIVLDEANKLLSQDFVQIMEDIILTLPKNRQILLYSATFPLSVQKFMNSHLQKPYEINLMEELTLKGVTQYYAYV

TERQKVHCLNTLFSRLQINQSIIFCNSSQRVELLAKKISQLGYSCFYIHAKMRQEHRNRVFHDFRNGLCRNLVCTDLFTR

GIDIQAVNVVINFDFPKLAETYLHRIGRSGRFGHLGLAINLITYDDRFNLKSIEEQLGTEIKPIPSNIDKSLYVAEYHSE

PVEDEKQ

>TDH11563.1 hypothetical protein EPR50_G00062100 [Perca flavescens]

MATARTANPAQMIGLNKPANGQLRGQAALLAAAQQSSALQKRTSIPQSSGGIKFGDDWKKCLELPPKDTRMRTADVTSTK

GNEFEDYCLKRELLMGIFEMGWEKPSPVQEESIPIALSGRDILARAKNGTGKSGAYLIPLLERIDLKKDHIQAIVMVPTR

ELALQVSQISIQLSKHLGGVKVMATTGGTNLRDDIMRLDETVHVVIATPGRILDLIKKGVAKMDKAQLIVMDEADKLLSQ

DFVVLIEDIISFMPKDRQILLYSATFPISVQKFMNKHLKKPYEINLMEELTLKGITQYYAYVTERQKVHCLNTLFSRLQI

NQSIIFCNSTQRVELLAKKITQLGYSCFYIHAKMMQEYRNRVFHDFRNGLCRNLVCTDLFTRGIDIQAVNVVINFDFPKN

AETYLHRIGRSGRFGHLGLAINLITSEDRYNLKNIEDQLVTDIKPIPSSIDKSLQKQGTGRNLSEWRNAAPGIHDTILAM

HPTRFQVMISVP

>XP_027005728.1 LOW QUALITY PROTEIN: probable ATP-dependent RNA helicase ddx6 [Tachysurus fulvidraco]

MATARTQIPTSVMGVTKQNGQAGTQTATVLSASQKGSSIPHTSGGIRFGDDWKKSLQLPEKDTRVKTSDVTATKGNEFED

YCLKRELLMGIFEMGWEKPSPIQEESIPIALSGRDILARAKNGTGKSGAYLIPLLERIDLKKDHIQAIVMVPTRELALQV

SQICINMSRHMGGVKVMATTGGTNLKDDIMRLDEMVHVVIATPGRLLDLMKKGVAKTGKVQMMVMDEADKLLSQDFVTLI

EEIIGFTDKNRQILLYSATFPTTVQKFMSKHLKKPYEINLMDELTLKGITQYYAYVTERQKVHCLNTLFSRLQINQSIIF

CNSTQRVELLAKKITQLGYSCFYIHAKMMQEYRNRVFHDFRNGLCRNLVCTDLFTRGIDIQAVNVVINFDFPKNAETYLH

RIGRSGRFGHLGLAINLITSEDRFNLKAIEDQLVTDIKPIPGSIDKSLYVAEFHSINHEEEEEKDAALSAS

>XP_035493406.1 probable ATP-dependent RNA helicase ddx6 [Scophthalmus maximus]

MATARTANPAPMIPLNKAANGQFRGQTRPAVPQPGLLSTSQQSCAPQKRSSVPQNSGGIKFGDDWKKCLELPAKDTRMKT

SDVTSTKGNEFEDYCLKRELLMGIFEMGWEKPSPIQEESIPIALSGRDILARAKNGTGKSGAYLIPMLERIDLKKDHIQA

MVMVPTRELALQMSQISIQLSKHLGGVKVMATTGGTNLRDDIMRLEEIVHVVIATPGRILDLIKKGVAVVDKTHIMVMDE

ADKLLSQDFIVLIEDIISFLPKHRQILLYSATFPISVQKFMNKHLQKPYEINLMEELTLKGITQYYAYVNERQKVHCLNT

LFSKLQINQSIIFCNSTQRVELLAKKITQLGYSCFYIHAKMMQEYRNRVFHDFRNGLCRNLVCTDLFTRGIDIQAVNVVI

NFDFPKNAETYLHRIGRSGRFGHLGLAINLITSEDRYNLKTIEDQLVTDIKPIPSSIDKSLYVAEFHSVDPDDDDGDGRA

KNEELGAA

>XP_019948818.1 PREDICTED: probable ATP-dependent RNA helicase DDX6 [Paralichthys olivaceus]

MATARTANPGTMIGLNKAANGQFRGQTRPAGQQSGLLAAAQQSSDPMKRSSFPQSSGGIKFGDDWKRCLELPPKDTRLRT

SDVTSTKGNEFEDYCLKRELLMGIFEMGWEKPSPIQEESIPIALSGRDILARAKNGTGKSGAYLIPMLERIDLRKDHIQA

MVMVPTRELALQMSQISIQLSKHLGGVKVMATTGGTNLRDDIMRLEETVHVVIATPGRILDLIKKGVAIVDKTQMMVMDE

ADKLLSQDFVVLIEDIISFLPKNRQILLYSATFPVSVQTFMNKHLQKPYEINLMEELTLKGITQYYAYVTERQKVHCLNT

LFSKLQINQSIIFCNSVQRVELLAKKITQLGYSCFYIHAKMMQEYRNRVFHDFRNGLCRNLVCTDLFTRGIDIQAVNVVI

NFDFPKNAETYLHRIGRSGRFGHLGLAINLITIDDRNNLKTIEDQLVTDIKPIPSCIDKSLYVAEFHSVDPDDEGDGKAK

NNELGEA

>XP_017555820.1 PREDICTED: probable ATP-dependent RNA helicase DDX6 [Pygocentrus nattereri]

MATARTEIPASVMRLTKQNGQPKPMGLQSGSVTGLTQPGKALGAPPKGSSILQTSGGIKFGDDWKKCLQLPPKDLRVKTS

DVTATKGNEFEDYCLKRELLMGIFEMGWEKPSPIQEESIPIALSGRDILARAKNGTGKSGAYLIPLLERIDLKKDHIQAI

VMVPTRELALQVSQISINMSKHLGGVKVMATTGGTNLKDDIMRLDDTVHVVIATPGRIVDLIKKGVAKVGKVQMMVMDEA

DKLLSQDFVMLIEEIISFLDKNRQILLYSATFPISVQKFMAKHLRKPYEINLMDELTLKGITQYYAYVTERQKVHCLNTL

FSRLQINQSIIFCNSTQRVELLAKKITQLGYSCFYIHAKMMQEYRNRVFHDFRNGLCRNLVCTDLFTRGIDIQAVNVVIN

FDFPKNAETYLHRIGRSGRYGHLGLAINLITSDDRFSLKAIEEQLVTDIKPIPGSIDKSLYVAEFHHANHDEEEEREAVL

>NXS70969.1 DDX6 helicase [Pandion haliaetus]

DVTSTKGNEFEDYCLKRELLMGIFEMGWEKPSPIQEESIPIALSGRDILARAKNGTGKSGAYLIPLLERLDLKKDNIQAM

VIVPTRELALQVSQICIQVSKHMGGAKVMATTGGTNLRDDIMRLDDTVHVVIATPGRILDLIKKGVAKVEHVQMIVLDEA

NKLLSQDFVQIMEDIILTLPKNRQILLYSATFPLSVQKFMNSHLQKPYEINLMEELTLKGVTQYYAYVTERQKVHCLNTL

FSRLQINQSIIFCNSSQRVELLAKKISQLGYSCFYIHAKMRQEHRNRVFHDFRNGLCRNLVCTDLFTRGIDIQAVNVVIN

FDFPKLAETYLHRIGRSGRFGHLGLAINLITYDDRFNLKSIEEQLGTEIKPIPSNIDKSLYVAEYHSEPVEDEKQ

>KAF5899384.1 putative ATP-dependent RNA helicase DDX6 [Clarias magur]

MATARTQIPTTVMGVSKQNGQARSADLQSGSQPSTVLNVSQKGSSIPHTSGGIRFGDDWKKSLQLPQKDMRVKTSDVTAT

KGNEFEDYCLKRELLMGIFEMGWEKPSPIQEESIPIALSGRDILARAKNGTGKSGAYLIPLLERIDLKKDHIQAIVMVPT

RELALQVSQICITMSRHMGGVKVMATTGGTNLKDDIMRLDETVHVVIATPGRLLDLMKKGLAKADKVHMMVMDEADKLLS

QDFVILIEEIIGFLDRNRQILLYSATFPITVQKFMSKHLKKPYEINLMDELTLKGITQYYAYVTERQKVHCLNTLFSRLQ

INQSIIFCNSTQRVELLAKKITQLGYSCFYIHAKMMQEYRNRVFHDFRNGLCRNLVCTDLFTRGIDIQAVNVVINFDFPR

NAETYLHRIGRSGRFGHLGLAINLITSDDRFNLKAIEDQLVTEIKPIPGSIDKSLYVAEFHAINQEEEEEKEAALDSPLE

NRWMVPGILHIIC

>XP_016141620.1 PREDICTED: probable ATP-dependent RNA helicase ddx6 [Sinocyclocheilus grahami]

MAMARTEIPASVMPMSKQNGQSKPMNLQPGSLTSSLQSGKTPVMSQKGCNIPQSSGGIRFGDDWKKCLHLPPKDMRAKTT

DVTATKGNEFEDYCLKRELLMGIFEMGWEKPSPIQEESIPIVLSGRDILARAKNGTGKSGAYLIPLLERIDMKKDYVQAI

VLVPTRELALQMSQISINMSKHLGGVKVMATTGGTNLRDDIMRLDETVHVIIATPGRILDLMKKGVAKVDKAQMIVMDEA

DKLLSQDFVVLIEDIISFLPKNRQVLLYSATFPTSVQKFMTKHLQKPYEINLMDELTLKGITQYYAYVTERQKVHCLNTL

FSRLQINQSIIFCNSTQRVELLAKKITQLGYSCFYIHAKMMQEYRNRVFHDFRNGLCRNLVCTDLFTRGIDIQAVNVVIN

FDFPKNAETYLHRIGRSGRYGHLGLAINLITSEDRFNLKGIEDQLMTDIKPIPSSIDKSLYVAEFHSMNPDEEEAAHKAG

ELN

>XP_019751662.1 PREDICTED: probable ATP-dependent RNA helicase DDX6 [Hippocampus comes]

MAAARTANPAQMIGLMKTANRQIKGGKPAGFGGQQPQKRSSVPQSSGGIKFGDDWKKCLELPPKDNRIRTSDVTSTKGNE

FEDYCLKRELLMGIFEMGWEKPSPIQEESIPVALSGRDILARAKNGTGKSGAYLIPLLERIDLTKGHIQALVIVPTRELA

LQMSQISIQLSKHLGGVKVMATTGGTNLRDDILRLDETVHVVIATPGRILDLIKKGLAKVEKIQMMVMDEADKLLSQDFV

AIIEDIIRFLPKNRQILLYSATFPISVQTFMNKHLQKPYEINLMEELTLKGITQYYAYVTERQKVHCLNTLFSRLQINQS

IIFCNSTQRVELLAKKITQLGYSCFYIHAKMMQEYRNRVFHDFRNGLCRNLVCTDLFTRGIDIQAVNVVINFDFPKSAET

YLHRIGRSGRFGHLGLAINLITSEDRHNLKSVEDQLVTDIKPIPGNIDKSLYVAEFHSVEHDDPDGGPTNAELEAA

>KAA0703286.1 putative ATP-dependent RNA helicase ddx6 [Triplophysa tibetana]

MKETDTMRNVLLGLCPYIGKIPASVMAMTKQNGQSKPMTLQPGSFTSSTQSGKAPVVPPKGSSIPQISGGIRFGDDWKKC

LLLPPKDTRVRTTDVTATKGNEFEDYCLKRELLMGIFEMGWEKPSPIQEESIPIALSGRDILARAKNGTGKSGAYLIPLL

ERIDLKKDYVQAIVLVPTRELALQVSQISINMSKHLGGVKVMATTGGTNLRDDIMRLDEIVHVIIATPGRILDLLRKGVA

KVDKAQMIVMDEADKLLSQDFVVLVEDIISFLPKNRQVLLYSATFPISVQKFMTKHLQKPYEINLMDELTLKGITQYYAY

VTERQKVHCLNTLFSRLQINQSIIFCNSTQRVELLAKKITQLGYSCFYIHAKMMQEYRNRVFHDFRNGLCRNLVCTDLFT

RGIDIQAVNVVINFDFPKNAETYLHRIGRSGRYGHLGLAINLITAEDRFNLKGIEDQLITDIKPIPSSIDKSLYVAEERS

RRTLEVQL

>KAF0045581.1 hypothetical protein F2P81_002110 [Scophthalmus maximus]

MIPLNKAANGQFRGQTRPAVPQPGLLSTSQQSCAPQKRSSVPQNSGGIKFGDDWKKCLELPAKDTRMKTSDVTSTKGNEF

EDYCLKRELLMGIFEMGWEKPSPIQEESIPIALSGRDILARAKNGTGKSGAYLIPMLERIDLKKDHIQAMVMVPTRELAL

QMSQISIQLSKHLGGVKVMATTGGTNLRDDIMRLEEIVHVVIATPGRILDLIKKGVAVVDKTHIMVMDEADKLLSQDFIV

LIEDIISFLPKHRQILLYSATFPISVQKFMNKHLQKPYEINLMEELTLKGITQYYAYVNERQKVHCLNTLFSKLQINQSI

IFCNSTQRVELLAKKITQLGYSCFYIHAKMMQEYRNRVFHDFRNGLCRNLVCTDLFTRGIDIQAVNVVINFDFPKNAETY

LHRIGRSGRFGHLGLAINLITSEDRYNLKTIEDQLVTDIKPIPSSIDKSLYVAEFHSVDPDDDDGDGRAKNEELGAA

>KAF7708677.1 hypothetical protein HF521_017734 [Silurus meridionalis]

MATARTQIPASVIGITKQNGQTRPAGLRSGLQPEMVFSASQKESSIPHTSGGIRFGDDWKKALHLPQKDTRVRTSDVTAT

KGNEFEDYCLKRELLMGIFEMGWEKPSPIQEESIPIALSGRDILARAKNGTGKSGAYLIPLLERIDLKKDHIQAIVMVPT

RELALQVSQICINMSRHMGGVKVMATTGGTNLKDDIMRLDETVHVVIATPGRLLDLMKKGVAKADKVHMMVMDEADKLLS

QDFVVIIEEIIGFLDKNRQILLYSATFPITVQKFMSKHLKKPYEINLMDELTLKGITQYYAYVTERQKVHCLNTLFSRLQ

INQSIIFCNSTQRVELLAKKITQLGYSCFYIHAKMMQEYRNRVFHDFRNGLCRNLVCTDLFTRGIDIQAVNVVINFDFPK

NAETYLHRIGRSGRFGHLGLAINLITSDDRFNLKAIEDQLVTDIKPIPGSIDKSLYVAEFHSINHEEEDEKEALFGAP

>XP_030013410.1 probable ATP-dependent RNA helicase ddx6 [Sphaeramia orbicularis]

MATARTASPATMIGVNKPTNGHFKGQTKPTGQQSGLHGTSQLPSAPIIRSSIPQSSGGIKFGDDWKKCLELPQKDNRVKT

SDVTSTKGNEFEDYCLKRELLMGIFEMGWEKPSPIQEESIPIALSGRDILARAKNGTGKSGAYLIPLLERIDLKKDHIQA

IVMVPTRELALQVSQISIQISKHLGGVKVMATTGGTNLRDDIMRLDEIVHVVIATPGRILDLIKKGVAKVNKAQIMVMDE

ADKLLSQDFVILIEDIISFLPKDRQILLYSATFPISVQKFMNKHLKKPYEINLMEELTLKGITQYYAYVTEKQKVHCLNT

LFSRLQINQSIIFCNSTQRVELLAKKITQLGYSCFYIHAKMMQEYRNRVFHDFRNGLCRNLVCTDLFTRGIDIQAVNVVI

NFDFPKSAETYLHRIGRSGRFGHLGLAINLITSDDRYNLKSIEDQLVTDIKPIPGSIDKSLYVAEYHSVDPDDDDGSDGG

LKNKEQGAA

>XP_028819413.1 probable ATP-dependent RNA helicase ddx6 [Denticeps clupeoides]

MAAARTENPGPVMMNLTKSNGQLRGPPKAASLSGSSAASVPTGRAPGLKPSGGTQEGPGIKFGDDWKKSLHLPPKDNRIK

TSDVTATKGNEFEDYCLKRELLMGIFEMGWEKPSPIQEESIPIALSGRDILARAKNGTGKSGAYLIPLLERIDLKKDYIQ

AVVMVPTRELALQVSQISIQISKHLGGVKVMATTGGTNLRDDIMRLDETVHVVIATPGRVLDLIKKGVAKVDKVQTMVMD

EADKLLSQDFMVLIGDIISFLPKNRQILLYSATFPISVQKFMAKYLQKPYEINLMEELTLKGITQYYAYVTERQKVHCLN

TLFSRLQINQSIIFCNSTQRVELLAKKITQLGYSCFYIHAKMMQEYRNRVFHDFRNGLCRNLVCTDLFTRGIDIQAVNVV

INFDFPKNAETYLHRIGRSGRFGHFGLAINLITSDDRFNLKAIEDQLVTDIKPIPGTIDKSLYVAEFHCSKPDCEEDEEK

EDFRRLPINP

>XP_026145863.1 probable ATP-dependent RNA helicase DDX6 [Carassius auratus]

MTTKMENSGPGVMGLSTHNRQHLGQPKASSQLGPQTASAQPGKEPMVTQKPANASQEGPGIRFGDDWKKSLQLPSKDNRV

KTSDVTATKGNEFEDYCLKRELLMGIFEMGWEKPSPIQEESIPIALSGRDILARAKNGTGKSGAYLIPLLERIDIKKDHI

QAIVIVPTRELALQVSQISIQISKHLGGVNVMATTGGTNLRDDIMRLDEKVHVVVATPGRILDLMKKGIAKVDKVQMMVM

DEADKLLSQDFVVIIEDIIGFLSKNRQILLYSATFPISVQKFMAKHLQKPYEINLMEELTLKGITQYYAYVTERQKVHCL

NTLFSRLQINQSIIFCNSTQRVELLAKKITQLGYSCFYIHAKMMQEYRNRVFHDFRNGLCRNLVCTDLFTRGIDIQAVNV

VINFDFPKNAETYLHRIGRSGRFGHLGLAINLISAEDRFNLKTIEDQLITDIKPIPGSIDKSLYVAEFHSVDPDCEAEEP

QRGEGSEAH

>XP_016387513.1 PREDICTED: probable ATP-dependent RNA helicase ddx6 [Sinocyclocheilus rhinocerous]

MATARTEIPSSVMPMSKQNGQTKPLTLQSGSLTSSTQSGKTPAMPQKGSSIHQSSGGIRFGDDWKKCLQLPPKDLRVKTT

DVTATKGNEFEDYCLKRELLMGIFEMGWEKPSPIQEESIPIVLSGRDILARAKNGTGKSGAYLIPLLERIDLKKDYVQAI

VFVPTRELALQVSQISINMSKHLGGVKIMATTGGTNLRDDIMRLDETVHVIIATPGRILDLIKKGVAKVDKAQMIVMDEA

DKLLSQDFVVLIEDIISFMPKKRQILLYSATFPSSVQKFMSKHLQKPYEINLMDELTLKGITQYYAYVTERQKVHCLNTL

FSRLQINQSIIFCNSTQRVELLAKKITQLGYSCFYIHAKMMQEYRNRVFHDFRNGLCRNLVCTDLFTRGIDIQAVNVVIN

FDFPRNAETYLHRIGRSGRYGHLGLAINLITSEDRFNLKGIEDQLMTDIKPIPSSIDKNLYVAEFHTMNPDEEEAAHKAG

ELN

>XP_026140736.1 probable ATP-dependent RNA helicase ddx6 [Carassius auratus]

MATARTEIPASVMPMSKQNGQSKPMNLQSGSLTSSQSGKTPGMSQKGSSIPQSSGGIRFGDDWKKCLQLPPKDMRMKTTD

VTATKGNEFEDYCLKRELLMGIFEMGWEKPSPIQEESIPIVLSGRDILARAKNGTGKSGAYLIPLLERIDLKKDYVQAIV

LVPTRELALQVSQISINMSKHLGGVKVMATTGGTSLRDDIMRLDETVHVIIATPGRILDLIKKGVAKVDKAQMIVMDEAD

KLLSQDFVVIIEDIISFLPKNRQVLLYSATFPISVQKFMSKHLQKPYEINLMDELTLKGITQYYAYVTERQKVHCLNTLF

SRLQINQSIIFCNSTQRVELLAKKITQLGYSCFYIHAKMMQEYRNRVFHDFRNGLCRNLVCTDLFTRGIDIQAVNVVINF

DFPKNAETYLHRIGRSGRYGHLGLAINLITSEDRFNLKGIEDQLMTDIKPIPSSIDKNLYVAEFHSMNPDEEEAAHKAGE

LN

>XP_026172870.1 probable ATP-dependent RNA helicase ddx6 [Mastacembelus armatus]

MATARTANPAPLLGLNKQTNGQLRGQSKPAGPQPGLLATAQQPNTPEKRSSIPQKSGGIKFGDNWKKCLELPPKDNRVKT

SDVTSTKGNEFEDYCLKRELLMGIFEMGWEKPSPIQEESIPIALSGRDILARAKNGTGKSGAYLIPLLERIDLKKDHIQA

IVMVPTRELALQMSQISIQLSKHLGGVKIMATTGGTSLRDDIMRLDETVHVVIGTPGRLLDLVKKGVAKVDKTQMMVMDE

ADKLLSQDFVVLIEDIISFLSKDRQILLYSATFPISVQKFMSDHLQKPYEINLMEELTLKGITQYYAYVTERQKVHCLNT

LFSRLQINQSIIFCNSTQRVELLAKKITQLGYSCFYIHAKMMQEYRNRVFHDFRNGLCRNLVCTDLFTRGIDIQAVNVVI

NFDFPKSAETYLHRIGRSGRFGHLGLAINLITSDDRYNLKTIEEQLVTDIKPIPSSIDKSLYVAEFHSVDPDDDSCDAGT

KKKELGAA

>XP_012694823.1 probable ATP-dependent RNA helicase ddx6 [Clupea harengus]

MATARTENPTSVMGLGKPNGQLRGPPKPAGFQSLPLSSATLQTKASSGSQVGSTVSQTSDGIRFGDDWKKCLQLPPKDCR

MRTSDVTSTKGNEFEDYCLKRELLMGIFEMGWEKPSPIQEESIPIALSGRDILARAKNGTGKSGAYLIPLLERIDLKKDH

IQAMVLVPTRELALQVSQISIQISKHMGGVKVMATTGGTNLRDDIMRLDETVHVVIATPGRILDLIKKGVAKVDRVQILV

MDEADKLLSQDFVVLIEEIISFTAKHRQILLYSATFPVTVQKFMSKHLQKPYEINLMDELTLKGITQYYAYVTERQKVHC

LNTLFSRLQINQSIIFCNSTQRVELLAKKITQLGYSCFYIHAKMMQEYRNRVFHDFRNGLCRNLVCTDLFTRGIDIQSVN

VVINFDFPKNAETYLHRIGRSGRYGHLGLAINLITSEDRFNLKGIEDQLVTDIKPIPGTIDKSLYVAEFHSLDPDAEEIE

REAPRKSGEPNAR

>XP_036070118.1 probable ATP-dependent RNA helicase ddx6 [Oryzias melastigma]

MATARTENVGPMVMGLNKQNGQLRGQNKPPAVQPAPQSQGKGLGALQKAGGAPQDGGGIKFGDDWKKSLLLPPKDNRVKT

SDVTATKGNEFEDYCLKRELLMGIFEMGWEKPSPIQEESIPIALSGRDILARAKNGTGKSGAYLIPLLERIDLKKDHIQG

ELXXXTRELALQVSQICIQISKHLGGVKVMATTGGTNLRDDIMRLDETVHVVIATPGRILDLIKKGVAKVDRVQMIVMDE

ADKLLSQDFVVLIEDIISFLAKNRQILLYSATFPISVQKFMAKHLQKPYEINLMEELTLKGITQFYAYVTERQKVHCLNT

LFSRLQINQSIIFCNSTQRVELLAKKITQLGYSCFYIHAKMMQEYRNRVFHDFRNGLCRNLVCTDLFTRGIDIQAVNVVI

NFDFPKNAETYLHRIGRSGRFGHLGLAINLITSEDRFNLKAIEEQLVTDIKPIPSSIDKSLYVAEYHSSAGDGDAEDKPE

RQQDST

>XP_034455309.1 probable ATP-dependent RNA helicase ddx6 [Hippoglossus hippoglossus]

MATARTENLGTVVMGLNKQNGQLRGQTKPASVQPAPTTLGKAMGALQKAGCPPQEGGGIKFGDDWKKSLKLPPRDTRVKT

SDVTSTKGNEFEDYCLKRELLMGIFEMGWEKPSPIQEESIPIALSGRDILARAKNGTGKSGAYLIPMLERIDLKKDYIQA

MVMVPTRELALQVSQISIQISKHLGGVKVMATTGGTNLRDDIMRLDEIVHVVIATPGRILDLIKKGVAKVDRVQMMVMDE

ADKLLSQDFVVLIEDIISFLAKNRQILLYSATFPISVQKFMAKHLQKPYEINLMEELTLKGITQFYAYVTERQKVHCLNT

LFSRLQINQSIIFCNSTKRVELLAKKITLLGYSCFYIHAKMMQEYRNRVFHDFRNGLCRNLVCTDLFTRGIDIQAVNVVI

NFDFPKSAETYLHRIGRSGRFGHLGLAINLITSEDRINLKATEEQLVTDIKPIPSSIDKSLYVAEYHSSSGDCDVEEIEE

KSGHPDDGI

>XP_026999655.1 probable ATP-dependent RNA helicase DDX6 [Tachysurus fulvidraco]

MATAKMENVGAVVMGLGKQNGQLRGLSSQSGTAIQSNPFARGPKPAADSQEGPGIRFGEDWKKSLQLPPKDNRVKTSDVT

ATKGNEFEDYCLKRELLMGIFEMGWEKPSPVQEESIPIALSGRDILARAKNGTGKSGAYLIPMLERIDLKKEYIQALVMV

PTRELALQVSQISIQLSKHLHGLKVMATTGGTNLRDDIMRLDEIVHVVIATPGRILDLIKKGVAKVDKVQIMVMDEADKL

LSQDFVVLIEDIISFLHKNRQILLYSATFPVTVQKFMAKHLKKPYEINLMEELTLKGITQYYAYVTERQKVHCLNTLFSR

LQINQSIIFCNSTQRVELLAKKITQLGYSCFYIHAKMMQEYRNRVFHDFRNGLCRNLVCTDLFTRGIDIQAVNVVINFDF

PRNSETYLHRIGRSGRFGHLGLAINLITAEDRFNLKAIEDQLVTDIKPIPGSIDKSLYVADFHTTNDEAEEQETAEEP

>XP_035286716.1 probable ATP-dependent RNA helicase ddx6 [Anguilla anguilla]

MATARTENLASVVVGLNGQMRGQPKPASPQPGPFALGSQPGKAPGAPPKDGIGAQDGGGIRFGDDWKKNLQLPPKDTRMR

TSDVTATKGNEFEDYCLKRELLMGIFEMGWEKPSPIQEESIPIALSGRDILARAKNGTGKSGAYLIPLLERIDLKKDFIQ

AMVMVPTRELALQVSQISIQVSKHLGGVKVMATTGGTNLRDDIMRLDETVHVVIATPGRILDLIKKGVAKVDRVQIMVMD

EADKLLSQDFVVLIEDIISFLAKGRQILLYSATFPISVQKFMAKHLQKPYEINLMDELTLKGITQYYAYVTERQKVHCLN

TLFSRLQINQSIIFCNSTQRVELLAKKITQLGYSCFYIHAKMMQEYRNRVFHDFRNGLCRNLVSTDLFTRGIDIQAVNVV

INFDFPKNAETYLHRIGRSGRYGHLGLAINLITSEDRFNLKATEDQLVTDIKPIPGSIDKSLYVAEFHSADAEAEAAGAE

PEAAEAEAEPVAAEAEPQAALLQES

>XP_016348239.1 PREDICTED: probable ATP-dependent RNA helicase ddx6 [Sinocyclocheilus anshuiensis]

MATKMENTGPAVMGLNTHNRQLLGQPKAPSQLGPQTVSSQSGKEPMVTQKPANASQERPGIRFGDDWKKSLQLPAKDSRV

KTSDVTATKGNEFEDYCLKRELLMGIFEMGWEKPSPIQEESIPIALSGRDVLARAKNGTGKRGAYLIPLLERIDIKKDHI

QAVVIVPTRELALQVSQISIQISKHLGGVKVMATTGGTNLRDDIMRLDEEVHVVIATPGRILDLIKKGIAKVDKVQMMVM

DEADKLLSQDFVVIIEDIIGLLSKNRQILLYSATFPISVQKFMAKHLQKPYEINLMEELTLKGITQYYAYVTERQKVHCL

NTLFSRLQINQSIIFCNSTQRVELLAKKITQLGYSCFYIHAKMMQEYRNRVFHDFRNGLCRNLVCTDLFTRGIDIQAVNV

VINFDFPRNAETYLHRIGRSGRFGHLGLAINLISAEDRFNLKTTEDQLMTDIKPIPGSIDKSLYVAEFHSVNPDCEAEAP

QPGEGSEAQ

>XP_016106333.1 PREDICTED: probable ATP-dependent RNA helicase DDX6 [Sinocyclocheilus grahami]

MATKMENTGPAVMGLNTHNRQLLGQPKAPSQLGPQTVSAQPGKEPMVTQKPANASQERPGIRFGDDWKKSLQLPAKDGRV

KTSDVTATKGNEFEDYCLKRELLMGIFEMGWEKPSPIQEESIPIALSGRDILARAKNGTGKSGAYLIPLLERIDIKKDHI

QAVVIVPTRELALQVSQISIQISKHLGGVKVMATTGGTNLRDDIMRLDEEVHVVIATPGRILDLIKKGIAKVDKVHMMVM

DEADKLLSQDFVVIIEDIIAFLSKNRQILLYSATFPISVQKFMAKHLQKPYEINLMEELTLKGITQYYAYVTERQKVHCL

NTLFSRLQINQSIIFCNSTQRVELLAKKITQLGYSCFYIHAKMMQEYRNRVFHDFRNGLCRNLVCTDLFTRGIDIQAVNV

VINFDFPRNAETYLHRIGRSGRFGHLGLAINLISAEDRFNLKTTEDQLMTDIKPIPGSIDKSLYVAEFHSVNPECEAEAP

QPGEESEAQ

>RXN10222.1 putative ATP-dependent RNA helicase ddx6 [Labeo rohita]

MATARTEIPASVMPMSKQNGQSKPMTLQSGSLGSSTQSGKIPVMPQKGSSIPQSSGGIRFGDDWKKCLQLPPKDTRVKTT

DVTATKGNEFEDYCLKRELLMGIFEMGWEKPSPIQEESIPIVLSGRDILARAKNGTGKSGAYLIPLLERIDLKKDYVQAI

VLVPTRELALQVSQISINMSKHLGGVKVMATTGGTNLRDDIMRLDETVHVIIATPGRILDLIKKGVAKVDRAQMVVMDEA

DKLLSQDFVVLIEDIISFLPKNRQILLYSATFPISVQKFMTKHLQKPYEINLMDELTLKGITQYYAYVTERQKVHCLNTL

FSRLQINQSIIFCNSTQRVELLAKKITQLGYSCFYIHAKMMQEYRNRVFHDFRNGLCRNLVCTDLFTRGIDIQAVNVVIN

FDFPKNAETYLHRIGRSGRYGHLGLAINLITSEDRFNLKGIEDQLMTDIKPIPSSIDKSLMGSVLAASSPNPPPASGGGS

APGGAGLVTVPPGFTMPPVSAVPPSSGTPGQPGTDAEASLQNPGTFEECHRKCKEVFPVQMEGVRLVVNKGLSNHFQVSH

TITLSTLGDSGYRFGATYVGSKQTGPAESFPVMVGDMDNTGSLNAQVIHQLTNRIRSKVAMQTQQHKFVNWQCDAEYRGE

DFTAAVTLGNPDVLVGSGSLDSNWVVGATLEKKLIPLPLSLALGAFLNHRKNKFQCGFGVTIG

>XP_016138972.1 PREDICTED: probable ATP-dependent RNA helicase ddx6 [Sinocyclocheilus grahami]

MATARTEIPSSVMPMSKQNGQSKPLTLQSGSLTSSTQSGKTPVMPQKGSSIHQSSGGIRFGDDWKKCLQLPPKDLRVKTT

DVTATKGNEFEDYCLKRELLMGIFEMGWEKPSPIQEESIPIVLSGRDILARAKNGTGKSGAYLIPLLERIDLKKDYVQAI

VFVPTRELALQVSQISINMSKHLGGVKIMATTGGTNLRDDIMRLDETVHVIIATPGRILDLIKKGVAKVDKAQMIVMDEA

DKLLSQDFVVLIEDIISFMPKKRQILLYSATFPSSVQKFMSKHLQKPYEINLMDELTLKGITQYYAYVTERQKVHCLNTL

FSRLQINQSIIFCNSTQRVELLAKKITQLGYSCFYIHAKMMQEYRNRVFHDFRNGLCRNLVCTDLFTRGIDIQAVNVVIN

FDFPRNAETYLHRIGRSGRYGHLGLAINLITSEDRFNLKGTEDQLMTDIKPIPSSIDKNLYVAEFHTMNPDEEEAAHKAG

ELN

>ROL45807.1 putative ATP-dependent RNA helicase DDX6 [Anabarilius grahami]

MENAGPAVMGLNTHNRQCIGQPKAPSQLGPQTAIAQPGKEQMGPQKPASAFQEGPGIRFGDDWKRSLQLPDKDNRVKTSD

VTATKGNEFEDYCLKRELLMGIFEMGWEKPSPIQEESIPIALSGRDILARAKNGTGKSGAYLIPLLERIDMKKDYIQAIV

MVPTRELALQVSQISIQISKHLGGVKVMATTGGTNLRDDIMRLDETVHVVIATPGRILDLIKKGIAKVDKVQMMVMDEAD

KLLSRDFVVLIEDIIGFLAKNRQILLYSATFPISVQTFMAKHLQKPYEINLMEELTLKGITQYYAYVTERQKVHCLNTLF

SRLQINQSIIFCNSTQRVELLAKKITQLGYSCFYIHAKMMQEYRNRVFHDFRNGLCRNLVCTDLFTRGIDIQAVNVVINF

DFPRNAETYLHRIGRSGRFGHLGLAINLITAEDRFNLKTIEDQLMTDIKPIPGSIDKSLYVAEFHSVDPDCEVEAEAEAE

AFQPTGGSDAP

>XP_028837456.1 probable ATP-dependent RNA helicase ddx6 [Denticeps clupeoides]

MATTRTENHPSAFGLNKQNGQFREQSKPVGLQSMPLAASSLPAKPPSSSQKGSGIPQTSGGIRFGDEWKKCLQLPPKDTR

VRTSDVTATKGNEFEDYCLKRELLMGIFEMGWEKPSPIQEESIPIALSGRDILARAKNGTGKSGAYLIPLLERIDLKKDH

IQAIVMVPTRELALQVSQISIQISKHMGGVKVMATTGGTVLRDDIMRLDETVHVIIATPGRILDLIKKGIAKVDRVQMMV

MDEADKLLSPDFVTLIEDTISFLAKKRQILLYSATFPISVQKFMTKHLQKPYEINLMDELTLKGITQYYAYVTERQKVHC

LNTLFSRLQINQSIIFCNSTQRVELLAKKITQLGYSCFYIHAKMMQEYRNRVFHDFRNGLCRNLVCTDLFTRGIDIQAVN

VVINFDFPKNAETYLHRIGRSGRYGHLGLAINLITSEDRFNLKGIEDQLMTDIKPIPGTIDKSLYVAEFHSVNPDADEAV

RDSGHKGVRKVK

>XP_016301389.1 PREDICTED: probable ATP-dependent RNA helicase ddx6 [Sinocyclocheilus anshuiensis]

MATARTEIPSSVMPMSKQNGQSKPLTLQSGSLTSSTQSGKTPVMPQKGSSIRQSSGGIRFGDDWKKCLQLPPKDLRVKTT

DVTATKGNEFEDYCLKRELLMGIFEMGWEKPSPIQEESIPIVLSGRDILARAKNGTGKSGAYLIPLLERIDLKKDYVQAI

VFVPTRELALQVSQISINMSKHLGGVKIMATTGGTNLRDDIMRLDETVHVIIATPGRILDLIKKGVAKVDKAQMIVMDEA

DKLLSQDFVVLIEDIISFMPKKRQILLYSATFPSSVQKFMSKHLQKPYEINLMDELTLKGITQYYAYVTERQKVHCLNTL

FSRLQINQSIIFCNSTQRVELLAKKITQLGYSCFYIHAKMMQEYRNRVFHDFRNGLCRNLVCTDLFTRGIDIQAVNVVIN

FDFPRNAETYLHRIGRSGRYGHLGLAINLITSEDRFNLKGTEDQLMTDIKPIPSSIDKNLYVAEFHTMNPDEEEAAHKAG

ELN

>XP_029476925.1 probable ATP-dependent RNA helicase ddx6 [Oncorhynchus nerka]

MSTARTENPTSIMGLNKPNGQFRGPLKPVGLSGSLATAPQPDAQQKGGNIPQGSGGIRFGDDWKKCLQLPQKDCRFRTSD

VTSTKGNEFEDYCLKRELLMGIFEMGWEKPSPVQEESIPIALSGRDILARAKNGTGKSGAYLIPLLERIDLKKDHIQAMV

MVPTRELALQVSQISIQVSKHMGGVKVMATTGGTNLRDDIMRLDETVHVVIATPGRILDLIKKGVAKVDKVQMMVMDEAD

KLLSQDFVVLIEDIISFLAKGRQILLYSATFPISVQKFMAKHLSKPYEINLMDELTLKGITQYYAYVTERQKVHCLNTLF

SRLQINQSIIFCNSTQRVELLAKKITQLGYSCFYIHAKMMQEYRNRVFHDFRNGLCRNLVCTDLFTRGIDIQAVNVVINF

DFPKNAETYLHRIGRSGRFGHLGLAINLITSEDRFNLKSIEDQLVTDIKPIPGSIDKGLYVAEFHSADPDAEDEIRGKDG

ELSAA

>XP_031658296.1 probable ATP-dependent RNA helicase ddx6 [Oncorhynchus kisutch]

MSTARTENPTSIMGLNKPNGQFRGPLKPVGLTSGSLASAPQPDAQQKGGNIPQGSGGIRFGDDWKKCLQLPQKDCRFRTS

DVTSTKGNEFEDYCLKRELLMGIFEMGWEKPSPVQEESIPIALSGRDILARAKNGTGKSGAYLIPLLERIDLKKDHIQAM

VMVPTRELALQVSQISIQVSKHMGGVKVMATTGGTNLRDDIMRLDETVHVVIATPGRILDLIKKGVAKVDKVQMMVMDEA

DKLLSQDFVVLIEDIISFLAKGRQILLYSATFPISVQKFMAKHLSKPYEINLMDELTLKGITQYYAYVTERQKVHCLNTL

FSRLQINQSIIFCNSTQRVELLAKKITQLGYSCFYIHAKMMQEYRNRVFHDFRNGLCRNLVCTDLFTRGIDIQAVNVVIN

FDFPKNAETYLHRIGRSGRFGHLGLAINLITSEDRFNLKSIEDQLVTDIKPIPGSIDKGLYVAEFHSADPDAEDEIRGKD

GELSAA

>KAF3847422.1 hypothetical protein F7725_020450, partial [Dissostichus mawsoni]

MSTARTENPMILGLSNQNGQLRGSVKTAGAPGGGVGGPQQQLNQMKGTINNGNSLPAPTTNAVIKRLEEKFEIAPKRHED

ENIGLYNIDVTATKGNEFEDYCLKRELLMGIFEMGWEKPSPIQEESIPIALSGRDILARAKNGTGKSGAYLIPLLERIDL

KRDCIQAMVIVPTRELALQVSQISIQVSKHMGGVKVMATTGGTNLRDDIMRLDETVHVVIATPGRILDLIKKGVAKVNQV

HMVVLDEADKLLSQDFVSMMEEMLGFLAKQRQILLYSATFPLSVQKFMNAHLQKPYEINLMEELTLKGVTQYYAYVTERQ

KVHCLNTLFSRLQINQSIIFCNSSQRVELLAKKISQLGYSCFYIHAKMRQEHRNRVFHDFRNGLCRNLVCTDLFTRGIDI

QAVNVVINFDFPKLGETYLHRIGRSGRFGHLGLAINLITYDDRFNLKGIEEQLGTEIKPIPGIIDKSLYISPSSIPLLYL

FFTPPPSPPPPVLEFSCFSFFMYSFCLSSFFGCHHEE

>XP_029535113.1 probable ATP-dependent RNA helicase ddx6 [Oncorhynchus nerka]

MSTARTENPTSIMGLNKPNGQFRGPLKPVGLTPGSLATTPQPDAHQKGGNIPQGSGGIRFGDDWKKCLQLPQKDCRFRTS

DVTSTKGNEFEDYCLKRELLMGIFEMGWEKPSPVQEESIPIALSGRDILARAKNGTGKSGAYLIPLLERIDLKKDHIQAM

VMVPTRELALQVSQISIQVSKHMGGVKVMATTGGTNLRDDIMRLDETVHVVIATPGRILDLIKKGVAKVDKVQMMVMDEA

DKLLSQDFVVLIEDIISFLAKGRQILLYSATFPISVQKFMAKHLSKPYEINLMDELTLKGITQYYAYVTERQKVHCLNTL

FSRLQINQSIIFCNSTQRVELLAKKITQLGYSCFYIHAKMMQEYRNRVFHDFRNGLCRNLVCTDLFTRGIDIQAVNVVIN

FDFPKNAETYLHRIGRSGRFGHLGLAINLITSEDRFNLKSIEDQLVTDIKPIPGSIDKGLYVAEFHSAETDAEDEIRGKD

GELSAA

>XP_021447222.1 probable ATP-dependent RNA helicase ddx6 [Oncorhynchus mykiss]

MSTARTENPTSIMGLNKPNGQFRGPLKPVGLTSGSLATAPQPDAQQKGGNIPQGSGGIRFGDDWKKCLQLPQKDCRFRTS

DVTSTKGNEFEDYCLKRELLMGIFEMGWEKPSPVQEESIPIALSGRDILARAKNGTGKSGAYLIPLLERIDLKKDHIQAM

VMVPTRELALQVSQISIQVSKHMGGVKVMATTGGTNLRDDIMRLDETVHVVIATPGRILDLIKKGVAKVDKVQMMVMDEA

DKLLSQDFVVLIEDIISFLAKGRQILLYSATFPISVQKFMAKHLSKPYEINLMDELTLKGITQYYAYVTERQKVHCLNTL

FSRLQINQSIIFCNSTQRVELLAKKITQLGYSCFYIHAKMMQEYRNRVFHDFRNGLCRNLVCTDLFTRGIDIQAVNVVIN

FDFPKNAETYLHRIGRSGRFGHLGLAINLITSEDRFNLKSIEDQLVTDIKPIPGSIDKGLYVAEFHSADPDAEDEIRGKD

GELSAA

>XP_014057064.1 PREDICTED: probable ATP-dependent RNA helicase ddx6 [Salmo salar]

MSTARTENPTSIMGLNKPNGQFRGPLKPVGLTSGSLATAPQPDAHQKGGNIPQSSGGIRFGDDWKKCLQLPQKDCRFRTS

DVTSTKGNEFEDYCLKRELLMGIFEMGWEKPSPVQEESIPIALSGRDILARAKNGTGKSGAYLIPLLERIDLKKDHIQAM

VMVPTRELALQVSQISIQVSKHMGGVKVMATTGGTNLRDDIMRLDETVHVVIATPGRILDLIKKGVAKVDKVQMMVMDEA

DKLLSQDFVVLIEDIISFLAKGRQILLYSATFPISVQKFMAKHLSKPYEINLMDELTLKGITQYYAYVTERQKVHCLNTL

FSRLQINQSIIFCNSTQRVELLAKKITQLGYSCFYIHAKMMQEYRNRVFHDFRNGLCRNLVCTDLFTRGIDIQAVNVVIN

FDFPKNAETYLHRIGRSGRFGHLGLAINLITSEDRFNLKSIEDQLVTDIKPIPGSIDKGLYVAEFHSADPDAEDEIRGKD

GELSAA

>XP_024002723.1 probable ATP-dependent RNA helicase ddx6 [Salvelinus alpinus]

MSSARTENPTSIMGLNKPNGQFRGPLKPVGLTSGSLATAPQPDAHQKGGNIPQGSGGIRFGDDWKKCLQLPQKDCRFRTS

DVTSTKGNEFEDYCLKRELLMGIFEMGWEKPSPVQEESIPIALSGRDILARAKNGTGKSGAYLIPLLERIDLKKDHIQAM

VMVPTRELALQVSQISIQVSKHMGGVKVMATTGGTNLRDDIMRLDETVHVVIATPGRILDLIKKGVAKVDKVQMMVMDEA

DKLLSQDFVVLIEDIISFLAKGRQILLYSATFPISVQKFMAKHLSKPYEINLMDELTLKGITQYYAYVTERQKVHCLNTL

FSRLQINQSIIFCNSTQRVELLAKKITQLGYSCFYIHAKMMQEYRNRVFHDFRNGLCRNLVCTDLFTRGIDIQAVNVVIN

FDFPKNAETYLHRIGRSGRFGHLGLAINLITSEDRFNLKSIEDQLVTDIKPIPGSIDKGLYVAEFHSADPDAEDEIRGKD

GELSAA

>XP_029600470.1 probable ATP-dependent RNA helicase ddx6 [Salmo trutta]

MSTARTENPTSIMGLNKPNGQFRGPLKPVGLTSGSLATAPQPDTHQKGGNIPQSSGGIRFGDDWKKCLQLPQKDCRFRTS

DVTSTKGNEFEDYCLKRELLMGIFEMGWEKPSPVQEESIPIALSGRDILARAKNGTGKSGAYLIPLLERIDLKKDHIQAM

VMVPTRELALQVSQISIQVSKHMGGVKVMATTGGTNLRDDIMRLDETVHVVIATPGRILDLIKKGVAKVDKVQMMVMDEA

DKLLSQDFVVLIEDIISFLAKGRQILLYSATFPISVQKFMAKHLSKPYEINLMDELTLKGITQYYAYVTERQKVHCLNTL

FSRLQINQSIIFCNSTQRVELLAKKITQLGYSCFYIHAKMMQEYRNRVFHDFRNGLCRNLVCTDLFTRGIDIQAVNVVIN

FDFPKNAETYLHRIGRSGRFGHLGLAINLITSEDRFNLKSIEDQLVTDIKPIPGSIDKGLYVAEFHSADPDAEDEIRGKD

GELSAA

>XP_017330489.1 PREDICTED: probable ATP-dependent RNA helicase DDX6 isoform X1 [Ictalurus punctatus]

MATARTQIPTSVMGVTKQNGQARSADLQSGSQPATVFSASQKGSSIPHTTGGIRFGDDWKKSLQLPQKDLRVKTSDVTAT

KGNEFEDYCLKRELLMGIFEMGWEKPSPIQEESIPIALSGRDILARAKNGTGKSGAYLIPLLERIDLKKDHIQAIVMVPT

RELALQVSQICINMSRHMGGVKVMATTGGTNLKDDIMRLDETVHVVIATPGRLLDLMKKGVAKADKVHMMVMDEADKLLS

QDFVILIEDIIGFLDKNRQILLYSATFPITVQKFMSKYQRKPYEINLMDELTLKGITQYYAYVTERQKVHCLNTLFSRLQ

INQSIIFCNSTQRVELLAKKITQLGYSCFYIHAKMMQEYRNRVFHDFRNGLCRNLVCTDLFTRGIDIQAVNVVINFDFPR

NAETYLHRIGRSGRYGHLGLAINLITSDDRFNLKTIEDQLVTDIKPIPGSIDKSLYVAEFHSINHEEEEEKEDALGAP

>XP_014010994.1 PREDICTED: probable ATP-dependent RNA helicase ddx6 [Salmo salar]

MSTARTENPTSIMGLNKPNGQFRGPLKPVGLTSGSLATAPQPDAHQKGGNIPQGSGGIRFGDDWKKCLQLPQKDCRFRTS

DVTSTKGNEFEDYCLKRELLMGIFEMGWEKPSPVQEESIPIALSGRDILARAKNGTGKSGAYLIPLLERIDLKKDHIQAM

VMVPTRELALQVSQISIQVSKHMGGVKVMATTGGTNLRDDIMRLDETVHVVIATPGRILDLIKKGVAKVDKVQMMVMDEA

DKLLSQDFVVLIEDIISFLAKGRQILLYSATFPISVQKFMAKHLSKPYEINLMDELTLKGITQYYAYVTERQKVHCLNTL

FSRLQINQSIIFCNSTQRVELLAKKITQLGYSCFYIHAKMMQEYRNRVFHDFRNGLCRNLVCTDLFTRGIDIQAVNVVIN

FDFPKNAETYLHRIGRSGRFGHLGLAINLITSEDRFNLKSIEDQLVTDIKPIPGSIDKGLYVAEFHSADPDAEDEIRGKD

GELSAA

>XP_035607455.1 probable ATP-dependent RNA helicase ddx6 [Oncorhynchus keta]

MSTARTENPTSIMGLNKPNGQFRGPLKPVGLTSGSLATAPQPDAQQKGGNIPQGSGGIRFGDDWKKCLQLPQKDCRFRTS

DVTSTKGNEFEDYCLKRELLMGIFEMGWEKPSPVQEESIPIALSGRDILARAKNGTGKSGAYLIPLLERIDLKKDHIQAM

VMVPTRELALQVSQISIQVSKHMGGVKVMATTGGTNLRDDIMRLDETVHVVIATPGRILDLIKKGVAKVDKVQMMVMDEA

DKLLSQDFVVLIEDIISFLAKGRQILLYSATFPISVQKFMAKHLSKPYEINLMDELTLKGITQYYAYVTERQKVHCLNTL

FSRLQINQSIIFCNSTQRVELLAKKITQLGYSCFYIHAKMMQEYRNRVFHDFRNGLCRNLVCTDLFTRGIDIQAVNVVIN

FDFPKNAETYLHRIGRSGRFGHLGLAINLITSEDRFNLKSIEDQLVTDIKPIPGSIDKGLYVAEFHSGDPDAEDEIRGKD

GELSAA

>XP_029593428.1 probable ATP-dependent RNA helicase ddx6 [Salmo trutta]

MSTARMENPTSIMGLNKPNGQFRGPLKPVGLTSGSLATAPEPDAHQKGGNIPQGSGGIRFGDDWKKCLQLPQKDCRFRTS

DVTSTKGNEFEDYCLKRELLMGIFEMGWEKPSPVQEESIPIALSGRDILARAKNGTGKSGAYLIPLLERIDLKKDHIQAM

VMVPTRELALQVSQISIQVSKHMGGVKVMATTGGTNLRDDIMRLDETVHVVIATPGRILDLIKKGVAKVDKVQMMVMDEA

DKLLSQDFVVLIEDIISFLAKGRQILLYSATFPISVQKFMAKHLSKPYEINLMDELTLKGITQYYAYVTERQKVHCLNTL

FSRLQINQSIIFCNSTQRVELLAKKITQLGYSCFYIHAKMMQEYRNRVFHDFRNGLCRNLVCTDLFTRGIDIQAVNVVIN

FDFPKNAETYLHRIGRSGRFGHLGLAINLITSEDRFNLKSIEDQLVTDIKPIPGSIDKGLYVAEFHSADPDAEDEIRGKD

GELSAA

>XP_035618274.1 probable ATP-dependent RNA helicase ddx6 [Oncorhynchus keta]

MSTARTENPTSIMGLNKPNGQFRGPLKPVGLTPGSLATTPQPDAHQKGGNIPQGSGGIRFGDDWKKCLQLPQKDCRFRTS

DVTSTKGNEFEDYCLKRELLMGIFEMGWEKPSPVQEESIPIALSGRDILARAKNGTGKSGAYLIPLLERIDLKKDHIQAM

VMVPTRELALQVSQISIQVSKHMGGVKVMATTGGTNLRDDIMRLDETVHVVIATPGRILDLIKKGVAKVDKVQMMVMDEA

DKLLSQDFVVLIEDIISFLAKGRQILLYSATFPISVQKFMAKHLSKPYEINLMDELTLKGITQYYAYVTERQKVHCLNTL

FSRLQINQSIIFCNSTQRVELLAKKITQLGYSCFYIHAKMMQEYRNRVFHDFRNGLCRNLVCTDLFTRGIDIQAVNVVIN

FDFPKNAETYLHRIGRSGRFGHLGLAINLITSEDRFNLKSIEDQLVTDIKPIPGSIDKGLYVAEFHSAEPDAEDEIRGKD

GELSAA

>XP_021480011.1 probable ATP-dependent RNA helicase ddx6 [Oncorhynchus mykiss]

MSTARTENPTSIMGLNKPNGQFRGPLKPVGLTPGSLATTPQPDAHQKGGNIPQGSGGIRFGDDWKKCLQLPQKDCRFRTS

DVTSTKGNEFEDYCLKRELLMGIFEMGWEKPSPVQEESIPIALSGRDILARAKNGTGKSGAYLIPLLERIDLKKDHIQAM

VMVPTRELALQVSQISIQVSKHMGGVKVMATTGGTNLRDDIMRLDETVHVVIATPGRILDLIKKGVAKVDKVQMMVMDEA

DKLLSQDFVVLIEDIISFLAKGRQILLYSATFPISVQKFMAKHLSKPYEINLMDELTLKGITQYYAYVTERQKVHCLNTL

FSRLQINQSIIFCNSTQRVELLAKKITQLGYSCFYIHAKMMQEYRNRVFHDFRNGLCRNLVCTDLFTRGIDIQAVNVVIN

FDFPKNAETYLHRIGRSGRFGHLGLAINLITSEDRFNLKSIEDQLVTDIKPIPGSIDKGLYVAEFHSADPDAEDEIRGKD

GELSAA

>XP_020329469.2 probable ATP-dependent RNA helicase ddx6 [Oncorhynchus kisutch]

MSTARTENPTSLMGLNKPNGQFRGPLKPVGLTPGSLATTPQPDDHQRGGNIPQGSGGIRFGDDWKKCLQLPQKDCRFRTS

DVTSTKGNEFEDYCLKRELLMGIFEMGWEKPSPVQEESIPIALSGRDILARAKNGTGKSGAYLIPLLERIDLKKDHIQAM

VMVPTRELALQVSQISIQVSKHMGGVKVMATTGGTNLRDDIMRLDETVHVVIATPGRILDLIKKGVAKVDKVQMMVMDEA

DKLLSQDFVVLIEDIISFLAKGRQILLYSATFPISVQKFMAKHLSKPYEINLMDELTLKGITQYYAYVTERQKVHCLNTL

FSRLQINQSIIFCNSTQRVELLAKKITQLGYSCFYIHAKMMQEYRNRVFHDFRNGLCRNLVCTDLFTRGIDIQAVNVVIN

FDFPKNAETYLHRIGRSGRFGHLGLAINLITSEDRFNLKSIEDQLVTDIKPIPGSIDKGLYVAEFHSAEPDTEDEIRGKD

GELSAA

>CAF90961.1 unnamed protein product, partial [Tetraodon nigroviridis]

MATTRTENVGPVIMGLNKQNGQLRGQTKAASVQSASATPGKPLGPSQSPAGAPQDGGGIKFGDDWKRNLKLPPKDNRVRT

SDVTATKGNEFEDYCLKRELLMGIFEMGWEKPSPIQEESIPIALSGRDILARAKNGTGKSGAYLIPMLERIDLKKDHIQA

LVLVPTRELALQVSQISIQIAKHLGGVKVMATTGGTNLRDDIMRLDETVHVVIATPGRILDLMKKGVAKVDKVQIMVMDE

VGKRTPKAALCGGVGAAGPCVWVVSPQADKLLSQDFVALVEDIISFLAKNRQILLYSATFPISVQKFMAKHLQKPYEINL

MEELTLKGITQYYAYVTERQKVHCLNTLFSRLQINQSIIFCNSTQRVELLAKKITQLGYSCFYIHAKMMQEYRNRVFHDF

RNGLCRNLVCTDLFTRGIDIQAVNVVINFDFPKNAETYLHRIGRSGRFGHLGLAINLITSDDRFNLKTIEEQLITDIKPI

PGSIDKSLYVAEFHSADCEVEE

>ROL52221.1 putative ATP-dependent RNA helicase DDX6 [Anabarilius grahami]

MENPVILGLSNQNGQMRGSVKPAGGPGGGGGGSQTMQPAQVKASSTVNNGNSQPAPTANTIIKPGDDWKKNLKLPPKDLR

MRTSDVTATKGNEFEDYCLKRELLMGIFEMGWEKPSPIQEESIPIALSGRDILARAKNGTGKSGAYLIPLLERIDLKKDS

IQALVIVPTRELALQVSQICIQVSKHMGGVKVMATTVHVVIATPGRILDLIKKGVAKVNQVQMVVLDEADKLLSQDFVQM

MEEMLSSLPKQRQILLYSATFPLSVQKFMNNHLQKPYEINLMEELTLKGVTQYYAYVTERQKVHCLNTLFSRLQINQSII

FCNSSQRVELLAKKISQLGYSCFYIHAKMRQEHRNRVFHDFRNGLCRNLVCTDLFTRGIDIQAVNVVINFDFPKLGETYL

HRIGRSGRFGHLGLAINLITYDDRFNLKGIEEQLGTEIKPIPSSIDKSLYVAEYHSESGEEVKLPIYCTGEVLRQVQMAK

QFDDDKYFVDMKLTTAPEVIVEAFANLTSGFPNKTVPSSELQKFLQTYFEEPGKEFEQWTPPDWHSKPKFLSKVSDSKYR

SWAEELHGLWKSLGRKIRDDVRDHPELYSQIYTPHPVVVPGGRFRELYYWDSYWVINGLLLSEMTETARGMILNFMFLVE

RYGFVPNGGRVYYERRSQPPFLPLMVESFYEVTRDKDFLGQVLPALEREYSFWMQNRSHVVAINGLTHILNQYNVPVDRP

RPESYSDDVELAEGLSTEAQKRLWMELTSGAESGWDFSSRWYIDSTGRNNGTLSDTQTSSILPADLNAIMCRNERLLASF

HRILGNEDKAAEYDKALSARIKAVESLLWDAERGAWFDFSLVNETRHLSFYPSNLAPLWARCYSKPEMGDQAVQYLRDSG

GLDYPNGVPTSLSESGQQWDMPNAWPPLQHMIIEGLSGLDSAHAKELAFSLAQRWMQTNWRAYIKYEAMFEKYDVNGDGK

PGGGGEYEVQLGFGWTNGIALQLLDQYGDRLSSGGCVLNASILIGLVYPILKSLFLF

>XP_024246779.1 probable ATP-dependent RNA helicase ddx6 [Oncorhynchus tshawytscha]

MSTARTENPTSIMGLNKPNGQFRGPLKPVGLTSGSLATAPQPDAQQKGGNIPQGSGGIRFGDDWKKCLQLPQNDCRFRTS

DVTSTKGNEFEDYCLKRELLMGIFEMGWEKPSPVQEESIPIALSGRDILARAKNGTGKSGAYLIPLLERIDLKKDHIQAM

VMVPTRELALQVSQISIQVSKHMGGVKVMATTGGTNLRDDIMRLDETVHVVIATPGRILDLIKKGVAKVDKVQMMVMDEA

DKLLSQDFVVLIEDIISFLAKGRQILLYSATFPISVQKFMAKHLSKPYEINLMDELTLKGITQYYAYVTERQKVHCLNTL

FSRLQINQSIIFCNSTQRVELLAKKITQLGYSCFYIHAKMMQEYRNRVFHDFRNGLCRNLVCTDLFTRGIDIQAVNVVIN

FDFPKNAETYLHRIGRSGRFGHLGLAINLITSEDRFNLKSIEDQLVTDIKPIPGSIDKGLYVAEFHSADPDAEDEIRGKD

GELSAA

>OXB59010.1 hypothetical protein ASZ78_007638 [Callipepla squamata]

MSTARTENPVIMGLSSQNGQLRGPVKPSGGPGGGGTQTQQQMNQLKNANTINNGTQQQAQSMTTTIKPGDDWKKTLKLPP

KDLRIKTSDVTSTKGNEFEDYCLKRELLMGIFEMGWEKPSPIQEESIPIALSGRDILARAKNGTGKSGAYLIPLLERLDL

KKDNIQAMVIVPTRELALQVSQICIQVSKHMGGAKVMATTGGTNLRDDIMRLDDTVHVVIATPGRILDLIKKGVAKVEHV

QMIVLDEASCSWSDPIVKLERRLFLVLFSLCSVVLPEDWKLSLISYFVLLLLQADKLLSQDFVQIMEDIILTLPKNRQIL

LYSATFPLSVQKFMNSHLQKPYEINLMEELTLKGVTQYYAYVTERQKVHCLNTLFSRLQINQSIIFCNSSQRVELLAKKI

SQLGYSCFYIHAKMRQEHRNRVFHDFRNGLCRNLVCTGLDFMEINVLHGLACLSDLFTRGIDIQAVNVVINFDFPKLAET

YLHRIGRSGRFGHLGLAINLITYDDRFNLKSIEEQLGTEIKPIPSNIDKSLYVAEYHSEPVEDEKQ

>XP_016146666.1 PREDICTED: probable ATP-dependent RNA helicase ddx6 [Sinocyclocheilus grahami]

MATKMENIGPAVIGLNTHNRHLIGQPKGPSQLGPQSVSSQPGKEPMGTQKPANASQEGPGIRFGDDWKKSLLLPAKDNRV

KTSDVTATKGNEFEDYCLKRELLMGIFEMGWEKPSPIQEESIPIALSGRDILARAKNGTGKSGAYLIPLLERIDIKKDHI

QAIVIVPTRELALQVSQISIQISKHLGGVKVMATTGGTNLRDDIMRLDEEVHVVIATPGRILDLIKKGIAKVNKVQMMVM

DEADKLLSQDFVVIIEDIIGFLSKNRQILLYSATFPISVQKFMAKHLQKPYEINLMEELTLKGITQYYAYVTERQKVHCL

NTLFSRLQINQSIIFCNSTQRVELLAKKITQLGCSCFYIHAKMMQEYRNRVFHDFRNGLCRNLVSTDLFTRGIDIQAVNV

VINFDFPKNAETYLHRIGRSGRFGHLGLAINLITAEDRFNLKTIEDQLMTDIKPIPGSIDKSLYVAEFHSVNPDCEAEAS

HLSGGSEAH

>XP_026087924.1 probable ATP-dependent RNA helicase ddx6 isoform X2 [Carassius auratus]

MMHLIGQPKGPSQSGKEPMGTPKPAIATQEGPGIRFGDDWKKSLLLPAKDNRFKTSDVTATKGNEFEDYCLKRELLMGIF

EMGWEKPSPIQEESIPIALSGRDILARAKNGTGKSGAYLIPLLERIDIKKDHIQAIVIVPTRELALQVSQISIQISKHLG

GVKVMATTGGTNLRDDIMRLDEEVHVVIATPGRILDLIKKGIAKVNKVQMMVMDEADKLLSQDFVVIIEDIISFLSKNRQ

ILLYSATFPISVQKFMAKHLQKPYEINLMEELTLKGITQYYAYVTEKQKVHCLNTLFSRLQINQSIIFCNSTQRVELLAK

KITQLGCSCFYIHAKMMQEYRNRVFHDFRNGLCRNLVCTDLFTRGIDIQAVNVVINFDFPKNAETYLHRIGRSGRFGHLG

LAINLITAEDRFNLKTIEDQLMTEITPIPGSIDKSLYVAEFHSVNPDCEAETSHPGGGSEAH

>PWA16789.1 hypothetical protein CCH79_00017865 [Gambusia affinis]

MAAARTENLGPVVMGLNKQNGQLKGQTKPAAGPPASTQGKNPAGSNQDGGGIKFGDDWKKCLQLPPKDNRVKTSDVTATK

GNEFEDYCLKRELLMGIFEMGWEKPSPIQEESIPIALSGRDILARAKNGTGKSGAYLIPLLERIDLKKDHIQAVVMVPTR

ELALQMSQICIQLSKHLGGVKVMATTGGTNLRDDIMRLDETVHVVIATPGRILDLIKKGVAKVDRVQMMVMDEADKLLSQ

DFVVLIEDIISFLAKNRQILLYSATFPISVQKFMVSQQDGPLRNRTVSPGRPWERQQIWEEFESKLKVKHLQKPYEINLM

EELTLKGITQFYAYVTERQKVHCLNTLFSRLQINQSIIFCNSTQRVELLAKKITQLGYSCFYIHAKMMQEYRNRVFHDFR

NGLCRNLVCTDLFTRGIDIQAVNVVINFDFPKNAETYLHRIGRSGRFGHLGLAINLITSEDRFNLKAIEEQLVTDIKPIP

SSIDKSLYVAEYHSGPVTDGDEDDAEEKPPRQQDST

>XP_026087921.1 probable ATP-dependent RNA helicase DDX6 isoform X1 [Carassius auratus]

MTTKMENTGPAFIGLNTHNRHLIGQPKGPSQSGKEPMGTPKPAIATQEGPGIRFGDDWKKSLLLPAKDNRFKTSDVTATK

GNEFEDYCLKRELLMGIFEMGWEKPSPIQEESIPIALSGRDILARAKNGTGKSGAYLIPLLERIDIKKDHIQAIVIVPTR

ELALQVSQISIQISKHLGGVKVMATTGGTNLRDDIMRLDEEVHVVIATPGRILDLIKKGIAKVNKVQMMVMDEADKLLSQ

DFVVIIEDIISFLSKNRQILLYSATFPISVQKFMAKHLQKPYEINLMEELTLKGITQYYAYVTEKQKVHCLNTLFSRLQI

NQSIIFCNSTQRVELLAKKITQLGCSCFYIHAKMMQEYRNRVFHDFRNGLCRNLVCTDLFTRGIDIQAVNVVINFDFPKN

AETYLHRIGRSGRFGHLGLAINLITAEDRFNLKTIEDQLMTEITPIPGSIDKSLYVAEFHSVNPDCEAETSHPGGGSEAH

>XP_016300016.1 PREDICTED: probable ATP-dependent RNA helicase ddx6 [Sinocyclocheilus anshuiensis]

MATKMENIGPAVIGLNTHNRHVIGQPKGRSQLGPQSVSSQPGKEPMGTQKPANASQEGPGIRFGDDWKKSLLLPAKDNRV

KTSDVTATKGNEFEDYCLKRELLMGIFEMGWEKPSPIQEESIPIALSGRDILARAKNGTGKSGAYLIPLLERIDIKKDHI

QAIVIVPTRELALQVSQISIQISKHLGGVKVMATTGGTNLRDDIMRLDEEVHVVIATPGRILDLIKKGIAKVNKVQMMVM

DEADKLLSRDFVVIIEDIIGFLSKNRQILLYSATFPISVQKFMAKHLQKPYEINLMEELTLKGITQYYAYVTERQKVHCL

NTLFSRLQINQSIIFCNSTQRVELLAKKITQLGCSCFYIHAKMMQEYRNRVFHDFRNGLCRNLVSTDLFTRGIDIQAVNV

VINFDFPKNAETYLHRIGRSGRFGHLGLAINLITAEDRFNLKTIEDQLMTDIKPIPGSIDKSLYVAEFHSVNPDCEAEAS

HPGGGSEAH

>4CT4_B Chain B, Probable Atp-dependent Rna Helicase Ddx6 [Homo sapiens]

RSMGNEFEDYCLKRELLMGIFEMGWEKPSPIQEESIPIALSGRDILARAKNGTGKSGAYLIPLLERLDLKKDNIQAMVIV

PTRELALQVSQICIQVSKHMGGAKVMATTGGTNLRDDIMRLDDTVHVVIATPGRILDLIKKGVAKVDHVQMIVLDEADKL

LSQDFVQIMEDIILTLPKNRQILLYSATFPLSVQKFMNSHLQKPYEINLMEELTLKGVTQYYAYVTERQKVHCLNTLFSR

LQINQSIIFCNSSQRVELLAKKISQLGYSCFYIHAKMRQEHRNRVFHDFRNGLCRNLVCTDLFTRGIDIQAVNVVINFDF

PKLAETYLHRIGRSGRFGHLGLAINLITYDDRFNLKSIEEQLGTEIKPIPSNIDKSLY

>NXQ21030.1 DDX6 helicase [Peucedramus taeniatus]

MSTARTENPVIMGLSSQNGQLRGPVKPSGGPGGGGTQTQQQMNQLKNANTINNGTQQQAQSMTTAIKPGDDWKKTLKLPP

KDLRIKTSDVTSTKGNEFEDYCLKRELLMGIFEMGWEKPSPIQEESIPIALSGRDILARAKNGTGKSGAYLIPLLERLDL

KKDNIQAMVIVPTRELALQVSQICIQVSKHMGGAKVMATTGGTNLRDDIMRLDDTVHVVIATPGRILDLIKKGVAKVEHV

QMIVLDEANKLLSQDFVQIMEDIILTLPKNRQILLYSATFPLSVQKFMNSHLQKPYEINLMEELTLKGVTQYYAYVTERQ

KVHCLNTLFSRLQINQSIIFCNSSQRVELLAKKISQLGYSCFYIHAKMRQSAFELIEINILCDLDCLSDLFTRGIDIQAV

NVVINFDFPKLAETYLHRIGRSGRFGHLGLAINLITYDDRFNLKSIEEQLGTEIKPIPSNIDKSLYVAEYHSEPVEDEKQ

>XP_035801069.1 probable ATP-dependent RNA helicase DDX6 isoform X2 [Amphiprion ocellaris]

MKTSDVTSTKGNEFEDYCLKRELLMGIFEMGWEKPSPIQEESIPIALSGRDILARAKNGTGKSGAYLIPLLERIDLKKDH

IQAIVMVPTRELALQMSQISIQLSKHLGGVKVMATTGGTNLRDDILRLDEIVHVVVATPGRILDLIKKGVAKVDQTQMMV

MDEADKLLSQDFVALIEDIISFLPKNRQILLYSATFPISVQKFMSKHLQKPYEINLMEELTLKGITQYYAYVTERQKVHC

LNTLFSRLQINQSIIFCNSTQRVELLAKKITQLGYSCFYIHAKMMQEYRNRVFHDFRNGLCRNLVCTDLFTRGIDIQAVN

VVINFDFPKNAETYLHRIGRSGRFGHLGLAINLITSDDRYNLKTIEDQLITDIKPIPSSIDKSLYVAEFHSVDPDEDGDE

GGAKNKDLGAA

>TNN89199.1 putative ATP-dependent RNA helicase DDX6 [Liparis tanakae]

MATARTANSAPMIGLNKPANGQLRGQAGLLGSAQQPNALQKRTGIPQSSGGIKFGDDWKKCLELPPRDTRMRTSDVTSTK

GNEFEDYCLKRELLMGIFEMGWEKPSPVQEESIPIALSGRDILARAKNGTGKSGAYLIPLLERIDLKKDHIQAIVMVPTR

ELALQMSQISIQLSKHLGGVKVMATTGGTNLRDDIMRLDETGRILDLIKKGVAKMDKAQLIVMDEADKLLSQDFVVLIED

IISFMPRDRQILLYSATFPISVQKFMSKHLKKPYEINLMEELTLKGITQYYAYVTERQKVHCLNTLFSRLQINQSIIFCN

STQRVELLAKKITQLGYSCFYIHAKMMQEYRNRVFHDFRNGLCRNLVCTDLFTRGIDIQAVNVVINFDFPKNAETYLHRI

GRSGRFGHLGLAINLITSEDRYNLKNIEDQLVTDIKPIPSCIDKSLYVAEFHSVDPDADDDDLGGEIGGAKSKGLGAI

>XP_020470396.1 probable ATP-dependent RNA helicase DDX6 [Monopterus albus]

MSTARTENPVILGLSNQNGQIRGSVKPTGAPGGGGGGLQQQQLNQMKGTVNNGNSLPAPTTNAVIKPGDDWKKNLKLPPK

DMRMKTSDVTATKGNEFEDYCLKRELLMGIFEMGWEKPSPIQEESIPIALSGRDILAXXXXXXXXLKKDCIQALVIVPTR

ELALQVSQICIQVSKHMGGVKVMATTGGTNLRDDIMRLDETVHVVIATPGRILDLIKKGVAKVNQVQMIVLDEADKLLSQ

DFVVMMEEILGFLSKQRQILLYSATFPLSVQKFMNAHLQKPYEINLMEELTLKGVTQYYAYVTERQKVHCLNTLFSRLQI

NQSIIFCNSSQRVELLAKKISQLGYSCFYIHAKMRQEHRNRVFHDFRNGLCRNLVCTDLFTRGIDIQAVNVVINFDFPKL

GETYLHRIGRSGRFGHLGLAINLITYDDRFNLKGIEEQLGTEIKPIPGIIDKSLYVAEYHSESGEEVKP

>RXM95798.1 putative ATP-dependent RNA helicase DDX6 [Acipenser ruthenus]

MSTARMETPVILGLSNQNGQLRGPVKPPGGPGGGGGGVTPTQQTNQIKNASVNNGNPYPAQTPNSTIKPGDDWKKNLQVP

TKDMRMKTSDVTATKGNEFEDYCLKRELLMGIFEMGWEKPSPIQEESIPIALSGRDILARAKNGTGKSGAYLIPLLERID

LKKDCIQVCMLVHSQHRPVEESIPIALSGRDILARAKNGTGKSGAYLIPLLERIDLKKDCIQALVVVPTRELALQVSQIS

IQVSKHMGGVKVMATTGGTNLRDDIMRLDETVHVVIATPGRILDLIKKGVAKVSKVQMIVLDEADKLLSQDFVQIMEEII

GTLAKNRQVLLYSATFPTSVQKFMSTHLQKPYEINLMEELTLKGVTQYYAYVTERQKVHCLNTLFSRLQINQSIIFCNSS

QRVELLAKKISQLGYSCFYIHAKMRQEHRNRVFHDFRNGLCRNLVCTDLFTRGIDIQAVNVVINFDFPKLAETYLHRIGR

SGRFGHLGLAINLITYDDRFNLKGIEEQLGTEIKPIPGSIDKSLYVAEYHSETGEEDKL

>XP_020514390.1 probable ATP-dependent RNA helicase DDX6 [Labrus bergylta]

MSTARTENPVILGLSNQNGQLRGSVKPAGAPGGGGGGGGGGGGPQLQLNQMKGAINNGNSQPVPTTNAVIKPGDDWKKNL

KLPPKDMRMRTSDVTATKGNEFEDYCLKRELLMGIFEMGWEKPSPIQEESIPIALSGRDILARAKNGTGKSGAYLIPLLE

RIDLKKDCIQAMVIVPTRELALQVSQICIQVSKHMGGVKVMATTGGTNLRDDIMRLDETGIMATTGGTNLRDDIMRLDEQ

VNVLADKLLSQDFVVMMEEILGFLAKQRQILLYSATFPLSVQKFMNSHLQKPYEINLMEELTLKGVTQYYAYVTERQKVH

CLNTLFSRLQINQSIIFCNSSQRVELLAKKISQLGYSCFYIHAKMRQEHRNRVFHDFRNGLCRNLVCTDLFTRGIDIQAV

NVVINFDFPKLGETYLHRIGRSGRFGHLGLAINLITYDDRFNLKGIEEQLGTEIKPIPGIIDKSLYVAEYHSESGEELKP

>XP_017378740.1 PREDICTED: probable ATP-dependent RNA helicase DDX6 [Cebus capucinus imitator]

MSTARTENPVIMGLSSQNGQLRGPVKPSGGPGGGGTQTQQQMNQLKNTNTINNGTQQQAQSMTTTIKPGDDWKKTLKLPP

KDLRIKTSDVTSTKGNEFEDYCLKRELLMGIFEMGWEKPSPIQEESIPIALSGRDILARAKNGTGKSGAYLIPLLERLDL

KKDNIQAMVIVPTRELALQVSQICIQVSKHMGGAKVMATTGGTNLRDDIMRLDDTVHVVIATPGRILDLIKKGVAKVDHV

QMIVLDEADKLLSQDFVQIMEDIILTLPKNRQILLYSATFPLSVQKFMNSHLQKPYEINLMEELTLKGVTQYYAYVTERQ

KVHCLNTLFSRLQINQSIIFCNSSQRVELLAKKISQLGYSCFYIHAKMRQVSIKLELAQIYLFTRGIDIQAVNVVINFDF

PKLAETYLHRIGRSGRFGHLGLAINLITYDDRFNLKSIEEQLGTEIKPIPSNIDKSLYVAEYHSEPVEDEKP

>KAF5167554.1 hypothetical protein HST_G00086550 [Hippoglossus stenolepis]

MATARTANPATMMGLNKAANGQFRGQTRPPGQQSGLLAAAQQSSDPMKRTGIPQSSGGIKFGDDWKRCLELPPKDTRLRT

SDVTSTKGNEFEDYCLKRELLMGIFEMGWEKPSPIQEESIPIALSGRDILARAKNGTGKSGAYLIPMLERIDLRKDHIQA

MVMVPTRELALQMSQISIQLSKHLGGVKVMATTGGTNLRDDIMRLEETVHVVIATPGRILDLIKKGVAVVDKLQMMVMDE

ADKLLSQDFVVLIEDIISFLPKNRQILLYSATFPVSVQTFMNKHLQKPYEINLMEELTLKGITQYYAYVTERQKVHCLNT

LFSKLQINQSIIFCNSVQRVELLAKKITQLGYSCFYIHAKMMQEYRNRVFHDFRNGLCRNLVCTDLFTRGIDIQAVNVVI

NFDFPKNAETYLHRIGRSGRFGHLGLAINLITIDDRNNLKTIEDQLMMTVMGKAKTMNWDKPESKEKRGTWHSQDSQVKV

SISHFAIKMV

>XP_015345257.1 PREDICTED: LOW QUALITY PROTEIN: probable ATP-dependent RNA helicase DDX6 [Marmota marmota marmota]

MSTARTENPVIMGLSSQNGQLRGPVKPSGGPGGGGTQSQQQMNQLKNTNTINNGTQQQAQSMTTTIKPGDDWKKTLKLPP

KDLRIKTSDVTSTKGNEFEDYCLKRELLMGIFEMGWEKPSPIQEESIPIALSGRDILARAKNGTGKSGAYLIPLLERLDL

KKDNIQAMVIVPTRELALQVSQICIQVSKHMGGAKVMATTGGTNLRDDIMRLDDTVHVVIATPGRILDLIKKGVAKVDHV

QMIVLDEVIYILEYTLYTDYFKFLAFXVEGRKILLSFKCVSSYFQNSHLQKPYEINLMEELTLKGVTQYYAYVTERQKVH

CLNTLFSRLQINQSIIFCNSSQRVELLAKKISQLGYSCFYIHAKMRQEHRNRVFHDFRNGLCRNLVCTDLFTRGIDIQAV

NVVINFDFPKLAETYLHRIGRSGRFGHLGLAINLITYDDRFNLKSIEEQLGTEIKPIPSNIDKSLYVAEYHSEPVEDEKP

>KAF4101670.1 hypothetical protein G5714_018102 [Onychostoma macrolepis]

MRTSDVTATKGNEFEDYCLKRELLMGIFEMGWEKPSPIQEESIPIALSGRDILARAKNGTGKSGAYLIPLLERIDLKKDS

IQALVIVPTRELALQVSQICIQVSKHMGGVKVMATTGGTNLRDDILRLDETVHVVIATPGRILDLIKKGVAKVNQVQMIV

LDEADKLLSQDFVQMMEELLSYLPKQRQILLYSATFPLSVQKFMNSHLQKPYEINLMEELTLKGVTQYYAYVTERQKVHC

LNTLFSRLQINQSIIFCNSSQRVELLAKKISQLGYSCFYIHAKMRQEHRNRVFHDFRNGLCRNLVCTDLFTRGIDIQAVN

VVINFDFPKLGETYLHRIGRSGRFGHLGLAINLITYDDRFNLKVRVERRLNCEQERSPILCPLSSTSVFFLPYCV

>XP_017330497.1 PREDICTED: probable ATP-dependent RNA helicase ddx6 isoform X2 [Ictalurus punctatus]

MEMLEALIIIFLSKTIDVSIWLMLIGQHSSKQGVLPLDTPVCSDLQLSLSPDVTATKGNEFEDYCLKRELLMGIFEMGWE

KPSPIQEESIPIALSGRDILARAKNGTGKSGAYLIPLLERIDLKKDHIQAIVMVPTRELALQVSQICINMSRHMGGVKVM

ATTGGTNLKDDIMRLDETVHVVIATPGRLLDLMKKGVAKADKVHMMVMDEADKLLSQDFVILIEDIIGFLDKNRQILLYS

ATFPITVQKFMSKYQRKPYEINLMDELTLKGITQYYAYVTERQKVHCLNTLFSRLQINQSIIFCNSTQRVELLAKKITQL

GYSCFYIHAKMMQEYRNRVFHDFRNGLCRNLVCTDLFTRGIDIQAVNVVINFDFPRNAETYLHRIGRSGRYGHLGLAINL

ITSDDRFNLKTIEDQLVTDIKPIPGSIDKSLYVAEFHSINHEEEEEKEDALGAP

>RXN09990.1 B-cell lymphoma 3 -like protein [Labeo rohita]

MTMSGSNHTVRSVPLDLTNKPRERGSCAQTGDQGRSRHTPERQVEPVHKCSNGHGVLPSEPQDTCDGPVSQTAQTDTVGV

RGNRTGEGPKQTRPISPSAKCNKNAAGKPSDSSFSNLPPRKRPFPVDVDHDRSGPRKHPPSPAPKVLKTPEAQTDTEQQR

IFSAFGENPRVNDPRLVPHLTPYHCPYFLPRQPPPLESTDRLMADIAEATRQDEDGDTPLHIAVVQENCQLVIWLTDIYR

RGRKDLDVFNNLRQTPLHLAVITHQPILVKALLEAGADPGALDRNGQTALHLCCEHGEADCLSVILRHYPQNPSPYLEIR

NYEGLTPLHLAVQNGDKKLARILLKSGAEINAGDNKSGRSPLVHAVENNFTDMVIFLIESGCDVNAQSYSGNTALHSACG

RGHIEIVRVLLKNGADSSVKNNHNDTAIMVANNKKVSDVLRGRGTRSCTVKTSSNGSLSPGSSSHSPRMTPTFQRSASHS

PVTPVLPRSQSAENISERQSPMNSHEKTLQMLPRTHHNVAVDNRAGYGMGLHHYPYLIPGVPVTYGSPSQGAGNRSRPSS

RSSDQSDMSTVSVNSEERGIVTATKGNEFEDYCLKRELLMGIFEMGWEKPSPIQEESIPIVLSGRDILARAKNGTGKSGA

YLIPLLERIDLKKDYVQAIVLVPTRELALQVSQISINMSKHLGGVKVMATTGGTNLRDDIMRLDETVHVIIATPGRILDL

IKKGVAKVDRAQMVVMDEADKLLSQDFVVLIEDIISFLPKNRQILLYSATFPISVQKFMTKHLQKPYEINLMDELTLKGI

TQYYAYVTERQKVHCLNTLFSRLQINQSIIFCNSTQRVELLAKKITQLGYSCFYIHAKMMQEYRNRVFHDFRNGLCRNLV

CTDLFTRGIDIQAVNVVINFDFPKNAETYLHRIGRSGRYGHLGLAINLITSEDRFNLKGIEDQLMTDIKPIPSSIDKSLL

ALCPPLHRLEQEVETFRRRAIADTLLTVSRMEKARTEYRGALLWMKDVSQELDPDTYKQLEKFRKVQAQVRGTKVHFDKL

KNDVCQKVDMLGASRCNMLSHSLCTYQTTLLQYWEKTAHVMSGIHEAFKGYVPYQFTTLKELRDPLDQIVSAQTEEDSKD

EDKKTQPDKMGLFRREASLKALVTDLLVSLEDEQLGESSDTVSTRAVDGQSRGSDSSLCASLDSVGRDLLSEAFDSAGVK

EEEGERGDLAFLQDLLSPGAAGGASEFSKAWQDAFGCFEAPPAPVTAPSPQPEMTNTPTGFLPSQLLDHSLSATGWVTPP

MFQAPPLQPPSSGAQPTQNSPHSSARAPKGNSRDMSAWFNLFADLDPLSNPDAIGRSDQEFLNA

>KAA0714695.1 putative ATP-dependent RNA helicase DDX6 [Triplophysa tibetana]

MSTARMENPVILGLSNQNGQMRGSVKPAGGPGGGGGGSQTTQSAQVKGSSTVNNGNSQPAPTANTIIKPGDDWKKNLKLP

PKDMRMKTSDVTATKGNEFEDYCLKRELLMGIFEMGWEKPSPIQEESIPIALSGRDILARAKNGTGKSGAYLIPLLERID

LKKDNIQAMVIVPTRELALQVSQISIQVSKHMGGVKVMATTVHVIIATPGRILDLIKKGVAKVSQADKLLSQDFVQMMEE

LLSSLSKQRQILLYSATFPLSVQKFMNTHLQKPYEINLMEELTLKGVTQYYAYVTERQKVHCLNTLFSRLQINQSIIFCN

SSQRVELLAKKISQLGYSCFYIHAKMRQEHRNRVFHDFRNGLCRNLVCTDLFTRGIDIQAVNVVINFDFPKLGETYLHRI

GRSGRFGHLGLAINLITYDDRFNLKGIEEQLGTEIKPIPSSIDKSLYVAEYHSESGEEVKLQIYCTGQILKQIQMARLFD

DDKHFVDMKLTAHPEVVVAAFENLTSGFPNKTVPSSDLLKFLNVYFEEPGKEFEMWTPPDWHSNPKFLSNISDPKYRRWA

EELHRLWKSLGRKIRDDVRDHPELYSQIYTPHPVVVPGGRFRELYYWDSYWIIKGLLLSEMTETARGMILNFKFLVERYG

FVPNGGRIYYERRSQPPFLSLMVESFFEVTEDKDFLRRVLPALEKEYSFWMQNRSHTVVKNGATHILNRYDVQVAGPRPE

SYSDDVELAEGLSTEAQQKLWAEIKAGAESGWDFTSRWYIDSLNANSGTLRDTQTSSILPADLNAIMCRNERLLASFHRT

LGNEQKATEYDQALSARIQALESLLWDPERKAWFDFSLLTQSRHTSFYPSNMAPLWARCYSKPEMGDQAMQYLKGSGGLD

YPNGVPTSLSASGQQWDMPNAWPPLQHMIVEGLSALESAHAKELAFDLTQRWIQTNWLAYDKYDAMYEKVKST

>TSO05420.1 putative ATP-dependent RNA helicase DDX6 [Bagarius yarrelli]

MENTAAVMMGISKQNGQLRAPSSRSGPGVLSNPFTSVPTVGQKPAADSPEGPGISIVDVTATKGNEFEDYCLKRELLMGI

FEMGWEKPSPVQEESIPIALSGRDILARAKNGTGKSGAYLIPMLERIDLKKDHVQALVLVPTRELALQISQISIQISKHL

GGVKVMATTGGTNLRDDIMRLDEIVHVIVATPGRILDLIRKGVAKVDKVQIMIMDEADKLLSQDFVLLVEDIISFLDKNR

QILLYSATFPITVQKFMAKHLKKPYEINLMEELTLKGITQYYAYVTERQKVHCLNTLFSRLQINQSIIFCNSTQRVELLA

KKITQLGYSCFYIHAKMMQEYRNRVFHDFRNGLCRNLVCTDLFTRGIDIQAVNVVINFDFPKNAETYLHRIGRSGRFGHL

GLAINLITAEDRFNLKAIEDQLVTEIKPIPSSIDKSLYVAEFHSADDQAEQQEMPDDP

>NXV79365.1 DDX6 helicase [Atlantisia rogersi]

MSTARTENPVIMGLSSQNGQLRGPVKPSGGPGGGGTQTQQQMNQLKNANTINNGTQQQAQSMTTAIKPGDDWKKTLKLPP

KDLRIKTSDVTSTKGNEFEDYCLKRELLMGIFEMGWEKPSPIQEESIPIALSGRDILARAKNGTGKSGAYLIPLLERLDL

KKDNIQAMVIVPTRELALQVSQICIQVSKHMGGAKVMATTGGTNLRDDIMRLDDTVHVVIATPGRILDLIKKGVAKVEHV

QMIVLDEANKLLSQDFVQIMEDIILTLPKNRQILLYSATFPLSVQKFMNSHLQKPYEINLMEELTLKGVTQYYAYVTERQ

KVHCLNTLFSRLQINQSIIFCNSSQRVELLAKKISQLGYSCFYIHAKMRQEHRNRVFHDFRNGLCRNLVCTDLFTRGIDI

QAVNVVINFDFPKLAETYLHRIGRSGEW

>KAA8591724.1 hypothetical protein FQN60_017098 [Etheostoma spectabile]

MATARTANPAQMIGLNKPANGQLRGQAALLAAAQQPSAPQKRTSIPQSSGGIKFGDDWKKCLELPPKDTRMRTADVTSTK

GNEFEDYCLKRELLMGIFEMGWEKPSPVQEESIPIALSGRDILARAKNGTGKSGAYLIPLLERIDLKKDHIQAIVMVPTR

ELALQVSQISIQLSKHLGGVKVMATTGGTNLRDDIMRLDETVHVVIATPGRILDLIKKGVAKMDKAQLIVMDEADKLLSQ

DFVVLIEDIISFMPKDRQILLYSATFPISVQKFMNKHLKKPYEINLMEELTLKGITQYYAYVTERQKVHCLNTLFSRLLA

KKITQLGYSCFYIHAKMMQEYRNRVFHDFRNGLCRNLVCTDLFTRGIDIQAVNVVINFDFPKNAETYLHRIGRSGRFGHL

GLAINLITSEDRYNLKNIEDQLVTDIKPIPSSIDKSLYVAEFHSVDPDDDENDYDDHGRAKKKELGGI

>KAF2977032.1 hypothetical protein EK904_013099 [Melospiza melodia maxima]

MPGLKQNGQLRGPVKPSGGPGGGGTQTQQQMNQLKNANTINNGTQQQAQSMTTAIKPGDDWKKTLKLPPKDLRIKTSDVT

STKGNEFEDYCLKRELLMGIFEMGWEKPSPIQEESIPIALSGRDILARAKNGTGKSGAYLIPLLERLDLKKDNIQAMVIV

PTRELALQVSQICIQVSKHMGGAKVMATTGGTNLRDDIMRLDDTDKLLSQDFVQIMEDIILTLPKNRQILLYSATFPLSV

QKFMNSHLQKPYEINLMEELTLKGVTQYYAYVTERQKVHCLNTLFSRINQSIIFCNSSQRVELLAKKISQLGYSCFYIHA

KMRQEHRNRVFHDFRNGLCRNLVCTDLFTRGIDIQAVNVVINFDFPKLAETYLHRIGRSGRFGHLGLAINLITYDDRFNL

KSIEEQLGTEIKPIPSNIDKSLYVAEYHSEPVEDEKQ

>KFP50442.1 putative ATP-dependent RNA helicase DDX6, partial [Cathartes aura]

MSTARTENPVIMGLSSQNGQLRGPVKPSGGPGGGGTQTQQQMNQLKNANTINNGTQQQAQSMTTAIKPGDDWKKTLKLPP

KDLRIKTSDVTSTKGNEFEDYCLKRELLMGIFEMGWEKPSPIQEESIPIALSGRDILARAKNGTGKSGAYLIPLLERLDL

KKDNIQAMVIVPTRELALQVSQICIQVSKHMGGAKVMATTGGTNLRDDIMRLDDTVHVVIATPGRILDLIKKGVAKVEHV

QMIVLDEANKLLSQDFVQIMEDIILTLPKNRQILLYSATFPLSVQKFMNSHLQKPYEINLMEELTLKGVTQYYAYVTERQ

KVHCLNTLFSRLQINQSIIFCNSSQRVELLAKKISQLGYSCFYIHAKMRQEHRNRVFHDFRNGLCRNLVCTDLFTRGIDI

QAVNVVINFDFPKLAETYLHRIGRSG
