# Supplementary material for: DDX6 Is Essential for Oocyte Development and Maturation in Locusta migratoria
Source: Insects. 2021 Jan 14;12(1):70. doi: 10.3390/insects12010070 (PMC7830464; doi:10.3390/insects12010070)
Supplement: Supplementary file 1 [file insects-12-00070-s001.zip › Supplementary material/Supplemental file 6.docx]

**500 sequences of DXX6 from plants**

>XP_030551180.1 ATP-dependent RNA helicase me31b-like [Rhodamnia argentea]

MLAEHYEGHWPFWLSPLQAQVIPINDNQIDYAKQVETEFRELGFWVDSDVATDKLNAKIRNASLKHYNLILLVGSKEESD

RSVTCRLVRSRQSSVGCQASVESGNNFDKQDKKFSNNSLSVHNLTIPLDKLKKAFVDYERRYVDKADIELLNDYGGRVAI

RHVSAASMLAARHKLRQRALKANYSSRLLTLQSKSQSCRFGNTLLYYIFKMDVQLRSIVKMPPKDRRKKTTDVTATKGQE

FEDLCLKRELLMGIFEKGWEKPSPIQEASIPISLLGRDVLARAKNGTGKTGAYIIPLLEKIDTKSDDIQALIIVPTRELA

LQTSQICIELSKHLGARVMATTGGTNLKDDIMRIYDNVHVIIATPGRILDLMEKKVAQMSFCKMLVLDEADKLLSQDFSG

MLDQVIALLPPERQILLFSATFPLTVRQFVDKHLTKRKPYEINLMEELTLKGVTQYYAYVQEKQKVHCLNTLFSKLQINQ

SIIFCNSTQRVELLAKKIAELGYSCYYIHAKMSQPHRNRVFHDFRNGLCRNLVCSDLFTRGIDIQAVNVVINFDFPKMSE

TYLHRIGRSGRFGHLGIAINLITYEDRFNLHRIEQELGTEIKPIPKVIDKSLYVAEYQQEDDE

>BAJ93616.1 predicted protein [Hordeum vulgare subsp. vulgare]

MATNNLSNDIKSDEAAGWKNKLNLPPKDKRVKTTDVTATKGHEFEDYCLKRELLMGIFEKGWETPSPIQESSVPIALTGR

DILARAKNGTGKTGAYVIPILERIDVSKDKIQALIIVPTRELALQTSQICIELSKHMGCKVMATTGGTNLKEDIMRLQQQ

VHVIIATPGRILDLMKKGLAVMDTCSMLVMDEADKLLSQDFKNMLDSVISYLPPDRQILLYSATFPCTVDQFIKKHMHNP

YEINLMEELTLKGITQYYAYVQEKQKVHCLNTLFSKLQINQSIIFCNSTQRVELLAKKITELGYSCFYIHAKMRQEHRNR

IFHDFRNGACRNLVCSDLFTRGIDIQAVNVVINFDFPKMSETYLHRIGRSGRFGHLGIAINLITYDDRYALHKIEQELGT

EIKPIPKVIDPHLYVAEYQTEEEFNQLKLSEQKEKDKQQDSSSVQQQQHQQPPTPLQQLVQAP

>ABR16163.1 unknown [Picea sitchensis]

MNSRGRYGQGAAVANGRVGQVNSYPKSVQQNFAQRNHQQWVSRVQIQAEKDAAAAKRGNPSEEEKRVQSGDVDPSVQDWK

SQLKIPPPDSRYKTEDVTATKGNEFEDYFLKRELLMGIYEKGFERPSPIQEESIPIALTGSDILARAKNGTGKTAAFCIP

ALEKIDPKKNSIQVLLLVPTRELALQTSQVCKELAKHLKIQIMVTTGGTSLKDDIMRLYQPVHILVATPGRVLDLTKKGV

CNLKDCAMLVMDEADKLLSPEFQPLVEQLIGFLPENRQILLYSATFPVTVKSFKDKYLRKPYVINLMDELTLKGITQFYA

FVEERQKVHCLNTLFSKLQINQSIIFCNSVNRVELLAKKITELGYSCFYIHAKMLQSHRNRVFHDFRNGACRNLVCSDLF

TRGIDIQAVNVVINFDFPKNSETYLHRVGRSGRFGHLGLAVNLITYEDRFNLYKIEQELGTEIQQIPPQIDQTVYCR

>XP_002987276.2 DEAD-box ATP-dependent RNA helicase 8 [Selaginella moellendorffii]

MQHYQPRLARPQGRVSQPFAQQQQQSQQQNPPLQNSNALAGGPAGDADGNAKVLASSSLQEWKAQLKTPPADARYRTEDV

TATKGNEFEDYFLKRELLMGIYEKGFERPSPIQEESIPIALTGSDILARAKNGTGKTAAFCIPAIEKIDPNKNAIQVLIL

VPTRELALQTSQVCKELAKHLKIETMVTTGGTSLKDDIMRLYQPVHLLVGTPGRVLDLANKGVCKLKDCSMMVMDEADKL

LSPEIQPLVERLLSFLPESRQVLLFSATFPVTVKQFKEKFLRKPYVINLMDELTLKGITQYYAFVEERQKVHCLNTLFSK

LQINQSIIFCNSVNRVELLAKKITELGYSCFYIHAKMLQSHRNRVFHDFRNGACRNLVSSDLFTRGIDIQAVNVVINFDF

PKNSETYLHRVGRSGRFGHLGLAVNLITYEDRFNLYRIEQELGTEIKPIPPQIDQAIYCR

>XP_031486568.1 DEAD-box ATP-dependent RNA helicase 8-like [Nymphaea colorata]

MNSRGRYAAGAANGRGGYVGTYHQLQNYVQRGSQFHQQQQWLRRSPAVPETDVGAAEGNMQNEEEKRVQSEAVESSSQDW

KAQLKLPPIDTRYRTEDVTATKGNEFEDYFLKRELLMGIYEKGFERPSPIQEESIPIALTGSDILARAKNGTGKTAAFCI

PALEKIDPNKNAIQVIILVPTRELALQTSQVCKELGKHLKVQIMVTTGGTSLKDDIMRLYQPVHLLVGTPGRILDLVKKG

VCILKDCSMLVMDEADKLLSQEFQPLVEQLISFLPTNRQILLFSATFPVTVKAFKDKLLLKPYVINLMDELTLKGITQYY

AFVEERQKVHCLNTLFSKLQINQSIIFCNSVNRVELLAKKITELGYSCFYIHAKMLQSHRNRVFHDFRNGACRNLVCSDL

FTRGIDIQAVNVVINFDFPKNSETYLHRVGRSGRFGHLGLAVNLITYEDRFNLYRIEQELGTEIKAIPAQIDQAIYCR

>XP_006827696.1 DEAD-box ATP-dependent RNA helicase 8 [Amborella trichopoda]

MNNRRGYLPSYGGTPQRLPQQIVQRGPLQNQQQQQQWLRRDQVEEEKRVTSEDAASSSQDWKAQLKIPPKDSRYKTEDVT

ATKGNEFEDYFLKRELLMGIYEKGFERPSPIQEESIPIALTGSDILARAKNGTGKTAAFCIPALEKIDQNNNVIQVIILV

PTRELALQTAQVCKELGKHLKIQIMVTTGGTSLKDDIMRLYQPVHLLVGTPGRVLDLAKKGICNLKDCTMLVMDEADKLL

SQEFQPSVEQLIGFLPPNRQILLFSATFPVTVKEFKDKFLRKSYIINLMDELTLKGITQYYAFVEERQKVHCLNTLFSKL

QINQSIIFCNSVNRVELLAKKITELGYSCFYIHAKMLQSHRNRVFHDFRNGACRNLVCSDLFTRGIDIQAVNVVINFDFP

RNSETYLHRVGRSGRFGHLGLAVNLITYEDRFSLYRIEQELGTEIKPIPPQIDQAIYCK

>XP_012846538.1 PREDICTED: DEAD-box ATP-dependent RNA helicase 8-like [Erythranthe guttata]

MYNNQGRGRYLPGIGNGRGGGGGGGGGGFNQNYQNRNPQYQQPQPRQQPQPPYPQRAAQNQPQQWMRRNPSASAVSDSYN

EVEKTVQSEAIGSTSEDWKAGLRIPPADNRFRTEDVTATKGNEFEDYFLKRELLMGIYEKGFERPSPIQEESIPIALTGS

DILARAKNGTGKTAAFCIPALEKIDSDNNVIQVVILVPTRELALQTSQVCKELGKHLKIQVMVTTGGTSLKDDIMRLYQP

VHLLVATPGRVLDLTKKGICNLKHCSVLVMDEADKLLSPEFQPSVNELISFLPPNRQILMYSATFPVTVKEFKDRHLRRP

YVINLMDELTLKGVTQYYAFVEERQKVHCLNTLFSKLQINQSIIFCNSVNRVELLAKKITELGYSCFYIHAKMLQDHRNR

VFHDFRNGACRNLVCTDLFTRGIDIQAVNVVINFDFPKNAETYLHRVGRSGRFGHLGLAVNLITYEDRFNLYRIEQELGA

EIKQIPPLIDQAIYCC

>XP_024389734.1 DEAD-box ATP-dependent RNA helicase 8-like isoform X1 [Physcomitrium patens]

MATSRAGPAPTAHKAASSSNVSRQQQQQHHQQQQHNSNNTSSGSGGNNNNSVSINNSSSSINNNTNNNSQDWKAQLKLPP

PDARYKTEDVTATKGNEFEDYFLKRELLMGIYEKGFERPSPIQEESIPIALTGSDILARAKNGTGKTAAFCIPAIEKIDQ

NKNAVQVLLLVPTRELALQTSQVCKELAKHLNIQIMVTTGGTSLRDDIMRLYQPVHLLVGTPGRVLDLANKGVCNLKECT

MLVMDEADKLLSPEFQPLVEQLIGFLPENRQILLYSATFPVTVKSFKDRFLRKPYVINLMDELTLKGITQYYAFVEERQK

VHCLNTLFSKLQINQSIIFCNSVNRVELLAKKITELGYSCFYIHAKMLQSHRNRVFHDFRNGACRNLVSSDLFTRGIDIQ

AVNVVINFDFPKNSETYLHRVGRSGRFGHLGLAVNLITYEDRFNLYRIEQELGTEIKPIPPQIDRGIYCR

>XP_024367950.1 DEAD-box ATP-dependent RNA helicase 6-like [Physcomitrium patens]

MMATSRAGPAPTAPKASSSSNVSRHNSSNSGNILSSNNNSSVNNNNSQDWKAQLKLPPPDARYKTEDVTATKGNEFEDYF

LKRELLMGIYEKGFERPSPIQEESIPIALTGSDILARAKNGTGKTAAFCIPAIEKIDQNKNAVQVLLLVPTRELALQTSQ

VCKELAKHLNIQIMVTTGGTSLRDDIMRLYQPVHLLVGTPGRVLDLANKGVCNLKECTMLVMDEADKLLSPEFQPLVEQL

IGFLPENRQILLYSATFPVTVKSFKDRFLRKPYVINLMDELTLKGITQYYAFVEERQKVHCLNTLFSKLQINQSIIFCNS

VNRVELLAKKITELGYSCFYIHAKMLQSHRNRVFHDFRNGACRNLVSSDLFTRGIDIQAVNVVINFDFPKNSETYLHRVG

RSGRFGHLGLAVNLITYEDRFNLYRIEQELGTEIKPIPPQIDRGIYCR

>XP_018446859.1 PREDICTED: DEAD-box ATP-dependent RNA helicase 12-like [Raphanus sativus]

MNNNRGRYPPESGTRRGAPPRPSYQSQHGQPHKHQQQQWSRRAQLPGNANEVQNTASSQPPVPSNDHKFNGSVTLSGDQD

WKATLKLPPPDTRYQTADVTATKGNEFEDYFLKRDLLKGIYEKGFEKPSPIQEESIPIALTGSDILARAKNGTGKTGAFC

IPVLERIDPNTNVIQAMILVPTRELALQTSQVCKELSKYLNIQVMVTTGGTSLRDDIMRLHQPVHLLVGTPGRILDLTKK

GVCVLKDCTMLVMDEADKLLSAEFQPSLEELIQFLPENRQFLMFSATFPVTVKAFKDRHLRKPYVINLMDQLTLMGVTQY

YAFVEERQKVHCLNTLFSKLQINQSIIFCNSVNRVELLAKKITELGYSCFYIHAKMVQDHRNRVFHEFRNGACRNLVCTD

LFTRGIDIQAVNVVINFDFPRTSESYLHRVGRSGRFGHLGLAVNLVTYEDRFKMYQTEQELGTEIKPIPSQIDQAIYCQ

>XP_013636400.1 PREDICTED: DEAD-box ATP-dependent RNA helicase 6 isoform X1 [Brassica oleracea var. oleracea]

MDNNSSRGRFPPGIGAPDPNFQSRNPNPNPNPPPQQFLHSRTPFPQQYAQSRTPLPDAQQYVQRGYTQPQQIQQQQQWST

RAQLPGDSSYVEEVEKTVQSEANSNQDWKATLKLPPRDNRYQTEDVTATKGNEFEDYFLKRDLLRGIYEKGFEKPSPIQE

ESIPIALTGSDILARAKNGTGKTGAFCIPTLEKIDPENNVIQAVILVPTRELALQTSQVCKELSKYLKIEVMVTTGGTSL

RDDIMRLYQPVHLLVGTPGRILDLTKKGVCVLKDCAMLVMDEADKLLSAEFQPSIDELIQFLPQNRQILMFSATFPVTVK

YFKDRYLRKPYIINLMDQLTLMGVTQYYAFVEERQKVHCLNTLFSKLQINQSIIFCNSVNRVELLAKKITELGYSCFYIH

AKMAQDHRNRVFHDFRNGACRNLVCTDLFTRGIDIQAVNVVINFDFPRTSESYLHRVGRSGRYGHLGLAVNLVTYEDRFK

MYQTEQELGTEIKPIPSLIDKAIYCQ

>XP_013636403.1 PREDICTED: DEAD-box ATP-dependent RNA helicase 6 isoform X2 [Brassica oleracea var. oleracea]

MDNNSSRGRFPPGIGAPDPNFQSRNPNPNPNPPPQQFLHSRTPFPQQYAQSRTPLPDAQQYVQRGYTQPQQIQQQQQWST

RAQLPGDSSYVEEVEKTVQSEANNQDWKATLKLPPRDNRYQTEDVTATKGNEFEDYFLKRDLLRGIYEKGFEKPSPIQEE

SIPIALTGSDILARAKNGTGKTGAFCIPTLEKIDPENNVIQAVILVPTRELALQTSQVCKELSKYLKIEVMVTTGGTSLR

DDIMRLYQPVHLLVGTPGRILDLTKKGVCVLKDCAMLVMDEADKLLSAEFQPSIDELIQFLPQNRQILMFSATFPVTVKY

FKDRYLRKPYIINLMDQLTLMGVTQYYAFVEERQKVHCLNTLFSKLQINQSIIFCNSVNRVELLAKKITELGYSCFYIHA

KMAQDHRNRVFHDFRNGACRNLVCTDLFTRGIDIQAVNVVINFDFPRTSESYLHRVGRSGRYGHLGLAVNLVTYEDRFKM

YQTEQELGTEIKPIPSLIDKAIYCQ

>EAY94957.1 hypothetical protein OsI_16765 [Oryza sativa Indica Group]

MATSMKANIERRKTREHQQHQHQHQQPPHPHHHQYVQRQPQPQQTPHNSQHQQWLRRNQIAAEAAGASEQKAPPVADGID

SSSQDWKAQLKLPPQDTRYRTEDVTATKGNEFEDYFLKRELLMGIYEKGFERPSPIQEESIPIALTGSDILARAKNGTGK

TAAFCIPALEKIDQDKNAIQVVILVPTRELALQTSQVCKELGKHLKIQVMVTTGGTSLKDDIVRLYQPVHLLVGTPGRIL

DLTKKGVCVLKNCSMLVMDEADKLLSPEFQPSIQELIRYLPSNRQILMFSATFPVTVKEFKDKYLPKPYVINLMDELTLK

GITQFYAFVEERQKVHCLNTLFSKLQINQSIIFCNSVNRVELLAKKITELGYSCFYIHAKMLQDHRNRVFHDFRNGACRN

LVCTDLFTRGIDIQAVNVVINFDFPKSAETYLHRVGRSGRFGHLGLAVNLITYEDRFNLYRIEQELGTEIKPIPPQIDRA

IYCQ

>VAI47452.1 unnamed protein product [Triticum turgidum subsp. durum]

MDPRARYPPGTGNGRGGNPNYYGRGPPLSQNNHHHHHQTSAAHQQQYVQRQPQPQPQQHHQNNQQQHHHQSNHQQQHHQN

HHQQQQQQQQQWLRRNQITAAGTSGPKVVAPPPAAVGNDPSSQDWKAQLKLPPADTRFRTEDVTATKGNEFEDYFLKREL

LMGIYEKGFEKPSPIQEESIPIALTGSDILARAKNGTGKTAAFCIPALEKIDQDKNAIQVVIVVPTRELALQTSQVCKEL

GKHLKIQVMVTTGGTSLKDDIVRLYQPVHLLVGTPGRILDLTKKGVCILKDCSMLVMDEADKLLSPEFQPSIEQLIRYLP

ASRQILMFSATFPVTVKEFKDKYLPKPYVINLMDELTLKGITQFYAFVEERQKVHCLNTLFSKLQINQSIIFCNSVNRVE

LLAKKITELGYSCFYIHAKMLQDHRNRVFHDFRNGACRNLVCTDLFTRGIDIQAVNVVINFDFPKSSETYLHRVGRSGRF

GHLGLAVNLITYEDRFNLYRIEQELGTEIKPIPPQIDQAIYCQ

>VAI47454.1 unnamed protein product [Triticum turgidum subsp. durum]

MDPRARYPPGTGNGRGGNPNYYGRGPPLSQNNHHHHHQTSAAHQQQYVQRQPQPQPQQHHQKQQQWLRRNQITAAGTSGP

KVVAPPPAAVGNDPSSQDWKAQLKLPPADTRFRTEDVTATKGNEFEDYFLKRELLMGIYEKGFEKPSPIQEESIPIALTG

SDILARAKNGTGKTAAFCIPALEKIDQDKNAIQVVIVVPTRELALQTSQVCKELGKHLKIQVMVTTGGTSLKDDIVRLYQ

PVHLLVGTPGRILDLTKKGVCILKDCSMLVMDEADKLLSPEFQPSIEQLIRYLPASRQILMFSATFPVTVKEFKDKYLPK

PYVINLMDELTLKGITQFYAFVEERQKVHCLNTLFSKLQINQSIIFCNSVNRVELLAKKITELGYSCFYIHAKMLQDHRN

RVFHDFRNGACRNLVCTDLFTRGIDIQAVNVVINFDFPKSSETYLHRVGRSGRFGHLGLAVNLITYEDRFNLYRIEQELG

TEIKPIPPQIDQAIYCQ

>VAI47453.1 unnamed protein product [Triticum turgidum subsp. durum]

MDPRARYPPGTGNGRGGNPNYYGRGPPLSQNNHHHHHQTSAAHQQQYVQRQPQPQPQQHHQQQQQQQQWLRRNQITAAGT

SGPKVVAPPPAAVGNDPSSQDWKAQLKLPPADTRFRTEDVTATKGNEFEDYFLKRELLMGIYEKGFEKPSPIQEESIPIA

LTGSDILARAKNGTGKTAAFCIPALEKIDQDKNAIQVVIVVPTRELALQTSQVCKELGKHLKIQVMVTTGGTSLKDDIVR

LYQPVHLLVGTPGRILDLTKKGVCILKDCSMLVMDEADKLLSPEFQPSIEQLIRYLPASRQILMFSATFPVTVKEFKDKY

LPKPYVINLMDELTLKGITQFYAFVEERQKVHCLNTLFSKLQINQSIIFCNSVNRVELLAKKITELGYSCFYIHAKMLQD

HRNRVFHDFRNGACRNLVCTDLFTRGIDIQAVNVVINFDFPKSSETYLHRVGRSGRFGHLGLAVNLITYEDRFNLYRIEQ

ELGTEIKPIPPQIDQAIYCQ

>VAI47455.1 unnamed protein product [Triticum turgidum subsp. durum]

MDPRARYPPGTGNGRGGNPNYYGRGPPLSQNNHHHHHQTSAAHQQQYVQRQPQQQHHQNHHQQQQQQQQQWLRRNQITAA

GTSGPKVVAPPPAAVGNDPSSQDWKAQLKLPPADTRFRTEDVTATKGNEFEDYFLKRELLMGIYEKGFEKPSPIQEESIP

IALTGSDILARAKNGTGKTAAFCIPALEKIDQDKNAIQVVIVVPTRELALQTSQVCKELGKHLKIQVMVTTGGTSLKDDI

VRLYQPVHLLVGTPGRILDLTKKGVCILKDCSMLVMDEADKLLSPEFQPSIEQLIRYLPASRQILMFSATFPVTVKEFKD

KYLPKPYVINLMDELTLKGITQFYAFVEERQKVHCLNTLFSKLQINQSIIFCNSVNRVELLAKKITELGYSCFYIHAKML

QDHRNRVFHDFRNGACRNLVCTDLFTRGIDIQAVNVVINFDFPKSSETYLHRVGRSGRFGHLGLAVNLITYEDRFNLYRI

EQELGTEIKPIPPQIDQAIYCQ

>VVA98297.1 unnamed protein product [Arabis nemorensis]

MNNNRGRFPPGIGAAAAPDPNFQSRNPNQPQQYLQSRAPFPQQPQQYVQSRTLNPPQPEASQYVQRGYPQNPQQIQQQQQ

WSRSAQLPGNPSYVDEVEKTVQSEANNDSNNQDWKATLKLPPRDDRYQTEDVTATKGNEFEDYFLKRDLLRGIYEKGFEK

PSPIQEESIPIALTGSDILARAKNGTGKTGAFCIPTLEKIDPEKNVIQAVILVPTRELALQTSQVCKELSKYLKIEVMVT

TGGTSLRDDIMRLYQPVHLLVGTPGRILDLTKKGVCVLKDCAMLVMDEADKLLSAEFQPSIEELIQFLPQNRQILMFSAT

FPVTVKSFKDRYLRKPYIINLMDQLTLMGVTQYYAFVEERQKVHCLNTLFSKLQINQSIIFCNSVNRVELLAKKITELGY

SCFYIHAKMVQDHRNRVFHDFRNGACRNLVCTDLFTRGIDIQAVNVVINFDFPRTSESYLHRVGRSGRYGHLGLAVNLVT

YEDRFKMYQTEQELGTEIKPIPSLIDKAIYCQ

>XP_017218906.1 PREDICTED: DEAD-box ATP-dependent RNA helicase 8 [Daucus carota subsp. sativus]

MNSRGRYPPGIGGAGGGNPNYQPRNPNPNQQYVQRNYMQNQQQFAQQPQQNTQQQWLRRGQIGGDSGAEEVEKTVQSEAA

DTSSNDWKARLKLPPQDTRYRTEDVTATKGNEFEDYFLKRELLMGIYEKGFEKPSPIQEESIPIALTGSDILARAKNGTG

KTAAFCIPALEKIDQDNNVIQVVILVPTRELALQTSQVCKELGKHLNIEVMVTTGGTSLKDDIMRLYQPVHLLVGTPGRI

LDLAKKGICNLQNCAMLVMDEADKLLSPEFQPSIEHLISFLPTNRQILMFSATFPVTVKDFKDRYLQKPYVINLMDELTL

KGITQFYAFVEERQKIHCLNTLFSKLQINQSIIFCNSVNRVELLAKKITELGYSCFYIHAKMLQDHRNRVFHDFRNGACR

NLVCTDLFTRGIDIQAVNVVINFDFPKSAETYLHRVGRSGRFGHLGLAVNLITYEDRFNLYRIEQELGTEIKQIPPHIDQ

AIYCQ

>KAF7079576.1 hypothetical protein CFC21_083791 [Triticum aestivum]

MDPRARYPPGTGNGRGGNPNYYGRGPPLSQNNHHHQQQTSAAHQQQYVQRQPQPQPQQHHQNNQQQHHHQSNHQQQHHQN

HHHQQQQQQQQWLRRNQITAAGTSGPKVVAPPPAAVGNDPSSQDWKAQLKLPPADTRFRTEDVTATKGNEFEDYFLKREL

LMGIYEKGFEKPSPIQEESIPIALTGSDILARAKNGTGKTAAFCIPALEKIDQDKNAIQVVIVVPTRELALQTSQVCKEL

GKHLKIQVMVTTGGTSLKDDIVRLYQPVHLLVGTPGRILDLTKKGVCILKDCSMLVMDEADKLLSPEFQPSIEQLIRYLP

ASRQILMFSATFPVTVKEFKDKYLPKPYVINLMDELTLKGITQFYAFVEERQKVHCLNTLFSKLQINQSIIFCNSVNRVE

LLAKKITELGYSCFYIHAKMLQDHRNRVFHDFRNGACRNLVCTDLFTRGIDIQAVNVVINFDFPKSSETYLHRVGRSGRF

GHLGLAVNLITYEDRFNLYRIEQELGTEIKPIPPQIDQAIYCQ

>XP_031482679.1 DEAD-box ATP-dependent RNA helicase 8-like [Nymphaea colorata]

MNSRARYPPPGIREGPGGPANMNQGQDPQWRGSQQQYVQRGLLQYQQQQQQQQHQQWLRRQASVEAGASTAENSIGVQPD

LTMIQQQSGIKSSSQDWKAQLKIPPPDTRYRTEDVTATKGNEFEDYFLKRELLMGIYEKGFERPSPIQEESIPIALTGSD

ILARAKNGTGKTAAFCIPALEKIDQEKNAIQVVILVPTRELALQTSQVCKDLGKHLKIQVMVTTGGTSLKDDIMRLYQPV

HLLVGTPGRILDLVKKGVCILKDCTMLAMDEADKLLSPEFQPSVEQLISFLPPTRQILMFSATFPITVKDFKDRYLRKPY

VINLMDELTLKGITQFYAFVEERQKVHCLNTLFSKLQINQSIIFCNSVTRVELLAKKITELGYSCFYIHARMLQAHRNRV

FHDFRNGACRNLVCTDLFTRGIDIQAVNVVINFDFPKNSETYLHRVGRSGRFGHLGLAVNLITFEDRFNLYRIEQELGTE

IRPIPPQIDQALYCS

>VAH34226.1 unnamed protein product [Triticum turgidum subsp. durum]

MCTVLHTTPQDWKAQLKLPPPDTRYQTEDVTATKGNEFEDYFLKRELLMGIYEKGFERPSPIQEESIPIALTGSDILARA

KNGTGKTAAFCIPALEKIDQDKNAIQVVILVPTRELALQTSQVCKELGKHLKIQVMVTTGGTSLKDDIVRLYQPVHLLVG

TPGRVLDLTKKGICILKDCSMLIMDEADKLLSPEFQPSVEHLIRYLPSSRQILMFSATFPVTVKAFKDKYLPKPYVINLM

DELTLKGITQFYAFVEERQKVHCLNTLFSKLQINQSIIFCNSVNRVELLAKKITELGYSCFYIHAKMLQDHRNRVFHDFR

NGACRNLVCTDLFTRGIDIQAVNVVINFDFPKTAETYLHRVGRSGRFGHLGLAVNLITYEDRFNLYRIEQELGTEIKPIP

PQIDRTIYCQ

>XP_009142369.1 DEAD-box ATP-dependent RNA helicase 6 [Brassica rapa]

MDNNNNRGRFPPGIGAPDPNFQSRNPNPNPNPPPQQFLHSRTPFPQQYVQSRTPLPPQQQPDAQQYVQRGYSQQNPPQQI

QQQQQWSTRAQLPGNPSYVDEVEKTVQSEANNDSNNQDWKATLKLPPRDNRYQTEDVTATKGNEFEDYFLKRDLLRGIYE

KGFEKPSPIQEESIPIALTGSDILARAKNGTGKTGAFCIPTLEKIDPENNVIQAVILVPTRELALQTSQVCKELSKYLKI

EVMVTTGGTSLRDDIMRLYQPVHLLVGTPGRILDLTKKGVCVLKDCAMLVMDEADKLLSAEFQPSIEELIQFLPQNRQIL

MFSATFPVTVKYFKDRYLRKPYIINLMDQLTLMGVTQYYAFVEERQKVHCLNTLFSKLQINQSIIFCNSVNRVELLAKKI

TELGYSCFYIHAKMAQDHRNRVFHDFRNGACRNLVCTDLFTRGIDIQAVNVVINFDFPRTSESYLHRVGRSGRYGHLGLA

VNLVTYEDRFKMYQTEQELGTEIKPIPSLIDKAIYCQ

>XP_013688265.1 DEAD-box ATP-dependent RNA helicase 6-like isoform X1 [Brassica napus]

MDNNSSRGRFPPGIGAPDPNFQSRNPNPNPNPPPQQFLHSRTPFPQQYVQSRTPLPDAQQYVQRGYPQQNPPQQIQQQPQ

WSTRAQLPGDSSYVEEVEKTVQSEANSNNQDWKATLKLPPRDNRYQTEDVTATKGNEFEDYFLKRDLLRGIYEKGFEKPS

PIQEESIPIALTGSDILARAKNGTGKTGAFCIPTLEKIDPENNVIQAVILVPTRELALQTSQVCKELSKYLKIEVMVTTG

GTSLRDDIMRLYQPVHLLVGTPGRILDLTKKGVCVLKDCAMLVMDEADKLLSAEFQPSIEELIQFLPQNRQILMFSATFP

VTVKYFKDRYLRKPYIINLMDQLTLMGVTQYYAFVEERQKVHCLNTLFSKLQINQSIIFCNSVNRVELLAKKITELGYSC

FYIHAKMAQDHRNRVFHDFRNGACRNLVCTDLFTRGIDIQAVNVVINFDFPRTSESYLHRVGRSGRYGHLGLAVNLVTYE

DRFKMYQTEQELGTEIKPIPSLIDKAIYCQ

>XP_013688267.1 DEAD-box ATP-dependent RNA helicase 6-like isoform X2 [Brassica napus]

MDNNSSRGRFPPGIGAPDPNFQSRNPNPNPNPPPQQFLHSRTPFPQQYVQSRTPLPDAQQYVQRGYPQQNPPQQIQQQPQ

WSTRAQLPGDSSYVEEVEKTVQSEANNNQDWKATLKLPPRDNRYQTEDVTATKGNEFEDYFLKRDLLRGIYEKGFEKPSP

IQEESIPIALTGSDILARAKNGTGKTGAFCIPTLEKIDPENNVIQAVILVPTRELALQTSQVCKELSKYLKIEVMVTTGG

TSLRDDIMRLYQPVHLLVGTPGRILDLTKKGVCVLKDCAMLVMDEADKLLSAEFQPSIEELIQFLPQNRQILMFSATFPV

TVKYFKDRYLRKPYIINLMDQLTLMGVTQYYAFVEERQKVHCLNTLFSKLQINQSIIFCNSVNRVELLAKKITELGYSCF

YIHAKMAQDHRNRVFHDFRNGACRNLVCTDLFTRGIDIQAVNVVINFDFPRTSESYLHRVGRSGRYGHLGLAVNLVTYED

RFKMYQTEQELGTEIKPIPSLIDKAIYCQ

>VAH34225.1 unnamed protein product [Triticum turgidum subsp. durum]

MSPQDWKAQLKLPPPDTRYQTEDVTATKGNEFEDYFLKRELLMGIYEKGFERPSPIQEESIPIALTGSDILARAKNGTGK

TAAFCIPALEKIDQDKNAIQVVILVPTRELALQTSQVCKELGKHLKIQVMVTTGGTSLKDDIVRLYQPVHLLVGTPGRVL

DLTKKGICILKDCSMLIMDEADKLLSPEFQPSVEHLIRYLPSSRQILMFSATFPVTVKAFKDKYLPKPYVINLMDELTLK

GITQFYAFVEERQKVHCLNTLFSKLQINQSIIFCNSVNRVELLAKKITELGYSCFYIHAKMLQDHRNRVFHDFRNGACRN

LVCTDLFTRGIDIQAVNVVINFDFPKTAETYLHRVGRSGRFGHLGLAVNLITYEDRFNLYRIEQELGTEIKPIPPQIDRT

IYCQ

>XP_027081480.1 DEAD-box ATP-dependent RNA helicase 8-like [Coffea arabica]

MNSRGRYPPGIWNGRGGGGAGGFGTVNANPNFQNRNPTYYHHQQQQQQFQQNYGQRNLQNQQHHHHFQQQQQWMRRNPGG

TPSDSSVNEVEKTVQPVTPDSGSQDWKARLKLPPPDTRYKTEDVTATKGNEFEDYFLKRELLMGIYEKGFERPSPIQEES

IPIALTGSDILARAKNGTGKTAAFCIPALEKIDTDYNVIQVVILVPTRELALQTSQVCKELGKHLKIQVMVSTGGTNLKD

DIMRLYQPVHLLVGTPGRVLDLTQKGVCILKDCAMLVMDEADKLLSPEFQPSLEQLIAFLPVNRQILMFSATFPVTVKEF

KDRYLKKPYVVNLMDELTLKGITQYYAFVEERQKVHCLNTLFSKLQINQSIIFCNSVNRVELLAKKITELGYSCFYIHAK

MLQDHRNRVFHDFRNGACRNLVCTDLFTRGIDIQAVNVVINFDFPKTSETYLHRVGRSGRFGHLGLAVNLITYEDRFNLY

RIEQELGTEIKQIPPLIDQAIYCR

>KAF3795544.1 DEAD-box ATP-dependent RNA helicase 8 [Nymphaea thermarum]

MNSRGRYAPGAANGRGGYGGAHQQQQNYVQRGSQFHQQQQWLRRSPAVPETDVGAAEGNMQHEEEKRVQSEAVESSSQDW

KAQLKLPPIDTRYKTEDVTATKGNEFEDYFLKRELLMGIYEKGFERPSPIQEESIPIALTGSNILARAKNGTGKTAAFCI

PALEKIDQNKNAIQVIILVPTRELALQTSQVCKELGKHLKVQIMVTTGGTSLKDDIMRLYQPVHLLVGTPGRILDLVKKG

VCILKDCSMLVMDEADKLLSQEFQPLVEQLISFLPTSRQILLFSATFPVTVKAFKDRFLLKPYVINLMDELTLKGITQYY

AFVEERQKVHCLNTLFSKLQINQSIIFCNSVNRVELLAKKITELGYSCFYIHAKMLQSHRNRVFHDFRNGACRNLVCSDL

FTRGIDIQAVNVVINFDFPKNSETYLHRVGRSGRFGHLGLAVNLITYEDRFNLYRIEQELGTEIKVIPVQIDQAIYCR

>EEC73681.1 hypothetical protein OsI_08237 [Oryza sativa Indica Group]

MDPRARYPPGIGNGRGGNPNYYNRGPPLQQHHHHNHHQQHQQQQQQWLRRNQIAREAAGTDRNSEPKAVAQSPAVDGIDS

SSQDWKAQLKLPPQDTRYRTEDVTATKGNEFEDYFLKRELLMGIYEKGFERPSPIQEESIPIALTGSDILARAKNGTGKT

AAFCIPALEKIDQEKNAIQVVILVPTRELALQTSQVCKELGKHLKIQVMVTTGGTSLKDDIIRLYQPVHLLVGTPGRILD

LTKKGICILKDCSMLIMDEADKLLSPEFQPSVEQLIRYLPASRQILMFSATFPVTVKEFKDKYLPKPYVINLMDELTLKG

ITQFYAFVEERQKVHCLNTLFSKLQINQSIIFCNSVNRVELLAKKITELGYSCFYIHAKMLQDHRNRVFHDFRNGACRNL

VCTDLFTRGIDIQAVNVVINFDFPKTAETYLHRVGRSGRFGHLGLAVNLITYEDRFNLYRIEQELGTEIKPIPPQIDQAI

YCQ

>PTQ47051.1 hypothetical protein MARPO_0009s0142 [Marchantia polymorpha]

MVYSRGAQQHYQPRPLGGGGSHGRGQSGGSYQPYQPHTQRAIHQNNHQQQLQQQQQQQQQQQQQQQQQQQNQQQSQLSGQ

HLALSQAQANVSTSAAPSADGGQGGHLAQGNSSLQDWKAQLKLPPTDSRYRTEDVTATKGNEFEDYFLKRELLMGIYEKG

FERPSPIQEESIPIALTGSDILARAKNGTGKTAAFCIPAIEKIDQNKNAIQVLILVPTRELALQTSQVCKELAKHLHIQV

MVTTGGTSLKDDIMRLYQPVHLLVGTPGRVLDLARKGVCNLGECTMLVMDEADKLLSPEFQPLVEQLIAFLPDNRQTLLY

SATFPVTVKSFKDRFLRKPYVINLMDELTLKGITQFYAFVEERQKVHCLSTLFSKLQINQSIIFCNSVNRVELLAKKITE

LGYSCFYVHAKMLQSHRNRVFHDFRNGACRNLVSSDLFTRGIDIQAVNVVINFDFPKNSETYLHRVGRSGRFGHLGLAVN

LITYEDRFNLYRIEQELGTEIKPIPPQVDQTIYCR

>XP_015627069.1 DEAD-box ATP-dependent RNA helicase 8 [Oryza sativa Japonica Group]

MDPRARYPPGIGNGRGGNPNYYNRGPPLQQQHNHHQQQQTSAPHHQQYVQRQPQQHHHHNHHQQHQQQQQQWLRRNQIAR

EAAGTDRNSEPKAVAQSPAVDGIDSSSQDWKAQLKLPPQDTRYRTEDVTATKGNEFEDYFLKRELLMGIYEKGFERPSPI

QEESIPIALTGSDILARAKNGTGKTAAFCIPALEKIDQEKNAIQVVILVPTRELALQTSQVCKELGKHLKIQVMVTTGGT

SLKDDIIRLYQPVHLLVGTPGRILDLTKKGICILKDCSMLIMDEADKLLSPEFQPSVEQLIRYLPASRQILMFSATFPVT

VKEFKDKYLPKPYVINLMDELTLKGITQFYAFVEERQKVHCLNTLFSKLQINQSIIFCNSVNRVELLAKKITELGYSCFY

IHAKMLQDHRNRVFHDFRNGACRNLVCTDLFTRGIDIQAVNVVINFDFPKTAETYLHRVGRSGRFGHLGLAVNLITYEDR

FNLYRIEQELGTEIKPIPPQIDQAIYCQ

>VDD16467.1 unnamed protein product [Brassica oleracea]

MDNNSSRGRFPPGIGAPDPNFQSRNPNPNPNPPPQQFLHSRTPFPQQYAQSRTPLPDAQQYVQRGYTQQNPPQQIQQQQQ

WSTRAQLPGDSSYVEEVEKTVQSEANSNQDWKATLKLPPRDNRYQTEDVTATKGNEFEDYFLKRDLLRGIYEKGFEKPSP

IQEESIPIALTGSDILARAKNGTGKTGAFCIPTLEKIDPENNVIQAVILVPTRELALQTSQVCKELSKYLKIEVMVTTGG

TSLRDDIMRLYQPVHLLVGTPGRILDLTKKGVCVLKDCAMLVMDEADKLLSAEFQPSIEELIQFLPQNRQILMFSATFPV

TVKYFKDRYLRKPYIINLMDQLTLMGVTQYYAFVEERQKVHCLNTLFSKLQINQSIIFCNSVNRVELLAKKITELGYSCF

YIHAKMAQDHRNRVFHDFRNGACRNLVCTDLFTRGIDIQAVNVVINFDFPRTSESYLHRVGRSGRYGHLGLAVNLVTYED

RFKMYQTEQELGTEIKPIPSLIDKAIYCQ

>XP_013749666.1 DEAD-box ATP-dependent RNA helicase 6 [Brassica napus]

MDNNNNRGRFPPGIGAQNPNPNPNPPPQQFLHSRTPFPQQYVQSRTPLPPQQQPDAQQYVQRGYSQQNPPQQIQQQQQWS

TRAQLPGNPSYVDEVEKTVQSEANNDSNNQDWKATLKLPPRDNRYQTEDVTATKGNEFEDYFLKRDLLRGIYEKGFEKPS

PIQEESIPIALTGSDILARAKNGTGKTGAFCIPTLEKIDPENNVIQAVILVPTRELALQTSQVCKELSKYLKIEVMVTTG

GTSLRDDIMRLYQPVHLLVGTPGRILDLTKKGVCVLKDCAMLVMDEADKLLSAEFQPSIEELIQFLPQNRQILMFSATFP

VTVKYFKDRYLRKPYIINLMDQLTLMGVTQYYAFVEERQKVHCLNTLFSKLQINQSIIFCNSVNRVELLAKKITELGYSC

FYIHAKMAQDHRNRVFHDFRNGACRNLVCTDLFTRGIDIQAVNVVINFDFPRTSESYLHRVGRSGRYGHLGLAVNLVTYE

DRFKMYQTEQELGTEIKPIPSLIDKAIYCQ

>ESQ39221.1 hypothetical protein EUTSA_v10001405mg [Eutrema salsugineum]

MLTSNQDWKAMLKLPPRDNRYQTEDVTATKGNEFEDYFLKRDLLRGIYEKGFEKPSPIQEESIPIALTGSDILARAKNGT

GKTGAFCIPTLEKIDPENNVIQAVILVPTRELALQTSQVCKELSKYLKIEVMVTTGGTSLRDDIMRLYQPVHLLVGTPGR

ILDLAKKGVCVLKDCTMLVMDEADKLLSAEFQPSIEELIQFLPQNRQILMFSATFPVTVKSFKDRYLRKPYIINLMDQLT

LMGVTQYYAFVEERQKVHCLNTLFSKLQINQSIIFCNSVNRVELLAKKITELGYSCFYIHAKMVQDHRNRVFHDFRNGAC

RNLVCTDLFTRGIDIQAVNVVINFDFPRTSESYLHRVGRSGRYGHLGLAVNLVTYEDRFKMYQTEQELGTEIKPIPSLID

KAIYCQ

>CDY27357.1 BnaC04g50480D [Brassica napus]

MDNNSSRGRFPPGIGAPDPNFQSRNPNPNPNPPPQQFLHSRTPFPQQYVQSRTPLPDAQQYVQRGYPQQNPPQQIQQQPQ

WSTRAQLPGDSSYVEEVEKTVQSEANSNQDWKATLKLPPRDNRYQTEDVTATKGNEFEDYFLKRDLLRGIYEKGFEKPSP

IQEESIPIALTGSDILARAKNGTGKTGAFCIPTLEKIDPENNVIQAVILVPTRELALQTSQVCKELSKYLKIEVMVTTGG

TSLRDDIMRLYQPVHLLVGTPGRILDLTKKGVCVLKDCAMLVMDEADKLLSAEFQPSIEELIQFLPQNRQILMFSATFPV

TVKYFKDRYLRKPYIINLMDQLTLMGVTQYYAFVEERQKVHCLNTLFSKLQINQSIIFCNSVNRVELLAKKITELGYSCF

YIHAKMAQDHRNRVFHDFRNGACRNLVCTDLFTRGIDIQAVNVVINFDFPRTSESYLHRVGRSGRYGHLGLAVNLVTYED

RFKMYQTEQELGTEIKPIPSLIDKAIYCQ

>KAE8807131.1 DEAD-box ATP-dependent RNA helicase 8 [Hordeum vulgare]

MDPRARYPPGTGNGRGGNPNYYGRGPPLSQNNHHHQQQTSAAHQQQYVQRQPQPQRQQHHQDHHHHQQQHHQNHHQQQQQ

QQQQQQWLRRNQITAAGTSGPKVVAPPPAAAGNDSSSQDWKAQLKLPPADTRFRTEDVTATKGNEFEDYFLKRELLMGIY

EKGFEKPSPIQEESIPIALTGSDILARAKNGTGKTAAFCIPALEKIDQDRNAIQVVIVVPTRELALQTSQVCKELGKHLK

IQVMVTTGGTSLKDDIVRLYQPVHLLVGTPGRILDLTKKGVCILKDCSMLVMDEADKLLSPEFQPSIEQLIRYLPASRQI

LMFSATFPVTVKEFKDKYLPKPYVINLMDELTLKGITQFYAFVEERQKVHCLNTLFSKLQINQSIIFCNSVNRVELLAKK

ITELGYSCFYIHAKMLQDHRNRVFHDFRNGACRNLVCTDLFTRGIDIQAVNVVINFDFPKSSETYLHRVGRSGRFGHLGL

AVNLITYEDRFNLYRIEQELGTEIKPIPPQIDQAIYCQ

>XP_027085068.1 DEAD-box ATP-dependent RNA helicase 8-like [Coffea arabica]

MNSRGRYPPGIWNGRGGGGAGGFGTVNANPNFQNRNPTYYHHQQQQQQFQQNYGQRNLQNQQHHHHHFQQQQQQHWMRRN

PSGTPSDSSVNEVEKTVQPVNPDSGSQDWKARLKLPPPDTRYKTEDVTATKGNEFEDYFLKRELLMGIYEKGFERPSPIQ

EESIPIALTGSDILARAKNGTGKTAAFCIPALEKIDTDYNVIQVVILVPTRELALQTSQVCKELGKHLKIQVMVSTGGTN

LKDDIMRLYQPVHLLVGTPGRILDLTQKGVCILKDCAMLVMDEADKLLSPEFQPSLEQLIAFLPVNRQILMFSATFPVTV

KEFKDRYLKKPYVINLMDELTLKGITQYYAFVEERQKVHCLNTLFSKLQINQSIIFCNSVNRVELLAKKITELGYSCFYI

HAKMLQDHRNRVFHDFRNGACRNLVCTDLFTRGIDIQAVNVVINFDFPKTSETYLHRVGRSGRFGHLGLAVNLITYEDRF

NLYRIEQELGTEIKQIPPLIDQAIYCR

>CDP09769.1 unnamed protein product [Coffea canephora]

MNSRGRYPPGIWNGRGGGGAGGFGTVNANPNFQNRNPTYYHHQQQQQQFQQNYGQRNLQNQQHHHHFQQQQQWMRRNPSG

TPSDSSVNEVEKTVQPVNPDSGSQDWKARLKLPPPDTRYKTEDVTATKGNEFEDYFLKRELLMGIYEKGFERPSPIQEES

IPIALTGSDILARAKNGTGKTAAFCIPALEKIDTDYNVIQVVILVPTRELALQTSQVCKELGKHLKIQVMVSTGGTNLKD

DIMRLYQPVHLLVGTPGRILDLTQKGVCILKDCAMLVMDEADKLLSPEFQPSLEQLIAFLPVNRQILMFSATFPVTVKEF

KDRYLKKPYVINLMDELTLKGITQYYAFVEERQKVHCLNTLFSKLQINQSIIFCNSVNRVELLAKKITELGYSCFYIHAK

MLQDHRNRVFHDFRNGACRNLVCTDLFTRGIDIQAVNVVINFDFPKTSETYLHRVGRSGRFGHLGLAVNLITYEDRFNLY

RIEQELGTEIKQIPPLIDQAIYCR

>GER30369.1 ATP-dependent RNA helicase DHH1 [Striga asiatica]

MSNNYNRGRYPPGIGNGRGGGSGDGVGVGNTNQNYQSRNPHYPQPQPPYQQRTAQNQPQQWMRRNPNAAVVSDSSNEVEK

TVQPDAAGSTSKDWKARLAIPPPDTRYKTEDVTATKGNEFEDYFLKRDLLMGIYEKGFERPSPIQEESIPIALTGSDILA

RAKNGTGKTAAFCIPALEKIDSDKNVIQVVILVPTRELALQTSQVCKELGKHLKIQVMVSTGGTSLKDDIMRLYQPVHLL

VGTPGRILDLTKKGICVLKDCSVLVMDEADKLLSPEFQPSIEQLIAFLPPNRQILMYSATFPVTVKDFKDKYLRKPYIIN

LMDELTLKGITQYYAFVEERQKVHCLNTLFSKLQINQSIIFCNSVNRVELLAKKITELGYSCFYIHAKMLQDHRNRVFHD

FRNGACRNLVCTDLFTRGIDIQAVNVVINFDFPKNSETYLHRVGRSGRFGHLGLAVNLITYEDRFNLYRIEQELGTEIKQ

IPPHIDQAIYCFGVWMGSHQDNCRKSLTLPVIGLGTVIFVVSVIGFLGALKNNSILLWMYLILLCLILVAILVFTVLAFI

VTNNGSGHNVTGLRYKEYQLHDYSSWFLNQLNNTENWEHLKSCLVKSEDCNNLAIKYKMAPKRKLGSDAGEPSNLTRITR

SAARTVSRSTRSGAVLGDAPTVNAAFSDPEPRRTKKPKTAADGPSSGAKTLIIEHCVASGLFKNKAAQVKKALEKAVSGV

NIVINPEKPRNGCFEIREEGGDIFVSLLDLESPFEQVKALNVSKVVSDIADRIK

>XP_003623902.2 DEAD-box ATP-dependent RNA helicase 8 [Medicago truncatula]

MNNNNRGRYPPGIGLGRGSGSGSGGGGGGLNSNPNNANAGFQQRPHYQQQQQQQYVQRHMMQNQNQHQQHYQHHQQNQQQ

YQQQQQQQQQWLRRNQLGGGTDTNVVEEVEKTVQSETNDPSSQDWKARLKIPAADTRYRTEDVTATKGNEFEDYFLKREL

LMGIYEKGFERPSPIQEESIPIALTGSDILARAKNGTGKTAAFSIPALEKIDQDKNVIQVVILVPTRELALQTSQVCKEL

GKHLQIQVMVTTGGTSLKDDIMRLYQPVHLLVGTPGRILDLAKKGVCVLKDCSMLVMDEADKLLSPEFKPSIEQLIQFLP

SNRQILLFSATFPVTVKDFNDRYLRKPYIINLMDELTLKGITQFYAFVEERQKVHCLNTLFSKLQINQSIIFCNSVNRVE

LLAKKITELGYSCFYIHAKMLQDHRNRVFHDFRNGACRNLVCTDLFTRGIDIQAVNVVINFDFPKNSETYLHRVGRSGRF

GHLGLAVNLVTYEDRFNLYRIEQELGTEIKQIPPFIDQAIYCR

>KAF3578969.1 hypothetical protein DY000_02036054, partial [Brassica cretica]

XNPPPQQFLHSRTPFPQQYVHSRTPLPDAQQYVQRGYTQPQQIQQQPQWSTRAQLPGDSSYVEEVEKTVQSEANNQDWKA

TLKLPPRDNRYQTEDVTATKGNEFEDYFLKRDLLRGIYEKGFEKPSPIQEESIPIALTGSDILARAKNGTGKTGAFCIPT

LEKIDPENNVIQAVILVPTRELALQTSQVCKELSKYLKIEVMVTTGGTSLRDDIMRLYQPVHLLVGTPGRILDLTKKGVC

VLKDCAMLVMDEADKLLSAEFQPSIEELIQFLPQNRQILMFSATFPVTVKYFKDRYLRKPYIINLMDQLTLMGVTQYYAF

VEERQKVHCLNTLFSKLQINQSIIFCNSVNRVELLAKKITELGYSCFYIHAKMAQDHRNRVFHDFRNGACRNLVCTDLFT

RGIDIQAVNVVINFDFPRTSESYLHRVGRSGRYGHLGLAVNLVTYEDRFKMYQTEQELGTEIKPIPSLIDKAIYCQ

>NP_001148960.2 ATP-dependent RNA helicase dhh1 [Zea mays]

MDPRARYPSGVGNGRGGTPNYYGRGPSPQQPHQHHQQQQQQKQTSGAHHHQQYAQRQQQQHRHSHNHQQQQQQHQSHQQQ

QQQQQWLRRNQIAREAAGAAVTSEPKALAPSTAADGVNSSSQDWKAQLKLPPPDTRYRTEDVTATKGNEFEDYFLKRELL

MGIYEKGFERPSPIQEESIPIALTGSDILARAKNGTGKTAAFCIPALEKIDQEKNAIQVVILVPTRELALQTSQVCKELG

KHLKIQVMVTTGGTSLKDDIVRLYQPVHLIVGTPGRILDLTKKGVCILKDCSMLIMDEADKLLSPEFQPSIEQLIRYLPA

SRQILMFSATFPVTVKEFKDKYLPKPYVINLMDELTLKGITQFYAFVEERQKVHCLNTLFSKLQINQSIIFCNSVNRVEL

LAKKITELGYSCFYIHAKMLQDHRNRVFHDFRNGACRNLVCTDLFTRGIDIQAVNVVINFDFPKNSETYLHRVGRSGRFG

HLGLAVNLITYEDRFNLYRIEQELGTEIKPIPPQIDQAIYCQ

>XP_015636229.1 DEAD-box ATP-dependent RNA helicase 6 [Oryza sativa Japonica Group]

MDPRARYPPGIGNGRGGNPNYYGRGPPPSQHQQHQHQHQQPPHPHHHQYVQRQPQPQQTPHNSQHQQWLRRNQIAAEAAG

ASEQKAPPVADGIDSSSQDWKAQLKLPPQDTRYRTEDVTATKGNEFEDYFLKRELLMGIYEKGFERPSPIQEESIPIALT

GSDILARAKNGTGKTAAFCIPALEKIDQDKNAIQVVILVPTRELALQTSQVCKELGKHLKIQVMVTTGGTSLKDDIVRLY

QPVHLLVGTPGRILDLTKKGVCVLKNCSMLVMDEADKLLSPEFQPSIQELIRYLPSNRQILMFSATFPVTVKEFKDKYLP

KPYVINLMDELTLKGITQFYAFVEERQKVHCLNTLFSKLQINQSIIFCNSVNRVELLAKKITELGYSCFYIHAKMLQDHR

NRVFHDFRNGACRNLVCTDLFTRGIDIQAVNVVINFDFPKSAETYLHRVGRSGRFGHLGLAVNLITYEDRFNLYRIEQEL

GTEIKPIPPQIDRAIYCQ

>ONK64101.1 uncharacterized protein A4U43_C07F22090 [Asparagus officinalis]

MNSLILTAAISGGESCSFVLGSHINLRKEVFGAELPRSPFRGIRGDWLKSLKRSGGGSGYRLNVTARIKKAQKHEYPWPD

NIDPNIESGHLSYLSHFKPLKEKPKPVTLPFEKPLLDLEKKIIDVRKMANETGLDFTDQINLLESKYQEALKELYTHLTP

IQRLTIARHPNRPTFLDHILNITDKWVELHGDRAGYDDPAIVTGIGSIDGKTYMFIGHQKGRNTKENIQRNFGMPTPHGY

RKALRMMRYADHHGFPIITFIDTPGAFADLKSEELGQGEAIAYNLRAMFGLKVPIVTVVIGEGGSGGALAIGCANKLFMM

DNSVFYVASPEACAAILWKSSQAAPKAAEKLKITANELCRLKIADGIIPEPLGGAHTDPAWTSQQIKLTVTKAMEELCEM

DTDSLLNHRHLKFRNLGGFLEGSPIEPEKKRNMKKKEADISQATADIEVEIEKLKKAALEGKSQSPGPVISNEAVEKLKQ

DLDKEMTNAFISMGLQEKLESLKMELSKSPSNSPDQSLSPALKEKADRLMQEFKHNLSRPGSYLGLKQKLQMLSEVNKLG

EHKIKAERLKSEINQKLHEQVKGKTEILKMARDRVAKGEKLNEDEIKEVNMIQEELKEMLKSLNLEVVGLRKKILPTKPP

VLEEKLTKADEVIKMEIAKAIDAAGLNSKIDELKTEIANGASKETVEKLINEIREGIIANLDSAQLKEKVASTLELPLQE

TVGAGNHSPDWKSQLKIPPPDTRYRTEDVTATKGNEFEDYFLKRELLMGIYEKGFERPSPIQEESIPIALTGSDILARAK

NGTGKTAAFCIPALEKIDQDKNVIQVVILVPTRELALQTSQVCKELGKHLKIQVMATTGGTSLKDDIMRLYQPVHLLVGT

PGRILDLTKKGVCVLKDCSMLIMDEADKLLSHEFQPSIEQLIHFLPSNRQILLFSATFPVTVKDFKDRYLPKPYIINLMD

ELTLKGITQYYAFVEERQKVHCLNTLFSKLQINQSIIFCNSVNRVELLAKKITELGYSCFYIHAKMLQDHRNRVFHDFRN

GACRNLVCTDLFTRGIDIQAVNVVINFDFPKNSETYLHRVGRSGRYGHLGLAVNLITYEDRFNLYRIEQELGTEIKQIPP

QIDQAIYCR

>XP_004976343.1 DEAD-box ATP-dependent RNA helicase 6 [Setaria italica]

MDPRARYPPGIGNGRGGNPNYYGRGPPPQQQQHHHQQPPPPSQAYHQQYVQRQPQPQPSQHLSQQQQQQQWLRRRQIAGE

AAGAGAQKATPAVDGIDSSSQDWKAQLKLPPQDTRYRTEDVTATKGNEFEDYFLKRELLMGIYEKGFERPSPIQEESIPI

ALTGSDILARAKNGTGKTAAFCIPALEKIDQDKNAIQVVILVPTRELALQTSQVCKELGKHLKIQVMVTTGGTSLKDDII

RLYQPVHLLVGTPGRILDLTKKGICVLKDCSMLIMDEADKLLSPEFQPSIEQLIRYLPSNRQILMFSATFPVTVKEFKDK

YLPKPYVINLMDELTLKGITQFYAFVEERQKVHCLNTLFSKLQINQSIIFCNSVNRVELLAKKITELGYSCFYIHAKMLQ

DHRNRVFHDFRNGACRNLVCTDLFTRGIDIQAVNVVINFDFPKNAETYLHRVGRSGRFGHLGLAVNLITYEDRFNLYRIE

QELGTEIKPIPPQIDRAIYCQ

>VAH34228.1 unnamed protein product [Triticum turgidum subsp. durum]

MDQRARYPPGIGNGRGGNPNYHGRGPPPTQQHQHQQQQQAQGHQQQHMERQSQRSQHHAPAAADDADLSPQDWKAQLKLP

PPDTRYQTEDVTATKGNEFEDYFLKRELLMGIYEKGFERPSPIQEESIPIALTGSDILARAKNGTGKTAAFCIPALEKID

QDKNAIQVVILVPTRELALQTSQVCKELGKHLKIQVMVTTGGTSLKDDIVRLYQPVHLLVGTPGRVLDLTKKGICILKDC

SMLIMDEADKLLSPEFQPSVEHLIRYLPSSRQILMFSATFPVTVKAFKDKYLPKPYVINLMDELTLKGITQFYAFVEERQ

KVHCLNTLFSKLQINQSIIFCNSVNRVELLAKKITELGYSCFYIHAKMLQDHRNRVFHDFRNGACRNLVCTDLFTRGIDI

QAVNVVINFDFPKTAETYLHRVGRSGRFGHLGLAVNLITYEDRFNLYRIEQELGTEIKPIPPQIDRTIYCQ

>GAU49575.1 hypothetical protein TSUD_179890 [Trifolium subterraneum]

MNNRGRYPPGMGLGRGGGGGGGLNSNTNTGYQQRPHQQQQQQYVQRHMVQNQHPQQYHQQNQQNHQYQQQQQQQQQQWLR

RNQLGGTDTNNVEEVEKTVQSEANDTSSQDWKARLKLPPVDTRYRTEDVTATKGNEFEDYFLKRELLMGIYEKGFERPSP

IQEESIPIALTGSDILARAKNGTGKTAAFCIPALEKIDQDNNIIQVVILVPTRELALQTSQVCKELGKHLQIQVMVTTGG

TSLKDDIMRLYQPVHLLVGTPGRILDLAKKGVCVLKDCSMLVMDEADKLLSQEFQPSIEQLIQFLPSNRQILMFSATFPV

TVKDFNDRYLRKPYIINLMDELTLKGITQFYAFVEERQKVHCLNTLFSKLQINQSIIFCNSVNRVELLAKKITELGYSCF

YIHAKMLQDHRNRVFHDFRNGACRNLVCTDLFTRGIDIQAVNVVINFDFPKNSETYLHRVGRSGRFGHLGLAVNLITYED

RFNLYRIEQELGTEIKQIPPFIDQAIYCR

>XP_020883116.1 DEAD-box ATP-dependent RNA helicase 6 [Arabidopsis lyrata subsp. lyrata]

MNNNRGRFPPGIGAAGPDPNFQSRNPNPSPHQSQPQQYLQSRTPFPQQPQTQPPQYLQSQPDVQQYVQRGYPQQIQQQQQ

QQQQQQQQQWSRRPQLPGDPSYVDEVEKTVQSEAISDSNNEDWKATLKLPPRDNRYQTEDVTATKGNEFEDYFLKRDLLR

GIYEKGFEKPSPIQEESIPIALTGSDILARAKNGTGKTGAFCIPTLEKIDPENNVIQAVILVPTRELALQTSQVCKELSK

YLKIEVMVTTGGTSLRDDIMRLYQPVHLLVGTPGRILDLAKKGVCVLKDCAMLVMDEADKLLSVEFQPSIEELIQFLPEN

RQILMFSATFPVTVKSFKDRYLRKPYIINLMDQLTLMGVTQYYAFVEERQKVHCLNTLFSKLQINQSIIFCNSVNRVELL

AKKITELGYSCFYIHAKMVQDHRNRVFHDFRNGACRNLVCTDLFTRGIDIQAVNVVINFDFPRTSESYLHRVGRSGRFGH

LGLAVNLVTYEDRFKMYQTEQELGTEIKPIPSLIDKAIYCQ

>XP_013648432.1 DEAD-box ATP-dependent RNA helicase 12-like [Brassica napus]

MNNNRGRYPPGNGTGRGAPPHPDYQPYGQQPQNHQQFQQQQQWRRAQLPGNANEVQNTTSSQPPVPSNDHKFSGSVTLSG

GQDWKATLKLPPPDTRYQTADVTATKGNEFEDYFLKRDLLKGIYEKGFEKPSPIQEESIPIALTGSDILARAKNGTGKTG

AFCIPLLEKIDPNTNVIQAMILVPTRELALQTSQVCKELSKYLNIQVMVTTGGTSLRDDIMRLHQPVHLLVGTPGRILDL

TKKGVCVLKDCTMLVMDEADKLLSAEFQPSLEELIQFLPENRQFLMFSATFPVTVKAFKDRHLRKPYVINLMDQLTLMGV

TQYYAFVEERQKVHCLNTLFSKLQINQSIIFCNSVNRVELLAKKITELGYSCFYIHAKMVQDHRNRVFHEFRNGACRNLV

CTDLFTRGIDIQAVNVVINFDFPRTSESYLHRVGRSGRFGHLGLAVNLVTYEDRFKMYQTEQELGTEIKPIPSQIDQAIY

CQ

>VDD27663.1 unnamed protein product [Brassica oleracea]

MNNNNNRGRYPPGIGAGRGAINPNPNFQSRPGYQQQPPPQYVQRGGYPAQQNHQQQFQQATSHQQQQQWLRRPQISGGNS

NGDAVAEVEKSVLSQAIDTNSEDWKARLKLPAPDTRFRTEDVTATKGNEFEDYFLKRELLMGIYEKGFERPSPIQEESIP

IALTGRDILARAKNGTGKTAAFCIPVLEKIDQDNNVIQALIIVPTRELALQTSQVCKELGKHLKIQVMVTTGGTSLKDDI

MRLYQPVHLLVGTPGRILDLTKKGVCVLKDCSVFVMDEADKLLSQEFQPSVEHLISFLPQNRQILMFSATFPVTVKDFKD

RFLTNPYIINLMDELTLKGITQFYAFVEERQKIHCLNTLFSKLQINQSIIFCNSVNRVELLAKKITELGYSCFYIHAKML

QDHRNRVFHDFRNGACRNLVCTDLFTRGIDIQAVNVVINFDFPKTAETYLHRVGRSGRFGHLGLAVNLITYEDRFNLYRI

EQELGTEIKQIPPHIDQAIYCQ

>ACG33648.1 ATP-dependent RNA helicase dhh1 [Zea mays]

MDPRARYPSDMGNGRGGTPNYYGRGPPPQQPHQHHHQQQQQTSGAHHHQQYAQRQQQQHRHSHNHQQQQQQHQSQQQQQQ

WLRRNQIAREAAGAAVTSEPKALAPSTAADGVNSSSQDWKTQLKLPPPDTRYRTEDVTATKGNEFEDYFLKRELLMGIYE

KGFERPSPIQEESIPIALTGSDILARAKNGTGKTAAFCIPALEKIDQEKNAIQVVILVPTRELALQTSQVCKELGKHLKI

QVMVTTGGTSLKDDIVRLYQPVHLIVGTPGRILDLTKKGVCILKDCSMLIMDEADKLLSPEFQPSIEQLIRYLPASRQIL

MFSATFPVTVKEFKDKYLPKPYVINLMDELTLKGITQFYAFVEERQKVHCLNTLFSKLQINQSIIFCNSVNRVELLAKKI

TELGYSCFYIHAKMLQDHRNRVFHDFRNGACRNLVCTDLFTRGIDIQAVNVVINFDFPKNSETYLHRVGRSGRFGHLGLA

VNLITYEDRFNLYRIEQELGTEIKPIPPQIDQAIYCQ

>ACG29146.1 ATP-dependent RNA helicase dhh1 [Zea mays]

MDPRARYPSGMGNGRGGTPNYYGRGPPPQQPHQHHHQQQQQTSGAHHHQQYAQRQQQQHRHSHNHQQQQQQHQSQQQQQQ

WLRRNQIAREAAGAAVTSEPKALAPSTAADGVNSSSQDWKTQLKLPPPDTRYRTEDVTATKGNEFEDYFLKRELLMGIYE

KGFERPSPIQEESIPIALTGSDILARAKNGTGKTAAFCIPALEKIDQEKNAIQVVILVPTRELALQTSQVCKELGKHLKI

QVMVTTGGTSLKDDIVRLYQPVHLIVGTPGRILDLTKKGVCILKDCSMLIMDEADKLLSPEFQPSIEQLIRYLPASRQIL

MFSATFPVTVKEFKDKYLPKPYVINLMDELTLKGITQFYAFVEERQKVHCLNTLFSKLQINQSIIFCNSVNRVELLAKKI

TELGYSCFYIHAKMLQDHRNRVFHDFRNGACRNLVCTDLFTRGIDIQAVNVVINFDFPKNSETYLHRVGRSGRFGHLGLA

VNLITYEDRFNLYRIEQELGTEIKPIPPQIDQAIYCQ

>RAL44388.1 hypothetical protein DM860_011665 [Cuscuta australis]

MVAAAAEGAVTEGAAVTVGVEEATVTAGVEEAVVTVGGGGGSGYGGGGFRSSQNSGGYNQPRNHHYQYTQQQQQPPQPQQ

YGQRALQNQSQQQQQWLRRNPSGSMSESSSKEILKTVQSEAIDSSSQDWKARLNIPPPDTRYQTEDVTATKGNEFEDYFL

KRELLMGIYEKGFERPSPIQEESIPIALTGSDILARAKNGTGKTAAFCIPALERIDPEINAIQVVILVPTRELALQTSQV

CKELGKHLKIQVMVSTGGTSLKDDIMRLHQPVHLLVGTPGRILDLAKKGICVLKECTMLAMDEADKLLSPEFQPSVEHLI

SFLPEHRQILMFSATFPVTVKDFKDRYLKKPYIINLMDELTLKGITQFYAFVEERQKVHCLNTLFSKLQINQSIIFCNSV

NRVELLARKITELGYSCFYIHAKMLQDHRNKVFHDFRNGACRNLVCTDLFTRGIDIQAVNVVINFDFPKNSETYLHRVGR

SGRFGHLGLAVNLITYEDRFNLHKIEQELGTEIKPIPPHIDQAIYCA

>VDC63145.1 unnamed protein product [Brassica rapa]

MNNNRGRYQPGTGTGRGAYQQQPQAQQHVQRGQPQNHQQQQWSRRAQLPGNATNANEVQTTSSQPPVASSDSNGQDWKAS

LRLPPPDTRYQTADVTATKGNEFEDYFLKRDLLKGIYEKGFEKPSPIQEESIPIALTGSDILARAKNGTGKTGAFCIPVL

ERIDPNTNVIQAMILVPTRELALQTSQVCKELSKYLNIHVMVTTGGTSLRDDIMRLHQPVHLLVGTPGRILDLTKKGVCV

LKDCTMLVMDEADKLLSAEFQPSLEELIQFLPQNRQFLMYSATFPVTVKAFKERHLRKPYVINLMDQLTLMGVTQYYAFV

EERQKVHCLNTLFSKLQINQSIIFCNSVNRVELLAKKITELGYSCFYIHAKMVQDHRNRVFHEFRNGACRNLVCTDLFTR

GIDIQAVNVVINFDFPRTSESYLHRVGRSGRFGHLGLAVNLVTYEDRFKMYQTEQELGTEIKPIPSNIDQAIYCQ

>XP_006652567.1 PREDICTED: LOW QUALITY PROTEIN: DEAD-box ATP-dependent RNA helicase 6-like [Oryza brachyantha]

MDPLARYPPGIGNGRGGNPNYYGRGPPPPPQQQHQHQHQHQQPPQPHHHQYVQRQHARRAPPPQPQQTQNTSQHQQWLRR

NQITNEAAGASEQKAPPVPDGIDSSSQDWKAQLKLPPQDTRYRTEDVTATKGNEFEDYFLKRELLMGIYEKGFERPSPIQ

EESIPIALTGSDILARAKNGTGKTAAFCIPALEKIDQDKNAIQVVILVPTRELALQTSQVCKELGKHLKIQVMVTTGGTS

LKDDIVRLYQPVHLLVGTPGRILDLTKKGVCILKNCSMLVMDEADKLLSPEFQPSVEQLIRYLPSNRQILMFSATFPVTV

KDFKDKYLPKPYVINLMDELTLKGITQFYAFVEERQKVHCLNTLFSKLQINQSIIFCNSVNRVELLAKKITELGYSCFYI

HAKMLQDHRNRVFHDFRNGACRNLVCTDLFTRGIDIQAVNVVINFDFPKNAETYLHRVGRSGRFGHLGLAVNLITYEDRF

NLYRIEQELGTEIKPIPPQIDRAIYCQ

>XP_006848084.1 DEAD-box ATP-dependent RNA helicase 8 isoform X1 [Amborella trichopoda]

MNPRYATGPGNGRFSQQTYVQRIPNSQSQQQFQQQVWMRRNESSPIEDGNQNEEEKRVQSEVVDPSSQDWKAQLKIPPVD

TRYKTEDVTATKGNEFEDYFLKRELLMGIYEKGFERPSPIQEESIPIALTGSDILARAKNGTGKTAAFCIPALEKIDQNK

NAIQVIILVPTRELALQTSQVCKELGKHLNIQVMVTTGGTSLKDDIMRLYQPVHLLVGTPGRVLDLTKKGVCVLKECTML

VMDEADKLLSPEFQPSVEQLIGFLPPNRQILLFSATFPVTVKAFKERFLRRPYIINLMDELTLKGITQYYAFVEERQKVH

CLNTLFSKLQINQSIIFCNSVNRVELLAKKITELGYSCFYIHAKMLQSHRNRVFHDFRNGACRNLVCSDLFTRGIDIQAV

NVVINFDFPKNSETYLHRVGRSGRFGHLGLAVNLITYEDRFNLYRIEQELGTEIKQIPPQIDQTIYCR

>KAF7002587.1 hypothetical protein CFC21_018059 [Triticum aestivum]

MDQRARYPPGIGNGRGGNPNYHGRGPPPTQQHQHQQQQQAQGHQQQHMERQSQRSQHHNQQLQHQQWLRRNQTASEAAAG

AAKASEHHAPAAADDADLSPQDWKAQLKLPPPDTRYQTEDVTATKGNEFEDYFLKRELLMGIYEKGFERPSPIQEESIPI

ALTGSDILARAKNGTGKTAAFCIPALEKIDQDKNAIQVVILVPTRELALQTSQVCKELGKHLKIQVMVTTGGTSLKDDIV

RLYQPVHLLVGTPGRVLDLTKKGICILKDCSMLIMDEADKLLSPEFQPSVEHLIRYLPSSRQILMFSATFPVTVKAFKDK

YLPKPYVINLMDELTLKGITQFYAFVEERQKVHCLNTLFSKLQINQSIIFCNSVNRVELLAKKITELGYSCFYIHAKMLQ

DHRNRVFHDFRNGACRNLVCTDLFTRGIDIQAVNVVINFDFPKTAETYLHRVGRSGRFGHLGLAVNLITYEDRFNLYRIE

QELGTEIKPIPPQIDRTIYCQ

>XP_018479123.1 PREDICTED: DEAD-box ATP-dependent RNA helicase 6 [Raphanus sativus]

MDNNNNNRGRFPPGIGAPDPNFQSRNPNPNPPPQQQYLHSRTPFPQQYVQSRTPLPPHQQQQPDAQQYVQRGHTQQNPPP

HQIQQQQQQQWSTRAQLPGNPSYVDEVEKTVQSEANNDSNNQDWKATLKLPPRDNRYQTEDVTATKGNEFEDYFLKRDLL

RGIYEKGFEKPSPIQEESIPIALTGSDILARAKNGTGKTGAFCIPTLEKIDPENNIIQAVILVPTRELALQTSQVCKELS

KYLNIEVMVTTGGTSLRDDIMRLYQPVHLLVGTPGRILDLTKKGVCVLKDCAMLVMDEADKLLSAEFQPSIEELIQFLPQ

NRQILMFSATFPVTVKYFKDRYLRKPYIINLMDQLTLMGVTQYYAFVEERQKVHCLNTLFSKLQINQSIIFCNSVNRVEL

LAKKITELGYSCFYIHAKMAQDHRNRVFHDFRNGACRNLVCTDLFTRGIDIQAVNVVINFDFPRTSESYLHRVGRSGRYG

HLGLAVNLVTYEDRFKMYQTEQELGTEIKPIPSLIDKAIYCQ

>PUZ48179.1 hypothetical protein GQ55_7G225100 [Panicum hallii var. hallii]

MDPRARYPPGIGNGRGGNPNYYGRGPPPPPQQQHHHHQPPPPSQAHHQQYMQRQPQPQPQPSQHLNQQQQQQQWHRRNQI

AAEAAGASAQRAPPAVDGIDSSSQDWKAQLKLPPPDTRYRTEDVTATKGNEFEDYFLKRELLMGIYEKGFEKPSPIQEES

IPIALTGSDILARAKNGTGKTAAFCIPALEKIDQDKNAIQVVILVPTRELALQTSQVCKELGKHLKIQVMVTTGGTSLKD

DIIRLYQPVHLLVGTPGRILDLTKKGICMLKDCSMLIMDEADKLLSPEFQPSIEQLIRYLPSNRQILMFSATFPVTVKEF

KDKYLPKPYVINLMDELTLKGITQFYAFVEERQKVHCLNTLFSKLQINQSIIFCNSVNRVELLAKKITELGYSCFYIHAK

MLQDHRNRVFHDFRNGACRNLVCTDLFTRGIDIQAVNVVINFDFPKNAETYLHRVGRSGRFGHLGLAVNLITYEDRFNLY

RIEQELGTEIKPIPPQIDRTIYCQ

>KAE9615581.1 putative RNA helicase [Lupinus albus]

MNNSNNNNNRPRYPPGIGYGRGGGGFTQNPNQNEPFQPRSSYQHQQQHYVQRNFVPQQQQQQQQQQQQQQWLRRVQLGGG

ESNHNDEVEKNVQTEADDSSSQDWKARLKAPPADTRYKTEDVTATKGNEFEDYFLKRELLMGIYEKGFERPSPIQEESIP

IALTGSDILARAKNGTGKTAAFCIPALEKIDQDTNVIQAVILVPTRELALQTSQVCKELGKHLKIEVMVTTGGTSLKDDI

MRLYQPVHLLVGTPGRILDLAKKGVCILKDCSMLVMDEADKLLSPEFQPSIQQLIQFLPSNRQILMFSATFPVTVKDFKD

RYLQKPYVINLMDELTLKGITQYYAFVEERQKVHCLNTLFSKLQINQSIIFCNSVNRVELLAKKITELGYSCFYIHAKML

QDHRNRVFHDFRNGACRNLVCTDLFTRGIDIQAVNVVINFDFPKNSETYLHRVGRSGRFGHLGLAVNLITYEDRFNLYRI

EQELGTEIKQIPPFIDQAVYCR

>AAK63966.1 At2g45810/F4I18.21 [Arabidopsis thaliana]

MNNNNNNRGRFPPGIGAAGPGPDPNFQSRNPNPPQPQQYLQSRTPFPQQPQPQPPQYLQSQSDAQQYVQRGYPQQIQQQQ

QLQQQQQQQQQQQEQQWSRRAQLPGDPSYIDEVEKTVQSEAISDSNNEDWKATLKLPPRDNRYQTEDVTATKGNEFEDYL

LKRDLLRGIYEKGFEKPSPIQEESIPIALTGSDILARAKNGTGKTGAFCIPTLEKIDPENNVIQAVILVPTRELALQTSQ

VCKELSKYLKIEVMVTTGGTSLRDDIMRLYQPVHLLVGTPGRILDLAKKGVCVLKDCAMLVMDEADKLLSVEFQPSIEEL

IQFLPESRQILMFSATFPVTVKSFKDRYLKKPYIINLMDQLTLMGVTQYYAFVEERQKVHCLNTLFSKLQINQSIIFCNS

VNRVELLAKKITELGYSCFYIHAKMVQDHRNRVFHDFRNGACRNLVCTDLFTRGIDIQAVNVVINFDFPRTSESYLHRVG

RSGRFGHLGLAVNLVTYEDRFKMYQTEQELGTEIKPIPSLIDKAIYCQ

>KAF7090181.1 hypothetical protein CFC21_092977 [Triticum aestivum]

MDPRARYPPGTGNGRGGNPNYYGRGPPLSQNNHHHHQQTSGAHQQQYVQRQPQPQPQQHHQNNHQQQHHQNNHQQQHHQN

HHHHHQQQQQQWLRRNQINAAGTSGPKVVGPPPAAVGNDPSSQDWKAQLKLPPADTRFRTEDVTATKGNEFEDYFLKREL

LMGIYEKGFENPSPIQEESIPIALTGSDILARAKNGTGKTAAFCIPALEKIDQDKNAIQVVIVVPTRELALQTSQVCKEL

GKHLKIQVMVTTGGTSLKDDIVRLYQPVHLLVGTPGRILDLTKKGVCILKDCSMLVMDEADKLLSPEFQPSIEQLIRYLP

ASRQILMFSATFPVTVKEFKDKYLPKPYVINLMDELTLKGITQFYAFVEERQKVHCLNTLFSKLQINQSIIFCNSVNRVE

LLAKKITELGYSCFYIHAKMLQDHRNRVFHDFRNGACRNLVCTDLFTRGIDIQAVNVVINFDFPKSSETYLHRVGRSGRF

GHLGLAVNLITYEDRFNLYRIEQELGTEIKPIPPQIDQAIYCQ

>XP_013712536.1 DEAD-box ATP-dependent RNA helicase 12 [Brassica napus]

MNNNRGRYQPGTGTGRGAYQQQPQAQQHVQRGQPQNHQQQQWSRRAQLPGNATNANEIQTSSSQPPVASSDSNGQDWKAS

LRLPPPDTRYQTADVTATKGNEFEDYFLKRDLLKGIYEKGFEKPSPIQEESIPIALTGSDILARAKNGTGKTGAFCIPVL

ERIDPNTNVIQAMILVPTRELALQTSQVCKELSKYLNIHVMVTTGGTSLRDDIMRLHQPVHLLVGTPGRILDLTKKGVCV

LKDCTMLVMDEADKLLSAEFQPSLEELIQFLPQNRQFLMYSATFPVTVKAFKERHLRKPYVINLMDQLTLMGVTQYYAFV

EERQKVHCLNTLFSKLQINQSIIFCNSVNRVELLAKKITELGYSCFYIHAKMVQDHRNRVFHEFRNGACRNLVCTDLFTR

GIDIQAVNVVINFDFPRTSESYLHRVGRSGRFGHLGLAVNLVTYEDRFKMYQTEQELGTEIKPIPSNIDQAIYCQ

>XP_019413830.1 PREDICTED: DEAD-box ATP-dependent RNA helicase 8-like [Lupinus angustifolius]

MNNTNNNNNRPRYPPGIGYGRGGGGFTQNPNQNEPFQPRSSYQHQQQHYGQRNFVPQQQQQQQQQQHQQQQHQQQQQQWL

RRAQLGGGESNLNDEVEKNVQTEADDSSSQDWKARLKAPPADTRYKTEDVTATKGNEFEDYFLKRELLMGIYEKGFERPS

PIQEESIPIALTGSDILARAKNGTGKTAAFCIPALEKIDQDTNVIQAVILVPTRELALQTSQVCKELGKHLKIEVMVTTG

GTSLKDDIMRLYQPVHLLVGTPGRILDLAKKGVCILKDCSMLVMDEADKLLSPEFQPSIQQLIQFLPSNRQILMFSATFP

VTVKDFKDRYLQKPYVINLMDELTLKGITQYYAFVEERQKVHCLNTLFSKLQINQSIIFCNSVNRVELLAKKITELGYSC

FYIHAKMLQDHRNRVFHDFRNGACRNLVCTDLFTRGIDIQAVNVVINFDFPKNSETYLHRVGRSGRFGHLGLAVNLITYE

DRFNLYRIEQELGTEIKQIPPFIDQAVYCR

>XP_020584221.1 DEAD-box ATP-dependent RNA helicase 8-like [Phalaenopsis equestris]

MNARGSYPSGIGNGRGGSAEPSANVYPRGSRIHNQYVERNPTQSQQNQQFQQQQQQQQWLRRNQMERRSGSSELVKSVSS

DAIDSSSQDWKAQLRIPTADTRYKTEDVTATKGNDFEDYFLKRELLMGIYEKGFESPSPIQEESIPIALTGRDILARAKN

GTGKTAAFCIPALEKIDQEKNVIQVIILVPTRELALQTSQVCKELSKHLKVQVMVTTGGTSLKDDIMRLYQPVHLISGTP

GRILDLAKKGVCNLKDCSMLIMDEADKLLSPEFQPSIEQLMRFLPENRQILLFSATFPLSVKDFKDRFLRKPYIINLMDE

LTLKGITQYYAFVEERQKVHCLNTLFSKLQINQSIIFCNSVNRVELLAKKITELGYSCFYIHAKMLQDHRNRVFHDFRNG

ACRNLVCTDLFTRGIDIQAVNVVINFDFPKNSETYLHRVGRSGRFGHLGLAVSLITYEDRFNLYRIEQELGTEIKQIPPQ

IDQAIYCS

>XP_025823960.1 DEAD-box ATP-dependent RNA helicase 6 [Panicum hallii]

MDPRARYPPGIGNGRGGNPNYYGRGPPPPQQQQHHHHQPPPPSQAHHQQYMQRQPQPQPQPSQHLNQQQQQQQQQWHRRN

QIAAEAAGASAQRAPPAVDGIDSSSQDWKAQLKLPPPDTRYRTEDVTATKGNEFEDYFLKRELLMGIYEKGFEKPSPIQE

ESIPIALTGSDILARAKNGTGKTAAFCIPALEKIDQDKNAIQVVILVPTRELALQTSQVCKELGKHLKIQVMVTTGGTSL

KDDIIRLYQPVHLLVGTPGRILDLTKKGICMLKDCSMLIMDEADKLLSPEFQPSIEQLIRYLPSNRQILMFSATFPVTVK

EFKDKYLPKPYVINLMDELTLKGITQFYAFVEERQKVHCLNTLFSKLQINQSIIFCNSVNRVELLAKKITELGYSCFYIH

AKMLQDHRNRVFHDFRNGACRNLVCTDLFTRGIDIQAVNVVINFDFPKNAETYLHRVGRSGRFGHLGLAVNLITYEDRFN

LYRIEQELGTEIKPIPPQIDRTIYCQ

>XP_010544383.1 PREDICTED: DEAD-box ATP-dependent RNA helicase 8-like [Tarenaya hassleriana]

MNTRGRYPPGIGAGRGAVNPNNPGFLSRPVYQQQQPQPPPPHYVQRGNYSQNHQQFHQAPQQQQQQQQQQWLRRAQFAGS

NNNSGSSGNNNGVAVDEVEKTVQSEAIDLNSQDWKARLKLPPPDTRYRTEDVTATKGNEFEDYFLKRELLMGIYEKGFER

PSPIQEESIPIALTGSDILARAKNGTGKTAAFCIPALEKIDQDNNVIQVVILVPTRELALQTSQVCKELGKHLKIQVMVT

TGGTSLKDDIMRLYQPVHFLVGTPGRILDLAKKGVCVLKNCSMLVMDEADKLLSQEFQPSVEQLIRFLPENRQILMYSAT

FPVNVKDFKDRYLRKPYIINLMDELTLKGITQFYAFVEERQKVHCLNTLFSKLQINQSIIFCNSVNRVELLAKKITELGY

SCFYIHAKMLQDHRNRVFHDFRNGACRNLVCTDLFTRGIDIQAVNVVINFDFPKNSETYLHRVGRSGRFGHLGLAVNLIT

YEDRFNLYRIEQELGTEIKQIPPHIDQAIYCQ

>XP_025828643.1 DEAD-box ATP-dependent RNA helicase 8 [Panicum hallii]

MDPRARYPPGMGNGRGGNPNYYGRGPPPHQPHQHNYQHQQTSGAHHHQQYAQRQQQQHHQNHNHNHHQQQQQHHNHHQQQ

QQQQWLRRNQIAREAVGAAGTSEPKALVPSTAADGVDSSSQDWKAQLKLPPQDTRYRTEDVTATKGNEFEDYFLKRELLM

GIYEKGFERPSPIQEESIPIALTGSDILARAKNGTGKTAAFCIPALEKIDPDNNAIQVVILVPTRELALQTSQVCKELGK

HLKIQVMVTTGGTSLKDDIVRLYQPVHLLVGTPGRILDLTKKGVCILKDCSMLIMDEADKLLSPEFQPSVEQLIRYLPAS

RQILMFSATFPVTVKEFKDKYLPKPYVINLMDELTLKGITQFYAFVEERQKVHCLNTLFSKLQINQSIIFCNSVNRVELL

AKKITELGYSCFYIHAKMLQDHRNRVFHDFRNGACRNLVCTDLFTRGIDIQAVNVVINFDFPKNSETYLHRVGRSGRFGH

LGLAVNLITYEDRFNLYRIEQELGTEIKPIPPQIDQAIYCQ

>XP_006396262.1 DEAD-box ATP-dependent RNA helicase 8 [Eutrema salsugineum]

MNNNNNNNRGRYPPGIGAGRGAFNPNPNFQSRPTYQQQPPPQYVQRGGYAQQNHQQQFQQATSQPHQQHQYQQQQQQQWL

RRPQISAGNSNGDAVVEVEKTVQSEAIDTNSEDWKARLKLPAPDTRYRTEDVTATKGNEFEDYFLKRELLMGIYEKGFER

PSPIQEESIPIALTGRDILARAKNGTGKTAAFCIPVLEKIDQDNNVIQAVIIVPTRELALQTSQVCKELGKHLKIQVMVT

TGGTSLKDDIMRLYQPVHLLVGTPGRILDLAKKGVCVLKDCSVLVMDEADKLLSQEFQPSVEHLISFLPQNRQILMFSAT

FPVTVKDFKDRFLTNPYIINLMDELTLKGITQFYAFVEERQKIHCLNTLFSKLQINQSIIFCNSVNRVELLAKKITELGY

SCFYIHAKMLQDHRNRVFHDFRNGACRNLVCTDLFTRGIDIQAVNVVINFDFPKNAETYLHRVGRSGRFGHLGLAVNLIT

YEDRFNLYRIEQELGTEIKQIPPHIDQAIYCQ

>XP_024010708.1 DEAD-box ATP-dependent RNA helicase 6 [Eutrema salsugineum]

MNNNNNNTGRFPPGIGAAAPDPNFQSRNPNPQQPPPQQYLHSRTPFPQTQQSRPPNPQQPDAHHYVQRAYPQNPQQIQQL

QQQQQWSTHPQLPANPSYVDEVEKTVQSEAHTNDSNNQDWKAMLKLPPRDNRYQTEDVTATKGNEFEDYFLKRDLLRGIY

EKGFEKPSPIQEESIPIALTGSDILARAKNGTGKTGAFCIPTLEKIDPENNVIQAVILVPTRELALQTSQVCKELSKYLK

IEVMVTTGGTSLRDDIMRLYQPVHLLVGTPGRILDLAKKGVCVLKDCTMLVMDEADKLLSAEFQPSIEELIQFLPQNRQI

LMFSATFPVTVKSFKDRYLRKPYIINLMDQLTLMGVTQYYAFVEERQKVHCLNTLFSKLQINQSIIFCNSVNRVELLAKK

ITELGYSCFYIHAKMVQDHRNRVFHDFRNGACRNLVCTDLFTRGIDIQAVNVVINFDFPRTSESYLHRVGRSGRYGHLGL

AVNLVTYEDRFKMYQTEQELGTEIKPIPSLIDKAIYCQ

>KAE8785102.1 DEAD-box ATP-dependent RNA helicase 6 [Hordeum vulgare]

MDQRASSQDWKAQLKLPPPDTRYQTEDVTATKGNEFEDYFLKRELLMGIYEKGFERPSPIQEESIPIALTGSDILARAKN

GTGKTAAFCIPALEKIDQDKNAIQVVILVPTRELALQTSQVCKELGKHLKIQVMVTTGGTSLKDDIVRLYQPVHLLVGTP

GRVLDLTKKGICILKDCSMLIMDEADKLLSPEFQPSVEQLIRYLPSSRQILMFSATFPVTVKAFKDKYLPKPYVINLMDE

LTLKGITQFYAFVEERQKVHCLNTLFSKLQINQSIIFCNSVNRVELLAKKITELGYSCFYIHAKMLQDHRNRVFHDFRNG

ACRNLVCTDLFTRGIDIQAVNVVINFDFPKNAETYLHRVGRSGRFGHLGLAVNLITYEDRFNLYRIEQELGTEIKPIPPQ

IDRTIYCQ

>CAA7017373.1 unnamed protein product [Microthlaspi erraticum]

MNNNNRGRYPPGIGAGRGAFNPNPNFQSRPGYQQQPPPQYVQRGGYQQQQNHQQQFQQATSQPHQYQQQHQQQQQQQWLR

RPQISGGNSNGDAVVEVEKTVQSEAIDTNSEDWKARLKLPAPDTRFRTEDVTATKGNEFEDYFLKRELLMGIYEKGFERP

SPIQEESIPIALTGRDILARAKNGTGKTAAFCIPVLEKIDQDNNVIQAVIIVPTRELALQTSQVCKELGKHLKIQVMVTT

GGTSLKDDIMRLYQPVHLLVGTPGRILDLAKKGVCVLKDCSVLVMDEADKLLSQEFQPSVEHLISFLPETRQILMFSATF

PVTVKDFKDRFLTNPYIINLMDELTLKGITQFYAFVEERQKIHCLNTLFSKLQINQSIIFCNSVNRVELLAKKITELGYS

CFYIHAKMLQDHRNRVFHDFRNGACRNLVCTDLFTRGIDIQAVNVVINFDFPKNAETYLHRVGRSGRFGHLGLAVNLITY

EDRFNLYRIEQELGTEIKQIPPHIDQAIYCQ

>TEY72195.1 ATP-dependent RNA helicase DDX6/DHH1 [Salvia splendens]

MSSRARYPPPGMGGGRGGGGGMNPNAGPNPSFQPRNPMHQYVQRSPAPSNQNQQMYQQQQWLRRNQLPPSDSAVDEVEKT

VQSEAIDSSSQDWKARLKLPPRDNRYRTEDVTATKGNEFEDYFLKRELLMGIYEKGFESPSPIQEESIPIALTGSDILAR

AKNGTGKTAAFCIPALEKIDQDKNAIQVVILVPTRELALQTSQVCKELGKHLQIQVMATTGGTSLKDDIMRLYQPVHLLV

GTPGRILDLANKNVCVLNECSMLVMDEADKLLSPEFQPSIEQLIRFMPTSRQILMFSATFPVTVKDFKERYLRKPYIINL

MDELTLKGITQFYAFVEERQKVHCLNTLFSKLQINQSIIFCNSVNRVELLAKKITELGYSCFYIHAKMLQDHRNRVFHDF

RNGACRNLVCTDLFTRGIDIQAVNVVINFDFPKNSETYLHRVGRSGRYGHLGLAVNLITYEDRFNLYRIEQELGTEIKQI

PPQIDQAVYCQ

>PIN27223.1 ATP-dependent RNA helicase [Handroanthus impetiginosus]

MNNNYARGRYPPGMGNGWGGGGGGGGTPNQNYQNRNPHYPQLQPPYRQRTAESQPQQWMRRNPSASTVSESANEVEKTVQ

SEAAGSTSQDWKGRPMIPPADRRYKTEDVTATKGNEFEDYFLKRELLLGIYEKGFEKPSPIQEESIPIALTGSDILARAK

NGTGKTAAFCIPALEKIDSDKNLIQAVILVPTRELALQTSQVCKELGKHLKIQVMVTTGGTSLKDDIMRLYQPVHLLVGT

PGRILDLTRKGICNLNECSMLVMDEADKLLSPEFQPSIEQLISFLPPNRQMLMYSATFPVTVKDFKDKYLRKPYIINLMD

ELTLKGITQYYAFVEERQKVHCLNTLFSKLQINQSIIFCNSVNRVELLAKKITELGYSCFYIHAKMLQDHRNKVFHEFRN

GACRNLVCTDLFTRGIDIQAVNVVINFDFPKNSETYLHRVGRSGRFGHLGLAVNLITYEDRFNLYRIEQELGAEIKQIPS

HIDHAIYCM

>KAF3796225.1 DEAD-box ATP-dependent RNA helicase 8 [Nymphaea thermarum]

MIQQQSGIKSSAQDWKAHLKIPPPDTRYRTEDVTATKGNEFEDYFLKRELLMGIYEKGFERPSPIQEESIPIALTGSDIL

ARAKNGTGKTAAFCVPALEKIDQEKNAIQVVILVPTRELALQTSQVCKDLGKHLKIQVMVTTGGTSLKDDIMRLYQPVHL

LVGTPGRILDLVKKGVCILKDCTMLAMDEADKLLSPEFQPSVEQLISFLPPTRQILMFSATFPITVKDFKDRYLRKPYVI

NLMDELTLKGITQFYAFVEERQKVHCLNTLFSKLQINQSIIFCNSVTRVELLAKKITELGYSCFYIHARMLQAHRNRVFH

DFRNGACRNLVCTDLFTRGIDIQAVNVVINFDFPKNSETYLHRVGRSGRFGHLGLAVNLITFEDRFNLYRIEQELGTEIR

PIPPQIDQTLYCS

>KAF1884126.1 hypothetical protein Lal_00046413 [Lupinus albus]

MIPIKISFLISDFVSIATPFEEIIISRGMNNRARYPPGIGLGRGGGSGGGGGGGGSGLNPNPGFQQRPPQQHVQRHIMQQ

QQYQQQQQQQQQQQQQQWLRRTQLGGSANTNVVEEVEKTVQSEAVDESSQDWKTKLKIPPADTRYRTEDVTATKGNEFED

YFLKRELLMGIYEKGFERPSPIQEESIPIALTGSDILARAKNGTGKTAAFCIPALEKIDQDNNVIQVVILVPTRELALQT

SQVCKELGKHLKIQVMVTTGGTSLKDDIMRLYQPVHLLVGTPGRILDLARKGVCVMKDCSMLVMDEADKLLSPEFQPSIE

QLIQFLPGNRQILMFSATFPVTVKDFKDRYLRKPYVINLMDELTLKGITQFYAFVEERQKVHCLNTLFSKLQINQSIIFC

NSVNRVELLAKKITELGYSCFYIHAKMLQDHRNRVFHDFRNGACRNLVCTDLFTRGIDIQAVNVVINFDFPKNSETYLHR

VGRSGRFGHLGLAVNLITYEDRFNLYRIEQELGTEIKQIPPHIDQAIYCHLASSAAF

>CDY58787.1 BnaA07g38020D [Brassica napus]

MNNNRGRYPPGNGTGRGAPPHPDYQPYGQQPQNHQQFQQQQQWRRAQLPGNANEVQNTTSSQPPVPSNDHKFSGSVTLSG

GQDWKATLKLPPPDTRYQTADVTATKGNEFEDYFLKRDLLKGIYEKGFEKPSPIQEESIPIALTGSDILARAKNGTGKTG

AFCIPLLEKIDPNTNVIQAMILVPTRELALQTSQVCKELSKYLNIQVMVTTGGTSLRDDIMRLHQPVHLLVGTPGRILDL

TKKGVCVLKDCTMLVMDEADKLLSAEFQPSLEELIQFLPENRQLLMFSATFPVTVKAFKDRHLRKPYVINLMDQLTLMGV

TQYYAFVEERQKVHCLNTLFSKLQINQSIIFCNSVNRVELLAKKITELGYSCFYIHAKMVQDHRNRVFHEFRNGACRNLV

CTDLFTRGIDIQAVNVVINFDFPRTSESYLHRVGRSGRFGHLGLAVNLVTYEDRFKMCQTEQELGTEIKPIPSQIDQAIY

CQ

>PUZ76779.1 hypothetical protein GQ55_1G317400 [Panicum hallii var. hallii]

MDPRARYPPGMGNGRGGNPNYYGRGPPPHQPHQHNYQHQQTSGAHHHQQYAQRQQQQHHQNHNHNHHQQQQQHHNHHQQQ

QQQQWLRRNQIAREAVGAAGTSEPKALVPSTAADGVDSSSQDWKAQLKLPPQDTRYRTEDVTATKGNEFEDYFLKRELLM

GIYEKGFERPSPIQEESIPIALTGSDILARAKNGTGKTAAFCIPALEKIDPDNNTIQVVILVPTRELALQTSQVCKELGK

HLKIQVMVTTGGTSLKDDIVRLYQPVHLLVGTPGRILDLTKKGVCILKDCSMLIMDEADKLLSPEFQPSVEQLIRYLPAS

RQILMFSATFPVTVKEFKDKYLPKPYVINLMDELTLKGITQFYAFVEERQKVHCLNTLFSKLQINQSIIFCNSVNRVELL

AKKITELGYSCFYIHAKMLQDHRNRVFHDFRNGACRNLVCTDLFTRGIDIQAVNVVINFDFPKNSETYLHRVGRSGRFGH

LGLAVNLITYEDRFNLYRIEQELGTEIKPIPPQIDQAIYCQ

>OAP10408.1 hypothetical protein AXX17_AT2G43410 [Arabidopsis thaliana]

MNNNNNNRGRFPPGIGAAGPGPDPNFQSRNPNPPQPQQYLQSRTPFPQQPQPPQYLQSQSDAQQYVQRGYPQQIQQQQQL

QQQQQQQQQQQEQQWSRRAQLPGDPSYIDEVEKTVQSEAISDSNNEDWKATLKLPPRDNRYQTEDVTATKGNEFEDYFLK

RDLLRGIYEKGFEKPSPIQEESIPIALTGSDILARAKNGTGKTGAFCIPTLEKIDPENNVIQAVILVPTRELALQTSQVC

KELSKYLKIEVMVTTGGTSLRDDIMRLYQPVHLLVGTPGRILDLAKKGVCVLKDCAMLVMDEADKLLSVEFQPSIEELIQ

FLPESRQILMFSATFPVTVKSFKDRYLKKPYIINLMDQLTLMGVTQYYAFVEERQKVHCLNTLFSKLQINQSIIFCNSVN

RVELLAKKITELGYSCFYIHAKMVQDHRNRVFHDFRNGACRNLVCTDLFTRGIDIQAVNVVINFDFPRTSESYLHRVGRS

GRFGHLGLAVNLVTYEDRFKMYQTEQELGTEIKPIPSLIDKAIYCQ

>KAF5196896.1 Atp-dependent rna helicase dhh1 [Thalictrum thalictroides]

MNTRGRYPPGIGYGRGGYANQNPNFQPRNPQQQYVQRNSMPNQQQYQQQQQQQQQQQQQQQQWLRRNPMSGDPGVNEVEK

TVQSETIDSSAQDWKARLKIPPSDTRYRTEDVTATKGNEFEDYFLKRELLMGIYEKGFERPSPIQEESIPIALTGSDILA

RAKNGTGKTAAFCIPALEKIDQENNVIQVVILVPTRELALQTSQVCKELGKHLKIQVMVTTGGTSLKDDIMRLYQPVHLL

VGTPGRILDLAKKGVCVLKDCSMLVMDEADKLLSPEFQPSVEQLIRFLPASRQILLFSATFPVTVKDFKDRYLQKPYVIN

LMDELTLKGITQYYAFVEERQKVHCLNTLFSKLQINQSIIFCNSVNRVELLAKKITELGYSCFYIHAKMLQDHRNRVFHD

FRNGACRNLVCTDLFTRGIDIQAVNVVINFDFPKNSETYLHRVGRSGRFGHLGLAVNLITYEDRFNLYRIEQELGTEIKQ

IPPQIDQAIYCR

>NP_182105.1 DEA(D/H)-box RNA helicase family protein [Arabidopsis thaliana]

MNNNNNNRGRFPPGIGAAGPGPDPNFQSRNPNPPQPQQYLQSRTPFPQQPQPQPPQYLQSQSDAQQYVQRGYPQQIQQQQ

QLQQQQQQQQQQQEQQWSRRAQLPGDPSYIDEVEKTVQSEAISDSNNEDWKATLKLPPRDNRYQTEDVTATKGNEFEDYF

LKRDLLRGIYEKGFEKPSPIQEESIPIALTGSDILARAKNGTGKTGAFCIPTLEKIDPENNVIQAVILVPTRELALQTSQ

VCKELSKYLKIEVMVTTGGTSLRDDIMRLYQPVHLLVGTPGRILDLAKKGVCVLKDCAMLVMDEADKLLSVEFQPSIEEL

IQFLPESRQILMFSATFPVTVKSFKDRYLKKPYIINLMDQLTLMGVTQYYAFVEERQKVHCLNTLFSKLQINQSIIFCNS

VNRVELLAKKITELGYSCFYIHAKMVQDHRNRVFHDFRNGACRNLVCTDLFTRGIDIQAVNVVINFDFPRTSESYLHRVG

RSGRFGHLGLAVNLVTYEDRFKMYQTEQELGTEIKPIPSLIDKAIYCQ

>KZV26732.1 DEAD-box ATP-dependent RNA helicase 8-like [Dorcoceras hygrometricum]

MGGGRGGGMHSNVGMDPSFQPRNPAQHYVQSGPAPYNKLNNQLFQNPQPQQWLPRTQLTLADANIEVEKTVQSEAADSSS

QDWKASLKLPPQDTRFKTEDVTATKGNEFEDYFLKRELLMGIYEKGFERPSPIQEESIPIALTGSDILARAKNGTGKTAA

FCIPALEKIDQDKNAIQVVILVPTRELALQTSQVCKELGKHLKIQVMVTTGGTSLRDDIMRLYQPVHLLVGTPGRILDLA

KKGICILKDCSMLAMDEADKLLSPEFQPSIEQLIGFLPANRQILMFSATFPVTVKDFKERYLKRPYIINLMDELTLKGIT

QYYAFVEERQKVHCLNTLFSKLQINQSIIFCNSVNRVELLAKKITELGYSCFYIHAKMLQDHRNRVFHDFRNGACRNLVC

TDLFTRGIDIQAVNVVINFDFPKNSETYLHRVGRSGRFGHLGLAVNLITYEDRFNLYRIEQELGTEIKQIPPHIDQAIYC

Q

>XP_014516622.1 DEAD-box ATP-dependent RNA helicase 8 [Vigna radiata var. radiata]

MNHHHNNRARYPPGMGLGRGGFHPNVGHNPNLNQNPGLNQNQGFQPRPSYQQQPHYVQRHLMQQPQQQQQWLRRDANAVD

EVEKTVQSEAVDSSSQDWKARLKIPPADTRYKTEDVTATKGNEFEDYFLKRELLMGIYEKGFERPSPIQEESIPIALTGS

DILARAKNGTGKTAAFCIPALEKIDQDNNVIQVVILVPTRELALQTSQVCKELGKHLKIQVMVTTGGTSLKDDIMRLYQP

VHLLVGTPGRILDLAKKGVCIMKDCSMLVMDEADKLLSPEFQPSIEQLIHFLPSNRQILMFSATFPVTVKDFKDRYLRKP

YVINLMDELTLKGITQYYAFVEERQKVHCLNTLFSKLQINQSIIFCNSVNRVELLAKKITELGYSCFYIHAKMLQDHRNR

VFHDFRNGACRNLVCTDLFTRGIDIQAVNVVINFDFPKNSETYLHRVGRSGRFGHLGLAVNLITYEDRFNLYRIEQELGT

EIKQIPPQIDQAIYCR

>CAA7016929.1 unnamed protein product [Microthlaspi erraticum]

MDNNNRGRFPPGIGADPNFQSRNPNPNPQQPQPQQYLQSRTPFPQQPQPQQYLHSPSPNPQQPAQQYVQRGYPQNPQQIQ

QQQQWSTTSAQLPGTNPNYVDEVEKTVQSEANNDSNSQDWKATLNLPPRDNRYQTEDVTATKGNEFEDYFLKRDLLRGIY

EKGFEKPSPIQEESIPIALTGSDILARAKNGTGKTGAFCIPTLEKIDPEKNVIQAVILVPTRELALQTSQVCKELSKYLK

IEVMVTTGGTSLRDDIMRLYQPVHLLVGTPGRILDLSKKGVCVLKDCAMLVMDEADKLLSAEFQPSIEELIQFLPQNRQI

LMFSATFPVTVKSFKDRYLSKPYVINLMDQLTLMGVTQYYAFVEERQKVHCLNTLFSKLQINQSIIFCNSVNRVELLAKK

ITELGYSCFYIHAKMVQDHRNRVFHDFRNGACRNLVCTDLFTRGIDIQAVNVVINFDFPRTSESYLHRVGRSGRFGHLGL

AVNLVTYEDRFKMYQTEQELGTEIKPIPSLIDKAIYCVLRRQIIQLWQPHKLIPEMKATHRILLPWQEVTV

>KAF7010002.1 hypothetical protein CFC21_024479 [Triticum aestivum]

MDQRARYPPGIGNGRGGNPNYYGRGPPPTQQHQHQQPPQQQQQAQGQRQSQHSQHHSQQLQHQQWLRRNQTAGEAAAGAA

RASEHHAPAAAGDADLSSQDWKAQLKLPPPDTRYQTEDVTATKGNEFEDYFLKRELLMGIYEKGFERPSPIQEESIPIAL

TGSDILARAKNGTGKTAAFCIPALEKIDQDKNAIQVVILVPTRELALQTSQVCKELGKHLKIQVMVTTGGTSLKDDIVRL

YQPVHLLVGTPGRVLDLTKKGICILKDCSMLIMDEADKLLSPEFQPSVEQLIRYLPSSRQILMFSATFPVTVKAFKDKYL

PKPYVINLMDELTLKGITQFYAFVEERQKVHCLNTLFSKLQINQSIIFCNSVNRVELLAKKITELGYSCFYIHAKMLQDH

RNRVFHDFRNGACRNLVCTDLFTRGIDIQAVNVVINFDFPKTAETYLHRVGRSGRFGHLGLAVNLITYEDRFNLYRIEQE

LGTEIKPIPPQIDRTIYCQ

>XP_004492712.1 DEAD-box ATP-dependent RNA helicase 8 [Cicer arietinum]

MNNRARYPPGIGLGRGGGGGLNSNPGFQQRPQQQQYVQRHMMQNQHPQHYQQNQQYQQQQQQQQQQQWLRRNQLGGTDTN

VVEEVEKTVQSEAADPSSQDWKARLKIPPPDTRYRTEDVTATKGNEFEDYFLKRELLMGIYEKGFERPSPIQEESIPIAL

TGSDILARAKNGTGKTAAFCIPALEKIDQDNNVIQVVILVPTRELALQTSQVCKELGKHLQIQVMVTTGGTSLKDDIMRL

YQPVHLLVGTPGRILDLAKKGVCVLKDCSMLVMDEADKLLSPEFQPSIEQLIQFLPSNRQILMFSATFPVTVKDFKDRYL

RKPYIINLMDELTLKGITQYYAFVEERQKVHCLNTLFSKLQINQSIIFCNSVNRVELLAKKITELGYSCFYIHAKMLQDH

RNRVFHDFRNGACRNLVCTDLFTRGIDIQAVNVVINFDFPKNSETYLHRVGRSGRFGHLGLAVNLITYEDRFNLYRIEQE

LGTEIKQIPPFIDQAIYCR

>BAJ89043.1 predicted protein [Hordeum vulgare subsp. vulgare]

MDQRARYPPGIGNGRGGNPNYHGRGPPPTQQQQQAQGHQEQYMQRHSQHSQHHDQQLQHQQWLRRNQAAGEAAGAARASV

HHAPPAADDADLSSQDWKAQLKLPPPDTRYQTEDVTATKGNEFEDYFLKRELLMGIYEKGFERPSPIQEESIPIALTGSD

ILARAKNGTGKTAAFCIPALEKIDQDKNAIQVVILVPTRELALQTSQVCKELGKHLKIQVMVTTGGTSLKDDIVRLYQPV

HLIVGTPGRVLDLTKKGICILKDCSMLIMDEADKLLSPEFQPSVEQLIRYLPSSRQILMFSATFPVTVKAFKDKYLPKPY

VINLMDELTLKGITQFYAFVEERQKVHCLNTLFSKLQINQSIIFCNSVNRVELLAKKITELGYSCFYIHAKMLQDHRNRV

FHDFRNGACRNLVCTDLFTRGIDIQAVNVVINFDFPKNAETYLHRVGRSGRFGHLGLAVNLITYEDRFNLYRIEQELGTE

IKPIPPQIDRTIYCQ

>PWA45646.1 ATP-dependent RNA helicase DEAD-box, conserved site [Artemisia annua]

MNNNNNNNNNRRYPPGINRGGYNNTNSSGGGGGGGGYQYQTNPNYQNQQQPRNPNQYQQQQYQQRQPQQQQYQQRQQQQQ

QWLRLNPNSGAANTVTGSSSSSNSNVNEVEKTVTTVDSSSQDWKAQLNIPAADSRFKTEDVTATKGNEFEDYFLKRELLM

GIYEKGFERPSPIQEESIPIALTGSDILARAKNGTGKTAAFCIPALEKIDTDKNAIQVVILVPTRELALQTSQVCKELGK

HLNIQVMVTTGGTSLKDDIMRLYQPVHLLVGTPGRILDLTKKQICKLDNCTMLVMDEADKLLSPEFQPSVEHLISFLPEN

RQILMFSATFPVTVKDFKDRYLKKPYVVNLMDELTLKGITQYYAFVEERQKVHCLNTLFSKLQINQSIIFCNSVNRVELL

AKKITELGYSCFYIHAKMLQDHRNRVFHDFRNGACRNLVCTDLFTRGIDIQAVNVVINFDFPRNAETYLHRVGRSGRFGH

LGLAVNLITYEDRFNLYRIEQELGTEIRQIPPQIDQAIYCQ

>XP_013660402.1 DEAD-box ATP-dependent RNA helicase 8 [Brassica napus]

MNNNNNNRGRYPPGIGAGRAAINSNPNFQSRPGYQQQPPPQYVQRGGYAQQNHQQQFQQATSQQVQQQHGYQQQQQWLRR

PQISGGNSNGDAVAEVEKSVLSEAVDTNSEDWKARLKLPAPDTRFRTEDVTATKGNEFEDYFLKRELLMGIYEKGFERPS

PIQEESIPIALTGRDILARAKNGTGKTAAFCIPVLEKIDQDNNVIQAVIIVPTRELALQTSQVCKELGKHLKIQVMVTTG

GTSLKDDIMRLYQPVHLLVGTPGRILDLTKKGVCVLKDCSVFVMDEADKLLSQEFQPSVEHLISFLPQNRQILMFSATFP

VTVKDFKDRFLTNPYIINLMDELTLKGITQFYAFVEERQKIHCLNTLFSKLQINQSIIFCNSVNRVELLAKKITELGYSC

FYIHAKMLQDHRNRVFHDFRNGACRNLVCTDLFTRGIDIQAVNVVINFDFPKTAETYLHRVGRSGRFGHLGLAVNLITYE

DRFNLYRIEQELGTEIKQIPPHIDQAIYCQ

>XP_009104295.1 DEAD-box ATP-dependent RNA helicase 12 [Brassica rapa]

MNNNRGRYPPGNGTGRGAPPHPDYQSYGQQPQNHQQFQQWSRRAQLPGNANEVQNTTSSQPPNDHKFSGSVTLSGGKDWK

ATLKLPPPDTRYQTADVTATKGNEFEDYFLKRDLLKGIYEKGFEKPSPIQEESIPIALTGSDILARAKNGTGKTGAFCIP

VLEKIDPNTNVIQAMILVPTRELALQTSQVCKELSKYLNIQVMVTTGGTSLRDDIMRLHQPVHLLVGTPGRILDLTKKGV

CVLKDCTMLVMDEADKLLSAEFQPSLEELIQFLPENRQFLMFSATFPVTVKAFKDRHLRKPYVINLMDQLTLMGVTQYYA

FVEERQKVHCLNTLFSKLQINQSIIFCNSVNRVELLAKKITELGYSCFYIHAKMVQDHRNRVFHEFRNGACRNLVCTDLF

TRGIDIQAVNVVINFDFPRTSESYLHRVGRSGRFGHLGLAVNLVTYEDRFKMYQTEQELGTEIKPIPSQIDQAIYCQ

>XP_010456256.1 PREDICTED: DEAD-box ATP-dependent RNA helicase 8 [Camelina sativa]

MNNRGRYPPGSGAGRGAINPNPNYQSRQGYYQQQPQPQYVQRGGYAQNHHQQAPSQPHQYQQQQQQQQWLRRAQIPSGGN

SNGDAVVEVEKTVQSEVIDPNSEDWKAKLKLPAPDTRYRTEDVTATKGNEFEDYFLKRELLMGIYEKGFERPSPIQEESI

PIALTGRDILARAKNGTGKTAAFCIPVLEKIDQDNNVIQAVIIVPTRELALQTSQVCKELGKHLKIQVMVTTGGTSLKDD

IMRLYQPVHLLVGTPGRILDLTKKGVCVLKDCSVLVMDEADKLLSQEFQPSVEHLISFLPENRQILMFSATFPVTVKAFK

DRFLTNPYVINLMDELTLKGVTQFYAFVEERQKIHCLNTLFSKLQINQSIIFCNSVNRVELLAKKITELGYSCFYIHAKM

LQDHRNRVFHDFRNGACRNLVCTDLFTRGIDIQAVNVVINFDFPKNAETYLHRVGRSGRFGHLGLAVNLITYEDRFNLYR

IEQELGTEIKQIPPHIDQAIYCQ

>VAH49576.1 unnamed protein product [Triticum turgidum subsp. durum]

MDQRARYPPGIGNGRGGNPNYYGRGPPPTQQHQHQQPPQQQQQAQGQRQSQHSQHHSQQLQHQQWLRRNQTAGEAAAGAA

RASEHHAPAAAGDADLSGLVLLDKGITVSKHELSVERAPGSQDWKAQLKLPPPDTRYQTEDVTATKGNEFEDYFLKRELL

MGIYEKGFERPSPIQEESIPIALTGSDILARAKNGTGKTAAFCIPALEKIDQDKNAIQVVILVPTRELALQTSQVCKELG

KHLKIQVMVTTGGTSLKDDIVRLYQPVHLLVGTPGRVLDLTKKGICILKDCSMLIMDEADKLLSPEFQPSVEQLIRYLPS

SRQILMFSATFPVTVKAFKDKYLPKPYVINLMDELTLKGITQFYAFVEERQKVHCLNTLFSKLQINQSIIFCNSVNRVEL

LAKKITELGYSCFYIHAKMLQDHRNRVFHDFRNGACRNLVCTDLFTRGIDIQAVNVVINFDFPKTAETYLHRVGRSGRFG

HLGLAVNLITYEDRFNLYRIEQELGTEIKPIPPQIDRTIYCQ

>KAF3566979.1 hypothetical protein DY000_02010751 [Brassica cretica]

MNNNRGRYPPGIGAGRGAINPNPNFQSRPGYQQQPPPQYVQRGGYTAQQNHQQQFQQATSQQQWLRRPQISGGNSNGDAV

AEVEKSVLSQAIDTNSEDWKARLQLPAPDTRFRTEDVTATKGNEFEDYFLKRELLMGIYEKGFERPSPIQEESIPIALTG

RDILARAKNGTGKTAAFCIPVLEKIDQDNNVIQAVIIVPTRELALQTSQVCKELGKHLKIQVMVTTGGTSLKDDIMRLYQ

PVHLLVGTPGRILDLTKKGVCVLKDCSVFVMDEADKLLSQEFQPSVEHLISFLPQNRQILMFSATFPVTVKDFKDRFLTN

PYIINLMDELTLKGITQFYAFVEERQKIHCLNTLFSKLQINQSIIFCNSVNRVELLAKKITELGYSCFYIHAKMLQDHRN

RVFHDFRNGACRNLVCTDLFTRGIDIQAVNVVINFDFPKTAETYLHRVGRSGRFGHLGLAVNLITYEDRFNLYRIEQELG

TEIKQIPPHIDQAIYCQ

>KAE8665822.1 DEAD-box ATP-dependent RNA helicase 8 [Hibiscus syriacus]

MNSRGRYPPGTGAGRGGGVNASPSYQSRPSQQQRNHLLTGNDSNVVDKVEKTVQSEAIDASSQDWKARLKMPPPDTRYRT

EDVTATKGNEFEDYFLKRELLMGIYEKGFERPSPIQEESIPIALTGSDILARAKNGTGKTAAFCIPALEKIDQDNNVIQA

VILVPTRELALQTSQVCKELGKHLQIQVMVTTGGTSLRDDIMRLYQPVHLLVGTPGRILDLAKKGVCILKDCSMLIMDEA

DKLLSPEFQPSIEQLIQYLPANRQILMFSATFPVTVKDFKDRYLKKPYIINLMDELTLKGITQYYAFVEERQKVHCLNTL

FSKLQINQSIIFCNSVNRVELLAKKITELGYSCFYIHAKMLQDHRNRVFHDFRNGACRNLVCTDLFTRGIDIQAVNVVIN

FDFPKNSETYLHRVGRSGRFGHLGLAVNLITYEDRFNLYRIEQELGTEIKQIPPHIDQAIYCR

>XP_033134584.1 DEAD-box ATP-dependent RNA helicase 12 [Brassica rapa]

MNNSRGRYPPGRGRGAPPNPDHQTYQQQPQRGQSQNHQQQQWTRRAQLPGNANEVQKTTSSQPPVASSDSNGQDWKSTLR

LPPPDTRYQTADVTATKGNEFEDYFLKRDLLKGIYEKGFEKPSPIQEESIPIALTGSDILARAKNGTGKTGAFCIPVLER

IDPNTNVIQAMILVPTRELALQTSQVCKELSKYLNIHVMVTTGGTSLRDDIMRLHQPVHLLVGTPGRILDLTKKGVCVLK

DCTMLVMDEADKLLSAEFQPSLEELIQFLPQNRQFLMYSATFPVTVKAFKDRHLRKPYVINLMDQLTLMGVTQYYAFVEE

RQKVHCLNTLFSKLQINQSIIFCNSVNRVELLAKKITELGYSCFYIHAKMVQDHRNRVFHEFRNGACRNLVCTDLFTRGI

DIQAVNVVINFDFPRTSESYLHRVGRSGRFGHLGLAVNLVTYEDRFKMYQTEQELGTEIKPIPSNIDQAIYCQ

>RZC18652.1 DEAD-box ATP-dependent RNA helicase 8 [Glycine soja]

MNHNNNNRARYPPGMGIGRGGFNPNLGQNPSLNQNPNLNQNHHAFQARPPYHHQQQQPQYVQRHLLQPPPPQQQQQWLRR

DANAVDEVEKTVQSEPMDSRTVSAMGVVVAACILQPYVISSKSLPGFMQDPIAPLYNIEIPAIHISTDDSSSQDWKARLK

IPPADTRYRTEDVTATKGNEFEDYFLKRELLMGIYEKGFERPSPIQEESIPIALTGSDILARAKNGTGKTAAFCIPALEK

IDQDNNVIQVVILVPTRELALQTSQVCKELGKHLKIQVMVTTGGTSLKDDIMRLYQPVHLLVGTPGRILDLAKKGVCILK

DCAMLVMDEADKLLSPEFQPSIEQLIHFLPTTRQILMFSATFPVTVKDFKDRYLRKPYVINLMDELTLKGITQFYAFVEE

RQKVHCLNTLFSKLQINQSIIFCNSVNRVELLAKKITELGYSCFYIHAKMLQDHRNRVFHDFRNGACRNLVCTDLFTRGI

DIQAVNVVINFDFPKNAETYLHRVGRSGRFGHLGLAVNLITYEDRFNLYRIEQELGTEIKQIPPQIDQAIYCR

>KAE9595535.1 putative RNA helicase [Lupinus albus]

MNNSNRGRYPPGIGYGRGSGGGGFNQNPNQNQNAAFQSRNNFQQQQNQQQHYVQRNLVPQQNQQQQQQQWLRRAQLGGGA

DSNVVDEVEKNVQNEANDSSSQDWKARLKAPPADTRYKTEDVTATKGNEFEDYFLKRELLMGIYEKGFERPSPIQEESIP

IALTGSDILARAKNGTGKTAAFCIPALEKIDQDTNVIQAVILVPTRELALQTSQVCKELGKHLKIEVMVTTGGTSLKDDI

MRLYQPVHLLVGTPGRILDLAKKGVCILKDCTMLVMDEADKLLSPEFQPSIQHLIQFLPSNRQILMFSATFPVTVKDFKD

RYLHKPYVINLMDELTLKGITQFYAFVEERQKVHCLNTLFSKLQINQSIIFCNSVNRVELLAKKITELGYSCFYIHAKML

QDHRNRVFHDFRNGACRNLVCTDLFTRGIDIQAVNVVINFDFPKNSETYLHRVGRSGRFGHLGLAVNLITYEDRFNLYRI

EQELGTEIKQIPPFIDQAVYCR

>XP_003521117.1 DEAD-box ATP-dependent RNA helicase 8 [Glycine max]

MNHNNNNRARYPPGMGLGRGGFNPNPSQNPNLNQNPSLNQNHHAFQARPPYHHQQQRVQYVQRHLLQQQQQQWLRRDGSA

AVDEVEKTVQSEAVDSSSQDWKARLKIPPADTRYKTEDVTATKGNEFEDYFLKRELLMGIYEKGFERPSPIQEESIPIAL

TGSDILARAKNGTGKTAAFCIPALEKIDQDNNVIQVVILVPTRELALQTSQVCKELAKHLKIQVMVTTGGTSLKDDIMRL

YQPVHLLVGTPGRILDLAKKGVCILKDCAMLVMDEADKLLSPEFQPSIEQLIHCLPTTRQILMFSATFPVTVKDFKDRYL

RKPYVINLMDELTLKGITQFYAFVEERQKVHCLNTLFSKLQINQSIIFCNSVNRVELLAKKITELGYSCFYIHAKMLQDH

RNRVFHDFRNGACRNLVCTDLFTRGIDIQAVNVVINFDFPKNAETYLHRVGRSGRFGHLGLAVNLITYEDRFNLYRIEQE

LGTEIKQIPPQIDQAIYCR

>XP_013612716.1 PREDICTED: DEAD-box ATP-dependent RNA helicase 8 [Brassica oleracea var. oleracea]

MNNNNNNNNRGRYPPGIGAGRGAINPNPNFQSRPGYQQQPPPQFQQATSQQQQVQQQWLRRPQISGGNSNGDAVAQVEKS

VLSQAIDTNSEDWKARLQLPAPDTRFRTEDVTATKGNEFEDYFLKRELLMGIYEKGFERPSPIQEESIPIALTGRDILAR

AKNGTGKTAAFCIPVLEKIDQDNNVIQALIIVPTRELALQTSQVCKELGKHLKIQVMVTTGGTSLKDDIMRLYQPVHLLV

GTPGRILDLTKKGVCVLKDCSVFVMDEADKLLSQEFQPSVEHLISFLPQNRQILMFSATFPVTVKDFKDRFLTNPYIINL

MDELTLKGITQFYAFVEERQKIHCLNTLFSKLQINQSIIFCNSVNRVELLAKKITELGYSCFYIHAKMLQDHRNRVFHDF

RNGACRNLVCTDLFTRGIDIQAVNVVINFDFPKTAETYLHRVGRSGRFGHLGLAVNLITYEDRFNLYRIEQELGTEIKQI

PPHIDQTIYCQ

>KAE9592226.1 putative RNA helicase [Lupinus albus]

MNNRARYPPGIGLGRGGGSGGGGGGGGSGLNPNPGFQQRPPQQHVQRHIMQQQQYQQQQQQQQQQQQQQWLRRTQLGGSA

NTNVVEEVEKTVQSEAVDESSQDWKTKLKIPPADTRYRTEDVTATKGNEFEDYFLKRELLMGIYEKGFERPSPIQEESIP

IALTGSDILARAKNGTGKTAAFCIPALEKIDQDNNVIQVVILVPTRELALQTSQVCKELGKHLKIQVMVTTGGTSLKDDI

MRLYQPVHLLVGTPGRILDLARKGVCVMKDCSMLVMDEADKLLSPEFQPSIEQLIQFLPGNRQILMFSATFPVTVKDFKD

RYLRKPYVINLMDELTLKGITQFYAFVEERQKVHCLNTLFSKLQINQSIIFCNSVNRVELLAKKITELGYSCFYIHAKML

QDHRNRVFHDFRNGACRNLVCTDLFTRGIDIQAVNVVINFDFPKNSETYLHRVGRSGRFGHLGLAVNLITYEDRFNLYRI

EQELGTEIKQIPPHIDQAIYCQ

>XP_009111462.1 DEAD-box ATP-dependent RNA helicase 8 isoform X1 [Brassica rapa]

MNNNNNNRGRYPPGIGAGRAAINSNPNFQSRPGYQQQPPPQYVQRGGYAQQNHQQQFQQATSQQVQQQHGYQQQQQWLRR

PQISGGNSNGDAVAEVEKSVLSEAVDTNSEDWKARLKLPAPDTRYRTEDVTATKGNEFEDYFLKRELLMGIYEKGFERPS

PIQEESIPIALTGRDILARAKNGTGKTAAFCIPVLEKIDQDNNVIQAVIIVPTRELALQTSQVCKELGKHLKIQVMVTTG

GTSLKDDIMRLYQPVHLLVGTPGRILDLTKKGVCVLKDCSVFVMDEADKLLSQEFQPSVEHLISFLPQNRQILMFSATFP

VTVKDFKDRFLTNPYIINLMDELTLKGITQFYAFVEERQKIHCLNTLFSKLQINQSIIFCNSVNRVELLAKKITELGYSC

FYIHAKMLQDHRNRVFHDFRNGACRNLVCTDLFTRGIDIQAVNVVINFDFPKTAETYLHRVGRSGRFGHLGLAVNLITYE

DRFNLYRIEQELGTEIKQIPPHIDQAIYCQ

>KHN27313.1 DEAD-box ATP-dependent RNA helicase 8 [Glycine soja]

MNHNNNNRARYPPGMGLGRGGFNPNPSQNPNLNQNPSLNQNHHAFQARPPYHHQQQRVQYVQRHLLQQQQQQWLRRDGSA

AVDAVEKTVQSEAVDSSSQDWKARLKIPPADTRYKTEDVTATKGNEFEDYFLKRELLMGIYEKGFERPSPIQEESIPIAL

TGSDILARAKNGTGKTAAFCIPALEKIDQDNNVIQVVILVPTRELALQTSQVCKELAKHLKIQVMVTTGGTSLKDDIMRL

YQPVHLLVGTPGRILDLAKKGVCILKDCAMLVMDEADKLLSPEFQPSIEQLIHCLPTTRQILMFSATFPVTVKDFKDRYL

RKPYVINLMDELTLKGITQFYAFVEERQKVHCLNTLFSKLQINQSIIFCNSVNRVELLAKKITELGYSCFYIHAKMLQDH

RNRVFHDFRNGACRNLVCTDLFTRGIDIQAVNVVINFDFPKNAETYLHRVGRSGRFGHLGLAVNLITYEDRFNLYRIEQE

LGTEIKQIPPQIDQAIYCR

>XP_010273108.1 PREDICTED: DEAD-box ATP-dependent RNA helicase 8 [Nelumbo nucifera]

MNTRGRYPPGIGNGRGGNVNANPNFQARNPQQQYVQRSTVQNPQQFQQQQQQQWLRRNPMGTESGSNEVEKTVQSEAVDS

SSQDWKARLKIPPTDTRYKTEDVTATKGNEFEDYFLKRELLMGIYEKGFERPSPIQEESIPIALTGSDILARAKNGTGKT

AAFCVPALEKIDQDNNVIQVVILVPTRELALQTSQVCKELGKHLKIQVMVTTGGTSLKDDIMRLYQPVHLLVGTPGRILD

LAKKGICILKYCSMLVMDEADKLLSPEFQPSVDQLIRFLPENRQILMFSATFPVTVKDFKERYLHKPYIINLMDELTLKG

ITQYYAFVEERQKVHCLNTLFSKLQINQSIIFCNSVNRVELLAKKITELGYSCFYIHAKMLQDHRNRVFHDFRNGACRNL

VCTDLFTRGIDIQAVNVVINFDFPKNSETYLHRVGRSGRFGHLGLAVNLITYEDRFNLYRIEQELGTEIKQIPPHIDQAI

YCR

>XP_010255444.1 PREDICTED: DEAD-box ATP-dependent RNA helicase 8-like isoform X1 [Nelumbo nucifera]

MNTRGRYPPGIGNGRGGNVNANPNFQARNPQQQYVQRNTVQNQQAFQQQQQWLRRNPMGTDSSSNEVEKTVQSEAVDSSS

QDWKARLKIPPRDTRYKTEDVTATKGNEFEDYFLKRELLMGIYEKGFERPSPIQEESIPIALTGSDILARAKNGTGKTAA

FCIPALEKIDQDTNVIQVVILVPTRELALQTSQVCKELGKHLKIQVMVTTGGTSLKDDIMRLYQPVHLLVGTPGRILDLA

KKGVCVLKDCSMLIMDEADKLLSPEFQPSIDQLIRFVPANRQILMFSATFPVTVKDFKDRYLQKPYIINLMDELTLKGIT

QYYAFVEERQKVHCLNTLFSKLQINQSIIFCNSVNRVELLAKKITELGYSCFYIHAKMLQDHRNRVFHDFRNGACRNLVC

TDLFTRGIDIQAVNVVINFDFPKNSETYLHRVGRSGRFGHLGLAVNLITYEDRFNLYRIEQELGTEIKQIPPQIDQAIYC

R

>XP_020156832.1 DEAD-box ATP-dependent RNA helicase 6 isoform X1 [Aegilops tauschii subsp. tauschii]

MDQRARHPPGIGNGRGGNPNYHGRGPPPTQQHHQQPSPPSPQQAQGHPQQYMQRQSQHSQHHSQQLQHQQWLRRNQTAGE

AASGAARASEHHAPAAADDADLSSQDWKAQLKLPPPDTRYQTEDVTATKGNEFEDYFLKRELLMGIYEKGFERPSPIQEE

SIPIALTGSDILARAKNGTGKTAAFCIPALEKIDQDKNAIQVVILVPTRELALQTSQVCKELGKHLKIQVMVTTGGTSLK

DDIVRLYQPVHLLVGTPGRVLDLTKKGICILKDCSMLIMDEADKLLSPEFQPSVEQLIRYLPSSRQILMFSATFPVTVKA

FKDKYLPKPYVINLMDELTLKGITQFYAFVEERQKVHCLNTLFSKLQINQSIIFCNSVNRVELLAKKITELGYSCFYIHA

KMLQDHRNRVFHDFRNGACRNLVCTDLFTRGIDIQAVNVVINFDFPKTAETYLHRVGRSGRFGHLGLAVNLITYEDRFNL

YRIEQELGTEIKPIPPQIDRTIYCQ

>VAH49578.1 unnamed protein product [Triticum turgidum subsp. durum]

MDQRARYPPGIGNGRGGNPNYYGRGPPPTQQHQHQQPPQQQQQAQGQRQSQHSQHHSQQLQHQQWLRRNQTAGEAAAGAA

RASEHHAPAAAGDADLRCVLLVRTRGLDRLKVQHGSQDWKAQLKLPPPDTRYQTEDVTATKGNEFEDYFLKRELLMGIYE

KGFERPSPIQEESIPIALTGSDILARAKNGTGKTAAFCIPALEKIDQDKNAIQVVILVPTRELALQTSQVCKELGKHLKI

QVMVTTGGTSLKDDIVRLYQPVHLLVGTPGRVLDLTKKGICILKDCSMLIMDEADKLLSPEFQPSVEQLIRYLPSSRQIL

MFSATFPVTVKAFKDKYLPKPYVINLMDELTLKGITQFYAFVEERQKVHCLNTLFSKLQINQSIIFCNSVNRVELLAKKI

TELGYSCFYIHAKMLQDHRNRVFHDFRNGACRNLVCTDLFTRGIDIQAVNVVINFDFPKTAETYLHRVGRSGRFGHLGLA

VNLITYEDRFNLYRIEQELGTEIKPIPPQIDRTIYCQ

>XP_019456295.1 PREDICTED: DEAD-box ATP-dependent RNA helicase 8-like [Lupinus angustifolius]

MDNSNNNNRGRYPPGIGYGRGGGGGGFNLNPNQNQNAAFQPRNNYQQQQNQQQHYVQRHLVPQQQQQQQQQQWLRRAQLG

GGADSNVVDEVEKNVQNEANDSSSQDWKARLKAPPADTRYKTEDVTATKGNEFEDYFLKRELLMGIYEKGFERPSPIQEE

SIPIVLTGSDILARAKNGTGKTAAFCIPALEKIDQDTNVIQAVILVPTRELALQTSQVCKELGKHLKIEVMVTTGGTSLK

DDIMRLYQPVHLLVGTPGRILDLAKKGVCILKDCTMLVMDEADKLLSPEFQPSIQHLIQFLPSNRQILMFSATFPVTVKD

FKDRYLQKPYVINLMDELTLKGITQYYAFVEERQKVHCLNTLFSKLQINQSIIFCNSVNRVELLAKKITELGYSCFYIHA

KMLQDHRNRVFHDFRNGACRNLVCTDLFTRGIDIQAVNVVINFDFPKNSETYLHRVGRSGRFGHLGLAVNLITYEDRFNL

YRIEQELGTEIKQIPPFIDQAVYCR

>XP_004953257.1 DEAD-box ATP-dependent RNA helicase 8 [Setaria italica]

MDPRARYPPGMGNGRGGNPNYYGRGPPPQQPQQPHHQHQQTSGAHHHQQYAQRQQQQHHHHNYNHQQQHHNHHQQQQQQQ

WLRRNQIAREAAGAAGTSEPKALAPSTAADGVDSSSQDWKAQLKLPPQDTRYRTEDVTATKGNEFEDYFLKRELLMGIYE

KGFERPSPIQEESIPIALTGSDILARAKNGTGKTAAFCIPALEKIDQDKNAIQVVILVPTRELALQTSQVCKELGKHLKI

QVMVTTGGTSLKDDIVRLYQPVHLLVGTPGRILDLTKKGICILKDCSMLIMDEADKLLSPEFQPSVEQLIRYLPASRQIL

MFSATFPVTVKEFKDKYLPKPYVINLMDELTLKGITQFYAFVEERQKVHCLNTLFSKLQINQSIIFCNSVNRVELLAKKI

TELGYSCFYIHAKMLQDHRNRVFHDFRNGACRNLVCTDLFTRGIDIQAVNVVVNFDFPKNSETYLHRVGRSGRFGHLGLA

VNLITYEDRFNLYRIEQELGTEIKPIPPQIDQAIYCQ

>XP_012066035.2 DEAD-box ATP-dependent RNA helicase 8 [Jatropha curcas]

MDPSKFIGKQPMTMDIEQMPDIPQRGSHHRRAHSDTSFRFDDLLLFDPSDLDLSSLDLPTPTPPRSGSGVPMAVDSGSVS

DDSASVSHSGPSTKPKPINHLRSLSVDSDFFDGLGLSSGGGDEKFGGKAVATAAAGAGGGTGERRVHHRHSNSMDGSTSS

FEIESLMIDGVKKAMGPDRLAELALIDPKRAKRILANRQSAARSKERKIRYTSELERKVQTLQTEATTLSAQVTMLQRDT

TGLTAENKELKLRLQAMEQQAQLRDALNEALREEVQRLKIATGQIPPANGNPFTRGLTPQFSSHQQALHHFPSSQSQPHQ

QQQQQQQQQQQQQWLRRSQLPAADSSVDEVEKTVQSEAVDSSSQDWKARLKIPPADTRYKTEDVTATKGNEFEDYFLKRE

LLMGIYEKGFERPSPIQEESIPIALTGSDILARAKNGTGKTAAFCIPALEKIDQDNNVIQVVILVPTRELALQTSQVCKE

LGKHLKIQVMVTTGGTSLKDDIMRLYQPVHLLVGTPGRILDLAKKGVCILKDCSMLVMDEADKLLSPEFQPSVEQLIRFL

PPTRQILMFSATFPVTVKDFKDRYLHKPYIINLMDELTLKGITQYYAFVEERQKVHCLNTLFSKLQINQSIIFCNSVNRV

ELLAKKITELGYSCFYIHAKMLQDHRNRVFHDFRNGACRNLVCTDLFTRGIDIQAVNVVINFDFPKNSETYLHRVGRSGR

FGHLGLAVNLITYEDRFNLYRIEQELGTEIKQIPPHIDQAIYCR

>RLM79415.1 DEAD-box ATP-dependent RNA helicase 8 [Panicum miliaceum]

MDPRARYPPGMGNGRGGNPNYYGRGPPPHQHNYQHQQTSGAHHHQQYAQRQQQQHHQNHNHNHHQQQQQHHNHHQQQQQR

QWLRRNQIAREAVGAAGTSEPKALVPSTAADGVDSSSQDWKAQLKLPPQDTRYRTEDVTATKGNEFEDYFLKRELLMGIY

EKGFERPSPIQEESIPIALTGSDILARAKNGTGKTAAFCIPALEKIDPDNNAIQVVILVPTRELALQTSQVCKDLGKHLK

IQVMVTTGGTSLKDDIVRLYQPVHLLVGTPGRILDLTKKGVCILKDCSMLIMDEADKLLSPEFQPSVEQLIRYLPASRQI

LMFSATFPVTVKEFKDKYLPKPYVINLMDELTLKGITQFYAFVEERQKVHCLNTLFSKLQINQSIIFCNSVNRVELLAKK

ITELGYSCFYIHAKMLQDHRNRVFHDFRNGACRNLVCTDLFTRGIDIQAVNVVINFDFPKNSETYLHRVGRSGRFGHLGL

AVNLITYEDRFNLYRIEQELGTEIKPIPPQIDQAIYCQ

>CDY51147.1 BnaA09g51820D [Brassica napus]

MNNNNNNRGRYPPGIGAGRAAVNPNPNFQSRPGYQQQPPPQYFQQATSQQVQQQHGYQQQQQWLRRPQISGGNSNGDAVA

EVEKSVLSEAVDTNSEDWKARLKLPAPDTRFRTEDVTATKGNEFEDYFLKRELLMGIYEKGFERPSPIQEESIPIALTGR

DILARAKNGTGKTAAFCIPVLEKIDQDNNVIQAVIIVPTRELALQTSQVCKELGKHLKIQVMVTTGGTSLKDDIMRLYQP

VHLLVGTPGRILDLTKKGVCVLKDCSVFVMDEADKLLSQEFQPSVEHLISFLPQNRQILMFSATFPVTVKDFKDRFLTNP

YIINLMDELTLKGITQFYAFVEERQKIHCLNTLFSKLQINQSIIFCNSVNRVELLAKKITELGYSCFYIHAKMLQDHRNR

VFHDFRNGACRNLVCTDLFTRGIDIQAVNVVINFDFPKTAETYLHRVGRSGRFGHLGLAVNLITYEDRFNLYRIEQELGT

EIKQIPPHIDQAIYCQ

>XP_019461914.1 PREDICTED: DEAD-box ATP-dependent RNA helicase 8-like [Lupinus angustifolius]

MNNSNNNRARYPPGIGYGRGGGGGGDGGGFNPNHNQNAPFQPRPSYQQQQQQHQHQQHYAQRNLVPQQQQQQQQWLRRAQ

LGAGGSESNVVDEVEKTVQTEASDSSSQDWKARLKAPPPDTRYKTEDVTATKGNEFEDYFLKRELLMGIYEKGFERPSPI

QEESIPIALTGSDILARAKNGTGKTAAFCIPALEKIDQDTNVIQAVILVPTRELALQTSQVCKELGKHLKIEVMVTTGGT

SLRDDIMRLYQPVHLLVGTPGRILDLTKKGVCVLKDCTMLVMDEADKLLSPEFQPSIQQLIQFLPSNRQILMFSATFPVT

VKDFKDRYLQKPYVINLMDELTLKGITQFYAFVEERQKVHCLNTLFSKLQINQSIIFCNSVNRVELLAKKITELGYSCFY

IHAKMLQDHRNRVFHDFRNGACRNLVCTDLFTRGIDIQAVNVVINFDFPKNSETYLHRVGRSGRFGHLGLAVNLITYEDR

FNLYRIEQELGTEIKQIPPFIDQAVYCR

>KAF7017324.1 hypothetical protein CFC21_030787 [Triticum aestivum]

MDQRARHPPGIGNGRGGNPNYHGRGPPPTQQHHQQPSPPSPQQAQGHPQQYMQRQSQHSQHSQHHSQQLQHQQWLRRNQT

AGEAASGAARASEHHAPAAADDADLSSQDWKAQLKLPPPDTRYQTEDVTATKGNEFEDYFLKRELLMGIYEKGFERPSPI

QEESIPIALTGSDILARAKNGTGKTAAFCIPALEKIDQDKNAIQVVILVPTRELALQTSQVCKELGKHLKIQVMVTTGGT

SLKDDIVRLYQPVHLLVGTPGRVLDLTKKGICILKDCSMLIMDEADKLLSPEFQPSVEQLIRYLPSSRQILMFSATFPVT

VKAFKDKYLPKPYVINLMDELTLKGITQFYAFVEERQKVHCLNTLFSKLQINQSIIFCNSVNRVELLAKKITELGYSCFY

IHAKMLQDHRNRVFHDFRNGACRNLVCTDLFTRGIDIQAVNVVINFDFPKTAETYLHRVGRSGRFGHLGLAVNLITYEDR

FNLYRIEQELGTEIKPIPPQIDRTIYCQ

>PSS11712.1 DEAD-box ATP-dependent RNA helicase [Actinidia chinensis var. chinensis]

MNTRGRYPPGIGGGGGRGGNLYPNPNFQPRNFQQQYVQRGTMQNHQQFQSQQQQQQQQQQQQQWLRRNQLGADSAVDEVE

KTVQSEAVDSSSQDWKAQLKIPPTDSRYRTEDVTATKGNEFEDYFLKRELLMGIYEKGFERPSPIQEESIPIALTGSDIL

ARAKNGTGKTAAFCIPALEKIDQDNNVIQAVILVPTRELALQTSQVCKELGKHLNIQVMVTTGGTSLRDDIMRLYQPVHL

LVGTPGRILDLAKKGVCVLKDCSMLVMDEADKLLSPEFQPSLEQLIRFLPSNRQVLMFSATFPVTVKDFKDRYLHKPYVI

NLMDELTLKGITQFYAFVEERQKVHCLNTLFSKLQINQSIIFCNSVNRVELLAKKITELGYSCFYIHAKMLQDHRNRVFH

DFRNGACRNLVCTDLFTRGIDIQAVNVVINFDFPKNSETYLHRVGRSGRFGHLGLAVNLITYEDRFNLYRIEQELGTEIK

QIPPHIDQAIYCQ

>XP_024440677.1 DEAD-box ATP-dependent RNA helicase 8 [Populus trichocarpa]

MNNNNRGRYPPGIGAGRGGGMSANPNFQSRVPQQQYVQRHFGQNHHQQQYNQHQQNHNQQQQQQQQQQQHQQQQWLRRSQ

LAAADSSVDEVEKTVQSEAVDSSSQDWKAKLKIPPADTRYQTEDVTATKGNDFEDYFLKRELLMGIYEKGFERPSPIQEE

SIPIALTGSDILARAKNGTGKTAAFCIPALEKIDQDNNFIQVVILVPTRELALQTSQVCKELGKHLKIQVMATTGGTSLK

DDIMRLYQPVHLLVGTPGRILDLAKKGVCILKDCSMLVLDEADKLLSPEFQPSIEQLIRFLPSSRQILMFSATFPVTVKD

FKDRYLEKPYVINLMDELTLKGITQYYAFVEERQKVHCLNTLFSKLQINQSIIFCNSVNRVELLAKKITELGYSCFYIHA

KMLQDHRNRVFHDFRNGACRNLVCTDLFTRGIDIQAVNVVINFDFPKNSETYLHRVGRSGRFGHLGLAVNLITYEDRFNL

YRIEQELGTEIKQIPPHIDQAIYCQ

>XP_020168342.1 DEAD-box ATP-dependent RNA helicase 8 [Aegilops tauschii subsp. tauschii]

MDPRARYPPGTGNGRGGNPNYYGRGPPLSQNNHHHHQQTSGAHQQQYVQRQPQPQPQQHHQNNHQQQHHQNNHQQQHHQN

HHHQQQQQQQQWLRRNQINAAGTSGPKVVGPPPAAVGNDPSSQDWKAQLKLPPADTRFRTEDVTATKGNEFEDYFLKREL

LMGIYEKGFENPSPIQEESIPIALTGSDILARAKNGTGKTAAFCIPALEKIDQDKNAIQVVIVVPTRELALQTSQVCKEL

GKHLKIQVMVTTGGTSLKDDIVRLYQPVHLLVGTPGRILDLTKKGVCILKDCSMLVMDEADKLLSPEFQPSIEQLIRYLP

ASRQILMFSATFPVTVKEFKDKYLPKPYVINLMDELTLKGITQFYAFVEERQKVHCLNTLFSKLQINQSIIFCNSVNRVE

LLAKKITELGYSCFYIHAKMLQDHRNRVFHDFRNGACRNLVCTDLFTRGIDIQAVNVVINFDFPKSSETYLHRVGRSGRF

GHLGLAVNLITYEDRFNLYSIEQELGTEIKPIPPQIDQAIYCQ

>TVU15066.1 hypothetical protein EJB05_38568 [Eragrostis curvula]

MDQRARYPPDMGNGRGGNPNYYGRGPPPLQQHHHQPPPPWQVHHQQYVQRQPQPYNQQQQPPPYNQQQQQQWLRRNQIAG

EAAGASAPRAPPPADGVDSSSQDWKAQLKLPPQDTRYRTEDVTATKGNEFEDYFLKRELLMGIYEKGFERPSPIQEESIP

IALTGSDILARAKNGTGKTAAFCIPALEKIDQDKNAIQVVILVPTRELALQTSQVCKELGKHLKIQVMVTTGGTSLKDDI

IRLYQPVHLLVGTPGRILDLTKKGVCILKDCSMLIMDEADKLLSPEFQPSIEQLIRYLPSNRQILMFSATFPVTVKEFKD

KYLPKPYVINLMDELTLKGITQFYAFVEERQKVHCLNTLFSKLQINQSIIFCNSVNRVELLAKKITELGYSCFYIHAKML

QDHRNRVFHDFRNGACRNLVCTDLFTRGIDIQAVNVVINFDFPKNSETYLHRVGRSGRFGHLGLAVNLITYEDRFNLYRI

EQELGTEIKPIPPQIDRAIYCQ

>XP_024451238.1 DEAD-box ATP-dependent RNA helicase 8 [Populus trichocarpa]

MNNNNRGRYPPGIGAGRGGGMNANPNFQSRVPQQQYVQRNFGQNHHQQQYYQHQQHHNQQQQQQQQQQWLRRNQLTAADS

SIDEVEKTVQSEAVDSSSQDWKAKLKIPPADTRYRTEDVTATKGNDFEDYFLKRELLMGIYEKGFERPSPIQEESIPIAL

TGSDILARAKNGTGKTAAFCVPALEKIDQDNNFIQVVILVPTRELALQTSQVCKELGKHLKIQVMATTGGTSLKDDIMRL

YQPVHLLVGTPGRILDLAKKGVCILKNCSMLVLDEADKLLSPEFQPSIEQLIRFLPSNRQILMFSATFPVTVKDFKDRYL

EKPYVINLMDELTLKGITQYYAFVEERQKVHCLNTLFSKLQINQSIIFCNSVNRVELLAKKITELGYSCFYIHAKMLQDH

RNRVFHDFRNGACRNLVCTDLFTRGIDIQAVNVVINFDFPKNAETYLHRVGRSGRFGHLGLAVNLITYEDRFNLYRIEQE

LGTEIKQIPPHIDQTIYCQ

>XP_034905043.1 DEAD-box ATP-dependent RNA helicase 8-like [Populus alba]

MNNNNRGRYPPGIGAGRGGGMNANPNFQSRVPQQQYVQRHFGQSHHQQQYNQHQQNHNQQQQQQQQQQHHQQQQWLRRSQ

LAAADSSVDEVEKTVQSEAVDSSSQDWKAKLKIPPADTRYQTEDVTATKGNDFEDYFLKRELLMGIYEKGFERPSPIQEE

SIPIALTGSDILARAKNGTGKTAAFCIPALEKIDQDNNFIQVVILVPTRELALQTSQVCKELGKHLKIQVMATTGGTSLK

DDIMRLYQPVHLLVGTPGRILDLAKKGVCILKDCSMLVLDEADKLLSPEFQPSIEQLIRFLPSNRQILMFSATFPVTVKD

FKDRYLEKPYVINLMDELTLKGITQYYAFVEERQKVHCLNTLFSKLQINQSIIFCNSVNRVELLAKKITELGYSCFYIHA

KMLQDHRNRVFHDFRNGACRNLVCTDLFTRGIDIQAVNVVINFDFPKNSETYLHRVGRSGRFGHLGLAVNLITYEDRFNL

YRIEQELGTEIKQIPPHIDQAIYCQ

>PIA49498.1 hypothetical protein AQUCO_01300357v1 [Aquilegia coerulea]

MNNRGRYPPGIGYGRGGYVNQNPNFQPRNPQQQYVQRNSMQNQQQYQQQQQQQQQQQQQQQQQWLRRNPMSGGDPGVNEV

EKTVQSETIDSSAQDWKARLKIPPSDTRYRTEDVTATKGNEFEDYFLKRELLMGIYEKGFERPSPIQEESIPIALTGSDI

LARAKNGTGKTAAFCIPALEKIDQENNVIQVVILVPTRELALQTSQVCKELGKHLKIQVMVTTGGTSLKDDIMRLYQPVH

LLVGTPGRILDLAKKGVCVLKDCSMLVMDEADKLLSPEFQPSVEQLIRFLPASRQILLFSATFPVTVKDFKDRYLQKPYI

INLMDELTLKGITQYYAFVEERQKVHCLNTLFSKLQINQSIIFCNSVNRVELLAKKITELGYSCFYIHAKMLQDHRNRVF

HDFRNGACRNLVCTDLFTRGIDIQAVNVVINFDFPKNSETYLHRVGRSGRFGHLGLAVNLITYEDRFNLYRIEQELGTEI

KQIPPQIDQAIYCR

>XP_006287568.1 DEAD-box ATP-dependent RNA helicase 8 [Capsella rubella]

MNNRGRYPPGIGAGRGATNPNPNYQSRPGYQQQPPPQYVQRGYAQNHQQAPSQPHQYQQQQQQQQQQWLRRAQIPPGGNS

NGDAVVEVEKTVQSEVIDPNSEDWKARLKLPAPDTRFRTEDVTATKGNEFEDYFLKRELLMGIYEKGFERPSPIQEESIP

IALTGRDILARAKNGTGKTAAFCIPVLEKIDQDNNVIQAVIIVPTRELALQTSQVCKELGKHLKIQVMVTTGGTSLKDDI

MRLYQPVHLLVGTPGRILDLAKKGVCVLKDCSVLVMDEADKLLSQEFQPSVEHLISFLPENRQILMFSATFPVTVKDFKD

RFLTNPYVINLMDELTLKGITQFYAFVEERQKIHCLNTLFSKLQINQSIIFCNSVNRVELLAKKITELGYSCFYIHAKML

QDHRNRVFHDFRNGACRNLVCTDLFTRGIDIQAVNVVINFDFPKNAETYLHRVGRSGRFGHLGLAVNLITYEDRFNLYRI

EQELGTEIKQIPPHIDQAIYCQ

>PIN19896.1 ATP-dependent RNA helicase [Handroanthus impetiginosus]

MMNNNYARGRYPPGMGNGWGGGGGGGGTPNHNYQNRNPHYPQLQPPYRQRTAESQPQQWMRRNPSASTVSESANEVEKTV

QSEAAGSTSQDWKGRPTIPPPDRRYKTEDVTATKGNEFEDYFLKRELLLGIYEKGFEKPSPIQEESIPIALTGSNILARA

KNGTGKTAAFCIPALEKIDSDKNVIQAVILVPTRELALQTSQVCKELGKHLKIQVMVTTGGTSLKDDIMRLYQPVHLLVG

TPGRILDLTRKGICNLNECSMLVMDEADKLLSPEFQPSIEQLISFLPPNRQMLMYSATFPVTVKDFKDKYLRKPYIINLM

DELTLKGITQYYAFVEERQKVHCLNTLFSKLQINQSIIFCNSVNRVELLAKKITELGYSCFYIHAKMLQDHRNKVFHEFR

NGACRNLVCTDLFTRGIDIQAVNVVINFDFPKNSETYLHRVGRSGRFGHLGLAVNLITYEDRFNLYRIEQELGAEIKQIP

SHIDHAIYCM

>XP_006576347.1 DEAD-box ATP-dependent RNA helicase 8 [Glycine max]

MNHNNNNRARYPPGMGIGRGGFNPNLGQNPSLNQNPNLNQNHHAFQARPPYHHQQQQPQYVQRHLLQPPPPQQQQQWLRR

DANAVDEVEKTVQSEPMDSSSQDWKARLKIPPADTRYRTEDVTATKGNEFEDYFLKRELLMGIYEKGFERPSPIQEESIP

IALTGSDILARAKNGTGKTAAFCIPALEKIDQDNNVIQVVILVPTRELALQTSQVCKELGKHLKIQVMVTTGGTSLKDDI

MRLYQPVHLLVGTPGRILDLAKKGVCILKDCAMLVMDEADKLLSPEFQPSIEQLIHFLPTTRQILMFSATFPVTVKDFKD

RYLRKPYVINLMDELTLKGITQFYAFVEERQKVHCLNTLFSKLQINQSIIFCNSVNRVELLAKKITELGYSCFYIHAKML

QDHRNRVFHDFRNGACRNLVCTDLFTRGIDIQAVNVVINFDFPKNAETYLHRVGRSGRFGHLGLAVNLITYEDRFNLYRI

EQELGTEIKQIPPQIDQAIYCR

>XP_010518223.1 PREDICTED: DEAD-box ATP-dependent RNA helicase 6-like [Camelina sativa]

MNNNNNNSRGRFPPGIGAAGPNFQSRTPNPNPPQQPQEYLQSRSPFPQQPQTQPPQFLQSQPDAHQFVQRSYPQTNPQQI

QQPQQQWSGGRAQLFSDPSYVDEVEKTVQSEANNDSNTQDWKATLKLPPRDERYQTEDVTATKGNEFEDYFLKRDLLRGI

YEKGFEKPSPIQEESIPIALTGSDILARAKNGTGKTGAFCIPTLEKIDPENNVIQAVILVPTRELALQTSQVCKELSKYL

NIEVMVTTGGTSLRDDIMRLYQPVHLLVGTPGRILDLSKKGVCVLKDCTMLVMDEADKLLSVEFQPSIEELIQFLPENRQ

ILMFSATFPVTVKSFKDRYLRKPYIINLMDQLTLVGVTQYYAFVEERQKVHCLNTLFSKLQINQSIIFCNSVNRVELLAK

KITELGYSCFYIHAKMVQDHRNRVFHDFRNGACRNLVCTDLFTRGIDIQAVNVVINFDFPRTSESYLHRVGRSGRYGHLG

LAVNLVTYEDRFKMYQTEQELGTEIKPIPSLIDKAIYCQ

>KAF1864022.1 hypothetical protein Lal_00031176 [Lupinus albus]

MNNSNRGRYPPGIGYGRGSGGGGFNQNPNQNQNAAFQSRNNFQQQQNQQQHYVQRNLVPQQNQQQQQQQWLRRAQLGGGA

DSNVVDEVEKNVQNEANDSRVPLLVANVLLYGVTLEKLGTCDSSQDWKARLKAPPADTRYKTEDVTATKGNEFEDYFLKR

ELLMGIYEKGFERPSPIQEESIPIALTGSDILARAKNGTGKTAAFCIPALEKIDQDTNVIQAVILVPTRELALQTSQVCK

ELGKHLKIEVMVTTGGTSLKDDIMRLYQPVHLLVGTPGRILDLAKKGVCILKDCTMLVMDEADKLLSPEFQPSIQHLIQF

LPSNRQILMFSATFPVTVKDFKDRYLHKPYVINLMDELTLKGITQFYAFVEERQKVHCLNTLFSKLQINQSIIFCNSVNR

VELLAKKITELGYSCFYIHAKMLQDHRNRVFHDFRNGACRNLVCTDLFTRGIDIQAVNVVINFDFPKNSETYLHRVGRSG

RFGHLGLAVNLITYEDRFNLYRIEQELGTEIKQIPPFIDQAVYCR

>XP_031390570.1 DEAD-box ATP-dependent RNA helicase 8-like [Punica granatum]

MNNRARYLPGIGAGRGGAVNGDPAFQSRPLQPQFPQQYVQRNQLGQQLYHQQQQQQQWLRRNQLGRSDSADEVEKTVQSE

AVGSSSADWKSNLRIPPADTRYQTEDVTATKGNEFEDYFLKRELLMGIYEKGFERPSPIQEESIPIALTGSNILARAKNG

TGKTAAFCIPTLEKIDQDNNVIQAMVLVPTRELALQTSQVCKELGKHLEIQVMVTTGGTSLRDDIMRLYQPVHLLVGTPG

RILDLAKKGVCILKDCSMLVMDEADKLLSPEFQPSVEQLIRFMPANRQILMFSATFPVTVKDFKDRYLRKPYIINLMDEL

TLKGITQYYAFVEERQKVHCLNTLFSKLQINQSIIFCNSVNRVELLAKKITELGYSCFYIHAKMLQDHRNRVFHDFRNGA

CRNLVCTDLFTRGIDIQAVNVVINFDFPKNSETYLHRVGRSGRFGHLGLAVNLITYEDRFTLYRMEQELGTEIKQIPPHI

DQAIYCR

>XP_011035912.1 PREDICTED: DEAD-box ATP-dependent RNA helicase 8 [Populus euphratica]

MNNNNRGRYPPGIGAGRGGGMSANPNFQSRVPQQQYVQRHFGQNHHQQQYNQHQQNHNQQQQQQQHQHQQQQWLRRNQLA

AADSSVDEVEKTVQSEAVDSSSQDWKAKLKIPPADTRYQTEDVTATKGNDFEDYFLKRELLMGIYEKGFESPSPIQEESI

PIALTGSDILARAKNGTGKTAAFCIPALEKIDQDNNFIQVVILVPTRELALQTSQVCKELGKHLKIQVMATTGGTSLKDD

IMRLYQPVHLLVGTPGRILDLAKKGVCILKDCSMLVLDEADKLLSPEFQPSIEQLIRFLPSNRQILMFSATFPVTVKDFK

DRYLEKPYVINLMDELTLKGITQYYAFVEERQKVHCLNTLFSKLQINQSIIFCNSVNRVELLAKKITELGYSCFYIHAKM

LQDHRNRVFHDFRNGACRNLVCTDLFTRGIDIQAVNVVINFDFPKNSETYLHRVGRSGRFGHLGLAVNLITYEDRFNLYR

IEQELGTEIKQIPPHIDQAIYCQ

>KAF0931688.1 hypothetical protein E2562_005685 [Oryza meyeriana var. granulata]

MDTRARYPPGIGNGRGGNPNYYNRGPPPPQQHNHHHQQQQTSAAHHQQYVQRQPQPPQHHHNHQQQHQQQQQQQQQQQWL

RRNQITREAAGTTRDSEPNAVALSQTADGIDSSSQDWKAQLKLPAQDTRYRTEDVTATKGNEFEDYFLKRELLMGIYEKG

FERPSPIQEESIPIALTGSDILARAKNGTGKTAAFCIPALEKIDQDKNAIQVVILVPTRELALQTSQVCKELGKHLKIQV

MVTTGGTSLKDDIIRLYQPVHLLVGTPGRILDLTKKGICILKDCSMLIMDEADKLLSPEFQPSVEQLIRYLPASRQILMF

SATFPVTVKEFKDKYLPKPYVINLMDELTLKGITQFYAFVEERQKVHCLNTLFSKLQINQSIIFCNSVNRVELLAKKITE

LGYSCFYIHAKMLQDHRNRVFHDFRNGACRNLVCTDLFTRGIDIQAVNVVINFDFPKNAETYLHRVGRSGRFGHLGLAVN

LITYEDRFNLYRIEQELGTEIKPIPPQIDQAIYCQ

>XP_003623900.1 DEAD-box ATP-dependent RNA helicase 8 isoform X2 [Medicago truncatula]

MNNNNRGRYPPGIGLGRGGSGGGGGGLTSNSNTGFQQRPHHQYQQQQQYVQRHMMQNQHQQHYQNQQQNQQQNQQQQQQQ

QWLRRNQLGGGTDTNVVEEVEKTVQSEANDSSSQDWKARLKLPPADTRYRTEDVTATKGNEFEDYFLKRELLMGIYEKGF

ERPSPIQEESIPIALTGSDILARAKNGTGKTAAFSIPALEKIDQDNNIIQVVILVPTRELALQTSQVCKELGKHLQIQVM

VTTGGTSLKDDIMRLYQPVHLLVGTPGRILDLAKKGVCVLKDCSMLVMDEADKLLSPEFQPSIEQLIQFLPPTRQILMFS

ATFPVTVKDFKDRYLRKPYIINLMDELTLKGITQFYAFVEERQKVHCLNTLFSKLQINQSIIFCNSVNRVELLAKKITEL

GYSCFYIHAKMLQDHRNRVFHDFRNGACRNLVCTDLFTRGIDIQAVNVVINFDFPKNSETYLHRVGRSGRFGHLGLAVNL

ITYEDRFNLYRIEQELGTEIKQIPPFIDQAIYCR

>RHN46904.1 putative RNA helicase [Medicago truncatula]

MNNNNRGRYPPGIGLGRGGSGGGGGLTSNSNTGFQQRPHHQYQQQQQYVQRHMMQNQHQQHYQNQQQNQQQNQQQQQQQQ

WLRRNQLGGGTDTNVVEEVEKTVQSEANDSSSQDWKARLKLPPADTRYRTEDVTATKGNEFEDYFLKRELLMGIYEKGFE

RPSPIQEESIPIALTGSDILARAKNGTGKTAAFSIPALEKIDQDNNIIQVVILVPTRELALQTSQVCKELGKHLQIQVMV

TTGGTSLKDDIMRLYQPVHLLVGTPGRILDLAKKGVCVLKDCSMLVMDEADKLLSPEFQPSIEQLIQFLPPTRQILMFSA

TFPVTVKDFKDRYLRKPYIINLMDELTLKGITQFYAFVEERQKVHCLNTLFSKLQINQSIIFCNSVNRVELLAKKITELG

YSCFYIHAKMLQDHRNRVFHDFRNGACRNLVCTDLFTRGIDIQAVNVVINFDFPKNSETYLHRVGRSGRFGHLGLAVNLI

TYEDRFNLYRIEQELGTEIKQIPPFIDQAIYCR

>RID43161.1 hypothetical protein BRARA_I00038 [Brassica rapa]

MNNNNNNRGRYPPGIGAGRGAINPNPNFQSRPGYQQQPPPQYVQRGGYAQQNHQQQFQQATSQQVQQQHGYQQQQQWLRR

PQISGGNNNGDAVAEVEKSVLSEAVDTNSEDWKARLKLPAPDTRFRTEDVTATKGNEFEDYFLKRELLMGIYEKGFERPS

PIQEESIPIALTGRDILARAKNGTGKTAAFCIPVLEKIDQDNNVIQAVIIVPTRELALQTSQVCKELGKHLKIQVMVTTG

GTSLKDDIMRLYQPVHLLVGTPGRILDLTKKGVCVLKDCSVFVMDEADKLLSQEFQPSVEHLISFLPQNRQILMFSATFP

VTVKDFKDRFLTNPYIINLMDELTLKGITQFYAFVEERQKIHCLNTLFSKLQINQSIIFCNSVNRVELLAKKITELGYSC

FYIHAKMLQDHRNRVFHDFRNGACRNLVCTDLFTRGIDIQAVNVVINFDFPKTAETYLHRVGRSGRFGHLGLAVNLITYE

DRFNLYRIEQELGTEIKQIPPHIDQAIYCQ

>XP_034926311.1 DEAD-box ATP-dependent RNA helicase 8-like [Populus alba]

MNNNNRGRYPPGIGAGRGGGMNANPNFQSRVPQQQYVQRNFGQNHHQQQYYQHQQHHNQQQQQQQWLRRNQLTAADSSVD

EVEKTVQSEAVDSSSQDWKAKLKIPPADTRYRTEDVTATKGNDFEDYFLKRELLMGIYEKGFERPSPIQEESIPIALTGS

DILARAKNGTGKTAAFCVPALEKIDQDNNFIQVVILVPTRELALQTSQVCKELGKHLKIQVMATTGGTSLKDDIMRLYQP

VHLLVGTPGRILDLAKKGVCVLKNCSMLVLDEADKLLSPEFQPSIEQLIRFLPSNRQILMFSATFPVTVKDFKDRYLEKP

YVINLMDELTLKGITQYYAFVEERQKVHCLNTLFSKLQINQSIIFCNSVNRVELLAKKITELGYSCFYIHAKMLQDHRNR

VFHDFRNGACRNLVCTDLFTRGIDIQAVNVVINFDFPKNSETYLHRVGRSGRFGHLGLAVNLITYEDRFNLYRIEQELGT

EIKQIPPHIDQAIYCQ

>RLM64539.1 hypothetical protein C2845_PM16G14880 [Panicum miliaceum]

MDPRARYPPGIGNGRGGNPNYYGRGPPPPQQQHHHQRPPPSQAHHQQYMQRQPQPQRQPSQHLNQQQQQQQWHRRNQIAA

EAAGASAQRAPPAVDGIDSSSQDWKAQLKLPPPDTRYRTEDVTATKGNDFEDYFLKRELLMGIYEKGFEKPSPIQEESIP

IALTGSDILARAKNGTGKTAAFCIPALEKIDQDKNAIQVVILVPTRELALQTSQVCKELGKHLRIQVMVTTGGTSLKDDI

IRLYQPVHLLVGTPGRILDLTKKGICMLKDCSMLIMDEADKLLSPEFQPSIEQLIRYLPSNRQILMFSATFPVTVKEFKD

KYLPKPYVINLMDELTLKGITQFYAFVEERQKVHCLNTLFSKLQINQSIIFCNSVNRVELLAKKITELGYSCFYIHAKML

QDHRNRVFHDFRNGACRNLVCTDLFTRGIDIQAVNVVINFDFPKNAETYLHRVGRSGRFGHLGLAVNLITYEDRFNLYRI

EQELGTEIKPIPPQIDRTIYCQ

>XP_020878086.1 DEAD-box ATP-dependent RNA helicase 8 [Arabidopsis lyrata subsp. lyrata]

MNNRGRYPPGIGAGRGASNPNPNYHSRPGYQQQQPPPQYVQRGGYAQNHQQQFQQSPTQPHQYQQQQQQQQWLRRAQIPG

GNSNSNGDAVVEVEKTVQSEVIDPNSEDWKARLKLPAPDTRYRTEDVTATKGNEFEDYFLKRELLMGIYEKGFERPSPIQ

EESIPIALTGRDILARAKNGTGKTAAFCIPVLEKIDQDNNVIQAVIIVPTRELALQTSQVCKELGKHLKIQVMVTTGGTS

LKDDIMRLYQPVHLLVGTPGRILDLAKKGVCVLKDCSVLVMDEADKLLSQEFQPSVEHLISFLPENRQILMFSATFPVTV

KDFKDRFLTNPYVINLMDELTLKGITQFYAFVEERQKIHCLNTLFSKLQINQSIIFCNSVNRVELLAKKITELGYSCFYI

HAKMLQDHRNRVFHDFRNGACRNLVCTDLFTRGIDIQAVNVVINFDFPKNAETYLHRVGRSGRFGHLGLAVNLITYEDRF

NLYRIEQELGTEIKQIPPHIDQAIYCQ

>XP_006294022.1 DEAD-box ATP-dependent RNA helicase 6 [Capsella rubella]

MNNNNNRGRFPPGIGAAGPGPDPNFQSRNPNPNPQQPQPHQYLQSRTPFPQQPQPQYLQSQPNAQQYVQRSYPQTNPQQI

QQQQQQWSGRPQLPTDPSYVDEVEKTVQSEANNDSNTEDWKATLKLPPRDDRYQTEDVTATKGNEFEDYFLKRDLLRGIY

EKGFEKPSPIQEESIPIALTGSDILARAKNGTGKTGAFCIPTLEKIDPENNVIQAVILVPTRELALQTSQVCKELSKYLN

IEVMVTTGGTSLRDDIMRLYQPVHLLVGTPGRILDLAKKGVCVLKDCSMLVMDEADKLLSVEFQPSIEELIQFLPENRQI

LMFSATFPVTVKSFKDRYLRKPYIINLMDQLTLMGVTQYYAFVEERQKVHCLNTLFSKLQINQSIIFCNSVNRVELLAKK

ITELGYSCFYIHAKMVQDHRNRVFHDFRNGACRNLVCTDLFTRGIDIQAVNVVINFDFPRTSESYLHRVGRSGRYGHLGL

AVNLVTYEDRFKMYQTEQELGTEIKPIPSLIDKAIYCQ

>XP_013600085.1 PREDICTED: DEAD-box ATP-dependent RNA helicase 12 isoform X2 [Brassica oleracea var. oleracea]

MNNNRGRYPPGMGTGRGAPPNPDYQSQNHHQQQWTRRAQLPGNANEVQKTTSPPPVASSDPKLCSPFTLSGQDWKATLRL

PPPDTRYQTADVTATKGNEFEDYFLKRDLLKGIYEKGFEKPSPIQEESIPIALTGSDILARAKNGTGKTGAFCIPVLERI

DPNTNVIQAMILVPTRELALQTSQVCKELSKYLNIQVMVTTGGTSLRDDIMRLHQPVHLLVGTPGRILDLTKKGVCVLKD

CTMLVMDEADKLLSAEFQPSLEELIQFLPQNRQFLMYSATFPVTVKAFKDRHLRKPYVINLMDQLTLMGVTQYYAFVEER

QKVHCLNTLFSKLQINQSIIFCNSVNRVELLAKKITELGYSCFYIHAKMVQDHRNRVFHEFRNGACRNLVCTDLFTRGID

IQAVNVVINFDFPRTSESYLHRVGRSGRFGHLGLAVNLVTYEDRFKMYQTEQELGTEIKPIPSNIDQAIYCQ

>XP_020689006.1 DEAD-box ATP-dependent RNA helicase 8-like [Dendrobium catenatum]

MNPRGRYPPGIGNGRGGSVEPNVNFYPRGHQLQQHYVERNPAQSQQNQQFQHQQQQWLRRNQMERHSGSNELVKPVPSDA

IDSSSQDWKAQLRLPAADTRYKTEDVTATKGNDFEDYFLKRELLMGIYEKGFERPSPIQEESIPIALTGRDILARAKNGT

GKTAAFCIPALEKIDQDKNVIQVVILVPTRELALQTSQVCKELAKHLKVQVMVTTGGTSLKDDVMRLYQPVHLIAGTPGR

ILDLAKKGVCVLQDCSMLIMDEADKLLSPEFQPSIEQLIHFLPENRQILLFSATFPVTVKDFKDRYLRKPYIINLMDELT

LKGITQYYAFVEERQKVHCLNTLFSKLQINQSIIFCNSVNRVELLAKKITELGFSCFYIHAKMLQDHRNRVFHDFRNGAC

RNLVCTDLFTRGIDIQAVNVVINFDFPKNSETYLHRVGRSGRFGHLGLAVNLITYEDRFNLYRIEQELGTEIKQIPSQID

QAIYCS

>VAI58940.1 unnamed protein product [Triticum turgidum subsp. durum]

MDPRARYPPGTGNGRGGNPNYYGRGPPLSQNNHHHHQQTSAAHQQQYVQRQPQPQPQQHHQQQQQQQQWLRRNQITAAGT

SGPKVVAPPPAGVGNDPSSQDWKAQLKLPPADTRFRTEDVTATKGNEFEDYFLKRELLMGIYEKGFENPSPIQEESIPIA

LTGSDILARAKNGTGKTAAFCIPALEKIDQDKNAIQVVIVVPTRELALQTSQVCKELGKHLKIQVMVTTGGTSLKDDIVR

LYQPVHLLVGTPGRILDLTKKGVCILKDCSMLVMDEADKLLSPEFQPSIEQLIRYLPASRQILMFSATFPVTVKEFKDKY

LPKPYVINLMDELTLKGITQFYAFVEERQKVHCLNTLFSKLQINQSIIFCNSVNRVELLAKKITELGYSCFYIHAKMLQD

HRNRVFHDFRNGACRNLVCTDLFTRGIDIQAVNVVINFDFPKSSETYLHRVGRSGRFGHLGLAVNLITYEDRFNLYRIEQ

ELGTEIKQIPPQIDQAIYCQ

>VAI58941.1 unnamed protein product [Triticum turgidum subsp. durum]

MDPRARYPPGTGNGRGGNPNYYGRGPPLSQNNHHHHQQTSAAHQQQYVQRQPQHHHQQQQQQQWLRRNQITAAGTSGPKV

VAPPPAGVGNDPSSQDWKAQLKLPPADTRFRTEDVTATKGNEFEDYFLKRELLMGIYEKGFENPSPIQEESIPIALTGSD

ILARAKNGTGKTAAFCIPALEKIDQDKNAIQVVIVVPTRELALQTSQVCKELGKHLKIQVMVTTGGTSLKDDIVRLYQPV

HLLVGTPGRILDLTKKGVCILKDCSMLVMDEADKLLSPEFQPSIEQLIRYLPASRQILMFSATFPVTVKEFKDKYLPKPY

VINLMDELTLKGITQFYAFVEERQKVHCLNTLFSKLQINQSIIFCNSVNRVELLAKKITELGYSCFYIHAKMLQDHRNRV

FHDFRNGACRNLVCTDLFTRGIDIQAVNVVINFDFPKSSETYLHRVGRSGRFGHLGLAVNLITYEDRFNLYRIEQELGTE

IKQIPPQIDQAIYCQ

>XP_006402503.1 DEAD-box ATP-dependent RNA helicase 12 [Eutrema salsugineum]

MNNRGRYPPGLGTGRGAAPNPDYQSYRQQPQAQHHVQRGQPQNPQQFQLQQQQQQQWSRRAQLPGNANEVQKTSQPVATS

DPNGQDWKATLRLPPPDTRYQTADVTATKGNEFEDYFLKRDLLKGIYEKGFEKPSPIQEESIPIALTGSDILARAKNGTG

KTGAFCIPVLEKIDPKNNVIQAMILVPTRELALQTSQVCKELSKYLNIQVMVTTGGTSLRDDIMRLHQPVHLLVGTPGRI

LDLTKKGVCVLKDCTMLVMDEADKLLSAEFQPSLEELIQFLPQNRQFLMFSATFPVTVKAFKDRHLRKPYVINLMDQLTL

MGITQFYAFVEERQKVHCLNTLFSKLQINQSIIFCNSVNRVELLAKKITELGYSCFYIHAKMVQDHRNRVFHEFRNGACR

NLVCTDLFTRGIDIQAVNVVINFDFPRTSESYLHRVGRSGRFGHLGLAVNLVTYEDRFKMYQTEQELGTEIKPIPSLIDQ

AIYCQ

>XP_027349617.1 DEAD-box ATP-dependent RNA helicase 8-like [Abrus precatorius]

MNNRARYPPGIGLGRGGGGLNNTNPGFQPRPHQQQYVQRHMVPHQHQHQHQHQNQYQQQYQQHHQHQQQQQQQQWLRRTQ

LGGTDTNAVEEVEKTVQSEAVDPSSQDWKARLKIPPPDTRYKTEDVTATKGNEFEDYFLKRELLMGIYEKGFERPSPIQE

ESIPIALTGSDILARAKNGTGKTAAFCIPALEKIDQDNNVIQVVILVPTRELALQTSQVCKELGKHLKIQVMVTTGGTSL

KDDIMRLYQPVHLLVGTPGRILDLAKKGVCILKDCSMLVMDEADKLLSPEFQPSVEQLIQFLPSNRQILMFSATFPVTVK

DFKDRYLRKPYVINLMDELTLKGITQFYAFVEERQKVHCLNTLFSKLQINQSIIFCNSVNRVELLAKKITELGYSCFYIH

AKMLQDHRNRVFHDFRNGACRNLVCTDLFTRGIDIQAVNVVINFDFPKNSETYLHRVGRSGRFGHLGLAVNLITYEDRFN

LYRIEQELGTEIKQIPPHIDQAIYCR

>VAI58939.1 unnamed protein product [Triticum turgidum subsp. durum]

MDPRARYPPGTGNGRGGNPNYYGRGPPLSQNNHHHHQQTSAAHQQQYVQRQPQQQHHQNHHHHQQQQQQQWLRRNQITAA

GTSGPKVVAPPPAGVGNDPSSQDWKAQLKLPPADTRFRTEDVTATKGNEFEDYFLKRELLMGIYEKGFENPSPIQEESIP

IALTGSDILARAKNGTGKTAAFCIPALEKIDQDKNAIQVVIVVPTRELALQTSQVCKELGKHLKIQVMVTTGGTSLKDDI

VRLYQPVHLLVGTPGRILDLTKKGVCILKDCSMLVMDEADKLLSPEFQPSIEQLIRYLPASRQILMFSATFPVTVKEFKD

KYLPKPYVINLMDELTLKGITQFYAFVEERQKVHCLNTLFSKLQINQSIIFCNSVNRVELLAKKITELGYSCFYIHAKML

QDHRNRVFHDFRNGACRNLVCTDLFTRGIDIQAVNVVINFDFPKSSETYLHRVGRSGRFGHLGLAVNLITYEDRFNLYRI

EQELGTEIKQIPPQIDQAIYCQ

>CAA0392829.1 unnamed protein product [Arabidopsis thaliana]

MNNRGRYPPGIGAGRGAFNPNPNYQSRSGYQQHPTPQYVQRGNYAQNHQQQFQQAPSQPHQYQQQQQQQQQWLRRGQIPG

GNSNGDAVVEVEKTVQSEVIDPNSEDWKARLKLPAPDTRYRTEDVTATKGNEFEDYFLKRELLMGIYEKGFERPSPIQEE

SIPIALTGRDILARAKNGTGKTAAFCIPVLEKIDQDNNVIQAVIIVPTRELALQTSQVCKELGKHLKIQVMVTTGGTSLK

DDIMRLYQPVHLLVGTPGRILDLTKKGVCVLKDCSVLVMDEADKLLSQEFQPSVEHLISFLPESRQILMFSATFPVTVKD

FKDRFLTNPYVINLMDELTLKGITQFYAFVEERQKIHCLNTLFSKLQINQSIIFCNSVNRVELLAKKITELGYSCFYIHA

KMLQDHRNRVFHDFRNGACRNLVCTDLFTRGIDIQAVNVVINFDFPKNAETYLHRVGRSGRFGHLGLAVNLITYEDRFNL

YRIEQELGTEIKQIPPHIDQAIYCQ

>NP_191975.2 RNAhelicase-like 8 [Arabidopsis thaliana]

MNNRGRYPPGIGAGRGAFNPNPNYQSRSGYQQHPPPQYVQRGNYAQNHQQQFQQAPSQPHQYQQQQQQQQQWLRRGQIPG

GNSNGDAVVEVEKTVQSEVIDPNSEDWKARLKLPAPDTRYRTEDVTATKGNEFEDYFLKRELLMGIYEKGFERPSPIQEE

SIPIALTGRDILARAKNGTGKTAAFCIPVLEKIDQDNNVIQAVIIVPTRELALQTSQVCKELGKHLKIQVMVTTGGTSLK

DDIMRLYQPVHLLVGTPGRILDLTKKGVCVLKDCSVLVMDEADKLLSQEFQPSVEHLISFLPESRQILMFSATFPVTVKD

FKDRFLTNPYVINLMDELTLKGITQFYAFVEERQKIHCLNTLFSKLQINQSIIFCNSVNRVELLAKKITELGYSCFYIHA

KMLQDHRNRVFHDFRNGACRNLVCTDLFTRGIDIQAVNVVINFDFPKNAETYLHRVGRSGRFGHLGLAVNLITYEDRFNL

YRIEQELGTEIKQIPPHIDQAIYCQ

>VAI58942.1 unnamed protein product [Triticum turgidum subsp. durum]

MDPRARYPPGTGNGRGGNPNYYGRGPPLSQNNHHHHQQTSAAHQQQYVQRQPQPQPQQHHQKQQQWLRRNQITAAGTSGP

KVVAPPPAGVGNDPSSQDWKAQLKLPPADTRFRTEDVTATKGNEFEDYFLKRELLMGIYEKGFENPSPIQEESIPIALTG

SDILARAKNGTGKTAAFCIPALEKIDQDKNAIQVVIVVPTRELALQTSQVCKELGKHLKIQVMVTTGGTSLKDDIVRLYQ

PVHLLVGTPGRILDLTKKGVCILKDCSMLVMDEADKLLSPEFQPSIEQLIRYLPASRQILMFSATFPVTVKEFKDKYLPK

PYVINLMDELTLKGITQFYAFVEERQKVHCLNTLFSKLQINQSIIFCNSVNRVELLAKKITELGYSCFYIHAKMLQDHRN

RVFHDFRNGACRNLVCTDLFTRGIDIQAVNVVINFDFPKSSETYLHRVGRSGRFGHLGLAVNLITYEDRFNLYRIEQELG

TEIKQIPPQIDQAIYCQ

>XP_012843679.1 PREDICTED: DEAD-box ATP-dependent RNA helicase 8 [Erythranthe guttata]

MNSRARYPPPGMGAGRGGGGGGGGGGMNPHAGPNPNFQPRYANQQYAERSPARNNQNQELFQNPQPQQWLRRNQLPPVDS

AVDEVEKTVQSEAVDSSSQDWKGRLKLPPVDTRYRTEDVTATKGNEFEDYFLKRELLMGIYEKGFERPSPIQEESIPIAL

TGSDILARAKNGTGKTAAFCIPALEKIDQDKNVIQAVILVPTRELALQTSQVCKELAKHLQIQVMVTTGGTSLRDDIMRL

HQPVHLLVGTPGRILDLARKGICILNECNMLVMDEADKLLSPEFQPSIEQLIRFLSANRQILMFSATFPVTVKDFKDRYL

RKPYVINLMDELTLKGITQFYAFVEERQKVHCLNTLFSKLQINQSIIFCNSVNRVELLAKKITELGYSCFYIHAKMLQDH

RNRVFHDFRNGACRNLVCTDLFTRGIDIQSVNVVINFDFPKNAETYLHRVGRSGRFGHLGLAVNLITYEDRFNLYRIEQE

LGTEIKQIPPQIDQAIYCQ

>KAE8733333.1 DEAD-box ATP-dependent RNA helicase 6 [Hibiscus syriacus]

MNSRGRYPPGIGAGRGGGVNASPSFQSRPSQQQYVQRNLVQNHQHFQQQQQQQHHHQQQLWLRRNQLPGGNDSNVVDEVE

KTVQSEAVDSSSQDWKARLKMPPQDTRYKTEDVTATKGNEFEDYFLKRELLMGIYEKGFERPSPIQEESIPIALTGSDIL

ARAKNGTGKTAAFCIPTLEKIDQDNNVIQAVILVPTRELALQTSQVCKELGKHLQIQVMVTTGGTSLKDDIMRLYQPVHL

LVGTPGRILDLAKKGVCILKDCSMLIMDEADKLLSPEFQPSIEQLIQFLPANRQILMFSATFPVTVKDFKDRYLKKPYII

NLMDELTLKGITQYYAFVEERQKVHCLNTLFSKLQINQSIIFCNSVNRVELLAKKITELGYSCFYIHAKMLQDHRNRVFH

DFRNGACRNLVCTDLFTRGIDIQAVNVVINFDFPKNSETYLHRVGRSGRFGHLGLAVNLITYEDRFNLYRIEQELGTEIK

QIPPHIDQAIYCR

>XP_011023369.1 PREDICTED: DEAD-box ATP-dependent RNA helicase 8-like [Populus euphratica]

MNNNNRGRYPPGIGAGRGGGMNANPNFQSRVPQQQYVQRNFGQNHHQQQYYQHQQHQQHHNQQQQQQQQWLRRNQLTAAD

SNVDEVEKTVQSEAVDSSSQDWKAKLKIPPADTRYRTEDVTATKGNDFEDYFLKRELLMGIYEKGFERPSPIQEESIPIA

LTGSDILARAKNGTGKTAAFCVPALEKIDQDNNFIQVVILVPTRELALQTSQVCKELGKHLKIQVMATTGGTSLKDDIMR

LYQPVHLLVGTPGRILDLAKKGVCILKNCSMLVLDEADKLLSPEFQPSIEQLIRFLPSNRQILMFSATFPVTVKDFKDRY

LEKPYVINLMDELTLKGITQYYAFVEERQKVHCLNTLFSKLQINQSIIFCNSVNRVELLAKKITELGYSCFYIHAKMLQD

HRNRVFHDFRNGACRNLVCTDLFTRGIDIQAVNVVINFDFPKNSETYLHRVGRSGRFGHLGLAVNLITYEDRFNLYRIEQ

ELGTEIKQIPPHIDQAIYCQ

>KAF7085059.1 hypothetical protein CFC21_088547 [Triticum aestivum]

MDPRARYPPGTGNGRGGNPNYYGRGPPLSQNNHHHHQQTSAAHQQQYVQRQPQPQPQQHHQNNHQQQHHQNNHQQQHHQN

HHHHQQQQQQQWLRRNQITAAGTSGPKVVAPPPAGVGNDPSSQDWKAQLKLPPADTRFRTEDVTATKGNEFEDYFLKREL

LMGIYEKGFENPSPIQEESIPIALTGSDILARAKNGTGKTAAFCIPALEKIDQDKNAIQVVIVVPTRELALQTSQVCKEL

GKHLKIQVMVTTGGTSLKDDIVRLYQPVHLLVGTPGRILDLTKKGVCILKDCSMLVMDEADKLLSPEFQPSIEQLIRYLP

ASRQILMFSATFPVTVKEFKDKYLPKPYVINLMDELTLKGITQFYAFVEERQKVHCLNTLFSKLQINQSIIFCNSVNRVE

LLAKKITELGYSCFYIHAKMLQDHRNRVFHDFRNGACRNLVCTDLFTRGIDIQAVNVVINFDFPKSSETYLHRVGRSGRF

GHLGLAVNLITYEDRFNLYRIEQELGTEIKQIPPQIDQAIYCQ

>GEW95977.1 DEAD-box ATP-dependent RNA helicase 8-like [Tanacetum cinerariifolium]

MSTXYGRKCRSPIRWVKFRDVQLTGPEIIHETTEKIMQIRQRLQAARDRQRSYANRGKVNPWYIGPFKILKRVGPVAYAL

ELPKELSNIYNTFHIFNLKKCLSDESFVIPMKELRLDDKLNFVEKPIEIMDREVKQLKQSRIPIVKVRWNSKSGPEFTWE

REDQIRAKXGGYQYQSNPNNYQNQQPRNPNQYQQQYQQRQPQQQQYQQRQQQQQQWLRLNANNANTTTTGSSSSNANDGA

TYEVEKTVTTVDSSSQDWKAQLNIPAADSRFKTEDVTATKGNEFEDYFLKRELLMGIYEKGFERPSPIQEESIPIALTGS

DILARAKNGTGKTAAFCIPALEKIDTEKNAIQVVILVPTRELALQTSQVCKELGKHLNIQVMVTTGGTSLKDDIMRLYQP

VHLLVGTPGRILDLTKKQICKLDNCTMLVMDEADKLLSPEFQPSVEHLISFLPENRQILMFSATFPVTVKDFKDRYLKKP

YVVNLMDELTLKGITQFYAFVEERQKVHCLNTLFSKLQINQSIIFCNSVNRVELLAKKITELGYSCFYIHAKMLQDHRNR

VFHDFRNGACRNLVCTDLFTRGIDIQAVNVVINFDFPRNAETYLHRVGRSGRFGHLGLAVNLITYEDRFNLYRIEQELGT

EIRQIPPQIDQAIYCQ

>XP_030540898.1 DEAD-box ATP-dependent RNA helicase 8-like [Rhodamnia argentea]

MMNNRARYPPGIGAGRGVNAGPAFQSRTPQPQYVQRGQLGQPPQYQQNHHHQQQQQHQQQQHQQWLRRTQLGGADSTVDE

VEKTVTVQSEAIDSSSQDWKARLKIPPPDTRYKTEDVTATKGNEFEDYFLKRELLMGIYEKGFERPSPIQEESIPIALTG

SDILARAKNGTGKTAAFCIPALEKIDQDNNVIQVVILVPTRELALQTSQVCKELGKHLQIQVMVTTGGTSLKDDIMRLYQ

PVHLLVGTPGRILDLSKKGVCILKDCSMLVMDEADKLLSPEFQPSVEQLIRFLPGNRQILMYSATFPVTVKDFKDRYLQK

PYVINLMDELTLKGITQYYAFVEERQKVHCLNTLFSKLQINQSIIFCNSVNRVELLAKKITELGYSCFYIHAKMLQDHRN

RVFHDFRNGACRNLVCTDLFTRGIDIQAVNVVINFDFPKNAETYLHRVGRSGRFGHLGLAVNLITYEDRFNLYRIEQELG

TEIKQIPPHIDQAIYCR

>CAD5326725.1 unnamed protein product [Arabidopsis thaliana]

MNNRGRYPPGIGAGRGAFNPNPNYQSRSGYQQHPPPQYVQRGNYAQNHQQQFQQAPSQPHQYEQQQQQQQQWLRRGQIPG

GNSNGDAVVEVEKTVQSEVIDPNSEDWKARLKLPAPDTRYRTEDVTATKGNEFEDYFLKRELLMGIYEKGFERPSPIQEE

SIPIALTGRDILARAKNGTGKTAAFCIPVLEKIDQDNNVIQAVIIVPTRELALQTSQVCKELGKHLKIQVMVTTGGTSLK

DDIMRLYQPVHLLVGTPGRILDLTKKGVCVLKDCSVLVMDEADKLLSQEFQPSVEHLISFLPESRQILMFSATFPVTVKD

FKDRFLTNPYVINLMDELTLKGITQFYAFVEERQKIHCLNTLFSKLQINQSIIFCNSVNRVELLAKKITELGYSCFYIHA

KMLQDHRNRVFHDFRNGACRNLVCTDLFTRGIDIQAVNVVINFDFPKNAETYLHRVGRSGRFGHLGLAVNLITYEDRFNL

YRIEQELGTEIKQIPPHIDQAIYCQ

>OVA14083.1 Helicase [Macleaya cordata]

MNPRGRYPPGIGNGRGGYVNSNPNFQPRNPQQHYVQRNPVQNQQQFHQQQQQWLRRNPMGNDSSVNEVEKAVQSETIDSS

SQDWKARLKIPPSDTRYRTEDVTATKGNEFEDYFLKRELLMGIYEKGFERPSPIQEESIPIALTGSDILARAKNGTGKTA

AFCIPALEKIDQDNNVIQVVILVPTRELALQTSQVCKELGKHLKIQVMVTTGGTSLKDDIMRLYQPVHLLVGTPGRILDL

AKKGVCVLKDCSMLVMDEADKLLSPEFQPSVEQLIRFLPASRQILLFSATFPVTVKDFKDRYLQKPYIINLMDELTLKGI

TQYYAFVEERQKVHCLNTLFSKLQINQSIIFCNSVNRVELLAKKITELGYSCFYIHAKMLQDHRNRVFHDFRNGACRNLV

CTDLFTRGIDIQAVNVVINFDFPKNSETYLHRVGRSGRFGHLGLAVNLITYEDRFNLYRIEQELGTEIKQIPPQIDQAIY

CR

>XP_020273140.1 DEAD-box ATP-dependent RNA helicase 8-like [Asparagus officinalis]

MNNRGRYPPGIGDGGGRGAGVGNPNPSYYGRIPPQQQQQQQQQQYVQRNLMQYQQQQNQQQQQQWLRRNQIGSDSGGSES

NRAVQSASIDTSSPDWKSQLKIPPPDTRYRTEDVTATKGNEFEDYFLKRELLMGIYEKGFERPSPIQEESIPIALTGSDI

LARAKNGTGKTAAFCIPALEKIDQDKNVIQVVILVPTRELALQTSQVCKELGKHLKIQVMATTGGTSLKDDIMRLYQPVH

LLVGTPGRILDLTKKGVCVLKDCSMLIMDEADKLLSHEFQPSIEQLIHFLPSNRQILLFSATFPVTVKDFKDRYLPKPYI

INLMDELTLKGITQYYAFVEERQKVHCLNTLFSKLQINQSIIFCNSVNRVELLAKKITELGYSCFYIHAKMLQDHRNRVF

HDFRNGACRNLVCTDLFTRGIDIQAVNVVINFDFPKNSETYLHRVGRSGRYGHLGLAVNLITYEDRFNLYRIEQELGTEI

KQIPPQIDQAIYCR

>XP_010506559.1 PREDICTED: DEAD-box ATP-dependent RNA helicase 6 [Camelina sativa]

MNNNNSNNRGRFPPGIGAAGPNFQSRTPNPNPPPPQPQEYLQSRSPFPQQSQTQPPQYLQSQPDAHQFVQRSYPQTNPQQ

IQQPQQQWSGGRAQLPSDPSYVDEVEKTVQSEANNDSNTQDWKATLKLPPRDDRYQTEDVTATKGNEFEDYFLKRDLLRG

IYEKGFEKPSPIQEESIPIALTGSDILARAKNGTGKTGAFCIPTLEKIDPENNVIQAVILVPTRELALQTSQVCKELSKY

LNIEVMVTTGGTSLRDDIMRLYQPVHLLVGTPGRILDLSKKGVCVLKDCTMLVMDEADKLLSVEFQPSIEELIQFLPENR

QILMFSATFPVTVKSFKDRYLRKPYIINLMDQLTLVGVTQYYAFVEERQKVHCLNTLFSKLQINQSIIFCNSVNRVELLA

KKITELGYSCFYIHAKMVQDHRNRVFHDFRNGACRNLVCTDLFTRGIDIQAVNVVINFDFPRTSESYLHRVGRSGRYGHL

GLAVNLVTYEDRFKMYQTEQELGTEIKPIPSLIDKAIYCQ

>XP_003623903.1 DEAD-box ATP-dependent RNA helicase 8 isoform X1 [Medicago truncatula]

MNNNNRGRYPPGIGLGRGSGGGGLNSNPNNANAGFQQRPHYQQQQYVQRHLMQNQNQHQQHYQHHQQNQQQYQQQNQQQQ

QQQWLRRNQLGGGTDTNVVEEVEKTVQSETNDPSSQDWKEKLKLPPADTRYRTEDVTATKGNEFEDYFLKRELLMGIYEK

GFERPSPIQEESIPIALTGSDILARAKNGTGKTAAFSIPALEKIDQDNNIIQVVILVPTRELALQTSQVCKELGKHLQIQ

VMVTTGGTSLKDDIMRLYQPVHLLVGTPGRILDLAKKGVCVLKDCSMLVMDEADKLLSPEFQPSIEQLIQFLPPTRQILM

FSATFPVTVKDFKDRYLRKPYIINLMDELTLKGITQFYAFVEERQKVHCLNTLFSKLQINQSIIFCNSVNRVELLAKKIT

ELGYSCFYIHAKMLQDHRNRVFHDFRNGACRNLVCTDLFTRGIDIQAVNVVINFDFPKNSETYLHRVGRSGRFGHLGLAV

NLITYEDRFNLYRIEQELGTEIKQIPPFIDQAVYCR

>RID47637.1 hypothetical protein BRARA_I04217 [Brassica rapa]

MNNNRGRYQPGTGTGRGAYQQQPQAQQHVQRGQPQNHQQQQWSRRAQLPGNATNANEIQTSSSQPPVASSDPNGQDWKAS

LRLPPPDTRYQTADVTATKGNEFEDYFLKRDLLKGIYEKGFEKPSPIQEESIPIALTGSDILARAKNGTGKTGAFCIPVL

ERIDPNTNVIQAMILVPTRELALQTSQVCKELSKYLNIHVMVTTGGTSLRDDIMRLHQPVHLLVGTPGRILDLTKKGVCV

LKDCTMLVMDEADKLLSAEFQPSLEELIQFLPQNRQFLMYSATFPVTVKAFKDRHLRKPYVINLMDQLTLMGITQYYAFV

EERQKVHCLNTLFSKLQINQSIIFCNSVNRVELLAKKITELGYSCFYIHAKMVQDHRNRVFHEFRNGACRNLVCTDLFTR

GIDIQAVNVVINFDFPRTSESYLHRVGRSGRFGHLGLAVNLVTYEDRFKMYQTEQELGTEIKPIPSNIDQAIYCQ

>TEY29713.1 ATP-dependent RNA helicase DDX6/DHH1 [Salvia splendens]

MNPNAGPNPNFQPRNPMHQYVQRSPAPSNQNQQLFQNQQQQQQWLRRNQLPPTDSAVDEVEKTVQSEAVDSSSQDWKASL

KLPPRDNRYRTEDVTATKGNEFEDYFLKRELLMGIYEKGFESPSPIQEESIPIALTGSDILARAKNGTGKTAAFCIPALE

KIDQDKNVIQVVILVPTRELALQTSQVCKELGKHLQIQVMATTGGTSLKDDIMRLYQPVHLLVGTPGRILDLANKNVCVL

NECSMLIMDEADKLLSPEFQPSIEQLIRFMPVNRQILMFSATFPVTVKDFKDRYLRKPYIINLMDELTLKGITQFYAFVE

ERQKVHCLNTLFSKLQINQSIIFCNSVNRVELLAKKITELGYSCFYIHAKMLQDHRNRVFHDFRNGACRNLVCTDLFTRG

IDIQAVNVVINFDFPKNSETYLHRVGRSGRFGHLGLAVNLITYEDRFNLYRIEQELGTEIKQIPPQIDQAVYCQ

>XP_010508105.1 PREDICTED: DEAD-box ATP-dependent RNA helicase 6 [Camelina sativa]

MNNNISRGRFPPGIGAAGPNFQSRTPNPNPTQQPHPQEYLQSRSPFPQQPQTQPPQYLQSQPDAHQFVQRAYPQTNPHQI

QQQQQQQWSGGRAQLPSDPSYVDEVEKTVQSEANNDSNTQDWKATVKLPPRDDRYQTEDVTATKGNEFEDYFLKRDLLRG

IYEKGFEKPSPIQEESIPIALTGSDILARAKNGTGKTGAFCIPTLEKIDPENNVIQAVILVPTRELALQTSQVCKELSKY

LKIEVMVTTGGTSLRDDIMRLYQPVHLLVGTPGRILDLSKKGVCVLKDCTMLVMDEADKLLSVEFQPSIEELIQFLPENR

QILMFSATFPVTVKSFKDRYLRKPYIINLMDQLTLVGVTQYYAFVEERQKVHCLNTLFSKLQINQSIIFCNSVNRVELLA

KKITELGYSCFYIHAKMVQDHRNRVFHDFRNGACRNLVCTDLFTRGIDIQAVNVVINFDFPRTSESYLHRVGRSGRYGHL

GLAVNLVTYEDRFKMYQTEQELGTEIKPIPSLIDKAIYCQ

>XP_013600086.1 PREDICTED: DEAD-box ATP-dependent RNA helicase 12 isoform X3 [Brassica oleracea var. oleracea]

MNNNRGRYPPGMGTGRGAPPNPDYQSQNHHQQQWTRRAQLPGNANEVQKTTSPPPVASSDPNGQDWKATLRLPPPDTRYQ

TADVTATKGNEFEDYFLKRDLLKGIYEKGFEKPSPIQEESIPIALTGSDILARAKNGTGKTGAFCIPVLERIDPNTNVIQ

AMILVPTRELALQTSQVCKELSKYLNIQVMVTTGGTSLRDDIMRLHQPVHLLVGTPGRILDLTKKGVCVLKDCTMLVMDE

ADKLLSAEFQPSLEELIQFLPQNRQFLMYSATFPVTVKAFKDRHLRKPYVINLMDQLTLMGVTQYYAFVEERQKVHCLNT

LFSKLQINQSIIFCNSVNRVELLAKKITELGYSCFYIHAKMVQDHRNRVFHEFRNGACRNLVCTDLFTRGIDIQAVNVVI

NFDFPRTSESYLHRVGRSGRFGHLGLAVNLVTYEDRFKMYQTEQELGTEIKPIPSNIDQAIYCQ

>XP_021904338.1 LOW QUALITY PROTEIN: DEAD-box ATP-dependent RNA helicase 8-like [Carica papaya]

MNSRGRYPPGIGAGRGAVNANPSFQSRTQPQQQYVQRGFAQNHQQFQHQHQQQQQQQHQHHQQQQWLRRAQLVGNSNDAA

VDEVEKTVQSEAVDPNAQDWKARLKIPPPDTRYKTEDVTATKGNEFEDYFLKRELLMGIYEKGFERPSPIQEESIPIALT

GSDILARAKNGTGKTAAFCIPALEKIDQDNNVIQVVIXVPTRELALQTSQVCKELGKHLKIQIMVTTGGTSLKDDIMRLY

QPVHLLVGTPGRILDLAKKGVCILKDCSMLVMDEADKLLSPEFQPSVEQLIRFLPANRQILMFSATFPVTVKDFKDRYLQ

KPYIINLMDELTLKGITQYYAFVEERQKVHCLNTLFSKLQINQSIIFCNSVNRVELLAKKITELGYSCFYIHAKMLQDHR

NRVFHDFRNGACRNLVCTDLFTRGIDIQAVNVVINFDFPKNSETYLHRVGRSGRFGHLGLAVNLITYEDRFNLYRIEQEL

GTEIKQIPPHIDQAIYCR

>XP_017235760.1 PREDICTED: DEAD-box ATP-dependent RNA helicase 8 [Daucus carota subsp. sativus]

MNSRGRFPPGLAGGRGGNFNPNPNYQNRNYQHQNHQGQRNSNSNVQVHQQQQFQQQQWLRRNPGGADSGNDEVEKTVQSE

AVDSGSQDWKAQLKLPPQDTRYRTEDVTATKGNEFEDYFLKRELLMGIYEKGFERPSPVQEESIPIALTGSDILARAKNG

TGKTAAFCIPALERIDQDKNVIQVVILVPTRELALQTSQVCKELGKHLKIEVMVTTGGTSLKDDIMRLYQPVHLLVGTPG

RILDLAKKGICDLKNCGMLVMDEADKLLSPEFQPSIQHLISFLPTNRQILMFSATFPVTVKDFKDRYLQKTYVINLMDEL

TLKGITQFYAFVEERQKVHCLNTLFSKLQINQSIIFCNSVNRVELLARKITELGYSCFYIHAKMLQDHRNRVFHDFRNGA

CRNLVCTDLFTRGIDIQAVNVVINFDFPKSAETYLHRVGRSGRFGHLGLAVNLITYEDRFNLYRIEQELGTEIKQIPPQI

DQAIYCQ

>XP_021613368.1 DEAD-box ATP-dependent RNA helicase 8-like [Manihot esculenta]

MNNYNRGRYTPGIGAGRGAGMNANPSFQSRVPQQQYVQRNSMQNHQQFQQQQQQQWLRRTQLPPADSSVDEVEKTVQSEA

VDSSSQDWKARLKIPPPDTRYKTEDVTATKGNEFEDYFLKRELLMGIYEKGFERPSPIQEESIPIALTGSDILARAKNGT

GKTAAFCIPALEKIDQDNNFIQVVILVPTRELALQTSQVCKELGKHLKIQVMVTTGGTSLKDDIMRLYQPVHLLVGTPGR

ILDLAKKGVCVLKDCSMLVMDEADKLLSPEFQPSIEQLIRFLPPNRQILMFSATFPVTVKDFKDRYLQKPYIINLMDELT

LKGITQYYAFVEERQKVHCLNTLFSKLQINQSIIFCNSVNRVELLAKKITELGYSCFYIHAKMLQDHRNRVFHDFRNGAC

RNLVCTDLFTRGIDIQAVNVVINFDFPKNSETYLHRVGRSGRFGHLGLAVNLITYEDRFNLYRIEQELGTEIKQIPPHID

QAIYCR

>XP_006445089.1 DEAD-box ATP-dependent RNA helicase 8 [Citrus clementina]

MNNSRGRYPPGIGSGRGGVNASNPSFQSSRPQQQYVQRNFLQNHYHNQQFQQQQQQQQQQQWLRRNNFPGADSSIVDEVE

KTVQSEAAVDPSSQDWKARLKIPPADTRYRTEDVTATKGNEFEDYFLKRELLMGIFEKGFERPSPIQEESIPIALTGSDI

LARAKNGTGKTAAFCIPALEKIDQDNNVIQVVILVPTRELALQTSQVCKELGKHLNIQVMVTTGGTSLKDDIMRLYQPVH

LLVGTPGRILDLSKKGVCILKDCSMLVMDEADKLLSPEFQPSVEQLIRFLPANRQILMFSATFPVTVKDFKDKYLQKPYV

INLMDELTLKGITQYYAFVEERQKVHCLNTLFSKLQINQSIIFCNSVNRVELLAKKITELGYSCFYIHAKMLQDHRNRVF

HDFRNGACRNLVCTDLFTRGIDIQAVNVVINFDFPKNSETYLHRVGRSGRFGHLGLAVNLITYEDRFNLYRIEQELGTEI

KQIPPHIDQAIYCR

>XP_021969140.1 DEAD-box ATP-dependent RNA helicase 6 [Helianthus annuus]

MNNSNYNNNNRRYPPGGTGNGRGGGSFHPNPNYYNQPRNPNQFHQQQRQQPPQSGQQQQQQHNQQQQQWLRRNPNPVGSS

SANEIDKSVQLDAGVDPSSQDWKAQLNIPAADTRYRTEDVTATKGNEFEDYFLKRELLMGIYEKGFEKPSPIQEESIPIA

LTGSDILARAKNGTGKTAAFCIPALEKIDTDKNKIQVVILVPTRELALQTSQVCKELGKHLQIEVMVTTGGTSLKDDIMR

LYQPVHLLVGTPGRILDLSKKGICKLSDCGMLVMDEADKLLSPEFQPSVEELIGFLPKQRQILLFSATFPVTVKDFKDRY

LKKPYVVNLMDELTLKGITQFYAFVEERQKVHCLNTLFSKLQINQSIIFCNSVNRVELLAKKITELGYSCFYIHAKMLQD

HRNRVFHDFRNGACRNLVCTDLFTRGIDIQAVNVVINFDFPKNAETYLHRVGRSGRFGHLGLAVNLITYEDRFNLYRIEQ

ELGTEIKQIPPQIDQAIYCR

>NP_001346307.1 uncharacterized protein LOC100194030 [Zea mays]

MDPRARYPSGMGNGRGGNLNYYGRGPPPQQPHQHHNHQQTSGAHHHQQTSGAHHHQQYAQRQHQHQHHGHNRHQQQQQHH

IYQQQQQQWLRRSQIARETSGAAVTSEPKSLAPSTAADGVDSSSQDWKAQLKLPPPDTRYRTEDVTATKGNEFEDYFLKR

ELLMGIYEKGFERPSPIQEESIPIALTGSDILARAKNGTGKTAAFCIPALEKIDQEKNAIQVVILVPTRELALQTSQVCK

ELGKHLKIQVMVTTGGTSLKDDIVRLYQPVHLLVGTPGRILDLTKKGVCILNDCSMLIMDEADKLLSPEFQPSIEQLIRY

LPASRQILMFSATFPVTVKEFKDKYLPKPYVINLMDELTLKGITQFYAFVEERQKVHCLNTLFSKLQINQSIIFCNSVNR

VELLAKKITELGYSCFYIHAKMLQDHRNRVFHDFRNGACRNLVCTDLFTRGIDIQAVNVVINFDFPKNSETYLHRVGRSG

RFGHLGLAVNLITYEDRFNLYRIEQELGTEIKSIPPQIDQAIYCQ

>VVB06481.1 unnamed protein product [Arabis nemorensis]

MNNRGRYPPGIGTGRGAAPNPDFQPYRQQSQDQQHVQRGQPXXXXXXXXXXXXXWSRRAQVPGNANNANEVQKTTSQPEA

SNDPNGQDWKATLRLPPPDTRYQTADVTATKGNEFEDYFLKRDLLKGIYEKGFEKPSPIQEESIPIALTGSDILARAKNG

TGKTGAFCIPVLEKIDPNNNVIQAMILVPTRELALQTSQVCKELSKYLNIQVMVTTGGTSLRDDIMRLHQPVHLLVGTPG

RILDLTKKGVCVLKDCTMLVMDEADKLLSAEFQPSLEELIQFLPQNRQFLMFSATFPVTVKAFKDRHLRKPYVINLMDQL

TLMGVTQYYAFVEERQKVHCLNTLFSKLQINQSIIFCNSVNRVELLAKKITELGYSCFYIHAKMVQDHRNRVFHEFRNGA

CRNLVCTDLFTRGIDIQAVNVVINFDFPRTSESYLHRVGRSGRFGHLGLAVNLVTYEDRFKMYQTEQELGTEIKPIPSLI

DKAIYCQ

>GEY34676.1 DEAD-box ATP-dependent RNA helicase 8 [Tanacetum cinerariifolium]

MNNHNYARGGGRYPPGIGVGNGGGGYYGNPNPNYQQQYVQRNVGSNQQFQQQQWLRRNPAVDEVEKTVQSEAIDARLVLT

TQGWPSGKRACDYHIKMCEVASSIPASSQDWKAQLRLPPADTRYRTEDVTATKGNEFEDYFLKRELLMGIYEKGFEKPSP

IQEESIPIALTGSDILARAKNGTGKTAAFCIPALEKIDPDKNVIQVVILVPTRELALQTSQVCKELGKHLKIQVMVTTGG

TSLKDDIMRLYQPVHLLVGTPGRILDLAKKGICKLDDCAMLAMDEADKLLSPEFQPSVEELIHFMPTNRQILMFSATFPV

TVKDFKDRFLKKPYVINLMDELTLKGITQFYAFVEERQKVHCLNTLFSKLQINQSIIFCNSVNRVELLAKKITELGYSCF

YIHAKMLQDHRNRVFHDFRNGACRNLVCTDLFTRGIDIQAVNVVINFDFPKNSETYLHRVGRSGRFGHLGLAVNLITYED

RFNLYRIEQELGTEIKQIPPFIDQAIYCR

>PIN09802.1 ATP-dependent RNA helicase [Handroanthus impetiginosus]

MSSRARYPPPGMGGGRGGGGGGVNPHGGPNPSFQPRYSNQQYVQRSPLQNYQNHQFQNPQPQQWLRRTQLPSADSTVDEV

EKTVQSEAADSSSQDWKGQLKLPPPDTRYKTEDVTATKGNEFEDYFLKRELLMGIYEKGFERPSPIQEESIPIALTGSDI

LARAKNGTGKTAAFCIPALEKIDQDKNVIQVIILVPTRELALQTSQVCKELGKHLKIQVMVSTGGTSLKDDIMRLYQPVH

LLVGTPGRILDLTKKGVCVLNECHMLVMDEADKLLSPEFQPSVEQLIHFMPPNRQILMFSATFPVTVKDFKDRYLRKPYI

INLMDELTLKGITQYYAFVEERQKVHCLNTLFSKLQINQSIIFCNSVNRVELLAKKITELGYSCFYIHAKMLQDHRNRVF

HDFRNGACRNLVCTDLFTRGIDIQAVNVVINFDFPKNSETYLHRVGRSGRFGHLGLAVNLITYEDRFNLYRIEQELGTEI

KQIPPHIDQAIYCQ

>XP_021686856.1 DEAD-box ATP-dependent RNA helicase 8-like isoform X1 [Hevea brasiliensis]

MMNNNNRGRYPPGIGAGRGGVMNANPSFQSRVPQQQYVQRNLMQNHQQFQHQQYQQQQQQQHQQQQQWLRRSQLPPADSS

VDEVEKTVQSEAVDSSSQDWKARLNIPPPDTRYKTEDVTATKGNEFEDYFLKRELLMGIYEKGFERPSPIQEESIPIALT

GSDILARAKNGTGKTAAFCIPALEKIDQDNNVIQVIILVPTRELALQTSQVCKELGKHLKIQVMVTTGGTSLKDDIMRLY

QPVHLLVGTPGRILDLAKKGVCILKDCSMLVMDEADKLLSPEFQPSIEQLIRFLPPARQILMFSATFPVTVKDFKDRYLK

KPYIINLMDELTLKGITQYYAFVEERQKVHCLNTLFSKLQINQSIIFCNSVNRVELLAKKITELGYSCFYIHAKMLQDHR

NRVFHDFRNGACRNLVCTDLFTRGIDIQAVNVVINFDFPKNSETYLHRVGRSGRFGHLGLAVNLITYEDRFNLYRIEQEL

GTEIKQIPPHIDQAIYCR

>PIN06180.1 ATP-dependent RNA helicase [Handroanthus impetiginosus]

MGSRPRYPPPGLARGRGGGGLVNPNAGPNPSFQLRNPTQHYLQRSPAPNNQSQQLLQNSQQQQWMRRNQLPSGDSTGDEV

EKTVQSEAVDSSSQDWKARLQLPPQDTRYRTEDVTATKGNEFEDYFLKRELLMGIYEKGFERPSPIQEESIPIALTGSDI

LARAKNGTGKTAAFCIPALEKIDQDKDAIQVVILVPTRELALQTSQVCKELGKHLRIQVMATTGGTSLKDDIMRLYQPVH

LLVGTPGRILDLTKKGVCVLNECCMLVMDEADKLLSPEFQPSVEQLIRFLPANRQILMFSATFPVTVKDFKDRYLRKPYI

INLMDELTLKGITQYYAFVEERQKVHCLNTLFSKLQINQSIIFCNSVNRVELLAKKITELGYSCFYIHAKMLQEHRNRVF

HDFRNGACRNLVCTDLFTRGIDIQAVNVVINFDFPKNSETYLHRVGRSGRFGHLGLAVNLITYEDRFNLYRIEQELGTEI

KQIPPHIDQAIYCQ

>RYR61186.1 hypothetical protein Ahy_A04g018312 [Arachis hypogaea]

MNNNRARSQDWKARLKVPPPDTRYKTEDVTATKGNEFEDYFLKRELLMGIYEKGFERPSPIQEESIPIALTGSDILARAK

NGTGKTAAFCIPALEKIDQDNNVIQVVILVPTRELALQTSQVCKELGKHLKIQVMVTTGGTSLKDDIMRLYQPVHLLVGT

PGRILDLAKKGVCVLKDCSMLVMDEADKLLSPEFQPSIEQLIQFLPPTRQILMFSATFPVTVKDFKDRYLRKPYIINLMD

ELTLKGITQYYAFVEERQKVHCLNTLFSKLQINQSIIFCNSVNRVELLAKKITELGYSCFYIHAKMLQDHRNRVFHDFRN

GACRNLVCTDLFTRGIDIQAVNVVINFDFPKNSETYLHRVGRSGRFGHLGLAVNLITYEDRFNLYRIEQELGTEIKQIPP

HIDQAIYCR

>KHN04659.1 DEAD-box ATP-dependent RNA helicase 8 [Glycine soja]

MGLVRGGFNPNLSQNPNQNQNPSLNQNHHAFQARPPYHQQQQAQYVQRHLLQQQQQQQWLRRDANAVDEVEKTVQSEAVD

SSSQDWKARLKIPPADTRYKTEDVTATKGNEFEDYFLKRELLMGIYEKGFERPSPIQEESIPIALTGSDILARAKNGTGK

TAAFCIPALEKIDQDNNVIQVVILVPTRELALQTSQVCKELGKHLKIQVMVTTGGTSLKDDIMRLYQPVHLLVGTPGRIL

DLAKKGVCILKDCAMLVMDEADKLLSPEFQPSIEQLIHFLPTTRQILMFSATFPVTVKDFKDRYLQKPYVINLMDELTLK

GITQFYAFVEERQKVHCLNTLFSKLQINQSIIFCNSVNRVELLAKKITELGYSCFYIHAKMLQDHRNRVFHDFRNGACRN

LVCTDLFTRGIDIQAVNVVINFDFPKNAETYLHRVGRSGRFGHLGLAVNLITYEDRFNLYRIEQELGTEIKQIPPQIDQA

IYCR

>XP_019460657.1 PREDICTED: DEAD-box ATP-dependent RNA helicase 8-like [Lupinus angustifolius]

MNNRARYPPGIGLGRGGGSGGGAGLNLNPGFQQRPPQQHVQRHIMQQQQYQQQQQQQQQWLRRTQLGGNTNTNVVEEVEK

TVQSEAVDESSQDWKTKLKIPPADTRYRTEDVTATKGNEFEDYFLKRELLMGIYEKGFERPSPIQEESIPIALTGSDILA

RAKNGTGKTAAFCIPALEKIDQDNNVIQVVILVPTRELALQTSQVCKELGKHLNIQVMVTTGGTSLKDDIMRLYQPVHLL

VGTPGRILDLARKGVCIMKDCSMLVMDEADKLLSPEFQPSIEQLIQFLPGNRQILMFSATFPVTVKDFKDRYLRKPYVIN

LMDELTLKGITQFYAFVEERQKVHCLNTLFSKLQINQSIIFCNSVNRVELLAKKITELGYSCFYIHAKMLQDHRNRVFHD

FRNGACRNLVCTDLFTRGIDIQAVNVVINFDFPKNSETYLHRVGRSGRFGHLGLAVNLITYEDRFNLYRIEQELGTEIKQ

IPPHIDQAIYCQ

>XP_015961689.1 DEAD-box ATP-dependent RNA helicase 8 [Arachis duranensis]

MNNNRARYPPGIGLGRGNGGFNTGFQPRPPQQQYVQRHMVQQQHPQHYQHYQQNQQQHQQQQQQQQQQQQQWLRRTQLGG

GGSNDNNVVEEVEKTVQSEAVDSSSQDWKARLKVPPPDTRYKTEDVTATKGNEFEDYFLKRELLMGIYEKGFERPSPIQE

ESIPIALTGSDILARAKNGTGKTAAFCIPALEKIDQDNNVIQVVILVPTRELALQTSQVCKELGKHLKIQVMVTTGGTSL

KDDIMRLYQPVHLLVGTPGRILDLAKKGVCVLKDCSMLVMDEADKLLSPEFQPSIEQLIQFLPPTRQILMFSATFPVTVK

DFKDRYLRKPYIINLMDELTLKGITQYYAFVEERQKVHCLNTLFSKLQINQSIIFCNSVNRVELLAKKITELGYSCFYIH

AKMLQDHRNRVFHDFRNGACRNLVCTDLFTRGIDIQAVNVVINFDFPKNSETYLHRVGRSGRFGHLGLAVNLITYEDRFN

LYRIEQELGTEIKQIPPHIDQAIYCR

>KAF3504060.1 hypothetical protein F2Q69_00039387 [Brassica cretica]

MNNNNNNNRGRYPPGIGAVRGAINPNPNFQSRPGYQQQPPPQYVQRGGYTAQQNHQQQFQQATSQQQQVQQQWLRRPQIS

GGNSNGDTNSEDWKARLQLPAPDTRFRTEDVTATKGNEFEDYFLKRELLMGIYEKGFERPSPIQEESIPIALTGRDILAR

AKNGTGKTAAFCIPVLEKIDQDNNVIQALIIVPTRELALQTSQVCKELGKHLKIQVMVTTGGTSLKDDIMRLYQPVHLLV

GTPGRILDLTKKGVCVLKDCSVFVMDEADKLLSQEFQPSVEHLISFLPQNRQILMFSATFPVTVKDFKDRFLTNPYIINL

MDELTLKGITQFYAFVEERQKIHCLNTLFSKLQINQSIIFCNSVNRVELLAKKITELGYSCFYIHAKMLQDHRNRVFHDF

RNGACRNLVCTDLFTRGIDIQAVNVVINFDFPKTAETYLHRVGRSGRFGHLGLAVNLITYEDRFNLYRIEQELGTEIKQI

PPHIDQAIYCQ

>XP_013629570.1 PREDICTED: DEAD-box ATP-dependent RNA helicase 8-like [Brassica oleracea var. oleracea]

MMNNNNNRGGGRGIGAGRGAINPNPNFQSRPGYQQPQYVQRGGYSHQQQLFQQATSQPRQYQHQQQWLPRPQISSGNTNG

GGDAVVEVEKTLLSDTNSEDWKARLKLPAPDTRYRTEDVTATKGNEFEDYFLKRELLMGIYEKGFERPSPIQEESIPIAL

TGRDILARAKNGTGKTAAFCIPVLEKIDQDNNVIQAVIIVPTRELALQTSQVCKELGKHLKIQVMVTTGGTSLKDDIMRL

YQPVHLLVGTPGRILDLTKKGVCVLKDCSVLAMDEADKLLSQEFQPSVEHLISFLPQNRQILMFSATFPVTVKYFKDRFL

TNPYIINLMDELTLKGITQFYAFVEERQKIHCLNTLFSKLQINQSIIFCNSVNRVELLAKKITELGYSCFYIHAKMLQDH

RNRVFHDFRNGACRNLVCTDLFTRGIDIQAVNVVINFDFPKNAETYLHRVGRSGRFGHLGLAVNLITYEDRFNLYRIEQE

LGTEIKQIPPHIDQAIYCQ

>XP_031098119.1 DEAD-box ATP-dependent RNA helicase 8-like [Ipomoea triloba]

MNSRGRYPPGIGNGRGGGGSGYGGGGFRSGHNSGGYNQPRNPHYQYAQQNPPQPQQYGQRTLQNQPQQQQQQWLRRNPSA

AASESSSNEVLKTIQSEAIDSSSQDWKARLNIPAPDTRYRTEDVTATKGNEFEDYFLKRELLMGIYEKGFERPSPIQEES

IPIALTGSDILARAKNGTGKTAAFCIPALEKIDTETNVIQVVILVPTRELALQTSQVCKELGKHLKIQVMVSTGGTNLKD

DIMRLYQPVHLLVGTPGRILDLAKKGICILKDCAMLVMDEADKLLSPEFQPSVEHLITFLPEYRQILMFSATFPVTVKDF

KDRYLRKPYVINLMDELTLKGITQYYAFVEERQKVHCLNTLFSKLQINQSIIFCNSVNRVELLAKKITELGYSCFYIHAK

MLQDHRNKVFHDFRNGACRNLVCTDLFTRGIDIQAVNVVINFDFPKNSETYLHRVGRSGRFGHLGLAVNLITYEDRFNLY

KIEQELGTEIKPIPPHIDQAIYCQ

>RID70925.1 hypothetical protein BRARA_C02900 [Brassica rapa]

MMNNNNNNNNRGGGGGGRYPPGIGAGRGAINPNPNFQSRPGYQQQQQQPQYVQRGAYSHQQQQFQQATSQPPRQYQQQQH

QQQWLPRPQISTGNSNGGGGGDAVVEVEKTVLSDTNSEDWKARLKLPAPDTRYRTEDVTATKGNEFEDYFLKRELLMGIY

EKGFERPSPIQEESIPIALTGRDILARAKNGTGKTAAFCIPVLEKIDQDNNVIQAVIIVPTRELALQTSQVCKELGKHLK

IQVMVTTGGTSLKDDIMRLYQPVHLLVGTPGRILDLTKKGVCVLKDCSVLAMDEADKLLSQEFQPSVEHLISFLPQNRQI

LMFSATFPVTVKYFKDRFLTNPYIINLMDELTLKGITQFYAFVEERQKIHCLNTLFSKLQINQSIIFCNSVNRVELLAKK

ITELGYSCFYIHAKMLQDHRNRVFHDFRNGACRNLVCTDLFTRGIDIQAVNVVINFDFPKNAETYLHRVGRSGRFGHLGL

AVNLITYEDRFNLYRIEQELGTEIKQIPPHIDQAIYCQ

>XP_026380125.1 DEAD-box ATP-dependent RNA helicase 8-like [Papaver somniferum]

MNNPRGRYPPGIGNGRGGGGGGGYVNSNPNFQPRNPQQQQYVQRNPVQNQPQYQQQQQQQQQQQWLRRNPMGGNESSVNE

VEKAVQSETIDSSSQDWKARLKIPPSDTRYRTEDVTATKGNEFEDYFLKRELLMGIYEKGFERPSPIQEESIPIALTGSD

ILARAKNGTGKTAAFCIPALEKIDQDNNVIQVVILVPTRELALQTSQVCKELGKHLKIQVMVTTGGTSLKDDIMRLYQPV

HLLVGTPGRILDLAKKGVCVLKDCSMIVMDEADKLLSPEFQPSVEQLIRFLPTSRQILLFSATFPVTVKDFKDRYLQKPY

IINLMDELTLKGITQFYAFVEERQKVHCLNTLFSKLQINQSIIFCNSVNRVELLAKKITELGYSCFYIHAKMLQDHRNRV

FHDFRNGACRNLVCTDLFTRGIDIQAVNVVINFDFPKNSETYLHRVGRSGRFGHLGLAVNLITYEDRFNLYRIEQELGTE

IKQIPPQIDQAIYCR

>XP_016193807.1 DEAD-box ATP-dependent RNA helicase 8 isoform X1 [Arachis ipaensis]

MNNNRARYPPGIGLGRGNGGFNTGFQPRPPQQQYVQRHMVQPQHPHQYQHYQQNQQQHQQQQQQQQQQQWLRRTQLGGGG

SNDNNVVEEVEKTVQSEAVDSSSQDWKARLKVPPPDTRYKTEDVTATKGNEFEDYFLKRELLMGIYEKGFERPSPIQEES

IPIALTGSDILARAKNGTGKTAAFCIPALEKIDQDNNVIQVVILVPTRELALQTSQVCKELGKHLKIQVMVTTGGTSLKD

DIMRLYQPVHLLVGTPGRILDLAKKGVCVLKDCSMLVMDEADKLLSPEFQPSIEQLIQFLPPTRQILMFSATFPVTVKDF

KDRYLRKPYIINLMDELTLKGITQYYAFVEERQKVHCLNTLFSKLQINQSIIFCNSVNRVELLAKKITELGYSCFYIHAK

MLQDHRNRVFHDFRNGACRNLVCTDLFTRGIDIQAVNVVINFDFPKNSETYLHRVGRSGRFGHLGLAVNLITYEDRFNLY

RIEQELGTEIKQIPPHIDQAIYCR

>KAE8697571.1 DEAD-box ATP-dependent RNA helicase 6 [Hibiscus syriacus]

MNSRGRYPPGIGAGRGGGVNASPSFQSRPSQQQYVQRNLVQNHQHFQQQQQLWLRRNELPGGNDSNVVDEVEKTVQSEAV

DSSSQDWKARLKMPPPDTRYKTEDVTATKGNEFEDYFLKRELLMGIYEKGFERPSPIQEESIPIALTGSDILARAKNGTG

KTAAFCIPALEKIDQDNNVIQAVIIVPTRELALQTSQVCKELGKHLQIQVMVTTGGTSLKDDIMRLYQPVHLLVGTPGRI

LDLAKKGVCILKDCSMLIMDEADKLLSPEFQPSIEQLIHFLPANRQILMFSATFPVTVKYFKDRYLKKPYIINLMDELTL

KGITQYYAFVEERQKVHCLNTLFSKLQINQSIIFCNSVNRVELLAKKITELGYSCFYIHAKMLQDHRNRVFHDFRNGACR

NLVCTDLFTRGIDIQAVNVVINFDFPKNSETYLHRVGRSGRFGHLGLAVNLITYEDRFNLYRIEQELGTEIKQIPPHIDQ

AIYCR

>XP_012482519.1 PREDICTED: DEAD-box ATP-dependent RNA helicase 8-like [Gossypium raimondii]

MNSRGRYPPPGIGVGRGGGVNANPSFQSRPSQQHYVQRNLVHNQQHFQQHNQQHFQQQQQHQQQQQWLRRNQLPSGNDSS

VIDEVEKTVQSEAVDSSSQDWKARLKIPPADTRYKTEDVTATKGNEFEDYFLKRELLMGIYEKGFERPSPIQEESIPIAL

TGSDILARAKNGTGKTAAFCIPALEKIDQDNNVIQVVILVPTRELALQTSQVCKELGKHLQIQVMVTTGGTSLKDDIMRL

YQPVHLLVGTPGRILDLAKKGVCILKDCSMLIMDEADKLLSPEFQPSIEQLIRFLPATRQILLFSATFPVTVKDFKDRYL

QKPYIINLMDELTLKGITQYYAFVEERQKVHCLNTLFSKLQINQSIIFCNSVNRVELLAKKITELGYSCFYIHAKMLQDH

RNRVFHDFRNGACRNLVCTDLFTRGIDIQAVNVVINFDFPKNSETYLHRVGRSGRFGHLGLAVNLITYEDRFNLYRIEQE

LGTEIKQIPPHIDQAIYCR

>RYR23756.1 hypothetical protein Ahy_B02g057246 [Arachis hypogaea]

MNNNNNYYNRARYPPPGIGGGGLGRGGGGGGPGFNQNSPFQPRPNYQQHQQQHQQLQQQHQQYVQRQMVQQQQQQQQQQQ

QQQWLRRAQMGAADSNVVDEVEKTVQSEAIDPSSQDWKARLKVPPPDTRYKTEDVTATKGNEFEDYFLKRELLMGIYEKG

FERPSPIQEESIPIALTGSDILARAKNGTGKTAAFCIPALEKIDQDNNVIQVVILVPTRELALQTSQVCKELGKHLKIQV

MVTTGGTSLKDDIMRLYQPVHLLVGTPGRILDLAKKGVCVLKDCAMLVMDEADKLLSPEFQPSIQQLIHFLPSHRQILMF

SATFPVTVKDFKDRYLQKPYVINLMDELTLKGITQYYAFVEERQKVHCLNTLFSKLQINQSIIFCNSVNRVELLAKKITE

LGYSCFYIHAKMLQDHRNRVFHDFRNGACRNLVCTDLFTRGIDIQAVNVVINFDFPKNSETYLHRVGRSGRFGHLGLAVN

LITYEDRFNLYRIEQELGTEIKQIPPHIDQAIYCRLK

>RRT82239.1 hypothetical protein B296_00018100 [Ensete ventricosum]

MNPRGRYPPPGMGNGRGGNASTNPGFYSRQQQQQQQYVQRNPVQVQPNQHFQQQQQWSIRNQLGGDSGSGDVVKSVQPES

SDTSGFSSQDWKAQLRMPPPDTRYKTEDVTATKGNEFEDYFLKRELLMGIYEKGFEKPSPIQEESIPIALTGSDILARAK

NGTGKTAAFCIPALEKIDQDHNAIQVVILVPTRELALQTSQVCKELGKHLKIQVMVTTGGTSLKDDIMRLYQPVHLLVGT

PGRILDLAKKGVCVLKDCSMLVMDEADKLLSPEFQPSIEQLIQFLPANRQILMFSATFPVTVKDFKDRYLPKPYIINLMD

ELTLKGITQYYAFVEERQKVHCLNTLFSKLQINQSIIFCNSVNRVELLAKKITELGYSCFYIHAKMLQDHRNRVFHDFRN

GACRNLVCTDLFTRGIDIQAVNVVINFDFPKNSETYLHRVGRSGRFGHLGLAVNLITYEDRFNLYRIEQELGTEIKQIPP

QIDQAIYCR

>XP_009134538.1 DEAD-box ATP-dependent RNA helicase 8 [Brassica rapa]

MMNNNNNNNRGGGGRYPPGIGAGRGAINPNPNFQSRPGYQQPQQPQYVQRGAYSHQQQQFQQATSQPPRQYHQQQHQQQW

LPRPQISSGNSNGGGEGDAVVEVEKTVLSDTNSEDWKARLKLPAPDTRYRTEDVTATKGNEFEDYFLKRELLMGIYEKGF

ERPSPIQEESIPIALTGRDILARAKNGTGKTAAFCIPVLEKIDQDNNVIQAVIIVPTRELALQTSQVCKELGKHLKIQVM

VTTGGTSLKDDIMRLYQPVHLLVGTPGRILDLTKKGVCVLKDCSVLAMDEADKLLSQEFQPSVEHLISFLPQNRQILMFS

ATFPVTVKYFKDRFLTNPYIINLMDELTLKGITQFYAFVEERQKIHCLNTLFSKLQINQSIIFCNSVNRVELLAKKITEL

GYSCFYIHAKMLQDHRNRVFHDFRNGACRNLVCTDLFTRGIDIQAVNVVINFDFPKNAETYLHRVGRSGRFGHLGLAVNL

ITYEDRFNLYRIEQELGTEIKQIPPHIDQAIYCQ

>XP_003528863.1 DEAD-box ATP-dependent RNA helicase 8 [Glycine max]

MNHNNSNNRARYPPGMGLVRGGFNPNLSQNPNQNQNPSLNQNHHAFQARPPYHQQQQAQYVQRHLLQQQQQQQQWLRRDA

NAVDEVEKTVQSEAVDSSSQDWKARLKIPPADTRYKTEDVTATKGNEFEDYFLKRELLMGIYEKGFERPSPIQEESIPIA

LTGSDILARAKNGTGKTAAFCIPALEKIDQDNNVIQVVILVPTRELALQTSQVCKELGKHLKIQVMVTTGGTSLKDDIMR

LYQPVHLLVGTPGRILDLAKKGVCILKDCAMLVMDEADKLLSPEFQPSIEQLIHFLPTTRQILMFSATFPVTVKDFKDRY

LQKPYVINLMDELTLKGITQFYAFVEERQKVHCLNTLFSKLQINQSIIFCNSVNRVELLAKKITELGYSCFYIHAKMLQD

HRNRVFHDFRNGACRNLVCTDLFTRGIDIQAVNVVINFDFPKNAETYLHRVGRSGRFGHLGLAVNLITYEDRFNLYRIEQ

ELGTEIKQIPPQIDQAIYCR

>XP_018476565.1 PREDICTED: DEAD-box ATP-dependent RNA helicase 8-like [Raphanus sativus]

MMNNNNNNNNRGGGGGGRYPPGIGAGRGAINPNPNFQSRPAYQQQPQYVQRGGGYSQQQQFQQATSQPPRQYQQQQQWLP

RPQISTNGGSAVAQVEKSEAVDTNSEDWKARLKLPAPDTRFRTEDVTATKGNEFEDYFLKRELLMGIYEKGFERPSPIQE

ESIPIALTGRDILARAKNGTGKTAAFCIPVLEKIDQDNNVIQAVIIVPTRELALQTSQVCKELGKHLKIQVMVTTGGTSL

KDDIMRLYQHVHLLVGTPGRILDLTKKGVCVLKDCSVLAMDEADKLLSQEFQPSVEHLISFLPENRQILMFSATFPVTVK

YFKDRFLTNPYIINLMDELTLKGITQFYAFVEERQKIHCLNTLFSKLQINQSIIFCNSVNRVELLAKKITELGYSCFYIH

AKMLQDHRNRVFHDFRNGACRNLVCTDLFTRGIDIQAVNVVINFDFPKNAETYLHRVGRSGRFGHLGLAVNLITYEDRFN

LYRIEQELGTEIKQIPPHIDQAIYCQ

>XP_021613851.1 DEAD-box ATP-dependent RNA helicase 8 isoform X1 [Manihot esculenta]

MNSNNRGRYPPGMGVGRGAVMNANPSFQSRVPQQQYVQRNLMQNHQQFQHQQQQQQHHQQQWLRRSQLPPVDSSVDEVEK

TVQSEAVDSSSQDWKARLNIPPPDTRYKTEDVTATKGNEFEDYFLKRELLMGIYEKGFERPSPIQEESIPIALTGSDILA

RAKNGTGKTAAFCIPALEKIDQDNNVIQVVILVPTRELALQTSQVCKELGKHLKIQVMVTTGGTSLKDDIMRLYQPVHLL

VGTPGRILDLAKKGVCILKDCSMLVMDEADKLLSPEFQPSVEQLIRFVPQNRQILMFSATFPVTVKDFKDRYLKKPYIIN

LMDELTLKGITQFYAFVEERQKVHCLNTLFSKLQINQSIIFCNSVNRVELLAKKITELGYSCFYIHAKMLQDHRNRVFHD

FRNGACRNLVCTDLFTRGIDIQAVNVVINFDFPKNSETYLHRVGRSGRFGHLGLAVNLITYEDRFNLYRIEQELGTEIKQ

IPPHIDQAIYCR

>KAF3960775.1 hypothetical protein CMV_014539 [Castanea mollissima]

MNNNRGRYPPGMGGAGRGGGMNANPPFQSRNPQQQYVQRSMLQNQQYQHHQQQQQQQQQQHHHHQQQQHHQQQQQHQHQH

HQQQQQHQQQQQQQQQQQQQHWLRRSQLGGVDSSVDEVEKTVQSEAVDSSSQDWKARLKIPPPDTRFRTEDVTATKGNEF

EDYFLKRELLMGIYEKGFERPSPIQEESIPIALTGSDILARAKNGTGKTAAFCIPALEKIDQDNNVIQVVILVPTRELAL

QTSQVCKELGKHLKIQVMVTTGGTSLKDDIMRLYQPVHLLVGTPGRILDLAKKGVCILKDCSMLVMDEADKLLSPEFQPS

VEQLIRFLPTSRQILMFSATFPVTVKDFKDRYLQKPYVINLMDELTLKGITQFYAFVEERQKVHCLNTLFSKLQINQSII

FCNSVNRVELLAKKITELGYSCFYIHAKMLQDHRNRVFHDFRNGACRNLVCTDLFTRGIDIQAVNVVINFDFPKNSETYL

HRVGRSGRFGHLGLAVNLITYEDRFNLYRIEQELGTEIKQIPPHIDQAIYCR

>KAB5527076.1 hypothetical protein DKX38_020923 [Salix brachista]

MNNNNRGRYPPGIGAGRGGGMNANPNFQSRVPQQQYVQRHFGQNHHQQQYNHHQQNLNQQQQQQQQQWLRRNQLAAADSS

VDEVEKTVQSEAVDSSSQDWKAKLKIPPADTRYQTEDVTATKGNDFEDYFLKRELLMGIYEKGFERPSPIQEESIPIALT

GSDILARAKNGTGKTAAFCIPALEKIDQDSNFIQVVILVPTRELALQTSQVCKELGKHLKIQVMATTGGTSLKDDIMRLY

QPVHLLVGTPGRILDLAKKGVCILKDCSVLVLDEADKLLSPEFQPSIEQLIRFLPSNRQILMFSATFPVTVKDFKDRYLE

KPYVINLMDELTLKGITQFYAFVEERQKVHCLNTLFSKLQINQSIIFCNSVNRVELLAKKITELGYSCFYIHAKMLQDHR

NRVFHDFRNGACRNLVCTDLFTRGIDIQAVNVVINFDFPKNSETYLHRVGRSGRFGHLGLAVNLITYEDRFNLYRIEQEL

GTEIKQIPPHIDQAIYCQ

>KAF3516406.1 hypothetical protein DY000_02061880, partial [Brassica cretica]

LGLLVSSFFEIEIEIVMNNNRGRYPPGNGTGRGAPPHPDYQSYGQQPQNHQQFQQQQQWSRRAQLPGNANEVQNKFSGSV

TLSGGQDWKATLKLPPPDTRYQTADVTATKGNEFEDYFLKRDLLKGIYEKGFEKPSPIQEESIPIALTGSDILARAKNGT

GKTGAFCIPVLEKIDPNTNVIQAMILVPTRELALQTSQVCKELSKYLNIQVMVTTGGTSLRDDIMRLHQPVHLLVGTPGR

ILDLTKKGVCVLKDCTMLVMDEADKLLSAEFQPSLEELIQFLPENRQFLMFSATFPVTVKAFKDRHLRKPYVINLMDQLT

LMGVTQYYAFVEERQKVHCLNTLFSKLQINQSIIFCNSVNRVELLAKKITELGYSCFYIHAKMVQDHRNRVFHEFRNGAC

RNLVCTDLFTRGIDIQAVNVVINFDFPRTSESYLHRVGRSGRFGHLGLAVNLVTYEDRFKMYQTEQELGTEIKPIPSHID

QAIYCQ

>OWM88222.1 hypothetical protein CDL15_Pgr003634 [Punica granatum]

MVNSPVVNMYPLSSYTFGTKEPKMEKDTSVADRLARMKVNYMKEGMRTSVEGILLVQEHRHPHILLLQIGNTFCKLPGGR

LKPGENGTLSLVAQRERRVKIYYLERAYSKLLSLLLNYKICYMKEGMRTSVEGILLVQEHRHPHILLLQIGNTFCKLPGG

RLKPGENEIEGLKRKLSSKLGANSPQLQPDWQIGECVAIWWRPNFETVMYPYCPPHITKPKECKKLFIVHLSERDYFAVP

KNLKLLAVPLFELYDNVHRYGPVISTIPQQLSRRNQLGRSDSADEVEKTVQSEAVGSSSADWKSNLRIPPADTRYQTEDV

TATKGNEFEDYFLKRELLMGIYEKGFERPSPIQEESIPIALTGSNILARAKNGTGKTAAFCIPTLEKIDQDNNVIQAMVL

VPTRELALQTSQVCKELGKHLEIQVMVTTGGTSLRDDIMRLYQPVHLLVGTPGRILDLAKKGVCILKDCSMLVMDEADKL

LSPEFQPSVEQLIRFMPANRQILMFSATFPVTVKDFKDRYLRKPYIINLMDELTLKGITQYYAFVEERQKVHCLNTLFSK

LQINQSIIFCNSVNRVELLAKKITELGYSCFYIHAKMLQDHRNRVFHDFRNGACRNLVCTDLFTRGIDIQAVNVVINFDF

PKNSETYLHRVGRSGRFGHLGLAVNLITYEDRFTLYRMEQELGTEIKQIPPHIDQAIYCR

>RDY09324.1 DEAD-box ATP-dependent RNA helicase 8, partial [Mucuna pruriens]

MKAPIATPFLEISVRGIGGMNHRERERYPPGMGLGRGLNSNPGFQPRPHPHQYVQRQMMQQQHPQQYQQNHHQHHHQQQQ

QHHQQQHQQQQQWLRRNQLGGGSDTNVVEEVEKTVQSEAVDSSSQDWKARLKIPPPDTRYKTEDVTATKGNEFEDYFLKR

ELLMGIYEKGFERPSPIQEESIPIALTGSDILARAKNGTGKTAAFCIPALEKIDQDTNVIQVVILVPTRELALQTSQVCK

ELGKHLKIQVMVTTGGTSLKDDIMRLYQPVHLLVGTPGRILDLAKKGVCILKDCSMLVMDEADKLLSPEFQPSIEQLIQF

LPRTRQILMFSATFPVTVKDFKDRYLRKPYVINLMDELTLKGITQYYAFVEERQKVHCLNTLFSKLQINQSIIFCNSVNR

VELLAKKITELGYSCFYIHAKMLQDHRNRVFHDFRNGACRNLVCTDLFTRGIDIQAVNVVINFDFPKNSETYLHRVGRSG

RFGHLGLAVNLITYEDRFNLYRIEQELGTEIKQIPPHIDQAIYCR

>BAT97920.1 hypothetical protein VIGAN_09150900 [Vigna angularis var. angularis]

MNHHHNNRARYPPGMGLGRGGFHPNVGHNPNLNQNPGLNQNQAFQPRPSYQQQPHYVQRHLMQQPQQQQQWLRRDANAVD

EVEKTVQSEAVDSSSQDWKARLKIPPADTRYKTEDVTATKGNEFEDYFLKRELLMGIYEKGFERPSPIQEESIPIALTGS

DILARAKNGTGKTAAFCIPALEKIDQDNNVIQVVILVPTRELALQTSQVCKELGKHLNIQVMVTTGGTSLKDDIMRLYQP

VHLLVGTPGRILDLAKKGVCIMKDCSMLVMDEADKLLSPEFQPSIEQLIHFLPSNRQILMFSATFPVTVKDFKDRYLHKP

YVINLMDELTLKGITQYYAFVEERQKVHCLNTLFSKLQINQSIIFCNSVNRVELLAKKITELGYSCFYIHAKMLQDHRNR

VFHDFRNGACRNLVCTDLFTRGIDIQAVNVVINFDFPKNSETYLHRVGRSGRFGHLGLAVNLITYEDRFNLYRIEQELGT

EIKQIPPQIDQAIYCR

>XP_013600084.1 PREDICTED: DEAD-box ATP-dependent RNA helicase 12 isoform X1 [Brassica oleracea var. oleracea]

MNNNRGRYPPGMGTGRGAPPNPDYQSQNHHQQQWTRRAQLPGNANEVQKTTSPPPVASSDPKKKLIIIRLCSPFTLSGQD

WKATLRLPPPDTRYQTADVTATKGNEFEDYFLKRDLLKGIYEKGFEKPSPIQEESIPIALTGSDILARAKNGTGKTGAFC

IPVLERIDPNTNVIQAMILVPTRELALQTSQVCKELSKYLNIQVMVTTGGTSLRDDIMRLHQPVHLLVGTPGRILDLTKK

GVCVLKDCTMLVMDEADKLLSAEFQPSLEELIQFLPQNRQFLMYSATFPVTVKAFKDRHLRKPYVINLMDQLTLMGVTQY

YAFVEERQKVHCLNTLFSKLQINQSIIFCNSVNRVELLAKKITELGYSCFYIHAKMVQDHRNRVFHEFRNGACRNLVCTD

LFTRGIDIQAVNVVINFDFPRTSESYLHRVGRSGRFGHLGLAVNLVTYEDRFKMYQTEQELGTEIKPIPSNIDQAIYCQ

>XP_023895835.1 DEAD-box ATP-dependent RNA helicase 8-like [Quercus suber]

MNNNNRGRYPPGMGGAGRGGGMNANPPFQSRNPQQQYVQRSMLQNQQYQHHQQQQQHHHHQQQQHHQQQQQHHQQQQQQQ

QQQQQQHWLRRGQLGGVDSSVDEVEKTVQSEAVDSSSQDWKARLKIPPPDTRFRTEDVTATKGNEFEDYFLKRELLMGIY

EKGFERPSPIQEESIPIALTGSDILARAKNGTGKTAAFCIPALEKIDQDNNVIQVVILVPTRELALQTSQVCKELGKHLK

IQVMVTTGGTSLKDDIMRLYQPVHLLVGTPGRILDLAKKGVCILKDCSMLVMDEADKLLSPEFQPSVEQLIRFLPTSRQI

LMFSATFPVTVKDFKDRYLQKPYVINLMDELTLKGITQFYAFVEERQKVHCLNTLFSKLQINQSIIFCNSVNRVELLAKK

ITELGYSCFYIHAKMLQDHRNRVFHDFRNGACRNLVCTDLFTRGIDIQAVNVVINFDFPKNSETYLHRVGRSGRFGHLGL

AVNLITYEDRFNLYRIEQELGTEIKQIPPHIDQAIYCR

>RWR91884.1 DEAD-box ATP-dependent RNA helicase 8 [Cinnamomum micranthum f. kanehirae]

MTIPSRPSLQRTKRYLRNEKPFIKHMNSPYYRFELERSSNQRFFGKKPSPAVCAEESSAESAAPVSAAAPAAAQQQWLRR

NPMGADPGASEVEKAVVLSEGVDSSSQDWKAQLKIPPRDTRYKTEDVTATKGNEFEDYFLKRELLMGIYEKGFERPSPIQ

EESIPIALTGSDILARAKNGTGKTAAFCVPALEKIDQDNNVIQVVILVPTRELALQTSQVCKELGKHLKIQVMVTTGGTS

LKDDIMRLYQPVHLLVGTPGRILDLAKKGVCILKDCSMLIMDEADKLLSPEFQPSVEQLIRFLPANRQILLFSATFPVTV

KDFKDRYLHKPYIINLMDELTLKGITQYYAFVEERQKVHCLNTLFSKLQINQSIIFCNSVNRVELLAKKITELGYSCFYI

HAKMLQDHRNRVFHDFRNGACRNLVCTDLFTRGIDIQAVNVVINFDFPKNSETYLHRVGRSGRFGHLGLAVNLITYEDRF

NLYRIEQELGTEIKQIPPQIDQAIYCR

>XP_016682161.1 PREDICTED: DEAD-box ATP-dependent RNA helicase 8-like [Gossypium hirsutum]

MNNRGRYPSVLGRGRGANANPSFQSRPEQPQYAQRNLVQNHHHFQQQQHHHHHLQQQQHQQQWLRRNQLPGGNDSTVADE

VEKTVQSEAVDSSSQDWKARLKMPPSDTRYKTEDVTATKGNEFEDYFLKRELLMGIYEKGFERPSPIQEESIPIALTGSD

ILARAKNGTGKTAAFCIPALEKIDQDNNVIQVVILVPTRELALQTSQVCKELGKHLQIQVMVTTGGTSLKDDIMRLYQPV

HLLVGTPGRILDLAKKGVCILKNCSMLIMDEADKLLSPEFQPSLEQLLCFLPPNRQILMFSATFPVTVKDFKDKYLKKPY

IINLMDELTLKGITQYYAFVEERQKVHCLNTLFSKLQINQSIIFCNSVNRVELLAKKITELGYSCFYIHAKMLQDHRNRV

FHDFRNGACRNLVCTDLFTRGIDIQAVNVVINFDFPKNSETYLHRVGRSGRFGHLGLAVNLITYEDRFNLYRIEQELGTE

IKQIPPHIDQAIYCR

>XP_025612142.1 DEAD-box ATP-dependent RNA helicase 8 [Arachis hypogaea]

MNNNNNYYNRARYPPPGIGGGGLGRGGGGGGPGFNQNSPFQPRPNYQQHQQQHQQLQQQHQQYVQRQMVQQQQQQQQQQQ

QQWLRRAQMGAADSNVVDEVEKTVQSEAIDPSSQDWKARLKVPPPDTRYKTEDVTATKGNEFEDYFLKRELLMGIYEKGF

ERPSPIQEESIPIALTGSDILARAKNGTGKTAAFCIPALEKIDQDNNVIQVVILVPTRELALQTSQVCKELGKHLKIQVM

VTTGGTSLKDDIMRLYQPVHLLVGTPGRILDLAKKGVCVLKDCAMLVMDEADKLLSPEFQPSIQQLIHFLPSHRQILMFS

ATFPVTVKDFKDRYLQKPYVINLMDELTLKGITQYYAFVEERQKVHCLNTLFSKLQINQSIIFCNSVNRVELLAKKITEL

GYSCFYIHAKMLQDHRNRVFHDFRNGACRNLVCTDLFTRGIDIQAVNVVINFDFPKNSETYLHRVGRSGRFGHLGLAVNL

ITYEDRFNLYRIEQELGTEIKQIPPHIDQAIYCR

>TYJ26995.1 hypothetical protein E1A91_A07G157300v1 [Gossypium mustelinum]

MNSRGRYPPPGIGVGRGGGVNANPSFQSRPSQQHYVQRNLVHNQQHFQQHNQQHFQQQQQWLRRNQLRGGNDSSVIDEVE

KTVQSEAVDSSSQDWKARLKIPPADTRYKTEDVTATKGNEFEDYFLKRELLMGIYEKGFERPSPIQEESIPIALTGSDIL

ARAKNGTGKTAAFCIPALEKIDQDNNVIQVVILVPTRELALQTSQVCKELGKHLQIQVMVTTGGTSLKDDIMRLYQPVHL

LVGTPGRILDLAKKGVCILKDCSMLIMDEADKLLSPEFQPSVEQLIRFLPATRQILLFSATFPVTVKDFKDRYLQKPYII

NLMDELTLKGITQYYAFVEERQKVHCLNTLFSKLQINQSIIFCNSVNRVELLAKKITELGYSCFYIHAKMLQDHRNRVFH

DFRNGACRNLVCTDLFTRGIDIQAVNVVINFDFPKNSETYLHRVGRSGRFGHLGLAVNLITYEDRFNLYRIEQELGTEIK

QIPPHIDQAIYCR

>XP_030931886.1 DEAD-box ATP-dependent RNA helicase 8-like [Quercus lobata]

MNNNNRGRYPPGMGGMGRGGGMNANPPFQSRNPQQQYVQRSMLQNQQYQHHQQQQQQHHHHQQQQHHQQQQQHHQQQQQQ

QQQQQQQHWLRRGQLGGVDSSVDEVEKTVQSEAVDSSSQDWKARLKIPPPDTRFRTEDVTATKGNEFEDYFLKRELLMGI

YEKGFERPSPIQEESIPIALTGSDILARAKNGTGKTAAFCIPALEKIDQDNNVIQVVILVPTRELALQTSQVCKELGKHL

KIQVMVTTGGTSLKDDIMRLYQPVHLLVGTPGRILDLAKKGVCILKDCSMLVMDEADKLLSPEFQPSVEQLIRFLPTSRQ

ILMFSATFPVTVKDFKDRYLQKPYVINLMDELTLKGITQFYAFVEERQKVHCLNTLFSKLQINQSIIFCNSVNRVELLAK

KITELGYSCFYIHAKMLQDHRNRVFHDFRNGACRNLVCTDLFTRGIDIQAVNVVINFDFPKNSETYLHRVGRSGRFGHLG

LAVNLITYEDRFNLYRIEQELGTEIKQIPPHIDQAIYCR

>XP_035823623.1 uncharacterized protein LOC100194030 isoform X1 [Zea mays]

MDPRARYPSGMGNGRGGNLNYYGRGPPPQQPHQHHNHQQTSGAHHHQQTSGAHHHQQYAQRQHQHQHHGHNRHQQQQQHH

IYQQQQQQWLRRSQIARETSGAAVTSEPKSLAPSTAADGVDSSYTGTVLIHMCSSQDWKAQLKLPPPDTRYRTEDVTATK

GNEFEDYFLKRELLMGIYEKGFERPSPIQEESIPIALTGSDILARAKNGTGKTAAFCIPALEKIDQEKNAIQVVILVPTR

ELALQTSQVCKELGKHLKIQVMVTTGGTSLKDDIVRLYQPVHLLVGTPGRILDLTKKGVCILNDCSMLIMDEADKLLSPE

FQPSIEQLIRYLPASRQILMFSATFPVTVKEFKDKYLPKPYVINLMDELTLKGITQFYAFVEERQKVHCLNTLFSKLQIN

QSIIFCNSVNRVELLAKKITELGYSCFYIHAKMLQDHRNRVFHDFRNGACRNLVCTDLFTRGIDIQAVNVVINFDFPKNS

ETYLHRVGRSGRFGHLGLAVNLITYEDRFNLYRIEQELGTEIKSIPPQIDQAIYCQ

>VDC92130.1 unnamed protein product [Brassica oleracea]

MMNNNNRGGGRGIGAGRGAINPNPNFQSRPGYQQPQYVQRGGYSHQQQQFQQATSQPRQYQQQQQWLPRPQISSGNTNGG

GDAVVEVEKTVLSDTNSEDWKARLKLPAPDTRYRTEDVTATKGNEFEDYFLKRELLMGIYEKGFERPSPIQEESIPIALT

GRDILARAKNGTGKTAAFCIPVLEKIDQDNNVIQAVIIVPTRELALQTSQVCKELGKHLKIQVMVTTGGTSLKDDIMRLY

QPVHLLVGTPGRVLDLTKKGVCVLKDCSVLAMDEADKLLSQEFQPSVEHLISFLPQNRQILMFSATFPVTVKYFKDRFLT

NPYIINLMDELTLKGITQFYAFVEERQKIHCLNTLFSKLQINQSIIFCNSVNRVELLAKKITELGYSCFYIHAKMLQDHR

NRVFHDFRNGACRNLVCTDLFTRGIDIQAVNVVINFDFPKNAETYLHRVGRSGRFGHLGLAVNLITYEDRFNLYRIEQEL

GTEIKQIPPHIDQAIYCQ

>XP_025631329.1 DEAD-box ATP-dependent RNA helicase 8 [Arachis hypogaea]

MNNNNNYYNRARYPPPGIGGGGLGRGGGGGGPGFNQNSPFQPRPNYQQHQQQHQQLQQQHQQYVQRQMVQQQQQQQQQQQ

QQQWLRRAQMGAADSNVVDEVEKTVQSEAIDPSSQDWKARLKVPPPDTRYKTEDVTATKGNEFEDYFLKRELLMGIYEKG

FERPSPIQEESIPIALTGSDILARAKNGTGKTAAFCIPALEKIDQDNNVIQVVILVPTRELALQTSQVCKELGKHLKIQV

MVTTGGTSLKDDIMRLYQPVHLLVGTPGRILDLAKKGVCVLKDCAMLVMDEADKLLSPEFQPSIQQLIHFLPSHRQILMF

SATFPVTVKDFKDRYLQKPYVINLMDELTLKGITQYYAFVEERQKVHCLNTLFSKLQINQSIIFCNSVNRVELLAKKITE

LGYSCFYIHAKMLQDHRNRVFHDFRNGACRNLVCTDLFTRGIDIQAVNVVINFDFPKNSETYLHRVGRSGRFGHLGLAVN

LITYEDRFNLYRIEQELGTEIKQIPPHIDQAIYCR

>CAA09203.1 RNA helicase [Arabidopsis thaliana]

MNTNRGRYPPGVGTGRGAPPNPNYHQSYRQQQPPQDQQYVQRGYSQNPQQMQLQQQHQQQQQQQQWSKRPQLPENASNAN

EVVQQTTQPEASSDANGQNWKATLRLPPPDTRYQTADVTATKGNEFENYFLKRDLLKGIYEKGFEKPSPIQEESIPIALT

GSDILARAKNGTGKTGAFCIPVLEKIDPNNNVIQAMILVPTRELALQTSQVCKELSKYLNIQVMVTTGGTSLRDDIMRLH

QPVHLLVGTPGRILDLTKKGVCVLKDCAMLVMDEADKLLSAEFQPSLEELIQFLPQNRQFLMFSATFPVTVKAFKDRHLR

KPYVINLMDQLTLMGVTQYYAFVEERQKVHCLNTLFSKLQINQSIIFCNSVNRVELLAKKITELGYSCFYIHAKMVQDHR

NRVFHEFRNGACRNLVCTDLFTRGIDIQAVNVVINFDFPRTSESYLHRVGRSGRFGHLGLAVNLVTYEDRFKMYQTEQEL

GTEIKPIPSNIDQAIYCQ

>XP_013735282.1 DEAD-box ATP-dependent RNA helicase 8-like isoform X1 [Brassica napus]

MMNNNNNNNRGGGGGRYPPGIGAGRGAINPNPNFQSRPGYQQQQQPQYVQRGGYSHQQQFQQATSQPPRQYQQQQHQQQW

LPRPQISTGNSNGGGGGDAVVEVEKTVLSDTNSEDWKARLKLPAPDTRYRTEDVTATKGNEFEDYFLKRELLMGIYEKGF

ERPSPIQEESIPIALTGRDILARAKNGTGKTAAFCIPVLEKIDQDNNVIQAVIIVPTRELALQTSQVCKELGKHLKIQVM

VTTGGTSLKDDIMRLYQPVHLLVGTPGRILDLTKKGVCVLKDCSVLAMDEADKLLSQEFQPSVEHLISFLPQNRQILMFS

ATFPVTVKYFKDRFLTNPYIINLMDELTLKGITQFYAFVEERQKIHCLNTLFSKLQINQSIIFCNSVNRVELLAKKITEL

GYSCFYIHAKMLQDHRNRVFHDFRNGACRNLVCTDLFTRGIDIQAVNVVINFDFPKNAETYLHRVGRSGRFGHLGLAVNL

ITYEDRFNLYRIEQELGTEIKQIPPHIDQAIYCQ

>XP_016745625.1 PREDICTED: DEAD-box ATP-dependent RNA helicase 8-like [Gossypium hirsutum]

MNSRGRYPPPGIGVGRGGGVNANPSFQSRPSQQHYVQRNLVHNQQQFQQHNQQHFQQQQQHQQQQQWLRRNQLPSGNDSS

VIDEVEKTVQSEAVDSSSQDWKARLKIPPADTRYKTEDVTATKGNEFEDYFLKRELLMGIYEKGFERPSPIQEESIPIAL

TGSDILARAKNGTGKTAAFCIPALEKIDQDNNVIQVVILVPTRELALQTSQVCKELGKHLQIQVMVTTGGTSLKDDIMRL

YQPVHLLVGTPGRILDLAKKGVCILKDCSMLIMDEADKLLSPEFQPSVEQLICFLPATRQILLFSATFPVTVKDFKDRYL

QKPYIINLMDELTLKGITQYYAFVEERQKVHCLNTLFSKLQINQSIIFCNSVNRVELLAKKITELGYSCFYIHAKMLQDH

RNRVFHDFRNGACRNLVCTDLFTRGIDIQAVNVVINFDFPKNSETYLHRVGRSGRFGHLGLAVNLITYEDRFNLYRIEQE

LGTEIKQIPPHIDQAIYCR

>KHN04661.1 DEAD-box ATP-dependent RNA helicase 8 [Glycine soja]

MGLVRGGFNPNLSQNPNQNQNPSLNQNHHAFQARPPYHQQQQAQYVQRHLLQQQQQQQWLRRDANAVDEVEKTVQSEAVD

SSSQDWKARLKIPPADTRYKTEDVTATKGNEFEDYFLKRELLMGIYEKGFERPSPIQEESIPIALTGSDILARAKNGTGK

TAAFCIPALEKIDQDNNVIQVVILVPTRELALQTSQVCKELGKHLKIQVMVTTGGTSLKDDILRLYQPVHLLVGTPGRIL

DLAKKGVCILKDCAMLVMDEADKLLSPEFQPSIEQLIHFLPTTRQILMFSATFPVTVKDFKDRYLQKPYVINLMDELTLK

GITQFYAFVEERQKVHCLNTLFSKLQINQSIIFCNSVNRVELLAKKITELGYSCFYIHAKMLQDHRNRVFHDFRNGACRN

LVCTDLFTRGIDIQAVNVVINFDFPKNAETYLHRVGRSGRFGHLGLAVNLITYEDRFNLYRIEQELGTEIKQIPPQIDQA

IYCR

>MBA0672704.1 hypothetical protein [Gossypium klotzschianum]

MNSRGRYPTPGIGVGRGGGVNANPSFQSRPSQQHYVQRNLIHNQQHFQQHNQQHFQQQQQHQQQQQWLRRNQLPSGNDSS

VIDEVEKTVQSEAVDSSSQDWKARLKIPPADTRYKTEDVTATKGNEFEDYFLKRELLMGIYEKGFERPSPIQEESIPIAL

TGSDILARAKNGTGKTAAFCIPALEKIDQDNNVIQVVILVPTRELALQTSQVCKELGKHLQIQVMVTTGGTSLKDDIMRL

YQPVHLLVGTPGRILDLAKKGVCILKDCSMLIMDEADKLLSPEFQPSVEQLIRFLPATRQILLFSATFPVTVKDFKDRYL

QKPYIINLMDELTLKGITQYYAFVEERQKVHCLNTLFSKLQINQSIIFCNSVNRVELLAKKITELGYSCFYIHAKMLQDH

RNRVFHDFRNGACRNLVCTDLFTRGIDIQAVNVVINFDFPKNSETYLHRVGRSGRFGHLGLAVNLITYEDRFNLYRIEQE

LGTEIKQIPPHIDQAIYCR

>KHG12667.1 DEAD-box ATP-dependent RNA helicase 8 -like protein [Gossypium arboreum]

MNSRGRYPPPGIGVGRGGGVNANPSFQSRPSQQHYVQRNLVHNQQHFQQHNQQHFQQQQQWLRRNHLPGGNDSSVIDEVE

KTVQSEAVDSSSQDWKARLKIPPADTRYKTEDVTATKGNEFEDYFLKRELLMGIYEKGFERPSPIQEESIPIALTGSDIL

ARAKNGTGKTAAFCIPALEKIDQDNNVIQVVILVPTRELALQTSQVCKELGKHLQIQVMVTTGGTSLKDDIMRLYQPVHL

LVGTPGRILDLAKKGVCILKDCSMLIMDEADKLLSPEFQPSIEQLIRFLPANRQILMFSATFPVTVKDFKDRYLKKPYII

NLMDELTLKGITQYYAFVEERQKVHCLNTLFSKLQINQSIIFCNSVNRVELLAKKITELGYSCFYIHAKMLQDHRNRVFH

DFRNGACRNLVCTDLFTRGIDIQAVNVVINFDFPKNSETYLHRVGRSGRFGHLGLAVNLITYEDRFNLYRIEQELGTEIK

QIPPHIDQAIYCR

>XP_024175594.1 DEAD-box ATP-dependent RNA helicase 8 [Rosa chinensis]

MNNNRGRYPPGIGAGRGGGMNANPPFQSRPPHQQQYVQRNLLPNHQQQQQYFQQQQHHQQQQQQHHHQQQQWLRRGQLGG

STSADSAVDEVEKTVQSEAVDPSSQDWKARLKIPPADTRFRTEDVTATKGNEFEDYFLKRELLMGIYEKGFERPSPIQEE

SIPIALTGSDILARAKNGTGKTAAFCIPALEKIDQDNNVIQVVILVPTRELALQTSQVCKELGKHLQIQVMVTTGGTSLK

DDIMRLYQPVHLLVGTPGRILDLSKKGVCILKDCSMLVMDEADKLLSPEFQPSVEQLIRFLPSNRQILMFSATFPVTVKD

FKERYLHKPYVINLMDELTLKGITQFYAFVEERQKVHCLNTLFSKLQINQSIIFCNSVNRVELLAKKITELGYSCFYIHA

KMLQDHRNRVFHDFRNGACRNLVCTDLFTRGIDIQAVNVVINFDFPKNSETYLHRVGRSGRFGHLGLAVNLITYEDRFNL

YRIEQELGTEIKQIPPHIDQAIYCR

>KAB2074469.1 hypothetical protein ES319_A07G155400v1 [Gossypium barbadense]

MNSRGRYPPPGIGVGRGGGVNANPSFQSRPSQQHYVQRNLVHNQQHFQQHNQQHFQQQQQWLRRNQLPGGNDSSVIDEVE

KTVQSEAVDSSSQDWKARLKIPPADTRYKTEDVTATKGNEFEDYFLKRELLMGIYEKGFERPSPIQEESIPIALTGSDIL

ARAKNGTGKTAAFCIPALEKIDQDNNVIQVVILVPTRELALQTSQVCKELGKHLQIQVMVTTGGTSLKDDIMRLYQPVHL

LVGTPGRILDLAKKGVCILKDCSMLIMDEADKLLSPEFQPSVEQLIHFLPATRQILLFSATFPVTVKDFKDRYLQKPYII

NLMDELTLKGITQYYAFVEERQKVHCLNTLFSKLQINQSIIFCNSVNRVELLAKKITELGYSCFYIHAKMLQDHRNRVFH

DFRNGACRNLVCTDLFTRGIDIQAVNVVINFDFPKNSETYLHRVGRSGRFGHLGLAVNLITYEDRFNLYRIEQELGTEIK

QIPPHIDQAIYCR

>RDY13126.1 DEAD-box ATP-dependent RNA helicase 8, partial [Mucuna pruriens]

MNNNNRARYPPGMGLGRGGFGPNLNQNPNLNQNVNQNPNLNQNHLFQARPPYQQQQPQYMQRHFVQPPQQQQQWLRRDSN

AVDEVEKTVQSEAVDSSSQDWKARLKIPPADTRYKTEDVTATKGNEFEDYFLKRELLMGIYEKGFERPSPIQEESIPIAL

TGSDILARAKNGTGKTAAFCIPSLEKIDQDNNVIQVVILVPTRELALQTSQVCKELGKHLKIQVMVTTGGTSLKDDIMRL

YQPVHLLVGTPGRILDLAKKGVCVLKDCSMLVMDEADKLLSPEFQPSIEQLIQFLPRNRQILMFSATFPVTVKDFKDRYL

QKPYVINLMDELTLKGITQFYAFVEERQKVHCLNTLFSKLQINQSIIFCNSVNRVELLAKKITELGYSCFYIHAKMLQDH

RNRVFHDFRNGACRNLVCTDLFTRGIDIQAVNVVINFDFPKNSETYLHRVGRSGRFGHLGLAVNLITYEDRFNLYRIEQE

LGTEIKQIPPQIDQAIYCR

>TYI73928.1 hypothetical protein E1A91_D07G163800v1 [Gossypium mustelinum]

MNSRGRYPPPGIGVGRGGGVNANPSFQSRPSQQHYVQRNLVHNQQQFQQHNQQHFQQQQQHQQQQQWLRRNQLPSGNDSS

VIDEVEKTVQSEAVDSSSQDWKARLKIPPADTRYKTEDVTATKGNEFEDYFLKRELLMGIYEKGFERPSPIQEESIPIAL

TGSDILARAKNGTGKTAAFCIPALEKIDQDNNVIQVVILVPTRELALQTSQVCKELGKHLQIQVMVTTGGTSLKDDIMRL

YQPVHLLVGTPGRILDLAKKGVCILKDCSMLIMDEADKLLSPEFQPSVEQLIRFLPATRQILLFSATFPVTVKDFKDRYL

QKPYIINLMDELTLKGITQYYAFVEERQKVHCLNTLFSKLQINQSIIFCNSVNRVELLAKKITELGYSCFYIHAKMLQDH

RNRVFHDFRNGACRNLVCTDLFTRGIDIQAVNVVINFDFPKNSETYLHRVGRSGRFGHLGLAVNLITYEDRFNLYRIEQE

LGTEIKQIPPHIDQAIYCR

>TYG61712.1 hypothetical protein ES288_D07G170900v1 [Gossypium darwinii]

MNSRGRYPPPGIGVGRGGGVNANPSFQSRPSQQHYVQRNLVHNQQQFQQQQQHQQQQQWLRRNQLPSGNDYSVIDEVEKT

VQSEAVDSSSQDWKARLKIPPADTRYKTEDVTATKGNEFEDYFLKRELLMGIYEKGFERPSPIQEESIPIALTGSDILAR

AKNGTGKTAAFCIPALEKIDQDNNVIQVVILVPTRELALQTSQVCKELGKHLQIQVMVTTGGTSLKDDIMRLYQPVHLLV

GTPGRILDLAKKGVCILKDCSMLIMDEADKLLSPEFQPSVEQLICFLPATRQILLFSATFPVTVKDFKDRYLQKPYIINL

MDELTLKGITQYYAFVEERQKVHCLNTLFSKLQINQSIIFCNSVNRVELLAKKITELGYSCFYIHAKMLQDHRNRVFHDF

RNGACRNLVCTDLFTRGIDIQAVNVVINFDFPKNSETYLHRVGRSGRFGHLGLAVNLITYEDRFNLYRIEQELGTEIKQI

PPHIDQAIYCR

>KHG21932.1 DEAD-box ATP-dependent RNA helicase 8 [Gossypium arboreum]

MNSRGRYPPPGIGVGRGGGVNANPSFQSRPSQQHYVQRNLVHNQQHFQQHNQQHFQQQQQWLRRNHLPGGNDSSVIDEVE

KTVQSEAVDSSSQDWKARLKIPPADTRYKTEDVTATKGNEFEDYFLKRELLMGIYEKGFERPSPIQEESIPIALTGSDIL

ARAKNGTGKTAAFCIPALEKIDQDNNVIQVVILVPTRELALQTSQVCKELGKHLQIQVMVTTGGTSLKDDIMRLYQPVHL

LVGTPGRILDLAKKGVCILKDCSMLIMDEADKLLSPEFQPSVEQLIRFLPATRQILLFSATFPVTVKDFKDRYLQKPYII

NLMDELTLKGITQYYAFVEERQKVHCLNTLFSKLQINQSIIFCNSVNRVELLAKKITELGYSCFYIHAKMLQDHRNRVFH

DFRNGACRNLVCTDLFTRGIDIQAVNVVINFDFPKNSETYLHRVGRSGRFGHLGLAVNLITYEDRFNLYRIEQELGTEIK

QIPPHIDQAIYCR

>XP_017609604.1 PREDICTED: DEAD-box ATP-dependent RNA helicase 8-like [Gossypium arboreum]

MNSRGRYPPPGIGVGRGGGVNANPSFQSRPSQQHYVQRNLVHNQQHFQQHNQQHFQQQQQWLRRNQLPGGNDSSVIDEVE

KTVQSEAVDSSSQDWKARLKIPPADTRYKTEDVTATKGNEFEDYFLKRELLMGIYEKGFERPSPIQEESIPIALTGSDIL

ARAKNGTGKTAAFCIPALEKIDQDNNVIQVVILVPTRELALQTSQVCKELGKHLQIQVMVTTGGTSLKDDIMRLYQPVHL

LVGTPGRILDLAKKGVCILKDCSMLIMDEADKLLSPEFQPSVEQLIRFLPATRQILLFSATFPVTVKDFKDRYLQKPYII

NLMDELTLKGITQYYAFVEERQKVHCLNTLFSKLQINQSIIFCNSVNRVELLAKKITELGYSCFYIHAKMLQDHRNRVFH

DFRNGACRNLVCTDLFTRGIDIQAVNVVINFDFPKNSETYLHRVGRSGRFGHLGLAVNLITYEDRFNLYRIEQELGTEIK

QIPPHIDQAIYCR

>XP_019171156.1 PREDICTED: DEAD-box ATP-dependent RNA helicase 8-like [Ipomoea nil]

MNSRGRYPPGIGNGNGRGGGGSGYGGGGFRSGHNSGGYNQPRNPHYQYAQQNPPQPQQYGQRTLQNQPQQQQQQQQQQWL

RRNPSAAASESSSNEVLKTIQSEGIDSSSQDWKARLNIPAPDTRYRTEDVTATKGNEFEDYFLKRELLMGIYEKGFERPS

PIQEESIPIALTGSDILARAKNGTGKTAAFCIPALEKIDTETNVIQVVILVPTRELALQTSQVCKELGKHLKIQVMVSTG

GTNLKDDIMRLYQPVHLLVGTPGRILDLAKKGICILKDCAMLVMDEADKLLSPEFQPSVEHLITFLPEYRQILMFSATFP

VTVKDFKDRYLRKPYVINLMDELTLKGITQYYAFVEERQKVHCLNTLFSKLQINQSIIFCNSVNRVELLAKKITELGYSC

FYIHAKMLQDHRNKVFHDFRNGACRNLVCTDLFTRGIDIQAVNVVINFDFPKNSETYLHRVGRSGRFGHLGLAVNLITYE

DRFNLYKIEQELGTEIKPIPPHIDQAIYCQ

>KAF6148694.1 hypothetical protein GIB67_003785 [Kingdonia uniflora]

MEMKDLDAALQYDKKIRDDKCFEDIVYSDKKKSTKEEGILQGSGISRGLCELKPNFQPRNPHQQYAQRHPVQNQNVPAAA

AAAATAKQQQQQQQWLRRVPTGTDSSWNEVEKRCSPRASIQEHETLVGKHYEYHNLSQLSSTKFIIDLEKEYILKEENGL

GISQDWKARLKIPPSDTRYRTEDVTATKGNEFEDYFLKRELLMGIYEKGFERPSPIQEESIPIALTGSDILARAKNGTGK

TAAFCIPALEKIDQDNNVIQVVILVPTRELALQTSQVCKELGKHLKIQVMVTTGGTSLKDDIMRLYQPVHLLVGTPGRIL

DLARKGVCVLKDCSMLVLDEADKLLSPEFQPSIEQLIRFLPGSRQILLFSATFPVTVKDFKDRYLQKPYVINLMDELTLK

GITQFYAFVEERQKVHCLNTLFSKLQINQSIIFCNSVNRVELLAKKITELGYSCFYIHAKMLQDHRNRVFHDFRNGACRN

LVCTDLFTRGIDIQAVNVVINFDFPKNSETYLHRVGRSGRFGHLGLAVNLITYEDRFNLYRIEQELGTEIKQIPPQIDQA

IYCR

>CAA09199.1 RNA helicase [Arabidopsis thaliana]

MNNRGRYPPGIGAGRGAFNPNPNYQSRSGYQQHPPPQYVQRGNYAQNHQQQFQQAPSQPHQYQQQQQQQQQWLRRGQIPG

GNSNGDAVVEVEKTVQSEVIDPNSEDWKARLKLPAPDTRYRTEDVTATKGNEFEDYFLKRELLMGIYEKGFERPSPIQEE

SIPIALTGRDILARAKNGTGKTAAFCIPVLEKIDQDNNVIQAVIIVPTRELALQTSQVCKELGKHLKIQVMVTTGGTSLK

DDIMRLYQPVHLLVGTPGRILDLTKKGVCVLKDCSVLVMDEADKLLSQEFQPSVEHLISFLPESRQILMFSATFPVTVKD

FKDRFLTNPYVINLMDELTLKGITQFYAFVEERQKIHCLNTLFSKLQINQSIIFCNSVNRVELLAKKITELGYSCSYIHA

KMLQDHRNRVFHDFRNGACRNLVCTDLFTRGIDIQAVNVVINFDFPKNAETYLHRVGRSGRFGHLGLAVNLITYEDRFNL

YRIEQELGTEIKQIPPHIDQAIYCQ

>XP_020881562.1 DEAD-box ATP-dependent RNA helicase 12 [Arabidopsis lyrata subsp. lyrata]

MNNRGRYPPGVGTGRGAPPNPDYQSYRQQQPQDQQYVQRGYSQNPQQMQLQQQHHQQQQQQQWSRRSQLPGNASNANEVQ

KTSQPEASSDANGQDWKATLRLPPPDTRYQTADVTATKGNEFEDYFLKRDLLKGIYEKGFEKPSPIQEESIPIALTGSDI

LARAKNGTGKTGAFCIPVLEKIDPSNNVIQAMILVPTRELALQTSQVCKELSKYLNIQVMVTTGGTSLRDDIMRLHQPVH

LLVGTPGRILDLTKKGVCVLKDCAMLVMDEADKLLSAEFQPSLEELIQFLPQNRQFLMFSATFPVTVKAFKDRHLRKPYV

INLMDQLTLMGVTQYYAFVEERQKVHCLNTLFSKLQINQSIIFCNSVNRVELLAKKITELGYSCFYIHAKMVQDHRNRVF

HEFRNGACRNLVCTDLFTRGIDIQAVNVVINFDFPRTSESYLHRVGRSGRFGHLGLAVNLVTYEDRFKMYQTEQELGTEI

KPIPSNIDQAIYCQ

>XP_010540277.1 PREDICTED: DEAD-box ATP-dependent RNA helicase 8 [Tarenaya hassleriana]

MNTGGRYPPGIGAGRGSINANNPGSQSRPVYQQQPQYGQRGNYAQNHQRFQQAPPPPPHQQQQQWLRRAQFTGGNNGDAV

DEVEKTVQSEAIDSNSQDWKARLKLPQPDTRYKTEDVTATKGNEFEDYFLKRELLMGIYEKGFERPSPIQEESIPIALTG

SDILARAKNGTGKTAAFCIPALEKIDQDNNAIQVVILVPTRELALQTSQVCKELGKHLKIQVMVTTGGTSLKDDIMRLYQ

PVHLLVGTPGRILDLTKKGICVLKDCAMLVMDEADKLLSQEFQPSVEQLIRFLPGNRQILMYSATFPVTIKDFKDRYLRK

PYIINLMDELTLKGITQFYAFVEERQKVHCLNTLFSKLQINQSIIFCNSVNRVELLAKKITELGYSCFYIHAKMLQDHRN

RVFHDFRNGACRNLVCTDLFTRGIDIQAVNVVINFDFPKNSETYLHRVGRSGRFGHLGLAVNLITYEDRFNLYRIEQELG

TEIKQIPPHIDQAIYCQ

>XP_027907687.1 DEAD-box ATP-dependent RNA helicase 8-like [Vigna unguiculata]

MNHHHNNRARYPPGMGLGRGGFHPNVGHNPNLNQNPGLNQNQAFQPRPPYQQQPHYVQRHLMQQPQQQQQWLRRDANAVD

EVEKTVQSEAVDSSSQDWKARLKIPPADTRYKTEDVTATKGNEFEDYFLKRELLMGIYEKGFERPSPIQEESIPIALTGS

DILARAKNGTGKTAAFCIPALEKIDQDNNVIQVVILVPTRELALQTSQVCKELGKHLKIQVMVTTGGTSLKDDIMRLYQP

VHLLVGTPGRILDLAKKGVCIMKDCSMLVMDEADKLLSPEFQPSIEQLIHFLPPNRQILMFSATFPVTVKDFKDRYLHKP

YVINLMDELTLKGITQYYAFVEERQKVHCLNTLFSKLQINQSIIFCNSVNRVELLAKKITELGYSCFYIHAKMLQDHRNR

VFHDFRNGACRNLVCTDLFTRGIDIQAVNVVINFDFPKNSETYLHRVGRSGRFGHLGLAVNLITYEDRFNLYRIEQELGT

EIKQIPPQIDQAIYCR

>XP_023752045.1 DEAD-box ATP-dependent RNA helicase 6-like [Lactuca sativa]

MNDSTGNNSYNRRYPPGIGNGRGGGGGWYGGGGGGGGNFQTNPNYHHQPRSHDQIQQQQHQSQYSQRQQSTQQHYNQKQQ

WLRTNPNVPVSSTANNNNYEVEKTVQSEGSVDSSSQDWKAQLNIPAADSRFKTEDVTATKGNEFEDYFLKRELLMGIYEK

GFERPSPIQEESIPIALTGSDILARAKNGTGKTAAFCIPALEKIDTDNNVIQVIILVPTRELALQTSQVCKELGKHLQIQ

VMVTTGGTSLKDDIMRLYQPVHLLVGTPGRILDLTKKGVCILNDCAMLVLDEADKLLSPEFQPSVEHLISFLPQNRQILM

FSATFPVTVKDFRDRYLKNPYVVNLMDELTLKGITQYYAFVEERQKVHCLNTLFSKLQINQSIIFCNSVNRVELLAKKIT

ELGYSCFYIHAKMLQDHRNRVFHDFRNGACRNLVCTDLFTRGIDIQAVNVVINFDFPRNAETYLHRVGRSGRFGHLGLAV

NLITYEDRFNLYRIEQELGTEIKQIPPQIDQAIYCL

>XP_018456211.1 PREDICTED: DEAD-box ATP-dependent RNA helicase 8 [Raphanus sativus]

MMNNNNNRGGGGGGGGGRYPPGIGAGRGAINNPNPNFQSRPGYQHQQQPPPQYVQRGGGGYAHQNHQQQFQQATSQQQQP

HRYQQQQQQQQQWLRRPPPQISPGNSNGDAVVEVEKTVLSEAIDTNSEDWKARLKLPAPDTRYRTEDVTATKGNEFEDYF

LKRDLLMGIYEKGFERPSPIQEESIPIALTGRDILARAKNGTGKTAAFCIPVLEKIDQDNNVIQAVIIVPTRELALQTSQ

VCKEMGKHLKIQVMVTTGGTSLKDDIMRLYQPVHLLVGTPGRILDLTKKGVCVLKDCSVFVMDEADKLLSQEFQPSVEHL

ISFLPQNRQILMFSATFPVTVKDFKDRFLTNPYIINLMDELTLKGITQFYAFVEERQKIHCLNTLFSKLQINQSIIFCNS

VNRVELLAKKITELGYSCFYIHAKMLQDHRNRVFHDFRNGACRNLVCTDLFTRGIDIQAVNVVINFDFPKTAETYLHRVG

RSGRFGHLGLAVNLITYEDRFNLYRIEQELGTEIKQIPPHIDQAIYCQ

>KAB2017434.1 hypothetical protein ES319_D08G163500v1 [Gossypium barbadense]

MNNRGRYPSVLGRGRGANANPSFQSRPEQPQYAQRNLVQNHHHFQQQQHHHHHLQQQQHQQQWLRRNQLPGGNDSTVADE

VEKTVQSEAVDSSSQDWKARIKMPPSDTRYKTEDVTATKGNEFEDYFLKRELLMGIYEKGFERPSPIQEESIPIALTGSD

ILARAKNGTGKTAAFCIPALEKIDQDNNVIQVVILVPTRELALQTSQVCKELGKHLQIQVMVTTGGTSLKDDIMRLYQPV

HLLVGTPGRILDLAKKGVCILKNCSMLIMDEADKLLSPEFQPSLEQLLCFLPPNRQILMFSATFPVTVKDFKDKYLKKPY

IINLMDELTLKGITQYYAFVEERQKVHCLNTLFSKLQINQSIIFCNSVNRVELLAKKITELGYSCFYIHAKMLQDHRNRV

FHDFRNGACRNLVCTDLFTRGIDIQAVNVVINFDFPKNSETYLHRVGRSGRFGHLGLAVNLITYEDRFNLYRIEQELGTE

IKQIPPHIDQAIYCR

>XP_004306806.1 PREDICTED: DEAD-box ATP-dependent RNA helicase 8 [Fragaria vesca subsp. vesca]

MNNNNRGRYPPGIGAGRGGGGGMNANPPFQSRPPHQQQQYVQRNLLPNQQQQQQYYQQQQQHHHQQQQHHQQQQQQWLRR

GQLGGSTSADSAVDEVEKTVQSEAVDPSSQDWKARLKIPPADTRFRTEDVTATKGNEFEDYFLKRELLMGIYEKGFERPS

PIQEESIPIALTGSDILARAKNGTGKTAAFCIPALEKIDQDNNVIQVVILVPTRELALQTSQVCKELGKHLKIQVMVTTG

GTSLKDDIMRLYQPVHLLVGTPGRILDLSKKGVCILKDCSMLVMDEADKLLSPEFQPSVEQLIRFLPSNRQILMFSATFP

VTVKDFKDRYLHKPYVINLMDELTLKGITQFYAFVEERQKVHCLNTLFSKLQINQSIIFCNSVNRVELLAKKITELGYSC

FYIHAKMLQDHRNRVFHDFRNGACRNLVCTDLFTRGIDIQAVNVVINFDFPKNSETYLHRVGRSGRFGHLGLAVNLITYE

DRFNLYRIEQELGTEIKQIPPHIDQAIYCR

>XP_020233165.1 DEAD-box ATP-dependent RNA helicase 8 isoform X1 [Cajanus cajan]

MNTNNNRERYPPGIGLARGAFNPNLHLNRNPNLNHNLNHNHNHSFQVRPPFQQQPHYVQRHLLQPLQQQQQQQQQQQQQQ

WLRRDVNAVDEVEKTVQSEAVDSSSSQDWKARLKIPPPDTRYRTEDVTATKGNEFEDYFLKRELLMGIYEKGFERPSPIQ

EESIPIALTGSDILARAKNGTGKTAAFCIPALEKIDQDINVIQVVILVPTRELALQTSQVCKELGKHLKIQVMVTTGGTS

LKDDIMRLYQPVHLLVGTPGRILDLAKKGVCILKDCAMLVMDEADKLLSPEFQPSIEQLIHFLPTNRQILMFSATFPVTV

KDFKDRYLHKPYVINLMDELTLKGITQFYAFVEERQKVHCLNTLFSKLQINQSIIFCNSVNRVELLAKKITELGYSCFYI

HAKMLQDHRNRVFHDFRNGACRNLVCTDLFTRGIDIQAVNVVINFDFPKNSETYLHRVGRSGRFGHLGLAVNLITYEDRF

NLYRIEQELGTEIKQIPPQIDQAIYCR

>XP_027108320.1 DEAD-box ATP-dependent RNA helicase 8-like [Coffea arabica]

MYPRARYPPGIGGPGAGRGGGNMNPNASPNFQPRNPQQYLPRNPMQFQNQPPPQQQQQWLRRTQLGSDSAVEEVEKTIQS

EGVDSSSQDWKAKLKIPPADTRYKTEDVTATKGNEFEDYFLKRELLMGIYEKGFERPSPIQEESIPIALTGSDILARAKN

GTGKTAAFCIPALEKIDQDNNVIQVVILVPTRELALQTSQVCKELGKHLNIEVMVTTGGTSLKDDIMRLYQPVHLLVGTP

GRILDLSKKGVCILKDCAMLIMDEADKLLSPEFQPSIEQLIRFLPGNRQILMFSATFPVTVKDFKDRYLKKPYVINLMDE

LTLKGITQYYAFVEERQKVHCLNTLFSKLQINQSIIFCNSVNRVELLAKKITELGYSCFYIHAKMLQDHRNRVFHDFRNG

ACRNLVCTDLFTRGIDIQAVNVVINFDFPKNSETYLHRVGRSGRFGHLGLAVNLITYEDRFNLYRIEQELGTEIKQIPPH

IDQAIYCR

>PWA82726.1 Helicase [Artemisia annua]

MNNNNGRGGGVGGGGGRYPPGINRGGGGGNYYGNTNPNFQQQRNYQQHQQQAQQVQQQQQQQWLRRNHSSGANEVEKTVQ

SETIDSSSEDWKAQLRLPPADTRYRTEDVTATKGNEFEDYFLKRDLLMGIYEKGFERPSPVQEESIPIALTGSDILARAK

NGTGKTAAFCIPVLEKIDQDNNVIQAVILVPTRELALQTSQVCKELGKHLNIQVMVTTGGTSLKDDIMRLYQPVHLLVGT

PGRILDLANKGICRLNNCAMLVMDEADKLLSPEFQPSVEQLIRFTPANRQILMFSATFPVTVKDFKERYLRKPYIINLMD

ELTLKGITQFYAFVEERQKVHCLNTLFSKLQINQSIIFCNSVNRVELLAKKITELGYSCFYIHAKMLQDHRNRVFHDFRN

GACRNLVCTDLFTRGIDIQAVNVVINFDFPKNSETYLHRVGRSGRFGHLGLAVNLITYEDRYNLYRIEQELGTEIKQIPP

FIDQAIYCR

>NP_191683.1 DEA(D/H)-box RNA helicase family protein [Arabidopsis thaliana]

MNTNRGRYPPGVGTGRGAPPNPDYHQSYRQQQPPQDQQYVQRGYSQNPQQMQLQQQHQQQQQQQQWSRRPQLPGNASNAN

EVVQQTTQPEASSDANGQDWKATLRLPPPDTRYQTADVTATKGNEFEDYFLKRDLLKGIYEKGFEKPSPIQEESIPIALT

GSDILARAKNGTGKTGAFCIPVLEKIDPNNNVIQAMILVPTRELALQTSQVCKELSKYLNIQVMVTTGGTSLRDDIMRLH

QPVHLLVGTPGRILDLTKKGVCVLKDCAMLVMDEADKLLSAEFQPSLEELIQFLPQNRQFLMFSATFPVTVKAFKDRHLR

KPYVINLMDQLTLMGVTQYYAFVEERQKVHCLNTLFSKLQINQSIIFCNSVNRVELLAKKITELGYSCFYIHAKMVQDHR

NRVFHEFRNGACRNLVCTDLFTRGIDIQAVNVVINFDFPRTSESYLHRVGRSGRFGHLGLAVNLVTYEDRFKMYQTEQEL

GTEIKPIPSNIDQAIYCQ

>XP_022770405.1 DEAD-box ATP-dependent RNA helicase 8-like [Durio zibethinus]

MNSRGRYPLGIGVGRGGGVNANPNFQSRLPQQHYVQRNLVQNHHQFQHQQQQQHQHQQQQQQQWLRRNHLPGGNDTSVVD

EVEKTVQSEAVDSSSQDWKARLKIPPPDTRYKTEDVTATKGNEFEDYFLKRELLMGIYEKGFERPSPIQEESIPIALTGS

DILARAKNGTGKTAAFCIPALEKIDQDSNVIQAVILVPTRELALQTSQVCKELGKHLQIQVMVTTGGTSLKDDIMRLYQP

VHLLVGTPGRIIDLAKKGVCILKDCSMLIMDEADKLLSPEFQPSVEQLIRFLPANRQILMFSATFPVTVKDFKDRYLQKP

YIINLMDELTLKGITQYYAFVEERQKVHCLNTLFSKLQINQSIIFCNSVNRVELLAKKITELGYSCFYIHAKMLQDHRNR

VFHDFRNGACRNLVCTDLFTRGIDIQAVNVVINFDFPKNSETYLHRVGRSGRFGHLGLAVNLITYEDRFNLYRIEQELGT

EIKQIPPHIDQAIYCQ

>CDP08841.1 unnamed protein product [Coffea canephora]

MYPRARYPPGIGGPGAGRGGGNMNPNASPNFQPRNPQQYLPRNPMQFQNQPPPQQQQQWLRRTQLGSDSAVEEVEKTIQS

EGVDSSSQDWKAKLKIPPADTRYKTEDVTATKGNEFEDYFLKRELLMGIYEKGFERPSPIQEESIPIALTGSDILARAKN

GTGKTAAFCIPALEKIDQDNNVIQVVILVPTRELALQTSQVCKELGKHLNIEVMVTTGGTSLKDDIMRLYQPVHLLVGTP

GRILDLAKKGVCILKDCAMLIMDEADKLLSPEFQPSIEQLIRFLPGNRQILMFSATFPVTVKDFKDRYLKKPYVINLMDE

LTLKGITQYYAFVEERQKVHCLNTLFSKLQINQSIIFCNSVNRVELLAKKITELGYSCFYIHAKMLQDHRNRVFHDFRNG

ACRNLVCTDLFTRGIDIQAVNVVINFDFPKNSETYLHRVGRSGRFGHLGLAVNLITYEDRFNLYRIEQELGTEIKQIPPH

IDQAIYCR

>XP_031407104.1 DEAD-box ATP-dependent RNA helicase 8-like [Punica granatum]

MNNRARYPPGIGAGRGGAGMNAGPAFQSRTPQMQYVQRNQLGQQQYQQNYPQQPQPQHPQHQHHHWPRRPQIGGAEASVD

EVEKTVQSEAVDTSSQDWKERLKIPPPDTRYRTEDVTATKGNEFEDYFLKRELLMGIYEKGFERPSPIQEESIPIALTGS

DILARAKNGTGKTAAFCIPALEKIDQDNNVIQVVILVPTRELALQTSQVCKELGKHLKIQVMVTTGGTSLKDDIMRLYQP

VHLLVGTPGRILDLAKKGICILKDCSMLVMDEADKLLSPEFQPSVEQLIRFMPANRQILMFSATFPVTVKDFKERYLQKP

YIINLMDELTLKGITQFYAFVEERQKVHCLNTLFSKLQINQSIIFCNSVNRVELLAKKITELGYSCFYIHAKMLQDHRNR

VFHDFRNGACRNLVCTDLFTRGIDIQAVNVVINFDFPKNSETYLHRVGRSGRFGHLGLAVNLITYEDRFNLYRIEQELGT

EIKQIPPHIDQAIYCR

>XP_010676491.1 PREDICTED: DEAD-box ATP-dependent RNA helicase 8-like [Beta vulgaris subsp. vulgaris]

MNHNARPRYPPGIGNGRGGGSGGGNYAQQNPNFQNRPSYHNNQYHDQPQYVQRRAPQQNYQQHQNQYQQQQQQHQQQQQQ

QQQQQWMMRRPANTSSDNAGSTQSSSNQVEKSNQTTNVDSSAEDWKASLKLPPPDTRYQTEDVTATKGNEFEDYFLKREL

LMGIYEKGFERPSPIQEESIPIALTGSDILARAKNGTGKTAAFCIPVLEKIDQDNNVIQAVILVPTRELALQTSQVCKEL

GKHLQIQVMVTTGGTSLKDDIMRLYQPVHLLVGTPGRILDLAKKGICILKDCSMLVMDEADKLLSPEFQPSLEQLIRFLP

GNRQVLMFSATFPVTVKDFKDRYLEKPYVINLMDELTLKGITQFYAFVEERQKVHCLNTLFSKLQINQSIIFCNSVNRVE

LLAKKITELGYSCFYIHAKMLQDHRNRVFHDFRNGACRNLVCTDLFTRGIDIQAVNVVINFDFPKNSETYLHRVGRSGRY

GHLGLAVNLITYEDRFNLYKIEQELGTEIKQIPPHIDQGIYCR

>TYI19448.1 hypothetical protein ES332_A07G165800v1 [Gossypium tomentosum]

MNSRGRYPPPGIGVGRGGGVNANPSFQSRPSQQHYVQRNLVHNQQHFQQHNQQHFQQQQQWLRRNQLPGGNDSSVIDEVE

KTVQSEAVDSSSQDWKARLKIPPADTRYKTEDVTATKGNEFEDYFLKRELLMGIYEKGFERPSPIQEESIPIALTGSDIL

ARAKNGTGKTAAFCIPALEKIDQDNNVIQVVILVPTRELALQTSQVCKELGKHLQIQVMVTTGGTSLKDDIMRLYQPIHL

LVGTPGRILDLAKKGVCILKDCSMLIMDEADKLLSPEFQPSVEQLIRFLPATRQILLFSATFPVTVKDFKDRYLQKPYII

NLMDELTLKGITQYYAFVEERQKVHCLNTLFSKLQINQSIIFCNSVNRVELLAKKITELGYSCFYIHAKMLQDHRNRVFH

DFRNGACRNLVCTDLFTRGIDIQAVNVVINFDFPKNSETYLHRVGRSGRFGHLGLAVNLITYEDRFNLYRIEQELGTEIK

QIPPHIDQAIYCR

>XP_027115146.1 DEAD-box ATP-dependent RNA helicase 8-like [Coffea arabica]

MYPRARYPPGIGGPGAGRGGGNMNPNASPNFQPRNPQQYLPRNPMQFQNQPPPQQQQQWLRRTQLGSDSAVEEVEKTIQS

EAVDSSSQDWKAKLKIPPADTRYKTEDVTATKGNEFEDYFLKRELLMGIYEKGFERPSPIQEESIPIALTGSDILARAKN

GTGKTAAFCIPALEKIDQDSNIIQVVILVPTRELALQTSQVCKELGKHLNIEVMVTTGGTSLKDDIMRLYQPVHLLVGTP

GRILDLSKKGVCILKDCAMLIMDEADKLLSPEFQPSIEQLIRFLPGNRQILMFSATFPVTVKDFKDRYLKKPYVINLMDE

LTLKGITQYYAFVEERQKVHCLNTLFSKLQINQSIIFCNSVNRVELLAKKITELGYSCFYIHAKMLQDHRNRVFHDFRNG

ACRNLVCTDLFTRGIDIQAVNVVINFDFPKNSETYLHRVGRSGRFGHLGLAVNLITYEDRFNLYRIEQELGTEIKQIPPH

IDQAIYCR

>XP_020094993.1 DEAD-box ATP-dependent RNA helicase 8-like [Ananas comosus]

MNPRGRYPPGMDNGRGGHMGGNPNYYGRNPQLQPQLQPQPQSQYVQRNPLQGQQQFQTQQPLQQQWLRRNHMGSDSGTSE

VVKQLQPDTIDSSSQDWKAQLKIPPQDTRYRTEDVTATKGNEFEDYFLKRELLMGIYEKGFERPSPIQEESIPIALTGSD

ILARAKNGTGKTAAFCIPALEKIDQDRNVIQVVILVPTRELALQTSQVCKELGKHLNIQIMVTTGGTSLKDDIMRLHQPV

HMLIGTPGRILDLTKKGICILKDCSMLIMDEADKLLSPEFQPSIEQLIRFLPANRQILLFSATFPVTVKDFKDRYLPRPY

VINLMDELTLKGITQFYAFVEERQKVHCLNTLFSKLQINQSIIFCNSVNRVELLAKKITELGYSCFYIHAKMLQDHRNRV

FHDFRNGACRNLVCTDLFTRGIDIQAVNVVINFDFPKNAETYLHRVGRSGRFGHLGLAVNLITYEDRFNLHRIEQELGTE

IKPIPPQIDQAIYCQ

>KAE8662485.1 DEAD-box ATP-dependent RNA helicase 6 [Hibiscus syriacus]

MNSRGRNPPPGIGVGRGGGVNANPSFQSRPSQQQYVQRNLVQNQQQFQHHNQQHFQQQQQQWFRRNQLPGGNDFNVIHEV

EKTIETEAVDSSSHDWKARLKIPPADTRYKTEDVTATKGNEFEDYFLKRELLMGIYEKGFERPSPIQEESIPIALTGSDI

LARAKNGTGKTAAFCIPALEKIDQDNNVIQVVILVPTRELALQTSQVCKELGKHLQIQVMVTTGGTSLKDDIMRLYQPVH

LLVGTPGRILDLAKKGICILKDCSMLIMDEADKLLSPEFQPSIEQLIRFLPATRQILMFSATFPVTVKDFKDRYLQKPYI

INLMDELTLKGITQYYAFVEERQKVHCLNTLFSKLQINQSIIFCNSVNRVELLAKKITELGYSCFYIHAKMLQEHRNRVF

HDFRNGACRNLVCTDLFTRGIDIQAVNVVINFDFPKNSETYLHRVGRSGRFGHLGLAVNLITYEDRFNLYRIEQELGTEI

KQIPPHIDQAIYCR

>XP_012475171.1 PREDICTED: DEAD-box ATP-dependent RNA helicase 8 [Gossypium raimondii]

MNNRGRYPSVLGRGRGANANPSFQSRPEQPQYAQRNLVQNHHHHFQQQQHHHHHLQQQQHQQQWLRRNQLPGGNDSTVAD

EVEKTVQSEAVDSSSQDWKARLKMPPSDTRYKTEDVTATKGNEFEDYFLKRELLMGIYEKGFERPSPIQEESIPIALTGS

DILARAKNGTGKTAAFCIPALEKIDQDNNVIQVVILVPTRELALQTSQVCKELGKHLQIQVMVTTGGTSLKDDIMRLYQP

VHLLVGTPGRILDLAKKGVCILKNCSMLIMDEADKLLSPEFQPSLEQLLCFLPPNRQILMFSATFPVTVKDFKDRYLKKP

YIINLMDELTLKGITQYYAFVEERQKVHCLNTLFSKLQINQSIIFCNSVNRVELLAKKITELGYSCFYIHAKMLQDHRNR

VFHDFRNGACRNLVCTDLFTRGIDIQAVNVVINFDFPKNSETYLHRVGRSGRFGHLGLAVNLITYEDRFNLYRIEQELGT

EIKQIPPHIDQAIYCR

>TYI69566.1 hypothetical protein E1A91_D08G163900v1 [Gossypium mustelinum]

MNNRGRYPSVLGRGRGANANPSFQSRPEQPQYAQRNLVQNHHHFQQQQHHHHHLQQQQHQQQWLRRNQLPGGNDSTLADE

VEKTVQSEAVDSSSQDWKARIKMPPSDTRYKTEDVTATKGNEFEDYFLKRELLMGIYEKGFERPSPIQEESIPIALTGSD

ILARAKNGTGKTAAFCIPALEKIDQDNNVIQVVILVPTRELALQTSQVCKELGKHLQIQVMVTTGGTSLKDDIMRLYQPV

HLLVGTPGRILDLAKKGVCILKNCSMLIMDEADKLLSPEFQPSLEQLLCFLPPNRQILMFSATFPVTVKDFKDKYLKKPY

IINLMDELTLKGITQYYAFVEERQKVHCLNTLFSKLQINQSIIFCNSVNRVELLAKKITELGYSCFYIHAKMLQDHRNRV

FHDFRNGACRNLVCTDLFTRGIDIQAVNVVINFDFPKNSETYLHRVGRSGRFGHLGLAVNLITYEDRFNLYRIEQELGTE

IKQIPPHIDQAIYCR

>XP_016743614.1 PREDICTED: DEAD-box ATP-dependent RNA helicase 8-like [Gossypium hirsutum]

MNSRGRYPPPGIGVGRGGGVNANPSFQSRPSQQHYVQRNLVHNQQHFQQHNQQHFQQQQQWLRRNQLPGGNDSSVIDEVE

KTVQSEAVDSSSQDWKARLKIPPADTRYKTEDVTATKGNEFEDYFLKRELLMGIYEKGFERPSPIQEESIPIALTGSDIL

ARAKNGTGKTAAFCIPALEKIDQDNNVIQVVILVPTRELALQTSQVCKELGKHLQIQVMVTTGGTSLKDDIMRLYQPVHL

LVGTPGRILDLAKKGVCILKDCSMLIMDEADKLLSPEFQPSVEQLIRFLPATRQILLFSATFPVTVKDFKHRYLQKPYII

NLMDELTLKGITQYYAFVEERQKVHCLNTLFSKLQINQSIIFCNSVNRVELLAKKITELGYSCFYIHAKMLQDHRNRVFH

DFRNGACRNLVCTDLFTRGIDIQAVNVVINFDFPKNSETYLHRVGRSGRFGHLGLAVNLITYEDRFNLYRIEQELGTEIK

QIPPHIDQAIYCR

>CAA7023017.1 unnamed protein product [Microthlaspi erraticum]

MNNNNRGRYPPGIGTGRGAPPNPDYQSYRQQPQNQQQQRGQPQNPQQFQPQQQQQQQQWSRRPQLPGNANEVQKSSQPEA

SNHPNGQDWKATLRLPPPDARFQTADVTATKGNEFEDYFLKRDLLKGIYEKGFEKPSPIQEESIPIALTGSDILARAKNG

TGKTGAFCIPVLEKIDPNNNVIQGMILVPTRELALQTSQVCKELSKYLNIQVMVTTGGTSLRDDIMRLHQPVHLLVGTPG

RILDLTKKGVCVLKDCSMLVMDEADKLLSAEFQPSLEELIQFLPQNRQFLMFSATFPVTVKAFKDRHLRKPYVINLMDQL

TLVGVTQYYAFVEERQKVHCLNTLFSKLQINQSIIFCNSVNRVELLAKKITELGYSCFYIHAKMVQDHRNRVFHEFRNGA

CRNLVCTDLFTRGIDIQAVNVVINFDFPRTSESYLHRVGRSGRFGHLGLAVNLVTYEDRFKMYQTEQELGTEIKPIPSLI

DRAIYCS

>XP_031259996.1 DEAD-box ATP-dependent RNA helicase 8 [Pistacia vera]

MNNSRGRYPPGIGGGRGGINAANPTFQSNRPHQQYVQRNLVQNHHQQQFQQHQQQQQQQWLRRNNFVGADSSAVDEVEKT

VQSEAAIDPSSQDWKARLKIPPPDTRYRTEDVTATKGNEFEDYFLKRELLMGIYEKGFERPSPIQEESIPIALTGSDILA

RAKNGTGKTAAFCVPALEKIDQDNNVIQVVILVPTRELALQTSQVCKELGKHLKIQVMVTTGGTSLKDDIMRLYQPVHLL

VGTPGRILDLAKKGVCILKDCSMLVMDEADKLLSPEFQPSVEQLIRFLPGNRQILMFSATFPVTVKDFKDRYLAKPYVIN

LMDELTLKGITQYYAFVEERQKVHCLNTLFSKLQINQSIIFCNSVNRVELLAKKITELGYSCFYIHAKMLQDHRNRVFHD

FRNGACRNLVCTDLFTRGIDIQAVNVVINFDFPKNSETYLHRVGRSGRFGHLGLAVNLITYEDRFNLYRIEQELGTEIKQ

IPPHIDQAIYCR

>XP_009408278.1 PREDICTED: DEAD-box ATP-dependent RNA helicase 6 [Musa acuminata subsp. malaccensis]

MNPRGRYPPPGMGNGRGGSASTNPGFYSRNSHHQHHHQQQQQQYVQRNPVQVQPNQHFQQQQWSRRNQLGGDSGSGEVVK

SVQPETSDTSLQDWKAQLKMPPPDTRYKTEDVTATKGNEFEDYFLKRELLMGIYEKGFEKPSPIQEESIPIALTGSDILA

RAKNGTGKTAAFCIPALEKIDQDHNAIQVVILVPTRELALQTSQVCKELGKHLKIQVMVTTGGTSLKDDIMRLYQPVHLL

AGTPGRILDLAKKGVCVLKDCSMLVMDEADKLLSPEFQPSIEQLIQFLPANRQILMFSATFPVTVKDFKDRYLPKPYIIN

LMDELTLKGITQYYAFVEERQKVHCLNTLFSKLQINQSIIFCNSVNRVELLAKKITELGYSCFYIHAKMLQDHRNRVFHD

FRNGACRNLVCTDLFTRGIDIQAVNVVINFDFPKNSETYLHRVGRSGRFGHLGLAVNLITYEDRFNLYRIEQELGTEIKQ

IPPQIDQTIYCR

>XP_021653066.1 DEAD-box ATP-dependent RNA helicase 8 [Hevea brasiliensis]

MNNYNRGRYPPGISAGRGAGMNANPSFQSRVPQQQYVQRNLMQNHQQFQHQQQQQQQQQQQQQQWLRRSQLPPADSSVDE

VEKTVQSEAVDSSSQDWKARLKIPPPDTRYKTEDVTATKGNEFEDYFLKRELLMGIYEKGFERPSPIQEESIPIALTGSD

ILARAKNGTGKTAAFCIPALEKIDQDNNVIQVVILVPTRELALQTSQVCKELGKHLKIQVMVTTGGTSLKDDIMRLYQPV

HLLVGTPGRILDLAKKGVCILKDCSMLVMDEADKLLSPEFQPSVEQLIRFLPPTRQILMFSATFPVTVKDFKDRYLQKSY

IINLMDELTLKGITQYYAFVEERQKVHCLNTLFSKLQINQSIIFCNSVNRVELLAKKITELGYSCFYIHAKMLQDHRNRV

FHDFRNGACRNLVCTDLFTRGIDIQAVNVVINFDFPKNSETYLHRVGRSGRFGHLGLAVNLITYEDRFNLYRIEQELGTE

IKQIPPHIDQAIYCR

>GFP79518.1 dead-box ATP-dependent RNA helicase 8 [Phtheirospermum japonicum]

MNGNHARGRYPPGIGNGRGGGGALNQNYHNRNPHYMQQQPQYPQRVAQYPPPQWTRGNPNASAVSDSFNEVEKTVQSETI

GSTSQDWKSGLRVPPPDTRYRTEDVTATKGNEFEDYFLKRELLMGIYEKGFERPSPIQEESIPIALTGSDILARAKNGTG

KTAAFCIPALEKIDTDNNVIQAVILVPTRELALQTSQVCKELGKHLRIQVMVTTGGTSLRDDIMRLHQPVHLLVGTPGRV

LDLTKKGICNLQDCSVLIMDEADKLLSPEFQPSIEQLISFLHPNRQILMYSATFPVTVKYFKERHLRNPYVINLMDELTL

KGITQYYAFVEERQKVHCLNTLFSKLQINQSIIFCNSVNRVELLAKKITELGYSCFYIHAKMLQNHRNKVFHDFRNGACR

NLVCTDLFTRGIDIQAVNVVINFDFPKNSETYLHRVGRSGRFGHLGLAVNLITYEDRFNLYRIEQELGAEIKQIPPLIDQ

AIYCR

>XP_028239602.1 DEAD-box ATP-dependent RNA helicase 8-like isoform X2 [Glycine soja]

MNHNNSNNSNNNRARYPPGMGLGRGGFNPNLSQNPNQNQNHHAFQARPPYHQQQQAQYVQRHLLQQQQQQQWLRRDANAV

DEVEKTVQSEAVDSSSQDWKARLKIPPADTRYKTEDVTATKGNEFEDYFLKRELLMGIYEKGFERPSPIQEESIPIALTG

SDILARAKNGTGKTAAFCIPALEKIDQDNNVIQVVILVPTRELALQTSQVCKELGKHLKIQVMVTTGGTSLKDDILRLYQ

PVHLLVGTPGRILDLTKKGVCILKDCAMLVMDEADKLLSPEFQPSIEQLIHFLPTTRQILMFSATFPVTVKDFKDRYLQK

PYVINLMDELTLKGITQFYAFVEERQKVHCLNTLFSKLQINQSIIFCNSVNRVELLAKKITELGYSCFYIHAKMLQDHRN

RVFHDFRNGACRNLVCTDLFTRGIDIQAVNVVINFDFPKNAETYLHRVGRSGRFGHLGLAVNLITYEDRFNLYRIEQELG

TEIKQIPPQIDQAIYCR

>XP_012083429.1 DEAD-box ATP-dependent RNA helicase 8 [Jatropha curcas]

MNGNNRGRYPPGIGAGRGAGMNANPSFQSRVPQQQYVQRNMVQNHQQFQHQQHQHHQQQQQQQQQQQQQWLRRSQLPAAD

SSVDEVEKTVQSEAVDSSSQDWKARLKIPPADTRYKTEDVTATKGNEFEDYFLKRELLMGIYEKGFERPSPIQEESIPIA

LTGSDILARAKNGTGKTAAFCIPALEKIDQDNNVIQVVILVPTRELALQTSQVCKELGKHLKIQVMVTTGGTSLKDDIMR

LYQPVHLLVGTPGRILDLAKKGVCILKDCSMLVMDEADKLLSPEFQPSVEQLIRFLPPTRQILMFSATFPVTVKDFKDRY

LHKPYIINLMDELTLKGITQYYAFVEERQKVHCLNTLFSKLQINQSIIFCNSVNRVELLAKKITELGYSCFYIHAKMLQD

HRNRVFHDFRNGACRNLVCTDLFTRGIDIQAVNVVINFDFPKNSETYLHRVGRSGRFGHLGLAVNLITYEDRFNLYRIEQ

ELGTEIKQIPPHIDQAIYCR

>KAE9605419.1 putative RNA helicase [Lupinus albus]

MNNSNNNNNRARYPPGIGYGRGGSGGGGGGGFNPNHNQNAPFQPRPSYQQQQQQQQQQQHQQHYVQRNLVPQQHQQQQQQ

QQWLRRAQLGAGGSDSNVVYEVEKTVQTEANDSSSQDWKARLKAPPPDTRYKTEDVTATKGNEFEDYFLKRELLMGIYEK

GFERPSPIQEESIPIALTGSDILARAKNGTGKTAAFCIPALEKIDQDTNVIQAVILVPTRELALQTSQVCKELGKHLNIE

VMVTTGGTSLKDDIMRLYQPVHLLVGTPGRILDLAKKGVCILKDCTMLVMDEADKLLSPEFQPSIQQLIQFLPSNRQILM

FSATFPVTVKDFKDRYLQKPYVINLMDELTLKGITQFYAFVEERQKVHCLNTLFSKLQINQSIIFCNSVNRVELLAKKIT

ELGYSCFYIHAKMLQDHRNRVFHDFRNGACRNLVCTDLFTRGIDIQAVNVVINFDFPKNSETYLHRVGRSGRFGHLGLAV

NLITYEDRFNLYRIEQELGTEIKQIPPFIDQAVYCR

>XP_004133869.1 DEAD-box ATP-dependent RNA helicase 8 [Cucumis sativus]

MNNRGRYPPGIGAGRGGGVNANPSFQSRPHQQQYVQRNLVPNQQYQQQHQHQQLQQQQQWLKRNQLGGGPADSNVDEVEK

TVQSEAVDSSSQDWKARLKIPPPDTRYKTEDVTATKGNEFEDYFLKRELLMGIYEKGFERPSPIQEESIPIALTGSDILA

RAKNGTGKTAAFCIPALEKIDQDNNVIQVVILVPTRELALQTSQVCKELGKNLNIQVMVTTGGTSLKDDIMRLYQPVHLL

VGTPGRILDLAKKGVCVLKDCSMLIMDEADKLLSPEFQPSIEHLIRFLPTNRQILMYSATFPVTVKDFKDRYLHKPYVIN

LMDELTLKGITQFYAFVEERQKVHCLNTLFSKLQINQSIIFCNSVNRVELLAKKITELGYSCFYIHAKMLQDHRNRVFHD

FRNGACRNLVCTDLFTRGIDIQAVNVVINFDFPKNSETYLHRVGRSGRFGHLGLAVNLITYEDRFNLYRIEQELGTEIKQ

IPPHIDQAIYCR

>KAF0893695.1 hypothetical protein E2562_029375 [Oryza meyeriana var. granulata]

MDPRARYPPGIGNGRGGNPNYYGRGPTPPQQPHQHQHQQPLQPHHHQYVQRQPQPQQSQHNSQHQQWLRRNQIAAEAAGA

SEQKAPPVADGIDSSSQDWKAQLKLPTPDTRYRTEDVTATKGNEFEDYFLKRELLMGIYEKGFERPSPIQEESIPIALTG

SDILARAKNGTGKTAAFCIPALEKIDQDKNAIQVVILVPTRELALQTSQVCKELGKHLKIQVMVTTGGTSLKDDIVRLYQ

PVHLLVGTPGRILDLTKKGVCILKNCSMLVMDEADKLLSPEFQPSVEQLIRYLPSNRQILMFSATFPVTVKEFKDKYLPK

PYVINLMDELTLKGITQFYAFVEERQKVHCLNTLFSKLQINQSIIFCNSVNRVELLAKKITELGYSCFYIHAKMLQDHRN

RVFHDFRNGACRNLVCTDLFTRGIDIQAVNVVINFDFPKNAETYLHRVGRSGRFGHLGLAVNLITYEDRFNLYRIEQELG

TEIKSIPPQIDRAIYCQ

>XP_022035799.1 DEAD-box ATP-dependent RNA helicase 8 [Helianthus annuus]

MNSNYARGGGGGRYPPGIGRGGGGGGGGGGGGGGNYGNPNPNFQQQKNYQQQQYAQRNPVHQQQFQQQQQQQQWLRRNPV

GNDSNAVDEVEKTVQSDATDSSSQDWKAQLRLPPADTRYRTEDVTATKGNEFEDYFLKRELLMGIYEKGFERPSPIQEES

IPIALTGSDILARAKNGTGKTAAFCIPALEKIDQEKNVIQVVILVPTRELALQTSQVCKELGKHLNIQVMVTTGGTSLKD

DIMRLYQPVHLLVGTPGRILDLAKKGICALKDCAMLVMDEADKLLSPEFQPSVEQLIHCMPRNRQILMFSATFPVTVKDF

KDRYLQKPYVINLMDELTLKGITQFYAFVEERQKVHCLNTLFSKLQINQSIIFCNSVNRVELLAKKITELGYSCFYIHAK

MLQDHRNRVFHDFRNGACRNLVCTDLFTRGIDIQAVNVVINFDFPKNSETYLHRVGRSGRFGHLGLAVNLITYEDRFNLY

RIEQELGTEIKQIPPFIDQAIYCR

>XP_002511831.1 DEAD-box ATP-dependent RNA helicase 8 [Ricinus communis]

MNNNNNNNNNRGRYPPGIGAGRGAGMNANPSFQSRVPQQQYVQRNMMQNHHQQFQHQQHHQHHLQQQQQQQQQQQQWLRR

SQLPPADSSVDEVEKTVQSEAVDSTSQDWKARLKIPPPDTRYKTEDVTATKGNEFEDYFLKRELLMGIYEKGFERPSPIQ

EESIPIALTGSDILARAKNGTGKTAAFCIPALEKIDQDNNVIQVVILVPTRELALQTSQVCKELGKHLKIQVMVTTGGTS

LKDDIMRLYQPVHLLVGTPGRILDLAKKGVCVLKDCSMLVMDEADKLLSPEFQPSVEQLIRFLPPTRQILMFSATFPVTV

KDFKDRFLQKPYVINLMDELTLKGITQFYAFVEERQKVHCLNTLFSKLQINQSIIFCNSVNRVELLAKKITELGYSCFYI

HAKMLQDHRNRVFHDFRNGACRNLVCTDLFTRGIDIQAVNVVINFDFPKNSETYLHRVGRSGRFGHLGLAVNLITYEDRF

NLYRIEQELGTEIKQIPPHIDQAIYCR

>TKY63892.1 DEAD-box ATP-dependent RNA helicase 8 [Spatholobus suberectus]

MMNNNNNNNNRDRARYPPGMGLGRGGFNPNLNLNQNQSPNLIQNPNLNHAFQARPPYQQQPQYVQRHLVQPPPQQQQQWL

RRDANAVDEVEKTVQSEAVDSSSQDWKARLKIPPADTRYKTEDVTATKGNEFEDYFLKRELLMGIYEKGFERPSPIQEES

IPIALTGSDILARAKNGTGKTAAFCVPALEKIDQDNNVIQVVILVPTRELALQTSQVCKELGKHLKIQVMVTTGGTSLKD

DIMRLYQPVHLLVGTPGRIIDLAKKGVCILKDCSMLVMDEADKLLSPEFQPSIEQLIHFLPRNRQILMFSATFPVTVKDF

KDRYLQKPYVINLMDELTLKGITQYYAFVEERQKVHCLNTLFSKLQINQSIIFCNSVNRVELLAKKITELGYSCFYIHAK

MLQDHRNRVFHDFRNGACRNLVCTDLFTRGIDIQAVNVVINFDFPKNSETYLHRVGRSGRFGHLGLAVNLITYEDRFNLY

RIEQELGTEIKQIPPQIDQAIYCR

>KHG21931.1 DEAD-box ATP-dependent RNA helicase 8 [Gossypium arboreum]

MNSRGRYPPPGIGVGRGGGVNANPSFQSRPSQQHYVQRNLVHNQQHFQQHNQQHFQQQQQWLRRNHLPGGNDSSVIDEVE

KTVQSEAVDSSSQDWKARLKIPPADTRYKTEDVTATKGNEFEDYFLKRELLMGIYEKGFERPSPIQEESIPIALTGSDIL

ARAKNGTGKTAAFCIPALEKIDQDNNVIQVVILVPTRELALQTSQVCKELGKHLQIQVMVTTGGTSLKDDIMRLYQPVHL

LVGTPGRILDLAKKGVCILKDCSMLIMDEADKLLSPEFQPSVEQLIRFLPATRQILLFSATFPVTVKDFKDRYLQKPYII

NLMDELTLKGITQYYAFVEERQKVHCLNTLFSKLQINQSIIFCNSVNRVELLAKKITELGYSCFYIHAKMLQDHRNRVFH

DFRNGACRNLVCTDLFTRGIDIQAVNVVINFDFPKNSETYLHRVGRSGRFGHLGLAVNLITYEDRFNLYRIEQELGTEIK

QIPPHIDQAIYCRVNEACLFKAFVLAHGWSPEMLAGYNRPGRVLDESCF

>XP_022924437.1 DEAD-box ATP-dependent RNA helicase 8-like [Cucurbita moschata]

MNNRGRYPPGIGAGRGGGVNANPSFQSRPHQQQYVQRNLVPNQQYQQQHHQNQHQHQQLQQHQQWLKRNQLGGGPADSNV

DEVEKTVQSEAVDSSSQDWKARLKIPPPDTRYRTEDVTATKGNEFEDYFLKRELLMGIYEKGFERPSPIQEESIPIALTG

SDILARAKNGTGKTAAFCIPALEKIDQDNNVIQVVILVPTRELALQTSQVCKELGKNLNIQVMVTTGGTSLKDDIMRLYQ

PVHLLVGTPGRILDLAKKGVCVLKDCSMLIMDEADKLLSPEFQPSIEHLIRFLPSNRQILMYSATFPVTVKDFKDRYLHK

PYIINLMDELTLKGITQFYAFVEERQKVHCLNTLFSKLQINQSIIFCNSVNRVELLAKKITELGYSCFYIHAKMLQDHRN

RVFHDFRNGACRNLVCTDLFTRGIDIQAVNVVINFDFPKNSETYLHRVGRSGRFGHLGLAVNLITYEDRFNLYRIEQELG

TEIKQIPPHIDQAIYCQ

>XP_010469048.1 PREDICTED: DEAD-box ATP-dependent RNA helicase 12 [Camelina sativa]

MNNNNRGRYQPGVGTGRGAPPSPDYHQSYRQQQPQDQQQQYVQRGYPQNPQQMQLQHQQQQHQHQQQQWSRRPQIPGNAN

NANEVQKTSQSEATSEPNGQDWKATLRLPPPDTRYQTADVTATKGNEFEDYFLKRDLLKGIYEKGFEKPSPIQEESIPIA

LTGSDILARAKNGTGKTGAFCIPVLEKIDPNNNIIQAMILVPTRELALQTSQVCKELSKYLNIQVMVTTGGTSLRDDIMR

LHQPVHLLVGTPGRILDLTKKGVCVLKDCAMLVMDEADKLLSAEFQPSLEELIQFLPQNRQFLMFSATFPVTVKAFKDRH

LRKPYVINLMDQLTLVGVTQYYAFVEERQKVHCLNTLFSKLQINQSIIFCNSVNRVELLAKKITELGYSCFYIHAKMVQD

HRNRVFHEFRNGACRNLVCTDLFTRGIDIQAVNVVINFDFPRTSESYLHRVGRSGRFGHLGLAVNLVTYEDRFKMYQTEQ

ELGTEIKPIPSNIDQAIYCQ

>XP_010053686.1 PREDICTED: DEAD-box ATP-dependent RNA helicase 8 [Eucalyptus grandis]

MMNNRARYPPGIGASRGVNAGPAFQSRAPQPQYVQRGQLGQLPQYQQNHHQQQPQQHHQQHHQQNQQQHHQQQQQHQHQQ

HQQWLRRTQLGSADSTVDEVEKTVQSEAIDSSSQDWKARLKIPPPDTRYRTEDVTATKGNEFEDYFLKRELLMGIYEKGF

ERPSPIQEESIPIALTGSDILARAKNGTGKTAAFCVPALEKIDQDNNVIQVVILVPTRELALQTSQVCKELGKHLQIQVM

VTTGGTSLKDDIMRLYQPVHLLVGTPGRILDLAKKGVCILKDCSMLVMDEADKLLSPEFQPSVEQLIRFLPGNRQILMYS

ATFPVTVKDFKDRYLQKPYVINLMDELTLKGITQFYAFVEERQKVHCLNTLFSKLQINQSIIFCNSVNRVELLAKKITEL

GYSCFYIHAKMLQDHRNRVFHDFRNGACRNLVCTDLFTRGIDIQAVNVVINFDFPKNSETYLHRVGRSGRFGHLGLAVNL

ITYEDRFNLYRIEQELGTEIKQIPPHIDQAIYCR

>XP_006290970.1 DEAD-box ATP-dependent RNA helicase 12 [Capsella rubella]

MNNRGRYPPGVGTGRGAPPNPDYHQSYRQQQPQDQQYVQRGYPQNPQQMQLQQQQQHQQQQWSRRPQLPGNASNANELQK

TSQSEASSGDPNGQDWKATLRLPPPDTRYQTADVTATKGNEFEDYFLKRDLLKGIYEKGFEKPSPIQEESIPIALTGSDI

LARAKNGTGKTGAFCIPVLEKIDPNNNVIQAMILVPTRELALQTSQVCKELSKYLNIQVMVTTGGTSLRDDIMRLHQPVH

LLVGTPGRILDLTKKGVCVLKDCAMLVMDEADKLLSAEFQPSLEELIQFLPQNRQFLMFSATFPVTVKAFKDRHLRKPYV

INLMDQLTLMGVTQYYAFVEERQKVHCLNTLFSKLQINQSIIFCNSVNRVELLAKKITELGYSCFYIHAKMVQDHRNRVF

HEFRNGACRNLVCTDLFTRGIDIQAVNVVINFDFPRTSESYLHRVGRSGRFGHLGLAVNLVTYEDRFKMYQTEQELGTEI

KPIPSNIDQAIYCQ

>XP_020218839.1 DEAD-box ATP-dependent RNA helicase 8 [Cajanus cajan]

MNNRENNRERYPPGMGLGRGLNPNHGFQPRGHHHQYVQRHMVQHHPQQYQQNHHQQHHHQQHHQQHHHQQQQHHHHQQQQ

QQQWLRRNQLGGGTDTNVVEEVEKTVQSEAVDPSSQDWKARLKIPPPDTRYKTEDVTATKGNEFEDYFLKRELLMGIYEK

GFERPSPIQEESIPIALTGSDILARAKNGTGKTAAFCIPALEKIDQDNNVIQVVILVPTRELALQTSQVCKELGKHLQIQ

VMVTTGGTSLKDDIMRLYQPVHLLVGTPGRILDLAKKGVCILKDCSMLVMDEADKLLSPEFQPSIEQLIQFLPGNRQILM

FSATFPVTVKDFRDRYLRKPYVINLMDELTLKGITQYYAFVEERQKVHCLNTLFSKLQINQSIIFCNSVNRVELLAKKIT

ELGYSCFYIHAKMLQDHRNRVFHDFRNGACRNLVCTDLFTRGIDIQAVNVVINFDFPKNSETYLHRVGRSGRFGHLGLAV

NLITYEDRFNLYRIEQELGTEIKQIPPHIDQAIYCR

>XP_016748210.1 PREDICTED: DEAD-box ATP-dependent RNA helicase 8-like [Gossypium hirsutum]

MNSRGRYPPGIGVGRGGGVNANPSFQSRPSQQHYVQRNLLQNHQHFQQQQQQHQQQQQHQQQLWLRRDQLPGGNDSSVVD

EVEKTVQSEAVDSSSQDWKARLNVPPPDTRYKTEDVTATKGNEFEDYFLKRELLMGIYEKGFERPSPIQEESIPIALTGS

DILARAKNGTGKTAAFCIPALEKIDQDNNVIQVVILVPTRELALQTSQVCKELGKHLQIQVMVTTGGTSLKDDIMRLYQP

VHLLVGTPGRILDLAKKGVCILKDCSMLIMDEADKLLSPEFQPSIEQLIRFLPANRQILMFSATFPVTVKDFKDKYLKKP

YIINLMDELTLKGITQYYAFVEERQKVHCLNTLFSKLQINQSIIFCNSVNRVELLAKKITELGYSCFYIHAKMLQDHRNR

VFHDFRNGACRNLVCTDLFTRGIDIQAVNVVINFDFPKNSETYLHRVGRSGRFGHLGLAVNLITYEDRFNLYRIEQELGT

EIKQIPPHIDQAIYCR

>XP_028949075.1 DEAD-box ATP-dependent RNA helicase 8-like [Malus domestica]

MNSNRGRYPPGIGAGRGGGMNANPAFQSRPPHQQQYVQRNLLPNHHHQQQYFQQQQQQQQHQQHQQQQLQQQQQWLRRGQ

LGGSTSADSAVDEVEKTVQSEAVDPSSQDWKARLKIPPADTRFRTEDVTATKGNEFEDYFLKRELLMGIYEKGFERPSPI

QEESIPIALTGSDILARAKNGTGKTAAFCIPALEKIDQDNNVIQVVILVPTRELALQTSQVCKELGKHLQIQVMVTTGGT

SLKDDIMRLYQPVHLLVGTPGRILDLAKKGVCILKDCSMLVMDEADKLMSPEFQPSVEQLIRFLPSHRQILMFSATFPVT

VKDFKDRYLQKPYVINLMDELTLKGITQFYAFVEERQKVHCLNTLFSKLQINQSIIFCNSVNRVELLAKKITELGYSCFY

IHAKMLQDHRNRVFHDFRNGACRNLVCTDLFTRGIDIQAVNVVINFDFPKNSETYLHRVGRSGRFGHLGLAVNLITYEDR

FNLYRIEQELGTEIKQIPPHIDQAIYCR

>XP_025984956.1 DEAD-box ATP-dependent RNA helicase 8 isoform X2 [Glycine max]

MCCCSLSFYVLLLVLFPELSQDWKARLKIPPADTRYKTEDVTATKGNEFEDYFLKRELLMGIYEKGFERPSPIQEESIPI

ALTGSDILARAKNGTGKTAAFCIPALEKIDQDNNVIQVVILVPTRELALQTSQVCKELGKHLKIQVMVTTGGTSLKDDIL

RLYQPVHLLVGTPGRILDLTKKGVCILKDCAMLVMDEADKLLSPEFQPSIEQLIHFLPTTRQILMFSATFPVTVKDFKDR

YLQKPYVINLMDELTLKGITQFYAFVEERQKVHCLNTLFSKLQINQSIIFCNSVNRVELLAKKITELGYSCFYIHAKMLQ

DHRNRVFHDFRNGACRNLVCTDLFTRGIDIQAVNVVINFDFPKNAETYLHRVGRSGRFGHLGLAVNLITYEDRFNLYRIE

QELGTEIKQIPPQIDQAIYCR

>CBI40324.3 unnamed protein product, partial [Vitis vinifera]

MGTGNPSGPNPNFQYRNPNQQQYVQRNVGQSYQQFQQQNQQQWLRRNQAGADSAVDEVEKTVQSEAVDSSSQDWKARLKI

PPPDTRYRTEDVTATKGNEFEDYFLKRELLMGIYEKGFERPSPIQEESIPIALTGSDILARAKNGTGKTAAFCIPALEKI

DQDNNVIQVVILVPTRELALQTSQVCKELGKHLKIQVMVTTGGTSLKDDIMRLYQPVHLLVGTPGRILDLSKKGVCILKD

CSMLVMDEADKLLSPEFQPSIEQLIHFMPLNRQILLFSATFPVTVKDFKDRYLQKPYIINLMDELTLKGITQYYAFVEER

QKVHCLNTLFSKLQINQSIIFCNSVNRVELLAKKITELGYSCFYIHAKMLQDHRNRVFHDFRNGACRNLVCTDLFTRGID

IQAVNVVINFDFPKNSETYLHRVGRSGRFGHLGLAVNLITYEDRFNLYRIEQELGTEIKQIPPHIDQAIYCR

>XP_007135054.1 hypothetical protein PHAVU_010G0977000g, partial [Phaseolus vulgaris]

SQDWKARLKIPPADTRYKTEDVTATKGNEFEDYFLKRELLMGIYEKGFERPSPIQEESIPIALTGSDILARAKNGTGKTA

AFCIPALEKIDQDNNVIQVVILVPTRELALQTSQVCKELGKHLQIQVMVTTGGTSLKDDIMRLYQPVHLLVGTPGRILDL

AKKGVCIMKDCSMLVMDEADKLLSPEFQPSIEQLIHFLPTNRQILMFSATFPVTVKDFKDRYLHKPYVINLMDELTLKGI

TQFYAFVEERQKVHCLNTLFSKLQINQSIIFCNSVNRVELLAKKITELGYSCFYIHAKMLQDHRNRVFHDFRNGACRNLV

CTDLFTRGIDIQAVNVVINFDFPKNSETYLHRVGRSGRFGHLGLAVNLITYEDRFNLYRIEQELGTEIKQIPPQIDQAIY

CR

>XP_024994568.1 DEAD-box ATP-dependent RNA helicase 8-like [Cynara cardunculus var. scolymus]

MNNRRYPPGIGNGRGSGGGYDGGGGGNFHPNPNYYHQSRNPNHQHQFQQQRQQQQQQQPQYVQRQQAGQQQNHHQQQWLR

RSPNASGSSATNVTDEVEKSVHSDGSVDTSSQDWKAQLNVPAADTRYRTEDVTATKGNEFEDYFLKRELLMGIYEKGFER

PSPIQEESIPIALTGSDILARAKNGTGKTAAFCIPALEKIDTDNNSIQVVILVPTRELALQTSQVCKELGKHLQIQVMVT

TGGTSLKDDIMRLYQPVHLLVGTPGRILDLTKKGICNLTDCAMLVMDEADKLLSPEFQPSVEQLIQFLPEQRQVLMFSAT

FPVTVKDFKDRYLKKPYVVNLMDELTLKGITQFYAFVEERQKVHCLNTLFSKLQINQSIIFCNSVNRVELLAKKITELGY

SCFYIHAKMLQDHRNRVFHDFRNGACRNLVCTDLFTRGIDIQAVNVVINFDFPRNAETYLHRVGRSGRFGHLGLAVNLIT

YEDRFNLYRIEQELGTEIKQIPPLIDQAIYCR

>MBA0854312.1 hypothetical protein [Gossypium schwendimanii]

MNNRGRYPPVLGRGRGANANPSFQSRPEQPQYAQRNLVQNHHHFQQQQHHHHHHHLQQQQHQQQWLRRNQLPGGNDSTVA

DEVEKTVQSEAVDSSSQDWKARLKMPPSDTRYKTEDVTATKGNEFEDYFLKRELLMGIYEKGFERPSPIQEESIPIALTG

SDILARAKNGTGKTAAFCIPALEKIDQDNNVIQVVILVPTRELALQTSQVCKELGKHLPIQVMVTTGGTSLKDDIMRLYQ

PVHLLVGTPGRILDLAKKGVCILKNCSMLIMDEADKLLSPEFQPSLEQLLCFLPPNRQILMFSATFPVTVKDFKDRYLKK

PYIINLMDELTLKGITQYYAFVEERQKVHCLNTLFSKLQINQSIIFCNSVNRVELLAKKITELGYSCFYIHAKMLQDHRN

RVFHDFRNGACRNLVCTDLFTRGIDIQAVNVVINFDFPKNSETYLHRVGRSGRFGHLGLAVNLITYEDRFNLYRIEQELG

TEIKQIPPHIDQAIYCR

>KAA0048977.1 Dead box ATP-dependent RNA helicase [Cucumis melo var. makuwa]

MNNRGRYPPGIGAGRGGGVNANPSFQSRPHQQQYVQRNLVPNQQYQQQHQHQQLQQQQQWLKRNQLGGGPADSNVDEVEK

TVQSEAVDSSSQDWKARLKIPPPDTRYKTEDVTATKGNEFEDYFLKRELLMGIYEKGFERPSPIQEESIPIALTGSDILA

RAKNGTGKTAAFCIPALEKIDQDNNVIQVVILVPTRELALQTSQVCKELGKNLNIQVMVTTGGTSLKDDIMRLYQPVHLL

VGTPGRILDLAKKGVCVLKDCSMLIMDEADKLLSPEFQPSIEHLIRFLPANRQILMYSATFPVTVKDFKDRYLHKPYVIN

LMDELTLKGITQFYAFVEERQKVHCLNTLFSKLQINQSIIFCNSVNRVELLAKKITELGYSCFYIHAKMLQDHRNRVFHD

FRNGACRNLVCTDLFTRGIDIQAVNVVINFDFPKNSETYLHRVGRSGRFGHLGLAVNLITYEDRFNLYRIEQELGTEIKQ

IPPHIDQAIYCRAMKLLGRTKDIWVRQEACFLSEYDGRKTQVVQSVCVGNSTISLESNRPEDINRKKIRIECRLVPSIKN

ENPRRDG

>RXI08030.1 hypothetical protein DVH24_014596 [Malus domestica]

MNSNRGRYPPGIGAGRGGGMNANPAFQSRPPHQQQYVQRNLLPNHHHQQQYFQQQQQQQQHQQHQQQQLQQQQQWLRRGQ

LGGSTSADSAVDEVEKTVQSEAVDPRMGRDKSLMKHLPHVQFSMDLYSPVGPPSIVCPRWGLERLHVSMGINRIEDPYTS

ELTEKEISKGHRHWSSSFLDPSIKISQDWKARLKIPPADTRFRTEDVTATKGNEFEDYFLKRELLMGIYEKGFERPSPIQ

EESIPIALTGSDILARAKNGTGKTAAFCIPALEKIDQDNNVIQVVILVPTRELALQTSQVCKELGKHLQIQVMVTTGGTS

LKDDIMRLYQPVHLLVGTPGRILDLAKKGVCILKDCSMLVMDEADKLMSPEFQPSVEQLIRFLPSHRQILMFSATFPVTV

KDFKDRYLQKPYVINLMDELTLKGITQFYAFVEERQKVHCLNTLFSKLQINQSIIFCNSVNRVELLAKKITELGYSCFYI

HAKMLQDHRNRVFHDFRNGACRNLVCTDLFTRGIDIQAVNVVINFDFPKNSETYLHRVGRSGRFGHLGLAVNLITYEDRF

NLYRIEQELGTEIKQIPPHIDQAIYCR

>TYG93310.1 hypothetical protein ES288_A11G099800v1 [Gossypium darwinii]

MNSRGRYPPGIGVGRGGGVNANPSFQSRPSQQHYVQRNLLQNHQHFQQQQHQQQQQHQQQLWLRRDQLPGGNDSSVVDEV

EKTVQSEAVDSSSQDWKARLNVPPPDTRYKTEDVTATKGNEFEDYFLKRELLMGIYEKGFERPSPIQEESIPIALTGSDI

LARAKNGTGKTAAFCIPALEKIDQDNNVIQVVILVPTRELALQTSQVCKELGKHLQIQVMVTTGGTSLKDDIMRLYQPVH

LLVGTPGRILDLAKKGVCILKDCSMLIMDEADKLLSPEFQPSIEQLIRFLPANRQILMFSATFPVTVKDFKDKYLKKPYI

INLMDELTLKGITQYYAFVEERQKVHCLNTLFSKLQINQSIIFCNSVNRVELLAKKITELGYSCFYIHAKMLQDHRNRVF

HDFRNGACRNLVCTDLFTRGIDIQAVNVVINFDFPKNSETYLHRVGRSGRFGHLGLAVNLITYEDRFNLYRIEQELGTEI

KQIPPHIDQAIYCR

>XP_003528862.1 DEAD-box ATP-dependent RNA helicase 8 isoform X1 [Glycine max]

MNHNNSNNNRARYPPGMGLGRGGFNPNLSQNPNQNQNHHAFQARPPYHQQQQAQYVQRHLLQQQQQQQQQQQQQQQQWLR

RDANAVDEVEKTVQSEAVDSSSQDWKARLKIPPADTRYKTEDVTATKGNEFEDYFLKRELLMGIYEKGFERPSPIQEESI

PIALTGSDILARAKNGTGKTAAFCIPALEKIDQDNNVIQVVILVPTRELALQTSQVCKELGKHLKIQVMVTTGGTSLKDD

ILRLYQPVHLLVGTPGRILDLTKKGVCILKDCAMLVMDEADKLLSPEFQPSIEQLIHFLPTTRQILMFSATFPVTVKDFK

DRYLQKPYVINLMDELTLKGITQFYAFVEERQKVHCLNTLFSKLQINQSIIFCNSVNRVELLAKKITELGYSCFYIHAKM

LQDHRNRVFHDFRNGACRNLVCTDLFTRGIDIQAVNVVINFDFPKNAETYLHRVGRSGRFGHLGLAVNLITYEDRFNLYR

IEQELGTEIKQIPPQIDQAIYCR

>TYH99931.1 hypothetical protein ES332_A11G099100v1 [Gossypium tomentosum]

MNSRGRYPPGIGVGRGGGVNANPSFQSRPSQQHYVQRNLLQNHQHFQQQQQHHQQQQQHQQQLWLRRDQLPGGNDSSVVD

EVEKTVQSEAVDSSSQDWKARLNVPPPDTRYKTEDVTATKGNEFEDYFLKRELLMGIYEKGFERPSPIQEESIPIALTGS

DILARAKNGTGKTAAFCIPALEKIDQDNNVIQVVILVPTRELALQTSQVCKELGKHLQIQVMVTTGGTSLKDDIMRLYQP

VHLLVGTPGRILDLAKKGVCILKDCSMLIMDEADKLLSPEFQPSIEQLIRFLPANRQILMFSATFPVTVKDFKDKYLKKP

YIINLMDELTLKGITQYYAFVEERQKVHCLNTLFSKLQINQSIIFCNSVNRVELLAKKITELGYSCFYIHAKMLQDHRNR

VFHDFRNGACRNLVCTDLFTRGIDIQAVNVVINFDFPKNSETYLHRVGRSGRFGHLGLAVNLITYEDRFNLYRIEQELGT

EIKQIPPHIDQAIYCR

>XP_002270379.1 PREDICTED: DEAD-box ATP-dependent RNA helicase 8 [Vitis vinifera]

MHARGRYPPGIHGGGRGGGMGTGNPSGPNPNFQYRNPNQQQYVQRNVGQSYQQFQQQNQQQWLRRNQAGADSAVDEVEKT

VQSEAVDSSSQDWKARLKIPPPDTRYRTEDVTATKGNEFEDYFLKRELLMGIYEKGFERPSPIQEESIPIALTGSDILAR

AKNGTGKTAAFCIPALEKIDQDNNVIQVVILVPTRELALQTSQVCKELGKHLKIQVMVTTGGTSLKDDIMRLYQPVHLLV

GTPGRILDLSKKGVCILKDCSMLVMDEADKLLSPEFQPSIEQLIHFMPLNRQILLFSATFPVTVKDFKDRYLQKPYIINL

MDELTLKGITQYYAFVEERQKVHCLNTLFSKLQINQSIIFCNSVNRVELLAKKITELGYSCFYIHAKMLQDHRNRVFHDF

RNGACRNLVCTDLFTRGIDIQAVNVVINFDFPKNSETYLHRVGRSGRFGHLGLAVNLITYEDRFNLYRIEQELGTEIKQI

PPHIDQAIYCR

>XP_010413440.1 PREDICTED: DEAD-box ATP-dependent RNA helicase 12 isoform X2 [Camelina sativa]

MNNNNRGRYPPEVGTGRGAPPNPDYHQSYRQQQPQDQQHQYVQRGYPQMQLQQQQQQQQHQQQQWSRRPQIPGNANNANE

VQKTSQSEATSEPNGQDWKATLRLPPPDTRYQTADVTATKGNEFEDYFLKRDLLKGIYEKGFEKPSPIQEESIPIALTGS

DILARAKNGTGKTGAFCIPVLEKIDPNNNIIQAMILVPTRELALQTSQVCKELSKYLNIQVMVTTGGTSLRDDIMRLHQP

VHLLVGTPGRILDLTKKGVCVLKDCAMLVMDEADKLLSAEFQPSLEELIQFLPQNRQFLMFSATFPVTVKAFKDRHLRKP

YVINLMDQLTLVGVTQYYAFVEERQKVHCLNTLFSKLQINQSIIFCNSVNRVELLAKKITELGYSCFYIHAKMVQDHRNR

VFHEFRNGACRNLVCTDLFTRGIDIQAVNVVINFDFPRTSESYLHRVGRSGRFGHLGLAVNLVTYEDRFKMYQTEQELGT

EIKPIPSNIDQAIYCQ

>RWW08282.1 hypothetical protein GW17_00028282 [Ensete ventricosum]

MNPRGRYPPPGMGNGRGNSHHHHHHQQQQQQQQYVQRNPVQVQPNQHFQQQQQWSIRNQLGGDSGSGDVVKSVQPESSHT

SGFSSQDWKAQLRMPPPDTRYKTEDVTATKGNEFEDYFLKRELLMGIYEKGFEKPSPIQEESIPIALTGSDILARAKNGT

GKTAAFCIPALEKIDQDHNAIQVVILVPTRELALQTSQVCKELGKHLKIQVMVTTGGTSLKDDIMRLYQPVHLLVGTPGR

ILDLAKKGVCVLKDCSMLVMDEADKLLSPEFQPSIEQLIQFLPANRQILMFSATFPVTVKDFKDRYLPKPYIINLMDELT

LKGITQYYAFVEERQKVHCLNTLFSKLQINQSIIFCNSVNRVELLAKKITELGYSCFYIHAKMLQDHRNRVFHDFRNGAC

RNLVCTDLFTRGIDIQAVNVVINFDFPKNSETYLHRVGRSGRFGHLGLAVNLITYEDRFNLYRIEQELGTEIKQIPPQID

QAIYCR

>XP_028239601.1 DEAD-box ATP-dependent RNA helicase 8-like isoform X1 [Glycine soja]

MNHNNSNNSNNNRARYPPGMGLGRGGFNPNLSQNPNQNQNHHAFQARPPYHQQQQAQYVQRHLLQQQQQQQWLRRDANAV

DEVEKTVQSEAVDSRSKRSHCLSSLIVRSCCGAAGFLSMCCCSLSFYVLLLVLFPELSQDWKARLKIPPADTRYKTEDVT

ATKGNEFEDYFLKRELLMGIYEKGFERPSPIQEESIPIALTGSDILARAKNGTGKTAAFCIPALEKIDQDNNVIQVVILV

PTRELALQTSQVCKELGKHLKIQVMVTTGGTSLKDDILRLYQPVHLLVGTPGRILDLTKKGVCILKDCAMLVMDEADKLL

SPEFQPSIEQLIHFLPTTRQILMFSATFPVTVKDFKDRYLQKPYVINLMDELTLKGITQFYAFVEERQKVHCLNTLFSKL

QINQSIIFCNSVNRVELLAKKITELGYSCFYIHAKMLQDHRNRVFHDFRNGACRNLVCTDLFTRGIDIQAVNVVINFDFP

KNAETYLHRVGRSGRFGHLGLAVNLITYEDRFNLYRIEQELGTEIKQIPPQIDQAIYCR

>XP_017980159.1 PREDICTED: DEAD-box ATP-dependent RNA helicase 8 [Theobroma cacao]

MNSRGRYPPGIGVGRGGGVNANPSFQSRPLQQHYVQRNLVQNHQQFQHHQQQQHHQQQQHQQHQQQQQWLRRNQLPGGND

SSVVDEVEKTVQSEAVDSSSQDWKARLKIPPPDTRYKTEDVTATKGNEFEDYFLKRELLMGIYEKGFERPSPIQEESIPI

ALTGSDILARAKNGTGKTAAFCIPALEKIDQDNNVIQVVILVPTRELALQTSQVCKELGKHLQIQVMVTTGGTSLKDDIM

RLYQPVHLLVGTPGRILDLAKKGVCILKDCSMLIMDEADKLLSPEFQPSVEQLIRFLPANRQILMFSATFPVTVKDFKDR

YLHKPYIINLMDELTLKGITQYYAFVEERQKVHCLNTLFSKLQINQSIIFCNSVNRVELLAKKITELGYSCFYIHAKMLQ

DHRNRVFHDFRNGACRNLVCTDLFTRGIDIQAVNVVINFDFPKNSETYLHRVGRSGRFGHLGLAVNLITYEDRFNLYRIE

QELGTEIKQIPPHIDQAIYCR

>XP_013589399.1 PREDICTED: DEAD-box ATP-dependent RNA helicase 12-like [Brassica oleracea var. oleracea]

MERDVELRRISIISRTLPGNANEVQNTTSSQPPVPSNDHNGSVTLSGGQDWKATLRLPPPHTRYQTADVTATKGNEFEDY

FLKRDLLKGIYEKGFEKPSPIQEESIPIALTGSDILARAKNGTGKTGAFCIPVLEKIDPNTNVIQAMILVPTRELALQTS

QVCKELSKYLNIQVMVTTGGTSLRDDIMRLHQPVHLLVGTPGRILDLTKMGVCVLKDCTMLVMDEADKLLSAEFQPSLEE

LIQFLPENRQFLMFSATFPVTVKAFKDRHLRKPYVINLMDQLTLMGVTQYYAFVEERQKVHCLNTLFSKLQINQSIIFCN

SANRVELLAKKITELGYSCFYIHAKMVQDHRNRVFHEFRNGACRNLVCTDLFTRGIDIQAVNVVINFDFPRTSESYLHRV

GRSGRFRHLGLAVNLVTYEDRFKMYQTEQELGTEIKPIPSHIDQAIYCQ

>TEY47128.1 ATP-dependent RNA helicase DDX6/DHH1 [Salvia splendens]

MSSRARYPPPGMGGGRGGSGGGGMNQYGGGGMNHYGGANPNFQPRFYNQQYGQRSQTQNFQNNQSMQNTQPQQWLRRTPP

ADSSADEVEKTVQSEAIDSRKLGILHTGFFFLISSQDWKERLKLPPPDTRYRTEDVTATKGNEFEDYFLKRELLMGIYEK

GFERPSPIQEESIPIVLTGSDILARAKNGTGKTAAFCIPALEKIDQDKNYIQAVILVPTRELALQTSQVCKELGKHLQIE

VMATTGGTSLKDDIMRLYQPVHLLVGTPGRILDLSRKGVCHLDECNMLIMDEADKLLSPEFQPSIEELISFLPTNRQILM

FSATFPVTVKNFKDRYLRRPYIINLMDELTLKGITQFYAFVEERQKVHCLNTLFSKLQINQSIIFCNSVNRVELLAKKIT

ELGYSCFYIHAKMLQDHRNRVFHDFRNGACRNLVCTDLFTRGIDIQAVNVVINFDFPKNSETYLHRVGRSGRFGHLGLAV

NLITYEDRFNLYRIEQELGTEIKQIPPHIDQGIYCL

>KAB2070207.1 hypothetical protein ES319_A08G141100v1 [Gossypium barbadense]

MNNRGSYPPVLGRGRGANANPSFQSRPEQPQYAQRNLVQNHHHFQQQQHHHLQQQQHQHQQQWLRRNQLPGGNDSTVADE

VEKTVQSEAVDSSSQDWKARLKLPPSDTRYKTEDVTATKGNEFEDYFLKRELLMGIYEKGFERPSPIQEESIPIALTGSD

ILARAKNGTGKTAAFCIPALEKIDQDNNVIQVVILLPTRELALQTSQVCKELGKHLQIQVMVTTGGTSLKDDIMRLYQPV

HLLVGTPGRILDLAKKGVCILKNCSMLIMDEADKLLSPEFQPSLEQLLRFLPPKRQILMFSATFPVTVKDFKDRYLKKPY

IINLMDELTLKGITQYYAFVEERQKVHCLNTLFSKLQINQSIIFCNSVNRVELLAKKITELGYSCFYIHAKMLQDHRNRV

FHDFRNGACRNLVCTDLFTRGIDIQAVNVVINFDFPKNSETYLHRVGRSGRFGHLGLAVNLITYEDRFNLYRIEQELGTE

IKQIPPHIDQAIYCR

>TKY65476.1 DEAD-box ATP-dependent RNA helicase 8 [Spatholobus suberectus]

MNNRERMNNRERYPPGMGFQPRPHQHQYVQRHMVHQYQQNHHHHHHHHQQQQQQQQHQQQQQWLRRNQLGGGTDTSVVVE

EVEKTVQSEAVDSSSQDWKARLKIPPADTRYKTEDVTATKGNEFEDYFLKRELLMGIYEKGFERPSPIQEESIPIALTGS

DILARAKNGTGKTAAFCIPALEKIDQDSNVIQVVILVPTRELALQTSQVCKELGKHLKIQVMVTTGGTSLKDDIMRLYQP

VHLLVGTPGRILDLAKKGVCILKDCSMLVMDEADKLLSPEFQPSIEQLIQFLPANRQILMFSATFPVTVKDFKDRYLHKP

YIINLMDELTLKGITQFYAFVEERQKVHCLNTLFSKLQINQSIIFCNSVNRVELLAKKITELGYSCFYIHAKMLQDHRNR

VFHDFRNGACRNLVCTDLFTRGIDIQAVNVVINFDFPKNSETYLHRVGRSGRFGHLGLAVNLITYEDRFNLYRIEQELGT

EIKQIPPHIDQAIYCR

>PWA65297.1 DEAD-box ATP-dependent RNA helicase 8 [Artemisia annua]

MSNNNYARGGGGGGRYPPGIGRGGGNGGGGGYYGNPNPNYQQQYVQRNVGGSNQQWLRRNPAVDEVEKTVQSEAIDASSQ

DWKAQLRLPPADTRYRTEDVTATKGNEFEDYFLKRELLMGIYEKGFEKPSPIQEESIPIALTGSDILARAKNGTGKTAAF

CIPALEKIDHDKNVIQVVILVPTRELALQTSQVCKELGKHLKTQVMVTTGGTSLKDDIMRLYQPVHLLVGTPGRILDLAK

KGICKLDDCAMLAMDEADKLLSPEFQPSVEELIHFMPTNRQILMFSATFPVTVKDFKDRFLKKPYVINLMDELTLKGITQ

FYAFVEERQKVHCLNTLFSKLQINQSIIFCNSVNRVELLAKKITELGYSCFYIHAKMLQDHRNRVFHDFRNGACRNLVCT

DLFTRGIDIQAVNVVINFDFPKNSETYLHRVGRSGRFGHLGLAVNLITYEDRFNLYRIEQELGTEIKQIPPFIDQAIYCR

>XP_008438061.1 PREDICTED: DEAD-box ATP-dependent RNA helicase 8 [Cucumis melo]

MNNRGRYPPGIGAGRGGGVNANPSFQSRPHQQQYVQRNLVPNQQYQQQHQHQQLQQQQQWLKRNQLGGGPADSNVDEVEK

TVQSEAVDSSSQDWKARLKIPPPDTRYKTEDVTATKGNEFEDYFLKRELLMGIYEKGFERPSPIQEESIPIALTGSDILA

RAKNGTGKTAAFCIPALEKIDQDNNVIQVVILVPTRELALQTSQVCKELGKNLNIQVMVTTGGTSLKDDIMRLYQPVHLL

VGTPGRILDLAKKGVCVLKDCSMLIMDEADKLLSPEFQPSIEHLIRFLPANRQILMYSATFPVTVKDFKDRYLHKPYVIN

LMDELTLKGITQFYAFVEERQKVHCLNTLFSKLQINQSIIFCNSVNRVELLAKKITELGYSCFYIHAKMLQDHRNRVFHD

FRNGACRNLVCTDLFTRGIDIQAVNVVINFDFPKNSETYLHRVGRSGRFGHLGLAVNLITYEDRFNLYRIEQELGTEIKQ

IPPHIDQAIYCR

>THU65630.1 hypothetical protein C4D60_Mb05t05650 [Musa balbisiana]

MNPRGRFPPPGMGNGRGGGASTNPGFYARNPQQHQHQQQYVQRNPVNGQPNRQFQHQHQQWSRRNWVGGDSGAGEVMKSV

QSEATDSSLQDWKAQLKIPPADSRYKTEDVTATKGNEFEDYFLKRELLMGIYEKGFERPSPIQEESIPIALTGSDILARA

KNGTGKTAAFCIPALEKIDQDCNAIQVVILVPTRELALQTSQVCKELGKHLKIQVMVSTGGTSLKDDIMRLYQPVHLLVG

TPGRILDLAKKGVCVLKDCSMLIMDEADKLLSPEFQPTIEQLIQFLPANRQILMFSATFPVTVKDFKDKYLPKPYIINLM

DELTLKGITQYYAFVEERQKVHCLNTLFSKLQINQSIIFCNSVNRVELLAKKITELGYSCFYIHAKMLQDHRNRVFHDFR

NGACRNLVCTDLFTRGIDIQAVNVVINFDFPKNSETYLHRVGRSGRFGHLGLAINLITYEDRFNLYRIEQELGAEIKQIP

PQIDRTIYCT

>KAD3641296.1 hypothetical protein E3N88_30520 [Mikania micrantha]

MSSNNNTNRRYPPGIGNGRGNGGGYDGGGGGNFQPNPNYYHQPRNPNHQNQFHQQRQQPQYAQKQQSGPQHNQQQQWLRR

NPNPPGSSAAIVNNEVEKSVYSDGSVDSSSQDWKEKLNIPAADTRFRTEDVTATKGNEFEDYFLKRELLMGIYEKGFERP

SPIQEESIPIALTGSDILARAKNGTGKTAAFCIPALEKIDTDKNKIQVIILVPTRELALQTSQVCKELGKHLKIQVMVTT

GGTSLKDDIMRLYQPVHLLVGTPGRILDLTKKGICKLSDCAMLIMDEADKLLSPEFQPSVEELIGFLPEQRQLLMFSATF

PVTVKDFKDRYLKKPYIVNLMDELTLKGITQFYAFVEERQKVHCLNTLFSKLQINQSIIFCNSVNRVELLAKKITELGYS

CFYIHAKMLQDHRNRVFHDFRNGACRNLVCTDLFTRGIDIQAVNVVINFDFPKNAETYLHRVGRSGRFGHLGLAVNLITY

EDRFNLYRIEQELGTEIKQIPPQIDQAIYCR

>XP_017699082.1 DEAD-box ATP-dependent RNA helicase 8-like [Phoenix dactylifera]

MNYRGGRYPPGIGNGRGGNVGGNPNIYARNPQQQQQYVQRNPVQGQHNQQQQWLRRHQLGNDSGTSEVMKSVQSASIDSS

SQDWKAQLNVPPPDMRYKTEDVTATKGNEFEDYFLKRELLMGIYEKGFERPSPIQEESIPIALTGSDILARAKNGTGKTA

AFCIPALEKIDQDNNVIQVAILVPTRELALQTSQVCKELGKHLRIQVMVTTGGTSLKDDIMRLYQPVHLLVATPGRVLDL

AKKGVCILKDCSMLIMDEADKLLSPEFQPSVEQLIRFLPANRQILLFSATFPVTVKDFKDRYLAKPYVINLMDELTLKGI

TQYYAFVEERQKVHCLNTLFSKLQINQSIIFCNSVNRVELLAKKITELGYSCFYIHAKMLQDHRNRVFHDFRNGACRNLV

CTDLFTRGIDIQAVNVVINFDFPKNAETYLHRVGRSGRYGHLGLAVNLITYEDRFNLYRIEQELGTEIKQIPPQIDQAVY

CR

>XP_022862472.1 DEAD-box ATP-dependent RNA helicase 8-like [Olea europaea var. sylvestris]

MSSRARYPPPGMEGGRGSYGGINPNPFHPRNPTQNDMTRGPAPGNMNHQTFHNPQQQQWLRRTQFPPPDLTVHEVEKVIQ

SEAVDSSLQDWKARLKLPPTDTRYKTEDVTATKGNEFEDYFLKRELLMGIYEKGFERPSPIQEESIPIALTGSDILARAK

NGTGKTAAFCIPALEKIDQDNNVIQVVILVPTRELALQTSQVCKELGKHLKIQVMVTTGGTSLKDDIMRLHQPVHLLVGT

PGRILDLTKKGVCVLKDCSMLVMDEADKLLSPEFQPSVEQLIHFMPSKRQILMFSATFPVTVKDFKDRYLQKPYTINLMD

ELTLKGITQYYAFVEERQKVHCLNTLFSKLQINQSIIFCNSVNRVELLAKKITEIGYSCFYIHAKMLQNHRNRVFHDFRN

GACRNLVCTDLFTRGIDIQAVNVVINFDFPKNSETYLHRVGRSGRFGHLGLAVNLITYEDRFNLYRIEQELGTEIKQIPP

QIDQAIYCR

>MBC9844858.1 hypothetical protein [Adiantum capillus-veneris]

MSTNRNYQYVRRQPTVGAPTNGQSSSRINTPVSSSLATFGNSYDWKSQIKRPPADTRYKTEDVTATKGNEFEDYFLKREL

LMGIFEKGFEKPSPIQEESIPIALTGSDILARAKNGTGKTAAFCIPVIEKTDPSKNFIQALILVPTRELAFQTSQVCKEL

AKYLNIEVMVTTGGTSLKDDIMRLYQPVHILVGTPGRVLDLANKGVCKLRGCSMLVMDEADKLLSPESLPSIIQVISYLP

QNRQVLLFSATFPVTVKSFKDKFLQKPYIINLMDELTLKGITQYYAFVEERQKVHCLNTLFSKLQINQSIIFCNSVNRVE

LLAKKITELGYSCFYIHAKMVQSHRNKVFHDFRNGACRNLVCSDVFTRGIDIQAVNVVINFDFPKNSETYLHRVGRSGRF

GHLGLAVNLITYEDRVNFYNIERELGTEIKTIPADIDQAIYCQ

>XP_031125234.1 DEAD-box ATP-dependent RNA helicase 8-like [Ipomoea triloba]

MNARGRYSPGTGNGRGGGGDGYGGGGFQSNHNSRGGYRQGRNPHSQPQQYDKRSLQNEPHQQQQQWLRRNASAAPPESSY

NEVRKNIQSGGIDSSSNVWKARLNIPPPDTRYRTEDVTATKGNEFEDYFLKRELLMGIYEKGFERPSPIQEESIPIALTG

SDILARAKNGTGKTAAFCIPALEKIDPDNNVIQVVILVPTRELALQTSQVCKELGKHLKIQVMVSTGGTNLKDDIMRLYQ

PVHLLVGTPGRILDLAKRGVCILKDCAMIAMDEADKLLSPEFQPSVEQLIMFLPQHRQILMFSATFPVTIKDFKDRYLQK

PYIINLMDELTLKGITQFYAFVEERQKVHCLNTLFSKLQINQSIIFCNSVNRVELLAKKITEIGYSCFYIHAKMLQDHRN

KVFHDFRNGSCRNLVCTDLFTRGIDIQAVNVVINFDFPKNSETYLHRVGRSGRFGHLGLAVNLITYEDRFNLYKIEQELG

TEIKPIPPQIDQAIYCQ

>CBI34696.3 unnamed protein product, partial [Vitis vinifera]

MQNQQQQQWLRRIPIATDSPANEVEKTVQSEVVDSSSQDWKAQLRVPPPDTRYKTEDVTATKGNEFEDYFLKRELLMGIY

EKGFERPSPIQEESIPIALTGSDILARAKNGTGKTAAFCIPALEKIDPDNNVIQVVILVPTRELALQTSQVCKELGKHLK

IEVMVTTGGTSLKDDIMRLYQPVHLLVGTPGRILDLSKKGICILKDCSVLVMDEADKLLSPEFQPSVEQLIRFLPQNRQI

LMFSATFPVTVKDFKDRYLKKPYVINLMDELTLKGITQYYAFVEERQKVHCLNTLFSKLQINQSIIFCNSVNRVELLAKK

ITELGYSCFYIHAKMLQDHRNRVFHDFRNGACRNLVCTDLFTRGIDIQAVNVVINFDFPKNSETYLHRVGRSGRFGHLGL

AVNLITYEDRFNLYRIEQELGAEIKQIPPHIDQAIYCR

>KZV21818.1 DEAD-box ATP-dependent RNA helicase 8 [Dorcoceras hygrometricum]

MGGGRGGGINFNAGTNPIFQPRNPTQQYVQRNLAPNNQNHQLSQNPQPQQWLRRTQLPPADSAVEEVEKTVQSEAVASSS

QDWKAQLKLPPQDTRFKTEDVTATKGNEFEDYFLKRELLMGIYEKGFERPSPIQEESIPIALTGSDILARAKNGTGKTAA

FCIPALEKIEQDKNAIQVVILVPTRELALQTSQVCKELGKHLKIEVMVTTGGTSLKDDIMRLYQPVHLLVGTPGRILDLA

KKGICVLKDCSMLAMDEADKLLSPEFQPSVEQLIRFLPANRQILMFSATFPVTVKDFKDRYLKRPYIINLMDELTLKGIT

QYYAFVEERQKVHCLNTLFSKLQINQSIIFCNSVNRVELLAKKITELGFSCFYIHAKMLQDHRNRVFHDFRNGACRNLVC

TDLFTRGIDIQAVNVVINFDFPKNSETYLHRVGRSGRFGHLGLAVNLITFEDRFNLYRIEQELGTEIKQIPPHIDQAIYC

Q

>XP_017623498.1 PREDICTED: DEAD-box ATP-dependent RNA helicase 8 [Gossypium arboreum]

MNNRGRYPPVLGRGRGANANPSFQSRPEQPQYAQRNLVQNHHHFQQQQHHHLQQQQHQHQQQWLRRNQLPGGNDSTVADE

VEKTVQSEAVDSSSQDWKARLKLPPSDTRYKTEDVTATKGNEFEDYFLKRELLMGIYEKGFERPSPIQEESIPIALTGSD

ILARAKNGTGKTAAFCIPALEKIDQDNNVIQVVILLPTRELALQTSQVCKELGKHLQIQVMVTTGGTSLKDDIMRLYQPV

HLLVGTPGRILDLAKKGVCILKNCSMLIMDEADKLLSPEFQPSLEQLLRFLPPKRQILMFSATFPVTVKDFKDRYLKKPY

IINLMDELTLKGITQYYAFVEERQKVHCLNTLFSKLQINQSIIFCNSVNRVELLAKKITELGYSCFYIHAKMLQDHRNRV

FHDFRNGACRNLVCTDLFTRGIDIQAVNVVINFDFPKNSETYLHRVGRSGRFGHLGLAVNLITYEDRFNLYRIEQELGTE

IKQIPPHIDQAIYCR

>XP_015933871.1 DEAD-box ATP-dependent RNA helicase 8 [Arachis duranensis]

MNNNNNYYNRARYPPPGIGGGGLGRGGGGGGPGFNQNSPFQPRPNYQQHQQQHQQLQQQHQQYVQRQMVQQQQQQQQQQQ

QQQWLRRAQMGAADSNVVDEVEKTVQSEAIDPSSQDWKARLKVPPPDTRYKTEDVTATKGNEFEDYFLKRELLMGIYEKG

FERPSPIQEESIPIALTGSDILARAKNGTGKIAAFCIPALEKIDQDNNVIQVVILVPTRELALQTSQVCKELGKHLKIQV

MVTTGGTSLKDDIMRLYQPVHLLVGTPGRILDLAKKGVCVLKDCAMLVMDEADKLLSPEFQPSIQQLIHFLPSHRQILMF

SATFPVTVKDFKDRYLQKPYVINLMDELTLKGITQYYAFVEERQKVHCLNTLFSKLQINQSIIFCNSVNRVELLAKKITE

LGYSCFYIHAKMLQDHRNRVFHDFRNGACRNLVCTDLFTRGIDIQAVNVVINFDFPKNSETYLHRVGRSGRFGHLGLAVN

LITYEDRFNLYRIEQELGTEIKQIPPHIDQAIYCR

>XP_010512458.1 PREDICTED: DEAD-box ATP-dependent RNA helicase 12 [Camelina sativa]

MNNNNRGRYPPGVGTGRGAPPNPDYHHQSYRQQQQPQDHQQQQYVQRGYPQNPQQMQLQHQQQQQHQQQQWSRRPQIPGN

ANNANEVQKTSQSEATSEPNGQDWKATLRLPPPDTRYQTADVTATKGNEFEDYFLKRDLLKGIYEKGFEKPSPIQEESIP

IALTGSDILARAKNGTGKTGAFCIPVLEKIDPNNNIIQAMILVPTRELALQTSQVCKELSKYLNIQVMVTTGGTSLRDDI

MRLHQPVHLLVGTPGRILDLTKKGVCVLKDCGMLVMDEADKLLSAEFQPSLEELIQFLPQNRQFLMFSATFPVTVKAFKD

RHLRKPYVINLMDQLTLVGVTQYYAFVEERQKVHCLNTLFSKLQINQSIIFCNSVNRVELLAKKITELGYSCFYIHAKMV

QDHRNRVFHEFRNGACRNLVCTDLFTRGIDIQAVNVVINFDFPRTSESYLHRVGRSGRFGHLGLAVNLVTYEDRFKMYQT

EQELGTEIKPIPSNIDQAIYCQ

>XP_021280529.1 DEAD-box ATP-dependent RNA helicase 8 [Herrania umbratica]

MNSRGRYPPGIGVGRGGGVNANPSFQSRPPQQHYVQRNLVQNHQQFQHHQPQQHHQQQQHQQQQQWLRRNQLPGGNDSSV

VDEVEKTVQSEAVDSSSQDWKARLKIPPPDTRYKTEDVTATKGNEFEDYFLKRELLMGIYEKGFERPSPIQEESIPIALT

GSDILARAKNGTGKTAAFCIPALEKIDQDNNVIQVVILVPTRELALQTSQVCKELGKHLQIQVMVTTGGTSLKDDIMRLY

QPVHLLVGTPGRILDLAKKGVCILKDCSMLIMDEADKLLSPEFQPSVEQLIRFLPAYRQILMFSATFPVTVKDFKDRYLH

KPYVINLMDELTLKGITQYYAFVEERQKVHCLNTLFSKLQINQSIIFCNSVNRVELLAKKITELGYSCFYIHAKMLQDHR

NRVFHDFRNGACRNLVCTDLFTRGIDIQAVNVVINFDFPKNSETYLHRVGRSGRFGHLGLAVNLITYEDRFNLYRIEQEL

GTEIKQIPPHIDQAIYCR

>XP_028770364.1 DEAD-box ATP-dependent RNA helicase 8-like isoform X1 [Prosopis alba]

MNNRARYPPGIGVGRGGVNSNAAFQPRPPHQQQYVQRHLASQHQQYRHHHQQQQYQQHQPQQQNQQHHHQEQQWLRRTQP

GAADSNAVDEVEKTVQSEAVDPSSQDWKARLKIPPPDTRYKTEDVTATKGNEFEDYFLKRELLMGIYEKGFERPSPIQEE

SIPIALTGSDILARAKNGTGKTAAFCIPALEKIDQDNNVIQVVILVPTRELALQTSQVCKELGKHLKIQVMVTTGGTSLK

DDIMRLYQPVHLLVGTPGRILDLVKKGVCVLKDCNMLVMDEADKLLSPEFQPSVEQLIHFLPTNRQILMFSATFPVTIKD

FKDRYLQKPYVINLMDELTLKGITQFYAFVEERQKLHCLNTLFSKLQINQSIIFCNSVNRVELLAKKITELGYSCFYIHA

KMLQDHRNRVFHDFRNGACRNLVCTDLFTRGIDIQAVNVVINFDFPKNSETYLHRVGRSGRFGHLGLAVNLITYEDRFNL

YRIEQELGTEIKQIPPHIDQAIYCR

>XP_017630453.1 PREDICTED: DEAD-box ATP-dependent RNA helicase 8 [Gossypium arboreum]

MNSRGRYPPGIGVGRGGGVNANPSFQSRPSQQHYVQRNLLQNHQHFQQQQQQQHQQQQQHQQQLWLRRDQLPGGNDSSVV

DEVEKTVQSEAVDSSSQDWKARLNVPPPDTRYKTEDVTATKGNEFEDYFLKRELLMGIYEKGFERPSPIQEESIPIALTG

SDILARAKNGTGKTAAFCIPALEKIDQDNNVIQVVILVPTRELALQTSQVCKELGKHLQIQVMVTTGGTSLKDDIMRLYQ

PVHLLVGTPGRILDLAKKGVCILKDCSMLIMDEADKLLSPEFQPSIEQLIRFLPANRQILMFSATFPVTVKDFKDRYLKK

PYIINLMDELTLKGITQYYAFVEERQKVHCLNTLFSKLQINQSIIFCNSVNRVELLAKKITELGYSCFYIHAKMLQDHRN

RVFHDFRNGACRNLVCTDLFTRGIDIQAVNVVINFDFPKNSETYLHRVGRSGRFGHLGLAVNLITYEDRFNLYRIEQELG

TEIKQIPPHIDQAIYCR

>XP_022757281.1 DEAD-box ATP-dependent RNA helicase 8-like [Durio zibethinus]

MNSRGRYPPGIGVGRGGGVNANPSFQSRPPQQHYVQRNLVQNHQHFQQQQQQQHHQRQQLWLRRNQLPGVNDSTVVVEVE

KNVQSEAVDSSSQDWKAGLKIPPPDTRYKTEDVTATKGNEFEDYFLKRELLMGIYEKGFERPSPIQEESIPIALTGSDIL

ARAKNGTGKTAAFCIPALEKIDQDNNVIQVVILVPTRELALQTSQVCKELGKHLQIQVMVTTGGTSLKDDIMRLYQPVHL

LVGTPGRILDLAKKGVCILKDCSMLIMDEADKLLSPEFQPSIEQLIHFLPANRQILMFSATFPVTVKDFKDRYLQKPYII

NLMDELTLKGITQYYAFVEERQKVHCLNTLFSKLQINQSIIFCNSVNRVELLAKKITELGYSCFYIHAKMLQDHRNRVFH

DFRNGACRNLVCTDLFTRGIDIQAVNVVINFDFPKNSETYLHRVGRSGRFGHLGLAVNLITYEDRFNLYRIEQELGTEIK

QIPPHIDQAIYCR

>PSS24366.1 DEAD-box ATP-dependent RNA helicase [Actinidia chinensis var. chinensis]

MNTRGRYPPGIGGGGGRGGGVNSNPNFQPRNFQQQYVQRSPMQNHQQFQSQQQQQQQQWLRRNQLGAELSVDEVEKTVQS

EAVESSSQDWKAQLRIPPPDTRYRTEDVTATKGNEFEDYFLKRELLMGIYEKGFERPSPIQEESIPIALTGSDILARAKN

GTGKTAAFCIPALEKIDQDNNVIQVVILVPTRELALQTSQVCKELGKHLKIQVMVTTGGTSLKDDIMRLYQPVHLLVGTP

GRILDLAKKGICVLKDCSVLVMDEADKLLSPEFQPSVEQLIHFLPANRQVLMFSATFPVTVKDFKDRYLHKPYVINLMDE

LTLKGITQYYAFVEERQKVHCLNTLFSKLQINQSIIFCNSVNRVELLAKKITELGYSCFYIHAKMLQDHRNRVFHDFRNG

ACRNLVCTDLFTRGIDIQAVNVVINFDFPKNSETYLHRVGRSGRFGHLGLAVNLITYEDRFNLYRIEQELGTEIKQIPPH

IDQAIYCQ

>MBA0708574.1 hypothetical protein [Gossypium laxum]

MNSRGRYPPGIGVGRGGGLNSNPGFQSRPPQQNYGQRNFVQNHHQFHNQHQHQQQQQWLRRNQLPGTNDSSVVDEVEKTI

QSEAFDSSSQDWKARLKMPPPDTRYKTEDVTATKGNEFEDYFLKRELLMGIYEKGFERPSPIQEESIPIALTGSDILARA

KNGTGKTAAFCIPALEKIDQDKNVIQVVILVPTRELALQTSQVCKELGKHLQIQVMVTTGGTSLKDDIMRLYQPVHLLVG

TPGRILDLAKKGVCILKDCSMLILDEADKLLSPEFQPSIEQLIRFLSANRQILMFSATFPVTVKDFKDRYLQKPYIINLM

DELTLKGITQYYAFVEERQKVHCLNTLFSKLQINQSIIFCNSVNRVELLAKKITELGYSCFYIHAKMLQDHRNRVFHDFR

NGACRNLVCTDLFTRGIDIQAVNVVINFDFPKNSETYLHRVGRSGRFGHLGLAVNLITYEDRFNLYRIEQELGTEIKQIP

PHIDQAIYCR

>KAD3069131.1 hypothetical protein E3N88_37011 [Mikania micrantha]

MNNNYARGGGGRYPPGIGRGGGGGGSYHGNPNSNFQQQKNYQQQYSQRLPVHQQQFQQQQQQQQQQWLRRNPVGNDSSAV

AEVEKSIQSETADSSSQDWKAQLRLPPADTRYRTEDVTATKGNEFEDYFLKRELLMGIYEKGFERPSPIQEESIPIALTG

SDILARAKNGTGKTAAFCIPALEKIDQDKNVIQVVILVPTRELALQTSQVCKELGKHLNIQVMVTTGGTSLKDDIMRLYQ

PVHLLVGTPGRILDLAKKGICALKDCAMLVMDEADKLLSPEFQPSVEQLIHCMPRNRQILMFSATFPVTVKDFKDRYLQK

PYVINLMDELTLKGITQFYAFVEERQKVHCLNTLFSKLQINQSIIFCNSVNRVELLAKKITELGYSCFYIHAKMLQDHRN

RVFHDFRNGACRNLVCTDLFTRGIDIQAVNVVINFDFPKNSETYLHRVGRSGRFGHLGLAVNLITYEDRFNLYRIEQELG

TEIKQIPPFIDQAIYCR

>MBA0741328.1 hypothetical protein [Gossypium gossypioides]

MNSRGRYPPGIGVGRGGGVNANPSLQSRPSQQHYVQRNLLQNHQHFQQQQQQHQQQQQHQQQLWLRRDQLPGGNDSSVVD

EVEKTVQSEAVDSSSQDWKARLNVPPPDTRYKTEDVTATKGNEFEDYFLKRELLMGIYEKGFERPSPIQEESIPIALTGS

DILARAKNGTGKTAAFCIPALEKIDQDNNVIQVVILVPTRELALQTSQVCKELGKHLQIQVMVTTGGTSLKDDIMRLYQP

VHLLVGTPGRILDLAKKGVCILKDCSMLIMDEADKLLSPEFQPSIEQLIRFLPANRQILMFSATFPVTVKDFKDRYLKKP

YIINLMDELTLKGITQYYAFVEERQKVHCLNTLFSKLQINQSIIFCNSVNRVELLAKKITELGYSCFYIHAKMLQDHRNR

VFHDFRNGACRNLVCTDLFTRGIDIQAVNVVINFDFPKNSETYLHRVGRSGRFGHLGLAVNLITYEDRFNLYRIEQELGT

EIKQIPPHIDQAIYCR

>PON43078.1 DEAD-box ATP-dependent RNA helicase [Parasponia andersonii]

MNSNRGRYPPGIGAGRGGPMNANPAFQSRGPQQQYVQRGLVQNQPQYYQHQQQQHHQQQHHQQQHPQQQQQQQQQWLRRG

QLGGVADSNVDEVEKTVQSEAVDSSSQDWKARLKIPPADTRYKTEDVTATKGNEFEDYFLKRELLMGIYEKGFERPSPIQ

EESIPIALTGSDILARAKNGTGKTAAFCIPALEKIDQDLNAIQVVILVPTRELALQTSQVCKELGKHLNIQVMVTTGGTS

LKDDIMRLYQPVHLLVGTPGRILDLAKKGVCVLKDCAMLVMDEADKLLSPEFQPSIEQLIRFLPETRQILMYSATFPVTV

KDFKDRYLQKPYIINLMDELTLKGITQFYAFVEERQKVHCLNTLFSKLQINQSIIFCNSVNRVELLAKKITELGYSCFYI

HAKMLQDHRNRVFHDFRNGACRNLVCTDLFTRGIDIQAVNVVINFDFPKNSETYLHRVGRSGRFGHLGLAVNLITYEDRF

NLYRIEQELGTEIKQIPPHIDQAIYCR

>VVB06820.1 unnamed protein product [Arabis nemorensis]

MNNNRGRYPPGIGAGRGAINPNPNFQSRPSYHQQPPPQYVQRGGYGHQNHQQQFQQAPPSQSQGXXXXXXXXXXWLRRAQ

ISGGNSNGDAVVEFEKTVQSEAIDTNSEDWKARLKLPAPDTRYRTEDVTATKGNEFEDYFLKRELLMGIYEKGFERPSPI

QEESIPIALTGRDILARAKNGTGKTAAFCIPVLEKIDQDNNVIQAVIIVPTRELALQTSQVCKELGKHLKIQVMVTTGGT

SLKDDIMRLYQPVHLLVGTPGRILDLSRKGVCILKDCSVLVMDEADKLLSHEFQPSVEQLIGFLPENRQILMFSATFPVT

VKDFKDRFLTNPYVINLMDELTLKGITQFYAFVEERQKIHCLNTLFSKLQINQSIIFCNSVNRVELLAKKITELGYSCFY

IHAKMLQDHRNRVFHDFRNGACRNLVCTDLFTRGIDIQAVNVVINFDFPKNAETYLHRVGRSGRFGHLGLAVNLITYEDR

FNLYRIEQELGTEIKQIPPHIDQAIYCQ

>KAF3322385.1 DEAD-box ATP-dependent RNA helicase 8-like protein [Carex littledalei]

MDPRTRYPPPGIGNGRSGYVSGNPNYYGRNPNQPQQPQQQYVQRSPMPGQQYQQTWMRRNAPMGSDAGPSEPAGVKSAQL

SGTDTIDSSSQDWKVQLNLPPPDTRYKTEDVTATKGNEFEDYFLKRELLMGIYEKGFERPSPIQEESIPIALTGSDILAR

AKNGTGKTAAFCIPALEKIDQDRNVIQVVILVPTRELALQTSQVCKELAKHLKIQVMVTTGGTSLKDDIIRLYQPVHLLV

GTPGRILDLSKKGICLLNDCSMLIMDEADKLLSPEFQPSIEQLIQFLPPNRQILMFSATFPVTVKQFKEKYLPKPYVINL

MDELTLKGITQFYAFVEERQKVHCLNTLFSKLQINQSIIFCNSVNRVELLAKKITELGYSCFYIHAKMLQDHRNRVFHDF

RNGACRNLVCTDLFTRGIDIQAVNVVINFDFPKNSETYLHRVGRSGRFGHLGLAVNLITYEDRFNLYRIEQELGTEIKTI

PPQIDQAIYCQ

>CAD1821029.1 unnamed protein product [Ananas comosus var. bracteatus]

MNPRGRYPPGYGNTGGGNTGGNPNYYARNPHPQQQYVPRNYAQNQQQQYVPRNYVSNRNQMGSDSGPSEVAKAAQPDGID

SSSQDWKAKLRIPPQDTRYKTEDVTATKGNEFEDYFLKRELLMGIYEKGFERPSPIQEESIPIALTGSDILARAKNGTGK

TAAFCIPALEKIDQDNNVIQVVILVPTRELALQTSQVCKELGKHLKIQIMVTTGGTSLKDDIMRLYQPVHLLVGTPGRIL

DLARKGVCILKDCSMLIMDEADKLLTPEFQPSVEQLIRFLPANRQILMFSATFPVTVKDFKDKYLPKPYIINLMDELTLK

GITQFYAFVEERQKVHCLNTLFSKLQINQSIIFCNSVNRVELLAKKITELGYSCFYIHAKMLQDHRNRVFHDFRNGACRN

LVCTDLFTRGIDIQAVNVVINFDFPRNAETYLHRVGRSGRFGHLGLAVNLITYEDRFNLYRIEQELGTEIKQIPPQIDQA

IYCR

>XP_024027687.1 DEAD-box ATP-dependent RNA helicase 8 [Morus notabilis]

MNSNRGRYPPGIGAGRGGGMHANPAFQSRAPQQQYVQRNLVQNQTQQHYIQQQQHHQHQHHHHQQQQQHQYQQQQQWLRR

GQLGGVADSGVVDEVEKTVQSEAVDSSSQDWKARLKIPPPDTRFRTEDVTATKGNEFEDYFLKRELLMGIYEKGFERPSP

IQEESIPIALTGSDILARAKNGTGKTAAFCIPALEKIDQDTNAIQVVILVPTRELALQTSQVCKELGKHLNIQVMVTTGG

TSLKDDIMRLYQPVHLLVGTPGRILDLAKKGVCVLKDCNMLVMDEADKLLSPEFQPSIEQLIRFVPENRQILMFSATFPV

TVKDFKDRYLQKPYVINLMDELTLKGITQFYAFVEERQKVHCLNTLFSKLQINQSIIFCNSVNRVELLAKKITELGYSCF

YIHAKMLQDHRNRVFHDFRNGACRNLVCTDLFTRGIDIQAVNVVINFDFPKNSETYLHRVGRSGRFGHLGLAVNLITYED

RFNLYRIEQELGTEIKQIPPHIDQAIYCR

>XP_010908424.1 DEAD-box ATP-dependent RNA helicase 8 isoform X1 [Elaeis guineensis]

MNSRGGRYPPGNGNGRGGSMGGNPNFYARNSQQQQQYVQRNPLQGQHNQQQQWLRRNLMGGDSGTSEVVKSVQSDSIDSS

SQDWKARLKVPPPDTRYKTEDVTATKGNEFEDYFLKRELLMGIYEKGFERPSPIQEESIPIALTGSDILARAKNGTGKTA

AFCIPALEKIDQDNNVIQVVILVPTRELALQTSQVCKELGKHLKIQVMVTTGGTSLKDDIMRLYQPVHLLVGTPGRVLDL

AKKGVCILKDCSMLIMDEADKLLSPEFQPSVEQLIRFLPANRQILLFSATFPVTVKDFKDRYLAKPYIINLMDELTLKGI

TQYYAFVEERQKVHCLNTLFSKLQINQSIIFCNSVNRVELLAKKITELGYSCFYIHAKMLQDHRNRVFHDFRNGACRNLV

CTDLFTRGIDIQAVNVVINFDFPKNAETYLHRVGRSGRYGHLGLAVNLITYEDRFNLYRIEQELGTEIKQIPPQIDQAVY

CR

>XP_017419046.1 PREDICTED: DEAD-box ATP-dependent RNA helicase 8-like [Vigna angularis]

MNNRDRERYPPGMGLGRGLNSNPGFQPRPHQQHQYVQRHMVQHHHPQQYQQNHHHHQQQQQQHHQHHQHQQQQQQQQQWL

RRNQLGGGTDTNVVEEVEKTVQSEAVDSSLQDWKARLNIPAPDTRYKTEDVTATKGNEFEDYFLKRELLMGIYEKGFERP

SPIQEESIPIALTGSDILARAKNGTGKTAAFCIPALEKIDQDNNVIQVVILVPTRELALQTSQVCKELGKHLKIQVMVTT

GGTSLKDDIMRLYQPVHLLVGTPGRILDLAKKGVCIMKDCSMLVMDEADKLLSPEFQPSIEQLIQFLPGTRQILMFSATY

PVTVKDFKDRYLRKPYVINLMDELTLKGITQYYAFVEERQKVHCLNTLFSKLQINQSIIFCNSVNRVELLAKKITELGYS

CFYIHAKMLQDHRNRVFHDFRNGACRNLVCTDLFTRGIDIQAVNVVINFDFPKNSETYLHRVGRSGRFGHLGLAVNLITY

EDRFNLYRIEQELGTEIKQIPPHIDQAIYCR

>XP_012489916.1 PREDICTED: DEAD-box ATP-dependent RNA helicase 8 [Gossypium raimondii]

MNSRGRYPPGIGVGRGGGVNANPNFQSRPSQQHYVQRNLLQNHHHFQQQHQQQQQQHQQQLWLRRDQLPGGNDSSVVDEV

EKTVQSEAVDSSSQDWKARLNVPPPDTRYKTEDVTATKGNEFEDYFLKRELLMGIYEKGFERPSPIQEESIPIALTGSDI

LARAKNGTGKTAAFCIPALEKIDQDNNVIQVVILVPTRELALQTSQVCKELGKHLQIQVMVTTGGTSLKDDIMRLYQPVH

LLVGTPGRILDLAKKGVCILKDCSMLIMDEADKLLSPEFQPSIEQLIHFLPANRQILMFSATFPVTVKDFKDRYLKKPYI

INLMDELTLKGITQYYAFVEERQKVHCLNTLFSKLQINQSIIFCNSVNRVELLAKKITELGYSCFYIHAKMLQDHRNRVF

HDFRNGACRNLVCTDLFTRGIDIQAVNVVINFDFPKNSETYLHRVGRSGRFGHLGLAVNLITYEDRFNLYRIEQELGTEI

KQIPPHIDQAIYCR

>GEX06215.1 DEAD-box ATP-dependent RNA helicase 8 [Tanacetum cinerariifolium]

MNNNARGGGGGRYPPGINRGGGGGGNYYGNTNPNFNQQRNYQQQQQQQVQHQQQQQQQWLRRNHSSGANEVEKTVQSETI

DSSSEDWKAQLRLPPADTRYRTEDVTATKGNEFEDYFLKRDLLMGIYEKGFERPSPVQEESIPIALTGSDILARAKNGTG

KTAAFCIPVLEKIDQDNNVIQAVILVPTRELALQTSQVCKELGKHLNIQVMVTTGGTSLKDDIMRLYQPVHLLVGTPGRI

LDLANKGICRLNNCAMLVMDEADKLLSPEFQPSVEQLIRFTLANRQILMFSATFPVTVKDFKERYLRKPYIINLMDELTL

KGITQFYAFVEERQKVHCLNTLFSKLQINQSIIFCNSVNRVELLAKKITELGYSCFYIHAKMLQDHRNRVFHDFRNGACR

NLVCTDLFTRGIDIQAVNVVINFDFPKNSETYLHRVGRSGRFGHLGLAVNLITYEDRYNLYRIEQELGTEIKQIPPFIDQ

AIYCR

>TYI13199.1 hypothetical protein ES332_A08G042100v1 [Gossypium tomentosum]

MNSRGRYLPAIGVGRGGGLNSNPGFQSRPPQQNYMQRNFVQNHHQFHNQHQHQQQQQQQWLRRNQLPGTNDSSVVDEVEK

TIQSEAFDSSSQDWKARLKMPPPDTRYKTEDVTATKGNEFEDYFLKRELLMGIYEKGFERPSPIQEESIPIALTGSDILA

RAKNGTGKTAAFCIPALEKIDQDKNVIQVVILVPTRELALQTSQVCKELGKHLQIQVMVTTGGTSLKDDIMRLYQPVHLL

VGTPGRILDLAKKGVCILKDCSMLIMDEADKLLSPEFQPSIEQLIRFLSAKRQILMFSATFPVTVKDFKDRYLQKPYIIN

LMDELTLKGITQYYAFVEERQKVHCLNTLFSKLQINQSIIFCNSVNRVELLAKKITELGYSCFYIHAKMLQDHRNRVFHD

FRNGACRNLVCTDLFTRGIDIQAVNVVINFDFPKNSETYLHRVGRSGRFGHLGLAVNLITYEDRFNLYRIEQELGTEIKQ

IPPHIDQAIYCR

>XP_002271357.1 PREDICTED: DEAD-box ATP-dependent RNA helicase 8 [Vitis vinifera]

MNPRGRYPPGIGNGRGGNFHSNPNFQNRNPNYQQQQYFQRPPMQNQQQQQWLRRIPIATDSPANEVEKTVQSEVVDSSSQ

DWKAQLRVPPPDTRYKTEDVTATKGNEFEDYFLKRELLMGIYEKGFERPSPIQEESIPIALTGSDILARAKNGTGKTAAF

CIPALEKIDPDNNVIQVVILVPTRELALQTSQVCKELGKHLKIEVMVTTGGTSLKDDIMRLYQPVHLLVGTPGRILDLSK

KGICILKDCSVLVMDEADKLLSPEFQPSVEQLIRFLPQNRQILMFSATFPVTVKDFKDRYLKKPYVINLMDELTLKGITQ

YYAFVEERQKVHCLNTLFSKLQINQSIIFCNSVNRVELLAKKITELGYSCFYIHAKMLQDHRNRVFHDFRNGACRNLVCT

DLFTRGIDIQAVNVVINFDFPKNSETYLHRVGRSGRFGHLGLAVNLITYEDRFNLYRIEQELGAEIKQIPPHIDQAIYCR

>KAE8709408.1 DEAD-box ATP-dependent RNA helicase 6 [Hibiscus syriacus]

MNSRGRNPPPGIGVGRGGGVIANPSFQSRPSQQHYVQRNLVQNQQQFQQQQHQQQQQWLRRNQFPGGNDSSVIHEVEKTV

QTEAVDSSSQDWKARLKIPPADTRYKTEDVTATKGNEFEDYFLKRELLMGIYEKGFERPSPIQEESIPIALTGSNILARA

KNGTGKTAAFCIPALEKIDQDNNVIQVVILVPTRELALQTSQVCKELGKHLQIQVMVTTGGTGLKDDIMRLYQPVHLLVG

TPGRILDLAKKGVCILKDCSMLIMDEADKLLSPEFQPSIEQLIHFLPATRQILMFSATFPVTVKDFKDRYLQKPYIINLM

DELTLKGITQYYAFVEERQKVHCLNTLFSKLQINQSIIFCNSVNRVELLAKKITELGYSCFYIHAKMLQNHRNRVFHDFR

NGACRNLVCTDLFTRGIDIQAVNVVINFDFPKNSETYLHRVGRSGRFGHLGLAVNLITYEDRFNLYRIEQELGTEIKQIP

PHIDQAIYCR

>XP_017625998.1 PREDICTED: DEAD-box ATP-dependent RNA helicase 8-like [Gossypium arboreum]

MNSRGRYPPGIGVGRGGGLNSNPGFQSRPPQQNYMQRNFVQNHHQFHNQHQHQQQQQQQWLRRNQLPGTNDSSVVDEVEK

TIQSEAFDSSSQDWKARLKMPPPDTRYKTEDVTATKGNEFEDYFLKRELLMGIYEKGFERPSPIQEESIPIALTGSDILA

RAKNGTGKTAAFCIPALEKIDQDKNVIQVVILVPTRELALQTSQVCKELGKHLQIQVMVTTGGTSLKDDIMRLYQPVHLL

VGTPGRILDLAKKGVCILKDCSMLIMDEADKLLSPEFQPSIEQLIRFLSAKRQILMFSATFPVTVKDFKDRYLQKPYIIN

LMDELTLKGITQYYAFVEERQKVHCLNTLFSKLQINQSIIFCNSVNRVELLAKKITELGYSCFYIHAKMLQDHRNRVFHD

FRNGACRNLVCTDLFTRGIDIQAVNVVINFDFPKNSETYLHRVGRSGRFGHLGLAVNLITYEDRFNLYRIEQELGTEIKQ

IPPHIDQAIYCR

>XP_009400273.1 PREDICTED: DEAD-box ATP-dependent RNA helicase 6-like [Musa acuminata subsp. malaccensis]

MNPRGRFPPPGMGNGRGGGASTNPGFYARNPQQHQHQHQQQYVQRNPGNGQPNRQFQHQHQQWSRRNWVGGDSGAGEVMK

SVQSEATDSSLQDWKAQLKIPPADSRYKTEDVTATKGNEFEDYFLKRELLMGIYEKGFERPSPIQEESIPIALTGSDILA

RAKNGTGKTAAFCIPALEKIDQDCNAIQVVILVPTRELALQTSQVCKELGKHLRIQVMVSTGGTSLKDDIMRLYQPVHLL

VGTPGRILDLAKKGVCVLKDCSMLIMDEADKLLSPEFQPTIEQLIQFLPANRQILMFSATFPVTVKDFKDKYLPKPYIIN

LMDELTLKGITQYYAFVEERQKVHCLNTLFSKLQINQSIIFCNSVNRVELLAKKITELGYSCFYIHAKMLQDHRNRVFHD

FRNGACRNLVCTDLFTRGIDIQAVNVVINFDFPKNSETYLHRVGRSGRFGHLGLAINLITYEDRFNLYRIEQELGAEIKQ

IPPQIDRTIYCT

>XP_022022288.1 DEAD-box ATP-dependent RNA helicase 6 [Helianthus annuus]

MNNNSSVNDGNNRRYPPGIGNNRSGGGWYGGGGGGGNLQQNPNYHNQPRNPNQIQQSQQQQQSQYVQRQQHAQQQYIQKQ

QWMKLNPNVSGSSTVNNNNSFEVEKAVQSEATVDSSSQDWKAQLNIPAADSRYKTEDVTATKGNEFEDYFLKRELLMGIY

EKGFERPSPIQEESIPIALTGSDILARAKNGTGKTAAFCIPALEKIDTDNNVIQVVILVPTRELALQTSQVCKELGKHLQ

IQVMVTTGGTSLKDDIMRLYQPVHLLVGTPGRILDLTKKGICKLDNCTMLVMDEADKLLSPEFQPSVEQLIGFLSVNRQI

LMFSATFPVTVKDFKDRYLRKPYVVNLMDELTLKGITQYYAFVEERQKVHCLNTLFSKLQINQSIIFCNSVNRVELLAKK

ITELGYSCFYIHAKMLQDHRNRVFHDFRNGACRNLVCTDLFTRGIDIQAVNVVINFDFPRNAETYLHRVGRSGRFGHLGL

AVNLITYEDRFNLYRIEQELGTEIKQIPPHIDQAIYCR

>KAF5731714.1 DEAD-box ATP-dependent RNA helicase 8-like [Tripterygium wilfordii]

MNNNNRGRYPPGIGVGRGGVNTNPNFQSRTPPQQQQYVQRNFLNNHQQFQQQQQQYHHNQQQHHHSQQQLQQQQQQQWLR

RGNIRGADSVDEVEKSVHSEAVDPSSQDWKARLKIPPADTRYKTEDVTATKGNEFEDYFLKRELLMGIYEKGFERPSPIQ

EESIPIALTGSDILARAKNGTGKTAAFCIPALEKIDQENNVIQVVILVPTRELALQTSQVCKELGKNMKIQVMVTTGGTS

LKDDIMRLYQPVHLLVGTPGRILDLAKKGICILKDCSVLVMDEADKLLSPEFQPSIEQLIRFLPASRQILMFSATFPVNV

KDFKDRYLQKPYVINLMDELTLKGITQFYAFVEERQKVHCLNTLFSKLQINQSIIFCNSVNRVELLAKKITELGYSCFYI

HAKMLQDHRNRVFHDFRNGACRNLVCTDLFTRGIDIQAVNVVINFDFPKNSETYLHRVGRSGRFGHLGLAVNLITYEDRF

NLYRIEQELGTEIKQIPPHIDQAIYCR

>XP_016695481.1 PREDICTED: DEAD-box ATP-dependent RNA helicase 8 [Gossypium hirsutum]

MNSRGRYPPGIGVGRGGGVNANPSFQSRPSQQHYVQRNLLQNHQHFQQQQHQQQQQHQQQLWLRRDQLPGGNDSSVVDEV

EKTVQSEAVDSSSQDWKARLNVPPPDTRYKTEDVTATKGNEFEDYFLKRELLMGIYEKGFERPSPIQEESIPIALTGSDI

LARAKNGTGKTAAFCIPALEKIDQDNNVIQVVILVPTRELALQTSQVCKELGKHLQIQVMVTTGGTSLKDDIMRLYQPVH

LLVGTPGRILDLAKKGVCILKDCSMLIMDEADKLLSPEFQPSIEQLIHFLPANRQILMFSATFPVTVKDFKDRYLKKPYI

INLMDELTLKGITQYYAFVEERQKVHCLNTLFSKLQINQSIIFCNSVNRVELLAKKITELGYSCFYIHAKMLQDHRNRVF

HDFRNGACRNLVCTDLFTRGIDIQAVNVVINFDFPKNSETYLHRVGRSGRFGHLGLAVNLITYEDRFNLYRIEQELGTEI

KQIPPHIDQAIYCR

>XP_007139882.1 hypothetical protein PHAVU_008G066400g [Phaseolus vulgaris]

MNNRDRERYPPGMGLGRGLNSNPGFQPRPHQQHQYVQRHMVQHHHPQQYQQNHHHQQQHQQHHQQHQHQQQQQQQQQQQW

LRRNQLGGGTDTNVVEEVEKTVQSEAVDPSSQDWKARLNIPAPDTRYKTEDVTATKGNEFEDYFLKRELLMGIYEKGFER

PSPIQEESIPIALTGSDILARAKNGTGKTAAFCIPALEKIDQDNNVIQVVILVPTRELALQTSQVCKELGKHLKIQVMVT

TGGTSLKDDIMRLYQPVHLLVGTPGRILDLAKKGVCIMKDCSMLVMDEADKLLSPEFQPSIEQLIQFLPGTRQILMFSAT

FPVTVKDFKDRYLRKPYVINLMDELTLKGITQYYAFVEERQKVHCLNTLFSKLQINQSIIFCNSVNRVELLAKKITELGY

SCFYIHAKMLQDHRNRVFHDFRNGACRNLVCTDLFTRGIDIQAVNVVINFDFPKNSETYLHRVGRSGRFGHLGLAVNLIT

YEDRFNLYRIEQELGTEIKQIPPHIDQAIYCR

>XP_003580213.1 DEAD-box ATP-dependent RNA helicase 6 [Brachypodium distachyon]

MDPRARYPPGTGNGRGGNPNYNGRGPPPAQQHHLQPPPPQVQQQYMQRQSQHHNQQLQQQQWLRRNQIAGEAEGAARASE

HHAPPATDGIDSSSQDWKTQLKLPPPDTRYQTEDVTATKGNEFEDYFLKRELLMGIYEKGFERPSPIQEESIPIALTGSD

ILARAKNGTGKTAAFCIPALEKIDQDKNAIQVAILVPTRELALQTSQVCKELGKHLKIQVMVTTGGTSLKDDIIRLHQPV

HLLVGTPGRVLDLTKKGICILKDCSMLIMDEADKLLSPEFQPSVEQLIRYLPMSRQILMFSATFPVTVKAFKDKYLPKPY

VINLMDELTLKGITQFYAFVEERQKVHCLNTLFSKLQINQSIIFCNSVNRVELLAKKITELGYSCFYIHAKMLQDHRNRV

FHDFRNGACRNLVCTDLFTRGIDIQAVNVVINFDFPKNAETYLHRVGRSGRFGHLGLAVNLITYEDRFNLYRIEQELGTE

IKPIPPQIDRTIYCQ

>KAA3471792.1 DEAD-box ATP-dependent RNA helicase 8-like [Gossypium australe]

MNSRGRYPPGIGVGRGGGLNSNLGFQSRPPQQNYVQRNFVQNHHQFHNQHQHQQQQQQQWLRRNQLPGTNDSSVVDEVEK

TIQSEAFDSSSQDWKARLKMPPPDTRYKTEDVTATKGNEFEDYFLKRELLMGIYEKGFERPSPIQEESIPIALTGSDILA

RAKNGTGKTAAFCIPALEKIDQDKNVIQVVILVPTRELALQTSQVCKELGKHLQIQVMVTTGGTSLKDDIMRLYQPVHLL

VGTPGRILDLAKKGVCILKDCSMLIMDEADKLLSPEFQPSIEQLIRFLSANRQILMFSATFPVTVKDFKDRYLQKPYIIN

LMDELTLKGITQYYAFVEERQKVHCLNTLFSKLQINQSIIFCNSVNRVELLAKKITELGYSCFYIHAKMLQDHRNRVFHD

FRNGACRNLVCTDLFTRGIDIQAVNVVINFDFPKNSETYLHRVGRSGRFGHLGLAVNLITYEDRFNLYRIEQELGTEIKQ

IPPHIDQAIYCRFPSSLLNFFLLANF

>MBA0860100.1 hypothetical protein [Gossypium schwendimanii]

MNSRGRYPPGIGVGRGGGVNANPSFQSRPSQQHYVQRNLLQNHQHFQQQQHQQQQQHQQQLWLRRDQLPGGNDSSVVDEV

EKTVQSEAVDSSSQDWKARLNVPPPDTRYKTEDVTATKGNEFEDYFLKRELLMGIYEKGFERPSPIQEESIPIALTGSDI

LARAKNGTGKTAAFCIPALEKIDQDNNVIQVVILVPTRELALQTSQVCKELGKHLQIQVMVTTGGTSLKDDIMRLYQPVH

LLVGTPGRILDLAKKGVCILKDCSMLIMDEADKLLSPEFQPSIEQLIHFLPASRQILMFSATFPVTVKDFKDRYLKKPYI

INLMDELTLKGITQYYAFVEERQKVHCLNTLFSKLQINQSIIFCNSVNRVELLAKKITELGYSCFYIHAKMLQDHRNRVF

HDFRNGACRNLVCTDLFTRGIDIQAVNVVINFDFPKNSETYLHRVGRSGRFGHLGLAVNLITYEDRFNLYRIEQELGTEI

KQIPPHIDQAIYCR

>XP_015062448.1 DEAD-box ATP-dependent RNA helicase 8-like [Solanum pennellii]

MNPRPRFQSPGMGGRGGGGGGSMHPNTNPNFQHRNSQQYIQRGPAPYQQPQPQQQQQHFQNQQTQQWLRRNQLPSPDSTV

AEVEKNVQSEAVDQSSQEWKARLKIPQPDTRYRTEDVTATKGNEFEDYFLKRELLMGIYEKGFERPSPIQEESIPIALTG

SDILARAKNGTGKTASFCIPALEKIDQDKNVIQAIILVPTRELALQTSQVCKELGKHLKIQVMVTTGGTSLKDDIMRLYQ

PVHLLVGTPGRILDLAKKGVCVLKDCSMFIMDEADKLLSPEFQPSIEQLICFLPANRQILMFSATFPVTVKDFKERYLRK

PYVINLMDELTLKGITQFYAFVEERQKIHCLNTLFSKLQINQSIIFCNSVNRVELLAKKITELGYSCFYIHAKMLQDHRN

RVFHDFRNGACRNLVCTDLFTRGIDIQAVNVVINFDFPKNSETYLHRVGRSGRFGHLGLAVSLITFEDRFNLYRIEQELG

TEIKQIPPQIDQAIYCQ

>XP_019260131.1 PREDICTED: DEAD-box ATP-dependent RNA helicase 8-like [Nicotiana attenuata]

MNPRARFQPPGLGRGGGGSGSMHPNTNPYFQPRNSQQYIQRGPAPYQQQHQQQHFQNQQTQQWLRRNQLPSADSTVDEIE

KTVQSEAVDQSSQDWKARLKIPPPDTRYRTEDVTATKGNEFEDYFLKRELLMGIYEKGFERPSPIQEESIPIALTGSDIL

ARAKNGTGKTAAFCIPAFEKIDQDKNAIQAIILVPTRELALQTSQVCKELGKHLKIQVMVTTGGTSLKEDIMRLYQPVHL

LVGTPGRILDLAKKGVCILKDCSMLIMDEADKLLSPEFQPSIVQLIRFIPANRQILMFSATFPVTVKDFKDRYLNKPYVI

NLMDELTLKGITQFYAFVEERQKIHCLNTLFSKLQINQSIIFCNSVNRVELLAKKITELGYSCFYIHAKMLQDHRNRVFH

DFRNGACRNLVCTDLFTRGIDIQAVNVVINFDFPKNSETYLHRVGRSGRFGHLGLAVNLITYEDRFNLYRIEQELGTEIK

QIPPQIDQAIYCQ

>XP_012473360.1 PREDICTED: DEAD-box ATP-dependent RNA helicase 8-like [Gossypium raimondii]

MNSRGRYPPGIGVGRGGGLNSNPGFQSRPPQQNYVQRNFVQNHHQFHNQHQHQQQQQWLRRNQLPGTNDSSVVDEVEKTI

QSEAFDSSSQDWKARLKMPPPDTRYKTEDVTATKGNEFEDYFLKRELLMGIYEKGFERPSPIQEESIPIALTGSDILARA

KNGTGKTAAFCIPALEKIDQDKNVIQVVILVPTRELALQTSQVCKELGKHLQIQVMVTTGGTSLKDDIMRLYQPVHLLVG

TPGRILDLAKKGVCILKDCSMLIMDEADKLLSPEFQPSIEQLIRFLSANRQILMFSATFPVTVKYFKDRYLQKPYIINLM

DELTLKGITQYYAFVEERQKVHCLNTLFSKLQINQSIIFCNSVNRVELLAKKITELGYSCFYIHAKMLQDHRNRVFHDFR

NGACRNLVCTDLFTRGIDIQAVNVVINFDFPKNSETYLHRVGRSGRFGHLGLAVNLITYEDRFNLYRIEQELGTEIKQIP

PHIDQAIYCR

>VAH16936.1 unnamed protein product [Triticum turgidum subsp. durum]

MNQNYYGGRNPHPQHHDYQQQQQQHAQRNSFPQQQQQQHAQRNSFSQQQQQQHQQQQHQQWLRRDQAAAQASGEGAARTV

ARLDAVDTSSQDWKAQLNIPAPDTRFRTEDVTATKGNEFEDYFLKRELLMGIYEKGFERPSPIQEESIPIALTGSDILAR

AKNGTGKTAAFCIPALEKIDPEKNAIQVVILVPTRELALQTSQVCKELGKYLNIEVMVSTGGTSLKDDIMRLYQPVHLLA

GTPGRILDLTKKGICMLNECSMLIMDEADKLLAPEFQPSVEQLIRYLPASRQLLLFSATFPVTVKDFKQKYLPRPYVINL

MDELTLKGITQYYAFVEERQKVHCLNTLFSKLQINQSIIFCNSVNRVELLAKKITELGYSCFYIHAKMLQDHRNRVFHDF

RNGACRNLVCTDLFTRGIDIQAVNVVINFDFPKTAETYLHRVGRSGRFGHLGLAVNLITYEDRFNMYRIEQELGTEIKTI

PPQIDLAEYCQ

>XP_016545103.1 PREDICTED: DEAD-box ATP-dependent RNA helicase 8-like [Capsicum annuum]

MNPRGRYPPPGMGGGGGGRGGGGNMYPNANPNFQPRNPQQQYVQRSLVNQQQQQQKNFQSQQTQQQWLRRNQQQLAASDA

NVDEVEKTVQSEAVDQSSQDWKARLKIPPADTRYRTEDVTATKGNEFEDYFLKRELLMGIYEKGFERPSPIQEESIPIAL

TGSDILARAKNGTGKTAAFCIPALEKIDQDINAIQVVILVPTRELALQTSQVCKELGKHLKIEVMVTTGGTSLKDDIMRL

YQPVHLLVGTPGRILDLARKGICVLKDCSMLIMDEADKLLSPEFQPSIVQLIRFLPANRQVLMFSATFPVTVKDFKERYL

QKPYVINLMDELTLKGITQFYAFVEERQKLHCLNTLFSKLQINQSIIFCNSVNRVELLAKKITELGYSCFYIHAKMLQDH

RNRVFHDFRNGACRNLVCTDLFTRGIDIQAVNVVINFDFPKNSETYLHRVGRSGRFGHLGLAVNLITYEDRFNLYRIEQE

LGTEIKQIPPHIDQAIYCL

>KAB2068538.1 hypothetical protein ES319_A08G037900v1 [Gossypium barbadense]

MNSRGRYPPAIGVGRGGGLNSNPGFQSRPPQQNYMQRNFVQNHHQFHNQHQHQQQQQQQWLRRNQLPGTNDSSVVDEVEK

TIQSEAFDSSSQDWKARLKMPPPDTRYKTEDVTATKGNEFEDYFLKRELLMGIYEKGFERPSPIQEESIPIALTGSDILA

RAKNGTGKTAAFCIPALEKIDQDKNVIQVVILVPTRELALQTSQVCKELGKHLQIQVMVTTGGTSLKDDIMRLYQPVHLL

VGTPGRILDLAKKGVCILKDCSMLIMDEADKLLSPEFQPSIEQLIRFLSAKRQILMFSATFPVTVKDFKDRYLQKPYIIN

LMDELTLKGITQYYAFVEERQKVHCLNTLFSKLQINQSIIFCNSVNRVELLAKKITELGYSCFYIHAKMLQDHRNRVFHD

FRNGACRNLVCTDLFTRGIDIQAVNVVINFDFPKNSETYLHRVGRSGRFGHLGLAVNLITYEDRFNLYRIEQELGTEIKQ

IPPHIDQAIYCR

>KAF3619040.1 DEAD-box ATP-dependent RNA helicase 6 [Capsicum annuum]

MGGGGGGRGGGGNMYPNANPNFQPRNPQQQYVQRSLVNQQQQQQKNFQSQQTQQQWLRRNQQQLAASDANVDEVEKTVQS

EAVDQSSQDWKARLKIPPADTRYRTEDVTATKGNEFEDYFLKRELLMGIYEKGFERPSPIQEESIPIALTGSDILARAKN

GTGKTAAFCIPALEKIDQDINAIQVVILVPTRELALQTSQVCKELGKHLKIEVMVTTGGTSLKDDIMRLYQPVHLLVGTP

GRILDLARKGICVLKDCSMLIMDEADKLLSPEFQPSIVQLIRFLPANRQVLMFSATFPVTVKDFKERYLQKPYVINLMDE

LTLKGITQFYAFVEERQKLHCLNTLFSKLQINQSIIFCNSVNRVELLAKKITELGYSCFYIHAKMLQDHRNRVFHDFRNG

ACRNLVCTDLFTRGIDIQAVNVVINFDFPKNSETYLHRVGRSGRFGHLGLAVNLITYEDRFNLYRIEQELGTEIKQIPPH

IDQAIYCL

>XP_020233166.1 DEAD-box ATP-dependent RNA helicase 8 isoform X2 [Cajanus cajan]

MNTNNNRERYPPGIGLARGAFNPNLHLNRNPNLNHNLNHNHNHSFQVRPPFQQQPHYVQRHLLQPLQQQQQQQQQQQQQQ

WLRRDVNAVDEVEKTVQSEAVDSSSQDWKARLKIPPPDTRYRTEDVTATKGNEFEDYFLKRELLMGIYEKGFERPSPIQE

ESIPIALTGSDILARAKNGTGKTAAFCIPALEKIDQDINVIQVVILVPTRELALQTSQVCKELGKHLKIQVMVTTGGTSL

KDDIMRLYQPVHLLVGTPGRILDLAKKGVCILKDCAMLVMDEADKLLSPEFQPSIEQLIHFLPTNRQILMFSATFPVTVK

DFKDRYLHKPYVINLMDELTLKGITQFYAFVEERQKVHCLNTLFSKLQINQSIIFCNSVNRVELLAKKITELGYSCFYIH

AKMLQDHRNRVFHDFRNGACRNLVCTDLFTRGIDIQAVNVVINFDFPKNSETYLHRVGRSGRFGHLGLAVNLITYEDRFN

LYRIEQELGTEIKQIPPQIDQAIYCR

>XP_020115296.1 DEAD-box ATP-dependent RNA helicase 8-like [Ananas comosus]

MNPRGRYPPGYGNAGGGNTGGNPNYYARNPHPQQQYVPRNYAQSQQQQYVPRNYVSNRNQMGSDSGPSEVAKAVQPDGID

SSSQDWKAQLRIPPQDTRYKTEDVTATKGNEFEDYFLKRELLMGIYEKGFERPSPIQEESIPIALTGSDILARAKNGTGK

TAAFCIPALEKIDQDNNVIQVVILVPTRELALQTSQVCKELGKHLKIQIMVTTGGTSLKDDIMRLYQPVHLLVGTPGRIL

DLARKGVCILKDCSMLIMDEADKLLTPEFQPSVEQLIRFLPSNRQILMFSATFPVTVKDFKDNYLPKPYIINLMDELTLK

GITQFYAFVEERQKVHCLNTLFSKLQINQSIIFCNSVNRVELLAKKITELGYSCFYIHAKMLQDHRNRVFHDFRNGACRN

LVCTDLFTRGIDIQAVNVVINFDFPRNAETYLHRVGRSGRFGHLGLAVNLITYEDRFNLYRIEQELGTEIKQIPPQIDQA

IYCR

>PHT70023.1 DEAD-box ATP-dependent RNA helicase 6 [Capsicum annuum]

MYPNANPNFQPRNPQQQYVQRSLVNQQQQQQKNFQSQQTQQQWLRRNQQQLAASDANVDEVEKTVQSEAVDQSSQDWKAR

LKIPPADTRYRTEDVTATKGNEFEDYFLKRELLMGIYEKGFERPSPIQEESIPIALTGSDILARAKNGTGKTAAFCIPAL

EKIDQDINAIQVVILVPTRELALQTSQVCKELGKHLKIEVMVTTGGTSLKDDIMRLYQPVHLLVGTPGRILDLARKGICV

LKDCSMLIMDEADKLLSPEFQPSIVQLIRFLPANRQVLMFSATFPVTVKDFKERYLQKPYVINLMDELTLKGITQFYAFV

EERQKLHCLNTLFSKLQINQSIIFCNSVNRVELLAKKITELGYSCFYIHAKMLQDHRNRVFHDFRNGACRNLVCTDLFTR

GIDIQAVNVVINFDFPKNSETYLHRVGRSGRFGHLGLAVNLITYEDRFNLYRIEQELGTEIKQIPPHIDQAIYCL

>XP_009340166.1 PREDICTED: DEAD-box ATP-dependent RNA helicase 8 [Pyrus x bretschneideri]

MNSNRGRYPPGIGAGRGGGMNANPAFQSRPPHQQQYVQRNLLPNHHHQQQYFQQQQQHQQHQQQQQQQQQQQQQQQWLRR

GQLGGSTSADSAVDEVEKTVQSEAVDPSSQDWKARLKIPAPDTRFRTEDVTATKGNEFEDYFLKRELLMGIYEKGFERPS

PIQEESIPIALTGSDILARAKNGTGKTAAFCIPALEKIDQDNNVIQVVILVPTRELALQTSQVCKELGKHLQIQVMVTTG

GTSLKDDIMRLYQPVHLLVGTPGRILDLAKKGVCILKECSMLVMDEADKLLSPEFQPSVEQLIRFLPSHRQILMFSATFP

VTVKDFKDRYLQKPYVINLMDELTLKGITQFYAFVEERQKVHCLNTLFSKLQINQSIIFCNSVNRVELLAKKITELGYSC

FYIHAKMLQDHRNRVFHDFRNGACRNLVCTDLFTRGIDIQAVNVVINFDFPKNSETYLHRVGRSGRFGHLGLAVNLITYE

DRFNLYRIEQELGTEIKQIPPHIDQAIYCR

>MBA0762541.1 hypothetical protein [Gossypium trilobum]

MNSRGRYPPGIGVGRGGGLNSNPGFQSRPPQQNYVQRNFVQNHHQFHNQHQHQQQQQWLRRNQLPGTNDSSVVDEVEKTI

QSEAFDSSSQDWKARLKMPPPDTRYKTEDVTATKGNEFEDYFLKRELLMGIYEKGFERPSPIQEESIPIALTGSDILARA

KNGTGKTAAFCIPALEKIDQDKNVIQVVILVPTRELALQTSQVCKELGKHLQIQVMVTTGGTSLKDDIMRLYQPVHLLVG

TPGRILDLAKKGVCILKDCSMLIMDEADKLLSPEFQPSIEQLIRFLSANRQILMFSATFPVTVKDFKDRYLQKPYIINLM

DELTLKGITQYYAFVEERQKVHCLNTLFSKLQINQSIIFCNSVNRVELLAKKITELGYSCFYIHAKMLQDHRNRVFHDFR

NGACRNLVCTDLFTRGIDIQAVNVVINFDFPKNSETYLHRVGRSGRFGHLGLAVNLITYEDRFNLYRIEQELGTEIKQIP

PHIDQAIYCQ

>KAB2623369.1 DEAD-box ATP-dependent RNA helicase 8 [Pyrus ussuriensis x Pyrus communis]

MNSNRGRYPPGIGAGRGGGMNANPAFQSRPPHQQQYVQRNLLPNHHHQQQYFQQQQQHQQQQQQQQQQQQWLRRGQLGGS

TSADSAVDEVEKTVQSEAVDPSSQDWKARLKIPAPDTRFRTEDVTATKGNEFEDYFLKRELLMGIYEKGFERPSPIQEES

IPIALTGSDILARAKNGTGKTAAFCIPALEKIDQDNNVIQVVILVPTRELALQTSQVCKELGKHLQIQVMVTTGGTSLKD

DIMRLYQPVHLLVGTPGRILDLAKKGVCILKECSMLVMDEADKLLSPEFQPSVEQLIRFLPSHRQILMFSATFPVTVKDF

KDRYLQKPYVINLMDELTLKGITQFYAFVEERQKVHCLNTLFSKLQINQSIIFCNSVNRVELLAKKITELGYSCFYIHAK

MLQDHRNRVFHDFRNGACRNLVCTDLFTRGIDIQAVNVVINFDFPKNSETYLHRVGRSGRFGHLGLAVNLITYEDRFNLY

RIEQELGTEIKQIPPHIDQAIYCR

>GER26432.1 dead box ATP-dependent RNA helicase [Striga asiatica]

MSYRARYPPPGMGGGTGGRNPNAGPNPNFQTRNPTQQYVQRGPSPSNQNHQLYQNPHQQQWLRRAQLSSAESNVDEVEKT

VQSEAIDSSSQDWKAHLKLPPQDTRYRTEDVTATKGNEFEDYFLKRELLMGIYEKGFERPSPIQEESIPIALTGSDILAR

AKNGTGKTAAFCIPALEKIDQDKNSIQVVILVPTRELALQTSQVCKELGKHLKVQVMATTGGTSLKDDIMRLYQPVHLLV

GTPGRILDLARKGVCILNECSMLVMDEADKLLSPEFQPSIEQLIRFLPANRQILMFSATFPVTVKDFKDRYLHRPYIINL

MDELTLKGISQFYAFVEERQKVHCLNTLFSKLQINQSIIFCNSVNRVELLAKKITELGYSCFYIHAKMLQDHRNRVFHDF

RNGACRNLVCTDLFTRGIDIQAVNVVINFDFPKNSETYLHRVGRSGRFGHLGLAVNLITYEDRFNLYRIEQELGTEIKQI

PPHIDQAIYCQ

>XP_031090832.1 DEAD-box ATP-dependent RNA helicase 6-like [Ipomoea triloba]

MHSRPRYPPPGMGGGGRGDGGLNSNANLAFQPRNPHQYVQRSPLPNQQWLRRNNQLTADSAVDEVEKTVQSEAIDSSSQD

WKAQLKLPPPDTRYRTEDVTATKGNEFEDYFLKRELLMGIYEKGFERPSPIQEESIPIALTGSDILARAKNGTGKTAAFC

IPALEKIDQDKNAIQCIILVPTRELALQTSQVCKELGKHLQIEIMVSTGGTSLKDDIMRLYQPVHLLVGTPGRILDLVRK

GVCILKDCSMLVMDEADKLLSPEFQPSIGQLIRFLPTNRQILMFSATFPVTVKDFKDRYLQKPYVINLMDELTLKGITQF

YAFVEERQKVHCLNTLFSKLQINQSIIFCNSVNRVELLAKKITELGYSCFYIHAKMLQDHRNRVFHDFRNGACRNLVCTD

LFTRGIDIQAVNVVINFDFPKNSETYLHRVGRSGRFGHLGLAVNLITYEDRFNLYRIEQELGTEIKQIPPHIDQGIYCR

>XP_024993230.1 DEAD-box ATP-dependent RNA helicase 8-like [Cynara cardunculus var. scolymus]

MNNNYARGGGGRYPPGIGRGGGGGNYYGNPNPNFQQQKNYQQQQYAQRNPVHHQQFQQQQQWLRRNPVENDSSVVDEVEK

TIRSEATDPSSQDWKAQLRLPPADTRYRTEDVTATKGNEFEDYFLKRELLMGIYEKGFERPSPIQEESIPIALTGSDILA

RAKNGTGKTAAFCIPALEKIDQDSNVIQVVILVPTRELALQTSQVCKELGKHLKIQVMVTTGGTSLKDDIMRLYQPVHLL

VGTPGRILDLSKKGICVLKDCAMLVMDEADKLLSPEFQPSVEQLIHFMPTNRQILMFSATFPVTVKDFKDRYLQKPYVIN

LMDELTLKGITQFYAFVEERQKVHCLNTLFSKLQINQSIIFCNSVNRVELLAKKITELGYSCFYIHAKMLQDHRNRVFHD

FRNGACRNLVCTDLFTRGIDIQAVNVVINFDFPKNSETYLHRVGRSGRFGHLGLAVNLITYEDRFNLYRIEQELGTEIKQ

IPPFIDQAIYCR

>XP_018676552.1 PREDICTED: DEAD-box ATP-dependent RNA helicase 8-like [Musa acuminata subsp. malaccensis]

MNPQGRYPPPGMGGSGAGANPNFYARNPQHQQQYVQRNPMQGQQNHQFQPQQHQHWSRRNQMGGDSGVGEVVKAVQSDAV

TLMDSSSQDWKALLKIPPSDTRYRTEDVTATKGNEFEDYFLKRELLMGIYEKGFERPSPIQEESIPIALTGSNILARAKN

GTGKTAAFCIPALEKIDQDRNVIQVVILVPTRELALQTSQVCKELGKHLKIQVMVTTGGTSLKDDIMRLYQPVHLLVGTP

GRILDLAKKGVCILKDCSMLIMDEADKLLAPEFQPSVEQLIRFLPASRQILLFSATFPVTVKDFKDRYLPKPYIINLMDE

LTLKGITQYYAFVEERQKVHCLNTLFSKLQINQSIIFCNSVNRVELLAKKITELGYSCFYIHAKMLQDHRNRVFHDFRNG

ACRNLVCTDLFTRGIDIQAVNVVINFDFPKNSETYLHRVGRSGRFGHLGLAVNLITYEDRFNLYRIEQELGTEIKQIPPQ

IDQAIYCR

>PHT35864.1 DEAD-box ATP-dependent RNA helicase 8 [Capsicum baccatum]

MNPRGRYPPPGMGGGGGGRGGGGNMYPNANPNFQPRNPQQQYVQRSLVNQQQQQQKNFQNQQTQQQWLRRNQQQLAASDA

NVDEVEKTVQSEAVDQSSQDWKARLKIPPADTRYRTEDVTATKGNEFEDYFLKRELLMGIYEKGFERPSPIQEESIPIAL

TGSDILARAKNGTGKTAAFCIPALEKIDQDINAIQVVILVPTRELALQTSQVCKELGKHLKIEVMVTTGGTSLKDDIMRL

YQPVHLLVGTPGRILDLARKGICVLKDCSMLIMDEADKLLSPEFQPSIVQLIRFLPANRQVLMFSATFPVTVKDFKERYL

QKPYVINLMDELTLKGITQFYAFVEERQKLHCLNTLFSKLQINQSIIFCNSVNRVELLAKKITELGYSCFYIHAKMLQDH

RNRVFHDFRNGACRNLVCTDLFTRGIDIQAVNVVINFDFPKNSETYLHRVGRSGRFGHLGLAVNLITYEDRFNLYRIEQE

LGTEIKQIPPHIDQAIYCL

>XP_027334499.1 DEAD-box ATP-dependent RNA helicase 8 isoform X1 [Abrus precatorius]

MNNNRARYPPGMGIGRPGFNPNPNPNPNHPFQARPPYQQQPHQQQYVQRHLVQQPQQQQQWLRRDSNVVDEVEKTVQSEA

VDPSSQDWKARLKIPPPDTRYRTEDVTATKGNEFEDYFLKRELLMGIYEKGFERPSPIQEESIPIALTGSDILARAKNGT

GKTAAFCIPALEKIDQDNNVIQVVILVPTRELALQTSQVCKELGKHLKIQVMVTTGGTSLKDDIMRLYQPVHLLVGTPGR

ILDLAKKGVCILKDCSMLVMDEADKLLSPEFQPSVEQLIHFLPRTRQILMFSATFPVTVKDFKDRYLQKPYVINLMDELT

LKGITQFYAFVEERQKVHCLNTLFSKLQINQSIIFCNSVNRVELLAKKITELGYSCFYIHAKMLQDHRNRVFHDFRNGAC

RNLVCTDLFTRGIDIQAVNVVINFDFPKNSETYLHRVGRSGRFGHLGLAVNLITYEDRFNLYRIEQELGTEIKQIPPHID

QAIYCR

>XP_004510805.1 DEAD-box ATP-dependent RNA helicase 8-like [Cicer arietinum]

MNNNRARYPPPGIGPGRGGSNPNLNLNLNQNTNQNPNQNPNMNNQFQHRPSYQHHNQQQQHQQQQQQQQQQQQQYYVQRQ

MVQQQQQQQQQQQQQQQWLRRAQLGGSESNNVVDEVEKTVQSEVADPSSQEWKARLKIPPPDTRYRTEDVTATKGNEFED

YFLKRELLMGIYEKGFERPSPIQEESIPIALTGSDILARAKNGTGKTAAFCIPALEKIDQDNNVIQVVILVPTRELALQT

SQVCKELGKHLKIQVMVTTGGTSLKDDIMRLYQPVHLLVGTPGRILDLAKKGVCVLKDCSMLVMDEADKLLSPEFQPSIQ

QLIHFLPTNRQILMFSATYPVTVKDFKDRYLHKPYVINLMDELTLKGITQFYAFVEERQKVHCLNTLFSKLQINQSIIFC

NSVNRVELLAKKITELGYSCFYIHAKMLQDHRNRVFHDFRNGACRNLVCTDLFTRGIDIQAVNVVINFDFPKNSETYLHR

VGRSGRFGHLGLAVNLITYEDRFNLYRIEQELGTEIKQIPPHIDQAFYCR

>XP_014498037.1 DEAD-box ATP-dependent RNA helicase 8 [Vigna radiata var. radiata]

MNNRDRERYPPGMGLGRGLNSNPGFQPRPHQQHQYVQRHMVQHHHPQQYQQNHHHHQQQQQHHQHHQHQQQQQQQQQWLR

RNQLGGGTDTNVVEEVEKTVQSEAVDSSSQDWKARLNIPAPDTRYKTEDVTATKGNEFEDYFLKRELLMGIYEKGFERPS

PIQEESIPIALTGSDILARAKNGTGKTAAFCIPALEKIDQDNNVIQVVILVPTRELALQTSQVCKELGKHLKIQVMVTTG

GTSLKDDIMRLYQPVHLLVGTPGRILDLAKKGVCILKDCSMLVMDEADKLLSPEFQPSIEQLIQFLPGTRQILMFSATFP

VTVKDFKDRYLRKPYVINLMDELTLKGITQYYAFVEERQKVHCLNTLFSKLQINQSIIFCNSVNRVELLAKKITELGYSC

FYIHAKMLQDHRNRVFHDFRNGACRNLVCTDLFTRGIDIQAVNVVINFDFPKNSETYLHRVGRSGRFGHLGLAVNLITYE

DRFNLYRIEQELGTEIKQIPPHIDQAIYCR

>XP_010940461.1 DEAD-box ATP-dependent RNA helicase 8 [Elaeis guineensis]

MNPRGRYPPGIGNGGGGNYGANSNYYERNPQLQQHYIPRNQIQSHQNQQFQQQQQQQQQWLRRNQMGSNSGSSAAAAKSV

QPDAVDSSSQDWKAQLKIPPPDTRYKTEDVTGTKGNEFEDYFLKRELLMGIYEKGFERPSPIQEESIPIALTGSDILARA

KNGTGKTAAFCIPALEKIDQDYNVIQVVILVPTRELALQTSQVCKELGKYLEIQVMVTTGGTSLKDDIMRLYQPVHLLVG

TPGRILDLAKKGVCILKDCAMLIMDEADKLLSPEFQPSIEQLICFLPANRQILMFSATFPVTVKQFKDRYLPKPYIINLM

DELTLKGITQYYAFVEERQKVHCLNTLFSKLQINQSIIFCNSVNRVELLAKKITELGYSCFYIHAKMLQDHRNRVFHDFR

NGACRNLVCTDLFTRGIDIQAVNVVINFDFPKNSETYLHRVGRSGRFGHLGLAVNLITYEDRFNLYRIEQELGTEIKQIP

PQIDQAIYCR

>KAE8780926.1 DEAD-box ATP-dependent RNA helicase 12 [Hordeum vulgare]

MHHPRARYPPGYDYGGGGGGGGGRGGNGGGGGGGGGGNQNYYSGRNPHPQQHDYHHQQQQHAPRNSFPQQQQQHAQRNSF

SQQQQQQQQQQQHQQWLRRDQAAAQSSGEGAARTVARLDAVDTSSQDWKAQLNIPAPDTRFRTEDVTATKGNEFEDYFLK

RELLMGIYEKGFERPSPIQEESIPIALTGSDILARAKNGTGKTAAFCIPALEKIDPEKNAIQVVILVPTRELALQTSQVC

KELGKYLNIEVMVSTGGTSLKDDIMRLYQPVHLLAGTPGRILDLTKKGICMLNECSMLIMDEADKLLAPEFQPSVEQLIR

YLPASRQLLLFSATFPVTVKDFKQKYLPRPYVINLMDELTLKGITQYYAFVEERQKVHCLNTLFSKLQINQSIIFCNSVN

RVELLAKKITELGYSCFYIHAKMLQDHRNRVFHDFRNGACRNLVCTDLFTRGIDIQAVNVVINFDFPKTAETYLHRVGRS

GRFGHLGLAVNLITYEDRFNMYRIEQELGTEIKTIPPQIDLAEYCQ

>QCE12285.1 ATP-dependent RNA helicase DDX6/DHH1 [Vigna unguiculata]

MNNRDRESSQDWKARLNIPAPDTRYKTEDVTATKGNEFEDYFLKRELLMGIYEKGFERPSPIQEESIPIALTGSDILARA

KNGTGKTAAFCIPALEKIDQDNNVIQVVILVPTRELALQTSQVCKELGKHLKIQVMVTTGGTSLKDDIMRLYQPVHLLVG

TPGRILDLAKKGVCILKDCSMLVMDEADKLLSPEFQPSIEQLIQFLPGTRQILMFSATFPVTVRDFKDRYLRKPYVINLM

DELTLKGITQYYAFVEERQKVHCLNTLFSKLQINQSIIFCNSVNRVELLAKKITELGYSCFYIHAKMLQDHRNRVFHDFR

NGACRNLVCTDLFTRGIDIQAVNVVINFDFPKNSETYLHRVGRSGRFGHLGLAVNLITYEDRFNLYRIEQELGTEIKQIP

PHIDQAIYCR

>XP_015697482.1 PREDICTED: LOW QUALITY PROTEIN: DEAD-box ATP-dependent RNA helicase 12-like [Oryza brachyantha]

MHHPRARYPPGYGSXGGNHNYYGRGPQPQPQHHHYQAHQQQQYAQRSNTQQQQQHQQQQWLRRNQASAAEPGEAAARTAA

QLDAVVDSSSEDWKSQLNLPAPDTRYKTEDVTATKGNEFEDYFLKRELLMGIYEKGFERPSPIQEESIPIALTGSDILAR

AKNGTGKTAAFCIPALEKIDPEKNAIQVVILVPTRELALQTSQVCKELGKYLNIQVMVSTGGTSLKDDIMRLYQPVHLLV

GTPGRILDLTRKGICVLKDCSMLIMDEADKLLAPEFQPSVEQLIRFLPANRQLLMFSATFPVTVKDFKEKYLPRPYVINL

MDELTLKGITQHYAFVEERQKVHCLNTLFSKLQINQSIIFCNSVNRVELLAKKITELGYSCFYIHAKMLQDHRNRVFHDF

RNGACRNLVCTDLFTRGIDIQAVNVVINFDFPKTSETYLHRVGRSGRFGHLGLAVNLITYEDRFNLYRIEQELGTEIKPI

PSQIDLAVYCQ

>XP_021717499.1 DEAD-box ATP-dependent RNA helicase 8-like isoform X1 [Chenopodium quinoa]

MNDNIRDRYPPGMGRGRGGMGVDIGGGGGGGKPNFQRRNYEQQQHYAQRGMMQNHHQQQHQFQRQQQQQQHQQHFQHHQQ

QLQQIQQQQNQQQKQMQQQQQQQQQWLRRGQLGGITEAGVDEVEKTVQSEAIDTSTQDWKARLKLPPPDTRFRTEDVTAT

KGNEFEDYFLKRELLMGIYEKGFERPSPIQEESIPIALTGSDILARAKNGTGKTAAFCIPALEKIDQDNNVIQVVILVPT

RELALQTSQVCKELGKHLNIQVMVTTGGTSLKDDIMRLYQPVHLLVGTPGRILDLAKKGICVLKDCSMLIMDEADKLLSP

EFQPSIEQLIQFMPTNRQILMFSATFPVTVKDFKDRYLHKPYVINLMDELTLKGITQFYAFVEERQKVHCLNTLFSKLQI

NQSIIFCNSVNRVELLAKKITELGYSCFYIHAKMLQDHRNRVFHDFRNGACRNLVCTDLFTRGIDIQAVNVVINFDFPKN

AETYLHRVGRSGRFGHLGLAVNLITYEDRFNLYKIEQELGTEIKQIPPQIDQAIYCR

>KOM37158.1 hypothetical protein LR48_Vigan03g053900 [Vigna angularis]

MVRRFHLGCNVKVFDRSCGRTVTAIATAFVCNFSVLLLLQDWKARLNIPAPDTRYKTEDVTATKGNEFEDYFLKRELLMG

IYEKGFERPSPIQEESIPIALTGSDILARAKNGTGKTAAFCIPALEKIDQDNNVIQVVILVPTRELALQTSQVCKELGKH

LKIQVMVTTGGTSLKDDIMRLYQPVHLLVGTPGRILDLAKKGVCIMKDCSMLVMDEADKLLSPEFQPSIEQLIQFLPGTR

QILMFSATYPVTVKDFKDRYLRKPYVINLMDELTLKGITQYYAFVEERQKVHCLNTLFSKLQINQSIIFCNSVNRVELLA

KKITELGYSCFYIHAKMLQDHRNRVFHDFRNGACRNLVCTDLFTRGIDIQAVNVVINFDFPKNSETYLHRVGRSGRFGHL

GLAVNLITYEDRFNLYRIEQELGTEIKQIPPHIDQAIYCR

>MBA0645164.1 hypothetical protein [Gossypium klotzschianum]

MNSRGRYPPGIGVGRGGGLNSNPGFQSRPPQQNYVQRNFVQNHHQFHNQHQHQQQQQWLRRNQLPGTNDSSVVDEVEKTI

QSEAFDSSSQDWKARLKMPPPDTRYKTEDVTATKGNEFEDYFLKRELLMGIYEKGFERPSPIQEESIPIALTGSDILARA

KNGTGKTAAFCIPALEKIDQDKNVIQVVILVPTRELALQTSQVCKELGKHLQIQVMVTTGGTSLKDDIMRLYQPVHLLVG

TPGRILDLAKKGVCILKDCSMLIMDEADKLLSPEFQPSIEQLIRFLSANRQILMFSATFPVTVKDFKDRYLQKPYIINLM

DELTLKGITQYYAFVEERQKVHCLNTLFSKLQINQSIIFCNSVNRVELLAKKITELGYSCFYIHAKMLQDHRNRVFHDFR

NGACRNLVCTDLFTRGIDIQAVNVVINFDFPKNSETYLHRVGRSGRFGHLGLAVNLITYEDRFNLYRIEQELGTEIKQIP

PHIDQAIYCR

>XP_016469763.1 PREDICTED: DEAD-box ATP-dependent RNA helicase 8-like isoform X1 [Nicotiana tabacum]

MNPRARFQPPGMGSGSMHPNTNSNFQPRNPQQYIQRGPAPYQHQQQQQHFQNQQTQQWLRRNQSPSADSTVDEVEKTVQA

EAVDQSSQDWKAKLKIPSPDTRYRTEDVTATKGNEFEDYFLKRELLMGIYEKGFERPSPIQEESIPIALTGSDILARAKN

GTGKTAAFCIPALEKIDQDKNAIQAVILVPTRELALQTSQVCKELGKHLKIQVMVTTGGTSLKDDIMRLYQPVHLLVGTP

GRILDLAKKGVCILKDCSMLIMDEADKLLSPEFQPSIVQLIRFIPANRQILMFSATFPVTVKDFKDRYLNKPYVINLMDE

LTLKGITQFYAFVEERQKIHCLNTLFSKLQINQSIIFCNSVNRVELLAKKITELGYSCFYIHAKMLQDHRNRVFHDFRNG

ACRNLVCTDLFTRGIDIQAVNVVINFDFPKNSETYLHRVGRSGRFGHLGLAVNLITFEDRFNLYRIEQELGTEIKQIPPQ

IDQAIYCQ

>BAJ88430.1 predicted protein [Hordeum vulgare subsp. vulgare]

MHHPRARYPPGYDYGGGGGGGRGGNGGGGGGGGGGNQNYYSGRNPHPQQHDYHHQQQQHAPRNSFPQQQQQHAQRNSFSQ

QQQQQQQQQQHQQWLRRDQAAAQSSGEGAARTVARLDAVDTSSQDWKAQLNIPAPDTRFRTEDVTATKGNEFEDYFLKRE

LLMGIYEKGFERPSPIQEESIPIALTGSDILARAKNGTGKTAAFCIPALEKIDPEKNAIQVVILVPTRELALQTSQVCKE

LGKYLNIEVMVSTGGTSLKDDIMRLYQPVHLLAGTPGRILDLTKKGICMLNECSMLIMDEADKLLAPEFQPSVEQLIRYL

PASRQLLLFSATFPVTVKDFKQKYLPRPYVINLMDELTLKGITQYYAFVEERQKVHCLNTLFSKLQINQSIIFCNSVNRV

ELLAKKITELGYSCFYIHAKMLQDHRNRVFHDFRNGACRNLVCTDLFTRGIDIQAVNVVINFDFPKTAETYLHRVGRSGR

FGHLGLAVNLITYEDRFNMYRIEQELGTEIKTIPPQIDLAEYCQ

>XP_018844913.1 DEAD-box ATP-dependent RNA helicase 8-like [Juglans regia]

MNNNRGRYPPGIGAGRGGGGGGGMNLNPAFQSRVPQQQYVQRGFMQQQNHHHQQQYHHQQQQQQQQQQQWLRRAQLGGSD

SIVDEVEKTVQSEAADSSSQDWKARLKIPPSDTRYRTEDVTATKGNEFEDYFLKRELLMGIYEKGFERPSPIQEESIPIA

LTGSDILARAKNGTGKTAAFCIPALEKIDQDNNAIQVVILVPTRELALQTSQVCKELGKHLNIQVMVTTGGTSLKDDIMR

LYQPVHLLVGTPGRILDLAKKGVCILKDCSVLVMDEADKLLSPEFQPSIEQLIRFMPANRQILMFSATFPVTVKDFKERY

LQRPYIINLMDELTLKGITQFYAFVEERQKVHCLNTLFSKLQINQSIIFCNSVNRVELLAKKITELGYSCFYIHAKMLQD

HRNRVFHDFRNGACRNLVCTDLFTRGIDIQAVNVVINFDFPKNSETYLHRVGRSGRFGHLGLAVNLITYEDRFNLYRIEQ

ELGTEIKQIPPHIDQAIYCR

>XP_028096854.1 DEAD-box ATP-dependent RNA helicase 8-like [Camellia sinensis]

MNNTRGRYPPGMGGGGGGRGGGGMHANPNFQPRNFQQQYVQRSPMQNHLQYQNQQHHHHQQQQQQQWLRRNQLGAESSVV

EVEKTVQSEAVDSSSQDWKARLKIPPPDTRYMTEDVTATKGNEFEDYFLKRELLMGIYEKGFERPSPIQEESIPIALTGS

DILARAKNGTGKTAAFCIPALEKIDQDNNVIQVVILVPTRELALQTSQVCKELGKHLQIQVMVTTGGTSLKDDIMRLYQP

VHLLVGTPGRILDLAKKGVCVLKDCAMLVMDEADKLLSPEFQPSVEQLIRFLPANRQILMFSATFPVTVKDFKDRYLQKS

YIINLMDELTLKGITQFYAFVEERQKVHCLNTLFSKLQINQSIIFCNSVNRVELLAKKITELGYSCFYIHAKMLQDHRNR

VFHDFRNGACRNLVCTDLFTRGIDIQAVNVVINFDFPKNAETYLHRVGRSGRFGHLGLAVNLITYEDRFNLYRIEQELGT

EIKQIPPHIDQGIYCR

>XP_019159556.1 PREDICTED: DEAD-box ATP-dependent RNA helicase 6-like isoform X1 [Ipomoea nil]

MHSRARYPPPGMGGGGRGDGGLNSNANPAFQPRNPHQYVQRSPLPNQQWLRRNNQLTADSAVDEVEKTVQSEAIDSSSQD

WKARLKLPPPDTRYRTEDVTATKGNEFEDYFLKRELLMGIYEKGFERPSPIQEESIPIALTGSDILARAKNGTGKTAAFC

IPALEKIDQDKNAIQCIILVPTRELALQTSQVCKELGKHLQIEIMVTTGGTSLKDDIMRLYQPVHLLVGTPGRILDLVRK

GVCILKDCSMLVMDEADKLLSPEFQPSIGQLIRFLPANRQILMFSATFPVTVKDFKDRYLQKPYVINLMDELTLKGITQF

YAFVEERQKVHCLNTLFSKLQINQSIIFCNSVNRVELLAKKITELGYSCFYIHAKMLQDHRNRVFHDFRNGACRNLVCTD

LFTRGIDIQAVNVVINFDFPKNSETYLHRVGRSGRFGHLGLAVNLITFEDRFNLYRIEQELGTEIKQIPPHIDQGIYCQ

>VAH05016.1 unnamed protein product [Triticum turgidum subsp. durum]

MNQNYYGGRNPHPQHHDYQQQQPHAQRNSFPQQQQQQHAQRNSFSQQQQQQHQQQQHQQWLRRDQAAAQASGEGAARTVA

RLDAVDTSSQDWKAQLNIPAPDTRFRTEDVTATKGNEFEDYFLKRELLMGIYEKGFERPSPIQEESIPIALTGSDILARA

KNGTGKTAAFCIPALEKIDPEKNAIQVVILVPTRELALQTSQVCKELGKYLNIEVMVSTGGTSLKDDIMRLYQPVHLLAG

TPGRILDLTKKGICMLNECSMLIMDEADKLLAPEFQPSVEQLIRYLPASRQLLLFSATFPVTVKDFKQKYLPRPYVINLM

DELTLKGITQYYAFVEERQKVHCLNTLFSKLQINQSIIFCNSVNRVELLAKKITELGYSCFYIHAKMLQDHRNRVFHDFR

NGACRNLVCTDLFTRGIDIQAVNVVINFDFPKTAETYLHRVGRSGRFGHLGLAVNLITYEDRFNMYRIEQELGTEIKTIP

PQIDLAEYCQ

>KAE9617707.1 putative RNA helicase [Lupinus albus]

MNNRGRYPPGMGLGRGGGGGGGLNPNPGFQPRPPQQQYVQRHIMQQPQQQQQYQQQQQQQQQKYQQQQQQQYQQQQQQPQ

QWLRRTQLGGGTDTNVVEEVEKTVQSEAIDQSSQDWKTGLKIPAADTRYRTEDVTATKGNEFEDYFLKRELLMGIYEKGF

ERPSPIQEESIPIALTGSDILARAKNGTGKTAAFCIPALEKIDQDNNVIQVVILVPTRELALQTSQVCKELGKHLKIQVM

VTTGGTSLKDDIMRLYQPVHLLVGTPGRILDLAKKGVCVLKDCSILAMDEADKLLSPEFQPSIEQLIQFLPGNRQILMFS

ATFPVTVKDFSDRHLRKPYIINLMDELTLKGITQFYAFVEERQKVHCLNTLFSKLQINQSIIFCNSVNRVELLAKKITEL

GYSCFYIHAKMLQDHRNRVFHDFRNGACRNLVCTDLFTRGIDIQAVNVVINFDFPKNSETYLHRVGRSGRFGHLGLAVNL

ITYEDRFNLYRIEQELGTEIKQIPPHIDQAIYCQ

>KAF6994742.1 hypothetical protein CFC21_011372 [Triticum aestivum]

MHHPRARYPPGYDYGGGRGGNGGGGGGGGGGGGGGGNQNYYGGRNPHPQHHDYQQQQQQHAQRSSFPQQQQQQHAQRNSF

SQQQQQQHQQQQHQQWLRRDQAAAQESGEGAARTVARLDAVDTSSQDWKAQLNIPAPDTRFRTEDVTATKGNEFEDYFLK

RELLMGIYEKGFERPSPIQEESIPIALTGSDILARAKNGTGKTAAFCIPALEKIDPEKNAIQVVILVPTRELALQTSQVC

KELGKYLNIEVMVSTGGTSLKDDIMRLYQPVHLLAGTPGRILDLTKKGICMLNECSMLIMDEADKLLAPEFQPSVEQLIR

YLPASRQLLLFSATFPVTVKDFKQKYLPRPYVINLMDELTLKGITQYYAFVEERQKVHCLNTLFSKLQINQSIIFCNSVN

RVELLAKKITELGYSCFYIHAKMLQDHRNRVFHDFRNGACRNLVCTDLFTRGIDIQAVNVVINFDFPKTAETYLHRVGRS

GRFGHLGLAVNLITYEDRFNMYRIEQELGTEIKTIPPQIDLAEYCQ

>TQE06739.1 hypothetical protein C1H46_007608 [Malus baccata]

MNSNRGRYPPGIGAGRGGGMNANPAFQSRPPYQQQYVQRNLLPNHHHQQQYFQQQQQHQQHQQYQQQQQQQQQQQQQWLR

RGQLGGSTSADSAVDEVEKTVQSEAVDPSYVIVPFGEMGFVMDMDVWHQLRSATNLPQSSPKISASQDWKARLKIPAPDT

RFRTEDVTATKGNEFEDYFLKRELLMGIYEKGFERPSPIQEESIPIALTGSDILARAKNGTGKTAAFCIPALEKIDQDNN

VIQVVILVPTRELALQTSQVCKELGKHLQIQVMVTTGGTSLKDDIMRLYQPVHLLVGTPGRILDLAKKGVCILKDCSMLV

MDEADKLLSPEFQPSVEQLIRFLPSHRQILMFSATFPVTVKDFKDRFLQKPYVINLMDELTLKGITQFYAFVEERQKVHC

LNTLFSKLQINQSIIFCNSVNRVELLAKKITELGYSCFYIHAKMLQDHRNRVFHDFRNGACRNLVCTDLFTRGIDIQAVN

VVINFDFPKNSETYLHRVGRSGRFGHLGLAVNLITYEDRFNLYRIEQELGTEIKQIPPHIDQAIYCRDDRVLSIQEAIAL

VVGIPCDASSLELCLCS

>XP_010671975.1 PREDICTED: DEAD-box ATP-dependent RNA helicase 8 [Beta vulgaris subsp. vulgaris]

MNDNVRGRYPPGMGRGRGGIGGDVGGLGGGGMNGNPNFQGRNFQQQPYAQRGMLPNHHQQQQQFEQQQYMQRQHQQNKQM

QQQQQQQWLRRNQSGGTTESSVDEVEKTVQSEAIDTSSQDWKARLKLPPQDTRYRTEDVTATKGNEFEDYFLKRELLMGI

YEKGFERPSPIQEESIPIALTGSDILARAKNGTGKTAAFCVPALEKIDQDNNVIQVVILVPTRELALQTSQVCKELGKHL

KIQVMVTTGGTSLKDDIMRLYQPVHLLVGTPGRILDLSKKGICVLKDCSMLIMDEADKLLSPEFQPSIEQLIRFMPTNRQ

ILMFSATFPVTVKDFKDRYLHKPYVINLMDELTLKGITQFYAFVEERQKVHCLNTLFSKLQINQSIIFCNSVNRVELLAK

KITELGYSCFYIHAKMLQDHRNRVFHDFRNGACRNLVCTDLFTRGIDIQAVNVVINFDFPKNAETYLHRVGRSGRFGHLG

LAVNLITYEDRFNLYKIEQELGTEIKQIPPHIDQAYYCH

>TMW87848.1 hypothetical protein EJD97_019395 [Solanum chilense]

MNPRGRYPPPGMGSGGGGGGGGGRGGGNMYPNANPNFQPRNPQQYVQRGPVNQQQQFQNQQAQQWLRRNQLASDSTVDEV

EKTVQSEAVDQSSQDWKARLKIPPADTRYRTEDVTATKGNEFEDYFLKRELLMGIYEKGFERPSPIQEESIPIALTGSDI

LARAKNGTGKTAAFCIPALEKIDQDVNAIQVVILVPTRELALQTSQVCKELGKHLKIEVMVTTGGTSLKDDIMRLYQPVH

LLVGTPGRILDLARKGICVLKDCSMLVMDEADKLLSPEFQPSIVQLIRFLPANRQVLMFSATFPVTVKDFKERYLQKPYV

INLMDELTLKGITQFYAFVEERQKLHCLNTLFSKLQINQSIIFCNSVNRVELLAKKITELGYSCFYIHAKMLQDHRNRVF

HDFRNGACRNLVCTDLFTRGIDIQAVNVVINFDFPKNSETYLHRVGRSGRFGHLGLAVNLITYEDRFNLYRIEQELGTEI

KQIPPHIDQAIYCL

>KAF3450176.1 hypothetical protein FNV43_RR06256 [Rhamnella rubrinervis]

MHNPRGRYPPGIGAGRGGGMNANPAFQSRAPQQQYVQRSLVQNQHYFHQQQQQHQQQQQQQQQWLRRGQLGSAADSNVDE

VEKTVQFEAVDSSSQDWKARLKLPPADTRYRTEDVTATKGNEFEDYFLKRELLMGIYEKGFERPSPIQEESIPIALTGSD

ILARAKNGTGKTAAFCIPALEKIDQDNNVIQVVILVPTRELALQTSQVCKELGKNLKIQVMVTTGGTSLKDDIMRLYQPV

HLLVGTPGRILDLAKKGVCILKDCSMLVMDEADKLLSPEFQPSIEQLIQFMPGNRQILMFSATFPVTVKDFKDRYLHKPY

VINLMDELTLKGITQYYAFVEERQKVHCLNTLFSKLQINQSIIFCNSVNRVELLAKKITELGYSCFYIHAKMLQDHRNRV

FHDFRNGACRNLVCTDLFTRGIDIQAVNVVINFDFPKNSETYLHRVGRSGRFGHLGLAVNLITYEDRFNLYRIEQELGTE

IKQIPPHIDQAIYCR

>XP_004248580.1 DEAD-box ATP-dependent RNA helicase 8-like [Solanum lycopersicum]

MNPRGRYPPPGMGSGGGGGGRGGGNMYPNANPNFQPRNPQQYVQRGPVNQQQQFQNQQAQQWLRRNQLASDSTVDEVEKT

VQSEAVDQSSQDWKARLKIPPADTRYRTEDVTATKGNEFEDYFLKRELLMGIYEKGFERPSPIQEESIPIALTGSDILAR

AKNGTGKTAAFCIPALEKIDQDVNAIQVVILVPTRELALQTSQVCKELGKHLKIEVMVTTGGTSLKDDIMRLYQPVHLLV

GTPGRILDLARKGICVLKDCSMLVMDEADKLLSPEFQPSIVQLIRFLPANRQVLMFSATFPVTVKDFKERYLQKPYVINL

MDELTLKGITQFYAFVEERQKLHCLNTLFSKLQINQSIIFCNSVNRVELLAKKITELGYSCFYIHAKMLQDHRNRVFHDF

RNGACRNLVCTDLFTRGIDIQAVNVVINFDFPKNSETYLHRVGRSGRFGHLGLAVNLITYEDRFNLYRIEQELGTEIKQI

PPHIDQAIYCL

>VAH05012.1 unnamed protein product [Triticum turgidum subsp. durum]

MHHPRARYPPGYDYGGGGGGRGGNGGGGGGGGGGGGNQNYYGGRNPHPQHHDYQQQQPHQQQQQHQQQQHQQWLRRDQAA

AQASGEGAARTVARLDAVDTSSQDWKAQLNIPAPDTRFRTEDVTATKGNEFEDYFLKRELLMGIYEKGFERPSPIQEESI

PIALTGSDILARAKNGTGKTAAFCIPALEKIDPEKNAIQVVILVPTRELALQTSQVCKELGKYLNIEVMVSTGGTSLKDD

IMRLYQPVHLLAGTPGRILDLTKKGICMLNECSMLIMDEADKLLAPEFQPSVEQLIRYLPASRQLLLFSATFPVTVKDFK

QKYLPRPYVINLMDELTLKGITQYYAFVEERQKVHCLNTLFSKLQINQSIIFCNSVNRVELLAKKITELGYSCFYIHAKM

LQDHRNRVFHDFRNGACRNLVCTDLFTRGIDIQAVNVVINFDFPKTAETYLHRVGRSGRFGHLGLAVNLITYEDRFNMYR

IEQELGTEIKTIPPQIDLAEYCQ

>XP_020149407.1 DEAD-box ATP-dependent RNA helicase 12 [Aegilops tauschii subsp. tauschii]

MHHPRARYPPGYDYGGGRGGNGGGGGGGGGGGGGGGNQNYYGGRNPHPQHHDYQQQQQQHAQRSSFPQQQQQHAQRNSFS

QQQQQQHQQQQHQQWLRRDQAAAQESGEGAARTVARLDAVDTSSQDWKAQLNIPAPDTRFRTEDVTATKGNEFEDYFLKR

ELLMGIYEKGFERPSPIQEESIPIALTGSDILARAKNGTGKTAAFCIPALEKIDPEKNAIQVVILVPTRELALQTSQVCK

ELGKYLNIEVMVSTGGTSLKDDIMRLYQPVHLLAGTPGRILDLTKKGICMLNECSMLIMDEADKLLAPEFQPSVEQLIRY

LPASRQLLLFSATFPVTVKDFKQKYLPRPYVINLMDELTLKGITQYYAFVEERQKVHCLNTLFSKLQINQSIIFCNSVNR

VELLAKKITELGYSCFYIHAKMLQDHRNRVFHDFRNGACRNLVCTDLFTRGIDIQAVNVVINFDFPKTAETYLHRVGRSG

RFGHLGLAVNLITYEDRFNMYRIEQELGTEIKTIPPQIDLAEYCQ

>XP_030490053.1 DEAD-box ATP-dependent RNA helicase 8 [Cannabis sativa]

MNNNNNNRGRYPPGIGAGRGGPMNANPAFQSRGPQQQYVQRGLVQNQPQYFHHQQQQHHQQQQQQYHQQQQQQQQQQQQQ

QWLRRGQLGGVADSSADEVEKTVQSEAVDSSSQDWKARLKIPPADTRYRTEDVTATKGNEFEDYFLKRELLMGIYEKGFE

RPSPIQEESIPIALTGSDILARAKNGTGKTAAFCIPALEKIDQDLNAIQVVILVPTRELALQTSQVCKELGKHLNIQVMV

TTGGTSLKDDIMRLYQPVHLLVGTPGRILDLSKKGVCILKDCAMLVMDEADKLLSPEFQPSIEQLIGFLPGNRQILMFSA

TFPVTVKDFKDRYLQKPYIINLMDELTLKGITQFYAFVEERQKVHCLNTLFSKLQINQSIIFCNSVNRVELLAKKITELG

YSCFYIHAKMLQDHRNRVFHDFRNGACRNLVCTDLFTRGIDIQAVNVVINFDFPKNSETYLHRVGRSGRFGHLGLAVNLI

TYEDRFNLYRIEQELGTEIKQIPPHIDQAIYCR

>XP_020260542.1 DEAD-box ATP-dependent RNA helicase 6-like [Asparagus officinalis]

MNQRGRYPPGIGNGRGADGPPNPSPNPNPNYYGRNPNQQHQLQQQFSQRGNQMQSPQQFQQQWLRRNQIGGDAGSSEVSK

PRQSQAIDSSSPDWKAQLKIPPRDTRYKTEDVTASKGNEFEDYFLKRELLMGIYEKGFESPSPIQEESIPIALTGSDILA

RAKNGTGKTAAFCIPALEKIDQDNNVIQVVILVPTRELALQTSQVCKELSKHLQIQVMVTTGGTSLKDDIMRLYQPVHLL

VGTPGRILDLAKKGVCVLKDCSMLIMDEADKLLSPEFLPSVEQLIRFLPANRQILLFSATFPVTVKDFKDRYLPKPYIIN

LMDELTLKGITQYYAFVEERQKVHCLNTLFSKLQINQSIIFCNSVNRVELLAKKITELGYSCFYIHAKMLQDHRNRVFHD

FRNGACRNLVCTDLFTRGIDIQAVNVVINFDFPKNSETYLHRVGRSGRFGHLGLAVNLITYEDRFNLYRIEQELGTEIKQ

IPAQIDQAIYCR

>TQE03838.1 hypothetical protein C1H46_010502 [Malus baccata]

MKHLPHVQFSMDLYSPVGPPSIVCPRWGLERLHVSMGINRIEDPYTSELTEKEISKGHRHCSQDWKARLKIPPADTRFRT

EDVTATKGNEFEDYFLKRELLMGIYEKGFERPSPIQEESIPIALTGSDILARAKNGTGKTAAFCIPALEKIDQDNNVIQV

VILVPTRELALQTSQVCKELGKHLQIQVMVTTGGTSLKDDIMRLYQPVHLLVGTPGRILDLAKKGVCILKDCSMLVMDEA

DKLMSPEFQPSVEQLIRFLPSHRQILMFSATFPVTVKDFKDRYLQKPYVINLMDELTLKGITQFYAFVEERQKVHCLNTL

FSKLQINQSIIFCNSVNRVELLAKKITELGYSCFYIHAKMLQDHRNRVFHDFRNGACRNLVCTDLFTRGIDIQAVNVVIN

FDFPKNSETYLHRVGRSGRFGHLGLAVNLITYEDRFNLYRIEQELGTEIKQIPPHIDQAIYCR

>XP_008794547.1 DEAD-box ATP-dependent RNA helicase 8-like [Phoenix dactylifera]

MNPRGGRYPPGIGNGRGGSAGGNPNFNGRNPQQHQQYVQRNPVQGQQLQQQQLQWSRRNQMGSDSGIREAMKSVQSDSID

SSSQDWKAQLNIPPPDTRYRTEDVTATKGNEFEDYFLKRELLMGIYEKGFERPSPIQEESIPIALTGSDILARAKNGTGK

TAAFCIPALEKIDQDNNVIQVVILVPTRELALQTSQVCKELGKHLKIQIMVTTGGTSLKDDIMRLYQPVHLVVGTPGRII

DLAKKGVCILKDCSMLIMDEADKLLSPEFQPSIEQLIRFLPANRQILLFSATFPVTVKEFKDRYLAKPYIINLMDELTLK

GITQYYAFVEERQKVHCLNTLFSKLQINQSIIFCNSVNRVELLAKKITELGYSCFYIHAKMLQDHRNRVFHDFRNGACRN

LVCTDLFTRGIDIQAVNVVINFDFPKNSETYLHRVGRSGRYGHLGLAVNLITYEDRFNLYRIEQELGTEIKQIPPQIDQT

IYCR

>GER46410.1 dead box ATP-dependent RNA helicase [Striga asiatica]

MSYRARYPPPGMGGGRGGVNPNVGPNPNFQPRNPTQQYVQRGPPASNQNHQLYQNPQPQQWPRRAQLASAGSDEVEKSVQ

SEATDSSSQDWKTRLKLPPQDTRYRTEDVTATKGNEFEDYFLKRELLMGIYEKGFESPSPIQEESIPIALTGSDILARAK

NGTGKTAAFCIPALEKIDQDKNSIQVVILVPTRELALQTSQVCKELGKHLKIQVMATTGGTSLKDDIMRLYQPVHLLVGT

PGRILDLARKGICILNECSILVMDEADKLLSPEFQPSIEQLIRFLPANRQILMFSATFPVTVKDFKDRYLQKPYIINLMD

ELTLKGITQFYAFVEERQKVHCLNTLFSKLQINQSIIFCNSVNRVELLAKKITEIGYSCFYIHAKMLQDHRNRVFHDFRN

GACRNLVCTDLFTRGIDIQAVNVVINFDFPKNSETYLHRVGRSGRFGHLGLAVNLITYEDRFNLYRIEQELGTEIKQIPP

HIDQAIYCH

>MBA0729609.1 hypothetical protein [Gossypium laxum]

MVSVLFTSFLTSSFSSQDWKARLNVPPPDTRYKTEDVTATKGNEFEDYFLKRELLMGIYEKGFERPSPIQEESIPIALTG

SDILARAKNGTGKTAAFCIPALEKIDQDNNVIQVVILVPTRELALQTSQVCKELGKHLQIQVMVTTGGTSLKDDIMRLYQ

PVHLLVGTPGRILDLAKKGVCILKDCSMLIMDEADKLLSPEFQPSIEQLIHFLPASRQILMFSATFPVTVKDFKDRYLKK

PYIINLMDELTLKGITQYYAFVEERQKVHCLNTLFSKLQINQSIIFCNSVNRVELLAKKITELGYSCFYIHAKMLQDHRN

RVFHDFRNGACRNLVCTDLFTRGIDIQAVNVVINFDFPKNSETYLHRVGRSGRFGHLGLAVNLITYEDRFNLYRIEQELG

TEIKQIPPHIDQAIYCR

>PON92041.1 DEAD-box ATP-dependent RNA helicase [Trema orientale]

MNSNRGRYPPGIGAGRGGPMSANPAFQSRGPQQQYVQRGLVQNQPQYYQHQQQQHHHQQHHQQQHPQQQQQQQWLRRGQL

GGVADSNVDEVEKTVQSEAVDSSSQDWKARLKIPPADTRYKTEDVTATKGNEFEDYFLKRELLMGIYEKGFERPSPIQEE

SIPIALTGSDILARAKNGTGKTAAFCIPALEKIDQDLNAIQVVILVPTRELALQTSQVCKELGKHLNIQVMVTTGGTSLK

DDIMRLYQPVHLLVGTPGRILDLAKKGVCVLKDCAMLVMDEADKLLSPEFQPSIEQLIRFLPETRQILMFSATFPVTVKD

FKDRYLQKPYIINLMDELTLKGITQFYAFVEERQKVHCLNTLFSKLQINQSIIFCNSVNRVELLAKKITELGYSCFYIHA

KMLQDHRNRVFHDFRNGACRNLVCTDLFTRGIDIQAVNVVINFDFPKNSETYLHRVGRSGRFGHLGLAVNLITYEDRFNL

YRIEQELGTEIKQIPPHIDQAIYCR

>XP_021717536.1 DEAD-box ATP-dependent RNA helicase 8-like isoform X1 [Chenopodium quinoa]

MNDNIRGRYPPGMGRGRGGMGVDVGGGGGGGNPNFQRRNYEQQQHYAQRGMMQNHHQQQHQFQRQQQQQQHQQHFQHHQQ

QLQQIQQQQNQQQKQMQQQQQQQQQWLRRGQLGGITEAGVDEVEKTVQSEAIDTSTQDWKARLKLPPPDTRFRTEDVTAT

KGNEFEDYFLKRELLMGIYEKGFERPSPIQEESIPIALTGSDILARAKNGTGKTAAFCIPALEKIDQDNNVIQVVILVPT

RELALQTSQVCKELGKHLNIQVMVTTGGTSLKDDIMRLYQPVHLLVGTPGRILDLAKKGICVLKDCSMLIMDEADKLLSP

EFQPSIEQLIQFMPTNRQILMFSATFPVTVKDFKDRYLHKPYVINLMDELTLKGITQFYAFVEERQKVHCLNTLFSKLQI

NQSIIFCNSVNRVELLAKKITELGYSCFYIHAKMLQDHRNRVFHDFRNGACRNLVCTDLFTRGIDIQAVNVVINFDFPKN

AETYLHRVGRSGRFGHLGLAVNLITYEDRFNLYKIEQELGTEIKQIPPQIDQAIYCR

>XP_021748887.1 DEAD-box ATP-dependent RNA helicase 8-like [Chenopodium quinoa]

MNDNSRYPPGMGRGRGGMGVDVGGGGGGNPNFQRRNYEHQQHYAQRGMMQNHHQQQHQFQRQQQQQQHQQHFQHHQQQLQ

QIQQQQIQQQKQQQQQQQWLRRGQVGGTTEAGVDEVEKTVQSEAIDTSTPDWKARLKLPPPDTRFRTEDVTATKGNEFED

YFLKRELLMGIYEKGFERPSPIQEESIPIALTGSDILARAKNGTGKTAAFCIPALEKIDQDNNVIQVVILVPTRELALQT

SQVCKELGKHLNIQVMVTTGGTSLKDDIMRLYQPVHLLVGTPGRILDLAKKGICVLKDCSMLIMDEADKLLSPEFQPSIE

QLIQFMPTNRQILMFSATFPVTVKDFKDRYLNKPYVINLMDELTLKGITQFYAFVEERQKVHCLNTLFSKLQINQSIIFC

NSVNRVELLAKKITELGYSCFYIHAKMLQDHRNRVFHDFRNGACRNLVCTDLFTRGIDIQAVNVVINFDFPKNAETYLHR

VGRSGRFGHLGLAVNLITYEDRFNLYKIEQELGTEIKQIPPQIDQAIYCR

>TYI54799.1 hypothetical protein E1A91_D11G098600v1 [Gossypium mustelinum]

MNSRGRYPPGIGVGRGGGVNANPSFQSRPSQQHYVQRNLLQNHQHFQQQQHQQQQQHQQQLWLRRDQLPGGNDSSVVGEV

EKTVQSEAVDSSSQDWKARLNVPPPDTRYKTEDVTATKGNEFEDYFLKRELLMGIYEKGFERPSPIQEESIPIALTGSDI

LARAKNGTGKTAAFCIPALEKIDQDNNVIQVVILVPTRELALQTSQVCKELGKHLQIQVMVTTGGTSLKDDIMRLYQPVH

LLVGTPGRILDLAKKGVCILKDCSMLIMDEADKLLSPEFQPSIEQLIHFLPANRQILMFSATFPVTVKDFKDRYLKKPYI

INLMDELTLKGITQYYAFVEERQKVHCLNTLFAKLQINQSIIFCNSVNRVELLAKKITELGYSCFYIHAKMLQDHRNRVF

HDFRNGACRNLVCTDLFTRGIDIQAVNVVINFDFPKNSETYLHRVGRSGRFGHLGLAVNLITYEDRFNLYRIEQELGTEI

KQIPPHIDQAIYCR

>MBA0610098.1 hypothetical protein [Gossypium davidsonii]

MNSRGRYPPGIGVGRGGGLNSNPGFQSRPPQQNYVQRNFVQNHHQFHNQHQHQQQQQWLRRNQLPGTNDSSVVDEVEKTI

QSEAFDSRSILVSSHCFVGIRLFVFLALSILKHIAWFYNYLPFPPTTFSSQDWKARLKMPPPDTRYKTEDVTATKGNEFE

DYFLKRELLMGIYEKGFERPSPIQEESIPIALTGSDILARAKNGTGKTAAFCIPALEKIDQDKNVIQVVILVPTRELALQ

TSQVCKELGKHLQIQVMVTTGGTSLKDDIMRLYQPVHLLVGTPGRILDLAKKGVCILKDCSMLIMDEADKLLSPEFQPSI

EQLIRFLSANRQILMFSATFPVTVKDFKDRYLQKPYIINLMDELTLKGITQYYAFVEERQKVHCLNTLFSKLQINQSIIF

CNSVNRVELLAKKITELGYSCFYIHAKMLQDHRNRVFHDFRNGACRNLVCTDLFTRGIDIQAVNVVINFDFPKNSETYLH

RVGRSGRFGHLGLAVNLITYEDRFNLYRIEQELGTEIKQIPPHIDQAIYCR

>KAF3450177.1 hypothetical protein FNV43_RR06257 [Rhamnella rubrinervis]

MHNPRGRYPPGMGAGRGGGMNPSPGFQPRPPQQQYVQRNFVQNQRQYLNQQQQQWLRRGQFGSAADSSVDEVEKTVQSEA

ADLSSQDWKARLNLPTPDTRYRTEDVTATKGNEFEDYFLKRELLMGIYEKGFERPSPIQEESIPIALTGSDILARAKNGT

GKTAAFCIPALEKIDQDNNVIQAVILVPTRELALQTSQVCKELGKHLSIQVMATTGGTSLKDDIMRLYQPVHLLVGTPGR

IIDLAKKGVCILKYCSMLVMDEADKLLSPEFQPSIEQLIHFLPSNRQILMFSATFPVTVKDFKDRYLHKPYVVNLMDELT

LKGITQYYAFVEERQKVHCLNTLFSKLQINQSIIFCNSVNRVELLAKKITELGYSCFYIHAKMLQDHRNRVFHDFRNGAC

RNLVCTDLFTRGIDIQAVNVVINFDFPKNAETYLHRVGRSGRFGHLGLAVNLITYEDRFNLYRIEQELGTEIKQIPPHID

QGIYCRYKV

>KAF6989253.1 hypothetical protein CFC21_006607 [Triticum aestivum]

MHHPRARYPPGYDYGGGGRGGNGGGGGGGGGGGGGNQNYYGGRNPHPQHHDYQQQQQQHAQRNSFPQQQQQQHAQRNSFS

QQQQQQHQQQQHQQWLRRDQAAAQASGEGAARTVARLDAVDTSSQDWKAQLNIPAPDTRFRTEDVTATKGNEFEDYFLKR

ELLMGIYEKGFERPSPIQEESIPIALTGSDILARAKNGTGKTAAFCIPALEKIDPEKNAIQVVILVPTRELALQTSQVCK

ELGKYLNIEVMVSTGGTSLKDDIMRLYQPVHLLAGTPGRILDLTKKGICMLNECSMLIMDEADKLLAPEFQPSVEQLIRY

LPASRQLLLFSATFPVTVKDFKQKYLPRPYVINLMDELTLKGITQYYAFVEERQKVHCLNTLFSKLQINQSIIFCNSVNR

VELLAKKITELGYSCFYIHAKMLQDHRNRVFHDFRNGACRNLVCTDLFTRGIDIQAVNVVINFDFPKTAETYLHRVGRSG

RFGHLGLAVNLITYEDRFNMYRIEQELGTEIKTIPPQIDLAEYCQ

>XP_008778709.1 DEAD-box ATP-dependent RNA helicase 6 [Phoenix dactylifera]

MNPRGRYPPGIGNGGGGHYGASPNYYGRNPQPQQHYIPRNQVQSQQNQQFQQLQQQQWLRRNQMGSNSGNSEAAAKSVQS

DAVDSSSQDWKARLKIPPPDTRYMTEDVTATKGNEFEDYFLKRELLMGIYEKGFERPSPIQEESIPIALTGSDILARAKN

GTGKTAAFCIPALEKIDQDSNVIQVVILVPTRELALQTSQVCKELGKYLKIQVMVTTGGTSLKDDIMRLYQPVHLLVGTP

GRILDLAKKGVCILKDSSMLILDEADKLLSPEFYPSIEQLICFLPANRQILMFSATFPVTVKQFKDRYLPKPYIINLMDE

LTLKGITQYYAFVEERQKVHCLNTLFSKLQINQSIIFCNSVNRVELLAKKITELGYSCFYIHAKMLQDHRNRVFHDFRNG

ACRNLVCTDLFTRGIDIQAVNVVINFDFPKNSETYLHRVGRSGRFGHLGLAVNLITYEDRFNLNRIEQELGTEIKQIPPQ

IDQTIYCR

>XP_022147355.1 DEAD-box ATP-dependent RNA helicase 8-like [Momordica charantia]

MNNRGRYPPGIGAGRGGGMNASPSFQSRPHQQQYVQRNLAPNQQYHQHQQLQQHQQWLKRNQLGGAPADTGVDEVEKTVQ

SEAVDSSSQDWKARLKIPPPDTRYKTEDVTATKGNEFEDYFLKRELLMGIYEKGFERPSPIQEESIPIALTGSDILARAK

NGTGKTAAFCIPALEKIDQDNNVIQVVILVPTRELALQTSQVCKELGKNLNIQVMVTTGGTSLKDDIMRLYQPVHLLVGT

PGRILDLAKKGVCVLKDCSMLIMDEADKLLSPEFQPSIEQLIRFLPANRQILMYSATFPVTVKDFKDRYLHKPYIINLMD

ELTLKGITQFYAFVEERQKVHCLNTLFSKLQINQSIIFCNSVNRVELLAKKITELGYSCFYIHAKMLQDHRNRVFHDFRN

GACRNLVCTDLFTRGIDIQAVNVVINFDFPKNSETYLHRVGRSGRFGHLGLAVNLITYEDRFNLYRIEQELGTEIKQIPP

HIDQAIYCR

>OAY71048.1 DEAD-box ATP-dependent RNA helicase 8 [Ananas comosus]

MNPRGRYPPGYGNAGGGNTGGNPNYYARNPHPQQQYVPRNYAQSQQQQYVPRNYVSNRNQMGSDSGPSEVAKAVQPDGID

SSSQDWKAQLRIPPQDTRYKTEDVTATKGNEFEDYFLKRELLMGIYEKGFERPSPIQEESIPIALTGSDILARAKNGTGK

TAAFCIPALEKIDQDNNVIQVVILVPTRELALQTSQVCKELGKHLKIQIMVTTGGTSLKDDIMRLYQPVHLLVGTPGRIL

DLARKGVCILKDCSMLIMDEADKLLTPEFQPSVEQLIRFLPANRQILMFSATFPVTVKDFKDNYLPKPYIINLMDELTLK

GITQFYAFVEERQKVHCLNTLFSKLQINQSIIFCNSVNRVELLAKKITELGYSCFYIHAKMLQDHRNRVFHDFRNGACRN

LVCTDLFTRGIDIQAVNVVINFDFPRNAETYLHRVGRSGRFGHLGLAVNLITYEDRFNLYRIEQELGTEIKQIPPQIDQA

IYCR

>TKV93024.1 hypothetical protein SEVIR_9G199600v2 [Setaria viridis]

MHVSVSQDWKAQLNIPAPDTRYRTEDVTATKGNEFEDYFLKRELLMGIYEKGFERPSPIQEESIPIALTGSDILARAKNG

TGKTAAFCIPALEKIDPEKNAIQVVILVPTRELALQTSQVCKELGKYLNIQVMVSTGGTSLKDDIMRLYQPVHLLVGTPG

RILDLTRKGICVLKDCSMLVMDEADKLLAPEFQPSVEALIHFLPPSRQLLMFSATFPVTVKEFKEKYLPKPYVINLMDEL

TLKGITQYYAFVEERQKVHCLNTLFSKLQINQSIIFCNSVNRVELLAKKITELGYSCFYIHAKMLQDHRNRVFHDFRNGA

CRNLVCTDLFTRGIDIQAVNVVINFDFPKTSETYLHRVGRSGRYGHLGLAVNLITYEDRFNLYRIEQELGTEIKTIPPQI

DLAVYCQ

>XP_010905111.1 DEAD-box ATP-dependent RNA helicase 8 [Elaeis guineensis]

MNPRGGRYPPGIGNGRGGSSGGNPNFNGRNPQQHQQYVQHYVQRHPMQGQQNQQLQQHQQLQWSRRNQMGTDSGTREAMK

SVQSDSIDSSSQDWKAQLNIPPPDTRYKTEDVTATKGNEFEDYFLKRELLMGIYEKGFERPSPIQEESIPIALTGSDILA

RAKNGTGKTAAFCIPALEKIDQDNNVIQVVILVPTRELALQTSQVCKELGKHLKIQIMVTTGGTSLKDDIMRLYQPVHLL

VGTPGRILDLAKKGVCVLKDCSMLIMDEADKLLSPEFQPSIDQLIHFLPTTRQILLFSATFPVTVKEFKDRYLAKPYIIN

LMDELTLKGITQYYAFVEERQKVHCLNTLFSKLQINQSIIFCNSVNRVELLAKKITELGYSCFYIHAKMLQDHRNRVFHD

FRNGACRNLVCTDLFTRGIDIQAVNVVINFDFPKNSETYLHRVGRSGRYGHLGLAVNLITFEDRFNLYRIEQELGTEIKQ

IPPQIDQAIYCR

>XP_028961543.1 DEAD-box ATP-dependent RNA helicase 8 [Malus domestica]

MNSNRGRYPPGIGAGRGGGMNANPAFQSRPPHQQQYVQRNLLPNHHHQQQYFQQQQQHQQHQQYQQQQQWLRRGQLGGST

SADSAVDEVEKTVQSEAVDPSSQDWKARLKIPAPDTRFRTEDVTATKGNEFEDYFLKRELLMGIYEKGFERPSPIQEESI

PIALTGSDILARAKNGTGKTAAFCIPALEKIDQDNNVIQVVILVPTRELALQTSQVCKELGKHLQIQVMVTTGGTSLKDD

IMRLYQPVHLLVGTPGRILDLAKKGVCILKDCSMLVMDEADKLLSPEFQPSVEQLIRFLPSHRQILMFSATFPVTVKDFK

DRFLQKPYVINLMDELTLKGITQFYAFVEERQKVHCLNTLFSKLQINQSIIFCNSVNRVELLAKKITELGYSCFYIHAKM

LQDHRNRVFHDFRNGACRNLVCTDLFTRGIDIQAVNVVINFDFPKNSETYLHRVGRSGRFGHLGLAVNLITYEDRFNLYR

IEQELGTEIKQIPPHIDQAIYCR

>KAF5733027.1 DEAD-box ATP-dependent RNA helicase 8 [Tripterygium wilfordii]

MNNNNRGRYPPGIDVGRGGGVNTNSNFQSRTPQQQQQQQYVQRNYVHNHQQFQQQQQHHHNQQQQQHHPHNQQQHYHNQQ

QLHHQQQQQWLRRGQLPGADAVVDEVEKTVQSEAVDPSSQDWKASLKIPPADTRYRTEDVTATKGNEFEDYFLKRELLMG

IYEKGFERPSPIQEESIPIALTGSDILARAKNGTGKTAAFCIPALEKIDLENNVIQVVILVPTRELALQTSQVCKELGKH

LKIQVMVTTGGTGLKDDIMRLYQPVHLLVGTPGRILDLAKKGICILKDCSMLVMDEADKLLSPEFQPSIEQLIRFLPASR

QILMFSATFPVTVKDFKDRYLQKPYVINLMDELTLKGITQFYAFVEERQKVHCLNTLFSKLQINQSIIFCNSVNRVELLA

KKITELGYSCFYIHAKMLQDHRNRVFHDFRNGACRNLVCTDLFTRGIDIQAVNVVINFDFPKNSETYLHRVGRSGRFGHL

GLAVNLITYEDRFNLYRIEQELGTEIKQIPPHINQEIYCR

>XP_009794053.1 PREDICTED: DEAD-box ATP-dependent RNA helicase 8-like isoform X1 [Nicotiana sylvestris]

MNPRARFQPPGMGRGGGGSGSMHPNTNSNFQPRNPQQYIQRGPLPYQHQQQQQHFQNQQTQQWLRRNQSPSADSTVDEVE

KTVQAEAVDQSSQDWKAKLKIPSPDTRYRTEDVTATKGNEFEDYFLKRELLMGIYEKGFERPSPIQEESIPIALTGSDIL

ARAKNGTGKTAAFCIPALEKIDQDKNAIQAVILVPTRELALQTSQVCKELGKHLKIQVMVTTGGTSLKDDIMRLYQPVHL

LVGTPGRILDLAKKGVCILKDCSMLIMDEADKLLSPEFQPSIVQLIRFIPANRQILMFSATFPVTVKDFKDRYLNKPYVI

NLMDELTLKGITQFYAFVEERQKIHCLNTLFSKLQINQSIIFCNSVNRVELLAKKITELGYSCFYIHAKMLQDHRNRVFH

DFRNGACRNLVCTDLFTRGIDIQAVNVVINFDFPKNSETYLHRVGRSGRFGHLGLAVNLITFEDRFNLYRIEQELGTEIK

QIPPQIDQAIYCQ

>XP_028769186.1 DEAD-box ATP-dependent RNA helicase 8-like [Prosopis alba]

MNNRARYPPGIGVGRGGGMNSNPAFQPRPPQQQYVQRHLVQNQQQYPHQYQQYQQQHQQLQQQQQQQQQWLRRTQLIGAD

SNVVDEVEKTVQSEAVDSSSQDWKARLKIPPPDTRYRTEDVTATKGNEFEDYFLKRELLMGIYEKGFERPSPIQEESIPI

ALTGSDILARAKNGTGKTAAFCVPALEKIDQDNNVIQVVILVPTRELALQTSQVCKELGKHLSIQVMVTTGGTSLKDDIM

RLYQPVHLLVGTPGRILDLAKKGVCVLKDCNMLVMDEADKLLSPEFQPSIEQLIHFMPPNRQILMFSATFPVTVKDFKDR

YLQKPYIINLMDELTLKGITQFYAFVEERQKVHCLNTLFSKLQINQSIIFCNSVNRVELLAKKITELGYSCFYIHAKMLQ

DHRNRVFHDFRNGACRNLVCTDLFTRGIDIQAVNVVINFDFPKNSETYLHRVGRSGRFGHLGLAVNLITYEDRFNLYRIE

QELGTEIKQIPPHIDQAIYCR

>XP_028783702.1 DEAD-box ATP-dependent RNA helicase 8-like [Prosopis alba]

MNNRARYPPGIGVGRGGGMNSNPAFQPRPPQQQYVQRHLVQNQQQYPHQYQQYQQQHQQLQQQQQQQQQQWLRRTQLIGA

DSNVVDEVEKTVQSEAVDSSSQDWKARLKIPPPDTRYRTEDVTATKGNEFEDYFLKRELLMGIYEKGFERPSPIQEESIP

IALTGSDILARAKNGTGKTAAFCVPALEKIDQDNNVIQVVILVPTRELALQTSQVCKELGKHLSIQVMVTTGGTSLKDDI

MRLYQPVHLLVGTPGRILDLAKKGVCVLKDCNMLVMDEADKLLSPEFQPSIEQLIHFMPPNRQILMFSATFPVTVKDFKD

RYLQKPYIINLMDELTLKGITQFYAFVEERQKVHCLNTLFSKLQINQSIIFCNSVNRVELLAKKITELGYSCFYIHAKML

QDHRNRVFHDFRNGACRNLVCTDLFTRGIDIQAVNVVINFDFPKNSETYLHRVGRSGRFGHLGLAVNLITYEDRFNLYRI

EQELGTEIKQIPPHIDQAIYCR

>KAF5744362.1 DEAD-box ATP-dependent RNA helicase 8 [Tripterygium wilfordii]

MNNNNRGRYPPGIGVGRGGEGNTNPNFQSRTPQQQQQYLQRNYVHNHQQFQQQQQHHHNQQQHHHNQQQHHHNQQQLQQQ

QQQQWLRRGQLPVAHSVVDEVEKTVQSEAVDPSSQDWKAGLKIPAADTRFKTEDVTATKGNEFEDYFLKRELLMGIYEKG

FERPSPIQEESIPIALTGSDILARAKNGTGKTAAFCIPALEKIDQENNVIQVVILVPTRELALQTSQVCKELAKHLKIQV

MVTTGGTSLKDDIMRLYQPVHLLVGTPGRILDLAKKGICILKDCSMLVMDEADKLLSPEFQPSIEQLIRFLSASRQILMF

SATFPVTVKDFKDRYLQKPYVINLMDELTLKGITQFYAFVEERQKVHCLNTLFSKLQINQSIIFCNSVNRVELLAKKITE

LGYSCFYIHAKMLQDHRNRVFHDFRNGACRNLVCTDLFTRGIDIQAVNVVINFDFPKNSETYLHRVGRSGRFGHLGLAVN

LITYEDRFNLHRIEQELGTEIKQIPPHINQAIYCR

>VAH05015.1 unnamed protein product [Triticum turgidum subsp. durum]

MHHPRARYPPGYDYGGGGGGRGGNGGGGGGGGGGGGNQNYYGGRNPHPQHHDYQQQQQQHQQQQHQQWLRRDQAAAQASG

EGAARTVARLDAVDTSSQDWKAQLNIPAPDTRFRTEDVTATKGNEFEDYFLKRELLMGIYEKGFERPSPIQEESIPIALT

GSDILARAKNGTGKTAAFCIPALEKIDPEKNAIQVVILVPTRELALQTSQVCKELGKYLNIEVMVSTGGTSLKDDIMRLY

QPVHLLAGTPGRILDLTKKGICMLNECSMLIMDEADKLLAPEFQPSVEQLIRYLPASRQLLLFSATFPVTVKDFKQKYLP

RPYVINLMDELTLKGITQYYAFVEERQKVHCLNTLFSKLQINQSIIFCNSVNRVELLAKKITELGYSCFYIHAKMLQDHR

NRVFHDFRNGACRNLVCTDLFTRGIDIQAVNVVINFDFPKTAETYLHRVGRSGRFGHLGLAVNLITYEDRFNMYRIEQEL

GTEIKTIPPQIDLAEYCQ

>KAA8520609.1 hypothetical protein F0562_014865 [Nyssa sinensis]

MNTRGRYPPGIGGGGRAGNVNANPNFQPRNPQQQYVQRSPMQNHQQFQNHQQFQQQQQQWLRRNQLVADSSVEEVEKTVQ

TEAIDSSSQDWKAQLKIPPADTRYQTEDVTATKGNEFEDYFLKRELLMGIYEKGFERPSPIQEESIPIALTGSDILARAK

NGTGKTAAFCIPALEKIDQDNNVIQVVILVPTRELALQTSQVCKELGKHLKIQVMVTTGGTSLKDDIMRLYQPVHLLVGT

PGRILDLAKKGVCILKDCSMLVMDEADKLLSPEFQPSVEQLIRFVSTNRQILMFSATFPVTVKDFKDRYLHKPYVINLMD

ELTLKGITQFYAFVEERQKVHCLNTLFSKLQINQSIIFCNSVNRVELLAKKITELGYSCFYIHAKMLQDHRNRVFHDFRN

GACRNLVCTDLFTRGIDIQAVNVVINFDFPRNSETYLHRVGRSGRYGHLGLAVNLITYEDRFNLYRIEQELGTEIKQIPP

HIDQAIYCR

>XP_027928340.1 DEAD-box ATP-dependent RNA helicase 8-like [Vigna unguiculata]

MNNRDRERYPPGMGLGRGLNSNPGFQPRPHQQHQYVQRHMVQHHHPQQYQQNHQHHQQQQQQHHQHHQHQQQQQQQQQQW

LRRNQLGGGTDTNVVEEVEKTVQSEAVDSSSQDWKARLNIPAPDTRYKTEDVTATKGNEFEDYFLKRELLMGIYEKGFER

PSPIQEESIPIALTGSDILARAKNGTGKTAAFCIPALEKIDQDNNVIQVVILVPTRELALQTSQVCKELGKHLKIQVMVT

TGGTSLKDDIMRLYQPVHLLVGTPGRILDLAKKGVCILKDCSMLVMDEADKLLSPEFQPSIEQLIQFLPGTRQILMFSAT

FPVTVRDFKDRYLRKPYVINLMDELTLKGITQYYAFVEERQKVHCLNTLFSKLQINQSIIFCNSVNRVELLAKKITELGY

SCFYIHAKMLQDHRNRVFHDFRNGACRNLVCTDLFTRGIDIQAVNVVINFDFPKNSETYLHRVGRSGRFGHLGLAVNLIT

YEDRFNLYRIEQELGTEIKQIPPHIDQAIYCR

>XP_012698600.1 DEAD-box ATP-dependent RNA helicase 12 [Setaria italica]

MHQPRARYPPGYGSGGGGRGGGGGNGGGGGGGGGGGNHNYYGRNPQPQPQHHHHYQHQQPQQQQQQQQLAHRNSSHLHQQ

WLRRDQAAASAAGPGDAAGRTASHLDAVDSSSQDWKAQLNIPAPDTRYRTEDVTATKGNEFEDYFLKRELLMGIYEKGFE

RPSPIQEESIPIALTGSDILARAKNGTGKTAAFCIPALEKIDPEKNAIQVVILVPTRELALQTSQVCKELGKYLNIQVMV

STGGTSLKDDIMRLYQPVHLLVGTPGRILDLTRKGICVLKDCSMLVMDEADKLLAPEFQPSVEALIHFLPPSRQLLMFSA

TFPVTVKEFKEKYLPKPYVINLMDELTLKGITQYYAFVEERQKVHCLNTLFSKLQINQSIIFCNSVNRVELLAKKITELG

YSCFYIHAKMLQDHRNRVFHDFRNGACRNLVCTDLFTRGIDIQAVNVVINFDFPKTSETYLHRVGRSGRYGHLGLAVNLI

TYEDRFNLYRIEQELGTEIKTIPPQIDLAVYCQ

>TKV93023.1 hypothetical protein SEVIR_9G199600v2 [Setaria viridis]

MARPLPPQAYLSTLPIRNPTLPDPPPKSKSETHERRNGETIGTLDLSRPSSRTLDPAMHQPRARYPPGYGSGGGGRGGGG

GNGGGGGGGGGGGNHNYYGRNPQPQPQHHHHYQHQQPPQQQQQQQQLAHRNSSHLHQQWLRRDQAPASAAGPGDAAGRTA

SHLDVDSSSQDWKAQLNIPAPDTRYRTEDVTATKGNEFEDYFLKRELLMGIYEKGFERPSPIQEESIPIALTGSDILARA

KNGTGKTAAFCIPALEKIDPEKNAIQVVILVPTRELALQTSQVCKELGKYLNIQVMVSTGGTSLKDDIMRLYQPVHLLVG

TPGRILDLTRKGICVLKDCSMLVMDEADKLLAPEFQPSVEALIHFLPPSRQLLMFSATFPVTVKEFKEKYLPKPYVINLM

DELTLKGITQYYAFVEERQKVHCLNTLFSKLQINQSIIFCNSVNRVELLAKKITELGYSCFYIHAKMLQDHRNRVFHDFR

NGACRNLVCTDLFTRGIDIQAVNVVINFDFPKTSETYLHRVGRSGRYGHLGLAVNLITYEDRFNLYRIEQELGTEIKTIP

PQIDLAVYCQ

>OMP00441.1 hypothetical protein COLO4_12680 [Corchorus olitorius]

MNSRGRYPPGIGVGRGGGVNANPNFQSRPPQQHYVQRNMVQNHQQFHQQQQQHHHQQQQQQQQQQQWLRRNALPSGNDSS

VVDEVEKTVQSEAVDSSSQDWKARLKIPPPDTRYRTEDVTATKGNEFEDYFLKRELLMGIYEKGFERPSPIQEESIPIAL

TGSDILARAKNGTGKTAAFCIPALEKIDQDNNVIQVVILVPTRELALQTSQVCKELGKNLQIQVMVTTGGTSLKDDIMRL

YQPVHLLVGTPGRILDLAKKGVCILKDCSMLIMDEADKLLSPEFQPSVEQLIRFLPAHRQILMFSATFPVTVKDFKDRYL

QKPYIINLMDELTLKGITQYYAFVEERQKVHCLNTLFSKLQINQSIIFCNSVNRVELLAKKITELGYSCFYIHAKMLQDH

RNRVFHDFRNGACRNLVCTDLFTRGIDIQAVNVVINFDFPKNSETYLHRVGRSGRFGHLGLAVNLITYEDRFNLYRIEQE

LGTEIKQIPPHIDQAIYCRVMKLVIQDVCPCTWVVHSKYQSSDNRGIVVFKVVPRARNSCLVLVGAFAHLFISINKRHWT

EDLVLACV

>KAF6983697.1 hypothetical protein CFC21_001827 [Triticum aestivum]

MHHPRARYPPGYDYGGGGGGRGGNGGGGGGGGGGGGNQNYYGGRNPHPQHHDYQQQQPHAQRNSFPQQQQQQHTQRNSFS

QQQQQQHQQQQHQQWLRRDQAAAQASGEGAARTVARLDAVDTSSQDWKAQLNIPAPDTRFRTEDVTATKGNEFEDYFLKR

ELLMGIYEKGFERPSPIQEESIPIALTGSDILARAKNGTGKTAAFCIPALEKIDPEKNAIQVVILVPTRELALQTSQVCK

ELGKYLNIEVMVSTGGTSLKDDIMRLYQPVHLLAGTPGRILDLTKKGICMLNECSMLIMDEADKLLAPEFQPSVEQLIRY

LPASRQLLLFSATFPVTVKDFKQKYLPRPYVINLMDELTLKGITQYYAFVEERQKVHCLNTLFSKLQINQSIIFCNSVNR

VELLAKKITELGYSCFYIHAKMLQDHRNRVFHDFRNGACRNLVCTDLFTRGIDIQAVNVVINFDFPKTAETYLHRVGRSG

RFGHLGLAVNLITYEDRFNMYRIEQELGTEIKTIPPQIDLAEYCQ

>THG00422.1 hypothetical protein TEA_019698 [Camellia sinensis var. sinensis]

MNNTRGRYPPGMGGGGGGRGGGGMHANPNFQPRNFQQQYVQRSPMQNHLQYQNQQHHHHQQQQQQQWLRRNQLGAESSVV

EVEKTVQSEAVDSSNESNLIVVCVIVMVYDTECSQDWKARLKIPPPDTRYMTEDVTATKGNEFEDYFLKRELLMGIYEKG

FERPSPIQEESIPIALTGSDILARAKNGTGKTAAFCIPALEKIDQDNNVIQVVILVPTRELALQTSQVCKELGKHLQIQV

MVTTGGTSLKDDIMRLYQPVHLLVGTPGRILDLAKKGVCVLKDCAMLVMDEADKLLSPEFQPSVEQLIRFLPANRQILMF

SATFPVTVKDFKDRYLQKSYIINLMDELTLKGITQFYAFVEERQKVHCLNTLFSKLQINQSIIFCNSVNRVELLAKKITE

LGYSCFYIHAKMLQDHRNRVFHDFRNGACRNLVCTDLFTRGIDIQAVNVVINFDFPKNAETYLHRVGRSGRFGHLGLAVN

LITYEDRFNLYRIEQELGTEIKQIPPHIDQGLTGMMVLDVVHNSASEGSLWKQDRFVEDFGLV

>VAH16935.1 unnamed protein product [Triticum turgidum subsp. durum]

MHHPRARYPPGYDYGGGGRGGNGGGGGGGGGGGGGNQNYYGGRNPHPQHHDYQQQQQQHQQQQHQQWLRRDQAAAQASGE

GAARTVARLDAVDTSSQDWKAQLNIPAPDTRFRTEDVTATKGNEFEDYFLKRELLMGIYEKGFERPSPIQEESIPIALTG

SDILARAKNGTGKTAAFCIPALEKIDPEKNAIQVVILVPTRELALQTSQVCKELGKYLNIEVMVSTGGTSLKDDIMRLYQ

PVHLLAGTPGRILDLTKKGICMLNECSMLIMDEADKLLAPEFQPSVEQLIRYLPASRQLLLFSATFPVTVKDFKQKYLPR

PYVINLMDELTLKGITQYYAFVEERQKVHCLNTLFSKLQINQSIIFCNSVNRVELLAKKITELGYSCFYIHAKMLQDHRN

RVFHDFRNGACRNLVCTDLFTRGIDIQAVNVVINFDFPKTAETYLHRVGRSGRFGHLGLAVNLITYEDRFNMYRIEQELG

TEIKTIPPQIDLAEYCQ

>KAF5733032.1 DEAD-box ATP-dependent RNA helicase 8 [Tripterygium wilfordii]

MNNNNRGRYPPGIDVGRGGGVNTNSNFQSRTPQQQQQQQYVQRNYVHNHQQFQQQQHNQQQQQHHPHNQQQHYHNQQQLH

HQQQQQWLRRGQLPGADAVVDEVEKTVQSEAVDPSSQDWKASLKIPPADTRYRTEDVTATKGNEFEDYFLKRELLMGIYE

KGFERPSPIQEESIPIALTGSDILARAKNGTGKTAAFCIPALEKIDLENNVIQVVILVPTRELALQTSQVCKELGKHLKI

QVMVTTGGTGLKDDIMRLYQPVHLLVGTPGRILDLAKKGICILKDCSMLVMDEADKLLSPEFQPSIEQLIRFLPASRQIL

MFSATFPVTVKDFKDRYLQKPYVINLMDELTLKGITQFYAFVEERQKVHCLNTLFSKLQINQSIIFCNSVNRVELLAKKI

TELGYSCFYIHAKMLQDHRNRVFHDFRNGACRNLVCTDLFTRGIDIQAVNVVINFDFPKNSETYLHRVGRSGRFGHLGLA

VNLITYEDRFNLYRIEQELGTEIKQIPPHINQEIYCR

>KAA8516023.1 hypothetical protein F0562_019202 [Nyssa sinensis]

MNTRGRYPPGIGGGGRGGNGNANPNFQPRNPHQQYVQRTLQNHQQFQHHQQFQHQHQQQQQQQWLRRNQLGANSSVDEVE

KTVQSEAVDSSSQDWKARLKIPPPDTRYRTEDVTATKGNEFEDYFLKRDLLMGIYEKGFERPSPIQEESIPIALTGSDIL

ARAKNGTGKTAAFCIPALEKIDQDNNVIQVVILVPTRELALQTSQVCKELGKHLQIQIMVTTGGTSLKDDIMRLYQPVHL

LVGTPGRILDLAKKGVCILKDCAMLVMDEADKLLSPEFQPSIEQLIRFLPVNRQILMFSATFPVTVKDFKDRYLHKPYII

NLMDELTLKGITQFYAFVEERQKVHCLNTLFSKLQINQSIIFCNSVNRVELLAKKITELGYSCFYIHAKMLQDHRNRVFH

DFRNGACRNLVCTDLFTRGIDIQAVNVVINFDFPKNAETYLHRVGRSGRFGHLGLAVNLITYEDRFNLYRIEQELGTEIK

QIPPHIDQAIYCR

>VAH05013.1 unnamed protein product [Triticum turgidum subsp. durum]

MHHPRARYPPGYDYGGGGGGRGGNGGGGGGGGGGGGNQNYYGGRNPHPQHHDYQQQQQQQQQQHQQQQHQQWLRRDQAAA

QASGEGAARTVARLDAVDTSSQDWKAQLNIPAPDTRFRTEDVTATKGNEFEDYFLKRELLMGIYEKGFERPSPIQEESIP

IALTGSDILARAKNGTGKTAAFCIPALEKIDPEKNAIQVVILVPTRELALQTSQVCKELGKYLNIEVMVSTGGTSLKDDI

MRLYQPVHLLAGTPGRILDLTKKGICMLNECSMLIMDEADKLLAPEFQPSVEQLIRYLPASRQLLLFSATFPVTVKDFKQ

KYLPRPYVINLMDELTLKGITQYYAFVEERQKVHCLNTLFSKLQINQSIIFCNSVNRVELLAKKITELGYSCFYIHAKML

QDHRNRVFHDFRNGACRNLVCTDLFTRGIDIQAVNVVINFDFPKTAETYLHRVGRSGRFGHLGLAVNLITYEDRFNMYRI

EQELGTEIKTIPPQIDLAEYCQ

>KAB2015652.1 hypothetical protein ES319_D08G040300v1 [Gossypium barbadense]

MNSRGRYPPGIGVGRGGGLNSNPGFQSRPPQQNYVQRNFVQNHHQFHNQHQHQQQQQWLRRNQLPGTNDSSVVDEVEKTI

QSEAFDSSSQDWNARLKMPPPDTRYKTEDVTATKGNEFEDYFLKRELLMGIYEKGFERPSPIQEESIPIALTGSDILARA

KNGTGKTAAFCIPALEKIDQDKNVIQVVILVPTRELALQTSQVCKELGKHLQIQVMVTTGGTSLKDDIMRLYQPVHLLVG

TPGRILDLAKKGVCILKDCSMLIMDEADKLLSPEFQPSIEQLIRFLSANRQILMFSATFPVTVKYFKDRYLQKPYIINLM

DELTLKGITQYYAFVEERQKVHCLNTLFSKLQINQSIIFCNSVNRVELLAKKITELGYSCFYIHAKMLQDHRNRVFHDFR

NGACRNLVCTDLFTRGIDIQAVNVVINFDFPKNSETYLHRVGRSGRFGHLGLAVNLITYEDRFNLYRIEQELGTEIKQIP

PHIDQAIYCR

>EOX96015.1 RNAhelicase-like 8 isoform 1 [Theobroma cacao]

MNSRGRYPPGIGVGRGGGVNANPSFQSRPLQQHYVQRNLVQNHQQFQHHQQQQHHQQQQHQQHQQQQQWLRRNLLPGGND

SSVVDEVEKTVQSEAVDSSSQDWKARLKIPPPDTRYKTEDVTATKGNEFEDYFLKRELLMGIYEKGFERPSPIQEESIPI

ALTGSDILARAKNGTGKTAAFCIPALEKIDQDNNVIQVVILVPTRELALQTSQVCKELGKHLQIQVMVTTGGTSLKDDIM

RLYQPVHLLVGTPGRILDLAKKGVCILKDCSMLIMDEADKLLSPEFQPSVEQLIRFLSANRQILMFSATFPVTVKDFKDR

YLHKPYIINLMDELTLKGITQYYAFVEERQKVHCLNTLFSKLQINQSIIFCNSVNRVELLAKKITELGYSCFYIHAKMLQ

DHRNRVFHDFRNGACRNLVCTDLFTRGIDIQAVNVVINFDFPKNSETYLHRVGRSGRFGHLGLAVNLITYEDRFNLYRIE

QELGTEIKQIPPHIDQAIYCR

>KAF5953321.1 hypothetical protein HYC85_006177 [Camellia sinensis]

MNNTRGRYPPGMGGGGGGRGGGGMHANPNFQPRNFQQQYVQRSPMQNHLQYQNQQHHHHQQQQQQQWLRRNQLGAESSVV

EVEKTVQSEAVDSSSQDWKARLKIPPPDTRYMTEDVTATKGNEFEDYFLKRELLMGIYEKGFERPSPIQEESIPIALTGS

DILARAKNGTGKTAAFCIPALEKIDQDNNVIQVVILVPTRELALQTSQVCKELGKHLQIQVMVTTGGTSLKDDIMRLYQP

VHLLVGTPGRILDLAKKGVCVLKDCAMLVMDEADKLLSPEFQPSVEQLIRFLPANRQILMFSATFPVTVKDFKDRYLQKS

YIINLMDELTLKGITQFYAFVEERQKVHCLNTLFSKLQINQSIIFCNSVNRVELLAKKITELGYSCFYIHAKMLQDHRNR

VFHDFRNGACRNLVCTDLFTRGIDIQAVNVVINFDFPKNAETYLHRVGRSGRFGHLGLAVNLITYEDRFNLYRIEQELGT

EIKQIPPHIDQGLTGIMVLDVVHNSASEGSLWKQDRFVEDFGLVPPSRPTTPTILTNLVLPSKT

>XP_006339542.1 PREDICTED: DEAD-box ATP-dependent RNA helicase 8-like [Solanum tuberosum]

MNPRPRFQSPGMGGRGGGGGGSMHPNTNPNFQNRNPQQYMQRGPAPYQQQQPQQQQQHFQNQQTQQWLRRNQLPSADSTV

AEVEKNVQSEAVDQSSQEWKARLKIPLPDTRYRTEDVTATKGNEFEDYFLKRELLMGIYEKGFERPSPIQEESIPIALTG

SDILARAKNGTGKTASFCIPALEKIDQDKNVIQAIILVPTRELALQTSQVCKELGKHLKIQVMVTTGGTSLKDDIMRLYQ

PVHLLVGTPGRILDLAKKGVCVLKDCSMFIMDEADKLLSPEFQPSIEQLLRFVPANRQILMFSATFPVTVKDFKERYLRK

PYVINLMDELTLKGITQFYAFVEERQKIHCLNTLFSKLQINQSIIFCNSVNRVELLAKKITELGYSCFYIHAKMLQDHRN

RVFHDFRNGACRNLVCTDLFTRGIDIQAVNVVINFDFPKNSETYLHRVGRSGRFGHLGLAVSLITFEDRFNLYRIEQELG

TEIKQIPPQIDQAIYCQ

>XP_023768266.1 DEAD-box ATP-dependent RNA helicase 8-like [Lactuca sativa]

MNNNGRGGGRYPPGIGRGGGGNYQGNTNPSFQQQRNYQQQYAQRNPIQNQQFQQQQQWLRRNSLGNHSSATVEVEKTMQS

EGNDSSSQDWKAQLRLPPADTRYKTEDVTATKGNEFEDYFLKRDLLMGIYEKGFERPSPVQEESIPIALTGSDILARAKN

GTGKTAAFCIPALEKIDQDKNVIQVVILVPTRELALQTSQVCKELGKHLNIQVMVTTGGTSLKDDIMRLYQPVHLLVGTP

GRILDLTKKGICILKDCAMLVMDEADKLLSQEFQPSIEQLIQFMAPNRQILMFSATFPVTVKDFRDRYLRNPYVINLMDE

LTLKGITQFYAFVEERQKVHCLNTLFSKLQINQSIIFCNSVNRVELLAKKITELGYSCFYIHAKMLQDHRNRVFHDFRNG

ACRNLVCTDLFTRGIDIQAVNVVINFDFPRNSETYLHRVGRSGRFGHLGLAVNLITYEDRFNLYRIEQELGTEIKQIPPF

IDQAIYCR

>XP_006341720.1 PREDICTED: DEAD-box ATP-dependent RNA helicase 8-like [Solanum tuberosum]

MNPRGRYPPPGMGGGGGRGGGHMYPNANPNFQPRNPQQYVQRGPVNQQQLFQNQQTQQWLRRNQLASDSTVDEVEKTVQS

EAVDQSSQDWKARLKIPPADTRYRTEDVTATKGNEFEDYFLKRELLMGIYEKGFERPSPIQEESIPIALTGSDILARAKN

GTGKTAAFCIPALEKIDQDVNAIQVVILVPTRELALQTSQVCKELGKHLKIEVMVTTGGTSLKDDIMRLYQPVHLLVGTP

GRILDLARKGICVLKDCSMLVMDEADKLLSPEFQPSIVQLIRFLPANRQVLMFSATFPVTVKDFKERYLQKPYVINLMDE

LTLKGITQFYAFVEERQKLHCLNTLFSKLQINQSIIFCNSVNRVELLAKKITELGFSCFYIHAKMLQDHRNRVFHDFRNG

ACRNLVCTDLFTRGIDIQAVNVVINFDFPKNSETYLHRVGRSGRFGHLGLAVNLITYEDRFNLYRIEQELGTEIKQIPPH

IDQAIYCL

>NP_001140760.1 putative DEAD-box ATP-dependent RNA helicase family protein [Zea mays]

MHQPRARYPPGYGSGGGGGAGRGGGGGNGGGGGNHNYYGRNPQQHHYHQQQQSQHSHRNSSYQQQWLRRDQAPAVAGAAS

GNAVAKTASQLDAVGSSSQDWKAQLNIPAPDTRYRTEDVTATKGNEFEDYFLKRELLMGIYEKGFERPSPIQEESIPIAL

TGSDILARAKNGTGKTAAFCIPALEKIDPEKNAIQVVILVPTRELALQTSQVCKELGKYLNIQVMVSTGGTSLKDDIMRL

YQPVHLLVGTPGRILDLTRKGICVLKDCSMLVMDEADKLLAPEFQPSVEALIHFLPPSRQLLMFSATFPVTVKEFKDKYL

PRPYVINLMDELTLKGITQYYAFVEERQKVHCLNTLFSKLQINQSIIFCNSVNRVELLAKKITELGYSCFYIHAKMLQDH

RNRVFHDFRNGACRNLVCTDLFTRGIDIQAVNVVINFDFPKTSETYLHRVGRSGRYGHLGLAVNLITYEDRFNLYRIEQE

LGTEIKTIPPQIDLSIYCQ

>KAE8721293.1 DEAD-box ATP-dependent RNA helicase 6 [Hibiscus syriacus]

MNNGGRYPPGIGVGRGGGVNANPSFRSRPSQQQYVQRNLVQNHQHFQQQQHQQQQLWLRRNQLPGGNDSNVVDEVEKTVQ

SGAVDSSSQDWKARLTLPPPDTRYKTEDVTATKGNEFEDYFLKRELLMGIYEKGFERPSPIQEESIPIALTGSDILARAK

NGTGKTAAFCIPALEKIDQDNNVIQVVILVPTRELALQTSQVCKELGKHLQIHVMVTTGGTSLKDDIMRLYQPVHFLVGT

PGRILDLAKKGVCILKDCSMLIMDEADKLLSPEFQPSIEQLIRFLPANRQILMFSATFPVTVKDFKDRYLKKPYIINLMD

ELTLKGITQYYAFVEERQKVHCLNTLFSKLQINQSIIFCNSVNRVELLAKKITELGYSCFYIHAKMLQDHRNRVFHDFRN

GACRNLVCTDLFTRGIDIQAVNVVINFDFPKNSETYLHRVGRSGRFGHLGLAVNLITYEDRFNLYRIEQELGTEIKQIPP

HIDQAIYCR

>PHT74497.1 DEAD-box ATP-dependent RNA helicase 6 [Capsicum annuum]

MHPNSNQNFQYRNPQQYMQRGPTPYQQQQQQQHFQNQLTQQQWLRRNQLPSTDSPVEEVEKTVQSEAVDQSSQDWKAGLK

TPLPDTRYKTEDVTATKGNEFEDYFLKRELLMGIYEKGFERPSPIQEESIPIALTGSDILARAKNGTGKTAAFCIPALEK

IDQDKNAIQVVILVPTRELALQTSQVCKELGKHLKIEVMVTTGGTSLKDDIMRLYQPVHLLVGTPGRILDLAKKGVCILK

DCSMLVMDEADKLLSPEFQPSIEQLIHFLPANRQILMFSATFPVTVKDFRDRHLRKPYVINLMDELTLKGITQFYAFVEE

RQKVHCLNTLFSKLQINQSIIFCNSVNRVELLAKKITELGFSCFYIHAKMLQDHRNRVFHDFRNGACRNLVCTDLFTRGI

DIQAVNVVINFDFPKNSETYLHRVGRSGRFGHLGLAVSLVTYEDRFNLYRIEQELGTEIKQIPPHIDQAIYCQ

>XP_004229903.1 DEAD-box ATP-dependent RNA helicase 8 [Solanum lycopersicum]

MNPRPRFQSPGMGGRGGGGGGSMHPNTNPNFQHRNPQQYIQRGPAPYQQPQPQQQQQQHFQNQQTQQWLRRNQLPSPDST

VAEVEKNVQSEAVDQSSQEWKARLKIPQPDTRYRTEDVTATKGNEFEDYFLKRELLMGIYEKGFERPSPIQEESIPIALT

GSDILARAKNGTGKTASFCIPALEKIDQDKNVIQAIILVPTRELALQTSQVCKELGKHLKIQVMVTTGGTSLKDDIMRLY

QPVHLLVGTPGRILDLAKKGVCVLKDCSMFIMDEADKLLSPEFQPSIEQLICFLPANRQILMFSATFPVTVKDFKERYLH

KPYVINLMDELTLKGITQFYAFVEERQKIHCLNTLFSKLQINQSIIFCNSVNRVELLAKKITELGYSCFYIHAKMLQDHR

NRVFHDFRNGACRNLVCTDLFTRGIDIQAVNVVINFDFPKNSETYLHRVGRSGRFGHLGLAVSLITFEDRFNLYRIEQEL

GTEIKQIPPQIDQAIYCQ

>TMX01555.1 hypothetical protein EJD97_024286 [Solanum chilense]

MNSRPRFQSPGMGGRGGGGGGSMHPNTNPNFQHRNPQQYIQRGPAPYQQPQLQQQQQQHFQNQQTQQWLRRNQFPSPDST

VAEVEKNVQSEAVDQSSQEWKARLKIPQPDTRYRTEDVTATKGNEFEDYFLKRELLMGIYEKGFERPSPIQEESIPIALT

GSDILARAKNGTGKTASFCIPALEKIDQDKNVIQAIILVPTRELALQTSQVCKELGKHLKIQVMVTTGGTSLKDDIMRLY

QPVHLLVGTPGRILDLAKKGVCVLKDCSMFIMDEADKLLSPEFQPSIEQLICFLPANRQILMFSATFPVTVKDFKERYLH

KPYVINLMDELTLKGITQFYAFVEERQKIHCLNTLFSKLQINQSIIFCNSVNRVELLAKKITELGYSCFYIHAKMLQDHR

NRVFHDFRNGACRNLVCTDLFTRGIDIQAVNVVINFDFPKNSETYLHRVGRSGRFGHLGLAVSLITFEDRFNLYRIEQEL

GTEIKQIPPQIDQAIYCQ

>KAB8113236.1 hypothetical protein EE612_052179, partial [Oryza sativa]

GGGGGGGGGGGGGRGNGGGGFGGGGGGGGGNHGYYGRGPQPQPQQQHYHHQAQQLHQHQQQQQHAQRNSSSQQQQWLRRD

QATAAAASGEVAARTAAQLEAVDSSSEDWKAQLNLPAPDTRYRTEDVTATKGNEFEDYFLKRELLMGIYEKGFERPSPIQ

EESIPIALTGSDILARAKNGTGKTAAFCIPALEKIDPEKNAIQVVILVPTRELALQTSQVCKELGKYLNIQVMVSTGGTS

LKDDIMRLYQPVHLLVGTPGRILDLTRKGICVLKDCSMLVMDEADKLLAPEFQPSIEQLIHFLPANRQLLMFSATFPVTV

KDFKEKYLPRPYVINLMDELTLKGITQYYAFVEERQKVHCLNTLFSKLQINQSIIFCNSVNRVELLAKKITELGYSCFYI

HAKMLQDHRNRVFHDFRNGACRNLVCTDLFTRGIDIQAVNVVINFDFPKTSETYLHRVGRSGRFGHLGLAVNLITYEDRF

NLYRIEQELGTEIKTIPPQIDLAVYCQ

>KAF3675829.1 DEAD-box ATP-dependent RNA helicase 6 [Capsicum annuum]

MGGRGGGGSMHPNSNQNFQYRNPQQYMQRGPTPYQQQQQQQHFQNQLTQQQWLRRNQLPSTDSPVEEVEKTVQSEAVDQS

SQDWKAGLKTPLPDTRYKTEDVTATKGNEFEDYFLKRELLMGIYEKGFERPSPIQEESIPIALTGSDILARAKNGTGKTA

AFCIPALEKIDQDKNAIQVVILVPTRELALQTSQVCKELGKHLKIEVMVTTGGTSLKDDIMRLYQPVHLLVGTPGRILDL

AKKGVCILKDCSMLVMDEADKLLSPEFQPSIEQLIHFLPANRQILMFSATFPVTVKDFRDRHLRKPYVINLMDELTLKGI

TQFYAFVEERQKVHCLNTLFSKLQINQSIIFCNSVNRVELLAKKITELGFSCFYIHAKMLQDHRNRVFHDFRNGACRNLV

CTDLFTRGIDIQAVNVVINFDFPKNSETYLHRVGRSGRFGHLGLAVSLVTYEDRFNLYRIEQELGTEIKQIPPHIDQAIY

CQ

>GFP93013.1 dead-box ATP-dependent RNA helicase 6 [Phtheirospermum japonicum]

MNSRPRYPPPGMGGGRGGGGGGGMNPNAGPNPTYQPRNANQQYVQRGPAQNNQNHQLFQNPQPQQWLRRNQLPPADSSVE

EVEKTVQSEAVDSSSQDWKGQLKLPPADTRYKTEDVTATKGNEFEDYFLKRELLMGIYEKGFERPSPIQEESIPIALTGS

DILARAKNGTGKTAAFCIPALEKIDQDKNIIQVVILVPTRELALQTSQVCKELGKHLKIQVMVTTGGTSLKDDIMRLHQP

VHLLVGTPGRILDLSKKGLCVLNECTMVVMDEADKLLSPEFQPSIEQLIGFTSANRQILMFSATFPVTVKDFKDRYLRKP

YIINLMDELTLKGITQFYAFVEERQKVHCLNTLFSKLQINQSIIFCNSVNRVELLAKKITELGYSCFYIHAKMLQDHRNR

VFHDFRNGACRNLVCTDLFTRGIDIQAVNVVINFDFPKNSETYLHRVGRSGRFGHLGLAVNLITYEDRFNLYRIEQELGT

EIKQIPPHIDQAIYCV

>CAB4089923.1 unnamed protein product [Lactuca saligna]

MNNYGRGGGRYPPGIGRGGGGNYQGNTNPSFQQQRNYQQQYAQRNPIQNQQFQQQQQQQQQWLRRNSLGNHSSATVEVEK

TMQSEGNDSSSQDWKAQLRLPPADTRYKTEDVTATKGNEFEDYFLKRDLLMGIYEKGFERPSPVQEESIPIALTGSDILA

RAKNGTGKTAAFCIPALEKIDQDKNVIQVVILVPTRELALQTSQVCKELGKHLNIQVMVTTGGTSLKDDIMRLYQPVHLL

VGTPGRILDLTKKGICILKDCAMLVMDEADKLLSQEFQPSIEQLIQFMAPNRQILMFSATFPVTVKDFRDRYLRNPYVIN

LMDELTLKGITQFYAFVEERQKVHCLNTLFSKLQINQSIIFCNSVNRVELLAKKITELGYSCFYIHAKMLQDHRNRVFHD

FRNGACRNLVCTDLFTRGIDIQAVNVVINFDFPRNSETYLHRVGRSGRFGHLGLAVNLITYEDRFNLYRIEQELGTEIKQ

IPPFIDQAIYCR

>PHU09617.1 DEAD-box ATP-dependent RNA helicase 8 [Capsicum chinense]

MNQRPRFQPPGMGGRGGGGSMHPNSNQNFQYRNPQQYMQRGPTPYQQQQQQHFQNQLTQQQWLRRNQLPSTDSPVEEVEK

TVQSEAVDQSSQDWKAGLKTPLPDTRYKTEDVTATKGNEFEDYFLKRELLMGIYEKGFERPSPIQEESIPIALTGSDILA

RAKNGTGKTAAFCIPALEKIDQDKNAIQVVILVPTRELALQTSQVCKELGKHLKIEVMVTTGGTSLKDDIMRLYQPVHLL

VGTPGRILDLAKKGVCILKDCSMLVMDEADKLLSPEFQPSIEQLIHFLPANRQILMFSATFPVTVKDFRDRHLRKPYVIN

LMDELTLKGITQFYAFVEERQKVHCLNTLFSKLQINQSIIFCNSVNRVELLAKKITELGFSCFYIHAKMLQDHRNRVFHD

FRNGACRNLVCTDLFTRGIDIQAVNVVINFDFPKNSETYLHRVGRSGRFGHLGLAVSLVTYEDRFNLYRIEQELGTEIKQ

IPPHIDQAIYCQ

>XP_022940370.1 DEAD-box ATP-dependent RNA helicase 8-like [Cucurbita moschata]

MNNRGRYPPGIGAGRGGGVNANPSFQSRPHQQQYVQRNLVPNQQYQQQQHQNQHQHQHQHQNQQLQQQQQWLKRNQLGGG

PADSNVDEVEKTVQSEAVDSSSQDWKARLKIPPPDTRYKTEDVTATKGNEFEDYFLKRELLMGIYEKGFERPSPIQEESI

PIALTGSDILARAKNGTGKTAAFCIPALEKIDQDNNVIQVVILVPTRELALQTSQVCKELGKNLNIQVMVTTGGTSLKDD

IMRLYQPVHLLVGTPGRILDLAKKGVCVLKDCSMLIMDEADKLLSPEFQPSIEQLIRFLPANRQVLMYSATFPVTVKDFK

DRYLHRPYIINLMDELTLKGITQFYAFVEERQKVHCLNTLFSKLQINQSIIFCNSVNRVELLAKKITELGYSCFYIHAKM

LQDHRNRVFHDFRNGACRNLVCTDLFTRGIDIQAVNVVINFDFPKNSETYLHRVGRSGRFGHLGLAVNLITYEDRFNLYR

IEQELGTEIKQIPPHIDQAIYCR

>PHT40569.1 DEAD-box ATP-dependent RNA helicase 8 [Capsicum baccatum]

MNQRPRFQPPGMGGRGGGGSMHPNSNQNFQYRNPQQYMQRGPTPYQQQQQQQHFQNQLTQQQWLRRNQLPSTDSPVEEVE

KTVQSEAVDQSSQDWKAGLKTPLPDTRYKTEDVTATKGNEFEDYFLKRELLMGIYEKGFERPSPIQEESIPIALTGSDIL

ARAKNGTGKTAAFCIPALEKIDQDKNAIQVVILVPTRELALQTSQVCKELGKHLKIEVMVTTGGTSLKDDIMRLYQPVHL

LVGTPGRILDLAKKGVCILKDCSMLVMDEADKLLSPEFQPSIEQLIHFLPANRQILMFSATFPVTVKDFRDRHLRKPYVI

NLMDELTLKGITQFYAFVEERQKVHCLNTLFSKLQINQSIIFCNSVNRVELLAKKITELGFSCFYIHAKMLQDHRNRVFH

DFRNGACRNLVCTDLFTRGIDIQAVNVVINFDFPKNSETYLHRVGRSGRFGHLGLAVSLVTYEDRFNLYRIEQELGTEIK

QIPPHIDQAIYCQ

>XP_016722208.1 PREDICTED: DEAD-box ATP-dependent RNA helicase 8-like [Gossypium hirsutum]

MNNRGRYPSVLGRGRGANANPSFQSRPEQPQYAQRNLVQNHHHFQQQQHHHLQQQQHQHQQQWLRRNQLPGGNDSTVADE

VEKTVQSEAVDSSSQDWKARLKLPPSDTRYKTEDVTATKGNEFEDYFLKRELLMGIYEKGFERPSPIQEESIPIALTGSD

ILARAKNGTGKTAAFCILALEKIDQDNNVIQVVILLPTRELALQTSQVCKELGKHLQIQVMVTTGGTSLKDDIMRLYQPV

HLLVGTPGRILDLAKKGVCILKNCSMLIMDEADKLLSPEFQPSLEQLLRFLPPKRQILMFSATFPVTVKDFKDRYLKKPY

IINLMDELTLKGITQYYAFVEERQKVHCLNTLFSKLQINQSIIFCNSVNRVELLAKKITELGYSCFYIHAKMLQDHRNRV

FHDFRNGACRNLVCTDLFTRGIDIQAVNVVINFDFPKNSETYLHRVGRSGRFGHLGLAVNLITYEDRFNLYRIEQELGTE

IKQIPPHIDQAIYCR

>XP_010234885.1 DEAD-box ATP-dependent RNA helicase 12 [Brachypodium distachyon]

MHHPRARYPPGYDYGGGGGGGGRGGGGGGERGGGSGGNQNYYGRNPQPQHHDYHQPQHAQRNSFSHQQQHQQHQHAQKNS

FSQQQHQQQQQQWLRRDQAAAQASGEAAARTAARLDAVDTSSQDWKAQLNIPAPDTRYRTEDVTATKGNEFEDYFLKREL

LMGIYEKGFERPSPIQEESIPIALTGSDILARAKNGTGKTAAFCIPALEKIDPEKNAIQVVILVPTRELALQTSQVCKEL

GKYLNIEVMVSTGGTSLKDDIMRLYQPVHLLAGTPGRILDLTRKGICMLNECSMLIMDEADKLLAPEFQPSVEQLISFLP

ASRQLLLFSATFPVTVKDFKQKYLPRPYVINLMDELTLKGITQYYAFVEERQKVHCLNTLFSKLQINQSIIFCNSVNRVE

LLAKKITELGYSCFYIHAKMLQDHRNRVFHDFRNGACRNLVCTDLFTRGIDIQAVNVVINFDFPKTAETYLHRVGRSGRF

GHLGLAVNLITYEDRFNMYRIEQELGTEIKTIPPQIDLAEYCQ

>XP_021817040.1 DEAD-box ATP-dependent RNA helicase 8 [Prunus avium]

MNSNRGRYPPGIGAGRGGGMNANPAFQSRPPHQQQYVQRNLLPNHHHQQYFQQQQHQQQQQQQQQQWLRRGQLGGSTSAD

SAVDEVEKTVQSEAVDPSSHDWKARLNIPAPDTRFRTEDVTATKGNEFEDYFLKRELLMGIYEKGFERPSPIQEESIPIA

LTGSDILARAKNGTGKTAAFCIPALEKIDQDNNVIQVVILVPTRELALQTSQVCKELGKHLQIQVMVTTGGTSLKDDIMR

LYQPVHLLVGTPGRILDLAKKGVCILKDCSMLVMDEADKLLSPEFQPSVEQLIRFLPSHRQILMFSATFPVTVKDFKDRY

LQKPYVINLMDELTLKGITQFYAFVEERQKVHCLNTLFSKLQINQSIIFCNSVNRVELLAKKITELGYSCFYIHAKMLQD

HRNRVFHDFRNGACRNLVCTDLFTRGIDIQAVNVVINFDFPKNSETYLHRVGRSGRFGHLGLAVNLITYEDRFNLYRIEQ

ELGTEIKQIPPHIDQAIYCR

>XP_034571600.1 DEAD-box ATP-dependent RNA helicase 12 [Setaria viridis]

MHQPRARYPPGYGSGGGGRGGGGGNGGGGGGGGGGGNHNYYGRNPQPQPQHHHHYQHQQPPQQQQQQQQLAHRNSSHLHQ

QWLRRDQAPASAAGPGDAAGRTASHLDVDSSSQDWKAQLNIPAPDTRYRTEDVTATKGNEFEDYFLKRELLMGIYEKGFE

RPSPIQEESIPIALTGSDILARAKNGTGKTAAFCIPALEKIDPEKNAIQVVILVPTRELALQTSQVCKELGKYLNIQVMV

STGGTSLKDDIMRLYQPVHLLVGTPGRILDLTRKGICVLKDCSMLVMDEADKLLAPEFQPSVEALIHFLPPSRQLLMFSA

TFPVTVKEFKEKYLPKPYVINLMDELTLKGITQYYAFVEERQKVHCLNTLFSKLQINQSIIFCNSVNRVELLAKKITELG

YSCFYIHAKMLQDHRNRVFHDFRNGACRNLVCTDLFTRGIDIQAVNVVINFDFPKTSETYLHRVGRSGRYGHLGLAVNLI

TYEDRFNLYRIEQELGTEIKTIPPQIDLAVYCQ

>KMZ67639.1 DEAD-box ATP-dependent RNA helicase 8 [Zostera marina]

MNSRDRYPPGMGNGGRNPNNNYYGRSTHQQQQQSQQQYVQRNAVQNQQSHQNQTYHRQMIMQQQQQQWTRRNQMESESSG

NDSTKVTQTASVVDPSSQDWKSQLTIPSTDTRYRTEDVTATKGNEFEDYFLKRELLMGIYEKGFERPSPIQEESIPIALT

GSDILARAKNGTGKTAAFCIPILEKINQDKNVIQAVILVPTRELALQTSQVCKELGKHLKIQVMVTTGGTSLKDDIMRLY

QPVHLLVGTPGRILDLTKKGICVLKDCAMLAMDEADKLLSPEFQPSVGHLIRFMPPTRQILLFSATFPVTVKDFKDRYLS

KAYIINLMDELTLKGITQYYAFVEERQKVHCLNTLFSKLQINQSIIFCNSVNRVELLAKKITELGYSCFYIHAKMLQDHR

NRVFHDFRNGACRNLVCTDLFTRGIDIQAVNVVINFDFPKNSETYLHRVGRSGRFGHLGLAVNLITYEDRYNLYRMEQEL

GTEIKQIPSQIDQAIYCR

>XP_007220665.1 DEAD-box ATP-dependent RNA helicase 8 [Prunus persica]

MNSNRGRYPPGIGAGRGGGMNANPAFQSRPPHQQQYVQRNLLPNHHHQQYFQQQQHQQQQQQQQQWLRRGQLGGSTSADS

AVDEVEKTVQSEAVDPSSHDWKARLNIPAPDTRFRTEDVTATKGNEFEDYFLKRELLMGIYEKGFERPSPIQEESIPIAL

TGSDILARAKNGTGKTAAFCIPALEKIDQDNNVIQVVILVPTRELALQTSQVCKELGKHLQIQVMVTTGGTSLKDDIMRL

YQPVHLLVGTPGRILDLAKKGVCILKDCSMLVMDEADKLLSPEFQPSVEQLIRFLPSHRQILMFSATFPVTVKDFKDRYL

QKPYVINLMDELTLKGITQFYAFVEERQKVHCLNTLFSKLQINQSIIFCNSVNRVELLAKKITELGYSCFYIHAKMLQDH

RNRVFHDFRNGACRNLVCTDLFTRGIDIQAVNVVINFDFPKNSETYLHRVGRSGRFGHLGLAVNLITYEDRFNLYRIEQE

LGTEIKQIPPHIDQAIYCR

>XP_016538043.1 PREDICTED: DEAD-box ATP-dependent RNA helicase 8 [Capsicum annuum]

MNQRPRFQPPGMGGRGGGGSMHPNSNQNFQYRNPQQYMQRGPTPYQQQQQQQHFQNQLTQQQWLRRNQLPSTDSPVEEVE

KTVQSEAVDQSSQDWKAGLKTPLPDTRYKTEDVTATKGNEFEDYFLKRELLMGIYEKGFERPSPIQEESIPIALTGSDIL

ARAKNGTGKTAAFWIPALEKIDQDKNAIQVVILVPTRELALQTSQVCKELGKHLKIEVMVTTGGTSLKDDIMRLYQPVHL

LVGTPGRILDLAKKGVCILKDCSMLVMDEADKLLSPEFQPSIEQLIHFLPANRQILMFSATFPVTVKDFRDRHLRKPYVI

NLMDELTLKGITQFYAFVEERQKVHCLNTLFSKLQINQSIIFCNSVNRVELLAKKITELGFSCFYIHAKMLQDHRNRVFH

DFRNGACRNLVCTDLFTRGIDIQAVNVVINFDFPKNSETYLHRVGRSGRFGHLGLAVSLVTYEDRFNLYRIEQELGTEIK

QIPPHIDQAIYCQ

>XP_022974606.1 DEAD-box ATP-dependent RNA helicase 8-like [Cucurbita maxima]

MNNRGRYPPGIGAGRGGGVNANPSFQSRPHQQQYVQRNLVPNQQYQQQQHQNQNQNQNQHQHQHQHQNQQLQQQQWLKRN

QLGGGPADSNVDEVEKTVQTEAVDSSSQDWKARLKIPPPDTRYKTEDVTATKGNEFEDYFLKRELLMGIYEKGFERPSPI

QEESIPIALTGSDILARAKNGTGKTAAFCIPALEKIDQDNNVIQVVILVPTRELALQTSQVCKELGKNLNIQVMVTTGGT

SLKDDIMRLYQPVHLLVGTPGRILDLAKKGVCVLKDCSMLIMDEADKLLSPEFQPSIEQLIRFLPANRQVLMYSATFPVT

VKDFKDRYLHRPYIINLMDELTLKGITQFYAFVEERQKVHCLNTLFSKLQINQSIIFCNSVNRVELLAKKITELGYSCFY

IHAKMLQDHRNRVFHDFRNGACRNLVCTDLFTRGIDIQAVNVVINFDFPKNSETYLHRVGRSGRFGHLGLAVNLITYEDR

FNLYRIEQELGTEIKQIPPHIDQAIYCR

>RLN18715.1 DEAD-box ATP-dependent RNA helicase 12 [Panicum miliaceum]

MHQPRARYPPGYGSGGGGRGGGGGNGGGGGGNHNYYGRNPQPQPHNHQHYQHQQPPPQQQPQQHAHRNSSHQHQQWLRRE

QGPASAAGSGDAAGRTAAQLDAVDSSSQDWKAQLNIPAPDTRYRTEDVTATKGNEFEDYFLKRELLMGIYEKGFERPSPI

QEESIPIALTGSDILARAKNGTGKTAAFCIPALEKIDPEKNAIQVVILVPTRELALQTSQVCKELGKYLNIQVMVSTGGT

SLKDDIMRLYQPVHLLVGTPGRILDLTRKGICVLKDCSMLVMDEADKLLAPEFQPSVEALIHFLPPSRQLLMFSATFPVT

VKEFKEKYLPKPYVINLMDELTLKGITQYYAFVEERQKVHCLNTLFSKLQINQSIIFCNSVNRVELLAKKITELGYSCFY

IHAKMLQDHRNRVFHDFRNGACRNLVCTDLFTRGIDIQAVNVVINFDFPKTSETYLHRVGRSGRYGHLGLAVNLITYEDR

FNLYRIEQELGTEIKTIPPQIDLAVYCQ

>CAB4269944.1 unnamed protein product [Prunus armeniaca]

MNSNRGRYPPGIGAGRGGGMNANPAFQSRPPHQQQYVQRNLLPNHHHQQYFQQQQHQQQQQQQQQQQWLRRGQLGGSTSA

DSAVDEVEKTVQSEAVDPSSHDWKARLNIPAPDTRFRTEDVTATKGNEFEDYFLKRELLMGIYEKGFERPSPIQEESIPI

ALTGSDILARAKNGTGKTAAFCIPALEKIDQDNNVIQVVILVPTRELALQTSQVCKELGKHLQIQVMVTTGGTSLKDDIM

RLYQPVHLLVGTPGRILDLSKKGVCILKDCSMLVMDEADKLLSPEFQPSVEQLIRFLPSHRQILMFSATFPVTVKDFKDR

YLQKPYVINLMDELTLKGITQFYAFVEERQKVHCLNTLFSKLQINQSIIFCNSVNRVELLAKKITELGYSCFYIHAKMLQ

DHRNRVFHDFRNGACRNLVCTDLFTRGIDIQAVNVVINFDFPKNSETYLHRVGRSGRFGHLGLAVNLITYEDRFNLYRIE

QELGTEIKQIPPHIDQAIYCRGSGWGRMVDLVHVDIPVYQLLRVSIGNS

>XP_024996579.1 DEAD-box ATP-dependent RNA helicase 8-like [Cynara cardunculus var. scolymus]

MNNNARGGGRYPPGIGRGGGGGNYHGNANPSFQQQRNYQQQNAQRNPMHQQQFQQQQQWLRRNTLGNHPSTTVEVEKNMQ

SEANDSSSRDWKAQLRLPPADTRYRTEDVTATKGNEFEDYFLKRDLLMGIYEKGFERPSPVQEESIPIALTGSDILARAK

NGTGKTAAFCIPALEKIDQDNNVIQVVILVPTRELALQTSQVCKELGKHLNIQVMVTTGGTSLKDDIMRLYQPVHLLVGT

PGRILDLARKGICILKDCAMLVMDEADKLLSPEFQPSVEQLIHFMPANRQILMFSATFPVTVKDFKERYLHKPYIINLMD

ELTLKGITQFYAFVEERQKVHCLNTLFSKLQINQSIIFCNSVNRVELLAKKITELGYSCFYIHAKMLQDHRNRVFHDFRN

GACRNLVCTDLFTRGIDIQAVNVVINFDFPKNSETYLHRVGRSGRFGHLGLAVNLITYEDRFNLYRIEQELGTEIKQIPP

FIDQAIYCR

>XP_025796722.1 DEAD-box ATP-dependent RNA helicase 12 [Panicum hallii]

MHQPRARYPPGYGSGGGGRGGGGGNGGGGGGNHNYYGRNPQPQPHHHQHYHHQQPPPQQQPQQHAHRNSSHQHQQWLRRD

QGHASAAGSGDAAGRTAAQLDAVDSSSQDWKAQLNIPAPDTRYRTEDVTATKGNEFEDYFLKRELLMGIYEKGFERPSPI

QEESIPIALTGSDILARAKNGTGKTAAFCIPALEKIDPEKNAIQVVILVPTRELALQTSQVCKELGKYLNIQVMVSTGGT

SLKDDIMRLYQPVHLLVGTPGRILDLTRKGICVLKDCSMLVMDEADKLLAPEFQPSVEALIHFLPPSRQLLMFSATFPVT

VKEFKEKYLPKPYVINLMDELTLKGITQYYAFVEERQKVHCLNTLFSKLQINQSIIFCNSVNRVELLAKKITELGYSCFY

IHAKMLQDHRNRVFHDFRNGACRNLVCTDLFTRGIDIQAVNVVINFDFPKTSETYLHRVGRSGRYGHLGLAVNLITYEDR

FNLYRIEQELGTEIKTIPPQIDLAVYCQ

>XP_022975246.1 DEAD-box ATP-dependent RNA helicase 8-like [Cucurbita maxima]

MVNVFEFLFLVSSICLSASVLDFGKLYCLAQIETDYYHLCIFFICSSQDWKARLKIPPPDTRYKTEDVTATKGNEFEDYF

LKRELLMGIYEKGFERPSPIQEESIPIALTGSDILARAKNGTGKTAAFCIPALEKIDQDNNVIQVVILVPTRELALQTSQ

VCKELGKNLNIQVMVTTGGTSLKDDIMRLYQPVHLLVGTPGRILDLAKKGVCVLKDCSMLIMDEADKLLSPEFQPSIEQL

IRFLPANRQVLMYSATFPVTVKDFKDRYLHRPYIINLMDELTLKGITQFYAFVEERQKVHCLNTLFSKLQINQSIIFCNS

VNRVELLAKKITELGYSCFYIHAKMLQDHRNRVFHDFRNGACRNLVCTDLFTRGIDIQAVNVVINFDFPKNSETYLHRVG

RSGRFGHLGLAVNLITYEDRFNLYRIEQELGTEIKQIPPHIDQAIYCR

>RLN42473.1 DEAD-box ATP-dependent RNA helicase 12 [Panicum miliaceum]

MHQPRARYPPGYGSGGGGGNGGGGGGNHNYYGRNPQPQPQPHHHQHYQHQQPPPQQQPQQHAHRNSSHQHQQWLRRDQGP

ASAAGSGDAAGRTAAQLDAVDSSSQDWKAQLNIPAPDTRYRTEDVTATKGNEFEDYFLKRELLMGIYEKGFERPSPIQEE

SIPIALTGSDILARAKNGTGKTAAFCIPALEKIDPEKNAIQVVILVPTRELALQTSQVCKELGKYLNIQVMVSTGGTSLK

DDIMRLYQPVHLLVGTPGRILDLTRKGICVLKDCSMLVMDEADKLLAPEFQPSVESLIHFLPPSRQLLMFSATFPVTVKE

FKEKYLPKPYVINLMDELTLKGITQYYAFVEERQKVHCLNTLFSKLQINQSIIFCNSVNRVELLAKKITELGYSCFYIHA

KMLQDHRNRVFHDFRNGACRNLVCTDLFTRGIDIQAVNVVINFDFPKTSETYLHRVGRSGRYGHLGLAVNLITYEDRFNL

YRIEQELGTEIKTIPPQIDLAVYCQ

>CAB4086296.1 unnamed protein product [Lactuca saligna]

MDNSSSNNRRYPPGIGNGRGSGGVYDGGGGGSYHPNPNYYHQPRNPTHQNQFQQQRQQPQYTQRQQTGQQNNTQQQWLRR

NPNANAPDSSAANVNNEVEKSVYSDVSVDSSSQEWKAQLNIPAADTRYRTEDVTATKGNEFEDYFLKRELLMGIYEKGFE

RPSPIQEESIPIALTGSDILARAKNGTGKTAAFCIPALEKIDTDKNNIQVVILVPTRELALQTSQVCKELGKHLQIQVMV

TTGGTSLKDDIMRLYQPVHLLVGTPGRILDLTKKGICNLTECAMLVMDEADKLLSPEFQPSVEQLIRFLPEPRQILMFSA

TFPVTVKDFRDRYLKNPYVVNLMDELTLKGITQFYAFVEERQKVHCLNTLFSKLQINQSIIFCNSVNRVELLAKKITELG

YSCFYIHAKMLQDHRNRVFHDFRNGACRNLVCTDLFTRGIDIQAVNVVINFDFPRNAETYLHRVGRSGRFGHLGLAVNLI

TFEDRFNLYRIEQELGTEIKQIPPLIDQAIYCR

>CAB4300333.1 unnamed protein product [Prunus armeniaca]

MNSNRGRYPPGIGAGRGGGMNANPAFQSRPPHQQQYVQRNLLPNHHHQQYFQQQQHQQQQQQQQQQWLRRGQLGGSTSAD

SAVDEVEKTVQSEAVDPSSHDWKARLNIPAPDTRFRTEDVTATKGNEFEDYFLKRELLMGIYEKGFERPSPIQEESIPIA

LTGSDILARAKNGTGKTAAFCIPALEKIDQDNNVIQVVILVPTRELALQTSQVCKELGKHLQIQVMVTTGGTSLKDDIMR

LYQPVHLLVGTPGRILDLSKKGVCILKDCSMLVMDEADKLLSPEFQPSVEQLIRFLPSHRQILMFSATFPVTVKDFKDRY

LQKPYVINLMDELTLKGITQFYAFVEERQKVHCLNTLFSKLQINQSIIFCNSVNRVELLAKKITELGYSCFYIHAKMLQD

HRNRVFHDFRNGACRNLVCTDLFTRGIDIQAVNVVINFDFPKNSETYLHRVGRSGRFGHLGLAVNLITYEDRFNLYRIEQ

ELGTEIKQIPPHIDQAIYCRGSGWGRMVDLVHVDIPVYQLLRVSIGNS

>XP_020082028.1 DEAD-box ATP-dependent RNA helicase 8-like [Ananas comosus]

MNPRGRYPPGYGNAGGGNTGGNPNYYARNPHPQQQYVPRNYAQSQQQQYVPRNYVSNRNQMGSDSGPSEVAKAVQPDGID

SSSQDWKAQLRIPPQDTRYKTEDVTATKGNEFEDYFLKRELLMGIYEKGFERPSPIQEESIPIALTGSDILARAKNGTGK

TAAFCIPALEKIDQDNNVIQVVILVPTRELALQTSQVCKELGKHLKIQIMVTTGGTSLKDDIMRLYQPVHLLVGTPGRIL

DLARKGVCILKDCSMLIMDEADKLLTPEFQPSVEQLIRFLPSNRQILMFSATFPVTVKDFKDNYLPKPYIINPMDELTLK

GITQFYAFVEERQKVHCLNTLFSKLQINQSIIFCNSVNRVELLAKKITELGYSCFYIHAKMLQDHRNRVFHDFRNGACRN

LVCTDLFTRGIDIQAVNVVINFDFPRNAETYLHRVGRSGRFGHLGLAVNLITYEDRFNLYRIEQELGTEIKQIPPQIDQA

IYCR

>PUZ38389.1 hypothetical protein GQ55_9G192400 [Panicum hallii var. hallii]

MHQPRARYPPGYGSGGGGRGGGGGNGGGGGGNHNYYGRNPQPQPHHHQHYHHQQPPPQQQPQQHAHRNSSHQHQQWLRRD

QGPASAAGSGDAAGRTAAQLDAVDSSSQDWKAQLNIPAPDTRYRTEDVTATKGNEFEDYFLKRELLMGIYEKGFERPSPI

QEESIPIALTGSDILARAKNGTGKTAAFCIPALEKIDPEKNAIQVVILVPTRELALQTSQVCKELGKYLNIQVMVSTGGT

SLKDDIMRLYQPVHLLVGTPGRILDLTRKGICVLKDCSMLVMDEADKLLAPEFQPSVEALIHFLPPSRQLLMFSATFPVT

VKEFKEKYLPKPYVINLMDELTLKGITQYYAFVEERQKVHCLNTLFSKLQINQSIIFCNSVNRVELLAKKITELGYSCFY

IHAKMLQDHRNRVFHDFRNGACRNLVCTDLFTRGIDIQAVNVVINFDFPKTSETYLHRVGRSGRYGHLGLAVNLITYEDR

FNLYRIEQELGTEIKTIPPQIDLAVYCQ

>THU56589.1 hypothetical protein C4D60_Mb11t18830 [Musa balbisiana]

MNPQGRYPPPGMGGSGAGANPNFYARNPQHQQQYVHRNPMQGQQNQQFQPQQHQHWSRRNQMGGDSGVGEVVKSVQSDAP

FFFLFLCLGGYSSQDWKAHLKIPPPDTRYRTEDVTATKGNEFEDYFLKRELLMGIYEKGFERPSPIQEESIPIALTGSNI

LARAKNGTGKTAAFCIPALEKIDQDRNVIQVVILVPTRELALQTSQVCKELGKHLKIQVMVTTGGTSLKDDIMRLYQPVH

LLVGTPGRILDLAKKGVCILKDCSMLIMDEADKLLAPEFQPSVEQLIRFLPASRQILLFSATFPVTVKDFKDRYLPKPYI

INLMDELTLKGITQYYAFVEERQKIHCLNTLFSKLQINQSIIFCNSVNRVELLAKKITELGYSCFYIHAKMLQDHRNRVF

HDFRNGACRNLVCTDLFTRGIDIQAVNVVINFDFPKNSETYLHRVGRSGRFGHLGLAVNLITYEDRFNLYRIEQELGTEI

KQIPPQIDQTIYCR

>XP_023539620.1 DEAD-box ATP-dependent RNA helicase 8-like [Cucurbita pepo subsp. pepo]

MNNRGRYPPGIGAGRGGGVNANPSFQSRPHQQQYVQRNLVPNQQYQQQQHQNQNQNQNQHQHQHQHQHQHQHQHQNQQLQ

QQQQWLKRNQLGGGHADSNVDEVEKTVQSEAVDSSSQDWKARLKIPPPDTRYKTEDVTATKGNEFEDYFLKRELLMGIYE

KGFERPSPIQEESIPIALTGSDILARAKNGTGKTAAFCIPALEKIDQDNNVIQVVILVPTRELALQTSQVCKELGKNLNI

QVMVTTGGTSLKDDIMRLYQPVHLLVGTPGRILDLAKKGVCVLKDCSMLIMDEADKLLSPEFQPSIEQLIRFLPANRQVL

MYSATFPVTVKDFKDRYLHRPYIINLMDELTLKGITQFYAFVEERQKVHCLNTLFSKLQINQSIIFCNSVNRVELLAKKI

TELGYSCFYIHAKMLQDHRNRVFHDFRNGACRNLVCTDLFTRGIDIQAVNVVINFDFPKNSETYLHRVGRSGRFGHLGLA

VNLITYEDRFNLYRIEQELGTEIKQIPPHIDQAIYCR

>XP_024922994.1 DEAD-box ATP-dependent RNA helicase 8 isoform X2 [Ziziphus jujuba]

MNANPAFQSRAPLQQYVQRSVVQNQHYLPQQQHQQYQQQQQWLRRGQLGSAADSNVDEVEKTVQFEAADSSSQDWKARLN

IPAPDTRFRTEDVTATKGNEFEDYFLKRELLMGIYEKGFERPSPIQEESIPIALTGSDILARAKNGTGKTAAFCIPALEK

IDQDNNVIQVIILVPTRELALQTSQVCKELGKHLNIQVMVTTGGTSLKDDIMRLYQPVHLLVGTPGRILDLAKKGVCVLK

DCSMLVMDEADKLLSPEFQPSIEQLIQFLPANRQILMFSATFPVTVKDFKDRYLHRPYIINLMDELTLKGITQFYAFVEE

RQKVHCLNTLFSKLQINQSIIFCNSVNRVELLAKKITELGYSCFYIHAKMLQDHRNRVFHDFRNGACRNLVCTDLFTRGI

DIQAVNVVINFDFPKNSETYLHRVGRSGRFGHLGLAVNLITYEDRFNLYRIEQELGTEIKQIPPHIDQAIYCR

>RLN42525.1 DEAD-box ATP-dependent RNA helicase 12 [Panicum miliaceum]

MHQPRARYPPGYGSGSGGGGRGGGGGNGGGGGGNHNYYGRNPQPHHHQHYQHQQPPPQQQPQQHAHRNSSHQHQQWLRRD

QGPASAAGSGDAAGRTAAQLDAVDSSSQDWKAQLNIPAPDTRYRTEDVTATKGNEFEDYFLKRELLMGIYEKGFERPSPI

QEESIPIALTGSDILARAKNGTGKTAAFCIPALEKIDPEKNAIQVVILVPTRELALQTSQVCKELGKYLNIQVMVSTGGT

SLKDDIMRLYQPVHLLVGTPGRILDLTRKGICVLKDCSMLVMDEADKLLAPEFQPSVESLIHFLPPSRQLLMFSATFPVT

VKEFKEKYLPKPYVINLMDELTLKGITQYYAFVEERQKVHCLNTLFSKLQINQSIIFCNSVNRVELLAKKITELGYSCFY

IHAKMLQDHRNRVFHDFRNGACRNLVCTDLFTRGIDIQAVNVVINFDFPKTSETYLHRVGRSGRYGHLGLAVNLITYEDR

FNLYRIEQELGTEIKTIPPQIDLAVYCQ

>XP_024985055.1 DEAD-box ATP-dependent RNA helicase 8-like [Cynara cardunculus var. scolymus]

MNDNSGNNSNNRRYPPGIGNGRGGGGWYGGGGGGNFQANPNYHHQPRNANQIQQHQHQHQQQQQSQYLQRQQPTQQQYNQ

KQQWLRLNPNIPGSSTANSNNYEVEKTVQSESSVDSSSQDWKAHLNIPAVDSRYKTEDVTATKGNEFEDYFLKRELLMGI

YEKGFERPSPIQEESIPIALTGSDILARAKNGTGKTAAFCIPALEKIDTDNNVIQVVILVPTRELALQTSQVCKELGKHL

QIQVMVTTGGTSLKDDIMRLYQPVHLLVGTPGRILDLTKKGVCILNDCAMLVMDEADKLLSPEFQPSVEHLISFLPESRQ

ILMFSATFPVTVKDFKDRYLKKPYVVNLMDELTLKGITQFYAFVEERQKVHCLNTLFSKLQINQSIIFCNSVNRVELLAK

KITELGYSCFYIHAKMLQDHRNRVFHDFRNGACRNLVCTDLFTRGIDIQAVNVVINFDFPRNAETYLHRVGRSGRFGHLG

LAVNLITYEDRFNLYRIEQELGTEIKQIPPQIDQAIYCQ

>XP_019428544.1 PREDICTED: DEAD-box ATP-dependent RNA helicase 8 isoform X3 [Lupinus angustifolius]

MNNRGRYPPGMGLGRGGGSGLNPNPGFQQRPPQQQYQQQQYQQQQQQQYQQQRQQQQQPQQWLRRTQLGGGNDTNVVEEV

EKTVQSEAIDQSSQDWKVGLKIPAADTRYRTEDVTATKGNEFEDYFLKRELLMGIYEKGFERPSPIQEESIPIALTGSDI

LARAKNGTGKTAAFCIPSLEKIDQDNNVIQVVILVPTRELALQTSQVCKELGKHLKIQVMVTTGGTSLKDDIMRLYQPVH

LLVGTPGRILDLAKKGVCVLKDCSVLAMDEADKLLSPEFQPSIEQLIQFLPRNRQILMFSATFPVTVKDFSDRHLRKPYI

INLMDELTLKGITQFYAFVEERQKVHCLNTLFSKLQINQSIIFCNSVNRVELLAKKITELGYSCFYIHAKMLQDHRNRVF

HDFRNGACRNLVCTDLFTRGIDIQAVNVVINFDFPKNSETYLHRVGRSGRFGHLGLAVNLITYEDRFNLYRIEQELGTEI

KQIPPHIDQAIYCQ

>EEC67267.1 hypothetical protein OsI_34232 [Oryza sativa Indica Group]

MHHPRARYPPGYTSGGGGGGGGGGGGGGGGRGNGGGGFGGGGGGGGGNHGYYGRGPQPQPQQQHYHHQAQQLHQHQQQQQ

HAQRNSSSQQQQWLRRDQATAAAASGEVAARTAAQLEAVDSSSEDWKAQLNLPAPDTRYRTEDVTATKGNEFEDYFLKRE

LLMGIYEKGFERPSPIQEESIPIALTGSDILARAKNGTGKTAAFCIPALEKIDPEKNAIQVVILVPTRELALQTSQVCKE

LGKYLNIQVMVSTGGTSLKDDIMRLYQPVHLLVGTPGRILDLTRKGICVLKDCSMLVMDEADKLLAPEFQPSIEQLIHFL

PANRQLLMFSATFPVTVKDFKEKYLPRPYVINLMDELTLKGITQYYAFVEERQKVHCLNTLFSKLQINQSIIFCNSVNRV

ELLAKKITELGYSCFYIHAKMLQDHRNRVFHDFRNGACRNLVCTDLFTRGIDIQAVNVVINFDFPKTSETYLHRVGRSGR

FGHLGLAVNLITYEDRFNLYRIEQELGTEIKTIPPQIDLAVYCQ

>XP_015614831.1 DEAD-box ATP-dependent RNA helicase 12 [Oryza sativa Japonica Group]

MHHPRARYPPGYTSGGGGGGGGGGGGGRGNGGGGFGGGGGGGGGNHGYYGRGPQPQPQQQHYHHQAQQLHQHQQQQQHAQ

RNSSSQQQQWLRRDQATAAAASGEVAARTAAQLEAVDSSSEDWKAQLNLPAPDTRYRTEDVTATKGNEFEDYFLKRELLM

GIYEKGFERPSPIQEESIPIALTGSDILARAKNGTGKTAAFCIPALEKIDPEKNAIQVVILVPTRELALQTSQVCKELGK

YLNIQVMVSTGGTSLKDDIMRLYQPVHLLVGTPGRILDLTRKGICVLKDCSMLVMDEADKLLAPEFQPSIEQLIHFLPAN

RQLLMFSATFPVTVKDFKEKYLPRPYVINLMDELTLKGITQYYAFVEERQKVHCLNTLFSKLQINQSIIFCNSVNRVELL

AKKITELGYSCFYIHAKMLQDHRNRVFHDFRNGACRNLVCTDLFTRGIDIQAVNVVINFDFPKTSETYLHRVGRSGRFGH

LGLAVNLITYEDRFNLYRIEQELGTEIKTIPPQIDLAVYCQ

>KAE8693807.1 DEAD-box ATP-dependent RNA helicase 6 [Hibiscus syriacus]

MNSGGRYPPGIGVGRGGGVNANPSFQSRPSQQQYVQRNLVQNHQHFQQHHHQQQQHQQQQLWLRRNQLPGGNDSNVVDEV

EKTVQSGAVDSSSQDWKARLTLPPPDTRYKTEDVTATKGNEFEDYFLKRELLMGIYEKGFERPSPIQEESIPIALTGSDI

LARAKNGTGKTAAFCIPALEKIDQDNNVIQVVILVPTRELALQTSQVCKELGKHLQIQVMVTTGGTSLKDDIMRLYQPVH

LLVGTPGRILDLAKKGVCILKDCSMLIMDEADKLLSPEFQPSIEQLIRFLPANCQILMFSATFPVTVKDFKDRYLKKPYI

INLMDELTLKGITQYYAFVEERQKVHCLNTLFSKLQINQSIIFCNSVNRVELLAKKITELGYSCFYIHAKMLQDHRNRVF

HDFRNGACRNLVCTDLFTRGIDIQAVNVVINFDFPKNSETYLHRVGRSGRFGHLGLAVNLITYEDRFNLYRIEQELGTEI

KQIPPHIDQAIYCR

>XP_021840285.1 DEAD-box ATP-dependent RNA helicase 8-like [Spinacia oleracea]

MNDNVRGRFPPGMGRGRGGLGVDVGGLGGGGRGGGGGGGGMGGNPNFQAGNFQQQQHYVQRGYNNNMQNHHQQQHQFQRQ

QQQQQHEQHFQHHQQQLQQHKQQQQHQQQQQQQQQQQQWLRRNQLGGTTETNVEEVEKTVQSEGIDSSSQDWKIGLKPPP

QDTRFRTEDVTATKGNEFEDYFLKRELLMGIYEKGFERPSPIQEESIPIALTGSDILARAKNGTGKTAAFCVPALEKIDQ

DNNVIQVVILVPTRELALQTSQVCKELGKHLKIQVMVTTGGTSLKDDIMRLHQPVHLLVGTPGRILDLAKKGICVLKDCS

MLVMDEADKLLSPEFQPSIEQLIQFMPTSRQILMFSATFPVTVKDFKDRYLHKPYVINLMDELTLKGITQFYAFVEERQK

VHCLNTLFSKLQINQSIIFCNSVNRVELLAKKITELGYSCFYIHAKMLQDHRNRVFHDFRNGACRNLVCTDLFTRGIDIQ

AVNVVINFDFPKNAETYLHRVGRSGRFGHLGLAVNLITYEDRFNLYKIEQELGTEIKQIPPFIDQTIYCR

>XP_020584087.1 DEAD-box ATP-dependent RNA helicase 8-like [Phalaenopsis equestris]

MNPRGRYPTGIGNGRGGSAGSNPNFYPRGPQPQQQYVQRNLISSQQRQQLQQQQEEWRRSQMNQVESSSSAFDAVNLVPS

EGRDSSSQDWKVQLKIPPPDTRYKTEDVTATKGNDFEDYFLKRELLMGIYEKGFEKPSPIQEESIPIALTGRNILARAKN

GTGKTAAFCIPALEKIDQDKNSIQVVILVPTRELALQTSQVCKELGKHLKVQVMVTTGGTSLKDDIMRLYQPVHLLIGTP

GRILDLTKKEVCVLKDCGLLILDEADKLLSPEFQPSIEQLIRFFPANRQIFLFSATFPVTVKDFKDRFLPNPYIINLMDE

LTLKGITQFYAFVEERQKVHCLNTLFSKLQINQSIIFCNSVNRVELLAKKITELGYSCFYIHAKMLQDHRNRVFHDFRNG

ACRNLVCTDLFTRGIDIQAVNVVINFDFPRNSETYLHRVGRSGRFGHLGLAVNLITYEDRFNLYRIEQELGTEIKQIPPQ

IDQAIYCK

>XP_018845393.1 DEAD-box ATP-dependent RNA helicase 8-like [Juglans regia]

MNNNRGRYPPGIGAGRGGGMNLNPAFQSRVPQQHYVQRSLVQQQNHHQQQQHQYQQQQQQYHQQQQQQQQQWLRRGQLGG

ADSIVDEVEKTVQSEAVDSSSQDWKARLKIPPPDTRYRTEDVTATKGNEFEDYFLKRELLMGIYEKGFERPSPIQEESIP

IALTGSDILARAKNGTGKTAAFCIPALEKIDQDNNAIQVVILVPTRELALQTSQVCKELGKHLNIQVMATTGGTSLKDDI

MRLYQPVHLLVGTPGRILDLAKKGVCILKDCSMLVMDEADKLLSPEFQPSIEQLIRFMPANRQILMFSATFPVTVKDFKD

RYLQKSYIINLMDELTLKGITQFYAFVEERQKVHCLNTLFSKLQINQSIIFCNSVNRVELLAKKITELGYSCFYIHAKML

QDHRNRVFHDFRNGACRNLVCTDLFTRGIDIQAVNVVINFDFPKNSETYLHRVGRSGRFGHLGLAVNLITYEDRFNLYRI

EQELGTEIKQIPPHIDQAIYCR

>XP_028057917.1 DEAD-box ATP-dependent RNA helicase 8-like [Camellia sinensis]

MNTRGRYPPGIGGGGGRGGSLNVNLNFHPRNFQQQYVQRGPMQNHHQQFQSQQQHQQHQQHQQPQWLRRNQLGAELSVDE

VEKTVQSEAVDSSSQDWKARLKIPPADTRYRTEDVTATKGNEFEDYFLKRDLLMGIYEKGFERPSPIQEESIPIALTGSD

ILARAKNGTGKTAAFCIPALEKIDQDNNVIQVVILVPTRELALQTSQVCKELGKYLNIQVMVTTGGTSLKEDIMRLYQPV

HLLVGTPGRILDLAKKGVCILKDCSMLVMDEADKLLSPEFRPSLDQLIHFLPVNRQILMFSATFPVTVKDFKDRYLHRPY

VINLMDELTLKGITQFYAFVEERQKVHCLNTLFSKLQINQSIIFCNSVNRVELLAKKITELGYSCFYIHAKMLQDHRNRV

FHDFRNGACRNLVCTDLFTRGIDIQAVNVVINFDFPKNSETYLHRVGRSGRFGHLGLAVNLITYEDRFNLYRIEQELGTE

IKQIPPHIDQGIYCR

>XP_021306631.1 DEAD-box ATP-dependent RNA helicase 12 [Sorghum bicolor]

MDQPRARYPPGYGSRGGGGAGRGGGGGNGGGGGGGGGNHNYYGRNPQPQQQHHYQQQQQQQQHVHRNSSHQQQWLRRDQA

PAVAGAASGNAAAKTAPQLDAIDSSSQDWKAQLNIPAPDTRYRTEDVTATKGNEFEDYFLKRELLMGIYEKGFERPSPIQ

EESIPIALTGSDILARAKNGTGKTAAFCIPALEKIDPEKTAIQVVILVPTRELALQTSQVCKELGKYLNIQVMVSTGGTS

LKDDIMRLYQPVHLLVGTPGRILDLTRKGICVLKDCSMLVMDEADKLLAPEFQPSVEALIHFLPPSRQLLMFSATFPVTV

KEFKEKYLPRPYVINLMDELTLKGITQYYAFVEERQKVHCLNTLFSKLQINQSIIFCNSVNRVELLAKKITELGYSCFYI

HAKMLQDHRNRVFHDFRNGACRNLVCTDLFTRGIDIQAVNVVINFDFPKTSETYLHRVGRSGRYGHLGLAVNLITYEDRF

NLYRIEQELGTEIKTIPPQIDLAVYCQ

>XP_019428542.1 PREDICTED: DEAD-box ATP-dependent RNA helicase 8 isoform X1 [Lupinus angustifolius]

MNNRGRYPPGMGLGRGGGSGLNPNPGFQQRPPQQQYVQRHIMQQQQQQYQQQQQQYQQQQQQQYQQQRQQQQQPQQWLRR

TQLGGGNDTNVVEEVEKTVQSEAIDQSSQDWKVGLKIPAADTRYRTEDVTATKGNEFEDYFLKRELLMGIYEKGFERPSP

IQEESIPIALTGSDILARAKNGTGKTAAFCIPSLEKIDQDNNVIQVVILVPTRELALQTSQVCKELGKHLKIQVMVTTGG

TSLKDDIMRLYQPVHLLVGTPGRILDLAKKGVCVLKDCSVLAMDEADKLLSPEFQPSIEQLIQFLPRNRQILMFSATFPV

TVKDFSDRHLRKPYIINLMDELTLKGITQFYAFVEERQKVHCLNTLFSKLQINQSIIFCNSVNRVELLAKKITELGYSCF

YIHAKMLQDHRNRVFHDFRNGACRNLVCTDLFTRGIDIQAVNVVINFDFPKNSETYLHRVGRSGRFGHLGLAVNLITYED

RFNLYRIEQELGTEIKQIPPHIDQAIYCQ

>XP_021838650.1 DEAD-box ATP-dependent RNA helicase 8-like [Spinacia oleracea]

MNHNSRPRYPPGIGNGRGGGYAQQNPNFQTRPPYHHQQHHNQPHYVPRNAPPQNQHQYQQQQHQQQQQQQQQQQQQQQQQ

WMMRRPANSADEIEKSAQTPRSAVDSSSEDWKANLKLPPPDTRYMTEDVTATKGNEFEDYFLKRELLMGIYEKGFERPSP

IQEESIPIALTGSDILARAKNGTGKTAAFCVPVLEKIDQDNNVIQAVILVPTRELALQTSQVCKELGKHLNVQVMVTTGG

TSLKDDIMRLYQPVHLLVGTPGRILDLAKRGICVLKDCSMLVMDEADKLLSPEFQPSLEQLISFLPGNRQILMFSATFPV

TVKDFKDRYLQKPYVINLMDELTLKGITQFYAFVEERQKLHCLNTLFSKLQINQSIIFCNSVNRVELLAKKITELGYSCF

YIHAKMLQDHRNRVFHDFRNGACRNLVCTDLFTRGIDIQAVNVVINFDFPKNSETYLHRVGRSGRYGHLGLAVNLITFED

RFNLYKIEQELGTEIKQIPPHIDQGIYCR

>XP_022733552.1 DEAD-box ATP-dependent RNA helicase 8 [Durio zibethinus]

MNSRGRYPPGIGVGRGGGLKTNPSFQSRSPQQQYVQRNFVQNHHQFQQQQQQQQLWLRRNQLPGGNDSSVVDEVEKTVQL

EAVDSSSQDWKARLKVPPPDTRYKTEDVTATKGNEFEDYFLKRELLMGIYEKGFERPSPIQEESIPIALTGSHILARAKN

GTGKTAAFCIPALEKIDQDKNVIQVVILVPTRELALQTSQVCKELGKHLQIQVMVTTGGTSLKDDIMRLYQPVHLLVGTP

GRILDLAKKGVCILKDCSMLIMDEADKLLSPEFQPSVEQLIRFLSANRQILMFSATFPVTVKDFKDRYLHKPYIINLMDE

LTLKGITQYYAFVEERQKVHCLNTLFSKLQINQSIIFCNSVNRVELLAKKITELGYSCFYIHAKMLQDHRNRVFHDFRNG

ACRNLVCTDLFTRGIDIQAVNVVINFDFPKNSETYLHRVGRSGRFGHLGLAVNLITYEDRFNLYRIEQELGTEIKQIPPH

IDQAIYCR

>XP_015883154.1 DEAD-box ATP-dependent RNA helicase 8-like [Ziziphus jujuba]

MHNPRGRYPPGIGAGRGGGMNANPAFQSRAPQQQYVQRSVVQNPHYLPQQQHQQHQQQHQQQQQWLRRAQLGSAADSNVD

EVEKTVQYEAVDSSSQDWKARLKMPPPDTRYKTEDVTATKGNEFEDYFLKRELLMGIYEKGFERPSPIQEESIPIALTGS

DILARAKNGTGKTAAFCVPALEKIDQDINVIQVVILVPTRELALQTSQVCKELGKHLNIQVMVTTGGTSLKDDIMRLYQP

VHLLVGTPGRILDLAKKGVCVLKDCSMLVMDEADKLLSPEFQPSIEQLIRFLSPNRQILMFSATFPVTVKDFKDRYLQKP

YVINLMDELTLKGITQFYAFVEERQKVHCLNTLFSKLQINQSIIFCNSVNRVELLAKKITELGYSCFYIHAKMLQDHRNR

VFHDFRNGACRNLVCTDLFTRGIDIQAVNVVINFDFPKNSETYLHRVGRSGRFGHLGLAVNLITYEDRFNLYRIEQELGT

EIKQIPPQIDQAIYCR

>XP_019428543.1 PREDICTED: DEAD-box ATP-dependent RNA helicase 8 isoform X2 [Lupinus angustifolius]

MNNRGRYPPGMGLGRGGGSGLNPNPGFQQRPPQQQYVQRHIMQQQQQQYQQQQQQYQQQQQQPQQWLRRTQLGGGNDTNV

VEEVEKTVQSEAIDQSSQDWKVGLKIPAADTRYRTEDVTATKGNEFEDYFLKRELLMGIYEKGFERPSPIQEESIPIALT

GSDILARAKNGTGKTAAFCIPSLEKIDQDNNVIQVVILVPTRELALQTSQVCKELGKHLKIQVMVTTGGTSLKDDIMRLY

QPVHLLVGTPGRILDLAKKGVCVLKDCSVLAMDEADKLLSPEFQPSIEQLIQFLPRNRQILMFSATFPVTVKDFSDRHLR

KPYIINLMDELTLKGITQFYAFVEERQKVHCLNTLFSKLQINQSIIFCNSVNRVELLAKKITELGYSCFYIHAKMLQDHR

NRVFHDFRNGACRNLVCTDLFTRGIDIQAVNVVINFDFPKNSETYLHRVGRSGRFGHLGLAVNLITYEDRFNLYRIEQEL

GTEIKQIPPHIDQAIYCQ

>XP_021754108.1 DEAD-box ATP-dependent RNA helicase 8-like [Chenopodium quinoa]

MNHNSRPRFPPGIGNGRGAGGGGYVQQNPNFQNRPPYHHQQQQQPQYVPRNAPPQNQHQYQQQQQQQQQQQQQQQQQQQW

MMRRPANSATDGGSNQSSVNQVEKSIQTAASGVDSSSQDWKANLKLPPADTRYMTEDVTATKGNEFEDYFLKRELLMGIY

EKGFERPSPIQEESIPIALTGSDILARAKNGTGKTAAFCIPALEKIDQDNNVIQAVILVPTRELALQTSQVCKELGKHLK

IQVMVTTGGTSLKDDIMRLYQPVHLLVGTPGRILDLAKRGICVLKDCSMLVMDEADKLLSPEFQPSVEQLIRFLPGNRQV

LMFSATFPVTVKDFKDRFLQKPYVINLMDELTLKGITQFYAFVEERQKLHCLNTLFSKLQINQSIIFCNSVNRVELLAKK

ITELGYSCFYIHAKMLQDHRNRVFHDFRNGACRNLVCTDLFTRGIDIQAVNVVINFDFPKNSETYLHRVGRSGRYGHLGL

AVNLITYEDRFNLYKIEQELGTEIKQIPPHIDQGIYCR

>KAF2914289.1 hypothetical protein DAI22_10g151100 [Oryza sativa Japonica Group]

MHHPRARYPPGYTSGGGGGGGGGGGGGRGNGGGGFGGGGGGGGGNHGYYGRGPQPQPQQQHYHHQAQQLHQHQQQQQHAQ

RNSSSQQQQWLRRDQATAAAASGEVAARTAAQLEAVDSSSEDWKAQLNLPAPDTRYRTEDVTATKGNEFEDYFLKRELLM

GIYEKGFERPSPIQEESIPIALTGSDILARAKNGTGKTAAFCIPALEKIDPEKNAIQVVILVPTRELALQTSQVCKELGK

YLNIQVMVSTGGTSLKDDIMRLYQPVHLLVGTPGRILDLTRKGICVLKDCSMLVMDEADKLLAPEFQPSIEQLIHFLPAN

RQLLMFSATFPVTVKDFKEKYLPSPYVINLMDELTLKGITQYYAFVEERQKVHCLNTLFSKLQINQSIIFCNSVNRVELL

AKKITELGYSCFYIHAKMLQDHRNRVFHDFRNGACRNLVCTDLFTRGIDIQAVNVVINFDFPKTSETYLHRVGRSGRFGH

LGLAVNLITYEDRFNLYRIEQELGTEIKTIPPQIDLAVYCQ

>GER44553.1 ATP-dependent RNA helicase DHH1 [Striga asiatica]

MMNNNYSRGRYPPGIGNGRGGGNGIGVGNANQNYPNRNPQYPQPQPPYPQRTTAQNQPQQWMRRNPNTAAVSDSSNEVEK

TVQPDSTGSTSQDWKARLAVPPPDTRYKTEDVTATKGNEFEDYFLKRDLLMGIYEKGFERPSPIQEESIPIALTGSDILA

RAKNGTGKTAAFCIPALEKIDSDNNVIQVPTRELALQTSQVCKELGKHLKIQVMVSTGGTSLKDDIMRLYQPVHLLVGTP

GRILDLTRKGICILKDCSVLVMDEADKLLSPEFQPSIEQLIAFLPPTRQILMYSATFPVTVKDFKDKYLRKPYIINLMDE

LTLKGITQYYAFVEERQKVHCLNTLFSKLQINQSIIFCNSVNRVELLAKKITELGYSCFYIHAKMLQDHRNRVFHDFRNG

ACRNLVCTDLFTRGIDIQAVNVVINFDFPKNSETYLHRVGRSGRFGHLGLAVNLITYEDRFNLYRIEQELGTEIKQIPPH

IDQAIYYINN

>XP_015883083.1 DEAD-box ATP-dependent RNA helicase 8 isoform X1 [Ziziphus jujuba]

MHNPRGRYPPGISAGRGGGMNANPAFQSRAPLQQYVQRSVVQNQHYLPQQQHQQYQQQQQWLRRGQLGSAADSNVDEVEK

TVQFEAADSSSQDWKARLNIPAPDTRFRTEDVTATKGNEFEDYFLKRELLMGIYEKGFERPSPIQEESIPIALTGSDILA

RAKNGTGKTAAFCIPALEKIDQDNNVIQVIILVPTRELALQTSQVCKELGKHLNIQVMVTTGGTSLKDDIMRLYQPVHLL

VGTPGRILDLAKKGVCVLKDCSMLVMDEADKLLSPEFQPSIEQLIQFLPANRQILMFSATFPVTVKDFKDRYLHRPYIIN

LMDELTLKGITQFYAFVEERQKVHCLNTLFSKLQINQSIIFCNSVNRVELLAKKITELGYSCFYIHAKMLQDHRNRVFHD

FRNGACRNLVCTDLFTRGIDIQAVNVVINFDFPKNSETYLHRVGRSGRFGHLGLAVNLITYEDRFNLYRIEQELGTEIKQ

IPPHIDQAIYCR

>XP_031128272.1 DEAD-box ATP-dependent RNA helicase 8-like [Ipomoea triloba]

MNYRARYPPPGMSSGGRGGAGLNPNANPDFKPRNPHQYAQRSPVPNQQHFQNQQTQQWLRRNKELGADFTADEVEKTVQS

EGIDASSQNWKAQLKIPPPDTRYRTEDVTATKGNEFEDYFLKRELLMGIYEKGFEKPSPIQEESIPIALTGSDILARAKN

GTGKTAAFCIPAIEKIDLDNNSIQVIILVPTRELALQTSQVCKELGKHLKIQVMVSTGGTSLKDDIMRLYQPVHLLVGTP

GRILDLTKKGVCILKDCSMLVMDEADKLLAPEFQPSVEQLIRFLPANRQILMFSATFPVTVKDFKDRFLHKPYVINLMDE

LTLKGITQYYAFVEERQKVHCLNTLFSKLQINQSIIFCNSVNRVELLAKKITELGYSCFYIHAKMLQDHRNRVFHDFRNG

ACRNLVCTDLFTRGIDIQAVNVVINFDFPKNAETYLHRVGRSGRYGHLGLAVNLITFEDRFNLYKIEQELGTEIKQIPPH

IDQAIYCQ

>ACG38116.1 ATP-dependent RNA helicase dhh1 [Zea mays]

MHQPRARYPPGYGSSGGGGGGGGGSGGGRGGSGGGGNHNYYGRNTHPQHQHHYQQQQQHSHRNSSHQQQWLRRDQGPAVA

GPVSGNAVPKTAPQLDAVDSSSQDWKAQLNIPAPDTRYRTEDVTATKGNEFEDYFLKRELLMGIYEKGFERPSPIQEESI

PIALTGSDILARAKNGTGKTAAFCIPALEKIDPENNAIQVVILVPTRELALQTSQVCKELGKYLNIEVMVSTGGTSLKDD

IMRLYQPVHLLVGTPGRILDLTRKGICVLKDCSMLVMDEADKLLAPEFQPSVEALIHFLPPSRQLLMFSATFPVTVKEFK

EKYLPRPYVINLMDELTLKGITQYYAFVEERQKVHCLNTLFSKLQINQSIIFCNSVNRVELLAKKITELGYSCFYIHAKM

LQDHRNRVFHDFRNGACRNLVCTDLFTRGIDIQAVNVVINFDFPKTSETYLHRVGRSGRYGHLGLAVNLITYEDRFNLYR

IEQELGTEIKTIPPQIDLAVYCQ

>TVU32086.1 hypothetical protein EJB05_23804 [Eragrostis curvula]

MHQPRARYPPGYGSGGRGGGGGNGGGGGGGGGGNYNYYGRNPQPQHQHHYHHHHQQQELQQQHAHRNAQHQQQQQQQQQQ

WLRRDQAAAAAGDPAGRTASQFDAVDPSSEDRDLTNDLVDSLLLSSQDWKAQLNIPAPDTRYRTEDVTATKGNEFEDYFL

KRELLMAIYEKGFERPSPIQEESIPIALTGSDILARAKNGTGKTAAFCIPALEKIDPEKNAIQVVILVPTRELALQTSQV

CKELGKYLNIQVMVSTGGTSLKDDIMRLYQPVHLLVGTPGRILDLTRKGICMLNECSILIMDEADKLLAPEFQPSVEQLI

HFLPASRQLLMFSATFPVTVKEFKEKYLPRPYVINLMDELTLKGITQHYAFVEERQKVHCLNTLFSKLQINQSIIFCNSV

NRVELLAKKITELGYSCFYIHAKMLQDHRNRVFHDFRNGACRNLVCTDLFTRGIDIQAVNVVINFDFPKTSETYLHRVGR

SGRYGHLGLAVNLITYEDRFNLYRIEQELGTEIKPIPPQIDVEVYCQ

>VDC81163.1 unnamed protein product [Brassica rapa]

MNNNNNNNNRGGGGGRYPPGIGAGRGAINPNPNFQSRPGYQQQQQPQYVQRGGYSHHQQQFQQATSQPPRQYQQQQHQQQ

WLPRPQISTGNSNGGGGGDAVVEVEKTVLSDTNSEDWKARLKLPAPDTRYRTEDVTATKGNEFEDYFLKRELLMGIYEKG

FERPSPIQEESIPIALTGRDILARAKNGTGKTAAFCIPVLEKIDQDNNVIQVFSLLPVLYVAVIIVPTRELALQTSQVCK

ELGKHLKIQVMVTTGGTSLKDDIMRLYQPVHLLVGTPGRILDLTKKGVCVLKDCSVLAMDEADKLLSQEFQPSVEHLISF

LPQNRQILMFSATFPVTVKYFKDRFLTNPYIINLMDELTLKGITQFYAFVEERQKIHCLNTLFSKLQINQSIIFCNSVNR

VELLAKKITELGYSCFYIHAKMLQDHRNRVFHDFRNGACRNLVCTDLFTRGIDIQAVNVVINFDFPKNAETYLHRVGRSG

RFGHLGLAVNLITYEDRFNLYRIEQELGTEIKQIPPHIDQAIYCQ

>KNA19892.1 hypothetical protein SOVF_057190 [Spinacia oleracea]

MNHNSRPRYPPGIGNGRGGGYAQQNPNFQTRPPYHHQQHHNQPHYVPRNAPPQNQHQYQQQQHQQQQQQQQQQQQQQQQQ

WMMRRPANSADEIEKSAQTPRSAVDSSSEDWKANLKLPPPDTRYMTEDVTATKGNEFEDYFLKRELLMGIYEKGFERPSP

IQEESIPIALTGSDILARAKNGTGKTAAFCVPVLEKIDQDNNVIQAVILVPTRELALQTSQVCKELGKHLNVQVMVTTGG

TSLKDDIMRLYQPVHLLVGTPGRILDLAKRGICVLKDCSMLVMDEADKLLSPEFQPSLEQSISFLPGNRQILMFSATFPV

TVKDFKDRYLQKPYVINLMDELTLKGITQFYAFVEERQKLHCLNTLFSKLQINQSIIFCNSVNRVELLAKKITELGYSCF

YIHAKMLQDHRNRVFHDFRNGACRNLVCTDLFTRGIDIQAVNVVINFDFPKNSETYLHRVGRSGRYGHLGLAVNLITFED

RFNLYKIEQELGTEIKQIPPHIDQGIYCR

>XP_012840184.1 PREDICTED: DEAD-box ATP-dependent RNA helicase 6-like [Erythranthe guttata]

MGSRARYPPPGMGGGRGGGGGGGGGGMNPNAGPNPSFQSRNPPHQYVQRSPAPNNQNYQLFQNPNPQQWQRRTQMLPADS

SVDEVEKTVQSEATDSSSQDWKAQLKLPPQDTRYRTEDVTATKGNEFEDYFLKRELLMGIYEKGFERPSPIQEESIPIAL

TGSDILARAKNGTGKTASFCIPALEKIDQDKNAIQVVILVPTRELALQTALVCKELGKHLQIQVMATTGGTSLKDDIMRL

YQPVHLLVGTPGRVLDLANKGVCILNECCMLVMDEADKLLSPEFQPSIEQLIRFMPTNRQILMFSATFPVTVKSFKDRYL

HKPYVINLMDELTLKGITQFYAFVEERQKIHCLNTLFSKLQINQSIIFCNSVNRVELLAKKITELGYSCFYIHAKMLQDH

RNRVFHDFRNGACRNLVCTDLFTRGIDIQAVNVVINFDFPKNSETYLHRVGRSGRFGHLGLAVNLITYEDRFNLYRIEQE

LGTEIKQIPPNIDQAIYCL

>XP_034709974.1 DEAD-box ATP-dependent RNA helicase 8-like [Vitis riparia]

MNPRGRYPPGIGDGRGGNFHSNPNFQNRNPNYQQQQYFQRPPMQNQQQQQWLRRIPIATDSPANEVEKTVQSEVVDSSSQ

DWKAQLRVPPPDTRYKTEDVTATKGNEFEDYFLKRELLMGIYEKGFERPSPIQEESIPIALTGSDILARAKNGTGKTAAF

CIPALEKIDPDNNVIQVVILVPTRELALQTSQVCKELGKHLKIEVMVTTGGTSLKDDIMRLYQPVHLLVGTPGRILDLSK

KGICILKDCSVLVMDEADKLLSPEFQPSVEQLIRFLPQNRQILMFSATFPVTVKDFKDRYLKKPYVINLMDELTLKGITQ

YYAFVEERQKVHCLNTLFSKLQINQSIIFCNSVNRVELLAKKITELGYSCFYIHAKMLQDHRNRVFHDFRNGACRNLVCT

DLFTRGIDIQAVNVVINFDFPKNSETYLHRVCGRSGRFGHLGLAVNLITYEDRFNLYRIEQELGAEIKQIPPHIDQAIYC

R

>XP_021735867.1 DEAD-box ATP-dependent RNA helicase 8-like [Chenopodium quinoa]

MNHNSRPRFPPGIGNGRGGYVQQNPNFQNRPPYHHQQPQQQPQYVPRNAPPQNQHQYQQQQQQQQVQQQQQQQQQQWMMR

RPANSATDGGSNQSSVNQVEKSMQTASSGVDSSSQDWKANLKLPPADTRYMTEDVTATKGNEFEDYFLKRELLMGIYEKG

FERPSPIQEESIPIALTGSDILARAKNGTGKTAAFCIPALEKIDQDNNVIQAVILVPTRELALQTSQVCKELGKHLKIQV

MVTTGGTSLKDDIMRLYQPVHLLVGTPGRILDLAKRGICVLKDCSMLVMDEADKLLSPEFQPSVEQLIRFLPGNRQVLMF

SATFPVTVKDFKDRFLQKPYVINLMDELTLKGITQFYAFVEERQKLHCLNTLFSKLQINQSIIFCNSVNRVELLAKKITE

LGYSCFYIHAKMLQDHRNRVFHDFRNGACRNLVCTDLFTRGIDIQAVNVVINFDFPKNSETYLHRVGRSGRYGHLGLAVN

LITYEDRFNLYKIEQELGTEIKQIPPHIDQGIYCR

>KHN27312.1 DEAD-box ATP-dependent RNA helicase 8 [Glycine soja]

MDSSSQDWKARLKIPPADTRYSPQDVTATKGNEFEDYFLKRELLMGIYEKGFERPSPIQEESIPIALTGSDILARAKNGT

GKTAAFCIPALEKIDQDNNVIQVVILVPTRELALQTSQVCKELGKHLKIQVMVTTSGTSLKDDIMCLYQPVHLLVGTAGR

ILDLAKKGVCILKDCAMLVMDEADKLLSPEFQPSIEQLIHFLPTTRQILMFSATFPVTVKDFKDRYLRKPYVINLMDELT

LKGITQFYAFVEERQKVHCLNTLFSKLQINQSIIFCNSVNRVELLAKKITELGYSCFYIHAKMLQDHRNRVFHDFRNGAC

RNLVCTDLFTRGIDIQAVNVVINFDFPKNAETYLHRVGRSGRFGHLGLAVNLITYEDRFNLYRIEQELGTEIKQIPPQID

QAIYCR

>XP_019176223.1 PREDICTED: DEAD-box ATP-dependent RNA helicase 6-like [Ipomoea nil]

MNYRARYPPPGMSSGGRGGAGLNPNANPDFKPRNPHQYVQRSPLPNQQHFQNQQTQQWLRRNNELGADFTADEVEKTVQS

EGIDASSQNWKAQLNIPPPDTRYRTEDVTATKGNEFEDYFLKRELLMGIYEKGFEKPSPIQEESIPIALTGSDILARAKN

GTGKTAAFCIPALEKIDIDINSIQVIILVPTRELALQTSQVCKELGKHLKIQVMVTTGGTSLKDDILRLYQPVHLLVGTP

GRILDLSKKGVCMLKDCSMLVLDEADKLLAPEFQPSVEQLIRFLPLNRQVLMFSATFPVTVKDFKDRYLHKPYVINLMDE

LTLKGITQYYAFVEERQKVHCLNTLFSKLQINQSIIFCNSVNRVELLAKKITELGYSCFYIHAKMLQDHRNRVFHDFRNG

ACRNLVCTDLFTRGIDIQAVNVVINFDFPKNSETYLHRVGRSGRYGHLGLAVNLITYEDRFNLYRIEQELGTEIKQIPPH

IDQTIYCR

>MBA0879908.1 hypothetical protein [Gossypium schwendimanii]

MNSRGRYPPPGIGVGRGGGVNANPSFQSRPSQQHYVQRNLVHNQQHFQQHNQQHFQQQQQQQWLRRNQLLSGNDSSVIDE

VEKTVQSEAVDSSSQDWKARLKIPPADTRYKTEDVTATKGNEFEDYFLKRELLMGIYEKGFERPSPIQEESIPIALTGSD

ILARAKNGTGKTAAFCIPALEKIDQDNNVIQVVILVPTRELALQTSQVCKELGKHLQIQVMVTTGGTSLKDDIMRLYQPV

HLLVGTPGRILDLAKKGVCILKDCSMLIMDEADKLLSPEFQPSVEQLIRFLPATRQILLFSATFPVTVKDFKDRYLQKPY

IINLMDELTLKGITQYYAFVEERQKVHCLNTLFSKLQINQSIIFCNSVNRVELLAKKITELGYSCFYIHAKMLQDHRNRV

FHDFRNGACRNLVCTGMLCHAVCFCLLDLFTRGIDIQAVNVVINFDFPKNSETYLHRVGRSGRFGHLGLAVNLITYEDRF

NLYRIEQELGTEIKQIPPHIDQAIYCR

>GFP93311.1 dead-box ATP-dependent RNA helicase 8 [Phtheirospermum japonicum]

MGSRARYPPPGMGGGRGGMNPNAGPNPNFELRNPTQQYVQRGPPPNNQNQHQLFQNPQPQQWLRRAKLPSNDLDEVEKTV

QSEAIDSSSQDWKARLKLPPQDTRYRTEDVTATKGNEFEDYFLKRELLMGIYEKGFERPSPIQEESIPIALTGSDILARA

KNGTGKTAAFCIPALEKIDQDKNSIQVVILVPTRELALQTSQVCKELGKHLNIQVMASTGGTSLKDDIMRLYQPVHLLVG

TPGRILDLAKKGICVLNDCCMLVMDEADKLLSPEFQPTIEQLIRFLPANRQILMFSATFPVTVKDFKDRYLHRPYIINLM

DELTLKGITQFYAFVEERQKVHCLNALFSKLQINQSIIFCNSVNRVELLAKKITELGYSCFYIHAKMLQDHRNRVFHDFR

NGACRNLVCTDLFTRGIDIQAVNVVINFDFPKNSETYLHRVGRSGRFGHLGLAVNLITYEDRFNLYRIEQELGTEIKQIP

PHIDQAIYCQ

>XP_002464413.1 DEAD-box ATP-dependent RNA helicase 12 [Sorghum bicolor]

MHQPRARYPPGYGSGGGGGAGRGGGGGNGGGGGGGGGNHNYYGRNPQPQQQHHHQQQQQQQQHVHRNSSHQQQWLRRDQA

PAVAGAASGNAAAKTAPQLDAIGSSSHDWKAQLNIPAPDTRYRTEDVTATKGNEFEDYFLKRELLMGIYEKGFERPSPIQ

EESIPIALTGSDILARAKNGTGKTAAFCIPALEKIDPEKTAIQVVILVPTRELALQTSQVCKELGKYLNIQVMVSTGGTS

LKDDIMRLYQPVHLLVGTPGRILDLTRKGICVLKDCSMLVMDEADKLLAPEFQPSVEALIHFLPPSRQLLMFSATFPVTV

KEFKEKYLPRPYVINLMDELTLKGITQYYAFVEERQKVHCLNTLFSKLQINQSIIFCNSVNRVELLAKKITELGYSCFYI

HAKMLQDHRNRVFHDFRNGACRNLVCTDLFTRGIDIQAVNVVINFDFPKTSETYLHRVGRSGRYGHLGLAVNLITYEDRF

NLYRIEQELGTEIKTIPPQIDLAVYCQ

>XP_023764404.1 DEAD-box ATP-dependent RNA helicase 8-like [Lactuca sativa]

MNNNYARGGGGRYPPGIGRGGGGNYHGNPNPNFQQQYAQRNPAHHQQFQQQQQQQQQQQWLRRNPVGNDSSVVDEVEKTI

QSEAADPSGQDWKAQLRLPPADTRYRTEDVTATKGNEFEDYFLKRELLMGIYEKGFERPSPIQEESIPIALTGSDILARA

KNGTGKTAAFCIPALEKIDQESNVIQVVILVPTRELALQTSQVCKELGKHLNIQVMVTTGGTSLKDDIMRLYQPVHLLVG

TPGRILDLSKKGICVLKDCAMLVMDEADKLLSPEFQPSVEQLIHFMPTNRQILMFSATFPVTVKDFKDRYLPKSYVINLM

DELTLKGITQFYAFVEERQKVHCLNTLFSKLQINQSIIFCNSVNRVELLAKKITELGYSCFYIHAKMLQDHRNRVFHDFR

NGACRNLVCTDLFTRGIDIQAVNVVINFDFPKNSETYLHRVGRSGRFGHLGLAVNLITYEDRFNLYRIEQELGTEIKQIP

PFIDQAIYCR

>XP_019156018.1 PREDICTED: DEAD-box ATP-dependent RNA helicase 8-like [Ipomoea nil]

MNARRRYSPGTGNGGGDGYGGGGFQSNHNSRGGYRQGRNPYYQYGQQHYPSQPQQYGKRSLQNEPHQQQQQWLRRNASGA

PPESSYNEVRKTIHSDGIDSSSNVWKARLNIPPPDTRYRTEDVTATKGNEFEDYFLKRELLMGIYEKGFERPSPIQEESI

PIALTGSDILARAKNGTGKTASFCIPALEKIDPDNNVIQVVILVPTRELALQTSQVCKELGKHLKIQVMVSTGGTSLKDD

IMRLYQPVHLLVGTPGRILDLAKRGVCILKDCAMIAMDEADKLLSPEFQPSVEQLIMFLPRHRQILMFSATFPVTVKDFK

DRYLQKPYIINLMDELTLKGITQFYAFVEERQKVHCLNTLFSMLQINQSIIFCNSVHRVELLAKKITDLGYSCFYIHAKM

LQDHRNKVFHDFRNGSCRNLVCTDLFTRGIDIQAVNVVINFDFPKNSETYLHRVGRSGRFGHLGLAVNLITYEDRFNLYK

IEQELGTEIKPIPPHIDQAIYCQ

>XP_004240107.1 DEAD-box ATP-dependent RNA helicase 8 [Solanum lycopersicum]

MNSRSRYPPPWMGGGRGGGGGGSMHPNANTSFQHRNQQQYMQRSSVPHQQQFQNQQTQQWMRRNQLSSDSAIDEVEKTVQ

SENGSSQDWKARLKIPPLDARYRTEDVTATKGNDFEDYFLKRELLMGIYEKGFERPSPIQEESIPIVLTGSDILARAKNG

TGKTAAFCIPALEKIDQDNNVIQVIILVPTRELALQTSQVCKELGKHLKIQVMVSTGGTSLKDDIMRLYQPVHLLVGTPG

RILDLARKGVCVLKDCSMLVMDEADKMLSPEFLPSIEQLIRFLPASRQILMFSATFPVTVKAFKDRYLQKPYVINLMDEL

TLKGITQFYAFVEERQKLHCLNTLFSKLQINQSIIFCNSVNRVELLAKKITELGYSCFYIHAKMLQDHRNRVFHDFRNGA

CRNLVCTDLFTRGIDIQAVNVVINFDFPKNSETYLHRVGRSGRFGHLGLAVNLITFEDRFNLYRIEQELGTEIKQIPPHI

DQAVYCG

>XP_020687132.1 DEAD-box ATP-dependent RNA helicase 8 [Dendrobium catenatum]

MNYRARYPPGNGSGRGGNSSNFYASRPQPQPQQQFVQGNLSASQHGQQLQQQQRWLRRNEIGNDSGNREEVGEVQSYGSN

HSIQDWKSHLNIPAPDTRYQTEDVTATKGNEFEDYFLKRELLMGIYEKGFESPSPIQEESIPIALTGRDILARAKNGTGK

TAAFCIPALEKIDQAKNVIQVVILVPTRELALQTSQVCKELGKYLKIQVMVTTGGTSLKDDIMRLYQPVHLVVGTPGRIL

DLARKDICVLKDCSMLIVDEADKLLSPEFQPSIEQLIGFLPAKRQILLFSATFPVTVKGFKDRYLSNPFTINLMDELTLK

GITHYYAFVEERQKVHCLNTLFSKLQINQSIIFCNSVNRVELLAKKITELGYSCFYIHAKMLQDHRNRVFHDFRNGACRN

LVCTDLFTRGIDIQAVNVVINFDFPKNSETYLHRVGRSGRFGHLGLAVNLITYEDRFNLYRIEQELAAEIKQIPPQIDQA

IYCK

>XP_006345594.1 PREDICTED: DEAD-box ATP-dependent RNA helicase 8 [Solanum tuberosum]

MNSRSGYPPPWMGGGGGSMHPNANTSFQQRNQQQYMQRSSVPHQQQFQNQQTQQWMRRSDSAIDEVEKTVQSENGSSQDW

KARLKIPPLDARYRTEDVTATKGNDFEDYFLKRELLMGIYEKGFERPSPIQEESIPIVLTGSDILARAKNGTGKTAAFCI

PALEKIDQDNNVIQVIILVPTRELALQTSQVCKELGKHLKIQVMVSTGGTSLKDDIMRLYQPVHLLVGTPGRILDLARKG

VCVLKDCSMLVMDEADKLLSPEFLPSIEQLIRFLPASRQILMFSATFPVTVKAFKDRYLQKPYVINLMDELTLKGITQFY

AFVEERQKLHCLNTLFSKLQINQSIIFCNSVNRVELLAKKITELGYSCFYIHAKMLQDHRNRVFHDFRNGACRNLVCTDL

FTRGIDIQAVNVVINFDFPKNSETYLHRVGRSGRFGHLGLAVNLITFEDRFNLYRIEQELGTEIKQIPPHIDQAVYCG

>CAB4074458.1 unnamed protein product [Lactuca saligna]

MNNNYARGGGGRYPPGIGRGGGGGNYHGNPNPNFQQQYAQRNPAHHQQFQQQQQQQQQQWLRRNPVGNDSSVVDEVEKTI

QSEAADPSGQDWKAQLRLPPADTRYRTEDVTATKGNEFEDYFLKRELLMGIYEKGFERPSPIQEESIPIALTGSDILARA

KNGTGKTAAFCIPALEKIDQDSNVIQVVILVPTRELALQTSQVCKELGKHLNIQVMVTTGGTSLKDDIMRLYQPVHLLVG

TPGRILDLSKKGICVLKDCAMLVMDEADKLLSPEFQPSVEQLIHFMPTNRQILMFSATFPVTVKDFKDRYLPKSYVINLM

DELTLKGITQFYAFVEERQKVHCLNTLFSKLQINQSIIFCNSVNRVELLAKKITELGYSCFYIHAKMLQDHRNRVFHDFR

NGACRNLVCTDLFTRGIDIQAVNVVINFDFPKNSETYLHRVGRSGRFGHLGLAVNLITYEDRFNLYRIEQELGTEIKQIP

PFIDQAIYCVSCLSVCLRKGPKGVWYVVSQVNPRED

>XP_016717650.1 PREDICTED: DEAD-box ATP-dependent RNA helicase 8-like [Gossypium hirsutum]

MNSRGRYPPAIGVGRGGGLNSNPGFQSRPPQQNYMQRNFVQNHHQFHNQHQHQQQRQQQWLRRNQLPGTNDSSVVGEVEK

TIQSEAFDSSSQDWKARLKMPPPDTRYKTEGILNCKGNEFEDYFLKRELLMGIYEKGFERPSPIQEESIPIALTGSDILA

RAKNGTGKTAAFCIPALEKIDQDKNVIQVVILVPTRELALQTSQVCKELGKHLQIQVMVTTGGTSLKDDIMRLYQPVHLL

VGTPGRILDLAKKGVCILKDCSMLIMDEADKLLSPEFQPSIEQLIRFLSAKRQILMFSATFPVTVKDFKDRYLQKPYIIN

LMDELTLKGITQYYAFVEERQKVHCLNTLFSKLQINQSIIFCNSVNRVELLAKKITELGYSCFYIHAKMLQDHRNRVFHD

FRNGACRNLVCTDLFTRGIDIQAVNVVINFDFPKNSETYLHRVGRSGRFGHLGLAVNLITYEDRFNLYRIEQELGTEIKQ

IPPHIDQAIYCR

>EPS67203.1 hypothetical protein M569_07571 [Genlisea aurea]

MSWRARYPPPGVGGGRVGGGGGGSGMHQVAGANSSFQPRNSTFQNHQRPPAPSFQNNFQQQWLPRGQLSAGESSVDEVEK

TVQSEATESKLQGWKAELNLPPKDTRYRTEDVTATKGNEFEDYFLKRELLMGIYEKGFERPSPIQEESIPIALTGSDILA

RAKNGTGKTAAFCIPALEKINQEKNAIQVVILVPTRELALQTAQVCKELGKHLMIQVMVTTGGTSLKDDIMRLHQPVHLL

VGTPGRILDLTRKGVCILNECNMLAMDEADKLLSQEFQPSVEQLIRFMPANRQILMFSATFPVTVKDFRDRYLNKPHVIN

LMDELTLKGVTQYYAFVEERQKVHCLNTLFSKLQINQSIIFCNSVNRVELLAKKITELGYSCFYIHAKMLQAHRNRVFHD

FRNGACRNLVSTDLFTRGIDIQSVNVVINFDFPKNSETYLHRVGRSGRFGHLGLAVNLITYEDRFSVHKIEAELNTELKQ

IPPQIDKDIYCR

>XP_009389244.1 PREDICTED: DEAD-box ATP-dependent RNA helicase 8 [Musa acuminata subsp. malaccensis]

MNPRSRYPPPGMGNGRGGGASTNPNFYARSAHQHQQYAQQSPAHGQPSQQFQQQQQWSRRNQLGGDFGAGQIVKSVQSEA

TNTSLQDWKAQLKMAPPDTRYKTEDVTATKGNEFEDYFLKRELLMGIYEKGFERPSPIQEESIPIALTGSDILARAKNGT

GKTAAFCIPALEKIDQDHNVIQVVILVPTRELALQTSQVCKELGKHLNIQVMVTTGGTSLKDDIMRLYQPVHLLVGTPGR

ILDLARKGVCVLKDCSMLIMDEADKLLSPEFQPSIEQLIQFLPANRQILMFSATFPVTIKHFKDRYLPKPYIINLMDELT

LKGITQFYAFVEERQKVQCLNTLFSKLQINQSIIFCNSVNRVELLAKKITELGYSCFYIHAKMLQDHRNRVFHDFRNGAC

RNLVCTDLFTRGIDIQAVNVVINFDFPKNSETYLHRVGRSGRYGHLGLAVNLITYEDRFNLYRIEQELGTEIKQIPPQID

QAIYCR

>XP_011091933.1 LOW QUALITY PROTEIN: DEAD-box ATP-dependent RNA helicase 8 [Sesamum indicum]

MSSRARYPPPGMGGGRGGAAAGMNPQGGPNPNFQPRNINQQYVQRSPAQNNQNHQLFQNPQPQQWLRRNQLPHTDSTVDE

VEKTVQSEAVESSSQDWKGRLKLPPADTRYRTEDVTATKGNEFEDYFLKRELLMGIYEKGFERPSPIQEESIPIALTGSD

ILARAKNGTGKTATFCXEKIDQDKNAIQVVILVPTRELALQTSQVCKELGKHLKIQVMVTTGGTSLKDDIMRLYQPVHLL

VGTPGRILDLAKKGVCILNECSVLVMDEADKLLSPEFQPSVEQLIRFLPANRQILMFSATFPVTVKDFKDRYLRKPYIIN

LMDELTLKGITQYYAFVEERQKVHCLNTLFSKLQINQSIIFCNSVNRVELLAKKITELGYSCFYIHAKMLQDHRNRVFHD

FRNGACRNLVCTDLFTRGIDIQAVNVVINFDFPKNSETYLHRVGRSGRFGHLGLAVNLITYEDRFNLYRIEQELGTEIKQ

IPPHIDQAIYCQ

>XP_019252209.1 PREDICTED: DEAD-box ATP-dependent RNA helicase 8-like [Nicotiana attenuata]

MNPRGGRYPPPGMGGGGGGRGGGNMYPNPNANPNFQQRNPQQQYVQRNPMNHQQQHPQQHYQNQQQQITQQQQWLRRNQQ

LAASDSSIDEVEKTVQSEALDQSSQDWKARLNIPAPDTRYRTEDVTATKGNEFEDYFLKRELLMGIYEKGFERPSPIQEE

SIPIALTGSDILARAKNGTGKTAAFCIPALEKIDQDVNAIQVVILVPTRELALQTSQVCKELGKHLKIEVMVTTGGTSLK

DDIMRLYQPVHLLVGTPGRILDLARKGICVLKDCSMLIMDEADKLLSPEFQPSIVQLIRFLPTNRQILMFSATFPVTVKD

FKDRFLQKPYVINLMDELTLKGITQFYAFVEERQKLHCLNTLFSKLQINQSIIFCNSVNRVELLAKKITELGYSCFYIHA

KMLQDHRNRVFHDFRNGACRNLVCTDLFTRGIDIQAVNVVINFDFPKNSETYLHRVGRSGRFGHLGLAVNLITFEDRFNL

YRIEQELGTEIKQIPPHIDQAIYCQ

>XP_025979654.1 DEAD-box ATP-dependent RNA helicase 8 isoform X2 [Glycine max]

MKNRDREKSPPRMGLGRGLNSNRGFQPRPQQYVQRHMVQHRHHLQPYQHNHHHHHHQQQRQQHHHHQQQRQWLRKDQLSG

GTNTNVVEEVEKTMQSEAINSSSQDWKARLNIPPPDTCHKTEDVTATKGNEFEDYFLKRELLMGIYEKGFERPSPIQEEC

IPIALTGSDILARAKNGTGKTAAFCIPALEKIDQDNDVIQVAILVPTRELALQTSQVCKDLGKHLKIQVMVTTGGTSLKD

DIMRLYQPVHLLVGTPGRILDLAKKGVCILNDCSMLVMDEADKLLSQEFQPSIEQLIQFLPGNRQILMFSATFPVTVKDF

KDRYLRKPYIVNLMDELTLKGITQYYAFLEERQKVHCLNTLFSKLQINQSIIFCNSVNRVELLAKKITELGYSCFYIHAK

MLQDHRNRVFHDFCNGACRNLVCTDLFTRGIDIQAVNVVINFDFPKNSETYLHRVGRSGRFGHLGLAVNLITYEDRFNLY

RIEQELGTEIKQIPPHIDQAIYCR

>KAF3327877.1 ATP-dependent RNA helicase dhh1 [Carex littledalei]

MNQRSRFPPGIGGEHGSNMGGNRNPDNNFYGRNPNPNNYNQQLQPQYQYVQRNQQPYYQQNQSHQQPRNQFRQNQLHQPQ

SHIQRNQSQHQQQSHFHQNQLHQQQQWARRNSNQIASGSGVEMIKSTPQIGTMDSSSENWKDKLNIPAADTRFKTEDVTA

TKGNEFEDYFLKRELLMGIFEKGYERPSPIQEEAIPIALTGSDILARAKNGTGKTAAFCIPVLEKIDNEKNVIQAVILVP

TRELALQTSQVCKEIGKYLNIEVMVTTGGTSLRDDIMRLYQPVHLLVGTPGRILDLTKKGICVLKDCSMVVLDEADKLLT

PEFQVSVAEMLSFIPPNRQILLFSATFPVTVKDFKDRYLPRAYVINLMDELTLKGITQYYAYVEEKQKVHCLNTLFSKLQ

INQSIIFCNSVNRVELLAKKITELGYSCFYIHAKMLQDHRNRVFHDFRNGACRNLVCTDLFTRGIDIQAVNVVINFDFPK

NAETYLHRVGRSGRYGHLGLAVNLITYEDRFNLYRIEKELGTEIKSIPQEIDRTVYCG

>XP_009757253.1 PREDICTED: DEAD-box ATP-dependent RNA helicase 8-like [Nicotiana sylvestris]

MNPRGGRYPPPGMGGGGGGRGGGNMYPNPNANPNFQQRNHQQQYVQRNPMNHPQQHPQQHYQNQQQITQQQQWLRRNQQL

ATSDSSIDEVEKTVQSEALDQSSQDWKARLNIPAPDTRYRTEDVTATKGNEFEDYFLKRELLMGIYEKGFERPSPIQEES

IPIALTGSDILARAKNGTGKTAAFCIPALEKIDQDVNAIQVVILVPTRELALQTSQVCKELGKHLKIEVMVTTGGTSLKD

DIMRLYQPVHLLVGTPGRILDLARKGICVLKDCSMLIMDEADKLLSPEFQPSIVQLIRFLPTNRQILMFSATFPVTVKDF

KDRFLQKPYVINLMDELTLKGITQFYAFVEERQKLHCLNTLFSKLQINQSIIFCNSVNRVELLAKKITELGYSCFYIHAK

MLQDHRNRVFHDFRNGACRNLVCTDLFTRGIDIQAVNVVINFDFPKNSETYLHRVGRSGRFGHLGLAVNLITFEDRFNLY

RIEQELGTEIKQIPPHIDQAIYCQ

>KAF5946294.1 hypothetical protein HYC85_016522 [Camellia sinensis]

MNTRGRYPPGIGGGGGRGGSLNVNLNFHPRNFQQQYVQRGPMQNHHQQFQSQQQHQQHQQHQQPQWLRRNQLGAELSVDE

VEKTVRSEAVDSSSQDWKARLKIPPADTRYRTEDVTATKGNEFEDYFLKRDLLMGIYEKGFERPSPIQEESIPIALTGSD

ILARAKNGTGKTAAFCIPALEKIDQDNNVIQVVILVPTRELALQTSQVCKELGKYLNIQVMVTTGGTSLKEDIMRLYQPV

HLLVGTPGRILDLAKKGVCILKDCSMLVMDEADKLLSPEFRPSLDQLIHFLPVNRQILMFSATFPVTVKDFKDRYLHRSY

VINLMDELTLKGITQFYAFVEERQKVHCLNTLFSKLQINQSIIFCNSVNRVELLAKKITELGYSCFYIHAKMLQDHRNRV

FHDFRNGACRNLVCTDLFTRGIDIQAVNVVINFDFPKNSETYLHRVGRSGRFGHLGLAVNLITYEDRFNLYRIEQELGTE

IKQIPPHIDQGLMGLMIQDGIRCSFTECYIRIRLASQSLLSFLTDC

>XP_006587857.1 DEAD-box ATP-dependent RNA helicase 8 isoform X1 [Glycine max]

MIPEKFPISPLDFVSLGPNHNPILRIFRSQSKIGGMKNRDREKSPPRMGLGRGLNSNRGFQPRPQQYVQRHMVQHRHHLQ

PYQHNHHHHHHQQQRQQHHHHQQQRQWLRKDQLSGGTNTNVVEEVEKTMQSEAINSSSQDWKARLNIPPPDTCHKTEDVT

ATKGNEFEDYFLKRELLMGIYEKGFERPSPIQEECIPIALTGSDILARAKNGTGKTAAFCIPALEKIDQDNDVIQVAILV

PTRELALQTSQVCKDLGKHLKIQVMVTTGGTSLKDDIMRLYQPVHLLVGTPGRILDLAKKGVCILNDCSMLVMDEADKLL

SQEFQPSIEQLIQFLPGNRQILMFSATFPVTVKDFKDRYLRKPYIVNLMDELTLKGITQYYAFLEERQKVHCLNTLFSKL

QINQSIIFCNSVNRVELLAKKITELGYSCFYIHAKMLQDHRNRVFHDFCNGACRNLVCTDLFTRGIDIQAVNVVINFDFP

KNSETYLHRVGRSGRFGHLGLAVNLITYEDRFNLYRIEQELGTEIKQIPPHIDQAIYCR

>BAF26914.2 Os10g0503700 [Oryza sativa Japonica Group]

MHHPRARYPPGYTSGGGGGGGGGGGGGRGNGGGGFGGGGGGGGGNHGYYGRGPQPQPQQQHYHHQAQQLHQHQQQQQHAQ

RNSSSQQQQWLRRDQATAAAASGEVAARTAAQLEAVDSSSEDWKAQLNLPAPDTRYRTEDVTATKGNEFEDYFLKRELLM

GIYEKGFERPSPIQEESIPIALTGSDILARAKNGTGKTAAFCIPALEKIDPEKNAIQVVILVPTRELALQTSQVCKELGK

YLNIQVMVSTGGTSLKDDIMRLYQPVHLLVGTPGRILDLTRKGICVLKDCSMLVMDEADKLLAPEFQPSIEQLIHFLPAN

RQLLMFSATFPVTVKDFKEKYLPRPYVINLMDELTLKGITQYYAFVEERQKVHCLNTLFLKLQINQSIIFCNSVNRVELL

AKKITELGYSCFYIHAKMLQDHRNRVFHDFRNGACRNLVCTDLFTRGIGIQAVNVVINFDFPKTSETYLHRVGRSGRFGH

LGLAVNLITYEDRFNLYRIEQELGTEIKTIPPQIDLAVYCQ

>XP_016436204.1 PREDICTED: DEAD-box ATP-dependent RNA helicase 8-like [Nicotiana tabacum]

MNPRGGRYPPPGMGGGGGGRGGGNMYPNPNANPNFQQRNPQQQYVQRNPMNHQQQHQQQHYQNQQQITQQQQWLRRSQQL

AASDSSIDEVEKTVQSEALDQSSQDWKARLNIPAPDTRYRTEDVTATKGNEFEDYFLKRELLMGIYEKGFERPSPIQEES

IPIALTGSDILARAKNGTGKTAAFCIPALEKIDQDVNAIQVVILVPTRELALQTSQICKELGKHLNIEVMVTTGGTSLKD

DIMRLYQPVHLLVGTPGRILDLARKGICVLKDCSMLIMDEADKLLSPEFQPSIVQLIRFLPANRQILMFSATFPVTVKDF

KDRFLQKPYVINLMDELTLKGITQFYAFVEERQKLHCLNTLFSKLQINQSIIFCNSVNRVELLAKKITELGYSCFYIHAK

MLQDHRNRVFHDFRNGACRNLVCTDLFTRGIDIQAVNVVINFDFPKNSETYLHRVGRSGRFGHLGLAVNLITFEDRFNLY

RIEQELGTEIKQIPPHIDQAIYCQ

>XP_009611007.1 DEAD-box ATP-dependent RNA helicase 8-like [Nicotiana tomentosiformis]

MNPRGGRYPPPGMGGGGGGRGGGNMYPNPNANPNFQQRNPQQQYVQRNPMNHQQQHQQQHYQNQQQITQQQQWLRRSQQL

AASDSSIDEVEKTVQSEALDQSSQDWKARLNIPAPDTRYRTEDVTATKGNEFEDYFLKRELLMGIYEKGFERPSPIQEES

IPIALTGSDILARAKNGTGKTAAFCIPALEKIDQDVNAIQVVILVPTRELALQTSQVCKELGKHLNIEVMVTTGGTSLKD

DIMRLYQPVHLLVGTPGRILDLARKGICVLKDCSMLIMDEADKLLSPEFQPSIVQLIRFLPANRQILMFSATFPVTVKDF

KDRFLQKPYVINLMDELTLKGITQFYAFVEERQKLHCLNTLFSKLQINQSIIFCNSVNRVELLAKKITELGYSCFYIHAK

MLQDHRNRVFHDFRNGACRNLVCTDLFTRGIDIQAVNVVINFDFPKNSETYLHRVGRSGRFGHLGLAVNLITFEDRFNLY

RIEQELGTEIKQIPPHIDQAIYCQ

>KAE9460133.1 hypothetical protein C3L33_07995, partial [Rhododendron williamsianum]

MHEVQNGWDMPSPDFGGICPLASQDWKAQLRIPAPDTRYRTEDVTATKGNEFEDYFLKRELLMGIYEKGFERPSPIQEES

IPIALTGSDILARAKNGTGKTAAFCIPALEKIDQDNNVIQVVILVPTRELALQTSQVCKELGKHLKIQVMVTTGGTSLKD

DIMRLYQPVHLLVGTPGRILDLAKKGICVLKDCSMLAMDEADKLLSPEFQPSIEQLIHFLHPNRQILMFSATFPVTVKDF

KDRYLHKPYVINLMDELTLKGITQFYAFVEERQKVHCLNTLFSKLQINQSIIFCNSVNRVELLAKKITELGYSCFYIHAK

MLQDHRNRVFHDFRNGACRNLVCTDLFTRGIDIQAVNVVINFDFPKNAETYLHRVGRSGRFGHLGLAVNLITYEDRFNLY

RIEQELGTEIKQIPPHIDQAIYCQ

>KAB5569175.1 hypothetical protein DKX38_002968 [Salix brachista]

MNYNNRGRYPPGIGAGRGGGMNANPNFQSRVPQQQYVQRNFGQNHHQQQQYYQHQQHHNQQQQQQWLRRNQLSAAESSVD

EVEKTVQSEAVDSSFQTTYAIIDASFGYLLETEILLFCILVCSSSQDWKAKLKIPPADTRYRTEEISKLNDKCKSCEVEV

YSLYAMDVTATKGNDFEDYFLKRELLMGIYEKGFERPSPIQEESIPIALTGSDILARAKNGTGKTAAFCIPALEKIDQDN

NFIQVVMLVPTRELALQTSQVCKELGKHLNIQVMSTTGGTSLKDDIMRLYQPVHLLVGTPGRILDLAKKGVCILKNCSML

VLDEADKLLSPEFQPSIEQLIRFLPSNRQILMFSATFPVTVKDFKDRYLEKPYVINLMDELTLKGITQFYAFVEERQKVH

CLNTLFSKLQINQSIIFCNSVNRVELLAKKITELGYSCFYIHAKMLQDHRNRVFHDFRNGACRNLVCTDLFTRGIDIQAV

NVVINFDFPKNAETYLHRVGRSGRFGHLGLAVNLITYEDRFNLYRIEQELGTEIKQIPPHIDQGIYCQ

>XP_017219623.1 PREDICTED: DEAD-box ATP-dependent RNA helicase 6 isoform X2 [Daucus carota subsp. sativus]

MCRYSRQESSQDWKAQVKKPPPDNRYKTEDVTATKGNEFEDYFLKRELLMGIYEKGFDKPSPIQEESIPIALTGSNILAR

AKNGTGKTAAFCIPALEKIDSDKNVIQVAILVPTRELALQTSQVCKELGKHLKIQVMVTTGGTSLKDDIMRLYQHVHLLV

GTPGRILDLTKKGVCLLNECSMLVMDEADKLLSPEFEPSVKELIDYLPQNRQILMFSATFPVTVKAFKDRYLGTPYVINL

MDELTLKGITQYYAFVEERQKIHCLNTLFSKLQINQSIIFCNSVSRVELLAKKITELGYSCFYIHAKMLQDHRNRVFHDF

RNGACRNLVCTDLFTRGIDIQAVNVVINFDFPRTSETYLHRVGRSGRFGHLGLSVNLITYEDRFNMYNIEKELGTEIKQI

PPAIDQAVYCR

>XP_017219615.1 PREDICTED: DEAD-box ATP-dependent RNA helicase 6 isoform X1 [Daucus carota subsp. sativus]

MNNQGRYQPGFRGTRGGGVFRGSPNYYQQRPGVQQNNQHQQLWLRKRPGGPGERRGGNTFQAQAHNDSGSQDWKAQVKKP

PPDNRYKTEDVTATKGNEFEDYFLKRELLMGIYEKGFDKPSPIQEESIPIALTGSNILARAKNGTGKTAAFCIPALEKID

SDKNVIQVAILVPTRELALQTSQVCKELGKHLKIQVMVTTGGTSLKDDIMRLYQHVHLLVGTPGRILDLTKKGVCLLNEC

SMLVMDEADKLLSPEFEPSVKELIDYLPQNRQILMFSATFPVTVKAFKDRYLGTPYVINLMDELTLKGITQYYAFVEERQ

KIHCLNTLFSKLQINQSIIFCNSVSRVELLAKKITELGYSCFYIHAKMLQDHRNRVFHDFRNGACRNLVCTDLFTRGIDI

QAVNVVINFDFPRTSETYLHRVGRSGRFGHLGLSVNLITYEDRFNMYNIEKELGTEIKQIPPAIDQAVYCR

>PPS00878.1 hypothetical protein GOBAR_AA19779 [Gossypium barbadense]

MPPPDTRYKTEDVTATKGNEFEDYFLKRELLMGIYEKGFERPSPIQEESIPIALTGSDILARAKNGTGKTAAFCIPALEK

IDQDKNVIQVVILVPTRELALQTSQVCKELGKHLQIQVMVTTGGTSLKDDIMRLYQPVHLLVGTPGRILDLAKKGVCILK

DCSMLIMDEADKLLSPEFQPSIEQLIRFLSAKRQILMFSATFPVTVKDFKDRYLQKPYIINLMDELTLKGITQYYAFVEE

RQKVHCLNTLFSKLQINQSIIFCNSVNRVELLAKKITELGYSCFYIHAKMLQDHRNRVFHDFRNGACRNLVCTDLFTRGI

DIQAVNVVINFDFPKNSETYLHRVGRSGRFGHLGLAVNLITYEDRFNLYRIEQELGTEIKQIPPHIDQAIYCR
