# Supplementary material for: DDX6 Is Essential for Oocyte Development and Maturation in Locusta migratoria
Source: Insects. 2021 Jan 14;12(1):70. doi: 10.3390/insects12010070 (PMC7830464; doi:10.3390/insects12010070)
Supplement: Supplementary file 1 [file insects-12-00070-s001.zip › Supplementary material/Supplemental file 7.docx]

**Translation initiation factor eIF4A [Saccharomyces cerevisiae S288C]**

NCBI Reference Sequence: NP_012985.3

[GenPept](https://www.ncbi.nlm.nih.gov/protein/NP_012985.3?report=genpept) [Identical Proteins](https://www.ncbi.nlm.nih.gov/ipg/NP_012985.3) [Graphics](https://www.ncbi.nlm.nih.gov/protein/NP_012985.3?report=graph)

>NP_012985.3 translation initiation factor eIF4A [Saccharomyces cerevisiae S288C]

MSEGITDIEESQIQTNYDKVVYKFDDMELDENLLRGVFGYGFEEPSAIQQRAIMPIIEGHDVLAQAQSGTGKTGTFSIAALQRIDTSVKAPQALMLAPTRELALQIQKVVMALAFHMDIKVHACIGGTSFVEDAEGLRDAQIVVGTPGRVFDNIQRRRFRTDKIKMFILDEADEMLSSGFKEQIYQIFTLLPPTTQVVLLSATMPNDVLEVTTKFMRNPVRILVKKDELTLEGIKQFYVNVEEEEYKYECLTDLYDSISVTQAVIFCNTRRKVEELTTKLRNDKFTVSAIYSDLPQQERDTIMKEFRSGSSRILISTDLLARGIDVQQVSLVINYDLPANKENYIHRIGRGGRFGRKGVAINFVTNEDVGAMRELEKFYSTQIEELPSDIATLLN

>MBG00781.1 hypothetical protein [Acidimicrobiaceae bacterium]

MDESNKTKLSFDDLSLKDHLLRGVYSYGFENPSKIQHLAIPKIATGKDLIAQAQSGTGKTGAFTISVLQNLKEEQKNTQVLILSPTHELVHQINEVITSLSNYMDVSIMEVIGGTNVYECKKQLEKCPQIIVGTPGRVLDMIQKKCLFTDRIHTIIFDEADEILSYGFKESIYQIVQQIPENTQICLFSATMPEDVIELSDRFMNNPDKILIKKEALTLEGITQFYINIKINEWKFDVLKDLYDTINVSQCIIYINSKNKLMDLYQNLTKENFPVSYIHGELSSQERKDVMENFRSGHSRILLSTDLLSRGIDVQQLSLVINFDLPKSKETYIHRIGRSGRYGRKGVSINLVTDRDISYMKDIETFYETKIEEMPHNIADFLSV

>MBA42999.1 hypothetical protein [Magnetococcales bacterium]

MDNNTVVINSFEEMGLKEELLRGVFGYGFETPSVIQERAIPAILTRRDVIAQAQSGTGKTSTFTISALQRIDENKPYVQCIILAHTHELAHQIKGVIDQISEYMKNIKTCLCIGGTLISDSIKEIKSGAQIIIGTPGRVLHMIQKSFVKKSNIEMLIMDEADELLSGSFQEQIKDIIKTGTDSESQICIFSATLPKDKLDLTLHFMNKPLTILVKKEQLTLEGISQFYDYIERDEYKFDYLCHIYDRISISQSIIYVNTVKKASLLAEKLHEKNFTVTVIHGNMPSHERTEVMKQFRNGTSRILISTDLLARGIDVQQVSIVINYDIPTNRESYIHRIGRSGRYGRKGVAINFVTRRDTDKMRELEEFYNTQIVEIPENIGSYL
