# Supplementary material for: DDX6 Is Essential for Oocyte Development and Maturation in Locusta migratoria
Source: Insects. 2021 Jan 14;12(1):70. doi: 10.3390/insects12010070 (PMC7830464; doi:10.3390/insects12010070)
Supplement: Supplementary file 1 [file insects-12-00070-s001.zip › Supplementary material/Table S1.docx]

**Table S1.** Primers used in this study

| Genes | Primer sequence (5’-3’) | Purpose |
| --- | --- | --- |
| *LmDDX6* | F: CAGGAAGATTTGGTCATCTCGG | RT-qPCR |
|  | R: GCGTCTTCGATTTTCGTTGC |  |
| *VgA* | F: CCCACAAGAAGCACAGAACG |  |
|  | R: TTGGTCGCCATCAACAGAAG |  |
| *VgB* | F: AACGCCGACAGTGTTGGTATTC |  |
|  | R: ACCATCAGAAGTCGCTGGAAGT |  |
| *VgR* | F: TTCAAGAGGCTGTCGGGTTCC |  |
|  | R: GCAGTCATGAGGTCGGTCTTCT |  |
| *Met* | F: GTGCCTGAAGAAGAAGAAC |  |
|  | R: GGAGGTGATGAAGGAGAG |  |
| *Grp78-1* | F: GGGGACACTCATTTGGGTG |  |
|  | R: TTGCTTTTTCTACTTCACGGC |  |
| *Grp78-2* | F: TGTTAGTAGGTGGAAGCACAAGGA |  |
|  | R: GCACAGCAGCACCATATACAACT |  |
| *β-actin* | F: CGAAGCACAGTCAAAGAGAGGTA |  |
|  | R: GCTTCAGTCAAGAGAACAGGATG |  |
| *LmDDX6* | F: taatacgactcactatagggAAACGAGAATTGCTGATGGG | dsRNA synthesis |
|  | R: taatacgactcactatagggGTGCAATGACATGATCCAGC |  |
| *GFP* | F: taatacgactcactatagggGACGTAAACGGCCACAAGTT |  |
|  | R: taatacgactcactatagggTGTTCTGCTGGTAGTGGTCG |  |
